# Characterizing the impact of simvastatin co-treatment of cell specific TCDD-induced gene expression and systemic toxicity

**DOI:** 10.1038/s41598-023-42972-8

**Published:** 2023-10-03

**Authors:** Amanda Jurgelewicz, Rance Nault, Jack Harkema, Timothy R. Zacharewski, John J. LaPres

**Affiliations:** 1 Department of Pharmacology and Toxicology, Michigan State University, East Lansing, MI USA; 2 Institute for Integrative Toxicological Sciences, Michigan State University, East Lansing, MI USA; 3 Department of Biochemistry and Molecular Biology, Michigan State University, 602 Wilson Rd, East Lansing, MI 48824 USA; 4 Department of Pathobiology and Diagnostic Investigation, Michigan State University, East Lansing, MI USA

**Keywords:** Biochemistry, RNA, Metabolic disorders

## Abstract

2,3,7,8-tetrachlorodibenzo-*p*-dioxin (TCDD) is associated with metabolic syndrome (MetS) in humans and elicits pathologies in rodents that resemble non-alcoholic fatty liver disease (NAFLD) in humans through activation of the aryl hydrocarbon receptor (AHR) pathway. Dysregulation of cholesterol homeostasis, an aspect of MetS, is linked to NAFLD pathogenesis. TCDD exposure is also linked to the suppression of genes that encode key cholesterol biosynthesis steps and changes in serum cholesterol levels. In a previous experiment, treating mice with TCDD in the presence of simvastatin, a 3-Hydroxy-3-Methylglutaryl-CoA Reductase competitive inhibitor, altered lipid and glycogen levels, AHR-battery gene expression, and liver injury in male mice compared to TCDD alone. The aim of this study was to deduce a possible mechanism(s) for the metabolic changes and increased injury using single-nuclei RNA sequencing in mouse liver. We demonstrated that co-treated mice experienced wasting and increased AHR activation compared to TCDD alone. Furthermore, relative proportions of cell (sub)types were different between TCDD alone and co-treated mice including important mediators of NAFLD progression like hepatocytes and immune cell populations. Analysis of non-overlapping differentially expressed genes identified several pathways where simvastatin co-treatment significantly impacted TCDD-induced changes, which may explain the differences between treatments. Overall, these results demonstrate a connection between dysregulation of cholesterol homeostasis and toxicant-induced metabolic changes.

## Introduction

Non-alcoholic fatty liver disease (NAFLD) is an umbrella term that encompasses a spectrum of progressive liver pathologies ranging from fat accumulation (steatosis) to fat accumulation with inflammation (steatohepatitis), leading to irreversible changes such as fibrosis or cirrhosis^1^. These changes can eventually progress to hepatocellular carcinoma (HCC). It is estimated that one quarter of the global population has NAFLD, and it is the fastest growing cause of HCC in the United States^2^. Although NAFLD has become the primary reason for liver transplantation, there are currently no recommended pharmaceutical interventions^1,2^. The liver is a vital metabolic organ and is essential for detoxifying environmental pollutants. Interestingly, some of these toxicants have been associated with inducing pathology similar to NAFLD, including 2,3,7,8-tetrachlorodibenzo-*p*-dioxin (TCDD)^3^. TCDD exposure in mice promotes steatosis and progression into steatohepatitis with fibrosis^4,5^. TCDD elicits, most, if not all, of its toxic effects through binding to the aryl hydrocarbon receptor (AHR)^6^. Upon TCDD binding, the AHR can alter cellular metabolism via changes in gene expression affecting multiple metabolic pathways^7–9^.

One major pathway that is impacted by TCDD is cholesterol biosynthesis by suppressing the expression of genes that encode key enzymes in the process, including the rate-limiting enzyme 3-hydroxy-3-methylglutaryl-CoA reductase (HMGCR)^10–12^. These expression changes correlate with an alteration in circulating free cholesterol levels, high-density lipoproteins (HDLs) and low-density lipoproteins (LDLs) in mice^10–12^. Dysregulation of cholesterol homeostasis is one of the conditions that can lead to the development of metabolic syndrome (MetS), and TCDD exposure is associated with an increased incidence of MetS in humans^13–15^. Epidemiology studies have shown that MetS increases the risk of NAFLD and doubles the cases with advanced fibrosis^16,17^. Furthermore, dysregulation of cholesterol homeostasis is linked to NAFLD pathogenesis as free cholesterol accumulation can lead to mitochondrial dysfunction and liver injury caused by activation of signaling pathways that promote inflammation and fibrogenesis^18–20^. The liver is the primary location of cholesterol biosynthesis, suggesting a strong link between cholesterol homeostasis, AHR-mediated signaling, and liver injury.

Previously, we characterized the impact of HMGCR repression in TCDD-induced liver injury by co-treating mice with TCDD and simvastatin, a commonly prescribed HMCGR competitive inhibitor. In that study, co-treatment decreased TCDD-mediated hepatic lipid accumulation in males and females, increased glycogen and decreased ketone bodies in female mice and increased alanine aminotransferase levels in male mice, suggesting statins alter TCDD-induced changes to metabolism and these changes might play a role in toxicant-induced injury^12^. As millions of people take statins, it is important to elucidate the additional risk statins present to populations exposed to TCDD and related compounds^21^.

In this study, we aimed to determine how simvastatin impacts TCDD-induced changes in metabolism by characterizing the impact of simvastatin co-treatment on cell-specific gene expression. A previous single-nuclei RNA sequencing (snRNAseq) study showed that TCDD alters relative proportions of cell types and elicits cell-specific gene expression changes^22^. Therefore, snRNASeq was used to investigate the cell-specific gene expression changes following TCDD and simvastatin co-treatment and the role of different cell (sub)types. When TCDD and co-treated mice were compared, we observed changes in the relative proportions of cell (sub)types including hepatocytes and macrophages which are important mediators of NAFLD progression. In addition, we identified several treatment-group-specific differentially expressed genes associated with NAFLD related pathways. Though serum alanine aminotransferase (ALT) levels were not significantly different between TCDD and co-treatment, TCDD and simvastatin elicited wasting and caused lethality in some mice. These results demonstrate that TCDD-induced AHR-mediated toxicity is exacerbated by simvastatin and this may be due to alterations in the level of AHR activity, inflammatory response and insulin signaling.

## Results

### Simvastatin leads to worse prognosis of TCDD-induced liver injury

**Figure 1:**
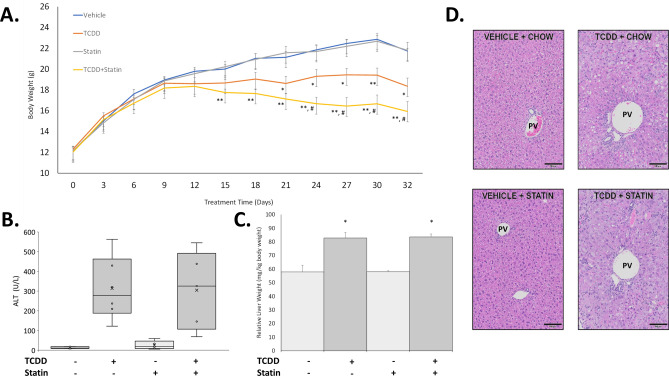
Simvastatin co-treatment induces wasting. (**A**) The average body weight of the mice in each respective group measured every 3 days. (**B**) Measured alanine aminotransferase (ALT) levels (U/L) in serum for each treatment. (**C**) Relative liver weight (mg of liver/kg body weight) for each treatment. (**D**) Representative H&E-stained liver samples for each treatment. The portal vessel (PV) is indicated on each image. Scale bar = 100 µm. Asterisks indicate statistical significance compared to respective control group: * (*P* ≤ 0.05), ** (*P* ≤ 0.01). Pound symbol indicates statistical significance between TCDD and T + S groups: # (*P* ≤ 0.05). N ≥ 5 mice per treatment group.

To characterize the impact of HMGCR inhibition on TCDD-induced, AHR-mediated signaling, mice were treated with TCDD (30 µg/kg body weight) in the presence and absence of simvastatin (~ 80 mg/kg body weight/day). As the experiment progressed, co-treated mice were more severely impacted compared to either TCDD or simvastatin alone. Simvastatin alone did not modulate weight gain compared to control, but there was a difference between TCDD and TCDD + Simvastatin (T + S) (Fig. 1A). TCDD reduced weight gain after 5 doses, while T + S reduced weight gain after only 3 doses with wasting after 4 doses (Fig. 1A). Co-treatment also led to two deaths over the course of the study. Across multiple past experiments utilizing a similar exposure paradigm, TCDD alone caused no deaths, suggesting simvastatin exacerbated TCDD-induced toxicity.

Interestingly, while failure to thrive was exacerbated by co-treatment, there was no difference in liver damage severity. Neither serum ALT levels nor liver weights between TCDD and co-treated groups were significantly different (Fig. 1B–C). However, one mouse had a much higher level of serum ALT compared to the other mice in the T + S group (Fig. 1B). Although this mouse is not a significant outlier based on Grubb's test analysis, the other T + S-treated mice, on average, had lower ALT levels compared to TCDD alone. Furthermore, histopathology between TCDD and co-treated mice were comparable. TCDD induced widespread vacuolization (score 4) with hepatocellular hypertrophy (score 4) and mild mixed inflammatory cell infiltrate (score 2) with minimal necrosis (Fig. 1D). Lesions were most severe in periportal regions. Co-treatment elicited similar lesions in both character and severity to TCDD-treated mice with the addition of mild biliary hyperplasia. One mouse in the co-treated group did not display biliary hyperplasia and had minimal vacuolization (score 1) and mild-moderate necrosis (score 2–3) (Fig. 1D). With the exception of this one mouse, which was not the mouse with higher ALT levels, all other mice had the same pathologies and severity scoring as the other mice in their respective treatment groups. No significant histopathology lesions were observed in control or simvastatin-treated mice.

### snRNASeq suggests differences in liver cell type proportions between treatments

**Figure 2:**
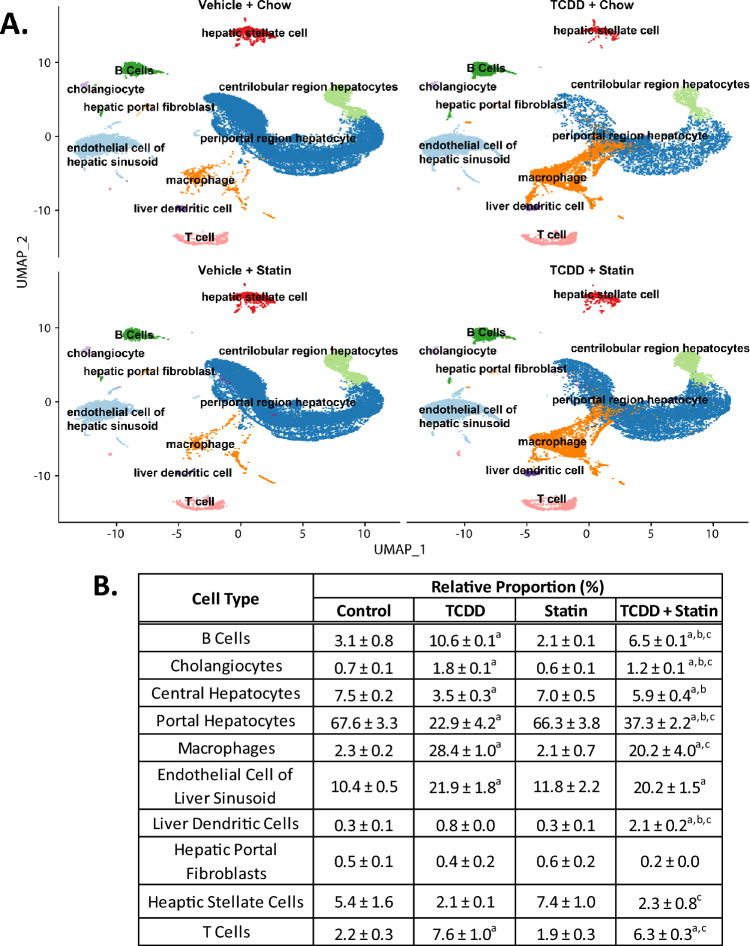
Simvastatin co-treatment effect on relative liver cell types. (**A**) UMAP visualization of nuclei isolated from livers of mice treated with vehicle control, TCDD, simvastatin or TCDD + simvastatin, clustered based on gene expression profile similarity. (**B**) Relative proportions of each cell type for each treatment group. Letters indicate statistical significance (*P* ≤ 0.05) in comparison to control (a), TCDD (b) or simvastatin (c). N = 3 mice per treatment group.

Although T + S elicited no significant changes in liver damage severity compared to TCDD alone, we hypothesized hepatic cell type and cell specific gene expression changes may explain the increased T + S toxicity (i.e. wasting). A total of 83,229 nuclei (average per group) were analyzed using snRNAseq. Relative proportions of liver cell nuclei clusters across treatments were identified as described in the material and methods (Fig. 2A). Simvastatin alone did not alter cell type proportions compared to control mice (Fig. 2B). TCDD elicited shifts in 7 of 10 cell types identified including an increase in macrophages (2.3–28.4%), B cells (3.1–10.6%) and T cells (2.2–7.6%) (Fig. 2A–B). This is consistent with previous reports using snRNAseq data as well as F4/80 staining of TCDD treated liver sections^22,23^.

T + S elicited shifts in proportions for 8 of 10 cell types identified compared to control mice, including the same 7 cell types impacted by TCDD alone (Fig. 2A–B). However, the proportions of B cells, cholangiocytes, centrilobular region (central) hepatocytes and periportal region (portal) hepatocytes differed between TCDD alone and co-treatment (Fig. 2B). Although co-treated mice had a smaller proportion of central (7.5–5.9%) and portal (67.6–37.3%) hepatocytes compared to control, this was increased compared to TCDD alone (3.5% and 22.9%, respectively). There was a large increase in the macrophage population in co-treated mice (2.3–20.2%) compared to control as well. However, the 8.2% difference between TCDD alone and T + S-treated mice was not significant due to one sample driving larger error in the co-treated group. Interestingly, the proportion of liver dendritic cells only increased in the co-treated mice (2.1%) compared to control (0.3%), statin alone (0.3%) and TCDD alone (0.8%) (Fig. 2B).

### Comparing TCDD and TCDD + simvastatin changes in portal hepatocyte pathways

**Figure 3:**
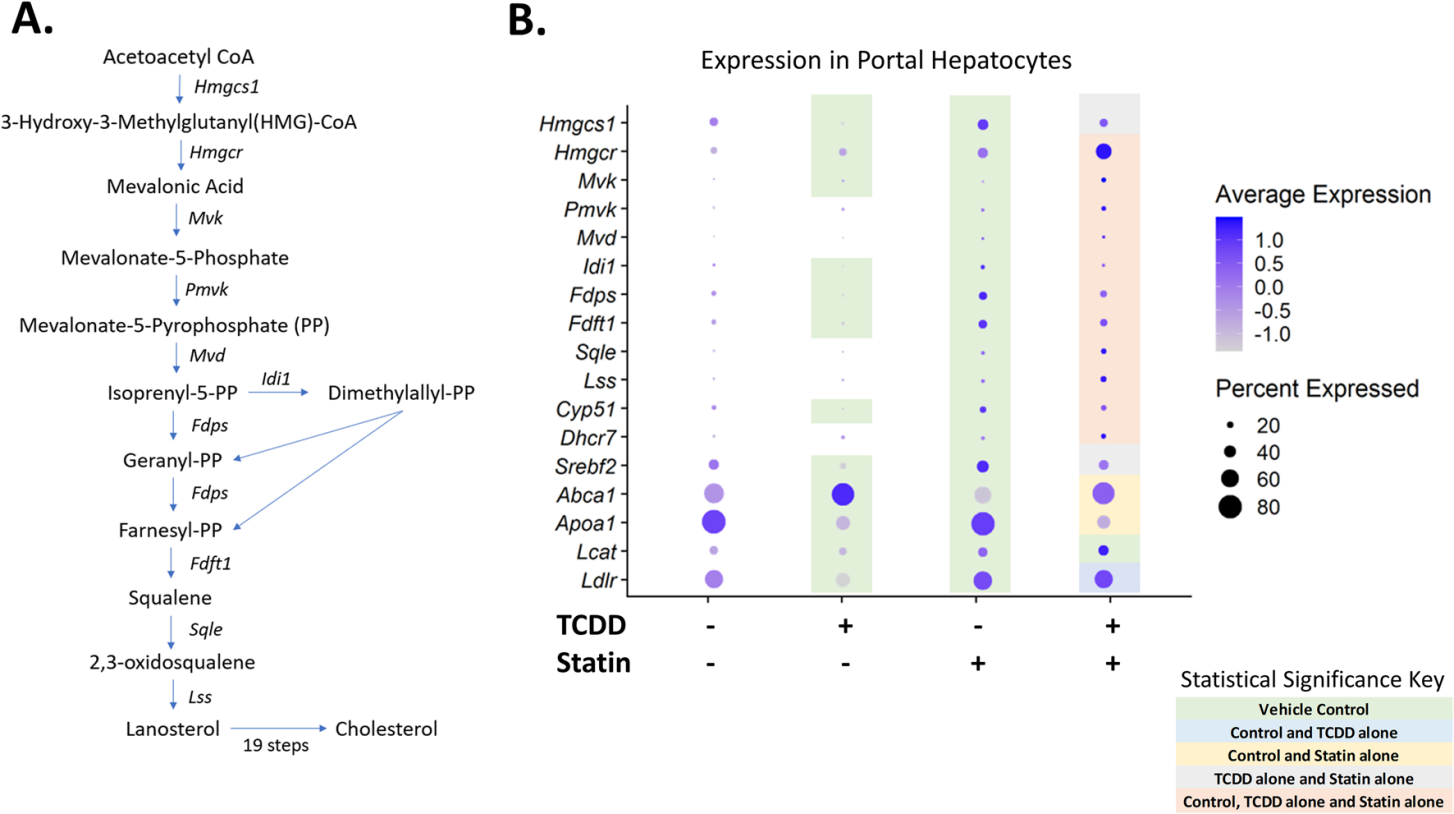
Cholesterol biosynthesis gene expression. (**A**) Cholesterol biosynthesis pathway. (**B**) Dot plot of genes involved in cholesterol biosynthesis and homeostasis in portal hepatocytes. Colors indicate significant differences in expression between treatment groups (*P* ≤ 0.05): green (vehicle); blue (vehicle and TCDD alone); yellow (vehicle and statin alone); gray (TCDD alone and statin alone) and peach (vehicle, TCDD alone and statin alone). N = 3 mice per treatment group.

Early stages of NAFLD are diagnosed by fat accumulation in hepatocytes. Hepatocytes exist as a gradient in the liver based on their lobule position ranging from being near the portal vein (periportal) to the central vein (centrilobular). Hepatocyte position in the lobule influences function with cholesterol biosynthesis primarily occurring in portal hepatocytes^24^. Furthermore, TCDD induces periportal toxicity despite preferential accumulation in the pericentral region due to CYP1A2 sequestration^22,25^. Thus, portal hepatotoxicity may play an important role in connecting cholesterol biosynthesis and TCDD-induced liver injury. As expected, TCDD decreased expression of many genes involved in cholesterol biosynthesis compared to control such as *Hmgcs1*, *Fdft1* and *Cyp51* (Fig. 3A–B). Furthermore, TCDD also decreased expression of additional genes involved in cholesterol homeostasis such as *Ldlr*, a receptor that mediates cholesterol re-uptake into the liver, and *Abca1*, a protein involved in cholesterol efflux (Fig. 3B). The enzymes involved in cholesterol biosynthesis that were assessed were more upregulated in co-treated mice compared to TCDD alone (Fig. 3B). In contrast, genes such as *Srebf2*, a transcription factor that regulates cholesterol homeostasis, had significantly lower expression in co-treated mice compared to simvastatin alone (Fig. 3B).

**Figure 4:**
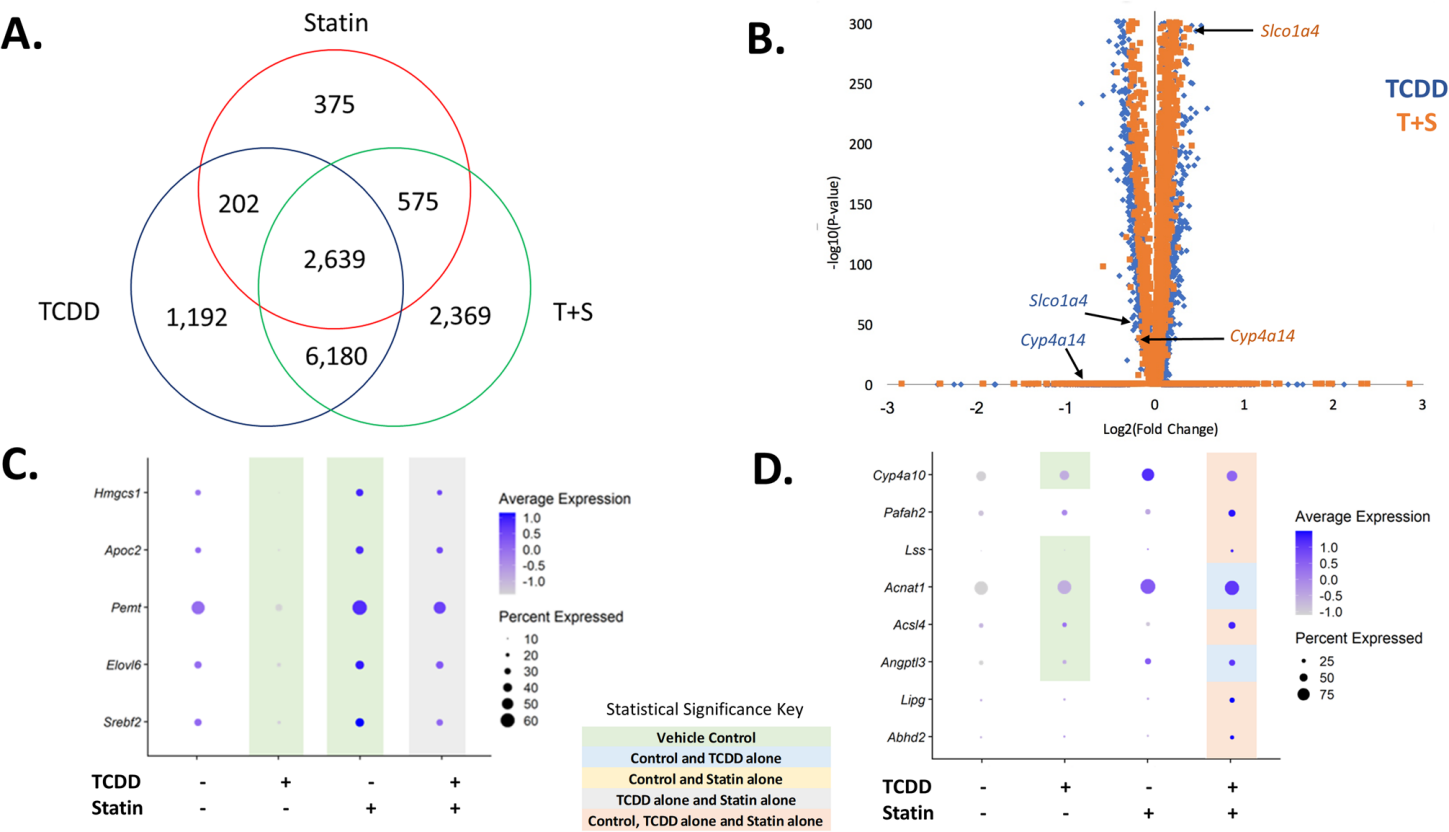
Simvastatin co-treatment impact on portal hepatocytes. (**A**) Venn diagram of the statistically significant (*P* ≤ 0.05) differentially expressed genes (DEGs) compared to control that are shared between treatment groups. (**B**) Volcano plot representing the significant DEGs compared to control that were shared between TCDD and T + S treatments where the fold changes for both treatments were also significantly different from each other. The gene list is available in supplementary table 3. The plot shows the log_2_ fold change of these DEGs for both TCDD (blue) and T + S (orange) corresponding to their respective p-values. (**C**) Dot plot of DEGs in the DAVID cluster involving lipid metabolism in TCDD-treated mice. The top significant clusters generated by DAVID are listed in supplementary table 5. (**D**) Dot plot of the DEGs in the DAVID cluster involving lipid metabolism in T + S-treated mice. The top significant clusters generated by DAVID are listed in supplementary table 5. Colors indicate statistical significance (P ≤ 0.05) in expression between treatment groups: green (vehicle); blue (vehicle and TCDD alone); gray (TCDD alone and statin alone) and peach (vehicle, TCDD alone and statin alone). N = 3 mice per treatment group.

Although there was a difference between the relative proportion of portal hepatocytes between TCDD (22.9%) and co-treated (37.3%) mice, the majority of differentially expressed genes (DEGs) between these two groups were the same compared to control (Fig. 4A). Interestingly, a large portion of these DEGs had expression levels that were different when comparing co-treatment to TCDD alone, including those that encode transporters like *Slco1a4* and enzymes involved in metabolism such as *Cyp4a14* (Fig. 4B). DAVID analysis of these DEGs with different expression levels indicated that pathways involving lipid and cholesterol metabolism were more upregulated in T + S treatment compared to TCDD alone (Supplementary Table 4). This is consistent with our previous simvastatin experiment where T + S co-treated mice had significantly reduced fat content in the liver^12^. DAVID analysis of the genes observed only in the TCDD or the T + S-treated groups indicated that there were also unique DEGs in both groups that were related to the lipid metabolism (Supplementary Table 5). In the TCDD-treated group, genes in this cluster, such as *Elovl6*, were downregulated (Fig. 4C). Notably, *Hmgcs1* and *Srebf2* are also represented in this cluster, which supports the importance of cholesterol homeostasis in TCDD-induced changes to metabolism (Fig. 4C). In the co-treated group, genes in the lipid metabolism cluster, such as *Cyp4a10* or *Lipg*, were upregulated (Fig. 4D). Notably, statins have been shown to activate peroxisome proliferator-activated receptors (PPARs), including PPARα, which is a major regulator of lipid metabolism in the liver^26–28^. These unique genes in this cluster include known PPARα target genes, *Cyp4a10*, *Acsl4* and *Lipg,* suggesting that PPARα is activated in co-treatment^26^. These genes are involved in processes such as ω-hydroxylation and β-oxidation of fatty acids^26,27^. In contrast, unique DEGs involved in lipid metabolism in TCDD-treated mice, such as *Elovl6* and *Apoc2*, are also PPARα target genes, but were downregulated^26,27^. Additionally, co-treatment also had a significant cluster associated with peroxisome-related genes, which are involved in long-chain fatty acid metabolism (Supplementary Table 5). The non-overlapping genes associated with the lipid metabolism clusters in both groups as well as the changes in expression of shared DEGs involving lipid metabolism between treatment groups may play an important role in differences previously seen in fat accumulation (Fig. 4B–D)^12^. For example, PPARα agonists have been suggested as potential therapeutic options for NAFLD to decrease steatosis^28^. Therefore, it’s possible that the activation of PPARα by statins in co-treatment may be protective against hepatocyte injury that was seen in most, but not all, of the T + S-treated mice as this could lead to less fat accumulation similar to what was observed in the previous study (Fig. 1B)^12^.

However, there could also be increased stress in other pathways that could be influencing systemic toxicity in the co-treated group. For example, there was enhanced expression of genes related to endoplasmic reticulum (ER) stress in the non-overlapping DEGs for co-treated mice (Supplementary Table 5). Some examples include *Sec16b*, an ER-stress inducible gene; *Mcfd2*, which accumulates in response to cell stress; and *Dnml1*, which has been shown to play a role in ER-stress-induced apoptosis^25,29–31^. ER stress can impact hepatocyte injury and can impact cholesterol homeostasis in hepatocytes, that would ultimately influence systemic metabolic homeostasis^32,33^.

### Comparing TCDD and TCDD + simvastatin changes in immune cells

**Figure 5:**
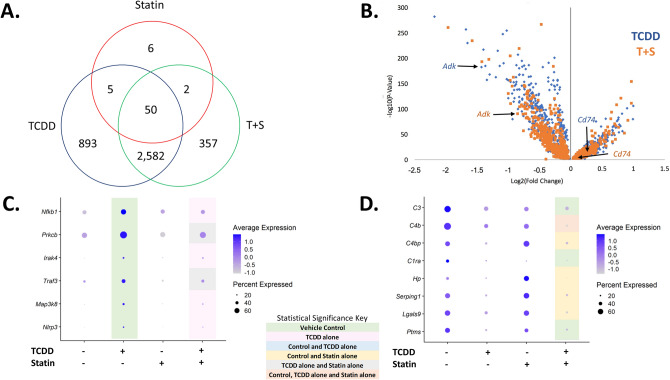
Simvastatin co-treatment impacts immune cells differently. (**A**) Venn diagram of the statistically significant (*P* ≤ 0.05) differentially expressed genes (DEGs) shared between treatment groups in macrophages. (**B**) Volcano plot representing the significant DEGs compared to control that were shared between TCDD and T + S treatments where the fold changes for both treatments were also significantly different from each other. The plot shows the log_2_ fold change of these DEGs for both TCDD (blue) and T + S (orange) corresponding to their respective p-values. The gene list is available in supplementary table 7. (**C**) Dot plot of genes associated with Nf-κB signaling in macrophages in TCDD-treated mice. The significant clusters generated by DAVID for these mice are listed in supplementary table 9. (**D**) Dot plot of genes in the immune pathway cluster in T + S-treated mice for liver dendritic cells. The significant clusters generated by DAVID for these mice are listed in supplementary table 10. Colors indicate statistically significant (*P* ≤ 0.05) differences in expression between treatment groups: green (vehicle); gray (TCDD alone and statin alone) and pink (TCDD alone). N = 3 mice per treatment group.

Macrophages are involved in the control of inflammatory responses in NAFLD. Hepatic lipid accumulation leading to lipotoxicity triggers Kupffer cell infiltration and subsequent pro-inflammatory responses^34^. The pro-inflammatory microenvironment produces further hepatocyte damage and liver injury^34^. Although the relative proportion of macrophages was not different between TCDD alone and co-treatment, statins tended to limit immune cell recruitment or proliferation (Fig. 2B). The majority of DEGs in macrophages were the same between treatment groups compared to control; however, a portion of these shared DEGs had expression that was different when comparing co-treatment to TCDD alone including genes that encode proteins involved in immune signaling like *Cd74* or *Adk* (Fig. 5A–B). Analysis of this group of genes indicated that immune system signaling was more repressed following co-treatment compared to TCDD alone (Supplementary Table 8). In contrast, the unique DEGs in the TCDD alone group associated with the immune response were upregulated (Supplementary Table 9). Interestingly, 6 genes in the immunity cluster generated by the DAVID database were all associated with activation of the NF-κB signaling pathway including *Prkcb, Nfkb1* and *Nlrp3* (Fig. 5C). NF-κB signaling in macrophages may play an important role in TCDD-induced changes in metabolism via NLRP3 inflammasome activation which has been associated with the progression of steatosis to steatohepatitis^32,35^.

A particularly interesting result of this study was that the proportion of liver dendritic cells significantly increased only in T + S-treated mice. Hepatic dendritic cells act as a bridge between the innate and adaptive immune process^36^. They can act as either perpetuators of inflammation in chronic inflammatory states, or interestingly, they can act as protective cells and avoid triggering inflammatory cascades^36^. DAVID analysis of non-overlapping DEGs in liver dendritic cells indicated that immune response, particularly regarding the complement system, was downregulated in co-treatment (Supplementary Table 10). Complement system activation recruits inflammatory cells, so repression of this pathway may be important for the differences seen in the proportion of immune cell types between TCDD alone and co-treatment (Fig. 5D).

### AHR activation and hepatic signatures of muscle wasting

**Figure 6:**
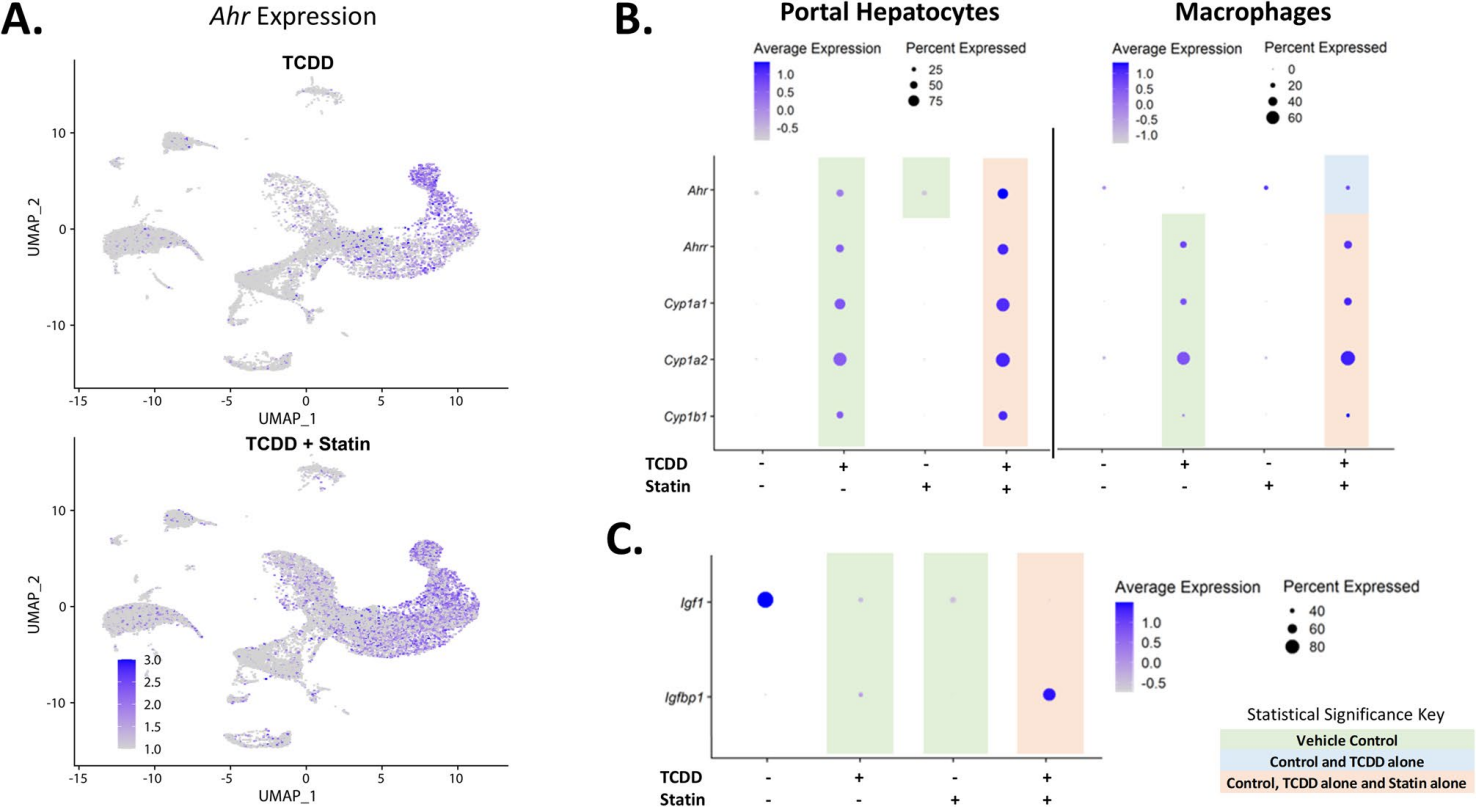
Simvastatin co-treatment induces AHR activity. (**A**) UMAP visualization of *Ahr* expression in all nuclei for TCDD and T + S-treated mice. (**B**) Dot plot of AHR and AHR target gene expression in portal hepatocytes and macrophages. Gene expression in other liver cell (sub)types is shown in supplementary table 11. (**C**) Dot plot of gene expression of GH/IGF-1 axis marker genes in portal hepatocytes. Colors indicate statistical significance (*P* ≤ 0.05) in expression between treatment groups: green (vehicle); blue (vehicle and TCDD alone); and orange (vehicle, TCDD alone and statin alone). N = 3 mice per treatment group.

**Figure 7:**
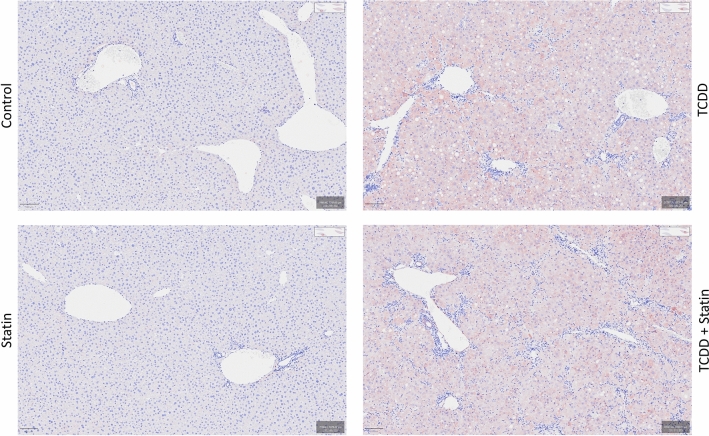
Immunostaining of CYP1A1 in mouse liver. Representative immunohistochemistry staining in mouse liver tissue using a human CYP1A1-specific antibody. Scale bar = 100 µm Images were taken from scanned slides using the QuPath software^56^.

Given the essential role that AHR activation plays in TCDD-induced injury, the impact of simvastatin co-treatment on AHR activation was explored. *Ahr* expression is highest in hepatocytes compared to other liver cell (sub)types, and interestingly, its expression is significantly higher in T + S treated mice compared to TCDD alone in portal hepatocytes (Fig. 6A and B). The increase *in Ahr* expression also correlates with increased AHR-mediated gene expression, such as *Cyp1a1* and *Cyp1a2* (Fig. 6B). This trend was also seen in Kupffer cells, but to a lesser degree (Fig. 6B). Although *Ahr* expression is smaller in other liver cell (sub)types, there was a general trend of increased AHR-mediated gene expression in T + S-treated mice compared to TCDD alone across multiple (sub)types (Supplementary Table 7). Notably, *Ahr* expression was significantly higher in T + S-treated mice compared to TCDD alone in B cells, T cells, liver dendritic cells and endothelial cells of the hepatic sinusoid (Supplementary Table 7). This co-treatment induced increase in CYP1A1 was verified using immunohistochemistry (IHC) (Fig. 7). Co-treated mice had more CYP1A1 staining compared to TCDD alone supporting the results seen at the gene expression level. Consequently, increased AHR activity may be involved in the worse prognosis seen in co-treated mice.

Simvastatin is also linked to muscle pain and muscle damage at high doses or when taken in combination with other drugs^37^. Therefore, we investigated hepatic gene signatures for mechanisms that link AHR activation by TCDD and perturbation by simvastatin to wasting such as the growth hormone (GH)/insulin-like growth factor (IGF)-1 axis. Hepatic steatosis-related alternations in the GH/IGF-1 axis is associated with decreased muscle myofibrillar protein content and muscle strength^38^. Lower IGF1 levels are reported in NAFLD patients, and the liver is the primary organ that contributes to plasma IGF1 concentration^38,39^. *Igf1* expression significantly decreased in T + S mice compared to TCDD alone in portal hepatocytes (Fig. 6C). A decrease in *Igf1* was also seen in simvastatin alone mice, which corresponds to previous reports^40^. Moreover, overexpression of IGFBP1, a modulator of IGF1 action, is associated with hepatic ER stress, hyperinsulinemia and glucose intolerance^38,41^. *Igfbp1* levels increased in T + S mice compared to TCDD alone (Fig. 6B) in portal hepatocytes. *Igfbp1* expression has been shown to be upregulated by the AHR in both humans and mice, so an increase in AHR activation in co-treated mice may explain the difference in expression between TCDD and co-treatment^42,43^. While statins have been shown to have no effect on IGFBP-1, there was a small (*P* = 0.04) decrease in expression in the simvastatin alone group compared to control in our study^40^. While these changes in expression are not huge in the liver, skeletal muscle will be examined in future experiments to determine if this possible mechanism is important for the wasting seen in co-treatment.

## Discussion

Many studies suggest that simvastatin may be protective against NAFLD. Our studies, however, suggest that simvastatin exacerbates aspects of TCDD-induced injury. Co-treatment with simvastatin impacted pathways involved in liver metabolism differently, which may explain differences in pathology between TCDD and co-treated mice. For example, we had previously reported that simvastatin co-treatment decreased hepatic fat accumulation^12^. Portal hepatocyte snRNAseq signatures suggest that the differential expression of key genes encoding proteins important for lipid homeostasis, such as the induction of PPARα target genes like *Cyp4a10* only seen in T + S-treated mice, may play a role in these differences in fat content.

An important observation in this study was that co-treatment led to wasting and caused the death of 2 mice compared to TCDD alone. However, there was not a significant increase in liver injury with co-treatment, likely due to following a different treatment paradigm in this study compared to our previous one^12^. In this study, mice underwent systemic exposure to TCDD treatment over the course of 28 days. While the liver is a key organ impacted by treatment, increased wasting and lethality in co-treated mice are likely due to systemic effects that may also involve other tissues. Therefore, hepatic snRNAseq results may not solely explain the T + S pathology. However, co-treatment may alter the GH/IGF-1 axis, which may impact the liver with subsequent effects on muscle protein and strength. Specifically, differences in hepatic *Igf1* and *Igfbp1* expression between TCDD and co-treatment suggests decreased IGF1 signaling may exacerbate muscle wasting, which can be explored in future studies by looking beyond the liver.

Simvastatin co-treatment also exhibited differences in immune cell infiltration evidenced by a decrease in immune cell populations and differences in pro-inflammatory pathways in macrophages compared to TCDD alone. TCDD-induced macrophage gene expression associated with the NF-κB pathway that was not present with T + S treatment. NF-κB signaling plays an important role in chronic liver injury. Upon steatosis-induced hepatocyte injury, factors such as IL-1α stimulate NF-κB activation leading to the release of pro-inflammatory mediators that further injure hepatocytes^44^. Therefore, the ability of simvastatin to partially inhibit this TCDD-induced upregulation of NF-κB-related genes could lead to a weaker inflammatory response in co-treatment. While immune cell infiltration decreased with co-treatment, the liver dendritic cell population increased. An increase in plasmacytoid dendritic cells (pDCs), for example, is associated with protection against immune-mediated acute liver injury in humans and mice^45^. Furthermore, non-overlapping DEGs in co-treated mice suggest a decrease in inflammatory pathways, particularly with the complement system. This includes *C1ra* and *Serping1*, which are involved in the C1 complement component; *C3*, which encodes the C3 complement component; and *C4b* and *C4bp*, which are involved in the C4 complement component. Complement components are responsible for the recruitment of inflammatory cells such as macrophages. Both the classical and lectin pathways are activated in NAFLD patients with severity correlated with accumulation of several components of both pathways such as C4d^46^. It is possible that liver dendritic cell recruitment with co-treatment plays an important role in decreasing macrophage recruitment by inhibiting the complement system. Therefore, the combination of liver dendritic cell recruitment and inhibition of TCDD-induced NF-κB pathway activation in Kupffer cells may explain the differences in immune cell infiltration between treatment groups, which may influence AHR-mediated changes in liver and systemic metabolism.

In conclusion, this study supports our previous experiment indicating that simvastatin co-treatment promotes a worse prognosis of TCDD-induced injury in mice. Overall, the snRNAseq results provided more insight on how simvastatin co-treatment impacts distinct liver cell populations, which may influence the extent of TCDD-induced changes in metabolism. Furthermore, this study suggests that liver dendritic cells may play an important mechanistic role in immune infiltration in liver injury. The role of liver dendritic cells in steatohepatitis progression remains unclear, so it is important to better understand their role in future studies.

## Materials and methods

### TCDD and statin mouse exposure

All animal experiments were approved by Michigan State University’s Institutional Animal Care & Use Committee and were carried out in accordance with this approval and all relevant guidelines and regulations, including ARRIVE^47^. Male C57BL/6NCrl mice were ordered from Charles River Laboratories and were delivered to Michigan State University on postnatal day 25 (PND25). Mice were acclimated for 7 days prior to treatment. Mice were housed in Innocages (Innovive, San Diego, CA) with ALPHA-Dri bedding (Shepherd Specialty Papers, Chicago, IL) under constant 12-h light/dark cycles, temperature, and humidity. Upon arrival, mice were randomly placed into a treatment group: (vehicle control) sesame oil (Sigma Aldrich) with standard mouse chow (Harlan Teklad Rodent Diet 8940); (TCDD alone) TCDD (30 µg/kg body weight every 4 days for 28 days for 7 total treatments; AccuStandard, New Haven, CT) with standard mouse chow; (simvastatin alone) sesame oil with chow containing simvastatin (500 mg/kg/food, Harlan Teklad Rodent Diet 8940; Sigma Aldrich, St. Louis, MO); or (TCDD and simvastatin) 30 µg/kg TCDD every 4 days for 28 days with chow containing 500 mg/kg simvastatin. Repeated dosing with 30 µg/kg TCDD using this paradigm has been shown to elicit steatohepatitis with mild fibrosis and negligible necrosis/apoptosis in male mice previously^4,22,23^. Furthermore, this dose was chosen to compensate for the differences in TCDD half-life between humans (1–11 years) and mice (8–12 days), the short duration of the study compared to lifelong cumulative human exposure to diverse AHR ligands and the bioaccumulative nature of AHR ligands^48–51^. Furthermore, the level of TCDD in mouse liver using this paradigm approximates levels of TCDD reported in human serum^4^. The simvastatin-laced chow was prepared at Envigo (Huntington, UK). Mice were acclimated to the simvastatin-laced chow for 3 days prior to treatment with TCDD. Chow was provided ad libitum for each treatment group and food consumption was measured daily. Each treatment group had a sample size of at least 6 mice. Group D started with 7 mice and ended up with 5 due to 2 mice dying by the end of the study (Supplementary Fig. 1). The average dose of simvastatin (mg/kg body weight/day) for the mice over the 32-day period was calculated to be 76.4 ± 3.1 and 85.9 ± 3.5 for statin alone and co-treatment, respectively, estimated from the daily chow consumption per cage (Supplementary Table 1). TCDD did not impact consumption of simvastatin-laced chow. Although this dose range in mice corresponds to approximately 350 mg/day in humans, simvastatin is 5–8 times less efficacious in mice in comparison^52^. Therefore, the effective dose used in this study is within range of what is relevant in humans with moderate to severe hypercholesterolemia. On day 32, the mice were sacrificed following a 6 h fasting period. Tissue samples were frozen in liquid nitrogen or fixed in formalin for histopathology.

### Serum clinical chemistry

Serum alanine aminotransferase (ALT, n ≥ 5) was measured spectrophotometrically (SpectraMax, Molecular Devices, San Jose, CA) using commercially available reagents (FUJIFILM Wako Diagnostics, Richmond, VA).

### Histology

Formalin-fixed liver was vacuum infiltrated with paraffin using a Tissue-Tek VIP 2000 and embedded with the HistoCentre III embedding station (Thermo Fisher, Waltham, MA). A Reichert Jung 2030 rotary microtome (Reichert, Depew NY) was used to prepare 4–5 µm liver sections that were stained with hematoxylin and eosin (H&E). Histological severity scoring of H&E stained liver sections was performed by a certified veterinary pathologist using the following criteria: 1, minimal, less than 25% of tissue; 2, mild, 25% to less than 50%; 3, moderate, 50% to less than 75%; 4, marked, 75–100%. Immunohistochemistry staining for CYP1A1 (sc-25304, 1:50; Santa Cruz Biotechnology, Dallas, TX) was also performed using liver sections from the paraffin-embedded tissue via TRIS/EDTA retrieval at pH 9.0. All paraffin-embedding and staining was performed by the Michigan State University Investigative Histopathology Laboratory.

### Nuclei isolation

Nuclei were isolated from frozen liver samples (n = 3) as previously described [22; https://doi.org/10.17504/protocols.io.3fkgjkw]. Briefly, livers were diced in EZ Lysis Buffer (Sigma-Aldrich, St. Louis, MO), homogenized using a disposable Dounce homogenizer, and incubated on ice for 5 min. The homogenate was filtered, transferred to a microcentrifuge tube and nuclei were isolated by centrifugation (500 × g and 4 °C, 5 min). The supernatant was removed and nuclei were resuspended in fresh EZ Lysis Buffer and incubated on ice (5 min.) ice followed by centrifugation. The nuclei pellet was washed twice with nuclei wash and resuspend buffer (1 × phosphate-buffered saline, 1% bovine serum albumin, 0.2 U/µL RNAse inhibitor) with 5 min incubations on ice. After washing, the pellet was resuspended in nuclei wash and resuspend buffer containing DAPI (10 µg/mL). The nuclei were filtered (40 µm) and underwent fluorescence-activated cell sorting using a BD FAC-Saria Ilu (BD Biosciences, San Jose, CA) with a 70 µm nozzle at the MSU Pharmacology and Toxicology Flow Cytometry Core.

### Single-nuclei RNA sequencing (snRNASeq)

Nuclei were immediately processed for snRNASeq, which was performed as previously described^22^. Briefly, 10 × Genomics Chromium Single Cell 3′ v3 libraries were submitted for 150-bp paired-end sequencing at a depth ≥ 60,000 reads/cell using a NovaSeq 6000 System (Illumina, San Diego, CA) at the MSU Research Technology Support Facility. Following sequencing quality control, Cell Ranger v3.0.2 (10× Genomics) was used to align reads to a custom reference genome (mouse mm 10 release 93 genome build) which included introns and exons to consider pre-mRNA and mature mRNA. Raw counts were further analyzed on Seurat v.3.1.1^53^. Each sample was filtered for genes expressed in at least 3 nuclei, nuclei that expressed at least 100 genes and ≤ 1% mitochondrial genes. Clustering of nuclei was performed using Seurat integration tools at a resolution of 0.05 and were annotated using a semiautomated strategy. Marker genes for individual nuclei clusters were also manually examined to verify annotation. Raw and processed data have been deposited in the Gene Expression Omnibus (GEO) with accession ID GSE211018 following the Minimum Information about Animal Toxicology Experiments (MIATE; https://doi.org/10.25504/FAIRsharing.wYScsE).

### DAVID

Functional analysis of differentially expressed genes (DEGs) from identified cell types was assessed using the Database for Annotation, Visualization and Integrated Discovery tool (DAVID, v. 6.8)^54,55^. DEGs with log_2_ fold changes ≥ 0.10 or ≤ − 0.10 were used to have enough genes to create clusters. Enrichment scores higher than 1.3 were considered significant.

### Statistical analysis

All analyses except for snRNAseq was performed using Astatsa. Differences were assessed with one-way analysis of variance (ANOVA) and Tukey’s pair-wise *posthoc* test with *P* ≤ 0.05 considered significant. Potential outliers were determined using Grubb’s test.

### Supplementary Information

Supplementary Information.

## Data Availability

The datasets generated during and/or analyzed during the current study are available in Gene Expression Omnibus (GEO) with accession ID GSE211018 following the Minimum Information about Animal Toxicology Experiments (MIATE; https://doi.org/10.25504/FAIRsharing.wYScsE).
