# Supplementary material for: Characterizing the impact of simvastatin co-treatment of cell specific TCDD-induced gene expression and systemic toxicity
Source: Sci Rep. 2023 Oct 3;13:16598. doi: 10.1038/s41598-023-42972-8 (PMC10547718; doi:10.1038/s41598-023-42972-8)
Supplement: Supplementary file 1 — Supplementary Information. [file 41598_2023_42972_MOESM1_ESM.pdf]

Supplementary Figures / Tables

Figure 1: Survival plot for mice over the course of the experiment. Plot indicates the percent survival of the mice in each treatment group over the course of the study.

Table 1: Food consumption measurements for mice given statin chow throughout the course of the experiment.

Table 2: All DEGs where at least 1 treatment group had expression that was significantly different from vehicle control in portal hepatocytes.

Table 3: Significant DEGs compared to control that were shared by TCDD and T+S-treated mice that were also significantly different from each other used to make the volcano plot in figure 4B.

Table 4: The top 25 significant DAVID clusters generated from DEGs with a log_2 fold change difference between T+S and TCDD ≥ 0.1 or ≤ -0.1 in portal hepatocytes.

Table 5: DAVID clusters generated from the non-overlapping DEGs in portal hepatocytes in TCDD and T+S-treated mice with a fold change cut off of 0.1 (upregulated genes) or -0.1 (downregulated genes). Only significant clusters, with enrichment scores of 1.3 or greater, are shown.

Table 6: All DEGs where at least 1 treatment group had expression that was significantly different from vehicle control in macrophages.

Table 7: Significant DEGs compared to control that were shared by TCDD and T+S-treated mice that were also significantly different from each other used to make the volcano plot in figure 5B.

Table 8: The top 25 significant DAVID clusters generated from DEGs with a log2 fold change difference between T+S and TCDD ≥ 0.1 or ≤ -0.1 in macrophages from the volcano plot in Figure 5B.

Table 9: DAVID clusters generated from the non-overlapping DEGs in macrophages in TCDD and T+S-treated mice with a fold change cut off of 0.1 (upregulated genes) or -0.1 (downregulated genes). Only significant clusters, with enrichment scores of 1.3 or greater, are shown.

Table 10: DAVID clusters generated from the non-overlapping DEGs in liver dendritic cells in T+S-treated mice compared to TCDD alone with a fold change cut off of 0.1 (upregulated genes) or -0.1 (downregulated genes). Only significant clusters, with enrichment scores of 1.3 or greater, are shown.

Table 11: AHR and AHR target gene expression in the liver cell (sub)types not shown in Figure 6. Colors indicate statistical significance ($P \leq 0.05$) in expression between treatment groups: green (vehicle) and blue (vehicle and TCDD alone).

Supplemental Figure 1

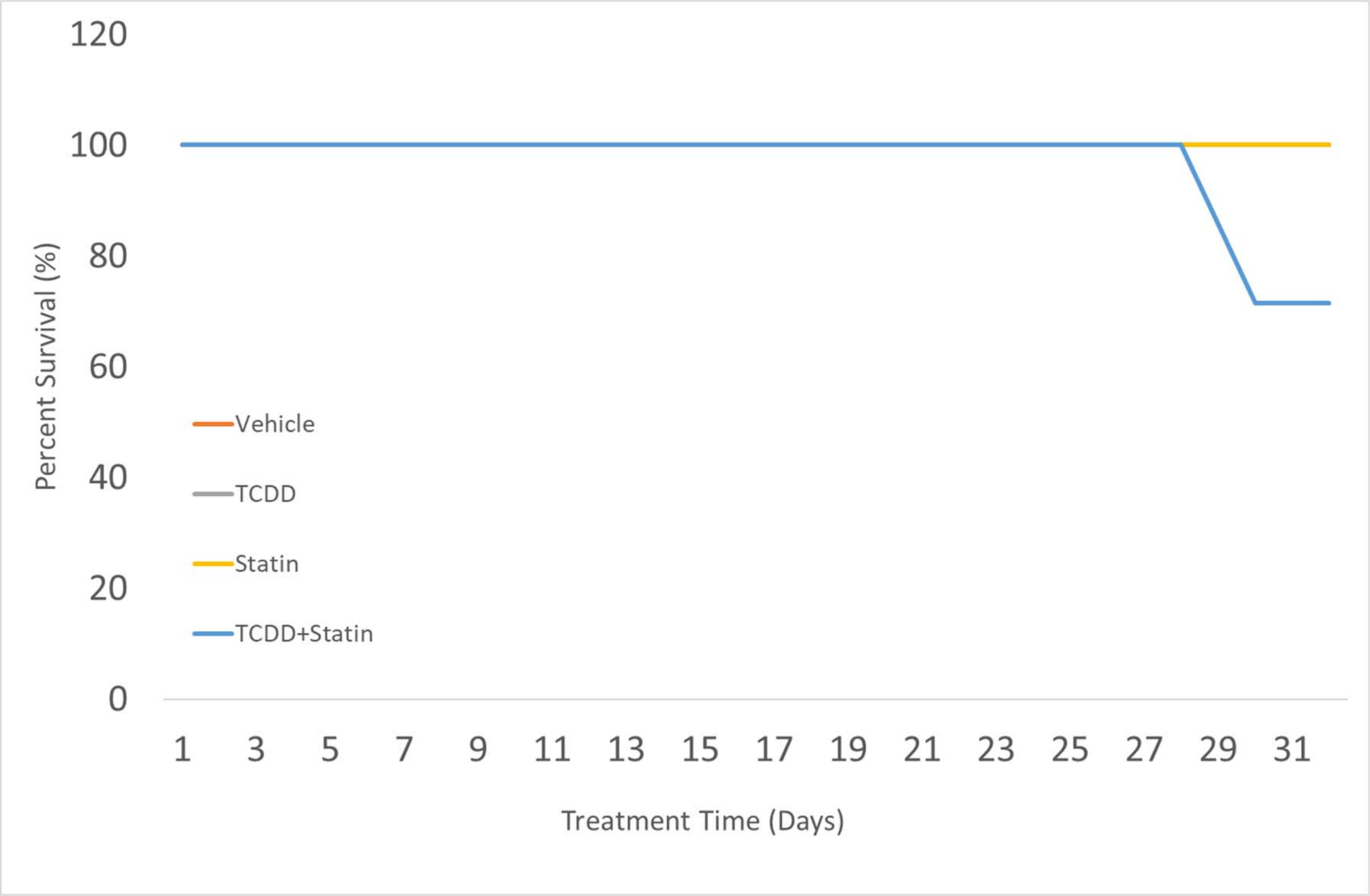

	TCDD (ug/l	Cage #	16-Jun	17-Jun	18-Jun	19-Jun	20-Jun	21-Jun	22-Jun	23-Jun	24-Jun	25-Jun	26-Jun	27-Jun	28-Jun	29-Jun	30-Jun	1-Jul
Average F	0	5		-5.5	-7.7	-9.2	-6.5	-8.6	-8.7	-10.3	-8.7	-12.2	-11.9	-9.5	-9.7	-9.4	-9.4	-10.4
Average V	0	5	11.88233	12.832	13.66933	14.54533	15.04733	16.10833	16.696	17.48833	17.67867	18.70433	19.10033	19.87767	19.668	19.89233	20.077	20.223
Dose of St	0	5		71.43599	93.88412	105.4175	71.99504	88.98086	86.84715	98.16068	82.01976	108.7092	103.8376	79.65388	82.19782	78.75731	78.03291	85.71099
Average F	0	6		-5.5	-7.3	-10.2	-6.2	-8.6	-9.7	-10.6	-8.4	-12.9	-12.1	-10.3	-11.6	-9.9	-10.7	-11.6
Average V	0	6	12.48267	13.41267	14.332	15.122	15.682	16.71367	17.501	18.05333	18.08233	19.04033	19.36667	19.821	19.466	19.72233	19.94133	20.23633
Dose of St	0	6		68.34336	84.89162	112.419	65.89296	85.75816	92.37567	97.8582	77.42364	112.9182	104.1308	86.60848	99.31847	83.6615	89.42899	95.53773
Average F	30	7		-6.6	-9	-10.2	-5.8	-9.2	-8.9	-10.2	-9.3	-12.1	-11.2	-9.5	-9.7	-8.6	-9.9	-10.5
Average V	30	7	11.345	13.305	14.142	14.81467	14.87233	15.65	16.24267	16.97467	17.148	17.684	18.01733	18.206	17.999	17.80633	17.65	17.38667
Dose of St	30	7		82.67569	106.067	114.7511	64.99765	97.97657	91.32326	100.1492	90.38955	114.0391	103.6039	86.96767	89.8198	80.4957	93.48442	100.6518
Average F	30	8		-8	-12	-12.8	-8.3	-12	-12.1	-14.2	-11.8	-16.2	-15.4	-13.6	-12.9	-12.7	-13.3	-13.8
Average V	30	8	12.553	13.696	14.763	15.41175	15.786	16.62675	17.084	17.873	17.90225	18.55675	18.8625	18.738	18.60225	18.518	18.35475	18.0215
Dose of St	30	8		73.01402	101.6054	103.8169	65.72279	90.21607	88.53313	99.31181	82.39188	109.1247	102.0543	90.72473	86.68306	85.7274	90.57601	95.719

	TCDD (ug/l	Cage #	2-Jul	3-Jul	4-Jul	5-Jul	6-Jul	7-Jul	8-Jul	9-Jul	10-Jul	11-Jul	12-Jul	13-Jul	14-Jul	15-Jul	16-Jul	17-Jul	Avg Dos	Std Deviation
Average F	0	5	-9.2	-10.5	-9.7	-9.2	-8.8	-9.7	-8.4	-9.6	-10.1				-7.5	-8.7	-10.2	-7.3		
Average V	0	5	20.271	20.98033	21.22867	20.186	21.46067	21.705	21.69333	21.94967	22.08467				22.44633	23.06633	22.95467	22.01067		
Dose of St	0	5	75.64172	83.41145	76.15488	75.96024	68.34208	74.48361	64.53596	72.89405	76.22181				55.68838	62.86218	74.05901	55.27623	74.53908	23.53776
Average F	0	6	-9.6	-9.9	-9.4	-12.1	-10	-11.1	-8.8	-9.9	-10.7				-8.5	-9.1	-9.8	-7.4		
Average V	0	6	20.45167	20.496	20.65067	20.88867	21.23233	21.448	21.39533	21.46767	21.30533				21.85933	22.29367	22.526	21.61567		
Dose of St	0	6	78.23323	80.50351	75.86519	96.54358	78.49663	86.25513	68.55077	76.85977	83.70361		No data for these days		64.80832	68.03128	72.5088	57.05738	78.3328	24.62654
Average F	30	7	-9.3	-10.3	-9.3	-12.5	-7.5	-10.6	-9.8	-9.4	-9.6				-6.3	-7.6	-7.5	-7.9		
Average V	30	7	17.39333	17.28467	17.09833	16.901	16.686	17.03	16.90167	16.63767	16.56733				15.856	15.98667	15.742	15.401		
Dose of St	30	7	89.1146	99.31731	90.65211	123.2669	74.9131	103.7385	96.63741	94.16385	96.57559				66.22099	79.23269	79.40541	85.49228	87.77078	23.17529
Average F	30	8	-13	-15.9	-15.3	-16	-12.5	-22.4	-1.1	-8.5	-10.2				-6.5	-6.1	-4.8	-5.3		
Average V	30	8	17.9645	18.06525	18.07575	17.86	17.4055	17.2025	16.8155	16.938	16.76775				#REF!	#REF!	#REF!	#REF!		
Dose of St	30	8	90.45618	110.0179	105.8047	111.9821	89.77047	162.767	8.17698	62.72878	76.03882				#REF!	#REF!	#REF!	#REF!	#REF!	#REF!

Gene	V_C_vs_adj_V_S	V_C_vs_adj_T_C	V_C_vs_adj_T_	V_C_vs_calculatedFCV_S	V_C_vs_calculatedFCT_C	V_C_vs_calculatedFCT_S
Fmo3	0.210936295	0.00E+00	0	-0.007661757	1.995998944	2.863612515
Reln	0.452639163	0.00E+00	0	0.015746907	2.120244598	2.3827768
Cdh18	0.001549696	0.00E+00	0	-0.064207019	2.307919831	2.367973087
Airn	1	0.00E+00	0	0.002837676	1.656621115	2.321610195
Cyp1a2	0.122930765	0.00E+00	0	-0.009310344	1.599452186	1.992748367
Nrg1	1	0.00E+00	0	0.002264112	1.490354057	1.955485419
Selenbp1	2.70761E-80	0.00E+00	0	0.107733795	1.348605909	1.874285111
Cyp2b9	2.79829E-21	0.00E+00	0	0.013931491	0.753667785	1.873966403
Esrrg	1	0.00E+00	0	-0.023301781	1.590254035	1.809250424
Cyp1a1	1	0.00E+00	0	-0.001025782	1.102878726	1.404616481
Nfe2l2	0.699525723	0.00E+00	0	-0.028625005	0.965370286	1.380085159
Por	0.002407833	0.00E+00	0	0.042709059	1.056808985	1.364840309
Samd4	1	0.00E+00	0	-0.001817945	1.068456151	1.278797615
Ugdh	1	0.00E+00	0	-0.011311537	0.999622163	1.254966798
Them7	7.4026E-22	0.00E+00	0	-0.055506436	0.935453614	1.240965958
Tiparp	1	0.00E+00	0	0.003312166	0.950407836	1.237608604
Tbc1d8	4.9976E-165	0.00E+00	0	0.169545042	0.964310711	1.225917137
Abcc4	1	0.00E+00	0	6.15262E-05	1.025759167	1.149359936
Myom1	0.000106382	0.00E+00	0	0.012281263	0.875006138	1.118367308
Dst	6.0604E-16	0.00E+00	0	0.059055064	0.806723471	1.098096199
Eif4g3	1.59554E-10	0.00E+00	0	0.045149944	0.836641617	1.057772207
Gclc	1	0.00E+00	0	-0.032136435	0.861738163	1.056958452
Robo1	1	0.00E+00	0	0.014541819	0.812385832	1.048652463
Ctdspl	6.20771E-32	0.00E+00	0	-0.031781384	0.48329705	1.032677747
Nrip1	1.06035E-44	0.00E+00	0	-0.084886145	0.795856138	1.029476374
Hjurp	2.42955E-13	0.00E+00	0	0.048535286	0.628917864	1.007919994
Gm20528	1.1715E-52	0.00E+00	0	0.093461641	1.111783404	0.998794767
Msrb3	1	0.00E+00	0	-0.00566475	0.675778172	0.988869359
Htatip2	1.0613E-40	0.00E+00	0	0.052180703	0.748587078	0.979439095
Pvt1	7.1886E-07	0.00E+00	0	-0.022016714	1.072733929	0.954594595
Zfhx3	1	0.00E+00	0	-0.016563423	0.691869788	0.92713921
Chd9	1	3.34E-230	0	0.01038088	0.58207716	0.912809656
Lpin2	0	0.00E+00	0	0.339960852	0.545018066	0.903271442
Prkca	1	0.00E+00	0	0.023240261	0.636242076	0.897328839
Kcnq1ot1	1.68546E-44	0.00E+00	0	0.099147381	0.534926109	0.89294221
Cyb5a	4.9133E-264	0.00E+00	0	0.248438327	0.551304806	0.882993658
Ahrr	1	0.00E+00	0	5.73198E-05	0.633342544	0.882620893
Itih2	1	1.57E-09	0	-0.023398215	-0.045057585	0.865108629
Adamts17	1	0.00E+00	0	-0.001578609	0.571825862	0.860485313
Cyp2a5	1	0.00E+00	0	-0.001293408	0.687572681	0.849246557
Wipf3	1	0.00E+00	0	-0.003804789	0.616543844	0.847158424
Tanc1	0.003680588	0.00E+00	0	-0.020133402	0.648631961	0.845986394
Pik3ap1	6.3187E-108	0.00E+00	0	0.147261867	0.694917366	0.844455334
Adh1	3.10001E-11	0.00E+00	0	-0.056603878	0.474962751	0.835190762
Gramd1c	9.25893E-10	3.73E-251	0	-0.051375141	0.424149337	0.834632009

Fabp12	2.23178E-23	0.00E+00	0	-0.011050653	0.496180714	0.815059062
Abhd6	1	0.00E+00	0	-8.53835E-05	0.572800666	0.814255045
Nipal2	1	0.00E+00	0	-0.000514557	0.620549437	0.807222473
Aox1	2.14008E-17	3.94E-299	0	-0.068336635	0.514634466	0.803935864
Glis3	1	0.00E+00	0	0.003706256	0.612401048	0.790126246
Ppard	5.0122E-61	0.00E+00	0	0.084001436	0.540847499	0.77816139
Tbc1d16	0.000114478	0.00E+00	0	0.027346498	0.417311046	0.76730069
Tbcel	1	0.00E+00	0	0.014461733	0.654364522	0.762065452
Lmo7	1	1.50E-267	0	-0.00650245	0.362375307	0.761007695
Gm43305	2.40444E-05	0.00E+00	0	0.049409548	0.706950902	0.75239693
Slc35d1	1.40522E-45	0.00E+00	0	-0.083074908	0.73249379	0.748811451
Zfp704	5.23703E-20	0.00E+00	0	-0.037658971	0.492980038	0.748015138
Gstm1	1	0.00E+00	0	-0.012847899	0.733139561	0.747376717
Npc1	1.30785E-19	0.00E+00	0	0.056281097	0.563841781	0.739661101
Zbtb16	0	9.51E-66	0	0.864835008	0.322362873	0.73898163
Hpgd	8.71026E-58	0.00E+00	0	0.071931341	0.421428789	0.738832193
Hsd17b6	1	1.77E-188	0	0.0034415	0.475579588	0.732261411
Son	1.1532E-177	0.00E+00	0	0.158818497	0.402625514	0.731409751
Acacb	9.2507E-199	0.00E+00	0	0.154037771	0.512022047	0.721072729
Gstm3	1	0.00E+00	0	0.000592425	0.610491586	0.710221416
Abca8b	1	0.00E+00	0	0.001441291	0.670671103	0.706509902
St3gal1	1.89696E-81	7.39E-182	0	-0.137287301	0.3726701	0.705394275
Elovl5	1.92528E-18	2.06E-43	0	-0.119665889	0.189539208	0.694769779
Notch1	1	0.00E+00	0	-0.008832596	0.377410132	0.690664997
Cyfip2	1	0.00E+00	0	-0.001248376	0.482436443	0.688526748
Nup155	0.01558892	0.00E+00	0	0.018287722	0.369098725	0.688244233
Setbp1	1	0.00E+00	0	0.00302379	0.395391807	0.687789339
Magi3	3.54766E-06	1.15E-77	0	-0.044393298	0.264376457	0.674852676
Gm29685	1	0.00E+00	0	-0.000208697	0.635190099	0.674634685
Patj	1	0.00E+00	0	-0.017497208	0.479372682	0.669984424
Nlrp12	1.44873E-77	0.00E+00	0	0.11237794	0.455547018	0.669155464
Hacd3	1	0.00E+00	0	-0.016428497	0.49645258	0.666544625
Cd36	2.09793E-28	0.00E+00	0	0.025620522	0.729340583	0.666144042
Aig1	1.10098E-07	0.00E+00	0	-0.038066076	0.542168321	0.663439958
Ftl1	0.098672495	0.00E+00	0	0.025465387	0.747911796	0.660948274
Tafa2	6.13999E-37	1.20E-239	0	0.034478804	0.204764446	0.654776376
Ahr	2.92567E-25	4.08E-171	0	0.058458017	0.306998654	0.650751037
Bdh1	1.02688E-40	0.00E+00	0	0.085044441	0.535705996	0.644069399
Cldn14	2.35874E-16	0.00E+00	0	-0.023635824	0.407667322	0.644054378
Fbp1	0	3.80E-183	0	-0.456818335	0.436321651	0.642387021
Entpd5	1	0.00E+00	0	-0.017878653	0.556440558	0.641315143
Shroom3	3.73127E-83	2.19E-130	0	-0.15372468	0.384135019	0.638066852
Sgk1	1	0.00E+00	0	0.001830712	0.334724599	0.633247007
Mybl1	1	0.00E+00	0	0.000953806	0.383381196	0.63134743
2900026A(	0.000103126	4.11E-211	0	-0.036536823	0.38771071	0.631209916
Cyp1b1	1	0.00E+00	0	-0.000343953	0.460035444	0.628547411
Pdzrn3	6.00932E-06	6.66E-39	0	-0.043547798	0.22610093	0.628126729

Arid1b	9.04856E-79	3.71E-299	0	-0.114589258	0.409018423	0.622835902
Pitpnc1	3.29216E-09	2.90E-226	0	-0.055028579	0.456819584	0.622661915
Lrp4	1	0.00E+00	0	0.007464777	0.400239608	0.622302623
Abcc3	9.0907E-127	0.00E+00	0	0.164194756	0.479025023	0.621304365
Igfbp1	0.04004694	4.33E-69	0	-0.017026749	0.193588961	0.619311422
Mgll	2.25273E-11	2.70E-51	0	0.061995747	0.276112724	0.615752247
Cyp2c68	1	4.62E-52	0	-0.017150462	0.198383043	0.610588328
Abcb4	6.6059E-222	4.59E-160	0	0.185313853	0.292825558	0.608590555
Sult2a2	1	0.00E+00	0	-9.38186E-05	0.188754007	0.608169627
Txnip	2.5959E-129	0.00E+00	0	0.064163668	0.436470813	0.607810529
Gtf2i	6.37455E-07	4.60E-282	0	0.038503395	0.398843585	0.604006826
Hectd2os	1.2034E-114	0.00E+00	0	-0.094327427	0.49170665	0.590754374
Acyp2	1	0.00E+00	0	0.013328057	0.46113475	0.590534168
Xdh	1.23816E-54	0.00E+00	0	0.096433701	0.467016899	0.586446346
Pde4d	8.17781E-11	5.54E-149	0	-0.05216445	0.379271646	0.584466385
Gm42047	3.83838E-85	1.49E-74	0	0.09961299	0.283626877	0.584351224
Fth1	2.38443E-13	0.00E+00	0	0.022601727	0.62401196	0.581132656
Tmlhe	0.953309713	1.01E-269	0	-0.013966649	0.337480678	0.580258513
Eml4	1.01906E-05	2.86E-194	0	-0.028252193	0.28985815	0.575972099
Exoc3	1	0.00E+00	0	0.006950782	0.343393905	0.575710387
Sytl5	5.78321E-82	0.00E+00	0	0.048648252	0.366296447	0.574556363
Slc25a21	4.46994E-26	9.00E-88	0	-0.088597212	0.299361763	0.574546436
Ecpas	0.000167072	0.00E+00	0	0.031615042	0.464931384	0.574075082
Metap1d	1	0.00E+00	0	-0.000428289	0.410339081	0.573246
Rapgef4os1	7.15293E-78	0.00E+00	0	-0.047539381	0.490298844	0.572038455
Lrmda	1.24069E-25	0.00E+00	0	0.048053905	0.841669935	0.569554707
Fabp1	1	2.63E-222	0	-0.020094157	0.404397754	0.565743442
Slc12a6	4.87699E-08	0.00E+00	0	-0.031688651	0.533127051	0.562575562
Eif1a	4.42927E-27	0.00E+00	0	0.042892812	0.444915488	0.559500297
Furin	4.10188E-43	1.18E-134	0	0.066414938	0.356065943	0.557948724
Txnrd1	1.85743E-07	0.00E+00	0	-0.031876205	0.639586128	0.554545089
Arhgap10	1	0.00E+00	0	-0.007813602	0.696517705	0.553557508
Dpf3	3.03821E-08	0.00E+00	0	-0.027940074	0.39940836	0.553376904
Cdk6	2.16281E-29	0.00E+00	0	-0.069545943	0.413371923	0.552248247
Chrm3	3.245E-151	0.00E+00	0	0.121534657	0.365940305	0.551000391
Gm50020	1	0.00E+00	0	-0.000558534	0.591036447	0.545969244
Blvrb	1	0.00E+00	0	0.005096761	0.533805746	0.545347817
Igf2r	1	0.00E+00	0	0.010651724	0.364657889	0.54384065
Cdh22	1	0.00E+00	0	-0.000779754	0.371574567	0.543176358
D630045J1	1	0.00E+00	0	-0.005441182	0.419393054	0.541904032
Pdk4	9.92999E-23	0.00E+00	0	0.020271344	0.341295627	0.540577085
Etl4	2.86714E-32	2.03E-294	0	-0.074158312	0.45439309	0.539422491
Cers6	7.90646E-51	5.99E-252	0	0.099332269	0.400685193	0.539043591
Gm19619	1	0.00E+00	0	4.87411E-05	0.54815119	0.532172276
Ube2e2	0.552837413	1.03E-111	0	-0.030668179	0.349732131	0.527800469
Ism2	1	0.00E+00	0	-0.000972248	0.39103725	0.526676285
Pon3	1	7.48E-241	0	0.016477751	0.324122138	0.52596337

Slc8b1	1	0.00E+00	0	0.011311077	0.530474175	0.52466165
Gbe1	6.62894E-27	1.35E-203	0	0.07332278	0.489485309	0.521367974
Pawr	5.5085E-17	1.27E-174	0	0.050231719	0.346912099	0.519256403
Antxr2	7.63322E-20	3.47E-131	0	-0.044771192	0.265064517	0.517663105
Srgap2	3.77966E-15	0.00E+00	0	0.051978436	0.425780759	0.516076348
Gm37273	1	0.00E+00	0	-0.000439409	0.408998612	0.515734361
Mapre3	1	2.13E-230	0	0.019214906	0.402724237	0.510650319
Wwox	0	2.61E-07	0	-0.2712277	0.115116571	0.51017023
Osbpl3	0.617005476	0.00E+00	0	0.004155274	0.650514161	0.503810594
9130230L2	1.51971E-54	0.00E+00	0	-0.053224335	0.639017205	0.502828912
Taf1d	0.085027945	0.00E+00	0	0.026449594	0.447213878	0.50102568
Rpl10a	9.36119E-69	0.00E+00	0	0.055816778	0.410191871	0.499279953
Gsta3	8.114E-115	5.54E-131	0	0.193395606	0.348270432	0.498120665
Ces1f	8.85987E-06	2.95E-189	0	-0.038717189	0.354483132	0.490646284
Malat1	0	2.64E-132	0	0.250556309	0.170610792	0.486036674
Gstt2	8.33232E-36	7.08E-182	0	0.060077202	0.263062903	0.485823248
Mbnl2	1.24258E-08	3.65E-281	0	-0.049348173	0.496075235	0.48503058
Mast2	1	8.14E-223	0	0.00014797	0.337038403	0.480786757
Herc1	1.00035E-36	0.00E+00	0	0.075453197	0.405646847	0.474532645
Abca8a	1.70071E-93	3.19E-215	0	-0.144486647	0.437697554	0.471789643
Eya3	1	1.10E-178	0	0.013548369	0.309711289	0.469412507
Stard13	3.15525E-80	4.83E-69	0	-0.124183958	0.229397918	0.466660485
Vtcn1	4.93369E-43	6.65E-247	0	0.047144839	0.251196067	0.464254159
Slc1a5	0.006462796	0.00E+00	0	0.004010475	0.311719826	0.46349017
Arfgef2	2.41012E-31	9.71E-271	0	0.068228356	0.341541588	0.462931173
Gm43700	1	0.00E+00	0	-0.000575166	0.178338798	0.458192695
Hectd1	3.92403E-97	1.30E-260	0	0.120050408	0.418583334	0.458138415
Alb	3.27997E-21	2.66E-07	0	0.125886804	0.113327936	0.455840502
Sybu	1	0.00E+00	0	0.000804367	0.356703773	0.449598182
Atoh8	1.92461E-49	1.85E-276	0	0.054468018	0.27686906	0.448483455
Lrrc8d	1	0.00E+00	0	-0.019744248	0.514072078	0.447586092
Gstm2	1	0.00E+00	0	-0.002488621	0.368442937	0.445000257
Me1	2.5341E-17	1.11E-193	0	0.065365613	0.339703874	0.4442192
Tbc1d1	1	0.00E+00	0	0.002466411	0.326916957	0.442671228
Gpld1	1	6.27E-142	0	-0.006306782	0.276915368	0.441408805
Parm1	1	0.00E+00	0	-0.000188001	0.345584953	0.438888559
Tox	1.97769E-11	4.14E-23	0	-0.097710653	0.133954268	0.438718539
B4galt6	1	0.00E+00	0	-0.000713583	0.326733023	0.438608466
Ccdc171	1	4.98E-202	0	0.00149107	0.396431901	0.437778451
Gsr	1	0.00E+00	0	0.008414012	0.517295023	0.436537029
Acox2	1	7.85E-135	0	0.003979993	0.305356697	0.435013238
Hmgcr	2.0264E-149	3.45E-03	0	0.166092316	0.048341866	0.428424129
Ppp1r9a	2.05036E-78	7.01E-176	0	-0.133258842	0.32321192	0.427581579
Tnnc1	1	0.00E+00	0	0.001612559	0.28649994	0.425934755
Ccs	1.49783E-87	3.38E-105	0	-0.118548443	0.238406154	0.424711305
Tnfaip2	8.52341E-15	0.00E+00	0	-0.032015235	0.407594438	0.424428368
Comt	2.9154E-149	3.43E-78	0	-0.175798988	0.229670156	0.423626952

Trf	1	1.05E-03	2E-198	-0.017191184	-0.043473373	0.422830393
Gm37359	1	0.00E+00	0	-0.00059589	0.252057143	0.422736151
Etv6	2.47929E-46	2.40E-51	0	-0.103169856	0.197661976	0.417912252
Tgfa	0.488866572	4.89E-131	0	-0.014514014	0.249779873	0.417135652
Pkp4	0.183044315	2.56E-90	0	0.023443446	0.272628968	0.413206702
Tacc2	6.24927E-12	9.95E-137	0	0.054381035	0.334704951	0.412448401
Sult5a1	1.91527E-54	0.00E+00	6E-280	-0.105901451	0.68523226	0.412244815
Slc22a27	1	0.00E+00	0	0.003694215	0.187673832	0.412026912
Srxn1	1	0.00E+00	0	-0.000192352	0.397343457	0.411296614
Rictor	0.000660667	2.52E-217	0	-0.028665238	0.301420612	0.411141763
Crybg1	1.99035E-06	2.74E-217	0	-0.022830438	0.311472299	0.410809778
Jazf1	1	0.00E+00	0	-0.011078502	0.293964845	0.410338318
Slc16a7	3.81811E-82	9.35E-91	0	0.094404593	0.206783112	0.410194775
Tapt1	1	1.24E-68	0	-0.002036362	0.251440086	0.41002433
Gm42711	1	0.00E+00	0	-0.000577848	0.129162406	0.409920057
Gm29966	0	1.00E+00	0	0.311681746	-0.042609211	0.409449108
Tpr	1.85632E-32	2.27E-198	0	0.073109238	0.303464095	0.407500058
Srsf10	1	5.28E-120	0	0.010362578	0.18947015	0.406516768
Snd1	1.85771E-16	1.90E-150	0	0.05546742	0.29700102	0.403233489
Trak2	1	1.44E-169	0	0.00079179	0.238100048	0.401650627
Tmem214	5.98289E-21	3.44E-288	0	0.03626251	0.296740243	0.400686247
Gm34777	1	0.00E+00	0	0.008046436	0.340262485	0.400662108
Slc25a22	2.20228E-53	3.26E-17	0	0.087251893	0.102532282	0.400640284
Gm36419	2.9333E-106	8.80E-129	0	0.080327408	0.160971245	0.400039021
Ppfibp2	1.09266E-35	2.84E-284	0	-0.086982151	0.44999817	0.399051789
Aopep	1	0.00E+00	0	-0.026951316	0.489956392	0.398623881
Tnfaip8l1	0.0582289	1.24E-126	0	0.023603103	0.232051013	0.398068184
Hgfac	0.024606646	1.23E-56	0	-0.022188123	0.142617679	0.397936496
B3galnt2	1	5.83E-193	0	0.006927231	0.274114686	0.396861946
Yars	5.60103E-09	1.36E-194	0	0.032078807	0.257905794	0.39404763
Slco1a4	1.977E-104	3.00E-55	2E-295	0.160436154	-0.162066412	0.393972864
Prom1	1	0.00E+00	0	-0.000155313	0.155721392	0.39101679
Baz1a	1.15889E-16	1.70E-261	0	0.045064075	0.354094507	0.390836231
Hpse2	1	7.47E-58	0	-0.000455992	0.062011101	0.389490862
Rnf217	3.74648E-78	3.48E-23	0	-0.128003973	0.138797188	0.387160214
Rpl41	1	0.00E+00	0	0.006355551	0.383900973	0.385875063
N4bp2l1	3.1541E-168	2.01E-128	0	0.175260592	0.282778181	0.384983445
Ptges	1	0.00E+00	0	0.001118157	0.173100669	0.382747499
Myo5b	1	1.11E-155	0	-0.017449101	0.297498263	0.382009194
Rab11fip3	0.183593121	1.26E-159	0	0.021987899	0.259442785	0.380494236
Cdcp1	0.002809756	0.00E+00	0	-0.014342093	0.379606567	0.380205525
Fbxw8	1	4.93E-95	0	0.005421464	0.194169774	0.378345478
Rapgef4	1	2.66E-37	0	0.017082314	0.106762488	0.376841259
Nedd4l	4.8383E-296	6.48E-51	0	0.206039583	0.156992626	0.374936007
4833422C1	1.36447E-09	0.00E+00	0	0.026009987	0.324049524	0.374480348
Nbr1	1.17125E-57	2.63E-150	0	0.093674181	0.262201588	0.374458408
Ankrd12	1	0.00E+00	0	-0.013465709	0.465510389	0.373605052

Gm47283	1	1.38E-26	0	0.013967332	0.157395523	0.369200376
Setd4	4.06088E-34	4.53E-246	0	0.040297126	0.209296702	0.368153632
Ephx1	1.36024E-09	2.25E-178	0	-0.037385639	0.311619238	0.367819401
Hdac8	2.48514E-06	2.32E-113	0	-0.048233222	0.284789346	0.366155555
Hnrnpa3	2.28423E-23	2.89E-146	0	0.056658508	0.239785172	0.365885373
Trpm8	1	0.00E+00	0	-0.000731542	0.292030243	0.364887905
Fhit	9.95406E-18	1.02E-11	1E-295	-0.077918459	0.125194969	0.363750725
Ext1	1	3.06E-11	0	-0.023362273	0.123349657	0.363700982
Vps13b	6.23095E-05	1.26E-230	0	0.027959264	0.32793066	0.36368926
Akap9	7.8597E-17	2.26E-80	0	0.052168574	0.208849053	0.362896133
Rapgef2	6.0966E-32	1.04E-95	0	0.073647484	0.254661556	0.362300331
Ces1c	0.455938835	1.00E+00	0	-0.034864648	-0.001040208	0.361168133
Gas5	1.86769E-90	0.00E+00	0	0.087554521	0.313771329	0.356459812
Ces1b	1.51523E-28	3.54E-272	0	-0.045224458	0.395651975	0.35420529
Psme4	3.84237E-26	3.02E-156	0	0.06384056	0.281345573	0.352627947
Itih5	7.52696E-07	1.20E-08	0	-0.017820816	0.05023742	0.351679815
Fbxw9	1	0.00E+00	0	0.008526005	0.203318602	0.3502599
Slc7a11	1	0.00E+00	0	-0.00029804	0.541359257	0.349308354
Dop1a	1	1.43E-146	0	0.014210718	0.238916787	0.348895288
Iffo2	1	9.10E-194	0	-0.010443649	0.191754381	0.347382772
Bcar3	1.28672E-11	6.77E-98	2E-273	0.049943831	0.295185867	0.346292974
Tmprss2	1	0.00E+00	0	-0.000345327	0.282656715	0.345999354
Aox3	1.13334E-21	1.73E-12	0	-0.134500264	0.039377122	0.344539934
Atp13a3	5.18364E-43	5.76E-174	0	0.08324455	0.314776616	0.343933766
Uhrf1bp1l	1.58336E-23	3.46E-30	0	0.061320475	0.116185539	0.342466203
Mycbp2	5.26751E-24	5.79E-238	0	0.063536916	0.356537288	0.342088518
Rap1gap2	1	0.00E+00	0	-0.000238165	0.322539269	0.342059136
Abcg8	7.78748E-28	7.11E-110	0	0.059968983	0.222207067	0.340337605
Ank3	2.64824E-25	2.51E-205	0	0.046669783	0.326169358	0.339977733
Aqp9	1	1.31E-07	6E-289	-0.034821011	0.089148141	0.337420369
Gtf2ird1	3.26343E-13	2.95E-32	0	-0.049371151	0.14271226	0.335776664
Abca6	6.3356E-147	2.99E-05	0	0.145857341	0.065264294	0.335613552
Diaph2	2.3591E-09	9.00E-165	0	-0.048714755	0.320178924	0.33513083
Slc46a3	3.55062E-18	9.03E-151	0	-0.032453769	0.21013508	0.334958844
Ntf3	1	1.37E-208	0	0.002574062	0.142677058	0.333475289
Fmo1	6.83041E-68	8.55E-127	0	0.0922348	0.251498093	0.333182781
Fndc3b	0.000118317	6.21E-93	0	-0.030405505	0.230046054	0.332952549
Epg5	1	2.56E-116	0	0.007818451	0.198881607	0.332485067
Rxra	1	2.57E-26	2E-281	0.009195483	0.147823509	0.331811183
Pcp4l1	8.95509E-06	0.00E+00	0	0.008364268	0.302359828	0.331719064
Zup1	1	0.00E+00	0	-0.001773453	0.289940675	0.331262773
Gnl3	3.99177E-31	0.00E+00	0	0.042257105	0.256814853	0.329631709
Tyw1	1	2.50E-282	0	-0.00223569	0.285058641	0.328903061
Fubp1	5.15524E-37	1.46E-194	0	0.074791844	0.288939685	0.327976273
Agl	2.99379E-18	5.08E-158	0	-0.044372925	0.274946459	0.3269154
Apex2	8.75919E-60	1.52E-11	0	0.099170989	0.09276074	0.326209648
Hspbap1	1.26869E-17	0.00E+00	0	0.029469262	0.291798112	0.325929397

Dnase2a	1	0.00E+00	0	0.006844915	0.296474467	0.325618699
Ranbp2	2.08787E-07	2.16E-183	0	0.038082002	0.274318326	0.32529927
Prpf39	4.65261E-06	2.76E-119	0	0.031455462	0.210418829	0.324994253
Gm42031	3.62358E-17	2.42E-121	0	0.033892274	0.240073681	0.324660202
Cyp3a11	0	1.00E+00	2E-120	0.379183483	0.036713179	0.324039448
Gclm	1	1.58E-147	0	-0.000697456	0.293455648	0.32401352
Pick1	0.029885147	6.22E-254	0	0.015653547	0.225498799	0.323698557
Arl6ip5	0.46833305	1.94E-160	0	0.013785872	0.202465083	0.323011091
Ssbp2	8.27436E-21	3.57E-176	0	-0.029508251	0.202316468	0.32241024
Dpyd	1.42924E-61	2.81E-91	0	-0.049466384	-0.396995645	0.321661334
Zfp791	0.000108307	2.76E-135	0	0.017098306	0.145615084	0.321312265
Xrcc4	1	1.20E-67	0	-0.017676823	0.190174205	0.320830882
Ccdc85c	1	1.30E-152	0	-0.009060337	0.199494752	0.319842733
Cp	1.04782E-90	1.00E+00	1E-303	0.121917704	-0.021414293	0.31841719
Ugt2b35	2.51964E-62	3.19E-161	0	-0.092856701	0.315696158	0.3183281
Slc35f5	1	2.08E-297	0	-0.005194684	0.26004232	0.317553207
Osgin1	0	1.75E-30	4E-255	-0.271322898	0.1616121	0.317519321
Adamts14	1	0.00E+00	0	0.000525932	0.134176108	0.316562388
Gss	1	9.62E-231	0	-0.015626312	0.284465434	0.316518657
Itpk1	1.01745E-20	3.59E-57	0	-0.035187209	0.157552331	0.316247657
Slc25a3	3.80421E-32	5.31E-159	0	0.060659594	0.250544157	0.316087341
Sertad2	1.23546E-06	3.50E-131	0	-0.027640287	0.253601369	0.315869585
Cbr1	2.49892E-45	0.00E+00	0	0.026678389	0.157207591	0.314742264
Jmjd1c	1	5.94E-104	2E-258	0.00966131	0.280612723	0.314002171
Tango2	1	7.04E-48	0	-0.003821132	0.161699202	0.313727516
Sec61a2	2.93629E-07	1.84E-153	0	-0.026288839	0.232823104	0.313506332
Coro7	1	4.83E-169	0	0.014287513	0.20372321	0.312846001
Ptpn4	1	2.15E-59	0	-0.006429937	0.151185733	0.312820125
Frmd4b	6.08515E-98	5.96E-143	0	0.13559323	0.31305824	0.312550954
Lacc1	0.001317853	5.17E-246	0	0.009508965	0.139774961	0.312512704
Pgd	1	0.00E+00	0	0.004705749	0.29960509	0.311310231
Gipc2	1	0.00E+00	0	0.000110935	0.27077411	0.310661682
Rb1cc1	1.26936E-33	3.45E-150	0	0.071410692	0.264289044	0.310182672
Trpm7	1	1.01E-217	0	0.017392917	0.329733645	0.308808793
Meis2	1	0.00E+00	0	-0.000118469	0.336802	0.308708109
F8	1	0.00E+00	0	-0.006548943	0.270253181	0.308471647
Lrrc20	1	6.97E-82	0	0.004552019	0.189045826	0.307975349
Rbm39	6.31751E-64	1.23E-257	0	0.092795341	0.350830919	0.307118467
Sfswap	2.79279E-05	7.61E-152	0	0.034563073	0.240904593	0.306106663
Sel1l3	0.653939758	2.40E-72	0	-0.016940442	0.183662045	0.305919017
Tenm3	1	7.45E-14	2E-154	-0.005653405	-0.15632865	0.305686498
Cdh1	1	3.67E-260	0	0.00870211	0.279876682	0.305604314
Cpq	1.27475E-09	1.00E+00	0	-0.053679768	0.019894036	0.305498329
Washc2	1	1.07E-116	0	0.012497984	0.225896182	0.305044836
Xxylt1	1	3.76E-268	0	-0.002578855	0.246273491	0.304935143
Foxo3	1.21503E-62	1.00E+00	1E-211	0.122586007	0.061925531	0.304620424
Tpp2	1	1.27E-185	0	0.018052484	0.273764356	0.304316003

Galnt10	1	4.67E-260	0	0.001091866	0.229128673	0.304221275
Gstp2	1.68728E-23	7.97E-276	0	-0.040613242	0.335292887	0.304019169
Gm13657	1	0.00E+00	0	-0.001762436	0.169104642	0.303220236
Abcg5	2.36583E-24	2.25E-03	0	0.06384995	0.068823217	0.301907879
Acnat1	0	1.00E+00	2E-217	0.316513381	0.031834147	0.301229387
Gadd45g	1	2.49E-99	0	0.012382633	0.127821882	0.301131408
Tasor2	7.97646E-07	1.71E-276	0	0.02716638	0.303812194	0.301124801
Ahsg	8.9656E-120	9.37E-78	3E-209	0.188491532	-0.244660073	0.300693541
Man1a	1	5.91E-84	0	-0.000863287	0.224473803	0.300448082
Cabin1	1	1.07E-157	0	0.013229504	0.201970605	0.30006344
Plekhm3	1	0.00E+00	0	0.001757414	0.378907845	0.299183903
Acot4	1.97212E-83	4.04E-220	0	0.063373787	0.202188972	0.29915801
Hacd2	1.66836E-53	1.60E-75	3E-276	0.098731794	0.228574784	0.298874077
Mpped2	6.22476E-07	0.00E+00	0	-0.009457455	0.163908168	0.298862676
Igsf11	0.187891506	5.85E-38	0	0.019676504	0.12108438	0.298684934
Eif2s3y	1.18871E-14	2.03E-285	0	0.039052131	0.308634215	0.298278414
Bptf	1	4.25E-128	0	0.004107702	0.240311161	0.297815362
Stau2	2.42792E-17	5.80E-23	0	-0.051959161	0.118498478	0.297578867
Xylb	2.10226E-38	2.43E-224	0	0.078268724	0.335721344	0.297161152
Parp9	0.000357698	6.09E-106	0	0.026150125	0.208348878	0.296771347
Tardbp	1	3.32E-149	0	0.02198664	0.241667213	0.296534197
Gm41333	0.002567565	0.00E+00	0	-0.004357593	0.312325651	0.2960769
Scfd2	0.000135096	4.91E-124	0	0.035495841	0.248346414	0.295830218
Mid1	1	1.80E-187	0	-0.009915131	0.208830706	0.295654743
Kdm3a	1	1.66E-101	0	0.000108558	0.191839472	0.294239021
Gm43449	1	1.47E-228	0	0.003837399	0.171946477	0.294214
Tpm1	1	6.92E-218	0	0.009538317	0.205019528	0.293607516
Cyp3a59	0	1.00E+00	4E-182	0.386531349	0.033715165	0.293045027
Swt1	0.000140306	2.29E-78	0	0.029953618	0.181336542	0.292878553
Cdip1	5.5504E-193	8.09E-11	2E-249	0.2070548	0.090551472	0.292313666
Rif1	1	7.42E-77	0	0.011616707	0.183806056	0.292024133
Usp4	1	1.52E-76	0	-0.008049451	0.165629181	0.291763215
Etnk1	1	9.22E-10	0	0.007662174	0.07575118	0.291676934
Ttc17	1	4.28E-141	0	-0.0147913	0.251725893	0.291565138
Ccdc162	1	6.89E-62	0	-0.005363593	0.117604301	0.291511638
Aldh2	1	1.35E-61	0	0.013317057	0.191004573	0.291455025
Apc	1	3.71E-105	0	0.009422652	0.219983316	0.289295429
Bin1	1	4.01E-198	0	-0.002011518	0.228797626	0.289076881
Tmem62	1	0.00E+00	0	0.005509764	0.197029315	0.288844154
Greb1l	1	1.07E-35	0	-0.006000736	0.062471014	0.288756258
Acaca	1	6.44E-06	5E-300	-0.000989133	0.07410061	0.28734931
Slc6a6	9.9945E-123	2.73E-115	0	-0.107967307	0.253522428	0.287321346
Fmn2	1	0.00E+00	0	-0.001008334	0.286886386	0.286705749
Dennd4c	1	1.33E-210	0	-0.017766347	0.327051721	0.286449675
Mpp7	1	1.93E-95	0	0.006358387	0.205758144	0.286133347
Smchd1	2.9513E-09	6.13E-121	0	0.036959026	0.228986562	0.28609263
Gsg1l	1	0.00E+00	0	3.68622E-05	0.20065228	0.285672572

Tsku	1.67696E-09	0.00E+00	0	0.027796201	0.337293695	0.285627479
Mecr	1	2.16E-110	0	0.014174582	0.185446195	0.285117465
Mad1l1	0.00015941	2.27E-111	0	0.028529994	0.231986574	0.285030051
Ivns1abp	2.2276E-117	8.78E-130	0	0.124737943	0.257103792	0.283921951
Cald1	1	1.00E+00	5E-236	0.005625274	0.018989048	0.283252918
Pxk	6.16374E-15	3.95E-78	0	0.047958776	0.185882627	0.28204953
Eif5	1.37718E-43	5.02E-91	0	0.07971083	0.195069203	0.282004238
Cyp2b13	1	6.54E-301	0	-0.000628613	0.105162641	0.281161903
Nt5e	1	8.46E-110	0	0.003404992	0.101606087	0.280573228
Bcl2l11	1	0.00E+00	0	-0.004588148	0.32178426	0.280298051
Aldh4a1	1	1.00E+00	5E-271	-0.004181964	0.061699406	0.280110176
Setdb2	3.04221E-59	1.00E+00	7E-138	0.081643268	0.055696793	0.280041292
Abcd2	1.01355E-27	1.70E-121	0	0.027199406	0.111205391	0.279174814
Dgkh	1	1.79E-123	0	0.006145044	0.206988663	0.278623873
Vti1a	2.32306E-08	1.13E-61	0	0.038372927	0.192882176	0.277855043
Utp14a	0.065146886	3.35E-155	0	0.017350304	0.197099287	0.277511353
Gtdc1	1	1.59E-05	4E-253	0.010665709	0.089335058	0.277406809
Safb2	8.94429E-44	2.29E-160	0	0.082336571	0.267913977	0.277248413
Kdm5a	1	1.86E-112	0	0.019845183	0.21740356	0.276493399
Trim2	1	5.69E-09	2E-265	-0.009035718	0.080360748	0.274629082
Akr1c14	1	1.71E-166	0	0.003695338	0.307442123	0.274359619
Pkm	1	0.00E+00	0	0.000429376	0.223607402	0.273851941
4732463B0	1.429E-78	0.00E+00	0	0.03819463	0.189026743	0.273153958
Sdk1	1	2.76E-87	0	-0.005969692	0.122711409	0.27232283
Herc2	5.65377E-20	1.00E-73	0	0.058983118	0.179433194	0.27196583
Sfxn1	1.7685E-14	3.71E-10	3E-249	0.054210891	0.094215939	0.271846215
Atg2b	0.000756069	1.43E-30	0	0.025499264	0.106556781	0.271765875
Creg1	1	2.75E-105	0	0.0208351	0.22718803	0.271687168
Rps2	0.000119698	4.81E-280	0	0.018548418	0.23885854	0.271306571
Cpeb2	5.05715E-21	4.47E-73	5E-186	0.064112102	0.246440859	0.270727306
Cpb2	2.62176E-20	6.00E-03	6E-224	-0.048844689	0.110300477	0.269696896
Rpl13a	1	1.16E-269	0	0.013261972	0.252264562	0.269522269
Mettl15	9.13323E-18	1.20E-151	0	-0.034070329	0.208576723	0.268792212
Kif21a	0.266892181	1.22E-36	6E-268	0.025119382	0.152652016	0.268747495
Chdh	9.51127E-12	6.62E-07	9E-279	-0.0450445	0.072980626	0.268547046
Mal2	0.507301404	3.60E-101	0	-0.017257517	0.175148719	0.268267124
Snx30	4.85299E-15	4.90E-163	0	-0.029437698	0.216725673	0.26797873
Sult1d1	0.175228087	1.27E-38	0	-0.028119719	0.124276156	0.267696615
Mfn2	1	1.67E-65	0	-0.008695557	0.136566933	0.266707635
Eif4a2	1.97703E-29	3.69E-139	0	0.056400894	0.214463794	0.266429218
Lpin1	0	1.86E-23	4E-114	0.544033168	0.06712041	0.266056306
Serpine1	1	2.01E-220	0	0.001494494	0.091334677	0.266041442
Stimate	0.001865784	6.38E-122	0	-0.016880405	0.15670381	0.265975225
Maff	1	0.00E+00	0	-0.001881412	0.239056809	0.265260485
Akr1c19	1	3.07E-185	0	0.000863933	0.247083941	0.264997889
Sf3b2	1.12089E-10	1.76E-102	0	0.033734951	0.178158025	0.262770456
Ralgapb	0.073559391	7.20E-115	0	0.021992328	0.205082623	0.261938632

Pde6c	0.350902101	0.00E+00	0	-0.004904151	0.155256086	0.261888072
Slc22a5	0.009438064	1.05E-135	0	0.023791645	0.228622244	0.261581707
Usp18	0.44202468	6.72E-206	0	-0.007944435	0.149523933	0.261332639
Pitpnm2	7.98849E-34	3.42E-54	4E-236	0.07790363	0.17570765	0.260447343
Micos10	1	3.58E-71	0	0.013545681	0.145037434	0.260328538
Iars	1.72891E-09	1.68E-212	0	0.024660451	0.206199236	0.260225282
Mast4	1	7.11E-57	0	-0.018029173	0.16558515	0.260109181
0610010F0	1	5.62E-138	0	-0.011420776	0.197932318	0.259949556
Pan2	1.9074E-06	1.47E-136	0	0.028515714	0.204362634	0.259886954
Myo1b	5.1188E-294	1.03E-02	4E-215	0.246553967	0.067873397	0.259722519
Stk40	5.30807E-07	1.65E-49	3E-298	0.037426617	0.16778552	0.259615721
Ccdc93	1	4.15E-111	0	-0.00816434	0.185996051	0.259510219
Ptpn9	2.1139E-48	2.57E-33	0	-0.069002464	0.134033157	0.259428374
Bcl9	0.010829724	9.58E-89	0	-0.01749734	0.158983671	0.259342868
Trpv4	1	0.00E+00	0	-0.00041449	0.194516074	0.258842338
Shroom1	0.015803702	5.24E-01	0	0.023032755	0.036814584	0.258769015
Dram2	1	7.54E-66	0	-0.010218828	0.154767714	0.258596696
Pgap1	0.000353408	1.08E-88	0	-0.016777976	0.147024176	0.257551778
Il1r1	1.2134E-272	5.57E-35	4E-85	0.348592056	0.247187716	0.257257726
Fto	1	4.58E-21	2E-276	-0.008875362	0.12326865	0.256476117
Sgms1	1	6.11E-116	1E-235	0.012007698	0.251475961	0.255337307
Desi2	9.82662E-21	3.70E-22	1E-229	0.063598274	0.117846049	0.25529481
Dapk2	0.001937555	4.76E-45	0	0.021033207	0.120426363	0.255188662
D630024D(	0.000220794	0.00E+00	0	-0.013295531	0.286409174	0.254774825
Camkmt	1	1.07E-145	1E-206	-0.000860522	0.322456454	0.254313109
Lrp5	5.36808E-09	3.99E-111	2E-259	0.046169681	0.22693545	0.253821754
Ttc37	0.016642297	1.66E-35	0	0.023371962	0.106485059	0.252207913
Ptpn1	1	1.43E-208	0	0.013285812	0.243239354	0.250962115
Tafa5	1	1.98E-176	0	0.002710555	0.138629028	0.250171913
Tfrc	3.34637E-16	4.03E-185	0	0.030571723	0.192362851	0.24979816
Ndufaf4	5.87838E-20	1.02E-247	0	0.032045381	0.215715158	0.249258362
Srpk1	1.30305E-11	4.90E-83	0	0.031736099	0.143221502	0.248307458
4632427E1	1	9.02E-29	0	-0.000774441	0.101563776	0.248226692
Scfd1	2.13007E-11	7.34E-07	0	0.042426417	0.062464721	0.24795628
Fam185a	1	5.21E-134	0	0.008001713	0.164623334	0.247426032
Cyp39a1	1.16005E-81	4.57E-72	6E-284	0.105018086	0.184680731	0.247194536
Rpl36	1.11426E-05	8.87E-189	0	0.018784103	0.178095161	0.246606918
Sec16a	0.005870712	1.00E+00	3E-268	0.029362981	0.024429297	0.24660131
Cyp4a10	0	1.00E+00	4E-84	0.44713956	0.040712284	0.246592919
Pdcd11	4.40119E-05	5.93E-117	0	0.024488476	0.174752251	0.24657353
Gm41804	1	0.00E+00	0	-0.002575816	0.182875721	0.246446739
Agpat3	6.04192E-05	1.79E-28	2E-232	0.033994108	0.134485622	0.246355357
Edem1	1	3.35E-15	7E-301	0.007398957	0.083520351	0.24629341
Grip1	1	1.37E-217	0	-0.011140542	0.243296414	0.246255501
Erbb3	4.71263E-10	1.02E-38	0	0.03737138	0.128057821	0.245887668
Agap1	2.24745E-10	1.78E-123	2E-208	-0.047890507	0.278768313	0.24584144
Sec14l4	1	1.00E+00	1E-215	0.01865852	-0.022148722	0.245499875

Nbas	7.75537E-15	6.99E-06	5E-294	0.047055669	0.062442612	0.245342458
Rptor	0.299848719	1.67E-55	5E-266	0.021180053	0.167568101	0.245271086
Pacs2	0.016203953	1.03E-166	0	-0.015270096	0.205793148	0.245067617
Reep3	0.000119505	1.77E-109	2E-202	0.034763951	0.247539233	0.244543697
Mindy1	0.129004742	1.93E-50	7E-292	0.023181539	0.149295961	0.244237399
Mdm2	1	1.92E-60	2E-276	0.018515725	0.175029103	0.24398804
Etnppl	7.74012E-06	3.29E-27	1E-109	-0.052744685	0.182785471	0.243884539
Mical2	0.088952891	3.16E-15	3E-199	-0.021996658	0.108882817	0.243266025
Aldh1l1	2.04035E-08	3.52E-03	1E-151	0.051620454	0.075115543	0.243163542
Fyb2	1	2.50E-52	4E-246	0.006881077	0.170132412	0.242297759
1110051M	2.31903E-07	2.35E-245	0	-0.015773321	0.199701643	0.241443273
Polr1a	3.81841E-05	1.41E-104	0	0.024505761	0.163807851	0.241358458
Kif16b	1.21253E-09	1.82E-24	3E-226	-0.037327983	0.140173613	0.241338301
Tm4sf4	1.22038E-15	1.02E-65	0	0.034970914	0.139953137	0.240443677
Col9a2	1	0.00E+00	0	0	0.217577635	0.24027138
Cep192	1	0.00E+00	0	-0.007440084	0.203991744	0.23984257
Ccar1	5.10792E-35	3.98E-120	1E-255	0.073267667	0.229972221	0.23966961
Derl1	0.000351628	8.04E-87	0	0.022011854	0.14263088	0.239573606
Ikbke	1	2.27E-137	0	0.003357826	0.176372887	0.239472467
Tns3	0.060161457	1.72E-173	1E-302	-0.018770808	0.294653966	0.238702422
H6pd	6.38481E-62	1.60E-38	3E-264	0.096012284	0.140768516	0.238663918
Trim24	1.145E-19	5.88E-10	0	0.040587489	0.056911089	0.238460666
Krit1	0.000108794	3.82E-169	0	0.026645502	0.248698108	0.238447827
Eef1a1	4.4572E-10	2.26E-101	0	0.034414247	0.186807861	0.238439004
Ppm1h	1	0.00E+00	0	-0.004523145	0.369033475	0.23788857
Wwtr1	1	1.32E-38	7E-288	-0.012828202	0.126841914	0.236550022
Sugp1	0.000889912	5.25E-57	0	0.018737751	0.104073534	0.236402097
Hspd1	1	1.15E-134	0	-0.008028482	0.190212396	0.236364213
AI463229	1	3.10E-67	0	-0.003561458	0.145327297	0.23577059
Eif4g1	2.22744E-25	4.01E-93	6E-242	0.062915473	0.209986557	0.235317726
4833420G1	4.19511E-20	2.04E-50	0	0.049111318	0.133006068	0.235257514
Pqlc1	1	1.00E+00	3E-190	0.009645161	0.010418905	0.234699606
Fam234b	0.995518795	5.68E-235	0	0.012273526	0.227983024	0.234602325
Polk	1	1.33E-92	0	0.007755485	0.139870716	0.234256738
Gm36723	1	0.00E+00	0	-0.001504341	0.222747116	0.233369736
Rpl13	1	4.06E-293	0	0.00599459	0.232410834	0.232468552
Il6ra	0	2.71E-03	6E-140	0.335916938	0.072356978	0.232263882
Ewsr1	1	3.24E-110	5E-280	0.022274721	0.204966774	0.232102843
Rpl3	1.08194E-10	3.45E-283	0	0.019516146	0.193404451	0.231067719
Snx10	3.33149E-62	2.44E-224	2E-300	-0.06715429	0.292501185	0.23042725
Nrn1	1	3.04E-64	0	0.011357076	0.140902094	0.230047384
Mocos	3.63132E-17	3.20E-93	3E-275	-0.038495586	0.196585421	0.22985004
Slc48a1	1	0.00E+00	0	0.002521211	0.231461167	0.229808591
Hipk1	1.47482E-08	2.82E-61	6E-292	0.033755038	0.162435942	0.229638216
Micu1	1	7.13E-54	1E-246	-0.017967175	0.157541862	0.229253815
Rhbdd1	1	1.20E-84	3E-298	-0.003329528	0.175829609	0.229061692
Net1	0.74978604	9.93E-32	0	0.016339932	0.094137107	0.229029913

Ascc2	1	4.98E-94	0	0.004832173	0.170815685	0.228696354
Cox7c	1	3.22E-86	0	0.010115417	0.151619128	0.227244859
Copa	1.1071E-33	1.40E-15	1E-256	0.071015442	0.09429787	0.227022153
Esr1	1.34267E-64	1.49E-03	9E-203	-0.083081875	0.074108569	0.226909232
Zfp532	1	7.79E-233	0	-0.003825929	0.195953178	0.226617474
Ivd	1	1.55E-01	1E-232	0.012013385	-0.026582874	0.226195194
Ccdc141	1.5901E-154	1.00E+00	5E-164	0.167196132	-0.009039963	0.226062858
Tbcd	1	4.05E-60	1E-293	-0.011048869	0.151261654	0.226030046
Trmt1	1.95458E-11	1.60E-70	0	0.026102127	0.109561963	0.225943015
Plbd2	6.70756E-06	2.69E-173	0	0.017990738	0.159468343	0.225605272
Zfp973	0.000856855	3.28E-60	0	0.020444991	0.117585342	0.225353979
Ubr2	9.92906E-41	4.37E-31	2E-193	0.081910936	0.136523755	0.225266227
Shtn1	7.8469E-23	8.35E-182	6E-206	-0.060781191	0.296566773	0.22513415
Arglu1	6.22137E-18	8.10E-171	5E-282	0.052264137	0.247411331	0.225117095
Nr1i3	1	1.00E+00	6E-102	0.007220719	0.018362822	0.224729549
Pir	8.09941E-20	4.31E-104	1E-202	-0.049787418	0.251282479	0.224455994
Arhgef10l	6.01143E-26	1.20E-53	5E-189	-0.066805644	0.174962587	0.224294669
Trappc9	1	1.54E-29	1E-185	0.004723304	0.138247254	0.224279512
Rab30	1	1.00E+00	5E-220	0.000107582	0.008344941	0.224036571
Mdn1	8.02981E-30	3.26E-79	0	0.049937562	0.146306113	0.223836028
Slc39a11	1	1.00E+00	4E-196	-0.008480918	0.040038717	0.223267544
4732419C1	2.5062E-09	1.21E-94	0	-0.022465751	0.143149199	0.222964397
Npas2	6.77542E-54	1.00E+00	1E-138	0.099360015	0.008898508	0.22262425
Tubgcp5	1	3.46E-160	0	0.010158669	0.147457659	0.222530359
Pgm3	5.71164E-05	9.84E-52	0	0.018726984	0.091649917	0.222464144
Ctps2	1	6.76E-174	0	-0.000502393	0.181059868	0.222345136
Rock2	8.07979E-13	9.44E-220	4E-166	-0.049129064	0.350141344	0.221986665
Lgr5	0.737358523	3.73E-13	6E-215	-0.017354828	0.098261398	0.221917641
Ttc41	1	6.92E-198	0	-0.002055927	0.149381408	0.221861346
Rbp4	4.7384E-121	1.00E+00	1E-202	0.134884185	0.000989565	0.221469087
Lrch1	3.15868E-07	1.60E-216	2E-209	-0.036614215	0.320137046	0.220945906
Cped1	0.009362622	3.47E-04	3E-242	-0.028850897	0.036198372	0.22046303
Riok2	1	6.13E-113	0	-0.01019861	0.176404783	0.220345354
Sorbs3	1	1.52E-115	0	0.003248802	0.173372882	0.219605661
Dclk3	8.29473E-12	1.78E-212	0	-0.014552698	0.157922195	0.219169316
6030443J0	0.128584905	1.21E-12	5E-276	-0.018422279	0.074689333	0.219084652
Tgfbr2	1.73565E-44	5.63E-171	0	-0.036520846	0.171962563	0.219054604
Plin2	1.86114E-07	2.28E-193	5E-82	-0.038029305	0.395802551	0.219040111
Gm33543	1	3.58E-30	8E-222	-0.00614239	0.135673164	0.218400961
Camk2d	0.534074044	2.69E-124	4E-168	-0.020220082	0.254511241	0.218214684
Col4a3	1	4.10E-241	0	0.000574643	0.115534917	0.217563999
Slc13a3	1	0.00E+00	0	-0.002442176	0.118819498	0.217085765
Marveld3	1	0.00E+00	0	0.004042933	0.165933933	0.216666731
Ggcx	1.81478E-15	7.44E-39	0	0.031539013	0.083375418	0.216493493
Atr	1	2.78E-164	0	-0.005398393	0.197915842	0.21634059
Ypel3	1.8749E-14	4.69E-112	0	0.031312673	0.160954938	0.216222363
Gon4l	1.65467E-09	1.32E-58	1E-237	0.041656305	0.152927803	0.216199128

Srrt	3.60963E-16	1.10E-18	3E-277	0.043071602	0.086226062	0.216019937
Utp20	0.000320861	1.47E-96	0	0.019871296	0.138020205	0.215366752
Slc10a7	0.009557115	7.83E-66	2E-193	-0.027026111	0.171980991	0.214925134
Tbx3os1	1	4.07E-24	2E-280	-0.010461546	0.076291877	0.214815156
Nfx1	9.14709E-17	9.02E-45	3E-222	0.050697895	0.141550238	0.214168348
Cluh	1.4745E-22	7.58E-10	1E-207	0.059960746	0.076321466	0.214125952
Tut4	8.54311E-42	4.20E-84	3E-203	-0.072393468	0.194648382	0.213596595
Deptor	7.35442E-97	1.00E+00	8E-127	-0.136454644	0.064852796	0.213538354
Ddc	8.6715E-262	3.20E-72	2E-79	-0.24877544	0.316658873	0.213300901
Gtf2a2	1	4.10E-70	0	0.006966214	0.131794801	0.21302864
Rmdn2	6.11538E-25	1.00E+00	6E-195	0.062511856	0.010299967	0.21234703
Secisbp2l	6.7813E-16	1.77E-37	2E-297	0.041008931	0.1094772	0.212310169
Srsf2	6.11016E-42	8.30E-53	2E-215	0.0781932	0.159495198	0.212093864
Cplane1	1	8.63E-96	0	0.004660903	0.146017014	0.211704207
Mpp6	1	1.80E-39	5E-159	-0.019312308	0.148754175	0.211293205
Rai1	0.374645792	9.47E-45	5E-201	-0.01996682	0.140040874	0.210734064
Dph5	1	4.06E-71	0	-0.009359443	0.132324543	0.210733818
Lrrk1	0.01164989	5.39E-76	0	-0.016929236	0.15307018	0.2107307
Camk2b	3.3753E-133	8.71E-78	4E-227	0.128705804	0.183911914	0.209944865
Cox10	1	1.89E-42	2E-285	0.009912838	0.120274837	0.209644673
Itfg1	1	4.46E-47	3E-235	0.01682428	0.139083233	0.209632979
Fbxo31	0	9.91E-79	2E-199	0.233246905	0.197620213	0.209097868
Wdr91	1	7.47E-69	2E-257	-0.004709796	0.15894954	0.208561949
Tpk1	7.05843E-59	2.25E-65	1E-143	-0.107815355	0.203184999	0.207853367
Atp9a	1	4.78E-39	9E-236	-0.010718613	0.124960204	0.207816618
Aco2	1.41767E-08	1.55E-03	1E-180	0.04038434	0.059268932	0.207808891
Ptcd3	1.31294E-05	1.26E-83	0	0.024592556	0.147352869	0.20747063
Snhg11	1	4.78E-93	1E-209	-0.001206003	0.223490084	0.206981742
Cdc42bpg	1	2.06E-220	0	-0.001578136	0.139414659	0.206832981
Zfc3h1	1	1.93E-98	6E-189	0.017198496	0.212317345	0.206649984
Gadd45b	1.66699E-16	0.00E+00	0	0.00755328	0.099861681	0.206018401
Cdk7	3.65228E-12	4.60E-37	4E-280	0.033872047	0.106569621	0.205906511
Tmem135	4.66178E-05	8.86E-45	1E-162	0.033533162	0.155368189	0.205825951
Ogt	1	3.71E-157	3E-209	0.00473027	0.260543478	0.205822248
Thada	2.52806E-11	2.71E-153	1E-198	0.044145985	0.248812637	0.205593924
Xpnpep1	1	2.34E-91	0	-0.000545354	0.122624514	0.205517342
Rtn4rl2	1	0.00E+00	0	-0.000126858	0.166217571	0.205515345
Afg3l2	1	4.29E-32	2E-247	0.017368505	0.108165276	0.205297845
Prkdc	1	1.04E-89	1E-263	0.002089311	0.174802678	0.205083231
Cdk11b	5.95778E-57	5.48E-75	4E-242	0.079847278	0.159072655	0.203919723
Nqo1	1	0.00E+00	0	0.00065719	0.121565801	0.20337918
Lncpint	1	8.53E-169	6E-191	-0.019339061	0.283826073	0.203234883
Traf3	0.757720835	1.47E-45	7E-214	-0.015850929	0.142393008	0.203129823
Phkb	1	1.88E-33	4E-159	0.019767847	0.139926332	0.202571383
Meis1	0.64788368	1.09E-74	0	0.015076829	0.125802825	0.201823795
Atg2a	1	3.31E-82	3E-276	0.015970814	0.152365903	0.200706911
Fmn1	7.24402E-13	1.00E+00	1E-101	0.055583585	0.041333808	0.200583711

Uty	1	3.16E-81	5E-158	0.01577973	0.207119496	0.200225533
Itsn2	0.026385763	3.86E-121	6E-184	0.027756056	0.233596246	0.199954365
Mon2	9.53089E-09	1.93E-45	9E-190	0.040659619	0.139063887	0.199923183
Slc20a1	6.20297E-06	1.88E-86	0	0.017577479	0.10856718	0.199781876
Ldhd	1	6.17E-96	0	0.001433138	0.110841833	0.199678906
Tmem176b	1	0.00E+00	0	0.003336788	0.171771077	0.199655375
Wwp2	1	2.09E-99	4E-253	0.006105496	0.191430193	0.199387364
2500002B1	1	0.00E+00	0	-0.005461901	0.182200153	0.199337917
Rps20	1	0.00E+00	0	0.003712067	0.210969012	0.198982864
Nfia	2.3075E-278	1.23E-11	6E-210	0.177856728	0.029765738	0.198778076
Tbk1	1	2.10E-127	2E-234	-0.00022508	0.221926965	0.198723126
Gm26911	1	8.25E-182	0	0.002168601	0.073374395	0.198712465
Nemf	2.66979E-32	3.71E-18	2E-201	0.06784169	0.092248421	0.197963455
Sh3gl1	0.001810802	4.85E-106	0	0.015046156	0.13048331	0.197884404
Slc22a3	1	4.96E-202	0	0.003994122	0.137656937	0.197460734
Dmxl1	1	3.14E-128	2E-165	-0.022690667	0.252365475	0.197460256
Clk4	1.2211E-20	8.41E-56	7E-190	0.054999505	0.151396779	0.197356153
Pigx	1	3.22E-108	0	-0.003311119	0.110676872	0.19708996
F5	1	1.64E-06	1E-130	-0.010531886	0.088217886	0.197083806
Mga	1	2.67E-98	2E-186	-0.006120714	0.214607125	0.196771559
Cox16	1	1.56E-02	9E-128	-0.007048894	-0.033507844	0.196282212
Lars	2.52689E-05	1.91E-55	8E-251	0.026519004	0.130756853	0.196142359
Rps8	1	5.79E-209	0	0.007380557	0.220801885	0.195841159
Fgf21	1.34574E-15	0.00E+00	0	0.007940277	0.135822356	0.19552046
Hmgb2	1	5.38E-214	0	-0.00704407	0.145300412	0.195503014
Wdr3	0.053978874	7.69E-156	0	0.013597021	0.155030781	0.195442174
Tcerg1	3.06419E-05	1.59E-24	2E-245	0.027123659	0.092196736	0.195223007
Rhou	1	7.38E-84	2E-285	-0.011623414	0.146663121	0.195182552
Enpp1	1	7.23E-62	2E-292	-0.003576494	0.122134628	0.19511754
Acsm5	2.0412E-33	1.39E-40	9E-155	-0.069059501	0.161054095	0.194956625
Itga1	1	2.27E-04	2E-177	0.010649655	0.060075052	0.194762231
Ralgapa1	1	5.56E-61	4E-195	0.009186702	0.170037761	0.194732403
Mst1	2.67495E-30	1.00E+00	6E-234	0.056807173	-0.003306589	0.194526232
Serinc3	1	3.48E-191	1E-219	0.00062692	0.266813063	0.194505048
Epn1	0.007097792	8.44E-50	0	0.020088281	0.111953467	0.19428837
Ipo7	1.08941E-12	1.40E-90	2E-287	0.03276636	0.152644917	0.194286861
Zfp64	1	5.91E-38	1E-225	-0.00931178	0.11401154	0.193633607
Lrpprc	0.00048697	1.02E-29	1E-140	-0.034921479	0.129837809	0.193500005
Pepd	0.001720362	6.66E-14	6E-229	-0.020750202	0.07475588	0.192851631
Srp72	1.16886E-30	9.19E-03	4E-228	0.058798889	0.042309143	0.192789804
Abhd4	1	5.07E-162	0	0.009557635	0.140771406	0.192099246
5330439B1	1	0.00E+00	0	-0.000135709	0.154213642	0.19193697
Dus1l	0.044055227	6.11E-20	0	0.012536369	0.048651923	0.191885117
Alcam	0.032089117	3.98E-31	2E-66	-0.035540153	0.226142497	0.191638033
Tomm40	0.00177907	3.97E-38	2E-270	0.021333452	0.101329484	0.191631526
Smg1	1	4.31E-124	1E-133	0.008887095	0.267900129	0.191521251
Arfgef1	1	1.60E-122	2E-173	-0.005756338	0.226301378	0.191073217

2410131K1	1	1.10E-214	0	0.004231998	0.13737858	0.190207091
Snx5	1	8.50E-148	0	0.003102777	0.186437087	0.189959267
Spon1	1	0.00E+00	0	-0.000489009	0.183893274	0.189907167
Slf1	1	3.41E-78	0	-0.004713682	0.121659576	0.189757106
Fam172a	1	9.51E-105	2E-123	-0.004857122	0.263321444	0.189624611
Aldh1a1	3.8638E-103	5.30E-24	3E-55	0.159797234	0.160465404	0.189531552
Col4a5	0.001925792	9.33E-89	0	0.009773353	0.089243929	0.189227527
Cdkal1	1	1.00E-38	5E-139	0.014975267	0.159169317	0.189134596
Serpinb6a	1	1.08E-97	0	0.004967215	0.14356994	0.189095008
Sult2a1	1	6.14E-157	0	-3.92791E-05	0.044696341	0.189014394
Snhg1	3.36105E-12	0.00E+00	0	0.018079597	0.184968567	0.188851656
Acsl4	4.34561E-08	1.00E+00	3E-152	-0.030218743	0.05264073	0.188818407
Rpl37a	1	2.07E-191	0	0.01037268	0.188522319	0.188663534
Rit1	1	5.66E-232	0	0.004299792	0.165704507	0.188656751
Glyr1	1	3.11E-12	2E-185	0.018222126	0.078172286	0.188508737
Mpp2	1	0.00E+00	0	-0.000183721	0.121453368	0.188501544
Usp40	2.18593E-13	1.11E-48	8E-146	0.049088935	0.159244063	0.187928869
Gpr107	1	9.95E-39	8E-216	0.001253809	0.116083547	0.187737178
Nop58	0.000436631	5.51E-67	0	0.020461977	0.117467272	0.187729548
Gramd4	1	7.34E-72	0	-0.001438851	0.111957007	0.187393674
Impact	1	2.70E-89	9E-295	0.006237181	0.142378734	0.187246714
Ppid	6.95066E-18	3.02E-76	0	0.028780595	0.098908352	0.186635353
Abce1	3.19909E-18	2.39E-138	0	0.032282005	0.154096024	0.186554638
Ano10	0.119994504	4.41E-57	0	-0.011874685	0.100562912	0.186394688
Gars	0.14867126	1.15E-139	0	0.010981885	0.132182274	0.186356245
Cyp2a4	1	0.00E+00	0	0.000964366	0.224295208	0.186242448
Mroh2a	1	0.00E+00	0	-0.003604833	0.145570911	0.186033859
Alkbh1	0.000126392	2.21E-73	2E-274	0.023326717	0.129244128	0.185919437
Hmox1	1	0.00E+00	0	-0.003378274	0.208911237	0.185653027
Nop56	2.29745E-14	3.48E-104	0	0.027521308	0.132848156	0.185049914
Arid2	1	7.76E-99	4E-152	0.004918558	0.212436589	0.184745444
Clcn3	1	3.43E-36	3E-176	-0.017749674	0.13029208	0.184727643
Ttc27	1	2.68E-77	8E-247	0.007779336	0.148634719	0.184706894
Srsf5	1.8384E-121	1.18E-71	4E-140	0.13495476	0.202123821	0.184699899
Ssb	4.4458E-06	2.19E-53	3E-291	0.024605575	0.109840764	0.184451978
Orc3	1	4.19E-52	3E-222	0.004786802	0.128412401	0.184111278
Ubxn4	7.59562E-51	8.39E-36	2E-142	0.092112218	0.134249524	0.184033477
Rpl10	1.1116E-14	7.48E-149	0	0.020666403	0.115259675	0.18393718
Eif2a	2.47578E-05	7.81E-101	0	0.020711821	0.138199169	0.183678689
Map4k3	1	6.27E-122	2E-130	-0.000114362	0.253864386	0.183511753
Hsd17b2	2.6691E-213	2.37E-29	3E-66	-0.254997747	-0.146793279	0.183423669
Echdc3	1.70476E-35	8.71E-11	2E-149	-0.062025192	0.092160758	0.183298365
Nptx1	1	0.00E+00	0	1.17019E-05	0.11755974	0.183139443
Ctr9	1	1.06E-43	5E-271	0.002246685	0.098545329	0.182647997
Preb	5.79659E-68	1.00E+00	6E-205	0.088774954	0.015909601	0.182536466
Top2b	5.36746E-15	2.17E-64	4E-216	0.041490903	0.145315195	0.182388145
Dmd	6.69293E-29	1.00E+00	3E-142	-0.063372526	0.032141518	0.182340435

Heatr1	2.10295E-07	1.82E-174	0	0.018088691	0.143842979	0.182270842
Kdm2b	1	1.63E-50	2E-241	0.010501222	0.118904986	0.18185729
Tcf4	1.79517E-22	2.90E-59	5E-125	-0.062880931	0.178281993	0.181456309
Pcsk6	1	1.28E-46	2E-92	0.008522578	0.198231023	0.181383072
Ddb1	1	1.63E-59	8E-298	0.010992796	0.110392493	0.181289159
Esd	0.000266042	7.18E-141	1E-271	0.020457791	0.191372429	0.180671747
Zfp981	2.23456E-62	3.22E-38	9E-251	0.070455713	0.10719152	0.1806009
Atad3a	5.48116E-26	1.00E+00	9E-186	0.054923866	0.02558788	0.180558513
Nomo1	1	4.81E-92	0	0.003547296	0.106170237	0.179205657
Mt1	1	2.31E-86	0	0.005634008	0.082288428	0.179151569
Sidt2	1.04585E-05	1.24E-52	6E-267	0.024243445	0.111012843	0.179111866
Zfp280c	1	4.24E-144	4E-303	0.003064635	0.163471175	0.178993398
H2afj	1	6.46E-213	0	0.002331395	0.133036455	0.17891426
Atg4c	1	4.12E-48	4E-208	0.010769992	0.123843797	0.178774895
Clk1	1	1.09E-112	3E-134	-0.002569258	0.242112172	0.177975263
Morc2a	1	5.24E-49	4E-220	0.001683562	0.117388371	0.177803034
Smarcad1	0.051336839	7.36E-52	2E-240	0.019847454	0.117360606	0.177621177
Slc1a2	2.73043E-38	1.00E+00	3E-91	0.046146406	0.040054736	0.17733256
Thrap3	1	2.00E-23	1E-159	-0.001874	0.101957303	0.177171173
Arap2	0.016804374	2.61E-35	2E-218	-0.017882559	0.103938377	0.176923218
Sdc4	1.8573E-270	8.17E-56	3E-84	0.240122978	-0.1604447	0.176736704
Btg3	0.037837653	2.29E-135	0	0.011088014	0.136128316	0.176680261
Zfp746	1	3.83E-43	1E-263	0.002741521	0.099643908	0.176530017
Rock1	7.53113E-20	1.62E-86	3E-139	0.056722577	0.202270997	0.176236424
Ik	3.82634E-11	1.39E-25	6E-240	0.031693646	0.086480317	0.175814009
Eif4a1	1	1.17E-108	1E-217	0.010716297	0.17561982	0.175765083
Krcc1	5.94493E-07	4.87E-70	2E-217	-0.02440256	0.145725031	0.175722741
Gtpbp2	3.41619E-10	7.39E-80	8E-264	0.028329825	0.139221037	0.175424396
Cul4a	0.199097895	1.56E-32	2E-144	-0.023227561	0.121137511	0.17541186
Selenok	1	2.26E-204	0	0.003538048	0.150450433	0.17527953
Fn1	1.0123E-79	6.95E-154	7E-53	-0.148807492	-0.364813873	0.175224182
Ints2	0.2411547	3.97E-85	0	0.010060402	0.088898572	0.174969361
Txn2	1	1.73E-32	3E-225	0.012259157	0.094219549	0.174736489
Ccnl2	3.8988E-43	1.86E-51	4E-125	0.078914226	0.174151328	0.174628669
Sinhcaf	1	0.00E+00	0	0.00205428	0.145918217	0.174493017
Rpl12	1.00924E-06	3.39E-283	0	0.015394473	0.184385885	0.174400896
Hspa9	1	2.02E-61	6E-179	0.017204869	0.150211196	0.174322322
Aen	1	2.04E-206	0	0.011011675	0.184424518	0.174204346
Stard5	1.07917E-15	8.75E-05	5E-160	0.047635265	0.056741453	0.173785962
Prep	1	4.60E-70	3E-253	-0.010313118	0.122465439	0.173721145
Xpo6	1	5.11E-26	5E-161	0.006228413	0.112714621	0.173543102
Tom1l2	0.009494903	2.02E-47	2E-110	0.031319921	0.166786194	0.173376939
Naa25	9.89185E-08	9.52E-31	2E-212	0.029372905	0.095227704	0.173163321
D10Wsu10	0.002323577	0.00E+00	0	-0.009451566	0.236357921	0.173160371
Rpl38	1	3.79E-163	7E-301	0.008392415	0.179479108	0.172965332
Lamc1	5.3723E-06	3.16E-35	2E-197	-0.022335218	0.109644589	0.172954274
Rsad1	1	8.20E-61	9E-300	0.003890314	0.103777965	0.17294842

Rps18	5.8203E-09	7.29E-120	0	0.023440717	0.149004666	0.17269961
Ublcp1	1	2.05E-90	1E-271	0.006165019	0.132306262	0.172618211
Alg14	7.08443E-11	4.86E-22	2E-158	0.037487699	0.100604558	0.172562979
Ccdc125	1	4.75E-61	2E-247	-0.002250134	0.123863315	0.172525958
Bcl2l1	1	2.54E-36	2E-102	-0.007344649	0.146093696	0.172479591
App	1	1.10E-226	7E-243	0.009853748	0.245394989	0.171997704
Clasp1	1	5.97E-49	1E-129	-0.010159552	0.159994489	0.171669257
Nkd1	1	1.93E-149	0	-0.000274377	0.073384477	0.171416961
Rplp1	1.06053E-10	0.00E+00	0	-0.019565794	0.244573867	0.171201862
Resf1	0.096508986	2.95E-36	3E-104	0.024322519	0.156471943	0.171150798
Eif4g2	1	5.24E-32	5E-174	0.016001422	0.114286124	0.171092829
Atp5b	0.087808663	8.97E-24	4E-206	0.021406295	0.084485276	0.170978321
Mcph1	3.25318E-15	3.48E-36	2E-181	-0.035975879	0.109178147	0.170888912
Dhps	3.61856E-07	3.93E-73	0	0.015741584	0.075266672	0.170733247
Igsf5	1	7.12E-14	6E-123	0.011828247	0.088557253	0.17066804
C2cd5	1	4.12E-43	8E-257	0.006621533	0.095168092	0.170537045
Zfp950	0.119078636	1.00E+00	1E-116	-0.025080016	-0.020130384	0.170465715
Pdpk1	1	2.22E-36	3E-135	-0.002836146	0.138274751	0.170457759
Btbd8	1	2.82E-165	0	-0.009220414	0.160199859	0.170196497
Terb1	1	3.57E-271	0	-0.002369223	0.141076557	0.170142222
Man2b1	1	1.36E-86	2E-301	0.006705801	0.12807863	0.169764925
A330015K0	1	1.33E-300	0	-0.000493346	0.116928267	0.169654171
Setd3	0.033716695	1.00E+00	7E-136	-0.021258961	0.011816747	0.169351884
Notum	1	1.08E-38	5E-278	0.002988506	0.080405968	0.169272761
Copb1	9.99124E-06	4.57E-29	8E-192	0.028316816	0.095452937	0.169267389
Pdss1	1	1.05E-141	0	0.00764006	0.113435275	0.169154032
Gm35190	1	0.00E+00	0	-0.000878867	0.162568093	0.169092727
Ppan	3.47013E-19	3.87E-107	0	0.02202771	0.092322978	0.169053319
Rps27a	1	7.78E-193	0	0.007594482	0.161994534	0.168754413
Dlg5	1	1.26E-136	0	-0.000163277	0.156586398	0.168367156
Nckap1	0.158609689	9.03E-40	2E-134	0.026061054	0.12853103	0.168165108
Dnm1l	1	1.00E+00	3E-161	-0.007545152	0.044127825	0.167745567
Knop1	0.001820554	1.41E-70	8E-251	0.019896051	0.12357324	0.167581687
Ryk	0.003970787	5.39E-129	6E-227	0.020022275	0.179802201	0.16755543
Psma1	1	8.00E-44	3E-281	0.011103276	0.086211805	0.167544911
Tshz1	7.53172E-15	1.00E+00	6E-95	-0.047383856	-0.002849076	0.167468624
Zmiz2	1	1.10E-75	0	0.007341932	0.092000076	0.167305577
Lpin3	1	1.61E-231	0	-0.001109794	0.1144718	0.166815167
Stradb	1	1.73E-47	8E-271	0.006248632	0.089340756	0.166688408
Cdkn1a	1.45088E-44	4.04E-120	0	0.046253354	0.134749388	0.166635848
Traf5	6.70354E-10	9.07E-64	1E-228	-0.027008715	0.125113872	0.166465971
Ndor1	1	4.25E-107	0	0.011610774	0.124506423	0.166064101
Zc3h7a	1	1.43E-61	5E-129	0.017334629	0.166984803	0.165983133
1700086O0	1	1.69E-152	0	0.00334766	0.13233715	0.165764509
Ap4b1	1	7.05E-203	0	0.008867588	0.14573681	0.165659036
Trrap	1	3.18E-18	2E-160	-0.010031793	0.093321734	0.165523659
Cebpz	8.15037E-09	9.37E-23	2E-197	0.031425089	0.079062026	0.165260896

Nyx	1	0.00E+00	0	9.6671E-05	0.16672296	0.164979546
Vps53	1	8.71E-01	1E-184	0.00136823	0.035285335	0.164960113
Zfp612	1	1.65E-57	3E-225	0.00122232	0.114376275	0.164616204
Scaf11	1.40795E-08	2.47E-71	6E-125	0.040282983	0.173948272	0.164594127
Dsg2	1	2.16E-07	1E-149	-0.006330928	0.061636905	0.164086304
Dis3l2	8.87417E-07	7.18E-66	6E-109	-0.036751825	0.177970225	0.163411835
Brwd1	1	4.52E-18	2E-109	-0.014074656	0.110020737	0.16328172
Rbm26	1	1.58E-34	5E-126	-0.019103436	0.128519511	0.163262387
BC005561	1	1.00E+00	1E-120	0.019200144	-0.00284829	0.163185035
mt-Co1	1	3.62E-124	2E-228	-0.012413039	0.183374212	0.163167356
Stxbp4	1	5.52E-31	4E-232	-0.001642559	0.081136705	0.163024095
Dvl1	7.42691E-19	1.33E-05	1E-184	0.043821427	0.051617138	0.162865828
Wdr26	1	1.51E-77	2E-126	-0.003240493	0.181783876	0.162576455
Rint1	1	2.18E-19	1E-198	0.017314155	0.076579718	0.162411146
Zfp445	3.65867E-49	1.00E+00	1E-117	-0.085121576	0.030130678	0.162360481
Dip2b	1	1.00E-89	3E-136	-0.009156553	0.188668669	0.162325903
Xkr9	0.548212602	8.11E-05	3E-144	0.018992144	0.061678141	0.162220403
Rpl28	1	2.70E-151	0	0.009132898	0.146326716	0.161840142
Tpd52	1	8.73E-115	4E-214	5.35935E-05	0.18552764	0.161593174
Ccdc122	1	6.26E-21	1E-261	-0.005070102	0.061900438	0.161504954
Washc4	1	5.93E-99	5E-210	0.010197007	0.163809822	0.161396437
Srpk2	1	1.51E-35	6E-118	-0.013119016	0.131205435	0.160876465
Cc2d1b	1	2.57E-168	0	0.003891126	0.145215047	0.160787444
Cdo1	0	1.00E+00	2E-64	0.35006423	0.062496187	0.16059965
Frmd6	1	1.30E-53	9E-252	-0.000113602	0.100652909	0.1603614
Taf1a	2.33445E-24	2.46E-75	4E-281	0.034609161	0.110099655	0.160314669
Map4	0.004420038	1.64E-154	4E-121	-0.026072174	0.262697753	0.160314393
Rars2	1	1.56E-37	8E-175	-0.00178466	0.107148911	0.160247221
Gm36388	1	0.00E+00	0	0.002373082	0.155612327	0.159824445
Anapc1	0.000912652	1.59E-27	2E-178	0.024929277	0.089626025	0.159710001
Wdr25	1	4.00E-54	2E-234	-0.005688285	0.10582527	0.159630403
Cep120	0.176527694	1.86E-72	3E-201	0.017060554	0.133584903	0.159481716
Ltn1	0.425759791	3.00E-28	4E-202	0.018601606	0.088026141	0.159292276
Gm41409	1	0.00E+00	0	-0.001108873	0.112277073	0.15926441
Dnajc10	1	6.17E-65	1E-264	0.003635599	0.102589031	0.159221536
Slc35a3	1	3.59E-10	1E-121	0.004371859	0.081619599	0.158679563
Snhg20	8.86885E-08	6.35E-47	1E-237	0.024459627	0.096645851	0.158677172
Luc7l	1	1.85E-40	3E-146	0.015737105	0.124128573	0.158514317
Hsph1	6.64444E-15	1.14E-30	2E-282	0.026435102	0.066940683	0.158474261
A230072E1	1	2.44E-267	0	-0.004139669	0.196703774	0.158432918
Rpl17	1	4.78E-166	5E-271	0.005212302	0.173021966	0.158280509
Spag1	1	6.52E-66	0	0.00662067	0.091194879	0.158260083
P4hb	7.96486E-21	1.00E+00	6E-149	0.05564866	0.007034103	0.15803853
Sptlc2	1	1.27E-113	2E-174	0.012269291	0.192382713	0.157897249
Srsf6	5.28899E-09	1.23E-04	1E-171	0.033297693	0.052673705	0.157867326
Atp7a	1	8.66E-66	5E-202	-0.009443455	0.13583129	0.157768331
Riok3	1	1.47E-25	3E-131	0.008710096	0.113004997	0.15775613

Ppcdc	1	1.94E-28	3E-210	0.011064616	0.08420949	0.157636559
Gtf2h1	1	2.60E-63	5E-200	0.015799688	0.127865033	0.157625089
Dalrd3	4.87666E-07	2.28E-68	0	0.016601878	0.083202004	0.157407897
Xpot	1.30004E-29	4.33E-67	1E-181	0.051870151	0.13727232	0.157181276
Usp37	1	4.94E-21	1E-182	0.012763892	0.078706062	0.15704704
Tjp2	1	1.00E+00	1E-81	-0.01278259	0.056335442	0.156893357
Arfgap2	0.583855924	1.21E-28	2E-215	0.015547477	0.081260412	0.156499341
Gm49980	2.7391E-126	4.43E-05	3E-98	0.145535636	0.058046178	0.156489457
Pik3r3	0.022601168	1.93E-218	0	0.006897208	0.100210133	0.156479612
Cd2ap	1	7.02E-53	7E-123	-0.012699766	0.14679573	0.156194306
Cbr3	1	0.00E+00	0	-0.000501901	0.124048102	0.156027728
Eef2	1	6.47E-52	2E-204	0.016233886	0.117022165	0.15581392
Ctnna1	7.3844E-28	5.90E-69	6E-102	-0.06524667	0.17892812	0.155687269
4931428L1	9.17022E-12	9.96E-50	1E-139	-0.03076178	0.138623338	0.155523657
Rpl6	1	1.62E-163	1E-273	0.010847077	0.168737246	0.155494406
Oplah	1	1.28E-52	1E-179	0.012012822	0.114755236	0.155388958
Lsg1	0.002839402	1.76E-19	2E-202	0.020316023	0.069845924	0.155270952
Ibtk	0.071183669	1.50E-08	5E-144	-0.018479968	0.066237399	0.15511013
Mvb12b	1	0.00E+00	0	0.002384344	0.146099893	0.155069132
Pfkfb3	1.47764E-06	1.27E-100	6E-261	0.017684952	0.125109236	0.155019656
Btaf1	3.33846E-06	2.22E-44	2E-125	0.033230636	0.139713536	0.154941331
Cyc1	3.48811E-24	9.60E-81	0	0.030405941	0.094946811	0.154743982
Tmem184b	1	4.90E-169	3E-257	-0.009429714	0.172269265	0.154468644
Hnrnpa2b1	1.12955E-06	5.22E-19	2E-94	0.035278734	0.118788178	0.1544518
Ankrd13c	1	8.63E-06	6E-100	0.011468074	0.063184998	0.154344951
Gsdme	1	6.90E-158	3E-263	0.009677506	0.160967521	0.154333268
Mia3	4.6506E-271	1.00E+00	6E-89	0.210174192	0.047085255	0.154308282
Ralbp1	1	3.36E-14	4E-179	0.009702428	0.070179941	0.15424766
Prpf8	6.66425E-12	2.54E-41	3E-242	0.028523876	0.086527644	0.154000108
Pip4p2	1	3.56E-68	1E-212	-0.007504885	0.125894829	0.153945834
Prune1	1	7.74E-80	3E-227	-0.007389138	0.12278248	0.15381015
Eif2s2	1.22833E-16	1.12E-20	1E-170	0.041263681	0.083158109	0.153748327
Pop1	4.14706E-17	4.55E-67	3E-282	0.029117296	0.09599896	0.153647882
Rnf123	0.347576133	2.76E-40	2E-181	0.017766742	0.103329267	0.153558414
Dhdds	1.11304E-07	1.77E-27	2E-202	0.025077842	0.079321514	0.153521608
Plcb4	0.072809521	0.00E+00	0	0.005206017	0.176260387	0.153247345
Phc2	2.80623E-05	2.34E-30	3E-124	-0.025506713	0.123421329	0.153142862
Hnrnpdl	4.39879E-45	1.02E-24	2E-141	0.072580066	0.103672402	0.153096078
Sf3b3	1	2.00E-38	1E-183	0.011698144	0.099566167	0.152892371
Senp2	0.000297235	3.25E-18	4E-173	0.02436139	0.077916805	0.152840206
Actn1	3.04131E-05	2.91E-23	4E-188	-0.020253695	0.086294369	0.152816194
Synpo	1	1.53E-91	0	-0.000713083	0.085000902	0.152780467
Ugt1a7c	1	2.72E-203	0	-0.001781629	0.124247551	0.152641884
Psmc3	8.97012E-06	1.95E-17	6E-267	0.01913151	0.055134104	0.152565829
St7	1	4.97E-04	4E-112	-0.008323578	0.058593359	0.152374281
Kcnab1	0.000337195	0.00E+00	0	0.009184132	0.145860424	0.152323053
Wwp1	6.21765E-06	2.40E-49	5E-92	0.034048701	0.181716883	0.152234409

Cemip2	1.4494E-16	3.79E-68	8E-275	0.025897943	0.107386129	0.152192248
Mapk8ip3	4.04025E-06	1.09E-15	6E-162	0.028189701	0.073783928	0.152128143
Wdr43	3.63009E-23	3.05E-47	2E-168	0.044216339	0.11330866	0.151947945
Ddx39b	1.02561E-29	7.03E-22	2E-131	0.060275312	0.098868717	0.151926449
Lipg	1	1.00E+00	8E-217	0.006062509	0.004502448	0.151794612
Gmnn	1	0.00E+00	0	0.003675667	0.144615649	0.151407655
Etnk2	5.57465E-33	4.42E-12	4E-66	0.080639576	-0.110101815	0.151349955
Birc6	2.60721E-06	3.39E-08	2E-96	0.033596295	0.098210707	0.151296853
Rasa2	1	2.57E-60	2E-201	-0.007297581	0.117414023	0.151222965
Cltc	1	1.83E-18	2E-115	0.013463402	0.108271662	0.151118582
Rfx7	0.537053171	1.52E-02	3E-82	-0.024856847	0.059925772	0.151070949
Tsc22d3	2.0414E-247	6.08E-15	6E-64	0.25813041	0.080959736	0.150729871
Ddx3y	1	1.64E-01	2E-121	0.009289238	0.047284851	0.150564189
Prkn	0.000419334	1.00E+00	3E-108	-0.033728326	0.009335959	0.150442312
Acer2	1	1.21E-79	0	0.005230396	0.082192214	0.150373142
Pinx1	1	9.51E-68	5E-274	0.00927368	0.099101224	0.150243638
Psmd14	0.987929835	1.00E+00	4E-120	0.020564432	7.85984E-05	0.150060348
Rps19	1	7.93E-219	5E-277	0.003194974	0.189076468	0.149938083
Vars	1.68726E-05	5.08E-166	0	0.013068046	0.116806615	0.149493962
Rpl19	1	7.07E-166	4E-295	0.009956103	0.153734193	0.14943789
Anapc10	1	7.77E-33	4E-180	0.006753598	0.092714846	0.149167095
Araf	1	1.27E-27	1E-164	0.014689404	0.088717846	0.149081821
Mcts1	1	1.90E-26	1E-172	-0.001318324	0.083296222	0.148916082
Safb	3.06959E-06	1.50E-42	1E-103	0.036505335	0.135738039	0.148891155
Plaa	0.009829452	7.57E-25	1E-147	0.023064717	0.091610411	0.148743829
Sema4d	1	0.00E+00	0	-0.000435978	0.195100482	0.148740818
Prkag1	3.73407E-05	1.65E-31	8E-186	0.024426712	0.089466586	0.148552722
Wdr70	1	9.68E-01	6E-108	-0.002761995	-0.020899744	0.148522372
Slx4ip	0.066900873	2.76E-22	6E-145	-0.019059538	0.094877718	0.148436352
Kansl2	2.26846E-06	7.99E-61	2E-161	0.025758166	0.131769148	0.148246995
Rtn4ip1	1	1.71E-60	4E-256	-0.001212605	0.093934743	0.148216951
Arhgef5	1	1.59E-49	7E-244	0.004531875	0.086753657	0.148121558
Gpatch2	0.621380414	9.88E-07	3E-98	-0.023961913	0.067424587	0.148115176
Pcm1	1	8.94E-63	8E-103	0.007300711	0.166611557	0.14791497
Zfp943	1	7.44E-36	3E-185	0.003521441	0.090790489	0.147711946
Map3k20	0.000140173	9.28E-29	5E-96	-0.029177647	0.125377252	0.147477407
Gm49961	2.01383E-16	2.99E-12	3E-209	0.030469289	0.04883281	0.147316471
Zfp282	1	8.28E-47	6E-225	-0.002588724	0.090727068	0.14717369
Rpl9	7.99425E-06	1.28E-116	0	0.016430845	0.121206048	0.147168932
Ttpa	1.5839E-205	1.00E+00	7E-87	0.186521603	0.000138686	0.147104697
Ddx5	1	1.12E-06	9E-86	0.018038528	0.099420884	0.14707509
A730090N1	1	8.01E-226	0	0.000170653	0.08508923	0.146874607
Tat	2.8034E-212	2.97E-56	2E-24	0.322822153	-0.251333196	0.146682791
Gmds	0.012371555	1.08E-129	0	0.010616122	0.114201329	0.146595908
Retreg1	1.7762E-79	4.21E-01	4E-86	0.113811041	0.057150989	0.146585723
Huwe1	4.19344E-34	6.48E-03	6E-84	0.069843718	0.072502029	0.146362897
Rpsa	1	2.71E-192	1E-225	-0.006957265	0.196403688	0.146055086

Cops7b	1	7.09E-04	4E-168	-0.009831552	0.04366354	0.146053527
Nek6	0.549047997	6.47E-42	3E-139	-0.015369301	0.122021368	0.145975174
Bsg	1	3.89E-45	3E-242	0.004273605	0.086019719	0.145873741
Naa15	1.03892E-08	5.14E-32	8E-124	0.035511714	0.116494122	0.145404389
Hspa5	1	1.00E+00	1E-106	0.013324221	0.00546798	0.145323257
Rsrp1	0	1.23E-47	8E-68	0.255584519	0.192064162	0.145072697
Slc16a11	1	2.49E-164	0	0.002254745	0.086343067	0.144907397
Brca2	1	1.25E-77	7E-259	-0.00696405	0.102249725	0.144690034
Dido1	1	2.58E-25	2E-95	0.007660922	0.10553121	0.144656378
Dcaf8	0.002097069	2.89E-21	9E-96	0.027640107	0.107992261	0.144644365
Prim2	1	9.82E-73	8E-224	-0.001868637	0.107517454	0.144628341
Ugp2	2.62044E-63	8.17E-13	9E-35	-0.138504801	0.134296674	0.144520177
Sigirr	1	8.35E-273	0	0.001768432	0.124266332	0.144460134
Eif1	1.62855E-27	5.34E-20	4E-175	0.045594114	0.074448588	0.144328117
Rhbdf2	1	1.15E-123	1E-212	0.002055969	0.151057983	0.144161127
Ddah1	0.001734165	6.32E-07	2E-39	-0.045210387	0.11609066	0.14381603
Rab3gap2	1	7.73E-18	3E-156	0.009533943	0.084540538	0.143794591
Copg1	4.78828E-12	5.35E-54	8E-277	0.023156068	0.079653534	0.143791695
Baiap2	2.5155E-114	4.54E-49	2E-74	0.137750509	-0.130960767	0.14375862
Uvssa	1	6.27E-27	1E-156	0.013958562	0.087049356	0.143474375
Zmynd12	1	5.77E-170	0	-6.52881E-05	0.102682856	0.143185796
Gm47990	1	0.00E+00	0	0.001419472	0.102626229	0.142981516
Ddx1	1	1.26E-28	2E-220	0.011994474	0.070138072	0.14282442
Helz	1	6.67E-02	1E-110	0.01003092	0.047501422	0.142782805
Trmt13	2.05192E-05	6.03E-22	7E-169	0.023649525	0.074603803	0.142751896
Lrif1	1	4.58E-74	7E-224	-0.00617319	0.114320628	0.1426502
2810403D2	8.27906E-10	2.25E-37	3E-154	-0.026102372	0.106123292	0.142626655
Grpel2	1	2.25E-18	5E-151	-0.000851854	0.073485307	0.142484505
Ssr4	1.03968E-13	7.04E-34	0	0.018261592	0.046649959	0.142341669
Gm14296	0.000235119	2.96E-29	2E-194	-0.016935981	0.078332914	0.142340593
Sars	1	2.50E-54	3E-211	0.010050321	0.098243397	0.142159718
Aldh1a7	1	6.00E-27	4E-145	0.009043428	0.091774983	0.141668381
Tmem87b	1	6.13E-45	2E-147	0.002341602	0.121417033	0.141638673
Nsdhl	1.62426E-63	4.82E-48	6E-151	0.068682633	-0.067434125	0.141581734
Gart	1.90439E-10	1.15E-87	6E-250	0.023580737	0.10880977	0.141504831
Thumpd3	0.248111669	2.84E-98	2E-201	0.014600412	0.137503015	0.141431957
Uggt1	4.23759E-19	2.75E-03	2E-131	0.048160567	0.044897712	0.141153441
Axin2	1.60411E-09	1.06E-88	0	0.016510954	0.083021452	0.141025511
Gne	0	3.65E-22	2E-77	0.281809796	0.107221447	0.140891084
Ppl	1	2.57E-214	6E-283	-0.007004048	0.155310689	0.140863817
Dnajc11	1	1.36E-04	3E-98	0.011518613	0.057891713	0.140848316
Rangap1	1	4.68E-70	6E-198	0.011165111	0.11237396	0.140726509
Ctps	1	3.61E-268	0	0.003851954	0.119476339	0.1406302
Sap30	1	4.02E-269	0	0.000583494	0.089777189	0.140567614
Zbtb11	2.20978E-08	1.51E-16	1E-95	0.037946542	0.094467072	0.140518329
Rps9	1	5.85E-95	2E-220	0.003521862	0.128153316	0.140467869
Cwc27	1	1.00E+00	3E-97	0.010183233	0.032746059	0.140421188

Plekhm2	0.01588989	4.55E-92	3E-150	0.020347001	0.197074414	0.140407521
Apoc4	1.05046E-37	2.99E-12	6E-89	0.072396746	-0.052356251	0.139867417
Brd8	2.20478E-08	3.26E-48	6E-162	0.029043793	0.109948172	0.139857268
Slc25a28	1	3.00E-76	1E-206	0.000670824	0.113861671	0.139519922
Cpped1	7.32115E-09	1.14E-09	3E-77	-0.041432341	0.076190003	0.139498099
Trappc12	1	2.07E-50	4E-179	0.005584885	0.103205468	0.139479686
Gm34654	0.002113204	2.78E-127	0	0.011222644	0.094178419	0.139368788
Ubac2	1	8.77E-04	4E-84	-0.01238505	0.053407413	0.139266085
Tex10	1	1.00E-124	7E-244	0.008376095	0.135023654	0.139263203
Ago2	1	4.59E-27	3E-144	0.009364632	0.100018626	0.139186471
C130080G1	1	0.00E+00	0	0.000404834	0.149926934	0.139005304
Vrk2	1	6.78E-60	9E-126	-0.012229287	0.151575965	0.138566669
Grb2	1	1.49E-98	3E-87	0.020403011	0.214502012	0.138277596
Tmem120b	1	3.06E-99	6E-252	-0.000210327	0.111248234	0.13801297
Eipr1	1	7.34E-13	7E-180	-0.004874728	0.055353968	0.137988434
Cyp4a31	2.29837E-44	2.07E-53	0	0.034518014	0.062827314	0.137972749
Slc1a4	1	3.24E-302	0	-7.17363E-05	0.113442038	0.137963431
Elmsan1	0.000621209	6.14E-11	2E-111	-0.022438544	0.077479857	0.137949771
Rnh1	1	6.08E-30	8E-117	-0.006796486	0.105602313	0.137930764
Ell	1	1.00E+00	6E-91	-0.018580697	0.038364205	0.137642131
Hltf	1	9.00E-113	4E-225	0.001875714	0.135204842	0.137600798
Rhbdd2	1	1.21E-68	9E-277	0.003648799	0.085710901	0.137491512
Nid1	1	4.52E-204	0	0.002044156	0.074336568	0.137361887
Hook2	0.000407391	2.64E-33	4E-114	-0.024206451	0.11319391	0.137295165
Git2	1	2.56E-15	1E-104	0.019407533	0.094159117	0.137111169
Sys1	1	6.76E-70	4E-236	0.00914642	0.097875543	0.136841303
Sec16b	0.546671848	1.00E+00	1E-85	0.02333174	-0.016142735	0.136815863
Zfp407	1.75315E-26	1.57E-31	8E-62	0.069639812	0.162683922	0.136742245
Rps29	1	8.00E-238	5E-268	-0.001009897	0.188804547	0.136665018
Prpf40a	0.008757378	1.00E+00	6E-97	0.025972906	0.033142818	0.136520226
Ucp2	0.015447404	0.00E+00	0	0.003027645	0.088830717	0.136475115
Prox1os	5.65507E-45	7.18E-04	5E-30	0.103094468	0.10659594	0.136457589
Clec16a	1.75208E-07	8.08E-04	9E-85	-0.032360281	0.062462131	0.136421382
Hspa4l	1	1.31E-29	2E-242	0.003449717	0.057812574	0.136387164
Tfdp1	1	6.62E-43	4E-178	0.001899472	0.095078648	0.136227733
Atp6v0b	3.39876E-16	1.22E-96	2E-128	0.041506817	0.189903259	0.136047533
Rbm5	3.31772E-11	6.13E-35	6E-83	0.044502851	0.133115944	0.136018529
Unk	1	2.66E-36	1E-161	-0.00676846	0.09125201	0.135984884
Hnrnpl	3.87786E-20	1.05E-15	3E-114	0.050450293	0.081403113	0.135940313
Acot3	3.18235E-23	1.01E-204	0	0.009337259	0.062729507	0.135932737
Gm47818	3.65834E-20	9.24E-254	0	0.020676746	0.145266841	0.13591182
Sympk	2.66151E-05	4.94E-34	2E-172	0.022556698	0.08384354	0.135880379
Sbds	1	3.96E-67	4E-191	0.004392974	0.110607878	0.135837898
Rnf43	1	1.00E+00	2E-92	0.020778039	0.033850703	0.135816996
Irak1	0.088176793	1.81E-32	2E-189	0.014916843	0.082524959	0.135697845
D1Ertd622	1	1.34E-161	1E-174	0.006779827	0.198153709	0.135687738
44450	1	5.33E-88	2E-257	-0.002456463	0.105845882	0.135599262

Dnm2	0.015869492	3.06E-86	9E-95	0.027982612	0.192263401	0.135514227
Tmem140	0.002577606	1.84E-84	9E-164	0.018307017	0.144775802	0.135478473
Phip	1	1.09E-106	6E-79	-0.019065828	0.234322338	0.13522803
Ttc14	1	7.36E-05	1E-104	0.019715301	0.062621154	0.135146091
Cenpc1	1	4.10E-37	4E-238	-0.003946915	0.07005471	0.135080799
Lrrc4	1	1.45E-120	5E-129	0.01403987	0.199153325	0.13497752
Rps12	1	4.35E-192	3E-274	0.007414325	0.155873814	0.134875421
Stx18	1	6.56E-03	8E-112	0.017397873	0.045397758	0.134684261
Gm15441	2.1366E-42	2.81E-57	0	0.027875758	0.051786858	0.134574295
Gcfc2	1	5.50E-42	3E-213	0.002774819	0.081359151	0.134558001
Mlkl	1	3.12E-143	0	-0.001066979	0.088881132	0.134523509
Eef1g	2.99127E-07	8.33E-40	4E-200	0.02207725	0.08248573	0.13447566
Myl12b	1	8.67E-43	5E-152	-9.81762E-05	0.099745144	0.134470753
Tug1	1	7.78E-19	1E-177	-0.008550819	0.064343986	0.134452118
Xylt2	1	3.52E-35	3E-218	-0.008773784	0.071213554	0.134314186
Gm26871	0.957131324	1.00E+00	1E-120	-0.012785205	0.02520488	0.134252738
Gm14403	5.79076E-30	1.32E-73	0	-0.021750797	0.07420192	0.134148638
Rab24	7.09171E-05	1.60E-55	3E-224	0.018380857	0.092435376	0.134118033
Ikbkg	1	3.83E-11	4E-92	-0.005174672	0.080238534	0.134008525
Nprl3	1.98706E-05	2.47E-63	1E-159	0.023641375	0.119392176	0.133973672
Colgalt1	1	2.33E-100	1E-224	-0.0047924	0.126060168	0.133925848
Abhd2	1	1.00E+00	2E-123	0.005813551	0.020048849	0.133905068
Tert	1.24635E-33	1.88E-71	2E-232	-0.029691527	0.095742307	0.133881303
Tm9sf4	1	2.81E-16	1E-150	0.011724788	0.070618479	0.133870314
Tgfbrap1	1	1.83E-67	5E-201	0.005740585	0.103731937	0.133682919
Dnaja2	1	9.22E-17	7E-124	0.008632152	0.076209249	0.133418671
Mysm1	1	2.89E-27	4E-112	0.018001202	0.101617003	0.133310647
Gpat3	1.13086E-34	9.18E-42	7E-147	0.042107024	0.097683041	0.132957085
Tcirg1	1	1.51E-176	2E-171	0.006657547	0.23159424	0.132714224
Sptan1	1	2.71E-05	6E-88	-0.007032829	0.064326004	0.13271155
Gm49959	1	0.00E+00	0	0.002041281	0.111322192	0.132690158
Pabpc4	1	7.81E-46	2E-155	0.006662647	0.100541003	0.132636954
Dap	1.6357E-156	1.00E+00	1E-61	-0.146632311	0.042253094	0.1325374
Pabpc1	1	7.91E-54	1E-120	0.009297722	0.127711576	0.132305948
Pex11a	5.4593E-124	1.00E+00	8E-94	0.127362669	0.025657206	0.132263691
Yme1l1	1	1.06E-26	2E-112	0.00127015	0.098733052	0.132245165
Rnf14	1	2.92E-40	1E-138	-7.15876E-05	0.103869896	0.132137274
Myef2	1	1.03E-72	2E-174	-0.003869247	0.119478635	0.132133336
Tnpo2	1	2.98E-88	2E-188	0.013003952	0.128235518	0.132073017
Pdgfc	3.73199E-12	2.28E-128	1E-84	-0.043803313	0.270649195	0.132010308
Iars2	1	2.83E-14	8E-93	0.011413532	0.083309493	0.131977027
Msmo1	3.91698E-87	1.34E-47	8E-70	0.115986369	-0.09647108	0.13197637
Mtpap	1	3.52E-44	1E-171	-0.000499622	0.092811329	0.131938192
Xpo5	1	5.17E-66	3E-214	0.006818694	0.098208299	0.131918552
Exosc10	0.020369376	3.95E-32	7E-180	0.016683091	0.077172151	0.13186514
Thoc1	1	8.06E-18	3E-135	0.006823141	0.072554877	0.131858457
Zfp160	1	2.89E-15	3E-144	0.002867058	0.064916342	0.131781155

Per2	1	2.88E-158	0	-0.002640821	0.103369544	0.131485958
Ttc39a	1	0.00E+00	0	-0.003424793	0.134847314	0.131446782
Tars2	1	1.06E-66	3E-252	0.007273735	0.086766206	0.131301254
Mrps7	1	3.16E-48	3E-283	0.006221311	0.066330013	0.131214179
Ddx24	1.09811E-05	4.03E-13	6E-141	0.025290769	0.064771325	0.131199088
Med25	1.80551E-07	8.02E-70	8E-275	0.017077957	0.080987312	0.131191081
Rev1	1	2.41E-07	7E-92	-0.005525385	0.068143654	0.131106927
Chchd3	8.10349E-27	1.58E-03	2E-74	-0.07367278	-0.04013959	0.130940468
Nol10	2.23187E-10	2.46E-59	2E-194	0.024791453	0.096434877	0.130818448
Ndufa10	1	1.21E-28	1E-165	0.006894353	0.076351938	0.1305372
Lekr1	1	3.39E-246	0	-0.000768505	0.09814581	0.130504252
Vcp	0.000125305	1.00E+00	8E-78	0.032643862	-0.017897059	0.13030264
Micu3	1	2.58E-35	1E-139	0.006347444	0.096928929	0.130279348
Smad7	4.7085E-127	2.59E-31	7E-127	0.103251717	0.096169014	0.1302375
Dctn6	1	1.01E-49	4E-246	0.003767421	0.073038241	0.130194708
Ercc6l2	1	2.57E-28	3E-126	7.07235E-05	0.090195895	0.130172631
Gm1976	0.003691285	2.25E-28	6E-159	-0.016792748	0.076279447	0.130130395
Bmp6	1	4.60E-92	0	0.000245998	0.060027616	0.130037094
Cars2	0.705476357	1.81E-22	7E-187	0.013491547	0.060998225	0.129986781
Htt	1	1.09E-03	4E-136	0.014659698	0.044101651	0.129931453
Dtd1	1	1.87E-15	1E-104	0.013381296	0.080540024	0.129897114
Rassf6	1	5.27E-76	1E-219	-0.004158791	0.100033951	0.129784653
Ddx21	0.125063842	2.25E-97	3E-190	0.014392255	0.126536389	0.129483539
Rexo1	1.90666E-35	6.18E-06	1E-128	0.056608697	0.046963792	0.129434016
Cars	1	1.33E-15	3E-147	0.004716343	0.063839039	0.129382528
Rpl37	1	4.21E-135	5E-259	0.008397754	0.129937492	0.129320891
Pign	5.46397E-08	1.00E+00	3E-84	-0.032868208	0.043336946	0.129260008
Rarb	1.29077E-34	5.31E-04	4E-161	-0.033408187	0.044277693	0.129054163
Fem1b	1	3.80E-43	1E-217	0.002119391	0.072860924	0.128954981
Srsf4	3.06131E-43	1.47E-11	1E-69	0.084378508	0.086795214	0.128767767
Golga5	5.96572E-08	2.82E-12	2E-121	0.03196677	0.066819193	0.128705544
Secisbp2	3.81683E-17	3.67E-17	9E-126	0.04022889	0.07263376	0.128525907
Elp1	1	1.16E-23	2E-173	0.01323397	0.065487443	0.128498895
Gfm1	1	1.29E-21	1E-170	0.004193224	0.063539417	0.128411014
Ppp4r3b	0.308075025	2.68E-23	9E-98	0.021493247	0.099622259	0.128345593
Rcan3	1	0.00E+00	0	-8.55817E-05	0.109018026	0.128325065
Gm32063	1	7.95E-02	2E-103	0.011664359	0.043714596	0.128213966
Gak	0.005878937	8.45E-09	6E-100	0.024704904	0.06887926	0.127987463
Nfkb1	0.017381114	9.85E-33	3E-78	-0.022406731	0.124143946	0.127853021
Enc1	0.015737565	2.33E-177	0	0.005289643	0.077687497	0.127814296
Gulo	5.6845E-25	4.12E-11	7E-80	-0.051401261	0.081394678	0.127722272
Ddx50	3.31469E-13	9.11E-06	6E-103	0.042412029	0.059060667	0.127696976
Cwf19l2	1	9.56E-02	7E-75	0.007541995	0.049103052	0.127637893
Mettl1	4.55988E-06	3.38E-76	1E-285	0.014956991	0.077395025	0.127626711
Vipas39	1	2.17E-21	4E-167	0.009815204	0.062503784	0.127611808
Smarca4	1	1.00E+00	2E-106	0.018732721	0.025416499	0.12757771
1110038B1	1.8966E-13	2.33E-166	0	0.015257235	0.098142203	0.127512113

Dus2	1	1.59E-73	8E-211	0.006223368	0.10166328	0.127428055
Tmem39a	1.19978E-09	1.00E+00	5E-158	0.02882707	0.023363047	0.127409262
Nol9	0.006378553	4.72E-90	3E-244	0.013518302	0.09844821	0.127263376
Spred1	1	1.19E-69	1E-120	-0.000240022	0.14359969	0.127247337
Csnk1g3	1.85118E-27	1.00E+00	2E-76	0.064056465	0.001735344	0.127211668
Tgfb2	1	0.00E+00	0	-0.000612327	0.103108683	0.127109775
Zfp672	1	2.02E-15	5E-177	0.006319634	0.054213757	0.127099906
Klf6	1	0.00E+00	0	0.002507646	0.148164517	0.127072774
Eif3a	2.2121E-33	1.46E-24	2E-73	0.072734513	0.11008757	0.12698931
Dock7	0.000184718	1.46E-27	6E-75	-0.027672314	0.1175396	0.126829432
Cux2	1	1.00E+00	0	-3.20494E-05	0.005708421	0.126809579
Cct6a	1	2.66E-38	2E-178	0.007232683	0.083225706	0.126734037
Vldlr	0.000126772	2.97E-137	0	0.006008951	0.061735617	0.126694015
Fau	1	1.09E-88	4E-170	-0.001399694	0.134397355	0.126670617
Uqcrc2	1	2.11E-08	3E-117	0.004341595	0.0555878	0.12661798
Nek9	9.12521E-07	3.15E-21	1E-132	0.027289741	0.07826122	0.126529899
Blmh	1	2.06E-02	3E-124	-0.009796219	0.039790574	0.126483631
Uba3	0.02370026	2.03E-46	6E-157	0.017032086	0.096866452	0.126394125
Tfb2m	1	2.70E-57	1E-235	0.009738352	0.07741721	0.126372253
Gpi1	1	2.60E-20	4E-119	0.004307631	0.074847751	0.126361948
Khdc4	0.050910544	4.38E-04	1E-79	0.025482566	0.059617921	0.126336119
Tmem248	1.56896E-09	3.07E-07	5E-114	0.034149579	0.056468191	0.126199212
Rplp2	0.105837148	2.23E-141	3E-221	-0.012053182	0.142297264	0.126194733
Crybg2	1	4.70E-264	0	-0.002777408	0.097682763	0.126114075
Atp6v1h	1	9.89E-26	4E-92	-0.002779021	0.109166108	0.126059702
Tubgcp2	1	2.75E-47	3E-237	-0.005656124	0.071333434	0.126055886
Mta3	0.005622233	2.17E-32	4E-136	-0.016748117	0.092878244	0.126040805
Tbc1d9	1	0.00E+00	0	-0.000486686	0.128078315	0.126010363
Rnf128	0.001352344	4.06E-16	9E-93	-0.023027015	0.089924861	0.125922431
Lpp	1.823E-53	1.88E-71	6E-57	-0.096842698	-0.168580377	0.125918838
Rpl34	6.25544E-05	4.31E-127	3E-230	0.015632694	0.12621022	0.125918336
Phrf1	6.33057E-11	1.95E-10	6E-111	0.035848713	0.063335284	0.125905654
Gmeb1	1	3.01E-73	2E-136	0.005342494	0.135257656	0.125813566
Spata17	1	3.11E-25	9E-269	-0.001283232	0.045124778	0.125777474
Eif2b3	0.252113331	2.09E-46	1E-152	0.015041051	0.09676267	0.12571396
Sec31a	1.77141E-16	1.00E+00	3E-73	0.055099358	0.024033861	0.125618948
Tnfrsf19	1	1.62E-167	0	-0.002803772	0.074214365	0.125593213
Klhdc2	1	7.90E-53	2E-213	0.002715713	0.081809251	0.125514196
Ide	1	1.17E-01	1E-101	0.011880152	0.039132279	0.125478302
Abcb7	0.009040579	1.00E+00	2E-73	-0.026411786	0.024435553	0.1254039
Arpp19	1	1.63E-40	2E-140	0.008771576	0.101769365	0.125253713
Anxa7	1	8.65E-63	5E-164	0.002870367	0.109225916	0.125187655
Snhg15	2.18764E-05	5.83E-205	0	0.008360662	0.092218135	0.125140378
Trp53	1	2.33E-74	8E-196	0.011380171	0.106208016	0.125095916
Elmo1	3.0154E-17	2.77E-110	3E-64	-0.041715199	0.296365539	0.125012695
Tuba8	1	0.00E+00	0	-0.00021066	0.079739338	0.124964059
Kif26b	1	1.18E-229	0	-0.000187973	0.102373657	0.124893715

Rps28	4.49773E-06	1.83E-167	7E-248	0.016041502	0.141334181	0.124797343
Pard3bos3	0.000999388	1.15E-275	2E-231	-0.007703692	0.193921033	0.12479526
Lmbr1	1	6.15E-14	1E-81	-0.011424918	0.082153696	0.124791622
Hspa8	0.000134048	1.00E+00	2E-108	-0.020521418	0.038582869	0.124581
Tle1	1.64782E-37	6.97E-19	6E-49	0.08376371	-0.089195821	0.124577953
Sec24b	1	3.88E-08	6E-97	0.019381894	0.066941771	0.124501693
Dglucy	3.19089E-17	1.69E-12	2E-60	-0.048394932	0.099008701	0.124475935
Capn10	8.84553E-07	1.57E-29	2E-217	0.01803828	0.058402661	0.124368828
Snx24	1.56432E-06	4.70E-131	2E-47	0.041751608	0.302468082	0.124335171
Ice2	1	6.09E-22	9E-138	0.001385554	0.071617826	0.124308895
Mipep	1	1.31E-21	4E-162	-0.008788438	0.063548719	0.124287094
Slc30a7	1	4.34E-26	1E-124	0.004343013	0.08624784	0.124284738
Cep128	1	9.24E-61	2E-111	0.009377883	0.141469397	0.12428455
Gpx1	1.40555E-23	3.43E-09	1E-112	-0.042321602	0.07143483	0.124261861
Map2k2	1	1.26E-07	5E-94	0.012169517	0.062722088	0.124195253
Nup153	1	7.98E-67	4E-135	0.01184201	0.128642791	0.124142414
Cfi	1.91005E-07	1.00E+00	1E-87	-0.031218774	0.031448847	0.124048542
Clint1	1	2.40E-27	7E-64	0.019696684	0.120478603	0.124047815
Srsf7	5.08878E-42	3.69E-41	2E-141	0.055522051	0.102352661	0.124040922
9530046B1	1	4.93E-50	8E-257	-0.000533593	0.066764747	0.12394636
Csde1	1	1.57E-02	6E-89	0.008984399	0.050840256	0.123883989
Eefsec	2.06311E-07	1.17E-64	2E-60	-0.03797272	0.183522182	0.123804081
Hectd4	1	1.66E-01	3E-74	-0.000918974	0.04821385	0.123724644
Xndc1	1	3.97E-49	3E-183	0.009135288	0.088325776	0.123647457
Usp16	3.47152E-09	1.70E-11	5E-115	0.032596513	0.062451711	0.123580307
Mettl27	1	5.02E-58	2E-185	-0.008800023	0.091236423	0.123528898
Zfp942	1	1.00E+00	2E-137	0.007288495	0.024398656	0.123510824
Pdhb	6.45506E-06	1.95E-13	3E-158	0.022539883	0.052052906	0.123428369
Exoc4	1	3.03E-29	1E-48	-0.004485052	0.14620633	0.123390257
Nudt5	1.67842E-07	1.69E-53	2E-190	0.020844153	0.086352153	0.123320581
Supt16	3.97314E-05	1.90E-18	4E-103	0.028639092	0.082827775	0.123311663
Psmd13	1	1.49E-16	1E-177	0.007796987	0.055597061	0.123263026
Slc16a5	4.16562E-11	5.33E-96	0	0.011575152	0.059053457	0.123149273
Osbp	0.005385263	2.04E-01	4E-107	0.022570885	0.035064568	0.123019771
Pot1b	1	1.37E-92	5E-207	-0.001203047	0.112909654	0.122985721
Rbm47	1.8118E-119	3.45E-83	2E-62	-0.147906206	0.214447335	0.122830181
Banp	1	4.79E-04	2E-69	2.59144E-05	-0.037829061	0.122829959
Hexa	1	1.32E-256	4E-213	-0.006213995	0.210277697	0.1227651
Mettl7a1	1	5.92E-17	7E-102	0.017453559	0.078345907	0.122692204
Ylpm1	7.36336E-07	4.23E-47	2E-68	-0.033391578	0.144572739	0.122650632
Cdkn1b	1	2.53E-57	6E-236	0.007159154	0.077575552	0.122642545
Rpl23	1	1.11E-145	9E-201	-0.00040821	0.151224986	0.122425754
Tep1	1	3.80E-90	9E-175	-0.00023547	0.129015815	0.122372642
Rtn4	0.000104561	2.34E-194	4E-156	0.017706412	0.217480679	0.122242466
Nupl2	1	2.24E-40	8E-196	-0.002677902	0.072400968	0.122143421
Mbtps2	0.487966781	9.53E-51	5E-145	-0.011740271	0.10407272	0.121855276
Pde7b	0.005351222	2.87E-18	4E-31	-0.040227127	0.113492704	0.121741322

Zfp692	1	1.25E-28	6E-229	0.009520922	0.053648106	0.121725671
Angptl3	1.65755E-97	1.00E+00	5E-74	0.106550541	0.009952785	0.121687887
Snhg17	0.110221193	1.99E-69	8E-286	0.009565927	0.069739213	0.121673653
Nom1	1	8.71E-12	2E-136	0.002384416	0.052624134	0.121493149
Cyp2c55	0.01719594	6.30E-73	5E-290	0.008005814	0.063653852	0.121351743
Med27	0.010492991	1.00E+00	1E-71	0.028425446	0.0211188	0.121264926
Suds3	0.047411745	5.10E-36	3E-107	0.022285973	0.104940208	0.121258598
Atp5d	0.000429604	4.84E-30	3E-200	0.017063387	0.062960975	0.12117566
Csnk1a1	1	2.53E-45	1E-67	0.001824285	0.140394339	0.120987643
Med13	1	1.78E-17	2E-52	0.014882371	0.102974794	0.12079172
Mtor	1	1.00E+00	6E-83	-0.008881781	0.018042604	0.120780149
Macf1	1	3.41E-16	3E-63	0.007768012	0.140026117	0.120730021
Rps15	1	7.43E-61	2E-186	0.000791471	0.096385831	0.120726742
Recql5	1	4.60E-02	2E-126	0.011655022	0.035436889	0.120633265
Fbp2	0.0033414	2.68E-151	0	-0.003042432	0.062324721	0.120551663
2410006H1	1	9.99E-180	0	0.00637785	0.113594279	0.120516535
Gm26944	1	4.47E-73	3E-193	0.003005152	0.098370039	0.120441004
Rad18	1	5.37E-117	3E-248	0.005295423	0.106121379	0.120337843
Cherp	0.000877654	2.24E-34	2E-154	0.018308767	0.078441197	0.120298828
Junos	1	2.79E-223	4E-258	0.001327839	0.142999136	0.12015667
Cse1l	1	4.31E-59	4E-181	0.008533757	0.090396963	0.120145724
Rplp0	1	1.03E-182	8E-287	0.0004934	0.126887933	0.120007832
Uspl1	0.001652076	1.68E-58	4E-185	0.015870326	0.088280351	0.119916067
Usp24	1	7.60E-39	2E-70	0.005664497	0.124504024	0.119793474
Brd7	1	9.64E-14	8E-123	0.013720882	0.064966709	0.119738479
Fastkd1	0.130124318	1.82E-30	6E-156	0.015831593	0.071428344	0.119709736
Rps27	0.292362407	2.13E-153	3E-207	0.011511104	0.143133079	0.119704154
Rell1	3.15656E-13	7.99E-06	3E-80	-0.033561606	0.062590273	0.119683703
Abca1	3.7389E-113	2.93E-21	1E-44	-0.141982632	0.158828571	0.119660894
Galnt11	1	5.45E-58	5E-123	-0.010730081	0.117392278	0.119632315
Aldh3b1	1	0.00E+00	0	-0.001763193	0.124892614	0.119153962
Sec11a	1	2.17E-07	5E-86	0.013396884	0.063243422	0.11911056
Rps24	1	2.27E-135	3E-193	0.002865042	0.140838705	0.119104816
Asph	1	1.00E+00	2E-65	-0.001418989	0.018856348	0.119037317
Rpl4	1	2.31E-72	1E-244	0.006005549	0.085885088	0.118964555
Cmip	0.000428857	6.09E-10	2E-30	0.0392257	0.141834595	0.118935733
Cfb	0.030756727	2.80E-26	1E-133	-0.014418795	0.076609904	0.118894414
Med12l	1	1.00E+00	4E-72	-0.015532526	0.041138721	0.118740841
Sdhb	1	4.09E-15	1E-107	0.005748763	0.069552083	0.118569314
Ufl1	2.27855E-11	1.29E-07	9E-103	0.034926779	0.052821261	0.11844739
Ldha	1.58967E-06	9.27E-30	1E-81	0.032887666	0.110366312	0.118415439
Usp19	1.16031E-19	1.27E-12	8E-133	0.037090731	0.053733019	0.118387371
Zfp970	2.74316E-59	1.00E+00	7E-111	-0.061558965	-0.009391103	0.118353499
Lgmn	1	0.00E+00	3E-172	0.003088893	0.377215538	0.118229636
Papss2	4.31927E-73	2.54E-11	9E-30	0.109604063	-0.047014816	0.118192087
Vps35	0.626068955	2.12E-46	3E-152	0.013827325	0.097834481	0.118123133
Gpnmb	1	0.00E+00	0	-0.000604814	0.395087036	0.117996961

Gca	1	7.24E-97	4E-271	-0.003247681	0.086008384	0.117966003
Nsf	1	1.48E-30	3E-81	0.018629748	0.11935674	0.117891556
Rpl27a	1	1.36E-127	2E-201	0.005668361	0.133246943	0.117879916
Dcbld1	4.95616E-07	3.91E-25	2E-208	0.017869404	0.052461845	0.117865833
Col6a3	1	1.84E-166	0	0.000975144	0.048236958	0.117845087
Dmxl2	0.109743453	1.00E+00	1E-57	0.025556378	0.039727502	0.117829257
Tbrg1	1	7.23E-32	1E-252	0.007019325	0.052884163	0.117680823
Tmem147	3.39637E-09	1.06E-48	1E-261	0.016853314	0.061416721	0.117642179
Wdr44	1	3.73E-25	6E-138	0.013159171	0.074627787	0.117627707
Pus10	1	4.04E-06	2E-84	-0.000179326	0.053999189	0.11736032
Mtss1	1.95816E-10	4.36E-02	1E-24	0.069555269	-0.063372347	0.117229326
2610203C2	1	4.06E-206	0	-0.002641376	0.109138769	0.117181939
Uqcr10	1	1.26E-33	4E-231	0.000799929	0.059126306	0.117166736
Sat1	1	3.03E-85	7E-130	-0.004103376	0.165076492	0.117134656
Map1lc3b	2.42288E-47	2.54E-32	2E-141	0.054610256	0.079362301	0.117104305
Mtmr10	1	3.41E-02	2E-92	0.010868201	0.037803487	0.117097492
Snx14	1	2.13E-11	1E-108	0.0086285	0.059342013	0.117015591
Rack1	1	1.36E-81	3E-162	0.010746098	0.118281053	0.116962709
Bfar	1	1.00E+00	7E-79	0.012990919	0.000824749	0.116951962
Rps21	1	7.17E-141	3E-187	0.006998295	0.148728945	0.116949875
Chmp4b	4.38528E-05	6.40E-30	1E-123	0.022227878	0.088042318	0.116946717
Twsg1	1	2.02E-41	2E-224	-0.005702224	0.062357119	0.116861902
Arfgap1	1	3.80E-01	6E-126	0.010510563	0.029336346	0.116737174
Timm9	0.062288924	1.00E+00	2E-78	0.023125378	0.01709423	0.11654545
Crem	7.37418E-80	3.65E-19	1E-60	-0.094250354	0.112124863	0.11654271
Rps10	1	7.66E-191	2E-204	0.002083632	0.159843237	0.116483854
Rras2	3.09361E-06	9.16E-04	3E-76	-0.027004578	0.052392133	0.116460397
Eef1b2	0.089841813	4.12E-108	1E-276	0.009539535	0.092591242	0.116421787
Parp6	1	7.34E-46	3E-165	-0.002591987	0.083818447	0.116299025
Atp5a1	1	8.51E-05	1E-75	0.009664824	0.055342598	0.116258783
AI506816	1	0.00E+00	0	-0.000386453	0.094482298	0.116234746
Nob1	0.044745976	1.39E-43	2E-183	0.013839674	0.07744064	0.116169097
Eif3b	4.35153E-06	1.64E-13	1E-150	0.02035908	0.049991149	0.116157878
Crebl2	1	1.48E-14	8E-101	0.0046172	0.073313664	0.116131707
Eprs	1.30555E-15	1.00E+00	3E-61	0.050864382	0.000700176	0.116116453
Arfip2	2.04082E-05	3.10E-08	3E-222	0.015021565	0.031160518	0.116083346
Fosl2	1.17058E-11	5.01E-150	9E-238	-0.015107296	0.123781967	0.116075191
Cnot1	1.4374E-05	1.99E-26	6E-61	0.033245753	0.111915889	0.115857592
Minpp1	0.908157001	6.19E-08	2E-123	0.014500957	0.043892274	0.115699316
Asb3	1	1.00E+00	1E-78	0.014897366	0.006683776	0.115615573
Frrs1	0.193151931	1.25E-142	8E-118	-0.015452837	0.211517801	0.115602924
Pkd2	1	1.42E-79	4E-125	-0.010427701	0.131333979	0.115579774
Pls1	1	9.27E-269	0	-0.000300384	0.091233593	0.115403224
Cox19	1	2.48E-15	2E-137	0.007814671	0.05801759	0.115275796
Golim4	1	1.00E+00	1E-48	0.014842595	0.007282741	0.115275226
Mdh2	1	5.96E-35	7E-152	0.000160499	0.075022813	0.115065498
Hps5	1	4.32E-26	7E-130	0.00605106	0.077128307	0.115044118

Dhx29	3.80896E-05	1.29E-109	3E-232	0.013945449	0.09986703	0.114959767
Irf3	0.035177285	1.00E+00	1E-132	0.016313767	0.022373596	0.114865294
Sall1	8.46758E-56	1.24E-07	7E-130	0.059219131	0.047684364	0.114830727
Pex19	0.25723339	6.18E-02	5E-106	0.017784356	0.035256467	0.114824315
Vps26c	1	3.06E-65	9E-187	0.005195941	0.094838604	0.114784712
Psmd9	8.96543E-35	1.00E+00	1E-55	0.073812032	0.03252849	0.114696539
Tinag	5.31479E-42	2.60E-281	0	0.012325346	0.081401815	0.11466835
CAAA0111[illegible]	1.09156E-07	3.31E-78	1E-126	0.024659785	0.135375496	0.11435133
Dctn2	1	2.17E-30	4E-142	0.008373145	0.074468157	0.114187901
Gcc2	9.22439E-16	1.00E+00	8E-73	0.046564507	0.008845071	0.114176752
Trappc10	1	3.42E-04	1E-95	0.010365185	0.046716423	0.113960578
Rps14	1	1.58E-104	2E-139	-0.009788286	0.147146085	0.113881943
Tmpo	1	1.47E-57	3E-158	0.010883762	0.098756292	0.11370849
Kmt2e	1.31703E-38	5.56E-21	2E-49	0.075271306	0.119878088	0.113485473
Pef1	1	1.02E-35	1E-167	-0.007062871	0.071373358	0.113483304
Bet1	1	6.09E-26	3E-120	-0.002908769	0.078262825	0.11343404
Zfp398	1	2.42E-18	9E-92	-0.001903738	0.079270784	0.113311738
Mtrex	1	1.62E-25	5E-121	0.010463238	0.074115649	0.113296443
Ammecr1l	1	1.00E+00	9E-62	0.002067716	0.005104393	0.113266374
Slc25a17	0.252281092	1.00E+00	8E-64	0.022722336	0.044327397	0.113192399
Srpr	1	4.03E-05	1E-121	0.015316859	0.042817227	0.113187303
Cox4i1	1	2.40E-48	2E-133	-0.004479507	0.104008556	0.113114359
Rpl26	1	5.58E-122	8E-208	-0.001733554	0.116714365	0.113058576
Slc30a5	0.005029353	5.80E-17	8E-107	0.020427926	0.068623955	0.113057622
Myh9	1	3.73E-64	6E-58	-0.011391695	0.185978295	0.113039406
Vps8	1	4.90E-50	2E-107	0.008885403	0.122192034	0.112985296
Nt5dc1	1	1.00E+00	9E-69	0.01324532	0.031339688	0.112972484
Stk16	4.41749E-05	3.58E-04	6E-171	0.017360852	0.029467088	0.112941253
Cyb5rl	1	1.35E-79	8E-274	-0.001202341	0.071610503	0.112901433
Pex1	0.57219152	1.00E+00	4E-67	-0.019641837	-0.014568243	0.112857183
Sdad1	1.61473E-13	1.35E-29	1E-177	0.024853569	0.060695805	0.112771842
Idh3b	6.87792E-11	2.27E-47	2E-175	0.022935639	0.077205168	0.112770125
Psmd5	1.04438E-05	1.16E-49	2E-198	0.016430386	0.070767027	0.112768283
Mtbp	1	2.24E-128	1E-281	-0.000973587	0.090515114	0.112697675
Tomm70a	1	2.06E-09	3E-66	-0.007545967	0.072833618	0.112587779
Mark3	0.000284156	2.00E-12	2E-65	0.031488428	0.075425452	0.112514394
Dusp11	9.55311E-21	4.35E-20	3E-75	0.050530032	0.091231923	0.112488807
Rbck1	1.11426E-05	1.53E-53	7E-173	0.017973503	0.084675674	0.112485723
Lonp1	1	1.23E-16	3E-157	0.008482838	0.053066777	0.112412805
Trub1	0.000155877	3.97E-07	8E-125	0.02085153	0.042021162	0.112348218
Gcn1	1	1.28E-04	9E-88	0.013569162	0.048182852	0.112300901
Adcy1	1	1.79E-92	0	-0.00382695	0.056977433	0.112144289
Rsl1d1	0.159302517	1.36E-47	8E-149	0.013708592	0.087533	0.112110173
Slc44a3	2.74497E-43	4.66E-36	7E-179	-0.032734766	0.066211382	0.1119966
Tia1	1	5.37E-15	1E-129	0.012262603	0.057084205	0.111932506
Bicra	1	1.00E+00	2E-61	0.007413416	0.042618197	0.111796777
Gm15489	1.01159E-10	1.49E-87	2E-217	0.018491284	0.089949225	0.111759456

Gm19950	0.00604599	2.01E-89	3E-189	-0.011029349	0.102041753	0.111703627
Usp20	1	4.64E-22	1E-129	-0.004735551	0.065174982	0.111689694
A630089N(	1	2.36E-09	5E-155	-0.009519078	0.039168326	0.111671411
Rps7	0.025893003	9.68E-125	8E-229	0.010655772	0.110063771	0.11154103
Cd59a	1	9.75E-18	5E-132	-0.010522386	0.060349888	0.111525689
Esf1	1.12603E-08	1.00E+00	3E-109	0.029188912	0.026586701	0.111504648
Fbxl4	2.06884E-10	1.17E-04	6E-114	-0.024762619	0.041743993	0.1113059
Agk	1	5.87E-99	5E-237	-0.006484934	0.091224835	0.111169447
Mbd5	1.03233E-32	1.00E+00	2E-44	-0.076639575	0.003921693	0.111143885
Slc7a1	1	0.00E+00	0	-0.000474696	0.101787744	0.110827485
Ttyh2	1	1.24E-03	4E-76	-0.010839412	0.057214379	0.110820835
Idh1	2.37846E-22	1.00E+00	1E-66	0.055747263	-0.009330042	0.110694259
Nop2	1	1.34E-102	2E-246	0.007905689	0.086515981	0.110676571
Grina	1.47437E-20	1.12E-77	8E-99	0.04130902	0.154487366	0.110593676
Nol8	4.11233E-14	1.13E-85	3E-161	0.026715291	0.109189463	0.110517471
Mars	0.042731016	5.75E-58	4E-218	0.011595497	0.070895245	0.110473987
Ssr3	1	4.23E-24	2E-157	0.007801561	0.059044886	0.110381695
Apba3	1.97922E-05	7.51E-47	5E-204	0.01502351	0.067518471	0.110378329
Jun	1	4.54E-153	3E-199	-0.004969175	0.130595707	0.110354887
Abhd18	1	8.34E-01	8E-47	-0.018709257	-0.023776057	0.110239733
Ap1b1	1	1.67E-03	9E-99	0.011616618	0.040710609	0.110157136
Nudt13	1	1.53E-33	2E-105	0.012948906	0.090010024	0.110087024
Prdm16	1	0.00E+00	0	-0.00090199	0.16192505	0.110086066
Lss	8.34864E-68	1.00E+00	1E-215	0.052057425	0.008197987	0.110084416
Gpr89	1	4.23E-22	3E-135	0.011636362	0.062799096	0.10991291
Tnfaip8l3	1.36425E-06	1.86E-177	0	-0.005659381	0.07787585	0.109887474
Scn8a	0.066264495	0.00E+00	0	0.003490254	0.107273378	0.10985009
Fbxo36	0.008324936	1.25E-09	1E-117	-0.015452454	0.047551562	0.109820368
Vapb	1	4.07E-43	2E-132	0.013654912	0.091015428	0.109792693
Pbdc1	1	1.07E-32	4E-135	0.002731322	0.076894723	0.109765833
Ankib1	1	1.01E-03	6E-70	0.011266288	0.054707965	0.109630383
Cct8	1	1.39E-19	2E-136	0.013215904	0.059978281	0.109553072
Nckap5	5.46009E-12	9.47E-60	1E-166	0.017960326	0.088252471	0.109538641
Trip12	1	2.19E-04	2E-42	0.019343772	0.080166742	0.109516921
Dicer1	3.8965E-09	5.56E-01	3E-76	0.036692519	0.038334014	0.109474742
Fdx1	6.14958E-23	2.51E-02	4E-107	-0.031978439	0.035096837	0.109359022
Coq2	1	5.49E-79	8E-201	-0.001724715	0.089110342	0.109333711
Rpl35	1	3.16E-170	1E-274	0.006513986	0.109409343	0.109319848
Clasp2	1	7.63E-18	8E-74	-0.007805088	0.090880699	0.109075061
Naa16	5.22157E-26	9.54E-02	1E-70	0.056604398	0.045810875	0.109020203
Mfge8	1	1.38E-287	0	0.000797303	0.069199602	0.108809223
Cox6a1	1	2.26E-71	2E-170	0.000858425	0.100061541	0.108801708
2810013P(	0.001304688	9.21E-59	4E-140	0.016197606	0.093904694	0.1087446
Acot1	7.7759E-93	8.80E-83	1E-201	0.056854559	0.086941825	0.108669674
Slc38a9	1	1.77E-04	4E-71	-0.015337929	0.054249665	0.108494225
St5	4.36973E-37	1.00E+00	3E-43	-0.079858585	0.034220624	0.108434386
Ppp1r12a	1	1.21E-10	7E-44	0.017990815	0.09922052	0.108362189

Rars	1	2.92E-15	4E-130	-0.001918416	0.053757569	0.108237201
Gfm2	1	1.00E+00	4E-72	0.01185942	0.020640559	0.108216853
Foxn2	1	1.41E-11	8E-84	0.001410392	0.070847878	0.108060943
Xrn1	1	1.10E-28	1E-57	-0.00501926	0.115205819	0.108046239
Ecd	0.215324729	2.34E-14	2E-98	0.017952779	0.061173931	0.107987276
Dstn	1	1.94E-18	2E-96	-0.000747965	0.071453483	0.107978442
Nrd1	5.84895E-07	2.25E-02	2E-71	0.032670381	0.049227826	0.107960016
Foxp2	1	1.00E+00	8E-34	-0.021282327	-0.000959379	0.107792437
Fgl1	2.21515E-52	1.00E+00	2E-76	-0.056035559	0.039066731	0.107746191
Blm	1	4.92E-78	3E-160	-0.007137355	0.100586077	0.107652786
Nmnat3	3.74442E-36	1.00E+00	4E-43	-0.071667203	0.037589396	0.107648989
Atp13a1	6.55751E-07	1.40E-23	7E-136	0.021336664	0.06223287	0.107559224
Mertk	1.48576E-06	2.51E-83	2E-54	-0.026783044	0.268173822	0.107332168
Utp11	0.796435977	4.32E-11	3E-145	0.012806911	0.041059419	0.107325813
Prmt9	1	1.24E-36	1E-142	-0.00173531	0.076537879	0.107300243
Efl1	1	4.59E-06	9E-73	-0.004555099	0.054974726	0.107232195
H2afy	1	7.94E-08	3E-83	-0.001669822	0.061719457	0.107226623
Zfp639	1	3.77E-48	5E-158	0.004380811	0.080382788	0.107135138
Snhg12	5.32165E-06	1.28E-73	4E-214	0.015016962	0.080791385	0.107063644
Zranb2	6.0029E-40	1.00E+00	7E-61	0.07618118	0.030195562	0.106868924
Pex26	1	7.82E-22	2E-135	-0.001634774	0.059812924	0.106774201
Tbc1d19	1	3.61E-101	3E-297	-0.005098939	0.073237152	0.106619626
Stxbp5	1	6.38E-11	3E-74	0.006727499	0.075476697	0.106608675
Apaf1	1	1.80E-178	6E-267	-0.004092027	0.118571652	0.106516831
Zswim8	3.46846E-15	1.00E+00	1E-109	0.033987068	0.028206981	0.10648255
Nvl	1	8.70E-10	2E-82	0.01691451	0.060229149	0.106463994
Opa1	1	1.00E+00	6E-65	-0.01010994	0.034527872	0.106395112
Cdk5rap1	1	7.05E-35	2E-159	0.005789641	0.065766607	0.106386856
Snx25	0.448925027	1.00E+00	3E-65	-0.017506697	0.007969083	0.106386245
Nck2	1	2.50E-157	8E-230	-0.006528683	0.123863284	0.106381846
Sec24c	1	3.73E-04	1E-87	-0.008074255	0.045565974	0.106381092
Rps15a	1	4.53E-119	3E-192	0.00159368	0.116569287	0.10628363
Sqle	8.09372E-75	1.00E+00	2E-195	0.051632937	-0.007160649	0.10627093
Cdk12	1	5.39E-05	2E-43	-0.01439496	0.074907176	0.106244028
Nup160	1	1.68E-30	2E-119	0.009073539	0.076150963	0.106204344
Fnbp1l	0.000254714	1.01E-08	2E-88	-0.020791752	0.059415024	0.106180808
Hnrnph1	3.62846E-10	4.43E-40	1E-67	0.036773442	0.129219784	0.10617533
Rab4a	8.29984E-42	3.42E-13	8E-102	0.055131097	0.056230455	0.106168972
Atp6v0d1	1	3.33E-44	4E-76	0.010483148	0.128059341	0.106150623
Dhx15	1	7.16E-16	6E-77	0.006523245	0.081674964	0.105962754
Snrnp200	1	3.92E-31	2E-126	0.011664328	0.073353159	0.10594411
Atp6v1a	0.3416594	8.94E-57	3E-92	-0.014038105	0.142842347	0.105870829
Mapk9	1	2.24E-13	1E-78	-0.003682311	0.070620979	0.105781908
Thoc2	1.96479E-06	9.00E-18	1E-48	0.03551608	0.096836323	0.105633608
Raf1	0.03125875	1.25E-19	1E-38	-0.030335294	0.118567305	0.105611191
Hspa4	6.61689E-08	1.12E-13	1E-58	0.036737592	0.084746925	0.10560577
Slc25a37	1	8.73E-35	2E-92	0.001803579	0.099900816	0.105529336

Pola1	1	3.02E-24	1E-89	0.005797963	0.080931647	0.105451968
Tmed7	1	4.07E-17	4E-167	0.003820518	0.044064564	0.10539885
Myo18a	0.002821276	1.00E+00	3E-37	-0.033857374	0.050145982	0.105398405
Strn3	4.65108E-08	9.02E-27	6E-35	0.039427122	0.141401961	0.105190074
Mt2	1	2.41E-58	2E-274	-0.000100592	0.049584142	0.105169695
Nme2	1	1.31E-55	9E-179	0.006675069	0.077753804	0.105118706
Fmo2	6.61403E-24	2.52E-110	0	0.018033635	0.06435397	0.105109308
Eif2ak4	1	1.84E-12	7E-106	-0.001093053	0.058946032	0.105068364
Dgkz	1	4.80E-105	9E-108	-0.006431724	0.168079469	0.105062508
Nol11	1	1.40E-35	2E-161	0.010358532	0.06641493	0.105014093
Pnpla2	3.56191E-09	1.93E-60	5E-198	0.017703127	0.075088301	0.105008064
Mrps27	1	1.00E+00	1E-111	0.013594195	0.023333963	0.104851156
Dock1	1	4.09E-19	4E-48	-0.004337668	0.09472649	0.104673673
Zfyve26	0.414853473	7.55E-01	4E-66	-0.017706893	0.042178829	0.104572456
Uqcrc1	1	2.15E-22	6E-121	0.003893474	0.06477229	0.10451136
Npepl1	2.3757E-07	1.74E-18	2E-135	0.021294563	0.057384951	0.104502655
Usp15	1	4.05E-37	5E-48	0.006202929	0.132953838	0.10447386
Trip11	1	4.26E-06	4E-49	0.017706294	0.06632387	0.104390941
Katnbl1	1	1.27E-56	2E-149	-0.003409297	0.092155198	0.104360423
Rps26	1	3.09E-173	1E-168	-0.000408145	0.156276235	0.104357316
Upf1	1	5.46E-39	5E-149	0.009500865	0.072920819	0.104353468
Pla2g12a	2.18996E-09	2.11E-59	5E-174	0.020325684	0.079400798	0.104285702
Frmd8	1.48508E-08	1.92E-126	3E-184	-0.014898407	0.116042785	0.104271931
Wapl	1.5831E-14	2.77E-01	1E-42	0.050198469	0.06085102	0.104232225
Ube4b	0.001505109	2.37E-03	6E-45	-0.033048094	0.059255962	0.104148581
Egln1	1	8.89E-49	6E-130	0.012381109	0.087982193	0.104103241
Grhpr	1	3.22E-05	2E-63	0.016756666	0.061781437	0.103832115
Aasdh	1	1.16E-24	4E-111	-0.010298779	0.07134075	0.103740664
Aldoa	1.72258E-06	6.98E-62	1E-160	0.016882153	0.087786292	0.103659094
Farsb	1	1.43E-06	5E-94	0.002151873	0.04725904	0.103618471
Ofd1	0.026980447	7.26E-52	2E-153	0.013299251	0.08019815	0.103423075
Gstp1	1.44921E-10	2.07E-22	2E-93	-0.026550018	0.072209161	0.103382362
Mrpl24	1	3.50E-45	7E-138	0.007697378	0.082497127	0.103331007
Mthfr	1	1.09E-02	3E-71	0.011761061	0.044857566	0.103281606
Mfn1	1	1.00E+00	6E-72	0.014299242	0.030363507	0.103262396
Kcnq1	0.002239113	1.15E-59	2E-199	0.012188268	0.074889234	0.103202582
Pigk	1	1.98E-22	3E-114	-0.003262787	0.067927818	0.103186326
Cope	2.53379E-24	5.97E-08	4E-133	0.037136125	0.040264611	0.103130447
Zfp426	1	2.24E-17	3E-113	-0.004142407	0.057586909	0.103089437
Eif2d	1	2.47E-22	1E-142	0.008267629	0.055531066	0.103059309
Gm36283	1	2.99E-186	0	-0.001773364	0.071552853	0.10300832
Gm20400	3.80738E-14	1.00E+00	6E-53	-0.043915823	0.010480523	0.102951479
Supt6	1	1.00E+00	7E-65	0.019583513	0.033328671	0.102924642
Setd5	6.85885E-08	4.09E-13	1E-41	0.038048871	0.095988769	0.102842219
Gsta1	1	0.00E+00	0	-0.001118464	0.162196182	0.102792383
Ganab	7.35807E-08	1.00E+00	9E-76	0.031586291	0.015928216	0.102780871
Rfwd3	1	8.21E-28	6E-111	-0.011124219	0.075776805	0.10277944

Mcfd2	1.71429E-40	1.00E+00	5E-108	0.051714172	0.022046409	0.102766124
Tnik	1	4.14E-13	8E-193	-0.003849333	0.033160921	0.102750177
Larp4	1	1.00E+00	3E-49	0.001846813	0.024312775	0.102439693
Psmd4	1.15646E-23	1.00E+00	5E-62	0.056006657	-0.019549698	0.102418395
Topbp1	1	5.04E-87	6E-171	-9.04218E-05	0.096748506	0.102367378
Mrpl15	1	3.85E-11	2E-125	0.007541392	0.045137488	0.102366972
Aars	1	3.42E-10	6E-75	-0.010460855	0.062631869	0.10230909
Taf2	1	8.03E-19	4E-115	0.009826659	0.063400816	0.102301765
Dhx8	0.003288579	3.54E-14	2E-99	0.019087392	0.058436949	0.102268564
Cacna1b	1	2.95E-192	0	-0.00052065	0.059802411	0.10225683
Pwp1	1	1.30E-36	8E-176	-5.68269E-05	0.059042556	0.102226449
Thoc7	1	1.42E-23	9E-120	-0.003807101	0.064042128	0.102178403
Rexo2	2.86128E-08	4.96E-29	2E-146	0.021031804	0.060625561	0.102017378
Hilpda	1	2.97E-227	0	0.002358485	0.075341288	0.101994734
Stx5a	3.22872E-22	2.45E-12	6E-53	0.059130032	-0.05977822	0.101861778
Dancr	0.119086285	6.76E-155	3E-263	0.007883603	0.095189536	0.101706017
Hadh	1	1.13E-21	2E-91	0.013367792	0.074873484	0.101669591
Rps16	1	2.59E-117	5E-180	0.007293296	0.11584195	0.101599998
Atp5f1	1	4.25E-18	7E-89	-0.000192727	0.070545939	0.101569679
Gspt1	1	1.49E-12	1E-66	0.017513679	0.07251327	0.101499851
Snapc3	0.00361462	3.81E-04	2E-69	0.024735835	0.051837386	0.101436364
Rps11	1	4.03E-108	1E-237	0.006180182	0.085945026	0.10137761
Mphosph1	3.12788E-10	1.53E-49	2E-193	0.018150246	0.064308652	0.101370889
Fech	3.43513E-17	2.92E-18	3E-52	0.050438869	0.098245829	0.101226511
Orc4	1	2.52E-15	2E-94	0.011606126	0.061901111	0.101157862
Eif2b4	0.010935721	8.35E-11	6E-141	0.015279382	0.040245424	0.101136935
Cry2	0.140012814	1.00E+00	1E-72	-0.016685476	0.02651899	0.101127027
Ilrun	0.568548197	7.46E-09	3E-41	0.022495147	0.074860032	0.101115194
Pafah2	1	1.00E+00	1E-67	0.015948688	0.02978507	0.101044026
Rpl18a	1	1.71E-90	2E-190	0.005612945	0.09608833	0.101019073
Edrf1	1	1.00E+00	6E-68	0.017578094	0.035642855	0.101015691
Psph	1	4.44E-36	2E-249	-0.00189757	0.044041483	0.100974742
Nudt12	1	5.68E-20	3E-84	-0.012380071	0.074006794	0.100795699
Rmnd1	1	1.32E-19	9E-69	-0.010967196	0.086633371	0.100790144
Plpp5	1	4.47E-09	2E-166	0.005605044	0.034358188	0.10078681
Slc25a46	1	1.40E-26	3E-110	-0.005726318	0.072952603	0.100767264
Abca3	1	2.88E-18	6E-95	-0.011533677	0.066319221	0.100657758
Rpl22l1	1	6.18E-100	3E-252	0.005834808	0.079835809	0.100630609
Plgrkt	1	3.24E-115	9E-176	-0.003949048	0.116689395	0.100516926
Med23	1	5.45E-43	2E-136	-0.002380654	0.079520757	0.100469312
Dtnbp1	2.52499E-13	9.55E-04	2E-42	-0.040750244	0.070382845	0.100361223
Ufsp2	1.86543E-09	1.11E-06	2E-120	0.02454157	0.040561224	0.100323931
Lancl1	1	2.80E-36	4E-131	0.005583378	0.073777731	0.100234342
Als2cl	1	5.45E-04	6E-71	0.018033374	0.044703833	0.100215652
Cep295	1	5.78E-14	2E-111	0.011032946	0.055030778	0.100215546
Ccdc15	1	4.93E-112	1E-123	-0.010573116	0.140185411	0.100138012
Gpatch3	1	1.40E-37	1E-160	-0.004963349	0.062421246	0.100115381

Npat	1	4.82E-22	7E-135	0.005318193	0.057681939	0.1000681
Nsun6	1	1.03E-32	3E-119	0.003878133	0.073214155	0.100039621
Ccz1	0.083569958	1.06E-09	2E-88	0.017705807	0.056534069	0.099953321
Fam83a	1	3.03E-261	0	0.000488119	0.072746339	0.099919598
Senp6	1	1.02E-30	9E-46	0.005218097	0.116997731	0.099905573
Bet1l	1.74206E-14	2.40E-07	8E-136	0.027229323	0.034601959	0.099809958
Pus7	1	1.02E-51	1E-142	0.004619021	0.082349547	0.099770216
Cog4	1	1.00E+00	9E-49	0.018784392	0.018194681	0.099760738
Rpl14	1	1.66E-92	3E-193	-0.000305253	0.094811029	0.099690492
Fdft1	1.019E-165	3.76E-30	5E-58	0.133342753	-0.060935739	0.099648325
Gm32540	0.00012273	6.09E-101	5E-213	-0.010249689	0.087298676	0.099617218
Prpsap1	1	6.43E-09	3E-58	0.01466619	0.067097613	0.099617096
Mybbp1a	0.014062886	2.02E-22	2E-139	0.014155392	0.054975493	0.099614547
Dync2h1	1	1.00E+00	2E-61	-0.013959495	0.041388493	0.09959697
Fan1	1	2.29E-26	2E-143	0.007701342	0.056868824	0.099585719
Slc33a1	1	8.85E-08	7E-72	0.007756112	0.057001771	0.099544098
Usp38	1	1.17E-08	4E-96	-0.006509884	0.047027413	0.099256022
Arhgap21	0.005717667	1.00E+00	1E-55	-0.022162055	0.018963354	0.099090273
Kdm6a	1	8.23E-03	1E-50	0.011518738	0.055634639	0.099005488
Rbm41	1	2.43E-26	6E-103	-0.009194737	0.07649409	0.098982866
Szt2	1	4.32E-25	4E-146	0.010664673	0.054751043	0.098971337
Ndufs2	2.06607E-06	3.67E-26	9E-136	0.018543599	0.059964044	0.098943373
Rxylt1	1	4.86E-26	6E-137	-0.001172046	0.056557353	0.098934944
Xpo1	1.84812E-07	2.03E-44	1E-104	-0.020478872	0.097129461	0.098927371
Snhg8	2.36305E-21	3.73E-49	4E-173	0.02715206	0.068798454	0.098873915
Ccdc66	1	1.57E-64	7E-199	0.002608239	0.072059771	0.098849531
Capzb	1	6.40E-19	9E-67	0.011051568	0.102999776	0.098775936
Yipf5	2.44817E-05	1.57E-13	3E-116	0.019079086	0.047691709	0.098587295
Klhl26	1	1.79E-05	8E-113	4.98245E-05	0.034631338	0.098554832
Atp2b2	7.01671E-05	2.70E-01	5E-112	-0.012672757	0.030263579	0.09849416
Ccdc47	1	1.85E-19	2E-116	0.004527768	0.056036108	0.098363025
Gm16116	1	0.00E+00	0	-0.001179665	0.116919818	0.098233651
Rpl27	1	1.11E-95	7E-196	0.003185855	0.095496563	0.098183628
Pum3	4.12177E-11	7.21E-25	2E-105	0.026009351	0.067116706	0.098168491
Fbxw5	8.02163E-11	5.07E-32	3E-181	0.018624502	0.054435936	0.098164404
Zfp131	0.113211972	6.69E-06	2E-84	0.017774183	0.046648122	0.09806552
Trpm6	1	1.27E-40	0	-0.001255926	0.031024042	0.09805094
Rpl32	1	3.20E-126	2E-197	0.004541347	0.106461178	0.09802164
Vps13d	7.64662E-20	7.95E-23	1E-35	0.049828011	0.122853155	0.098012612
Socs6	3.07726E-05	1.06E-10	3E-108	-0.015531668	0.051042786	0.097980808
Cdc73	1	8.68E-07	4E-53	0.016365904	0.06454879	0.097918601
Arhgap18	0.000249925	4.35E-01	8E-47	0.027923938	0.052800616	0.097910482
Nudcd2	1	1.27E-76	5E-221	0.004550109	0.070127133	0.097823964
Guf1	1	8.13E-109	3E-192	0.001376871	0.096931958	0.097641861
2610507B1	1	4.41E-05	2E-56	0.017228065	0.055334119	0.097544241
Gle1	1.65549E-06	6.59E-05	2E-87	0.026672139	0.040876742	0.09753877
Chordc1	1	8.47E-06	7E-88	0.003423684	0.045289361	0.09750039

Arrdc2	2.30655E-18	1.00E+00	3E-100	0.034600498	0.016036857	0.097472842
Depdc7	1	1.00E+00	6E-71	-0.013307778	0.031165782	0.097416679
5430405H0	1	2.63E-01	9E-78	0.013228727	0.034249486	0.097412975
Ndufv1	1	2.15E-31	2E-179	0.008373248	0.051901266	0.097356584
Xrn2	0.067271947	2.63E-11	4E-46	0.025657003	0.078490477	0.097344743
Dync1h1	1	1.94E-62	6E-37	0.024332438	0.183017924	0.097321154
Zadh2	9.62175E-11	6.02E-01	5E-72	-0.028771032	0.037983809	0.097250025
Tcf7l2	1.8803E-26	6.70E-07	5E-24	0.077145956	0.114682193	0.09723517
Npm1	0.05749822	2.18E-70	6E-122	0.013478342	0.10196821	0.097231436
Rabggtb	5.30566E-14	1.08E-18	7E-86	0.034064514	0.066513933	0.097156354
C2cd3	0.253942596	1.71E-01	2E-63	0.020105305	0.035822856	0.097017909
Csnk1g2	1	2.23E-06	1E-83	0.013287958	0.046928342	0.096676441
Itpr1	1.38574E-09	1.80E-05	8E-29	-0.050651748	0.090815148	0.096584874
9130409I2	2.09421E-77	1.28E-135	2E-131	-0.043780391	0.138210871	0.096446687
Rfesd	1	6.60E-43	1E-147	-0.001517666	0.068797618	0.096398274
Supt20	1	3.29E-10	1E-66	0.005537546	0.065232795	0.096376079
Ddx18	8.74855E-06	9.94E-24	4E-140	0.017838294	0.051975915	0.096301485
Acvr1	0.004063654	1.00E+00	4E-36	-0.026835399	-0.018860301	0.09622958
Chid1	1	1.19E-37	7E-160	0.009062029	0.060331811	0.096213043
Hnrnpa1	0.128123459	1.25E-53	3E-120	0.013847729	0.090084774	0.09619414
Iqck	1	5.15E-271	0	1.54233E-05	0.090546366	0.096153826
Psmc4	0.00078778	1.42E-20	1E-136	0.015146011	0.051663487	0.096151974
Trap1	1	2.02E-02	1E-86	0.010701669	0.034783949	0.09610022
Gsap	7.21384E-36	1.00E+00	1E-26	-0.066033462	0.064533194	0.096083304
Abcf1	2.70706E-09	2.29E-11	1E-76	0.031262978	0.060346702	0.096043982
Tbc1d9b	1	2.60E-15	3E-71	-0.004198738	0.074366844	0.095986527
Fndc9	1	0.00E+00	0	3.84256E-05	0.073952938	0.095953713
Rpl5	1	1.35E-71	4E-138	0.00859769	0.095011322	0.095879474
Copb2	0.215770057	9.37E-10	1E-124	0.013676605	0.039122079	0.095867174
Dpp4	1	3.63E-16	1E-32	-0.015112696	0.104474115	0.095840936
Cdc27	1	8.68E-01	5E-64	0.007821118	0.038035122	0.095826665
Napa	1	1.40E-13	4E-94	0.011973748	0.058550399	0.09582246
Mfsd6	0.000784298	2.83E-88	5E-175	-0.01004088	0.094510378	0.095807693
Dnaja3	9.43322E-28	1.00E+00	1E-64	0.053229945	0.028942726	0.0956959
Rbm25	1.49954E-05	6.16E-22	3E-29	0.033372184	0.13763968	0.095581505
Ddx56	1	2.38E-51	7E-177	0.009364733	0.06513918	0.095453864
Sod2	1	1.00E+00	2E-52	-0.008908042	0.012561508	0.095452464
Polb	1	1.56E-02	2E-72	-0.011111283	0.040724482	0.095395052
Taf1	5.35726E-13	1.00E+00	5E-47	0.043911039	-0.018406924	0.095392429
Trnt1	1	1.01E-33	1E-165	0.009602398	0.054313682	0.09529756
Zmym2	2.64987E-10	9.28E-61	2E-41	0.043255995	0.163323451	0.095286257
Gas2l3	1	7.44E-164	2E-248	-0.002773588	0.100052056	0.095228668
Ddx55	0.001444206	3.91E-14	5E-114	0.017570314	0.04971229	0.09520943
Bcat2	0.032835354	1.58E-07	8E-80	0.018905177	0.048810216	0.095197463
P2rx3	1	7.26E-95	7E-194	-0.004397045	0.08474531	0.094958368
Hmgxb3	1	5.45E-31	6E-87	0.010848072	0.081395306	0.094804012
Ovol1	1	0.00E+00	0	4.28875E-05	0.076275457	0.09476555

Psmd7	1.13053E-05	1.00E+00	3E-83	0.024116377	0.029462811	0.094597728
Rpl8	1	1.02E-85	3E-137	-0.00155746	0.10995014	0.094568367
Rps23	1	1.63E-118	1E-193	0.002450981	0.100509254	0.094544341
Slc17a1	1	6.56E-02	3E-85	-0.005174799	0.032665623	0.094465634
Bach1	9.30433E-09	1.58E-31	2E-42	-0.032492265	0.126593736	0.094411017
Zwint	1	1.00E+00	6E-50	-0.00802777	0.035466412	0.09439073
Tmem176a	1	9.69E-160	1E-277	-0.001034698	0.085740388	0.094370784
Ube4a	1	1.00E+00	2E-38	0.012141318	0.032409387	0.094318885
Slc37a4	1.1E-156	1.00E+00	3E-85	0.108044008	-0.005109276	0.094211094
Bdp1	4.03614E-06	9.52E-24	2E-46	0.032065187	0.105085087	0.094152469
Ss18	1	2.25E-10	5E-76	0.005100727	0.054888505	0.094140699
Ghitm	0.344938206	2.06E-37	2E-124	-0.011716317	0.07210354	0.094137363
Klhdc8b	1	1.00E+00	1E-97	0.004585444	0.005246609	0.09408645
Kif13a	1.55446E-06	1.00E+00	9E-47	-0.030139742	0.042957495	0.094033477
Cds2	1	3.00E-38	6E-69	-0.014733303	0.11180357	0.094012094
Clasrp	1	9.99E-07	7E-78	0.008185147	0.047310199	0.093956148
Slc25a12	0.069073587	6.90E-28	7E-64	-0.017271456	0.09641526	0.093939509
Dmgdh	2.7904E-128	3.51E-05	2E-23	0.164267341	-0.056006953	0.093926639
Traf7	0.001208875	5.05E-11	1E-122	0.016280616	0.041650392	0.093830653
Wdr45	0.000307355	5.15E-25	4E-142	0.015153294	0.053748306	0.093792248
Tmem82	0.029960735	5.23E-03	3E-112	0.014919716	0.029780974	0.093765484
Tnip1	1	1.89E-58	7E-164	0.001560828	0.077765889	0.093472696
AW549877	1	1.54E-47	9E-131	0.002339915	0.078030026	0.093469457
Rnf10	1	1.00E+00	3E-50	0.013995849	0.034873117	0.09342396
Pgk1	1.55424E-05	5.25E-42	2E-116	-0.015764802	0.078360078	0.093423324
Snx19	1	3.66E-04	2E-93	0.007952725	0.036140974	0.093391126
BC005537	5.37777E-08	5.17E-75	2E-42	-0.030859288	0.210762026	0.093390681
Pkn2	1	6.89E-19	1E-45	0.015854448	0.094193401	0.093367644
Zfyve16	1	4.99E-17	9E-124	-0.001074279	0.048980033	0.093355343
Tmem259	2.39348E-11	1.00E+00	3E-65	0.035116244	0.014436842	0.093210327
Rc3h2	1	4.32E-16	6E-67	-0.002604182	0.074383242	0.093012372
Aacs	3.41879E-09	1.00E+00	3E-59	0.035567089	0.004298687	0.0929291
Morc3	5.04276E-10	4.71E-15	9E-60	0.034088662	0.076509757	0.092842956
Lpl	1	0.00E+00	2E-275	-0.002608859	0.176065869	0.092761732
Zfp143	0.060296961	1.90E-21	4E-103	0.01510213	0.062012679	0.092757781
Pacs1	0.000245117	3.38E-43	5E-135	-0.013544867	0.081066168	0.092743899
Zdhhc3	1	1.36E-30	2E-116	-0.005651509	0.067833044	0.092690057
Prpf19	1	1.59E-38	9E-129	0.011672195	0.070959644	0.092674636
Nop14	0.001811704	7.98E-19	9E-104	0.017577406	0.058314536	0.092674561
Slc22a29	1	1.30E-129	0	-0.000590187	0.04367946	0.092669333
Tubb2a	1.61216E-05	6.55E-09	1E-118	0.017587956	0.037620172	0.092646917
Actb	1	1.38E-164	2E-104	-0.007078252	0.188037582	0.092643945
BC052040	3.42729E-20	1.39E-03	1E-55	-0.042187886	0.04671813	0.092632706
Il22ra1	1	8.72E-129	2E-180	-0.000946324	0.100244152	0.092590509
Zeb2	0.000101489	0.00E+00	7E-104	0.013880881	0.474725629	0.092583194
Psen1	1	4.25E-14	3E-76	0.001486175	0.065055176	0.092571497
Aplp2	1	1.00E+00	4E-33	-0.016525865	-0.013987772	0.092567232

Slc35g1	0.000920007	2.62E-14	2E-106	0.017161621	0.049473298	0.09254001
Zfp945	1	8.24E-27	4E-88	-0.011799004	0.074735139	0.092530776
Rps6ka1	5.3006E-07	7.96E-51	1E-117	-0.016289148	0.084737674	0.09252603
Wdr36	1	5.81E-76	2E-160	0.008024363	0.081924437	0.092512195
Cep83	1	4.15E-26	4E-79	-0.004345816	0.085108052	0.092435608
Stk10	1	2.69E-254	3E-200	0.0022519	0.159894924	0.092413033
Exoc6b	0.445250094	1.20E-37	3E-34	0.023185536	0.138491856	0.092392238
Dlst	1	1.00E+00	8E-52	0.004588534	0.024284825	0.092334499
Ddit4	1	4.27E-16	4E-166	-0.002177761	0.036591992	0.09217538
Wdr75	5.76815E-08	4.24E-38	3E-132	0.019631746	0.06527816	0.09212722
Dnajb11	1.00528E-11	1.52E-04	1E-109	0.025020061	0.030978124	0.092082478
Tomm40l	1	2.42E-11	2E-65	0.014829899	0.069313889	0.092043098
Tmem161b	1	6.40E-03	4E-65	0.004263595	0.041670779	0.091840837
Ddx52	0.000641897	5.08E-29	1E-119	0.015735099	0.061520314	0.09180346
Ino80	0.000372788	6.28E-17	1E-40	0.031967496	0.090620138	0.091648693
Ddx58	2.0881E-09	1.27E-05	2E-88	-0.021682243	0.043139777	0.091607237
Cep290	1	2.69E-21	5E-88	0.006911353	0.066033994	0.091605168
Zc3h7b	1	2.83E-17	3E-94	-0.008137728	0.057817742	0.091599501
Cramp1l	1	3.60E-08	1E-62	0.015444193	0.058466963	0.091497358
Camkk2	1	1.30E-12	2E-120	0.004936689	0.045073868	0.09138368
Eif2ak3	6.66082E-09	3.20E-07	8E-66	0.029794528	0.054629381	0.091332597
Mms22l	0.204348605	9.75E-54	1E-184	-0.006927494	0.060960789	0.091320143
Rassf1	1	2.27E-44	7E-103	-0.008241486	0.083791628	0.091317972
Pgap2	0.006316468	4.46E-11	5E-85	-0.017442105	0.051737649	0.091313858
Ttf2	1	4.49E-41	6E-174	-0.001645364	0.056493265	0.091286521
Xpo4	1	1.30E-04	1E-53	0.000516851	0.051573891	0.091276725
Smc4	1	1.18E-50	2E-113	0.008501233	0.083833011	0.091257879
Rps6	1	1.09E-58	1E-167	-0.002849226	0.071119289	0.091183081
Gstcd	1	2.68E-41	2E-132	-0.008383309	0.06791395	0.09117417
Eif4enif1	1	1.00E+00	6E-55	0.013377666	0.035952242	0.09113309
Id2	3.19554E-24	1.06E-23	3E-85	0.037791963	0.071208086	0.091095894
Zc3h13	0.306106388	7.44E-02	4E-17	0.024383931	0.004970076	0.091047816
Plpp3	1.25414E-16	1.00E+00	1E-11	0.077490678	0.004796441	0.091026699
Zfp438	1	7.32E-07	1E-69	-0.008377488	0.048973647	0.090986571
Nup93	1	8.16E-28	4E-112	-0.004352853	0.062523663	0.090856239
Scamp1	0.119147952	7.61E-06	5E-39	-0.021462197	0.071152306	0.090731476
Rps6kb2	1	2.35E-08	4E-124	0.008135234	0.035468408	0.090718673
Cyfip1	1	8.60E-16	4E-61	0.011293383	0.083281971	0.090705366
Tbck	1	1.00E+00	7E-41	-0.010642225	0.038091391	0.090627166
Mlh1	0.001396784	1.57E-14	1E-107	0.016406848	0.047915444	0.090626977
Pacrg	1	3.99E-291	9E-265	-0.002375807	0.122216811	0.090561826
Ccdc120	1	3.67E-164	0	-0.001349927	0.059923662	0.09051087
Zcchc8	9.85929E-11	1.00E+00	1E-61	0.035377106	0.027831392	0.090504681
Tkfc	1	1.00E+00	4E-68	0.006844581	-0.002346364	0.090503292
Apbb3	5.46242E-12	1.78E-01	2E-104	0.024680829	0.024414234	0.090424325
9230114K1	0.011482805	3.28E-61	3E-166	-0.009381687	0.069981662	0.090403746
Sec61a1	5.73495E-13	1.00E+00	9E-70	0.035979408	0.009374319	0.090371859

Rnf126	0.000540387	4.11E-30	4E-155	0.013082643	0.050993904	0.090353092
Smc6	1	9.83E-03	1E-63	-0.002391551	0.045198735	0.090318648
Birc2	1	6.63E-32	1E-83	0.010770218	0.081770021	0.090299973
Pdcd6ip	1	1.00E+00	2E-50	0.008894955	0.012600649	0.090228653
Gm28905	1	5.42E-38	4E-233	-0.003073755	0.036445109	0.090228635
Alg8	1	2.18E-69	1E-214	0.005196115	0.061761134	0.090109246
Ubb	0.053812929	2.64E-51	9E-148	0.011455007	0.072757308	0.090050616
Synj1	0.092051436	1.53E-30	3E-70	0.018206323	0.101549814	0.090047451
Nln	2.6846E-12	3.75E-22	4E-53	-0.031432818	0.089570646	0.089983066
Dhx30	3.75813E-10	4.71E-08	2E-88	0.026594147	0.045214751	0.089938107
44262	2.2265E-12	1.00E+00	2E-41	-0.037746396	0.032012673	0.089804629
Usp5	0.002319516	5.59E-16	2E-124	0.015119411	0.044223983	0.089677743
Rpl11	1	2.41E-69	1E-123	0.003654643	0.097900419	0.089661578
Rps25	1	5.06E-94	1E-149	0.004721665	0.096839104	0.089650387
Rps13	1	1.70E-120	5E-167	0.003183975	0.105439705	0.089646416
Cops5	1	1.71E-12	7E-88	0.007940806	0.050727294	0.089622562
Gnpat	1	5.11E-15	2E-87	0.003575069	0.055977033	0.089489255
Slc16a6	1	3.66E-35	2E-101	-0.010554557	0.07595123	0.089471965
Tmem209	1	4.13E-71	4E-134	0.009113137	0.08706731	0.089464137
Farsa	0.127869464	2.43E-11	2E-92	0.015203038	0.047098196	0.089413049
Ehd1	1	7.17E-65	6E-181	0.000232612	0.071157297	0.089397503
Slc9a8	0.03386064	3.44E-05	2E-53	0.022544959	0.053526105	0.089309912
Zzef1	1	1.16E-05	3E-41	0.01128892	0.059048723	0.089278924
Cltb	1	7.40E-13	3E-78	0.00169802	0.053428226	0.089272779
Trak1	1	2.32E-28	3E-31	-1.83323E-05	0.122089157	0.089255995
Stpg2	1	2.92E-68	6E-204	-0.004814153	0.060425738	0.089243487
Abr	1	0.00E+00	2E-194	0.000810104	0.338123315	0.089223438
Iqgap1	1	0.00E+00	1E-162	0.003655665	0.287823321	0.089198284
Nipal1	1.86218E-17	1.00E+00	3E-208	0.015970602	-0.004255982	0.089049205
Eftud2	1	1.83E-31	2E-117	0.008999013	0.062645404	0.089011131
Nsun2	0.053964373	2.17E-06	1E-68	0.016830256	0.047527029	0.088977336
Chchd6	1	7.50E-41	2E-150	0.004953047	0.060897319	0.088948191
Lias	1	5.69E-15	4E-97	0.008151672	0.051252198	0.088921234
Zc2hc1a	1	9.00E-145	0	-3.38274E-05	0.055190511	0.088912283
AC149090.	2.0769E-285	1.42E-12	5E-28	0.222867565	0.097775112	0.088867461
Hdlbp	1	5.40E-19	1E-27	0.016701958	-0.076280633	0.088842792
Ercc3	1	5.59E-45	4E-141	-0.001416962	0.065494969	0.088790533
Susd1	1	6.49E-83	2E-124	-0.000376794	0.106780088	0.088779975
Xbp1	1	8.03E-22	3E-64	0.000123404	0.082512198	0.088546327
Fam187b	9.0293E-07	1.11E-70	1E-170	-0.01226256	0.07155249	0.088514083
Aspscr1	1.1066E-15	1.00E+00	6E-45	0.046654923	-0.01442466	0.088474476
Fbxo30	1	8.49E-42	2E-126	0.002034982	0.069860053	0.088472251
Tmem8	1	8.45E-96	5E-222	0.005012111	0.068560944	0.088436685
Gm43948	1	1.34E-77	2E-283	0.002405783	0.04450132	0.088415556
Eif3g	7.50324E-07	4.32E-57	7E-204	0.012228323	0.056481417	0.088415249
St14	1	0.00E+00	0	-0.000151778	0.097171467	0.088407025
Scyl2	1	1.00E+00	5E-63	0.011738608	0.011659404	0.088398573

Bclaf1	3.54368E-21	1.00E+00	3E-31	0.057364356	0.017579628	0.088326412
Mettl6	1	1.13E-03	4E-88	0.008033822	0.033457751	0.088260294
Vdac1	1	2.73E-18	2E-77	-9.67855E-05	0.065230754	0.088178058
Naca	1	2.34E-22	6E-77	0.002971418	0.072021869	0.088175902
Srr	0.003369102	1.00E+00	6E-26	0.028183277	0.052494283	0.088116286
Pum2	0.005073325	1.45E-54	5E-36	-0.026194142	0.152259335	0.088054298
Slc35b1	6.1006E-21	3.09E-11	8E-153	0.026498931	0.031656756	0.088023324
Ipo11	1	5.45E-03	3E-68	-0.001817846	0.039233309	0.087967868
Golga3	1	1.00E+00	4E-68	0.007700788	0.019010485	0.087876703
Trmt2a	0.050063271	4.55E-53	2E-195	0.008817421	0.058244448	0.087875942
Rad51b	1.00628E-19	2.28E-42	8E-39	0.044502317	0.150353945	0.087873663
Cdc40	1.85016E-07	1.00E+00	4E-58	0.029814555	0.006696886	0.087872784
Hsf4	1	3.60E-98	2E-198	-0.005876292	0.07520821	0.087841104
Polr3b	0.002847335	1.10E-11	6E-69	0.021404936	0.059239887	0.087768441
Tmco1	1	1.15E-42	1E-111	0.000371795	0.074835261	0.087732801
Ubr1	0.275898462	6.74E-01	7E-47	0.020135809	0.041629256	0.08771558
Pacsin2	1	2.21E-04	2E-29	-0.002805064	0.075582858	0.087707112
Ttc23	1.45885E-14	2.58E-01	5E-29	-0.050469846	0.049154607	0.087664899
Zfp418	1	1.16E-166	1E-267	0.00218726	0.07885477	0.087627299
Tead1	1	6.32E-13	6E-34	-0.005518163	-0.062378781	0.087617139
Gstm4	1	1.14E-171	3E-234	-0.00302551	0.092627945	0.087581977
Ncbp1	0.008310084	2.65E-17	5E-100	0.014721898	0.049399265	0.087520785
Mlh3	1	1.40E-08	1E-92	-0.008110465	0.042025749	0.087379005
Epb41l4aos	0.011779654	6.48E-73	2E-224	0.00811414	0.056697026	0.087208104
Rps6ka3	0.472050289	8.73E-17	1E-64	-0.0156701	0.075152854	0.087191235
Ciapin1	1	1.27E-13	1E-70	0.006814262	0.058061757	0.087062766
Ssu72	1	9.52E-15	5E-63	0.004104454	0.071685602	0.08701176
Dvl3	1.50259E-07	1.68E-13	1E-100	0.02115623	0.04692345	0.087010856
Dmpk	8.74965E-05	8.66E-75	5E-193	0.009459861	0.075206044	0.086997095
Nup205	1	4.11E-33	2E-135	0.003147182	0.058250711	0.086958687
Kmt5a	1	2.50E-36	3E-103	-0.004313273	0.076465976	0.086940833
Lig3	1	6.80E-07	6E-101	0.003606075	0.035515267	0.086910584
Rpl21	1	4.30E-84	1E-137	0.00714867	0.094436409	0.086884157
Gm47790	1.02176E-21	1.92E-96	8E-115	-0.021165485	0.112356784	0.086856171
Mphosph8	3.71232E-71	1.00E+00	5E-59	0.08120411	-0.003866472	0.086832211
Psmc1	1	1.00E+00	7E-63	0.016670775	0.020125141	0.08680147
Ipo4	1.08285E-09	3.83E-49	1E-207	0.013919514	0.051591531	0.086776157
Ube2f	1	1.43E-05	5E-57	0.004160884	0.051275075	0.086770203
Hnf4a	1.88441E-20	1.00E+00	4E-20	0.058333595	-0.021955283	0.086765034
Glmn	1	1.34E-27	2E-109	-0.007283948	0.059932838	0.086652031
Eif6	1	2.74E-54	1E-170	0.003207312	0.064568073	0.086550667
Pon2	1	3.83E-29	7E-107	-0.002306909	0.064627612	0.086529675
Nolc1	1	2.34E-38	6E-96	0.010487713	0.07466013	0.086490177
Ift88	0.194588838	1.43E-16	2E-74	0.016763607	0.06035464	0.08648954
Fcf1	1	6.86E-28	3E-99	0.007586571	0.064380405	0.086397202
Tsc2	1	1.11E-06	6E-126	0.006178034	0.03052421	0.086383135
Wwc2	1	1.00E+00	1E-35	-0.013586735	-0.006836347	0.086381291

Gm30505	1	5.74E-09	1E-137	0.005388504	0.030173463	0.086376912
Ckap5	1	5.02E-05	4E-50	-0.011421587	0.053304168	0.086285552
Tpra1	0.502477777	3.86E-60	5E-132	0.010443176	0.081273068	0.086272806
Src	1	2.90E-283	0	0.00137471	0.077562411	0.086255951
Nup214	1	1.00E+00	1E-54	0.009932127	0.02858576	0.086243614
Pmm1	1	5.75E-247	0	-0.001793596	0.075871752	0.086228711
Togaram1	2.88893E-09	4.66E-16	3E-70	0.027197831	0.063407171	0.086218525
Mvk	9.64602E-15	1.22E-09	7E-240	0.014656493	0.016911187	0.086111804
E2f3	7.68128E-24	2.69E-06	3E-40	-0.047360968	-0.032674648	0.086089221
Brix1	1	9.01E-22	1E-100	0.004778634	0.056513731	0.086066926
Tmem234	0.387318007	1.00E+00	2E-58	0.018883387	0.027100948	0.0860288
Sae1	1	3.63E-40	1E-91	0.000611572	0.081083484	0.085974657
Egr1	5.0869E-15	1.18E-77	8E-91	-0.013521842	0.104686373	0.085951813
Usp1	1	3.46E-14	2E-128	0.007676562	0.039084771	0.085930159
Rpap2	1	3.80E-08	5E-69	-0.008857682	0.04947201	0.085811596
2210016F1	1	8.67E-75	1E-197	0.000349147	0.063721868	0.085728161
Exosc7	1	1.02E-49	9E-97	0.009206022	0.085446885	0.085694371
Pgs1	4.41002E-07	1.53E-36	1E-118	0.018260963	0.066014274	0.085673622
Josd1	6.63176E-05	1.75E-35	8E-96	-0.016780015	0.072560281	0.085662772
Ercc6	1	1.00E+00	5E-51	0.011467285	0.00179479	0.085591901
Dgat1	1	4.42E-03	2E-98	0.009084481	0.031306756	0.085513676
Nucb1	1	1.10E-16	7E-72	0.011129079	0.061036255	0.085489222
Lmna	1	1.04E-37	1E-88	0.009958358	0.078321973	0.085429489
Kif24	1	8.90E-03	9E-124	-0.008140917	0.021473362	0.085419888
Serpina1b	1	2.48E-80	1E-13	0.00192671	-0.15961106	0.085416138
Vwc2l	1	0.00E+00	2E-290	0.000504162	0.129483004	0.085400595
Dld	1	3.88E-20	2E-110	0.002765317	0.051713483	0.0853326
Orc5	1	1.07E-34	7E-78	-0.005561915	0.080908348	0.085296819
Deaf1	3.75328E-05	1.19E-24	1E-76	0.021227017	0.067964929	0.085224482
Fastk	1.05492E-05	6.62E-11	8E-121	0.016593343	0.036858892	0.085188533
Ankrd16	1	2.54E-05	6E-82	0.011267457	0.037441067	0.085183486
Naa40	1	1.31E-21	2E-99	-0.002172494	0.057241816	0.0851779
Gskip	1	7.04E-15	3E-87	-0.002356413	0.054831945	0.08514618
Zmat3	1	2.99E-14	2E-146	-0.000172037	0.03630275	0.085119633
Elp3	1	3.18E-13	1E-78	-0.00820291	0.053996232	0.085092004
Zc3h18	0.023526879	1.00E+00	9E-43	0.024814025	-0.004288251	0.085089998
Zfp951	1.58126E-10	3.04E-06	3E-81	0.023047712	0.039157628	0.085070598
Rrnad1	1	4.80E-02	5E-73	0.013246193	0.032633849	0.085042047
Hgs	0.028864852	1.18E-12	1E-83	0.015962219	0.050849691	0.085017799
Dnajc1	1	1.00E+00	9E-24	0.01161488	0.027531429	0.084985974
Gmps	0.015240252	1.00E+00	3E-52	0.021906001	0.01323881	0.084972203
Atpaf1	1	5.13E-29	5E-101	-0.008665617	0.06300045	0.084970938
Arhgap1	1	1.00E-20	2E-112	0.003665781	0.051666723	0.084917505
Sult2a5	1	2.53E-78	0	-0.000142048	0.021056079	0.08490757
Ncoa6	0.000998032	1.06E-07	2E-31	-0.027457138	0.068930957	0.084864149
Ces2c	8.64463E-06	2.67E-136	2E-205	-0.008755168	0.084614265	0.084847859
Chmp7	1	3.71E-23	4E-172	-0.001733177	0.039841939	0.084821833

Nedd1	0.000799415	4.62E-04	9E-51	-0.021082537	0.048912838	0.084819274
Cnbd2	1	3.03E-179	8E-168	-0.001191854	0.125497445	0.08480263
Mpv17l	0.021821772	7.85E-03	4E-68	-0.016993706	0.033758511	0.084784869
Gm41541	1	7.95E-100	8E-249	-0.002093722	0.05806466	0.084692358
Galk2	1	1.34E-13	1E-67	-0.01266775	0.063070185	0.084660385
Grhl1	1	1.22E-95	1E-277	-7.81073E-05	0.050016447	0.084647781
Zfas1	0.006222556	6.71E-106	5E-184	0.009317162	0.081747108	0.084631943
Hlcs	2.97104E-06	1.00E+00	3E-35	-0.029231299	0.044403584	0.084610492
Setdb1	1	2.93E-06	2E-46	-0.00259934	0.057548807	0.084551874
Wdr41	1	2.91E-06	4E-75	-0.006748551	0.044347894	0.084480668
Scly	1	2.67E-09	1E-36	0.007815084	-0.049787673	0.084448226
Tbrg4	1	1.53E-37	2E-135	0.008580856	0.058606529	0.084439387
Saysd1	1	2.19E-43	2E-109	-0.00142509	0.073293605	0.084292628
Gm45866	1	1.99E-75	7E-175	-0.006467224	0.069475162	0.084279729
Flad1	1	1.87E-09	7E-101	0.010330986	0.037667747	0.084246428
Rabl6	2.04657E-08	1.00E+00	2E-48	0.033993573	0.01076582	0.084206547
Dmtf1	1	4.58E-04	2E-50	0.016115383	0.048221192	0.084157645
Otud4	1	6.20E-08	2E-51	0.006218107	0.056533135	0.084125922
Tubb4b	1	3.03E-50	8E-163	0.005303519	0.06105092	0.084118509
Eif4ebp1	1	5.88E-110	5E-158	0.007584804	0.09331118	0.084100376
Pomgnt1	1	2.17E-37	3E-147	0.000469969	0.054116519	0.084082996
Dpp8	1	2.89E-20	5E-40	0.013075206	0.096691084	0.084038513
Pstpip2	0.490152095	2.08E-04	3E-36	-0.015334136	0.061883819	0.084005127
Cdc34	0.154953731	5.70E-81	5E-154	0.009734889	0.082215661	0.083949409
Fam102a	1	3.21E-31	1E-85	-0.000572017	0.069825466	0.083891634
Tor1b	0.003346322	1.00E+00	3E-68	0.019043504	0.024109502	0.083804728
Capn15	0.000204801	1.00E+00	7E-56	0.023003484	0.010363034	0.083757015
Rnf167	5.64101E-10	2.00E-16	2E-120	0.020133434	0.046323997	0.083708672
Tbc1d15	1	7.92E-15	1E-41	0.002235454	0.083630906	0.083695095
Ftsj3	5.73189E-14	3.93E-12	1E-106	0.025226317	0.041962114	0.083692941
Rpa1	1	4.57E-34	9E-118	-0.002072837	0.059296596	0.083609381
Gm10475	1	0.00E+00	0	8.68693E-05	0.113741924	0.083589564
Dclre1a	1	7.96E-61	1E-109	-0.002685674	0.085089458	0.083576725
Sapcd2	1	2.20E-245	0	0	0.055902766	0.083547586
Ddt	0.0003424	1.64E-10	4E-145	0.013636533	0.032605426	0.083525475
Srp19	0.765363278	1.29E-20	3E-110	0.011348133	0.051018806	0.08349256
Stard10	1.6107E-54	1.00E+00	6E-26	0.08836913	0.035010743	0.083486235
6030458C1	1	4.52E-15	1E-123	0.001102678	0.039346867	0.08345924
Gsta4	1.31603E-06	4.77E-197	6E-212	-0.008052436	0.104870319	0.083375694
Foxred1	0.009190849	7.00E-14	7E-140	0.011846446	0.038370374	0.0833518
Myo19	1	3.45E-42	3E-122	0.002116864	0.062854095	0.083307366
Cyp2c39	1.7446E-13	3.02E-165	6E-242	0.010573045	0.082274358	0.083286192
Brinp3	1	1.87E-170	9E-165	-0.00079012	0.123359665	0.083249313
Fbxo45	1	1.72E-15	2E-126	-0.004872286	0.040605897	0.083197059
Adrm1	0.004347246	1.98E-10	4E-135	0.012712238	0.032790465	0.083169388
Gm14326	0.003978889	5.88E-21	1E-94	-0.01371502	0.060726954	0.083074427
Hpn	1	1.00E+00	2E-27	-0.020280116	0.013058014	0.083051264

Coq8b	1	1.55E-24	2E-82	-0.008785528	0.062035181	0.08305059
Nipa1	1	2.31E-147	5E-286	-0.003171672	0.062232971	0.082888881
Nup88	1	1.75E-12	2E-78	0.001986232	0.049458951	0.082861729
Cct2	5.8819E-08	2.19E-33	2E-122	0.017561169	0.059888306	0.082815432
Cdyl	1	1.00E+00	2E-34	-0.004667473	0.029733651	0.082777118
Phka1	1	1.00E+00	1E-48	0.002859517	0.032657191	0.082747819
Cul5	8.85239E-06	1.00E+00	2E-44	0.027843565	0.035425576	0.082747749
mt-Atp6	1	4.94E-35	2E-165	-0.003815018	0.052721836	0.082747562
Dhx40	1	2.63E-10	9E-52	0.011493	0.064753684	0.08269595
Nars	2.58093E-20	6.03E-07	2E-66	0.037780159	0.050101525	0.082673895
Noc3l	1	3.17E-35	1E-120	0.008373649	0.058728363	0.082645123
Rnf20	1	1.00E+00	1E-55	0.014459103	0.009914335	0.082594815
Odr4	1	1.00E+00	2E-53	-0.000368931	0.014131937	0.082582966
Fanca	1	6.30E-202	7E-276	-0.000262875	0.076826717	0.082581396
Ehmt2	1	7.76E-18	8E-67	0.001556431	0.064872921	0.082437136
Mrps10	0.937987558	6.39E-14	1E-88	0.012720441	0.047422366	0.082424853
Asns	1	4.08E-188	0	-0.000945674	0.058844554	0.082406609
6330562C2	1	1.66E-166	5E-207	-0.005143461	0.095310329	0.082362169
Klc1	1	1.18E-30	2E-40	0.016708316	0.11037334	0.082350218
Lyplal1	1	1.74E-71	3E-149	-0.005504964	0.07256747	0.082335838
Vac14	1	1.28E-15	7E-64	0.011292493	0.064114459	0.082314099
Usp36	1.96975E-10	2.53E-02	7E-61	0.031380023	0.034664576	0.082311316
Limk2	1.92783E-05	3.81E-16	1E-78	-0.019571483	0.059248657	0.082257588
Smg5	0.01037064	3.13E-05	1E-69	0.018276337	0.041068392	0.082233082
Pds5a	1	1.04E-34	2E-27	0.011211092	0.129610292	0.082210956
Zmpste24	1	1.21E-35	2E-109	-0.004311026	0.063066522	0.082199239
Polrmt	0.250914106	4.00E-42	1E-116	0.011713916	0.066069299	0.082177965
Hars	1	6.36E-09	3E-93	-0.004020138	0.041786661	0.082114051
Rnmt	1	4.64E-23	1E-86	0.001447686	0.059893792	0.082041186
Sumf1	1	5.51E-09	9E-53	0.01215719	0.073301797	0.082004672
Slc38a10	1	1.36E-08	2E-82	0.011416061	0.04515106	0.081999078
Tmem41b	1	1.00E+00	6E-66	0.007352105	0.013647929	0.081944952
1110032A0	0.449941708	1.26E-19	1E-110	0.010688234	0.046783	0.081917827
A930007I1	1	7.11E-95	4E-139	-0.004252821	0.096131318	0.081883928
0610040J0	1	3.62E-12	2E-20	0.012583151	-0.075533767	0.081788113
Rnf144a	5.70724E-05	1.00E+00	3E-65	0.020838477	0.023930163	0.081778696
Morf4l2	1	3.25E-02	3E-81	0.006378145	0.031802242	0.081729521
Tsc22d4	1	3.68E-28	2E-111	-0.007317047	0.060931728	0.081691245
Gm48236	1	1.20E-114	1E-287	0.000679972	0.045310222	0.081643848
Dcxr	1	9.38E-12	3E-186	-0.005354271	0.025600702	0.081630008
Hells	1	6.23E-14	4E-101	-0.002897518	0.042191191	0.081628253
Mtrr	1	9.29E-106	2E-185	-0.001083095	0.076983924	0.081526188
Trim39	3.09322E-05	5.39E-16	6E-81	0.019922029	0.053685432	0.081484901
Rfc1	1	9.03E-02	3E-57	-0.008428724	0.034974769	0.081461155
Terf2	1	1.48E-03	1E-48	-0.005860522	0.044086775	0.081435254
Ppib	1	5.70E-06	6E-110	0.009310133	0.030656661	0.081385233
Pak1ip1	0.090930301	2.53E-12	4E-90	0.013810983	0.044783086	0.081305501

Tipin	1	5.37E-59	5E-149	-0.000644564	0.067368834	0.081223919
Gm17359	1	2.60E-168	2E-220	-0.001866762	0.086366102	0.081173359
Bag6	6.45823E-10	7.01E-03	9E-98	0.022074275	0.027433338	0.081165003
B230217O1	0.002928922	3.00E-13	2E-76	0.0164153	0.050439794	0.081114217
Atad1	0.001171178	1.09E-02	2E-59	0.020617448	0.03651914	0.08111418
Ppp2r2d	1.09524E-43	2.03E-13	1E-30	-0.067786797	0.082637155	0.081091111
Dpp7	1	1.23E-156	1E-230	0.002248069	0.084882125	0.080956754
Ciao3	0.692347492	1.70E-17	2E-113	0.010747214	0.045891191	0.080930301
Rab5if	1	9.84E-43	2E-73	0.009337333	0.088368938	0.080875657
Tom1l1	1	2.54E-02	5E-58	-0.003694597	0.034749503	0.080865781
Arhgap39	1.32541E-10	1.40E-25	4E-59	-0.026865241	0.085705231	0.080794548
Snrpa1	1	5.94E-25	5E-82	-0.001148447	0.064700137	0.080674928
Eif3c	4.95277E-10	1.00E+00	1E-51	0.032860808	0.024821226	0.080659234
Sult1e1	1	1.61E-60	7E-226	-0.000794969	0.029457511	0.080615224
Nek4	1	1.69E-23	2E-82	-0.003132905	0.061186865	0.080524425
Mrps28	1	1.00E+00	2E-39	0.010511283	0.01851118	0.080479168
Sipa1l2	2.44502E-18	8.23E-20	2E-70	-0.027730142	0.070387303	0.080473488
Ralgps2	1.74058E-31	3.71E-06	5E-19	-0.065475681	-0.035632084	0.080466444
Ints4	1	6.53E-05	6E-72	0.002749737	0.03580781	0.080428228
Polg	1	1.72E-10	2E-83	0.009662308	0.047111637	0.080361649
Pmepa1	1	4.30E-116	3E-212	0.000795187	0.070478483	0.080338024
Nktr	8.17269E-11	1.00E+00	3E-22	0.044139766	0.010711294	0.080322499
Nbl1	1	1.08E-222	0	0.00019659	0.057270986	0.080316438
Fam92a	1	1.26E-31	1E-184	-0.000213996	0.039359741	0.080309657
Hspa14	1	1.13E-15	2E-123	-0.000319099	0.039342971	0.08030228
Fmo4	1	2.69E-47	4E-141	-0.000842024	0.059664051	0.080208201
Mid2	1	1.68E-24	2E-83	-0.011299768	0.062029514	0.080201546
Slc22a23	2.6943E-123	1.84E-02	1E-12	-0.174437575	0.089336634	0.080173926
Rad17	1.26754E-08	1.93E-13	6E-61	0.029227279	0.062128978	0.080137824
Trmt6	1	3.48E-57	1E-153	0.006343662	0.064394891	0.080069075
Alk	1	1.04E-212	3E-275	0.002842553	0.082950459	0.080019852
Cox5b	1	2.02E-30	1E-156	0.003171164	0.047616289	0.079799134
Helq	1	3.00E-40	1E-117	0.003537436	0.063007496	0.079789889
Rps6kb1	0.000121451	3.62E-01	3E-28	0.03224134	0.046545066	0.079776721
Med1	1	1.00E+00	1E-45	0.001490798	0.029737353	0.07972084
Phtf2	1	4.59E-16	1E-49	-0.007175995	0.075562596	0.079714778
Scaf8	1	7.17E-43	9E-28	0.0217244	0.135585134	0.079679335
Prepl	1	7.36E-15	6E-105	0.004261092	0.041994059	0.079621421
Gdap2	4.67357E-05	1.00E+00	5E-43	0.027516445	0.013389274	0.079466647
Usp45	0.274220228	1.00E+00	8E-38	-0.017436589	0.022051953	0.079459568
Sass6	1	4.05E-82	5E-170	0.003940399	0.069270187	0.079396097
Esco1	1	1.00E+00	2E-47	0.014335238	0.034513059	0.079334877
Psmd6	1	1.30E-29	2E-122	0.009078613	0.05271828	0.079298357
G2e3	0.001169714	1.36E-31	5E-97	-0.013448393	0.067577746	0.07927851
Gm3776	1	0.00E+00	0	-0.000319791	0.116151938	0.079181576
Trappc11	1	1.45E-07	2E-77	0.008775421	0.039411485	0.079163767
Mpp1	1	1.67E-27	2E-116	-0.002588018	0.056565742	0.079146926

Rps5	1	1.00E-126	5E-164	0.005031541	0.093883553	0.079074247
Pprc1	1.05829E-13	6.81E-26	6E-99	0.024404659	0.057792424	0.078967654
Pnpla7	1.55858E-08	5.32E-138	2E-24	0.038177276	0.27991112	0.078964358
Krt18	1	4.30E-08	4E-53	-0.003103904	0.052697029	0.078949343
D230025D1	2.18118E-25	1.00E+00	2E-25	0.066659475	-0.028833452	0.078948587
Gm12498	1	0.00E+00	0	-0.000185668	0.128484425	0.078938053
Atp5g1	1	1.03E-33	5E-130	0.003762806	0.053318312	0.078872047
Poc5	1	2.96E-38	8E-129	-0.003712833	0.055291254	0.078841333
Ncln	4.6317E-08	2.09E-28	1E-117	0.01756114	0.05080026	0.078825131
Wdr83	1	3.61E-12	1E-139	0.003759797	0.031966602	0.078727708
Rps3a1	1	1.89E-62	1E-112	0.004494271	0.085133178	0.0786979
Ythdc2	1	6.45E-35	2E-81	0.005252642	0.071671474	0.078695531
Virma	1	1.76E-01	3E-47	-0.000942246	0.036262532	0.078687758
8030462N1	0.011824277	4.50E-02	2E-34	-0.021700481	0.047421231	0.078670153
Actg1	1	3.45E-116	3E-195	-0.000880502	0.075440359	0.078639856
Gemin5	1	1.17E-39	1E-80	0.001021084	0.07889488	0.078569841
Parp2	1	1.56E-35	2E-163	0.003141629	0.04600723	0.078434184
Gm34829	0.012692022	1.97E-154	0	0.002922409	0.045379557	0.078414994
E2f4	5.10219E-10	7.64E-55	3E-156	0.015263733	0.05963079	0.078310436
Galt	1	7.38E-24	7E-152	0.006579953	0.040030713	0.078268072
Prpf3	1	1.40E-15	3E-70	0.003993198	0.055029371	0.078154719
Ppcs	1	5.50E-46	7E-135	0.006576507	0.058064621	0.078149672
Rer1	0.013527574	1.75E-40	4E-120	0.011376901	0.060608457	0.078106397
Atp5k	1	3.36E-31	6E-106	0.001138455	0.062034478	0.078085399
Gm17690	1	8.97E-64	3E-140	-0.004314919	0.067033809	0.078082768
Rmnd5b	1	1.80E-28	2E-120	0.004490432	0.051360273	0.07804132
Arhgef1	0.010901023	3.78E-36	3E-59	0.017733596	0.100010456	0.077990272
3110082I1	1.78694E-08	1.93E-37	6E-70	-0.020332276	0.089259791	0.077972783
Ccnd1	1	3.42E-81	2E-267	0.000807429	0.042086032	0.077962235
Actr8	1	9.60E-26	2E-81	0.00231122	0.060420374	0.077960527
Bax	0.014669554	1.30E-80	2E-122	0.010859723	0.088917691	0.077915106
Clcn7	1	3.90E-60	1E-77	0.007782568	0.105675687	0.077906165
Qrsl1	1	3.58E-33	1E-156	0.00162866	0.043528781	0.077847548
Lzic	1	1.35E-34	3E-119	-0.001232881	0.053821001	0.077832075
1200007C1	1	0.00E+00	5E-270	-0.001321762	0.105754948	0.077818069
Hspe1	3.58256E-13	3.99E-31	3E-85	-0.022367004	0.068890675	0.077808692
Dnajb14	1	1.54E-20	1E-68	0.002452841	0.064772562	0.077782949
F12	4.18527E-11	1.35E-17	1E-30	0.04176499	-0.075068597	0.077702153
Dtymk	0.098672495	1.45E-42	4E-151	0.008944428	0.051850383	0.07769839
Ift43	1	6.24E-44	8E-87	-0.001694292	0.080808521	0.077649808
Zfp930	1	2.56E-37	2E-159	-0.001705674	0.04706968	0.077581889
Atp5j2	1	6.25E-08	4E-85	-0.003948412	0.039300803	0.077558623
Nono	1	1.07E-05	3E-65	0.008084406	0.041077989	0.077520542
Keap1	1	5.93E-24	4E-72	0.010151815	0.063694149	0.077410609
Prdx3	1	8.34E-48	2E-125	0.009358881	0.062483486	0.077304262
Gm28836	2.27605E-40	7.64E-72	7E-102	0.040515206	0.097179289	0.077248421
Ddx49	1	1.44E-13	1E-94	0.009612941	0.045180968	0.077245901

Bbs9	1	5.42E-07	1E-39	-0.002888105	0.061368234	0.077245109
1700042O1	6.39573E-30	9.99E-104	2E-62	-0.033072004	0.155238107	0.077226726
Atox1	1	6.46E-07	5E-63	0.011804004	0.045236563	0.077223367
Cln6	1	7.37E-107	2E-284	-0.001429293	0.044701198	0.077061571
Rorc	7.7772E-14	2.50E-58	9E-59	-0.025340868	0.110901142	0.077021371
Pkhd1	1.072E-37	4.97E-08	2E-19	-0.074663891	-0.053643497	0.076958965
Psmd8	0.140191666	7.79E-14	3E-122	0.01011376	0.038719621	0.076958657
Tmem167	1	2.11E-10	3E-53	0.008167607	0.054572369	0.076907626
Rttn	1	5.70E-63	9E-124	0.001125697	0.076567558	0.076853081
Gar1	1	1.59E-107	1E-218	0.005353885	0.061338401	0.07684765
Gm49970	1	2.16E-07	1E-84	0.01167003	0.035396917	0.076742901
Zfp54	1	1.74E-45	2E-185	0.003524412	0.043737957	0.07673629
Mpzl3	0.318252335	1.35E-07	1E-83	-0.01193613	0.036531687	0.076709728
Nme1	1	3.67E-79	8E-177	0.006035715	0.062534724	0.076523356
Med24	1	3.19E-28	7E-146	0.004559868	0.042107352	0.076489196
Golga1	0.259156161	1.00E+00	6E-38	0.021782767	0.01411691	0.076480289
Trub2	1	6.10E-34	8E-115	0.005291707	0.05745761	0.07647469
Emc8	1	3.59E-20	5E-70	0.010946793	0.060077207	0.076469153
Eme2	1	7.07E-79	5E-239	0.001568361	0.046023549	0.076418646
Phtf1	1	3.88E-19	1E-77	0.002524961	0.056774085	0.076345052
Atm	1	9.32E-81	3E-57	0.006943167	0.13423433	0.076336862
Prmt3	0.154480654	2.75E-38	4E-67	0.01471485	0.08443632	0.07633108
Aprt	1	1.62E-34	1E-136	0.003466563	0.050943846	0.076296793
Rccd1	1	8.09E-96	3E-157	0.001145654	0.07532086	0.076295721
Mtif3	1	6.46E-14	5E-81	-0.002210804	0.045760318	0.076220852
Gdf15	1	1.74E-126	2E-113	0.005321705	0.110359574	0.076132788
Borcs5	0.000812099	1.00E+00	2E-54	0.020628979	0.017555656	0.076071841
Heph	1	3.51E-177	2E-205	-0.000948259	0.103087917	0.076066303
Rnpepl1	1	1.35E-13	2E-69	0.006072653	0.055529429	0.07606376
Ift20	1	2.15E-06	3E-103	0.008749556	0.03120291	0.075890559
Riok1	0.001101875	1.64E-06	1E-61	0.019003385	0.04325732	0.075768033
Ipo5	1	8.39E-41	8E-84	0.005990177	0.074581368	0.075698395
Mast3	3.34011E-55	1.00E+00	2E-41	0.072798646	-0.001474801	0.075652613
Cdc37l1	1	1.00E+00	1E-26	-0.022700394	-0.003704059	0.075621376
Uck2	1	3.05E-07	8E-83	0.00333183	0.036738583	0.075602099
Glrx3	1	1.00E+00	9E-54	-0.002378984	0.020737169	0.075560724
Timm13	1	3.32E-34	7E-135	0.004013142	0.050753647	0.075503524
Ccdc59	1	2.93E-10	4E-81	0.008345301	0.040774759	0.075479558
Hdgfl2	1	4.26E-02	5E-56	0.01225858	0.031384416	0.075455914
A730004F2	1	1.45E-91	2E-133	-0.000169746	0.055842071	0.075427589
Ets2	1	2.40E-57	3E-142	-0.000908633	0.06299971	0.075417756
Kin	1	4.93E-04	4E-67	0.01134527	0.034394469	0.075409204
Mroh9	1	0.00E+00	0	0.000158164	0.093170668	0.075402147
Fam120b	1	8.17E-01	6E-36	0.003108054	0.037210701	0.075293892
Dhx57	1	1.00E+00	3E-44	0.011183788	0.016586605	0.075236392
Tmem154	1	0.00E+00	8E-298	-0.000258178	0.10123555	0.075184088
Rrp12	0.65872295	1.13E-108	4E-194	0.0058123	0.066283033	0.075116502

Gale	0.001424068	1.96E-09	2E-225	0.006738453	0.015893806	0.07510578
Fip1l1	0.117312358	2.47E-04	6E-30	0.02410321	-0.037749378	0.075068415
Clptm1l	1.0254E-08	8.00E-08	4E-106	0.018227356	0.032180965	0.075062166
Zfp408	1	7.06E-04	2E-95	0.005189549	0.026913078	0.075060245
Chm	1	1.00E+00	4E-46	-0.004553666	0.017545202	0.075046198
Akr1a1	1	1.42E-23	2E-32	0.000787492	0.106313767	0.074986107
Gm48228	0.000660978	1.00E+00	9E-49	-0.02247124	0.001082407	0.074977727
Zbtb40	0.278733515	5.43E-06	5E-46	0.016789726	0.049445392	0.074952348
Tcf7l1	0.035197744	1.00E+00	6E-21	-0.036924269	-0.034314494	0.074947081
Ears2	1	1.01E-23	1E-98	0.006590668	0.051098146	0.074897849
Grem2	6.48969E-75	3.01E-05	1E-06	-0.084717085	-0.016704129	0.074891161
Cr1l	1	1.17E-01	4E-55	0.007029978	0.032998119	0.074876591
Prdm15	1	1.00E+00	4E-57	0.014425727	0.023415063	0.074863511
Dnajb12	1	2.64E-01	3E-41	0.006962724	0.03639809	0.074810933
Rpl24	1	4.62E-57	1E-84	-0.005736956	0.090091572	0.074793079
Thoc5	1	1.86E-38	4E-104	0.000374015	0.061447291	0.074757284
Fes	1	1.99E-45	2E-99	0.002495969	0.068390057	0.074734794
Hes1	7.46771E-14	0.00E+00	3E-178	0.013550349	0.153386935	0.074704716
Prkcsh	1	1.85E-11	6E-116	0.00661158	0.032952802	0.074615553
Il4ra	0.8527078	3.84E-70	3E-84	-0.008955031	0.09837971	0.074589244
Pomt1	1	1.40E-23	1E-142	0.002808646	0.03754043	0.074577983
Aldh9a1	1	1.00E+00	2E-40	0.011870458	0.000484002	0.074525456
Zfp53	1	1.00E+00	1E-55	-0.005919667	0.028915871	0.074488563
Arel1	1	3.27E-01	2E-32	-0.003661282	0.045114326	0.07444975
Gnpnat1	1	1.17E-10	5E-76	-0.002240468	0.043663997	0.074417621
Mnat1	1	1.00E+00	3E-29	-0.014005426	0.016717777	0.074399623
Ccng1	1	2.31E-34	3E-157	0.003012769	0.042802758	0.074317781
Tmem86b	1	9.18E-05	2E-88	0.006475715	0.029596701	0.074314755
Usp14	5.41894E-07	1.00E+00	3E-42	0.028477467	0.003503485	0.074294297
Psmd2	3.48048E-07	7.96E-07	4E-73	0.021675542	0.039319379	0.074250546
Ddx20	0.011882104	1.67E-69	2E-156	0.009213693	0.062859471	0.074244225
Ccnt2	1	1.00E+00	6E-28	-0.006874565	0.03017909	0.074193734
Zfp119a	1	1.82E-01	8E-117	-0.00159797	0.016884136	0.074096359
Ube3a	1	2.21E-08	7E-24	-0.012319729	0.071894841	0.073996064
Psmc6	5.08567E-08	1.28E-02	1E-45	0.029675482	0.042058616	0.073984015
Pi4kb	1	1.00E+00	7E-34	-0.001144773	0.010718277	0.073966592
Mctp2	1.24879E-98	9.46E-02	1E-18	-0.123456164	-0.037983716	0.073938234
Nkiras1	1	1.00E+00	5E-54	0.002776656	0.000630401	0.073932484
Eps15l1	1	1.00E+00	7E-33	0.016517941	0.01402493	0.073874419
Mthfd2	1	6.25E-62	5E-106	0.003681809	0.076717434	0.073865593
Synj2	1	2.64E-110	3E-156	-0.00037522	0.080875037	0.073847097
Actr6	9.17954E-17	1.00E+00	3E-65	0.031833212	-0.003902915	0.073752133
Ccdc186	1	3.12E-10	1E-66	0.01319113	0.048940479	0.073704827
Agbl3	1	3.86E-116	6E-133	-0.003544073	0.094611912	0.073689009
Rab3gap1	1	1.00E+00	1E-30	-0.004927977	0.01969543	0.073674098
Gtf3c3	1	6.34E-18	1E-70	-0.004902232	0.053246154	0.073634266
Commd3	1	4.05E-29	3E-127	0.005013204	0.045554297	0.07346401

Tmem179b	5.23759E-16	1.22E-41	3E-124	0.020945881	0.056257793	0.073443107
Syvn1	9.95058E-56	1.00E+00	3E-56	0.061151958	0.021452933	0.073434604
Map2k3	1	2.26E-18	1E-53	-0.007520057	0.065959412	0.073320394
Ankrd49	1	2.30E-101	4E-154	0.00276258	0.078086773	0.073302143
Naa20	0.000868029	1.01E-43	1E-91	0.014143465	0.073056212	0.073267466
Sharpin	1	8.06E-12	2E-115	0.007235335	0.032287711	0.073246216
Prrg2	1	1.00E+00	4E-65	-0.004854536	0.010505408	0.073226822
Ly96	1	1.31E-40	1E-99	-0.003968214	0.06635371	0.073210207
Gm49890	1	2.73E-56	4E-148	-0.005112355	0.056030513	0.073209864
Prdm10	1	1.00E+00	2E-23	0.009248357	0.018800515	0.073183425
Prkci	1	1.00E+00	1E-58	0.009981388	0.020388669	0.073108243
Ercc4	1	2.53E-18	6E-69	0.006960823	0.054267557	0.07300375
Letm1	2.57147E-06	9.13E-04	1E-79	0.018269108	0.029868275	0.07297749
Plekha5	1.39893E-09	4.76E-14	3E-13	-0.049302591	0.111017255	0.072933853
Lman1	6.21362E-35	2.23E-02	7E-33	0.062708844	-0.027400755	0.072795618
Golga7	1	1.46E-03	3E-68	0.008233268	0.031994452	0.072759239
Ndufs1	1	1.58E-11	1E-37	0.015946476	0.064204522	0.072720845
Mbp	1	2.73E-281	1E-273	-0.000252549	0.084970605	0.072705552
Qars	1	8.11E-19	4E-110	0.00586229	0.039635974	0.072663555
Ppox	8.61982E-08	1.00E+00	6E-92	0.018613584	0.012348527	0.072654046
Atg9a	0.003487003	9.84E-02	1E-81	0.014598731	0.024290033	0.072635923
Edc4	0.040993448	6.19E-08	5E-86	0.012894195	0.035783185	0.072621522
Rbks	0.640379181	1.00E+00	2E-28	-0.01896648	0.012134235	0.072604303
Ndufaf2	1	2.20E-02	2E-45	-0.01113274	0.036200466	0.072522582
Wdr11	1	6.83E-01	9E-49	0.008030878	0.034984267	0.072522545
Tcea1	8.21998E-17	1.00E+00	2E-34	0.04550571	0.022726063	0.07249756
Aifm1	1	1.00E+00	3E-35	0.00388239	-0.009422499	0.072447446
Ahctf1	1.0359E-115	1.25E-01	1E-22	0.126776534	-0.028558148	0.072436529
Rora	3.16058E-26	5.35E-01	4E-16	0.07461043	-0.030514348	0.072434686
Snapc4	1	3.39E-07	2E-88	0.008557937	0.03216506	0.072397312
Rnft2	1	3.27E-205	5E-278	0.000742679	0.06576864	0.072373599
Cep135	1	5.42E-23	4E-71	-0.0007116	0.060444884	0.07236683
Ing3	1	4.05E-04	9E-71	0.011621997	0.031286747	0.072362742
Prpf4	1	4.68E-25	3E-87	0.011325072	0.054252734	0.072290886
Dnajc8	0.136821556	1.46E-19	7E-61	0.015231506	0.062278529	0.072236483
Smad4	1	1.00E+00	1E-29	-0.007430071	0.006741422	0.072108268
Tsr1	1	1.12E-42	1E-130	0.005298275	0.051773014	0.072106347
Ptpn23	1	5.13E-17	3E-95	0.002295361	0.045211991	0.072081452
Gtf2f2	1	1.59E-01	9E-35	0.000466196	0.041501068	0.072070681
Ctsd	1	1.11E-264	2E-117	0.003019892	0.19294689	0.072052106
Ncapd3	1	2.00E-07	3E-33	0.005051959	-0.03907554	0.072012985
Eif3e	0.032437283	7.36E-10	1E-73	0.013879677	0.039019897	0.071956596
Tmem104	1	2.86E-39	9E-85	-0.003526452	0.079326461	0.071954872
Cdc5l	1	1.00E+00	3E-34	0.006667325	0.009173553	0.071918308
Wdr12	6.38425E-06	3.29E-13	2E-55	0.021033914	0.053100447	0.071797584
Eppk1	1	1.00E+00	2E-42	-0.002623181	5.15212E-05	0.071795744
Usp33	1	1.00E+00	2E-23	-0.011545379	0.001460234	0.071794444

Otud6b	1	1.43E-32	2E-95	-0.003115434	0.054560485	0.071791519
Zfp955b	1	1.13E-04	8E-101	0.009688594	0.024232629	0.071759532
Tubd1	1	1.56E-42	8E-102	0.002221508	0.064536261	0.071719137
BC003965	8.691E-13	3.58E-12	3E-99	0.021507919	0.035235163	0.07167297
Mthfsd	1	1.84E-28	3E-124	-0.001800778	0.043188248	0.071562383
Iscu	0.002946638	1.12E-08	6E-107	0.012650167	0.031848086	0.071481595
Gm32511	1.43663E-21	1.00E+00	9E-48	-0.034148546	0.022784499	0.071479186
Acadvl	6.81811E-05	1.61E-07	1E-61	0.019338765	0.04058784	0.071363366
Gm4258	1	5.52E-88	1E-220	0.001662102	0.048690402	0.071347843
Stat6	1	5.03E-27	3E-60	-0.007836567	0.074418145	0.071234875
Acot8	1	1.22E-05	7E-64	-0.001932241	0.034011756	0.0712138
Mrps18b	0.003327142	1.24E-36	8E-143	0.009645888	0.04358986	0.070977265
Gm12536	1.63705E-14	3.14E-83	1E-171	-0.011066719	0.05891535	0.070973824
Zcchc14	8.00133E-32	1.00E+00	3E-28	0.061864256	0.018315653	0.070959627
Mroh1	1	5.76E-26	2E-47	-0.012713892	0.08442676	0.070956611
Bnipl	0.003522613	1.60E-92	9E-198	-0.005576322	0.05512123	0.070868019
Dpm1	1	1.00E+00	1E-34	0.012787698	0.028371462	0.070686454
Akr1c13	1	1.89E-71	5E-103	0.000213893	0.079378246	0.070667523
Atp5h	1	1.00E+00	4E-70	0.008767159	0.0210308	0.070640104
Stx17	3.37937E-12	1.00E+00	5E-37	-0.032122347	0.019103254	0.070571666
Drg2	0.000534113	1.76E-09	1E-102	0.013701359	0.031560258	0.070550325
Gm16559	1	3.26E-72	9E-213	0.004196174	0.044928937	0.070550008
Fbh1	1	1.42E-08	6E-68	0.007568837	0.040443906	0.070549863
Rab11fip2	1	6.56E-25	3E-58	-0.001329282	0.06877257	0.07054647
Ric8a	1	9.06E-46	9E-137	0.007929411	0.051245148	0.070470055
Mrap	2.10588E-06	1.00E+00	5E-45	-0.020799674	0.004535516	0.070464693
Ppp4r2	1	1.00E+00	3E-31	-0.009643424	0.027557765	0.070380017
Exosc8	1	2.06E-31	2E-143	-0.000656508	0.040085975	0.070356937
Ubqln1	1	1.00E+00	4E-29	-0.012591724	-0.006951311	0.070327479
Arhgef16	1	1.27E-141	6E-199	-0.003043727	0.069543174	0.070223723
Utp4	1	4.62E-21	2E-73	-0.000939168	0.052290799	0.070191553
Mapk3	1	2.50E-87	1E-180	0.000175364	0.061152372	0.07012248
Acat2	8.3755E-12	1.00E+00	3E-63	0.027444672	-0.002569395	0.070107199
Capns1	1	5.71E-18	3E-88	0.001985258	0.045312	0.070095609
Sema4b	4.10047E-09	6.68E-71	3E-102	-0.014296907	0.077056383	0.070013555
Ciz1	1	1.32E-07	1E-60	0.010126864	0.040586789	0.069993675
Rpl36al	0.301235734	3.49E-21	2E-87	0.011225105	0.050001897	0.069930936
Plcd3	1	1.36E-168	1E-165	0.000988044	0.091637631	0.06992848
Slc18a1	1	8.44E-15	6E-59	-0.01074021	0.052249897	0.0698646
Ssbp1	1	1.36E-31	4E-45	0.001437657	0.0909075	0.0698107
Lcorl	1	1.00E+00	1E-15	-0.003673947	0.029482755	0.069736214
Hectd2	1	4.09E-27	2E-39	-0.003968057	0.08708091	0.069732651
Stk39	1.35308E-10	1.70E-25	4E-66	-0.016612935	0.06503984	0.069671162
Pla2g7	1	0.00E+00	2E-250	-0.000406252	0.147064536	0.069641462
Sh3yl1	1	4.12E-31	4E-96	-0.001431587	0.051428804	0.069619448
Mrtfb	1	6.75E-88	4E-09	-0.006197113	0.253998097	0.069594318
Crnkl1	8.89184E-05	5.43E-43	2E-104	0.013146144	0.059551487	0.069573928

Pnpt1	2.45283E-06	1.18E-13	8E-74	0.018431247	0.045244456	0.069499357
Adat1	1	1.21E-10	1E-45	0.00074916	0.05391032	0.069484466
Spns1	0.031511829	7.21E-08	9E-77	0.013408149	0.035357489	0.069437705
Rpl7a	0.003096393	8.92E-76	3E-101	0.012327	0.08552612	0.069436713
Fap	0.236880965	1.88E-147	7E-147	-0.006053354	0.096434391	0.069408879
Mtmr1	1	1.46E-01	1E-39	-0.012713331	0.041843548	0.069337759
Phb	1	4.99E-13	2E-57	-0.003668845	0.049324326	0.069209349
Nup210	1	6.61E-11	4E-62	-0.010118985	0.044523555	0.069162879
Tank	1	8.16E-11	7E-38	-0.001833364	0.069094815	0.069155891
Asxl2	1	4.03E-18	2E-20	-0.003519137	0.095489972	0.069154931
Stk11	0.000307545	2.45E-10	5E-39	0.02422446	0.059435266	0.069037297
Mettl16	1	1.03E-02	5E-46	-0.002611605	0.035312834	0.069022182
Icam1	0.023880315	1.43E-98	4E-123	-0.006703949	0.086242676	0.068936495
Rps17	1	2.90E-78	2E-102	0.003041142	0.085714258	0.068899813
Myl12a	1	1.35E-11	4E-114	-0.005808638	0.030186361	0.068888698
2810004N2	1	1.00E+00	2E-32	0.016024744	0.020031145	0.068871717
Spopl	0.368720773	4.46E-34	5E-35	-0.016701285	0.105348284	0.068718583
Rrn3	1	8.48E-27	5E-59	-0.000954288	0.066570261	0.068702036
Kmt2b	3.46388E-10	1.00E+00	3E-44	0.030965408	0.019801391	0.068609208
Cops3	1	1.00E+00	5E-40	0.007090539	0.017290828	0.068570626
Mak16	1	9.81E-38	4E-102	0.008391397	0.056581278	0.068504492
mt-Nd1	1	9.64E-36	7E-131	-0.004042274	0.048236794	0.068484756
Ube3b	1	1.00E+00	2E-41	0.01497595	-0.002730842	0.068450362
Senp3	1	3.07E-28	6E-77	0.010060832	0.05954627	0.068444036
4931406C0	1	8.88E-08	3E-32	-0.011018916	0.059008276	0.068383399
Smo	0.000286374	2.05E-01	2E-97	-0.011158608	0.017207111	0.068339296
Ncl	1	2.88E-09	6E-37	-0.012077893	0.056824043	0.068324068
Ppp2r3c	1	1.62E-20	1E-78	0.004286303	0.050781391	0.068274905
Uggt2	1	2.40E-19	3E-122	-0.004024939	0.034349233	0.068273877
Kcnj8	1	5.91E-177	3E-296	0.000117081	0.043481595	0.068268901
Itgb3bp	1	2.82E-53	8E-99	-0.000587484	0.069864977	0.068229189
mt-Co2	0.000621889	3.02E-30	3E-90	-0.012480012	0.06140681	0.068157045
Timm10b	0.003001727	9.80E-28	3E-145	0.009050218	0.038205586	0.068133878
Trappc13	1	1.34E-01	7E-63	0.010206542	0.024059222	0.068130371
Mettl23	1	9.30E-52	4E-84	0.008697292	0.07499037	0.068110896
Cct3	1	5.85E-05	2E-50	0.004731047	0.037232876	0.068071661
Nr2c1	1	4.56E-06	1E-31	0.007659268	0.055691036	0.068063242
Zswim4	1	7.76E-10	1E-71	0.003578779	0.040407404	0.067981383
Spata5	1	1.00E+00	3E-26	0.003654491	0.032476274	0.067935508
Asah2	4.23996E-11	2.99E-04	5E-60	-0.021452163	0.035251184	0.067934349
Ppfia1	1	1.00E+00	8E-24	0.001388286	-0.011026391	0.067921073
Dhx33	1.2195E-06	1.80E-13	9E-76	0.01818999	0.042831057	0.067872866
Tomm5	1	6.86E-134	7E-205	0.003623973	0.063633191	0.067862578
Mafk	1	8.27E-75	2E-121	-0.005094311	0.070928412	0.067793053
Car2	1	5.01E-113	2E-238	-0.00041018	0.045937275	0.067669108
Arg1	4.6935E-230	7.50E-46	3E-20	0.259065337	-0.245638706	0.067655617
Gnptab	1	5.02E-34	6E-73	-0.004357892	0.068312497	0.067630456

Rpl36a	1	5.73E-64	2E-91	-0.001151881	0.078807844	0.067586579
Smad1	1	1.00E+00	8E-24	0.007852251	-0.012833602	0.067526701
Nipa2	1	4.65E-07	1E-33	-0.000225081	0.05655276	0.067491951
Dis3	1	4.10E-46	1E-95	0.005265716	0.062204816	0.067449168
Trim11	0.092356639	2.68E-03	7E-50	0.016803457	0.035455425	0.067435657
Nmd3	1	3.05E-10	6E-46	-0.001073442	0.05117172	0.067433288
Bop1	1	2.94E-41	1E-101	0.005007793	0.057143112	0.067402981
Gstp3	1	2.93E-102	3E-202	0.000882655	0.052908365	0.067382495
Pigq	1	5.17E-10	3E-105	0.001832766	0.029141599	0.067290459
Arhgef7	9.94456E-62	1.78E-42	2E-13	-0.088614444	-0.114353626	0.067283917
Tbc1d31	0.111950281	1.41E-06	2E-52	-0.011969761	0.038677048	0.067260878
Vps11	1.41084E-09	1.92E-05	2E-53	0.025580471	0.035931811	0.067221118
Tirap	1	2.74E-01	9E-66	0.007225737	0.022830655	0.067217869
Rpl30	1	7.79E-102	1E-122	0.00322825	0.08197714	0.067197011
Nfs1	1	2.03E-02	1E-36	-0.010544493	0.037040557	0.067010326
Gm14964	1	0.00E+00	1E-263	6.91421E-05	0.111962494	0.066966786
Snhg14	1	5.64E-54	3E-110	-0.005549472	0.061461892	0.066952686
Gm13391	1	1.12E-122	2E-271	0.000662627	0.037789021	0.066935845
Mthfd2l	1	2.65E-40	3E-124	-0.004059607	0.049026113	0.066931505
Cfl2	1	7.44E-16	2E-78	0.000103093	0.044706828	0.066928591
Sap30bp	1	1.53E-01	1E-50	0.011752715	0.029129417	0.066905719
Ptp4a3	0.000245487	4.29E-139	4E-222	0.005882756	0.057932725	0.066899676
Pkp1	1	7.92E-170	2E-286	3.39637E-05	0.040916293	0.06689432
Trmu	1	2.40E-04	4E-77	0.008356027	0.02660607	0.066860294
Trmt11	1	1.00E+00	8E-57	0.000833156	0.020841294	0.066826439
Zfp330	1	2.01E-18	2E-92	0.009118092	0.041498539	0.066809613
2010001A1	1	6.56E-142	3E-155	-0.003357968	0.087405704	0.066791034
Klhl20	1	7.74E-04	8E-48	0.003586058	0.037744711	0.066785082
Pnn	3.35973E-62	2.20E-05	1E-17	0.097542357	0.06317589	0.066781294
Tulp4	8.29734E-05	1.00E+00	3E-19	-0.028076347	-0.022446089	0.066764602
Focad	1	5.35E-02	2E-25	-0.007427537	-0.028084027	0.066757992
Alkbh8	1	1.00E+00	4E-40	-0.009032418	-0.001847637	0.066679592
Snf8	1	3.32E-07	8E-66	0.011919437	0.036011189	0.066507258
Atp6v0e	1	5.21E-19	1E-56	-0.002981635	0.057092723	0.066475089
Rnf40	1	2.95E-15	4E-76	0.008652532	0.042861171	0.066473705
Sf3b1	1	1.81E-29	5E-21	0.009441434	0.117625646	0.066383429
Klhl22	1	5.97E-22	8E-63	-0.000608399	0.056047037	0.066382747
Gm10447	1	1.50E-77	2E-260	-0.000417686	0.030142234	0.06621018
Gm42982	1	2.57E-66	2E-122	-0.001523949	0.058325942	0.066205179
Mcm7	1	1.30E-53	3E-118	0.006484757	0.057501393	0.066196373
Tmbim4	0.810025709	4.37E-20	4E-69	0.011979072	0.052574691	0.066179707
Nub1	1	4.80E-23	3E-39	0.006388497	0.080955129	0.066164816
Elp2	1	4.91E-11	4E-68	0.009246026	0.040998484	0.066139494
Mrpl12	1	1.13E-18	5E-79	0.003175128	0.045947029	0.06601075
Mpp5	5.19434E-51	1.00E+00	2E-11	-0.07120547	0.005208573	0.066000712
Ddx27	3.60996E-13	5.27E-02	6E-58	0.026882678	0.025167469	0.065951475
2610021A0	1	2.03E-29	1E-78	0.010142986	0.053579447	0.065945309

Sirt7	6.94679E-10	1.00E+00	1E-37	0.031317808	-0.001047955	0.065900011
BC017158	1	1.52E-25	1E-94	-0.003536039	0.044777298	0.065857335
Wipi2	1	1.00E+00	6E-25	0.005604248	-0.005805854	0.065851058
Brpf1	3.19069E-09	1.54E-01	2E-61	0.022527672	0.027735992	0.065844102
Dhx32	1	4.99E-10	7E-43	-0.004852917	0.049187716	0.065819679
Plekhj1	0.550040455	3.14E-34	1E-123	0.007686163	0.045458352	0.065811727
Rap1gap	1	3.02E-41	5E-174	0.004468386	0.037621717	0.065739996
Dpp3	1	1.00E+00	4E-55	0.00504779	0.016587629	0.065735964
Ift52	1	1.25E-16	1E-47	0.000425661	0.062068007	0.065714324
Lmf1	5.65876E-16	1.00E+00	3E-36	-0.032914533	7.3819E-05	0.065640566
Hmcn2	1	2.47E-16	2E-93	0.000560801	0.03675755	0.065628319
Os9	4.00808E-22	2.91E-01	9E-33	0.045230753	-0.021130821	0.065550224
Exosc1	5.43138E-12	4.34E-17	2E-90	0.020068205	0.038143315	0.065529856
Ptgfrn	1	6.31E-173	3E-246	-0.000540552	0.056795245	0.065504701
Ptbp2	7.24658E-05	3.55E-19	1E-28	-0.023281983	0.087970775	0.065498761
Cul2	1	1.00E+00	1E-29	0.01406408	0.003753864	0.065496062
Tm9sf2	1.01241E-14	1.00E+00	4E-27	0.041446863	0.031338241	0.065448916
Hps3	1	2.24E-55	2E-63	0.009272446	0.090791128	0.065440955
Atp6v0a2	1	7.49E-08	5E-46	0.01392541	0.044334566	0.065435297
Mmadhc	2.1879E-05	9.96E-21	2E-58	0.018989833	0.057018222	0.065424668
Golga2	1	1.00E+00	2E-52	0.008153025	0.014304933	0.06540166
Ago4	3.50556E-13	1.00E+00	5E-41	-0.027180308	0.007276829	0.06539952
Slc17a9	1	5.25E-01	4E-74	0.001141436	0.021671609	0.065384012
Ipo13	1	2.74E-30	1E-126	0.004116981	0.041589924	0.065367608
Ap4e1	1	1.45E-19	1E-52	-0.007689808	0.060890327	0.06535317
Ube2o	1	2.26E-24	3E-53	0.002369508	0.068270941	0.065298516
Sap30l	1	7.10E-42	6E-111	0.002836625	0.052767542	0.065256305
Asrgl1	0.042967188	2.54E-41	1E-123	-0.006768275	0.045708754	0.065252744
Ndufb9	1	2.50E-35	2E-81	-0.000349671	0.060791444	0.065220491
Ip6k2	6.7608E-146	1.74E-01	5E-22	0.138611114	-0.025251366	0.06519885
Casp8ap2	1	1.00E+00	2E-45	0.001861058	0.025732714	0.065161941
Gm28863	5.51444E-05	1.50E-68	1E-105	-0.010892205	0.070260304	0.065143469
Dcaf12	1	4.12E-04	1E-36	0.003142423	0.043553398	0.065126276
Psmd1	9.49399E-20	1.00E+00	4E-18	0.058915367	0.020081006	0.065075259
Eif4e	1	1.00E+00	6E-39	-0.001787778	0.023413088	0.065068095
Ripk4	0.126330863	5.78E-53	4E-109	-0.007873818	0.057239935	0.064948599
Atic	1	6.51E-107	7E-134	0.005450492	0.077071444	0.064894016
Senp1	1	1.40E-08	6E-40	0.005641391	0.050882311	0.064837349
Tnfrsf1a	1	1.03E-49	1E-105	-0.001015088	0.060638236	0.064828174
Ankrd6	1	1.89E-199	1E-147	0.003464712	0.10994304	0.064799267
Gm44767	6.92054E-07	4.52E-19	3E-106	-0.011686231	0.035506032	0.064762356
Pdss2	1	5.12E-08	1E-12	-0.015302969	0.081649605	0.064760092
Prag1	1	3.38E-150	7E-221	0.000727346	0.057467116	0.064757976
Gm14319	0.118883129	2.20E-136	7E-215	0.004404401	0.05613966	0.064734428
Rtcb	0.332208391	1.27E-13	2E-81	0.010719613	0.040021423	0.064732098
Abraxas1	1	2.95E-18	3E-77	0.008028392	0.042158884	0.064729707
Uqcr11	1	6.20E-39	7E-86	-0.008234761	0.064705382	0.064678846

Ubap2	1	1.00E+00	2E-26	0.012885958	0.019908002	0.064672587
Cd46	1	2.30E-16	6E-51	-0.002860926	0.055058981	0.064647866
Add1	3.19844E-07	7.39E-06	2E-39	-0.022907112	0.052311047	0.064603131
Vps36	1	1.81E-17	1E-73	0.006659741	0.044463335	0.064550628
Vps50	1	9.43E-01	2E-30	-0.001308549	0.03414589	0.064544633
Psmd12	1	1.00E+00	1E-31	0.018938589	-0.018751465	0.064526844
Cnst	1.50283E-38	1.00E+00	1E-39	0.052503539	0.023104341	0.064502973
Mettl17	0.000145694	1.00E+00	8E-58	0.017562233	0.002426139	0.064486427
Cdkl3	1	4.49E-125	8E-214	-0.001094358	0.0542827	0.064463915
Ntmt1	1	4.78E-08	4E-63	-0.004072644	0.035049729	0.064455736
Chd1	4.82686E-06	1.00E+00	6E-20	0.032173844	0.032942714	0.064430348
Cct4	1	3.28E-14	2E-55	0.003346341	0.049307355	0.064425116
Tm7sf3	1	2.59E-06	9E-46	0.012513868	0.040975124	0.064418653
Mrpl48	1	1.00E+00	5E-38	-0.005698106	-0.005373537	0.064352184
Fam72a	1	4.64E-77	7E-202	0.001305922	0.040412537	0.064331984
Ext2	0.000100692	5.88E-05	1E-21	-0.029056982	-0.046171251	0.064330937
Smc3	1.42931E-15	1.00E+00	2E-30	0.039402257	0.00441717	0.064302704
Apoc3	1	1.08E-03	4E-16	-0.003078259	-0.028126164	0.064275827
Rexo4	0.220707791	1.00E+00	2E-64	0.012213286	0.017298583	0.064275677
Arcn1	0.005320741	1.00E+00	4E-31	0.022661532	0.029912457	0.064260691
Exoc7	1	4.15E-11	1E-76	0.004144948	0.035780664	0.064192438
Gtf2h2	1	4.72E-01	2E-48	-0.000534294	0.025493566	0.06416596
Kdf1	1	3.80E-71	3E-177	-0.002403958	0.04592176	0.064150298
Mios	1	4.31E-07	1E-58	0.002595765	0.034831414	0.064108583
Mknk1	1	3.55E-13	4E-61	-0.007626876	0.045400636	0.06403688
Epb41l1	1	4.41E-207	4E-139	0.004693896	0.118535226	0.063970371
Eif4ebp2	4.59151E-38	1.00E+00	1E-17	0.073621851	0.042028735	0.063946222
Bcap29	1	1.90E-40	1E-69	-0.004316413	0.067221454	0.063938014
Hs2st1	3.17856E-08	1.00E+00	3E-18	-0.033504324	-0.009272013	0.063935596
Smpd4	1	1.39E-03	2E-72	0.006787428	0.025062653	0.06390403
Ppil4	3.02154E-11	1.00E+00	8E-34	0.03415869	0.006218524	0.063884488
Gmppa	1.77489E-10	1.00E+00	1E-80	0.02005264	0.016271697	0.063872108
Chpf2	1	7.74E-05	2E-76	0.003397089	0.026579505	0.063851382
Terf1	0.058883246	5.40E-06	2E-35	0.019148637	0.047703042	0.063822921
Prodh	4.40532E-08	5.27E-39	7E-22	0.033705385	-0.093961589	0.063816743
Caprin2	2.57247E-05	2.15E-42	5E-121	-0.009128215	0.048490825	0.063807186
Mat2b	0.046017547	5.24E-09	9E-73	0.01301006	0.035453915	0.063785394
Chfr	1	1.00E+00	5E-31	0.01782057	0.030681304	0.063775875
Nt5c	0.07394806	1.00E-43	4E-152	0.006832556	0.040162336	0.063762694
Tma7	1	2.57E-17	4E-81	0.002085827	0.04341732	0.063728273
Sergef	1	1.94E-09	8E-44	-0.004285672	0.053121542	0.063703045
Gm42067	1	4.27E-31	1E-126	0.002003055	0.0382072	0.063695251
Zc3h8	1	7.61E-05	2E-80	-0.003505426	0.025225521	0.063653903
Ankrd26	1	7.45E-06	1E-42	-0.005839684	0.037297362	0.063634527
Gm9967	1	4.17E-217	3E-196	-5.88057E-05	0.082732831	0.063627603
Ccdc17	1	8.90E-52	4E-188	-0.001142689	0.034822347	0.06360449
Crppa	1	4.74E-01	2E-34	-0.014391113	0.035938596	0.06359782

Rpl31	1	7.10E-45	4E-100	-0.003079644	0.058955213	0.063558193
Ap2a1	1	1.02E-01	1E-53	0.010698602	0.026827316	0.063519494
Cers2	0.168755772	1.00E+00	2E-39	0.015872062	-0.00881992	0.063505356
Cc2d1a	1	1.14E-18	2E-102	0.00032281	0.036695286	0.063486406
Fbxl5	1	3.58E-96	5E-67	-0.003893852	0.120368157	0.063485496
Hint1	1	1.42E-08	6E-52	0.002068064	0.044506987	0.063438688
Rpn2	4.69014E-06	1.00E+00	2E-53	0.019575238	0.012024085	0.063417755
mt-Co3	1.13119E-32	1.31E-10	1E-43	-0.038472662	0.059057155	0.063364086
Dhx34	1	1.03E-27	7E-86	-0.003620943	0.048240958	0.063361375
Inppl1	1	6.38E-16	3E-59	0.004147485	0.050585523	0.063344739
Kctd18	1	1.88E-42	6E-100	-0.001774523	0.054583814	0.063297172
Spidr	1	2.46E-02	5E-29	-0.003933663	0.044196181	0.063289394
Uros	1	1.00E+00	2E-37	0.010032736	0.004182734	0.063288304
Fbxo7	1	5.26E-21	1E-61	0.00254337	0.055644445	0.063247063
Recql	1	2.26E-42	1E-138	0.001342118	0.04261217	0.063183842
Sestd1	1	6.76E-76	4E-116	-0.003442238	0.074051531	0.063168877
Tagap1	1.04976E-08	3.80E-20	4E-80	0.01776582	0.043702695	0.063069791
Gdi2	1	6.15E-08	1E-23	0.01164596	0.067909616	0.063011424
Phb2	0.054966313	8.47E-04	7E-59	0.01400674	0.029036255	0.062991694
Scamp5	1	6.65E-30	3E-100	-0.000284189	0.046107204	0.062976123
Fyb	1	0.00E+00	5E-82	-0.004259017	0.265836399	0.062924509
Fnip2	1.32861E-13	4.17E-136	3E-13	-0.045965947	0.303345416	0.06288449
Blzf1	1	2.09E-05	3E-68	0.010491484	0.027441404	0.062820299
Abcb1a	0.004623936	1.58E-94	7E-177	0.004951052	0.048978236	0.062813829
D330041H(	1	2.84E-29	7E-90	-0.002924722	0.046495109	0.062803888
Mast1	1	5.16E-182	2E-153	-0.00081742	0.092374882	0.062777121
Dusp4	1	4.37E-225	4E-214	-0.001300112	0.078387306	0.062758352
Taf1b	4.30479E-10	8.91E-28	5E-73	-0.01774501	0.056097733	0.062757187
Serinc2	1	1.28E-108	2E-127	0.006171566	0.0733549	0.062747531
Gm14325	0.001360324	1.29E-06	5E-84	-0.010996143	0.025387908	0.062717715
Ubap1	0.002709149	1.00E+00	1E-18	-0.025304274	0.03991415	0.062665712
Ctse	4.7984E-122	3.66E-121	2E-98	0.065707489	0.103646785	0.062550414
Tbc1d2	1	1.25E-93	7E-186	0.003758277	0.049725143	0.062522065
Bod1l	5.55707E-05	1.00E+00	4E-20	0.030984613	-0.020439362	0.062515508
Atp5o	1	3.09E-16	2E-77	-0.00115125	0.039702508	0.062512289
Tc2n	0.002073294	3.57E-29	1E-60	-0.011851997	0.061870309	0.062493356
Stk19	1	2.82E-05	2E-43	0.002893765	0.039664841	0.062486905
Mri1	4.79902E-07	3.37E-02	1E-43	0.023847568	0.030654717	0.062482808
Urb1	1	8.15E-17	3E-93	0.007442562	0.036231538	0.062465451
Tatdn1	1	5.78E-03	8E-43	5.75522E-05	0.032426143	0.062403516
Pop5	1	3.42E-26	5E-150	0.004057378	0.03069455	0.062396019
Lysmd2	4.326E-05	1.34E-170	1E-245	0.004523823	0.052875196	0.062385955
Sec23b	1.02445E-13	1.00E+00	6E-31	0.037113832	-0.006527673	0.062324511
Vps52	0.010268291	4.32E-07	7E-73	0.013036565	0.031926786	0.062300866
Aaas	1	1.07E-53	2E-115	0.001599488	0.054895874	0.06225543
Mrpl3	1	1.71E-02	1E-45	0.011172981	0.030800193	0.062239182
Ap1s3	1	1.29E-116	1E-176	0.000527329	0.060684884	0.062060607

Med8	1	2.71E-01	1E-62	0.000526348	0.02115066	0.062044175
Cnot11	1	2.02E-56	2E-123	0.000151943	0.053009723	0.062029289
Rnf219	1	4.45E-32	1E-96	0.001189764	0.048753472	0.062013894
Cdk18	0.000554625	1.00E+00	7E-45	-0.015726317	0.010402335	0.061980022
Taldo1	1	1.84E-72	2E-80	0.002665267	0.0891764	0.061962076
Ndufa6	1	3.52E-05	1E-60	0.004444095	0.031908885	0.061957789
Spata7	0.045356361	9.41E-18	2E-76	-0.010882328	0.042424949	0.061916066
Atad5	1.27278E-32	6.59E-28	6E-57	-0.031040766	0.061880723	0.061903271
Uqcc1	1	1.00E+00	7E-20	-0.015248196	0.023732984	0.061900768
Lilr4b	1	0.00E+00	1E-198	0.000946591	0.281145534	0.061884126
Eif3d	1	1.58E-08	7E-88	0.003204443	0.02908522	0.061873067
Zfp638	1	1.36E-22	6E-14	0.013871404	0.111575054	0.061839984
Setd6	1	1.99E-32	2E-134	0.004959267	0.03640481	0.061740683
Aggf1	1	1.00E+00	1E-32	0.004193735	0.011211457	0.061740029
Eef1d	1	1.74E-16	1E-62	0.006289455	0.045977825	0.061691378
Gm13067	1	4.68E-202	6E-234	0.001051504	0.063845225	0.06166425
Tmem181a	1	1.22E-67	1E-99	-0.000671458	0.073927222	0.061620654
Bola3	1	6.13E-26	5E-70	0.004915794	0.052877083	0.061616929
Aup1	1.85188E-05	2.26E-19	1E-78	0.01451393	0.04511417	0.061594014
Hmg20b	0.004925359	1.45E-14	5E-63	0.014134927	0.045390273	0.061588986
Stambpl1	1	2.04E-200	3E-241	0.000363991	0.062290504	0.061559565
Tmem186	1	1.37E-38	3E-92	-0.00023937	0.052655115	0.06155379
Rhot2	1	8.09E-13	3E-67	0.001777874	0.036884932	0.061513786
Pyroxd1	1	2.72E-02	7E-62	-0.006720029	0.02477129	0.061499665
Uckl1	1	1.96E-05	6E-61	0.00413831	0.03216227	0.061470403
Zfp956	1	2.84E-38	2E-86	-0.000487017	0.053975801	0.061456647
Zfp169	3.88117E-06	1.00E+00	2E-53	-0.016237007	-0.000154978	0.061429028
Klhl25	2.0842E-13	1.00E+00	1E-83	0.019780951	0.012652528	0.061409058
Tnfrsf10b	1	3.05E-98	5E-246	-0.000611739	0.032980123	0.061392021
2310061I0	1	7.11E-33	1E-94	0.007470973	0.047552947	0.061357007
Flvcr1	1	3.96E-65	2E-134	0.001400121	0.054687571	0.06132204
Mfsd8	1	4.69E-02	3E-61	0.010300406	0.025978019	0.061292566
Plekhg3	3.30227E-07	1.00E+00	3E-13	0.038372704	-0.004078802	0.061268344
Polr3a	0.283091823	3.02E-01	2E-36	0.016556068	0.029864389	0.061258111
Gm42477	1	1.76E-43	1E-151	0.003415672	0.037688991	0.06125766
Unkl	1	8.19E-03	5E-38	-0.008039435	0.033165783	0.061251479
Psmd3	6.83431E-10	1.00E+00	4E-51	0.022700698	0.000232565	0.061192047
Marveld2	1	8.71E-15	1E-41	0.004552559	0.053645819	0.061155795
0610009E0	1	6.96E-14	2E-86	0.006967447	0.033332262	0.061122854
Gps1	8.54945E-05	3.91E-53	2E-144	0.009068315	0.045496368	0.061115692
Dnase1l3	1	1.93E-55	1E-156	0.001286087	0.042283326	0.061111359
Usp8	1	1.00E+00	4E-24	-0.000687987	0.009132531	0.061105366
Lilrb4a	1	0.00E+00	4E-171	0.001387403	0.252498124	0.061100008
Gstt3	1.2249E-05	3.59E-12	1E-104	0.012060518	0.027718077	0.061091481
Smad3	7.90243E-05	1.25E-10	0.0004	0.041043831	0.10761436	0.061090234
Dynll1	1	9.13E-75	8E-191	0.001913332	0.041443317	0.061071397
Mcm3ap	0.016012016	2.30E-04	3E-46	0.016119429	0.032298249	0.061004704

Twnk	1	2.41E-14	2E-77	0.007586997	0.035890851	0.060982169
Ogdh	1	1.19E-07	5E-10	0.008311258	0.075923576	0.060940302
Zfp112	1	1.52E-18	1E-89	-0.000852484	0.03591339	0.060926869
Slc39a7	7.77475E-09	4.36E-02	3E-60	0.021150658	0.025611247	0.060919722
Tcp1	0.008757378	4.27E-03	3E-32	0.020248673	0.037464751	0.060913903
Fzd6	1.6813E-16	1.92E-26	1E-73	-0.020232875	0.050091848	0.06089053
2210408F2	0.004867582	8.70E-08	7E-07	-0.035628069	0.100369191	0.060868684
Supv3l1	1	5.84E-25	7E-69	0.005103598	0.051047809	0.060860816
Sf1	6.97617E-07	1.00E+00	2E-24	0.031282639	0.030230674	0.060858105
Cpsf3	1	2.26E-04	5E-39	0.002773678	0.037074065	0.060831521
Cycs	1	3.18E-25	9E-109	-0.001674884	0.040311729	0.060802977
Rapgef1	1	3.98E-23	7E-20	-0.00324972	0.09602692	0.060781654
Tmf1	7.49907E-06	1.00E+00	8E-38	0.023181274	0.017988826	0.0607562
Fnbp1	1.01866E-42	9.06E-167	6E-14	-0.074108895	0.286867808	0.060746921
Gmip	1	2.12E-88	2E-106	0.007275719	0.086754899	0.060745238
Mtdh	1.80287E-30	2.28E-14	6E-15	0.068697445	0.086125032	0.060735942
Zfand5	1	1.00E+00	2E-17	0.000415534	0.036504592	0.060723684
Cep162	1	2.12E-06	1E-52	-0.002560432	0.034245223	0.060668809
Entr1	1	5.25E-19	1E-54	0.010983488	0.052279418	0.060646085
Abcc5	1	1.62E-200	4E-142	0.003009343	0.123082186	0.060627051
Kdm5c	1	1.00E+00	4E-20	0.012827275	0.012427973	0.060619905
Ly6d	1	2.37E-197	2E-260	-3.56951E-05	0.048583295	0.060619829
Rims2	7.77189E-27	1.00E+00	5E-52	-0.024121967	-0.009479368	0.06060292
Trib3	0.112901793	1.00E-84	9E-125	0.006970152	0.060557741	0.060597373
Tpp1	6.13415E-05	2.26E-07	5E-56	0.01630364	0.038055324	0.060492918
Gpr157	0.73612758	4.29E-175	2E-183	-0.003676046	0.071648804	0.060438827
Lin9	1	1.76E-36	2E-75	-0.007131408	0.055310224	0.060431816
Mgst3	1	4.39E-170	2E-210	0.002382062	0.060183512	0.060389129
Gm47071	1	2.31E-24	6E-87	-0.002076475	0.037053692	0.060376405
Nbeal1	1	3.37E-09	2E-11	-0.00536941	0.08081791	0.060369498
Atad2b	1	2.35E-07	2E-13	0.019024302	0.071851138	0.060346278
Diaph3	1	9.51E-48	8E-123	-0.00154978	0.041477965	0.060334673
Gm36231	1.34718E-39	1.56E-11	1E-142	0.023710156	0.020187051	0.060327444
Gm49958	1	1.64E-198	6E-266	0.000517453	0.04909155	0.060303569
Ubp1	1	1.00E+00	2E-25	0.011563084	0.026841645	0.06029989
Setx	0.000526755	1.00E+00	6E-20	-0.023114398	0.012507547	0.060218194
Amn	1	7.99E-223	2E-268	-0.00030999	0.052227474	0.060197459
Vav2	1	1.00E+00	8E-18	-0.014159852	0.02062718	0.060179123
Bzw2	1	2.14E-12	2E-58	-0.002387279	0.040604692	0.060175667
Tbc1d24	1	4.70E-09	3E-29	0.000512524	0.055169867	0.060170435
Washc5	0.001502479	1.85E-26	2E-52	-0.014268033	0.066673831	0.060055151
Nup35	1	9.71E-47	7E-77	0.006451344	0.063094916	0.060031376
Tmem106a	2.62272E-31	1.00E+00	7E-26	-0.045279657	0.036127222	0.060016567
Polr2b	1	3.36E-05	8E-47	-0.003210145	0.034140822	0.059983545
Unc45a	1	4.34E-15	5E-68	0.00243237	0.038534692	0.059970913
Apeh	1	1.75E-09	4E-70	0.0036912	0.031783454	0.0599226
Brip1os	1.21336E-07	2.62E-26	1E-14	-0.031145655	0.123967973	0.059918768

44446	1.25805E-07	2.74E-02	2E-33	0.026611232	0.041069822	0.059912002
Ogfod1	1	1.00E+00	8E-44	0.006066197	0.012126451	0.059822622
Ints14	1	1.06E-06	8E-61	0.005638385	0.031613278	0.059798555
Egf	1	3.27E-29	8E-95	-0.005182285	0.043808656	0.059745234
Micos13	1	3.41E-24	2E-120	0.00059537	0.034978616	0.059725568
Zfp62	1	3.76E-10	4E-31	-0.000328656	0.05284377	0.05970564
Eri2	1	1.10E-10	3E-72	0.009289626	0.032644818	0.059638876
Rbbp7	0.001939062	2.62E-01	8E-37	-0.01525423	0.030771197	0.059606342
Rps4x	1	3.61E-73	4E-106	0.000991611	0.068674819	0.059574397
Fam214b	1	7.17E-02	2E-66	-0.002081749	0.021455888	0.059568522
Ccdc84	5.88366E-07	1.00E+00	3E-44	0.022495671	0.010433545	0.05955422
Plod1	1	5.41E-04	6E-49	-0.010018503	0.034559934	0.05952673
Mto1	0.159679094	2.31E-03	3E-45	0.015075693	0.029613595	0.05951236
Lrrc45	1	1.00E+00	3E-63	-0.002501858	0.015927934	0.059437827
Psmb3	1	2.29E-09	6E-56	0.009692969	0.039120344	0.05941125
Galnt7	1	4.78E-57	2E-71	-0.00721439	0.087888623	0.059409805
Ulk1	1	1.00E+00	5E-21	-0.017380109	-0.005023315	0.059406496
Crybg3	1	1.00E+00	2E-11	-0.012094225	0.015000693	0.059405725
Atpif1	1	5.04E-53	6E-137	0.00093062	0.045976294	0.059359417
Agps	1	2.65E-23	2E-32	0.00435807	0.07493048	0.059354063
Afp	1	1.34E-89	4E-212	0.000371766	0.036080653	0.059343992
Gm14410	0.383616794	8.86E-08	3E-60	-0.009577897	0.035936014	0.059342815
Csnk1e	3.95854E-05	1.64E-65	1E-104	-0.009432416	0.063428466	0.059328846
Ap1m1	1	2.19E-13	1E-69	0.009968302	0.039555232	0.059286281
Smc2	1	2.37E-33	7E-134	-0.000732564	0.036407195	0.059266747
Gm12602	9.63372E-06	1.79E-60	1E-197	-0.004662964	0.031357082	0.059198235
Tmem161a	1	1.00E+00	8E-63	0.0007733	0.017910184	0.059165234
Clk2	1	5.06E-04	5E-33	0.010212187	0.039292152	0.059162616
Esco2	1	1.16E-129	2E-202	-0.001388222	0.048168086	0.059147138
Fxr2	1	1.00E+00	3E-28	0.013377986	0.0164995	0.059128041
Ocrl	0.008300483	2.35E-14	1E-71	-0.010718705	0.040571212	0.059047608
Snhg6	0.06847581	1.41E-101	1E-167	0.005810429	0.053131394	0.059025953
Nckipsd	1	1.52E-99	4E-163	0.002461121	0.055045849	0.059023927
Tbx3	1	8.78E-04	3E-32	0.011766476	0.039272096	0.059011047
Gm10076	1	5.96E-124	3E-180	0.000160565	0.057318488	0.058978469
Phospho2	1	5.71E-11	5E-77	0.00355043	0.032337851	0.058944585
Dgkq	0.374267381	1.00E+00	4E-30	0.016578149	-0.010678317	0.058943167
Tmem208	1	3.40E-04	7E-65	0.00739981	0.027392085	0.058909458
L3mbtl3	1.59511E-16	2.09E-04	1E-19	-0.040352311	0.055797131	0.05884783
Man1a2	2.12373E-14	1.00E+00	1E-19	0.045392266	0.034333737	0.058847613
Met	7.08792E-15	4.77E-06	1E-11	-0.054048811	-0.054459288	0.058824535
Cox6b1	1	2.60E-08	2E-52	-0.003554649	0.039299391	0.058811245
Rcan1	1	1.00E+00	1E-38	0.004466121	0.029459031	0.058803984
Eogt	1	1.22E-14	2E-68	0.00545191	0.038507695	0.058794932
Tmcc3	1	8.10E-103	2E-20	-0.002614975	0.217288111	0.058785938
Gga3	1	7.19E-25	1E-61	0.00310931	0.052132774	0.058737045
Tmem242	1	3.42E-22	2E-80	0.000554204	0.04234254	0.058735489

Lsr	1.20021E-37	6.89E-08	3E-24	-0.054789828	0.054758949	0.058701621
Cdk10	1	1.00E+00	1E-48	0.002033713	0.020666129	0.058668576
Tm9sf1	0.264366131	1.70E-15	2E-57	0.01168161	0.045588151	0.058653425
Nf2	1	5.07E-01	2E-24	0.012467371	0.038004193	0.058599289
Smarce1	1	5.43E-03	3E-48	0.006538388	0.028301085	0.058585534
Abcc10	1	1.00E+00	1E-45	0.010532933	0.006186706	0.05857898
Chrna9	1	1.61E-209	3E-232	-0.000328715	0.060584199	0.05854962
Mmp12	1	0.00E+00	7E-190	-8.92378E-06	0.198422541	0.058534874
Rngtt	1	1.00E+00	5E-17	-0.008488161	0.022655246	0.058502069
Gm34934	0.892052607	5.49E-74	9E-109	-0.006287084	0.061201524	0.058482115
M6pr	1	1.94E-09	4E-68	0.004311749	0.034475823	0.058418854
Esrp2	0.00172492	1.00E+00	9E-40	0.018129745	0.007128237	0.058368191
Ctu2	2.43726E-07	2.00E-31	4E-117	0.011890356	0.037662436	0.058366505
Ssh1	2.31897E-09	7.55E-18	4E-49	-0.019170156	0.051661974	0.058365133
Max	1	1.49E-01	2E-36	0.011557039	0.032198255	0.058357168
Vps13a	0.002288554	3.13E-19	4E-13	-0.026820324	0.098826886	0.058307137
Lztr1	0.006046852	1.00E+00	1E-56	0.014425406	0.017714806	0.05828978
Mrto4	1	2.00E-36	1E-137	0.003485168	0.035219807	0.058242847
Ankle2	1	2.08E-05	5E-39	0.010012724	0.037414002	0.058216783
Samm50	0.911419542	3.33E-16	4E-56	0.011495429	0.044797362	0.058065374
Polr3c	1	1.00E+00	6E-44	-0.002280525	0.020302007	0.058048809
Gm42836	1	5.28E-153	5E-150	0.003045429	0.073803168	0.058033671
Nsmaf	1	1.00E+00	7E-26	0.00950036	0.025714957	0.058027225
Gtf3c1	1	2.89E-08	3E-19	0.000761947	-0.050577939	0.057953476
Mtap	1	2.96E-53	1E-75	-0.002022616	0.065437526	0.057950683
Atp8b5	1	7.12E-67	7E-193	-0.000324069	0.034767063	0.057937427
Slc25a5	0.000483692	6.87E-23	2E-90	0.011578288	0.043230514	0.05792025
Unc5b	1	8.94E-19	3E-121	0.001994159	0.024077022	0.057890281
Exosc9	1	8.49E-42	5E-127	0.001972792	0.038895636	0.057868268
Tgs1	1	1.00E+00	1E-25	0.016532207	-0.009779397	0.057848902
Zmym1	1	5.33E-22	5E-95	-0.001469958	0.036464725	0.0578215
Tbl3	1	3.78E-42	2E-104	0.004410549	0.046742751	0.057781907
Man2c1	0.030064963	8.14E-06	9E-68	0.011229068	0.02641154	0.057768924
Pex3	0.109283733	1.00E+00	5E-31	-0.016023639	-0.015251078	0.057741653
B3gntl1	0.001963407	3.53E-04	1E-47	-0.014595227	0.034362863	0.057734306
Ipp	1	5.88E-21	2E-68	0.001653378	0.044336059	0.057702818
Bcor	2.66493E-07	1.00E+00	5E-35	0.020021397	0.011562022	0.057697296
Cog2	1	2.00E-11	2E-47	0.006659535	0.043769391	0.057693211
Wdfy1	1	1.00E+00	2E-24	-0.004602779	0.016812989	0.057666183
C2cd2l	1	1.00E+00	3E-58	0.009287785	0.018082671	0.057640084
Uba5	3.03471E-26	1.00E+00	7E-31	0.044972999	-0.018393745	0.057632221
Elob	1	6.51E-23	2E-91	0.003559932	0.041860284	0.05763193
Uhrf1bp1	1	1.95E-14	1E-48	0.001383877	0.046061343	0.057597052
Yipf3	1	3.70E-17	5E-92	0.004515005	0.034603918	0.057587428
Fntb	1	3.47E-30	4E-79	0.00736278	0.047833053	0.057582644
Rpl39	1	9.96E-165	1E-123	-0.001004269	0.095550697	0.057578003
Rbm19	1	4.18E-09	2E-64	0.00548409	0.031316288	0.05753836

4930581F2	0.242774662	6.23E-30	9E-14	-0.020044513	0.127943031	0.057536986
Casp6	1	8.83E-22	6E-81	0.004031692	0.039826314	0.057518484
Abhd16a	0.325970847	1.09E-11	6E-58	0.011633956	0.039477234	0.057507677
Srsf1	1	1.00E+00	3E-30	0.012343856	0.02328175	0.057470108
Pigb	1	6.99E-18	4E-56	-0.003892542	0.047522648	0.05745543
Eif3h	1	1.00E+00	8E-26	0.016569136	0.015751269	0.057398621
Fbxo4	1	7.16E-17	6E-61	-0.005497122	0.043390988	0.057313431
Timm44	1	1.00E+00	1E-29	0.010279158	0.00558577	0.057244011
Zfp777	1	1.13E-04	1E-46	-0.003112462	0.029993529	0.057242105
Dusp14	1	1.11E-84	1E-217	0.00060656	0.031323352	0.057194371
Acer3	8.03114E-18	3.50E-25	5E-20	0.044969583	0.112989541	0.057163969
Rpl18	1	3.27E-45	6E-61	-0.002633714	0.074269444	0.057150198
Plcg2	1	1.02E-263	1E-205	-0.001156649	0.094535073	0.057078521
Atp5c1	1	1.00E+00	2E-19	0.002287172	0.017836093	0.057059877
Bud23	0.002335238	2.65E-15	2E-77	0.011685854	0.034882056	0.056976473
Elovl7	1	5.37E-185	2E-217	0.000263711	0.056145775	0.056945188
Arl14ep	1	3.02E-19	1E-70	-0.001790665	0.040706803	0.056932175
Ybx3	1	3.60E-31	2E-85	0.003155685	0.045571915	0.056926095
Slc25a19	1	4.90E-15	5E-74	0.007404094	0.036029771	0.05688309
1700109H0	1	1.31E-02	2E-43	0.001223158	0.02685207	0.056857693
Dbf4	1	2.04E-50	4E-114	-0.000305812	0.046263239	0.05683236
Nek1	0.000101621	1.00E+00	6E-15	-0.028553808	-0.008162546	0.056782917
Zfp800	1	1.00E+00	2E-39	-0.006900771	0.014945371	0.056759028
Rph3al	0.000840155	2.01E-31	4E-49	-0.01504788	0.06588964	0.056716741
Atad2	1	6.84E-22	3E-51	-0.000864362	0.053249469	0.056713958
Txnl4a	1	3.88E-27	1E-101	-0.000846008	0.037444135	0.056707782
Slc19a2	6.99453E-28	2.78E-06	3E-18	0.05678846	-0.044016793	0.056703571
Psma5	1	1.00E+00	2E-33	0.006194785	0.019504803	0.05667898
Orc2	1	1.53E-01	4E-45	0.006132141	0.02440813	0.0566716
Clpp	6.34008E-09	1.00E+00	7E-32	0.026994083	0.019984496	0.056661138
Fis1	1	2.12E-18	2E-41	0.006780232	0.056783362	0.05664424
Ercc8	1	1.53E-07	4E-65	0.008204391	0.02864078	0.056621134
Gm12153	1	0.00E+00	1E-158	-0.000289171	0.120486276	0.056601956
Tpi1	1	4.68E-12	6E-43	0.006808913	0.046543077	0.056593798
Arfgap3	5.41069E-05	1.00E+00	5E-56	0.016087596	0.009399054	0.056576494
Snx33	7.81197E-07	1.00E+00	3E-57	-0.015725983	-0.001705053	0.056570709
Dnajc2	0.000753054	7.13E-18	1E-60	0.014448173	0.043643126	0.056547727
Dnmt1	1	3.70E-27	3E-82	-0.00204112	0.046055695	0.056547246
Lnx1	8.16214E-05	3.46E-118	9E-110	-0.00693983	0.089426167	0.056537887
6030442K2	1	1.77E-42	7E-94	0.000260229	0.049302318	0.056534331
Disp1	2.71664E-07	1.46E-45	7E-86	-0.011916158	0.058003533	0.056524472
Akap8	4.00255E-14	1.00E+00	9E-22	0.03990563	-0.005866037	0.056521833
Fdps	3.5395E-121	2.16E-51	2E-24	0.109245742	-0.077691238	0.05651162
Wdr74	0.014564955	1.04E-09	4E-83	0.009942429	0.027193982	0.056510946
Smarca5	1	3.98E-07	9E-23	-0.001533128	0.054816957	0.05650036
1700021F0	1	1.79E-10	5E-49	-0.000286708	0.039303244	0.056491398
Dhcr7	2.49298E-12	1.00E+00	8E-56	0.023925698	0.014692576	0.056482017

Ice1	0.037284494	1.00E+00	1E-29	0.018280071	0.025871026	0.056479592
Smndc1	1	4.33E-11	5E-46	0.003347123	0.041919887	0.056473406
Cul9	5.37162E-10	2.28E-06	3E-39	-0.02079451	0.03883731	0.056346485
Galk1	1	1.28E-05	3E-90	-0.004626784	0.021885064	0.056334265
Clptm1	1	2.74E-08	3E-39	0.008588006	0.043174451	0.056325449
Cyb5r4	1	1.00E+00	9E-39	-0.000986276	0.013843457	0.056308795
Tyk2	1	2.82E-03	4E-40	-0.007661461	0.032013665	0.056290646
Lims1	1	2.07E-18	3E-26	-0.009406891	0.083210203	0.056283307
Rpl23a	1	2.33E-79	4E-109	-0.000837404	0.06414975	0.056280371
Akr1e1	1	2.57E-01	3E-47	0.00297092	0.021226443	0.056259202
A930001C0	1	2.37E-21	1E-67	-0.005597257	0.043140881	0.056203572
Cox20	1	1.30E-16	4E-66	0.003000765	0.039835937	0.056161024
Vps4b	1	1.00E+00	1E-25	0.004111182	0.029622987	0.056128958
Chaf1a	1	9.58E-94	2E-156	-0.0030812	0.04994151	0.056082466
Kdm4a	1	1.49E-03	4E-29	0.002295416	0.040075439	0.056072103
Zmym5	1	1.00E+00	1E-13	0.00016988	0.039736625	0.05606945
Smdt1	1	2.60E-25	2E-94	0.003084731	0.03746978	0.056054489
Ndufa2	1	4.46E-06	6E-72	-0.006824241	0.027780431	0.056044553
Cnnm2	1	7.43E-07	2E-22	0.01219709	0.056565324	0.056042555
Aqr	1	1.20E-02	2E-26	0.006959362	0.035896932	0.056029713
Ap2m1	1	4.53E-04	2E-69	-0.004261819	0.023270965	0.056007083
Use1	1	2.18E-03	3E-78	0.004232429	0.020899856	0.05596291
Kantr	2.29623E-07	1.24E-20	5E-64	-0.013656619	0.046913416	0.055933072
Tkt	1	7.00E-52	9E-43	-0.003617216	0.088062098	0.055917497
mt-Cytb	1	8.35E-17	6E-103	-0.002981617	0.031164778	0.055889377
Nup85	1	8.69E-15	6E-69	0.008840585	0.03422739	0.055882153
Pparg	2.67496E-79	1.63E-36	1E-43	-0.081186804	0.085857209	0.055843751
Rbl2	1	1.00E+00	2E-20	0.005772468	0.013433945	0.055758699
Cenpt	1	2.98E-43	2E-181	0.000794844	0.0275671	0.055744274
Csnk1g1	1	1.00E+00	6E-10	-0.016092703	0.022245562	0.055741714
Rpap1	1	1.00E+00	9E-57	0.001179425	0.017351389	0.055735049
Dcun1d5	0.463657466	1.00E+00	4E-33	0.014634428	0.022008321	0.055676282
Nip7	1	5.17E-32	3E-111	0.005331892	0.037557851	0.055675389
Tmx3	1	1.00E+00	2E-33	-0.003892288	0.027517972	0.055668071
Trp53cor1	1	1.82E-90	4E-87	-0.000205716	0.077694143	0.055635631
Zcchc9	0.048045024	1.00E+00	2E-48	0.013657041	0.016416199	0.055632242
Zfand2a	1	2.19E-20	6E-96	-0.001675869	0.03574641	0.05561538
Gpr19	1	3.19E-07	5E-55	-0.008256326	0.031476957	0.055611783
Zfp560	1	9.62E-06	4E-57	0.004520576	0.029072099	0.055601057
Polr2m	1	1.70E-07	4E-48	-0.003665062	0.033720144	0.055600643
Noc2l	0.014997432	6.06E-16	2E-37	0.016387103	0.058620894	0.055536415
Ap3m1	5.09186E-28	1.00E+00	6E-14	0.069188226	-0.033790918	0.055502981
Chka	7.2971E-39	6.56E-36	0.0003	-0.109626271	-0.147676907	0.055502075
Parn	1	1.99E-06	1E-16	0.006075933	0.057326699	0.055500315
Pcsk7	1	1.00E+00	5E-20	-0.002958578	-0.019868694	0.055497932
Cdkl2	1	2.69E-41	2E-110	-0.002043404	0.040939824	0.055492656
Plekhh1	1	1.44E-172	4E-239	-0.000216048	0.044041568	0.055476596

St6galnac2	1	7.37E-224	7E-237	-3.91103E-05	0.05576552	0.055461245
Serf2	0.015213246	1.77E-08	2E-40	0.015295484	0.044122426	0.055433498
Scamp3	2.27717E-10	2.88E-06	8E-65	0.019329593	0.027319737	0.055430094
Tmem33	1	7.75E-01	3E-48	0.007061985	0.021422629	0.055382315
Limd1	1	1.00E+00	8E-26	-0.009423079	0.019984389	0.055371538
Psap	0.002970782	8.20E-136	3E-17	0.020433397	0.276579398	0.055359343
Ecsit	1	3.15E-13	4E-48	0.003954898	0.040705482	0.05533331
Sbno1	3.21068E-14	1.00E+00	1E-13	0.046190625	0.040626298	0.055287866
Mvp	1	2.29E-41	4E-31	-0.006386396	0.095440693	0.055236001
Gm14963	1	4.05E-84	6E-121	0.00159541	0.058222158	0.055208593
Gde1	1	6.06E-22	3E-28	0.000626059	0.073993	0.055170093
Slc3a2	1	1.01E-61	5E-95	0.005844116	0.065976306	0.055136759
Susd6	1.1971E-182	1.00E+00	4E-06	0.205384859	0.053223792	0.05510636
Dctn1	1	6.85E-02	6E-37	-0.00125086	0.027660294	0.055087315
Rragc	1	8.67E-38	1E-39	-0.006173078	0.082270789	0.055071172
Pex11b	1	3.53E-13	6E-69	0.003001623	0.032320142	0.055069048
Endov	1	1.02E-03	4E-81	0.004476156	0.020150095	0.055059852
Gm49539	1	9.65E-30	2E-96	-0.0051734	0.038657547	0.055053264
Arfrp1	1	4.31E-19	5E-74	0.003479895	0.040303541	0.055029243
Hrk	1	6.62E-240	7E-218	-0.000554397	0.065209483	0.055018001
Tbc1d23	1	1.94E-04	1E-16	-0.007180255	0.078855964	0.054944578
Hprt	1	2.77E-11	4E-47	0.000373185	0.043681723	0.054937593
Mrpl52	0.004142704	1.11E-34	2E-63	0.012454555	0.058310826	0.054935078
Ankzf1	0.16977541	1.00E+00	7E-36	0.015128967	0.014203471	0.054906019
Stxbp2	1	3.54E-02	3E-41	0.009013412	0.03152897	0.054898713
Uqcrh	1	1.00E+00	2E-36	-0.003996915	0.018103816	0.054871502
Hsf3	1	2.24E-180	2E-157	-0.000315989	0.077898662	0.054850611
Id1	9.30822E-13	3.54E-123	1E-180	0.008388192	0.049761864	0.054761812
Idh3a	1	7.80E-09	8E-55	-9.90464E-05	0.030227067	0.054724432
Myc	1	4.99E-137	2E-166	-0.000959634	0.05997324	0.054717271
Ezh1	1	1.91E-15	1E-38	-0.010700511	0.053395202	0.05468474
Bbs5	1	3.61E-26	6E-107	-0.001829109	0.032829224	0.054683881
Siah1a	1	3.52E-20	2E-44	0.008891053	0.054493186	0.054655758
Higd1a	0.000428692	4.57E-02	2E-41	-0.015121684	0.025426958	0.054650207
Dbt	1	1.00E+00	3E-41	0.007798773	0.009710927	0.054649061
Gpatch4	1	1.27E-30	2E-80	0.001439977	0.046110136	0.054630817
Atp5g3	1	2.94E-10	1E-39	-0.006408024	0.04776662	0.054620097
Spats2	1	1.62E-05	2E-45	-0.003144601	0.035917731	0.054619393
Pphln1	1	1.00E+00	2E-24	0.002553921	0.024826075	0.054609679
Pmvk	4.1364E-14	1.00E+00	2E-69	0.023686837	0.010949924	0.05458728
Stoml2	1	1.89E-08	9E-64	0.007355743	0.028901942	0.054559233
Rbfa	1	4.99E-07	2E-86	0.002108496	0.022831359	0.05454373
Mpzl1	1.15948E-11	1.00E+00	6E-38	0.024038336	0.002350529	0.054532495
Prpf38b	1	1.00E+00	3E-12	0.021295368	0.019654165	0.05452176
Ehhadh	1.36748E-07	1.00E+00	1	-0.029853019	0.029558875	0.054499771
Rpgrip1l	2.14973E-05	1.28E-03	2E-58	-0.013361056	0.023002053	0.054490712
Sf3a1	1	6.39E-09	6E-57	0.005584275	0.032915681	0.05448416

Dcaf10	1	1.00E+00	2E-14	-0.009508593	0.013183459	0.054462861
Adgb	1	7.46E-234	4E-138	-0.001467707	0.136039644	0.054435137
Rpp14	1	2.34E-12	3E-56	0.009656906	0.037749034	0.05442544
Id3	1	2.66E-28	2E-65	-0.001563841	0.053831182	0.054340295
Gm10501	1	1.15E-119	6E-82	0.002141301	0.09479059	0.054319635
Fam91a1	1	1.00E+00	4E-24	-0.009277717	0.001207522	0.054317938
Nipal3	1	1.54E-58	3E-81	-0.003531875	0.062165656	0.054299888
Gm15738	1	1.20E-111	1E-33	0.007096121	0.160324163	0.054292953
Sugt1	1	5.02E-04	2E-34	0.008436312	0.034206407	0.05427453
Tdo2	0	1.46E-70	0.0002	0.503767127	-0.278507963	0.054264626
Rnf139	1	7.67E-30	5E-21	-0.012661294	0.089700286	0.054253073
Paf1	1	4.26E-11	4E-55	0.007933434	0.037688189	0.054250621
Tmem71	1	2.25E-110	1E-191	-0.001492363	0.042265253	0.054223212
Fam135a	3.36273E-14	1.00E+00	4E-09	-0.050149152	-0.026957197	0.054181072
Zfp958	1	3.72E-18	5E-54	0.000963172	0.045221149	0.054152129
Mrpl47	1	1.55E-15	1E-75	0.003830435	0.032044367	0.054140791
Anks1	1	1.99E-30	1E-14	-0.005922408	0.111533375	0.054138822
Zfp820	1	5.52E-07	3E-127	-0.003993184	0.015974429	0.054119812
Pomp	1	1.07E-05	2E-45	-0.006690904	0.034734752	0.054097144
Fermt1	3.17804E-12	1.09E-36	3E-194	0.0067867	0.018793547	0.05409632
Fbrs	0.51861243	1.00E+00	3E-36	0.014679985	0.028502316	0.054058712
Cdc25a	1	3.69E-40	1E-117	0.002111733	0.038609311	0.054051737
Tut1	1	2.18E-03	2E-51	0.004927047	0.025083238	0.054012907
Kif17	1	6.01E-128	2E-174	-0.000676083	0.050355501	0.053999786
Ppp6r1	1	1.00E+00	6E-33	-0.000249426	0.028412743	0.053999563
Dnttip1	1	8.38E-10	1E-39	0.011466463	0.042984544	0.053947093
Pik3c3	1	4.98E-01	3E-25	-0.005250442	0.032298319	0.05392864
Ppil3	1	3.68E-27	9E-68	0.000165966	0.046170298	0.053916178
Trim68	1	1.29E-58	5E-173	-0.001319474	0.032109422	0.053885467
Rogdi	1	1.00E+00	3E-71	0.004837479	0.013298245	0.053847344
Fam83f	3.11976E-08	1.58E-77	1E-134	-0.007380612	0.049783474	0.053834471
Rpp21	1.01432E-08	4.23E-10	2E-78	0.015565801	0.028108992	0.053825874
Zfp317	1	1.07E-02	7E-50	0.005196243	0.024690532	0.053780663
Strn4	1	1.10E-11	2E-39	0.01147213	0.047060118	0.053769508
Pot1a	1	4.85E-11	2E-51	-0.008551977	0.038559291	0.053722093
Lamp1	1	6.29E-15	5E-28	-0.007173456	0.068517972	0.053707933
Eif2b5	1	2.20E-21	3E-76	0.004485363	0.039695877	0.053698051
D430041D(	1	3.46E-254	7E-222	2.61117E-05	0.065131332	0.053629707
Ppp1r12c	1.80495E-13	4.87E-04	1E-46	0.024795384	0.032780017	0.053624264
Eif3l	1	1.10E-25	5E-70	0.008508153	0.047004351	0.05359975
Khsrp	1	8.82E-07	6E-37	0.010712099	0.038361749	0.053563284
Sirt4	1	1.03E-09	5E-81	-0.003162927	0.026348899	0.05354075
Atp5l	1	1.00E+00	8E-27	-0.012506612	0.027291194	0.053532543
1700095A2	1	3.99E-07	1E-92	-0.003086962	0.019911031	0.053523732
Cast	8.4011E-23	1.00E+00	1E-12	-0.048951622	0.028974755	0.05350071
Dhx38	0.003762258	2.40E-01	1E-59	0.013008184	0.01878157	0.05347801
Rpl29	1	6.78E-78	8E-112	0.002641296	0.059625395	0.053459713

Hspg2	1	1.00E+00	5E-85	-0.004003741	0.00934056	0.053401014
Atp9b	1	2.63E-07	2E-09	0.016382981	0.077288242	0.053389559
AI314278	1	1.68E-82	4E-223	-0.000554782	0.02291604	0.05337833
5530601H(	1	2.73E-10	1E-44	-0.0016114	0.039039278	0.053318045
Kpnb1	1	1.65E-21	1E-42	0.004525952	0.053446128	0.053288255
Rpf2	4.66621E-09	9.29E-23	1E-80	0.0149219	0.03790594	0.053286066
Sirpa	1	0.00E+00	4E-142	-0.002431413	0.198129385	0.05326015
Trappc8	1	5.09E-14	2E-11	0.009742888	0.085058953	0.053243939
Mblac2	1	1.00E+00	6E-25	-0.00790137	0.025531164	0.053205059
Hyou1	1.94035E-09	1.00E+00	4E-22	0.03082956	-0.00462884	0.053167916
Snx13	0.016574971	1.88E-27	1E-11	-0.024261328	0.113024098	0.053151116
Fam160a2	1	1.00E+00	2E-30	-0.007809565	0.01785097	0.053021824
Adam2	0.082821869	1.00E+00	1E-52	-0.008837241	0.017951394	0.053008884
Mtx1	1	1.10E-09	1E-73	0.001316944	0.027251676	0.052986289
Ralgds	1	3.33E-210	9E-210	-0.000504293	0.062908968	0.052871787
Gm42997	1	4.36E-132	3E-138	-0.000909878	0.064947038	0.05285274
Wars	1	2.23E-04	3E-70	0.005449673	0.022410701	0.052851923
Pfkfb1	1.0694E-172	1.63E-35	3E-13	0.153585266	-0.105629186	0.052816632
Lztfl1	1	3.28E-10	1E-50	0.007032849	0.037481229	0.052722348
Zfp760	1	1.52E-07	5E-68	-0.006060498	0.02602754	0.05271324
Vps45	0.479214717	1.00E+00	2E-29	-0.013242482	0.006450139	0.052704283
Dlat	2.85888E-53	1.00E+00	1E-15	0.076954641	-0.005477096	0.052682651
Yipf1	1	1.00E+00	8E-23	0.007162994	0.021038525	0.052638007
Ccdc137	1	7.49E-12	2E-64	0.003356041	0.033116012	0.052630566
D930048N1	1	9.80E-08	1E-61	-0.002491455	0.026542386	0.052612038
Pigg	1	6.74E-17	1E-60	-0.002586137	0.04014217	0.05256938
Sra1	1	1.00E+00	3E-53	0.00517241	0.004039002	0.05256731
Bms1	0.461945964	1.00E+00	7E-28	0.0156489	0.016050082	0.052566467
Trib1	1	1.00E+00	4E-26	0.001915378	0.026239669	0.05255472
Slc8a1	1	1.38E-294	3E-62	-0.002199771	0.289368312	0.052553773
Dapk3	1	1.00E+00	9E-65	0.00763834	0.016061901	0.05251294
Cln5	1	5.70E-33	2E-104	-0.002410411	0.038314331	0.05251173
Rab36	0.276214404	1.95E-47	4E-153	-0.004066686	0.03121677	0.05243432
Tbca	1	1.00E+00	2E-17	-0.01008109	0.028589636	0.052429655
Dimt1	1	1.77E-37	2E-89	0.004733402	0.044800903	0.052427707
Mrpl4	0.003715412	4.69E-13	2E-57	0.012457599	0.037543276	0.052398203
Fbxo22	1	1.00E+00	3E-23	0.011721358	0.003109238	0.052397094
Mrps35	1	3.51E-08	9E-60	0.008211223	0.028740594	0.052396263
Il20rb	1	5.78E-29	4E-48	0.002232131	0.058896207	0.052375352
Fbxl12	1	6.89E-02	1E-37	0.001442902	0.028542262	0.052354732
Cwc22	1	2.70E-01	1E-29	0.001397432	0.028477795	0.052308066
Tor1a	1	2.42E-09	5E-53	0.002488803	0.033033128	0.052287122
Snrnp40	1	2.24E-07	2E-38	0.007436607	0.035048875	0.05227555
Txlna	1	1.05E-01	6E-36	0.012982282	0.027484468	0.05226401
Ttc33	1	1.00E+00	2E-31	-0.006389587	0.025277889	0.052248335
Dpysl2	3.42454E-11	3.47E-44	8E-174	0.006892684	0.026449252	0.052229159
Vps39	1	8.43E-04	7E-37	0.001441141	0.034270545	0.05221545

Ostm1	1	4.20E-21	1E-64	-0.002452237	0.041477863	0.052195789
Grk6	1	1.51E-19	2E-77	-0.00140451	0.038786831	0.052142215
Mettl2	0.163014039	6.09E-10	1E-61	0.010724299	0.029382087	0.052098189
Impdh2	0.0408006	4.93E-32	1E-98	0.00768612	0.037410509	0.052079292
Aebp2	0.242104394	1.00E+00	6E-10	-0.017541133	-0.007426538	0.052048745
Slc35a1	1	6.03E-10	2E-63	-0.000247697	0.029402131	0.052012223
Kazn	1	1.54E-165	1E-175	0.000868372	0.058941976	0.052001439
Tstd2	1	3.45E-04	8E-36	0.001518508	0.032301189	0.051927273
Nox1	6.28017E-06	7.80E-50	2E-72	-0.01061825	0.058110334	0.051916608
Eml5	1	1.00E+00	3E-18	0.003006935	0.010511527	0.05189443
Mtmr2	1	1.00E+00	2E-31	0.005313989	0.013211253	0.051892694
Hnrnpab	1	1.09E-17	1E-43	-0.005014251	0.047003671	0.051846984
Serpina6	1	1.21E-16	7E-95	0.003094714	0.027690555	0.051846776
Angel2	1	4.40E-10	2E-40	-0.000237258	0.042257205	0.051834104
Ppwd1	1	1.00E+00	8E-34	-0.000749965	0.01883523	0.051807571
Kctd13	1	5.76E-22	8E-58	-0.004329598	0.043525363	0.051762525
Adh5	1	1.00E+00	3E-28	-0.007341399	0.006114998	0.051693223
Atp5j	1	1.00E+00	2E-46	-0.001865313	0.017281215	0.051684758
Eif2s3x	1	1.37E-14	1E-47	0.008153818	0.042484639	0.051658996
Ttll3	1	7.25E-87	2E-115	0.004149077	0.064549712	0.051648967
Ccdc62	1	1.37E-26	5E-41	-0.007675165	0.060295824	0.05161102
Lcat	1.13208E-05	1.00E+00	3E-20	0.026116425	-0.014720656	0.051571641
Azi2	1	2.59E-03	5E-23	0.007489716	0.043557053	0.051552964
Eef1akmt1	1	1.67E-10	3E-47	-0.000770617	0.035487074	0.051517277
Lysmd3	1	1.00E+00	9E-33	-0.003187752	0.009142755	0.051512042
Fam118b	0.869031544	1.90E-06	3E-38	0.012250089	0.039463281	0.051473393
Scyl1	1	2.21E-08	7E-70	0.002440417	0.026494733	0.051471048
Gm33699	9.74714E-05	9.25E-51	4E-180	0.004926358	0.024914375	0.0514183
Smyd1	1	2.68E-85	1E-118	-0.000406037	0.0539315	0.051385271
Epb41	0.003937334	3.44E-14	0.008	0.029098777	-0.069007458	0.051380916
Anapc4	1	6.86E-18	3E-59	0.006906942	0.040834269	0.05137559
Sgsm3	1	2.67E-04	4E-50	0.00231734	0.025076555	0.051352359
Mki67	1	1.29E-30	2E-136	-0.001041397	0.016621479	0.051328328
Snrpa	1	2.59E-03	8E-43	0.006412472	0.027615047	0.051301363
Celf2	1	2.28E-36	7E-09	-0.002940182	0.158017083	0.051296819
Ate1	1	1.00E+00	6E-37	0.011817499	0.003260847	0.051243979
Pdzd11	1	1.68E-12	6E-100	0.004614784	0.023445218	0.051239723
Morc4	1	1.18E-28	1E-145	0.000715531	0.022097716	0.051228956
Rbis	1	2.80E-52	4E-114	0.002274445	0.043318301	0.051223542
Dgke	1	1.41E-28	2E-105	0.000384487	0.034099895	0.051221478
Myo9b	1	7.63E-50	1E-15	-0.003408527	0.159083091	0.051215328
Cox17	1	2.88E-27	2E-78	0.002413188	0.041085273	0.051185136
Lrig2	1	1.00E+00	7E-14	0.012150582	0.002272889	0.051124012
4833439L1	1	2.51E-01	7E-28	0.009561225	0.027271001	0.051063724
Grb10	1	2.38E-06	9E-147	0.000770943	0.011762301	0.051055314
Ptprm	1	1.50E-89	2E-79	0.005079351	0.108741809	0.051043918
Zdhhc21	0.000130449	3.32E-07	3E-44	-0.014916956	0.032442494	0.05103093

Mettl3	1	1.17E-21	5E-57	0.001220042	0.044530706	0.051017125
Ap1s1	1	7.76E-27	6E-72	-0.007183107	0.04119849	0.050998233
Naa35	1	1.00E+00	3E-22	0.006753294	0.027903863	0.050998039
Mul1	1	5.32E-02	7E-61	0.003323366	0.018593121	0.050989579
Ldb1	1	1.00E+00	3E-30	0.00621002	0.004579206	0.050899834
Cd247	1	1.79E-04	2E-101	-0.000255622	0.017133636	0.050841443
Eif2s1	1	5.83E-12	1E-39	-0.000573896	0.043755609	0.050818433
Slc39a13	1	4.17E-29	2E-73	0.000231241	0.042705459	0.050687086
Wdr18	1	9.74E-04	3E-61	0.006622812	0.021436035	0.050666562
Trim23	1	1.34E-03	1E-41	0.003382019	0.02710602	0.050631196
Mpzl2	1	1.00E+00	1E-21	-0.011770225	-0.009952172	0.05056474
Malt1	0.00029185	1.98E-03	7E-22	-0.018160743	0.048489735	0.050538243
Gpr180	1	5.28E-14	1E-45	0.00225631	0.044120003	0.050521059
Tmem51	1.57324E-10	3.19E-73	2E-32	-0.020605651	0.120153963	0.050482397
Drosha	1	1.00E+00	3E-28	-0.009499328	0.020256494	0.050468796
mt-Nd2	1	1.80E-38	2E-105	-0.003206547	0.04046877	0.050464256
Rap1gds1	0.508776496	4.27E-13	7E-11	-0.019301431	0.077676938	0.050356663
E030042O2	1	3.20E-50	1E-180	-0.000865972	0.02396896	0.050343897
Zmynd8	1	6.64E-16	9E-07	0.01789252	0.10843776	0.050333022
Rad50	1	1.00E+00	2E-18	0.001871364	0.01743447	0.050315483
Skiv2l	1	1.00E+00	9E-26	-0.002243564	0.001670895	0.050279661
9330159M	1	6.04E-09	2E-24	-0.010970527	0.051386259	0.05027728
Cep83os	1	3.21E-20	2E-59	-0.004606377	0.040431116	0.050233734
Commd10	1	2.99E-05	3E-29	-0.002714604	0.040023959	0.05023074
Rrp8	1	2.19E-35	6E-73	0.006890148	0.04779594	0.050164198
Tdp2	1	1.00E+00	1E-33	-0.004081148	0.00917214	0.050159412
Rbm42	1	1.07E-16	3E-50	0.002096654	0.041342088	0.050150013
Mfsd10	1	3.85E-49	3E-132	0.000576561	0.035744704	0.050146904
Frk	1	1.00E+00	3E-34	0.001329547	0.008967203	0.050144359
Hars2	0.000272118	1.00E+00	1E-36	0.016670196	0.013553745	0.050090243
Pak2	0.00333139	5.66E-04	1E-10	0.026477026	0.069430051	0.050040102
Setd1a	1.5207E-15	1.00E+00	3E-19	0.039155673	-0.013959886	0.050003308
Mrps26	0.114237071	8.22E-21	1E-84	0.008154721	0.032968703	0.049974168
Gdpd2	1	1.23E-130	2E-208	0	0.032155913	0.049969989
Znhit6	1	7.34E-01	7E-30	0.005072756	0.023599019	0.049967317
Rab3ip	1	2.06E-06	1E-11	-0.009553152	-0.043307477	0.049964661
Gm44507	1	0.00E+00	9E-134	-0.003104932	0.154021333	0.049947349
Slc25a1	3.07959E-07	1.00E+00	1E-48	0.017338973	-0.00359523	0.049927961
Aarsd1	2.29979E-05	4.82E-26	1E-62	0.013649146	0.044038663	0.049886034
Stam2	1	3.54E-11	3E-23	0.003275161	0.060449098	0.049823211
Srrm4	1	2.11E-11	1E-60	-0.000718304	0.031106235	0.049815406
Ubl5	0.251728357	2.97E-19	6E-90	0.007044927	0.032471469	0.049812998
Atp5mpl	1	4.93E-12	3E-50	-0.000696543	0.035716331	0.049775586
Gfer	1	9.65E-01	3E-65	0.004761883	0.015211879	0.049761622
Srrm4os	1	1.91E-19	9E-35	0.007751658	0.055106619	0.049722353
Babam1	1	1.00E+00	1E-44	0.002601926	0.018248948	0.049713966
Slc22a4	0.005427999	4.70E-12	1E-29	-0.013082689	0.054815018	0.049712272

Usp7	4.13573E-05	1.00E+00	1E-14	0.027891344	0.018566608	0.049690095
C87436	1.48096E-11	6.90E-03	4E-23	0.031725015	0.037216461	0.049672194
Uap1l1	1	1.59E-209	2E-197	-0.000256842	0.058274364	0.049614341
Atp6v0a4	1	4.01E-136	2E-159	-0.000644247	0.052177649	0.049613023
Gm4924	1	8.19E-01	2E-58	-0.001693223	0.015470843	0.049611267
Kctd9	1	1.80E-03	3E-32	-0.007874104	0.03182084	0.04960877
Ifnlr1	1	2.40E-204	1E-218	-0.000101818	0.049263934	0.049557019
2700097O(	1	1.00E+00	1E-16	0.011544065	-0.018655556	0.049540915
Slc30a6	1	2.68E-05	2E-43	0.009304666	0.02893664	0.049536322
Ric1	1	1.00E+00	1E-10	0.000814548	-0.002305117	0.049514427
Gm49359	1	1.00E+00	5E-42	-0.003035941	0.01789101	0.049491831
Ptcd2	0.008079091	1.24E-24	1E-79	0.008853144	0.038638676	0.049446185
Gm7072	1	1.00E+00	7E-29	0.004473389	0.009587701	0.049434878
Aifm3	8.3336E-19	3.49E-19	7E-63	-0.016704938	0.036272875	0.049422138
Aagab	0.172570199	9.62E-04	2E-25	0.01572412	0.037333773	0.049408519
A430105J0	1	2.59E-77	1E-130	0.000972374	0.044970499	0.049393408
Mmp27	1	0.00E+00	5E-168	-0.000446602	0.144052875	0.049379463
Syt11	1	7.09E-43	3E-94	-0.004999153	0.042176691	0.049372382
Cdc16	0.092449877	3.31E-04	1E-45	0.012493256	0.026334553	0.049347373
4930481A1	0.032614087	1.00E+00	3E-22	0.018818334	-0.016433662	0.04929785
Rcc2	1	1.27E-57	8E-35	0.007582236	0.091831615	0.049294427
Snhg9	1.24087E-11	4.65E-07	4E-19	0.029383661	0.052290904	0.049291125
Ccdc77	1	1.00E+00	5E-27	-0.001079165	0.014047668	0.049262224
Lmbrd1	1	1.00E+00	2E-11	-0.003765002	0.019652257	0.049251033
9030624G2	4.22114E-11	1.34E-01	0.0002	-0.056022754	-0.030221864	0.049249992
Prim1	1	4.62E-47	9E-126	0.000362529	0.033841423	0.04923467
Bccip	1	1.00E+00	1E-44	0.006435416	0.015565931	0.049229875
Arl2bp	1	5.15E-69	2E-129	-0.000108122	0.041124176	0.049201765
Gm17494	1	1.08E-88	5E-69	0.006168844	0.077349152	0.049169681
Unc93b1	1	9.83E-59	3E-32	-0.011564117	0.110392314	0.049141804
E4f1	0.033502549	3.42E-04	6E-40	0.012848458	0.028058065	0.049044191
Uso1	1.49303E-16	2.41E-03	7E-10	0.051809967	-0.033350944	0.04900755
Spryd3	0.000508829	3.61E-27	8E-63	0.012123131	0.045225263	0.048991961
Kpna4	1	5.01E-54	3E-09	0.003792307	0.170587067	0.048977043
Pikfyve	0.003027629	1.00E+00	5E-12	0.026744641	0.011948798	0.048927051
Rfng	1	3.69E-29	2E-114	0.001900013	0.029743539	0.048920219
Pet100	1	3.42E-16	3E-49	0.00146054	0.041673686	0.04890476
Dmac2	1	1.00E+00	1E-24	0.008577523	0.024828609	0.048853333
Elac2	0.005650875	4.29E-04	6E-71	0.010192494	0.017567685	0.048847878
Mta2	1	5.05E-04	9E-41	0.01005513	0.02814841	0.048813733
Sgta	0.016232594	1.00E+00	3E-29	0.016578538	0.012111624	0.048808213
Actl6a	0.211774922	1.14E-01	3E-37	0.012795566	0.02257417	0.048775881
Mrpl45	1	6.69E-01	2E-44	-0.00235768	0.018539965	0.048744459
Mtf2	1	1.00E+00	6E-17	-0.004441398	0.015146577	0.048701653
Runx1	1	0.00E+00	4E-91	-0.001944021	0.21966164	0.04868675
Cdc123	1	1.00E+00	1E-16	0.01489091	0.015555443	0.048636804
BC002059	1	1.00E+00	3E-28	-0.001752843	0.017897869	0.048616202

Myo7b	1	1.13E-111	2E-185	-0.000929799	0.036253406	0.048612151
Dclre1c	8.67779E-16	1.46E-14	3E-11	-0.036092758	0.083443976	0.048588093
Ccnc	1	2.98E-13	6E-44	-0.000585988	0.039070476	0.048560885
Nr2c2ap	0.001028722	2.95E-02	3E-47	0.014572517	0.022444401	0.048535613
Rps3	1	6.72E-68	6E-67	9.35756E-05	0.069709338	0.048529808
Ccdc82	1	1.85E-23	8E-37	-0.000937453	0.058934219	0.048509451
Cfap44	1	1.39E-57	4E-201	-0.000142048	0.015103855	0.048505745
Ints10	1	4.59E-08	3E-75	-0.000227223	0.023408677	0.048494726
Adamts1	0.367348069	4.15E-66	3E-130	0.0050506	0.04031544	0.048490486
Ppil2	1	1.00E+00	3E-21	0.015712476	0.006078656	0.048484666
Polm	1	5.86E-04	2E-50	0.006551361	0.022896176	0.048476289
Myo10	1	1.00E+00	1E-07	-0.015626882	0.01231464	0.048468106
Ttf1	1	1.00E+00	9E-19	0.008889086	0.011760863	0.0484125
Nat9	0.004290155	3.01E-05	3E-46	-0.012171176	0.027318064	0.04841038
Utp18	0.153324864	3.96E-22	4E-50	0.011049596	0.045370131	0.048400177
Gm42161	1	9.14E-69	5E-119	-0.000494963	0.044719641	0.048349083
Mrpl13	1	8.89E-05	7E-50	-0.003124767	0.024083997	0.048329773
Abcc9	1	1.66E-100	7E-143	0.000814963	0.047350487	0.048323928
Plcl2	1	8.49E-53	2E-07	0.000797673	0.170573213	0.048295733
4930527F1	0.790110418	2.11E-26	3E-89	-0.005208599	0.032351504	0.048231207
Ttc1	1	2.62E-03	1E-37	0.009637353	0.028077446	0.04822705
Cox6c	1	3.33E-18	2E-33	-0.005394259	0.05866804	0.048179416
Chmp2b	1	5.68E-14	1E-64	-0.001673004	0.031602556	0.048170315
Cmas	1	1.00E+00	2E-20	0.00717719	0.027913662	0.048149671
Gtf2f1	1	1.53E-01	1E-61	0.000745913	0.016840027	0.048147727
Bcs1l	1	9.97E-15	1E-74	-9.50782E-05	0.027896655	0.048140487
Atp6ap1	1	2.11E-06	9E-53	0.006214257	0.031307018	0.04810464
Fbxo38	1	1.00E+00	3E-19	0.012467182	0.000206703	0.048101888
1300017J0	3.37489E-16	1.77E-25	3E-08	-0.048344319	-0.093719837	0.048099816
Ndufb10	1	4.20E-22	3E-61	0.001033091	0.03934986	0.048084891
Atp10a	1	9.02E-102	2E-168	0.000430872	0.036441637	0.048075763
Ctsa	1	4.36E-36	2E-24	-0.001629353	0.099413481	0.048051478
Psmb4	1	1.06E-09	3E-57	-0.001758549	0.031069623	0.048045085
Mettl5	1	4.85E-21	9E-45	-0.005466652	0.046215004	0.048034475
Cir1	1	1.00E+00	5E-17	0.015929533	0.01473811	0.047999452
Gapdh	1	2.40E-10	4E-72	0.003502494	0.025455484	0.047998752
Zc3h14	1	1.00E+00	1E-19	0.008366967	-0.003841164	0.047973004
Cox18	1	7.77E-05	3E-70	-0.0012418	0.018700913	0.047945889
Naxd	1	1.00E+00	1E-29	0.013112003	0.012332443	0.047858202
Comtd1	1	1.85E-106	4E-185	0.001622113	0.036545405	0.047832965
Zfp618	1.97742E-11	1.00E+00	7E-44	-0.018047231	0.016357605	0.047751329
Smox	1	1.60E-118	8E-121	-0.003003039	0.070436156	0.047739065
Dctn5	1	2.32E-13	6E-40	0.00383179	0.042593638	0.047700349
Pwp2	1	1.36E-19	2E-78	0.00602341	0.030711476	0.047663333
Fgd1	0.148169607	4.20E-33	2E-100	-0.005067636	0.033721609	0.047657589
Stt3a	2.25426E-07	1.00E+00	7E-27	0.022233077	0.004414325	0.047599329
Bud31	1	1.22E-08	3E-64	0.00222695	0.025364155	0.047589808

Gm49390	1	1.08E-217	4E-190	-0.000580753	0.062850049	0.047561863
Ank	5.83394E-25	1.00E+00	0.0009	-0.056361466	0.04588804	0.04755149
Wdpcp	7.35757E-06	1.00E+00	3E-09	-0.02387825	0.024812979	0.047538215
Hdac3	1	1.00E+00	5E-31	-0.002922334	0.012380673	0.047535621
Immp1l	1.579E-05	1.00E+00	4E-12	-0.026868479	-0.010442637	0.047487707
Cops2	1	4.71E-04	6E-30	0.002153328	0.031208986	0.047481778
Coro6	1	1.72E-110	2E-197	0.000158164	0.031307978	0.047474844
Nmrk1	1.05825E-07	2.50E-02	2E-14	-0.027486542	-0.026534525	0.047474068
Slc35a4	2.3266E-05	4.93E-05	1E-66	0.012511532	0.019883767	0.047461171
Tmed4	0.000301362	8.23E-10	1E-67	0.01112778	0.024280126	0.047449356
Mrpl19	1	3.51E-12	1E-76	0.003005489	0.025912905	0.047446201
Prpf31	1	7.73E-15	6E-78	0.002419375	0.02779735	0.047424044
Zfp78	1	5.85E-34	1E-91	-0.001806668	0.036041398	0.047420214
Nudt21	1	1.00E+00	1E-35	-0.000216949	0.018607747	0.047408325
Ech1	1.15196E-07	1.00E+00	3E-21	0.026629467	0.020096974	0.047383242
Phactr2	1	1.48E-65	5E-93	-0.002576829	0.06678457	0.04735192
Dock6	1	1.00E+00	2E-33	-0.004496553	-0.004062256	0.047333938
Aptx	1	1.10E-12	2E-53	-0.006078199	0.032251508	0.047329936
mt-Nd4	1	2.91E-24	4E-81	-0.00408346	0.036424347	0.047285678
A930015D0	1	1.00E+00	2E-19	0.006860813	0.009683516	0.047274735
Sf3a3	1	5.82E-02	3E-39	0.009141952	0.021529268	0.047238296
Atrip	1	1.00E+00	4E-53	0.007343975	0.011976334	0.047233705
Prorp	1	1.00E+00	7E-16	0.005643502	-0.001892231	0.047223532
Mapre2	0.56961036	1.11E-83	2E-39	0.010507137	0.11152155	0.047216802
Xpc	1	3.28E-06	6E-39	-0.001674423	0.030548452	0.047151412
Cdk15	1	3.10E-134	7E-177	-0.00066343	0.043038958	0.047102568
Kctd3	1	1.00E+00	1E-24	0.004617372	0.023887221	0.047084775
Wdr77	1	7.77E-09	4E-51	-1.1682E-05	0.028376424	0.047044517
Med20	1	2.17E-02	1E-28	-0.006767668	0.028578358	0.047039105
Thpo	1	1.00E+00	9E-42	-0.002711609	-0.001245261	0.047037031
Fcsk	1	1.17E-16	4E-66	0.006010238	0.030873055	0.047035762
Mrpl37	1	1.00E+00	7E-46	0.002023504	0.013447617	0.047025913
Rrm1	1	1.14E-24	5E-92	-0.002894216	0.031588292	0.047011951
Edem2	1	8.37E-01	1E-21	0.00772689	0.029452443	0.046988892
Aspa	2.04604E-08	1.00E+00	2E-24	-0.023561024	0.0145411	0.046963877
Chd1l	0.00046483	1.00E+00	5E-20	-0.026669831	-0.001528224	0.046954536
Ptprc	1	0.00E+00	8E-89	-0.000316059	0.202163516	0.046932826
Cpsf1	1	1.00E+00	6E-41	0.00473345	0.017338747	0.04691508
Gyg	1	6.29E-84	7E-129	-0.001241973	0.045532337	0.046889401
Tma16	1	1.22E-27	9E-73	0.00065244	0.03659541	0.046887381
Tomm6	1	1.63E-19	2E-72	0.005909434	0.033844927	0.046849321
Wdr61	1	1.00E+00	5E-25	-0.004711111	0.008103308	0.046778214
Ifih1	1.69688E-10	1.65E-09	6E-22	-0.024517233	0.055749917	0.046776474
Utp25	0.001227746	1.79E-10	5E-33	0.015783857	0.040731511	0.046765239
Eln	1	8.87E-29	4E-154	-0.000518269	0.016794984	0.046692226
Zfp948	1	1.00E+00	5E-43	-0.002863009	0.012206135	0.046660161
Reep5	1	9.57E-78	3E-111	0.004167304	0.048830878	0.046639263

Ttc13	1	1.00E+00	9E-28	-0.003117027	0.017317849	0.046625882
Mrpl20	1	6.41E-10	2E-55	-0.000534214	0.029490044	0.046622115
Pik3c2b	0.002222835	3.65E-59	6E-81	-0.00760399	0.053367389	0.046574288
Ces1h	1	1.50E-300	2E-203	-0.000122396	0.071560164	0.046455556
Adra2a	1	1.97E-87	5E-201	-1.78476E-05	0.020978746	0.046449606
Fabp3	1	5.81E-127	1E-156	0.000463828	0.047443494	0.046449331
L3mbtl2	1	3.02E-08	9E-50	0.006893893	0.028972901	0.046406135
Pde7a	1	1.00E+00	5E-16	0.000682387	0.007780192	0.046387569
Mmut	1	1.00E+00	4E-24	0.001726551	0.005046473	0.046378726
Mvd	3.5158E-70	1.00E+00	1E-95	0.034687337	-0.004531565	0.046351089
Dnajb9	0.313470167	1.56E-07	1E-53	0.009993358	0.026580134	0.046314806
Ubxn2a	1	1.73E-02	2E-23	-0.010250707	0.029408089	0.04628802
Sec13	0.020953509	3.26E-13	2E-75	0.008684008	0.026559993	0.046265658
Cpsf2	1	4.04E-05	6E-36	0.003989725	0.029111717	0.046251906
Rsrc1	0.664875681	2.69E-03	8E-06	-0.025441579	0.063567427	0.046214694
Nhlrc2	6.34739E-07	3.04E-14	1E-06	-0.031636592	0.097197654	0.046203655
Gm10130	1	7.86E-02	1E-49	-0.000770663	0.017702891	0.04620218
Lnpk	1	5.33E-10	2E-30	-0.009400081	0.041794967	0.046200851
Mpg	1	8.46E-07	6E-68	0.000633738	0.021070361	0.046191293
Kif5a	1	2.95E-03	4E-47	0.006242175	0.024183581	0.046182202
C77080	3.13948E-64	1.00E+00	4E-14	-0.065146471	0.031838728	0.04618183
Zfp758	1	1.00E+00	2E-57	-0.004573003	0.012515817	0.046177288
Ltbr	1	2.54E-38	2E-39	0.003812345	0.064010533	0.046173868
Srcap	1	1.19E-01	6E-08	0.001286837	0.044618161	0.046168969
Piezo1	1	1.34E-88	2E-86	-0.000358698	0.074313817	0.046167242
Angptl8	1	5.44E-01	2E-31	0.000100629	0.021504035	0.046069101
Cebpb	2.96174E-16	1.22E-12	3E-13	0.041613907	0.073265695	0.046066114
Ppt1	1	3.37E-32	2E-38	-0.00703302	0.067924525	0.046044707
Prkd2	1	1.83E-18	1E-44	0.002464442	0.045204616	0.046034422
Stam	0.070595931	1.00E+00	1E-17	0.018502197	-0.000424641	0.046032018
Ldlr	8.36228E-11	1.54E-45	0.0229	0.054002441	-0.143594232	0.046030796
Sytl2	1	6.63E-37	1E-166	0.000249663	0.014246767	0.046023763
Acad8	0.976945225	1.00E+00	8E-20	0.015548017	0.025940457	0.046006047
Kbtbd12	1	7.89E-100	4E-114	-0.000772304	0.053937445	0.045981201
Lncppara	1	1.00E+00	2E-07	-0.000977185	-0.008776387	0.045969314
Fam219a	1	1.25E-04	5E-14	-0.005944458	0.07309711	0.04588115
Rims4	1	1.69E-153	7E-186	6.34655E-05	0.041724121	0.045879262
Lgr4	1.78409E-20	2.21E-13	0.0128	-0.064622498	0.119428564	0.04587738
Tti2	1	1.84E-07	5E-56	0.006988141	0.025444042	0.045873792
Llph	1	2.02E-05	7E-52	0.004800381	0.023501526	0.045851055
Incenp	1	2.76E-54	4E-132	-0.000298864	0.033848504	0.04584498
Rpl7	1	7.88E-45	3E-58	-0.001910907	0.057944372	0.045836854
Ston2	0.000749688	1.00E+00	3E-09	-0.024667825	0.044326869	0.045824307
Ppp1r15a	1	3.47E-10	3E-67	0.002543322	0.024815362	0.045822432
Osgepl1	0.006625969	5.70E-14	2E-47	-0.010087093	0.03758475	0.045818299
Mcm6	1	8.55E-29	3E-86	-0.0036177	0.035180568	0.045795342
BC029722	1	6.38E-44	3E-135	0.000995054	0.028935705	0.045790647

Orai1	8.59952E-13	4.52E-09	2E-32	0.023821284	0.037684372	0.045780097
Pgghg	1	1.00E+00	2E-60	0.000606668	0.005647128	0.045746447
Adss	1	1.00E+00	4E-17	-0.009407057	-0.004713315	0.045707522
Mtx3	0.00039535	1.00E+00	8E-28	-0.016370546	0.021533559	0.045693314
Ap5z1	1	9.23E-01	9E-36	0.001642903	0.022061087	0.045680558
Becn1	1	1.00E+00	3E-18	0.012561959	0.00121205	0.045657421
Ltv1	1	5.45E-25	1E-61	-0.001240318	0.039185268	0.045643632
Cep78	1	2.52E-25	6E-67	-0.002955154	0.03746352	0.045607715
Gm19705	1	1.21E-13	2E-73	0.005720481	0.028948041	0.045605464
Psd4	1	1.02E-108	8E-130	-0.003243733	0.052711991	0.045568399
Isca2	6.62012E-07	1.00E+00	4E-61	0.013484259	0.014185313	0.045518408
Tsen2	0.003776791	4.34E-13	3E-39	-0.011815335	0.038276398	0.045515173
Txndc9	1	2.25E-11	3E-42	-0.003011266	0.03633561	0.045503292
Gm19605	1	4.40E-18	1E-57	-0.002540385	0.033436947	0.045497816
Epb41l3	1	3.61E-153	5E-126	-0.000740967	0.063189334	0.045496155
Zfp335	0.782738639	9.89E-04	3E-47	0.009531585	0.021726122	0.045475753
Kctd5	1	3.11E-15	2E-50	-0.001665553	0.035400349	0.045469426
Epha1	1	1.17E-05	5E-36	-0.004114273	0.028398761	0.045461077
Pdcd10	1	1.00E+00	4E-25	-0.005479271	4.00022E-05	0.045439134
Primpol	0.138214562	1.50E-04	3E-41	-0.01030667	0.026487434	0.045417877
Cnep1r1	1	3.23E-01	2E-32	0.005502058	0.02381071	0.045413612
Sec61b	1	1.07E-03	7E-62	0.007755413	0.019039411	0.045401541
Reep4	1	1.05E-59	9E-94	-0.002281668	0.045548995	0.045320613
Dkk3	3.90425E-06	2.98E-125	1E-107	-0.004974661	0.064071094	0.045233118
Cep89	1	3.09E-02	2E-45	-0.001378506	0.019278374	0.045231116
Ostc	1	1.00E+00	3E-41	0.009812909	0.015854428	0.045222123
Polr2k	1	1.26E-41	1E-93	0.001372053	0.037814459	0.04520996
Gm35330	1	3.16E-92	2E-120	-0.000337639	0.039834363	0.045181719
Gpn2	1	4.74E-21	2E-57	-0.0031062	0.037776483	0.045177495
Gabrb3	1	4.67E-13	6E-84	0.003285802	0.02260151	0.045174148
Gm32017	1	7.58E-05	7E-43	-0.001914205	0.020788245	0.045161132
Ints6	1.65487E-27	1.00E+00	4E-07	0.066893948	0.000254634	0.04513694
Tmem258	2.63481E-06	5.26E-14	6E-96	0.009359278	0.021392121	0.04512371
Rab11fip5	1	1.09E-158	2E-177	-0.000828835	0.047184793	0.045118955
Gpr35	1	4.40E-03	3E-62	-0.001497215	0.018194516	0.045115756
Dhrs1	1	2.16E-02	2E-53	-0.003881931	0.018510451	0.045062326
Lrrc59	1	1.36E-03	3E-48	0.007658323	0.021988611	0.045040109
Eif3i	1	5.15E-16	6E-54	0.007666082	0.033341805	0.045035576
Btbd10	1	4.95E-13	2E-43	-0.001496889	0.036153	0.045016677
Pdxdc1	1	1.00E+00	4E-08	0.011359733	-0.014448316	0.044983534
Ppp1r7	1	1.70E-10	6E-52	0.004907886	0.030902581	0.044980333
Tmx2	1	8.15E-12	2E-78	0.001008872	0.0234535	0.044955545
Ppa1	1.09125E-11	1.17E-01	5E-11	0.034927609	-0.026822215	0.044920771
Pkp3	1	9.95E-139	5E-182	0.000189206	0.039519262	0.044884469
Zfp568	1	1.00E+00	9E-09	0.002434589	-0.01297863	0.0448638
Rrp15	1	5.05E-15	1E-43	0.008735387	0.037892151	0.044852195
Psmc2	1	1.00E+00	4E-27	0.009849242	0.0120932	0.044850269

Uvrag	1	1.00E+00	0.0127	-0.009573294	0.021170793	0.044846147
Actr3	1	5.23E-55	7E-14	-0.006596461	0.139765683	0.044832596
Ndufb11	3.20662E-07	5.42E-07	1E-68	0.01278712	0.02121518	0.044819837
Cln8	1	2.35E-16	6E-52	0.003194915	0.036419157	0.04481955
Dact1	1	1.00E+00	2E-120	-0.000221769	0.006140993	0.044810801
Mpdu1	0.12382773	1.00E+00	3E-22	-0.013434013	0.018365437	0.04479769
Zdhhc6	1	8.22E-01	6E-13	0.00763406	0.034664383	0.044796894
Nop10	0.00080959	5.30E-18	8E-95	0.008184381	0.024516757	0.044789038
Idh2	1	1.88E-06	3E-37	-0.005964288	0.031280839	0.044743004
Ccny	1	1.00E+00	1E-06	0.000500559	0.028463969	0.044727883
Taf4b	1	2.92E-04	1E-35	0.008566538	0.026048918	0.044686504
Smyd3	1.14307E-52	4.55E-13	0.0019	-0.111592777	0.113654435	0.044678821
Ypel5	0.02460951	2.91E-01	3E-13	-0.016127913	0.038336617	0.044675181
Slc6a12	1	1.00E+00	1E-31	0.011018398	0.004511573	0.044643114
H13	9.14385E-06	1.00E+00	3E-10	0.030750316	4.43556E-05	0.044635061
Ppp4r1	1	3.12E-20	7E-13	-0.002507673	0.089540494	0.044584326
Ints3	1	4.70E-14	8E-25	0.000449627	0.051753207	0.044581182
Akt1	1	7.35E-36	4E-69	-0.00204856	0.045137633	0.044550382
Vtn	1	1.00E+00	3E-07	0.000579227	0.008936705	0.044540098
Rnaseh2c	1	6.00E-20	3E-88	1.90147E-05	0.027026666	0.044527273
Mlec	1	1.00E+00	8E-18	0.004278565	0.008702325	0.044521073
Gm15764	1	8.22E-54	7E-54	0.006014968	0.06207922	0.044494987
Mkrn2os	1	3.17E-17	4E-56	-0.006351334	0.033201949	0.044474049
Spint2	1.15755E-13	4.52E-05	4E-16	-0.033207312	0.046681554	0.04445731
Dpf2	1	2.56E-02	3E-24	0.001331428	0.027357784	0.044428838
Idua	1	3.55E-23	2E-72	0.002039291	0.03449574	0.044411417
Chaf1b	1	5.52E-30	4E-137	-0.000497807	0.0207067	0.044375192
Anapc2	1	2.63E-05	1E-37	0.004675685	0.029863808	0.044352591
Gjb2	1	3.77E-04	2E-34	-0.006716604	0.028344121	0.04434677
Nae1	0.558104679	8.64E-03	8E-24	0.013475384	0.029803832	0.044316487
Tada3	1	6.86E-15	2E-55	0.001507411	0.031625488	0.044305502
Dhx37	1	1.07E-17	7E-59	-0.000169405	0.033872672	0.044291887
Nup133	1	1.96E-20	5E-41	-0.001586516	0.043452281	0.044267279
Rpe	1	1.94E-13	5E-37	0.001556624	0.039414481	0.044244156
Eef1aknmt	0.660942401	6.46E-13	1E-74	0.0065351	0.023318635	0.044242161
Fastkd2	1	2.80E-02	2E-54	0.00258696	0.016241383	0.044190555
Ran	1	2.16E-24	4E-79	0.004028321	0.032598756	0.04418893
Fam133b	4.48886E-18	1.00E+00	8E-12	0.045644212	-0.012249333	0.044182766
Gm16322	1	4.24E-99	3E-184	5.90036E-05	0.024738787	0.044169884
Xkr6	0.58374471	1.00E+00	3E-13	-0.014215823	-0.003756413	0.04406239
H2-D1	1.83923E-05	2.25E-68	2E-25	0.016618946	0.12439214	0.044061363
Ranbp3	1	1.00E+00	2E-18	0.013220515	0.008401064	0.044045265
Snhg5	0.191159372	2.28E-31	5E-87	0.006236232	0.032534016	0.044008084
Ankfy1	1	1.61E-08	2E-15	0.004873606	0.061844103	0.043996693
Cox8a	1	5.66E-09	1E-31	0.003810775	0.0381819	0.043982953
Fgf9	1	2.40E-146	2E-148	-7.79071E-05	0.05217959	0.043978568
Triap1	1	8.22E-27	7E-108	0.003169413	0.025371307	0.043970828

Kri1	1	1.00E+00	5E-29	0.003827892	0.014348183	0.043958858
Leprotl1	1	2.49E-18	2E-73	0.003809784	0.029290938	0.043945453
Gm10785	1.45864E-06	5.45E-27	1E-47	0.015309788	0.047268006	0.04392522
Utp6	1	6.53E-06	5E-31	0.007382179	0.03134714	0.043910474
Gm14399	1	5.73E-19	3E-69	-0.00227161	0.030771013	0.043795258
Jtb	1	1.00E+00	8E-32	0.008215976	-0.009118678	0.043788192
Cdc23	1	8.27E-08	4E-49	0.00731419	0.027509767	0.043770787
Fam98a	1	5.55E-02	6E-32	0.006144842	0.021447184	0.043770131
Trappc6a	1	8.65E-13	1E-79	0.003388463	0.02277585	0.043767907
Stx3	1	1.30E-213	2E-167	-0.000124692	0.065896547	0.043764888
Dnajc16	1	2.43E-12	1E-44	-0.000101073	0.032960067	0.043760928
Apmap	1	4.01E-10	2E-53	0.004799743	0.026790033	0.043759484
Cog6	1	1.00E+00	2E-37	0.001343753	0.007853308	0.043717565
Tspan4	9.40155E-11	2.09E-61	1E-74	-0.010299166	0.05099682	0.043707957
Serhl	1	2.21E-04	1E-32	-0.00649611	0.029133778	0.043700612
Nin	1	4.47E-135	4E-112	-0.000648933	0.066755131	0.043694937
Epdr1	1	3.25E-139	5E-189	-4.01094E-05	0.034378727	0.043666813
Cenpl	1	1.67E-19	2E-62	0.000719338	0.033593246	0.043651398
Pou2f2	1	1.28E-258	6E-82	0.00051067	0.158966781	0.043644322
Uaca	7.56776E-06	4.24E-07	7E-52	-0.011178196	0.023914372	0.043634391
Actr1a	1	1.00E+00	1E-18	-0.006186808	0.026866704	0.043633746
Zfp750	2.18029E-12	3.57E-18	4E-74	0.014316436	0.027209429	0.0436256
Carm1	0.0784762	1.28E-14	7E-26	0.015038049	0.046937429	0.043611518
Haus5	1	1.15E-50	1E-84	0.001136847	0.04259887	0.04360107
Cd5l	1	5.18E-290	1E-94	-0.001619916	0.149364203	0.043600237
Zbtb34	1	1.00E+00	4E-32	0.001805956	0.019275026	0.043582948
2700049A0	9.80326E-12	1.00E+00	7E-09	-0.033559046	0.008978379	0.043571176
Cipc	1	8.18E-02	1E-10	0.010011567	0.043818841	0.043540231
Pgls	1	2.11E-09	3E-32	0.00143826	0.03644911	0.043507176
Zfp995	1	6.56E-01	4E-44	-0.005421686	0.018038687	0.043468781
Dars2	1	1.37E-26	3E-40	-0.003492343	0.051584307	0.043453088
Haus6	1	5.60E-26	4E-57	0.000954779	0.041460695	0.043452577
Nup107	1	1.20E-14	2E-50	-0.000928035	0.032805683	0.043443745
Gm26632	4.58672E-66	5.86E-38	6E-24	0.061736805	0.075033448	0.043440072
Ddx47	1	1.00E+00	4E-34	0.00593155	0.011177651	0.043437159
Hdhd2	1	1.00E+00	3E-26	-0.00441257	0.016405516	0.043436495
AU041133	1	1.00E+00	2E-33	-0.00207532	0.001490857	0.043388466
Exog	1	4.25E-11	4E-64	0.001837734	0.024454703	0.043343769
Brf1	1	1.00E+00	3E-15	0.009897098	0.022627504	0.043254574
Brca1	1	9.05E-35	2E-103	0.001312908	0.028353766	0.043239704
Cd84	1	0.00E+00	1E-89	8.18873E-05	0.241782534	0.043231116
Sec61g	1	2.36E-18	4E-69	0.003567299	0.030803933	0.043198002
Gm16090	0.166788219	1.65E-28	8E-68	-0.00686409	0.036944979	0.043194627
Traf6	1	7.17E-07	1E-33	0.003638229	0.034014271	0.043144029
Tpt1	1	1.08E-23	1E-26	-0.010835769	0.065017101	0.0431371
Zfp397	1	1.00E+00	4E-16	-0.001606206	0.023282849	0.043129234
Ost4	1	2.11E-34	2E-82	0.004817975	0.035749794	0.043126925

Mre11a	1	6.29E-15	4E-31	-0.002685899	0.046167057	0.043098196
Umps	1	1.32E-36	2E-91	-0.003075101	0.032918918	0.043091341
Slc25a39	1	1.00E+00	3E-22	-0.001665633	0.017618471	0.043051508
Cox15	1	1.22E-11	4E-53	0.005451566	0.029075826	0.042999
4921511C1	1	3.95E-37	2E-53	0.006401036	0.048705983	0.042988321
Taf6	1	1.00E+00	5E-35	0.008545653	0.017785917	0.042950998
Chd8	1	9.28E-06	1E-07	0.016853332	-0.040342502	0.042940421
Romo1	1	3.91E-28	1E-73	-0.001630587	0.036134978	0.042938678
Wdr1	1	1.21E-40	4E-51	0.000545163	0.058994852	0.042925202
Anks3	1	1.00E+00	3E-20	0.008915226	0.005845782	0.042877396
Ly6e	1	6.36E-109	6E-66	0.0036321	0.085381231	0.042866997
Spout1	1	9.17E-09	3E-80	0.005823691	0.018982677	0.04284165
E2f6	1	5.77E-18	2E-49	0.001148694	0.038555246	0.042817746
Ctdp1	0.011142548	3.46E-07	4E-23	0.016513784	0.04261877	0.042815959
Coro1c	1	8.81E-34	1E-34	-0.006547725	0.076185153	0.042806707
Crtc1	1	1.00E+00	7E-23	0.010074575	0.013022602	0.042805665
Actr2	1	1.52E-17	3E-15	0.013529241	0.085272313	0.042801822
Exoc1	1	4.19E-05	3E-32	-0.006363827	0.031918157	0.042786286
Zdhhc4	1	1.38E-06	1E-53	0.003591823	0.023502567	0.042718896
Sectm1a	1	1.60E-73	3E-172	-0.000299093	0.022766467	0.042710514
Ccpg1	0.051767826	1.04E-15	3E-18	0.016513395	0.061784225	0.042705501
Wdr60	1	1.00E+00	5E-20	-0.001125791	-0.015048644	0.042680208
Tsr3	1	2.68E-03	2E-73	0.005696759	0.015151832	0.042673574
Zfp949	1	1.00E+00	3E-19	-0.000498406	0.021272336	0.042648543
1600010M	0.001002844	5.72E-29	2E-26	0.016523451	0.066211682	0.042638094
Hemk1	1	8.22E-02	9E-39	-0.007851708	0.017485192	0.042635624
Pdk2	1	1.56E-02	6E-26	0.003740176	0.0266123	0.0426264
Alkbh2	1	1.37E-11	2E-57	0.002363592	0.02574803	0.04262149
Mdfic	3.1816E-64	1.00E+00	5E-07	-0.082397017	0.049893043	0.04257932
Med14	1	1.00E+00	9E-18	0.001016984	0.015809683	0.042577089
Asb6	0.00012404	3.27E-14	3E-77	0.008841267	0.024531222	0.042576644
Jkamp	1	3.37E-01	8E-39	0.004102401	0.017952732	0.04256805
Thyn1	1	1.71E-16	2E-80	0.002521994	0.023997118	0.042557295
Dnttip2	1	1.00E+00	6E-43	0.00403785	0.010350548	0.042550655
Manba	4.672E-51	1.00E+00	0.0004	-0.076063118	0.045456589	0.042511655
Rbmxl1	1	1.39E-30	9E-70	0.004773191	0.037550866	0.042469982
Gpatch1	1.26711E-06	1.00E+00	1E-16	0.024685433	0.008812964	0.042420934
Retsat	6.58751E-49	7.91E-13	3E-14	0.056778159	0.059090408	0.04241717
Fbxl18	1	1.00E+00	1E-26	-0.00143103	0.016205572	0.042376873
Jade3	1	2.04E-15	9E-24	0.005142697	0.052274275	0.042359017
Mif4gd	3.96972E-08	1.00E+00	6E-18	0.025842307	0.011366514	0.042356678
Zfp597	1	1.00E+00	4E-33	0.003335783	0.01920788	0.042304984
Micu2	7.98471E-33	1.00E+00	5E-10	-0.05476863	-0.007945974	0.042292811
Dock2	1	0.00E+00	5E-71	-0.00063021	0.25370169	0.042265431
Slc38a6	1	1.31E-01	2E-12	-0.009932083	0.050843982	0.042256
Cog8	1	8.99E-01	9E-46	0.005572598	0.014750945	0.042230881
Hnrnpu	0.201334871	1.69E-05	0.0077	0.022534795	0.078703854	0.042207381

Coq5	1	2.01E-01	4E-21	0.001581409	0.02483383	0.042196524
Dna2	0.429631695	1.00E+00	1E-14	-0.014294474	0.021420031	0.042177617
B4galt3	1	1.00E+00	1E-47	0.001205939	0.015427057	0.042164398
Nmt2	1	1.00E+00	7E-08	-0.014640073	-0.004383846	0.042120993
Nsun5	3.68652E-05	1.02E-07	1E-71	0.009884722	0.018793208	0.042105586
Zw10	1	1.00E+00	7E-33	0.006528945	0.012449269	0.042101984
Srbd1	6.11445E-14	1.70E-03	4E-05	-0.045279559	-0.04001868	0.042094948
Gpn3	1	3.05E-05	1E-51	0.002287204	0.021888665	0.042064717
Pakap.1	1	1.17E-05	1E-47	0.0049225	0.028294801	0.04204618
Arpc1a	1	8.16E-04	8E-21	-0.005842559	0.032305194	0.041990066
Prmt7	1	1.00E+00	2E-23	0.006387698	0.015309912	0.041982348
Prrc2c	1.22265E-31	1.00E+00	0.001	0.070582881	0.043769344	0.041971049
Stard7	1	1.00E+00	2E-06	0.008613737	-0.004249632	0.041953893
Washc3	1	1.00E+00	6E-21	-0.012029626	0.015752071	0.041943696
Fdx2	1	6.15E-20	4E-67	0.002768248	0.030622579	0.041932325
Pomk	1	1.17E-32	7E-85	-0.002065667	0.03582303	0.041914208
Dhx36	7.63033E-54	4.56E-09	2E-07	0.087328984	-0.055690445	0.041909475
Acpp	1	3.01E-55	4E-92	-0.001653025	0.04195876	0.041897397
Cox7b	1	2.05E-01	2E-30	0.001860785	0.023638305	0.041895875
Rbm28	9.06915E-17	1.00E+00	9E-10	0.045474423	-0.013850128	0.041878772
Fibp	1	1.00E+00	2E-42	0.005494017	0.012947587	0.041870371
Bysl	1	1.10E-34	1E-75	0.003220084	0.037103372	0.041851303
2210016L2	1	1.00E+00	2E-44	0.005470213	0.013656034	0.041838705
Cep57l1	5.07021E-08	1.00E+00	2E-10	-0.029763843	-0.004614821	0.041826059
2700062C0	1	1.58E-08	1E-61	0.001335703	0.022577876	0.041806926
Gm2814	1	2.66E-07	3E-69	0.004057788	0.019514589	0.041783017
Mrpl57	0.005775631	4.57E-45	1E-105	0.006216761	0.032166679	0.04177566
Pgam5	1	3.15E-06	3E-35	0.00213051	0.028250239	0.041775041
Zfp933	1	1.00E+00	2E-16	-0.004464908	0.011450001	0.041767022
Aldh5a1	0.081601007	5.23E-09	1E-19	0.015559378	0.043203995	0.041747899
D6Wsu163	1	5.59E-10	2E-51	-0.006231882	0.025289458	0.041736208
Cenpj	0.00732471	5.52E-26	9E-47	-0.008867142	0.042462564	0.041725173
Snw1	1	1.73E-03	5E-23	0.012987729	0.030624949	0.041683899
Wdr76	1	7.50E-13	2E-51	0.003670445	0.029596967	0.041644504
Rbl1	1	1.57E-67	7E-106	-0.001616355	0.040094836	0.041640336
Rpl22	1	1.48E-12	9E-26	0.008557626	0.044846424	0.041618331
Gm12689	1	1.10E-15	1E-56	-0.003146118	0.02968448	0.041612946
Camsap1	1	1.00E+00	5E-12	0.011582822	-0.009712021	0.041592716
Mynn	1	1.00E+00	1E-28	-0.000655553	0.019149417	0.041583049
Nudcd1	1	1.00E+00	8E-18	0.000795808	0.017822312	0.041549643
Fhdc1	1	2.02E-122	4E-175	1.88466E-05	0.031645378	0.041540429
Uba52	1	3.89E-103	8E-99	-0.001442686	0.056038907	0.041534583
Setd7	0.001137441	4.37E-43	6E-88	0.00799666	0.038730016	0.041461091
Ap5b1	1	9.97E-60	5E-161	7.98959E-05	0.022195685	0.041458013
Myo1f	1	0.00E+00	7E-99	-1.64152E-05	0.248936406	0.04145299
Slc25a27	0.001512315	3.13E-05	2E-36	0.013176647	0.024970705	0.041449721
Rae1	0.00018002	1.30E-02	4E-25	0.016812539	0.025885127	0.041435952

Ints1	1	1.14E-01	2E-50	0.005839643	0.017294715	0.041428379
Cox11	1	8.20E-01	3E-29	0.000330651	0.019845812	0.04139749
1810010H2	1	2.60E-39	5E-116	0.002056582	0.02526447	0.041381314
Las1l	1	1.00E+00	6E-26	0.003662716	0.014706653	0.041370688
Parp10	0.361679755	1.48E-18	1E-27	-0.010888551	0.050177427	0.041358601
Eif2b1	1	2.36E-30	2E-67	0.006007231	0.035387265	0.04134872
Gkap1	0.671286847	1.00E+00	9E-13	-0.014292431	0.001260536	0.041343308
Rps27l	1	5.91E-28	2E-47	-0.000946064	0.043598163	0.041335583
Cct5	1	7.45E-06	7E-37	0.001982673	0.029460195	0.041333227
Gm45044	1	1.46E-17	1E-53	-0.003097385	0.034562031	0.041330409
Dock10	1	0.00E+00	1E-64	0.001907411	0.224860593	0.041310242
Ncstn	1	4.03E-01	2E-20	0.003256209	0.027946388	0.041184461
Ikbkb	1	1.00E+00	5E-09	0.004902756	0.011437515	0.041134381
Polr3h	0.000963661	1.93E-15	3E-28	0.015119322	0.044507631	0.041133274
Dhx35	1	1.49E-09	5E-28	-0.002041429	0.038346724	0.041085672
Dguok	0.000198732	9.23E-04	4E-18	0.02033915	0.033378488	0.041078275
Lrrc58	2.5842E-121	1.00E+00	0.0146	-0.138304278	0.043221205	0.041077009
Arv1	1	1.44E-04	1E-32	0.005029209	0.025629227	0.041076821
Urm1	1	7.55E-06	3E-40	0.005563555	0.023397217	0.041074391
Mterf3	1	1.21E-12	2E-21	0.00943121	0.04919768	0.041066385
Prpf38a	1	7.30E-03	1E-51	0.002013306	0.017363733	0.041050819
Otud7b	2.09544E-12	7.85E-02	7E-05	0.049119395	-0.032496931	0.041042992
Slc45a4	1	9.19E-19	2E-46	-0.001877647	0.03769501	0.041019401
Card6	1	1.00E+00	6E-42	0.001917772	0.014113417	0.04101843
Nelfa	2.03652E-13	4.26E-02	4E-19	0.030486992	0.028932474	0.040988836
Fbxo21	1.04232E-35	1.00E+00	3E-05	0.073393102	0.043999487	0.040985707
Smyd4	1	2.69E-38	1E-48	-0.004960802	0.051571025	0.040975453
Gm13165	1	8.95E-15	5E-63	-0.004832987	0.025715242	0.040961709
Rcbtb1	0.003103177	1.00E+00	2E-08	0.02400741	0.005058263	0.040953453
Glrx5	1	3.32E-12	2E-32	-0.000191862	0.037617627	0.040940303
Map2k3os	1	4.11E-38	2E-80	0.001545969	0.035431778	0.040925148
Gm40916	0.000420765	1.00E+00	2E-41	-0.011283527	-0.005052054	0.040916581
Fry	1	2.58E-33	6E-58	0.001992426	0.046293517	0.040888671
Ube2v2	1	1.00E+00	1E-12	-0.006610437	0.010797803	0.04088591
Asf1a	1	6.61E-24	2E-62	0.00066195	0.033010443	0.040872369
Ankrd10	1	1.00E+00	5E-20	0.007775258	0.01983904	0.040872095
Ppp1r3c	1.31033E-35	1.00E+00	5E-11	-0.048943597	0.009361707	0.040850705
Nup98	0.015748146	1.00E+00	2E-07	-0.022147717	0.006857397	0.040824188
Kif5c	1	4.75E-75	1E-156	3.75721E-05	0.021668182	0.040819806
Zmat5	1	4.35E-08	5E-40	-0.005708272	0.026963242	0.040784693
Tab1	1	4.56E-12	9E-32	-0.003492588	0.037618204	0.040744951
Mrpl33	1	3.19E-04	1E-28	0.00270207	0.029401957	0.040744388
Dchs1	1	5.50E-105	6E-166	-0.000161579	0.029096127	0.040733436
Zgrf1	1	1.02E-14	2E-74	-0.001454914	0.023428807	0.040723994
Cnot10	1	1.00E+00	3E-09	0.011413222	-0.012374278	0.040722947
Mtrf1l	1	1.59E-05	3E-33	0.001626714	0.02503116	0.04069436
Gm49719	4.21738E-11	1.37E-20	2E-70	0.013486868	0.027823505	0.040674725

Agpat5	1	1.32E-23	2E-61	-0.00235402	0.038559896	0.040651635
Epha2	1	5.97E-02	2E-16	0.000392321	-0.020873734	0.040638635
Abcf3	1.66025E-07	1.00E+00	3E-23	0.020219328	0.000779301	0.040614967
Poli	1	7.72E-23	3E-50	-0.006933536	0.03579107	0.040614746
Mrpl42	1	7.82E-01	3E-37	0.007738665	0.018101631	0.040584906
Scyl3	0.473496566	1.00E+00	2E-13	0.01692635	-0.020745579	0.040565312
Gm15545	1	3.63E-22	7E-44	0.003873468	0.040337573	0.04055336
Inpp5k	1	2.88E-01	1E-18	0.000394565	0.029567545	0.040552999
Gm48678	1.78809E-06	3.65E-45	6E-32	0.016013824	0.070182784	0.040509298
Nol6	5.68674E-05	4.62E-18	1E-82	0.008587725	0.023091591	0.040492886
Msto1	1	1.36E-38	2E-95	0.002376127	0.031602516	0.040415254
Ttc39aos1	1	3.15E-151	8E-176	0.00020466	0.036474344	0.040412311
Corin	1	1.28E-75	3E-84	-0.002280204	0.045013437	0.040373072
Cmpk1	1	2.73E-02	1E-17	-0.004095165	0.030795062	0.04037213
Vps28	1	1.72E-14	2E-77	0.001767549	0.022822663	0.040352724
Rnf39	1	4.85E-32	2E-49	0.003717786	0.043136195	0.040347933
Cnksr1	1	2.33E-167	1E-180	3.84256E-05	0.037773209	0.040331798
Zcchc4	1	1.00E+00	5E-18	-0.012630596	0.004978649	0.040324484
Scaper	7.00189E-25	4.01E-11	0.0005	-0.058786061	-0.058591084	0.04031973
Krt8	1.68533E-13	3.92E-17	2E-21	-0.017176366	0.045878549	0.040311588
Gm44899	1	5.73E-04	3E-26	-0.008027435	0.029017916	0.040311138
Ints8	1	1.00E+00	2E-11	0.003440117	0.021023534	0.040272655
Crls1	1	1.00E+00	6E-17	0.004910053	0.012369135	0.040254963
5730455P1	1	1.18E-05	1E-28	0.006675097	0.029125387	0.040251113
Trabd	0.63675626	4.14E-02	7E-11	0.018105321	-0.02394131	0.040234675
Gm17189	1	5.35E-230	2E-180	0	0.05272831	0.040160517
Rbm22	1	3.04E-11	6E-38	0.008149706	0.031914924	0.040153882
2410002F2	0.528434092	4.60E-01	1E-34	0.00958779	0.018508157	0.040123321
Rpl35a	1	2.79E-18	3E-27	-0.004978323	0.05283421	0.040104288
Rbm17	1	1.00E+00	5E-23	0.008298769	0.021947796	0.040104203
Gm7854	1	6.25E-30	4E-23	-0.000339894	0.064470559	0.040077684
Eif3k	1	8.73E-08	2E-35	0.009476923	0.029832683	0.040059224
Clpb	1	1.00E+00	1E-12	0.009706524	-0.007102449	0.040052621
Pus1	1	4.71E-28	4E-49	0.004598786	0.040833889	0.0400343
Itga9	1	3.12E-226	2E-58	0.004046075	0.181330923	0.040009917
Zfp994	1	7.69E-01	1E-36	-0.00404154	0.01778077	0.039994267
Riox2	1	1.00E+00	3E-34	0.000164225	0.014188848	0.039926325
Rusc2	1.81189E-05	1.00E+00	1	-0.033894351	-0.003645044	0.039922109
Plxna1	1	1.39E-37	1E-46	-0.003269223	0.06810325	0.039881401
Rbm27	0.000149076	1.00E+00	4E-10	0.024847161	0.010266631	0.039860067
Zfp251	1	7.50E-24	3E-67	-0.00274327	0.03200798	0.039856484
Clcn5	0.017526019	1.54E-02	2E-16	-0.014991055	0.035057903	0.039817631
Adsl	1	4.77E-07	4E-41	0.001722346	0.024900413	0.039804918
Top2a	1	9.93E-43	5E-111	-0.001804277	0.023125587	0.039804072
Vamp1	1	8.01E-19	2E-48	0.000673897	0.034922349	0.039801649
Gm4890	1	5.21E-76	7E-59	-0.002149983	0.061743966	0.039769495
Gm38304	1	5.36E-08	5E-56	-0.005291665	0.020992711	0.039761378

Tmc6	0.122580146	2.19E-31	3E-61	-0.006200058	0.038480211	0.039751298
Polr2e	0.928456248	5.91E-23	5E-77	0.005729268	0.028106235	0.039746976
Tmtc3	1	2.23E-11	7E-31	0.003296243	0.037008808	0.039731624
Dhx9	1	1.00E+00	3E-07	0.004453402	0.028130349	0.0397147
Fam160b2	1	2.87E-03	2E-33	-0.000810419	0.022821722	0.039705865
Gm28055	4.57304E-14	5.54E-05	9E-18	-0.026920859	0.032874422	0.039693508
Appl1	1.46381E-09	1.00E+00	1E-15	-0.023828118	0.007858787	0.039665829
Sri	1	4.27E-16	4E-39	-0.00522614	0.03533364	0.039655678
Emg1	1	2.66E-16	5E-37	0.008144223	0.037882068	0.039607624
Crybb3	1	8.42E-43	2E-122	-0.001282099	0.022903595	0.03956069
Katna1	1	6.88E-01	7E-21	-0.001076793	0.024100282	0.039555518
Mrps9	1	1.00E+00	9E-11	-0.014030011	0.006291062	0.039548257
Add3	1	3.25E-01	5E-18	-0.002262629	0.032150259	0.039537499
Ttc4	1	1.00E+00	1E-17	0.009435357	0.013322128	0.039533954
Cops8	0.152434695	4.73E-07	4E-36	0.010595153	0.028579676	0.039461749
Wdr93	1	2.65E-13	1E-23	-0.004851102	0.04240623	0.03942137
Tmem199	0.375592338	1.57E-18	2E-56	0.008001369	0.030776524	0.03941934
Stx4a	1	1.00E+00	8E-21	0.007187694	0.016418479	0.039413782
Ttll7	1	2.66E-37	2E-126	0.002947571	0.020149248	0.039406139
Mtmr7	1	2.34E-31	4E-55	-0.001242618	0.039722922	0.03940022
Ubxn2b	1	3.40E-10	2E-48	-0.001086009	0.025763126	0.039396689
Gpn1	1	1.14E-02	2E-43	0.003308376	0.01803537	0.039368533
Dph3	1	3.10E-35	2E-70	0.000626238	0.03606197	0.039366503
Fam3c	1	4.20E-11	4E-34	-0.006583105	0.036162409	0.039353587
Zfp872	1	3.88E-83	6E-120	-0.002078064	0.037985856	0.039352515
Emilin2	1	7.80E-192	2E-147	0.000330053	0.059109415	0.039321571
6820408C1	1	1.17E-73	3E-168	1.61161E-05	0.019000937	0.03931754
Ncoa7	1	1.57E-127	7E-112	0.002110289	0.060720048	0.039311809
Casz1	1	7.19E-49	4E-107	-0.000679194	0.02954253	0.039299792
Ankmy2	1	1.00E+00	3E-37	-0.000309065	0.01363875	0.039280433
Ranbp1	0.009305517	1.59E-15	2E-56	0.00884483	0.027768913	0.039277461
Fkbp11	1	2.74E-05	1E-96	-0.002735059	0.011229751	0.039266149
Guca1b	1	1.11E-43	1E-90	-0.001566958	0.032207582	0.039264109
Zfp341	0.000779135	1.92E-07	8E-37	0.012881023	0.027059303	0.03925713
Abcb8	1	1.00E+00	8E-27	0.009917873	0.004898163	0.039252239
Pcgf6	1	1.00E+00	1E-22	0.011051185	0.014427363	0.039244881
Snhg16	1	2.01E-67	9E-76	0.002903156	0.047597972	0.039215578
Bin3	0.391848045	2.55E-01	5E-18	0.015013794	0.032043748	0.039203781
Catspere2	1	1.00E+00	6E-15	-0.001553729	0.016912614	0.039174845
Armc10	1	3.73E-15	1E-53	0.001524043	0.028945235	0.039147608
Fam57a	1	6.51E-15	5E-36	0.001891224	0.035066894	0.039129879
Gm14164	1	7.25E-166	7E-119	-0.000758429	0.061018294	0.039069881
Surf2	0.001825892	4.08E-04	8E-62	0.008905433	0.016338307	0.03904677
Ash2l	1	5.98E-04	8E-23	8.05909E-05	0.029415742	0.039005681
Scrn3	1	1.54E-03	4E-07	0.015689561	-0.034643921	0.039001132
Aatf	1	1.00E+00	2E-05	0.004197169	0.02656259	0.038981266
Hdac10	1	4.60E-05	3E-53	-0.000105116	0.018743737	0.038954113

Arhgap15	1	1.98E-185	2E-43	0.000909225	0.154056455	0.038948154
Eif4h	1	1.00E+00	7E-12	0.000480986	0.022113168	0.038932635
Pcnx3	0.062057303	4.89E-04	7E-35	0.01068712	0.022746113	0.038930095
Pfas	1	1.00E+00	2E-34	-0.006454218	0.007949771	0.038916799
Fbxl6	1.05307E-10	5.95E-04	2E-41	0.017093639	0.020566727	0.038905517
Akr1b7	1	4.89E-36	4E-161	0.000932917	0.010366695	0.038880989
Pik3c2a	6.98909E-17	1.35E-12	1E-06	0.052858516	0.079581657	0.038880197
Tmem68	1	2.46E-05	9E-30	0.000826295	0.027518193	0.038866803
Mrvi1	1	9.60E-109	5E-110	-0.002264382	0.046893385	0.038825391
Odf2l	1	1.00E+00	7E-15	-0.011732313	0.010167268	0.038792398
Malsu1	1	1.00E+00	5E-21	0.002060456	0.018111671	0.038785677
Gm26725	1.60875E-13	6.34E-15	3E-43	-0.0152164	0.031701649	0.038777869
Ndufaf7	1	1.42E-10	1E-66	0.003543896	0.020254789	0.038764341
Arsk	1	1.00E+00	3E-22	-0.007097152	0.014987819	0.038738936
Grsf1	1	1.74E-02	8E-20	0.009690769	0.026448206	0.038733388
Arhgap11a	1	2.91E-41	6E-112	-0.002765261	0.026652418	0.038729254
Ccdc148	1	2.44E-95	3E-64	0.002266727	0.085450877	0.038693283
Ripor1	1	2.46E-05	5E-41	0.000116107	0.023215296	0.038692587
Uxs1	1	1.00E+00	5E-17	-0.00369335	0.020372671	0.038676659
Polr2l	1	3.00E-60	3E-96	0.002350988	0.038588876	0.038622931
Zcchc2	1.4912E-09	1.00E+00	1E-05	-0.033665457	0.040085802	0.038546821
Phykpl	1	3.06E-06	5E-17	0.002785569	0.038615631	0.03854361
Ccdc34	1	5.57E-48	1E-84	-0.00290069	0.036379609	0.03854123
Osbpl2	1	1.00E+00	7E-14	0.002275793	0.021067139	0.038525083
Ajuba	1	5.37E-39	5E-109	0.000799413	0.025067488	0.038521599
Col4a4	1	2.61E-64	3E-110	0.002776998	0.03238582	0.038520546
Zfp120	1	1.00E+00	9E-35	0.005988047	0.008895871	0.038520489
Sars2	0.000986969	2.91E-03	4E-45	0.01130387	0.016917078	0.038517612
Cactin	1	1.80E-09	4E-49	0.006568168	0.023972505	0.038508102
Sesn2	1	1.00E+00	5E-25	0.003747752	0.017192375	0.038506541
Srek1ip1	1	1.00E+00	1E-19	0.002046742	0.007081633	0.038496265
Ifi207	1	5.07E-281	2E-84	0.001247551	0.144409752	0.038472527
Med17	1	1.00E+00	8E-16	0.008022152	0.020839841	0.038460021
Myo1c	1	1.18E-45	7E-50	-0.001358733	0.056598483	0.038448559
Zfp788	1	9.26E-27	4E-72	-0.000886058	0.029991864	0.038419018
Llgl2	1	1.00E+00	4E-15	-0.002250153	0.015456227	0.038401158
Gm47695	1	3.16E-223	2E-164	-0.000220631	0.052711043	0.038391345
Manbal	1	3.97E-09	5E-34	0.003362809	0.030903786	0.038389909
Spg7	0.017903839	1.00E+00	3E-16	0.016393948	0.024292147	0.038328179
Fbl	1	5.91E-17	6E-36	0.003678225	0.038806859	0.038295642
3010003L2	1	9.26E-93	7E-137	4.59573E-05	0.03300267	0.038282042
Serinc1	1	1.00E+00	1E-15	0.000521535	0.011737959	0.038260326
BC004004	1	1.00E+00	3E-08	0.006014203	-0.017489842	0.038257449
Ttll4	1	1.00E+00	9E-12	0.016369289	0.00558808	0.038251864
Wbp11	1	1.00E+00	1E-17	-0.003592054	0.020878053	0.038249879
Pla2g6	1	2.64E-01	5E-18	0.006830684	0.024549366	0.038246372
Atp6v0a1	1	8.44E-01	4E-08	0.005646866	0.061529519	0.038205006

BC016579	0.839558737	6.53E-101	4E-141	-0.001675808	0.033742053	0.038200271
Bbs7	1	6.74E-01	8E-28	-0.0061879	0.018684562	0.038194298
Fnbp4	7.91467E-23	1.92E-04	4E-05	0.057290249	-0.036742433	0.038190885
Atg16l1	0.01738505	2.18E-01	4E-06	0.026165424	-0.025746625	0.038171813
Anxa9	1	2.90E-77	8E-120	0.000308226	0.034949003	0.038150528
Pigt	1	9.35E-10	6E-36	0.001227327	0.030989033	0.038112281
Taf10	1	4.79E-22	1E-66	0.002437639	0.028482615	0.038090726
Cbwd1	1	1.00E+00	1E-21	-0.009156878	0.008292516	0.038079503
Zfp386	1	4.72E-09	5E-44	-0.004017622	0.02444179	0.038066163
Baz2a	0.059873647	4.23E-04	0.0001	-0.022513945	-0.035471149	0.038060533
Wdhd1	1	1.43E-16	2E-59	-0.002333816	0.028759035	0.038052409
G6pdx	1	1.80E-140	5E-89	3.06377E-05	0.069141736	0.038046929
Spata1	1	2.97E-04	3E-11	-0.009132353	0.041747535	0.038033577
Alg5	0.587666681	1.60E-17	8E-40	-0.00782431	0.035857575	0.038026019
Krtcap2	1	3.74E-24	2E-79	0.00265361	0.027156627	0.037996271
Acot6	1	5.29E-50	5E-138	0.000685071	0.021866881	0.037944481
Cdk2ap1	1	4.14E-22	2E-60	-0.002600348	0.030668261	0.037930305
Dedd2	0.209459409	2.77E-06	6E-23	-0.010758162	0.034532156	0.037922448
Nadsyn1	1	1.00E+00	2E-16	0.00983194	0.019071394	0.037917721
Tgm2	1	1.90E-17	3E-23	-0.005776461	0.048615412	0.037892419
Gm7160	1.27603E-28	2.57E-25	8E-15	-0.035805626	0.073656082	0.037892413
Vmn1r184	1	1.60E-194	5E-166	0	0.045391924	0.037875151
Zc3h4	1	4.50E-19	1E-11	0.003478647	0.071236927	0.037869505
Ccne2	1	2.11E-41	5E-88	6.10069E-05	0.029372115	0.037864959
Rbm3	1	1.00E+00	3E-28	-0.001747918	0.014827285	0.037859921
Cd9	2.81768E-22	1.93E-51	5E-20	-0.023769846	0.096326144	0.037850613
Anxa5	0.00053619	1.33E-06	5E-32	-0.01108207	0.027935517	0.037847084
Ccdc14	1	4.74E-40	1E-66	-0.004842238	0.03775335	0.037838082
Srp54c	0.316358764	6.96E-06	4E-46	0.008027892	0.021053029	0.037787241
Camk1	1	4.00E-33	5E-62	-0.002088362	0.038845703	0.037786068
Dnah7a	1	1.10E-40	7E-45	-0.005342528	0.052431417	0.037783684
Dnajc17	1	1.00E+00	2E-39	0.002910979	0.014467472	0.037760811
Dmac2l	1	6.67E-15	1E-41	-0.001037295	0.031939695	0.037739491
1810055G0	1	1.54E-10	2E-09	0.00811303	-0.043379397	0.037724994
Epo	2.09625E-29	1.00E+00	1E-10	0.053690396	0.019788202	0.037712687
Ndufb1-ps	1	7.35E-06	2E-16	-0.004578206	0.042233191	0.037698696
Timm21	1	9.88E-06	3E-46	0.004284177	0.020912849	0.037695566
Bbip1	1	7.13E-15	1E-24	-0.005407891	0.043777121	0.03769131
Gnl2	1.13993E-15	1.93E-01	5E-12	0.034286889	-0.022972482	0.03763046
Spindoc	1	1.00E+00	7E-29	-0.004924432	0.007983222	0.037624607
Wdr48	1	1.00E+00	5E-17	0.001663143	0.017616482	0.037623901
Pygb	1	1.24E-56	2E-108	0.002063533	0.030851442	0.037621673
Oxa1l	1	2.40E-08	9E-20	0.010657229	0.036429322	0.037606457
Atp5e	0.158545247	1.85E-07	2E-31	-0.009858964	0.032530109	0.037577114
Arhgdia	1	2.93E-56	4E-58	0.001862957	0.053124001	0.037567458
Aoc2	1	2.79E-52	1E-104	0.0022798	0.030290528	0.037565786
Zfp729a	1	1.00E+00	4E-27	0.001929179	0.010917417	0.037553575

Rnd3	1.03311E-19	1.47E-01	3E-11	-0.030463066	0.033077949	0.037547431
H3f3b	1	1.00E+00	6E-12	-0.00576658	0.017802131	0.037546397
Man1b1	1	4.63E-02	3E-20	0.00698042	0.027688082	0.037492098
Rarg	1	2.03E-31	2E-95	-0.003299814	0.024925929	0.037471714
Zfp451	1	1.00E+00	6E-08	0.003617605	0.020590674	0.037453861
Mrps15	1	1.00E+00	7E-36	0.000488598	0.016726366	0.037432862
Zer1	1	5.18E-03	1E-22	0.003465471	0.024144072	0.037418361
Bub3	1	6.09E-08	5E-33	0.002977021	0.029314044	0.037413881
Vangl1	0.000114906	1.00E+00	5E-39	-0.010804688	0.010309087	0.037389958
Gm31323	1	5.20E-65	1E-68	0.004690981	0.048528479	0.037364017
Dbnl	1	7.78E-13	3E-34	0.001295302	0.035823223	0.03734804
Rtel1	1	7.54E-15	2E-40	-0.000535529	0.032516206	0.037342714
Map3k7	1	1.00E+00	2E-07	-0.001654971	-0.002137608	0.037313524
A2ml1	1	7.06E-55	2E-129	0.000478752	0.024640792	0.037295112
Rpl7l1	1	1.00E+00	5E-20	0.003869648	0.01469931	0.037240891
Nr1h2	1.9773E-05	1.49E-01	1E-27	0.015629009	0.021163941	0.03723537
G3bp1	1	1.00E+00	3E-06	0.006230772	0.006955689	0.037223264
Hook1	1	3.23E-08	3E-10	0.00524327	-0.043365853	0.037221447
Srrm1	4.41877E-16	1.00E+00	0.0003	0.049977031	-0.006120473	0.03721694
Eid1	1	1.34E-46	3E-145	0.001562279	0.018671573	0.037197596
Itm2b	1	7.19E-05	0.0004	-0.001797378	0.068609199	0.037197041
Pdhx	1	1.00E+00	1E-14	-0.003331706	-0.006809448	0.03717806
Arhgef2	1	4.71E-229	1E-131	-0.000505099	0.079115371	0.037158127
Zmym6	1.86533E-15	2.58E-07	2E-15	-0.024939131	0.044025157	0.037121729
Rmc1	2.17328E-17	6.46E-03	2E-29	0.023659087	0.02235406	0.037097683
Acox3	1	1.00E+00	2E-22	-0.009553832	0.020230792	0.03709464
Nit1	1	1.00E+00	1E-09	0.009293182	-0.016101315	0.037073436
Haus8	1	8.79E-75	5E-65	-0.000492077	0.060140718	0.03704357
Lrba	1.37721E-06	2.68E-01	0.0475	-0.042939356	0.056802838	0.037042512
Ubxn1	1	2.37E-02	1E-10	0.00055987	0.035135503	0.037034273
Fam104a	4.26739E-06	1.00E+00	3E-20	0.018373459	0.021764324	0.03700106
Slc36a4	1.059E-07	6.71E-24	1E-57	-0.009522043	0.033617315	0.036994346
Atxn1l	1	3.71E-01	3E-35	-0.002343	0.015831036	0.03697599
Tepsin	1	2.38E-05	2E-41	0.005510681	0.022001917	0.036958719
Idh3g	0.010558704	1.00E+00	9E-22	0.014363686	-0.002416448	0.036931071
Gm4107	1	5.37E-37	1E-82	-0.002408815	0.031301692	0.036926063
Thap12	1	1.62E-03	6E-24	-0.008637932	0.025287334	0.036918605
Chchd1	1	9.88E-02	3E-40	0.003058963	0.016721104	0.036918547
Dhrs9	1	1.97E-15	2E-43	-0.004120594	0.031050541	0.036910899
Scand1	1	4.32E-22	4E-57	-0.000938047	0.031641514	0.036891186
Asah1	1	3.51E-12	4E-25	0.002971998	0.044210967	0.036891043
Alg1	1	4.93E-12	8E-49	0.006870637	0.024924849	0.036887915
Gtf2h5	1	1.21E-10	2E-34	-0.002216361	0.030813456	0.036827707
Mtfmt	1	1.00E+00	4E-32	-0.002657005	0.012468559	0.036825256
Nup50	1	1.00E+00	1E-24	-0.002775158	0.020308919	0.036800105
Ppp4c	1	6.29E-01	1E-14	0.002622678	0.026908835	0.036761452
Pfdn4	1	2.37E-18	3E-68	0.005487587	0.02428464	0.036727121

Zfp512b	1	1.00E+00	7E-34	-0.003914519	0.011962414	0.036716795
Ippk	1	1.00E+00	2E-27	0.004485683	0.012770334	0.036704781
Rbak	1	4.37E-14	9E-43	0.001094926	0.03001702	0.036673912
2610020C0	0.007438811	2.39E-38	1E-21	0.014046601	0.06829556	0.0366567
Rnf181	0.001715388	1.00E+00	3E-22	0.015087459	0.015216008	0.036654028
Kif20b	1	4.37E-27	1E-70	-0.001866077	0.027290679	0.036633949
Syt12	1	4.10E-51	8E-91	-0.001519213	0.033359147	0.036622472
Pdzk1	1	2.02E-03	3E-08	-0.014015556	-0.027727359	0.036611082
Lrp10	1	1.45E-04	2E-37	0.008047524	0.022795804	0.036608527
Ppp1cc	1	6.23E-03	5E-14	0.012897136	0.03573703	0.036605289
Stk4	0.020382725	2.34E-04	9E-11	-0.01729277	0.050794135	0.036588896
Entpd6	1	1.23E-17	1E-48	-0.003854738	0.028851565	0.036557211
Abhd17a	1	3.03E-17	4E-46	-0.000801357	0.030717132	0.036542332
Isy1	1	1.00E+00	9E-23	0.007898595	0.014772798	0.036514243
Nxf1	6.64052E-12	1.01E-05	3E-10	0.034421334	-0.032800738	0.036505568
Nifk	1	5.05E-15	5E-40	0.002150101	0.031509904	0.036492302
Pcbp2	1	1.00E+00	3E-05	-0.008978329	0.041154317	0.036492029
Tpx2	1	1.66E-13	3E-91	-0.002175353	0.015094179	0.036458383
Strip1	1	1.00E+00	3E-26	0.010744287	0.018180333	0.03643814
Rnf166	1	2.67E-01	4E-28	0.000634619	0.019942468	0.036435248
Nrbf2	1	1.00E+00	3E-10	0.010900628	0.010169139	0.03642381
Kdm4b	1	1.00E+00	5E-10	-0.001376164	0.002309258	0.03639656
Nrde2	1	1.00E+00	1E-25	0.008913509	0.011234198	0.036394986
Aftph	1	1.00E+00	0.002	0.009132855	0.03473275	0.036394709
Slc4a7	1	1.00E+00	2E-10	-0.003399051	0.022536998	0.036391703
C330018D2	1	2.78E-13	1E-29	-0.005177126	0.03584346	0.036385266
Timm8b	1	7.77E-33	4E-75	0.001972614	0.029450021	0.036353032
Lamtor3	1	4.72E-06	2E-25	0.002707423	0.031160239	0.036338912
Hsd3b7	6.30634E-53	1.00E+00	0.0002	0.090329664	-0.015129666	0.036323799
Galns	1	2.54E-26	1E-43	-0.005300346	0.041670801	0.036296284
Pi4k2b	1	1.00E+00	4E-14	-0.00668265	-0.003922673	0.036284686
Areg	1	1.05E-110	1E-123	0.000196637	0.033852813	0.036263278
Pus3	1	1.57E-12	3E-60	0.001473499	0.023033058	0.0362575
Ctns	1	1.00E+00	8E-18	-0.003040158	0.02130048	0.03625462
Thumpd2	1	9.94E-07	6E-34	0.00165443	0.027326952	0.036250313
Tusc2	1	1.29E-29	4E-59	0.003395629	0.034406734	0.036201633
Acsf3	1	1.00E+00	1E-33	-0.005642336	0.015007802	0.036177215
Gm26588	1	2.06E-36	1E-41	0.005289688	0.047414843	0.036175162
Klf3	1.19976E-93	2.67E-01	0.9983	-0.120408192	0.053099294	0.036173006
Ifi27l2b	1	2.09E-41	4E-138	0.001096216	0.016218192	0.036159156
Phka2	0.000658008	1.00E+00	2E-06	-0.022615151	-0.011743295	0.036115604
Ankrd24	1	4.70E-18	5E-32	0.007728011	0.068919004	0.03611127
Cul4b	1	1.00E+00	4E-17	0.004060331	0.016902552	0.036105182
Pvr	1	4.96E-18	1E-46	0.000316277	0.030253811	0.036093858
Zfp174	1	5.63E-12	4E-35	0.002025275	0.029852957	0.036088271
Nedd9	1	6.07E-115	3E-52	0.00214163	0.09631239	0.036070145
Timmdc1	1	1.00E+00	8E-13	0.007356291	0.013655355	0.036054623

Actr1b	1	1.00E+00	2E-26	0.006594784	0.009445336	0.036044017
Fam49b	1	7.01E-04	3E-05	-0.003294055	0.086149404	0.036037348
Trpv1	1	2.12E-23	2E-126	-0.001144164	0.013438903	0.036009692
Phf5a	1	2.47E-09	3E-39	0.005364421	0.024931524	0.035996914
Cdipt	1	4.05E-19	2E-55	0.005242834	0.028720342	0.03598282
Ccdc86	1	5.67E-18	3E-48	0.004429441	0.029755289	0.035962469
Btc	0.306578306	1.05E-15	2E-38	-0.005562425	0.030691496	0.035917091
Zfp518a	1	1.00E+00	3E-36	-0.003475836	0.006464075	0.035916353
Slc4a2	1	1.00E+00	2E-19	0.002730145	0.016173285	0.035896069
Specc1	1	2.74E-39	8E-34	0.00649016	0.068864208	0.035879271
Gcc1	1	4.91E-14	1E-36	-0.003041302	0.0304075	0.035853639
Abcd4	0.00123051	1.16E-02	1E-10	0.019748786	-0.026663391	0.035851529
Snx11	1	5.12E-05	7E-37	-0.002500832	0.023135642	0.035836671
2900076A0	1	1.00E+00	1E-25	0.005023433	0.008079126	0.03581104
Mllt11	1	7.76E-36	7E-70	0.000140698	0.032813541	0.035789979
Mrpl43	1	6.02E-08	4E-43	0.007616778	0.022661114	0.035780269
Itgb2l	1	1.34E-46	2E-84	0.00050405	0.032263117	0.035761618
Manf	5.86252E-18	1.00E+00	2E-16	0.031357406	0.017867037	0.035743397
Npm3	1	6.32E-05	2E-48	-0.00157593	0.018340352	0.035741533
Eaf1	1	3.59E-06	6E-32	-0.002568794	0.025333913	0.035729006
Oma1	1.16385E-15	1.00E+00	1E-09	-0.033533255	-0.003422137	0.035718986
Gab2	1	2.33E-126	0.0006	-0.007848844	0.256102114	0.035687181
Dctn4	1.93234E-07	1.00E+00	1E-09	0.02755307	0.020443069	0.035676686
Dnajc5	1	2.22E-01	0.0006	0.009975651	-0.023879152	0.035673582
Ica1	1	1.00E+00	9E-47	-0.001200359	0.013615374	0.035670564
Gm13528	1	2.65E-52	7E-108	0.000129414	0.0271178	0.035668283
Creb3	0.000131672	1.93E-01	1E-41	0.011222009	0.014295827	0.035648352
Tspyl2	1	1.00E+00	4E-41	0.005205373	0.00368583	0.035634825
Mrpl21	1	1.00E+00	4E-22	0.002940878	0.002132709	0.035616393
Plscr2	7.23065E-07	1.00E+00	3E-19	0.019072344	-0.003234856	0.035608161
Fancm	1	1.00E+00	6E-17	-0.003607359	0.017669609	0.035540768
Gemin2	0.003970206	3.81E-03	2E-14	0.018153139	0.031695055	0.035470114
Phf11c	1	6.21E-10	1	-0.009287689	-0.03802371	0.035445111
Smim1	1	5.99E-06	9E-81	0.000959083	0.012409642	0.035432735
Arpc1b	0.368462537	3.98E-25	3E-27	-0.008221239	0.057190086	0.035428107
Ankrd52	1	2.82E-10	2E-14	-0.005340327	0.046820391	0.035426244
2510009E0	1	8.73E-24	5E-39	-0.002746257	0.053049997	0.03542091
Lsm6	1	1.65E-18	6E-38	-0.001299815	0.034517856	0.035417429
Capn3	0.006851068	3.45E-38	4E-49	-0.007088051	0.043703678	0.035406459
Sec22b	0.203612059	1.00E+00	1E-11	0.016731538	-0.017915151	0.035402057
Surf6	1	8.35E-07	9E-28	0.004618063	0.027086081	0.035380812
Rab31	1	1.07E-175	6E-65	-0.001286594	0.105673604	0.035377444
B930025P0	1	7.50E-21	6E-23	-0.003694553	0.047751819	0.035363828
Prg4	1	7.54E-16	5E-51	0.007270007	0.026926513	0.035359137
Ctc1	1	4.13E-03	2E-35	-0.004881011	0.018201456	0.035358444
B930036N1	1	7.33E-123	1E-118	-0.000340427	0.042970353	0.035352556
Bag1	1	8.64E-06	3E-27	0.002307757	0.027488589	0.035343384

Hax1	1	1.00E+00	2E-23	0.006792271	0.009417921	0.035342704
Rabl3	1	1.00E+00	8E-16	-0.001443477	-0.006897558	0.035342529
Gatb	1	1.00E+00	4E-09	-0.007587746	0.027260969	0.035339941
Hcfc1	1	1.00E+00	8E-21	0.001379277	0.006064673	0.03533941
Cuta	1	3.73E-09	5E-52	0.004055288	0.022394678	0.035330801
Atxn7	1	7.50E-11	0.0288	-0.007783985	0.08461205	0.035325884
Sumf2	1	1.00E+00	4E-17	0.003432348	0.016726511	0.035318741
Ncapg2	1	2.95E-54	1E-98	-0.001017702	0.029381794	0.035310784
Ap1m2	1	1.37E-162	8E-157	-4.73494E-05	0.037231371	0.035296677
Csgalnact2	1	3.88E-17	5E-33	0.00511431	0.038107512	0.035271502
Ubr7	1	2.22E-09	6E-27	0.004720588	0.032315644	0.03523053
Lrrc41	1	1.00E+00	7E-10	-0.00796953	0.010748831	0.03521148
Lmnb2	1	6.58E-27	2E-62	0.004993133	0.029266238	0.03520588
Pkn1	1	8.15E-47	1E-14	-0.003803712	0.100000826	0.035201006
Polr1b	1	1.30E-09	4E-25	0.006086145	0.032647543	0.0351948
Elp6	1	6.98E-03	1E-31	0.001088234	0.018743292	0.035176585
Intu	1	2.92E-08	2E-50	-0.001349364	0.021723211	0.03516953
C1qbp	1	1.15E-38	1E-54	-0.002528274	0.040099792	0.035137853
Gm50314	1.53294E-20	1.64E-02	5E-37	-0.014830013	0.018696608	0.035130162
Wdr73	1	1.00E+00	2E-17	0.003832561	0.019493513	0.035123129
Ube3c	1	1.00E+00	0.0122	-0.004134666	0.01148567	0.035121745
Zfp267	1	1.41E-01	5E-36	0.000406339	0.016679847	0.035103594
Gm6712	1	1.56E-02	4E-13	-0.000610898	-0.023407776	0.035079691
Pms2	1	3.58E-20	1E-65	-0.002041555	0.024790327	0.035058903
Atp8a2	1	4.49E-62	5E-79	-0.000468896	0.039705459	0.035040266
Gna11	1	1.00E+00	7E-22	-0.008266286	0.005283977	0.035030225
Trmt44	1	4.49E-08	6E-47	-0.001915541	0.020268589	0.035021671
Puf60	0.016093031	1.48E-01	9E-23	0.013928158	0.021277162	0.035016982
Urod	1	2.12E-03	3E-59	0.003006392	0.013159613	0.035016353
Pitpnb	1	1.54E-03	3E-07	0.01442857	-0.030238966	0.034998987
Hsf1	0.391954234	1.00E+00	1E-15	0.013148038	0.007762831	0.034988325
Cdk5rap3	3.8453E-09	1.00E+00	5E-26	0.018087963	-0.006620501	0.034958443
Armt1	1	1.00E+00	5E-19	-0.002618097	0.015199422	0.034940949
Rpp30	1	4.77E-12	6E-41	0.006470869	0.027151511	0.034915567
Gm42375	1	4.77E-241	1E-138	0.000244085	0.063759639	0.034911778
Prkab1	1	7.11E-06	2E-31	0.006756368	0.023699692	0.03490426
Ccdc130	1	4.07E-06	6E-39	-0.00148647	0.020550111	0.03490403
Gm9725	3.47857E-13	1.00E+00	1E-21	-0.019701311	0.009874012	0.034898376
Flywch1	1	1.00E+00	7E-15	0.001213591	-0.008345741	0.034884321
Kif5b	2.96077E-05	1.00E+00	0.0001	0.031869961	0.000612186	0.034870282
Tmx4	4.72617E-16	1.00E+00	1E-09	-0.02696064	-0.005752228	0.03485001
Slc6a8	1	7.84E-68	3E-97	-0.000898718	0.035834865	0.034845789
Tubgcp6	1	1.00E+00	4E-18	0.002145324	0.014302033	0.034819782
Zfp971	1	1.00E+00	6E-31	-5.35449E-05	0.009529007	0.034816704
Rad1	1	2.98E-17	5E-63	0.002528471	0.023429349	0.034803618
Champ1	1	3.99E-06	3E-36	-0.003553934	0.021560113	0.034800309
Trmt10a	1	1.69E-06	1E-30	-0.001111634	0.026026748	0.034779455

Mrps31	1	1.00E+00	3E-21	-0.001926679	0.001188627	0.034773103
Pgam1	1	2.68E-18	3E-40	-0.000567904	0.034656305	0.03477239
Zfp511	0.451170522	2.96E-06	1E-38	0.008159982	0.02076936	0.034767195
Alg12	0.142549858	1.00E+00	3E-28	0.011059483	0.005439242	0.034756805
Rfc2	1	3.37E-04	2E-33	-0.003639006	0.022563214	0.034753524
Nags	1	1.00E+00	1E-32	0.000407462	0.011832564	0.034748127
Rasl11b	1	1.23E-20	2E-97	0.000868782	0.016894562	0.034733276
1700017B0	8.5982E-43	6.76E-06	8E-17	-0.038676492	0.033530811	0.034721602
Fut1	1	4.74E-100	4E-112	0.000374792	0.036751435	0.034716488
Heatr5b	1	1.00E+00	1E-11	-0.005389572	0.006860946	0.034713235
Ndufa13	1	1.42E-01	2E-53	0.00024011	0.012496186	0.034696468
Atp2c1	1	1.00E+00	1E-06	0.003432364	0.033426881	0.034694554
Itga10	1.30943E-09	1.21E-01	5E-27	-0.015692984	0.01937577	0.034682117
Rpn1	6.3059E-09	1.00E+00	8E-27	0.01767109	0.00780786	0.034677329
Atp6v1e1	1	1.00E+00	7E-05	0.001826288	0.01535796	0.034676462
Eif3j1	1	1.00E+00	0.0003	0.019513663	-0.019946236	0.034653386
Zkscan8	1	1.26E-01	4E-24	-0.002598009	0.020152003	0.034640632
Cep152	0.047867675	1.00E+00	3E-18	0.014178887	0.003258432	0.034625894
Sec23a	1	1.00E+00	0.0015	-0.000582766	-0.010361544	0.034612677
Gm14858	1	8.90E-39	4E-40	0.005631066	0.046993683	0.034585117
Papolg	1	1.00E+00	2E-18	0.002424207	0.011404497	0.034556459
Necap1	1	1.00E+00	2E-15	0.008285238	0.003089893	0.034549127
Ccar2	1	1.00E+00	3E-33	0.003120902	0.012512607	0.034532113
Bora	1	2.30E-04	8E-44	-0.004166441	0.017386401	0.034506673
Psma4	0.011905725	2.28E-03	7E-12	0.018136326	-0.028168144	0.034503154
Tnks2	0.16828636	1.18E-09	0.0775	0.024984699	0.081100996	0.034494585
Spg11	1	3.83E-03	2E-09	-0.011138833	0.041179568	0.034486656
Btbd1	1.70231E-12	1.00E+00	3E-07	0.035477918	0.037832357	0.034480912
Aga	1	1.77E-06	1E-30	-0.004627386	0.025536851	0.034468151
Baz1b	1	6.44E-04	0.0029	0.009374004	0.052980301	0.034467646
Zfp507	0.005256295	1.00E+00	1E-08	0.020318136	0.009593857	0.034456057
Trpt1	1	2.77E-15	7E-74	-0.000476971	0.018619862	0.034448689
Fkbp15	1	1.00E+00	0.0003	0.018852706	-0.012872159	0.034432638
Zfp770	1	6.95E-08	3E-54	0.00209668	0.017749405	0.034403102
Bola2	1	8.97E-23	6E-56	0.004867875	0.030769148	0.034394079
Nubp1	1	7.10E-06	3E-45	0.000479181	0.018479615	0.034388544
Slc52a2	1	1.29E-23	7E-71	9.17029E-05	0.024759331	0.034382092
Srsf3	1	1.00E+00	6E-07	-0.005320818	0.030252908	0.034378154
1810019D2	1	1.00E+00	7E-28	-0.005785447	0.001153375	0.034348186
Asb8	1	1.00E+00	8E-18	-0.000529467	0.015032938	0.034339025
Ipo9	1	1.00E+00	4E-15	-0.010687917	0.01442573	0.034307657
Klhl28	1	1.96E-11	6E-22	-0.006932624	0.038765732	0.034292201
Mrc1	1	4.07E-110	8E-23	0.000104497	0.135800646	0.034284971
Xrcc5	1	1.00E+00	5E-11	-0.005642284	0.010309708	0.034276244
Ddx54	1	1.07E-05	6E-21	0.006552876	0.031085403	0.034275181
Cep104	1	1.00E+00	2E-17	0.00424575	0.010494328	0.034247875
Adpgk	1	1.85E-01	3E-20	0.009894632	0.022171586	0.03423969

1810062G1	1	4.45E-44	4E-76	-0.00336187	0.032329516	0.034201859
Timm23	1	1.00E+00	4E-06	0.003920521	0.011877028	0.034201179
Gm14167	1	5.30E-112	1E-95	-0.001354316	0.047569332	0.034193861
Ppp2r1a	1	1.93E-01	1E-23	-0.001767539	0.019535421	0.034192371
Eif3m	1	1.15E-01	2E-20	0.008601442	0.022038125	0.034192329
Clcn6	1	3.94E-09	8E-47	0.005349846	0.022680746	0.034176977
Lgals3	1	0.00E+00	8E-114	-2.45585E-05	0.175710548	0.034155346
Rfx5	1	8.20E-02	5E-41	0.000439579	0.014347791	0.034129515
Ogfod2	1	1.00E+00	6E-46	0.001279441	0.011573443	0.034124956
Nkapd1	1	1.00E+00	2E-19	0.00527876	0.002330638	0.03412345
Arrb1	1	2.61E-155	2E-109	0.000792005	0.067585213	0.034109648
Gpkow	1	1.00E+00	5E-13	0.009147334	-0.000837796	0.034108134
Zfp617	1	1.00E+00	5E-19	-0.003088717	-0.00466524	0.034100178
Mmab	5.57174E-07	1.00E+00	3E-28	0.015530293	-0.007983099	0.034094676
Ebag9	1	1.68E-04	1E-28	0.005100582	0.021934171	0.034094612
Kcng3	1	7.91E-37	3E-92	-0.000917436	0.020277587	0.034079691
Narf	1	1.67E-01	2E-07	-0.012254332	-0.020803277	0.034056774
Stab2	0.075787899	1.15E-107	9E-08	0.014952949	0.155501043	0.034050779
Rbm48	0.868911115	1.00E+00	1E-25	0.00955674	0.006327015	0.034038122
Fkbp1a	1	6.85E-31	5E-32	0.001226589	0.048798333	0.034031753
Zwilch	1	1.98E-12	2E-76	-0.001760278	0.016140109	0.034025127
Usp43	1	1.90E-161	3E-145	4.1156E-05	0.039641531	0.034003193
Pigo	1	1.00E+00	9E-36	0.007651577	0.010199472	0.03399527
Adgre5	1	2.21E-263	2E-68	0.001018381	0.134811679	0.033968236
Ezh2	0.007084051	1.00E+00	2E-09	-0.016683629	0.009467084	0.0339654
Sat2	1	7.57E-06	2E-62	0.000578259	0.013688398	0.033943979
Cpne3	1	1.00E+00	4E-07	0.014283388	-0.002468914	0.033930522
Metap1	1	1.00E+00	1E-13	-0.001635781	0.019109704	0.033927766
Stk25	1	1.00E+00	7E-15	0.010365605	-0.006189768	0.033917122
Gm26740	1	3.59E-277	1E-59	-0.001331625	0.161690711	0.0338993
AI480526	0.000153654	1.62E-01	8E-10	0.022265024	0.03295618	0.033887973
Mcm4	1	1.72E-24	2E-91	-0.001079169	0.019646227	0.033887119
Pole	0.000501542	3.42E-08	3E-62	-0.005381044	0.016736355	0.033886239
Cyp2a22	1	1.98E-109	6E-99	0.002520764	0.043925906	0.033874815
Ppdpf	0.105248191	1.33E-03	5E-48	0.007611601	0.015998036	0.033871808
Kat5	1	8.05E-10	2E-38	0.002867205	0.025186048	0.033835919
Gba	1	8.41E-13	4E-69	0.001754042	0.018800007	0.03381316
Pgm2	1	1.52E-32	3E-92	3.85876E-05	0.023060427	0.033781947
Gtpbp8	1	1.32E-07	6E-26	-0.002320605	0.029690648	0.033775734
Atf6b	1	1.06E-04	6E-46	0.005675465	0.016922871	0.033774599
Tsfm	1	2.29E-09	3E-46	0.003893214	0.020822486	0.033747556
Ctss	1	0.00E+00	7E-80	-0.000264575	0.174271156	0.033715054
Hps1	1	3.70E-10	8E-36	0.002917043	0.026987979	0.033711702
Parp4	0.004008587	1.00E+00	0.0006	-0.021008558	0.036592361	0.033706358
Rrs1	1	2.32E-01	2E-34	0.005376735	0.015632433	0.033663877
Mkln1os	1	1.00E+00	2E-19	-0.003317548	0.009539215	0.033633825
Coa6	1	7.73E-17	1E-49	0.003243975	0.025879242	0.033597422

Gm15461	1	4.43E-84	8E-76	0.002814873	0.045723752	0.033583956
4930452B0	1.27925E-16	8.79E-07	3E-38	-0.01354679	0.021390745	0.033527573
Megf8	1	3.17E-03	1E-25	-0.003902532	0.021656271	0.033523252
Dxo	1	5.44E-09	5E-40	0.001889537	0.022996928	0.033522369
Zfp955a	1	1.00E+00	2E-44	0.00333033	0.010157161	0.033521414
Pkd2l2	1	4.41E-08	1E-50	0.00433078	0.018469413	0.033502681
2700046G0	1	2.16E-25	1E-105	0.000863791	0.016655313	0.033500663
Serp1	1	1.00E+00	3E-20	0.003775454	0.016922325	0.033469318
Pak7	0.004423185	5.43E-90	4E-69	-0.004123944	0.054135145	0.033468439
Rfxank	1	1.00E+00	1E-22	-0.003652934	0.005554432	0.033458275
Ptpn21	1	1.00E+00	3E-11	-0.005269967	-0.013500056	0.033456034
Naxe	1	1.77E-04	3E-54	0.002639808	0.0146991	0.033388735
Ggct	1	2.18E-12	7E-41	-0.001915016	0.024671403	0.033383253
Mpc1	0.300563086	1.00E+00	0.0462	0.023862193	0.023802401	0.033348991
Abhd17b	9.41952E-17	1.00E+00	0.0067	-0.043618458	-0.01173776	0.033344263
Tigd2	1	1.32E-02	1E-39	-0.003342708	0.015704836	0.033296767
Zfat	1	1.00E+00	4E-13	-0.003338299	0.000314812	0.033264545
Xab2	1	1.00E+00	9E-37	0.003994553	0.010409087	0.033227638
Bzw1	1	6.78E-07	3E-12	-0.006222098	0.038990111	0.033208342
Rnasek	1	4.33E-12	2E-43	0.00612981	0.025078922	0.03317152
Parl	1	1.00E+00	1E-13	0.00674196	0.008335777	0.033133661
Srp68	0.002965016	1.00E+00	1E-09	0.019693807	-0.017584947	0.033131061
Cstf1	1	7.73E-01	9E-29	0.006879042	0.013994944	0.033115085
Cyp51	6.57587E-34	1.02E-43	0.0044	0.058585813	-0.067318254	0.033103604
Ccdc97	0.60609118	1.00E+00	3E-30	0.008764138	0.006971705	0.033103305
Fam171a1	1	2.20E-10	2E-85	0.00152741	0.014912299	0.033081137
Adgrg1	1	8.38E-101	1E-136	-0.000407298	0.028041128	0.033074232
Ndufa9	1	4.31E-04	2E-20	0.007970709	0.024960562	0.033055785
Med21	1	1.00E+00	5E-20	-0.009599528	0.013531513	0.033035253
Pmpca	1	9.30E-04	5E-30	0.003759348	0.02110613	0.033022917
Polr3d	0.004823337	1.09E-06	8E-37	0.00964691	0.021616286	0.033010212
Pa2g4	1	1.20E-01	5E-14	0.011663617	0.026083304	0.032987474
Gtf3c2	0.008976957	1.18E-03	4E-05	0.0254922	-0.03058107	0.032973597
Gm10734	1	3.40E-03	3E-60	0.004643621	0.011549766	0.032933615
Nfxl1	3.25973E-22	3.93E-02	6E-07	0.043779566	-0.022733753	0.032912292
Ndufaf5	1	3.59E-04	1E-46	-0.001194938	0.015323358	0.03290863
Zfp608	9.56362E-06	8.76E-08	2E-10	0.018596486	0.050677552	0.032881735
Synrg	1	1.00E+00	0.0004	0.002384599	-0.000417954	0.032879835
Cops9	1	8.50E-28	3E-57	0.003068567	0.030359926	0.032871275
Nt5m	1	1.83E-02	2E-15	-0.003237523	0.026770364	0.032869514
C130046K2	1	8.24E-03	1E-30	-0.003760887	0.017824514	0.032863136
Tiam1	1	1.77E-17	4E-59	0.000225134	0.021313276	0.032848039
Vps33b	1	1.00E+00	1E-28	0.002372867	0.014508506	0.032843186
2810402E2	1	1.20E-12	1E-32	0.00351523	0.02795419	0.032818197
Nme5	1	3.02E-97	4E-81	-0.003274088	0.046649887	0.032817625
Mrpl10	0.161170907	1.00E+00	3E-21	0.011375387	0.010591998	0.032808257
Fam32a	1	7.13E-22	5E-24	0.007191209	0.04474591	0.0327738

Lsm7	1.97307E-05	1.00E+00	4E-22	0.01511129	-0.001617872	0.032764752
Gsta2	1.79548E-10	1.17E-46	0.0008	-0.031045583	0.135385312	0.032755735
Gm26770	1	2.92E-19	4E-93	0.002381641	0.015874575	0.032722426
Mrpl30	1	1.01E-04	6E-32	-0.001078788	0.019904721	0.032703649
Mep1a	1	3.08E-33	2E-131	0	0.008777842	0.032698392
Psenen	1	8.62E-04	3E-41	0.003981639	0.017093254	0.032679753
Txndc5	3.90323E-19	4.03E-07	8E-05	0.040353681	-0.033719122	0.032675242
4933421D2	1	4.48E-198	3E-138	0.000271757	0.049988783	0.032657175
Il17rb	1	1.00E+00	9E-37	-0.002310006	0.009074829	0.032654961
Doc2g	1	1.61E-15	1E-32	0.003230451	0.031170133	0.032647133
Caml	1	1.00E+00	5E-11	0.003310258	0.00999271	0.032644463
Phlpp2	1	1.50E-03	6E-19	-0.006536557	0.026645134	0.032623513
Tdg	1	1.00E+00	6E-15	0.005093562	0.012243939	0.032616237
Zfp641	1	2.95E-15	2E-105	-0.000859539	0.011795765	0.032614344
Cluap1	1	1.00E+00	2E-14	-0.004723961	0.00351419	0.032610806
Zfp36	1	1.77E-01	1E-08	-0.001096577	0.030767781	0.032597405
Eed	1	1.00E+00	1E-06	0.003550058	-0.000854588	0.032597088
Emc4	1	1.00E+00	2E-39	0.000237167	0.0071369	0.03257126
Mlx	1	2.16E-04	1E-37	0.006565112	0.017712886	0.032568594
Chkb	2.82298E-46	3.97E-07	7E-06	0.068748473	-0.041040397	0.032550238
Gm10138	0.042661141	6.49E-36	1E-36	0.009117496	0.043981034	0.032531518
Mrm3	1	2.15E-12	2E-50	0.001934426	0.021015875	0.032515676
Bloc1s2	1	6.36E-01	3E-18	0.002613248	0.021780268	0.032506953
Rhpn2	5.30485E-30	1.00E+00	0.0237	0.053176581	0.004368317	0.032496695
Scmh1	0.003748501	1.18E-01	0.0085	-0.025425009	-0.03041435	0.032487334
Rnf8	1	1.00E+00	5E-10	0.002126262	0.014025484	0.032453911
Ssr2	0.12676286	1.00E+00	1E-30	0.009291207	0.002787215	0.032433924
Sde2	1	1.00E+00	1E-19	0.002188407	0.015745739	0.03242964
Gabpb1	1	1.00E+00	9E-07	-0.013289116	0.016584091	0.032420423
9430037G0	1	2.23E-18	4E-42	0.004205804	0.028846807	0.032406333
Miga2	1	6.70E-04	0.002	-0.008263141	-0.032130045	0.032401181
Wdr46	1	1.22E-14	3E-56	0.001095051	0.022501552	0.032326053
Abcg1	1	0.00E+00	3E-107	0.000466952	0.134210734	0.032324603
Catsperd	0.706662419	5.28E-09	3E-47	-0.004931173	0.019303664	0.032322708
Snrpe	1	6.22E-22	3E-40	0.004597321	0.032421756	0.032322116
Gtf2b	6.85305E-19	1.00E+00	1E-11	-0.029972617	0.012123409	0.032279074
Cfap47	1	9.07E-35	5E-84	-0.001865143	0.024113117	0.032268902
Pi4k2a	1	9.79E-02	2E-05	-0.0051645	-0.022678345	0.032265112
Urad	5.54625E-77	6.26E-03	9E-28	-0.030495932	0.019069504	0.032256884
Rpgr	1	7.43E-09	1E-17	-0.00757474	0.039450921	0.032233133
Kdm4c	1	1.00E+00	0.0025	-0.013791404	0.000914436	0.03222109
Hsd17b7	3.77865E-17	2.49E-09	3E-14	0.033619835	-0.030471532	0.032157097
Kctd1	1	4.01E-25	8E-42	0.004057756	0.032200038	0.03215224
1500015A0	1	3.96E-01	3E-40	-0.000603547	0.011445442	0.032134494
Gas8	1	3.82E-18	3E-69	-0.000873903	0.020424675	0.032105989
1110038F1	0.002276461	1.00E+00	5E-28	0.01178871	0.013656576	0.032105075
Tchp	1	1.00E-03	4E-43	0.002403868	0.015379602	0.032103668

Pcyox1l	1	4.85E-27	1E-73	-0.000283331	0.023340332	0.032093529
Tmem165	1	5.74E-10	7E-23	0.002318877	0.033435742	0.032006331
Hace1	0.073093721	1.00E+00	7E-10	-0.014330239	0.022164808	0.031997963
Arntl2	1	7.43E-01	2E-36	0.003458295	0.011972063	0.031978026
Abhd5	0.010257583	7.45E-01	5E-16	-0.012373805	0.021430671	0.031940911
Zfp938	1	3.68E-01	3E-38	0.002007685	0.013055742	0.031933758
Nr1i2	1.7823E-104	1.00E+00	1	0.136073965	-0.005446419	0.03191859
Zkscan7	1	8.51E-03	6E-20	-0.009094549	0.021579072	0.031916859
Slc25a40	1	1.00E+00	1E-20	-0.007990125	0.011968257	0.031914654
Txnl1	1	1.00E+00	8E-08	9.91658E-05	-0.011075175	0.03191419
Smyd2	0.054874035	1.00E+00	3E-16	0.013028058	0.011827122	0.03190071
Utp15	1	2.10E-11	8E-31	0.002949372	0.027512959	0.031893906
Gtf2h3	1	4.25E-07	5E-37	0.004337475	0.019937009	0.031887126
Styxl1	1	1.92E-17	6E-46	-0.001804455	0.027258326	0.031880921
Myl6	1	9.16E-06	2E-14	0.010919171	0.03900419	0.031872207
Sdf4	1	1.00E+00	3E-12	-0.001129155	0.002995417	0.031868228
H2-DMa	1	1.81E-20	3E-42	0.001209683	0.030456259	0.031866137
Yju2	1	1.85E-17	5E-35	0.004857829	0.031316871	0.031829387
Ighmbp2	0.097571407	1.00E+00	5E-22	0.01097938	0.002341168	0.031828431
6430550D2	1	2.40E-02	4E-15	-0.005640242	0.026104912	0.031827032
Hacd1	1	1.00E+00	4E-16	-6.01242E-05	-0.001079489	0.031796034
Pip4p1	1	1.00E-07	4E-23	0.003200329	0.031538603	0.03177673
Apopt1	1	1.00E+00	2E-05	-0.005660368	0.010547926	0.031774398
Isoc2b	1	6.51E-01	6E-24	-0.002504324	0.015633645	0.031772563
Tomm7	1	1.01E-31	3E-65	0.001883016	0.028433736	0.031765324
Gps2	4.51247E-13	1.00E+00	4E-15	0.025653291	0.002684658	0.031764188
Tmem9b	1	1.02E-11	3E-32	-0.002260791	0.029011214	0.031759197
Ccdc9b	1	4.36E-75	3E-119	0.001620715	0.02557986	0.031694038
3830406C1	1	1.91E-02	5E-14	-0.003281538	0.028087169	0.031690031
Washc1	1	1.48E-01	5E-28	0.001072939	0.015436661	0.031670079
Ttc9c	1	1.00E+00	5E-19	0.00041068	0.013408999	0.031654893
Coq6	0.756916083	1.13E-01	2E-43	0.006719045	0.011713556	0.031650932
Nubpl	1	2.21E-06	5E-07	-0.013007331	0.043350064	0.031617005
Erap1	1	1.00E+00	9E-05	-0.00066952	0.011610307	0.031602521
Nudt8	1	2.61E-32	6E-71	0.001683228	0.026443157	0.031572547
Gm15883	1.10584E-09	1.83E-14	6E-13	0.027890995	0.052901267	0.031547857
Tada2a	1	1.00E+00	8E-10	-0.00683736	0.011569243	0.03153792
Pip4k2a	1	2.64E-211	3E-47	0.00132034	0.140340402	0.031505374
Nphp1	1	2.39E-47	4E-82	0.001741156	0.028171336	0.03148657
Mks1	1	2.49E-17	2E-56	0.001772939	0.021904748	0.031486444
4931406G0	1	3.05E-08	5E-50	-0.002950047	0.016556068	0.031484408
Snrnp48	0.006052538	8.49E-01	1E-05	0.022370274	-0.019552191	0.031476611
Hnrnpa0	0.03794905	1.20E-15	1E-23	0.010825098	0.036582542	0.031476335
Mettl4	1	2.10E-05	3E-25	-0.007905015	0.023833756	0.031461004
Apoa4	0.003656158	4.39E-41	2E-83	0.00482811	0.02427889	0.031457853
Cdk5	1	1.00E+00	2E-24	0.005094329	-0.003563901	0.031404113
Apbb1ip	1	1.15E-301	1E-70	-0.000294076	0.147494244	0.031396639

Ilf2	1	5.00E-03	1E-13	0.001570379	0.027276948	0.031393938
Rala	1	6.45E-01	3E-08	-0.011218229	0.032529025	0.031384477
Pdia4	7.75748E-41	1.40E-02	1E-07	0.052641166	-0.024214247	0.031375872
Pdia6	0.000200248	1.16E-03	1E-08	0.020124085	-0.023240928	0.031366518
Hccs	1	7.41E-03	5E-35	-0.006262593	0.016342213	0.031344903
Gm48163	1	1.01E-19	2E-23	-0.007912817	0.040390987	0.031342997
Dkc1	1	3.03E-19	2E-31	0.003884635	0.033281694	0.031336754
Rrp1	1	1.00E+00	1E-19	0.007131705	0.007661424	0.031304317
Foxp4	1	1.00E+00	4E-05	0.001121528	0.005650958	0.031293747
Rai2	1	1.00E+00	4E-34	-0.005088322	-7.69854E-05	0.031272817
Adam23	1	7.07E-43	8E-45	0.004566689	0.055534546	0.031269897
Ssrp1	1	1.00E+00	1E-15	0.008053046	0.014502976	0.031257245
Ufm1	1	2.19E-07	5E-42	0.002510046	0.019002153	0.031240739
Gm10563	1	5.82E-36	2E-45	0.002959874	0.036293146	0.031165434
Srp54a	1	1.00E+00	7E-25	0.002859063	0.009647052	0.031148753
Rnf213	1.45522E-06	1.68E-06	1E-05	-0.032826871	0.098741595	0.031143959
Arhgap22	1	3.70E-242	7E-61	-0.000606183	0.135284261	0.031121618
Psme3	1	4.54E-05	8E-27	0.000486454	0.022584154	0.0311182
Chchd2	1	1.70E-06	7E-23	-0.004669573	0.02926446	0.031103525
Rnft1	1	2.06E-07	6E-18	-0.001935979	0.031931596	0.031098301
Paxip1	1	1.00E+00	2E-09	0.001424412	0.016495942	0.031092639
Wdr37	1	8.30E-01	1E-05	0.01317077	-0.020321913	0.031084842
Ankrd40	1	3.43E-04	1E-11	0.007853821	0.032619847	0.031043555
Zfp280b	4.98776E-11	6.60E-04	2E-41	0.013370165	0.015321812	0.031020058
Mettl9	1	1.00E+00	3E-10	-0.009120107	-0.002763241	0.031006529
Tbc1d30	5.9584E-61	2.10E-23	1E-11	-0.045758311	0.062161775	0.030982604
Aldh3b3	1	1.36E-70	4E-136	0.000422729	0.01774678	0.030972107
Lrtm1	5.47743E-12	3.65E-03	6E-38	-0.009839661	0.014068807	0.030959661
Msantd3	1	4.81E-61	4E-136	-0.00014651	0.015544767	0.030956711
Stil	1	3.49E-39	2E-79	-0.001787064	0.024072512	0.030948597
Tsta3	0.000130825	1.00E+00	6E-32	0.011228394	0.011206367	0.030942108
Kpna2	1	7.09E-11	2E-30	-0.002246423	0.026733056	0.030935992
Usp22	1	1.00E+00	9E-21	-0.002893371	0.00548283	0.030922106
Rrp1b	1	7.46E-06	5E-31	0.001815064	0.021633781	0.030903158
2610044O1	1	1.00E+00	5E-26	-0.000330312	0.008620747	0.030892017
Rnf168	1	1.00E+00	1E-11	0.004556295	0.021870073	0.030881675
Tmem184c	1	1.14E-22	6E-57	-1.0309E-05	0.02698801	0.030878714
Ndc1	0.202291433	1.02E-06	2E-23	-0.008357287	0.025131463	0.030862758
Slc25a26	1	1.00E+00	2E-05	0.01478162	0.010061618	0.03085174
Sirt1	0.478172116	3.35E-01	3E-08	-0.014692129	0.028998415	0.030832409
Tmem69	1	2.15E-18	3E-37	0.003126101	0.029146992	0.030814365
Arhgap27	1	1.03E-139	1E-109	-6.10109E-05	0.046363586	0.030809474
Ankrd13a	0.330251726	7.49E-02	2E-19	0.010343956	0.024413849	0.030809439
Trmt61a	1	1.16E-43	1E-88	0.002905829	0.024496434	0.030806134
Cry1	1	1.65E-02	7E-09	0.00873397	0.034108909	0.030794509
Mrpl54	1	5.94E-30	3E-69	-0.000176642	0.025367	0.030775866
Nup54	1	1.00E+00	4E-08	0.004853476	-0.000782797	0.030729754

Rbm8a	1	1.00E+00	8E-19	0.008653218	0.013748625	0.030728308
Timm8a1	1	3.21E-28	6E-50	0.000190447	0.031379353	0.030699168
Sft2d1	0.000720273	6.65E-06	6E-07	0.021986757	0.050621903	0.030669293
Trmt5	1	8.15E-16	4E-62	0.00108687	0.019317326	0.030664932
Pes1	1	5.56E-07	1E-27	0.00444269	0.023698852	0.030650249
Adam10	1	1.00E+00	0.0079	-0.003694672	0.026581777	0.030648356
Ahsa1	1	1.35E-08	5E-29	2.50819E-05	0.025511672	0.030641738
Wdr34	1	9.90E-10	2E-22	0.000546638	0.029320555	0.030632124
Rabggta	1	1.00E+00	4E-18	0.003485899	0.004002372	0.030623746
Timeless	1	1.55E-12	5E-56	-0.004004279	0.018378483	0.03062295
Ggnbp1	1	1.00E+00	1E-05	0.000540129	-0.014451498	0.030622174
Uckl1os	1	5.28E-09	5E-43	4.72485E-05	0.019632088	0.030614716
Nsa2	1	2.38E-21	1E-36	0.001232515	0.032491325	0.030613866
Mcee	1	1.00E+00	5E-18	-0.004809573	0.016181582	0.030586604
Polr2f	1	1.41E-09	3E-29	0.004301353	0.026073311	0.030575347
Bscl2	1	1.00E+00	1E-22	-0.000263024	0.015386464	0.030554359
Phf20l1	1	8.31E-12	1	-0.001119635	0.093781753	0.03055411
Hnf1b	1	1.00E+00	3E-05	-0.003726443	0.016289707	0.030552036
Mcm2	1	1.26E-79	5E-106	-0.001059267	0.028857692	0.030531084
Creld2	1.88555E-21	1.00E+00	2E-35	0.020255361	-0.000533038	0.03053046
Tstd3	1	4.29E-04	2E-41	-0.005182028	0.014650806	0.030526467
Park7	1	5.08E-06	5E-13	0.00893348	0.033689929	0.030504414
Apoo	1	1.42E-01	5E-17	-0.002519412	0.020060545	0.030497725
Srp14	1	1.81E-04	4E-31	0.004679182	0.018525063	0.03049076
Prpf18	0.005172873	1.00E+00	9E-05	-0.019136033	0.006363627	0.030479723
Tmem43	1	8.19E-32	3E-60	0.00329699	0.028542974	0.030461601
Rpp38	1	9.42E-29	2E-76	-0.000390809	0.021297423	0.030441862
4732440D0	1	4.01E-14	2E-36	-0.001496199	0.026910677	0.030435565
Brox	1	4.81E-01	7E-15	0.007152357	0.023680877	0.030387203
Ndufa7	1	1.00E+00	8E-13	0.012397318	0.003717579	0.030382492
Gm20457	0.159361363	4.13E-03	9E-28	-0.008137079	0.017902283	0.030382414
Zfp993	1	2.06E-18	2E-71	-0.000225351	0.019665703	0.030378108
Aimp1	1	1.00E+00	9E-11	0.009679034	0.002685197	0.030365418
Npdc1	1	2.89E-59	1E-121	0.000849993	0.019129225	0.030359505
Gm5617	0.239657419	4.41E-34	2E-76	0.004309653	0.024883056	0.030340048
Cfap97	1	1.00E+00	1E-10	-0.006601941	0.001382724	0.030327171
Stub1	1.50675E-05	1.00E+00	2E-09	0.02059291	-0.003443697	0.030307978
Fbxl2	1	5.33E-07	1E-28	0.003029797	0.023419445	0.030304699
Gspt2	1	2.29E-24	3E-125	0.000910607	0.008262153	0.030302382
Isyna1	1	1.19E-19	3E-88	4.64873E-05	0.015833329	0.030290408
Cep170b	1	2.26E-03	8E-19	0.003112345	0.023412084	0.030271147
Naga	1	2.07E-02	2E-29	0.002076928	0.019681144	0.03026297
Ssr1	1	1.00E+00	2E-12	0.00559238	0.004462755	0.030257581
Prdm4	1	1.53E-13	1E-34	-0.00389138	0.02647186	0.030255913
Psma3	2.04452E-05	1.00E+00	0.0411	0.032623445	0.027742267	0.030228869
Eral1	2.14093E-05	1.00E+00	1E-35	0.010876557	0.010202079	0.030210604
Acot2	7.79493E-05	2.40E-74	5E-106	0.00377007	0.025828128	0.030154453

Lin37	1	2.18E-08	1E-36	0.002754391	0.020483391	0.030149031
Prkcb	1	3.75E-258	4E-44	-0.000381833	0.183320174	0.030141924
Snupn	1	1.00E+00	4E-20	-0.005631576	0.012729929	0.030117535
Ercc2	1	1.00E+00	6E-21	0.002086465	0.011752478	0.030086136
Eif1ad	1	2.20E-11	2E-52	0.004321905	0.018624196	0.030084708
Aoc1	1	5.02E-112	2E-133	0.000582577	0.026155461	0.030079972
Taf1c	1	1.11E-40	1E-69	0.001402737	0.02824704	0.030071177
Ppa2	1	1.00E+00	1E-09	0.004348799	0.009705503	0.030070898
Mark4	1	1.00E+00	5E-12	0.00318035	0.002416377	0.030050011
9930104L0	0.090986705	1.51E-02	7E-38	0.00760505	0.01296817	0.030043858
Gnpda2	1	1.88E-10	9E-52	-0.003388325	0.018380324	0.030042694
Entpd1	1	4.48E-293	2E-78	0.001054254	0.137476102	0.030034206
F2	1	5.92E-39	0.2813	0.014982215	-0.113042417	0.0300257
Zfp229	1	4.74E-01	2E-24	-0.00479265	0.014238632	0.03002443
Gpr137b	1	1.76E-267	5E-82	-0.000316868	0.131491246	0.030018591
Mbnl3	1	1.05E-45	1E-77	0.001358329	0.027883222	0.03000746
Get4	1	1.17E-02	5E-22	0.005689987	0.018967949	0.03000104
Entpd7	1	1.48E-06	2E-37	0.005035367	0.019176005	0.029997375
Ano6	0.000613327	9.52E-38	0.0049	0.019819459	0.129406761	0.029970182
Dad1	1	1.00E+00	1E-15	0.010865208	0.006898818	0.0299294
Mrps22	1	6.92E-02	1E-37	0.003548937	0.012627417	0.029884284
Ptpn11	1	1.00E+00	6E-06	0.006787239	0.001052647	0.029879744
Miga1	1	2.30E-29	3E-57	0.000461164	0.027290174	0.029852994
Atg13	0.011238685	5.93E-05	0.0002	0.023846513	-0.037479135	0.029850582
Zfp941	1	9.44E-34	5E-75	-0.000959117	0.023913457	0.029844668
Ube2m	1	1.00E+00	2E-18	0.006002079	0.016632933	0.02980518
Snrpc	1	4.00E-06	4E-30	0.003327712	0.020477289	0.029781851
Rab11fip4	1	9.20E-27	5E-32	0.006196371	0.037369728	0.029779564
Dnajc15	1	1.00E+00	7E-11	-0.010342606	0.017553037	0.029768504
Nup188	1	1.00E+00	7E-10	0.003026928	0.005910556	0.02975624
Polh	1	3.13E-06	1E-25	-1.57813E-05	0.021292343	0.029751348
Mrps18a	1	1.28E-04	2E-19	0.003562073	0.024985148	0.029740328
Map3k14	1	1.32E-21	5E-13	-0.000420486	0.056158929	0.029686219
Klhdc4	1	1.00E+00	2E-31	0.004519261	0.012395314	0.029666402
Tmem168	1	2.59E-06	1E-18	-0.004424505	0.027433299	0.029657033
Capn11	1	1.00E+00	1E-86	-0.001422535	0.000189718	0.029645127
Tfec	1	1.61E-121	1E-69	0.000461266	0.069965905	0.029640406
Plekha4	1	8.76E-28	7E-56	-0.001310366	0.025425362	0.029637339
Rab20	1	9.95E-21	8E-14	-0.005629979	0.05773118	0.029614055
mt-Nd5	1	6.67E-43	7E-62	-0.000890609	0.032413801	0.029591808
Cstb	1	1.29E-57	4E-65	-0.000616103	0.03840746	0.02959067
Rad9b	7.45846E-12	5.98E-05	1E-21	-0.017494957	0.024858669	0.029581354
Cmc2	1	5.93E-01	1E-24	-0.002546395	0.015343421	0.029574642
Uhmk1	1	1.00E+00	2E-11	-0.00418241	0.014045006	0.029567952
Faim	1	3.76E-05	4E-32	4.35257E-06	0.018290632	0.029554341
Sgo2a	1	5.20E-44	7E-103	-0.000184406	0.018217376	0.029514643
Tmco6	0.073365804	1.46E-11	2E-32	0.008254089	0.024531591	0.029513551

Mkrn1	1	3.00E-05	8E-20	0.001372493	0.025287638	0.02951313
Eif4a3	1	3.65E-01	4E-17	0.004704219	0.018380472	0.029495944
Gtpbp3	1	3.22E-09	1E-43	-0.003006074	0.018643743	0.02949109
Nsmf	1	2.50E-01	3E-05	0.014972516	-0.025586747	0.029469601
2310058D1	1	7.93E-02	5E-12	0.001634993	0.024722546	0.029467146
Mmp14	0.000545744	2.54E-02	1E-21	0.012366483	0.021947775	0.029461269
Trim8	1	2.20E-03	6E-14	-0.006150916	0.02563339	0.029458633
Ptpru	1	2.58E-115	9E-107	5.7745E-06	0.03533281	0.02944489
Tmem87a	1	1.00E+00	0.0006	0.015341119	-0.001037475	0.029440843
Rsph3a	1	7.11E-02	3E-16	0.00065989	0.021075347	0.029436582
Psmb2	1	1.00E+00	0.0019	-0.002935689	-0.010076629	0.029429485
Ifngr1	0.03595237	1.64E-05	5E-21	-0.00977803	0.028247697	0.029411402
Ccdc173	1	2.26E-11	9E-56	-0.001340952	0.017059714	0.029407377
Ophn1	1.89418E-07	1.00E+00	1E-07	-0.020482707	-0.00996674	0.029356896
Zfp729b	1	1.00E+00	1E-17	0.003404711	0.007778563	0.029331816
Snrpd1	1	8.69E-03	4E-20	-0.003444896	0.020538147	0.029319208
Gm12092	1	3.43E-225	2E-115	0	0.059566478	0.029314802
Mrpl27	1	1.00E+00	5E-26	0.001732049	0.009878473	0.029311939
Surf1	1	2.94E-06	1E-55	0.002739156	0.013346961	0.029299692
Myo6	1.25842E-09	1.00E+00	1	-0.048455957	-0.016883271	0.029278303
Mrps34	1	4.99E-12	9E-55	0.000957412	0.017572555	0.029257292
Nedd4	1.41817E-11	1.00E+00	1	-0.049899179	0.020424195	0.02919564
Selenop	5.0862E-218	1.11E-04	1	0.203648933	-0.051846659	0.029195208
Gm34574	1	2.55E-153	2E-119	0.000183325	0.039749108	0.029186651
Cnpy2	1	1.96E-01	7E-22	0.003440712	0.016297528	0.029152328
Ubl7	1	1.00E+00	1E-13	-3.80324E-05	0.018121485	0.029134305
Zfp983	1	1.00E+00	7E-20	-0.001815129	0.011127268	0.029131069
Arpc5	1	2.07E-07	1E-13	0.001124428	0.03713452	0.029129943
A530020G2	3.85013E-06	1.00E+00	4E-23	-0.01197086	0.004173847	0.02912696
Ptpn3	4.2899E-111	1.00E+00	0.1714	-0.129366238	-0.00403735	0.029106781
Gpaa1	1	1.00E+00	6E-20	0.008883379	0.014902585	0.029102305
Kat8	1	1.00E+00	4E-14	0.005290243	0.000323069	0.029089818
Gm44206	1	8.54E-53	6E-59	0.00089604	0.035950333	0.029080987
Prelid1	1	5.85E-04	1E-17	-0.001973107	0.02508933	0.029070106
Sufu	1	1.00E+00	6E-09	-0.00901712	-0.00015577	0.029068478
Nat10	1	2.34E-37	0.178	-0.004984011	-0.095693474	0.029063772
Borcs7	0.087340217	1.00E+00	4E-22	0.010363468	0.005142631	0.029053431
Ptdss2	1	1.00E+00	7E-19	-0.007361525	0.01311005	0.029050924
Sfi1	1.45853E-05	4.22E-13	0.0078	0.03144827	-0.050818035	0.02904968
Fam129b	1	1.36E-222	1E-102	0.000110298	0.084468015	0.029047813
Yars2	1	6.22E-14	7E-55	0.003663797	0.018870755	0.029047541
Vdac2	0.892671031	7.80E-03	8E-10	0.012407807	0.030764602	0.029034819
Tmem63a	1	6.63E-30	1E-41	-0.001969393	0.033152428	0.029028329
Mtmr12	4.70689E-20	1.00E+00	1E-10	0.030581691	0.01516083	0.029027636
Thop1	1	6.22E-32	2E-76	0.001718534	0.021873941	0.029027413
Cchcr1	1	3.89E-03	2E-33	-0.004830918	0.01476406	0.029007567
Dhrs7b	1	4.53E-09	6E-19	-0.007611056	0.030610776	0.028996599

Gm46210	0.456123034	2.77E-01	8E-33	-0.006155607	0.013684864	0.028990518
Edem3	1	1.00E+00	0.0001	0.010435876	-0.000523082	0.028976793
Kif11	1	2.55E-09	1E-53	0.001150921	0.014777084	0.028966678
Gucy2c	1	8.12E-182	4E-99	0.000297869	0.055501719	0.028965453
Sun2	6.67711E-07	4.33E-02	0.0183	-0.026212525	-0.021415731	0.028937824
Nif3l1	1	2.65E-18	3E-39	0.000748262	0.026093942	0.028933254
Zfp874b	0.010906783	2.14E-04	2E-16	-0.01099403	0.026578348	0.028930718
Ces2g	1	3.25E-34	1E-77	-0.001104161	0.022584766	0.028922197
Aurkaip1	1	4.17E-12	1E-40	0.004049908	0.021936222	0.028893089
Capn2	1	1.00E+00	9E-11	-0.008280152	0.015738187	0.028883779
Rpia	7.67009E-07	2.20E-12	2E-37	0.010810023	0.023464136	0.028853372
Gm4189	1	1.44E-06	7E-51	0.002891067	0.01376436	0.02885325
Hadhb	1	1.00E+00	0.0423	0.001327254	-0.021153574	0.028846426
Txn1	0.715338359	6.89E-11	3E-08	-0.012326428	0.053634888	0.028836746
2310030G0	1	1.00E+00	4E-19	0.008236661	0.004400523	0.02882722
Ecel1	1	2.14E-116	5E-122	0	0.029373517	0.028797905
Abcf2	1	1.00E+00	9E-19	0.006045667	0.010386339	0.02879759
Gm10373	1	4.38E-10	8E-59	-0.001343529	0.015049871	0.028793601
Pcgf1	1	2.14E-08	2E-46	0.004120696	0.017313374	0.028773898
Stx12	1	1.00E+00	3E-08	-0.001431001	0.016182474	0.028768047
Ubox5	1	1.00E+00	1E-10	-0.008141748	0.013059133	0.028737456
Brat1	1	2.10E-09	2E-29	0.002387563	0.024717348	0.028704512
Vps16	1	1.00E+00	7E-11	0.01056465	0.019045281	0.02870419
Zfp934	1	3.65E-33	2E-49	-0.002314038	0.035810791	0.028688057
Rnf4	0.000493279	1.00E+00	4E-05	-0.020707659	-0.014260652	0.028650735
Arpc5l	1	1.00E+00	3E-16	0.009235208	0.020984539	0.028650176
Spg21	0.786764066	3.18E-03	5E-19	-0.008348008	0.020823273	0.028640223
Gm5150	1	6.91E-196	2E-76	-0.000448923	0.095109678	0.02862914
Cyp4f16	1	2.34E-50	1E-90	-0.001645234	0.024709078	0.028615416
Osbpl10	1	4.20E-41	2E-79	-6.44408E-06	0.02444343	0.028597441
Gsk3a	1	1.00E+00	1E-11	0.004498928	0.015465341	0.02856693
Armc1	1	1.00E+00	6E-10	0.004529464	-0.004158917	0.028561305
Dusp6	1	2.06E-01	1E-29	0.005013435	0.014655731	0.028558664
Gas2l1	1	8.68E-06	3E-34	0.003940745	0.01832525	0.028553279
Eif2b2	1	1.31E-15	1E-37	0.002946682	0.025809631	0.028529797
Zdhhc16	1	1.00E+00	5E-27	0.003691199	0.00733095	0.028419207
Pex12	1	8.81E-22	3E-55	0.004372592	0.022280758	0.02841229
Vdac3	1	1.00E+00	1E-08	-0.001200146	0.017788661	0.028406308
Pno1	1	2.78E-05	1E-36	0.000414064	0.016859768	0.028405144
Nudc	1	1.40E-04	8E-15	-0.003227091	0.025864622	0.028398278
Nfrkb	1	1.00E+00	1E-06	0.01312107	0.003029232	0.028371987
Psat1	1	8.70E-67	2E-113	0.00069617	0.018550342	0.028369253
Mfsd11	0.033496883	1.00E+00	4E-06	0.018283773	0.030786013	0.028364603
Rbm45	1	4.25E-08	3E-29	-0.004312486	0.022139892	0.028353352
Qtrt1	1	5.73E-15	1E-30	0.001501887	0.028782318	0.028347966
Slirp	1	9.90E-03	1E-47	0.001052064	0.011464242	0.02834031
Nfkbia	1.33202E-53	7.93E-12	0.0004	0.066247876	0.068128199	0.028318719

Znrd2	1	9.54E-18	4E-54	0.003056157	0.020310849	0.028315477
Irak4	1	1.00E+00	2E-12	-0.001086218	0.02005439	0.028312789
Anapc7	1	1.00E+00	3E-12	0.00178017	0.013144259	0.0282842
Gm19689	1	2.83E-75	6E-115	-4.73494E-05	0.019457017	0.028274029
AW146154	1	1.00E+00	2E-07	-9.39234E-05	-0.005012321	0.028241053
Zfp931	1	2.24E-10	5E-40	-0.003393651	0.019051618	0.028214512
Rrp9	1	2.83E-05	2E-34	0.003492591	0.016904736	0.028198286
Usp31	1	1.00E+00	5E-12	0.002064167	0.017576142	0.02818031
Mrpl28	1	1.00E+00	7E-24	-0.001844355	0.009775678	0.028161544
Rufy2	1	1.92E-06	1E-13	0.001324473	0.030720956	0.028158624
Slc25a11	1	1.00E+00	8E-23	-0.001006865	0.005297294	0.028156943
Cobl	1.1359E-182	7.29E-09	0.7152	-0.16526137	-0.064173762	0.02815341
Hpf1	1	4.02E-09	3E-31	0.001134111	0.024210885	0.028151893
Vps51	1	1.00E-05	3E-28	-0.002003508	0.021065695	0.028147727
Phkg2	1	8.19E-03	6E-08	-0.000458825	-0.02326867	0.02813399
Inpp5d	1	5.02E-302	4E-51	0.001275512	0.162624639	0.028119178
Lmf2	1	3.65E-07	4E-37	0.002798646	0.018952364	0.028106672
Tufm	1	4.21E-08	4E-42	0.004023628	0.018643333	0.028099581
Zfp740	1	1.00E+00	0.0036	0.018334554	0.018347552	0.028083625
Gm9929	1	1.89E-33	9E-42	0.005888252	0.033127257	0.02806438
Nsmce1	1	7.79E-05	2E-35	-0.001582114	0.016114543	0.028036495
Hras	1	8.48E-30	3E-44	0.004618327	0.030897177	0.028035228
Gm14827	1	4.55E-23	2E-76	0.000614522	0.016954229	0.028034748
Exosc2	1	1.00E+00	2E-21	0.002196943	0.01330333	0.028023318
Gtf2h4	1	1.00E+00	9E-23	-0.001616777	0.012228976	0.028022688
Kif4	1	3.80E-43	2E-89	-0.00119695	0.020866028	0.027999475
Sart1	0.550373059	1.00E+00	2E-11	0.012130462	0.009062032	0.027998068
Lats2	1	3.43E-02	3E-05	0.016305512	0.036624012	0.027982626
Slc35c2	1	1.29E-11	5E-22	-0.006187264	0.03055082	0.02797967
Tbc1d7	1	1.33E-03	7E-41	0.001442024	0.01297551	0.027977779
Eif3f	1	5.22E-04	8E-25	0.002599025	0.019991963	0.027956174
Spsb3	1	6.86E-08	1E-37	5.87409E-05	0.018394594	0.027952427
Erh	1	2.91E-05	2E-22	0.006475401	0.021567964	0.027945197
Trip6	1	1.00E+00	8E-40	0.00261912	0.009177887	0.02793009
Elmo3	0.048516743	1.64E-12	2E-38	0.007357942	0.021554323	0.027889136
Avpi1	1	1.17E-10	7E-60	3.87206E-05	0.014743799	0.027833111
Krr1	0.241328018	1.00E+00	3E-18	0.010033457	0.014695237	0.027829408
Sgsm1	1	1.00E+00	3E-43	-8.50175E-05	-0.001655459	0.027823552
Smco4	1	5.44E-04	8E-14	-0.006222991	0.024809393	0.027818394
Wbp4	1	1.00E+00	1E-07	0.01421378	-0.000190191	0.027811095
2900093K2	1	1.15E-05	2E-36	0.004827982	0.017327979	0.027804239
Efcab7	1	7.20E-10	8E-41	-0.00283602	0.018543248	0.027803332
Nop53	1	1.00E+00	4E-18	-0.001227689	0.01170272	0.027802363
2900052L1	1	4.08E-13	2E-22	0.004206006	0.030140622	0.027798984
Cd2bp2	1	5.16E-08	5E-34	0.001971073	0.018805339	0.027794608
Ttc8	0.130473996	1.00E+00	2E-13	-0.010672202	-0.001765096	0.0277923
Arpc3	1	1.55E-03	4E-13	-0.00149079	0.02817716	0.027782226

Ndufs6	1	2.82E-07	4E-24	0.003879709	0.023984222	0.027772892
Polr2c	1	1.00E+00	3E-23	0.004679812	0.004650316	0.027768872
Ndufc1	1	1.00E+00	2E-22	-0.000466732	0.018058948	0.027752586
Cbr4	1	5.39E-09	3E-20	-0.004070271	0.027240142	0.02775014
Gm17018	1	1.62E-06	4E-36	0.001121669	0.017613776	0.027745098
Pmpcb	1	1.00E+00	6E-12	-0.004972156	-0.00857711	0.027707047
Gm13613	1.17329E-27	1.00E+00	7E-18	0.028365095	-0.009263488	0.027673999
Tmem128	1	3.94E-11	5E-27	0.000116588	0.026095635	0.027668765
Ccdc134	1	7.29E-02	5E-24	0.000227379	0.015472042	0.027635361
Sec23ip	0.12681051	1.00E+00	4E-05	0.019052115	-0.00881193	0.027631179
Gm47352	1	3.56E-24	2E-37	0.000240662	0.032528429	0.027631086
Gm16008	5.75151E-05	3.71E-02	2E-21	-0.010786945	0.017799087	0.027627003
N4bp1	1	1.00E+00	5E-05	0.009752808	0.033867744	0.027621849
Nprl2	1	4.97E-03	4E-33	0.003163262	0.014328373	0.027583439
Tbx6	1	2.75E-36	4E-64	-0.001329914	0.024556136	0.027581474
Ints9	1	1.00E+00	1E-05	-0.007578416	-0.005847432	0.027580597
Ankrd42	0.000105006	1.00E+00	9E-26	-0.009394989	0.012145193	0.027577747
Gabpa	1	1.00E+00	1E-07	0.007243789	0.005004217	0.027574551
Commd8	1	1.93E-07	2E-27	0.003575965	0.02218645	0.027564995
Rftn1	1	1.64E-270	2E-49	-0.000793415	0.161661512	0.027559159
Gm6288	1	2.06E-59	8E-75	-0.001496462	0.029706315	0.027553872
D030028A(	1	2.05E-23	1E-24	0.000924842	0.037283547	0.027546874
Gm11655	1	2.68E-51	7E-80	0.000526957	0.025357035	0.027538779
Rcn2	1	6.53E-26	1E-47	-0.003532498	0.025040353	0.027522195
Cbl	1	3.42E-10	0.002	0.005016949	0.083387773	0.027522118
Gm15787	1	1.00E+00	4E-11	0.003995252	0.011453684	0.027521633
Thnsl2	0.63161785	1.00E+00	1E-14	-0.00857633	-0.004696992	0.0275177
Wnk3	1	1.04E-61	9E-32	-0.003371802	0.054282781	0.027506349
Cep95	1	1.00E+00	6E-06	0.009559869	-0.011387468	0.027495865
Rragd	1	2.32E-142	8E-106	-0.000357218	0.039220814	0.027493633
Srfbp1	1	6.11E-01	2E-16	0.000960011	0.016543861	0.02746399
0610012G(	1	3.97E-03	5E-42	0.000673811	0.012338327	0.027456297
Gm26749	2.48804E-14	7.28E-14	2E-19	0.020187039	0.033542286	0.027454291
Crb3	1	4.70E-14	3E-32	-0.001073777	0.024596474	0.027443453
Armc8	0.000275379	1.00E+00	6E-06	-0.018638432	0.028044329	0.027440591
Cenpp	1	1.00E+00	6E-07	-0.003708003	0.005083386	0.027425955
Vps33a	1	2.29E-03	4E-06	-0.001582772	0.036377077	0.027413713
Bpnt1	1	1.00E+00	8E-16	0.006581254	0.015147625	0.027402895
Usp12	1	7.84E-03	0.0049	-0.009810952	0.049893901	0.027384268
Slc9a9	1	1.12E-114	3E-24	5.06128E-06	0.12828347	0.027383251
Glmp	1	2.90E-01	1E-33	0.006333418	0.012514659	0.027381976
Ptpn6	1	9.15E-71	7E-30	0.002525288	0.069750577	0.027370439
Elp5	1	1.00E+00	2E-10	-0.005116975	0.007784263	0.027359997
Vsig4	1	1.88E-93	3E-74	0.000913325	0.04564038	0.027330786
Zscan25	1	3.53E-03	6E-21	-0.001956266	0.018907244	0.027329637
Zfp944	1	1.00E+00	1E-09	-0.004223124	-0.001501972	0.027326234
Usp34	5.67031E-06	1.00E+00	1	0.031228232	0.003507015	0.027322952

Itga6	1	2.69E-34	1E-53	0.000342608	0.029590007	0.027307644
Dclre1b	1	1.00E+00	1E-26	0.003599968	0.010146579	0.02730541
Gm14295	1	3.37E-16	1E-60	-0.000909985	0.017040566	0.027291051
N4bp3	1	5.12E-08	2E-48	-0.000850186	0.015386846	0.027280909
Cystm1	1	4.09E-18	1E-27	0.000564878	0.02996296	0.027274007
Telo2	1	2.67E-17	4E-37	-0.001887272	0.025152245	0.027258305
Nsun3	1	1.00E+00	3E-12	-0.008771802	0.008195753	0.027228856
Slc35f2	1	1.21E-69	4E-83	-0.000294196	0.028462349	0.027214702
Dnaaf5	1	1.00E+00	4E-14	0.007873752	0.013102136	0.027199183
Cacng4	1	1.99E-100	2E-118	3.39637E-05	0.023433152	0.027179409
Ssna1	0.107531596	3.95E-17	2E-42	0.006324215	0.023053021	0.027179129
BC025920	1	6.40E-89	4E-60	-0.000554692	0.05827215	0.027158474
Srprb	0.05321119	1.00E+00	2E-21	0.009705785	0.003594013	0.027155678
Dlx1as	1	1.97E-40	2E-80	0.001270883	0.021297459	0.027144699
Gadd45gip	1	4.86E-03	2E-39	0.001579466	0.012850075	0.027125373
Rnf44	1	1.00E+00	1E-08	-0.000121717	0.016173171	0.027125177
Rnf121	1	1.00E+00	5E-07	-0.003525337	0.007765309	0.027122294
Ttll11	1	5.70E-05	2E-32	-0.002474362	0.017819813	0.027118282
Adam12	1	3.94E-16	5E-21	0.000411844	0.03205031	0.027106833
1810030O(	1	1.00E+00	1E-24	0.003429657	0.003760152	0.027103832
Fbxo25	1	1.64E-04	2E-21	-0.00101496	0.020439927	0.0270998
Gm5165	1	1.00E+00	2E-10	0.005363724	0.002860465	0.027094688
Tex264	1	1.00E+00	3E-09	-0.01027968	0.004051898	0.027091366
Top3b	1	2.19E-02	1E-05	-0.006536565	-0.02584432	0.027083173
Nr3c2	2.4874E-131	1.00E+00	1	-0.182374427	0.058835487	0.027068799
Mmp19	1	1.00E+00	8E-12	-0.004210359	0.008297994	0.027065678
Psma8	1	1.94E-50	1E-61	-0.000913104	0.030307027	0.027038541
Tomm22	1	2.47E-06	3E-36	0.00414246	0.0174623	0.026991351
Ruvbl1	0.147615758	1.00E+00	7E-09	-0.012785211	0.009051352	0.026983038
Bcr	4.65276E-05	3.14E-29	1	-0.028213825	-0.091245534	0.026962798
Il18bp	1	1.33E-18	1E-47	0.002577442	0.023327482	0.026942695
Gm49336	1	1.00E+00	3E-08	-0.001003726	0.022271634	0.026935821
Mlxip	1	3.38E-10	9E-06	0.001813174	0.068368637	0.026925119
Sox5os4	3.44007E-06	1.14E-31	4E-30	0.011327961	0.040336886	0.026904687
Ndufaf8	1	1.77E-26	1E-46	-0.001428301	0.025861524	0.026892812
Gm48767	1	9.98E-11	8E-71	-0.001223804	0.011924361	0.026888275
Ppia	0.700779208	2.19E-03	2E-07	-0.011025534	0.033981573	0.02688515
Mrps30	1	4.01E-14	3E-39	7.10435E-05	0.022019546	0.026883688
Uchl3	1	1.00E+00	2E-11	-0.002731449	0.019288112	0.026878135
Cep76	1	2.49E-25	6E-32	0.002115092	0.032408438	0.02686224
S100a10	6.9902E-38	1.00E+00	8E-11	0.039507404	0.018799178	0.026853075
Snrpb2	1	5.55E-12	1E-38	0.003894409	0.020891891	0.026845364
Csnk2b	1	1.00E+00	4E-20	0.008008233	0.006440924	0.026824158
Fam3a	1	1.00E+00	1E-24	0.004978231	0.007183183	0.026819368
Polr1c	1	1.72E-07	5E-35	0.001431697	0.018018818	0.026801239
Ubald1	0.002841519	2.18E-08	1E-25	0.009662951	0.02225783	0.026782809
Kars	1	1.19E-10	4E-27	0.000271766	0.023461018	0.026781813

Dcaf4	1	1.82E-09	1E-49	-0.002596425	0.01536231	0.026779141
Pgp	0.729834458	2.85E-08	1E-31	0.006364009	0.019909301	0.026776324
Cdc37	1	1.00E+00	2E-10	-0.002608269	0.019778159	0.026754092
Ttpal	1	4.44E-03	8E-14	0.00130177	0.024075206	0.026733571
E130317F2	1	1.02E-12	3E-45	-0.000277651	0.01825331	0.026719255
Nt5c3b	1	2.90E-24	8E-78	-0.000600577	0.016651604	0.026719029
Prmt5	0.292149985	1.00E+00	2E-18	0.009547874	0.01480811	0.02671735
Csf3r	1	3.74E-273	4E-57	-0.000573977	0.139094176	0.026716381
Pask	1	1.30E-35	6E-64	-0.001860584	0.023589531	0.026714432
Tex9	1	3.82E-05	2E-50	0.001366694	0.011985714	0.026701721
Tmem223	1	1.00E+00	2E-26	0.005828302	0.00916078	0.026698795
Snx17	1	1.00E+00	6E-19	0.006811108	0.013814182	0.026676941
Prelid3b	1	7.70E-01	3E-24	-0.000653799	0.013732059	0.026647458
Sntb2	7.99994E-05	1.00E+00	0.0043	-0.022744361	0.033198108	0.026647108
Rtca	1	2.00E-04	9E-28	0.002762122	0.017030486	0.026636358
Cog1	1	1.00E+00	7E-12	0.008591572	0.017548352	0.026630364
Tubgcp4	1	3.66E-12	1E-07	0.002318479	0.052622796	0.02662277
Dnlz	1	2.27E-08	1E-59	0.001847487	0.012202565	0.026613395
Ugt2b34	8.78145E-13	1.00E+00	0.0005	-0.032466281	0.006603775	0.026603869
Nelfb	1	1.00E+00	2E-10	0.006158585	0.007671993	0.026594854
Nipbl	0.016792355	1.00E+00	1	0.022597166	0.053656716	0.026593102
Hmgcs1	1.22448E-40	4.95E-122	1	0.086280073	-0.160787891	0.026588821
Mir100hg	0.054977724	2.59E-03	2E-40	0.004387607	0.012712439	0.02657626
Fam8a1	1	1.19E-01	3E-15	0.003171662	0.018900467	0.026556629
Tefm	1	1.95E-09	1E-33	0.003038906	0.020100321	0.026545433
Gm15601	1	5.61E-17	5E-60	0.000278775	0.015428109	0.026525164
Hsbp1l1	0.730753138	2.18E-13	2E-44	-0.004859542	0.018480764	0.026513864
Snhg10	1	2.32E-104	4E-87	0.001153839	0.035964156	0.026486111
Dph6	1	1.00E+00	0.0007	-0.007897653	0.00060554	0.026483585
Ccdc158	1	1.21E-30	2E-57	-0.00127186	0.023647126	0.02647397
Filip1l	1	3.48E-34	0.0001	0.00341591	0.092699246	0.026452397
Zdhhc13	0.47284199	1.02E-01	3E-10	-0.009804347	0.021309127	0.026445233
Tmed9	2.07119E-05	6.50E-01	4E-07	0.020848831	-0.016921903	0.026381155
Orc6	1	4.04E-04	7E-29	0.001831779	0.016449619	0.026379185
Tigar	1	2.09E-11	3E-19	-8.45474E-05	0.029223266	0.026371817
Ssh3	1	2.19E-09	4E-36	0.000766669	0.019626009	0.026362819
9530052E0	0.00015555	1.00E+00	2E-14	-0.012121785	0.017413537	0.026358233
Samd9l	1	5.97E-28	3E-17	-0.006357534	0.053570985	0.02634321
Chchd5	1	4.20E-14	8E-42	7.2879E-05	0.021243373	0.02633998
Pnrc2	1	4.78E-04	3E-24	0.000627373	0.018843097	0.026319251
Cdk4	1	1.51E-01	2E-26	0.001469033	0.013152704	0.026315526
Mier2	1	3.54E-01	2E-29	0.005939773	0.011537707	0.026310543
Mtfr2	1	4.42E-16	2E-47	1.65799E-05	0.019278934	0.026302205
Snhg3	3.9829E-24	6.41E-20	3E-45	0.015960179	0.022332974	0.026283878
Msl3	1	1.00E+00	2E-08	0.006987201	0.012459982	0.026281539
Gpx2	1	1.10E-129	6E-117	0	0.030418932	0.026244702
Zfp101	1.58147E-05	1.00E+00	4E-06	-0.018586884	-0.015801107	0.026236367

Gm31763	0.001931847	3.00E-36	2E-14	0.012942006	0.059482827	0.026220349
Tmbim1	1	1.82E-04	1E-13	0.007323242	0.025764443	0.026205366
Ankrd11	1	6.93E-14	1	-0.012677598	0.113467143	0.026197328
Gm45890	1	2.71E-08	6E-21	-0.00047496	0.023924974	0.026195012
Mbtps1	1	1.00E+00	0.0014	0.004125071	-0.007246609	0.026181196
Dmap1	1	1.00E+00	3E-26	-0.001980665	0.009486548	0.026176842
Gm15856	1	3.64E-75	2E-114	9.30149E-05	0.018211664	0.026173349
Mrps14	1	1.98E-05	2E-32	0.00206493	0.016917023	0.026170947
Plekha1	0.041125711	5.55E-08	1E-08	0.01354954	0.042635063	0.02615685
Eef1akmt4	1	1.16E-25	1E-48	0.002762233	0.024335016	0.026142704
Fam83h	1	2.31E-01	1E-25	0.000214147	0.012615668	0.026120779
Gm10371	1	7.46E-80	3E-72	0.000568401	0.034478335	0.026100306
Lins1	1	1.38E-06	2E-28	0.001541755	0.01881409	0.026096143
Gm44026	1	5.52E-107	8E-101	0.001310979	0.03132097	0.026088267
Sult1a1	2.7752E-36	8.88E-43	0.0001	0.047725406	-0.0711026	0.026086907
Prss8	1	3.02E-31	6E-83	-0.000446142	0.016832218	0.026061088
Sun3	1	4.80E-48	5E-75	-0.001563705	0.024273235	0.026056058
Gga1	1	1.00E+00	2E-08	0.012536494	0.014377071	0.026048843
Ruvbl2	0.008452838	1.93E-29	3E-45	0.00649817	0.026903889	0.02604807
Smpdl3a	1	4.40E-23	2E-40	0.001141916	0.02749497	0.026037939
Fryl	4.35047E-05	2.65E-18	1	-0.028927927	0.100924476	0.026035843
Coa3	1	1.11E-15	3E-50	0.001706775	0.01847001	0.026034033
Rpa2	1	2.08E-24	1E-45	0.001192607	0.02390284	0.026033486
Ick	1	1.50E-02	0.0054	-0.001373578	-0.030436579	0.026027134
Hus1	1	8.42E-04	1E-29	2.44224E-05	0.014976395	0.026024223
Trit1	1	1.00E+00	0.0024	-0.006112794	0.00141168	0.025992225
Pex13	1.78842E-10	1.00E+00	0.6083	-0.034346337	-0.017790387	0.025984701
Nufip1	1	1.00E+00	8E-12	0.003258998	0.011478671	0.025951425
Mfap1b	1	1.00E+00	3E-11	0.0006762	0.007358821	0.025940815
Rfc4	1	5.78E-49	5E-74	0.00250631	0.02438413	0.025931554
Hip1	1	1.03E-234	6E-43	0.000616062	0.142009072	0.025928687
Zfp346	1	1.00E+00	1E-05	-0.002830912	0.017468438	0.02592365
Ints12	1	2.20E-03	2E-24	0.001144022	0.016900219	0.025904205
Ctdnep1	1	1.00E+00	3E-16	0.001789466	0.014893441	0.025898028
Rnaseh1	1	7.88E-10	8E-29	0.003111363	0.021930294	0.025897327
2610507I0	1	1.00E+00	1E-14	-0.000695949	-0.004179312	0.025886628
Maml2	1	8.63E-139	6E-20	0.004738532	0.146048974	0.025877044
Nagpa	1	4.07E-02	5E-30	0.003996991	0.013352719	0.025874332
Nepro	1	2.24E-08	2E-26	0.005429158	0.021344329	0.025867512
Apoc2	1.08885E-36	3.42E-66	1	0.059074697	-0.113461604	0.025855532
Nras	1	1.00E+00	2E-08	-0.001954274	0.014186084	0.02584789
Scnm1	1	4.72E-01	2E-25	0.006671905	0.012138189	0.025844231
Hnrnpk	1.28108E-12	1.00E+00	0.0023	0.035159574	0.018876545	0.025837597
Pelp1	1	1.00E+00	2E-15	-0.003279738	0.008639291	0.025836079
Sec24d	8.62163E-17	1.00E+00	1	0.061594595	-0.02863066	0.025783317
Emid1	1	1.88E-47	4E-106	-8.91901E-05	0.01402703	0.025723201
Ubxn6	1	1.00E+00	6E-13	0.009186065	0.016907156	0.025705388

Zscan21	1	1.00E+00	0.0002	0.012010823	-0.001437249	0.025705289
Tmsb10	1	5.93E-72	2E-75	0.000972329	0.034004831	0.025699082
BC005624	1	1.00E+00	3E-07	0.004099861	0.008598807	0.025685145
Ybey	1	3.20E-10	6E-42	-0.000410149	0.016923144	0.025679547
Clcf1	1	2.91E-65	8E-91	0.001935096	0.023815915	0.025669833
Fam110b	1	1.48E-01	3E-38	-0.000955507	0.01102584	0.025659713
Rad23a	1	1.87E-02	2E-17	0.009006005	0.018252797	0.025658744
Phf2	1	1.00E+00	0.0003	0.003204316	0.010915023	0.025653411
Ppih	1	1.00E+00	7E-16	-0.001669871	0.015139966	0.025650656
Timm50	1	3.77E-05	2E-32	0.000137266	0.015375929	0.025649872
Dram1	1	7.15E-109	8E-87	-0.000801089	0.041627882	0.025649705
Agfg1	1	1.36E-01	0.0002	-0.001975035	0.035726345	0.025646493
Rhno1	1	1.00E+00	6E-23	0.002280863	0.00722775	0.025634544
Cept1	3.23025E-05	9.51E-08	1	0.034836135	-0.040239095	0.025633228
1700123O2	1	7.70E-04	2E-22	-0.001204763	0.017947728	0.025629116
Plk4	1	1.12E-37	5E-54	-0.001142204	0.026938948	0.025625437
Zbtb41	1	9.59E-08	5E-10	-0.008852403	0.031130758	0.025624892
Cwc15	1	1.00E+00	1E-10	0.005468824	0.017709922	0.025622691
1810037I1	1	5.53E-07	3E-38	0.003748041	0.016463269	0.025619576
Lrp12	0.376420161	3.62E-09	5E-07	-0.012140576	0.053312901	0.025593312
Hmga2	1	4.41E-24	1E-68	-4.00366E-05	0.01238031	0.025589443
Cyren	1	1.74E-04	6E-29	0.005861561	0.016978079	0.025583525
Rab26os	1.00791E-08	2.39E-07	1E-45	0.009449813	0.01403621	0.025577224
Tdp1	0.757605313	2.12E-06	1E-20	-0.006954626	0.022876005	0.025568325
Zswim9	0.667837495	1.04E-15	3E-27	-0.005919172	0.026443808	0.025562539
AC160336.	1	1.33E-03	1E-59	-0.000251064	0.008966506	0.025561045
Nmnat1	1	1.00E+00	5E-09	-0.005255421	-0.000147011	0.025554459
Tatdn2	1	1.15E-05	2E-12	-0.00068598	0.028982801	0.025549834
Grik5	1	6.66E-02	6E-06	-0.004450938	0.028987451	0.025528458
Rab23	1	1.00E+00	7E-18	0.00303208	0.010423549	0.025502955
Zdhhc23	1	2.16E-23	1E-71	-0.00111347	0.01598494	0.025498818
Eqtn	1	3.28E-114	3E-92	0.000648088	0.034916211	0.025480446
Sdcbp	0.000840751	7.87E-09	3E-07	-0.01377147	0.055020826	0.025478631
Cacfd1	1	1.00E+00	1E-15	-0.002183929	0.003013432	0.02547054
Ppat	1	1.00E+00	2E-05	0.009301693	0.016165653	0.02545506
Pramef8	1	2.95E-05	1E-37	-0.002100702	0.014340842	0.025444171
Tmem79	1	3.95E-01	2E-19	0.002282623	0.014765335	0.025441002
Fam118a	1	3.33E-41	1E-76	-0.00070171	0.020700577	0.025424961
Vars2	1	1.00E+00	1E-15	0.000851225	0.00972743	0.025413204
Mterf4	1	1.00E+00	3E-25	-0.000570408	0.011018806	0.025397901
Chek2	7.61139E-06	6.66E-10	5E-27	-0.008742986	0.021796286	0.025395404
Ehd4	1	1.67E-70	1E-45	-0.00090905	0.053238426	0.025392356
Gm16751	1	2.98E-71	1E-60	-0.001657259	0.035266693	0.025382325
Wdr92	1	1.00E+00	8E-11	0.008465267	0.006365225	0.025377993
Crebzf	7.13011E-07	1.00E+00	3E-05	0.023634571	0.013848816	0.025367686
Itga2b	1	1.00E+00	4E-23	-0.005165039	0.009979767	0.025366883
Haus1	1	1.00E+00	5E-19	-0.004365842	0.012832161	0.025359149

Cep41	0.284187616	9.05E-13	6E-38	-0.004910553	0.019729853	0.02535851
Cd74	1	1.68E-188	5E-58	0.000209642	0.085632352	0.025326425
Rnps1	1	1.00E+00	5E-07	0.001213683	0.004868807	0.025310618
Ctsf	1	3.47E-11	6E-27	0.001817312	0.022336042	0.025294768
Gm26747	1	3.43E-83	4E-100	0.000104622	0.022291087	0.025292237
Gm17399	1	2.70E-35	1E-38	-0.001176663	0.032419732	0.025286891
Selenok-ps	1	1.31E-13	1E-50	-0.002896616	0.016321047	0.02528003
Cks2	1.28008E-09	1.00E+00	2E-09	-0.018078936	0.000837588	0.025277861
Ugt1a10	1	8.27E-17	4E-42	0.000275398	0.020804381	0.025240277
Ints11	1	1.37E-03	1E-15	0.002983391	0.021685701	0.025239101
Itgb5	1	1.91E-16	0.0093	-0.005067047	0.080479364	0.025238849
Gmpr	1	5.95E-52	3E-59	0.002430807	0.027946468	0.025237747
Enox1	1	8.96E-19	1E-45	-0.00174305	0.019339966	0.025210982
E530011L2	1	4.96E-03	7E-39	0.004202947	0.011407193	0.025205859
Prrg4	0.387559534	1.54E-22	2E-68	0.003343697	0.015464448	0.025170979
Tmem268	1	5.94E-36	2E-45	-0.001415254	0.030427984	0.025170344
Sun1	0.292007809	1.00E+00	4E-06	-0.012589117	0.002583877	0.025155077
Faap24	1	4.44E-34	4E-73	-0.000491686	0.019172592	0.025138429
Chmp2a	1	7.11E-07	1E-19	0.001462611	0.025301075	0.025137856
Myh14	7.47239E-08	1.00E+00	1E-09	-0.018674526	0.00195816	0.025129941
B230118H(	2.76304E-09	1.00E+00	2E-10	-0.018135009	0.003734048	0.025120654
Ap3s2	1	1.00E+00	0.0002	0.002439034	0.022916508	0.02509274
Npnt	1	8.57E-01	3E-34	-0.000677781	0.008805426	0.025083539
Ccnk	1	1.00E+00	5E-06	0.000542816	-0.009504977	0.02508352
9530051G(	1	1.44E-36	7E-66	-0.001285466	0.021078669	0.025074917
Slc37a3	1	5.98E-01	8E-06	-0.000714521	-0.01736586	0.025074183
Ereg	1	5.76E-47	1E-68	5.36154E-05	0.018580342	0.02507045
Ilk	1	4.88E-02	1E-14	0.00493541	0.019840399	0.025064272
Tinf2	1	8.31E-01	5E-29	-0.001951035	0.010397221	0.02505608
Dusp13	1	1.09E-37	3E-88	0.000704409	0.015339898	0.025048692
Dnajc4	1	9.76E-04	3E-30	-0.002346953	0.013946598	0.025015138
Ddx19a	1	1.00E+00	4E-12	0.000251421	0.00563683	0.025010337
Sgsh	1	1.72E-18	3E-63	-0.000715828	0.015240195	0.025003368
Neil1	1	2.84E-03	3E-29	0.001442052	0.014137017	0.025002015
Ikbip	1	6.90E-12	1E-56	0.000393124	0.014575583	0.0249945
Gm36371	1	1.39E-01	3E-34	-0.001053529	0.010611149	0.024993625
Slc22a7	4.19916E-11	1.83E-01	3E-27	-0.009498428	0.01194828	0.024991888
2310039H(	6.39586E-14	1.00E+00	1E-22	0.016951935	0.008098407	0.024987532
Tmem202	1	9.20E-102	5E-98	-0.00024021	0.031191513	0.024963372
1700087I2	0.006964143	1.00E+00	4E-23	-0.007834511	0.005802243	0.024951623
Cd82	1.37773E-06	1.00E+00	0.0078	-0.021050149	0.026320564	0.024922048
Mok	1	1.08E-41	2E-76	0.000114316	0.019999968	0.024920932
Per1	9.48546E-64	1.00E+00	4E-12	0.049103552	0.016556476	0.024918518
Bcas2	1	1.98E-01	1E-11	0.00170066	0.021168584	0.024907988
Leng8	6.51519E-06	1.00E+00	0.0014	0.025624165	0.011218595	0.024882326
Ndufa3	1	6.06E-01	2E-15	0.005411845	0.018215407	0.02487178
Ier3	1	1.70E-92	7E-96	-0.000222194	0.028418276	0.024853702

Prelid2	1	7.82E-51	3E-44	-0.000490287	0.034063299	0.024843495
Aldh16a1	1	1.00E+00	6E-05	-0.002351855	-0.009028542	0.024838962
Ccdc124	1	8.36E-07	1E-25	0.001488524	0.01884361	0.024825442
Nmral1	1	2.43E-18	8E-56	0.002078558	0.016541103	0.024816838
Gmpr2	1	3.46E-12	2E-41	0.001689553	0.019030103	0.024809531
Il17ra	1	1.73E-14	1E-12	-0.000903956	0.039000979	0.024807093
Ino80b	1	2.55E-07	3E-17	0.002424255	0.023987849	0.024804849
Flcn	0.224743925	1.49E-11	0.0005	0.015337702	0.053212437	0.024804134
Gm4117	1	7.81E-35	5E-41	0.002369391	0.02835361	0.024799868
Anxa2	1	1.34E-77	1E-64	-0.001071511	0.037146743	0.024782011
Gm15910	1	1.02E-76	1E-90	-0.000316014	0.024222746	0.024779112
Oxld1	1	3.27E-19	1E-41	0.000277469	0.021639494	0.024723947
Dpy19l4	1.10316E-10	1.00E+00	0.0004	-0.026929008	0.002887903	0.024721277
Gmfb	1	7.31E-06	1E-07	-0.010537069	0.033669242	0.024707158
Bbof1	1	6.53E-04	2E-19	-0.002522989	0.017670357	0.024701814
Rft1	0.056965343	1.00E+00	9E-06	-0.015106445	0.011066643	0.024699828
Trim6	1	4.09E-28	3E-95	0.000553268	0.010857794	0.024691069
Mcmbp	0.013684485	1.00E+00	0.0145	0.020415218	0.031583471	0.024678648
Myof	1	0.00E+00	2E-73	0.000374885	0.143124602	0.024676969
Zfp518b	0.856743151	7.26E-31	8E-58	-0.003054625	0.021350206	0.024670316
Zfp839	1	1.00E+00	2E-05	0.011962634	-0.004577764	0.024668548
Rpgrip1	1	1.00E+00	2E-20	-0.006432839	0.010758967	0.024656318
Prdx2	1	1.61E-02	2E-14	-0.004785156	0.020540262	0.024649219
Snrpd2	1	9.63E-21	4E-40	-0.000806243	0.023503223	0.02462962
Rabepk	1	1.00E+00	9E-19	0.006160041	0.010202222	0.024628024
Ndufb7	1	1.15E-02	6E-16	-0.006176266	0.020425675	0.024626141
Ndufv2	1	1.00E+00	0.0004	0.004832851	0.009911879	0.024625192
Eif4ebp3	2.70969E-19	1.00E+00	4E-09	0.029640883	-0.011143671	0.024620027
Plpp1	1.66471E-07	1.00E+00	6E-10	0.014089793	0.031003305	0.024614894
1810044D0	1	1.68E-31	9E-55	0.00110214	0.023513565	0.024613733
4833418N0	1	1.00E+00	1E-14	-0.00095198	0.014312379	0.024601356
Asna1	1	8.38E-03	4E-22	0.005319019	0.015233499	0.024594517
Fam98b	1	1.00E+00	8E-16	0.002837551	0.007156332	0.024587058
Fuom	1	1.14E-02	2E-25	-0.002603083	0.014512139	0.024584926
Rgl1	1	1.59E-123	2E-28	0.001272696	0.119378167	0.024571623
Mab21l3	1	1.46E-38	1E-57	-0.00024195	0.024136724	0.024569363
Gm11762	1	1.09E-58	7E-110	0	0.01347796	0.024547926
Ndufs7	0.308086031	1.00E+00	3E-10	0.011506883	0.008879203	0.024547822
Gtpbp10	1	5.83E-05	3E-13	-0.002129806	0.023582485	0.024543783
Tbccd1	1	1.00E+00	0.0002	-0.002268419	-0.002351911	0.024543157
Lyrm4	1	1.00E+00	1E-08	-0.003699905	0.006266196	0.024528242
Zfp763	1	1.48E-01	1E-18	0.001829087	0.013816971	0.024516035
Srp9	1	1.00E+00	1E-15	0.006983333	0.007366119	0.024494048
Ndufa5	1	5.35E-02	4E-16	-0.000616161	0.017453508	0.024487197
Dpm3	1.24452E-08	2.01E-12	1E-39	0.009525909	0.018577756	0.024475312
Lrrc57	1	1.65E-05	2E-20	0.002816335	0.021123453	0.024474464
Usp46	1	1.00E+00	0.0001	0.001376037	0.023809545	0.024470741

Zfp142	1	1.00E+00	5E-12	0.005532514	0.008570809	0.024468278
Afg3l1	1	1.00E+00	0.0041	0.006933837	-0.009420364	0.024462125
Dpagt1	0.011528962	1.00E+00	1E-13	0.011571578	0.002166088	0.024455552
933016201	3.54336E-10	1.00E+00	7E-07	-0.021174848	0.000772335	0.024446844
Ddost	1	2.26E-04	3E-37	0.002832751	0.012459268	0.024441741
Slc37a1	2.07958E-11	6.10E-31	1E-30	-0.010286613	0.034647782	0.024440757
Akr1b10	1	7.80E-62	5E-94	-0.000614591	0.021575917	0.024427283
Nhp2	1	1.31E-37	4E-59	-0.001264128	0.023755249	0.024421045
Smad6	1	1.52E-01	1E-08	-0.003714825	0.029581482	0.024411413
Pkd1l1	0.439889854	4.92E-113	6E-80	0.00256317	0.037408736	0.024411113
Det1	1	1.26E-11	5E-23	0.002143548	0.024643469	0.024392155
Hscb	1	9.06E-13	2E-28	0.001815214	0.023167195	0.024365891
Rcn1	1	1.01E-66	8E-86	0.000482925	0.024447494	0.024361155
Kif9	1	9.65E-19	1E-34	-0.000850583	0.024796403	0.024353677
Mcoln1	1	3.16E-07	2E-11	-1.03416E-05	0.030807491	0.024346246
Thap7	0.049308708	1.15E-03	2E-26	0.007614341	0.014183485	0.024342991
Alg3	1	3.33E-17	6E-55	-0.000291595	0.016032786	0.024341906
Adgre1	1	1.21E-141	2E-45	-0.000715734	0.083931754	0.024314932
Nbn	1	1.00E+00	1E-08	0.008792788	0.008543286	0.024313439
Itpa	1	1.00E+00	4E-14	0.004397719	0.011320289	0.024305197
Pigs	1	1.00E+00	9E-17	0.00017771	0.011094418	0.024300328
Accs	1	2.31E-02	6E-35	-0.002902673	0.011211052	0.024288537
Pin4	1	1.00E-05	1E-29	0.003058293	0.016880049	0.024286848
Smim13	1	1.00E+00	0.0328	0.001027461	-0.01464566	0.024275789
Mta1	1	1.00E+00	3E-05	-0.004523663	0.004480224	0.024273308
Ctnnal1	1	1.00E+00	4E-16	-0.004997676	0.012772021	0.024256542
Aoah	1	6.97E-171	1E-58	0.002156487	0.094407643	0.02425287
Rpf1	1	2.40E-01	2E-12	0.00844626	0.017875725	0.0242505
Ppp2r3d	0.000967406	2.74E-07	2E-11	0.014191631	0.032901819	0.024243411
Cct7	1	8.21E-01	6E-14	-0.006873472	0.017346091	0.024238382
Wipf1	1	7.78E-206	2E-34	0.001950205	0.129806241	0.024232849
Wdr7	4.52299E-06	3.26E-25	1	-0.037052276	0.115172306	0.024228766
Zkscan14	1	1.00E+00	2E-23	-0.004393803	0.010096659	0.024223419
Vav3	0.581125404	1.04E-198	3E-33	0.004238946	0.140071475	0.024207352
Gm15511	3.80935E-06	2.68E-19	6E-21	0.012195366	0.032580134	0.02420445
Ube2cbp	1	1.00E+00	2E-06	-0.000881724	0.010029514	0.02419674
Pam	0.021880243	1.48E-35	3E-11	0.010469476	0.061716776	0.024191604
U2surp	2.57024E-07	1.59E-08	1	0.035464473	-0.052049997	0.024183681
Unc50	1	3.68E-15	3E-19	0.003550781	0.032242776	0.02417414
Plekha7	3.39433E-06	9.41E-01	1	-0.031239667	-0.022896459	0.024159456
Itga3	1	1.22E-02	5E-20	-0.004949955	0.015955974	0.024154635
Eml3	1	1.00E+00	2E-05	0.005744596	0.000271339	0.024152505
Fahd2a	1	1.58E-05	8E-25	0.003448476	0.016756197	0.024145295
Ift46	1	5.92E-01	1E-06	0.001696462	0.023912629	0.024135253
Hnf1a	1	1.00E+00	1E-06	-0.00583817	0.004191528	0.024129558
2410004B1	1	1.00E+00	2E-21	0.005057895	0.012459676	0.024119718
Psmb5	1	9.43E-02	3E-20	-0.000297765	0.015495577	0.024081236

Coro2b	1	2.42E-60	1E-56	-0.001658161	0.034173838	0.024079314
Cad	1	3.03E-01	2E-31	0.004241591	0.010528476	0.024047821
Bap1	1	1.00E+00	6E-18	0.003151627	0.005423387	0.024046851
Zbtb17	1	1.00E+00	3E-12	0.003272427	0.006138997	0.024026473
Man1c1	1	1.16E-219	8E-45	7.64323E-05	0.117985755	0.024021367
Rbm10	0.514973527	1.59E-03	0.2744	0.020770252	-0.031034436	0.024019109
Psmc5	1	1.00E+00	1E-06	0.005778079	0.015924279	0.024018663
Zfp808	1	2.13E-07	2E-42	-0.001444819	0.014838626	0.024011718
Neu3	0.003329177	1.95E-06	7E-40	-0.005444844	0.013647004	0.0240083
Rad51	1	6.79E-21	4E-37	0.000567635	0.024168553	0.024004198
Pebp1	1	3.18E-01	6E-12	-0.005163115	0.019924285	0.023988627
Cenpe	1	8.50E-18	2E-56	-0.000246656	0.013529805	0.023985801
Vnn3	1	1.00E+00	4E-09	-0.000706523	0.009367629	0.023972129
Acp1	1	1.00E+00	4E-13	0.005413103	0.005999495	0.023962771
Ppp1r37	1	5.01E-04	0.0287	-0.014778662	-0.034340297	0.023959647
Dip2a	0.032617607	2.82E-05	2E-20	-0.006878274	0.021761898	0.023924584
Ddx31	1	1.00E+00	4E-05	-0.001444316	0.023273798	0.023922574
Myo5a	1	3.09E-161	2E-41	0.000322741	0.10788253	0.023911492
Odc1	1	4.83E-09	9E-28	0.005344432	0.018909316	0.023895461
Dpm2	0.126671673	5.35E-02	4E-30	0.006714813	0.012570634	0.023892838
Ift74	1	1.00E+00	4E-11	0.002143042	0.001912023	0.023892358
Lrrc14b	1	5.64E-72	3E-55	0.000763307	0.041971338	0.023885064
Grk2	1	3.23E-15	8E-10	0.003697063	0.052586299	0.023853637
Tmem63b	0.000770222	1.00E+00	0.2149	-0.021706489	0.024217588	0.023851496
Supt7l	1	1.42E-05	1E-18	0.001610595	0.019755973	0.023847389
Mfsd14a	1	2.21E-05	0.0038	0.011986286	-0.031640834	0.023824824
Brwd3	1	1.00E+00	0.045	-0.010936707	-0.003320491	0.023792929
Lrrcc1	0.027037848	1.79E-09	1E-24	-0.006670908	0.023152476	0.023756129
Kat2a	1	1.00E+00	1E-16	0.007406875	0.013039196	0.023729099
Chmp1a	1	1.00E+00	5E-13	-0.001603001	0.014959576	0.023712627
Lyrm1	0.367219935	1.00E+00	1E-09	-0.009886905	0.017508939	0.02370966
2810001G2	1	1.01E-01	8E-32	-0.000722529	0.011009658	0.023697725
Gm16278	1	2.42E-06	6E-36	0.001173222	0.014277684	0.023684364
Cenpf	1	3.06E-03	2E-57	-0.000442019	0.005618697	0.023678681
Mus81	1	1.00E+00	6E-20	0.00457638	0.011327541	0.02367694
Ndufaf1	1	1.00E+00	4E-23	-0.004292211	0.006573612	0.023673511
Tubb6	1	1.95E-43	2E-58	-0.000765524	0.025110387	0.023673308
Ddr1	1	1.25E-90	8E-90	0.000177131	0.02704497	0.023666806
Gm9895	1	1.00E+00	7E-35	-0.002208836	0.008411411	0.023662865
Ndufs4	1.16917E-12	6.63E-01	1	-0.043054577	-0.027677455	0.023661976
Fam173a	1	4.98E-02	8E-22	0.003294238	0.014742392	0.023652836
Uxt	1	1.00E+00	1E-17	-0.002519966	0.007866902	0.02363371
Gm11755	1	1.09E-20	9E-92	-0.000243866	0.007188992	0.023612443
Fance	1	1.00E+00	1E-07	0.008346028	0.009304475	0.02360416
Fam76b	1	1.00E+00	0.006	0.00903192	0.002355026	0.023571087
Rsph3b	1.06998E-08	1.00E+00	0.0003	-0.022298938	0.022376755	0.023549065
Ift140	0.00017006	1.00E+00	0.0005	-0.017242654	0.005869685	0.023547283

Mrpl41	1	4.92E-11	1E-41	0.004214332	0.016719792	0.023542147
1810024B0	1	1.04E-05	2E-14	0.003270888	0.023680312	0.023538394
Hspa13	0.001373528	1.00E+00	2E-27	0.008521387	0.003153932	0.023533686
Cmc1	6.10177E-11	1.00E+00	0.033	-0.032666782	0.015807784	0.02352166
Knl1	1	1.87E-09	3E-51	-0.001222483	0.010119484	0.023520935
Poldip3	1	1.00E+00	3E-05	0.000845246	0.021342923	0.023519308
Mrpl22	1	3.24E-04	5E-20	0.007397988	0.017409177	0.02351513
Mtmr9	1	5.77E-05	1E-20	-0.001431046	0.018318547	0.023507553
Pdap1	1	1.00E+00	8E-12	0.001699394	0.01551378	0.02350121
Efcab14	0.000224647	1.00E+00	4E-05	-0.017224991	-0.000682869	0.023482595
Slc39a9	1	1.00E+00	0.0047	-0.000280777	-0.016020482	0.023482557
Ciao2a	1	9.81E-03	2E-14	0.005922511	0.019038672	0.023478268
Gm14023	1	1.48E-15	3E-40	-0.000785087	0.018594169	0.023473554
Psmb6	1	1.00E+00	1E-18	0.002725542	0.009734468	0.023472698
Sbf1	0.007649601	1.00E+00	6E-06	0.017049386	-0.005992594	0.023470433
D8Ertd738	1	1.00E+00	3E-14	0.007939911	0.012085184	0.023469867
4933434E2	1	1.00E+00	0.0004	0.012335698	0.016538501	0.023456886
Gm12258	1	1.00E+00	4E-19	0.001910994	0.012778112	0.023452345
Cryzl2	0.012465752	1.00E+00	1E-12	-0.010249465	0.006988387	0.023448113
Mrps16	1	3.74E-12	1E-31	-0.001178584	0.019007442	0.02344621
Mrps17	1	1.00E+00	4E-09	-0.00327164	0.010681396	0.023434428
Gan	1	1.00E+00	0.0033	-0.003537396	0.006176014	0.023429741
Ddx39	1	6.97E-06	7E-19	0.002332831	0.021098827	0.023428462
Erich1	1	1.00E+00	3E-06	-0.002882304	-0.013429388	0.023427792
Akip1	0.541330809	5.65E-07	4E-22	-0.006052814	0.01943934	0.023398435
Pdlim7	1	1.81E-01	3E-11	-0.005217402	0.020280138	0.023389817
9530034E1	1	3.02E-08	1E-20	-0.005075261	0.021193013	0.023385693
Tfip11	1	1.00E+00	4E-27	0.004559739	0.005980834	0.023358206
Akt1s1	1	1.00E+00	8E-10	0.00223793	0.004972125	0.023356679
Stk17b	1	3.62E-25	3E-23	-0.001647727	0.039571157	0.023353914
Rab1b	1	3.58E-08	3E-20	0.003736357	0.024072133	0.023338666
Elf4	1	6.69E-158	7E-59	-0.000855932	0.069742756	0.023336132
Etaa1	1	2.89E-13	7E-14	0.000503395	0.031187205	0.023333129
Uevld	0.323961	1.07E-01	2E-07	-0.010713401	0.024229561	0.023324903
Jcad	0.007476902	5.18E-16	5E-54	-0.003665164	0.015635001	0.023318881
Tusc3	1	2.57E-06	2E-15	-0.005418772	0.02556435	0.023302533
Hgh1	1	6.56E-26	2E-44	0.001400008	0.022654223	0.023300146
Anp32b	1	3.48E-12	5E-07	0.004773977	0.0445963	0.023296401
Lmntd2	1	1.00E+00	9E-38	0.001871976	0.0041616	0.023262655
Prc1	1	1.74E-25	6E-70	-0.000998437	0.013011767	0.023251569
Spcs3	1	7.73E-01	2E-14	0.000647671	0.015188935	0.023247849
Ybx1	1	1.00E+00	0.0094	0.003341132	0.017163671	0.02324386
Zfp738	1	1.00E+00	2E-06	-0.002097258	-0.002344707	0.023242127
Aim2	1	6.07E-143	8E-56	-0.000402593	0.068652919	0.023235752
Mis18bp1	1	1.93E-52	6E-78	0.000748247	0.021236058	0.023234379
Flot1	1	1.00E+00	5E-18	-0.006159051	0.008731191	0.02323159
Rdm1	1	6.93E-10	6E-12	-0.008225167	0.031906424	0.023221834

Hirip3	1	1.45E-11	3E-29	0.000169011	0.020113092	0.02321867
Armc7	1	1.00E+00	3E-13	-0.000504429	0.011819404	0.023215282
Set	1	1.35E-11	4E-06	0.006474831	0.044322471	0.023211944
Gal3st1	1	6.46E-01	6E-19	-0.002907475	0.011761144	0.023197553
Chmp4c	1	2.07E-13	2E-30	-0.003289139	0.020695115	0.023186836
Ces2e	3.74873E-12	8.37E-04	7E-22	-0.011612956	0.016013185	0.023175004
Hsp90b1	1.03688E-24	2.05E-48	1	0.066124172	-0.137469807	0.023174354
Rsf1os2	1	2.74E-35	7E-19	0.005055742	0.045028749	0.023157612
Cc2d2a	0.127324672	1.00E+00	2E-14	-0.008875196	0.012591758	0.023139702
St8sia3	1	3.83E-21	7E-50	-0.002752608	0.018561214	0.023133909
Copz2	1	1.00E+00	9E-17	-0.002599117	0.008810637	0.023123948
2310009A0	1	8.91E-20	4E-49	0.003688941	0.018552605	0.023121974
Supt4a	1	3.24E-03	6E-20	0.001676168	0.015746022	0.023101337
Uqcrfs1	1	6.05E-04	3E-17	0.000407299	0.020597132	0.023099281
Prss36	1	1.91E-19	7E-37	0.001869221	0.021380661	0.02308823
Mknk2	0.001370509	1.00E+00	3E-05	0.016353669	-0.00569016	0.023087878
Grn	8.04854E-25	9.71E-05	3E-06	0.034658344	0.04359034	0.023083034
Lcp2	1	1.44E-219	1E-51	0.001582472	0.099577771	0.023074208
Gnai2	0.000580876	6.67E-16	9E-08	-0.012835577	0.049472917	0.02306422
2200002D0	3.0631E-05	6.08E-13	7E-41	-0.005603322	0.017248307	0.023063712
Acp2	1	1.00E+00	0.0002	-0.008059248	-0.007447082	0.023056496
Morn4	1	4.75E-13	4E-28	-0.004819485	0.020926322	0.023041804
Mpi	1	1.28E-03	2E-37	0.000215687	0.010719248	0.023041215
Soat1	1	3.33E-283	5E-59	3.36878E-05	0.1185069	0.0230266
Angpt2	1	3.34E-23	4E-33	-0.00194871	0.025369137	0.023010386
Mrpl16	1	2.66E-17	8E-38	-0.002497245	0.020740751	0.022987853
Plpp2	1	1.07E-09	6E-49	-0.000437491	0.01247216	0.022985264
Gm28379	0.013113319	5.77E-22	3E-34	0.006312827	0.023198992	0.02298122
Grpel1	1	7.41E-02	4E-14	-0.001321263	0.017244655	0.022966996
1110008P1	1	2.82E-18	2E-53	0.003517173	0.015609147	0.022958393
Spint1	1	5.18E-154	5E-100	-4.73494E-05	0.036351382	0.022923476
Zfp991	1	1.40E-22	1E-43	-0.001270079	0.024832654	0.022922403
Smim8	1	1.61E-07	2E-35	0.000348618	0.014387837	0.022912871
Jpt2	1	3.43E-03	1E-21	0.001006031	0.015177574	0.022911646
Gm49984	1	6.00E-144	2E-89	0	0.038443302	0.022903482
Gipc1	0.118021118	5.30E-16	4E-23	-0.006532968	0.027042585	0.022902633
Sag	1	5.52E-87	1E-70	0.000238276	0.033001303	0.022896145
Mybl2	1	2.87E-24	4E-35	-0.003590305	0.024315123	0.022875482
Napg	0.000192803	1.00E+00	0.0045	0.021377281	-0.000818055	0.022863092
H2-T23	1	1.00E+00	4E-12	0.002805381	0.008912047	0.022861011
Miip	1	1.00E+00	9E-08	0.010920845	-0.013554832	0.022829909
Sec11c	1	1.00E+00	4E-15	0.007511331	0.005923445	0.022824716
Ginm1	1	2.65E-05	1E-09	0.001466044	0.027417817	0.022820878
Mcrip1	1	5.98E-03	1E-17	0.000418119	0.017861547	0.022816251
Rela	1	1.00E+00	0.0012	-0.003645296	-0.001516864	0.022813156
Twf1	1	1.00E+00	2E-10	0.005653165	0.013499927	0.022804435
Mib2	1	1.00E+00	2E-07	0.000480171	-0.003566421	0.022802281

Trim37	1	1.13E-07	0.0001	-0.005242088	0.043662328	0.02279121
BC024139	1	2.22E-03	3E-35	-0.002576602	0.010639619	0.022768332
Urb2	1	9.62E-06	3E-12	-0.001183465	0.023542223	0.022761276
Cd44	1	2.93E-193	4E-43	-0.000834498	0.107039665	0.022753028
Fli1	1	1.66E-153	3E-21	0.001920312	0.11425169	0.022743685
Lrrc8b	1	5.78E-69	8E-64	-0.001279043	0.028956334	0.022735269
Tbp	1	1.00E+00	1E-08	0.003152258	0.008901589	0.022726151
Sf3a2	0.140191666	1.26E-05	1E-14	0.00954911	0.02219143	0.022722803
Nmb	1	1.10E-14	2E-35	0.001437377	0.019340082	0.022711081
Imp4	1	3.72E-06	5E-21	0.006062787	0.020521281	0.022710102
Psme2	1	8.47E-12	9E-16	0.005995571	0.03293096	0.022682959
Gm2415	1	2.30E-07	3E-69	-0.000586506	0.007773735	0.022678995
Gm47904	1	7.99E-11	1E-29	-0.000803351	0.018364488	0.022663107
Dera	5.4002E-21	3.93E-02	0.0578	-0.041789358	-0.030284893	0.022660956
Ciao1	1	1.00E+00	5E-11	-0.002444296	0.007362147	0.022656324
Ung	0.720853233	1.69E-12	3E-50	0.003995394	0.013659459	0.022635966
Olfr267	0.016429884	1.60E-02	6E-41	-0.004103477	0.010598628	0.022629931
Calcoco1	1	1.00E+00	0.0009	0.005045905	0.005142113	0.022627153
Csf2ra	1	1.69E-161	9E-63	0.000536046	0.066368471	0.022626354
Arhgap31	1	2.66E-134	7E-26	0.00309265	0.101967465	0.022622623
1110059G1	1	2.98E-01	2E-19	-0.004630599	0.01304996	0.022611492
Gripap1	0.001351821	3.94E-07	1	0.029347755	-0.041156465	0.022601625
3930402G2	1	3.30E-04	3E-41	0.004091574	0.010631779	0.022595582
Actr5	1	1.00E+00	6E-11	0.000294197	0.002165739	0.022593973
Cdk16	1	1.00E+00	6E-05	0.003594666	-0.006328431	0.022583146
Wwc1	0.000281746	7.47E-08	1	-0.039362265	-0.066720888	0.022576883
Rnaseh2b	1	2.59E-22	5E-28	-0.002509444	0.02823894	0.022553646
Cic	1	1.00E+00	7E-07	0.005080267	0.007435837	0.022527815
Commd4	4.47168E-06	1.00E+00	3E-18	0.011796177	0.01316596	0.022512015
Zfp146	0.055254682	1.00E+00	5E-05	-0.012240843	-0.004866973	0.022501597
Mfsd4b4	1	1.36E-08	2E-17	-0.001259243	0.023844768	0.022500536
Pus7l	1	2.99E-17	6E-37	-0.000486102	0.020298111	0.022469535
Map9	1	5.47E-17	2E-51	-0.000462581	0.014853354	0.022455342
Zfp182	1	1.00E+00	0.0477	0.001580738	-0.004658756	0.02244488
Slc26a11	1	3.10E-08	6E-05	-0.002075869	0.04922785	0.022419379
Tmsb4x	1	5.02E-157	2E-33	-0.002651271	0.085291313	0.022414586
Gm11520	1	2.19E-06	8E-27	-0.001985295	0.016097915	0.022411037
2900005J1	1	1.00E+00	5E-08	0.002330842	-0.006506401	0.02239828
Hmbs	1	1.00E+00	7E-15	0.005221342	0.010571204	0.022390357
Sh3kbp1	1	1.64E-180	3E-27	-0.0002498	0.120432668	0.022375503
Gm3587	1	9.15E-105	2E-92	-0.000284096	0.025399775	0.022373628
2310022A1	1	2.52E-04	6E-17	0.003603818	0.017767462	0.022362893
Fndc4	1	1.00E+00	5E-14	0.003737272	0.004438044	0.022360421
1700066B1	1	1.27E-49	8E-80	0.000573041	0.0186497	0.022359708
Lyrm2	1	2.83E-02	7E-25	0.002212955	0.012721121	0.022359654
Map4k4	2.13467E-80	4.77E-17	1	0.083574044	0.087549545	0.022357516
Ddx51	1	1.00E+00	4E-21	-3.62572E-06	0.010857317	0.022346424

Palb2	1	1.25E-12	6E-27	-0.003943217	0.021894053	0.022345769
Hypk	1	1.31E-15	3E-32	0.004863707	0.020993996	0.02234204
AU019990	1	3.05E-154	4E-89	-9.3748E-05	0.043761196	0.022340124
Pnkp	1	1.00E+00	1E-06	0.002268816	-0.007350892	0.022326021
Lemd2	1	1.49E-08	4E-07	0.008308726	0.036203245	0.022326011
Gm11963	2.38184E-37	1.00E+00	4E-10	-0.025134723	0.013840256	0.022313778
Cdca8	1	3.15E-04	4E-37	-0.001409204	0.011028654	0.022305913
Pdha1	1	1.00E+00	2E-09	-0.000533496	0.004459771	0.022300671
Zfp626	1	1.00E+00	1E-08	-0.005503629	0.007491033	0.022289205
Adamtsl1	1	1.00E+00	2E-28	-0.000966744	0.008364402	0.022284276
Gys1	1	1.00E+00	1E-06	-0.007774348	0.00662742	0.022281985
Mrpl51	1	1.73E-02	9E-16	-0.001807856	0.018253745	0.022281614
Cdt1	1	8.23E-39	8E-62	-0.00060338	0.020676442	0.022257385
Slc25a53	1	2.13E-10	3E-15	-0.006196395	0.0274103	0.022255887
Nop9	1	1.00E+00	5E-13	0.003629309	0.014188327	0.022233105
Chek1	1	1.19E-02	4E-48	0.001130763	0.007756662	0.02221965
Man2b2	1	1.43E-09	2E-27	-0.00235152	0.018456576	0.022217576
Bcl10	1	6.49E-07	9E-16	-0.003338172	0.024979357	0.022215849
Amz2	1	1.00E+00	2E-08	-0.003567118	0.015558085	0.022188894
Mtg2	1	1.00E+00	2E-05	0.005336074	0.017544075	0.022181387
Dph1	1	1.31E-06	3E-19	0.001373768	0.019266564	0.022175535
Dnase1l1	1	3.63E-37	3E-17	-0.000931382	0.053774204	0.022146551
4933431E2	1	3.62E-22	1E-40	-0.000459223	0.020723657	0.022134684
Nagk	1	2.58E-03	3E-10	-0.005638837	0.026593266	0.022132702
Spc25	0.430762301	2.72E-12	6E-18	-0.006420506	0.025995665	0.022130413
Ift172	1	1.00E+00	4E-08	0.001018409	0.002563222	0.022126847
Blvra	0.06764324	1.04E-07	4E-12	-0.008170017	0.030957908	0.022123296
Idi1	8.27923E-44	4.65E-25	4E-06	0.046108422	-0.037500427	0.022120484
Fbxw11	1	1.48E-09	1	0.014642425	-0.051634115	0.022110844
Rtraf	1	2.05E-01	7E-14	0.005670056	0.017116591	0.022109207
Tmem251	1	8.65E-18	3E-38	0.002189745	0.020803695	0.022092737
Zfp248	1	4.65E-03	2E-52	-0.000190418	0.007396671	0.022069165
Tacc3	1	1.35E-18	6E-36	0.002339911	0.02033544	0.022058153
Zfp106	2.62779E-07	1.00E+00	1	0.037065131	-0.003793715	0.022049774
Sh2d4b	1	2.42E-82	8E-58	-0.000650731	0.038636024	0.022045429
Hdac9	1	8.34E-75	9E-21	-0.002750021	0.078484071	0.022041996
Slc24a5	0.24448594	4.97E-23	4E-46	0.004398664	0.019296328	0.022039746
Tor2a	0.009000064	8.53E-08	8E-28	0.007178454	0.016670698	0.022038525
Daxx	1	1.00E+00	2E-12	0.004414549	0.01457382	0.022018004
Recql4	1	1.59E-08	1E-45	0.000514535	0.011707088	0.022012655
Phf12	4.81717E-09	1.00E+00	1	0.039816463	-0.012736329	0.022005029
Pdrg1	1	1.00E+00	3E-10	0.004084772	0.012570551	0.021998841
Mdh1	1	2.41E-07	0.5294	0.008097648	-0.037422324	0.021996285
Dcp1b	1	1.17E-03	1E-09	-0.006371462	0.023280847	0.021976478
Tsen54	1	7.02E-03	2E-32	0.001520701	0.010629666	0.021949606
Lsm2	1	5.55E-09	2E-18	0.004047988	0.02257225	0.021948955
Grwd1	1	2.34E-16	2E-41	0.00419225	0.017060218	0.021945022

Elmod3	4.27376E-41	1.00E+00	1	0.06283033	0.025563292	0.0219227
Mrps18c	1	1.00E+00	1E-09	-0.003138669	0.00605858	0.021903456
Calr3	1	1.88E-49	2E-47	0.000462315	0.029252542	0.021903234
Marco	1	3.57E-131	4E-77	-5.43962E-05	0.041620878	0.021890253
Mrpl17	1	1.41E-08	2E-23	-0.000539734	0.019436192	0.021885956
Abcb6	1	3.17E-03	9E-09	-0.00594177	0.022714411	0.02188337
Ncapd2	1	6.82E-04	2E-23	-0.000591801	0.013921628	0.021879868
Pidd1	1	1.31E-15	1E-34	0.000778657	0.01936446	0.021868427
Cerk	1	4.86E-253	5E-36	0.001617375	0.138206535	0.021864952
Noc4l	1	3.60E-07	3E-33	0.002393926	0.014414972	0.021844299
Ndufab1	1	1.00E+00	2E-05	-0.003512711	0.007075776	0.021842391
Nle1	0.007519169	1.10E-34	4E-40	0.005587151	0.026726371	0.021841752
Ptrh2	1	2.11E-02	2E-16	0.00020527	0.014977592	0.021832497
Ncaph2	1	1.00E+00	0.0002	-0.001494149	0.00399821	0.021813996
Asnsd1	1	2.84E-17	3E-32	-0.00117076	0.02158766	0.021813561
Cenpo	1	1.00E+00	9E-10	-0.002614469	0.012474819	0.021799955
Gosr1	1	1.00E+00	0.0197	-0.003733113	-0.002485179	0.021798093
Mettl22	1	4.69E-08	1E-21	0.001677379	0.018490837	0.021796346
Ddx23	1	1.00E+00	0.0038	0.000877838	0.004515205	0.021784992
Mbip	1	1.00E+00	4E-07	-0.003745961	0.00199317	0.021784093
Tbc1d22b	1	1.00E+00	0.0066	0.001837256	-0.004401776	0.021783231
Ptprt	1	1.23E-84	4E-35	0.001351239	0.048613574	0.021783206
Aste1	1	1.00E+00	3E-13	0.001048113	0.006128075	0.021734369
Unc119b	1	9.18E-02	1E-07	-0.002757111	0.018940789	0.021729922
Chchd4	1	2.15E-17	1E-12	-0.001735352	0.032941648	0.021721899
Hat1	1.57649E-10	2.54E-10	1E-07	-0.016591457	0.036676269	0.021711374
Zfp3	1	1.00E+00	2E-09	-0.000614316	0.015277034	0.021708086
Ptpdc1	1	1.58E-11	2E-33	-0.000632875	0.017023595	0.021707786
Polr1d	3.04105E-08	1.00E+00	0.0041	-0.021803601	-0.007124837	0.021686079
Cndp2	1	1.67E-09	1E-15	-0.000233743	0.028037113	0.021684056
Vsig10	1	6.63E-61	9E-78	-0.000736723	0.022734142	0.02167531
AC163638.	1	4.88E-33	2E-54	2.84161E-06	0.01976788	0.021661622
Dysf	1	7.15E-57	8E-43	0.001634658	0.044975227	0.021642698
Plk2	3.20697E-05	9.11E-28	1E-18	0.008901478	0.045227347	0.021637567
Mtmr11	8.18143E-12	7.35E-03	2E-57	0.007424727	0.006396475	0.021632486
Med7	1	1.00E+00	2E-11	-0.00230016	0.014215829	0.021602294
Tmem41a	1	1.30E-01	4E-37	0.00028247	0.008676836	0.021589311
Wdr83os	1	2.32E-19	2E-51	0.0028084	0.016028797	0.021589227
1700054A0	1	4.99E-12	2E-35	-0.000679333	0.016377642	0.021557911
Pole3	1	1.97E-03	4E-39	0.000999709	0.00934054	0.021544606
Atl1	1	1.71E-04	2E-15	-0.002961523	0.019497234	0.021539296
Pofut2	1	1.56E-04	2E-19	0.006929108	0.016638578	0.02153746
Ndufc2	1	9.54E-04	4E-13	-0.00281758	0.019773673	0.021532511
Timm10	1	6.52E-16	2E-35	0.002517248	0.018559558	0.021512339
Ift22	1	8.92E-06	2E-40	0.000118772	0.011213698	0.021509664
Rnf5	1	1.00E+00	1E-21	0.002033708	0.008283295	0.02150625
Gm9750	1	2.85E-14	8E-39	-0.002039353	0.014105917	0.021499267

H2-T22	1	1.00E+00	4E-11	-0.002184288	0.012167336	0.021486488
Cox7a2	1	8.34E-01	8E-06	-0.002886214	0.020656407	0.021475322
Kpna6	0.079036121	1.00E+00	0.005	0.017047989	0.000183663	0.021470041
Bco1	1	4.06E-07	2E-20	0.000755123	0.01903267	0.021439749
Mcm8	1	6.23E-29	9E-39	-0.001155465	0.023725159	0.021436583
Cdan1	1	1.00E+00	5E-13	-4.15849E-05	0.008920619	0.021433173
Gm45871	0.020051564	1.00E+00	5E-11	-0.009325077	-0.009726326	0.021432225
Casp3	1	1.00E+00	4E-12	-0.004662117	0.012658101	0.021413192
Mrps5	4.59762E-29	1.00E+00	0.0002	0.041027092	-0.01281393	0.021403404
Ccp110	1	1.00E+00	4E-07	0.007447038	0.002485786	0.02139587
Dohh	1	9.96E-20	1E-38	0.004169798	0.018953137	0.021395124
Msr1	1	4.46E-151	4E-40	0.002144231	0.085942274	0.021393366
E130311K1	1	1.00E+00	1E-18	-0.004360765	0.005147156	0.021389069
Srm	0.02772916	2.40E-20	3E-53	0.004220461	0.016103946	0.02138506
Gm38832	1	8.36E-72	2E-49	0.001979984	0.035207835	0.021384771
Gm32856	1	1.00E+00	2E-25	0.001357388	-0.001213858	0.02138314
Coq7	1	1.00E+00	9E-16	0.004410668	0.001514694	0.021374692
Faap100	1	3.01E-03	2E-21	-0.003705226	0.01524789	0.021371863
Mcm5	1	3.89E-38	4E-76	-0.000215002	0.015356973	0.021367873
Pemt	2.3849E-37	1.02E-96	1	0.08364787	-0.19987664	0.021365911
Dek	1	1.00E+00	3E-05	-0.001870795	0.019524342	0.021351028
5430416N(	1	1.00E+00	9E-10	-0.001298287	0.011130016	0.021343503
2310011J0	1	1.00E+00	3E-13	-0.004642881	0.012129057	0.021335927
Pcsk9	9.28257E-43	2.17E-17	1E-08	0.037312869	-0.03150088	0.021327001
Pim3	4.52881E-56	1.00E+00	0.7151	0.071019318	-0.01367431	0.021320001
Tfam	1	1.00E+00	7E-09	0.004410341	0.016105934	0.021309672
Zc3h3	1	1.00E+00	0.0029	0.008116284	-0.01145769	0.021288766
Fam83g	1	4.32E-08	4E-41	-0.00130848	0.012384905	0.021284396
Ncf2	1	1.35E-233	7E-53	-0.000424736	0.093671432	0.021263386
Ccdc149	1	1.00E+00	6E-13	0.002466895	-0.00887985	0.021253168
Gm34121	1	1.30E-04	1E-33	0.000714747	0.011946941	0.02125284
Zdhhc8	1	5.44E-08	6E-15	-0.002823249	0.022546827	0.021242743
Lrig3	0.00043283	1.00E+00	1	-0.020878284	-0.014808071	0.021240106
Smpd2	1	1.00E+00	2E-08	0.002689207	-0.009023473	0.021228961
Bank1	1	6.27E-85	2E-20	-0.00168672	0.071589108	0.021219028
Gm34411	3.38284E-11	1.67E-55	2E-29	-0.008060773	0.039741254	0.021202636
Btf3	1	1.33E-01	8E-11	-0.003917938	0.01732041	0.021194105
Pdcd2	1	2.44E-02	9E-19	0.003955145	0.013944057	0.021190391
Nek3	1	1.00E+00	1E-15	-0.00545307	0.005438743	0.021184455
Spry1	1	3.45E-19	5E-73	0.000843752	0.009879442	0.021119849
Ifrd2	1	1.00E+00	3E-15	0.002639644	0.010578577	0.021117524
Yipf4	1	8.56E-04	8E-07	-0.000643169	0.025707563	0.021104735
Sirt6	1	1.29E-13	2E-46	0.001283298	0.013977285	0.021088356
Rrm2b	0.006771944	1.00E+00	1E-06	-0.0111291	0.018266497	0.021077357
Mapkbp1	1	7.91E-02	5E-06	-0.001897565	0.023148213	0.021052362
Nup43	1	6.47E-06	3E-21	0.000143422	0.015650186	0.021037305
Ttll8	1	1.00E+00	3E-18	6.00203E-05	0.002009883	0.021017624

Smn1	0.001460163	1.00E+00	2E-09	0.012129946	0.004547155	0.021015606
Ndufb5	1	1.00E+00	1E-07	0.004629912	0.012558396	0.021012386
A930002C0	1.14282E-08	1.00E+00	2E-22	-0.009663478	0.008476742	0.021005586
Zfp874a	0.022200735	1.00E+00	5E-06	-0.012103497	0.002361692	0.021005442
Acot9	1	7.66E-36	2E-47	0.001218507	0.024429302	0.020996435
Lsm4	1	5.25E-03	5E-21	0.004461268	0.013216662	0.020992707
Mcl1	0.213811458	3.74E-15	0.0008	-0.011779534	0.06421774	0.020991813
Zfp952	1	1.00E+00	2E-08	0.002674075	0.011756238	0.020984056
Rhof	1	3.77E-22	3E-32	-0.001165094	0.023342498	0.020975999
Poglut2	0.107193508	1.00E+00	5E-16	0.008364023	0.009023452	0.020966511
Adprhl2	1	1.00E+00	3E-07	0.004778724	0.002894645	0.020962405
9630014M	1	3.10E-10	4E-60	-2.8731E-05	0.00962034	0.020920656
Rxrb	0.001977347	1.00E+00	3E-08	0.013160773	0.009711044	0.020920386
4930480G2	3.37753E-09	1.00E+00	9E-33	0.009590766	0.005006658	0.02091533
Gm30173	1	1.43E-66	8E-65	-0.00073048	0.02501745	0.02090709
Gm50368	1	8.60E-65	2E-57	-0.000555248	0.028256296	0.020898271
Atp5md	1	1.00E+00	0.0001	-0.005468613	0.007315385	0.020897455
Shq1	0.000344172	1.00E+00	0.2636	-0.021497185	-0.016470614	0.02089618
Ralb	1	6.03E-09	9E-12	-0.000794264	0.031514951	0.02087761
Rgs1	1	2.39E-232	2E-69	0.000411027	0.087418493	0.020877085
Slu7	1	1.00E+00	1E-05	0.009078678	0.002544386	0.020875909
Ptcd1	1	1.00E+00	5E-09	-0.002650374	0.007474464	0.020870615
Capn1	0.237256993	4.59E-09	4E-14	-0.007443113	0.025269412	0.020857341
Hist1h1c	1	7.50E-07	5E-18	0.000769034	0.017844414	0.020855248
Slc45a1	1	1.43E-46	1E-79	0.000768559	0.01541856	0.020850281
Cd38	1	1.09E-98	7E-30	0.000738207	0.075137305	0.020841776
Parp8	1	4.24E-119	3E-42	0.001368311	0.071015864	0.02081912
Ptpn13	1	3.54E-11	2E-45	-0.001086265	0.013613191	0.02081644
Gm43691	7.05449E-14	1.00E+00	3E-11	0.019526294	0.005977081	0.020815942
Acd	1	1.00E+00	4E-22	0.000157892	0.008362682	0.020813959
Mrpl38	5.01449E-14	1.00E+00	0.0048	0.033746666	-0.016212491	0.020806909
Gm36172	1	1.09E-11	6E-42	0.003079713	0.014259085	0.02080471
Csf1r	1	9.05E-163	3E-48	-0.000164248	0.077354116	0.020797527
Gm36028	1	3.74E-17	1E-35	-0.003651716	0.018250943	0.020784118
Tsnax	1	1.00E+00	0.0017	-0.005342704	0.011342007	0.020769608
Brip1	2.29126E-17	1.00E+00	0.1823	-0.027042307	-0.012693441	0.020762418
Nat2	1	7.81E-02	2E-09	-0.002624844	0.018572301	0.020762027
Wiz	1	1.00E+00	2E-06	0.004118323	0.010146697	0.02073806
9330020H0	1	1.00E+00	2E-30	0.003089962	0.006519294	0.020735739
Dus4l	1	1.94E-12	5E-10	0.001045225	0.03271803	0.020705078
Kif18a	0.370543979	2.83E-21	1E-27	-0.004284239	0.024395806	0.020698528
Hibch	1.84487E-08	1.60E-14	1	-0.033877693	-0.068176332	0.02069808
Acyp1	1	3.37E-04	3E-11	-0.005074109	0.021549022	0.020684741
BC024063	1	1.89E-01	4E-15	-0.006589492	0.013494788	0.020679499
Samhd1	1	2.14E-51	2E-06	-0.003560594	0.09935582	0.020677419
Cdc42se1	1	5.21E-22	7E-28	-0.000727212	0.025154036	0.02067137
Htra1	1	3.49E-176	5E-68	0.000185935	0.057687139	0.020655495

Spdl1	1	2.39E-35	6E-57	-0.001052027	0.019917449	0.020614949
A230108P1	1	2.47E-18	1E-19	-0.003859997	0.026929437	0.020606741
Cops7a	0.702367269	1.00E+00	3E-16	0.007455066	0.011738426	0.020602062
Traf3ip2	1	1.00E+00	6E-09	0.00178025	0.015377619	0.020600945
Necap2	1	1.00E+00	2E-11	-0.000727095	0.012257439	0.020582279
Lockd	1	6.80E-11	2E-60	-0.000672475	0.008726962	0.020559462
Pola2	0.026464947	1.00E+00	5E-06	-0.010704683	0.018216288	0.020549889
Gm46404	1	4.09E-31	7E-56	-7.8928E-05	0.017588892	0.020547975
Polr2g	1	7.14E-14	1E-22	0.000212551	0.023530994	0.020545901
Kctd6	1	1.00E+00	7E-08	0.002101647	0.010293711	0.020541863
Vamp8	1	1.00E+00	6E-06	-0.000365535	0.016797847	0.020541635
A930033H1	0.84086616	1.00E+00	2E-17	0.006722476	0.003718839	0.020529071
Cenpu	1	1.10E-08	4E-27	-0.003473157	0.018841724	0.020527462
Nacc1	1	1.00E+00	5E-05	0.001350721	-0.011368557	0.020523555
Gm11752	1	2.00E-50	1E-72	0.000140774	0.018149322	0.020518174
Morn2	1	2.72E-06	7E-37	0.001771068	0.011480257	0.020499301
Rapgef4os2	1	2.03E-03	3E-08	-0.004431047	0.022008347	0.020492337
Tmem184a	0.016792215	1.19E-07	2E-15	-0.007536215	0.021203443	0.020477747
Wdr55	1	3.07E-06	1E-37	0.003508541	0.012459301	0.020474321
Zgpat	1	1.00E+00	8E-07	0.001278429	0.011414009	0.020472552
Syk	1	1.60E-277	9E-47	0.000422478	0.126240571	0.020464625
Eps15	6.26265E-55	3.34E-01	1	-0.068755776	0.044272011	0.020457924
Slc23a2	9.27969E-19	1.00E+00	1	-0.044478698	0.039096783	0.020456028
Glg1	0.000626125	1.00E+00	1	0.028510942	0.021893713	0.020452506
Mrrf	1	1.00E+00	0.0002	0.005930076	-0.000641006	0.020446802
Adap1	1	7.84E-125	8E-74	-0.000295605	0.039921592	0.020445891
Yif1a	1	3.06E-11	4E-33	-0.001975375	0.015837774	0.020431883
Eif5a	1	1.30E-07	6E-08	0.002141345	0.031514174	0.020430936
Adam1a	1	1.94E-01	9E-24	-0.00176483	0.01013606	0.020428661
Lcn2	1	2.20E-69	4E-46	-0.001596179	0.035214085	0.020421053
Avil	1	1.00E+00	4E-23	-0.003554216	0.010860338	0.020388114
Dnah7c	1	4.26E-02	9E-25	-0.001450006	0.010664674	0.020383909
Snrpd3	5.38211E-12	1.00E+00	0.0137	0.028877175	0.004980612	0.020381224
Elovl1	1	1.00E+00	2E-16	-4.94736E-05	0.011580706	0.020380938
Gng5	1	1.00E+00	5E-12	0.000781841	0.009187981	0.020362796
Lhx1os	1	6.20E-44	3E-81	-0.000308185	0.012503838	0.020345781
Cdk2ap2	0.693940934	1.00E+00	1E-11	0.008031577	0.010086274	0.020343648
Tpcn2	1	2.41E-113	7E-25	-0.000382324	0.091279761	0.020341111
Mon1a	1	1.00E+00	0.0126	0.004091685	-0.005813767	0.020334802
1700010I14	1	5.21E-07	4E-30	0.001532795	0.013163237	0.020326545
Erlin1	4.07818E-13	1.00E+00	0.1433	-0.029436297	0.017086502	0.020303749
Smg9	1	1.00E+00	2E-07	-0.000911288	0.000535525	0.020283387
Hinfp	1	1.00E+00	7E-05	-0.002152019	0.005675026	0.020245904
Vxn	1	8.84E-106	7E-80	-0.000192813	0.028553362	0.020235739
Mrpl53	1	1.00E+00	9E-13	0.002339581	0.007885484	0.02020473
Cpsf4	1	1.00E+00	8E-07	0.001701708	0.012872638	0.020189806
Ccdc88c	1	2.98E-15	1E-32	-0.002450738	0.019586958	0.020186091

Ankrd54	1	1.00E+00	2E-10	0.002799319	0.013138205	0.020182895
Ncdn	0.00018842	3.19E-04	5E-20	0.009322574	0.014094291	0.020180214
Igsf8	1	9.05E-15	2E-39	-0.000767084	0.018313555	0.020177105
Tomm20	0.011166857	4.03E-01	3E-07	-0.011793621	0.019416966	0.020163229
Qpct	1	1.00E+00	2E-20	0.001373538	0.005914482	0.020156532
Tceal8	1	1.73E-05	2E-41	0.001889447	0.010218652	0.020155225
Mettl25	1	1.00E+00	0.0031	0.00235059	-0.009236873	0.020137451
Prdx5	1	1.00E+00	4E-10	-0.001715521	0.015330498	0.020124833
Tyms	1	1.00E+00	1E-12	-0.002538363	0.008828187	0.020118453
Gm11714	1	6.59E-24	7E-37	-0.000194899	0.021034569	0.020092166
Stambp	1	1.00E+00	4E-07	-0.004304817	0.001587625	0.020082557
Kptn	1	1.00E+00	4E-08	0.000514211	0.003236435	0.020079958
Abcc1	1	1.01E-259	7E-59	0.000562613	0.104765269	0.020077128
Ndufa4	1	1.31E-01	0.0078	-0.010990911	0.032277091	0.020066672
Mrps12	1	1.00E+00	1E-13	0.003592348	0.008622789	0.02004937
Ermard	1	1.00E+00	0.0007	-0.004618449	0.018731864	0.020044055
D5Ertd579	1.9795E-08	3.09E-01	1	-0.041927744	-0.033302183	0.020033906
Wbp1	1	9.50E-02	9E-15	0.002099115	0.014152103	0.020022212
Msi2	1	9.85E-05	1	-0.027668652	-0.038722396	0.019976681
Gm11084	1	1.00E+00	2E-12	0.007662997	0.012605311	0.019975007
Cd274	1	1.09E-127	8E-37	-0.003570437	0.080213273	0.019973247
Tbxas1	0.475714288	1.85E-157	3E-22	-0.003748149	0.109774482	0.019968487
Gpank1	1	3.55E-03	2E-19	0.00024341	0.013439438	0.01994444
Zfp709	1	3.50E-02	8E-23	-0.000647579	0.011570832	0.019934468
Ubald2	1	1.05E-08	6E-14	0.003231844	0.024502373	0.019916804
Irgq	2.6429E-05	1.00E+00	4E-12	-0.010187573	0.010356834	0.019911548
Gm47882	1	3.59E-15	2E-12	-0.004897478	0.029795003	0.019908384
Zfp84	1	1.70E-04	4E-08	0.000921141	0.023058362	0.019877023
Tmem60	1	9.13E-13	3E-18	0.003342425	0.023448932	0.019866227
Coil	1	1.00E+00	1E-08	0.002796764	0.002967379	0.01985888
Dach2	1	2.91E-08	9E-26	-0.001476279	0.01621279	0.019858402
Ccdc174	1	1.00E+00	0.0054	0.012278776	-0.003762243	0.019844348
2010310C0	1	2.24E-28	3E-65	-0.000229336	0.014116199	0.019819539
Gpatch11	1	7.10E-01	3E-11	0.0049846	0.014575936	0.019806798
Stard3nl	1	1.00E+00	7E-12	-0.004389951	0.014704067	0.019798433
Nelfe	1	1.00E+00	2E-10	-0.001174499	0.012440667	0.019797084
Ccnj	1	2.08E-05	1E-26	0.0018063	0.01364129	0.019784676
Gm50337	1	1.81E-83	7E-78	-0.000229554	0.021978411	0.019783401
Usp39	1	1.00E+00	4E-06	0.003487755	0.006507628	0.019766063
Jrkl	1	1.00E+00	4E-11	-0.002909056	0.007793269	0.019758728
Tulp2	1	5.31E-01	0.0495	0.007714958	0.023249909	0.01974197
Mcm3	1	1.56E-30	3E-53	-0.00122443	0.017360963	0.019739123
Ap1ar	1	1.00E+00	0.0467	-0.005620619	0.01979453	0.01973709
Cdc45	1	1.00E+00	4E-23	-0.002015901	0.00215422	0.019732799
Foxq1	1.33281E-72	4.42E-18	7E-06	-0.04308881	0.052368471	0.019729623
Apom	0.431527043	1.00E+00	1E-06	-0.009356835	0.000882037	0.019727442
Gm14569	1	1.74E-120	3E-66	7.33883E-05	0.036948943	0.019717533

Gm26797	1	3.56E-69	3E-66	0.000324808	0.025236571	0.019715676
Caskin2	1	1.00E+00	8E-24	-0.000459282	0.008011654	0.019712961
Gpr160	1	8.42E-48	3E-58	-0.000721508	0.021221878	0.019698578
Ctsz	1	2.94E-05	0.0011	-0.004715913	0.0409509	0.019698102
Thsd1	1	1.00E+00	5E-13	-0.004755788	0.01204421	0.019697173
Agbl4	1	2.15E-01	3E-11	-0.006092575	0.014454496	0.019686588
St6galnac3	1	3.68E-13	4E-11	0.004672317	0.039966369	0.019682739
Clp1	1	4.51E-03	2E-10	-0.001750606	0.018210969	0.019673421
Arhgap25	1	3.71E-271	9E-52	0.000325372	0.117078056	0.019665409
Cd300a	1	4.92E-246	5E-42	-0.001289677	0.111687083	0.019640454
B230208H1	1	9.77E-05	1E-27	0.002116337	0.012315893	0.019637651
Ddhd2	8.55099E-16	1.00E+00	1	0.044492701	0.027081992	0.019635304
Cyp17a1	1.21067E-25	1.00E+00	3E-35	0.013279693	-0.002539683	0.019631682
Gm45086	1	1.00E+00	4E-39	-0.000922832	0.002870076	0.019630743
Vta1	1	1.00E+00	0.0499	-0.00589251	0.004424122	0.019586462
Rnf215	1	1.00E+00	1E-07	0.003836347	-0.010606661	0.019530968
Gm15834	1	2.64E-26	4E-31	-0.000389491	0.024607663	0.019516895
Maip1	1	1.00E+00	2E-09	0.000998134	0.004684396	0.019515663
Yap1	1	4.29E-15	1	-0.004858489	-0.080592102	0.019508406
Calr	0.551133485	1.23E-02	1	0.015936969	-0.018661077	0.019501506
Slc38a1	1	1.98E-146	7E-53	-0.000570513	0.060799023	0.019496032
Atp6v1b2	1	1.15E-06	1	-0.006051367	0.069925848	0.01949079
Pitpnm2os	1	5.68E-16	1E-30	0.000793367	0.018146503	0.019481746
Tmem116	0.364005431	1.00E+00	2E-17	-0.005656388	0.010337244	0.019461055
Ubiad1	1	1.58E-03	9E-16	0.003870072	0.015746664	0.019454046
B230354K1	1	1.00E+00	1E-08	-0.002072469	0.001684268	0.019410891
Gm11827	1	1.76E-05	2E-36	-0.000363648	0.011178201	0.019410494
1700030K0	1	1.00E+00	9E-05	4.48027E-05	0.006150299	0.019398903
4933436I2(	0.002852028	1.00E+00	5E-13	-0.008057379	0.007481528	0.019387539
Serac1	1	1.00E+00	2E-06	-0.004284981	0.016868346	0.019368959
Gm16023	0.054966313	4.35E-12	4E-16	0.007924433	0.023186756	0.019367548
Cript	1	1.00E+00	3E-08	0.005164334	0.009806726	0.019360515
Prorsd1	1	1.00E+00	8E-14	0.005107585	0.008759686	0.019360499
Gm9828	1	1.40E-09	5E-19	0.004448077	0.01936401	0.019358541
Camsap3	1	1.00E+00	0.0026	-0.003943353	-0.011643585	0.019350238
Aifm2	1	1.45E-02	0.1137	-0.005929585	-0.028441555	0.019333934
Msrb2	1	1.00E+00	4E-06	0.00182408	-0.009774011	0.01932896
Mterf2	1	1.63E-07	2E-28	-0.000605585	0.013970967	0.019322411
Pomgnt2	1	6.74E-14	5E-17	-0.001245185	0.024067619	0.01931255
Plekhg5	2.60292E-12	1.00E+00	0.0061	-0.022119327	0.011711098	0.019309651
Slc18b1	1	1.00E+00	9E-05	-0.003685118	9.15915E-05	0.019299628
Itfg2	1	1.00E+00	0.0002	0.010221069	0.000457968	0.019290982
Fbxo44	1	1.00E+00	6E-17	-0.002003254	0.005233037	0.019284195
Zfp11	1	1.00E+00	1E-24	-0.003828999	0.003530222	0.019271747
Gm16565	1	1.00E+00	5E-29	-0.002145603	0.000602317	0.019267755
D030024E0	1	6.00E-38	2E-45	-0.000440608	0.02181414	0.019263925
A230103J1	1	2.25E-16	3E-49	-0.001055369	0.013187834	0.01924448

Ppm1f	1	1.00E+00	6E-08	-0.006775817	0.003752944	0.019235436
Ect2	2.65768E-19	2.38E-06	8E-20	-0.010121019	0.014959056	0.019228445
Kif15	1	1.20E-22	8E-42	-0.00198546	0.017746136	0.019220705
Spcs2	1	1.10E-04	0.0563	0.005148966	-0.026151606	0.019211717
Tcta	1	1.00E+00	9E-08	-0.001546439	-0.001863194	0.019196276
Zfyve19	1	1.00E+00	5E-05	0.003371509	0.001763678	0.019196229
Gm40578	1	3.89E-50	2E-54	-3.26253E-05	0.021536253	0.019193177
Dcaf1	2.71683E-15	1.00E+00	1	0.043107673	-0.016097186	0.019179684
Lrrc14	1	1.06E-01	2E-13	0.002160152	0.014157806	0.019176122
Fabp2	1	8.01E-10	9E-28	0.003010224	0.015064941	0.019164842
Git1	1	5.86E-06	4E-22	0.000626776	0.015784715	0.019152433
Pfkm	1	1.00E+00	3E-10	0.000276811	0.012538077	0.019143703
Gramd1b	1	6.69E-120	3E-28	0.001038309	0.075131852	0.019138592
Lamb3	0.000638956	1.00E+00	6E-09	-0.010486926	0.008455738	0.019116159
Clip2	1	9.01E-26	1E-44	-0.001278706	0.020183573	0.019115663
Kifap3	1	2.68E-01	2E-07	-0.000874052	0.018420358	0.019110725
Gm11906	1	1.54E-13	8E-16	0.003104998	0.024929049	0.019089106
Rab8b	1	1.13E-197	3E-33	0.001190279	0.104138594	0.01908533
Polr3k	1	4.82E-03	3E-17	-0.002584122	0.013716977	0.019083991
Fndc3a	1.44694E-20	1.00E+00	1	0.058251773	0.035957207	0.019078007
Hsp90aa1	1.09914E-21	1.00E+00	1	-0.033196185	-0.002080111	0.019077074
G6pc2	1	1.00E+00	2E-17	-0.004417442	-0.006699397	0.019069509
Ndufa8	1	1.00E+00	2E-08	0.003024827	0.00044261	0.019065477
Fam160b1	4.54391E-06	1.00E+00	1	0.030915052	-0.023077028	0.019057536
Gm16253	1	1.41E-31	3E-59	-0.000843003	0.015799756	0.019039593
Ngdn	1	1.00E+00	5E-05	0.00805944	0.012139695	0.019032622
Qtrt2	1	1.00E+00	0.0001	0.007578292	0.001006965	0.019027181
Hist1h1e	2.06522E-07	1.00E+00	4E-13	0.012685229	0.007858353	0.019025625
Mrpl11	1	3.90E-05	3E-29	0.001847827	0.011680876	0.019017894
Cib1	1	1.00E+00	5E-10	0.006358589	0.010164099	0.019014383
Mphosph6	1	6.35E-03	2E-25	-0.001458703	0.01097494	0.018992675
Mgrn1	2.36699E-06	1.00E+00	1	0.033305927	0.016294764	0.018991366
Arhgap45	1	2.41E-159	2E-19	-0.000327076	0.104424562	0.018989199
Prpsap2	1	1.00E+00	0.0066	0.004070128	-0.001562991	0.018980792
Igf1r	0.819450097	1.87E-84	9E-20	0.003609427	0.068202399	0.018967902
Gm10851	1	4.93E-92	1E-76	-0.00025279	0.024665292	0.018960937
Gm14305	1	1.82E-06	9E-48	0.001623305	0.008284384	0.018958797
Lrch3	1	8.80E-04	1	0.008017838	0.052088291	0.018957456
Tyw5	1	1.00E+00	2E-06	0.003282608	-0.001693984	0.018942595
Tmem18	1	1.99E-09	9E-24	0.002340554	0.016918911	0.018941288
Tarbp2	1	1.00E+00	2E-14	0.00187829	0.008648544	0.018938661
Gm2115	1	2.57E-15	6E-42	-0.001175302	0.014682772	0.018921448
Rad52	1	1.00E+00	2E-05	-0.003744639	0.005092148	0.018912924
Slc27a4	1	1.00E+00	1E-10	0.001655822	0.012026025	0.018887876
Fundc2	1	4.22E-09	3E-23	-0.001443777	0.01651221	0.018884242
Gm34664	1	3.67E-34	3E-39	0.000247491	0.021845056	0.018878427
Rrp36	1	1.00E+00	0.0039	-0.006556963	-0.002706583	0.01887488

Gata3	1	6.41E-45	1E-72	0.000210926	0.013827219	0.018871101
Lpxn	1	4.44E-30	2E-12	-0.003138045	0.045307324	0.018864343
Gsto1	0.004838456	1.40E-02	6E-09	-0.00901531	0.018944495	0.018839036
Usp2	1.72691E-24	1.84E-07	4E-05	-0.027676665	0.035622164	0.018819539
Mrpl58	1	4.06E-01	5E-18	0.000915606	0.010956752	0.018819493
Yipf6	1	1.00E+00	7E-09	-0.000911106	0.011482494	0.018816127
Bmp8b	1	2.23E-35	4E-47	0.001514433	0.02018492	0.018812924
3300002I0	0.002005669	1.00E+00	0.0003	-0.013178939	-0.011047764	0.018809038
Sorl1	1	5.69E-202	5E-33	-0.000785947	0.099714282	0.018806274
Echdc1	0.0693917	1.68E-09	0.8793	0.020227857	-0.044875611	0.018803523
Smoc2	1	3.63E-13	6E-63	-0.00096742	0.008495158	0.018790979
Foxo1	4.1086E-269	1.00E+00	1	0.238895858	0.024402003	0.018763109
Cggbp1	1	1.05E-01	4E-05	0.007647294	0.021671862	0.018760527
2510017J1	1	1.11E-01	1E-23	0.000313358	0.009474193	0.018752824
Saal1	1	5.91E-04	5E-21	-0.002517187	0.012966722	0.018749172
Gm29650	1	2.08E-28	7E-36	-0.000964696	0.022142794	0.018748796
Mrpl36	1	4.31E-02	5E-19	0.003843673	0.011589212	0.018741562
Gpr108	1	1.00E+00	3E-05	-0.00250826	0.001753848	0.018739827
Sptlc1	1	1.00E+00	0.0318	0.004667396	0.004031231	0.018735125
Ovgp1	1	3.12E-01	2E-17	-0.000380151	0.010259623	0.018731128
Htra2	1	1.03E-12	1E-15	0.002471744	0.025385963	0.018726364
1700126G0	1	4.84E-03	7E-25	0.000230009	0.01096499	0.01872467
Cep19	1	1.12E-02	1E-23	0.000894778	0.01104727	0.018723672
Cyp2c37	2.72729E-54	1.45E-14	1	-0.065620382	-0.045801177	0.018723229
Manea	1	1.00E+00	1E-06	0.004236497	0.01197869	0.018721079
Gid8	1	1.00E+00	2E-07	-0.004215236	0.009330891	0.018720279
Gins4	0.004732628	1.00E+00	5E-18	-0.006487462	0.006237755	0.018709057
Tmem29	1	1.02E-06	0.2023	-0.011098799	-0.032129568	0.018706112
Cdkl4	1	2.69E-48	1E-33	-0.002818443	0.030952462	0.018701766
Cptp	1	1.17E-01	6E-25	0.000324578	0.009468089	0.018688472
Ptpn22	1	1.08E-46	2E-44	0.0010328	0.028891678	0.018684488
Ndst2	1	1.00E+00	0.0005	-0.000743085	-0.001954477	0.018681536
4933421A0	7.78742E-05	3.62E-20	4E-36	0.006168895	0.017566452	0.018677311
Dynlt1b	1	3.50E-17	1E-29	0.00096233	0.01872754	0.018649789
Gm27241	1	1.77E-07	2E-39	0.00174782	0.010227405	0.018649168
Smyd5	1	1.00E+00	4E-11	-0.001181315	0.009958805	0.018639804
Polr2j	1	4.07E-04	2E-15	0.000606312	0.015611695	0.018635814
Ptprb	9.50003E-07	2.62E-03	0.9568	0.014249732	0.048466161	0.018635189
Ess2	1	1.00E+00	6E-17	0.002727543	0.008858332	0.018618023
Prf1	1	2.97E-22	1E-58	-0.001302401	0.012102782	0.018613787
Tspan15	1	6.37E-34	7E-62	9.43024E-05	0.016067548	0.018605796
Sf3b4	1	1.27E-09	4E-24	0.004660251	0.016430389	0.018595546
Ccnb1ip1	0.057807588	8.89E-43	1E-33	-0.003714737	0.028050288	0.01859336
Nme6	1	1.00E+00	0.0153	0.007733917	0.003519525	0.018584462
Larp7	1	1.00E+00	6E-09	-0.006962244	0.013126338	0.018582555
Zfp691	1	1.00E+00	5E-12	0.002774877	0.003644824	0.018567748
Dnajc25	2.68342E-18	9.11E-02	0.3879	0.040453329	-0.024243009	0.018557643

Smad9	3.33822E-77	1.00E+00	1	-0.073570767	0.041383161	0.018553397
Tapbpl	1	1.00E+00	6E-20	-0.003020207	0.006298328	0.018530624
A930037H(	0.847859513	1.39E-09	0.0173	-0.010783874	0.041620056	0.01852999
C2cd2	1	5.19E-33	1	-0.019857037	0.151628394	0.018527427
Edf1	1	1.00E+00	2E-08	0.00034505	0.004525328	0.018527154
Wrap73	1	1.36E-03	4E-13	0.000629817	0.016149407	0.018492943
Cenpk	1	1.00E+00	3E-24	-0.000761874	0.008709401	0.018489127
Zfp458	1	1.00E+00	1E-10	-0.00401078	-0.002977105	0.01848033
Impad1	1	1.00E+00	0.0004	-0.005327672	-0.008596888	0.018473966
Zfp846	1	1.00E+00	3E-11	-0.002181364	0.011547897	0.018472231
Pak1	1	3.98E-39	1E-43	-0.000375148	0.025422806	0.018460229
Kcnk13	1	2.65E-148	1E-49	-8.53409E-05	0.069235996	0.018451151
Dyrk3	1	4.96E-21	1E-43	0.002629801	0.015991961	0.018448573
Pfkp	1	4.09E-91	4E-54	0.000104152	0.034188756	0.018444368
Gm40915	0.883510363	6.66E-04	4E-24	-0.004376025	0.011141793	0.018441105
Pitrm1	1	1.00E+00	5E-06	-0.002101644	0.015940868	0.018419922
Mat2a	1	1.99E-02	1	0.005792367	0.040840472	0.01841301
Vkorc1	1	1.00E+00	4E-11	0.004568798	0.012406102	0.018395027
Skp2	1	1.00E+00	2E-10	-0.000124262	0.013622447	0.018389217
Rgp1	0.004459057	1.00E+00	7E-09	0.011092328	0.003092238	0.018387829
Kif3b	0.941612938	1.00E+00	0.0039	-0.011144473	8.67261E-05	0.018385087
AU020206	1	1.89E-63	4E-25	-0.001178629	0.048601323	0.018367664
Pik3r4	1	1.00E+00	0.004	-0.007045591	-0.007155479	0.018353814
Pcdh17	1	5.04E-32	3E-42	0.000247928	0.026070531	0.018347411
Pex10	1	1.00E+00	7E-15	0.002531979	0.008287429	0.018331869
Wdr53	1	1.00E+00	7E-09	0.004389028	-0.000394971	0.018317278
Gosr2	1	1.00E+00	1E-06	0.008760447	0.00119571	0.018315565
5730480H(	1	1.00E+00	0.0146	-0.008531757	-0.008954222	0.01831259
Ywhab	1	1.00E+00	0.0012	0.001803943	0.0156849	0.018310582
Lrrc29	1	1.00E+00	0.0001	-0.000375834	-0.001134771	0.018301457
Rpl15	1	4.79E-18	1E-29	0.001157068	0.0192047	0.018291643
Thumpd1	1	1.40E-04	1E-14	0.002007982	0.017175759	0.018285841
Slc38a7	1	1.72E-01	0.0014	0.007063506	0.02414922	0.018281294
Elovl6	3.02628E-18	4.12E-35	1	0.061757395	-0.110939679	0.018279116
Rptoros	1	8.76E-15	2E-33	0.000667167	0.015135514	0.018270161
Slc11a1	1	1.83E-203	1E-45	0.000120173	0.078102756	0.018231847
Gm11713	1	5.61E-16	4E-25	0.002086427	0.019377309	0.018217298
Taf5	1	1.02E-01	3E-10	0.003299939	0.015495119	0.018202397
Nosip	1	1.00E+00	3E-05	-0.000516873	0.015029898	0.018183307
Gls	1	7.76E-68	6E-16	-0.002338511	0.066178844	0.018182962
Gm13561	1	1.32E-26	2E-10	0.006271864	0.039598746	0.018163341
Myo7a	1	1.00E+00	1E-06	-0.001117047	0.01509351	0.018145399
Pfdn5	1	1.00E+00	2E-06	-0.002437755	0.01188709	0.018131126
Ptgr1	1	5.00E-43	1E-66	-0.000231118	0.016175938	0.018126198
Trps1	1	5.04E-148	3E-30	0.003193626	0.096017001	0.018126002
B130034C1	1	1.93E-11	3E-06	-0.001623188	0.032476998	0.018123539
Bag2	1	1.00E+00	7E-17	-0.003109344	0.007743322	0.018119766

Smarcb1	1	1.00E+00	0.0129	0.001945644	-0.006158624	0.018119441
Abracl	1	2.68E-13	3E-24	-0.001216476	0.018670413	0.018085462
Gm36279	0.029435178	4.33E-21	6E-23	0.006296696	0.023522919	0.018082297
Khnyn	1	1.75E-06	1	0.002967612	0.056976475	0.018062357
4930522L1	1	1.00E+00	4E-12	-0.006165008	-0.002973407	0.01805128
Mcat	1	2.70E-04	8E-18	0.00363061	0.013700358	0.018049339
Rex1bd	1	1.26E-06	5E-19	0.001881313	0.01625394	0.018045391
Nsfl1c	1	2.18E-01	0.0227	0.002201922	-0.018196372	0.018043632
Map11	1	1.00E+00	5E-07	0.007922062	0.010927796	0.018033839
Cby1	1	7.81E-08	2E-17	-0.001716532	0.016994416	0.018024471
Arhgap19	1	5.61E-61	8E-38	-0.000363589	0.03540481	0.018020836
Jagn1	0.005226793	1.00E+00	1E-07	0.011432625	0.011579308	0.018018502
Gm11789	1	1.00E+00	2E-09	-0.00070142	0.002112509	0.017998515
Trmt10c	1	3.01E-01	8E-12	0.001992987	0.013451298	0.017989364
E130309D0	1	5.21E-04	2E-16	0.00149161	0.013794142	0.017985029
Cenph	1	1.05E-20	8E-47	-0.001209265	0.013431173	0.017972315
1700022N2	1	1.00E+00	4E-25	-3.89325E-05	0.006560086	0.017968624
Pde2a	1	1.96E-31	3E-27	3.35401E-05	0.034113871	0.017968532
Acin1	0.004530836	5.33E-03	1	0.029573015	0.052306871	0.017948862
Cttn	0.041746981	1.00E+00	0.0035	-0.013536454	-0.008115066	0.017941444
Msantd4	1	1.00E+00	1E-12	-0.001752234	0.002233826	0.017932664
Inip	1	1.00E+00	6E-12	-0.001788011	0.013196426	0.017920071
Ier3ip1	1	1.00E+00	0.0008	-0.000169037	-0.003079182	0.017912545
4921504E0	1	1.00E+00	3E-08	0.001905641	0.011400643	0.017911575
Amd1	4.65874E-07	1.00E+00	1	-0.022864454	0.016355201	0.01789536
Tmod3	0.492753863	1.00E+00	0.0062	0.011815291	0.017314464	0.017879547
Mcrip2	0.003746246	2.80E-01	4E-07	0.011617791	0.016067016	0.01787701
9030025P2	1	5.23E-26	4E-19	-0.00140458	0.029204494	0.017874207
Gdpd3	0.000620157	1.44E-20	8E-15	-0.007579336	0.029361796	0.017868867
Ppp1r14b	1	2.23E-15	3E-16	0.002429457	0.023539856	0.017867712
Plod3	1	1.00E+00	2E-07	-0.002930433	0.003413479	0.01786741
Mrpl9	1	1.00E+00	3E-06	0.003932497	0.010152053	0.017858677
Tcf7	1	1.00E+00	7E-26	-0.002444987	0.002733158	0.017842604
Ubash3b	1	3.01E-142	7E-22	0.001290175	0.08887532	0.017838257
Men1	0.017202778	1.00E+00	3E-15	0.007766915	0.007581658	0.017819207
Gm18280	1	4.48E-06	7E-35	-0.001396446	0.009825711	0.017808816
Tnfsf13b	1	1.01E-33	2E-53	0.000557166	0.016143002	0.017802778
Tlr4	1	1.72E-172	6E-39	0.000691023	0.076740278	0.017801561
Plekhg1	0.361132562	2.55E-22	6E-10	0.00695381	0.053827732	0.017799701
Cpd	3.57891E-19	1.00E+00	0.0055	-0.026276188	0.006984804	0.01779942
Nol12	1	1.00E+00	2E-15	-0.003446721	0.008021147	0.017798919
Gm31793	1	1.00E+00	1E-30	-0.000442566	0.003634401	0.017789136
Tmem101	1	1.72E-15	5E-14	0.003836545	0.02602152	0.017786163
Timm22	1	1.00E+00	2E-08	-0.004861851	0.006274319	0.017780845
1110046J0	1	5.87E-39	2E-47	-0.000380187	0.019148493	0.017770193
Nhsl1	1.3457E-46	1.06E-06	1	-0.095244072	-0.059983909	0.01775324
Matn2	1	1.17E-39	4E-47	-0.000105087	0.020199485	0.01774681

Kifc5b	1	1.29E-07	1E-24	-0.001403479	0.014396795	0.017731668
Uprt	1	8.80E-15	6E-29	-0.001908282	0.016925754	0.017731434
Slx4	1	1.00E+00	3E-12	0.003529816	0.006525655	0.017723226
Ube2t	1	4.53E-19	3E-38	0.000871934	0.015108871	0.017716299
Sprtn	1	1.00E+00	1E-07	0.000435049	0.014624813	0.017715059
Ska2	1	3.66E-05	1E-22	-0.001398799	0.013294563	0.017705015
Obscn	1	6.10E-18	1E-33	0.000275727	0.016835285	0.017676391
Oser1	1	1.00E+00	0.0003	-0.001421684	0.007381613	0.017662196
Atmin	1	9.06E-04	5E-13	0.000808952	0.014874431	0.01765354
Mdc1	1	2.52E-02	4E-07	-0.004939584	0.018759646	0.017644646
Nek8	1	1.00E+00	1E-11	-0.001774013	0.007802396	0.017638213
Dus3l	0.195465204	1.00E+00	8E-09	0.008522449	0.00116614	0.017622878
4930429F2	1	5.43E-14	1E-26	-0.001188405	0.016763032	0.01761945
Rhbdf1	1	1.00E+00	2E-30	0.00012933	0.005154283	0.017613077
Gusb	1	4.12E-43	2E-07	0.001000874	0.069534383	0.017612662
Tspan17	1	1.56E-31	3E-56	0.000302331	0.014587671	0.017609044
Mrpl50	1	1.00E+00	2E-08	0.002334869	0.009858357	0.0176027
Stk11ip	1	2.97E-05	2E-21	0.002175086	0.013832135	0.017588027
Tatdn3	1	1.00E+00	1E-07	-0.003794378	9.5707E-05	0.017587703
Ptbp1	1	1.00E+00	0.0068	0.011127908	-0.000377833	0.01756596
Tctn3	0.107065697	5.14E-28	7E-41	0.00379493	0.018075299	0.017561403
Lratd2	1	7.27E-41	7E-48	0.00110151	0.019996051	0.0175568
Lrrc40	1	1.00E+00	3E-07	-0.005191014	0.010304114	0.017531663
Arid5a	1	3.63E-07	5E-24	-0.003660639	0.015218144	0.017530017
Ndufa11	1	2.12E-03	2E-13	-0.000869396	0.016024202	0.01752368
Wdfy4	1	6.94E-146	4E-29	-0.000212738	0.08223016	0.017520276
Ift80	1	1.00E+00	0.0002	-0.002637276	-0.005244654	0.017511521
Cdca2	1	2.45E-01	9E-35	-4.47486E-05	0.006673989	0.01750865
Cd180	1	1.40E-108	2E-33	-0.000720074	0.062431462	0.017504207
Zfp235	1	1.45E-01	1E-10	0.002959595	0.01294184	0.017497856
Lrrc47	0.330436575	1.00E+00	5E-10	0.008322221	0.012386914	0.017472994
Gm31108	1	3.05E-42	3E-65	-0.000558318	0.014062062	0.017469126
Zdhhc1	1	2.18E-19	5E-34	0.001459813	0.018894521	0.017468725
Pold4	1	4.02E-06	5E-28	0.002100615	0.012872759	0.017466936
Slc39a4	1	3.45E-12	4E-28	0.000672014	0.015245092	0.017452821
1810058N1	1	2.94E-49	4E-78	0	0.011441089	0.017449217
Fzd7	1	6.35E-04	2E-14	-0.002806164	0.01490835	0.017440911
Klhl42	1	3.46E-05	1E-07	-0.000138892	0.022435395	0.017437978
Cyp20a1	1	5.93E-02	4E-11	0.006017846	0.017324964	0.017432285
Gm12576	1	1.82E-02	2E-10	-0.000972025	0.015207634	0.017428406
Plec	5.40359E-70	1.00E+00	1	-0.083548824	0.041850387	0.017422264
Pusl1	1	2.05E-05	3E-36	0.001413596	0.009612439	0.017412221
Slc37a2	1	2.29E-72	1E-16	-0.003336803	0.060201651	0.017387805
Taf7	1	1.00E+00	8E-08	0.003130256	0.005409237	0.017383998
Nubp2	1	4.66E-09	4E-31	0.001575778	0.013452398	0.017366707
Ksr2	1	2.50E-218	3E-40	0.000269335	0.122172112	0.017345992
1700010K2	1	1.09E-03	5E-24	-0.000471394	0.010679304	0.017341144

Ss18l2	1	1.00E+00	2E-12	0.001615727	0.004634779	0.017336315
Dse	1	1.88E-111	3E-23	0.001886426	0.076910689	0.017306597
Flna	1	4.99E-137	5E-23	0.000532519	0.07926042	0.017288909
Psmg2	1	6.38E-03	3E-06	0.002723181	0.019813268	0.017283721
Hmgxb4	0.000432518	3.32E-01	0.0903	-0.016556285	-0.019957065	0.017274658
B230303O1	1	3.31E-67	1E-63	0.000116276	0.020841353	0.017263822
Dab2ip	4.13097E-07	1.00E+00	0.003	-0.014326391	0.01781407	0.017257032
Mgat4b	1	5.04E-03	0.0011	-0.001876396	0.027116898	0.017256626
Psrc1	1	1.30E-29	1E-64	-0.000514577	0.010052324	0.017245298
Fpgt	1	1.00E+00	7E-08	-0.000282423	0.009439589	0.017244673
Ets1	0.461656827	8.15E-21	1E-14	0.005620683	0.041105004	0.01722526
Cenpm	1	1.00E+00	5E-44	-0.00140666	0.002906028	0.017216044
Sorcs3	1	5.52E-104	4E-39	-0.000308724	0.044150348	0.017212023
U2af2	1.79459E-11	1.00E+00	0.7799	0.0302689	-0.009786731	0.017202719
BC049715	1	1.16E-08	1E-26	-0.002424505	0.013279974	0.017199374
Ankrd13b	1	1.00E+00	6E-17	-0.002202495	0.005543417	0.017196208
Dtx3	1	2.58E-33	2E-36	-6.87167E-05	0.023882964	0.017176802
Ubl4a	1	3.74E-04	1E-18	-0.00011499	0.012141214	0.017171037
Cntrob	0.518513281	1.00E+00	0.0019	-0.010100359	-0.000439086	0.017164872
3110040N1	1	1.00E+00	6E-09	0.000721031	0.008926201	0.017163938
Zfp28	1	1.65E-02	8E-20	-0.003497951	0.011135475	0.017155417
Stpg1	0.003181002	4.38E-17	2E-20	-0.00512467	0.022258261	0.017153835
Rnaseh2a	1	2.59E-11	7E-07	-0.006875992	0.029829138	0.017137331
Golt1b	1	5.25E-08	2E-20	0.001346685	0.014854524	0.017136034
Ndufv3	1	1.00E+00	0.0013	0.00128196	0.005684555	0.017132701
Lage3	1	1.00E+00	1E-08	0.000839419	0.004316398	0.017121064
Dab2	1	3.07E-32	3E-16	0.003992897	0.041365036	0.017110398
Gm17767	1	2.17E-08	5E-27	-0.001277174	0.012728174	0.017089128
Uqcrb	1	1.00E+00	2E-07	0.00376603	0.011017829	0.017076549
Hs1bp3	0.289428484	2.39E-04	5E-08	-0.007649419	0.020620248	0.017073552
Lyar	1	2.66E-05	3E-13	-0.001153313	0.016550631	0.017072505
Tmem70	1	1.00E+00	2E-06	-0.001490216	0.006477143	0.017072115
Tmed3	1	1.00E+00	1E-21	0.004209282	0.006824137	0.017070627
Ier2	1	1.29E-21	5E-06	0.006907754	0.040301617	0.017069114
9630013D2	1	1.00E+00	0.0003	0.001635378	-0.014321092	0.017029733
Vmn2r29	1	1.00E+00	4E-12	-0.001087559	0.007829648	0.017029616
Dtl	1	3.22E-06	8E-39	-0.001391591	0.007832585	0.017004799
Rwdd1	1	1.00E+00	4E-07	0.007776727	0.002983351	0.016993339
Cables2	1	1.00E+00	0.0079	0.002139522	0.009567163	0.016987451
Gm49454	1	5.17E-24	3E-59	5.10788E-05	0.010197268	0.016984685
Nrxn2	1	2.99E-131	1E-61	-0.000111547	0.035330164	0.016975379
Gm28940	1	7.77E-32	7E-29	0.001169668	0.023734119	0.016972942
Mob3a	1	7.10E-15	4E-32	-0.000801194	0.016293642	0.016970114
Gpr137	1	1.00E+00	9E-05	0.009295085	0.00587208	0.016957223
Prkrip1	1	1.00E+00	0.0007	0.00287071	0.006409961	0.016955261
Ndufs8	1	2.47E-02	6E-20	0.003156869	0.011068825	0.016952376
Poldip2	1	1.00E+00	0.0338	-0.01036684	0.001773773	0.016951932

Ccdc167	1	1.00E+00	2E-06	0.003439166	-0.004209747	0.016946071
Gm3417	1	8.69E-33	5E-27	-0.001402844	0.02472239	0.016945834
Kntc1	1	3.45E-16	2E-47	-0.000919975	0.010845414	0.016941719
4930556J2	1	1.97E-10	2E-14	0.002702115	0.019881534	0.016937677
Hmmr	1	1.87E-07	9E-51	-0.00039467	0.005987574	0.016925454
Plrg1	1	6.77E-04	7E-12	0.001860874	0.01517906	0.016918049
Mrps23	1	1.38E-02	1E-16	0.002387383	0.012752197	0.016914083
Gm45894	1	9.41E-23	1E-26	0.000140131	0.022775799	0.016894791
Fzr1	1	1.00E+00	5E-07	0.000600325	0.001227123	0.016883684
Xlr3a	1	1.69E-101	8E-27	-0.000940671	0.046893848	0.01688079
Abcg3	1	1.82E-125	3E-34	0.000209868	0.068634072	0.016880186
Ccdc146	1	3.26E-65	2E-38	-0.002434696	0.034155569	0.016878127
Mcrs1	1	1.00E+00	3E-12	0.002059481	0.006580611	0.016877978
Myo16	1	6.44E-30	5E-37	-0.001377327	0.01772056	0.01687772
Gm15938	1	2.30E-06	2E-07	-0.003876707	0.023529657	0.016874659
Aldh1b1	1	4.30E-26	9E-33	-0.00165085	0.019091034	0.016873696
Diablo	1	1.00E+00	8E-19	0.003107572	0.004662195	0.016868615
Faap20	1	1.00E+00	3E-15	-0.002341838	0.010373657	0.016867855
Lair1	1	2.78E-161	7E-46	0.000578788	0.066494237	0.016852987
Phf6	1	1.00E+00	9E-05	-0.00369244	0.006570917	0.016851653
Cebpzos	1	6.77E-08	1E-28	0.001567382	0.01201987	0.016850216
Dut	1	1.30E-01	6E-16	0.003941148	0.011211387	0.016846623
Gm16348	1	1.00E+00	0.0065	-0.002749467	0.006807396	0.016844339
Dctn3	1	1.00E+00	1E-11	0.001462956	0.00211154	0.016844243
Gm44127	1	4.73E-42	3E-49	0.000246105	0.019057932	0.016828725
Mrm2	1	7.55E-11	9E-18	0.00323377	0.019035907	0.016818439
Ifi204	1	9.76E-138	1E-29	0.001169322	0.080841842	0.016807635
Ppif	1	7.62E-02	1E-15	0.002417068	0.010890196	0.016802904
Dgcr6	1	1.00E+00	5E-10	-0.002093216	0.00560975	0.016798158
1700001L0	0.444636482	1.00E+00	4E-16	-0.00529685	0.007562081	0.016794401
Gm14391	0.072184135	1.00E+00	6E-11	-0.007129556	0.006434275	0.016789558
Gpx4	0.086230412	3.06E-06	0.0979	-0.014393442	0.042729016	0.016784704
Pced1a	1	1.00E+00	0.0039	-0.000174948	0.000449651	0.016784043
Eri1	1	1.06E-05	2E-06	-0.001465588	0.025068336	0.016780404
1810041H1	1	1.43E-30	8E-52	-0.000114059	0.014610781	0.016778195
4930506C2	1	1.85E-13	1E-17	0.005152122	0.019799431	0.01677787
Ppp1r11	1	1.00E+00	2E-08	-0.00111986	0.01010908	0.016774379
Krt10	1	1.43E-13	2E-35	0.000979716	0.012904056	0.01675726
Gm38393	0.004258284	1.38E-15	1E-19	-0.00561893	0.020114505	0.016756058
Arhgap44	1	1.00E+00	2E-10	-0.005316747	-0.002209118	0.016734136
Cenpa	1.64011E-05	1.00E+00	2E-07	-0.009741312	0.00165678	0.016730085
Erg28	3.48681E-30	1.66E-21	1	0.043569133	-0.047645135	0.016728096
Neurl3	1	1.16E-114	2E-41	0.000172225	0.051495634	0.016721799
Trappc2l	1	1.00E+00	7E-14	0.001459591	0.005534563	0.016721162
Disp3	1	4.54E-37	2E-66	-0.000331445	0.010175746	0.016714576
2810021J2	1	1.76E-01	4E-17	0.002460535	0.010062993	0.016713438
BC048403	1	2.24E-01	4E-11	-0.002057362	0.01220388	0.016701208

Srl	1	5.23E-07	4E-18	-0.003283417	0.016393455	0.016697271
Slco1a6	1	6.13E-12	5E-25	-0.002180588	0.015998987	0.016695829
Stard9	1	3.90E-75	4E-11	-5.19017E-05	0.073232126	0.016676126
Gm11716	1	1.48E-24	2E-43	6.13635E-05	0.014592791	0.01667023
Mettl14	1	1.00E+00	1E-05	-0.000804813	0.011646663	0.016666028
Apex1	1	3.76E-04	2E-29	0.001987945	0.009145954	0.016660231
Scd2	1.76322E-25	1.51E-79	2E-66	0.007241038	0.023244134	0.016656396
Mrpl46	1	1.00E+00	6E-20	0.00016509	0.007155693	0.016639143
Ndufb6	1	3.12E-03	7E-12	-0.001399672	0.015132067	0.016633934
Ccdc32	1	1.30E-02	4E-17	0.000440602	0.011508204	0.016630811
Psmb7	1	2.55E-08	1	0.002635439	-0.047545438	0.016627842
1700082M	1	2.00E-22	2E-34	-0.001049883	0.017359908	0.016627745
Evl	1	1.89E-224	3E-33	0.000422646	0.112935233	0.016621261
Bcl2l12	1	2.34E-01	7E-12	0.001659917	0.012650854	0.016601978
Fasn	3.0346E-30	7.84E-12	0.1749	0.038144798	-0.035018635	0.016594475
Gm43713	1	1.44E-12	1E-05	0.005660984	0.03254912	0.016593618
Fdxacb1	0.027676366	1.00E+00	6E-11	0.007986335	0.005575242	0.016586789
Anapc13	1	3.29E-02	3E-09	-0.002517012	0.015557393	0.016583571
Gm15886	1	7.79E-23	9E-08	0.002046945	0.039665968	0.01658015
Insyn2a	1	1.84E-08	6E-13	0.004675401	0.019939778	0.016574819
Msn	1	2.68E-189	2E-27	0.000896454	0.093290155	0.016569504
Basp1	1	6.30E-229	7E-44	0.00022887	0.092567193	0.016550899
Rwdd3	4.80802E-08	1.00E+00	1	-0.025762058	-0.020089443	0.016537148
Htr1f	1.16689E-08	8.33E-04	0.0004	-0.016871207	0.02343706	0.016531225
Ms4a6c	1	2.44E-104	1E-46	0.000148628	0.041318315	0.016530653
Katnal1	1	1.00E+00	5E-10	-0.002616371	0.003907502	0.016527556
Gm13421	1	1.00E+00	9E-10	-0.000384784	0.007184461	0.01650394
Gm16156	1	1.00E+00	5E-13	0.000782033	0.006731079	0.016495907
Mrps2	1	3.34E-04	1E-22	0.00246368	0.011237654	0.016491399
Snx8	1	7.14E-14	0.1363	0.007990917	0.056272019	0.016475376
Ogg1	1	1.00E+00	2E-13	0.000596651	0.00778429	0.016469386
Zfp9	0.001422401	1.00E+00	9E-16	0.007882308	0.001096588	0.01646922
Gng2	1	1.09E-101	4E-36	0.000232935	0.049480949	0.016460356
Nbdy	1	3.08E-04	4E-33	-0.0001257	0.008835533	0.01645384
Tubb5	1	9.00E-14	2E-23	0.000850757	0.017740677	0.016440275
Hddc2	1	6.00E-06	1E-36	0.001436949	0.008533725	0.016408805
Naf1	1	1.00E+00	1E-06	-0.003328878	0.010848276	0.016400551
Ing4	0.029943158	1.62E-01	1	0.020007179	-0.021787868	0.016395219
Clns1a	1	1.00E+00	0.0037	0.005810546	0.011823885	0.016390636
Ndufa1	1	5.41E-05	5E-14	-0.004258774	0.016093566	0.016377585
Trip13	1	1.27E-15	2E-17	-0.003371549	0.020043375	0.01637666
Gm16794	1	6.28E-08	4E-13	-0.002645547	0.017951883	0.016346522
Fam71f2	1	4.42E-90	4E-42	0.000300202	0.033639839	0.016342161
Nampt	1	1.20E-13	1	-0.000848231	0.091074215	0.016340309
Zfp646	0.00598159	1.00E+00	0.0627	0.015115097	-0.000165746	0.016332577
Rtkn	0.125924475	2.80E-02	8E-06	-0.008755588	0.018150673	0.016326067
Ckap2	1	1.42E-01	7E-40	-0.001451521	0.004875788	0.016319336

4930547M	1	9.53E-40	1E-39	-0.000496101	0.020502151	0.016311877
Atp6v1f	1	3.44E-03	9E-15	0.003560539	0.012903154	0.016306081
Fgfbp1	1	5.25E-37	1E-67	0	0.009235142	0.016254735
Gm3448	5.55251E-05	1.00E+00	1	-0.020620099	0.010081802	0.016252038
Rhbg	0.014972794	1.00E+00	5E-39	-0.002583323	0.00317915	0.016228971
Polr3e	1	1.77E-01	0.0035	0.008375752	0.020073173	0.016227502
Efcab11	1	1.00E+00	6E-27	4.26951E-05	0.004876175	0.016226427
Cenpx	1	4.35E-09	1E-26	0.003332408	0.012736923	0.016220697
Pmm2	1	1.69E-17	1	0.00156149	-0.063725802	0.016184388
Gypc	1	2.10E-02	0.0237	0.00037842	0.022268448	0.016181507
Yrdc	1	1.00E+00	1E-06	0.002332791	0.004536132	0.016179894
Gm26981	1	1.07E-01	2E-13	0.002010698	0.01087305	0.016179702
Cttnbp2nl	1	5.45E-48	1E-07	-0.000833482	0.061564294	0.016177392
Supt5	0.000658779	3.50E-16	1	0.028397167	-0.066968501	0.016176489
Znrd1	1	1.00E+00	3E-14	0.003474211	0.00403769	0.016164608
Cinp	1	1.00E+00	0.0006	0.000701111	0.013432643	0.016149694
4931407E1	1	1.00E+00	3E-15	-0.001020373	0.005094555	0.016142672
Zfp69	1	2.13E-02	5E-15	-0.002566977	0.011872069	0.016132501
1700113A1	1	1.18E-03	2E-12	-0.003445979	0.013717299	0.016127005
Smcr8	1	7.82E-01	0.0004	-0.002680283	0.017747438	0.016114681
Npc2	1	9.90E-14	0.0003	0.004957769	0.042051023	0.016109022
Ebna1bp2	1	3.00E-02	6E-11	0.004390482	0.013594231	0.016105194
Lrrc8dos	1	1.03E-38	2E-28	-0.002351383	0.025002602	0.016099303
Fus	1.10141E-09	1.16E-23	1	-0.041652915	0.119594271	0.016097608
Gnl3l	1	1.00E+00	0.0002	0.000772548	0.002474563	0.016094484
Ap4m1	1	9.32E-06	0.0191	-0.003132723	0.031474468	0.016069201
Vav1	1	1.90E-179	3E-43	-0.00016841	0.068757333	0.016065234
9330160F1	1	1.00E+00	4E-21	0.000350987	0.004616964	0.016062733
Qsox2	1	2.16E-01	1E-10	0.00196322	0.011113002	0.016046184
Gm44777	1	4.71E-12	6E-22	0.000509151	0.016671437	0.016036027
Vcan	1	4.56E-36	1E-47	9.4061E-06	0.016455466	0.016022681
Bub1b	1	2.52E-47	2E-40	-0.000599039	0.021437172	0.016001771
Atf3	1	9.97E-103	5E-38	0.000222701	0.044183223	0.015996201
Gm9949	1	5.75E-47	1E-70	8.1313E-05	0.011070903	0.015983706
Plcg1	1	3.74E-12	1	-0.010289236	-0.051806488	0.015977623
Dvl2	1	1.77E-06	2E-10	0.000700205	0.019348376	0.015969089
Tmem192	1	1.00E+00	0.0006	-0.003728066	0.015039687	0.015967035
Elof1	1	3.14E-01	8E-16	0.002889201	0.010385156	0.015936271
Ccdc163	1	1.00E+00	2E-07	0.003256291	0.012596612	0.015930875
Frg1	1	1.00E+00	0.0034	0.004362663	0.00880563	0.015928545
Spc24	1	1.00E+00	4E-17	-0.001985995	0.006057519	0.0159283
Gm11457	1	3.19E-19	5E-21	0.00211073	0.020715383	0.015917978
Mustn1	1	1.72E-09	1E-31	-0.000266917	0.011492286	0.015909985
Ccdc142os	1	2.27E-07	7E-22	-0.001101088	0.013060256	0.015897816
Nfkbiz	6.68644E-07	1.00E+00	1	0.018468403	0.019813876	0.015896313
2310057M	1	2.21E-04	3E-33	-0.000661359	0.007742656	0.015894696
Thoc6	1	1.27E-02	1E-21	0.003326649	0.00946579	0.015892393

9530062K0	0.000925227	1.78E-12	6E-06	-0.009399761	0.031241012	0.015887159
Gm16168	1	1.67E-57	6E-52	-0.000388452	0.019412313	0.015873864
Rasal2	3.39544E-15	7.76E-08	1	-0.045912259	0.062346316	0.015866905
4930579G1	1	2.88E-11	7E-18	-0.001297497	0.017949117	0.015852084
Bcl2l15	1	8.36E-01	3E-18	-0.003466035	0.009115346	0.015829659
H2afx	1	1.18E-07	7E-21	-0.000137504	0.013850724	0.015820842
Jmjd6	1	1.00E+00	0.0095	0.004936206	-0.009236448	0.015802336
Gm45330	1	2.66E-08	8E-44	0.000747347	0.00795209	0.01579659
BC051226	1	8.95E-18	4E-28	0.001279787	0.016582417	0.015784479
Dll1	1	1.00E+00	1E-15	-0.000811773	0.005034708	0.015766913
Aldh18a1	1	2.96E-63	5E-53	-1.58978E-05	0.021494152	0.0157627
Tradd	1	1.85E-02	7E-11	0.000702623	0.013007652	0.015761041
Nrros	1	3.61E-161	2E-14	-0.001132893	0.106303014	0.015754547
Cd3e	1	7.61E-53	9E-59	-0.00073255	0.015993653	0.015745166
Natd1	1	1.00E+00	7E-07	-0.003465434	0.010068889	0.015741154
Dcp2	0.118575784	1.00E+00	0.0019	-0.010093394	0.017148762	0.015731652
Cenps	1	1.17E-22	2E-54	-0.000314283	0.009827082	0.015728407
Gatc	1	2.85E-10	2E-16	-5.85578E-05	0.017306112	0.015725656
Gtf3c5	1	1.00E+00	4E-14	0.003179639	0.008928935	0.015720215
Znhit3	1	7.50E-02	1E-25	0.002016282	0.007583106	0.015715822
Tnfrsf12a	1	1.75E-14	8E-28	0.001204883	0.014781427	0.015715754
Tmed8	1	3.19E-02	2E-14	-0.000372286	0.010622392	0.015713813
Rdh13	1	1.00E+00	0.0003	0.007639335	0.003700875	0.01571285
Ptk2	1	6.92E-08	1	-0.009990651	0.062049917	0.015708008
Zfp457	1	1.00E+00	9E-11	-0.000797753	0.00901102	0.015701359
Zfp422	1	4.40E-05	1E-16	0.002784172	0.012765115	0.015694126
Rpusd4	1	3.09E-07	2E-17	0.003120741	0.014204895	0.015691567
Hormad2	0.010835988	6.17E-07	2E-37	0.003863821	0.008624465	0.015670216
AA792892	1	1.00E+00	9E-24	-0.000201428	0.005950473	0.01566471
Igdcc4	1	5.01E-40	1E-63	0.000205514	0.011446574	0.015659509
Mndal	1	4.18E-69	5E-06	0.0029463	0.096809547	0.015654595
Mrps24	1	1.00E+00	6E-05	0.001781739	0.001516751	0.015648465
Ramac	1	1.00E+00	7E-12	-0.001312884	0.00459435	0.015646412
Mpv17	1	1.00E+00	0.0053	0.002914315	0.009776025	0.015632097
Bckdk	1	1.00E+00	2E-08	-0.000465568	0.011328377	0.015613137
Vcpkmt	1	1.90E-14	2E-32	0.00065491	0.012565378	0.015612575
Stab1	1	3.75E-64	7E-06	0.001543078	0.079771306	0.015596403
Wee1	1	5.43E-35	1E-23	0.000203738	0.025434284	0.01559614
Dnajc14	1	1.00E+00	9E-05	-0.001968014	0.008534135	0.015592193
Tha1	1	6.24E-01	5E-12	0.00055053	0.012491815	0.015591858
8430429K0	1	6.90E-01	4E-06	-0.004984766	0.013723692	0.015589354
Lysmd4	0.08960524	6.67E-03	0.0705	0.01371078	0.028234507	0.015582987
Polr1e	1	3.87E-14	1E-26	0.001882111	0.014644835	0.015579124
Gm128	1	2.55E-19	8E-53	-0.00046084	0.009651974	0.015577798
Nckap1l	1	4.35E-197	6E-36	2.35744E-05	0.07859658	0.015575427
Mrpl44	1	4.21E-03	1E-18	0.002792024	0.010532635	0.015570457
Ankle1	1	2.98E-11	4E-55	-0.000240162	0.005846487	0.015567228

Irak2	3.02359E-06	2.08E-38	1	-0.035986535	0.161350145	0.015558713
Ttll12	1	3.58E-06	1E-22	-0.00032641	0.011391802	0.015556017
Cmtm7	1	7.26E-76	8E-34	-0.001928477	0.038413577	0.015551914
Kifc1	1	3.38E-25	1E-51	-4.62299E-05	0.010566783	0.015540229
Slc22a21	1	1.00E+00	1E-12	-0.000875248	0.007041993	0.015524149
1700055D1	1	3.72E-09	8E-26	0.000107419	0.012369119	0.015508561
P2ry14	1	4.04E-21	2E-16	0.002371122	0.028988209	0.015507629
Cetn1	1	1.00E+00	3E-44	-0.001492604	0.002803635	0.015494493
Gab3	1	2.29E-98	3E-28	0.000313996	0.055052328	0.015493286
Mtln	1	2.15E-31	4E-30	0.001420401	0.020727217	0.015483809
Rad9a	1	1.80E-02	8E-15	-0.00164027	0.011822885	0.015454461
Wdyhv1	1	2.62E-02	1E-07	0.00332622	0.016033638	0.015446349
Gm26569	1	3.36E-60	1E-45	0.000406121	0.024616423	0.015442193
Casp7	2.3853E-29	1.00E+00	0.715	-0.031130435	0.00159079	0.015439135
Mydgf	0.125669872	1.00E+00	0.0006	0.010976616	-0.004869726	0.015438709
Vps37a	1	2.22E-07	1	-5.77144E-05	-0.047601273	0.015436793
Gm41492	1	3.74E-03	2E-45	-0.001036863	0.00472326	0.015428671
Tecta	1	4.85E-12	8E-50	0.000227186	0.007903753	0.015421271
Cavin2	1	1.00E+00	1E-29	0.00136349	0.004618999	0.015416592
Gm11973	1.22945E-19	5.39E-19	0.1084	-0.023126096	0.05053101	0.015402032
Arl1	1	1.00E+00	2E-05	0.002333981	0.005108462	0.015390054
Calml4	1	1.50E-19	1E-47	0.00035882	0.010304369	0.015389048
Zfp35	1	1.00E+00	4E-06	-0.005659555	0.004561187	0.01538577
Rraga	1	2.46E-05	6E-19	0.002875657	0.012180729	0.015382961
Dusp5	1	6.36E-30	8E-42	-0.001280018	0.017457418	0.015370972
Mfap3	1	1.00E+00	0.0107	-0.001842531	0.000837928	0.015360066
Zfp937	1	1.00E+00	2E-20	-0.000746361	0.004825444	0.015356918
A930009A1	1	1.00E+00	6E-07	-0.001056552	0.001563194	0.015353162
Hgsnat	0.003772972	1.00E+00	1	-0.01766606	0.020386003	0.015340404
Parvb	1	7.60E-109	3E-39	2.81316E-05	0.040798881	0.015338578
Glrx	2.4938E-59	1.00E+00	0.0385	0.05128447	-0.011338548	0.015337227
Cip2a	1	2.58E-03	8E-28	-0.001874522	0.007762376	0.015334072
Zfp623	1	1.00E+00	7E-05	0.00642778	0.011777108	0.015332712
Gm20658	1	1.38E-131	3E-53	0	0.047677386	0.015332488
Defb1	4.01086E-20	1.31E-08	2E-17	0.013888518	0.014750235	0.015329339
Slc2a8	1	5.23E-04	2E-16	0.002188184	0.011727379	0.015322793
Bnc2	1	6.92E-30	2E-35	2.64729E-05	0.016990667	0.015318839
Birc3	1.78529E-08	1.00E+00	1	-0.024718652	0.053217007	0.015313242
Trdmt1	1	6.11E-23	1E-31	-0.000988127	0.017051251	0.015310939
Dynlrb1	0.003671406	1.00E+00	0.0022	0.012770682	-0.002116442	0.015297616
Tctn1	1	1.00E+00	4E-09	0.00024485	0.006641137	0.015293533
Gm20186	1	8.30E-06	6E-10	-0.001457539	0.017144941	0.015278546
Ccdc18	1	1.00E+00	0.0001	-0.000864787	-0.00223238	0.015258147
Commd6	1	5.74E-01	7E-09	0.00168005	0.012688383	0.015244604
9430085M	1	3.56E-06	1E-29	-0.002585834	0.009086925	0.0152349
Pced1b	1	5.63E-07	1	-0.020001619	-0.040887792	0.015229399
Ikzf1	1	1.20E-135	6E-22	-0.000892431	0.079092482	0.015209269

Ndufaf3	1	3.56E-11	7E-36	0.001121115	0.010227789	0.015206629
Cxcl1	1	1.48E-37	0.0009	0.003546861	0.046213836	0.015202202
Zfp513	0.894288222	1.00E+00	2E-06	0.007805774	0.006075307	0.015201847
Cyb5d1	1	1.00E+00	4E-22	-0.000349766	0.005764072	0.015190096
Exo5	1	1.00E+00	9E-25	-0.001755253	0.005559537	0.015183113
Elp4	1.97347E-12	1.00E+00	1	-0.053575174	0.020139465	0.015178935
Rcsd1	0.078912468	1.68E-69	6E-23	0.003484367	0.046801161	0.01515212
Bcdin3d	1	1.00E+00	5E-11	-0.00042133	0.010143679	0.015127922
Slc41a3	1.19907E-10	3.98E-08	7E-16	-0.007748825	0.016269897	0.015093821
Zbtb6	1	1.00E+00	2E-07	-0.001875131	0.006493936	0.015072893
Gm20125	1	1.00E+00	4E-28	-0.001666878	-0.000879497	0.015064998
Ttc32	1	5.64E-04	7E-06	-0.005065201	0.018549197	0.015062958
Tonsl	1	1.00E+00	7E-19	-0.002090015	0.005383019	0.015060703
Fabp4	1	6.35E-18	6E-47	-0.000405373	0.011399134	0.015029914
Nkap	1	2.59E-01	0.0288	0.005195843	0.01906972	0.015026113
Smim12	1	3.71E-07	7E-15	-0.000238599	0.015472444	0.015024776
Ecm2	1	5.42E-35	2E-43	0.000537609	0.015687397	0.015018769
Gm17590	1	3.62E-22	2E-24	-0.00144709	0.018989584	0.015005926
Nemp1	2.13067E-05	7.61E-09	3E-06	-0.010967304	0.025405414	0.015005234
Twistnb	1	1.00E+00	0.008	0.008901359	0.002276441	0.01498462
Mei4	1	1.00E+00	6E-10	0.002301729	0.003570591	0.014974389
Evc2	1	5.61E-16	9E-41	-0.001165578	0.010496782	0.014971326
A730036I1	1	1.87E-11	5E-38	0.001928712	0.00941664	0.014971072
Zfp51	1	1.00E+00	7E-14	0.001475193	0.008278353	0.014969965
Haus3	1	8.30E-06	9E-27	-0.000439273	0.009828496	0.014964453
Haghl	1	1.42E-01	2E-19	-0.001132937	0.008996214	0.014952347
Cops6	1	1.00E+00	0.0005	0.002358803	0.003912107	0.014946114
Ppp1r9b	1	6.47E-81	4E-47	0.000314007	0.029425171	0.014936062
Zfp772	1	1.00E+00	1E-16	0.002372952	0.003678866	0.014935969
1500004A1	1	1.30E-07	0.0006	0.006604919	0.02832171	0.014934323
Gm3235	1	2.09E-05	2E-12	0.00038872	0.014583066	0.014928811
Cxxc1	5.8451E-07	1.00E+00	0.0637	0.018418977	-0.007522433	0.014919823
F420014N2	1	3.17E-17	6E-45	0.000158431	0.010640454	0.014916356
Prkch	0.003590235	2.31E-75	6E-15	0.005507653	0.06473892	0.01491571
E2f1	1	5.96E-05	2E-13	-0.003742982	0.013139391	0.014911218
Pfn1	1	1.33E-10	0.0004	-0.004250182	0.033935548	0.014909737
4932422M	1	4.31E-04	6E-12	0.004379306	0.013604918	0.014894707
1700054M	1	2.22E-26	7E-48	-0.000886937	0.01221327	0.014889322
9430060I0	1	4.95E-18	2E-22	0.003092126	0.01757683	0.014886885
Gm5122	0.002308739	1.48E-06	6E-49	0.002913884	0.005486111	0.014886424
Gm43821	0.000957982	7.14E-18	2E-15	-0.006383543	0.022798719	0.014878327
Cyb561	1	2.14E-30	4E-34	0.00023752	0.017383403	0.014858625
Hpgds	1	4.99E-59	2E-17	-0.000861891	0.042770484	0.014857225
Uchl5	1	2.27E-03	0.0292	-0.002113225	0.02495251	0.014855505
Ufd1	1	1.42E-03	1	0.003716225	-0.031080655	0.014854665
Nlrx1	1	2.18E-02	2E-09	-0.003188724	0.013252966	0.014849733
Nfya	1	1.00E+00	0.0069	-0.003815979	0.017205238	0.014845131

Pirb	1	1.58E-166	2E-33	-0.000534625	0.067438206	0.014839041
Gm36445	1	1.51E-02	4E-11	0.000860304	0.011944765	0.014834152
Ankrd9	1	1.67E-36	9E-41	0.000288356	0.017118679	0.014833061
Slc41a2	0.000171479	1.00E+00	1	-0.020912686	0.021849984	0.014815015
Rabl2	0.101593377	2.81E-02	8E-15	0.006080554	0.01052184	0.014813295
9130401M	1	8.62E-09	5E-13	0.001445668	0.017031223	0.014809011
Arhgap30	1	3.18E-151	6E-30	-0.000471087	0.064044409	0.014806664
Gm10516	0.000265815	2.59E-04	3E-06	-0.009926103	0.016892169	0.014805946
2410018L1	1	4.97E-03	2E-36	0.00023094	0.00476994	0.01480551
Zfp27	1	1.00E+00	2E-09	0.003392903	-9.71885E-05	0.014804577
Tlr3	0.001484013	1.00E+00	7E-09	-0.00782262	0.011385134	0.01479424
Psmg1	1	1.00E+00	0.002	-0.00547937	-0.002029116	0.014787564
Grm7	1	1.01E-02	7E-10	0.001661615	0.013092796	0.01478718
Gm37233	1	4.01E-82	9E-60	-0.000155434	0.020511284	0.014780663
Mogs	1	1.00E+00	2E-09	0.00516174	0.00624257	0.014774148
Tmub2	1	1.00E+00	1E-08	0.002918232	0.003949103	0.014756281
G6pc3	1	5.21E-01	7E-05	0.008071285	0.015876775	0.014752772
Cldnd1	1	5.46E-03	8E-10	5.19588E-05	0.013942008	0.014747276
Mpeg1	1	7.78E-166	2E-32	-0.00050173	0.066364059	0.014745278
Mob3b	1	6.37E-06	1	0.00487414	-0.074921151	0.014740033
Gm15492	1	5.26E-10	1E-13	0.001407505	0.017164237	0.014739688
Anapc15	1	2.61E-25	1E-07	0.001116071	0.049364147	0.014739381
Macc1	1	8.12E-08	3E-34	-0.001236937	0.009336549	0.014734595
Psmg4	1	1.12E-15	2E-17	0.001329119	0.018432503	0.014733971
Rgmb	1	1.00E+00	1E-15	-0.001804318	0.005588187	0.014720981
Samd10	1	1.07E-17	5E-42	-0.001403365	0.011062118	0.014720686
Dmac1	1	1.00E+00	1E-22	0.00129762	0.005683485	0.01471463
Slc26a10	0.015817295	6.34E-08	1E-19	0.005034976	0.014817747	0.014693992
Lyrm9	1	1.00E+00	5E-06	-0.006423364	0.008742822	0.014692074
Coq4	1	8.47E-02	1E-12	0.001417692	0.01035711	0.014684548
Gm28375	1	4.46E-24	0.0002	0.004027302	0.043110005	0.014677079
AW112010	1	6.89E-03	6E-10	-0.0002793	0.015338688	0.014664001
Vmn1r4	1	8.65E-14	2E-12	-0.003378319	0.022161703	0.014659597
Gm12264	1	2.12E-03	5E-17	-0.002296404	0.010069696	0.014652552
Spag5	1	6.17E-04	4E-27	0.000179165	0.007941752	0.014646
Ppie	1	1.00E+00	0.0003	0.004891138	0.010968666	0.01464525
Pigw	1	1.00E+00	2E-13	0.001968548	0.006705914	0.014644394
Mrpl40	1	7.60E-01	3E-10	0.002904624	0.010558747	0.014625086
Sh3pxd2b	0.865068872	1.16E-124	1E-20	-0.002413095	0.071480326	0.014622882
Chadl	1	1.38E-07	9E-24	-0.000308828	0.011488336	0.01459271
Btg2	1	1.00E+00	6E-08	-0.002441962	0.005585681	0.014573628
Sgo1	1	3.66E-16	1E-47	0.000679369	0.008296413	0.014572578
Itga4	1	1.73E-90	6E-22	0.00256173	0.054191035	0.014568705
Chmp5	1	1.00E+00	0.0158	0.004229221	0.005896471	0.014565027
Fam111a	1	1.79E-75	2E-06	0.003397787	0.066517993	0.014563234
Dusp16	1.61078E-09	1.00E+00	1	0.045546571	-0.029952563	0.014548267
Tprgl	1	5.50E-07	3E-12	0.001103576	0.017237722	0.014541703

Cstf2	1	1.00E+00	6E-05	-0.003101945	0.006168406	0.014526318
Gm29927	1	1.17E-42	5E-61	8.57749E-05	0.010372727	0.014510656
Map4k1	1	1.62E-49	8E-38	0.000136457	0.021077608	0.014506484
Calm3	1	1.92E-05	2E-16	-0.00172244	0.012314591	0.014499089
Fcer1g	1	7.92E-34	4E-18	0.001715217	0.029325568	0.014498031
Mrpl49	1	2.10E-08	4E-20	0.001906587	0.012733324	0.014468319
Zfp830	1	1.00E+00	0.006	-0.003201355	-0.001645033	0.01446656
Lrrc8c	1	5.56E-43	1E-20	0.002650122	0.03527559	0.014465693
Exoc2	0.000113325	1.00E+00	1	-0.023242553	-0.001171921	0.014464589
Zbtb49	1	1.00E+00	1E-05	0.000308681	-0.001055367	0.014462604
Pik3cd	1	4.63E-199	2E-27	-0.000195162	0.088069819	0.014457409
Cyyr1	1	6.03E-10	7E-11	0.001364838	0.027233215	0.014451659
Imp3	0.001161242	1.00E+00	1E-07	0.009427631	0.011982399	0.014449994
Acad9	1	1.00E+00	3E-05	0.000704951	0.002171918	0.014435481
Snrpg	1	1.00E+00	2E-05	-0.000968299	0.010898487	0.014434095
Otub2	1	1.60E-12	1E-24	0.000472283	0.013525809	0.014426729
Cybc1	1	2.02E-01	1E-13	0.002728753	0.01144404	0.014425863
Pigf	1	3.67E-07	2E-07	-0.002753499	0.018687288	0.014425745
Mrps33	1	1.00E+00	0.0001	-0.002675813	0.010148322	0.014420054
D430001F1	1	1.02E-13	7E-39	0.001208428	0.009037208	0.014418395
Abitram	1	1.04E-10	1E-26	-0.000427787	0.012365427	0.01441467
Ccdc78	1	3.34E-23	1E-37	0.000778748	0.013265029	0.014404535
Mtrf1	1	1.00E+00	1E-05	0.00062502	-0.001175384	0.014402473
Mdp1	1	1.74E-03	3E-07	0.005419796	0.015885276	0.014398232
Poglut1	1	5.86E-07	7E-08	0.006095953	0.019469152	0.014397791
Gm13402	1	1.56E-04	0.0007	0.003224	0.021471759	0.014392817
P4ha2	1	3.49E-01	1E-13	-0.000481045	0.009302964	0.014380093
Pcif1	1	1.00E+00	0.0029	0.005818182	0.001309497	0.014371551
Slc9a3r2	1	6.74E-09	1E-18	0.001498891	0.014570557	0.014355729
Gm13565	1	2.17E-37	1E-58	0.000235015	0.009982219	0.014349428
Ifi203	1	7.97E-80	1E-13	0.003355601	0.06877458	0.014336855
Rpa3	1	3.35E-06	6E-19	0.00018879	0.011935088	0.014323693
Pigh	1	1.71E-10	1E-30	-0.000524604	0.010305732	0.014323416
Etfrf1	1	1.00E+00	0.0054	-0.002116251	-0.001798127	0.014311963
Izumo1	1	6.14E-26	9E-60	-8.92378E-06	0.006865274	0.014310458
Itgal	1	5.18E-89	2E-26	-8.23445E-05	0.047707093	0.014306306
Gm26737	1	1.03E-14	5E-38	-0.000736625	0.010001237	0.014268622
Atp8b4	1	3.70E-59	7E-05	-0.003560721	0.070930759	0.014266719
Mmrn2	1	3.23E-11	2E-22	-0.002693341	0.013791203	0.014266275
Sec22a	3.18153E-10	1.00E+00	1	-0.030889578	0.0181522	0.014262839
Prr13	1	1.00E+00	0.0262	-0.00035937	0.019384942	0.0142628
Ak7	1	2.47E-42	9E-31	-0.000517773	0.022024563	0.014253863
Ndufb4	1	1.00E+00	0.0008	-0.003241968	0.008711641	0.014251271
Fktn	1	1.00E+00	0.0209	-0.008201909	0.016241943	0.014249646
Cdk14	1	2.77E-127	2E-10	0.004023667	0.099721054	0.014247952
Mbd6	0.000508218	2.70E-01	1	0.01906469	-0.01864616	0.014244527
Alkbh4	1	1.00E+00	1E-10	0.002425492	0.00888956	0.014241701

Abhd11	1	1.00E+00	0.0009	0.006135311	-0.006730662	0.01421906
Gm17036	1	1.98E-10	9E-19	0.004336813	0.015046671	0.014214956
Gm33037	8.75775E-18	1.00E+00	1	-0.038241684	-0.022639369	0.014199003
Clec4f	1	2.57E-37	3E-07	-0.001171854	0.048913348	0.014196682
Gm34517	1	6.47E-12	4E-60	-9.46987E-05	0.003690818	0.014192414
Lrp11	1	1.43E-09	2E-25	-0.00108178	0.011329136	0.014191691
Gm49164	1	1.07E-16	3E-33	0.00155803	0.012453774	0.014179058
Poll	1	6.40E-02	5E-14	0.000838614	0.009724449	0.01417244
Dnaja4	1	7.18E-18	6E-27	-0.001601155	0.014441744	0.01416058
Oxct1	1	7.51E-53	1E-20	-0.000500791	0.040716039	0.014156396
Il7r	1	3.29E-131	2E-33	-0.000293801	0.060252807	0.014154924
Exosc4	1	2.59E-03	3E-13	0.001063502	0.010752486	0.01414823
C1qb	1	1.29E-100	6E-33	-0.001003536	0.043569505	0.014146074
Tsr2	1	8.12E-02	7E-12	-0.002641245	0.010407808	0.01412335
Snapc5	1	6.61E-11	2E-15	6.02671E-05	0.017088709	0.014113798
Gm48884	1	9.26E-32	1E-40	-0.000987829	0.014468313	0.014106938
Mettl21a	1	1.00E+00	3E-05	-0.002415574	0.008265522	0.014103399
Usp30	1	1.00E+00	0.0011	0.002703863	0.006034951	0.014095475
Scrn1	1	3.64E-28	7E-54	-0.000376064	0.009583103	0.014092842
Ddx28	1	1.21E-01	2E-16	-0.000124997	0.008541032	0.014089768
Mir142hg	1	2.04E-179	9E-37	-1.93382E-05	0.067236421	0.0140799
Otulinl	1	3.19E-168	7E-37	-0.000349858	0.066219919	0.014073304
Plxnc1	1	1.43E-23	4E-07	0.001893665	0.040270125	0.014059506
Ly86	1	2.13E-95	2E-23	-0.001407298	0.057989456	0.014042557
Gm20686	1	1.06E-26	8E-39	0.000463225	0.013269971	0.014023511
Eif1b	1	1.08E-06	7E-12	0.003467017	0.015437986	0.014016318
Entpd2	1	3.18E-59	1E-61	0.000424413	0.013599686	0.014016219
Fkbp3	1	1.00E+00	0.0001	0.001252967	0.015085272	0.014004937
Fam98c	1	1.00E+00	0.0014	-0.000253432	0.003672172	0.013997707
Pbxip1	1	2.09E-02	6E-06	0.001224084	0.020980858	0.013990115
Hk3	1	6.34E-201	2E-29	-0.000889618	0.091388455	0.013978329
Fem1a	1	3.55E-01	0.0095	0.004856207	0.018362763	0.01396819
Mapk13	1	4.78E-26	1E-54	-3.83528E-05	0.008604641	0.013961934
Llgl1	1	1.00E+00	4E-07	0.000688325	0.008898419	0.013960369
Tes	0.738773174	4.84E-30	2E-33	-0.002151839	0.01837812	0.0139513
Maz	1	1.49E-03	0.0038	0.002347141	0.022651319	0.013942129
Csf1	1	5.91E-28	2E-12	0.000775421	0.034411536	0.013934481
Asb7	6.22488E-06	2.30E-15	1	-0.021014479	-0.050708038	0.013933669
Morrbid	1	3.45E-124	9E-13	0.000508261	0.091485075	0.013931006
Hist1h4h	1	7.43E-17	2E-23	0.001307507	0.015153292	0.01392237
Rusc1	1	1.20E-02	1E-14	0.002865171	0.010671543	0.013922192
Cln3	1	1.56E-35	0.0173	0.005410311	0.066507373	0.01391694
Mad2l1	1	4.43E-16	4E-33	-0.000446757	0.011529439	0.013909282
Slc7a8	1	3.41E-124	2E-39	0.000782895	0.050255032	0.013908601
Ncapg	1	5.41E-15	5E-46	0.000463837	0.00734152	0.013905211
Tex30	1	3.78E-02	0.0077	-0.001723594	0.019317686	0.013874997
Gm38948	1	2.06E-06	1E-18	-0.001906127	0.011929157	0.013852974

Cdk9	2.83274E-15	1.00E+00	1	0.029792422	-0.008496999	0.013851307
Hsbp1	1	2.39E-02	4E-06	-0.001228525	0.015141998	0.01385009
Baz2b	0.008695253	7.26E-18	1	0.029622116	0.129609804	0.013848601
Ulk3	1	1.00E+00	8E-10	0.003293065	0.008071721	0.013847538
Pon1	0.005532227	7.78E-74	1	0.032901213	-0.173705253	0.013838989
Pik3r5	1	1.12E-242	2E-35	-0.000606545	0.100236523	0.013830344
Cfl1	1	8.45E-12	2E-05	-0.003031086	0.028146992	0.013827711
Gm27252	1	8.74E-32	3E-28	0.001094344	0.018984538	0.013826582
Gm42567	1	3.54E-05	3E-23	-0.003077549	0.009229667	0.013822383
Dennd2c	1	9.59E-15	3E-31	0.00096675	0.012029355	0.013821366
Slc34a2	1	4.45E-18	1E-41	-0.000782364	0.010627609	0.01382014
Pars2	1	7.14E-14	4E-34	-0.000815328	0.010800569	0.013817492
Map1s	1	2.90E-18	1E-11	0.001672945	0.025109888	0.013814791
1700084C0	1	1.00E+00	0.0478	0.001137738	0.008175372	0.013814403
Rab35	1	1.00E+00	0.0003	-0.00116824	0.01075892	0.013811226
Mtch2	2.62632E-06	1.23E-03	1	0.030850669	-0.037509897	0.013806318
A530013C2	1	3.57E-02	4E-07	-0.004090323	0.01469608	0.013800606
4930412C1	1.34511E-06	1.00E+00	1	-0.018812942	-0.004810907	0.013791042
Smim26	1	9.20E-05	2E-23	0.003429786	0.009257409	0.013769516
Map3k11	1	1.00E+00	0.008	0.001340921	0.014580067	0.013745246
Gm14762	1	1.16E-23	2E-27	-0.001511821	0.015811527	0.013737217
Adam8	1	6.11E-89	4E-39	0.000594472	0.036279567	0.013735596
Nusap1	1	3.13E-02	2E-22	-0.001143801	0.007192623	0.013709834
Ropn1l	1	1.00E+00	1E-09	0.00019456	0.005060309	0.013704832
Pla2g4a	1	6.81E-103	1E-34	-1.11719E-05	0.048566109	0.01370471
Hck	1	5.65E-163	2E-28	-0.000428536	0.074558346	0.01369972
Mrtfa	0.000387339	1.24E-16	1	-0.026890831	0.109503714	0.013696703
Ccdc115	1	3.56E-04	2E-11	0.004186899	0.012937228	0.013685207
Ush2a	1	5.14E-24	2E-24	-0.002145427	0.018265998	0.013665857
Yipf2	1	1.00E+00	0.0029	-0.001108808	-0.002654044	0.013640427
U2af1l4	1	3.11E-04	1E-23	0.002359994	0.008595331	0.013628269
Phf3	2.16114E-12	1.00E+00	1	0.047592093	0.017713186	0.013624734
Lrwd1	1	2.06E-01	8E-06	0.000189263	0.014307611	0.013611022
Gm26901	1	2.44E-01	8E-10	0.002464249	0.010151489	0.013609146
Gm10767	1	5.36E-07	6E-26	0.000886341	0.00934724	0.013600312
Pcna	1	3.77E-20	1E-11	-0.00016681	0.025720194	0.013585244
Sdr39u1	1	1.43E-08	4E-23	0.000990158	0.011880997	0.013584613
Nudt18	1	2.49E-18	6E-32	0.000479535	0.013011064	0.013580415
Bpgm	1	2.47E-01	1E-05	0.00104319	0.013219966	0.013568456
1700030N0	1	3.37E-12	1E-21	8.04767E-05	0.012747367	0.013534553
Lrch4	1	1.18E-05	3E-09	0.004041995	0.016129497	0.01352883
Zfp984	1	4.67E-03	5E-09	-0.004342996	0.016087044	0.013517803
Gm16110	1	1.44E-31	8E-58	3.39637E-05	0.008325695	0.013496693
Lsm8	1	1.18E-04	6E-18	0.001613494	0.010027	0.013494125
Glipr1	1	1.26E-79	5E-26	-0.000189743	0.041076801	0.013478788
Ccdc51	1	1.00E+00	0.0083	0.000297127	0.014475162	0.013473604
Dr1	1	1.00E+00	0.0009	0.000357057	0.011655506	0.013461916

Zfp97	1	1.00E+00	0.0003	0.004072712	-0.001128357	0.01345929
Emc9	0.000331842	1.00E+00	0.0158	-0.011910901	0.009826706	0.013447802
Pard6b	1	1.00E+00	8E-07	-0.001573742	0.006997105	0.01343564
Klhl41	1	9.82E-09	4E-23	-0.001166244	0.011065472	0.01343003
Arsg	0.139492885	2.57E-09	1	-0.022371671	-0.047373058	0.013423451
Cox5a	0.000148193	1.00E+00	1	-0.018530831	0.003844985	0.013420765
Gm10032	1	2.09E-09	6E-34	-0.000408668	0.007620326	0.013415416
Zfp442	0.020402957	1.00E+00	9E-07	-0.007151661	-0.000501739	0.013414569
Lgalsl	1.00298E-11	1.00E+00	5E-09	0.012888164	0.006511685	0.013399312
Ccdc181	1	6.30E-08	2E-14	-0.002004949	0.013850054	0.01339245
9330151L1	1	1.00E+00	4E-06	0.004573238	0.002742533	0.013390538
Polq	1	1.24E-11	2E-35	-0.000241773	0.008674039	0.01338871
Zfp593	1	1.67E-09	9E-35	0.001353254	0.008360134	0.013380277
Dbr1	1	9.47E-04	5E-09	-0.001101373	0.013029811	0.013367333
Gnptg	1	1.70E-01	1E-06	0.003982659	0.012986958	0.013355262
Pheta1	1	1.00E+00	5E-09	-0.00443043	0.002472978	0.013351759
Six4	1	3.41E-27	2E-55	3.84256E-05	0.00711631	0.01335048
Ms4a4a	1	3.04E-69	1E-49	-8.92378E-06	0.029924312	0.013347288
Pin1	1	2.02E-01	3E-10	0.001790092	0.010505525	0.013344797
Eml2	1	1.00E+00	0.0128	-0.007315449	0.015548494	0.013334407
Bspry	1	1.38E-57	8E-51	-0.000272442	0.016394322	0.013323711
Zfpl1	1	1.00E+00	3E-07	0.003537881	0.006922159	0.013319275
Kif22	1	2.70E-10	2E-29	-0.000109308	0.009741028	0.013316164
Fam129a	1	4.87E-67	3E-25	-9.50216E-05	0.037639544	0.013307504
Lyz2	1	1.65E-151	2E-28	-0.001453769	0.057772686	0.013303942
Cntnap2	1	1.00E+00	0.0002	0.000895758	0.011997874	0.013302095
2810407A1	1	4.04E-28	5E-37	0.000612345	0.014089069	0.013293551
Zcchc3	1	8.89E-09	2E-45	-0.000626125	0.005342612	0.01329165
Sdhaf4	1	1.00E+00	0.0021	-0.002995165	0.002512202	0.013284045
Il11ra1	1	3.47E-09	1	-0.009086212	-0.039007908	0.013282697
Cyth4	1	9.07E-165	1E-28	2.62321E-05	0.067171395	0.013280636
Arl8a	1	8.56E-12	0.0021	-0.002876669	0.034797538	0.013278973
Dph7	1	7.63E-03	1E-08	0.002084002	0.012788966	0.013273494
Wnt10a	1	1.61E-61	1E-56	0	0.013969872	0.013237259
1110065P2	1	8.03E-03	3E-14	0.002146401	0.010374993	0.013222849
2700038G2	1	1.33E-46	9E-36	0.000654307	0.019600796	0.013218966
Insl6	1	2.31E-35	1E-54	-0.000284096	0.010000443	0.013209699
Spag16	1	1.28E-16	5E-16	-0.000914592	0.018862547	0.013202459
Nudt19	1	1.69E-01	6E-08	-0.004905701	0.010689583	0.013185652
2010320M	1	1.00E+00	2E-14	0.003568026	0.004114429	0.013182292
Zfp65	1	1.00E+00	0.0027	-0.002329951	-0.005033068	0.013171028
Cd151	1	1.82E-02	2E-25	-9.20728E-05	0.007085017	0.013165995
Slc50a1	1	1.50E-02	8E-16	-0.000732591	0.010472366	0.013140626
Ccne1	1	1.74E-19	3E-45	0.000107352	0.00859067	0.013136553
Gm48441	1	1.06E-37	1E-45	-3.02342E-05	0.013265312	0.013131547
Ube2k	0.35668386	1.34E-05	1	0.021725336	0.076599615	0.013129097
A830052D1	1	2.18E-02	2E-15	-0.001638687	0.008999041	0.013115307

Tcof1	1	1.00E+00	0.0334	-0.000339582	0.011382046	0.013102792
Epsti1	1	1.23E-75	3E-18	-0.00053597	0.055347117	0.013093797
Trp53rkb	1	1.00E+00	8E-11	0.0026936	0.006077069	0.013091781
E2f5	1	1.00E+00	0.0003	0.002187814	-0.001191773	0.013088069
Gmppb	1	7.72E-04	5E-22	0.003128576	0.007891266	0.013072676
Ddias	1	1.55E-17	4E-27	0.000322066	0.013076832	0.013069491
Wdr35	1	1.00E+00	2E-05	-0.00389063	0.010733637	0.013058534
Maf1	0.015572679	1.00E+00	0.0059	0.010859598	0.006158787	0.013056962
Cdca4	1	1.00E+00	1E-14	-0.000273215	0.006897915	0.013051197
Nrxn1	4.7999E-135	1.00E+00	1	0.073819343	0.029585672	0.013040585
Sptlc3	1	8.64E-18	2E-11	-0.000135457	0.021777067	0.013036996
Phf11d	0.354335415	1.68E-15	2E-11	0.005470491	0.022240722	0.013030806
Tbl2	1	1.00E+00	2E-06	0.003317843	0.009425327	0.013014195
Cd300lf	1	2.30E-163	6E-36	0.000545414	0.063791822	0.013011952
Ptgs1	1	1.31E-78	1E-20	0.001064156	0.045060634	0.013010006
Gemin7	1	9.69E-03	8E-05	-0.005155938	0.015715942	0.012992775
Gtf3c4	1	1.00E+00	0.0027	-0.005224525	-0.003454293	0.012988521
Aspm	1	2.69E-02	1E-19	-0.002193365	0.007849806	0.01298789
Ccdc73	0.067278589	5.61E-02	2E-06	0.007992014	0.013040263	0.012976811
Gm10433	1	2.16E-29	9E-30	-0.00140322	0.016020716	0.012968303
Tmem30b	1	6.53E-07	7E-16	0.003791999	0.012062176	0.012935012
Id4	1	1.64E-34	2E-42	-0.000579653	0.013381901	0.012932837
Tamm41	1	1.00E+00	1E-04	-0.002776111	0.002991952	0.01293063
Dlec1	1	4.31E-02	7E-21	-0.00110926	0.006679516	0.012924822
Tnfrsf11a	1	6.44E-119	1E-33	-0.000527769	0.050825316	0.012919583
Fastkd3	1	5.44E-02	5E-12	-0.000872256	0.009413156	0.012916123
Abcb1b	1	1.42E-17	2E-37	0.000767615	0.010693673	0.012913835
1810064F2	1.58127E-21	3.01E-37	0.6078	-0.028329046	0.085130333	0.012910066
Gm47438	1	4.22E-05	6E-30	8.26991E-05	0.006833593	0.012900511
Tmprss4	1	5.64E-17	3E-34	0.000424042	0.010526878	0.01287447
Zmynd19	1	2.61E-05	8E-10	-0.00090429	0.013685783	0.012860429
Gm10603	1	1.81E-15	3E-23	0.000500259	0.01341495	0.012859482
Rhbdl1	1	1.00E+00	2E-07	-0.001562875	0.003578241	0.012857149
Vcam1	1	2.42E-69	1E-23	0.000886026	0.035695924	0.012852698
Osbp2	1	1.00E+00	0.0002	-0.003948586	0.006750024	0.012850256
Magohb	1	4.74E-05	2E-18	-0.000668116	0.010050039	0.012846995
Gm44955	1	4.85E-37	3E-46	0.000940061	0.012327286	0.01284587
Nans	0.092467382	6.07E-04	3E-11	-0.004804093	0.012327043	0.012827411
Efcc1	1	1.00E+00	1E-35	-3.98911E-05	0.003486848	0.012824164
Snrnp35	1	1.00E+00	1E-05	-0.004975764	0.008112	0.012819829
Exosc3	1	1.00E+00	4E-07	0.001640878	0.007814782	0.012800698
Gm16230	1	1.20E-28	6E-27	9.89356E-05	0.01628001	0.012795321
Plekho1	1	8.93E-21	1E-11	-0.001850984	0.02995351	0.012786747
Teddm2	1	9.73E-06	2E-13	0.002713283	0.01294657	0.012783706
Cfap126	1	4.74E-11	1E-11	-0.0029039	0.016651082	0.012778491
Ahnak2	1	4.12E-89	5E-42	-0.000320549	0.033327568	0.012774332
Fam49a	1	7.76E-96	2E-13	0.000438218	0.069277638	0.012759744

Slc15a2	1.1301E-101	1.00E+00	0.2758	0.068666804	0.006772004	0.01273934
Gm28856	1	6.69E-05	3E-25	0.000812953	0.007537307	0.012737484
Clec4d	1	6.33E-166	4E-41	-0.000150972	0.057383992	0.012728713
Gpr141	1	6.38E-141	4E-31	-0.000190035	0.065292242	0.01272827
Twf2	1	7.58E-51	3E-29	-0.000308629	0.02477274	0.012727825
Fam161b	1	2.29E-03	5E-17	0.000229393	0.008695587	0.012723605
Gm43568	0.04104964	1.04E-06	3E-11	-0.004523199	0.013802248	0.012721788
Gm17354	1	3.67E-04	3E-07	-0.004497682	0.014424003	0.012716879
Slamf7	1	8.33E-181	4E-37	-0.000247354	0.069540284	0.012694789
Bak1	1	2.99E-07	1E-08	-0.003551153	0.017491552	0.012693311
Gm16064	1	7.75E-22	2E-43	-0.000642145	0.009412332	0.012691749
Txndc17	1	3.40E-03	1E-08	0.001678073	0.012514572	0.012687645
2810454H0	1	1.00E+00	1E-09	-0.000565343	0.005030122	0.012679436
Clec4a1	1	2.51E-24	8E-30	-0.000501658	0.01715044	0.01267458
Axl	1	2.51E-136	2E-27	0.000754704	0.057902729	0.012673006
Tmem237	1	1.00E+00	1E-21	-0.001071523	0.003066212	0.0126706
1520401A0	1	6.25E-14	6E-43	0.00091068	0.006888703	0.012652121
Meak7	1	1.41E-05	4E-15	-0.000768695	0.010665971	0.012648018
Gm42849	1	3.70E-17	1E-47	0.000700584	0.006778833	0.012638609
Ints5	1	1.00E+00	5E-08	-0.000595336	0.002289684	0.012636805
Nudt12os	1	6.12E-06	2E-08	0.002963014	0.015135486	0.012630865
4930432B1	1	2.27E-07	7E-06	-0.006091027	0.020006034	0.01262639
Ro60	1	1.15E-11	7E-10	0.003983645	0.019548526	0.012608398
Fzd3	1	1.29E-25	2E-39	-0.000476741	0.010998608	0.012604295
AW495222	1.50369E-24	1.00E+00	2E-09	0.017124024	0.004897357	0.012597642
Fam110c	1	4.39E-41	6E-43	9.84282E-05	0.014468469	0.012596397
Cd52	1	2.82E-66	1E-35	-0.000267523	0.024861215	0.012586224
Gm39929	1	4.21E-12	2E-21	-0.002375053	0.012207787	0.012583294
BC055324	1	6.12E-09	3E-20	-0.000144712	0.011305004	0.012582163
Fancg	1	1.00E+00	1E-14	-0.002401244	0.0069471	0.012577323
Slc7a15	0.01256891	2.88E-04	0.0045	0.010999224	0.020423069	0.012559312
Gm39139	1	9.79E-05	1E-18	-0.00116677	0.009012977	0.012557108
Slc35f6	1	8.59E-10	3E-05	-0.0032404	0.026295239	0.012553152
Zfp273	1	1.30E-02	3E-08	0.004368173	0.011527365	0.012551454
Gm12367	1	1.91E-02	0.0002	-0.000496308	0.014922674	0.012546908
Glrx2	1	1.00E+00	0.0005	0.00117266	0.009097374	0.012537225
Pkmyt1	1	9.63E-07	3E-23	0.000897874	0.009886918	0.012525389
Tmem14a	1	2.69E-11	5E-25	-0.000584533	0.010742723	0.012521844
Cgrrf1	0.000257573	1.00E+00	1	-0.01415862	-0.000638749	0.012513549
Mars2	1	4.54E-08	3E-11	-0.000390341	0.014296949	0.012508899
Fyn	1	2.93E-17	2E-05	0.001494659	0.034295447	0.01249082
Zfp960	1	1.00E+00	4E-08	-0.000978739	0.004600698	0.012489126
Ghdc	1	1.00E+00	2E-06	0.000320771	-0.001042049	0.012486515
Gpr146	1.63126E-59	1.00E+00	1	-0.045516998	-0.000215283	0.012483419
Tial1	1.59695E-08	1.00E+00	1	0.035375204	-0.017155927	0.012480945
Tmem126b	1	1.00E+00	6E-05	-0.002886455	0.007185725	0.012470826
Epb41l4a	1	1.00E+00	3E-32	0.000824531	0.002411572	0.012460224

Abca2	1	1.00E+00	0.0085	0.008427632	-0.000706738	0.012438059
Pold1	1	6.51E-02	5E-13	-0.000635944	0.008476747	0.01242768
Trpm2	1	2.29E-91	1E-24	0.00039656	0.045666664	0.012405184
Maoa	0.237000033	1.00E+00	0.0016	-0.006077639	-0.000849913	0.012405124
Frmd3	1	8.11E-04	1E-10	-0.001572199	0.011306746	0.012398974
Gm14455	1	4.06E-04	7E-12	-0.000892293	0.011870237	0.012396347
Cmc4	1	1.00E+00	1E-05	-0.001318608	0.004651506	0.012382983
Gm17089	1	2.61E-06	2E-20	-0.000795999	0.009183637	0.012379063
Ftsj1	1	4.88E-02	1	0.002507197	-0.018306535	0.012378537
Lama5	1	2.01E-10	8E-20	-0.00126591	0.011945666	0.012369293
Olfml3	0.00018773	2.44E-10	8E-14	-0.004920973	0.014512203	0.012364771
Rad54b	0.040020771	1.00E+00	0.0334	-0.00717117	0.001276236	0.012336781
Asb4	1	3.03E-51	8E-44	-9.90402E-05	0.014596104	0.012328268
Jpt1	1	4.39E-02	0.002	-0.004115814	0.016996731	0.012321594
Cdkn2aipnl	1	1.00E+00	0.0001	0.002514673	0.009737436	0.01231428
Gm49066	1	1.00E+00	3E-16	-0.000423084	0.004620082	0.012307349
Zfp414	1	1.00E+00	3E-18	0.001166506	0.006595505	0.012297212
Neil3	1	1.15E-06	2E-29	-0.000425951	0.00836894	0.012293613
Kif23	1	3.42E-52	1E-35	3.67669E-05	0.02141887	0.012288553
H2afz	1	3.35E-03	0.0031	0.001864768	0.018978819	0.012287063
Pycr2	1	6.80E-07	4E-25	0.001097879	0.008806317	0.012286089
Zmynd10	1	7.05E-12	2E-31	0.000818027	0.009123404	0.012282284
Ppip5k2	2.10799E-67	1.60E-11	1	0.095415846	-0.075769509	0.012272594
Ccdc3	1	1.54E-16	9E-27	0.000264638	0.009317438	0.012270855
Prmt1	1	1.00E+00	2E-05	0.003549225	-0.00103638	0.012269677
Cfap298	1	1.00E+00	2E-09	0.002936422	0.008131688	0.012267524
E230001N0	1	7.58E-06	0.0002	0.000876483	-0.015820767	0.0122671
Dennd6b	1	1.00E+00	0.0109	0.004596892	-0.00241421	0.012262033
H2-Aa	1	3.75E-96	2E-26	-0.000286319	0.040872842	0.012256307
Tmem238	1	1.30E-01	9E-08	0.001040486	0.010732976	0.012249975
Trmt112	1	5.84E-03	1E-07	-0.000681277	0.013730024	0.01223999
Sh3glb1	1	3.58E-02	1	-0.000738437	0.042819508	0.012233253
Racgap1	1	2.55E-12	6E-36	-0.000122251	0.007453505	0.012225179
Gm47795	1	2.48E-59	5E-44	-0.000276904	0.016685124	0.012223654
A930024E0	1	7.60E-16	1E-25	0.000588545	0.011938365	0.012219751
Tcte2	0.000798877	8.83E-07	0.1886	-0.011035384	0.027672803	0.012216784
Ntpcr	1	8.73E-02	4E-14	-0.002373404	0.008511499	0.012200508
Cenpn	1	7.29E-07	4E-21	0.00101807	0.009212881	0.012197121
Ythdf3	1	2.63E-16	1	-0.015475497	0.095138274	0.012178236
Sdhaf1	1	3.38E-02	3E-11	0.002895587	0.009995379	0.012176402
Gm28068	0.011772898	1.00E+00	2E-05	-0.007754705	0.005431562	0.012175878
Ap3m2	0.010470605	3.28E-14	4E-21	-0.003026268	0.013482829	0.012170736
Calhm2	1	8.13E-41	3E-16	0.000735731	0.030870439	0.012150066
Dctpp1	1	1.78E-09	3E-13	-0.001604508	0.014319457	0.012136998
Rsl24d1	1	1.00E+00	7E-07	-0.000916026	0.009737687	0.012128929
Clspn	1	1.00E+00	1E-19	-0.001373718	0.00355759	0.012126029
Ptger3	1	1.50E-47	7E-41	4.76378E-05	0.016577557	0.012114729

Mterf1a	1	2.61E-15	5E-12	0.002462659	0.019053129	0.012110812
Zfp446	1	2.06E-04	4E-07	-0.000635419	0.014300547	0.012105981
Pdcl3	1	1.00E+00	0.0036	-0.002785458	0.002922906	0.012105039
Gm36037	1	1.48E-30	6E-31	7.9233E-05	0.014811009	0.012096173
Coro1b	1	5.89E-02	3E-09	-0.003480725	0.010955675	0.012086176
Zfp263	1	2.48E-02	1	0.010778838	-0.018463553	0.012082272
Gm6980	7.69324E-14	5.62E-01	6E-14	-0.005015302	0.008148263	0.012077801
Aktip	1	5.90E-03	4E-05	-0.005229015	0.014844263	0.012068806
Klc2	1	1.00E+00	0.0312	-0.003507027	0.005405598	0.012064847
Wdr19	0.120854317	1.00E+00	0.0023	-0.007497378	-0.002189488	0.012044191
Sap25	0.120945273	3.85E-04	9E-07	0.006753537	0.017109435	0.012042411
Tm2d2	1	2.83E-05	2E-09	0.001012054	0.013790359	0.012042267
Gm19541	1	1.73E-45	3E-51	0	0.010830028	0.012034739
D630033O1	1	2.79E-07	1E-08	-0.002113973	0.01564598	0.012033391
Sh3glb2	1	1.00E+00	0.0473	0.001425321	-0.003191803	0.012023899
Gm28913	1	1.37E-06	7E-35	0.000313865	0.006051374	0.012016269
Xylt1	1	1.58E-164	8E-09	0.002333328	0.11316474	0.012014843
Rhob	0.001690768	1.59E-30	4E-14	0.005605947	0.025554588	0.012010799
Hecw2	0.363662874	8.47E-06	0.0011	0.005587754	0.025060344	0.012004744
D17H6S53E	1	1.00E+00	8E-15	-0.000538167	0.00485621	0.011991336
Grik3	1	1.51E-27	2E-18	-0.000117725	0.020335896	0.011980149
Smarcd3	1	1.74E-10	3E-09	-0.004103412	0.016767268	0.011966879
Gm43625	1	1.16E-05	1E-18	-0.00148393	0.009346427	0.011965841
Arsb	2.14449E-21	1.00E+00	1	-0.050062288	-0.020566942	0.011963186
1600012H0	1	7.93E-02	2E-05	0.001079254	0.012953805	0.011962182
Jmjd8	1	1.00E+00	8E-07	0.000292961	0.002684389	0.011958576
Usp49	0.00144759	2.22E-05	1	-0.016346936	-0.029817526	0.011953725
Adgrl4	1	1.49E-03	2E-05	0.004978205	0.019603142	0.011944226
Glis1	1	7.67E-64	2E-25	-0.000860044	0.027948743	0.011942276
0610040B1	1	2.55E-03	7E-05	-0.005519941	0.015281835	0.011930895
2010320O0	1	1.59E-44	9E-30	0.000382839	0.019121608	0.011916221
Colq	0.003915499	9.80E-13	1E-25	-0.002392362	0.010416158	0.011893001
Emc1	1	1.26E-03	8E-19	-0.001359897	0.007824265	0.011892848
Pcdh15	1	1.00E+00	0.0094	0.003148708	0.008771162	0.011890238
Poc1a	1	1.00E+00	0.0146	-0.005485902	-0.000731663	0.01188951
Ptpa	7.66914E-09	1.00E+00	1	0.032531571	0.017806236	0.011883526
Ywhah	0.154588006	3.97E-05	0.0112	-0.007683817	0.022387485	0.011878042
Pole2	1	3.72E-15	5E-20	-0.001399022	0.013658607	0.011877297
Fbxl12os	1	1.14E-16	6E-28	-0.000590873	0.011398785	0.011872888
Plek2	1	1.56E-31	2E-43	-7.13902E-05	0.01083726	0.01186737
Gm39214	1	1.38E-14	6E-07	0.0050769	0.023381577	0.01186581
Tagln2	1	3.78E-66	3E-30	-0.000442584	0.026636284	0.011864244
Eps8l1	4.74E-12	2.59E-26	3E-12	0.010124307	0.024155708	0.011859695
Zscan18	1	8.31E-05	3E-10	-0.00287606	0.011787958	0.011829202
Cdk1	1	6.08E-13	2E-31	-0.000497341	0.008668362	0.011819008
Uqcc3	1	5.55E-05	1E-09	0.00337738	0.012867647	0.01181166
Ulbp1	1	4.94E-89	5E-26	-4.61823E-05	0.040146474	0.01178732

Vbp1	1	1.00E+00	5E-07	0.000357944	0.008057835	0.011786482
Mien1	1	1.00E+00	7E-11	0.004816487	0.007903262	0.0117847
Dhrs13	1	5.06E-17	1E-14	0.001382869	0.017278296	0.011783739
Gm26532	1	4.25E-62	4E-42	0.000149361	0.019563285	0.011775432
Snrnp25	1	1.56E-08	7E-33	-0.000782172	0.007217343	0.011772459
6530401F1	1	1.40E-02	4E-05	-0.002718745	0.013013818	0.011768669
Klhl31	1	2.76E-34	2E-33	-0.000977221	0.014320584	0.011767454
Mppe1	1	1.00E+00	0.0004	0.000355529	0.000467962	0.011756067
Ptprn2	1	6.69E-05	3E-20	0.000129268	0.008596704	0.01175364
Spata22	0.077228763	1.71E-04	3E-05	-0.006569783	0.016407105	0.011746477
Mlst8	1	4.08E-01	4E-10	0.001261524	0.008041854	0.011744596
Coro2a	1	2.00E-114	4E-21	-9.87259E-05	0.052208103	0.01174336
Gm15956	1	3.25E-02	8E-20	-0.000446043	0.00655726	0.011741041
Hypm	1	2.04E-31	2E-44	0.000752395	0.010405435	0.011732253
Mif	1	4.59E-09	8E-05	-0.003148664	0.025240704	0.011716622
Aimp2	1	2.51E-04	4E-10	-9.98826E-05	0.012268395	0.011710295
E230029C0	1	4.75E-54	5E-12	-0.001003032	0.044224779	0.01168944
Gm36287	3.49974E-17	1.00E+00	1	-0.019499361	0.006210256	0.011683429
Clhc1	1	2.69E-40	6E-28	-0.0005187	0.017771159	0.011678869
Csf2rb	1	2.93E-119	5E-08	-0.000264679	0.084447071	0.011678013
Zfp526	1	1.00E+00	5E-10	-0.001700034	0.007270895	0.011676613
Abca14	1	1.00E+00	0.005	-0.002795499	-0.006539794	0.011673584
Zfp759	1	1.00E+00	3E-05	0.000775299	-0.003088949	0.011664181
Gpr18	1	1.00E+00	2E-07	0.001499174	0.004912602	0.01166345
St18	1	1.52E-158	2E-19	-0.000376797	0.093369746	0.011656442
Dnal1	1	1.00E+00	2E-05	-0.000863556	0.004010239	0.011647819
Eif4e3	1	7.23E-39	9E-33	0.00061173	0.017002998	0.01164492
Alpk1	1	2.24E-50	5E-08	0.002398636	0.047191403	0.01164038
Gltp	1	4.33E-83	3E-43	-0.000130394	0.024365331	0.011636899
Fancb	1	2.24E-16	2E-21	-0.000417277	0.013286199	0.011626101
Katnb1	1	1.81E-08	1E-21	0.000439378	0.010492197	0.011620767
Shkbp1	1	5.77E-05	5E-07	-0.001799769	0.017475369	0.011619848
mt-Nd3	1	1.00E+00	1E-12	-0.000529928	0.007233838	0.011618469
Map3k6	1	3.72E-29	2E-30	-0.000308701	0.014258802	0.011612674
Gm49692	1	3.03E-08	5E-28	0.000454492	0.007280411	0.011607855
Satb2	0.006903629	1.00E-02	1	-0.01441808	0.028009321	0.011603374
Gm37406	1	3.16E-42	2E-31	-0.001481143	0.017081842	0.011602714
Bloc1s6os	1	2.06E-27	4E-31	-4.08167E-05	0.013191465	0.011600606
Laptm5	1	6.94E-130	7E-23	-0.001338805	0.055640441	0.01159857
Gins3	1	1.04E-04	2E-16	-0.000133816	0.009618315	0.011598451
Brf2	1	9.42E-02	5E-10	-9.27166E-05	0.00883522	0.011584546
Bbs4	0.07299113	1.00E+00	0.0008	-0.007307117	0.000651962	0.01158353
Dreh	1	3.75E-12	7E-28	0.00077858	0.009283005	0.011580898
Utp23	1	1.00E+00	3E-08	-0.000778169	0.007750105	0.011580507
Gatd3a	0.111996046	1.00E+00	0.0498	-0.008721237	-0.005783589	0.011576871
Zfp786	1	1.21E-11	2E-26	-0.000168264	0.009007713	0.011557731
Cmtr2	1	1.14E-05	3E-12	-0.001554392	0.011019501	0.011556286

Eef1akmt3	1	8.08E-34	1E-39	0.001142965	0.011678366	0.011555576
Pop4	1	1.45E-03	5E-08	-0.000877737	0.012327488	0.011552805
5033403F0	1	8.81E-06	3E-14	-0.001453404	0.010452262	0.011547005
Tm6sf1	1	1.10E-78	6E-28	0.000218719	0.032988981	0.011538989
Rab29	1	5.58E-01	4E-08	0.00416998	0.008831094	0.01152195
Gm49768	1	3.64E-18	2E-29	0.000808935	0.011007184	0.011520221
Gm3435	1	1.67E-07	5E-08	0.000290953	0.015565011	0.011512381
Gm4013	1	1.60E-16	7E-17	0.000628236	0.015355746	0.011508905
A630052C1	1	2.19E-04	7E-14	-0.001277271	0.009757545	0.01150882
Commd2	1	1.00E+00	9E-05	-0.002440501	0.005268324	0.011508086
C630043F0	1	1.00E+00	6E-07	-0.000299536	-0.006426912	0.011505294
P3h1	0.113023319	4.47E-10	1	0.01250314	-0.029963268	0.011500804
Slc43a1	8.27744E-14	1.00E+00	3E-09	-0.007748404	0.002563965	0.011494478
4930412O1	1	1.21E-26	4E-50	0	0.0063143	0.011491685
Lig4	1	1.00E+00	0.0003	-0.002561466	0.004162492	0.011484314
Gm20621	1	6.81E-01	4E-38	0.000267368	0.002611274	0.011484223
Gatd1	1	1.00E+00	0.0147	-0.002100141	-0.000399143	0.011477868
Wdr5b	1	1.97E-05	5E-19	-0.000747676	0.00910467	0.011473698
Gm12905	1	9.98E-09	1	-0.00499749	0.038492594	0.011471414
Mrpl34	1	9.43E-03	7E-15	0.001836686	0.008584107	0.011464766
Akt3	0.128368511	1.68E-70	4E-07	0.004923317	0.061826495	0.011464671
Pde12	1	7.34E-05	5E-11	-0.001866581	0.011114167	0.011460064
Gm14102	0.037695591	2.10E-14	8E-46	0.001537803	0.005083568	0.011459302
2810410L2	0.000113938	1.00E+00	4E-07	0.008601304	0.009323568	0.01144998
Gm26533	1	1.00E+00	2E-06	-0.002092619	0.004575714	0.011440856
Lipo3	1	6.38E-15	1E-13	0.000649128	0.016727217	0.011432187
Nbeal2	1	1.00E+00	0.012	-0.000443244	0.01257836	0.011431996
Hmga1	1	7.73E-04	6E-14	-0.001372187	0.009286518	0.011431396
Iqank1	1	1.00E+00	0.0179	-0.003359302	-0.000628889	0.011424407
Naa38	1	1.43E-08	6E-25	0.000725205	0.008826782	0.011423399
Clmp	1	1.61E-27	6E-16	-0.002366082	0.021583504	0.011423225
1110002J0	1	1.00E+00	1E-17	-0.002300837	0.00236091	0.011411099
Srd5a3	1	1.00E+00	2E-06	-0.000731597	0.008573146	0.011409016
Hspa2	1	1.00E+00	4E-34	4.23708E-05	0.002625564	0.01139883
Ptrhd1	1	2.74E-03	1E-07	-0.001197807	0.012191417	0.011387076
Dnajc9	1	1.00E+00	5E-12	-0.001933776	0.006838993	0.011382518
Gm6787	1	4.42E-03	3E-15	-0.002480142	0.007535332	0.011379902
Zfp202	1	9.81E-04	2E-12	-0.002952371	0.009181832	0.011378686
Tyrobp	1	8.22E-90	2E-31	-0.000492904	0.032523691	0.011378565
Apool	0.007792298	1.00E+00	1	-0.013849652	0.001971576	0.011377747
Nek10	1	3.67E-03	7E-22	-0.001586424	0.005641283	0.011373428
4930544F0	0.019924083	9.23E-07	0.0001	-0.006415181	0.017043823	0.011363736
Mrps11	1	1.00E+00	2E-06	-0.001550658	0.003315596	0.011353776
Il1rn	1	1.33E-91	2E-32	-0.000208856	0.031991417	0.011339549
2610528J1	1	5.59E-23	2E-32	0.000541687	0.011112874	0.01133828
Gm13830	1	7.54E-14	2E-25	-0.000144813	0.010646346	0.011330813
Castor2	7.53568E-06	1.00E+00	1	-0.015670899	-0.005734356	0.011330273

Czib	1	1.00E+00	0.0029	0.00703673	0.009175273	0.011308054
Lamtor2	1	4.14E-01	4E-05	0.000857713	0.011438557	0.011306031
Fignl1	1	1.51E-22	1E-38	-0.000604646	0.008533723	0.011295347
Sema6a	1	1.00E+00	0.0015	0.003054646	0.016536024	0.011293696
Akr1b3	1	2.95E-35	1E-25	-0.000390038	0.018417163	0.011280851
Eef2kmt	1	1.00E+00	0.0127	-0.003427266	0.002873302	0.01127448
Cdca3	1	1.00E+00	2E-18	0.000451368	0.004714687	0.01127257
Irf8	1	3.33E-84	3E-10	-0.002839827	0.054820186	0.011266557
Plekhn1	1	1.00E+00	0.0001	0.001220656	0.01091473	0.011265008
Tifa	1	2.63E-17	0.0194	0.000186113	0.033853542	0.011258499
Med18	1	3.18E-10	2E-17	0.001025852	0.011620045	0.011250331
Mrpl55	1	1.00E+00	5E-07	-0.000579916	0.006399834	0.011249639
Adgrf5	1	9.10E-01	0.0061	0.001494858	0.016302629	0.011242124
Mdfi	1	2.84E-100	3E-41	-0.000272442	0.025525629	0.011238179
Exo1	1	2.18E-21	8E-30	2.27918E-05	0.011709024	0.011237962
Med12	4.66019E-11	1.57E-01	1	0.023621879	-0.016134066	0.011220714
Zfand2b	0.015920653	1.00E+00	0.0097	0.009834784	-0.006121537	0.011218795
Ccdc159	1	1.00E+00	0.0087	0.002310863	-0.004770988	0.011214968
Med19	1	1.00E+00	2E-05	0.005561337	0.003808882	0.011189786
Cybb	1	1.90E-134	7E-26	-0.000640316	0.060441899	0.011182703
Gm11802	1	4.66E-02	2E-26	-0.000768857	0.004587355	0.011175253
Cd320	1	1.00E+00	7E-08	0.000895935	0.004108081	0.011173558
Shprh	0.000123327	2.88E-02	1	-0.027656207	-0.032738031	0.011169708
Anxa3	1	1.00E-117	6E-18	-0.000484352	0.056791434	0.011159318
Slc7a5	1	1.52E-13	1E-18	-0.000191329	0.012546248	0.011155043
Nkain2	4.97976E-59	1.00E+00	0.0103	0.040083911	0.013058066	0.011153624
BC030867	1	8.59E-30	9E-38	-0.000442188	0.01016389	0.011145583
Apobec3	1	1.07E-62	2E-22	0.000326685	0.033501981	0.01114465
Commd9	1	1.00E+00	7E-05	0.004033723	0.003839337	0.011144082
H2-Eb1	1	3.11E-104	3E-30	-9.62125E-05	0.037831373	0.01114121
Tnfaip3	1	3.77E-53	4E-11	0.002212725	0.036068654	0.01113868
Pip5k1b	1	8.09E-06	5E-06	-0.000246936	0.018829174	0.011111452
Efr3a	1	2.45E-05	1	0.002116962	-0.038976826	0.011111224
Jrk	1	2.12E-07	2E-25	0.001008255	0.00786286	0.011106144
Vat1	1	2.06E-39	2E-20	0.000477851	0.022896339	0.011100647
Gm12185	1	7.09E-104	2E-07	-0.001662871	0.067346974	0.011098923
Cd3eap	1	1.00E+00	7E-07	0.001494229	0.00766098	0.011098172
Nkiras2	1	1.69E-05	4E-10	-0.00047714	0.012109242	0.011097885
Zbtb25	0.00014607	1.55E-04	0.4195	-0.011074925	0.022982815	0.011084468
Sox5os2	2.58509E-23	1.00E+00	0.0008	-0.015221457	0.001135105	0.011083881
Wdr24	1	1.00E+00	0.001	0.003337071	0.000888994	0.011078988
Slfn2	1	1.08E-98	7E-24	-0.000915488	0.041365267	0.011078357
A430073D2	0.006091518	1.00E+00	1E-05	-0.006452382	-0.000870664	0.0110769
Clock	2.97622E-18	1.00E+00	1	0.052218427	-0.019024594	0.011070715
Gm26779	1	2.75E-01	2E-12	-0.001594525	0.007202197	0.011065137
Gm14308	1	1.00E+00	0.0162	-0.000759927	0.008501956	0.011063623
Adcy7	1	1.22E-99	4E-08	-3.9117E-05	0.062308007	0.011057528

Gm4779	1	1.00E+00	2E-43	0	0	0.011055762
Pfdn2	1	1.01E-03	1	0.002061469	-0.032769437	0.011055602
Dock11	1	2.73E-85	0.0011	-0.002227689	0.071133392	0.011051348
Gm16897	1	2.93E-16	1E-15	-0.001260642	0.014988501	0.011049665
Gimap9	0.752186005	1.00E+00	0.0018	0.007529735	0.000347193	0.01104298
0610010K1	1	5.59E-04	4E-09	0.003442119	0.011921715	0.011040501
Mad2l1bp	1	9.30E-03	9E-09	-0.002410418	0.010429578	0.011034498
Rrp7a	1	1.00E+00	0.0001	0.000952734	0.008473118	0.011031231
Tlr7	1	2.67E-123	2E-31	-0.000177575	0.046059528	0.011025779
Nup37	0.001378244	1.00E+00	0.1837	-0.009971214	0.011502531	0.011020672
0610009B2	1	1.00E+00	5E-06	0.000251687	0.00550466	0.011007011
Rapgefl1	1	4.14E-07	2E-19	-0.001772491	0.008644409	0.011006876
Emc6	1	1.00E+00	0.0341	0.004499786	0.007250304	0.011000231
Sdcbp2	1	8.60E-19	3E-39	0.000353023	0.007258007	0.010999984
Tmem236	1	5.76E-62	1E-30	-0.000175602	0.021845147	0.010998981
Nol4l	1	1.37E-05	2E-05	-0.001964504	0.016443751	0.010989333
Cdc6	1	1.80E-16	4E-28	-0.000200737	0.010010974	0.010984329
Abhd13	1	1.00E+00	0.0271	-0.004068115	0.007119321	0.010982805
Rhoc	1	1.46E-22	5E-20	-0.000971279	0.015338593	0.010977592
2810414N0	1	3.64E-03	1E-19	-0.000276323	0.006625744	0.010977401
Gm26724	1	9.52E-05	3E-06	0.004683547	0.013740365	0.010961844
Gm44427	1	3.68E-18	2E-47	0	0.004719415	0.010956535
Tmem102	1	1.00E+00	2E-11	-0.000433521	-0.000268497	0.010955259
Klhl6	1	4.54E-35	2E-22	9.24757E-05	0.021233653	0.010952501
Gm13912	1	6.05E-81	1E-29	0.000738277	0.026288197	0.010947417
Ms4a7	1	5.45E-116	9E-41	0.0001242	0.03683353	0.010945575
Adat2	1	9.97E-13	1E-24	0.000709895	0.009561935	0.010938506
Socs3	1	3.06E-12	1E-07	0.003417464	0.01917965	0.010935635
Srrd	1	1.00E+00	0.0004	-0.002626437	0.009171684	0.010921962
Ggta1	1	7.13E-142	9E-25	-0.000397255	0.063347202	0.010916677
H2-Ab1	1	3.95E-104	6E-24	-0.00019138	0.041554756	0.010913102
Abcb9	0.539954058	1.00E+00	0.0006	-0.005912907	0.007271213	0.010911539
4930595D1	1	8.81E-20	2E-05	0.002958005	0.026669679	0.01090828
C1qc	1	6.57E-75	4E-25	-4.25732E-05	0.031580931	0.010898983
Cyba	1	3.24E-124	2E-20	-0.000744527	0.054398807	0.010898262
1700029J0	1	1.76E-05	3E-07	-0.000716148	0.013700776	0.010897369
Smtn	1	1.02E-02	1E-17	-0.00024041	0.006864005	0.010876561
Cdc26	1	2.50E-02	1	0.014390161	-0.023860948	0.010870287
Mrpl32	1	1.00E+00	0.041	0.002161787	-0.000624598	0.010864803
Rapgef5	0.00057645	8.57E-37	0.0281	0.007903524	0.054327035	0.010853615
B230206H0	1	1.00E+00	2E-05	-0.000924553	-0.00070144	0.010852365
Lef1	1	9.81E-10	6E-22	-0.000220317	0.008658409	0.010847642
Orc1	1	2.35E-11	1E-26	-0.00057044	0.008728573	0.010830816
Gm7480	1	5.81E-70	8E-37	0.000210974	0.0212935	0.010809137
Mpz	1	1.37E-38	1E-38	0.00056746	0.012623094	0.01080479
Gm41392.1	1	2.07E-30	5E-45	0	0.007248198	0.010797595
Cd48	1	7.72E-124	2E-28	-0.000357928	0.046830487	0.010796748

Gm14091	1	7.50E-29	8E-39	-0.000495803	0.009593264	0.01079562
Agrp	1	4.86E-04	0.0007	-0.00104529	0.015349929	0.010787157
Igf2bp2	1	3.29E-16	0.0004	-0.001402436	0.038982494	0.010782287
Trpv2	1	7.45E-81	1E-32	0.000116276	0.027714132	0.010777548
Slc25a4	1	3.37E-15	2E-36	0.00066662	0.006416231	0.010773859
Clec4g	1	4.60E-06	8E-08	0.000516541	0.016404319	0.010743552
Nfam1	1	6.07E-122	4E-24	-0.00036239	0.047607994	0.010730582
6330418K0	1	4.17E-03	8E-13	-0.000667142	0.008438079	0.010729085
1700056N1	1	1.00E+00	3E-06	0.000881746	0.008068533	0.010719769
Ulk4	1	1.26E-13	2E-05	-0.003016924	0.022616534	0.01071442
2310010J1	1	4.66E-05	5E-09	-0.000392267	0.01170208	0.010702665
Tuba1b	1	3.62E-11	1E-12	-0.001741062	0.014006493	0.010702053
AU022793	1	4.35E-122	3E-32	0	0.039805578	0.010694026
Gm16146	1	3.41E-15	2E-19	0.002174408	0.011930218	0.010690275
Trappc5	1	1.00E+00	6E-05	-0.002443836	0.000852405	0.010690147
Vmn2r1	1	1.48E-06	2E-24	-8.31767E-05	0.007015403	0.01068619
Map3k8	1	9.94E-17	4E-05	-0.001454026	0.030489821	0.010684891
Cyhr1	0.103311429	4.40E-03	1	0.014471858	-0.020944417	0.010673918
Acap3	1	1.00E+00	1E-07	0.000428298	0.002926283	0.010648443
Gm17029	1	1.68E-04	0.0001	0.003185702	0.01420261	0.010648108
Ccdc24	1	3.30E-01	3E-09	-0.002535451	0.008137888	0.010641337
Kit	1	3.89E-16	4E-11	0.002316489	0.021097722	0.010633256
5330417C2	1	1.03E-05	9E-14	-0.000224184	0.010662242	0.010629089
Hk2	1	6.45E-40	4E-32	-0.000602011	0.016616717	0.010626334
Col16a1	1	5.41E-17	8E-39	0.000334249	0.006798485	0.010621528
Fcgr3	1	2.02E-66	1E-25	-0.000941526	0.025856557	0.010613305
Pde1b	1	3.58E-99	5E-29	-0.000176577	0.033609407	0.010584967
1700027J0	1	2.28E-05	1E-10	0.000759448	0.010345139	0.010584263
Fermt3	1	1.14E-26	0.0234	0.000972915	0.043706504	0.010577503
Gtpbp6	0.001434278	1.00E+00	1	0.012801528	0.013685673	0.01057371
Zfp93	1	1.00E+00	1E-08	0.001602252	0.005373389	0.010573153
1700015O1	1	1.94E-11	1E-28	0.000717867	0.007690362	0.010571859
Rbp1	5.19511E-11	1.00E+00	2E-05	-0.008822403	-0.0045871	0.010571045
Zfp992	0.000802927	6.42E-02	0.0004	-0.008268917	0.018331547	0.01056403
Gemin6	1	7.02E-07	2E-16	4.43074E-06	0.009498216	0.010562662
Adgrb1	1	2.26E-47	8E-41	0	0.012137019	0.010560148
Myo1g	1	6.06E-72	7E-24	-0.000161554	0.02901001	0.010558617
Coa4	1	1.65E-11	6E-19	0.00105694	0.010759112	0.010552908
Med10	1	1.00E+00	4E-05	0.004202888	0.008324686	0.01054935
Ces1a	1	2.04E-26	5E-19	-0.001885542	0.015577705	0.010547115
Wnk4	1	1.00E+00	9E-07	-0.000714139	0.003547547	0.010541672
Zfp687	1	1.00E+00	0.0269	-0.000981826	0.000537062	0.010522844
Col11a2	1	2.14E-07	6E-15	0.00268629	0.010455907	0.01050727
Ebf1	1	4.99E-18	7E-05	-0.002436896	0.033248447	0.010500536
1700096K1	1	4.08E-08	3E-13	0.000204847	0.011542297	0.010500122
Orm3	1	8.40E-12	1E-32	0.000320153	0.006781937	0.010490418
AI413582	1	1.18E-20	3E-35	0.000441576	0.008566219	0.010484622

Prr5	1	1.00E+00	2E-06	-0.003009459	0.006874742	0.010479616
Gm44040	1	9.07E-09	4E-09	0.001351456	0.014844709	0.010478984
D330023K1	0.01092717	1.00E+00	1	-0.010186243	0.010849859	0.010475114
Dbndd2	1	4.82E-17	5E-14	-0.000157028	0.015388805	0.010473886
Slc15a3	1	1.39E-200	4E-26	-0.000288365	0.076766794	0.010461584
Large2	1	5.59E-01	4E-09	-0.000540606	0.007830261	0.010451621
Junb	1	1.10E-17	1	-0.006558858	0.03906399	0.010437098
Sccpdh	1	3.85E-01	2E-19	-0.000945338	0.005183631	0.010418448
Mtg1	1	1.00E+00	0.0065	-0.000932739	-0.000112151	0.010413876
Slc25a45	0.002141762	1.00E+00	1	-0.010536953	0.008881361	0.010412964
Angptl1	1	1.81E-05	7E-09	-0.00113633	0.011193694	0.010406709
Gm16299	1	6.10E-04	4E-15	-0.001074828	0.007793672	0.010397921
Pdcd5	1	3.63E-01	0.0017	0.003403546	0.011861213	0.010384628
Me2	1	3.98E-27	2E-21	0.000430283	0.017491271	0.010382917
Ccdc28b	1	1.00E+00	4E-05	0.000964297	0.004599927	0.010382513
Armc6	1	2.31E-01	0.0001	0.003740387	0.010524169	0.010380505
mt-Nd4l	1	2.63E-01	4E-18	0.000173225	0.005580281	0.010368658
Gopc	1.96463E-06	1.00E+00	1	-0.024815584	-0.013066606	0.010358082
Prkcg	1	4.66E-04	2E-09	-0.001662873	0.010966748	0.010355538
Ppil1	1	1.00E+00	5E-06	0.003546274	0.007909651	0.010352194
Bola1	0.087808663	8.50E-07	1E-11	0.00463671	0.011161121	0.010340657
BC049762	1	1.00E+00	5E-05	-0.003876454	0.005798794	0.010334367
Arf6	1	1.07E-05	4E-09	-0.000830235	0.012244835	0.010331014
1810012K1	1	9.70E-30	1E-31	0.000782944	0.012201079	0.010330638
Gbp8	1	3.86E-26	2E-07	0.000151141	0.029160068	0.010329797
Col15a1	1	3.86E-14	0.0017	-0.00481436	0.025345759	0.010327122
Slc22a1	2.63509E-05	4.05E-03	1	-0.036036637	-0.044946951	0.010320155
Hist1h4i	0.017762399	5.41E-08	2E-10	0.005225205	0.012786456	0.010315788
Clec1b	1	2.91E-78	7E-24	0.000935793	0.034831181	0.01031326
Aco1	0.008140425	3.17E-13	1	0.025126554	-0.06072815	0.010310906
Kif20a	1	5.12E-06	6E-23	0.000161209	0.006702728	0.010307217
Gm32647	1	2.67E-03	2E-09	-0.001608691	0.009571362	0.010298641
Gm9920	1	3.42E-05	3E-12	7.26717E-05	0.009387392	0.010297204
Abcc2	2.49972E-81	6.43E-181	1	0.108397707	-0.338342296	0.010292687
Syngr2	1	1.00E+00	0.0013	0.002260714	0.005149738	0.010281105
6330415G1	1	4.40E-01	0.011	-0.003018735	0.012005483	0.010278481
Snrpn	1	1.43E-03	2E-21	-0.000241627	0.006070993	0.010274171
Gm14547	1	2.25E-04	3E-14	0.000672504	0.008161954	0.010254556
Itpkb	1	1.72E-126	1E-06	0.000877029	0.091316237	0.010251831
Gm26590	1	1.15E-04	0.0017	-0.003271912	0.015575883	0.010248
Sox5os3	1	2.68E-16	2E-16	0.000463612	0.013345053	0.010247047
Ndc80	1	3.11E-03	3E-29	-4.62299E-05	0.005017306	0.010245154
Myorg	1	8.12E-03	2E-07	-0.00397492	0.010206451	0.010243415
Gm41760	1	9.57E-12	2E-30	-0.000738743	0.006917952	0.010239496
Rpp25l	1	1.00E+00	4E-05	0.002309477	0.00134315	0.01023875
4930505A0	1	1.04E-26	6E-33	-0.000498461	0.010140991	0.010238568
Cotl1	1	2.97E-93	9E-10	-0.001872058	0.056998659	0.010233703

Bard1	1	2.26E-04	1E-17	-0.000395305	0.007693534	0.010206043
Gm15558	1	4.38E-12	1E-12	-0.000126648	0.013102241	0.010205551
Casp4	1	7.77E-100	6E-23	0.001031339	0.043057309	0.010194978
Atp6v0d2	1	2.54E-75	0.0003	-0.002537937	0.077052164	0.010191602
Tfap2d	1	7.75E-11	7E-27	-0.000737696	0.007395223	0.01018245
Mecom	1	1.54E-07	4E-13	8.63075E-05	0.010495634	0.010176298
Gm42917	1	5.72E-06	0.0047	-0.001619673	0.018685912	0.010171344
Uhrf1	1	4.88E-10	5E-27	-0.000740596	0.006258885	0.010167257
Gm45406	1	1.36E-70	5E-40	1.89419E-05	0.01774678	0.010159185
Eef1e1	1	1.00E+00	0.0116	-0.005411665	0.01052647	0.010157286
Knstrn	1	3.85E-09	3E-21	-0.000730552	0.008629585	0.010155952
Lrrc69	0.033812808	9.95E-04	0.0074	-0.007589925	0.015365795	0.010144519
Drc3	1	6.19E-07	4E-12	-0.000840113	0.010514816	0.010139532
Zfp105	1	6.25E-27	2E-37	1.44323E-05	0.008836026	0.010138079
Gm32687	1	1.00E+00	5E-08	-0.001054281	0.007280738	0.010137058
Cd300lb	1	1.34E-224	8E-31	5.92193E-05	0.081928638	0.010134885
Tstd1	1	1.00E+00	1E-09	0.000412743	0.004433381	0.010118801
Endog	1	1.00E+00	2E-06	0.004152961	0.001874431	0.010116842
Gm49691	0.575034968	1.59E-09	2E-28	0.002080248	0.00636786	0.010107225
Gm14393	1	2.10E-26	5E-28	3.96654E-05	0.011623289	0.010102919
Unc79	0.012129751	3.42E-14	1E-12	0.00462553	0.013713896	0.010098236
Fgd2	1	1.90E-63	1E-20	0.000509721	0.031375746	0.010081796
Sh3d21	6.15974E-06	1.00E+00	3E-05	-0.007719619	0.007676494	0.01007691
Slc28a1	1	4.12E-17	1E-16	-0.002522197	0.013423291	0.010074778
Tmem129	1	1.00E+00	4E-09	0.000281056	0.005167899	0.010068739
1700086P0	1	8.63E-06	1E-19	-0.000951062	0.007585678	0.010051463
Nwd2	1	1.11E-06	5E-15	-0.001264514	0.009137309	0.010043799
A630001G2	1	4.35E-110	6E-22	0.00064282	0.043266197	0.010034169
Gm20939	1	1.00E+00	5E-05	-0.001646571	-0.004926336	0.01002648
Pld6	1	2.81E-31	1E-26	0.000221018	0.013716437	0.010022533
Gm11423	1	1.45E-05	3E-10	0.001846489	0.010721256	0.010017719
Tada2b	1	1.00E+00	0.0019	0.001713365	0.002536416	0.010012175
4930549C1	1	1.25E-04	4E-14	0.003350348	0.008036663	0.009999671
3110009E1	1	8.24E-07	0.0021	-0.003449368	0.01764221	0.009990348
Nkrf	0.695011988	1.00E+00	5E-05	-0.00503551	0.004730677	0.009983672
Banf1	1	4.65E-01	3E-10	0.003190791	0.007268066	0.009977295
Elk3	1	2.51E-17	2E-05	-0.00143362	0.026343087	0.009962473
Rassf4	2.86992E-12	3.25E-08	0.0006	0.012005673	0.019966381	0.009956254
Gm40437	1	1.02E-14	7E-22	-0.001042345	0.010097067	0.009949387
Mmp13	1	1.87E-107	3E-37	-4.73494E-05	0.037826292	0.009944384
Stoml1	1	1.23E-01	0.0002	-0.003198987	0.011993904	0.009929658
Mpv17l2	1	1.00E+00	6E-08	-0.000911144	0.007806192	0.00992601
Cd163	1	1.41E-12	4E-14	0.000390462	0.014816908	0.00992078
Cd53	1	3.37E-108	1E-19	-0.000129106	0.043766435	0.00991196
Gm31717	1	2.08E-14	8E-17	-0.001360922	0.011753097	0.00991099
Kirrel3	1	1.00E+00	2E-13	-0.000777756	0.00308357	0.009908467
Tmem50b	1	1.00E+00	0.0039	0.001327193	0.004172301	0.009908313

Tnfsf13os	1	3.76E-11	2E-10	-0.002015162	0.013815195	0.009907725
Gm17746	1	7.21E-32	1E-31	-0.000857604	0.011664958	0.009906145
Tspan7	1	1.00E+00	0.0296	-0.000190739	0.011575361	0.009900775
4930417H(	1	1.00E+00	2E-13	-0.001474033	0.004093575	0.009893832
Efcab5	1	3.07E-15	6E-17	-0.000950942	0.01141519	0.009881437
Aqp1	1	5.86E-07	2E-08	-0.001401751	0.016492917	0.009878243
Ms4a6b	1	2.51E-60	1E-21	-0.000163948	0.028345933	0.009869577
Cnn3	2.63636E-16	1.91E-34	1	-0.04691312	-0.103659985	0.009868482
9930111J2	1	1.50E-83	2E-20	-0.00018479	0.037191815	0.009867973
Sema6d	3.63427E-13	1.54E-26	1	0.01354391	0.052338708	0.009867916
Adgrl3	1	6.68E-05	5E-10	0.003850205	0.012953022	0.00985543
Syt14	1	4.24E-20	1E-30	-0.000339419	0.008252412	0.009849488
4931423N1	1	3.24E-19	5E-20	-0.000159293	0.011889816	0.00984762
Ccdc71	1	1.00E+00	0.0153	-0.005068552	0.009662749	0.009847362
Il10ra	1	5.86E-118	2E-17	-0.000166438	0.058601615	0.009832135
C030034I2	1	1.00E+00	7E-05	-0.001789123	0.00878022	0.009829377
Hfe	1	1.00E+00	0.01	-0.004556495	0.010571254	0.009823802
Hhipl2	1	1.90E-05	4E-27	-0.000422415	0.005088897	0.00982318
Gm26788	1	9.65E-33	6E-40	6.34655E-05	0.008725485	0.009822602
Ehd3	1	1.00E+00	2E-05	0.004350579	0.010316413	0.009822408
C1qa	1	3.49E-53	3E-13	-0.000485411	0.032129701	0.009819714
Tas1r2	1	6.69E-01	1E-26	0.000349607	0.003156886	0.009819015
B3gat2	1	1.00E+00	9E-07	0.002661557	0.007374165	0.009818789
9130019P1	7.57119E-05	2.34E-09	0.1186	-0.008952562	0.024482567	0.009817104
Ssbp4	1	4.73E-42	1E-24	-0.000182769	0.017997848	0.009814796
Zfp113	5.60561E-07	7.76E-02	1	-0.015379462	-0.015956683	0.009800045
Mrpl35	1	1.00E+00	5E-05	-0.001245324	0.003963228	0.009792053
Sirpb1b	1	2.99E-96	7E-24	-5.19431E-06	0.041456713	0.009788085
Gm42809	1	7.05E-04	2E-19	0.000811786	0.006257144	0.009785574
1700034H1	1	1.82E-21	1E-10	-0.000326749	0.019209672	0.009784741
Ms4a6d	1	2.48E-123	8E-31	-0.000134856	0.038813798	0.009784264
R3hcc1	1	1.00E+00	2E-06	-0.000287501	0.007678126	0.009780606
Kctd10	1	1.00E+00	4E-07	-0.002342637	0.00781629	0.009772635
Plscr1	3.43386E-07	1.00E+00	1	0.014312328	0.009990023	0.009751301
Tmem203	1	1.68E-05	7E-12	0.002039181	0.009749337	0.009747421
Slc16a1	5.47834E-19	1.00E+00	1	0.023359886	0.005225246	0.009737982
Trarg1	1	7.16E-12	4E-38	0.000176132	0.004685685	0.009736277
P2rx7	1	3.95E-87	3E-09	-0.002006724	0.050976626	0.009729073
Lig1	1	1.53E-04	7E-08	-0.002267292	0.010626166	0.009719319
Zfp781	1	7.99E-03	7E-23	0.00116542	0.004681172	0.009708957
Stamos	1	6.73E-35	5E-16	-0.000547034	0.019698653	0.00970728
Chst11	1	3.66E-198	2E-17	-0.000502707	0.093204513	0.009705373
B430306N(	1	8.18E-162	1E-26	-0.000232212	0.059449221	0.009686616
Nenf	1	5.14E-16	7E-15	-0.002508816	0.013443732	0.00968406
Zfp688	1	2.00E-01	0.0003	0.004609718	0.010938496	0.009680778
Epb41l2	0.101484724	1.37E-60	1	0.007445815	0.089518147	0.009670902
Trappc1	1	1.98E-01	4E-11	0.000577447	0.006828585	0.00966044

Gm12758	1	7.65E-06	2E-07	0.001230815	0.011909264	0.009653943
Sowahb	1	1.99E-08	8E-16	0.000713961	0.009217611	0.009653609
Polr2i	1	1.00E+00	0.0008	0.005820806	0.007969435	0.009650675
Jam2	1	7.08E-13	2E-10	0.0026158	0.017601552	0.009647679
Zdhhc12	1	1.00E+00	4E-07	0.001555541	0.001341422	0.009638775
Spats2l	1	2.85E-02	4E-20	0.000206309	0.005839726	0.009637779
Adrb2	1	2.24E-07	3E-32	0.00056746	0.005121284	0.009630621
Gm14443	1	1.00E+00	9E-17	-0.001786309	0.003973577	0.009628682
Cytip	1	2.29E-62	1E-25	1.73077E-05	0.026572767	0.009625411
Fbxl7	1	3.72E-09	1	0.007420989	0.040125235	0.009625283
Gm19710	0.000212201	4.85E-15	1	0.015389546	0.042105746	0.009623221
Fbxw17	1	1.23E-54	7E-25	-0.000421369	0.021909637	0.009618669
Rasal1	1	1.26E-21	3E-34	-0.000248328	0.008383062	0.009613318
Tnfrsf23	1	5.66E-15	1E-25	0.000223795	0.008904647	0.00960997
Gm11940	1	1.00E+00	1E-08	0.000670538	0.006306688	0.009602789
Anln	1	1.00E+00	2E-14	-0.001746152	0.004277253	0.009598456
Tmem106b	1.01461E-28	1.00E+00	1	0.05480601	0.014681505	0.009597426
Zfp661	1	1.00E+00	0.0124	0.000788448	0.002618505	0.00959645
Fosl1	1	3.15E-23	1E-23	-0.000221412	0.010391985	0.009589113
Dnal4	1	9.10E-03	4E-12	0.001781663	0.007838763	0.00958171
Cdc7	1	9.84E-22	2E-31	-8.73859E-05	0.00868057	0.00957747
Eaf2	0.201300877	5.83E-03	1	-0.012383386	-0.018893855	0.009574669
Traip	1	1.95E-04	1E-18	-0.000594361	0.006445871	0.009569138
Xist	1	7.70E-10	8E-20	0.000299036	0.008468452	0.009563342
1810013D1	0.058058226	4.24E-04	2E-07	-0.004524161	0.010821876	0.009557189
Plekho2	1	1.12E-122	8E-12	-0.000678332	0.060193357	0.009555321
Bicc1	1	1.28E-06	0.0009	-8.38622E-05	0.019035614	0.00955211
Cep164	1	4.23E-03	1	0.001587793	-0.028333841	0.009551133
Mycbpap	1	1.14E-03	7E-37	0.000158164	0.002133122	0.00954272
Gm9961	1	4.67E-13	3E-07	0.001684491	0.017281477	0.009536359
Cd63	0.117878666	6.83E-25	0.0005	-0.005109379	0.030293294	0.009531357
Jmjd7	1	6.08E-03	1E-11	-0.001024843	0.007443457	0.009522287
Ift122	4.47323E-05	1.00E+00	0.9088	-0.011135543	0.003528457	0.009513841
Gm38102	1	4.02E-07	1E-10	0.000525493	0.010696737	0.009504349
Tyw3	1	1.00E+00	0.0003	-0.002678935	0.005338741	0.009503922
Cage1	0.079128305	6.17E-03	0.0412	-0.006994591	0.01444522	0.009503893
Esyt2	1	1.62E-13	1	0.015538053	-0.060480172	0.009502206
Wdr49	1	1.00E+00	1E-08	-0.001665206	0.002762499	0.009481773
Ciao2b	1	2.09E-01	8E-09	0.002035226	0.008035533	0.009480259
Gm16000	1	8.03E-48	3E-06	3.59683E-05	0.034107302	0.009464924
Snrpf	1	1.71E-06	0.0853	0.005979456	0.020238172	0.009464417
4930539E0	1	1.04E-33	8E-22	-5.76427E-05	0.015161335	0.009462249
Ust	1	3.65E-21	8E-05	-0.000138604	0.028507297	0.00943771
Cdca7l	1	2.39E-25	2E-35	0.00030299	0.00825287	0.009427574
AC154200.	1	1.00E+00	0.0012	-0.003643458	0.002015397	0.00942748
Gtse1	1	1.00E+00	3E-12	0.001101825	0.002075012	0.009426204
Ube4bos1	1	5.39E-04	0.0022	0.004479264	0.014031482	0.009422707

Nav1	1	3.63E-41	1	-0.000540105	0.064291676	0.009414735
Alyref	1	6.22E-02	5E-07	0.00057181	0.008477841	0.009414489
A230083N1	1	3.13E-06	3E-16	-0.000621227	0.007735313	0.009406774
Abi3	1	1.80E-84	2E-22	0.001130721	0.035276219	0.009406026
Gm36355	0.112422002	2.10E-07	2E-13	-0.002917353	0.009721555	0.00940024
Lsm3	1	1.23E-07	3E-11	0.0020876	0.011512989	0.009400213
2810006K2	1	1.00E+00	0.0086	-0.002079317	0.008196167	0.009399814
Selplg	1	2.01E-94	6E-20	-0.000148567	0.039650561	0.009395589
Gtf3c6	1	1.00E+00	0.0052	-0.000125386	0.000363317	0.009392823
Dusp8	1	5.19E-13	2E-20	-2.55064E-05	0.009247245	0.009381396
Thnsl1	1	7.59E-07	7E-12	-0.001335711	0.009732809	0.009374772
Mcmdc2	1	1.05E-19	4E-07	-0.000670454	0.018669491	0.00936476
Rasa4	1	1.16E-146	2E-22	1.90874E-05	0.059803461	0.00935933
Lsp1	1	5.22E-108	2E-18	-0.000408813	0.044370741	0.009358293
Plat	1	1.21E-15	2E-29	-5.18112E-05	0.006066172	0.009353071
Gm26760	1	8.56E-66	1E-17	-0.001518402	0.025786609	0.009345363
Gm3055	1	7.51E-01	3E-11	0.001898082	0.005835625	0.009330129
Gpr68	1	1.15E-18	2E-08	-0.001931758	0.018423613	0.009324563
Spi1	1	1.14E-190	9E-19	0.000139848	0.072982262	0.00932419
Sox6os	1	1.34E-06	5E-19	0.000284724	0.007191604	0.00932356
Gnb1l	0.000283786	1.00E+00	1	-0.010989243	0.008922672	0.009306564
Ltk	1	1.40E-26	3E-31	-3.74266E-05	0.009451216	0.009304657
Mdga1	1	9.30E-21	3E-31	0.000219898	0.008382854	0.009304657
Csf2rb2	1	3.40E-160	2E-31	2.0578E-05	0.053221489	0.009300403
Yif1b	1	5.83E-01	2E-07	-0.003099687	0.00745743	0.009287954
Rnf180	1	6.53E-75	4E-27	0.000813298	0.026034255	0.009272884
Evi5l	0.001938677	3.36E-01	1	-0.012417598	-0.015862514	0.009249469
Rundc3b	1	1.00E+00	1E-05	0.003087676	0.007809079	0.009246852
Ttk	1	1.00E+00	1E-24	-0.000643997	0.00319753	0.009244194
Rpusd1	1	1.74E-11	2E-23	-0.000380381	0.007672825	0.009234874
Dynlt1c	1	2.57E-06	9E-12	-0.000392116	0.009189669	0.009233717
Gm34921	1	1.49E-10	1E-20	0.001980098	0.007450931	0.009228367
Alkbh6	1	1.00E+00	4E-05	0.000787609	0.004800589	0.009221111
Trp53rka	1	2.55E-01	0.0022	-0.003049984	0.01139601	0.009206628
Gtf3a	1	8.33E-30	2E-09	-7.39336E-05	0.022551105	0.009200134
Tmprss15	1	1.33E-04	2E-13	-0.000755205	0.007464273	0.009190927
Hdac11	9.63929E-09	1.00E+00	1	-0.01957711	0.002265727	0.009184218
Cetn3	1	3.63E-01	0.0153	0.000552058	0.012532921	0.009180075
Gm43409	1	5.06E-20	3E-25	0.00032191	0.009528026	0.009179018
2810002D1	1	1.78E-10	2E-16	7.2116E-05	0.009651903	0.009175838
Gm48770	1	1.00E+00	0.0002	0.00253024	0.006712894	0.009173088
C130050O1	1	1.40E-19	4E-14	-0.001496992	0.018261319	0.009170215
Zfp433	1	1.00E+00	0.0419	-0.004727016	-0.001467213	0.009162999
Phf14	1	6.97E-05	1	-0.005082169	0.071814691	0.009161712
Rfc5	1	1.00E+00	4E-07	-0.000699864	0.002025091	0.009161201
Med9	1	3.57E-02	6E-07	-0.002678075	0.008915278	0.009157311
Bmf	1	2.29E-08	1E-05	-0.000753877	0.014635198	0.009154249

Fam241b	1	1.04E-18	1E-28	0.00013244	0.008267833	0.009138605
Dph2	1	9.56E-11	4E-17	0.001216004	0.009604342	0.009134953
Gata2	1	9.02E-20	3E-34	0.000187666	0.00662595	0.009129961
Vwa5a	1.1815E-06	2.60E-04	1	-0.014775756	0.031989277	0.009128592
Mirt1	1	7.81E-170	3E-18	-0.000308895	0.072431548	0.00912619
Pnpla3	1	1.00E+00	1E-17	0.002157795	0.003295336	0.009118688
Fblim1	1	1.03E-124	3E-20	-0.000142733	0.045956902	0.009102282
Ergic2	1	2.50E-52	1	-0.012868097	-0.121504749	0.009090888
Gm39375	1	4.20E-19	4E-38	0	0.004803952	0.009086644
Rasip1	1	5.16E-13	1E-12	0.000653441	0.013786324	0.009081264
1190007I07	1	3.02E-03	0.0175	-0.00029946	0.013707206	0.009075761
Igsf6	1	1.19E-57	2E-07	-0.001281217	0.04294448	0.009071336
Gm17259	1	4.52E-02	2E-05	0.001636624	0.009985133	0.00905336
Trim13	1	2.24E-03	8E-14	-0.001751466	0.006726687	0.009039389
9030407P2	1	6.38E-05	5E-10	-0.00143385	0.009299858	0.009038312
Slc25a36	1	5.83E-60	9E-09	0.000855418	0.03772417	0.00903655
S100a9	1	1.15E-59	1E-24	-0.000404688	0.023272192	0.009022977
Gm15265	1	1.00E+00	0.0099	-0.003503118	0.003311122	0.009015847
Gm13778	1	2.54E-02	2E-08	-0.000847592	0.007839505	0.009013634
0610005C1	1	3.46E-47	1	-0.006499737	-0.134901058	0.008984179
Tmem273	1	6.10E-89	8E-22	-0.000433191	0.033866391	0.008983423
Klra2	1	6.20E-38	4E-17	-0.000393551	0.024069901	0.00897702
Gm28874	1	4.16E-05	2E-07	0.000277104	0.010609444	0.008969765
Plppr3	1	1.00E+00	5E-12	0.00066559	0.005171886	0.00896559
E330020D1	1	1.98E-87	3E-22	-5.35427E-05	0.031696914	0.008964494
Ugt1a1	1	1.07E-07	2E-21	7.10674E-05	0.006676725	0.008955926
Samd12	1	1.00E+00	9E-09	0.000177099	0.004399756	0.00894709
Zfp811	1	2.73E-07	9E-13	-0.002009502	0.008979521	0.008943757
D230022J0	0.041182217	2.04E-03	0.2865	-0.00650875	0.014727691	0.008942774
Zfp72	1	5.86E-04	0.6581	-0.000662883	0.016450534	0.00894091
2510046G1	1	2.62E-06	1E-14	0.001941437	0.008551363	0.008935529
Bbs1	1	1.66E-13	6E-12	-0.000717489	0.012277153	0.008930972
Zfp954	1	1.00E+00	1E-05	-0.000854936	0.006365008	0.008922267
Parpbp	1	3.98E-02	5E-18	-0.000695783	0.004034155	0.008908711
Cep72	1	1.28E-08	5E-11	-0.001049836	0.010912169	0.008906299
Pde1c	1	7.21E-01	0.0041	-0.000999328	0.011046135	0.008904306
Plek	1	1.51E-118	7E-22	0.000205707	0.050001717	0.008900911
Fam229b	1	6.27E-02	7E-20	-0.000339225	0.004228563	0.0088936
Med31	1	1.72E-03	5E-05	0.001473168	0.010693453	0.008888904
Kif3a	0.002456939	1.00E+00	0.0563	-0.007220614	0.011009059	0.008888449
Bcl2	1	6.13E-25	0.0035	0.002470371	0.032839288	0.0088841
Slfn4	1	1.34E-120	4E-24	0.000153702	0.04895536	0.008873844
Gm29773	1	6.21E-06	2E-18	-0.000582876	0.00786712	0.008871889
Chrna1os	1	1.00E+00	4E-08	-0.002458326	0.004165553	0.008871586
Lama2	1	2.29E-06	5E-08	0.0001237	0.010335368	0.008855185
Mfsd3	1	7.68E-03	5E-07	0.00044222	0.008715189	0.008844605
Mirt2	1	2.51E-18	5E-25	0.000200319	0.009264922	0.008843231

Pla2g15	1	1.92E-13	0.0004	-0.002508918	0.018697144	0.008838192
Napepld	1	1.00E+00	9E-05	0.001894069	-0.000718636	0.008837238
Gm17720	1	1.55E-05	3E-13	0.001152301	0.007885055	0.008830345
Gm10451	1	2.74E-01	2E-18	-0.00103157	0.003981655	0.008812419
Gm41764	1	1.00E+00	0.004	-0.000586857	0.005221992	0.008801982
Mccc1	0.00246656	4.81E-05	1	-0.018613039	-0.034050528	0.008801657
Eda2r	1	2.37E-06	6E-19	0.000200108	0.006076317	0.008799145
Shisa9	1	4.15E-18	1E-20	0.001472727	0.010652726	0.008798508
Acta1	1	3.68E-18	1E-27	0	0.00543066	0.008797144
4930503L1	1	1.59E-19	1E-09	0.001140762	0.020844596	0.008792055
Rbm43	0.582424171	4.49E-06	6E-06	-0.003723299	0.012379969	0.008789945
Nudt14	1	5.96E-01	6E-11	-0.001029378	0.005679639	0.008789709
Gm20069	1	7.00E-05	7E-25	-1.858E-05	0.004548084	0.008781707
Borcs8	1	8.29E-04	2E-20	-0.000824325	0.005567258	0.008780379
0610038B2	1	2.03E-39	2E-29	-0.000169673	0.012545826	0.008774187
4930509E1	1	1.61E-04	5E-16	-0.000539987	0.006498019	0.008771793
Gm26636	1	1.00E+00	3E-15	0.001064649	0.003389136	0.008765521
Scn3a	1	1.84E-05	7E-23	0.000519473	0.005317035	0.00876205
Rab7b	1	3.40E-109	6E-30	-2.65769E-06	0.033784993	0.008759544
Aurka	1	5.18E-01	1E-11	-0.000852466	0.005325328	0.008751128
Cenpw	1	5.06E-10	5E-17	-8.45093E-05	0.008472369	0.00875056
Ldb2	2.45085E-31	8.22E-02	1	0.020445194	0.021805337	0.008748098
Gm1604a	1	5.42E-41	1E-20	0.000487193	0.019844117	0.008744457
Adcy10	0.042923204	1.00E+00	1	-0.008641479	0.008158787	0.00874181
Gm48293	1	7.72E-21	2E-11	2.26965E-05	0.016655809	0.008739285
Rab40b	1.65306E-13	1.00E+00	0.0006	-0.009202597	-0.004954495	0.008716727
Ccdc88b	1	5.06E-54	2E-21	-0.000504486	0.024540679	0.008710393
Bmi1	1	1.00E+00	0.016	0.001562686	0.004686788	0.008709461
Gm34590	0.053504528	2.53E-08	2E-05	-0.004381535	0.014299102	0.008704716
Gm47823	1	1.00E+00	3E-17	0.000194271	0.003399174	0.008704335
Fcnaos	1	3.00E-09	6E-14	-0.000448895	0.009276355	0.008683351
Gm15943	1	6.50E-90	2E-09	-0.001763146	0.054853759	0.008683235
Prr5l	1	2.85E-34	3E-12	-0.000453383	0.024329	0.008680794
Itm2c	1	1.00E+00	0.0016	-0.001298493	0.009411386	0.008676711
Tcf19	1	5.74E-13	2E-25	-0.000141049	0.006886796	0.008671433
Fchsd1	1	1.47E-06	5E-22	8.69671E-05	0.005658853	0.008660754
5730419F0	1	7.20E-34	2E-33	0.000453915	0.009726029	0.008646983
Fbxl16	1	6.47E-12	1E-35	-5.18112E-05	0.003809968	0.008644085
Kbtbd4	1	1.00E+00	0.0163	-0.000654667	-0.000874334	0.008629968
St8sia4	1	6.05E-55	1E-13	-0.000205885	0.030823279	0.008622183
Lst1	1	3.99E-87	9E-25	-0.000390376	0.031931253	0.00862123
Hist2h4	1	8.49E-18	5E-08	-0.001712533	0.017328368	0.008615989
Cdpf1	1	1.00E+00	3E-07	0.00106196	0.00641211	0.008614003
Sla	1	1.37E-83	3E-19	-7.21233E-05	0.034697951	0.008607743
5730507C0	1	1.00E+00	7E-11	0.001349659	-0.002352931	0.00858844
Zfp213	1	1.00E+00	0.0048	0.001760252	0.006686347	0.008581994
Rpusd3	1	1.00E+00	0.0341	0.001680603	-3.87635E-05	0.008570425

Hspa12a	1	1.00E+00	5E-08	-0.001037725	0.006369961	0.008552283
Gm15880	1	1.48E-05	9E-08	-0.000411766	0.009521293	0.008541468
4930532G1	1	6.61E-05	5E-17	-0.000309603	0.005957769	0.008533799
Gm26670	1	1.30E-08	2E-18	0.000272096	0.007590051	0.008525758
Ano2	1	6.80E-12	2E-21	-5.68851E-05	0.008027293	0.008521609
1600002D2	1	1.58E-04	3E-15	-0.000840368	0.00623594	0.008515262
Fbxl8	1	3.10E-01	0.0003	-0.0027692	0.009150895	0.00851424
4831440D2	1	1.91E-01	0.0002	0.003761563	0.00853698	0.008512336
Gm49027	1	1.39E-01	2E-14	-0.001405734	0.004890188	0.008489942
Gm30146	3.21311E-12	5.44E-04	2E-35	0.004428287	0.00142581	0.00848852
Cap1	1	4.10E-05	1	-0.004029019	0.041971398	0.008481735
Arrdc1	1	2.38E-15	0.0002	0.000417783	0.021941259	0.008478446
Oasl2	1	4.95E-83	4E-07	-0.001316994	0.05300178	0.008477436
Rasgrp3	1	5.10E-08	1E-05	0.001075894	0.01809934	0.008477413
Unc13d	1	3.78E-21	1E-14	0.000335825	0.014126508	0.00847694
Mgme1	1	7.87E-03	0.3469	0.003254209	0.014645933	0.008475488
Pold2	1	3.85E-01	6E-07	0.003817629	0.006786106	0.008468129
Gm47962	1	5.07E-02	5E-05	-0.000766697	0.00869762	0.008464149
Mapkapk5	1	1.00E+00	4E-07	0.001719559	0.00349864	0.008463454
Csmd3	1	1.00E+00	0.0017	0.00023005	0.007467352	0.008461017
Vps29	0.025983227	1.00E+00	1	-0.010153838	0.009454766	0.008459991
Lrmp	1	6.77E-63	2E-11	-0.000728989	0.031207031	0.008459491
Gm50397	1	3.47E-14	7E-23	-0.000420683	0.007311037	0.008440959
Gm16556	1	2.74E-126	4E-26	-1.78476E-05	0.042565962	0.008440876
Ndufaf6	0.726630617	1.67E-04	1	0.016522367	-0.03585663	0.008437125
Efhd2	1	5.53E-04	1	-0.004044928	0.032546061	0.008433009
Hbegf	1	1.55E-07	6E-15	-0.000340861	0.008465202	0.008430729
4930455H0	0.00048393	1.00E+00	0.0001	-0.005238551	0.004459807	0.008427142
Gm16172	1	1.83E-04	7E-13	0.000302645	0.006781764	0.008420691
AW046200	1	3.17E-17	3E-15	-0.001452373	0.012342281	0.008411206
Ccl6	1	1.09E-47	2E-27	0.000119859	0.015934154	0.008407515
Neurl1b	1	5.20E-03	5E-27	3.40841E-05	0.003221662	0.008407515
Gm30961	1	1.93E-07	5E-30	-0.000162553	0.004086336	0.008405884
Wfs1	1	1.00E+00	0.0172	0.004236756	0.004633068	0.00840473
Gm16351	1	7.04E-02	2E-18	-0.000650238	0.004604004	0.00840341
Mea1	1	1.00E+00	5E-07	-0.001072516	0.003136814	0.008402911
Nyap1	1	1.40E-26	7E-27	-0.000466301	0.009332066	0.008401198
Sytl4	1	1.00E+00	0.0037	-0.003344101	-0.0014589	0.008399788
Nrcam	1	6.53E-05	1E-09	-0.000992612	0.008748985	0.008394164
Pkib	1	9.87E-40	5E-12	-0.000589236	0.024225351	0.008384806
Tmprss5	1	1.52E-05	3E-06	0.001212269	0.010601436	0.008374742
Dyrk1a	0.012187324	9.12E-13	1	0.029838618	0.094827464	0.008370406
Spire2	1	4.45E-18	5E-22	-5.51537E-05	0.009080887	0.008370259
F630028O1	1	1.97E-94	3E-24	0.000258371	0.032387867	0.008368772
Txnl4b	1	1.00E+00	0.0002	0.000363953	0.005611085	0.008362443
Gm20635	1	7.52E-03	0.0006	-0.003696044	0.010266076	0.008359865
Pacc1	1	2.87E-23	0.0001	-0.001404646	0.02618788	0.008342146

Smc1b	1	2.17E-04	3E-15	-0.000282025	0.005982008	0.008335424
Gm37982	1	4.27E-06	5E-11	0.00013231	0.008489623	0.008329017
Gm12781	0.037197865	1.31E-04	0.0022	-0.005459262	0.012823935	0.008325559
Gm17477	1	3.40E-03	2E-07	0.000493406	0.008587252	0.008320158
A730017L2	1	5.35E-05	4E-10	0.002626784	0.008456212	0.008318004
Gm11464	1	5.01E-04	2E-08	-0.001937867	0.009582484	0.00831733
Zfp382	1	6.19E-27	1E-28	0.00040042	0.009312623	0.008316987
Cklf	1	3.62E-27	4E-09	-0.001346633	0.020394356	0.008316604
Cd300c2	1	9.20E-90	6E-22	5.9124E-05	0.030642484	0.00831585
1700102P0	0.000967629	7.77E-01	3E-06	-0.004604717	0.007434776	0.008315132
Gm39078	1	9.82E-23	7E-29	-0.000234016	0.008011116	0.008311981
Snx12	0.000535345	1.00E+00	1	-0.012214313	0.014939828	0.008305102
Il4i1	1	1.00E+00	0.0004	0.001006649	-0.000779105	0.00830061
Phtf1os	1	1.00E+00	2E-09	-0.001309986	0.003065648	0.008300454
Gm35533	1	3.15E-16	7E-18	0.000469514	0.011579567	0.008288584
1700112M	1	6.52E-02	4E-06	-0.002347749	0.007911734	0.008279326
Cfap54	0.000181202	1.00E+00	1	-0.014391781	0.003602423	0.008277647
4933400F2	1	2.10E-04	1E-05	-0.001755983	0.01051383	0.008276873
Rskr	0.002787668	1.00E+00	0.0041	-0.005906983	-8.78238E-05	0.008267262
Gm48529	1	4.12E-03	2E-08	0.002221339	0.007985152	0.0082653
Gm37853	1	3.84E-11	1E-30	-8.30445E-05	0.004462346	0.008263841
Olfr1564	1	1.32E-04	6E-15	-0.000962764	0.00643638	0.008232296
Gm37083	1	2.79E-01	3E-10	-0.000177991	0.005752002	0.008217372
Mfsd12	1	1.78E-56	6E-09	-0.000823817	0.035037714	0.008210766
4833438C0	1	1.00E+00	2E-07	-0.000829034	0.002473074	0.008205213
Clec9a	1	8.90E-42	6E-15	0.000608194	0.022460047	0.008199651
Acoxl	1	1.67E-42	4E-18	-1.94586E-05	0.016354413	0.008196473
Ccdc112	1	1.36E-10	5E-21	-0.000451916	0.007133352	0.008192935
Dlx4os	1	1.47E-11	5E-35	0	0.003174456	0.008189458
Zfp13	1	3.59E-03	1E-10	-0.000581898	0.006697756	0.008184861
C8g	0.000315735	3.34E-08	1	0.01863938	-0.033221929	0.00818477
Pcdh11x	1	1.00E+00	0.0005	-0.001768544	0.005838363	0.008179975
Gm49595	1	5.94E-10	4E-08	0.000783695	0.011886363	0.00817284
Trappc2	1	1.00E+00	4E-06	-0.000534702	0.003337465	0.008170181
Sfxn4	1	1.00E+00	1E-07	0.001492827	0.006275916	0.008165125
Gm36660	1	1.00E+00	1E-13	-0.001733692	0.002269384	0.008160004
Aff3	1	5.02E-16	0.2276	-0.00066901	0.029482035	0.008152705
Ccsap	1	1.47E-14	2E-27	-0.000299166	0.006249732	0.008146588
Wnt2b	1	6.78E-18	1E-07	-0.000744185	0.01618221	0.008136901
Zbtb45	1	1.00E+00	2E-05	0.001256061	0.006922759	0.008126786
Golm1	1	1.86E-11	4E-18	-7.65845E-05	0.008552105	0.00812527
Myo9a	1	3.17E-66	1	-0.002972129	0.134889704	0.008119201
Gm11250	1	5.02E-03	6E-05	0.003947531	0.009215677	0.008116294
Gm50023	1	5.30E-10	2E-07	-0.001308771	0.012353931	0.008113885
Lsm5	1	1.00E+00	0.0376	0.001130229	0.005575205	0.008112865
Gm49191	1	8.49E-60	5E-33	5.90512E-05	0.013959711	0.008094759
A330035P1	1	4.30E-02	9E-09	-0.000349372	0.006669144	0.008087849

Itgam	1	9.35E-150	4E-18	-0.00022775	0.059215332	0.008082733
Ncoa2	1.70782E-58	1.01E-05	1	-0.099447088	-0.039239821	0.008080711
Sdc3	1	1.23E-78	4E-06	-0.00014516	0.047004679	0.008080599
1700047K1	1	5.56E-07	1E-13	0.000584793	0.007726385	0.008073822
Gm14221	1	1.05E-82	6E-17	-0.000340176	0.036482404	0.008071986
4930586N(	1	4.33E-01	3E-05	0.000346715	0.007200925	0.00806611
Rapgef4os3	1	2.35E-03	5E-05	-0.002628043	0.009534597	0.00806024
Tmprss7	1	4.33E-02	7E-14	-0.001558391	0.004619494	0.00805502
D930015M	1	6.45E-04	2E-08	-0.001272318	0.008061894	0.008052567
Slc22a26	1	1.12E-20	2E-30	1.61161E-05	0.006710488	0.008051757
Gm32031	1	1.00E+00	1E-06	0.00108031	0.001934656	0.008039287
Gm40918	1	2.56E-02	1E-09	-0.001374114	0.006321076	0.008039114
Tmem185b	1	1.00E+00	0.0022	-0.00217633	0.006892622	0.008034244
Spcs1	1	1.00E+00	6E-05	0.001142034	0.003060382	0.008033973
Cyp2d11	1	1.12E-02	1E-13	-0.000331864	0.005377971	0.008023247
Zfp607b	1	7.85E-03	3E-08	-0.001207857	0.007225831	0.008018991
Fam216a	1	1.00E+00	2E-05	0.000207819	0.002763699	0.008017976
1810059C1	1	2.68E-10	1E-14	0.000967483	0.008961721	0.008015068
Btla	1	7.99E-23	2E-10	-0.000525051	0.023367453	0.008012098
D730003I1	1	1.09E-02	1E-05	-0.000971479	0.010002631	0.008010588
Drc1	0.000458429	2.50E-04	8E-06	-0.003897305	0.009028601	0.008009279
Tmem191c	1	1.00E+00	0.004	0.002755549	0.002565237	0.008006782
4932438H2	1	2.51E-06	9E-18	-1.85072E-05	0.005738029	0.007999976
Oas2	1	1.10E-67	2E-09	-0.000684105	0.038203071	0.00799596
A930012O1	1	1.08E-01	5E-15	-0.000612497	0.004530514	0.007984145
Iqgap3	1	3.66E-05	1E-23	-0.00039218	0.003502973	0.007983483
Hist1h1b	1	5.14E-19	2E-28	-0.000162553	0.006580508	0.007974603
Gm20512	1	3.58E-11	5E-13	-0.000725849	0.009022003	0.007962237
Akap5	4.84171E-24	2.20E-04	1	-0.024710762	-0.022009832	0.007954105
Gm33447	1	1.81E-29	8E-34	0.000300212	0.00716366	0.007939194
Arnt2	1	1.75E-11	2E-13	-0.000291022	0.011899685	0.00793662
Tmem125	1	1.17E-02	0.0043	-0.004159518	0.010905472	0.007935106
Btbd11	1	8.44E-38	5E-08	0.000898544	0.032239061	0.00793466
Chn1os3	1	1.00E+00	0.0427	-0.004357404	0.001779523	0.007933216
Btk	1	2.81E-105	1E-15	0.000133811	0.043338253	0.007925539
Apol7d	1	1.00E+00	2E-06	-0.000277969	0.00333415	0.007919795
1700019D(	1	1.45E-01	4E-12	0.000182906	0.005162428	0.007917265
Ptges2	1	2.89E-02	0.416	0.001753497	0.012676168	0.007910536
Eme1	1	9.38E-03	1E-13	-0.000311432	0.005928754	0.007908486
Adgre4	1	1.44E-10	2E-11	0.001138937	0.012093776	0.007899642
Zfp947	1	1.00E+00	3E-05	-0.001801553	0.001915801	0.007895066
Gm12212	1	4.84E-07	4E-29	0.00103203	0.003443911	0.007881358
Ccdc114	1	1.70E-16	4E-20	-0.000531425	0.008928678	0.007875345
Clec4n	1	8.49E-73	9E-20	6.8974E-05	0.028386489	0.007872546
Gm34552	1	1.44E-16	2E-27	3.22322E-05	0.006153482	0.00787153
Dnaaf2	1	1.00E+00	0.0035	-0.002125986	0.006280119	0.00786369
Vwf	1	3.40E-55	2E-18	-0.000426925	0.02657642	0.007860372

Ppil6	0.017911021	1.00E+00	1	-0.006944873	0.007318458	0.007858827
Taco1os	1	1.00E+00	8E-12	0.000256005	0.003096307	0.007855603
G630016G(	1	4.40E-03	7E-12	-0.000270888	0.006120106	0.007854286
Prr22	1	1.41E-02	9E-17	0.001139575	0.004588588	0.007851468
Arf2	1	1.00E+00	0.003	-7.28008E-05	0.006441815	0.00784925
Mgat4a	1	8.97E-59	5E-18	0.000475923	0.025576528	0.007848488
Gm35438.1	1	1.00E+00	4E-24	4.02298E-05	0.002522008	0.007842458
Arhgef10	1	6.40E-08	0.0115	0.002172248	0.018348819	0.007827376
Gm10964	1	4.58E-07	1E-05	-0.001595547	0.011203468	0.007807356
A730011C1	1	1.00E+00	2E-09	0.001975402	0.003493026	0.007801582
Anks1b	1	9.29E-02	0.001	-0.003684846	0.009084177	0.007800726
Pclaf	1	2.07E-07	2E-10	0.001198938	0.007828053	0.007800547
Dlgap5	1	4.81E-07	7E-25	-3.28694E-05	0.003920508	0.007799568
Marcks	1	1.15E-74	4E-06	0.000134473	0.042665332	0.007799523
Gm17203	1	2.06E-01	7E-08	-0.001442064	0.006381801	0.0077994
Thg1l	1	2.75E-01	2E-08	0.001603799	0.00587528	0.007794784
Malrd1	1	2.44E-01	0.0027	-0.001410646	0.008195195	0.00778305
A630001O1	1	1.65E-17	9E-05	-0.001094922	0.01862246	0.007772123
Gm16867	1	1.47E-11	2E-33	4.28875E-05	0.003055306	0.007758176
Gm16845	1	2.32E-04	1	-0.00023577	0.016515453	0.007752237
Sp100	1	1.34E-63	1	0.001645678	0.079677808	0.007750372
4930554G2	1	2.62E-01	5E-11	0.00129905	0.006726328	0.007742707
Nek2	1	1.00E+00	2E-10	0.000283823	0.003604061	0.007742696
Cfap43	1	3.78E-11	1E-05	-0.001286388	0.014153717	0.007735744
Platr25	1	1.84E-16	4E-08	-0.001003606	0.014607465	0.007735381
Gngt2	1	9.20E-49	2E-17	-0.000353562	0.01967991	0.007734744
Gm14397	1	5.47E-21	5E-33	0	0.005092177	0.007723553
Gm13562	1	4.20E-02	3E-07	0.001803288	0.006872479	0.007723361
Tgfbi	1	4.65E-58	3E-07	-0.001892276	0.041870875	0.007716429
Gm17103	1	1.00E+00	2E-08	0.002076487	0.005296045	0.0077115
Gm11697	1	5.86E-14	7E-16	0.001293325	0.009069704	0.007698801
Med29	1	1.00E+00	0.0416	-0.001617299	0.003641641	0.007698389
Kitl	1	1.65E-45	4E-07	0.001354608	0.036799541	0.007693942
Gm26873	1	3.38E-01	5E-06	-0.001372969	0.006776967	0.007690967
Tbcc	1	2.92E-12	4E-10	0.002185472	0.011241745	0.007687571
D130040H1	1	5.20E-06	1E-09	-0.001405517	0.009901664	0.007674056
Gm33370	1	9.03E-07	3E-23	-0.000346563	0.004706325	0.007673753
Ehbp1l1	1	1.51E-41	1	-0.003838471	0.060628348	0.00767352
Gm47708	1	1.00E+00	4E-22	1.71151E-05	0.001871573	0.007673094
1700120C1	1	1.00E+00	1E-07	0.000639179	0.005917119	0.007670328
Dnah1	1	3.20E-35	7E-29	0.000541494	0.009740674	0.007663472
Fut2	1	7.08E-59	9E-29	-0.00016348	0.015088225	0.00765346
Mocs3	1	1.00E+00	8E-05	0.00184578	0.004591771	0.007653038
Itga2	1	2.87E-01	0.0004	0.001312128	0.007947859	0.00765058
Chmp6	1	2.74E-03	3E-06	0.00075975	0.008568834	0.00763993
B3galt2	1	7.21E-06	0.0746	0.004396227	0.01522291	0.007637397
Atp13a2	1	4.86E-02	1	0.003598596	0.016077908	0.007634004

Fen1	1	1.00E+00	1E-15	7.66713E-05	0.003625548	0.007621977
Itgb2	1	1.81E-113	1E-16	-0.000337494	0.044093809	0.007620585
Zfp334	1	1.00E+00	6E-09	-0.00188708	0.003261641	0.007607042
Gm50186	1	1.47E-11	3E-32	0	0.003055306	0.007605509
Gm17638	1	4.65E-03	2E-13	0.000863524	0.005008137	0.007603769
C1qtnf12	0.980947737	1.00E+00	0.0002	0.004272567	0.002376189	0.007593609
Ddx41	2.04213E-11	1.00E+00	1	0.023718202	-0.011131959	0.007591105
Fgf12	1	1.00E+00	5E-05	0.001728193	0.004155516	0.007590129
Suv39h2	1	5.50E-05	2E-07	0.000106394	0.00813482	0.007589619
Ppp2r5b	1	1.00E+00	0.0186	-0.000821248	0.008270489	0.007587235
D16Ertd47	0.049369673	1.00E+00	1	-0.015175647	0.000374604	0.007576225
Cplane2	1	3.17E-03	3E-13	0.000727767	0.005496552	0.007574287
Gm15155	1	1.02E-04	5E-06	-0.00017414	0.009346856	0.007573724
Spp1	1	7.18E-42	7E-16	-0.000409883	0.019449693	0.007563733
Lhx1	1	1.75E-05	9E-29	-0.000189397	0.002662222	0.007562507
Col5a2	1	2.67E-01	2E-11	0.001773494	0.005986875	0.007562017
Engase	1	1.68E-02	0.0004	-0.000445272	0.009649754	0.007558677
Bag5	1	1.00E+00	0.0352	-0.002763811	0.002636935	0.007550829
Gm41077	1	1.00E+00	0.0002	-0.001214299	0.004673125	0.007547246
Tacr3	1	1.00E+00	3E-09	-0.000374533	0.003244566	0.007546313
Rnf17	1	1.49E-10	8E-08	-0.001328198	0.010449006	0.007544011
Gm43573	1	1.00E+00	4E-05	0.001295483	0.003007465	0.007540002
Arhgef33	1	2.61E-01	0.0003	0.001783027	0.007806061	0.007539934
Gm45435	1	4.13E-09	0.0008	0.001159038	0.014678834	0.007529113
Gm13710	1	7.66E-01	3E-09	0.000570228	0.005999682	0.007528123
Jaml	1	1.00E+00	0.0022	-0.002058735	0.00562782	0.007526808
Gm11465	1	1.00E+00	0.0132	0.002435818	0.005395691	0.007525128
Ficd	1	1.00E+00	4E-07	0.00102132	0.002144557	0.007515633
Il1r2	1	1.48E-83	3E-15	2.8817E-05	0.037358118	0.007501608
Prox2os	1	8.06E-05	0.0001	-0.002383444	0.009983109	0.007494899
Tek	1	2.76E-02	0.0235	0.001155334	0.012514731	0.007488087
Anxa10	1	2.13E-02	0.0174	-0.000933275	0.010312371	0.007486867
Fhitos	1	1.00E+00	0.0002	-0.000296261	0.003622655	0.007485722
E130114P1	1	1.00E+00	0.0003	0.001560121	0.004822761	0.007472332
9130023H2	1	1.00E+00	1E-06	-0.000273422	0.003588573	0.00746571
C530005A1	1	4.13E-02	0.0031	0.000472721	0.009158961	0.007465663
Jdp2	1	4.77E-88	9E-14	0.000773525	0.037474216	0.007465298
Sox17	1	2.61E-15	2E-14	0.000776966	0.010928887	0.007463558
Vstm2a	1	7.27E-01	1E-07	0.000661249	0.005366294	0.007463344
Fam110a	1	1.16E-04	4E-06	-0.000443877	0.009535791	0.00745957
Ckap2l	1	1.00E+00	4E-22	-0.000177743	0.002077163	0.007459518
4930590L2	1	1.00E+00	0.0139	-4.8588E-05	0.00793795	0.007459378
Tchh	1	7.48E-29	8E-24	-6.69283E-05	0.009712413	0.00745877
Dctd	1	1.42E-21	2E-22	0.000177011	0.008229093	0.007448942
Gm12002	1	2.31E-07	0.0001	0.00012772	0.012349481	0.007442071
Gm28172	2.05176E-05	1.00E+00	3E-10	0.004874019	0.000476955	0.00742801
Necab2	1	1.00E+00	1E-09	-0.001251058	0.005471712	0.007421589

Nwd1	1	3.25E-37	1E-27	7.28103E-05	0.010342244	0.00741901
5430427O1	1	3.33E-17	4E-17	-0.00037485	0.010119097	0.007418793
6330408M	1	3.71E-07	9E-16	0.000939329	0.00623474	0.007405545
Ccdc88a	1	1.54E-67	1	0.001334832	0.070578474	0.007397978
Lrp8	1	1.43E-34	3E-21	-0.000292947	0.012798059	0.00739724
Enoph1	1	1.25E-01	0.0371	0.001474217	0.009554352	0.007381614
Gm13166	0.358826213	1.86E-01	1E-09	-0.002245731	0.005142776	0.007376638
Psma7	1	1.61E-08	1	0.006637463	-0.033740881	0.00736855
Inpp4b	1	1.70E-09	1	0.000125327	0.027790379	0.007368303
Znhit2	1	7.16E-07	5E-08	0.000176053	0.009387425	0.007365285
Rab27a	1	4.64E-14	0.0002	0.000345665	0.02074123	0.007362346
Amz1	1	6.47E-70	4E-10	-0.00085551	0.032246683	0.007361924
2210406O1	1	1.96E-03	0.1101	-0.003534425	0.012308773	0.007361022
Adgrb3	1	1.00E+00	0.0002	-0.000260575	0.005412764	0.007357998
Slc22a15	4.74096E-09	1.00E+00	1	-0.026237798	0.003537035	0.007357798
Unc5c	1	2.22E-05	3E-12	-0.000583043	0.006643314	0.007353637
Foxred2	1	8.75E-22	7E-24	-3.2917E-05	0.008127689	0.007352003
Tlr13	1	2.68E-91	8E-23	-0.000187593	0.029348892	0.007352002
Zfp109	1	1.00E+00	0.0292	0.002459751	0.003977537	0.007338558
Rftn2	1	1.86E-36	1E-09	-0.001225188	0.019687402	0.007337005
Gm47414	1	3.20E-03	3E-15	-0.000861945	0.004724392	0.007336299
4930405A1	1	3.51E-53	2E-25	0.000637119	0.014705763	0.00733493
Slc44a2	1	1.27E-39	0.0151	-0.001018735	0.038561265	0.007333798
Vopp1	1	4.65E-07	4E-09	0.000157061	0.010538766	0.007325406
Gm29642	0.028124682	1.00E+00	1	-0.011540544	-0.007851599	0.007321614
Sac3d1	1	3.81E-02	3E-12	0.002098289	0.004724041	0.007319672
Wrb	1	4.61E-04	0.0006	0.000943676	0.010675738	0.007317887
Slc28a2	1	1.16E-56	2E-16	7.51925E-05	0.024143628	0.007313342
Pram1	1	3.67E-15	0.006	-0.000303078	0.022068816	0.007312928
Cd27	1	5.21E-05	0.001	-0.003509381	0.010960258	0.00731207
Adam19	1	1.28E-28	0.0014	-0.002023259	0.029968157	0.007306543
Gm49171	1.69693E-07	1.66E-01	0.554	-0.008426586	0.010719434	0.007304684
Pgbd1	0.076533497	1.00E+00	5E-06	-0.003195182	0.002721927	0.007301305
Pfkfb4	0.000494358	1.02E-24	1E-07	0.004238705	0.019824877	0.007288114
St3gal2	1	4.39E-10	0.0008	0.001161077	0.016460582	0.007285895
1700048O2	6.52607E-05	7.95E-01	0.008	-0.005369042	0.006887999	0.007273388
Ccna2	1	1.00E+00	3E-12	0.00044652	-0.000133085	0.007266522
Csf3	1	4.31E-02	4E-29	0	0.001018435	0.007265552
Fkbp7	1	1.00E+00	0.0074	0.000141614	0.004431975	0.007256867
Mef2c	1	1.51E-16	0.0025	-0.000220771	0.022566295	0.007252347
Exoc8	1	4.94E-05	0.0393	-0.002071612	0.012283194	0.007250064
Ercc1	1	2.52E-02	0.001	0.001196322	0.008255455	0.007243373
Irf2bpl	1	1.94E-20	1	-0.001094541	0.029547227	0.007234665
Sntg1	1.06314E-09	1.00E+00	1	0.015431721	0.011277639	0.007232869
Homer3	1	6.64E-11	6E-11	0.001169365	0.010538448	0.007228853
Gm37245	1	4.60E-16	4E-16	-0.000429534	0.009763201	0.007222941
C5ar2	1	2.24E-65	4E-20	0.000169818	0.022609716	0.007201533

Galnt13	1	1.00E+00	0.0039	0.000434105	0.005973514	0.007199249
Papss1	1	1.00E+00	0.033	-0.001220081	0.00672887	0.007191691
Pld4	1	2.65E-33	3E-09	-0.001032255	0.020194437	0.00719006
Gm17092	1	1.00E+00	2E-06	0.001945527	0.005719931	0.00718439
4933426K0	1	5.12E-26	3E-26	-0.000164358	0.007768689	0.007177916
Gm48882	1	1.00E+00	0.0021	-0.000175317	0.005017632	0.007175314
Gm6598	0.647631819	1.00E+00	0.0014	0.004567535	0.005670362	0.007173733
Gabra5	0.001326052	1.49E-01	5E-05	0.005570411	0.006951053	0.007173494
Fcgr4	1	1.94E-76	1E-18	-0.000455573	0.027754632	0.007172565
Bicdl1	1	1.00E+00	2E-09	0.000761141	0.002321744	0.007168904
Stmn1	1	4.77E-02	0.0053	-0.003450122	0.00944335	0.007153148
Dffb	0.005228716	2.28E-01	1	0.011748069	-0.014608127	0.007132189
Stx8	3.19731E-13	1.83E-11	1	-0.050051829	0.090052029	0.007127737
Gm16063	1	5.35E-10	2E-24	-8.12403E-05	0.004496288	0.007127664
4930520O0	1	3.01E-08	6E-27	0.000144779	0.003382272	0.007105772
Gm13938	1	8.84E-11	3E-19	-0.000183937	0.006037436	0.007105382
Jak3	1	1.00E+00	0.0013	0.001884398	0.005490137	0.00710403
Itk	1	1.10E-06	4E-17	-9.90151E-05	0.006008044	0.00710252
Hnrnpf	1	2.30E-04	1	-0.001721585	0.053109486	0.007078265
Limk1	1	4.11E-08	3E-12	0.000120341	0.008156655	0.007073299
Duox1	1	3.46E-05	2E-20	-0.000150851	0.003944288	0.007061451
Gm15777	1	2.21E-14	3E-23	-0.000191129	0.005974245	0.007060663
Gm47460	1	1.00E+00	0.0015	-0.001038334	0.002180759	0.00706004
F2rl1	1	2.09E-14	3E-26	-0.000331445	0.005321867	0.007058423
Tox4	1	5.01E-04	1	-0.013197126	-0.029816319	0.007051027
1600022D1	1	6.04E-56	7E-23	-0.000306406	0.015555123	0.007043771
Fbxl14	1	8.42E-13	9E-07	0.000597831	0.013025321	0.007034828
Gm16685	1	2.12E-08	1E-07	-0.000153395	0.008245354	0.007033709
Gm42397	1	4.88E-22	3E-14	-1.67281E-05	0.011666155	0.007031682
Gm48250	1	1.00E+00	3E-07	-0.001301941	0.001569272	0.007026129
Gins2	1	5.03E-05	0.0001	-0.001884108	0.009100863	0.007014163
Cyp26a1	3.18591E-12	1.00E+00	0.0083	-0.007185649	-0.005908333	0.007004964
Fkbpl	1	4.77E-01	1E-06	-0.000394112	0.006590176	0.006995194
Kbtbd8	1	3.65E-01	8E-06	-0.001296144	0.00621417	0.006993867
Rhbdl3	1	3.36E-10	1E-12	0.000128339	0.008031155	0.006986842
Gm45188	1	1.00E+00	1E-06	-0.001867523	0.005306206	0.006986162
Gm50209	1	1.00E+00	5E-05	-0.002733029	0.004404874	0.006985108
Gm14435	1	4.26E-02	7E-13	-0.000526627	0.004501935	0.006979401
Clic1	1	5.88E-12	1	-0.00191971	0.027149912	0.006978753
Tnfrsf4	1	1.00E+00	2E-09	0.000796764	0.001792894	0.006976944
Gm34907	1	1.00E+00	9E-12	-0.000204958	0.002265044	0.006967935
Mis18a	1	6.82E-01	5E-06	-0.001006948	0.006381127	0.006967163
AY512931	1	4.29E-02	4E-19	-0.000747715	0.003092563	0.006963709
Adcy2	1	1.00E+00	0.001	-0.000809309	0.000476067	0.006957731
Tnfaip8	1	1.54E-81	0.0002	0.000792001	0.051538667	0.00695568
Ncaph	1	1.13E-01	1E-08	-0.001501455	0.005309376	0.006951211
4833445I07	1	2.50E-06	1E-12	-0.000134589	0.006610606	0.006932052

Vsir	1	5.89E-90	2E-14	-0.000600159	0.038080124	0.006916673
Ptprq	9.09765E-07	1.00E+00	1	-0.008768794	0.003448585	0.006905949
Edil3	1	1.00E+00	3E-08	-0.000852586	0.003657761	0.006895895
Msi1	1	6.10E-01	4E-24	0.000182278	0.001181687	0.006893074
Gm29856	1	3.99E-15	5E-21	-0.000536886	0.006571899	0.006892366
Txk	1	1.62E-12	0.0012	-0.001827522	0.017392395	0.006889735
Rhog	1	1.34E-04	0.4132	-0.002788152	0.014919433	0.006889035
Creb5	1	1.42E-85	2E-12	0.000598008	0.040166953	0.006886635
Vim	1	7.32E-55	2E-13	0.000597562	0.025251313	0.006879568
Gm45285	1	7.59E-43	6E-28	3.39637E-05	0.010650791	0.006878063
Gm30091	1	1.50E-04	8E-09	-0.00069008	0.00691845	0.006872554
Scel	1	3.56E-10	2E-22	-0.000164358	0.004544307	0.00686631
Pih1d2	1	1.00E+00	2E-10	0.001422108	0.002991236	0.006865178
Ubxn11	1	5.52E-15	9E-15	-0.000250649	0.009106889	0.00685674
Dapp1	1	2.33E-59	2E-10	0.000517284	0.028641523	0.006847001
B130024G1	1	6.74E-01	5E-09	0.000771749	0.005806103	0.006845706
Emb	1	6.92E-81	1E-13	-0.000743205	0.033539624	0.00683648
Mmp8	1	8.55E-74	9E-24	0	0.023537191	0.006826639
Cd55	0.000286366	5.83E-09	0.1091	0.006631193	0.023258144	0.006825494
Gm46224	1	2.92E-81	9E-18	0.000455839	0.027081478	0.006808331
Cbarp	1	2.59E-06	1E-05	-0.001650971	0.009225726	0.006794382
Fam83b	1	1.21E-26	3E-25	4.28875E-05	0.007079122	0.006787103
Inafm2	1	1.00E+00	5E-05	-0.001998123	0.005233242	0.006786399
Cntd1	1	1.00E+00	1E-06	-0.00010458	0.005647813	0.006779559
Nme4	1	3.78E-02	3E-18	-0.000375114	0.003596188	0.006779134
Scrg1	1	1.32E-20	1E-27	0.000167088	0.00635182	0.006772746
C430049B0	1	6.42E-13	2E-10	0.000157552	0.010763745	0.006769899
0610009L1	1	1.07E-08	1E-15	0.00044611	0.006613743	0.006767523
Esrp1	1	1.80E-32	8E-23	-7.41207E-05	0.009633907	0.006767264
Gm12790	1	2.57E-03	2E-07	-0.000446116	0.006758513	0.006766705
Mmachc	1	1.00E+00	2E-05	0.001178581	0.004174302	0.006764381
Morc1	1	1.00E+00	3E-05	0.001509196	0.006138137	0.006761017
Gm12762	1	5.14E-01	2E-05	0.00095273	0.005804011	0.006757916
Cd200r1	1	2.84E-66	3E-13	0.000848261	0.030028991	0.006757877
B230398E0	1	1.00E+00	2E-10	-0.000691079	0.003217822	0.006756962
2610001J0	1	1.00E+00	0.0021	0.001541864	0.007077871	0.006752014
D730045A0	1	1.47E-11	3E-27	0	0.003055306	0.006750849
P2ry12	1	3.18E-24	4E-09	0.000673321	0.018000067	0.006738479
Nlrp1b	1	4.42E-84	4E-09	-0.000532183	0.04085764	0.006736998
2610306M	1	2.46E-02	3E-11	0.001714249	0.004630575	0.006731239
Apol7c	1	1.97E-39	2E-24	0.000149288	0.01317401	0.00672846
Gm38560	1	1.17E-02	9E-05	-0.001454549	0.00847775	0.006728298
Lrrc39	0.000913214	8.55E-06	1	0.008845507	0.016478971	0.006726339
Gm11728	1	6.13E-05	5E-28	0	0.001986945	0.006722498
Gm44987	1	5.11E-01	1E-07	-4.56265E-05	0.004838358	0.006722127
Tmem185a	1	1.00E+00	0.0313	0.000527119	0.004195157	0.006715991
AV356131	1	5.63E-04	8E-09	0.000911897	0.006572816	0.006715731

Ifi30	1	9.74E-08	0.0057	-0.002194808	0.013791057	0.006710404
Ank2	1.14554E-06	1.83E-04	1	-0.009955047	0.0440612	0.00670942
Gm14418	1	1.51E-10	1E-10	-0.000755519	0.008767508	0.006706577
2010308F0	1	1.00E+00	1E-08	-0.001029597	0.00354154	0.006704738
C3ar1	1	2.08E-106	7E-21	-0.000117008	0.034246115	0.006704426
Coro1a	1	6.80E-68	2E-09	-0.000919661	0.032109683	0.006703394
Mdm1	1	1.00E+00	0.0013	-0.002109477	-0.000231418	0.006700186
Arl16	1	1.00E+00	0.0036	0.000625544	0.003946623	0.006699933
Bmper	1	4.70E-12	9E-08	0.002838095	0.013585532	0.006698574
Ncf4	1	4.96E-112	3E-20	0.000129757	0.037128979	0.006689639
Dok3	1	8.44E-10	1	0.001208772	0.021262432	0.006677588
Hmgn2	1	6.56E-06	2E-08	-0.000362929	0.008144909	0.006676565
Gm44764	1	4.06E-25	7E-23	-0.000175061	0.008189221	0.006667889
Abhd10	0.045540824	1.00E+00	1	-0.010592282	-0.001076678	0.006665587
Xkr4	1	7.16E-03	0.0082	-0.000911697	0.00970174	0.006663724
Gm29865	1	5.87E-05	0.0007	0.000943785	0.009892679	0.006659115
Dok2	1	9.64E-08	6E-05	0.003090055	0.011364635	0.006651479
Pitpnm2os	1	2.15E-04	0.0004	0.001419226	0.009417422	0.006650746
Gm17300	1	2.62E-02	2E-06	-0.001067708	0.006274859	0.006650242
Hist1h2ap	1	1.32E-02	1E-11	0.001232469	0.005006793	0.006642681
Atf4	0.056990834	9.48E-12	1	-0.018057508	-0.049220429	0.006641843
Slc4a5	1	3.16E-08	8E-14	-0.000717973	0.006554909	0.006636417
Gm14437	1	6.97E-28	6E-24	-0.000386841	0.008202629	0.00663472
Aoc3	1	3.27E-17	2E-22	0.000305768	0.006313753	0.006631488
Fam227b	1	6.39E-02	3E-06	-0.000809601	0.006340165	0.006622539
Shcbp1	1	1.00E+00	1E-08	-0.00063298	0.002159973	0.00661979
Skap1	1	1.14E-10	0.0962	-0.001859923	0.018366718	0.006618452
Cnpy4	1	1.00E+00	1E-05	-0.001035451	0.002674009	0.006595627
Tmem35b	1	1.00E+00	0.0002	-0.00038924	0.005032801	0.006589286
Fuca2	1	3.93E-04	0.9883	-0.002892304	0.015506977	0.006587936
Pdzrn4	1	3.09E-19	9E-08	-0.000709	0.014981227	0.006583394
Rad54l	1	1.28E-11	3E-12	0.000618586	0.008017802	0.006580506
Gpr183	1	3.24E-01	2E-07	-5.00884E-05	0.005471293	0.006577181
Tas1r1	1	1.00E+00	2E-10	-0.001159258	0.00350728	0.006576465
4930554H2	1	3.99E-01	6E-08	-0.000198714	0.00491552	0.006570589
Gm20559	1	1.36E-34	0.911	-0.000393198	0.038486266	0.006566776
Gm28578	1	1.44E-13	2E-09	-0.001602468	0.010960675	0.006563266
Nell1	1	9.87E-04	0.0014	3.78803E-05	0.009036825	0.006546542
Gm26810	1	5.64E-16	5E-15	-0.000280367	0.00852455	0.006544957
Hist1h1d	1	6.05E-06	7E-09	-0.001614074	0.007059717	0.006537485
Ncf1	1	3.36E-59	2E-10	0.000112908	0.027569292	0.006524781
E2f2	1	3.65E-12	2E-07	-0.000520215	0.010971616	0.006522919
Nlrc4	1	8.74E-38	8E-09	-0.000656359	0.019930894	0.006504607
Gm47767	1	5.47E-21	2E-26	0	0.005092177	0.006500585
Ccr5	1	4.93E-53	2E-10	0.000834875	0.025971713	0.006496289
Gm16093	9.39306E-15	1.00E+00	1	-0.026427923	-0.016623774	0.006495304
A930018P2	1	2.22E-06	7E-21	0.000413758	0.003781037	0.00648944

Gm16223	1	1.00E+00	7E-05	-0.000399258	0.00444051	0.006486552
4732491K2	1	3.64E-02	0.002	0.000706638	0.007731398	0.00648409
Gm48708	1	5.06E-01	0.0211	-0.000932101	0.007364957	0.006480247
Gm16068	1	1.00E+00	0.0273	0.00047182	0.003907495	0.006475889
Cd72	1	6.48E-13	9E-11	0.001804366	0.009851513	0.006475367
Ly6m	1	1.73E-38	2E-26	-4.46189E-06	0.009714316	0.006473684
Rab3a	1	1.00E+00	0.0002	0.002173207	0.003222305	0.006473359
Gimap4	1	3.15E-18	2E-07	0.00045554	0.015715453	0.006464026
Cachd1	1	3.24E-03	0.0127	0.002746736	0.012304651	0.006463745
P2ry6	1	3.94E-69	6E-13	-0.000423293	0.027184146	0.00646158
Olfr1393	1	1.08E-02	3E-06	-0.000435341	0.006380952	0.006457677
Sipa1	1	8.42E-14	0.882	0.001587629	0.029797909	0.006456836
Cdk17	1	1.45E-25	1	-0.008748724	0.114089047	0.006455922
Gm28494	1	5.11E-08	0.0012	0.001063571	0.011939711	0.006454752
Klf2	1	1.33E-17	8E-05	-0.001021924	0.017394344	0.006448642
Nuf2	1	1.00E+00	5E-20	3.95199E-05	0.002233501	0.006447096
Naip5	1	5.44E-40	5E-08	-0.000724022	0.022318187	0.006444244
Ltbp2	1	3.05E-04	7E-07	-0.000285957	0.007000622	0.006441262
Slc26a8	1	1.00E+00	2E-05	-0.000267158	0.001942705	0.006438811
Duoxa1	1	8.14E-24	7E-27	0	0.006060687	0.006437612
Snx20	1	1.33E-82	4E-19	-0.000207245	0.028208279	0.006435164
Hacd4	1	1.63E-43	6E-05	-0.001090114	0.030506028	0.006433859
Themis2	1	6.01E-75	1E-10	-0.000827055	0.034702732	0.00642063
Wasl	1	1.06E-14	1	0.017161463	-0.068707181	0.006419431
Luzp2	1	2.19E-05	0.0017	-0.000396575	0.009775895	0.00640259
H2afy2	1	3.12E-08	3E-12	-0.00015322	0.006396439	0.00639158
Bcl2a1b	1	3.35E-48	1E-18	-0.000202783	0.016406171	0.00638983
4930458D0	1	1.00E+00	3E-07	0.001860363	0.003732701	0.00638798
Kif2c	1	1.32E-01	2E-14	-0.00024282	0.003219758	0.006380038
Msh2	0.000100019	1.00E+00	1	-0.011839203	-0.000450199	0.006378988
Sf3b5	1	3.42E-06	0.001	0.001744766	0.011063744	0.006373108
Phkg1	1	1.00E+00	0.001	-0.001654108	0.001478207	0.006368502
Tulp3	1	1.02E-03	2E-08	7.21385E-05	0.006346582	0.006368397
Svop	1	7.95E-11	4E-18	-1.33129E-05	0.005121638	0.006362221
Cdadc1	1	2.70E-07	1	-0.014435995	-0.044981832	0.006361646
Gm48371	1	4.20E-19	2E-26	0	0.004803952	0.00635419
Mrps6	0.030807033	1.00E+00	1	-0.010015354	0.005973925	0.006348855
Tm7sf2	3.30286E-05	4.90E-18	1	0.014271398	-0.031080482	0.006345851
Gm11832	1	1.00E+00	0.004	0.003202374	-0.001342083	0.006343484
Lgals1	1	8.47E-25	7E-09	-0.00129682	0.015114949	0.006327973
Fcor	1	1.72E-12	8E-18	-0.000260788	0.006097523	0.006324837
Gm12743	1	1.30E-02	0.1749	0.000872309	0.011430151	0.00632117
Gla	1	1.58E-12	3E-07	-0.000726679	0.01143486	0.006319544
D930016D0	1	1.04E-09	1	-0.008937223	-0.049686544	0.006315356
Mir155hg	1	4.07E-19	1E-19	-0.000182205	0.007529365	0.006314876
Rpusd2	1	5.94E-09	7E-10	0.000378377	0.007836857	0.006314271
Tex38	1	5.39E-17	2E-19	-8.47283E-05	0.006622495	0.00631132

Gm10642	1	1.18E-01	4E-10	0.001657761	0.004359887	0.006302203
Gm6209	1	4.24E-27	1E-08	-0.000307823	0.020787014	0.006300231
Olfr1443	1	1.46E-08	4E-13	-0.0007789	0.006372659	0.006295484
Arfip1	1	1.00E+00	0.0191	0.003512464	0.007234684	0.006288273
Abcc12	1	1.40E-75	3E-06	-0.002163252	0.035029517	0.006280549
Bub1	1	1.00E+00	6E-14	-0.000319718	0.000799964	0.006272659
Fancd2	1	1.00E+00	0.0017	-0.001806525	0.005059838	0.006270038
Tgfb1	1	6.88E-61	1E-05	-0.000185114	0.0344096	0.006266717
Ascl4	1	8.33E-03	5E-05	0.00280104	0.006706684	0.006264385
Cnn2	1	7.32E-33	1E-09	-0.000147784	0.017533965	0.006263282
Gm49463	1	6.09E-03	2E-05	-0.000635176	0.006879951	0.006259701
Arhgdib	1	1.10E-38	8E-09	-0.000116431	0.021516604	0.006251163
Gmfg	1	3.68E-04	0.007	-0.002412758	0.01140411	0.006246618
Gm14066	1	1.00E+00	0.0067	-0.003122238	0.005853197	0.00624583
Gm34655	1	1.00E+00	1E-09	0.000731446	0.003068961	0.006245353
Flrt2	1	3.29E-80	1E-15	0.000759635	0.03292758	0.006238654
Cacna1c	1	9.51E-04	0.0005	-0.000260699	0.008567172	0.006234564
A930004D1	1	2.14E-11	1E-05	-0.001769952	0.010929281	0.006233327
Pde11a	1	6.33E-19	8E-13	0.001137913	0.009603955	0.006233163
Ccdc150	1	1.86E-04	8E-05	-0.00115543	0.008270731	0.006231348
Gm1647	1	9.41E-15	7E-14	-0.000619763	0.00815648	0.006230128
Kcnk6	1	8.35E-46	2E-14	6.26071E-05	0.018583307	0.006229253
Sel1l	1	1.00E-21	1	-0.012279439	-0.086690891	0.006220405
Depdc1b	1	2.14E-04	5E-14	-0.000120423	0.004514351	0.006219043
Agtrap	1	3.85E-33	1E-12	-0.000150515	0.015441682	0.006215619
Ube2c	1	4.68E-02	4E-20	-0.000286754	0.00238504	0.006215035
Cdc20	1	3.73E-06	3E-18	-0.000410028	0.004366622	0.006207451
Necab3	1	3.84E-33	7E-20	0.000205634	0.010281487	0.006202309
Cdca7	0.452507292	1.38E-02	0.0067	0.004416823	0.008138773	0.006199203
Cd80	1	3.88E-43	2E-12	0.000601424	0.019047378	0.006192935
Gm13599	1	4.32E-75	1E-11	-0.000194519	0.026028759	0.006186605
Dynlt1a	1	7.83E-05	0.0002	-0.001116957	0.008770227	0.006186106
Gm50340	1	4.29E-18	2E-15	-0.00016782	0.00848581	0.006182131
Dennd1c	1	6.02E-61	2E-09	-0.001092474	0.0283067	0.006180797
Palmd	0.019337192	1.00E+00	1	-0.027872854	-0.025892435	0.006180264
Dgki	1	1.13E-11	0.0021	-0.00171085	0.015681702	0.006171337
Cd86	1	6.44E-54	6E-09	0.000128266	0.029946217	0.006163257
1700111N1	1	1.00E+00	0.0047	-0.00219151	0.002934258	0.00615793
Polr2d	1	1.00E+00	0.0061	-0.000681731	0.005591431	0.00615535
Gm49774	1	1.23E-06	9E-05	0.001375903	0.009858449	0.006141198
BC026762	1	3.22E-17	1E-25	0	0.004277429	0.00613855
Gm16341	1	4.23E-30	2E-13	-4.18884E-05	0.011813832	0.006136234
Trim43c	1	2.24E-09	0.0033	-0.001082726	0.012469125	0.006135899
4930533B0	0.019456601	1.00E+00	9E-05	0.004404734	-0.000671956	0.006133942
1700061I1	1	9.44E-13	1E-13	0.000431846	0.006609969	0.00613319
Gm14211	1	8.11E-32	2E-21	0.000749665	0.009202083	0.006131619
Gm46102	1	1.00E+00	0.0012	-0.002398948	0.004949816	0.006122723

C030034L1	1	2.94E-21	8E-16	-0.00025801	0.009596367	0.006122713
Pcyt1b	1	1.00E+00	3E-08	-0.000946421	0.000833554	0.00612091
Mutyh	1	1.00E+00	2E-13	0.000615808	0.002620202	0.006115124
Gm3604	1	1.19E-02	6E-12	-0.000154362	0.004264675	0.006105949
Blnk	1	2.12E-67	2E-05	0.000115194	0.036041113	0.006105584
D930032P0	1	1.39E-21	2E-22	5.90036E-05	0.006556727	0.006104717
Rgs2	1	2.70E-42	2E-16	0.000298481	0.015960583	0.006104377
Gm13199	1	4.61E-05	2E-11	0.00100633	0.005521604	0.006103183
Gm28644	1	6.15E-13	2E-10	0.000277243	0.008254074	0.006096911
Car7	1	4.57E-06	1	-0.004860719	0.013723815	0.006091817
Gm28085	1	1.46E-05	7E-06	0.001869049	0.00797259	0.006091289
Cxcr5	1	5.21E-27	0.0013	-0.002684044	0.020648109	0.006090271
3222401L1	1	7.13E-12	4E-09	-0.000423075	0.00890084	0.006086885
Cd93	1	5.97E-80	4E-13	-6.04942E-05	0.029259946	0.006072505
Ddit4l	1	5.19E-18	1E-22	0.00032079	0.00570479	0.006068645
Mageb18	1	1.00E+00	0.0026	-0.001428605	0.005348161	0.006063767
Nlrp9c	1	3.34E-24	2E-20	-0.000297482	0.007517652	0.006060266
9230116L0	1	5.14E-09	4E-19	-2.39932E-05	0.004402259	0.006048988
Gm1123	0.000556175	5.54E-19	0.4174	-0.004918194	0.024955138	0.006045351
Ikzf3	1	1.22E-18	2E-06	-0.000760667	0.016930541	0.006041495
4930402D1	1	1.00E+00	9E-17	0.000298481	0.000137568	0.006035981
Gm15726	1.05601E-09	1.00E+00	0.2477	-0.006622843	0.009412754	0.006035762
Fanci	1	1.13E-02	9E-08	-0.001102275	0.004646098	0.006021388
Ednrb	0.193611625	9.17E-27	3E-10	0.002286025	0.016472346	0.006020283
Zfp1	1	7.96E-04	1	-0.005007785	-0.025687248	0.006011844
Srgn	1	1.63E-43	9E-06	-0.00069784	0.029529666	0.006008317
Rmi2	1	7.22E-03	2E-11	0.00048551	0.00442791	0.005999978
Hist2h2be	1	7.14E-12	5E-09	1.10673E-05	0.008976557	0.005997993
Ctla2b	1	1.68E-19	7E-14	0.000523104	0.009754465	0.005997601
Gm29282	1	1.83E-40	9E-17	-0.000259056	0.016193923	0.005986197
Lat2	1	4.48E-03	1	0.000501826	0.013016411	0.005982395
AB124611	1	4.22E-75	0.0004	-0.00073019	0.041552768	0.005980183
Gm16549	1	1.77E-01	0.0005	0.003114216	0.006186723	0.005976069
D830044D1	1	8.11E-02	2E-06	-0.00099473	0.005229641	0.005975692
9130017K1	1	6.34E-14	1E-17	0.000172644	0.006066367	0.00597347
Fcgr2b	1	1.26E-17	1	-0.000376107	0.0299074	0.005972455
Pou4f1	1	6.92E-06	6E-25	0.00017155	0.001833184	0.005971707
Wls	1	3.21E-31	0.0074	0.002655335	0.027123993	0.005970803
4930470G0	0.002081906	1.13E-27	1	0.022681912	-0.064363752	0.005963879
Card19	1	2.60E-02	0.7989	-6.13868E-05	0.012085672	0.005960175
Irak3	1	1.65E-32	4E-05	0.001851389	0.021790019	0.005958075
2010003K1	0.024633746	1.00E+00	7E-05	0.004094025	0.003500402	0.005957282
Gm9774	0.052170191	1.05E-03	4E-08	0.003082091	0.005511091	0.005957235
Trerf1	1	2.28E-19	0.0177	0.000521255	0.022848839	0.005946381
Gm19610	1	7.36E-02	1E-09	0.000817859	0.004226448	0.005942274
Insyn1	1	9.30E-25	2E-24	0	0.006026075	0.005937084
9130230N0	1	1.00E+00	0.0051	0.000526558	0.004725273	0.005936275

Gm28526	1	1.00E+00	0.0001	3.37645E-05	0.001930429	0.005930047
Ticrr	1	6.62E-06	5E-18	-0.000561048	0.003588956	0.005925
Ldb3	1	1.23E-15	1E-12	-0.000938844	0.008301986	0.005924607
Gm32036	1	1.39E-03	0.0001	-0.001025938	0.007870372	0.005924436
Adgrg3	1	2.32E-19	5E-07	0.001098875	0.016204706	0.005911525
Gm9958	1	1.00E+00	0.0029	-0.000874221	0.002714513	0.005907885
Slfn5	1	1.68E-64	0.0024	-0.00025363	0.045431966	0.005901575
Espn	1	7.96E-04	1E-12	-0.00096374	0.004368848	0.005899484
Stap1	0.885512699	2.35E-20	0.0184	-0.002969777	0.024054451	0.005896201
Ap3d1	2.86775E-17	2.84E-04	1	0.044582833	-0.028962259	0.005892421
Loxl3	1	4.13E-60	3E-12	0.000391616	0.023734419	0.005890388
Gm5463	1	9.30E-25	8E-22	0	0.006264374	0.005889917
Myrfl	1	1.53E-15	2E-15	0.000622049	0.00715057	0.005887283
Espl1	1	7.20E-07	5E-15	-0.000367141	0.004212861	0.005881669
Dck	1	2.33E-07	1	-0.000663121	0.016769789	0.005877373
Spata5l1	1	2.99E-03	2E-07	0.002102257	0.005805667	0.005876849
Pitpnm1	1	4.55E-05	0.0582	0.000985515	0.013288964	0.005875007
Frmd4a	1	6.66E-61	1	-0.000223053	0.056290345	0.005873592
Rhod	1	3.14E-05	1	0.006412161	-0.031754694	0.005869737
Mmgt2	1	1.31E-01	1E-08	0.000508963	0.004994284	0.005868999
Nsmce4a	1	7.57E-01	0.0369	-0.000492093	0.006790867	0.005868345
5830454E0	1	1.40E-02	2E-09	0.001500474	0.004826887	0.005863533
R3hdml	3.3385E-09	1.00E+00	1	-0.012977129	1.79828E-05	0.005859893
Slc44a4	1	3.86E-55	2E-21	-2.23094E-05	0.013852274	0.005818382
Gm1604b	1	6.30E-14	1E-08	-0.000626834	0.009765949	0.005817825
Haspin	1	4.80E-21	1E-10	-0.000362533	0.010748454	0.00581671
Cr2	1	1.61E-12	0.0007	-0.001175035	0.011943694	0.005806676
Kcnj16	1	6.32E-14	2E-09	-2.82619E-05	0.009864335	0.005803375
Gm12542	1	3.11E-06	5E-16	5.46091E-06	0.004036202	0.005800964
Tspan5	1	2.71E-23	1	0.001169825	0.037176034	0.005795503
Ier5	1	3.48E-11	0.0235	-0.000452779	0.014421701	0.005788302
Kif21b	1	2.00E-39	3E-08	3.53239E-05	0.020430777	0.005786429
Rassf2	1	8.95E-47	6E-07	-0.000528575	0.023930564	0.005782105
Rad51ap1	1	1.64E-02	1E-08	-0.000465278	0.004719818	0.005781852
Gm48202	1	7.81E-20	2E-21	-0.000193859	0.005873866	0.005769583
Mef2a	2.70963E-08	4.19E-15	1	-0.040725949	0.117075005	0.005769035
Fam3b	1	8.25E-13	6E-06	0.001573273	0.010804909	0.005768755
Myadml2o	1	1.36E-09	2E-12	-0.000370603	0.006405719	0.005767264
Selenot	0.022762975	1.00E+00	1	-0.015370535	0.000844875	0.005758126
Gm17108	1	1.54E-10	3E-06	-0.000383554	0.009752532	0.005757483
Gm44759	1	1.00E+00	0.038	0.001601792	0.005365096	0.005753544
Mageh1	1	5.22E-02	1E-12	0.000402249	0.003437065	0.005750048
Sowahc	1	2.89E-09	0.0011	0.000206335	0.012739331	0.005738135
Gm47272	1	1.00E+00	0.0152	0.001926851	0.005696329	0.005729419
Gm36495	1	3.52E-34	9E-24	0	0.008062946	0.005721443
Gm16083	1	1.00E+00	0.0001	0.001336742	0.005112713	0.005715128
Asgr2	0.012124255	1.00E+00	1	-0.016685583	0.005416077	0.005713663

Aox2	1	9.04E-03	2E-09	0.0001557	0.004360239	0.005708161
1700095J1	1	3.02E-12	4E-14	-0.000411759	0.006352689	0.005705607
Gm17382	1	3.95E-02	0.0155	-0.001838155	0.008290201	0.005694393
Nexmif	1	1.04E-02	6E-07	-0.000267624	0.004882562	0.005693002
Trim46	1	1.12E-07	2E-19	0.000478954	0.003729559	0.005676334
Spx	1	1.00E+00	4E-11	0.000604274	0.002820642	0.005673218
Gm10101	1	1.00E+00	9E-09	-6.12743E-05	0.00319739	0.005662068
Arhgap9	1	7.40E-61	1E-09	-0.000199812	0.024771441	0.00565698
Kif18b	1	1.76E-09	6E-17	-0.000383257	0.004293271	0.005651063
Siglec1	1	5.12E-39	2E-11	-0.000281077	0.018756456	0.005649735
Aurkb	1	4.13E-09	7E-19	-6.96588E-05	0.003943407	0.005646716
Gm10602	1	9.88E-02	0.0322	-0.002089308	0.007435026	0.005642431
Zfyve9	1	1.08E-08	1	-0.016993733	-0.052250306	0.005641808
1700022A2	1	4.96E-02	3E-08	4.32268E-05	0.004535877	0.005641758
Gm11008	1	9.07E-03	2E-06	6.29997E-05	0.005606109	0.005627376
1300002E1	1	1.00E+00	9E-05	-7.0857E-05	0.004185628	0.005625056
Sbk1	5.35234E-18	1.53E-05	1	0.031230478	-0.028437395	0.005618453
D430020J0	1	6.55E-06	5E-18	3.66941E-05	0.00370666	0.005617707
Gm50311	1	1.51E-04	2E-07	-0.000649118	0.006037615	0.005609374
Ankrd27	2.8054E-07	1.00E+00	1	-0.02623906	0.022133453	0.005607474
Tuba1c	0.040993949	2.23E-01	1	-0.009468274	0.019359417	0.005601246
1700007L1	1	2.70E-06	2E-11	0.000145898	0.005203248	0.005599626
Rab34	1	1.00E+00	0.0254	-0.002049415	-0.001090338	0.00559739
Prr15	1	8.20E-10	7E-15	-0.000172187	0.005677411	0.005595942
4933424L2	1	4.88E-11	9E-09	0.000327323	0.008198115	0.005592169
2310026L2	1	1.39E-27	2E-23	0	0.006637136	0.005589224
Casc1	1	6.89E-07	6E-11	0.00025937	0.00648071	0.005558077
Zfp326	1	7.04E-05	1	-0.005182999	-0.030077166	0.005557646
Zfp52	1	6.85E-08	1	0.001178439	0.016344117	0.005556452
Gnat3	1	6.82E-75	2E-17	4.91536E-05	0.023321911	0.005556257
Gm14009	1	1.04E-03	9E-16	-0.000205441	0.003033289	0.005549114
Nsl1	1	7.21E-07	9E-16	-0.000266176	0.004304313	0.005547007
Adgrg6	1	3.56E-05	0.0013	-0.001017092	0.009594551	0.005544679
1700105P0	1	1.88E-07	1E-22	3.84256E-05	0.002516045	0.005541875
Slfn8	1	1.72E-64	5E-07	4.7831E-05	0.034670436	0.005540196
Gm16759	6.58162E-11	1.00E+00	1	-0.016307153	0.006823337	0.005534461
Fgfr3	5.48036E-28	1.74E-02	1	-0.033556622	-0.022589003	0.005530137
Fam71e1	1	1.00E+00	0.016	-0.000511708	-0.001387278	0.005523468
Gm5148	1	1.00E+00	1E-13	0.000369139	0.002289108	0.005515419
Dnaaf4	1	9.22E-05	4E-06	1.0646E-05	0.006973276	0.005498383
Cep55	1	3.50E-04	7E-18	-8.75064E-05	0.003108336	0.005498214
S100a8	1	6.97E-38	1E-12	-0.000125932	0.014471319	0.005493646
Cd37	1	1.30E-47	8E-08	-5.68851E-05	0.023234884	0.005491724
Ptger4	1	8.52E-09	1	0.000382273	0.018660544	0.005486377
Gm39323	1	3.57E-16	6E-16	-0.000245671	0.006017466	0.005477932
Il1b	1	2.61E-68	3E-14	0.000186908	0.031280275	0.005474981
Fgd3	1	4.54E-72	2E-13	-3.11128E-05	0.026805703	0.005458762

S100a11	1	6.52E-27	4E-16	0.000361994	0.010228629	0.005457272
Wfdc17	1	1.81E-21	4E-13	-0.000496656	0.010532712	0.005452303
Atp8a1	1	3.91E-46	1	0.000887952	0.095003396	0.005449777
Plppr2	1.44606E-06	4.51E-04	1	0.016197186	-0.020483301	0.005447553
Egfl7	1	3.39E-04	1	-0.001700019	0.024789734	0.005445905
Rhoj	1	3.95E-04	1	0.001564147	0.012678544	0.005444869
Zglp1	1	3.48E-02	9E-07	-0.000449703	0.00498612	0.005441874
Efcab8	1	1.00E+00	0.0135	-0.001926996	0.004654358	0.005441703
Gm39556	1	6.30E-37	4E-12	-0.000408224	0.015873277	0.005439717
Gm26936	1	1.49E-06	3E-19	0.000169866	0.003332345	0.005437347
Serpinb3b	1	1.00E+00	0.0004	0.00013302	0.002605033	0.005435659
Gm30541	1	1.00E+00	7E-05	-0.001799694	0.004477201	0.005433616
Cmtm3	1	1.12E-14	2E-10	0.00039603	0.008403033	0.005430726
Tarbp1	1	2.21E-02	1	-0.006869991	0.02147243	0.005429629
Pecam1	1	5.55E-10	1	-0.000111965	0.021983425	0.005428509
Gm28535	1	3.06E-07	5E-12	0.000734621	0.005583594	0.005428436
Gm16178	1	1.00E+00	0.0006	0.001474234	0.004668931	0.005425831
Gssos1	1	3.89E-01	8E-09	3.60821E-05	0.003678259	0.005424536
Tm6sf2	0.998187007	6.97E-03	1	0.005527368	-0.010017392	0.0054244
Gm41555	1	2.69E-10	3E-06	0.000464176	0.008943514	0.005423862
Osgin2	1	1.39E-03	0.0017	-0.000849783	0.007834316	0.00542358
Selenoh	1	1.00E+00	1E-06	-0.000973396	0.002647404	0.005412261
Gm35279	1	1.34E-11	9E-08	0.000156747	0.008419339	0.005407124
Galnt3	1	8.66E-39	2E-19	-1.33857E-05	0.010349828	0.005406281
Gm10635	1	9.47E-04	5E-08	0.000731158	0.006012491	0.005405491
Tex22	1	1.78E-05	8E-12	-0.000129395	0.004803054	0.005398166
Mitd1	1	2.84E-03	1	-0.007361753	-0.023525148	0.005394731
Pilra	1	6.13E-45	3E-10	-0.000119545	0.019186582	0.00538179
Gm26672	1	1.00E+00	3E-09	-0.000367562	0.002532417	0.005380564
Tctex1d2	1	1.64E-04	0.0001	-0.000779271	0.00796934	0.005375647
Csrp1	1	4.23E-19	9E-07	-0.000469571	0.012587845	0.005370251
Tceal5	1	1.00E+00	5E-21	0.000368187	0.000611061	0.005367311
Cacnb1	1	1.12E-02	2E-07	-0.001135265	0.004996632	0.005359637
Lgi4	1	1.28E-09	3E-13	0.000623853	0.005520404	0.005355502
Angptl7	1	2.21E-03	0.0431	-0.002128853	0.008463092	0.005354387
Cgref1	1	1.66E-21	8E-10	0.000279948	0.010290063	0.005341778
Gm43599	1	8.55E-07	4E-06	0.001037757	0.007720812	0.00534025
Tmem117	1	4.27E-18	0.0002	-0.000836689	0.014425632	0.005335815
Prr18	1	1.72E-07	1E-07	-0.000584042	0.007085651	0.005333531
Lrrn4	1	1.63E-01	8E-19	0.000517428	0.001596437	0.00533203
1700063J0	1	1.00E+00	3E-08	0.000273755	0.00345492	0.005321463
Cpsf4l	1	1.49E-03	1	0.001769657	0.021068838	0.005321203
Prmt2	1	1.20E-01	1E-11	0.000301332	0.003519696	0.005313948
Enkd1	1	1.00E+00	1E-05	-0.000338911	0.002354804	0.005312217
Armh1	1	1.03E-05	2E-13	0.000206513	0.00421699	0.005311056
Tmprss11d	1	1.53E-06	1E-05	0.000153236	0.007486642	0.005306417
A730071L1	1	1.00E+00	2E-05	-7.43708E-05	0.000547873	0.005290788

Fmnl1	1	2.59E-44	7E-06	-0.000218633	0.024284959	0.005290728
Sbsn	1	1.00E+00	7E-06	0.000206947	0.00448147	0.005290655
Lzts1	1	1.25E-07	4E-06	-0.000763301	0.007601764	0.005284579
Samsn1	1	5.14E-16	0.0457	-0.000468498	0.020370333	0.005284491
Alms1	4.03091E-07	1.00E+00	1	-0.020288533	-0.00225153	0.00528365
Casp1	1	1.26E-38	1E-10	0.000120785	0.015989905	0.005278676
Gm46516	2.20895E-07	4.66E-12	1	-0.010875174	0.027299725	0.005272556
Tbx2	1	4.71E-12	1E-05	0.000966183	0.010989716	0.005257151
Spic	1	8.40E-60	9E-14	0.000192296	0.021294654	0.005255011
Gm15663	1	6.47E-10	5E-08	-6.49303E-05	0.007709451	0.005248661
Gimap3	1	5.79E-20	3E-06	-7.36384E-05	0.014623141	0.005233213
Rims3	1	6.80E-72	5E-13	6.62436E-05	0.024223898	0.005229133
Tbc1d22a	0.436448171	3.61E-07	1	-0.016611738	0.077694763	0.005227802
Pck2	1	1.86E-09	9E-08	0.000672202	0.007981017	0.005227285
Ebi3	1	4.21E-18	2E-18	0.00019659	0.005871289	0.005226712
Spata6	1	6.67E-26	1E-06	0.000397366	0.017835826	0.005225167
B3galnt1	1	2.57E-17	2E-11	-0.000764709	0.009012542	0.005222015
Gm12227	1	2.92E-05	3E-07	0.000954903	0.00625961	0.005217264
Rps6ka4	1	1.98E-19	3E-07	-0.000104209	0.011597462	0.005208231
Exoc3l2	1	1.00E+00	0.0068	-0.000154733	0.006967979	0.005198392
Tmem17	1	3.74E-03	0.326	-0.00244738	0.009349671	0.0051942
1110006O2	1	4.74E-01	1E-08	-0.000497341	0.003660722	0.005192475
1700003G1	1	1.00E+00	0.0296	-0.00019014	0.004079035	0.005185044
Cdh12	1	3.11E-03	0.2755	0.000659761	0.008965344	0.005182454
44441	1	1.00E+00	8E-05	-1.29008E-05	0.000663105	0.005179663
C030006K1	1	1.00E+00	0.0009	-0.000821328	-0.001190082	0.005178008
Ezr	1	2.93E-26	0.0082	0.000357331	0.022796633	0.005176377
Nrip2	1	1.00E+00	6E-05	0.00082807	0.004129033	0.00516984
Eno4	1	1.15E-03	2E-09	-0.000806598	0.004511778	0.005169135
Snai1	1	4.68E-02	2E-16	0.000366456	0.002293588	0.005164529
Clec12a	1	6.12E-40	5E-10	-0.000390256	0.01711918	0.005162593
Relb	1	1.52E-11	1	0.00052756	0.024955275	0.005158195
Gm11634	1	1.56E-08	3E-07	-0.000618005	0.007145739	0.005157518
Prr11	1	1.00E+00	2E-11	-0.000398326	0.001542031	0.005141497
Ptges3l	1	1.00E+00	5E-05	9.53749E-05	0.000106557	0.005141024
Gm15510	1	2.02E-01	0.0003	-0.000612399	0.00509151	0.005138906
4933401D0	1	1.00E-08	5E-21	8.57749E-05	0.002563394	0.005137495
Rps6ka2	1	2.39E-26	0.0016	-0.000384506	0.022355283	0.005137333
Siglece	1	1.58E-23	8E-12	0.000138633	0.010829861	0.005134388
Bloc1s3	1	2.18E-03	5E-06	0.000910994	0.005654691	0.005132192
Btnl9	1	4.65E-01	1E-06	0.001855635	0.004516996	0.005123882
Gm26861	1	5.95E-04	6E-05	-0.000229215	0.006076355	0.005123813
2610300M	1	5.46E-02	0.0006	-0.000700736	0.005620047	0.005122289
Gm15675	1	2.27E-05	1	0.003079963	0.017220771	0.005118214
Hist1h3e	1	1.00E+00	0.0006	-0.000212125	0.001040455	0.005114304
Ppp1r16b	1	4.80E-13	0.0004	0.000801585	0.012960061	0.005112398
Bmp2	1	6.34E-03	0.0952	0.000296175	0.010310154	0.005110138

Csgalnact1	1	2.81E-02	1	0.001839407	0.011980687	0.005109875
Cenpi	1	1.63E-03	4E-11	-0.000655699	0.003796878	0.005104767
E2f7	1	1.00E+00	6E-07	-0.001186084	8.68613E-05	0.005104478
Zfp82	1	1.00E+00	1E-08	0.001176387	0.001633131	0.005103445
Mgst2	1	1.13E-20	2E-17	3.85936E-05	0.006767328	0.00509014
Shisa5	1	1.67E-19	0.2146	-0.001745812	0.021910644	0.005089541
Gm17545	1	1.13E-02	1	-0.001497296	0.010362225	0.005083032
Gm6225	2.03908E-16	1.00E+00	1	-0.015137864	-0.003969145	0.005078379
Nlrp3	1	5.71E-60	7E-13	0.000260055	0.022688689	0.005073522
1700123M	1	7.63E-09	7E-10	-0.000774487	0.006544058	0.005066258
Wdr54	1	1.00E+00	3E-08	0.000286167	0.001423411	0.005064607
Tmem267	0.599395464	7.01E-17	1	-0.00569984	0.05334349	0.005062278
Sult3a2	1	5.16E-08	3E-11	-0.000206246	0.005477853	0.005061583
Arl6ip4	0.038893849	1.00E+00	1	0.010473339	-0.009305764	0.00506156
Sell	1	1.68E-38	4E-07	-0.000610009	0.019036683	0.005060728
Kif14	1	1.74E-05	2E-16	0.000110815	0.003054281	0.005053597
Gm10754	1	1.00E+00	0.0022	-0.000965228	0.004063232	0.005050931
1700120E1	1	3.51E-12	6E-07	0.002320014	0.008430945	0.005050422
Aunip	1	3.01E-01	1E-11	-7.13902E-05	0.002416866	0.005034731
Ighm	1	8.41E-23	6E-05	0.00059742	0.016149256	0.005021076
Gm33906	1	2.54E-03	0.1718	-0.000557591	0.00793692	0.00501909
Capg	1	6.16E-78	7E-13	-0.000257252	0.026923759	0.005016861
Ptpn18	1	1.14E-09	9E-05	-0.000425	0.009509444	0.005009055
Tmod1	1	5.80E-07	0.06	0.000781228	0.010951121	0.005008177
Stpg4	1	2.71E-01	4E-11	-0.000115961	0.002743831	0.00500717
Ampd1	1	1.00E+00	3E-09	0.000198635	0.00149433	0.005006644
Ryr2	1	1.00E+00	4E-06	0.001249512	0.003532401	0.005003534
F830045P1	1	1.23E-30	6E-16	-0.000159896	0.009718445	0.005001242
Gm45774	1	4.56E-05	2E-16	-7.41207E-05	0.002723861	0.004987067
Acvr1b	4.391E-05	1.00E+00	1	-0.013505969	0.018192656	0.004984808
Rnf157	1	8.08E-06	0.0113	0.000267404	0.009650594	0.004980493
2900092C0	1	8.11E-04	0.0346	-0.00115543	0.008305343	0.004980029
Rpl10-ps3	1	1.34E-04	0.0001	-0.000104846	0.006698459	0.004978862
Rab39	1	1.90E-26	9E-08	0.000399687	0.015260894	0.004976368
Taok3	0.042217041	9.54E-12	1	0.038200577	-0.069572449	0.004971057
B930059L0	1	3.32E-05	3E-07	0.000943959	0.005848569	0.004970699
Pla2g4c	1	1.29E-10	1E-12	-0.000281366	0.005707894	0.004970308
Slc18a2	1	3.01E-03	1	0.000660734	0.010882155	0.004965286
Gdpd1	1	1.00E-19	1E-05	0.000751903	0.013477	0.004959795
Gm26820	0.511911046	2.88E-03	0.0207	-0.002794078	0.007207523	0.004954577
Cds1	1	7.28E-29	7E-12	-8.66333E-05	0.011367837	0.004948747
Ftx	0.001091716	1.00E+00	1	0.021050835	-0.005766806	0.00494242
Gm17114	1	2.87E-03	1	-0.001088866	0.012428656	0.004940866
Gm47849	1	3.99E-23	2E-17	0.000162626	0.006015914	0.004938927
Peak1os	1	6.16E-20	4E-10	0.000302353	0.010502213	0.004935817
Gm47794	1	6.47E-06	3E-07	-6.30785E-05	0.005980456	0.004934627
Apba1	1	1.37E-34	0.0006	-0.001022767	0.024373528	0.004928156

Spata48	1	2.27E-29	1E-11	-0.000248208	0.012068535	0.004926935
Tnfsf14	1	3.50E-11	9E-14	0.000237746	0.005040348	0.004926333
Slc10a3	1	8.08E-03	3E-06	0.001036926	0.0050624	0.00492559
Slc38a2	3.58148E-24	1.00E+00	1	-0.066861767	-0.029771557	0.004924969
Neurl1a	1	6.45E-02	1E-09	-0.00033937	0.003393633	0.00492454
Ccr1	1	1.19E-111	1E-17	0.000214437	0.037627904	0.00492199
Gm15243	1	1.97E-04	0.0045	0.001156884	0.007597712	0.00492016
Egflam	1	1.00E+00	0.0045	-0.000429952	0.002267236	0.00492016
Cd83	1	1.30E-57	2E-10	0.00018152	0.020537618	0.004918748
D5Ertd615	1	1.59E-02	0.0007	-0.000256744	0.005862826	0.004917794
Gm35867	1	1.77E-04	2E-07	-0.000106086	0.005008488	0.004916292
Nat8f3	1	7.27E-56	9E-08	-0.000437437	0.01975592	0.004914843
Rbmx2	1	1.00E+00	6E-07	0.000695558	0.003414485	0.004912731
Pwwp2b	1	1.03E-09	0.8007	-0.000109079	0.014846947	0.004910561
Shroom4	1	3.07E-04	0.0082	0.002541274	0.00953312	0.00490437
Gm21284	1	1.00E+00	2E-06	-0.000565052	0.003004326	0.004892421
Gm10974	1	1.77E-21	0.0002	0.000818172	0.013947539	0.004891879
Gldn	1	1.00E+00	5E-10	6.89264E-05	0.002373645	0.004890575
Actr3b	1	7.10E-03	1E-10	-0.000247354	0.005263163	0.004888502
Prr7	1	1.00E+00	2E-06	-0.000842293	0.003675682	0.004881664
Gm43091	1	4.60E-10	1E-11	0.000244938	0.005595451	0.004881223
Mefv	1	1.55E-58	3E-15	2.95018E-05	0.019824264	0.004879033
Gm41496	1	1.03E-05	8E-11	-0.000320645	0.004971716	0.00485273
Kcnv2	1	3.22E-17	2E-18	0	0.005746893	0.004852608
Psmc3ip	1	1.00E+00	4E-05	-0.00076971	0.003827751	0.004851328
Tlr1	1	1.82E-55	7E-14	-0.00012147	0.017519022	0.004850024
Gm11712	1	1.64E-03	2E-11	0.000116276	0.00343943	0.0048466
Gm45605	1	9.04E-20	6E-13	-6.06623E-05	0.007637684	0.004840009
Klhdc1	1	1.00E+00	3E-05	-0.000229926	0.003763535	0.00483926
Tmem260	0.043225652	1.42E-09	1	-0.022010175	-0.054553484	0.004839224
4921513I0	1	1.30E-10	7E-20	0.000128662	0.002851619	0.004838433
Tnpo1	1	2.99E-24	1	-0.017226409	0.109509859	0.004835809
Ecscr	1	1.17E-20	2E-10	0.000128856	0.009344869	0.004833731
6430573P0	1	1.00E+00	2E-09	3.94974E-05	0.000565794	0.00483321
Adig	1	5.39E-12	0.1908	-0.002784409	0.01305309	0.004829394
Prdm1	1	9.00E-38	9E-11	-0.000536886	0.013551168	0.004827418
A930019D1	1	1.98E-07	0.3511	-0.001227385	0.011255968	0.004823063
Pik3r6	1	3.70E-40	1E-06	0.000201677	0.022606644	0.004822905
Ly9	1	1.13E-68	4E-11	2.77704E-05	0.023701315	0.004822646
Txndc16	1	2.24E-05	1	-0.003246284	0.021141857	0.00482167
Cilp	1	1.68E-03	3E-09	-9.09692E-05	0.003875384	0.004815666
Melk	1	1.15E-01	2E-12	-0.000134856	0.002603687	0.004811845
Mctp1	1	1.43E-26	1	-0.000621034	0.028374694	0.004808065
Plxnd1	1	9.98E-12	1	0.002674434	0.018412588	0.004798963
Trim59	1	1.20E-05	7E-13	0.000322763	0.003641104	0.004793971
Gm5784	1	1.00E+00	5E-05	-0.001436283	0.003085154	0.004792391
Gm46336	1	1.41E-10	7E-11	0.000278902	0.005908126	0.004786524

Gm16196	1	9.38E-01	0.0059	0.000109706	0.005755122	0.00478595
Gm45175	1	1.68E-12	1E-18	0	0.003497292	0.004783362
Trim35	1	3.98E-37	6E-07	0.000225599	0.019042387	0.00478281
Xrcc2	1	4.14E-02	0.0493	-0.000799631	0.006699058	0.004781328
Ttll10	1	1.00E+00	2E-12	-0.000519039	0.002195642	0.004774319
Smim3	1	1.36E-13	1	-0.001476739	0.017909474	0.004753228
Dync1i1	1	1.00E+00	0.0134	-0.001020419	0.000427146	0.004752835
4933424G(	1	1.79E-04	0.3028	0.000146227	0.009572831	0.004751634
Cyp2s1	1	1.82E-05	5E-14	0.00034322	0.003247137	0.004746627
Satb1	1	6.68E-20	0.0541	-0.000510125	0.017962046	0.004746139
Gm26739	1	1.00E+00	1E-05	0.0011602	0.003319116	0.004745883
Gm28286	1	4.61E-24	3E-18	0.000248401	0.0064579	0.004743734
Lrrc7	1	3.29E-06	0.092	0.000231353	0.0097537	0.004743415
Arl9	1	8.48E-07	0.7628	-0.002293473	0.010906104	0.004743198
Lca5l	1	1.03E-03	9E-08	0.000167666	0.004620766	0.004740751
2700033N1	1	4.02E-02	7E-08	-0.000440973	0.00389334	0.004740613
Rap2b	1	1.05E-35	9E-08	0.000546292	0.016590258	0.004738699
Gm48796	1	2.64E-01	3E-06	-0.000110428	0.003777087	0.004734611
Fam89b	0.007355418	1.00E+00	1	0.0059055	0.00376668	0.00472728
Gm11808	1	8.20E-10	4E-11	-2.57247E-05	0.005677411	0.004727107
Smcp	1	1.00E+00	1E-15	-0.000189397	1.42897E-05	0.004726184
Zpbp	1	1.00E+00	0.0074	0.001378751	0.005101209	0.004722315
Bvht	1	1.70E-22	6E-14	0.000119859	0.008260262	0.004721174
Dlx4	1	3.22E-17	4E-19	0.00017155	0.004396578	0.004720388
Gm26766	1	3.41E-13	1E-10	0.000266341	0.00665915	0.004716539
Gm12279	1	1.00E+00	6E-06	0.000445257	0.002366237	0.004714959
E230013L2	1	1.00E+00	2E-05	-0.00035593	0.003384884	0.004708495
Gm20618	1	1.99E-01	5E-05	0.000168354	0.004316666	0.004705263
Crtc3	2.73174E-19	1.00E+00	1	-0.0580126	0.016642406	0.004702592
Cxcl16	1	2.39E-46	9E-09	0.000257686	0.018996183	0.004701506
2810457G(	1	1.87E-03	2E-14	-8.75064E-05	0.002700962	0.004698624
Krt23	1	5.38E-08	1	0.000706975	0.012936981	0.004698453
4833419F2	1	1.61E-11	0.007	-0.000418419	0.011208011	0.004698403
Ocstamp	1	8.72E-01	6E-05	-0.000222048	0.003966803	0.004694644
Fmr1nb	1	1.00E+00	4E-06	0.000668435	-0.000422617	0.004692463
Rhoh	1	7.42E-52	5E-08	0.00010835	0.025224752	0.004691678
Akap14	1	1.83E-18	1E-06	0.001287984	0.009994674	0.004691152
Gm12976	1	7.87E-13	2E-14	0.000224455	0.00486972	0.004676728
Nol3	1	1.00E+00	2E-07	-0.000383941	0.002667551	0.004675067
Itgb4	1	1.00E+00	0.0381	-0.001367968	0.003964053	0.004674376
Il1f9	1	2.80E-100	3E-13	8.57749E-05	0.036243775	0.004671862
Fgd5	1	1.51E-04	0.4434	0.001279856	0.012544778	0.004666241
Rrh	1	1.17E-08	1E-14	0.000199368	0.003851285	0.004662552
Slc7a4	1	7.77E-08	2E-12	0.000173427	0.004279193	0.004658195
Gm34047	1	1.00E+00	2E-10	1.99737E-06	0.002111316	0.004656082
Gm15716	1	4.42E-03	0.0003	-0.001048275	0.005770594	0.004655664
Ly6a	1	2.32E-40	7E-11	-0.00010616	0.014643164	0.004653372

Mmp16	1	1.00E+00	0.0019	-4.32805E-05	0.002755371	0.00465111
Stac2	1	7.25E-02	9E-07	-0.000122492	0.004263526	0.004649471
Slc43a2	1	6.25E-47	1	-0.004136956	0.116723322	0.004648269
Raly	1	1.00E+00	0.0014	0.000687826	0.002510579	0.004642406
4933439K1	1	1.09E-73	2E-12	-0.000198321	0.027027014	0.004639389
Tlr8	1	1.04E-46	4E-10	0.000158285	0.01720677	0.004639337
Dnmt3bos	1	1.00E-08	1E-18	8.57749E-05	0.002444245	0.004636967
Gm28609	1	2.01E-22	2E-14	-0.000211707	0.007467726	0.004627929
Rnasel	1	5.07E-13	2E-05	-0.000386599	0.010160268	0.004626445
Itga11	1	7.31E-03	1E-07	-0.0005108	0.004042764	0.004623365
Tet2	1	4.17E-15	1	-0.009850833	-0.055868146	0.004622964
Cracr2b	1	3.91E-04	0.0031	-0.000305068	0.007402732	0.004621642
Fmo6	1	2.09E-40	1E-15	-0.000189397	0.011298299	0.004614412
Gm37168	1	1.34E-112	4E-14	0	0.036958586	0.004613801
Trem2	1	2.80E-100	8E-17	8.57749E-05	0.025772722	0.004610247
Cxcl10	1	1.03E-07	1	-6.18426E-05	0.011385989	0.004610222
Plcxd1	1	1.00E+00	0.0009	-0.001160965	0.004068945	0.004609237
B4galnt4	1	2.48E-15	2E-18	0.000128662	0.003989204	0.004602344
Gm36888	1	4.86E-09	4E-16	2.0578E-05	0.003429621	0.004601686
Trim5	1	3.80E-13	1	-0.004858574	0.024643309	0.004601359
Ssc4d	1	3.45E-03	0.0007	0.000145191	0.005883711	0.004596378
C130036L2	1	1.93E-04	0.1991	-0.002399973	0.008753572	0.004595296
Emcn	1	2.35E-03	0.0098	-0.000315435	0.007205856	0.004591548
Meig1	1.89036E-08	1.31E-37	0.0258	-0.00400841	0.021969656	0.004586199
Gm13091	1	3.18E-04	0.1057	-0.000875748	0.008778094	0.004586136
Icosl	1	6.23E-03	3E-09	-0.000324133	0.004151079	0.004585712
1700120G1	1	1.14E-04	6E-10	0.000493339	0.004074942	0.004585059
3110045C2	1	1.00E+00	5E-05	0.000341635	0.000105357	0.00457436
Gm36992	1	1.00E+00	1E-05	0.001439835	0.002010904	0.004565524
Cracr2a	1	2.08E-27	2E-06	-0.000409955	0.013623495	0.004563293
Gm12925	1	1.00E+00	7E-06	-0.000227509	0.002985907	0.004560053
Gm15987	1	4.00E-08	0.008	-0.0009803	0.010671411	0.004559698
Gm44949	1	1.00E+00	9E-05	-0.000674206	0.003797057	0.004559521
Gpx7	1	1.00E+00	5E-05	-0.001268437	0.000125009	0.004559389
2310034O0	1	3.01E-08	1E-09	-0.000286681	0.00544278	0.004556566
Impdh1	1	4.73E-04	8E-09	0.000509503	0.003980243	0.004553334
Fxyd5	1	1.62E-45	0.0006	-0.000920924	0.023891157	0.004551908
Gpr176	1	3.30E-04	2E-06	-0.000805624	0.004852151	0.004546536
Ppp1r18	1	1.25E-31	2E-05	-0.000266836	0.017285512	0.004536893
Gm32089	1	1.30E-10	1E-17	0	0.003174456	0.004533098
Fmnl3	1	7.99E-24	7E-06	0.002106909	0.016413472	0.004524309
Gm16741	1	1.00E+00	0.0001	-0.000680256	0.000823711	0.004523449
Sirpb1c	1	1.51E-42	2E-14	5.45894E-05	0.013951287	0.004516998
9330175M	1	1.00E+00	2E-06	0.001361179	0.002717334	0.004516731
Sult2a4	1	1.00E+00	2E-11	-0.000333129	0.000929417	0.004514186
Lrrc46	1	1.00E+00	1E-10	0.000230626	0.001313223	0.004509221
Gm16070	1	1.00E+00	6E-06	2.97684E-05	0.002711972	0.004509147

Gm29087	1	2.58E-17	5E-16	5.45894E-05	0.005069279	0.004509094
Wbp2	1	3.70E-03	1	0.011953415	-0.021861108	0.004509015
Gm10640	1	1.07E-02	1E-08	-0.000159702	0.003579113	0.004508558
Gm31881	1	8.14E-24	2E-18	0	0.005703239	0.004504748
Zfp967	1	6.42E-02	0.0001	-0.000813596	0.004567703	0.004502348
Mrpl1	0.159736726	4.79E-11	1	-0.017389731	-0.048803347	0.004496908
Gm11373	1	3.22E-17	1E-17	0	0.004277429	0.0044843
9430065F1	1	7.99E-03	0.0031	-0.000484495	0.005898212	0.004483938
Matk	1	3.34E-30	1E-08	-0.000377423	0.012047065	0.004478803
C5ar1	1	6.38E-70	6E-14	-4.45712E-05	0.022207348	0.004477323
Slfn1	1	2.12E-54	7E-09	-8.30445E-05	0.023592058	0.004475534
Prex1	1	6.39E-79	1	-0.002657753	0.068745288	0.004470109
Lrrc8e	1	3.41E-09	1E-06	0.00014585	0.006320511	0.004457972
Mylip	1	1.33E-28	1	8.48098E-05	0.036945833	0.004453309
1700065L0	1	9.74E-01	0.0003	-0.000816254	0.004181532	0.004452096
Gm11099	1	5.15E-27	1E-07	0.000238888	0.013116722	0.004450123
Pnliprp1	1	8.86E-08	7E-15	0	0.002240558	0.004448318
Card11	1	5.81E-52	0.8768	-0.000320137	0.038287585	0.004446568
Siva1	1	1.33E-11	3E-07	0.000106619	0.007308002	0.004446369
Tbc1d10c	1	6.56E-05	5E-07	-0.00029629	0.005580767	0.004443329
H2-T24	1	1.57E-34	1	-0.002402342	0.031634683	0.004439193
Plac8	1	2.40E-07	0.0316	-0.001807788	0.011697695	0.004430311
Kcnab2	1	2.43E-46	7E-12	7.96293E-05	0.015137441	0.004425197
Zfp366	1	4.16E-18	0.0061	0.000800322	0.014660281	0.004424453
1700122E1	1	2.55E-04	0.009	-7.00042E-05	0.006853564	0.004424192
Scml2	1	4.56E-05	2E-14	-0.000117008	0.002723861	0.004423566
Gm30934	1	1.00E+00	3E-07	-2.91399E-05	0.002932877	0.004421247
Gm11739	1	8.12E-04	1	-0.002857791	0.009931598	0.004419515
Acvrl1	1	5.72E-02	0.0051	-0.000262084	0.006672629	0.004412782
Gm45538	1	1.00E+00	2E-05	-0.000591066	0.003295336	0.004412203
Gm17088	1	1.00E+00	0.0084	-0.000478085	0.001414981	0.004397285
Hvcn1	1	3.29E-43	1E-06	0.000253982	0.019583256	0.004389996
Zfp677	1	9.60E-06	5E-05	-0.000401764	0.006399543	0.004389648
Hdac7	1	6.94E-14	1	0.001519788	0.017687196	0.004383558
Dscc1	1	7.87E-02	5E-11	0.000271709	0.00294339	0.004379905
Lrrc9	1	2.38E-27	6E-15	0.000393227	0.007550712	0.004378324
Cebpd	0.04933754	1.00E+00	1	0.00693217	-0.001096478	0.004373596
Eml1	1	6.55E-01	0.0143	0.000874565	0.006651068	0.004369646
Scg3	1	7.91E-02	1E-10	-0.000159775	0.002592856	0.004366383
Gnb2	1	1.00E+00	0.0171	-0.000643585	0.004442235	0.004364111
Nr5a2	0.006543024	1.00E+00	1	0.029047994	-0.014166684	0.004359222
Bicdl2	1	1.06E-25	3E-17	0.000214437	0.006229762	0.00435208
Soga1	1	2.46E-56	0.0022	-0.000307728	0.027880368	0.004346401
Slc19a3	1	1.07E-04	7E-13	-0.000254594	0.002832849	0.004343043
Gm44702	1	1.00E+00	0.0088	0.001521341	0.002728344	0.004341429
Armc4	1	4.23E-05	0.0006	-0.00026923	0.005816954	0.004339253
Dusp19	1	3.02E-03	1E-07	-5.25437E-05	0.003828034	0.004337035

Tube1	1.92938E-05	1.00E+00	1	-0.006421285	0.005828567	0.004335451
Stk32c	1	3.93E-03	7E-06	0.00030221	0.004345949	0.004333002
Ap1s2	1	3.04E-24	7E-05	-0.000143466	0.013744408	0.004327849
Rps27rt	1	1.35E-03	0.0547	-0.001246499	0.007065928	0.004326533
Rasgrp4	1	7.06E-18	5E-06	-0.000302724	0.012286222	0.004315602
Cyp2c65	1	1.00E+00	3E-15	-4.10833E-05	0.00084869	0.004314554
Gm26537	1	3.46E-07	6E-09	0.000362067	0.004861993	0.004307098
Ciita	1	1.18E-19	0.1311	-0.000758056	0.019557687	0.004305873
Mboat4	1	1.30E-10	3E-17	0	0.002851619	0.004303282
Fhad1	1	3.30E-11	1	-0.00030172	0.017661571	0.004301
Gm47800	1	1.00E+00	3E-05	0.000196856	0.002630011	0.004294955
Gm13872	1	6.28E-05	2E-11	0.000754247	0.003345965	0.00429279
Gm7568	1	6.79E-04	2E-06	0.000347924	0.004467675	0.004291925
Plch1	1	1.73E-13	7E-14	0.000517428	0.004924654	0.004282176
Il1a	1	1.10E-27	0.0001	0.000862789	0.015799769	0.004273579
Gm12843	1	1.35E-02	6E-12	-9.64301E-05	0.002487114	0.00427024
Pja2	0.070154661	1.72E-15	1	-0.018866239	-0.059308631	0.004264988
Armcx4	1	2.35E-14	2E-09	0.000103816	0.007854844	0.004258162
Gm50431	1	1.00E+00	0.0125	0.000426893	0.002969219	0.004257961
Ufsp1	1	1.00E+00	2E-05	0.000848166	0.002081822	0.004257297
Catsper4	1	3.22E-17	3E-17	0	0.004277429	0.004254484
Srgap1	1	1.81E-04	1	0.001089255	0.013885712	0.004250134
Gm1600	0.012873072	1.06E-03	1	-0.004823891	0.01244097	0.004242627
Plcb2	1	2.52E-69	1E-07	0.000185935	0.025353947	0.004241549
Gm1715	1	3.87E-06	0.0009	0.000532079	0.006832712	0.004240807
Il18rap	1	7.90E-29	4E-08	-0.000105354	0.013788223	0.004240146
Trpc5	1	1.00E+00	0.0344	-0.001339538	0.004261908	0.004239937
A730035I1	1	4.20E-19	1E-15	0	0.004923102	0.004234036
Lpcat2	1	1.10E-83	3E-06	0.000342463	0.031532016	0.004233123
Gm19466	1	1.75E-05	0.3432	0.000352895	0.00876497	0.004226005
Map3k15	1	7.99E-03	1	0.000810791	0.010209213	0.00422391
Tmc8	1	1.83E-15	7E-05	-0.000343591	0.009721409	0.004219771
Clcn1	1	1.00E+00	5E-08	-8.19978E-05	0.001834384	0.004216751
Lilra5	1	3.00E-23	9E-09	8.86258E-05	0.010396009	0.004216614
Ntng1	1	1.17E-02	0.0003	0.000724964	0.004776752	0.00421087
Hist1h2br	1	6.47E-12	1E-15	-8.92378E-06	0.003690818	0.004208583
Prkcq	1	3.34E-05	0.0177	0.00161611	0.009584283	0.004202678
5830411N(	1	1.03E-03	3E-10	-4.46189E-06	0.001939595	0.004201448
Gm15567	1	1.07E-01	2E-05	-0.000277636	0.003667216	0.004200124
Hist1h2ae	1	1.00E+00	3E-06	0.000935672	0.002828226	0.00419564
H2-DMb1	1	1.17E-28	2E-06	0.000173691	0.013965225	0.004191434
Flacc1	1	1.00E+00	0.001	0.000967267	0.001603848	0.004189764
Ccnq	1	1.00E+00	0.0136	0.000807444	0.003073409	0.004181512
Gm13031	1	3.41E-07	9E-07	-0.000560927	0.005573434	0.004180015
Cd59b	6.2672E-16	1.77E-04	1	-0.018576926	-0.019426802	0.004177638
Gm50071	1	6.87E-04	1	0.001258969	0.00809372	0.004174432
Plekha2	1	1.83E-101	0.1319	-0.000398697	0.050046359	0.004172862

Cdh5	1	4.39E-07	0.8885	-0.001455596	0.011089282	0.004170872
E430024I0	0.170730696	4.70E-03	1	-0.012401376	-0.024878762	0.004166779
Gm31227	1	2.11E-17	1E-07	0.000122758	0.00831885	0.004162805
Clnk	1	1.72E-03	0.0003	0.000347022	0.005982216	0.004161144
Lhfpl2	1	8.14E-27	1	-0.002348777	0.02921765	0.004158033
Stard6	1	1.00E+00	7E-06	-0.00048837	0.00239986	0.004141831
Clec1a	1	1.46E-13	0.0001	0.001768718	0.010809276	0.004141035
Slc1a1	1	7.22E-02	3E-10	0.000381598	0.002364718	0.004140077
Mannr	1	1.00E+00	6E-05	-0.000223094	0.002113648	0.00413939
Gm26512	1	1.00E+00	0.0004	-0.000458062	0.002658942	0.004135883
1500011B0	1	1.15E-06	0.0014	-0.000440899	0.007829127	0.004134699
Notumos	1	1.00E+00	4E-07	0.000358531	0.002605912	0.004121394
Gm26852	1	1.00E+00	0.0206	2.10629E-05	0.003623713	0.004118533
Fos	1	1.06E-24	4E-05	-0.000866407	0.012696319	0.004116241
mt-Atp8	1	1.00E+00	2E-05	-0.000230287	0.002204218	0.004115254
Ikzf2	1	2.03E-06	0.001	0.0006948	0.009443533	0.004112999
Gm5129	1	1.00E+00	1E-13	0.001017718	0.000910329	0.004109533
Mcam	1	6.58E-18	3E-08	0.001041026	0.009282875	0.004107739
Lrrc75aos2	1	1.00E+00	0.004	0.000425678	0.001787884	0.004100867
Gm45765	1	3.87E-38	3E-15	-0.000122396	0.009366359	0.004097465
Gm43661	1	3.98E-44	4E-10	-0.000473494	0.017322197	0.004092332
Cd22	1	1.06E-17	0.0039	-0.000855583	0.01150276	0.004090636
Gm2682	1	1.01E-02	1	-0.000595345	0.012021596	0.004090479
H2al3	1	1.30E-10	2E-16	0	0.002851619	0.004087641
Pstpip1	1	2.24E-48	1E-10	0.000218046	0.016267312	0.004083466
Fxyd4	1	1.43E-06	1E-05	-0.000633244	0.005971529	0.004081755
Ankrd55	1	3.08E-25	4E-05	-0.001174737	0.01390106	0.004080493
Gm20707	1	1.00E+00	9E-14	0.000239477	0.001151874	0.004079262
Ampd3	1	1.03E-63	0.0066	-0.000375919	0.030234634	0.004075619
Hgf	5.16077E-29	3.42E-10	1	0.019688319	0.032997356	0.004073489
Havcr2	1	3.19E-44	5E-11	0.000242255	0.0156731	0.004072331
Gm10419	1	3.31E-16	1E-11	7.19234E-06	0.005528131	0.004070882
4930426I2	1	1.00E+00	2E-07	1.11943E-06	0.001905514	0.004068902
Naip6	1	2.78E-59	2E-05	0.000344483	0.022923393	0.004066839
Ankrd37	1	2.35E-06	1E-06	0.000430968	0.005251267	0.004064874
Rpl9-ps6	1	1.05E-02	1E-05	-4.97411E-05	0.004259609	0.004064539
Uba7	1	5.67E-11	1	-0.001415255	0.020315459	0.004054026
5430427M	1	2.33E-11	0.004	0.001108653	0.009036081	0.004052068
Gm13963	1	1.99E-29	1E-12	-5.62731E-05	0.009147003	0.004051093
Rinl	1	5.52E-10	0.0007	0.00020768	0.008571757	0.004049513
Chtf18	1	1.00E+00	7E-08	-0.000645897	0.00182221	0.004048975
Sh3bp1	1	8.62E-23	1E-05	-0.00016841	0.011679983	0.004047653
Gbp2b	1	3.76E-11	5E-11	0.000896764	0.006187277	0.004044634
Gm16302	1	1.00E+00	0.0002	-0.000304408	0.001872773	0.004034072
Gadd45a	1	6.16E-09	4E-05	0.001015349	0.007593965	0.004031649
Nfatc2	1	2.20E-41	2E-09	-0.000216169	0.016456146	0.00403139
Serpina3g	0.266892181	9.12E-12	2E-06	0.00196794	0.008237891	0.004028665

Gm36278	1	1.38E-02	0.005	-0.00069381	0.004866089	0.004027274
Elac1	4.28311E-05	1.00E+00	1	-0.011586882	-0.00848692	0.004024672
Cntnap5b	1	4.44E-05	1	-0.000597894	0.00961461	0.004020529
Tmem160	1	5.01E-04	1	-0.002844191	0.010258656	0.00402041
Htr2b	0.012617391	3.94E-01	1	0.007853627	0.010729306	0.004019984
Gm14022	1	1.11E-03	0.8736	-0.002293572	0.00806055	0.004019068
Hapln3	1	2.82E-16	3E-15	0	0.004073742	0.004018395
Ska3	1	3.20E-01	8E-05	-0.000748473	0.003128518	0.004018173
Snhg4.1	1	2.31E-03	0.0187	-0.000587384	0.006057585	0.004016123
Fam189a1	1	2.24E-07	1	-0.001088117	0.010817848	0.00401032
4930432K2	1	1.15E-09	0.0001	-0.000498172	0.007345297	0.004009278
Dap3	1	8.62E-05	1	0.006902602	-0.030321689	0.004006865
4930579K1	1	6.99E-01	1E-05	0.00030221	0.003446663	0.004005589
4632411P0	1	5.12E-01	7E-07	8.78683E-05	0.002976098	0.004001901
B430219N1	1	1.00E+00	0.0005	0.000145045	0.002738999	0.003998791
Prrc1	1	6.06E-05	1	-0.001351299	-0.030281151	0.003996937
C030037D0	0.024523302	1.00E+00	1	-0.006741285	0.001827197	0.00399668
Rerg	0.005013634	1.00E+00	1	0.004901392	0.002207494	0.003989105
Slc4a10	1	1.00E+00	4E-05	-0.000195955	0.003350341	0.003988234
Rac3	1	1.00E+00	0.0003	0.000230868	0.002928397	0.003986723
Zmynd15	1	2.24E-12	0.6228	5.86575E-05	0.013911964	0.003985567
Tmem40	1	1.06E-04	2E-11	-0.00015363	0.003400898	0.00398166
Lrrc25	1	4.78E-34	5E-08	-0.000338565	0.012344214	0.003979735
Sap30bpos	1	9.28E-01	0.0007	-0.000168553	0.003615387	0.003974787
Gm29707	1	7.06E-08	2E-10	9.57454E-05	0.00430756	0.003972627
Dlgap4	1	2.03E-02	1	-0.006038965	-0.021888623	0.003965199
Slco3a1	1	1.56E-39	1	0.00047758	0.052968177	0.003964789
Il16	1	1.40E-15	0.0362	-0.000111233	0.011809722	0.003963824
Igfbp7	1	5.00E-06	1	0.002368115	0.024499233	0.003962494
Gm19325	1	1.84E-40	1E-07	0.000109131	0.015042	0.003962144
Ipcef1	1	6.05E-12	0.0042	-0.000129322	0.012530946	0.003961345
Galnt6	1	2.84E-67	3E-05	-0.00057417	0.025711945	0.003953101
Lrrc27	1	1.65E-42	0.0022	0.000683904	0.021847833	0.003951962
Cd244a	1	7.96E-49	1E-09	0.000262881	0.017673641	0.003947969
Zbtb32	1	8.48E-04	3E-06	-0.000374187	0.004323613	0.003936677
Gm42851	1	2.74E-15	4E-13	0.000277903	0.004855431	0.003934316
D930030I0	1	6.93E-01	0.0004	-0.000724166	0.003524248	0.003932447
Ifitm1	1	1.01E-17	0.0023	-0.000334342	0.013341315	0.003931047
Trim30b	1	2.02E-61	1E-06	-3.83528E-05	0.025294757	0.003921274
Dppa5a	1	9.30E-25	1E-15	0	0.005906926	0.003920799
Cacng1	1	7.82E-07	1E-15	0	0.002036871	0.003920799
Platr8	1	5.36E-08	1	0.00041992	0.012398568	0.003918269
AI662270	1	1.98E-39	3E-08	-9.36044E-05	0.01410782	0.003911284
Cers4	1	1.46E-08	1	0.001078682	0.011307399	0.003907105
T2	1	3.60E-18	6E-10	-9.45783E-05	0.008856009	0.003901137
Far1	1	1.77E-39	1	0.001001218	0.028070963	0.003889216
Ccdc91	0.001545687	1.00E+00	1	-0.020751338	-0.003392996	0.003888398

Gm47126	1	2.91E-05	0.0001	-0.000453648	0.005606812	0.003888336
1700056E2	1	5.78E-04	7E-09	0.00022175	0.003241105	0.003887752
Treml4	1	8.31E-72	8E-10	-2.67713E-05	0.024536095	0.003887102
Atg10	0.021443657	1.00E+00	1	-0.023923946	-0.010748971	0.003885686
Pira2	1	6.65E-54	4E-06	0.000151971	0.020629047	0.003884519
Gm35154	1	1.40E-32	4E-07	0.0005175	0.013989835	0.003882929
Fam171b	0.002932975	1.00E+00	0.8797	0.004755448	0.004374674	0.003879138
Flt4	1	3.82E-06	1	0.001841837	0.014733964	0.003878964
Zfp219	1	9.21E-03	1	0.002466786	-0.024984403	0.003878456
Pla2g4f	1	1.00E+00	6E-08	-0.000631585	0.001792397	0.003877923
9930014A1	1	1.82E-05	5E-11	-8.30445E-05	0.003036536	0.003877138
Hps4	8.32463E-05	1.54E-01	1	-0.020260885	-0.023202113	0.003872552
Mnt	0.14765606	1.32E-09	1	-0.009811905	0.03422768	0.003872003
Ydjc	1	1.00E+00	0.0065	-8.89712E-05	0.003705605	0.00387105
A830082K1	1	1.00E+00	0.0065	0.000239864	0.003006564	0.003869464
Ptafr	1	4.86E-88	4E-07	-4.72766E-05	0.031881129	0.003868965
Stat4	1	1.77E-05	1	-0.000848245	0.011307629	0.003865178
Pax9	1	1.97E-07	0.0003	-0.000722554	0.00636338	0.003861429
Gm14634	1	1.50E-04	1	-0.004342035	0.016796961	0.003859681
4930579F0	3.53967E-09	1.00E+00	1	-0.008814403	0.002529757	0.0038591
Sarm1	1	9.70E-06	0.1845	-0.000464963	0.00779159	0.003855209
4930556M	2.07018E-24	1.00E+00	1	-0.030545394	0.002303631	0.003851188
Gm15713	1	5.68E-05	1	-0.001428532	0.00967795	0.0038497
4930470O(	1	1.00E+00	0.0018	-0.00025879	0.003160663	0.003844016
5033421B(	0.175639982	4.61E-38	1	-0.005952603	0.06159167	0.003838469
Olfr1392	1	1.00E+00	0.0017	-0.001176444	0.002382535	0.003827787
6430562O1	1	7.15E-02	8E-05	0.001114414	0.003686868	0.003823117
Cfap221	1	1.00E+00	2E-10	-0.000177743	0.00143149	0.003817062
Churc1	1	7.29E-03	2E-05	0.000493412	0.004161914	0.0038096
Mcf2l	1	3.79E-08	1E-06	-3.11605E-05	0.005562599	0.003808677
Gm44877	1	2.46E-04	0.0061	0.000364065	0.005948948	0.003807939
Gm4566	1	1.32E-01	8E-05	-0.000451916	0.003993508	0.003806231
Pacsin1	1	1.00E+00	0.0009	-0.000490102	0.003461936	0.003805758
Ska1	1	1.23E-03	3E-13	-8.92378E-06	0.001976784	0.003805653
1700084E1	1	6.50E-03	0.0338	0.001174705	0.006166112	0.003805163
9330162B1	1	1.24E-30	2E-11	-0.000117008	0.008630787	0.003804994
Cmklr1	1	7.97E-86	7E-06	6.53901E-05	0.035490297	0.00380092
Gm34961	1	2.66E-03	1	-0.001052322	0.010909947	0.003796023
Micall2	1	5.86E-01	0.0001	-0.000902055	0.003425209	0.003794624
Gm12364	1	1.00E+00	0.0075	-0.001096767	0.002234561	0.00379231
Gm16133	1	1.00E+00	3E-06	-0.000488611	0.001762584	0.003791924
Gm50146	1	1.00E+00	0.0004	-7.03185E-05	0.002826322	0.003789285
Pax5	1	1.06E-09	0.5782	-0.000687569	0.012410515	0.003786888
A930029G2	1	1.20E-06	0.0061	0.001660586	0.006995081	0.003786839
Gm26510	1	1.41E-48	0.3136	-0.000579954	0.024710591	0.003783655
Rpl3l	1	1.60E-02	0.0104	-0.000346224	0.005205346	0.003774435
Tmem132c	1	9.25E-01	0.0032	-0.001026302	0.003734536	0.003773322

Akna	1	9.32E-37	0.0016	-0.000383111	0.018103248	0.003772868
Depdc1a	1	1.86E-03	5E-08	2.33561E-05	0.003023799	0.003766951
Cyp4f18	1	1.09E-29	8E-08	-0.000405566	0.012796767	0.003766339
Gm42903	1	3.35E-03	5E-07	-0.000484955	0.003445674	0.003766146
Gm45708	1	6.01E-03	3E-06	0.000604154	0.003562708	0.003762914
Pafah1b3	1	1.00E+00	0.033	4.95657E-05	0.001887131	0.003757023
AI429214	1	9.60E-03	2E-10	0.000621171	0.002350428	0.003753293
Kcnj14	1	1.28E-09	4E-07	-0.000188398	0.005428952	0.003749397
Gm45016	1	1.00E+00	0.0011	-0.000789508	0.002819408	0.003747737
Gm26620	1	1.00E+00	0.0014	-0.000340055	0.001479688	0.003747078
Rapgef3	1	3.30E-01	0.0034	0.000203433	0.00413732	0.003746099
Cdkn2c	1	1.00E+00	0.0218	-2.26323E-05	0.002338858	0.003741729
Magee1	1	1.13E-02	0.0042	0.000443669	0.004666882	0.003739357
Fam102b	1	1.36E-08	0.0609	0.000406026	0.009076263	0.003738556
C78197	1	3.58E-02	0.0022	0.000448083	0.004559445	0.003735801
Map10	1	2.11E-05	2E-09	3.22322E-05	0.003301863	0.00373509
Gm35996	1	1.00E+00	0.0006	0.000686466	0.002208347	0.003733561
Nudt16	1	3.46E-02	1	-0.00033221	0.007056616	0.003733513
Cyp2b19	1	1.81E-29	4E-14	0	0.007282809	0.003733509
D630003M	1.44352E-07	1.00E+00	0.5377	-0.004077214	0.0003179	0.003731485
Snhg4	1	3.10E-07	1E-12	0.000582577	0.002865909	0.003731401
Efcab9	1	1.61E-03	2E-05	0.000944596	0.003956675	0.003726837
Gm16764	1	5.44E-04	2E-14	0	0.00142581	0.003719333
Emp1	1	6.58E-48	2E-10	-0.000103622	0.016344532	0.003718857
Cyb5r2	1	6.79E-04	8E-05	-0.000237311	0.004559127	0.003714901
Tspan3	1	1.00E+00	0.0007	-0.000236313	0.003899085	0.003709748
Gm2245	1	1.38E-56	3E-09	5.45417E-05	0.019081745	0.003707533
Kcnh7	1	3.64E-11	1	-0.001714795	0.011096661	0.003705231
Gpx3	1	3.64E-08	6E-12	0.000293093	0.003793983	0.003705153
AC103362.	1	1.00E+00	0.0066	-0.000786825	0.003460601	0.003701968
Gm17660	1	2.08E-03	1	-0.000974424	0.009240831	0.003696496
Ankrd22	1	5.92E-01	0.0449	-0.000639268	0.004961314	0.003695828
Gm12484	1	1.00E+00	0.0001	0.000164624	0.001976081	0.00369525
Cxcr2	1	3.56E-19	7E-10	7.23893E-05	0.007683263	0.003682785
Slc7a7	1	3.62E-06	1	-0.001624735	0.02242776	0.003682255
Edaradd	1	3.35E-10	6E-08	2.77704E-05	0.005276069	0.003681468
Dpysl3	1	5.15E-03	0.1753	-0.000109286	0.008076222	0.003680725
E230032D2	1	1.10E-63	5E-05	-0.000441261	0.025173354	0.003678838
Pabpc2	1	4.31E-02	3E-13	0.000128662	0.001018435	0.003676943
Adcy3	1	4.52E-03	0.0275	0.00044272	0.005838265	0.003675714
Plxdc1	1	1.32E-41	1	-5.66605E-05	0.034889889	0.003669538
Bhlhe41	1	4.74E-25	0.0002	-0.000793285	0.01156177	0.003667561
Nat8f6	7.76991E-11	1.44E-73	1	-0.008673097	0.050334177	0.003667071
Gm12195	1	2.67E-04	0.0007	0.001124264	0.005070127	0.003661417
Ms4a4b	1	3.47E-19	0.0739	0.000510718	0.012563869	0.003649526
Xpnpep2	1	3.64E-08	1E-11	-0.000150972	0.003345083	0.003649429
Gm15612	1	1.54E-08	0.0004	-0.000659161	0.006481647	0.003649349

Il18r1	1	5.60E-06	0.0003	0.000610347	0.005533214	0.003646369
Wnt9b	1	4.14E-09	2E-11	1.61161E-05	0.003705108	0.003636703
Gm10399	1	9.63E-02	2E-11	-0.000155434	0.001752775	0.003636703
Cryab	1	1.07E-01	0.0004	0.000355164	0.003667216	0.003636623
Gm11695	1	6.13E-05	4E-14	0	0.001629497	0.003635912
Fam81b	1	1.86E-17	0.0001	-0.000465302	0.0093164	0.003635832
Itpka	0.000482555	1.00E+00	1	-0.003770495	0.00156299	0.003633215
C2	2.00397E-47	1.00E+00	1	-0.052793185	0.008361347	0.003628481
Disc1	1	1.45E-34	2E-05	0.000315691	0.015785671	0.003622974
Zfp449	1	1.32E-01	0.0012	-0.000124692	0.003996297	0.003620198
P2rx6	1	2.82E-16	3E-13	0	0.004073742	0.003615465
Cfap70	1	2.34E-07	8E-08	-0.000105354	0.004141062	0.003605768
Gm45509	1	4.23E-06	1	0.004909544	0.01437602	0.003599359
Tra2b	1	4.97E-02	1	0.022300257	-0.028125683	0.003591495
A030001D2	1	1.00E+00	1E-10	0.000379841	0.001298051	0.003589353
44447	1	5.25E-38	0.0314	-0.000393526	0.020092327	0.003588
Gm47204	1	3.22E-17	4E-14	0.000428875	0.004277429	0.003587114
4930430A1	1	4.80E-04	2E-05	0.00025213	0.00419822	0.003584795
Grm6	1	1.14E-09	6E-13	0	0.002647932	0.003580842
Tgm3	1	3.43E-22	1E-10	-9.46987E-05	0.010646495	0.003579197
Gm29051	1	1.00E+00	0.0012	-0.000333909	0.003033256	0.003577996
Gm5420	1	8.90E-14	6E-13	-9.46987E-05	0.004217342	0.003575836
Clec4a2	1	1.12E-31	4E-09	0.000187666	0.010941973	0.00357536
Raet1e	1	2.03E-32	0.0031	-2.11424E-05	0.015528479	0.003571454
Fabp5	1	1.31E-24	0.0171	-0.00084744	0.016213722	0.00357073
9830144P2	1	8.26E-02	2E-07	0.000434336	0.002610392	0.003569038
BC053393	1	5.53E-04	0.0013	-0.000381211	0.004804801	0.003567377
Gm14104	1	2.78E-03	6E-11	-2.67713E-05	0.002279298	0.003553281
Slc22a12	1	3.68E-18	1E-13	0	0.004481116	0.003552491
Best3	1	1.00E+00	1E-13	0	0.000203687	0.003552491
Rgs11	1	8.25E-29	4E-08	6.18293E-05	0.009295756	0.003548458
Cyp2c66	1	9.26E-05	1	0.000947369	0.009734226	0.003547401
Gm26571	1	1.00E+00	0.0005	0.000371989	0.001409122	0.003536782
Prkx	1	1.13E-03	1	-0.001919606	0.012876192	0.003534557
Gm15747	1	2.95E-08	0.0023	-0.000277636	0.007149194	0.003532754
Gm49763	1	1.76E-01	0.0086	0.000129049	0.004114213	0.003529983
Dlg4	1	8.39E-01	0.0016	-0.00046345	0.00383285	0.003516992
Oca2	1	3.26E-01	0.043	0.000816127	0.004279478	0.003501293
Hbb-bs	1	1.95E-11	1	-0.00452617	0.022581789	0.00349788
Nanos3	1	3.97E-08	8E-12	2.95018E-05	0.002913258	0.003493864
A930014D0	1	4.71E-46	9E-08	-8.56545E-05	0.015960938	0.003489036
Hist1h3d	1	2.70E-10	7E-07	-0.000242724	0.005193438	0.003484821
Tnfsf13	1	3.09E-03	0.005	-0.000836857	0.005796736	0.003484746
Pnpla8	0.905079039	1.52E-15	1	-0.020628835	-0.075737437	0.003482976
8430419K0	1	3.72E-12	0.0001	0.000584718	0.006965302	0.003480926
Ccl4	1	5.25E-37	9E-12	0.00017155	0.010903233	0.003476973
4930548K1	1	2.28E-01	0.0048	0.000334443	0.003743145	0.00347202

Lxn	1	6.46E-03	0.0015	-0.000577801	0.00417945	0.003471888
Fgfr2	3.83979E-34	2.04E-68	1	0.079941743	0.203340626	0.003468123
Gm28809	1	1.00E+00	0.0014	-0.000725285	0.001935645	0.003467536
Il7	1	1.00E+00	3E-08	-8.19985E-05	0.000618187	0.003463258
Gbp2	1	4.85E-13	0.0003	0.000328982	0.00936603	0.003460899
G730003C1	1	1.00E+00	0.0001	0.000328982	0.001540127	0.003455473
4930524O0	1	6.85E-04	0.4948	-0.000865262	0.006683818	0.003454856
Gm10614	1	4.49E-03	0.1058	-0.000649166	0.005321694	0.00345249
4932414N0	1	2.03E-02	0.0272	0.001014449	0.004767261	0.003451705
5033430I1!	1	1.04E-02	3E-07	0.00020656	0.002861429	0.003449545
Gm36640	1	1.45E-38	1E-08	-9.19683E-05	0.011804217	0.003448754
A830008E2	1	1.00E+00	3E-05	-0.000204515	0.002607815	0.003443273
Gm12043	1	2.47E-04	0.0001	-0.000233017	0.004463546	0.003442746
Gng3	1	1.00E+00	0.0275	0.000160235	0.003560662	0.003435943
Epha5	1	2.99E-02	0.3545	-8.35103E-05	0.005535861	0.003435868
Sp110	1	9.39E-32	0.4508	0.000700212	0.021864044	0.003430647
Gm5524	0.00422736	1.00E+00	1	-0.013534711	-0.012414207	0.003428781
Tnfrsf22	1	1.23E-03	9E-12	0.000573701	0.001976784	0.003423169
Gm16401	1	4.20E-19	6E-13	0	0.004684803	0.003420271
Catsperg1	1	4.36E-24	2E-05	0.000277976	0.011360868	0.003417024
Shc4	1	1.00E+00	0.0067	-0.000252549	0.001292194	0.00341471
Gm26801	1	1.00E+00	0.0181	-0.001119245	0.002998395	0.003414375
Dnmt3aos	1	2.97E-27	1	-0.001527791	0.027441491	0.00341155
Rbpms	2.473E-66	5.02E-08	1	0.115579013	-0.067660516	0.003409636
Clec4a3	1	1.11E-20	0.1605	-0.000796507	0.014274382	0.003409368
Slc26a3	1	1.00E+00	4E-08	0.00031023	0.001526189	0.003404961
Radx	1	1.00E+00	5E-08	-0.00043953	0.001672366	0.003401405
Gm48399	1	4.16E-04	6E-06	-8.29717E-05	0.00361542	0.003400741
Gm21188	1	7.66E-87	4E-09	-1.33857E-05	0.030455963	0.003397898
Ttc30b	1	1.00E+00	0.0022	0.00158454	0.001758986	0.003394733
Clba1	1	4.13E-04	1E-05	-0.000373286	0.003881416	0.003393816
Cxcl2	1	3.26E-53	9E-07	8.41388E-05	0.022414618	0.003388473
Gimap6	1	2.22E-09	0.5373	0.000157539	0.011260629	0.003386003
Gm44933	1	2.48E-15	6E-13	0	0.004819599	0.003385648
Orm2	1	2.34E-16	1E-05	-0.00026074	0.007935714	0.003385258
Mrgpre	1	1.30E-10	2E-07	-0.000281366	0.005231297	0.003377401
Slc9b1	1	1.31E-22	6E-05	-0.000760223	0.00926693	0.00337627
C530008M	1	5.06E-04	0.4493	-0.000455211	0.006483056	0.003373942
Gm26850	1	1.00E+00	0.0005	-6.14674E-05	-0.000544093	0.003373501
Prkar1b	1	4.81E-02	1	0.001597751	0.007480713	0.003373053
Lpcat1	1	1.38E-21	0.0009	0.00022911	0.011460577	0.00337046
Gcnt1	1	6.82E-45	8E-05	-0.000187052	0.019515187	0.003370328
Kcnk1	1	1.00E+00	2E-08	-0.000189397	0.000540813	0.003369502
Arhgef4	1	2.42E-04	0.0058	5.58132E-06	0.005434841	0.003365596
Gnat2	1	4.71E-09	3E-05	0.000252178	0.005490591	0.003357086
Sult2a6	1	1.00E+00	8E-10	-0.000112546	0.000734339	0.003351816
Gm48341	1	1.21E-26	2E-12	0	0.006552599	0.003351025

Cd300ld	1	2.01E-25	1E-05	-0.000195591	0.011154201	0.003350681
Gm10847	1	2.75E-08	7E-05	0.000346829	0.005515042	0.003345808
Col4a2	7.56711E-07	1.00E+00	1	-0.013619915	-0.009799769	0.003343318
Alox12	1	1.00E+00	0.0041	0.00066774	0.002906731	0.003336765
Gm13274	1	5.83E-02	0.0005	-0.000461767	0.003474924	0.003335184
Gm32950	1	1.00E+00	0.0434	-0.00069108	0.003173821	0.003333989
4930544I0	1	1.37E-02	1	-0.001367332	0.006038933	0.003333922
Orai2	1	6.99E-20	0.0246	-3.62118E-05	0.013181294	0.0033326
D630023F1	1	2.02E-12	2E-07	1.44323E-05	0.005696181	0.003332159
Ankrd66	1	2.07E-30	9E-12	0	0.010062218	0.003330578
Inka2	1	1.22E-05	0.0002	-0.000402836	0.005632971	0.003326019
Foxm1	1	2.84E-03	1E-07	-9.64301E-05	0.0029291	0.003317984
Bcl11a	1	4.39E-05	1	-0.000245741	0.009567433	0.003316626
Nfkbid	1	3.48E-51	1E-07	0.000356606	0.017802038	0.003311574
Fancc	3.27029E-09	1.00E+00	1	-0.024118945	0.000316984	0.003309
Gm8113	1	2.25E-24	1E-06	-0.000161579	0.009384423	0.003298985
Il15	1	2.35E-14	1	-0.000742308	0.038555114	0.003297748
Gm33251	1	8.43E-03	0.9711	-0.000587505	0.007053597	0.0032943
Cfap157	1	7.78E-09	0.0003	-0.000421489	0.005772322	0.003293311
Adcy4	1	2.33E-06	0.0058	0.000877715	0.006005483	0.003293122
Rtkn2	1	8.68E-10	2E-06	-0.000108012	0.00502694	0.003291077
Gm26655	1	2.70E-01	0.0015	-0.000298214	0.003292902	0.003289421
H19	1	1.00E+00	3E-08	0	0	0.003286693
Olfr1442	1	1.25E-07	0.0982	-0.001513086	0.007164157	0.003286311
Klhl23	1	1.00E+00	1E-05	0.00041834	0.000726612	0.003281248
Shank3	1	3.74E-04	1	0.002055447	0.013627731	0.003276842
Il17rd	1	1.00E+00	0.0009	5.12469E-05	0.002038953	0.003275108
Ccdc68	0.006934491	1.00E+00	0.6121	0.003894991	0.003111954	0.003273706
Clec2i	1	9.46E-23	4E-06	-4.72766E-05	0.009974345	0.003272736
Gm10865	0.006315931	1.00E+00	1	-0.005555465	0.000865635	0.003269508
D630036H	1	1.35E-02	1E-07	0.000375332	0.002487114	0.003269185
Gm45949	1	5.44E-04	4E-12	0.000128662	0.00142581	0.003267604
Cd226	1	5.05E-04	0.7778	-0.000734088	0.007094325	0.003266991
Asxl3	1	1.00E+00	0.0041	-0.000265887	0.002085069	0.003266866
Maged2	1	1.00E+00	0.0157	9.5181E-05	0.001651549	0.003266861
Gm17749	1	1.97E-97	2E-06	-0.000125932	0.036896281	0.003266469
Gm4285	1	3.89E-03	1	-0.002884854	0.00963236	0.003264955
B130046B2	1	1.42E-06	0.0009	0.000229821	0.005268134	0.003264626
Fcrl1	1	3.20E-24	3E-07	-2.05053E-05	0.009421078	0.00325777
Gm44658	1	1.00E+00	0.0005	-0.000721749	0.002274005	0.003256581
Gm29797	1	1.00E+00	0.0046	-0.000560001	0.00059762	0.003256247
C78859	1	6.51E-02	0.0001	-0.000694978	0.003099939	0.00325579
Myh6	1	3.22E-17	4E-12	8.57749E-05	0.004515728	0.003253429
Actl9	1	1.00E+00	0.0016	9.95225E-05	0.00272919	0.003253349
Rnf182	1	1.54E-11	0.0001	0.000403102	0.006149002	0.003251768
Gm31479	1	5.50E-11	2E-11	-9.46987E-05	0.003367982	0.003242151
Gm16251	1	1.00E+00	0.0003	-0.000935333	0.002626234	0.00324049

Amd2	1	1.00E+00	0.0357	0.000127293	0.002928397	0.003235932
Ahsa2	1	6.85E-10	1	-0.002540303	-0.030967833	0.003233833
Pdk3	1	4.48E-10	3E-06	-0.000527962	0.006021256	0.003233108
Cd209c	1	9.67E-11	0.641	0.001044997	0.009210837	0.003232163
Plk1	1	1.18E-02	0.0004	0.000342415	0.003416532	0.00323132
Recql5os1	1	1.00E+00	0.0043	0.000313912	0.001725044	0.003230794
Cccd201	1	4.31E-02	4E-12	0	0.001018435	0.003218806
Ubqln4	0.000325242	1.00E+00	1	0.016923838	-0.015423784	0.003215358
Itpr3	1	8.20E-11	0.2063	-0.001237914	0.010958663	0.003204997
Atp23	0.003229975	1.00E+00	1	-0.008812564	-0.001965232	0.003203495
C030014I2:	1	2.36E-04	0.0001	-0.000669084	0.003908444	0.00320297
Crtap	1	2.60E-10	0.0071	-0.000416954	0.007009616	0.003197949
Mas1	1	1.00E+00	0.0017	-0.000923679	0.001768265	0.003193932
Slc25a24	1	1.06E-13	0.0004	-0.00024021	0.007982517	0.003182522
Plaur	1	1.75E-48	0.0003	-0.000103502	0.01901887	0.003178204
2900060B1	1	1.60E-04	1	-0.001570726	0.008772469	0.003176609
Tifab	1	8.92E-41	2E-06	3.67418E-05	0.013016032	0.003172383
Tmem156	1	2.84E-41	1E-05	-0.00026798	0.016173406	0.003171725
Gm29374	1	6.24E-22	4E-12	0	0.005856999	0.003170008
C230035I1(	1	1.00E+00	0.0001	0.000536227	0.002445445	0.003166898
5430427N1	1	1.00E+00	7E-06	-0.000676277	0.00204668	0.003164658
Gm26811	1	1.80E-16	0.0216	-0.000415175	0.010095512	0.003161548
Myzap	1	3.48E-03	0.0226	0.000623264	0.004834614	0.003160752
Gpr55	1	1.00E+00	0.0005	-0.000761979	0.001910999	0.003154166
Ppp1r18os	1	1.06E-06	4E-08	-4.90331E-05	0.00396528	0.0031518
Mtcp1	1	1.00E+00	0.0121	-3.36494E-05	0.002085069	0.003148822
Rdh9	1	4.63E-06	1	0.001096689	-0.019461159	0.003146108
Mcoln2	1	1.88E-40	7E-06	-0.000286754	0.014322023	0.003142624
Upp1	1	1.13E-09	0.0279	0.000465835	0.0079771	0.003140771
Itgax	1	3.43E-73	3E-07	9.03572E-05	0.022463442	0.003139726
Fxyd3	1	4.83E-03	9E-12	8.57749E-05	0.001222123	0.003135385
Gm49957	1	7.21E-12	1E-08	-2.49671E-05	0.004434648	0.003133272
Usp48	0.000281578	1.66E-05	1	0.028024967	-0.035467326	0.003131477
A430057M	1	5.09E-26	3E-08	0.000307405	0.008126137	0.003124898
Il17d	1	1.00E+00	1E-06	7.27615E-08	0.001189945	0.003124892
Gm2117	1	3.03E-04	0.0187	-0.000307114	0.004731199	0.003124351
Gm13212	1	2.79E-26	5E-05	0.000104622	0.010888591	0.003120865
Skida1	1	1.00E+00	8E-05	0.000364724	0.000886197	0.003120207
Cap2	1	1.00E+00	0.0294	-0.00027137	0.000489514	0.003111296
Gm13381	1	1.93E-05	4E-05	0.000382524	0.004060653	0.003106031
Cpne7	1	2.91E-01	4E-08	-3.12332E-05	0.001824575	0.003103001
Jakmip1	1	7.19E-03	1E-06	-0.000388524	0.003149192	0.003097195
Gm31243	1	6.92E-04	0.3654	-0.000430147	0.006785603	0.003092094
Dynlt1f	1	2.55E-05	0.3471	-0.00050897	0.006606897	0.003085832
Gm16286	1	1.27E-09	0.0001	-1.68485E-05	0.005667251	0.003082027
Olfr46	1	4.00E-01	0.008	-0.000131126	0.003245553	0.003075229
Scimp	1	4.64E-26	3E-05	-5.35427E-05	0.009550921	0.003061448

Ighd	1	1.50E-16	0.3028	-0.00022912	0.013748198	0.003060242
Adrb1	1	3.08E-07	7E-06	0.000235183	0.004049148	0.003059203
Tnc	1	2.30E-08	2E-07	-0.000119618	0.004106658	0.003054856
Cd276	1.27565E-07	1.00E+00	1	-0.00779664	0.001106541	0.003054114
Gm43388	1	3.77E-06	0.0004	-5.43485E-05	0.004276616	0.003049399
Gm20546	1	7.62E-08	2E-07	-4.0157E-05	0.003647598	0.003042925
Slc5a10	1	1.00E+00	0.0095	-6.59293E-05	0.002583013	0.003041264
Cysltr2	1	2.57E-07	0.0028	0.000746465	0.005825348	0.003040336
Gm27019	1	1.31E-04	0.003	0.000317448	0.004473037	0.003034014
Ptger2	1	4.07E-23	0.001	0.000506772	0.010709355	0.003032574
Dnah17	1	1.30E-12	0.041	0.000388936	0.009166446	0.003031897
Gm49694	0.350660216	1.85E-10	3E-07	0.001413555	0.004653295	0.003030199
Gm16036	1	1.00E+00	1E-06	0.000171623	0.000986258	0.003027296
Gm40645	1	4.86E-44	1E-04	-0.000334103	0.016258299	0.003026029
A930028N(	1	1.00E+00	0.0017	0.000635508	0.001403441	0.003021946
Gm31630	1	1.00E+00	8E-06	-0.000105354	0.001781354	0.003021819
Tinagl1	1	1.94E-05	0.5085	0.001397163	0.006997405	0.003017245
Trim30a	1	7.26E-45	1	-0.002027324	0.100852375	0.003011891
1700020N(	1	4.38E-13	1E-10	-4.73494E-05	0.003857317	0.003004614
Cacna1e	1	1.63E-02	1	0.00040249	0.005647986	0.002989351
Zfp459	1	1.00E+00	7E-05	-0.000183889	0.002203018	0.002985089
Cd200	1	1.04E-17	0.0006	0.000522936	0.00832019	0.002980266
Gm48689	1	1.00E+00	2E-05	0.000447721	0.002056841	0.00297447
Rgs10	1	5.80E-36	1E-06	-1.50695E-05	0.012037362	0.00297368
Btbd16	1	1.12E-02	0.0003	-4.44985E-05	0.00406499	0.002973016
Mns1	1	5.81E-03	3E-07	-7.85826E-05	0.002269137	0.002972231
Ugt1a9	1	1.07E-06	9E-08	1.16542E-05	0.003250384	0.002970782
Gm10791	1	5.64E-10	2E-08	0.000144779	0.003908795	0.002969333
Gm38325	1	8.86E-08	5E-11	0	0.002240558	0.002968542
4921528I0	1	1.06E-02	0.0017	0.000728354	0.003753658	0.002964774
Akr1b8	1	4.29E-19	0.0008	-0.000350809	0.009883776	0.002963187
Gm2479	1	1.00E+00	0.0002	-3.11605E-05	0.001707859	0.00296029
Gm10479	1	1.07E-04	4E-07	0.000224455	0.002832849	0.002959504
Lypd6b	1	9.09E-21	1E-04	-0.000119666	0.011484066	0.002955288
Gm48984	1	1.00E+00	0.0056	0.000886519	0.002558563	0.002954945
C030013G(	1	8.88E-12	2E-09	1.17019E-05	0.003728007	0.002944538
Krt25	1	1.00E+00	2E-09	-0.000142048	0.000469013	0.002944538
Nsrp1	0.014138736	5.85E-01	1	0.020340312	-0.020476401	0.002942138
Gssos2	1	2.89E-01	0.0002	0.000468299	0.002515694	0.002939189
Gm13054	1	1.35E-02	2E-06	0.000160895	0.002606263	0.0029355
Ren1	1	1.00E+00	6E-08	-0.000155434	0.000530652	0.00293471
Ccdc80	1	2.27E-03	0.4726	0.000751999	0.005924706	0.002934619
BE692007	1	2.95E-07	0.0567	-3.00661E-05	0.007312298	0.002933702
Gm34184	1	5.19E-18	1E-08	-2.23094E-05	0.00570479	0.002933261
Tm4sf19	1	1.37E-03	0.0157	-0.000110453	0.004728873	0.00293239
Trim9	1	3.35E-24	4E-07	-0.000110742	0.007847403	0.002931807
Tubb4a	1	6.09E-01	7E-08	-4.73494E-05	0.000971086	0.002929096

Gm20627	1	1.76E-10	0.3028	-0.001437329	0.007913138	0.002927041
Tmcc2	1	1.82E-02	0.0586	0.001160055	0.00474039	0.002925328
Gm50019	1	1.53E-02	0.0054	-9.6237E-05	0.003630591	0.002922896
Fam83e	1	1.00E+00	0.0008	-0.00036441	0.000900486	0.002916634
Zfp36l2	0.0039027	1.00E+00	1	0.012704604	0.013204894	0.002916507
Ces2h	2.81825E-06	3.10E-01	1	-0.004207224	0.006136126	0.00291493
Cnrip1	1	2.48E-20	0.057	0.000968925	0.013621792	0.002905162
Gm33180	1	2.17E-14	3E-10	0	0.004669488	0.002899296
Asb15	1	3.40E-05	0.0032	0.000286142	0.004377457	0.002894079
Gm8369	1	7.85E-18	0.0002	5.9124E-05	0.008466575	0.002891702
Elavl3	1	2.15E-07	6E-08	0.000208292	0.003131235	0.00288736
Itpripl2	1	1.14E-25	0.5499	0.000223867	0.013390803	0.002880686
Gm48045	1	1.00E+00	0.0032	0.000109156	0.001749176	0.002879108
Bach2os	4.24634E-05	5.76E-12	1	-0.008596438	0.02852802	0.002878944
Grap2	1	4.46E-05	1	-0.001420335	0.008508159	0.002875993
Crip1	1	1.36E-15	0.9653	-0.00149602	0.011152649	0.002874209
Znfx1	1	2.81E-02	1	0.002870742	0.034315791	0.002870441
Gm41144	1	4.31E-03	8E-08	-0.000236747	0.002003811	0.002864015
Got1l1	1	1.00E+00	0.0002	-6.24665E-05	0.001408592	0.002854977
Gm47595	1	4.33E-02	0.0002	-0.000170551	0.00276673	0.002854319
Gm45606	1	2.50E-35	2E-07	-5.18112E-05	0.010620162	0.002853396
Gm16796	1	1.00E+00	1E-07	-6.96588E-05	0.000938026	0.002851288
Kcnq5	3.75701E-08	1.12E-06	1	0.007541015	0.016793335	0.002850363
Plxna3	1	1.00E+00	0.0003	-0.00053062	0.000746264	0.002849171
Zfp707	2.22502E-07	8.43E-18	1	0.024005413	-0.052406757	0.002848538
Gm49974	1	1.00E+00	0.0058	0.0006052	0.002366481	0.002848507
Fpr1	1	3.74E-14	0.001	-0.000421562	0.007691592	0.002845802
4930545L2	1.36265E-27	1.00E+00	1	0.018313419	0.004876675	0.002842704
Chst8	1	1.17E-02	0.0655	-2.57723E-05	0.003893129	0.002842565
Gm16118	1	1.59E-07	2E-07	0.000440529	0.003334922	0.002838562
Msx1	1	5.17E-09	4E-06	-7.67784E-05	0.004255412	0.002837108
Gm34933	1	1.00E+00	0.0008	-1.68485E-05	0.000947836	0.002831764
Procr	1	1.13E-31	7E-07	-0.000112546	0.010107928	0.002830841
Mark1	1	1.00E+00	0.0294	0.000995745	0.001833837	0.002825624
Rac2	1	3.96E-19	0.0003	8.24074E-05	0.009121263	0.002825359
1700001J0	1	1.64E-17	2E-09	0.000119739	0.004912941	0.002825045
Gm49101	1	2.68E-31	3E-07	2.95018E-05	0.009485154	0.002820222
Gm49484	1	2.00E-04	4E-06	-0.000306406	0.002941838	0.002816007
Ppp1r36	1	9.68E-05	8E-08	2.77811E-06	0.002649484	0.002815217
Pde8b	1	2.26E-12	1	0.001139178	0.012909516	0.002807791
BB557941	1	1.00E+00	0.0058	-0.000259789	0.000722695	0.002801816
Cd200r4	1	2.55E-32	6E-05	0.000217168	0.010780628	0.002800565
Parvg	1	3.85E-17	6E-05	0.000131393	0.006364753	0.002800565
Gm34885	1	4.58E-04	1	-0.001589719	0.006874097	0.002800208
Gm43660	1	1.30E-07	1	-0.000368314	0.013671839	0.002798301
Thbs1	1	1.90E-34	9E-06	-0.000245671	0.01497711	0.002797667
Folr2	1	1.43E-08	1	-8.96566E-05	0.008826782	0.002796387

C130083M	1	2.63E-07	0.0008	-0.000355486	0.004780702	0.002795692
Gm48342	0.001168299	1.00E+00	1	0.004529643	-0.001693919	0.002795532
Prkar2b	1	8.77E-15	0.005	0.000526377	0.008110679	0.002787175
Dzip1	1	1.00E+00	0.0006	-5.96884E-05	0.002221241	0.002785073
Klrc2	1	7.63E-01	8E-06	-7.85826E-05	0.002134673	0.002784941
Thbd	1	5.25E-10	0.0001	-0.000167015	0.005568631	0.002783486
Glrp1	1	1.39E-27	3E-08	8.57749E-05	0.00743657	0.00277498
Gm5103	1	1.00E+00	0.0003	4.38865E-05	0.001951982	0.002770897
Gm13431	1	7.29E-07	4E-06	9.29673E-05	0.003883319	0.002770765
Gm10639	1	1.37E-12	3E-05	-0.000186667	0.005529683	0.002770107
Gm12678	1	1.00E+00	8E-08	0.000345878	0.000170627	0.002766418
Lrrc8a	0.000609314	1.00E+00	1	-0.016605753	-0.007680942	0.002758931
Zfp850	1	3.79E-02	9E-05	1.04667E-06	0.002548753	0.002758829
9230109A2	1	6.16E-04	0.0621	-0.000152558	0.004759567	0.002758293
ccdc198	1	6.41E-11	5E-07	0.000226092	0.004149671	0.002755141
Cd160	1	2.50E-03	0.0001	0.00020395	0.003306678	0.002754477
Gm11791	1	4.31E-02	3E-10	0	0.001018435	0.002752901
Gm43646	1	2.88E-01	0.0026	2.16247E-05	0.002634843	0.002751899
Gm44752	1	1.86E-02	0.0104	-0.000133857	0.00346801	0.002750582
Pdgfb	1	4.86E-48	2E-05	5.00798E-05	0.016155135	0.002750318
Dok6	1	1.77E-02	1	-0.00145784	0.00980465	0.002744701
Spire1	1	2.93E-12	0.0197	0.00088944	0.00750957	0.002743473
Ttc28	1	1.13E-02	1	-0.000608009	0.017566935	0.002743033
Tmem212	1	1.54E-04	5E-09	3.39637E-05	0.002180471	0.002741624
Gm5914	1	1.00E+00	0.0031	1.71151E-05	0.002601783	0.002737065
Il10rb	1.20507E-12	9.90E-36	1	-0.011630493	0.059399395	0.00273681
Gm48935	1	5.44E-03	0.0003	5.46091E-06	0.003136916	0.002734825
Sema3f	1	4.19E-05	1	7.82365E-05	0.006573167	0.002728138
F730035M(	1	9.90E-03	1	-0.000544893	0.00671896	0.002726403
Scml4	1	3.49E-04	1	-6.71002E-06	0.006520596	0.002724647
Hif3a	1	1.10E-05	8E-05	0.000121711	0.003733226	0.002718268
Eldr	1	2.91E-05	1E-04	-0.000207245	0.003475945	0.002715695
4921501I0!	1	7.02E-05	0.0306	-8.9165E-05	0.004520387	0.002713056
Rasl10b	1	7.91E-02	0.0002	-0.000285828	0.002501404	0.00271148
Adamdec1	1	1.74E-05	2E-07	-0.00014651	0.003173431	0.002709899
Fabp7	1	8.63E-28	4E-05	-0.000404688	0.010611569	0.002705541
Mmp24	1	2.11E-05	6E-05	0.000203782	0.003301863	0.002699412
Gm42109	2.08709E-73	4.95E-14	1	-0.063259296	-0.049848917	0.002699015
Cend1	1	1.00E+00	0.0003	5.28103E-05	0.000213496	0.002698754
Gm36913	1	3.68E-18	5E-09	0	0.004957713	0.002697831
Trim17	1	1.88E-02	1	-0.003120166	0.01078769	0.002695663
Igsf3	1	1.21E-08	3E-05	5.02479E-05	0.004840998	0.002693611
Gm36617	1	1.00E+00	0.0002	-0.000170456	0.001227803	0.002691691
Tafa1	1	2.07E-03	1	-0.000314388	0.011439528	0.002690356
Bsn	1	3.36E-20	1	-0.000849267	0.017258881	0.002688332
Pbk	1	1.00E+00	3E-05	-0.00043953	0.001061305	0.002685237
Gm33055	1	6.13E-05	2E-09	0	0.001629497	0.002683655

Gm26648	1	1.68E-03	0.0334	0.000723893	0.003875384	0.002681336
Pik3cg	1	3.24E-14	0.0003	-0.000416222	0.00656886	0.002680742
Gm20036	1	3.40E-05	0.0107	0.001033029	0.004377457	0.002678438
Ifi27l2a	1	1.15E-08	0.0029	-0.000347422	0.005991935	0.002678357
Pou2af1	1	1.31E-11	0.0005	0.000168135	0.006748203	0.002676857
Slc30a1	0.000847348	2.34E-07	1	-0.007225845	0.022117832	0.002673293
Arrb2	1	2.33E-17	1	0.000487447	0.040170961	0.002671732
Mfsd5	1	5.14E-03	1	-0.001089068	0.007609191	0.002671655
Gm43699	1	6.24E-22	8E-10	0.000128662	0.005295864	0.00266948
Gm32786	1	5.47E-21	8E-10	0.000214437	0.005211327	0.00266948
A330084C1	1	1.14E-09	8E-10	0.000128662	0.002767082	0.00266948
8030474K0	1	1.00E+00	8E-10	0	0	0.00266948
Acod1	1	9.55E-58	2E-06	0	0.025166806	0.002661849
Tspan8	1	3.96E-08	1E-05	0.000286827	0.003389856	0.00266146
Erich5	1	2.11E-04	0.1153	-0.000388451	0.004671362	0.002658123
Ccno	1	1.00E+00	0.0009	0.000296797	0.001686655	0.002654914
Tes3-ps	1	2.60E-03	0.0492	-0.000398301	0.004137463	0.00265298
Ccn2	1	3.24E-09	0.0608	0.000937572	0.006415418	0.002652843
Ifi209	1	1.44E-41	1	-0.000685981	0.025177161	0.002650284
Mal	1	1.47E-11	5E-09	4.28875E-05	0.003293605	0.002649032
Pqlc3	1	4.81E-14	0.2148	-0.00050819	0.008655413	0.002648953
Svopl	1	5.54E-01	0.0003	1.52626E-05	0.001976995	0.002645466
Gm48508	1	1.00E+00	0.0005	-2.84551E-05	0.001468679	0.002644341
Rnf212	1	2.25E-02	0.0057	-0.000134783	0.003113347	0.002638673
Gm14341	1	2.31E-08	3E-06	0.000226092	0.003538609	0.002637097
Psmb1	1	3.18E-05	1	0.014868761	-0.034887511	0.002636785
Ifi211	1	2.74E-40	0.0652	-0.000176624	0.021202144	0.002635627
4930412F0	1	4.22E-05	1	0.000579549	0.006749056	0.002631794
Cdnf	1	1.00E+00	0.0191	-0.000472495	0.002124161	0.002631089
Psmb11	1	1.00E+00	0.0191	-0.000300945	0.001478488	0.002631089
Gm44710	1	3.86E-02	1	-0.001053048	0.012648173	0.002630842
Gm41361	1	3.05E-13	0.0024	0.000718432	0.006138167	0.002630166
4930417O1	1	3.76E-06	0.0017	0.000233284	0.004009174	0.002620338
Gm47257	1	1.00E+00	0.005	-0.000518112	0.002108319	0.002619679
Hist1h3a	1	1.00E+00	0.0107	0.000188665	0.001990722	0.002619021
AC142100.	1	4.50E-26	0.0024	4.74221E-05	0.013029193	0.002616644
Tmem139	1	1.14E-03	6E-08	0.000158164	0.002013972	0.002610853
Tspan13	1	3.88E-19	0.0088	-0.000269664	0.009313433	0.002610509
Trim80	0.000376106	1.00E+00	1	0.006942926	-0.002939679	0.002607126
Impg1	1	2.78E-03	0.8599	-0.000457111	0.004631138	0.002604962
Gm43689	1	1.00E+00	0.0056	0.000666741	0.002584247	0.002602601
Rnf113a1	1	1.00E+00	0.0005	0.001041074	0.001658958	0.002600361
Bcar1	3.94741E-23	1.00E+00	1	-0.037006999	-0.015925312	0.00259881
Arap3	1	3.53E-17	1	0.001098145	0.023410912	0.002589496
Trim16	1	2.18E-08	3E-08	0.000381525	0.002600583	0.002587508
Gm31219	1	2.17E-14	2E-09	0	0.003666368	0.002586059
Podxl	1	1.86E-05	0.0572	4.70379E-05	0.005203918	0.002585188

Usp26	1	1.00E+00	0.0051	-0.000453842	-0.000453842	0.002584082
Gpr65	1	1.31E-12	2E-05	5.90036E-05	0.0055922	0.002580577
Rgs14	1	7.69E-20	2E-06	0.000106353	0.007591883	0.002579128
Cyp2g1	1	6.05E-19	3E-05	0.000169818	0.006690166	0.00257847
Adam15	1	4.10E-13	0.3797	0.000114569	0.009137792	0.002565064
Prrx2	1	3.32E-03	0.0108	0.000347707	0.003803122	0.002563626
Sp140	1	2.09E-13	1	-0.003109498	0.026294301	0.002557052
Bhlha15	1	1.00E+00	2E-06	0.000341416	0.000123278	0.002552226
A230009B1	1	3.68E-18	5E-09	0	0.004600265	0.002551436
4732490B1	1	1.47E-11	5E-09	4.28875E-05	0.003055306	0.002551436
Tmem51os	1	1.32E-06	0.7479	-5.85689E-05	0.006013516	0.002550692
Lipm	1	1.00E+00	0.0054	-0.000269711	0.001402559	0.002545428
Chst3	1	1.67E-13	0.0639	-0.000766393	0.010558311	0.002545423
Gm47760	1	1.03E-03	8E-06	0.000178742	0.002591974	0.002540949
Gm49380	3.2583E-05	1.00E+00	1	-0.007971604	0.007684653	0.00254087
Bin2	1	9.45E-15	1	-0.001202612	0.017169707	0.002540738
Sparc	1	5.14E-06	1	-0.000838107	0.009722323	0.002538529
Troap	1	1.00E+00	1E-04	-0.000182157	0.001692688	0.002534677
4933406P0	1	1.00E+00	0.0351	-0.000299093	0.001125377	0.002532696
Dubr	1	4.23E-38	0.0103	1.00909E-05	0.014411167	0.002531906
Tomt	1	4.26E-02	1	-0.000103308	0.00416068	0.002531041
Gm33103	1	1.11E-33	1E-05	-0.000108084	0.011546475	0.00253033
Lyzl4	1	1.13E-37	0.0559	0.000626559	0.01682681	0.002529149
4930535L1	1	1.00E+00	1E-07	0.000115277	0.0006727	0.002527432
C130026I2	1	1.30E-05	1	-8.90816E-05	0.019524664	0.002526417
Styk1	1	6.09E-02	0.0024	-5.35427E-05	0.002402576	0.002524802
Magoh	6.19761E-05	1.00E+00	1	-0.009914594	-0.001937883	0.002520377
Bcas1os2	1	1.30E-10	1E-08	0	0.002851619	0.002516813
Rasal3	1	2.97E-23	0.0088	0.000570286	0.010627394	0.002512912
Aif1	1	1.41E-13	0.0333	-0.000115914	0.007026267	0.002512912
Ripk3	1	8.60E-22	3E-06	0.000153702	0.007400933	0.002508433
Gm30889	1	6.13E-05	5E-09	0	0.001748646	0.002502638
Ptpro	1	1.45E-27	0.018	-0.000274126	0.011356531	0.002500186
Was	1	1.18E-29	0.0002	-0.00012147	0.010658902	0.002500054
Ticam1	1	1.64E-03	1	-0.002251182	-0.01837043	0.002495958
Strip2	1	1.03E-02	0.0083	-0.00011874	0.003457175	0.002489521
Tbkbp1	1	3.77E-04	1	-0.001746006	0.008735925	0.002485034
Gm5431	1	2.72E-15	3E-05	7.96293E-05	0.006121301	0.002484429
Traf1	1	6.51E-06	0.001	-0.000104476	0.004516271	0.002482133
Gm45740	1	3.69E-02	1	-5.83023E-05	0.005573931	0.002481499
Hr	1	2.89E-16	6E-07	6.79274E-05	0.005021929	0.002480083
Gm43137	1	2.18E-01	0.0004	-1.73144E-06	0.002089901	0.002474601
Cdr2	1	2.92E-05	4E-05	0.000589817	0.003322184	0.002471703
Dnajb13	1	7.60E-17	7E-08	0.000252863	0.004910364	0.002469464
Gm17080	5.95466E-09	1.00E+00	1	0.008108202	0.001606394	0.002468401
Gm19299	1	7.41E-04	0.3572	0.000220897	0.004358687	0.002467935
Siglecg	1	5.75E-13	0.0142	-0.000367141	0.006557254	0.00246767

Anxa11os	1	1.23E-02	1	0.00102715	0.005558759	0.002458817
Gm26881	1	2.03E-03	1	-0.000665894	0.007022496	0.002452667
Asap1	6.71905E-50	7.89E-01	1	-0.098827743	0.068541027	0.002451256
Slc6a19	1	6.56E-06	0.0002	-0.000306406	0.003587511	0.002447699
Gm15584	1	2.69E-07	6E-06	0.000235015	0.003022247	0.002446909
Sox4	1	3.77E-02	0.0017	-0.000351951	0.002997653	0.002446245
Aldh1l2	1	1.28E-06	0.0032	1.08007E-05	0.004001129	0.002445449
Rdh11	1.57319E-20	6.89E-03	1	0.021306787	-0.012493063	0.002444527
Fam167b	1	7.59E-02	0.0199	-8.7306E-06	0.003358559	0.002444132
Gja4	1	1.00E+00	0.0028	0.000411147	0.001989378	0.002437075
Smpd5	1	4.60E-17	9E-06	0.000101891	0.005657441	0.002434182
Cdc25c	1	4.30E-03	2E-06	-0.000236747	0.00224211	0.002432733
9230111E0	1	5.47E-09	1	-0.001051955	0.008690096	0.002432106
Zfp827	1	1.33E-06	1	-0.000341955	0.007321936	0.002430403
Ccr2	1	2.91E-24	0.0004	-7.41207E-05	0.010282321	0.002427864
Relt	1	6.57E-10	8E-06	0.000282365	0.003953568	0.002425012
Gm47027	1	2.47E-09	4E-05	-0.000250132	0.003898634	0.002422905
Pcbd1	1	2.89E-04	1	0.006946451	-0.024748859	0.002420362
A930006L0	1	1.00E+00	0.0197	0.000326251	0.001762584	0.002414657
Cnr2	1	2.59E-18	0.1238	4.30806E-05	0.010009839	0.002414515
Tmem229b	1	2.86E-22	0.0042	0.000463885	0.009467936	0.002413867
Evc	1	1.00E+00	0.0042	-0.000204515	0.00074002	0.002413867
Pygm	1	4.58E-21	1	0.000186981	0.011434012	0.002412074
Gm49226	1	1.77E-09	6E-05	-4.0157E-05	0.004174121	0.002410178
1700065D1	1	1.00E+00	6E-05	-8.30445E-05	-0.000222457	0.002410178
Gm49965	1	3.94E-17	0.18	-0.000418879	0.010642565	0.002403375
Nme3	1	7.44E-05	1	0.000659888	0.006768707	0.002401182
Gm16098	1	2.99E-01	0.0045	-0.000423414	0.002525854	0.00240114
Gm15169	1	3.00E-01	0.0045	0.000262786	0.002406705	0.00240114
Rasgef1c	1	2.10E-05	0.0005	0.000118007	0.003862998	0.00240035
Rangrf	1	1.00E+00	0.0002	0.000422681	0.001516028	0.002398901
Batf	1	6.19E-14	0.009	-3.38909E-05	0.008210668	0.002393282
Aldh1a2	1	1.00E+00	0.0303	-0.00024294	0.000871907	0.00239197
Gsn	1	1.45E-24	0.0562	-2.9261E-05	0.011256217	0.002390333
Rgs19	1	5.06E-26	0.0002	-2.05053E-05	0.009170783	0.00238744
S100a6	1	1.35E-10	0.2176	1.77816E-05	0.007756249	0.002385736
A230028O0	1	4.08E-43	0.0004	0.000523646	0.014459395	0.002385379
Gm13362	1	4.19E-13	0.2947	-0.000138319	0.007206177	0.002383723
Gm10010	6.23681E-05	1.65E-01	1	-0.004070611	0.006580126	0.002383611
Tectb	1	1.09E-14	0.1075	-7.32418E-07	0.007623712	0.002380825
1110019D1	1.607E-30	7.27E-11	1	-0.046582938	-0.045615067	0.002371073
Gm14146	1	1.00E+00	0.0367	0.000321789	0.000900486	0.002367308
Gm49123	1	2.51E-06	8E-07	-1.33857E-05	0.002505884	0.002360589
Gm4473	1	1.14E-03	8E-07	-0.000142048	0.001894823	0.002360589
Rasgrp1	1	2.25E-09	0.0675	-0.000226824	0.006464107	0.002358138
Dpep2.1	1	1.01E-04	0.1237	-4.79865E-05	0.003846804	0.002358138
Cd101	1	2.26E-24	6E-06	-4.46189E-06	0.00931927	0.002357827

Gm34425	1	8.14E-24	4E-07	0	0.006687394	0.002354883
Epn3	1	1.00E+00	2E-07	0	0	0.00234997
Pilrb1	1	3.52E-12	0.0071	0.000177889	0.007013068	0.002349302
Dzip1l	1	1.82E-05	0.0001	0.000436283	0.003127988	0.002347858
Tmeff1	1	3.01E-04	0.0639	-0.000365336	0.004350638	0.002345228
Pdcd1lg2	1	1.31E-20	2E-06	3.84256E-05	0.006320859	0.002343652
Lix1	0.016703261	1.00E+00	1	-0.007499402	-0.001557111	0.002342123
Gm29417	1	1.60E-04	1	-0.000372335	0.005117477	0.002339404
2310039L1	1	1.00E+00	0.004	-0.000200563	0.000114279	0.002331373
Zeb2os	1	1.59E-33	0.0002	-6.96588E-05	0.01160296	0.002330313
Cyp26b1	1.38135E-06	1.00E+00	1	-0.003627379	-0.000753714	0.002329807
Kbtbd2	1.75727E-05	1.00E+00	1	-0.017406226	0.003835103	0.002328751
Snai3	1	1.00E+00	0.0004	-0.000387719	0.00147884	0.002328206
Rwdd2a	1	1.00E+00	0.0001	0.00051738	0.000388604	0.002326757
Slpi	1	3.09E-02	1	-0.000476741	0.004580805	0.002321402
Insyn2b	1	1.92E-24	0.656	-0.000767367	0.012784983	0.002321265
Ccnb1	1	1.00E+00	0.0064	-0.000337591	0.001120367	0.002319827
Smc2os	1	1.25E-07	0.0089	-0.000208976	0.004920173	0.002317719
Arl13a	1	1.21E-06	0.0366	-0.000168747	0.004787616	0.002316265
Klrd1	1	3.17E-08	0.002	-0.000205441	0.004528322	0.002316133
Gpr132	1	3.24E-14	0.3503	-0.000551956	0.009512565	0.002309334
Gm26693	1	9.26E-09	1	-0.001271893	0.014737383	0.002308591
Gm28981	1	2.44E-15	0.0023	-0.000192055	0.007284023	0.002305514
Platr31	1	4.01E-05	1	-0.000351693	0.009335914	0.002301967
Gm35028	1	1.68E-12	7E-08	0	0.003378143	0.002301172
Crlf1	1	1.14E-09	7E-08	0	0.00300538	0.002301172
8030453O2	1	4.23E-30	0.1228	-0.000213438	0.011579183	0.002298721
Sult2a3	1	1.00E+00	0.0164	0.00054966	0.002618502	0.002295823
9330198I0	1	1.82E-05	0.0003	0.000260055	0.003036536	0.002292134
Tg	1	2.42E-08	0.0284	0.000215484	0.005215982	0.002286653
Lbh	1	2.52E-10	1	-0.000832154	0.011335571	0.002284455
Dusp2	1	5.12E-26	0.0043	-0.000248328	0.009401498	0.00228151
Tekt5	1	6.59E-10	8E-06	-6.0735E-05	0.003715269	0.002278617
Dmrt2	1	1.00E+00	0.0002	2.23822E-05	0.000611943	0.002277163
Oas1g	1	5.48E-16	0.1238	-0.000269711	0.008651089	0.002274717
Oas3	1	1.89E-62	0.0392	-0.000263518	0.022642276	0.002274584
Gm49312	1	6.27E-05	0.0028	-0.000186667	0.003254513	0.002269579
Cxcr4	1	1.00E-29	0.0004	0.000515792	0.009887852	0.002268789
Mylk4	1	3.98E-03	0.5572	-0.000706444	0.004875079	0.002268303
Gm19510	1	2.17E-14	4E-07	0	0.004023816	0.002266549
Gm47827	1	7.05E-06	1	0.000543644	0.012565839	0.002262874
Efhc1	1	7.44E-08	0.2964	-0.000603815	0.005930882	0.002261327
Gm41031	1	9.91E-03	0.0192	1.16542E-05	0.002793084	0.002261021
Nlrc3	1	2.85E-13	1	-0.000340811	0.012534128	0.002260573
Ptrh1	1	1.00E+00	0.0154	3.94722E-05	0.002332328	0.002259751
Vipr2	1	5.33E-17	0.0071	-2.40409E-05	0.007099092	0.002258302
Hcst	1	5.69E-18	0.0002	-0.000168819	0.007198798	0.002257511

Tnfrsf26	1	9.95E-18	0.7664	0.000111862	0.011368859	0.00225633
Mansc1	1	1.00E+00	0.0335	-0.000125812	0.000749511	0.002255399
Mmp9	1	1.72E-13	0.0047	2.0578E-05	0.006178069	0.002251851
Gm41168	1	1.10E-05	0.0008	0.000194906	0.003192874	0.002244785
Tktl1	1	1.00E+00	0.0003	-0.000297482	0.00140704	0.002243336
Gm26912	1	6.47E-12	9E-07	0.000205514	0.003690818	0.002241096
AC121792.	1	7.93E-06	0.0346	-0.000513651	0.004108002	0.002235747
Entpd3	1	1.21E-15	3E-05	0.000248401	0.006139046	0.002234824
Foxo6	1	9.25E-01	3E-05	2.0578E-05	0.00139275	0.002231268
Nmbr	1	1.35E-02	1	0.000550659	0.003914124	0.002230397
Cfap69	1	3.09E-03	1	6.25551E-05	0.007679228	0.002226867
Gm43136	1	1.00E+00	5E-05	0.000169866	0.000734339	0.002218541
Gm11290	0.001443893	1.00E+00	1	-0.013225199	0.010122163	0.002218398
Gm26686	1	6.13E-05	2E-07	4.28875E-05	0.002017906	0.002217751
Nucb2	1	2.11E-02	1	0.001319145	0.004266981	0.00221735
Trpc6	1	3.15E-03	1	0.000141678	0.005702711	0.002213903
H2-M3	1	8.30E-05	1	-0.00014717	0.006468571	0.002209286
Gm36752	1	2.87E-03	0.0346	0.000127051	0.003265556	0.002208049
Gm44731	1	1.60E-01	0.0002	-7.85826E-05	0.001861763	0.002207264
Fgl2	1	5.83E-13	0.0163	0.000134219	0.006540388	0.00220389
4930562C1	1	1.36E-10	0.2188	0.000251204	0.007510257	0.002199624
Gm16141	1	9.48E-01	0.0008	0.000427143	0.001767065	0.002195987
Gm5546	1	6.28E-03	2E-05	-0.000189397	0.001847474	0.002195196
Gm30551	1	1.37E-12	0.0009	0.00066389	0.004805504	0.002176197
Ms4a4c	1	7.89E-35	0.2232	-0.000390256	0.017125239	0.002171972
Gm47851	1	3.22E-17	4E-07	0	0.004515728	0.002168953
Tmem213	1	3.85E-01	2E-07	0.000128662	0.000814748	0.002168953
Frmd7	1	1.00E+00	0.0006	0.00032803	0.001729876	0.002159915
Tshr	1	1.04E-02	1	0.000883199	0.004132101	0.002159044
Cd14	1	8.87E-14	2E-06	0.000162626	0.004897626	0.002157675
Gm48707	1	2.76E-07	2E-06	-8.92378E-06	0.002553234	0.002157675
Hk1	1	1.10E-09	1	-0.000586699	0.009451248	0.002155797
Gm12708	1	3.08E-33	1E-05	0	0.009458126	0.002154913
Pik3cb	1.16377E-06	1.00E+00	1	-0.025607502	0.048407939	0.00215128
Gm36756	1	2.11E-05	0.005	-0.00043953	0.003540161	0.002150086
Gm17224	1	1.00E+00	0.0002	0.000255641	3.87403E-05	0.002149296
Tlr2	1	5.03E-14	2E-06	0.00027795	0.00438384	0.002135779
Gm7932	1	1.47E-11	4E-07	0	0.003055306	0.00213433
Arl4c	1	5.41E-27	0.0485	0.000463933	0.010303006	0.002131087
Phf11b	1	7.87E-07	1	0.000112296	0.007825773	0.002129224
Nckap5l	1	2.23E-13	0.0619	0.000249448	0.007703975	0.00212898
Cdca5	1	1.77E-09	0.0014	0.00017428	0.004174121	0.002125292
Gm17160	1	8.16E-10	0.0043	0.000336906	0.004486797	0.002114014
Tigd3	1	6.32E-05	0.0017	0.000495118	0.002989187	0.002112565
Gm16059	1	3.50E-04	0.0017	-0.000259056	0.002904649	0.002112565
Klc3	1	1.00E+00	4E-05	2.50399E-05	0.001236412	0.002111775
Atp2a3	0.02439121	5.77E-02	1	-0.00196617	0.00591352	0.002109101

Gm28095	1	3.11E-03	1	-0.000230287	0.004325627	0.002098969
Zfp867	0.013880764	1.00E+00	1	-0.010144231	-0.013335933	0.002092917
Sycp2	1	4.90E-21	0.0045	-1.33857E-05	0.00981569	0.002088519
Stxbp1	1	4.98E-03	1	0.001864488	0.008223513	0.002088461
Gbp5	1	8.60E-08	1	5.46867E-05	0.011485419	0.002087719
Lrr1	1	1.88E-07	2E-06	-4.73494E-05	0.002635194	0.00208698
Gm41392	1	1.30E-10	4E-07	0	0.002851619	0.002085531
Gm44066	1	1.00E+00	4E-07	0	0.000407374	0.002085531
Spatc1	1	1.82E-05	0.0014	0.000885567	0.003274835	0.002076493
Zfp583	1	1.00E+00	0.0014	-0.000168819	0.001933563	0.002076493
Cnnm1	1	1.01E-03	1	-0.003364107	0.010305552	0.002076478
4930469K1	1	2.62E-10	0.4208	0.000335223	0.006378018	0.00207391
Klrk1	1	1.45E-09	0.4208	0.000535154	0.006328091	0.00207391
Fam43a	1	1.42E-04	1	-0.001611054	0.007861695	0.002073081
Retnlg	1	6.13E-05	9E-05	4.28875E-05	0.001867795	0.002071492
Hsf2bp	1	1.54E-03	0.024	-5.80046E-05	0.002966288	0.002068114
Tedc1	1	3.64E-08	0.0002	2.77811E-06	0.003345083	0.002064425
Plin1	1	1.28E-04	4E-05	0.000110815	0.002254848	0.002062976
She	1	7.30E-04	1	0.000386612	0.005180349	0.002051482
AC106834.	1	3.01E-07	1	-0.000498388	0.006577241	0.00204489
Gm48682	1	1.63E-06	2E-06	-4.73494E-05	0.002193209	0.002038182
Ltc4s	1	1.00E+00	0.0192	0.000155506	0.000455605	0.00203773
Col1a1	1	2.95E-39	0.0002	0.00016443	0.013364289	0.002036728
Gm10874	1	1.89E-05	0.1101	0.000129709	0.004162935	0.002034894
Kcng2	1	1.83E-14	0.0111	-0.000164358	0.005681892	0.002034149
Ccl3	1	4.20E-19	6E-06	0	0.006145556	0.002030461
Mmp7	1	4.83E-03	6E-06	0	0.00142581	0.002030461
Fndc10	1	1.00E+00	3E-05	-0.000142048	0.000876387	0.002026904
Lsmem1	0.000209508	1.00E+00	1	-0.006692107	0.002600784	0.002026505
Pcdhga1	1	4.32E-02	0.1234	-0.000145463	0.003005029	0.002020106
Gimap5	1	5.71E-04	0.2247	0.00018876	0.0037454	0.002010936
Il2rb	1	4.09E-04	1	0.000267416	0.004499391	0.002010931
Gm20275	1	3.48E-06	1	0.00109405	0.010805342	0.002009578
Klf4	1	2.43E-08	0.0563	-0.000281366	0.004977684	0.002008038
Hpse	1	3.98E-20	0.2301	0.000359289	0.01057503	0.002006584
Atg12	1	7.12E-06	1	-0.005320351	-0.027383926	0.002004798
Gm35550	1	1.00E+00	0.0008	-3.12332E-05	0.001501738	0.00200435
Gm16894	1	1.03E-03	6E-06	0.000209975	0.001939595	0.002003559
Gm49736	1	6.13E-05	1E-06	8.57749E-05	0.001629497	0.00200211
Hnrnpul2	1.09547E-05	3.89E-01	1	0.02652306	-0.02258065	0.00199476
Gm5878	1	1.00E+00	0.0163	0.000727355	0.00135969	0.001994521
B3galt4	1	1.00E+00	0.0077	0.000126931	0.000544942	0.001994521
Il21r	1	1.33E-11	0.4159	-3.41522E-06	0.006152457	0.001989039
Hotairm1	1	7.63E-01	0.0111	0.000332492	0.002134673	0.001985351
Thy1	1	1.59E-07	0.0039	9.74292E-05	0.003573221	0.001983902
Tmem127	0.001267363	1.00E+00	1	-0.016155047	-0.0106688	0.001981611
Ackr3	1	2.67E-07	0.0004	-0.000168772	0.003617993	0.001981004

Cdkn1c	1	2.91E-01	0.0008	0.000463006	0.001796877	0.001976652
Gm41556	1	6.13E-18	0.6832	-2.39205E-05	0.008036449	0.001971303
Kirrel3os	1	1.00E+00	8E-05	3.39637E-05	0.00072005	0.001970385
Tenm4	1	2.91E-42	1	-0.000609082	0.031009411	0.001969078
Dusp7	1	1.40E-04	1	-0.000255086	0.005678823	0.001964631
Acap1	1	5.99E-07	1	0.002482148	0.011012433	0.001963941
Gm14223	1	5.54E-08	0.4087	0.000227138	0.005222688	0.001961479
Gm30211	1	9.33E-05	0.2129	-5.17385E-05	0.0040507	0.001959893
Gja1	1	2.58E-03	1	0.000365843	0.004734655	0.001957724
Acss1	1	3.05E-06	0.7788	-8.55341E-05	0.005526097	0.00195699
Nron	1	1.21E-04	6E-06	-4.46189E-06	0.001785834	0.001954761
A430088P1	1	8.77E-03	6E-06	0.0001242	0.00137846	0.001954761
Gm32364	1	1.77E-19	0.0565	-3.65486E-05	0.008181282	0.001952643
Pmaip1	1	1.82E-15	0.0019	-9.91606E-05	0.006057085	0.001951386
Ppic	1	1.18E-02	0.4164	0.00038274	0.003766604	0.001946508
Slc35g2	1	5.98E-03	0.2307	0.000354827	0.003099057	0.001945059
Grip1os2	1	1.59E-13	0.0002	6.79274E-05	0.004530017	0.001944932
Cntnap1	0.007847336	1.00E+00	1	-0.003933589	0.00064067	0.001939385
Gm47925	1	8.45E-11	1	-0.000461767	0.006291931	0.00193747
Hrh1	1	3.17E-07	0.0008	0.000115277	0.003305318	0.001937211
Slc24a1	1	5.77E-04	0.0216	0.000339732	0.002894488	0.001934445
Sgpp2	1	1.14E-06	0.0394	-8.80336E-06	0.004205486	0.001933649
Fhl3	1	1.54E-16	0.4219	6.00979E-05	0.008042126	0.001927515
Gm16235	1	1.15E-06	0.4219	0.000653235	0.004202701	0.001927515
Lrrk2	1	2.51E-07	1	0.000927858	0.014820584	0.001926787
Gm14327	1.97219E-33	1.49E-09	1	-0.032512558	-0.033221893	0.001924646
Plau	1	3.71E-44	0.0013	-0.000189397	0.014863837	0.001924485
6030466F0	1	1.00E-08	2E-06	0.0003431	0.002444245	0.001918689
Gm49130	1	4.31E-02	2E-06	0	0.001018435	0.001918689
Zfp831	1	5.45E-13	1	-0.000989802	0.010212293	0.001915097
Gm48166	1	8.00E-04	1	-0.000199738	0.005306523	0.00191405
Ola1	1	2.93E-13	1	-0.010854026	-0.053514976	0.001913261
Gm44706	1	3.46E-03	1	0.002046599	0.005720459	0.001904349
Batf3	1	1.04E-18	1	-0.000142613	0.009075626	0.001902385
Slc51b	1	5.56E-01	0.0004	0.000291288	0.001127424	0.001901139
Npl	1	8.30E-04	1	0.000169243	0.006276083	0.001899282
Aox4	1	4.31E-03	0.0009	2.0578E-05	0.002003811	0.001897583
Lncbate6	1	1.91E-05	0.1103	-1.56337E-06	0.003896939	0.001896919
Gm36246	1	1.00E+00	0.0487	7.06578E-05	0.001947853	0.001887096
Gsg1	1	1.00E-08	6E-06	0.000128662	0.002444245	0.001884066
Gm48680	1	1.20E-02	1	6.89264E-05	0.003307543	0.001879507
Ermp1	0.000107456	1.00E+00	1	-0.013023067	0.003494508	0.001877213
Pla2g3	1	3.64E-08	0.0047	-0.000108084	0.003345083	0.001877135
Pdlim4	1	2.30E-30	0.0003	2.95018E-05	0.009708138	0.001874237
Gm4724	1	1.00E+00	0.0051	0.000521842	0.001454389	0.001873579
Ltb	1	2.53E-06	1	-0.000769929	0.007287445	0.001873367
Gnb4	1	7.26E-06	1	0.000193393	0.008176307	0.001872034

Arg2	1	6.11E-23	1	-0.000472495	0.014415604	0.001866258
Sh2d3c	1	1.91E-13	1	0.000481113	0.032286259	0.001866022
A930041C1	1	2.00E-04	0.0394	0.000722894	0.002941838	0.00186375
1700001K1	1	2.19E-14	0.002	0.00045674	0.00497458	0.00186296
Cryzl1	0.017245913	1.00E+00	1	0.019330417	-0.011322667	0.001861997
Ssmem1	1	2.51E-06	0.0001	2.95018E-05	0.002625033	0.001860062
H2-Ob	1	9.69E-10	1	-0.000361487	0.008656923	0.001850339
Rab6b	1	1.01E-04	1	-0.000352804	0.004240309	0.001850172
Cd33	1	1.10E-33	0.1219	8.8553E-05	0.012346241	0.00184892
4930557K0	1	1.26E-03	1	0.005584051	0.019640914	0.001847789
Wdr31	1	1.00E+00	0.0191	-0.000147363	-0.000168864	0.001842978
Gm13572	1	4.75E-04	1	0.000285168	0.003833397	0.001841981
Wfdc3	1	1.00E+00	0.0111	9.29673E-05	-0.000175108	0.001838956
Inhbb	1	9.96E-03	0.0039	0.000122517	0.002231949	0.001837507
Meltf	1	4.31E-02	6E-06	0	0.001018435	0.001835268
Slc25a43	1	6.09E-27	0.6972	-5.35427E-05	0.010112057	0.001830576
Gm50358	1	3.32E-03	0.0137	2.73045E-06	0.002425475	0.00182623
Arhgap27o	1	4.90E-09	0.0003	0.000183252	0.003355244	0.001825439
Gm12236	1	1.54E-04	8E-05	3.39637E-05	0.002265009	0.00182399
Gm48860	1	9.70E-05	0.0009	0.000378868	0.002621786	0.001821087
Gm37027	1	1.14E-02	0.7715	0.000412074	0.002970417	0.001818641
Naip1	1	3.56E-16	0.0192	-3.12332E-05	0.006290376	0.001817059
Gm44734	1	1.34E-02	0.0896	7.51197E-05	0.003132787	0.001816401
Gm15759	1	1.00E+00	3E-05	0	0.000203687	0.00181482
5830428M	1	3.29E-10	1	-0.000189277	0.006859235	0.001812231
Kbtbd11	1	2.02E-03	0.408	-0.000109816	0.003790173	0.001806573
Grap	1	1.18E-02	1	-0.000114157	0.003507983	0.001805909
Slit1	1	4.81E-07	0.1292	-3.28694E-05	0.004338569	0.001805782
Grip1os3	1	1.23E-03	0.0004	0.000334176	0.001738485	0.001803542
Oit3	1	8.64E-03	1	0.00034155	0.00696209	0.001801918
Gm15551	1	2.78E-03	0.0029	8.40911E-05	0.002160149	0.001801435
Gm12414	1	1.14E-09	1E-05	0	0.002647932	0.001800645
Adam34	1	5.69E-03	1	0.00025213	0.003671697	0.001798325
2010016I18	1	2.16E-04	1	-0.000402836	0.004241441	0.001796086
Gm47456	1	3.79E-02	0.2133	0.000361946	0.002548753	0.001792397
Adgrf4	1	2.35E-04	1	0.000574652	0.004146742	0.001784808
Lmo3	1	6.31E-03	1	-0.00019286	0.003463881	0.001783359
Chil3	1	7.47E-02	0.0061	-9.46987E-05	0.00156941	0.001783095
Mical1	1	4.18E-21	1	-0.000444845	0.010069926	0.00177887
Rmrp	1	1.68E-02	1	-0.003281399	0.008305131	0.001773784
Clec3b	1	4.56E-05	0.0192	0.000165404	0.002723861	0.001768261
Tcerg1l	1	2.91E-01	0.0192	0.000440529	0.001824575	0.001768261
Trdc	1	1.63E-06	0.003	-4.46189E-06	0.002669806	0.001767471
Zfp980	1	9.70E-05	0.002	0.000535228	0.002411185	0.001765363
4930473A0	1	3.86E-02	1	0.000627734	0.009729283	0.001764794
Dlk1	1	4.31E-02	0.0012	0	0.00142581	0.001758254
Mapk7	1	7.10E-04	1	0.000241645	0.006424625	0.001757349

Napsa	1	2.10E-10	0.7538	-4.72766E-05	0.005523859	0.001756978
Gm30655	1	1.00E+00	0.0241	0.000436067	0.001250702	0.001755535
Rbp7	1	1.00E+00	0.0004	0.000119739	0.000923737	0.001754744
Cyp27b1	1	1.00E+00	0.0087	-3.12332E-05	-0.000127758	0.001754086
Matn4	1	1.00E+00	0.0002	0	0.00099947	0.001751846
Sash3	1	4.13E-25	0.5522	0.000113593	0.009796804	0.001750711
Gm29459	1	1.77E-09	0.0293	4.56179E-05	0.004174121	0.001742808
Gm15406	1	1.00E+00	0.0293	-0.000297482	0.000999665	0.001742808
Gfpt2	1	1.14E-03	0.0008	0.000183252	0.002013972	0.001742018
Plxnb3	1	5.97E-02	0.0008	-3.11856E-05	0.001487449	0.001742018
Apobr	1	8.21E-11	1	-0.00026798	0.006714949	0.001740883
Sh3tc1	1	1.13E-07	1	-0.000763914	0.010873225	0.001737681
Gm14321	1	2.17E-04	1	-0.00072609	0.005806019	0.001736589
Phex	1	3.85E-03	1	-9.67755E-05	0.00648493	0.001735055
4930579C1	1	9.95E-12	0.0327	0.000298528	0.005388513	0.001732189
Ptpn7	1	5.15E-18	0.0106	2.77811E-06	0.006623373	0.00173074
Arhgap27o	1	1.00E+00	0.0013	-0.000189397	0.000625351	0.001729291
Itgb8	1	2.91E-05	0.2339	0.000630094	0.003771084	0.001725942
Klrb1c	1	1.34E-02	1	-0.00011423	0.003082861	0.001725213
Gm43434	1	1.13E-02	1	0.000308404	0.00352552	0.001724733
Runx3	1	7.22E-21	0.0167	-7.85349E-05	0.007327083	0.001723019
Arhgap27o	1	4.66E-10	0.0011	-9.46987E-05	0.003552704	0.001720121
Wtip	1	1.15E-04	1	0.000846625	0.006001656	0.001719246
Emp3	1	1.87E-22	0.1476	-1.73144E-06	0.00833896	0.001709634
Gm10184	1	2.05E-03	0.4086	0.000533496	0.002956127	0.001708976
Ankub1	0.013052492	1.00E+00	1	-0.005140689	-0.000189648	0.001705098
Tmem86a	1	9.43E-12	1	0.000253862	0.006370434	0.001702045
Slc19a1	1.25439E-05	1.00E+00	1	0.012597395	-0.004013291	0.001698035
Oas1a	1	1.25E-29	1	-0.000383016	0.013677758	0.001697688
8030456M	0.014351247	7.13E-01	1	-0.004065004	0.006801066	0.001697628
A930033M	1	1.87E-03	0.0717	0.000323568	0.002700962	0.001695459
Htra3	1	8.74E-12	0.0421	3.67418E-05	0.004472507	0.00168484
Grm4	1	6.13E-05	9E-05	8.57749E-05	0.002309781	0.0016826
Lum	1	6.05E-04	0.062	0.000128783	0.003078062	0.001680488
Sgcb	1	2.86E-03	1	3.86664E-05	0.003595306	0.001679824
A530076I1	1	4.67E-10	0.0004	4.91536E-05	0.003913415	0.001678248
Fcgr1	1	9.04E-15	0.6588	-0.000152656	0.007019562	0.00167646
Gm26947	1	1.94E-14	0.0385	0.000110815	0.005329451	0.001674221
5031425F1	1	1.99E-29	0.0045	-3.11856E-05	0.009801021	0.001672772
Prdm8	1	2.85E-04	0.0192	0.000311866	0.002639323	0.001670665
St6galnac2	1	7.82E-07	3E-05	0	0.002036871	0.001668425
Epgn	1	4.83E-03	3E-05	0	0.001222123	0.001668425
Gm12409	1	4.31E-02	3E-05	0	0.001018435	0.001668425
Gm17182	1	1.00E+00	3E-05	0	0.000526523	0.001668425
Gpr174	1	7.05E-08	1	-0.000100892	0.00457682	0.001665183
Ccdc28a	7.9057E-05	1.00E+00	1	-0.019330362	0.003279587	0.001664496
Sfxn3	1	2.37E-15	0.0072	0.000289652	0.005564099	0.001660045

Fa2h	1	8.31E-11	0.0106	-2.23094E-05	0.004075294	0.001647319
Selenon	1	4.93E-13	1	-0.000231238	0.007540053	0.001645526
Gm20540	1	1.21E-02	1	0.000127004	0.003160696	0.001636169
Plxdc2	1	1.97E-10	1	-3.13343E-06	0.019568649	0.001634403
Gna15	1	4.97E-08	1	5.63459E-05	0.004371984	0.001626866
Pdxp	1	4.68E-02	0.3016	-0.000259056	0.002293588	0.001626213
Aanat	1	3.77E-06	0.7711	-0.000452916	0.003770876	0.001625555
Gm48346	1	3.81E-12	0.0002	0.000167088	0.003415331	0.001621076
Cyp2c69	0.02126816	1.00E+00	1	-0.003424642	-0.002992645	0.001620593
4930430E1	1	4.60E-36	0.0005	0	0.009742701	0.001613354
Fam177a	1	1.83E-10	0.1478	-0.000216169	0.005249042	0.001612038
Bmx	1	1.34E-07	1	-5.17385E-05	0.00462715	0.001612033
Pmp22	1	1.07E-06	0.4202	0.000606859	0.003790736	0.001608997
Pdp1	1	9.97E-05	1	-0.000373286	0.004442551	0.001607347
Ms4a1	1	6.30E-05	1	-0.000420683	0.005378001	0.001598921
Cd69	1	1.27E-04	0.0386	0.000239477	0.003119854	0.0015908
Cdc42ep3	1	1.79E-02	1	-0.000267739	0.005584613	0.001590263
Gm33263	1	3.85E-03	0.755	0.00066662	0.00279979	0.001589483
Hist1h2bb	1	3.13E-02	0.1109	0.000264517	0.00206545	0.001588692
Gm48704	1	6.92E-06	9E-05	0	0.001833184	0.001585004
Gm47761	1	1.00E+00	0.0011	0.000462838	0.000312675	0.001573726
Zfp57	1	1.72E-03	0.0917	0.000327056	0.002677198	0.001573721
Stx11	1	1.75E-31	0.4889	0.000100255	0.012323342	0.001568245
Siglecf	1	8.14E-24	0.0005	0	0.006418135	0.001564556
Gm35733	1	2.48E-15	0.0005	0.000214437	0.005680955	0.001564556
5830408C2	1	1.87E-03	1	-0.000165994	0.002820112	0.001556967
Ctla2a	1	3.77E-02	1	-0.000152703	0.002906201	0.001556309
Gimap8	1	3.60E-05	1	0.0004804	0.013692842	0.00155513
Gpr171	1	3.16E-04	0.7319	0.000224408	0.003408274	0.001553411
Gm8797	1	1.72E-03	0.0916	0.000336954	0.002316487	0.00155262
Gm47557	1	2.29E-13	1	-0.000403762	0.00692677	0.001551957
1700013F0	1	2.23E-14	0.0328	0.000555853	0.004808081	0.001551171
Cyp2d22	2.61681E-25	1.08E-82	1	0.060985183	-0.163801086	0.001550174
Fcrla	1	3.13E-04	1	-5.80046E-05	0.004088558	0.001545644
3110006O0	1	1.00E+00	0.0106	0.000115277	0.000876387	0.001540552
Dnali1	1	2.92E-05	0.1109	0.000178742	0.002999348	0.001539894
Gm47593	1	5.81E-03	0.1109	5.00798E-05	0.002269137	0.001539894
1700112J0	1	1.04E-03	0.332	-9.45783E-05	0.002710453	0.001536991
Gm11100	1	2.17E-02	1	0.000734548	0.002814079	0.001532305
Klri1	1	1.21E-15	0.0146	-8.92378E-06	0.005185851	0.001531336
Gm49046	1	5.76E-04	1	-3.29647E-05	0.003268803	0.001530856
Gm14548	1	1.46E-17	1	-0.000204467	0.009673311	0.001526691
Vill	1	1.49E-03	1	0.000382524	0.003891577	0.001521028
Armc2	1	7.31E-09	1	-0.000226729	0.004871272	0.001520237
Gm16096	1	8.14E-24	0.0005	0	0.007994386	0.001515758
Sult3a1	1	1.00E+00	0.0005	0	0.000933898	0.001515758
Cldnd2	1	1.00E+00	0.0242	-0.000236747	0.000170627	0.0015151

Fbn1	1	1.60E-03	1	0.001327288	0.00780781	0.001514351
Fgfbp3	1	3.17E-03	1	-0.000311457	0.003838195	0.00151199
9430069I0	1	4.38E-13	0.0012	-4.46189E-06	0.003976466	0.001503031
Gm48940	1	2.82E-16	0.0002	0	0.004073742	0.001501583
Ly6g6g	1	2.48E-15	0.0002	0	0.003870055	0.001501583
Adgrf3	1	5.44E-04	0.0002	0.000257325	0.001695069	0.001501583
P2ry13	1	8.28E-11	0.1222	-0.000108084	0.004432742	0.001494652
Myo1a	1	1.89E-05	0.0026	0.000334176	0.002145859	0.001490305
Bend4	1	1.64E-11	1	9.84758E-05	0.007278856	0.001489302
Gm20685	1	2.00E-04	0.6606	0.000619319	0.002941838	0.001481267
Gm15419	1	1.00E+00	0.0167	0.000110815	0.001032725	0.001479028
Hepacam	1	1.00E+00	0.0167	2.50399E-05	0.000421664	0.001479028
Gm49313	1	1.04E-03	1	3.66941E-05	0.002738151	0.0014735
Lfng	1	4.50E-09	1	-0.000426024	0.006491343	0.001469984
Fbxo48	1	6.13E-05	0.0005	4.28875E-05	0.001748646	0.00146696
Dio2	1	6.13E-05	0.0005	0	0.001629497	0.00146696
Cacnb3	1	2.29E-06	0.084	3.39637E-05	0.002857105	0.001466467
4930533K1	1	1.18E-02	1	0.000504067	0.003269685	0.001458049
Arhgef28	1	1.13E-02	1	1.61889E-05	0.003503855	0.001457258
Olfr111	1	4.16E-34	0.0251	-9.91606E-05	0.010622738	0.001457131
Gm47754	1	1.11E-07	0.2341	-0.000250132	0.004444454	0.001456473
4930556G(	1	5.36E-03	1	0.00037021	0.004819973	0.001455729
Zfp410	1	1.17E-10	1	0.011481828	-0.048394431	0.001449902
Rab26	1	3.06E-04	1	-3.74266E-05	0.003425141	0.001448884
Gm38604	1	2.90E-07	1	0.001787433	0.017471717	0.001447185
G530011O(	1	2.52E-02	1	3.66941E-05	0.002330776	0.001446644
Bcl2a1d	1	2.72E-15	0.1223	-0.000108084	0.005570326	0.001445854
Gm13256	1	1.00E+00	0.0107	-0.000142048	0.001080075	0.001442956
Dok1	1	1.12E-07	0.49	2.23822E-05	0.004178458	0.00144295
Tnfrsf21	1	3.01E-21	1	0.001106018	0.015544922	0.001436736
Pde6b	1	3.49E-02	1	0.000357557	0.003283974	0.001435494
Cd40	1	1.31E-17	1	-0.000248087	0.010373605	0.001435305
1700120B2	1	2.85E-03	1	-0.00026798	0.002690801	0.001433918
Klrb1b	1	1.41E-05	0.0074	-4.46189E-06	0.00222782	0.001433786
Lilra6	1	1.00E-34	0.107	-4.73494E-05	0.011962839	0.001427513
Tmem150b	1	1.74E-09	1	4.56179E-05	0.005783675	0.001423299
Gm27042	1	4.68E-06	0.4897	-0.000378795	0.003322184	0.00142185
Gm16497	1	3.22E-17	0.0005	0	0.004634877	0.001418161
Gm15743	1	1.47E-11	0.0005	0.000128662	0.003055306	0.001418161
Olfr457	1	4.83E-03	0.0005	0	0.001222123	0.001418161
Platr14	1	1.00E+00	0.0005	0	0.000611061	0.001418161
Tas2r143	1	1.00E+00	0.0005	0	0.000407374	0.001418161
4930520M	1	1.00E+00	0.0005	0	0.000203687	0.001418161
Prima1	1	1.00E+00	0.0005	4.28875E-05	0	0.001418161
Gm10134	1	5.18E-09	0.0387	0.000151092	0.003841332	0.001416707
Gm33926	1	1.00E-08	0.0184	0	0.003209067	0.001411889
Hoxb4	1	1.75E-13	1	4.18058E-05	0.01099786	0.00141002

Mpp3	1	1.00E+00	0.0061	0.000101939	0.001281185	0.001406884
Gm13205	1	1.00E+00	0.0061	0.000205514	-9.46987E-05	0.001406884
Il3ra	1	3.36E-12	1	-0.001051053	0.011015312	0.001401398
Cxcl14	1	2.88E-10	0.018	2.06257E-05	0.003246256	0.001399163
Cd19	1	1.76E-19	1	-0.000812839	0.012159297	0.001397957
Rs1	1	4.35E-02	1	1.82346E-05	0.004184461	0.001397761
Dnajc27	1	4.53E-03	1	-0.000606989	0.005895105	0.001395643
Eps8l3	1	1.28E-04	0.0386	0.000239477	0.002254848	0.001395606
Gm49579	1	9.09E-04	0.0386	-6.0735E-05	0.002051161	0.001395606
Akap3	1	4.20E-02	0.0386	-0.000189397	0.001643786	0.001395606
Ankar	1	1.19E-02	1	-7.92476E-06	0.003198555	0.001390257
Vinac1	1	2.37E-10	1	-0.001295088	0.008672139	0.001388059
Gm35363	1	6.09E-01	0.0074	3.84256E-05	0.000971086	0.001384987
Gm20219	1	3.17E-08	1	0.000352096	0.00461286	0.001384324
Wasf3	1	4.31E-02	0.0012	8.57749E-05	0.001287695	0.001383538
Gm16079	1	1.00E+00	0.0012	0	0.000611061	0.001383538
Sectm1b	1	1.00E+00	0.0012	0	0	0.001383538
Panx1	1	1.14E-06	1	-0.000411664	0.005086672	0.001378189
Gm33280	1	6.34E-14	1	0.000190444	0.005981829	0.001377398
Kcna2	1	7.16E-03	1	9.97045E-06	0.003120049	0.001372529
Gm43913	1	9.71E-03	0.0147	3.39637E-05	0.001534798	0.001372261
Cd68	1	1.52E-23	1	-0.000136619	0.020914629	0.001371342
Inpp5j	1	2.50E-09	0.003	0.00027795	0.00280427	0.001370812
Gm48754	1	3.15E-02	1	-0.000224094	0.00342066	0.001368493
Scd3	1	4.16E-02	1	0.00022436	0.002776891	0.001364014
Ppfia4	1	4.29E-13	1	3.66941E-05	0.00613559	0.001363223
Slamf6	1	1.55E-10	1	-0.000310747	0.006771984	0.001362559
Gm50270	1	9.58E-03	1	-0.000216169	0.002378126	0.001361774
Gm26838	1	1.28E-04	0.0886	0.000239477	0.002254848	0.001360983
Gm47540	1	1.00E+00	0.0251	-1.33857E-05	0.000265326	0.001359534
Gm12333	1	3.47E-07	1	0.000283411	0.004515376	0.001352736
Nudt17	1	4.31E-02	0.0031	0.000300212	0.001018435	0.001348915
Armh2	1	1.95E-06	0.1223	-0.000193859	0.00281856	0.001348257
Cdh23	1	2.89E-14	1	-0.000118667	0.010174645	0.001347249
Calcrl	1.09413E-13	1.00E+00	1	0.013302687	0.015574009	0.001344894
Yod1	0.000179584	1.00E+00	1	0.016443176	-0.017039885	0.001343624
Cavin1	1	6.17E-03	1	0.000320201	0.003916028	0.001342908
Slc16a9	1	1.64E-03	1	-8.66515E-05	0.005180557	0.001339999
Gm34728	1	3.18E-08	1	-0.000119666	0.004409173	0.001335525
Aldh3b2	1	1.30E-10	0.0012	0	0.002851619	0.00133474
Gm28387	1	7.82E-07	0.0012	0.000128662	0.002036871	0.00133474
Gm50394	1	6.13E-05	0.0012	0.000428875	0.001748646	0.00133474
Gm20471	1	5.44E-04	0.0012	0	0.001544959	0.00133474
Dusp18	1	3.85E-01	0.0012	0	0.001137585	0.00133474
Gm42425	1	4.31E-02	0.0012	0.00017155	0.001018435	0.00133474
Gm47734	1	3.85E-01	0.0012	0	0.000933898	0.00133474
Hist1h2ak	1	1.00E+00	0.0012	0	0.000611061	0.00133474

Gm32172	1	1.00E+00	0.0012	0	0.000407374	0.00133474
Gm38064	1	1.00E+00	0.0012	0	0.000203687	0.00133474
Cass4	1	4.88E-08	1	-0.000276856	0.00587575	0.001333559
Gm43464	1	1.72E-04	1	-0.000448454	0.00380291	0.001332947
Palm	1	2.87E-08	1	-0.000498267	0.007495632	0.001326609
A930001M	1	2.06E-02	1	-0.000658565	0.009039226	0.001319407
Trim36	1	2.87E-16	0.0886	0.00016099	0.005855974	0.001312185
Igsf9	1	3.73E-06	1	-0.000581578	0.004366622	0.001306045
Gm47507	1	1.15E-08	1	-0.000340369	0.005526412	0.001305255
Gm45349	3.58167E-07	6.86E-02	1	-0.007530584	0.010230267	0.001303203
Zfyve28	1	5.37E-11	1	-0.000143779	0.006310151	0.001303147
Arl5c	1	1.19E-05	1	-0.000358217	0.003815648	0.001303147
4921517D1	1	6.82E-05	0.3468	-4.45712E-05	0.002567523	0.001300908
D330050G	1	1.00E-08	0.0031	4.28875E-05	0.002444245	0.001300117
Mybphl	1	6.13E-05	0.0031	0.000128662	0.001629497	0.001300117
Mmp25	1	3.80E-12	0.1071	3.84256E-05	0.005202571	0.001293799
Gm35853	1	1.63E-06	0.1071	-4.46189E-06	0.003111792	0.001293799
Ntng2	1	2.70E-05	1	-0.000676937	0.010711117	0.001291981
Gm42962	1	6.78E-19	1	-0.000211707	0.00769071	0.001291079
Ckap4	1	1.74E-09	1	-1.50695E-05	0.005246465	0.001291079
Gm21057	1	1.98E-06	1	-0.00092756	0.009288386	0.001290378
1700006J1	1	8.38E-03	0.1371	-9.91606E-05	0.001691136	0.001290289
1700021A0	1	1.03E-03	0.0074	0.0001242	0.001785834	0.001287391
Galnt12	1	4.73E-15	1	7.06578E-05	0.007332237	0.0012827
Nod2	1	1.96E-12	1	-0.000310867	0.00732966	0.001281251
Nlrp1a	1	6.46E-29	1	0.000308066	0.011003895	0.00128046
Trim29	1	5.47E-21	0.0184	0	0.006333597	0.001279669
Tnfrsf13b	1	2.71E-02	0.6021	0.00013144	0.001919273	0.001279011
Gm35239	1	3.08E-07	1	-0.000387719	0.003838547	0.001278353
Phlda2	1	4.90E-09	0.0589	0.000655014	0.003236095	0.001276113
Tal1	1	1.08E-03	1	0.000216314	0.003771966	0.001272546
Gm12089	1	2.85E-03	1	0.000143095	0.002690801	0.001267075
Faiml	1	2.07E-30	0.0075	0	0.008439691	0.001265494
Grip1os1	1	4.83E-03	0.0075	0	0.001222123	0.001265494
Pycard	0.011121233	9.53E-08	1	-0.004487252	0.016122266	0.001265191
Lman2	1	8.13E-10	1	0.012928012	-0.044757434	0.001258138
Igkv6-32	1	7.82E-07	0.0031	0	0.002036871	0.001251319
Gprin2	1	6.13E-05	0.0031	0	0.001629497	0.001251319
Hist1h2bf	1	4.83E-03	0.0031	0	0.001460421	0.001251319
Gm39228	1	1.00E+00	0.0031	0	0.000730211	0.001251319
Gm31597	1	1.00E+00	0.0031	8.57749E-05	0.000407374	0.001251319
Gm47480	1	1.00E+00	0.0031	0	0.000407374	0.001251319
Rubcnl	1	1.59E-08	1	-0.000250866	0.007047487	0.001248537
Cgas	1	5.71E-09	1	-0.000725969	0.008715005	0.001248124
Ckb	1	1.54E-12	1	-0.000175578	0.009070121	0.001243841
Adora2b	1	8.37E-03	0.1372	-1.33857E-05	0.002133122	0.00124149
Nfkb2	1	2.70E-02	1	-0.001248496	0.015012884	0.001240114

Calr4	1	1.00E+00	0.0352	3.39637E-05	0.000312675	0.001240041
Gm15524	1	1.00E+00	0.0352	-9.46987E-05	0.000312675	0.001240041
Grxcr1	1	5.43E-03	1	7.36549E-05	0.005240403	0.001239091
Loxl1	1	2.48E-03	1	0.0003617	0.004090182	0.001232175
Tnf	1	7.13E-23	0.0184	0	0.006894732	0.001230871
Kcnj10	1	1.07E-11	1	-0.000178669	0.005811889	0.001230866
Gask1b	1	4.50E-10	1	-0.000341223	0.006012524	0.001230156
Iglc1	1	9.70E-03	1	7.68511E-05	0.002042356	0.001224507
Atp8b2	1	1.03E-03	1	0.000436067	0.002949422	0.001220384
Gm35167	1	2.78E-03	0.7462	0.000144779	0.002160149	0.001217486
Gm12589	1	2.17E-14	0.0075	0	0.004500413	0.001216696
4930455G(	1	1.28E-26	0.1373	0.000130467	0.007316751	0.001213793
Bcl2l14	1	1.73E-02	1	-0.000340369	0.002460087	0.001207658
Far1os	1	1.52E-04	0.3185	0.000243939	0.002336809	0.001206867
Gm42743	1	5.06E-11	1	-0.00013927	0.004663456	0.001206209
Clec4e	1	8.75E-39	1	-0.000182253	0.014671345	0.0012053
Mkx	3.87415E-24	1.00E+00	1	-0.015303427	-0.00671218	0.001204213
A330033J0	1	1.88E-07	0.018	-4.73494E-05	0.002516045	0.001203969
Gm40457	1	1.63E-06	0.018	-4.73494E-05	0.002193209	0.001203969
Sfn	1	1.18E-02	1	-0.000714509	0.004444315	0.001197293
Trbc1	1	5.29E-06	1	-0.000168747	0.005925533	0.001197166
Gm29128	1	1.14E-03	0.1372	0.000329714	0.001894823	0.001192692
Agpat4	1	6.96E-22	1	-0.000814065	0.01989676	0.001186387
Lrrc4b	1	1.18E-07	1	-0.000139318	0.004032073	0.001183654
Anxa13	7.62714E-43	1.00E+00	1	0.012840922	0.002430694	0.001182853
Ccr7	1	2.74E-11	1	-0.000387719	0.005721989	0.001174484
Fbxw15	1	1.30E-10	0.0075	0	0.003258994	0.001167898
Hist1h4k	1	6.13E-05	0.0075	0.00017155	0.001748646	0.001167898
Gm43657	1	6.13E-05	0.0075	0	0.001629497	0.001167898
Olfr455	1	4.83E-03	0.0075	0	0.001341272	0.001167898
Tmem249	1	4.83E-03	0.0075	0.000471762	0.001222123	0.001167898
4930554I0(	1	3.85E-01	0.0075	0	0.000814748	0.001167898
Gm21962	1	1.00E+00	0.0075	0.000214437	0.000611061	0.001167898
Gm48281	1	1.00E+00	0.0075	0	0.000203687	0.001167898
Gm49698	1	1.00E+00	0.0075	4.28875E-05	0	0.001167898
Ccdc34os	1	8.74E-09	1	-0.000368872	0.005453402	0.001166237
Gm49871	1	1.27E-03	1	0.000304941	0.005464764	0.001160229
Gm11831	1	4.55E-03	1	-0.00035783	0.004845868	0.001159894
Ankrd13d	1	7.57E-08	1	0.000297723	0.004545397	0.001152263
4933432I0:	1	6.43E-12	1	-8.92378E-06	0.005524334	0.001150348
Grin1os	1	1.00E+00	0.0452	0	0.000611061	0.00114745
4930445B1	1	3.79E-02	1	0.000308404	0.003202684	0.001140784
Rncr4	1	5.44E-04	0.0184	0	0.00142581	0.001133275
Pea15a	1	6.73E-03	1	-0.003367794	0.013119598	0.001125746
Card9	1	7.85E-09	1	0.000298481	0.00436507	0.001125686
Tmem26	1	1.16E-08	1	-0.000168819	0.004716291	0.001124237
Gm11725	1	1.52E-04	0.7342	-0.000142048	0.002336809	0.001123446

Gm27011	1	3.13E-02	1	0.000135855	0.0021846	0.001122788
Ifit2	1	4.38E-21	1	-0.00110476	0.019458291	0.001121068
Gm14401	1	1.59E-02	1	0.001009156	0.00433667	0.001119805
Mdk	1	1.89E-03	1	-0.00081367	0.004794178	0.001118218
Gm34035	1	1.90E-07	1	0.000675979	0.007317248	0.001114455
Trim30c	1	9.81E-12	1	-0.000516645	0.011993574	0.001114196
Hic1	1	1.07E-11	1	-0.000108084	0.004278981	0.001112169
Slc16a3	1	2.88E-24	1	0.000140317	0.009976372	0.001100891
Celf4	1	4.22E-02	1	0.00041495	0.005385445	0.001100801
Tgtp2	1	1.99E-08	1	8.77899E-05	0.004530616	0.001100614
Syn1	1	2.46E-16	1	-0.000346563	0.009303272	0.001097649
Ifi213	1	2.12E-17	1	-0.000463799	0.018487754	0.001096105
Samd3	1	3.74E-02	1	-0.000342053	0.003501948	0.001090404
Asprv1	1	9.07E-04	1	-0.00014651	0.002408609	0.001090272
5430425K1	1	5.78E-03	1	-0.000335907	0.002626585	0.001088165
H2-DMb2	1	1.25E-07	1	-0.000665549	0.005130774	0.001087501
Pf4	1	7.60E-17	0.2598	-4.73494E-05	0.004875752	0.001085925
Gm26626	1	3.22E-17	0.0184	4.28875E-05	0.005411695	0.001084476
Gm48719	1	1.00E-08	0.0184	0	0.002563394	0.001084476
Cd8a	1	6.92E-06	0.0184	0.000128662	0.002309781	0.001084476
Gpr61	1	7.82E-07	0.0184	0	0.002036871	0.001084476
Gm34060	1	5.44E-04	0.0184	0.000128662	0.00142581	0.001084476
Slc5a8	1	5.44E-04	0.0184	0	0.00142581	0.001084476
Slc38a8	1	4.83E-03	0.0184	0	0.001222123	0.001084476
Cort	1	1.00E+00	0.0184	0	0.000611061	0.001084476
Platr27	1	1.00E+00	0.0184	0	0.000407374	0.001084476
A1bg	1	1.00E+00	0.0184	0	0.000203687	0.001084476
Spink1	1	1.00E+00	0.0184	0	0	0.001084476
Tmem173	1	1.21E-09	1	-4.72289E-05	0.005136807	0.001083813
Mak	1	1.20E-14	1	0.000298481	0.006759389	0.001076887
Cd164l2	1	9.71E-03	0.2	-9.46987E-05	0.001534798	0.001073199
Gm21750	1	9.71E-03	0.2	-9.46987E-05	0.001534798	0.001073199
A530064D0	1	3.68E-18	0.1111	0	0.005876296	0.001064029
1700029H1	1	6.13E-05	0.1111	4.28875E-05	0.001952333	0.001064029
Tmem45a2	1	1.04E-11	1	-1.78476E-05	0.003884344	0.001061921
Gm16191	1	1.28E-04	1	6.79274E-05	0.002254848	0.001061921
Arhgef6	1	7.07E-03	1	0.002362722	0.00973851	0.001060614
Cysltr1	1	1.08E-03	1	-0.00041449	0.003889283	0.00105723
Mroh5	1	7.20E-03	1	-0.000186667	0.002762601	0.001052883
Syt8	1	1.68E-12	0.0452	0	0.00385474	0.001049853
Tbx1	1	1.00E+00	0.0452	0	0.000611061	0.001049853
Tmem89	1	1.00E+00	0.0452	0	0	0.001049853
Gcdh	1	7.56E-69	1	0.00749657	-0.137847772	0.001049246
Dpep2nb	1	1.02E-05	1	0.000311134	0.005017097	0.001048325
Gm3848	1	4.74E-02	1	-0.001553008	0.008184186	0.00104066
Kctd12	1	4.72E-04	1	-0.000806284	0.007273227	0.001040056
Mybpc3	1	2.51E-06	1	-9.91606E-05	0.002863332	0.001040025

Cd24a	1	2.28E-06	0.4738	-8.92378E-06	0.003064442	0.001038576
Cd79b	1	1.16E-02	1	-0.000540422	0.004215965	0.001034017
Ptgs2	1	1.47E-11	0.6721	4.28875E-05	0.004308722	0.001029406
Gm8013	1	3.09E-07	1	6.79274E-05	0.003630731	0.001027298
Prune2	1	1.16E-02	1	-6.86598E-05	0.004669947	0.00102625
Gm15964	1	1.02E-07	1	-7.92476E-06	0.005304649	0.001021949
Akr1c18	1	1.97E-15	1	4.12288E-05	0.007030751	0.001017597
Nupr1	1	4.03E-06	1	-0.000561853	0.005049695	0.001016143
Gm35455	1	3.22E-17	0.1111	0.000128662	0.004923102	0.00101523
Hist1h2ad	1	6.56E-04	1	-6.51969E-05	0.002207498	0.001014572
4930422I2	1	1.52E-04	1	0.000173354	0.002070812	0.001012327
Ifi206	1	1.60E-19	1	-7.47101E-05	0.01279257	0.00101212
Gm8739	1	8.32E-04	1	2.33085E-05	0.003003477	0.001005534
Gm29686	1	1.12E-07	1	-0.000250132	0.00434062	0.001004744
Pi16	1	7.14E-03	1	-0.000300139	0.003449799	0.001004738
A130051J0	1	1.72E-03	1	0.000311866	0.002723861	0.001003295
Gm49548	1	6.13E-05	0.0452	0.000471762	0.001629497	0.001001055
Gm20531	1	4.83E-03	0.0452	0.000686199	0.001222123	0.001001055
Mrgprx2	1	4.31E-02	0.0452	0	0.001018435	0.001001055
1700028I1	1	3.85E-01	0.0452	0	0.000814748	0.001001055
Hsd3b1	1	1.00E+00	0.0452	8.57749E-05	0.000611061	0.001001055
Lrit3	1	1.00E+00	0.0452	0	0.000611061	0.001001055
Ankrd53	1	1.00E+00	0.0452	0.000128662	0	0.001001055
4930413F2	1	1.00E+00	0.0452	0	0	0.001001055
Gm47152	1	1.00E+00	0.0452	0	0	0.001001055
Gm10521	1	2.52E-24	1	-0.000198321	0.008120983	0.000995573
Col1a2	1	9.85E-13	1	-0.000256085	0.009827864	0.000994378
Klre1	1	3.30E-08	0.4738	0.000402151	0.003621927	0.000989778
BB365896	0.000519844	1.00E+00	1	0.007204924	0.002157794	0.000989452
Ms4a14	1	2.17E-14	0.273	0	0.004500413	0.000980607
Dppa3	1	7.82E-07	0.273	8.57749E-05	0.002598006	0.000980607
Pianp	1	5.44E-04	0.273	0	0.00142581	0.000980607
Gm26611	1	1.67E-02	1	0.000316328	0.001956462	0.000967223
Gm12703	1	6.13E-05	0.1111	0.000300212	0.001748646	0.000966432
Slc10a4	1	5.44E-04	0.1111	0	0.00142581	0.000966432
Odf3l1	1	4.83E-03	0.1111	0	0.001222123	0.000966432
P2rx5	1	1.07E-04	1	0.000264733	0.002805152	0.00096432
Il27ra	1	8.28E-04	1	1.80884E-05	0.003543828	0.000964314
Krt80	1	4.18E-02	1	-0.000189397	0.002477832	0.000958053
Gm48853	1	5.81E-03	1	0.000307405	0.002269137	0.000955945
Gm10684	1	8.77E-03	0.2598	-4.73494E-05	0.001616759	0.000953706
Gm15972	1	1.03E-03	0.2598	8.1313E-05	0.001582147	0.000953706
BC035044	1	1.64E-06	1	-0.000384988	0.005584069	0.000949805
Hrh2	1	4.64E-10	1	0.000162626	0.004236639	0.000947387
Lpar6	0.003139678	1.00E+00	1	-0.01506487	0.000187404	0.000945514
Meis3	1	2.59E-10	1	0.00017428	0.005415541	0.000936947
Gm48530	1	8.90E-03	1	-0.000324253	0.003348187	0.000923562

Crispld2	1	1.72E-02	1	-0.000282292	0.003028136	0.000923424
Gm28505	1	8.23E-03	1	0.000481926	0.003786929	0.000922235
F13a1	1	1.29E-04	1	-0.000528768	0.007050697	0.000918212
Slc7a9	1	1.00E-08	0.1111	0.00015375	0.002444245	0.000917634
Slc35g3	1	5.44E-04	0.1111	0.00017155	0.001664108	0.000917634
Gm36208	1	5.44E-04	0.1111	4.28875E-05	0.00142581	0.000917634
Siah3	1	3.31E-03	1	-8.30445E-05	0.002663774	0.000908596
Rab44	1	7.27E-11	1	2.95018E-05	0.00506961	0.000907805
Gm21859	1	3.14E-04	1	0.00018152	0.003884871	0.000906624
Gm26887	1	1.53E-04	1	-8.92378E-06	0.002503307	0.000906356
4930471E1	1	3.28E-09	1	-0.000245671	0.004387969	0.000899426
Tlr6	1	1.72E-13	1	2.0578E-05	0.0055204	0.000896528
Aph1c	0.005994429	5.90E-19	1	-0.003054134	0.018727879	0.000893855
Alox5ap	1	5.48E-20	1	-0.000396642	0.009197456	0.000892495
Hlx	1	1.83E-09	1	-0.000328595	0.006105989	0.000888275
Nes	1	2.10E-05	1	0.000347634	0.003750762	0.000885245
Sirpb1a	1	4.43E-04	1	-0.000198321	0.002602135	0.000883801
Gm10425	1	6.13E-05	0.273	0	0.001629497	0.000883011
B930018H1	1	4.83E-03	0.273	8.57749E-05	0.001341272	0.000883011
Gm30198	1	7.59E-17	0.6306	3.84256E-05	0.005590648	0.000870284
Gm5086	1	3.13E-24	1	-0.000584236	0.01192093	0.000865542
Gm39321	1	1.63E-06	1	-4.46189E-06	0.003446624	0.000864012
Slfn3	1	7.82E-09	1	0.000338758	0.005067583	0.0008619
Clec2g	1	9.55E-03	1	-8.75064E-05	0.002616424	0.000861246
Gm11670	1	9.57E-03	1	0.000588181	0.002497275	0.000861246
Sbspon	1	5.98E-03	1	0.000183277	0.003099057	0.000860583
Gm14419	1	3.36E-05	1	-0.000193593	0.005186593	0.000859256
Def6	1	1.26E-06	1	-0.000704779	0.007861318	0.000850415
Hcar2	1	3.68E-18	0.6721	0	0.00648273	0.000848388
Serpinb1b	1	1.00E-08	0.6721	0	0.002682544	0.000848388
9430041J1	1	4.83E-03	0.6721	4.28875E-05	0.001341272	0.000848388
F730311O2	1	1.13E-04	1	-0.000621735	0.00443239	0.000845278
Cd79a	1	1.84E-02	1	-0.000735014	0.005006331	0.000839599
Ccrl2	1	7.58E-13	1	0.000205514	0.004977536	0.00083711
Scd4	1	1.41E-05	1	-4.73494E-05	0.00222782	0.000835661
Gm50105	1	1.63E-06	1	0.0001242	0.002193209	0.000835661
Comp	1	8.77E-03	1	0.000252863	0.00137846	0.000835661
9930022D1	1	6.92E-06	0.6721	0	0.002190632	0.000834213
Hist1h3i	1	5.44E-04	0.273	0.000686199	0.001544959	0.000834213
Gm34078	1	5.44E-04	0.273	0	0.00142581	0.000834213
Depp1	5.8595E-207	1.70E-07	1	0.103621184	-0.026089204	0.000830676
Gm38405	1	6.28E-05	1	0.000298481	0.003766005	0.000826624
Slc14a1	1	3.14E-04	1	4.29602E-05	0.004126433	0.00082372
Gm47416	1	3.30E-08	1	3.39637E-05	0.00287607	0.000822935
Ear2	1	5.44E-04	1	0	0.00142581	0.000813765
Tnip3	1	6.27E-03	1	0.000239477	0.001966623	0.000811658
Gm50130	1	6.28E-03	1	0.000264565	0.001847474	0.000811658

Ttc12	1	1.10E-03	1	-0.000634927	0.006025915	0.000808669
F730043M	1	1.27E-04	1	-9.98454E-05	0.005007815	0.000808548
Rdh12	1	6.52E-04	1	-0.000236747	0.003041544	0.000805339
Pilrb2	1	1.16E-08	1	2.73045E-06	0.00468533	0.000804727
Slc36a2	1	2.82E-16	1	0	0.004788638	0.00079959
A430108G(	1	1.14E-09	0.6721	0	0.002886231	0.00079959
Iglc2	1	1.06E-04	1	0.000181568	0.003547745	0.000790552
Ppm1j	1	1.53E-04	1	0.000419951	0.00229962	0.000788312
Ece2	1	5.24E-03	1	-0.000349293	0.002534464	0.000779274
Gm20647	1	6.30E-05	1	4.1156E-05	0.003227486	0.000777825
Ctsw	1	5.96E-03	1	-0.00020271	0.003218207	0.000777162
Gm34662	1	1.14E-03	1	7.23893E-05	0.001894823	0.000775586
Ifi205	1	3.15E-04	1	-0.000214365	0.004380047	0.000774922
Adora1	1.75454E-30	6.35E-31	1	-0.03474072	0.082313801	0.000772872
Slc2a6	1	2.06E-14	1	-0.000202783	0.006748008	0.000767206
Gbgt1	1	1.49E-06	1	-0.000155434	0.002974897	0.000765757
Bcl2a1a	1	1.30E-10	1	0	0.002970769	0.000764967
Gm17111	1	6.56E-04	1	2.0578E-05	0.002207498	0.000764308
Lancl3	1	2.72E-02	1	-6.51969E-05	0.001800124	0.000764308
Metrnl	1	1.81E-06	1	6.73789E-05	0.01132049	0.000762726
Runx2os1	1	1.23E-03	1	-9.46987E-05	0.001857634	0.000753689
Il1bos	1	1.03E-03	1	-4.46189E-06	0.001820446	0.00075224
Kcnj2	1	1.49E-06	1	-0.000115156	0.003185498	0.000752235
Treml1	1	4.20E-19	0.6721	0	0.005399699	0.000750791
Hmx1	1	4.80E-20	0.6721	0	0.00500764	0.000750791
Gm38335	1	2.48E-15	0.6721	0	0.00443119	0.000750791
Hmcn1	8.64978E-06	1.00E+00	1	0.007571154	0.002715006	0.000746672
9930038B1	1	4.68E-02	1	-0.000173281	0.002174439	0.000743202
4921509O(	1	7.33E-11	1	-5.62731E-05	0.003643469	0.000740963
Itgb7	1	2.11E-05	1	-0.001095962	0.007772388	0.000733622
Adgrg5	1	1.34E-02	1	-0.000167015	0.003174312	0.000732578
Gm12968	1	1.75E-05	1	-0.000189397	0.002577684	0.000728236
Ak8	1	2.73E-20	1	-0.000235748	0.011426974	0.000727234
Prrc2a	2.7061E-08	6.21E-04	1	0.029993722	-0.025597375	0.00072635
Samd11	1	9.98E-03	1	0.000455719	0.002204251	0.000725333
Gm17268	1	1.03E-03	1	-3.21595E-05	0.00228466	0.000724543
Gm13270	4.23664E-09	1.00E+00	1	-0.009399552	-0.004288495	0.000723533
Il2rg	1	1.73E-13	1	-0.000351024	0.008121988	0.000722887
Xlr4a	0.008879199	1.21E-26	1	0.001326287	0.006790897	0.000716168
4930412L0	1	1.14E-09	1	0	0.003531904	0.000716168
Acta2	1	5.44E-04	1	4.28875E-05	0.001664108	0.000716168
Zbtb3	1	4.31E-02	1	8.57749E-05	0.001018435	0.000716168
Gm2164	1	3.10E-24	1	-0.000243866	0.011831569	0.000715505
Mapk12	1	3.46E-06	1	0.001614692	0.009183625	0.000714873
Inf2	3.06047E-09	1.00E+00	1	-0.018344499	-0.004153064	0.000712326
Dbn1	1	8.92E-03	1	-0.000195591	0.003313575	0.000707921
Rab19	1	7.59E-13	1	7.68511E-05	0.004251953	0.000704891

CN725425	1	1.88E-05	1	-9.46987E-05	0.002587845	0.000704891
Npc1l1	1	1.21E-04	1	-4.73494E-05	0.001785834	0.000703442
Gm49249	1	2.71E-02	1	0.00014663	0.002249024	0.000701988
Glipr2	1	1.46E-05	1	0.000129661	0.00500762	0.000699541
Chil1	1	1.15E-12	1	-0.000245671	0.007278182	0.00069796
Rnf24	3.09235E-06	1.00E+00	1	-0.019232718	-0.001477917	0.000695778
2010103J0	1	9.58E-03	1	0.000255593	0.002378126	0.000694404
Fam241a	8.62662E-07	1.00E+00	1	-0.015257764	0.02045587	0.000693495
B3gnt5	1	3.84E-03	1	-0.000125764	0.00312954	0.00069295
Snn	1	4.12E-02	1	-0.000418952	0.003522748	0.000688876
Zfp598	1	3.13E-12	1	0.014183481	-0.043571088	0.000683468
P2ry10b	1	2.36E-05	1	-0.000726284	0.004787616	0.000682463
Rnase6	1	1.12E-07	1	0.000108157	0.004297608	0.000677984
Gm12766	1	3.34E-03	1	-0.000651212	0.003747727	0.000672963
Slc9a5	1	1.73E-02	1	0.000302943	0.002460087	0.000672507
Gm44993	1	2.16E-16	1	-9.91606E-05	0.006107011	0.000671717
Gm41300	1	2.51E-06	1	-0.000142048	0.002625033	0.000671717
Cd300c	1	6.92E-06	1	0.000128662	0.002071482	0.00066737
Gm35248	1	5.44E-04	1	0.00015375	0.001544959	0.00066737
Ly6g6d	1	4.83E-03	1	0.00015375	0.001341272	0.00066737
Gm17173	1	1.21E-09	1	-2.9429E-05	0.005255956	0.000666706
Cpe	1	2.14E-07	1	0.000124566	0.004572338	0.000666658
Mcub	1	5.37E-11	1	-0.000171477	0.00660529	0.000656878
Dennd2a	1	1.20E-04	1	0.000360323	0.008693993	0.000656713
Tpm4	1	1.27E-06	1	-0.005355545	0.025883714	0.000656428
Axdnd1	1	1.52E-03	1	0.000413758	0.004625769	0.000649953
Gm2396	1	1.34E-02	1	-0.000139318	0.002844562	0.000648504
Xlr	1	9.66E-05	1	4.56656E-05	0.002887783	0.000646264
Tspan32	1	4.30E-09	1	-0.000283976	0.005949456	0.0006456
Camkk1	1	1.71E-03	1	-0.000202783	0.002824045	0.000634987
Gm49173	1	4.43E-04	1	5.90036E-05	0.002482985	0.000633538
Rnf208	1	2.78E-03	1	-0.000284096	0.002279298	0.000633538
Arl11	1	2.17E-14	1	0	0.004142965	0.000632747
Gm11527	1	4.31E-02	1	8.57749E-05	0.001018435	0.000632747
Fam78b	1	1.09E-10	1	-0.000896834	0.010337406	0.000631213
St6galnac4	1	1.00E-07	1	-0.000199885	0.006041773	0.000625943
Catip	1	9.71E-03	1	0.000119739	0.001773097	0.00062147
1700099I0!	1	3.81E-12	1	2.06257E-05	0.003772779	0.000620021
Wsb2	0.030190933	1.00E+00	1	0.01644447	0.010185722	0.000612274
Hoxaas3	1	9.94E-03	1	0.000327056	0.002561699	0.000607289
Gm26520	1	1.31E-11	1	-8.38503E-05	0.005710009	0.000607284
Rab37	1	5.00E-14	1	-0.000346563	0.008614974	0.000603394
Gm38411	1	9.10E-07	1	0.000425412	0.004348877	0.000603394
Gm36043	1	2.84E-04	1	-0.000288558	0.002877622	0.000600364
Timp1	1	5.44E-04	1	0.000110862	0.001783258	0.000598124
Gm16140	1	1.39E-05	1	2.0578E-05	0.002734022	0.000597466
Shisa3	1	4.54E-05	1	-0.000117008	0.003081309	0.000586188

Il9r	1	1.63E-06	1	8.1313E-05	0.002788955	0.000585398
Gm15728	1	8.77E-03	1	-4.73494E-05	0.001616759	0.000585398
Gm36569	1	7.82E-07	1	0	0.003074603	0.000583949
Gm16169	1	4.31E-02	1	0.000128662	0.001018435	0.000583949
Gm26812	1	4.31E-02	1	0	0.001018435	0.000583949
Lif	1	4.31E-02	1	0	0.001018435	0.000583949
Gm32098	1	1.27E-04	1	-6.0735E-05	0.003208042	0.000575569
Gm16287	1	1.88E-05	1	-5.18112E-05	0.002587845	0.000572671
Cpxm1	1	9.71E-03	1	0.000162626	0.001773097	0.000572671
Nos1	1	1.74E-12	1	0.000218161	0.008375335	0.000566249
Celsr3	1	1.67E-02	1	-0.000155434	0.002433059	0.000564292
Ace	1	2.71E-02	1	-9.28945E-05	0.002249024	0.000562001
Gm11851	1	1.75E-05	1	0.000325252	0.002577684	0.000561394
Gm17546	1	3.18E-04	1	0.00071397	0.003050826	0.000552356
Gm20406	1	5.63E-10	1	0.000273441	0.004027944	0.000550116
Unc93a	1	1.66E-02	1	0.000487878	0.002671358	0.000550116
AI427809	1	1.91E-13	1	6.7975E-05	0.003939278	0.000549326
Stfa2l1	1	4.83E-03	1	0	0.002087129	0.000549326
Gm40117	1	6.13E-05	1	4.28875E-05	0.001748646	0.000549326
Bex1	1	4.83E-03	1	0	0.001222123	0.000549326
Ccl2	1	1.59E-13	1	4.02298E-05	0.005828277	0.000547872
Gcgr	1.67994E-80	9.91E-29	1	0.110594987	-0.112669554	0.0005406
9330158H0	1	3.13E-02	1	0.000607617	0.0021846	0.000538839
Pigp	0.000351628	1.00E+00	1	-0.008710539	0.002547947	0.000538443
Gm36161	1	2.97E-21	1	-5.18112E-05	0.006830994	0.000538048
Slc27a6	1	1.03E-03	1	0.000209975	0.002597264	0.000536599
Gdpd5	1	1.63E-07	1	0.000359555	0.008678342	0.000533404
Gm7008	1	5.75E-04	1	-0.000393864	0.003387952	0.000529801
1700128A0	1	4.68E-02	1	-1.73144E-06	0.002174439	0.000527561
Slamf8	1	5.24E-32	1	-7.85349E-05	0.010202626	0.000526771
Ceacam16	1	1.59E-13	1	-6.0735E-05	0.004768316	0.000526771
Jph2	1	6.26E-03	1	0.000325252	0.00243957	0.000526771
Tnfrsf8	1	8.37E-03	1	0.000292371	0.002348804	0.000525322
Gm5547	1	1.52E-04	1	-5.62731E-05	0.00209851	0.000525322
Gm13708	1	4.43E-04	1	-8.74587E-05	0.002482985	0.000515493
Gm26535	1	1.30E-10	1	0	0.004043112	0.000514703
Arfgef3	1	1.94E-06	1	0.00032079	0.003971791	0.000514044
Trim67	1	6.55E-04	1	-0.000150972	0.002326648	0.000514044
Ndufs3	1	7.98E-12	1	0.001339901	-0.051575715	0.000513247
Bend6	1	3.77E-02	1	-0.000195591	0.002906201	0.000506456
Gm34084	1	3.29E-08	1	-8.92378E-06	0.003948414	0.000503425
Traf3ip3	1	1.04E-13	1	0.000523348	0.014567127	0.000502793
4833407H1	1	3.22E-17	1	0.000239525	0.006183818	0.000500528
Gm46189	1	1.14E-09	1	0	0.003124529	0.000500528
Srpx	1	8.86E-08	1	0.00017155	0.002359707	0.000500528
Prss30	1	6.13E-05	1	4.28875E-05	0.001867795	0.000500528
Lrp8os2	1	6.92E-06	1	0	0.001833184	0.000500528

Cdhr3	1	6.13E-05	1	0	0.001748646	0.000500528
Gm26685	1	6.13E-05	1	0	0.001629497	0.000500528
Lrtm2	1	5.44E-04	1	0	0.001544959	0.000500528
4933417O1	1	4.83E-03	1	0.00017155	0.001341272	0.000500528
Gm35520	1	4.83E-03	1	4.28875E-05	0.001222123	0.000500528
Gm13074	1	4.83E-03	1	0	0.001222123	0.000500528
Gm13073	1	4.31E-02	1	0	0.001137585	0.000500528
Gm12977	1	4.31E-02	1	0.000128662	0.001018435	0.000500528
Speg	1	2.54E-04	1	-0.000711947	0.004492227	0.000497741
Gm10863	1	1.83E-10	1	-0.000301944	0.005571878	0.000492939
3300005D0	1	1.88E-05	1	5.90512E-05	0.002503307	0.00048925
Kcnj6	1	9.43E-07	1	-0.000549346	0.006591845	0.000484199
Gm17455	1	4.31E-02	1	0	0.001256734	0.00048008
Dhx16	4.22083E-08	1.00E+00	1	0.027479459	-0.016475617	0.000467485
Ctbp2	1	1.91E-13	1	8.57749E-05	0.003700979	0.000465905
Gm43351	1	1.47E-11	1	0	0.003055306	0.000465905
Gm48277	1	4.83E-03	1	0	0.002106094	0.000465905
Best1	1	5.44E-04	1	0	0.001664108	0.000465905
Gm15448	1	4.83E-03	1	4.28875E-05	0.001341272	0.000465905
Dennd2d	3.58613E-05	1.00E+00	1	-0.010115335	-0.000953442	0.000465247
Dusp23	1	1.75E-02	1	0.006602584	-0.012695588	0.000461016
Trac	1	1.54E-04	1	-8.92378E-06	0.002180471	0.000454627
Tnfaip8l2	1	8.35E-04	1	-0.000608276	0.004899708	0.000447956
Ndst3	1	2.48E-02	1	-0.000269254	0.004368496	0.0004463
4930503O0	1	6.01E-10	1	2.95018E-05	0.003439782	0.000441901
Gm19434	1	3.97E-08	1	-1.33857E-05	0.002913258	0.000441901
Klhl14	1	4.32E-02	1	-0.000136587	0.003502426	0.000438
Zan	1	4.32E-02	1	-0.000274126	0.003174104	0.000435102
Gm46606	1	1.88E-05	1	-9.46987E-05	0.003395624	0.000432684
Tnni2	1	1.30E-10	1	0	0.003531904	0.000431282
Syngr1	1	7.75E-20	1	0.000192128	0.006911598	0.000430623
Gm45572	1	4.41E-03	1	-0.000718165	0.003998662	0.000429753
Fcer2a	1	1.08E-04	1	-0.000806598	0.006518019	0.000428299
Bst1	1	1.07E-13	1	4.56179E-05	0.006979796	0.000422244
Klhl38	1	8.77E-03	1	8.1313E-05	0.00149761	0.000418555
Gm50334	1	1.14E-09	1	8.57749E-05	0.003328217	0.000417106
Gm35037	1	5.44E-04	1	4.28875E-05	0.002190632	0.000417106
Gm31063	1	6.92E-06	1	0	0.001952333	0.000417106
Sgo2b	1	6.13E-05	1	0.0003431	0.001629497	0.000417106
A430048G1	1	5.44E-04	1	0.000128662	0.001544959	0.000417106
A630035G1	1	5.44E-04	1	0	0.001544959	0.000417106
Adam30	1	5.44E-04	1	0	0.001544959	0.000417106
Fbxw20	1	4.31E-02	1	0	0.001018435	0.000417106
Gm26877	1	1.97E-05	1	-5.62731E-05	0.002540496	0.000407278
Gm45155	1	2.29E-06	1	0.000248401	0.002349546	0.000405829
Treml2	1	4.78E-07	1	1.00181E-05	0.004547215	0.000400301
Lad1	1	6.69E-07	1	-0.001931479	0.010704387	0.000398364

Gm13479	1	6.26E-03	1	-0.00014651	0.002612296	0.000394551
Slc7a6	1	2.11E-05	1	0.000148387	0.003723065	0.000391643
Trem3	1	4.96E-11	1	-0.000250132	0.006381473	0.000386172
Trem1	1	1.88E-07	1	-4.73494E-05	0.003738499	0.000383932
Fam170b	1	5.44E-04	1	0	0.00142581	0.000382483
Tubb1	1	4.31E-02	1	0	0.001375883	0.000382483
Gm19385	1	4.31E-02	1	0	0.001256734	0.000382483
Cd300ld3	1	4.83E-03	1	0	0.001222123	0.000382483
H2-Oa	1	4.31E-02	1	0	0.001018435	0.000382483
Trim43a	1	1.07E-02	1	0.000170817	0.003515359	0.000378715
Col3a1	1	1.88E-10	1	0.000853421	0.013106426	0.000376512
Prkg1	1.37044E-76	1.00E+00	1	0.05217627	0.035436206	0.00037146
Ankdd1a	1	1.89E-33	1	-9.46987E-05	0.010124599	0.000371206
Marcksl1	1	2.82E-03	1	0.000115397	0.003616298	0.000370542
Mag	1	1.03E-03	1	-4.46189E-06	0.001582147	0.000369757
Timp2	1	6.32E-05	1	0.000144141	0.005559072	0.000369545
Vwa3a	1	1.92E-04	1	-0.000149095	0.00387337	0.00036843
Tmem229a	1	6.55E-04	1	0.00014924	0.002445797	0.000361377
Kctd4	1	1.74E-02	1	0.003405736	0.009264328	0.000359231
Nxpe5	1	1.52E-04	1	-5.62731E-05	0.00209851	0.000358479
Gm47448	1	1.66E-02	1	5.90036E-05	0.002924971	0.000348651
5830432E0	1	5.18E-03	1	8.99654E-06	0.003629036	0.000348646
4930512J1	1	7.82E-07	1	0	0.00300538	0.00034786
Phf11a	1	6.35E-19	1	-6.51969E-05	0.006265926	0.000347202
Dgkg	1	1.12E-08	1	-0.001811589	0.011216545	0.000340633
Gm30329	1	1.88E-07	1	-4.73494E-05	0.003108141	0.000335134
Gm21860	1	1.30E-08	1	0.00028441	0.007336014	0.000335054
Olfr592	1	1.00E-08	1	0	0.002563394	0.000333685
Rab11fip4c	1	6.13E-05	1	4.28875E-05	0.001867795	0.000333685
Gm15961	1	6.92E-06	1	0	0.001833184	0.000333685
Ush1c	1	5.44E-04	1	0	0.001783258	0.000333685
Cryba4	1	4.83E-03	1	4.28875E-05	0.001460421	0.000333685
Gm36548	1	5.44E-04	1	0	0.00142581	0.000333685
5330411J1	1	4.83E-03	1	0	0.001341272	0.000333685
Lrrc75b	1	4.83E-03	1	0	0.001341272	0.000333685
Gm48362	1	4.31E-02	1	0.000128662	0.001256734	0.000333685
Glp1r	1	4.31E-02	1	0.00017155	0.001018435	0.000333685
Gm14225	1	4.31E-02	1	0	0.001018435	0.000333685
Gsx1	1	4.31E-02	1	0	0.001018435	0.000333685
Skint3	1	2.67E-07	1	-0.000193859	0.00424105	0.000312579
Ffar1	1	1.27E-04	1	-6.0735E-05	0.002731445	0.00031113
Lrrc6	1	4.20E-02	1	0.000110815	0.001762936	0.00031113
Lyl1	1	2.27E-02	1	-0.000441261	0.003508653	0.00030723
Egr3	1	1.30E-10	1	0	0.003597476	0.000299062
Gm27007	1	6.13E-05	1	0.000214437	0.002344393	0.000299062
Slco4c1	1	4.31E-02	1	0	0.001645143	0.000299062
Gm39459	1	4.31E-02	1	0	0.001137585	0.000299062

Txndc2	1	4.31E-02	1	0	0.001137585	0.000299062
Nectin4	1	4.90E-09	1	8.75791E-05	0.003804144	0.000296159
Pglyrp1	1	5.19E-03	1	-0.000195543	0.00339947	0.000292922
Nkain1	1	9.71E-03	1	0.000205514	0.001653947	0.000287785
Adam33	1	2.73E-06	1	0.000231625	0.006424345	0.000286909
Map7d3	1	1.21E-04	1	0.0001242	0.002262432	0.000286336
Gm20513	1	1.63E-06	1	0.000252863	0.002193209	0.000286336
Ism1	1	4.20E-02	1	0.000168892	0.001616089	0.000283433
Gm16239	1	4.68E-02	1	0.000384256	0.002174439	0.000277298
Eno2	1	1.52E-04	1	2.95018E-05	0.002336809	0.000275058
Gm27201	1	6.54E-04	1	-0.000108084	0.002564946	0.000263781
Gm4793	1	2.71E-02	1	0.000431653	0.002122961	0.000263781
Lipn	1	1.68E-12	1	8.57749E-05	0.003497292	0.000250264
Olr1	1	8.86E-08	1	4.28875E-05	0.003224714	0.000250264
Zfp469	1	7.82E-07	1	4.28875E-05	0.002394319	0.000250264
Dpep2	1	7.82E-07	1	0	0.002036871	0.000250264
Guca2a	1	5.44E-04	1	0	0.00142581	0.000250264
Grhl3	1	4.31E-02	1	0.000214437	0.001137585	0.000250264
Slfn5os	1	4.31E-02	1	6.7975E-05	0.001137585	0.000250264
Gm30275	1	4.31E-02	1	0.000128662	0.001018435	0.000250264
Gm11537	1	4.31E-02	1	8.57749E-05	0.001018435	0.000250264
Pelo	1	4.31E-02	1	0	0.001018435	0.000250264
BC147527	1	2.72E-02	1	-0.000276952	0.002074479	0.000229486
Lexm	1	2.73E-05	1	-0.000634243	0.006236512	0.000226382
Lman1l	1	3.30E-03	1	-0.000371482	0.004153656	0.000220905
Tspan2	1	3.40E-06	1	4.74949E-05	0.0060094	0.000220063
Gm45463	1	2.13E-07	1	5.90036E-05	0.003416883	0.000216431
AI839979	1	8.77E-03	1	0.00029575	0.001735908	0.000202914
Pdlim2	1	5.21E-03	1	-0.000195543	0.002891911	0.000195325
Ryr1	1	1.03E-03	1	2.23822E-05	0.003040873	0.000191632
Clca3a1	0.025164787	1.00E+00	1	0.004012623	0.006715602	0.000182752
Gm50295	1	1.95E-06	1	-0.000193859	0.003141396	0.000180359
Phgdh	1	5.78E-03	1	0.000108157	0.003006262	0.000177456
Fbxw4	1	1.85E-11	1	0.003423282	-0.052793921	0.000171312
A530032D1	1	4.59E-10	1	-0.000159896	0.00454173	0.000169082
Gm29264	1	1.21E-04	1	-4.46189E-06	0.002466119	0.000168291
Ticam2	1	2.17E-14	1	4.28875E-05	0.0047041	0.000166843
Trim15	1	2.17E-14	1	0.00017155	0.003666368	0.000166843
Gm454	1	8.86E-08	1	0	0.002359707	0.000166843
4933402J0	1	5.44E-04	1	0.000128662	0.001544959	0.000166843
Gm38397	1	4.31E-02	1	0	0.001495033	0.000166843
Psd2	1	4.83E-03	1	4.28875E-05	0.001460421	0.000166843
Dok7	1	5.44E-04	1	0	0.00142581	0.000166843
Gm13134	1	5.44E-04	1	0	0.00142581	0.000166843
Gm1720	1	4.83E-03	1	4.28875E-05	0.001341272	0.000166843
6430531B1	1	4.83E-03	1	0	0.001222123	0.000166843
Gm35657	1	4.83E-03	1	0	0.001222123	0.000166843

Gm44850	1	4.31E-02	1	0	0.001137585	0.000166843
B020031H(	1	4.31E-02	1	0	0.001018435	0.000166843
Gm37229	1	1.23E-05	1	-0.000260426	0.00650968	0.000162204
Rgs18	1	8.37E-03	1	-9.91606E-05	0.002317843	0.000157014
Gm39822	1	2.29E-06	1	-2.67237E-05	0.002587845	0.000155565
Gm36486	1	3.22E-17	1	0.000128662	0.004634877	0.00013222
Gm38171	1	4.83E-03	1	0	0.00157957	0.00013222
Mrgprg	1	5.44E-04	1	0	0.00142581	0.00013222
Kctd19	1	4.31E-02	1	0	0.001137585	0.00013222
Ttc36	1	1.58E-05	1	0.001896211	-0.026744652	0.000115569
Gm5608	1	1.03E-03	1	-4.46189E-06	0.001582147	8.48702E-05
Tmem179	1	6.92E-06	1	0	0.00227517	8.34213E-05
Creg2	1	6.13E-05	1	0	0.001629497	8.34213E-05
Gm15413	1	4.83E-03	1	0	0.001460421	8.34213E-05
Gm48837	1	4.83E-03	1	0	0.001341272	8.34213E-05
Plcz1	1	4.83E-03	1	0	0.001222123	8.34213E-05
Gm39458	1	4.31E-02	1	0	0.001137585	8.34213E-05
Epha8	1	4.31E-02	1	0	0.001018435	8.34213E-05
Gm43401	0.009049283	6.32E-03	1	-0.003830134	0.008258172	7.78797E-05
Il1rl2	1	1.62E-09	1	-0.000821391	0.012460103	6.61056E-05
Ifit1bl1	1	1.71E-03	1	-0.000273368	0.003119184	5.79632E-05
Ntn1	1	3.08E-05	1	0.001483842	0.010773551	4.69496E-05
Ppp1r1c	1	3.62E-05	1	-0.000611933	0.004410055	4.60221E-05
Prkag3	0.036185266	1.00E+00	1	-0.002849546	-0.002874974	3.39162E-05
Pou3f1	1	1.14E-03	1	-0.000142048	0.002252271	2.47944E-05
Alox5	1	1.93E-03	1	-0.000653701	0.008174451	1.74398E-05
Nxpe4	1	2.34E-12	1	0.00209633	0.020457825	1.60276E-05
Dmrt1	1	1.34E-02	1	-0.000396642	0.002844562	1.57565E-05
BC023719	1	1.47E-11	1	0.00017155	0.003174456	0
Sbk2	1	6.92E-06	1	8.57749E-05	0.002221593	0
Cstdc4	1	4.31E-02	1	0	0.001764292	0
Gm33045	1	5.44E-04	1	0	0.00142581	0
Ms4a2	1	4.31E-02	1	0	0.001375883	0
Pip5k1bos	1	4.31E-02	1	0	0.001256734	0
4930500L2	1	4.83E-03	1	8.57749E-05	0.001222123	0
Fam124b	1	4.31E-02	1	0	0.001137585	0
Mcoln3	1	4.31E-02	1	0	0.001137585	0
Ring1	1	1.75E-03	1	0.007948143	-0.019099542	-2.84744E-06
Shisa4	1	9.69E-03	1	0.000291288	0.002130545	-1.12775E-05
Clec5a	1	1.38E-05	1	-0.000108084	0.003687216	-2.1106E-05
2310043M	0.013691626	1.00E+00	1	-0.00336678	0.00082442	-2.41267E-05
Gbp9	1	2.03E-03	1	-4.63559E-05	0.003580733	-3.55637E-05
Rhox8	1	2.71E-02	1	-2.23094E-05	0.002038423	-6.99043E-05
Smu1	1	1.79E-02	1	0.005075206	-0.020912036	-7.09699E-05
Pnp	9.60379E-07	1.00E+00	1	-0.014821169	0.002735401	-7.48629E-05
Rab3il1	1	3.72E-11	1	-0.000888621	0.009692598	-7.69146E-05
Gm4131	1	3.31E-08	1	-0.000931411	0.011637811	-0.000102708

Nrg3	1	2.77E-03	1	-0.000367534	0.006526247	-0.000112149
Timd4	1	1.97E-03	1	-0.001060098	0.007977347	-0.00012203
Gm13986	1	2.53E-06	1	-0.000196276	0.009782426	-0.000142128
Xlr3b	0.068958456	6.37E-10	1	0.00146824	0.004109905	-0.000153326
Anxa1	1	1.85E-10	1	-0.00018426	0.012274001	-0.000166538
Gm15494	0.001950359	1.00E+00	1	0.005254057	0.005761987	-0.000190391
Renbp	1	4.26E-10	1	-0.002617668	0.021636295	-0.000224929
Tfpt	1	3.28E-02	1	0.002949383	-0.013299091	-0.00022926
Il34	0.008972397	1.00E+00	1	0.003366757	0.001376161	-0.000247596
Mamld1	1	1.34E-04	1	-0.000187111	0.006970169	-0.000259392
Gtsf1	1	8.97E-04	1	-0.000601109	0.003943055	-0.0002661
Hbb-bt	1	5.98E-05	1	-0.001057957	0.006686285	-0.000285514
Lingo4	1	3.40E-05	1	-0.003212992	0.010514745	-0.000294999
Sel1l2	3.78214E-11	1.00E+00	1	-0.00778642	-0.001105429	-0.000310856
Olfr56	0.031114141	1.67E-09	1	-0.003917716	0.018240646	-0.000323969
Tns4	1	3.27E-03	1	-0.000231286	0.00351123	-0.000338244
Eef2k	1.72041E-07	1.00E+00	1	-0.014223864	-0.00062973	-0.000341605
Saa2	1	5.01E-06	1	0.000266876	0.007494369	-0.000403831
1700025F2	0.021976947	1.00E+00	1	-0.001479411	-0.000761188	-0.000444884
Gm15091	1	4.59E-03	1	0.000828093	0.005389365	-0.000447203
Cited2	4.44551E-57	1.00E+00	1	0.041175997	-0.011797229	-0.000483123
Noxred1	1	2.10E-03	1	-0.001969102	-0.013155039	-0.000500786
Hba-a1	0.00214049	5.11E-04	1	-0.006058014	0.014224334	-0.000507276
Prkcd	1	3.49E-21	1	-0.002968884	0.080594229	-0.00051155
Ints6l	1	3.53E-04	1	0.003457566	0.016256885	-0.000532395
9530026P0	2.18478E-32	1.00E+00	1	0.01755824	0.000717719	-0.000559459
Cpne8	1.30851E-22	1.00E+00	1	0.022405032	0.007597785	-0.000563724
Cdk5r1	0.158583675	3.59E-02	1	-0.005409373	-0.009370832	-0.000563801
Fhl1	0.029168916	1.00E+00	1	0.00262203	0.000969001	-0.000572758
Clk3	0.002380333	1.24E-07	1	0.021186093	-0.036947449	-0.000596543
Ptpn12	9.31337E-08	1.00E+00	1	0.034794173	0.041597027	-0.000601425
Dot1l	0.648103925	1.60E-05	1	0.019120852	-0.032768189	-0.000616509
Agmat	0.05850355	4.36E-44	1	0.024665252	-0.1184431	-0.000622538
Ptk7	0.008043497	1.00E+00	1	-0.003241774	0.000269526	-0.000662149
Gm49198	1	1.17E-02	1	-0.000176311	0.005154835	-0.000675045
Klf9	7.63097E-51	1.00E+00	1	-0.099683504	-0.023694127	-0.000679504
Fgr	0.01337334	1.77E-23	1	-0.006413126	0.051456492	-0.000688924
Pcdh7	1.01825E-06	1.00E+00	1	0.006073501	0.003654968	-0.000722476
Sqstm1	5.06744E-07	6.83E-11	1	-0.037647327	0.089695538	-0.00076898
Rnf150	1	8.94E-14	1	0.000420643	0.026943651	-0.000794392
Arl4d	7.84582E-54	7.76E-11	1	0.045744199	-0.029991156	-0.000829297
Cyp4f39	1	2.31E-03	1	-0.000958182	0.006029888	-0.000898805
Ano8	0.021264577	1.00E+00	1	-0.004954164	-0.001968307	-0.000918745
Nat8	1	8.50E-05	1	-0.001162938	0.006138557	-0.000930982
Agxt2	1.45755E-68	5.18E-97	1	-0.11779819	-0.209738658	-0.000940936
Hand2os1	6.14532E-13	1.00E+00	1	0.008469505	-0.003436816	-0.000989242
Wdsub1	5.05242E-06	1.00E+00	1	-0.00926639	0.001436326	-0.000990824

Col14a1	0.03895761	1.00E+00	1	0.005788502	0.003632178	-0.00103203
Ngf	0.00040357	1.00E+00	1	0.00447149	0.004213801	-0.0010914
Gm4070	2.27141E-06	1.91E-18	1	-0.004759593	0.026952662	-0.001182852
Tgfbr3l	1	6.36E-15	1	0.00275315	-0.027896588	-0.001184452
Strada	7.87906E-05	1.00E+00	1	-0.015478021	-0.00181751	-0.001230199
Tafa4	0.000973765	1.00E+00	1	-0.005025351	-0.002001966	-0.00125702
Hcls1	1	1.40E-05	1	-0.001413139	0.016442668	-0.0013088
Hist1h2be	0.000169304	1.96E-04	1	-0.008995874	0.017887407	-0.001357058
6430548M	1	1.39E-07	1	-0.000610502	0.018678727	-0.001358348
Gucy1a1	1.35188E-06	1.00E+00	1	0.004891768	-1.24908E-05	-0.001360161
Fam131a	0.001160914	1.00E+00	1	-0.002715617	0.000709886	-0.001374672
Gm12823	2.04115E-12	1.00E+00	1	-0.009514963	0.000540368	-0.001374732
Asb2	2.217E-09	1.00E+00	1	-0.004998777	0.001741627	-0.001377465
Rec114	7.05412E-19	1.00E+00	1	-0.011635387	-0.005162502	-0.001393122
Gm32200	0.028027489	1.00E+00	1	-0.003833406	-0.001711917	-0.001424968
Slc25a18	1.08416E-07	1.00E+00	1	-0.008682096	0.002568	-0.001492154
Gm14027	0.000535207	1.00E+00	1	-0.004007193	0.003064064	-0.001511603
Gm13184	1.51619E-14	1.00E+00	1	-0.010102975	0.009323572	-0.001568613
Tbc1d8b	1	2.33E-07	1	-0.008291187	-0.036927095	-0.001582153
Plagl2	0.046952453	1.00E+00	1	-0.009490636	-0.004737113	-0.001604703
Adam24	8.36471E-06	1.00E+00	1	-0.002073545	-0.002202208	-0.001618259
Zranb3	3.88228E-11	1.00E+00	1	-0.026116059	0.007150338	-0.001644698
Cyp2c40	2.24096E-15	2.05E-02	1	-0.008212425	-0.008420153	-0.001655388
Vcpip1	0.001719435	4.98E-10	1	-0.022075339	-0.047448945	-0.001657995
Fam81a	6.23189E-14	1.00E+00	1	-0.004915048	-0.003627765	-0.001692684
Tpte	0.003013238	1.00E+00	1	-0.004562539	-0.000324205	-0.00173848
Dnhd1	1	2.73E-06	1	-0.003567745	-0.025009262	-0.001748217
Tecpr1	1	4.19E-02	1	0.002889413	0.047615822	-0.0017496
Igbp1	1	4.96E-02	1	-0.004992403	-0.019727374	-0.001751833
Gm40055	1	1.00E+00	0.048	-0.000765514	0.001507067	-0.001751926
Ccnl1	3.55581E-08	1.00E+00	1	0.038889966	0.018033575	-0.001761068
Lcmt2	0.00085154	1.00E+00	1	-0.010648781	-0.005492563	-0.00180539
Aven	1	1.37E-09	1	-0.003150067	-0.057610017	-0.001812438
Gm10800	1	2.18E-02	0.2103	0.000300526	0.004622988	-0.001833108
Riiad1	1	1.06E-03	1	-0.000621985	0.008217236	-0.001839991
Vps37c	1	3.23E-04	1	0.002486583	-0.026612397	-0.001917416
Mettl26	1.78057E-08	2.83E-05	1	0.024371028	-0.029142583	-0.001948287
Gm12405	0.110542445	1.00E+00	0.015	-0.001497259	-0.001969021	-0.001969021
Dnajb2	1	8.90E-03	1	0.002436514	-0.026647229	-0.001985964
Cyp2d34	1	1.00E+00	0.0112	-1.58495E-05	-0.001581299	-0.001988673
Gm9902	1	1.00E+00	0.0112	-0.001088036	-0.001988673	-0.001988673
6330576A1	0.016375674	1.00E+00	1	-0.002744998	-0.00066088	-0.001989098
Fzd8	0.046577987	1.00E+00	1	-0.005355808	-0.000855245	-0.002033441
Sugp2	1	2.52E-08	1	0.002892154	-0.040242447	-0.002060586
Lhfp	5.77531E-07	1.00E+00	1	0.007990144	-0.002558178	-0.002062871
Slc25a38	1	1.98E-24	1	0.003689231	-0.052569755	-0.002155055
Tcaim	3.3544E-12	1.00E+00	1	-0.032670853	-0.016086448	-0.002162336

Prr36	1	1.00E+00	0.0093	-0.000670274	-0.00204943	-0.002169696
Gm48488	1	1.00E+00	0.0035	-0.000207857	-0.002205768	-0.002205768
Ubap2l	5.11508E-06	1.00E+00	1	0.035388005	0.034752279	-0.00222498
Mup10	1	1.00E+00	0.0026	0.000440891	-0.00222542	-0.00222542
Pyroxd2	0.000799951	5.63E-08	1	0.017962809	-0.033326299	-0.002225858
Cacnb2	1.90796E-06	1.00E+00	1	0.008115151	0.003161668	-0.002267045
Lor	1	1.00E+00	0.0354	0.000936887	-0.00261226	-0.002278575
Cabcoco1	1	7.42E-15	1	-0.012763131	-0.050219438	-0.002286334
Zfp706	0.004440117	1.00E+00	1	-0.012048806	0.014294981	-0.002294091
Rec8	0.010378926	1.00E+00	1	-0.004764083	-0.003271795	-0.002315392
Gm14764	2.56839E-16	1.00E+00	1	0.010029068	-0.001235031	-0.002328403
Mup13	1	1.00E+00	0.0011	-0.000394644	-0.002367468	-0.002367468
Cstf3	1.66496E-09	1.33E-05	1	-0.042844016	-0.054818452	-0.002375572
Cntn2	1	1.00E+00	0.0093	-0.000823351	-0.002631912	-0.002381648
B4galnt1	4.66628E-25	7.93E-16	1	0.035653605	-0.038880244	-0.002386538
Oat	5.15718E-07	1.00E+00	1	-0.007295593	-0.005910797	-0.002390014
2810405F1	0.003532676	1.00E+00	1	-0.00632372	0.000266981	-0.002403563
Gm31683	6.06083E-07	1.00E+00	1	0.010121957	0.001229011	-0.002437779
Bace2	5.41212E-15	1.00E+00	1	-0.010718349	0.004050212	-0.002440996
1810044K1	1.63012E-18	1.00E+00	1	0.008512876	-0.00324569	-0.002475435
Ugt2b38	0.004243589	1.00E+00	0.0063	-0.002090651	-0.002476638	-0.002476638
Ankrd44	2.61298E-15	4.50E-05	1	-0.02537802	0.081095205	-0.002506861
H2-K1	8.71879E-09	1.00E+00	1	0.035660794	0.04966529	-0.002512214
Zfp780b	1	2.31E-18	1	0.010307057	-0.0612306	-0.002524865
Gm30692	1	1.00E+00	0.0358	-0.00096811	-0.002219247	-0.002533468
Gm36543	1	1.00E+00	0.0207	-0.001280018	-0.002223656	-0.002537877
Dscam	0.016621379	1.00E+00	1	-0.003575373	0.000209422	-0.002546943
AY512915	1	1.00E+00	0.0041	-0.00091449	-0.002315676	-0.002556208
Prok1	1.04199E-09	1.00E+00	0.0003	-0.002513978	-0.002556865	-0.002556865
5730420D1	2.79035E-05	1.00E+00	1	-0.003139175	-0.003825488	-0.002589328
1700037H0	6.02864E-05	1.00E+00	1	-0.004244866	-0.001889313	-0.002611051
Chrna7	1	1.00E+00	0.0397	-0.001171478	-0.002437191	-0.002619192
Dnajc24	1	3.44E-04	1	-0.009409489	-0.030746937	-0.002632772
Dlgap2	1.39287E-05	1.00E+00	1	-0.008403843	-0.004344093	-0.002633547
Pmel	1	1.00E+00	0.027	-0.000727653	-0.001077144	-0.002637261
Vnn1	0.555751905	1.52E-05	1	0.010812122	-0.023616514	-0.002642795
4930447A1	1	1.00E+00	0.0005	-0.000256938	-0.002522924	-0.002643189
Acad10	1.48123E-26	1.00E+00	1	-0.04041855	-0.007120717	-0.002645553
Kdm4d	0.005515617	1.00E+00	1	-0.005772158	-0.004335188	-0.002721129
Cyb5b	6.20341E-23	1.00E+00	1	0.063915877	-0.00980418	-0.002721971
Ctsk	0.001835511	1.00E-05	1	-0.006340763	0.02671669	-0.002729556
Slc35b4	1	4.22E-02	1	-0.001900286	-0.01275391	-0.002749797
Gm5105	1	1.00E+00	0.0269	-0.001075358	-0.002297821	-0.002769153
Ccser2	0.00611035	3.60E-08	1	-0.020288653	-0.040365185	-0.002785556
Nat14	1	1.00E+00	0.0122	5.13454E-05	-0.001921705	-0.00280041
Plekhf2	5.73358E-07	1.00E+00	1	-0.012866124	0.00575104	-0.002810863
Kmt5c	1	2.30E-06	1	0.001481949	-0.025759222	-0.002897213

Nr2f6	1	1.49E-02	1	0.007474568	-0.023900721	-0.002901281
Ednra	1	1.00E+00	0.0086	0.002342822	-0.003018824	-0.002913717
Prpf40b	0.05290706	2.82E-06	1	-0.016634064	-0.038825934	-0.00292508
Tent4b	1	5.98E-32	1	0.011609411	0.128836539	-0.002930804
Gm9946	1	1.00E+00	5E-05	-0.001485532	-0.002128957	-0.002943706
Fam120c	1	3.23E-34	1	-0.010593953	-0.086256956	-0.002964288
Igfbp5	1	1.00E+00	0.0055	-0.000857999	-0.002379675	-0.002970156
Dach1	3.89899E-05	1.00E+00	1	0.005970711	-0.001641494	-0.002992496
Inpp1	2.1101E-05	1.00E+00	1	-0.015467771	-0.003034945	-0.003024157
Frmd8os	1.41945E-23	1.00E+00	1	-0.02100043	-0.001828232	-0.00304138
Tctex1d4	0.000938078	1.00E+00	0.0236	-0.002820238	-0.000951432	-0.003064069
Colec10	1.09553E-28	1.00E+00	1	0.013866564	-0.003401111	-0.0030848
Bmyc	1	1.00E+00	0.0008	-0.000599797	-0.002412172	-0.003084968
Gm16277	1	1.00E+00	0.0012	-0.001615	-0.002858849	-0.003089649
Adamtsl5	1	1.00E+00	0.014	-0.001125245	-0.000910013	-0.00309078
Ihh	1	1.00E+00	0.0456	-0.000243381	-0.001325535	-0.003104498
Frmpd3	1	1.00E+00	0.0019	-0.001781161	-0.002337688	-0.003131856
Tnfsf10	1	1.00E+00	0.048	-0.000743767	-0.000620237	-0.003140237
Cyp2a12	2.32001E-49	2.05E-13	0.0042	0.084766717	-0.043886273	-0.003144537
Gm4737	0.006676252	1.00E+00	1	0.007086827	-0.002880644	-0.00317682
Tbc1d10a	0.033790497	7.24E-02	1	-0.011596171	-0.017346373	-0.003178707
Gcsh	0.000116497	2.24E-02	1	-0.012851786	-0.017667022	-0.003190062
Iyd	1	2.18E-02	1	-0.004856114	-0.019708349	-0.00322482
Gm5096	1	1.00E+00	0.0004	0.001697185	-0.003020549	-0.003251349
4930594M	1	1.00E+00	0.004	-0.001389618	-0.003337705	-0.003271664
Rbfox1	0.000639126	1.00E+00	1	0.00843446	-0.004701679	-0.00327459
4930592A0	0.028166732	1.00E+00	1	-0.003911422	-0.002065797	-0.003279291
Slc4a9	1	1.00E+00	0.0111	-0.000174026	-0.000919606	-0.003285845
4930477N0	1	1.00E+00	0.0066	-0.00138791	-0.003353797	-0.003287756
Zfp647	0.039553477	1.00E+00	1	-0.003143107	-0.003467862	-0.003293514
4933405D1	1.39067E-17	7.49E-02	1	-0.01503085	-0.013439105	-0.003301443
Ganc	1	1.17E-06	1	-0.006315743	-0.040071453	-0.003313636
Acaa1a	1	6.77E-12	1	0.017720618	-0.054272367	-0.003322578
Gm15348	0.00022459	1.00E+00	0.0005	-0.002846155	-0.00161351	-0.003378431
1700092C1	1	5.32E-01	3E-06	-0.001709603	-0.003389502	-0.003389502
Appbp2os	0.000246484	1.00E+00	1	-0.007629017	-0.004234333	-0.003394286
Nmt1	0.010092244	8.30E-13	1	0.023273222	-0.054357293	-0.003408358
Gm30127	1	1.00E+00	2E-05	-0.001436162	-0.001840414	-0.003423334
Gm26627	0.017216384	4.72E-01	2E-06	-0.002236002	-0.003436851	-0.003436851
Acsl3	4.68531E-08	5.75E-38	1	0.031611797	-0.075559091	-0.003439561
Arhgap33o	0.001737372	6.99E-02	0.0004	-0.002693379	-0.004194441	-0.003443649
Gm10801	1	1.00E+00	5E-05	0.001512513	0.005291591	-0.003445889
Gm48500	1.02561E-09	1.00E+00	0.1646	-0.004363873	-0.003878238	-0.003447094
Tfpi2	1	5.86E-63	1	-0.015053376	-0.159743761	-0.003458603
Zfyve27	1	1.92E-05	1	-0.003231923	-0.028341844	-0.003475676
Rnf186	1	1.00E+00	0.0041	0.002510567	-0.003235881	-0.003481839
Saa1	1	8.96E-03	0.8229	-0.002592481	0.010378901	-0.003488531

Olfr16	1	5.23E-03	0.0463	-0.002163299	-0.005458584	-0.003493318
Zfp595	0.162641312	1.37E-06	1	-0.011810486	-0.029438577	-0.003497292
Amelx	1	1.00E+00	0.0002	-0.000915342	-0.002545292	-0.003507419
Gm32281	1	4.18E-01	1E-06	-0.000570463	-0.003511898	-0.003511898
Gm10300	1	1.00E+00	0.0125	-0.001181397	-0.003737072	-0.003517126
Bglap3	1	1.00E+00	0.0009	0.001452617	-0.001608165	-0.003528463
Gm48775	0.207110826	1.61E-01	2E-05	-0.002190384	-0.003862995	-0.00352931
Fam186a	0.012024398	1.00E+00	1	-0.005277539	-0.001122084	-0.003555916
C920021L1	0.000756069	8.79E-06	1	-0.014165075	-0.026373527	-0.003575492
Tro	1	1.00E+00	0.037	0.000907629	-0.00189496	-0.003596063
Spata3	1	3.69E-01	0.0003	-0.002133064	-0.004023813	-0.003596975
Gm36899	0.946071852	1.00E+00	0.0098	-0.002459975	-0.003932713	-0.003606676
AC125149.	0.585506303	1.00E+00	0.0032	-0.002428742	-0.003601268	-0.003608916
Ptk2b	3.38421E-30	1.00E+00	1	-0.060890097	0.045483425	-0.003665366
Rnase13	1	1.00E+00	6E-05	-5.73767E-05	-0.003519625	-0.003665887
Cntn4	0.00146229	1.00E+00	1	-0.00755475	-0.002095984	-0.003676546
Pln	0.030244765	1.00E+00	1	0.007725461	0.002190757	-0.003686427
Gm7967	1	5.32E-01	0.0008	-0.001984625	-0.0041597	-0.003693795
Kctd15	2.23287E-11	1.00E+00	1	-0.02201719	0.002237475	-0.003711686
6430710C1	3.76321E-10	1.00E+00	1	-0.006719226	-0.002614266	-0.003745616
Kiz	9.09159E-09	1.00E+00	1	-0.030998577	-0.007514467	-0.003753594
Srms	1	3.40E-01	2E-06	-0.001278046	-0.003801356	-0.00375478
Olfr541	1	6.14E-01	3E-07	-0.001984823	-0.003564609	-0.003768296
Wnt5a	0.000355523	1.50E-02	0.7463	-0.004163481	-0.006444031	-0.003770245
Sult2a7	1	2.05E-01	0.0002	0.000155952	-0.004280095	-0.003779567
4930524O(	1	1.00E+00	8E-05	9.38115E-05	-0.003602149	-0.003786372
Sowaha	1	2.11E-01	7E-06	0.001114546	-0.004018451	-0.003805032
Tfb1m	0.006561216	1.00E+00	1	-0.012109288	-0.015699981	-0.003806122
Gm45469	1	1.35E-03	1	-0.002747471	0.014687673	-0.003826477
Gm19585	6.11374E-09	1.00E+00	1	-0.005717915	-0.00231549	-0.003832512
Sdr16c5	2.07735E-09	2.39E-02	3E-05	-0.003677061	-0.004620585	-0.003869794
Gdi1	1	4.44E-03	1	0.004214292	-0.019089837	-0.00388648
Ciart	2.19255E-11	1.00E+00	1	-0.006889851	-0.002303604	-0.003886672
Pla2r1	1	1.00E+00	0.0152	0.001128962	-0.001910894	-0.003905332
Nrtn	1	3.41E-01	7E-05	-0.000584533	-0.004226619	-0.003920046
Zbtb12	0.000280102	1.00E+00	1	-0.006677837	0.008060492	-0.003928279
Arap1	3.89324E-05	6.49E-05	1	-0.024900488	-0.03207868	-0.00393281
Stk3	4.35942E-08	1.00E+00	1	-0.035211853	0.037002773	-0.003952676
Gm9530	0.006985793	1.00E+00	1	-0.005463161	0.001340112	-0.003972169
Gm5820	0.076437421	1.00E+00	0.0135	-0.003037849	-0.003506602	-0.00397585
Sox13	0.001692854	2.29E-11	1	-0.016535016	-0.03980515	-0.003982833
A530001N2	4.73054E-09	1.00E+00	0.0009	-0.004145142	-0.001410424	-0.004018312
Cmya5	6.06363E-10	1.00E+00	1	-0.01047354	-0.005942519	-0.004022047
Acbd6	2.53774E-10	3.68E-07	1	-0.035018157	-0.045015754	-0.004027346
Sppl2b	1	6.30E-08	1	0.006551661	-0.026803747	-0.004030027
Espnl	1	5.30E-02	0.0068	-0.000832741	-0.005354075	-0.004036716
Impg2	1	3.13E-10	1	0.001103474	-0.027539821	-0.004054959

Plet1	1	1.00E+00	2E-05	0.001175753	-0.003938957	-0.004062428
Itga8	1	1.00E+00	0.0145	0.000489349	-0.002833169	-0.004063767
Zfp354c	1	1.00E+00	1E-05	-0.000620891	-0.003588992	-0.004094934
Snai2	1.73903E-09	1.64E-06	1	0.011342569	-0.013174232	-0.004100927
Fam126b	0.335462054	6.63E-21	1	0.024701692	-0.082553999	-0.004137646
Fyttd1	1	2.98E-08	1	-0.009550879	-0.045304334	-0.004140137
1810046K0	0.000946347	4.47E-03	0.0075	-0.003703494	-0.005906432	-0.004155557
Olfr45	1	1.00E+00	1E-07	-0.00032686	-0.003725427	-0.004169646
Epm2aip1	1	1.47E-11	1	0.009892745	-0.035555657	-0.004183904
Tmem243	1	1.98E-08	1	0.015906336	0.091131629	-0.004191231
Kbtbd3	0.000108671	1.00E+00	1	-0.013029685	-0.004253921	-0.004192516
Atp4a	1	2.69E-02	7E-07	-0.001687196	-0.004628631	-0.004211524
C230013L1	1	1.00E+00	0.0026	-0.00023922	-0.004267757	-0.004243988
Slc6a21	1	6.99E-02	2E-08	-0.000554226	-0.004249836	-0.004249836
Lepr	8.26501E-25	1.00E+00	1	0.016165668	-0.000710706	-0.004264933
Gpc6	2.02619E-38	1.00E+00	1	0.029050425	-0.007135063	-0.004274604
Tmem98	1	1.00E+00	0.0007	-0.002422283	-0.004617058	-0.004279067
Slc22a22	6.48491E-07	9.23E-01	1	-0.009755468	0.014226762	-0.004280788
9530018F0	1	4.21E-01	5E-05	0.000105179	-0.004299232	-0.004304647
Gm20663	0.211277481	1.54E-02	1	-0.005227374	0.021340141	-0.0043075
4930546C1	1	1.00E+00	0.0003	-0.002294666	-0.004320117	-0.004316917
Robo2	2.80876E-06	1.00E+00	1	0.007980585	-0.001104142	-0.004351018
A1cf	1.1393E-169	9.92E-99	1	-0.166739779	-0.239331082	-0.004363669
Sptb	1	1.00E+00	0.0276	-0.002705091	-0.002987174	-0.004380443
Myrf	1	1.28E-03	1	0.00080487	-0.020912679	-0.004402782
Mycn	1	4.16E-01	8E-09	0.001427689	-0.003893058	-0.004419582
Gm40787	0.009739932	1.00E+00	1	-0.005571459	-0.001998505	-0.004424826
Col7a1	1	1.00E+00	1E-05	0.003125413	-0.00415517	-0.004435752
Gm45941	0.926461591	1.00E+00	0.0003	-0.002694534	-0.002522677	-0.004436081
Sall2	1	2.23E-01	0.0153	-0.000558986	-0.005644135	-0.004439398
Hcn3	0.156014257	2.52E-01	0.0048	-0.003258575	-0.005424675	-0.004458381
Slc12a3	1	1.00E+00	0.0115	0.001927993	-0.001673701	-0.004483059
Pcnx4	4.12143E-09	1.00E+00	1	-0.021009443	-0.007914001	-0.004501658
Aatk	1.16597E-05	1.00E+00	0.004	-0.004830378	0.00468525	-0.004535468
Serpina9	0.530823236	2.73E-02	5E-06	-0.002665464	-0.005156638	-0.004562957
Phldb1	0.014714005	1.00E+00	0.0053	-0.003958772	-0.002514595	-0.004586933
Gm28100	0.106332336	9.23E-01	2E-07	-0.002636697	-0.004206085	-0.004591773
Sppl2a	9.35314E-09	1.00E+00	1	-0.039638763	-0.007418199	-0.004605987
Myom3	5.59462E-06	1.00E+00	1	-0.006613773	-0.006418204	-0.004610064
Pam16	0.001176063	1.00E+00	1	0.015304931	-0.011110265	-0.00461407
Tgm1	1.83009E-14	1.00E+00	0.6776	-0.007470743	-0.00166696	-0.004627172
Slc13a4	1	1.00E+00	6E-06	-0.001560854	-0.003343669	-0.004655744
Dcn	2.03253E-05	1.00E+00	0.4454	0.008535008	0.000209545	-0.004666471
Sebox	1	1.19E-02	5E-06	-0.002474214	-0.005480037	-0.004670716
Tpm2	1	1.00E+00	3E-07	-0.00114729	-0.003523117	-0.004674733
4930459C0	1	1.00E+00	0.0073	-0.001470548	-0.004037241	-0.004693299
Gm15723	1	3.86E-02	4E-09	0.000457729	-0.004700118	-0.004700118

Pkdcc	0.005666909	1.00E+00	1	-0.010836082	-0.009792278	-0.004735299
Gm10135	0.033790497	1.00E+00	1	0.009209241	0.002380959	-0.004743109
Golph3l	0.008966356	1.00E+00	1	0.014078541	-0.012184926	-0.004750112
Gm48395	1	1.00E+00	1E-07	-0.000511146	-0.003071747	-0.004762968
1810062O1	1	4.36E-02	1	-0.005108625	-0.012987426	-0.004784936
Gimd1	1	1.00E+00	1E-06	0.000858907	-0.00384038	-0.004791658
Cdh9	1	1.00E+00	4E-07	-0.000772325	-0.004433049	-0.004793846
Gm40604	1	2.16E-01	6E-08	0.000856081	-0.004363555	-0.004795808
Ly75	2.91431E-13	1.00E+00	1	-0.01187225	-0.001728466	-0.004827767
Hba-a2	0.000170326	4.30E-02	1	-0.006952156	0.012238021	-0.004831346
Car15	1	1.00E+00	0.0037	-0.001075032	-0.002005715	-0.004860533
Zfp493	1	1.00E+00	0.01	0.000489113	-0.003817228	-0.00487043
Rgs16	1.1871E-197	1.00E+00	0.1784	0.063095296	-0.005855341	-0.004887802
Gm29682	0.000247524	1.00E+00	0.0057	-0.004477077	-0.003661772	-0.00489377
Kctd7	1	2.99E-04	5E-05	-0.001397987	-0.006705758	-0.004907335
Gm2762	0.00257514	4.22E-02	3E-06	-0.003445435	-0.005429122	-0.004945975
Gm43824	4.69413E-07	1.00E+00	0.0011	-0.004874719	-0.003731776	-0.004996308
Gm28040	1	1.65E-03	1	-0.005456129	-0.019492229	-0.004998977
Crbn	1	1.80E-03	1	0.000211902	-0.021419775	-0.005000867
Mroh8	1	1.00E+00	0.0007	-0.000711283	-0.004941622	-0.005016421
Oxnad1	1.32787E-06	2.61E-01	1	-0.017397673	-0.017785452	-0.005043843
Gm49076	1	4.39E-02	2E-05	-0.000861243	-0.005704291	-0.005044569
Gm35986	1	2.97E-01	1E-06	-0.001219486	-0.004954747	-0.005045817
Zfp658	1	4.13E-03	0.0255	-0.000358593	-0.007843091	-0.005093532
Slc30a8	1	3.70E-02	6E-08	-0.001096044	-0.005173293	-0.005103914
Gm11837	1	1.00E+00	4E-07	-0.000343984	-0.004263824	-0.005186416
Pdcd2l	1	1.80E-02	1	4.04191E-05	-0.015715898	-0.005189528
Syt16	1	1.00E+00	0.0023	-0.001867184	0.000234178	-0.005203781
Bnip2	1	4.04E-06	1	0.011286865	-0.033680562	-0.005204265
Isg15	0.010965769	1.00E+00	0.0002	-0.003883234	0.000478605	-0.00522976
B9d2	1	1.00E+00	3E-07	0.00044045	-0.003783114	-0.005236997
Prickle4	1	1.40E-02	0.0971	-0.003579565	-0.008004829	-0.005238028
Gm17662	1	1.00E+00	9E-09	-0.000592242	-0.002817145	-0.005239693
Gm16174	1	7.07E-03	0.0024	-0.000617987	-0.00721471	-0.005259181
Gm32468	0.246882105	3.04E-02	2E-09	-0.002923404	-0.00526119	-0.00526119
Lhx6	1	1.64E-01	5E-06	-0.001043895	-0.005575332	-0.005271832
Sh3bp4	9.42107E-09	1.00E+00	1	-0.007917704	-0.005003864	-0.005277053
Pappa2	1	1.00E+00	0.036	0.00042696	0.00077517	-0.005297774
Tcf24	0.74249397	7.23E-01	1E-08	-0.002704647	-0.004512198	-0.005306366
Ampd2	1	1.66E-09	1	0.001104055	-0.030154709	-0.005328962
Oas1c	1	1.00E+00	1E-06	-0.001996559	-0.002702828	-0.005357159
S1pr5	1	3.91E-02	9E-11	-0.001937208	-0.005053893	-0.005377846
Vezt	2.91209E-24	6.22E-02	1	-0.044122361	-0.022897768	-0.005390117
Pdcd6	1	2.94E-04	1	-0.009093972	-0.023437736	-0.005393853
Klhl11	0.018651819	1.00E+00	1	-0.009633526	-0.00676576	-0.0054077
Cfap299	1	1.00E+00	0.0053	-0.00169287	-0.0024768	-0.005416516
Milr1	0.047549186	1.00E+00	0.0026	-0.004343711	0.003927245	-0.005416875

Mzb1	1	5.98E-02	2E-09	-0.001327787	-0.005195059	-0.005425859
Gm16587	1	1.00E+00	1E-05	-0.001083438	-0.004597439	-0.005437216
Dnpep	1	4.07E-06	1	-0.000822246	-0.016553406	-0.005444355
Gm49602	1	1.00E+00	1E-05	-0.001624859	-0.002487216	-0.005456873
Piezo2	1	1.00E+00	1E-05	0.003347426	-0.002691099	-0.005462011
Slc2a5	1	8.29E-03	2E-07	-0.002213918	-0.006202804	-0.00548984
Nmnat2	1	1.00E+00	5E-06	-0.001728602	-0.004175105	-0.005491491
Adamts13	1	1.56E-01	2E-07	0.000864656	-0.004928432	-0.005496098
Wdr63	1	1.00E+00	0.0004	-0.002292043	-0.003537049	-0.005511222
Trmt2b	1.49467E-06	5.51E-10	1	-0.02357123	-0.042495995	-0.005519708
Fam161a	1	1.00E+00	0.0174	-0.002585554	-0.004770849	-0.005535975
P2ry4	1	7.49E-03	6E-12	-0.000987274	-0.005344233	-0.00554792
Psmb9	1	1.00E+00	0.0465	-0.000954423	-0.000897096	-0.005563104
Gm31458	1	6.87E-02	7E-06	0.002680315	-0.005880319	-0.005569441
Sox12	1	1.78E-02	1E-06	-0.001255156	-0.006047029	-0.005589878
Mgat5	1.24495E-47	3.88E-10	1	-0.068029774	0.150596729	-0.005606049
Apcs	1	9.27E-03	2E-06	0.001182693	-0.006322203	-0.005612567
Abi3bp	1	1.00E+00	0.0003	0.003295389	-0.00557061	-0.005622485
Fetub	7.4611E-115	1.00E+00	1	-0.09295243	0.014630642	-0.005627895
Gm15848	1	1.00E+00	2E-07	-0.001802085	-0.003631363	-0.00562819
Gm48952	1	1.00E+00	0.0084	0.002096898	-0.003460522	-0.005671894
Ttbk1	1	1.00E+00	6E-06	-0.00314918	-0.003477748	-0.00569342
Gm44957	1	1.65E-02	5E-08	-0.001393477	-0.005863876	-0.005703565
Gm26935	0.0071707	1.00E+00	1	-0.006845558	0.000191633	-0.005719065
Gm5600	1	1.00E+00	0.007	-0.000764749	-0.000433226	-0.00572715
Gpr34	1	1.00E+00	0.0385	-0.001583975	-0.000376881	-0.005748792
Cyp2d10	8.89073E-41	1.11E-61	1	0.071502635	-0.132682018	-0.005750497
A330041J2	1	1.00E+00	0.0221	-0.002512262	-0.003502861	-0.005756637
P3h2	1	1.00E+00	0.0121	0.000605029	-0.005202268	-0.005772757
Gm8016	1	1.00E+00	5E-06	-0.002346801	-0.005126891	-0.0058236
Gm6034	1	1.00E+00	0.0024	-0.002983556	-0.001115885	-0.005849473
Gm7030	1	1.00E+00	0.002	-0.003551508	0.000200272	-0.00585403
Hpcal1	0.000160291	1.00E+00	1	-0.019617976	0.060966476	-0.005859655
Abhd3	1.04262E-14	2.91E-04	1	-0.032914411	-0.030543148	-0.005861696
Dcstamp	1	9.56E-02	8E-12	-0.001378624	-0.004830236	-0.005872446
Zfp454	1	2.60E-02	4E-09	-0.00146079	-0.005821325	-0.00587555
Emsy	1	5.43E-06	1	0.010190291	-0.038624753	-0.005894211
Col25a1	1	1.00E+00	0.0044	-0.002868847	-0.00569371	-0.005904083
Naaa	1	9.00E-01	0.0122	-0.004227207	-0.007262202	-0.005943492
Hspa1b	1	1.63E-01	1E-08	-0.001796529	-0.005649816	-0.005955288
Hmg20a	1	1.85E-11	1	0.006123688	-0.059878706	-0.005956991
Cyp46a1	1	1.00E+00	0.0494	0.000583206	-0.004284303	-0.005957457
2810408I1	1	1.00E+00	7E-07	-0.002552714	-0.005039005	-0.00595997
Pde3a	1.41412E-13	1.00E+00	0.0063	0.011003967	0.001630706	-0.005993773
Crip3	1	7.76E-04	3E-11	-0.002471441	-0.006306463	-0.006009623
Gzf1	0.703089601	3.47E-11	1	0.015790654	-0.040729899	-0.006010066
Dennd5b	3.0351E-280	5.02E-82	1	0.191306391	-0.189361419	-0.006032331

Rundc3a	1	2.53E-03	7E-07	-0.000882266	-0.007026513	-0.006033106
Leap2	7.96873E-30	6.11E-12	1	0.030341843	-0.02785559	-0.006051345
Gm45510	1	1.48E-03	1	-0.000814443	0.01453889	-0.006056901
1110013H1	1	9.50E-01	0.0005	-0.002288289	-0.006146843	-0.006101514
Bmp4	1	3.94E-03	3E-11	-0.001566879	-0.00596214	-0.006108402
9330175E1	1	1.00E+00	4E-05	-0.00152168	-0.002119992	-0.006139496
D130009I1	0.012558851	1.00E+00	0.3	-0.006042931	-0.006498572	-0.006152096
Spatc1l	1	1.00E+00	6E-07	-0.000963315	-0.00463018	-0.006153591
Mtnr1a	7.26808E-05	8.50E-03	1	-0.009907583	-0.014380759	-0.00615605
Tmem144	1	1.16E-11	1	-0.003500039	-0.028336528	-0.006180146
Gm14703	1	3.79E-01	0.0021	4.24025E-05	-0.007074848	-0.00619754
Trir	0.000467568	1.41E-06	1	0.020799312	-0.034440373	-0.006234202
Ifngr2	0.000532531	1.00E+00	1	-0.009350078	0.014505291	-0.006238022
Trim12a	1.28909E-07	7.52E-04	1	-0.016483613	0.035499238	-0.006239321
Vegfd	9.54165E-15	1.00E+00	1	-0.031053302	-0.016863376	-0.00625037
Pld1	9.1168E-07	1.00E+00	1	-0.022215479	0.018099915	-0.006250442
Fam227a	5.55838E-11	1.00E+00	1	-0.014492431	-0.006575555	-0.006252924
Gm10856	1	1.59E-03	0.0001	-3.63977E-05	-0.008058634	-0.006263542
Gm50046	0.033288711	2.18E-01	0.0006	-0.004948898	-0.007308854	-0.006264166
Ggt6	1	3.54E-01	0.0026	-0.000631288	-0.007163553	-0.006284996
Saa3	1	1.00E+00	1E-09	-0.001249754	-0.002289463	-0.006341352
Dclk2	0.00966227	4.55E-03	0.0019	-0.00532251	-0.008091584	-0.006348463
Gm36855	6.28583E-10	8.91E-03	6E-14	-0.004393773	-0.005741798	-0.006352859
Zfp592	1	2.34E-08	1	0.000851799	-0.050863993	-0.006360788
Firre	1	6.21E-33	1	0.010135901	-0.096250506	-0.006391958
Vipr1	1	3.77E-01	1E-08	0.001017515	-0.005298337	-0.006394628
Rnaset2b	3.65759E-09	1.00E+00	1	-0.016127599	-0.013756462	-0.006396141
1700047A1	1	1.00E+00	0.028	-5.52761E-05	0.005486541	-0.006400157
Gm50399	0.181149844	1.88E-02	0.5135	-0.005660492	-0.010777163	-0.006410359
Spink5	2.24523E-08	1.00E+00	4E-05	-0.006085032	-0.004615962	-0.006411206
Gm12592	1	1.00E+00	2E-11	-0.002827776	-0.004397194	-0.006439858
D030055H(	1	1.00E+00	2E-05	-0.001213517	-0.004427759	-0.006440999
Gm49067	1	1.00E+00	0.0009	-0.000692757	-0.003618343	-0.006441881
Gm11767	0.211774922	1.00E+00	0.0189	-0.004952261	-0.006625234	-0.00644784
Plag1	1	1.00E+00	0.0092	-0.004267532	-0.007198704	-0.006456542
Nfasc	1	1.00E+00	4E-06	-0.002028499	-0.005687952	-0.006487685
Brd9	1	5.94E-03	1	0.00926292	-0.020184711	-0.006516323
Adnp2	0.000616508	1.00E+00	1	-0.012247803	0.00613674	-0.00653043
Gm28229	1	1.00E+00	0.0014	-0.003700698	-0.000488654	-0.006543239
Cdk20	0.001170397	1.00E+00	1	0.010045968	-0.001937397	-0.006560746
Txndc12	1	9.67E-10	1	0.001328987	-0.035341528	-0.006591272
Zfpm2	6.02318E-33	1.00E+00	0.0035	0.02062883	0.004015312	-0.006607248
B430119L0	1	4.87E-05	8E-07	-0.003460923	-0.008700708	-0.006610871
Htr4	1	1.00E+00	8E-07	-4.67043E-05	-0.003451523	-0.006620695
Gbf1	8.32267E-06	4.13E-08	1	0.035330249	-0.050852228	-0.006654553
Unc93a2	1	6.36E-01	0.006	-0.00292758	-0.007635948	-0.006663403
Dynlt3	0.002126406	1.00E+00	1	-0.008972197	-0.004949409	-0.006677921

Tomm34	1	4.39E-02	1	-0.002501002	-0.019693729	-0.006719123
Mup16	1	3.16E-02	6E-13	-0.00272662	-0.005810282	-0.006722494
Gm16188	1	1.00E+00	4E-07	-0.002441969	-0.000369758	-0.00672535
Xk	3.27448E-16	1.66E-02	1	-0.018795343	-0.016049505	-0.006729621
Sema3d	1	1.00E+00	6E-06	0.001061515	-0.00427224	-0.006760972
Derl2	0.007455206	6.34E-02	1	0.021587274	-0.023462676	-0.006769285
Atp1a3	0.05019901	1.00E+00	0.0007	-0.004868357	0.001551365	-0.006770847
Pth1r	1	1.14E-03	1E-08	-0.001642351	-0.007467587	-0.006794795
Gm45502	1	1.00E+00	7E-07	0.004179296	-0.004807441	-0.0068033
Pcgf5	3.68793E-16	1.00E+00	1	-0.057197571	0.034148567	-0.006814484
Dhrs7	1	1.00E+00	0.0003	-0.003391129	-0.002690409	-0.006865802
Gm49741	1	1.00E+00	0.0036	0.002088171	-0.005331312	-0.006877673
Cfap100	1	3.53E-01	3E-10	-0.002571966	-0.005782055	-0.006881828
Cxcl17	1	1.00E+00	2E-06	0.000129932	-0.001884267	-0.006888711
Ptk6	1	1.64E-04	7E-15	-0.001987803	-0.006859412	-0.006896257
Ifit1	0.187900085	1.00E+00	4E-07	-0.004526824	0.000443398	-0.006910229
2700054A1	6.00369E-05	1.00E+00	1	-0.009354378	0.0019205	-0.006927983
Gm15635	1	2.22E-01	6E-12	-0.001595645	-0.005652494	-0.006928842
Gm16170	1	7.70E-01	1E-08	-0.003213521	-0.005858898	-0.006939207
Zfp804b	6.21963E-13	9.67E-01	0.0552	0.014215874	-0.007045138	-0.00694241
Fam209	1	6.56E-01	0.006	0.002878234	-0.007876857	-0.006945466
Gm39326	1	1.00E+00	0.0369	0.001208971	0.000140896	-0.006967851
Gm12866	0.028826997	9.78E-04	5E-10	-0.003839154	-0.007441927	-0.006985893
Gamt	2.06248E-18	1.01E-01	1	-0.017728436	-0.014260683	-0.007001526
Stard8	1	1.00E+00	0.0128	0.000254242	0.008947574	-0.007007053
Cers5	1	1.00E+00	0.0116	-0.001083453	0.004568837	-0.007008238
Rgl3	2.66485E-05	7.32E-06	1	-0.022137369	-0.034515049	-0.007022507
Ormdl1	0.00032284	1.00E+00	1	-0.008375937	-0.008412726	-0.007034069
A530088E0	0.000188964	1.00E+00	4E-12	-0.004172405	-0.004109799	-0.007034952
Ccl9	1	1.00E+00	7E-08	0.002462656	-0.002271993	-0.007038927
Nr1h5	0.000472389	4.69E-03	2E-10	0.005935477	-0.00684453	-0.007054615
Gm12898	1	5.01E-01	2E-06	-6.7942E-05	-0.006691568	-0.007065578
Zfp128	1	1.30E-03	0.0045	0.000626943	-0.010584346	-0.007101248
Tmem38b	5.1287E-15	1.00E+00	1	0.034487245	-0.016992184	-0.007149568
Pja1	1	7.96E-05	1	-0.004905042	-0.021761553	-0.007153462
Meg3	0.008732055	1.56E-01	2E-08	0.007205175	-0.006033702	-0.007155612
Maml3	1.28621E-38	5.88E-56	1	-0.086121298	0.193835661	-0.00715568
Plet1os	1	4.14E-09	0.0023	0.001037475	-0.013926432	-0.00715599
Ccn6	4.22408E-06	1.00E+00	9E-09	-0.00534649	-0.004444173	-0.007225435
Gm12474	1	1.00E+00	0.0084	-0.003599863	-0.006860261	-0.00723762
Ctf1	1	1.49E-02	7E-09	-0.0004944	-0.007274898	-0.007243619
Cnmd	1	8.44E-02	8E-12	-0.000507549	-0.006075625	-0.007261418
Ube2s	2.43254E-07	1.00E+00	1	-0.011199901	-0.003651538	-0.007261928
Gm9917	1	3.49E-03	0.0026	-0.000920398	-0.010241289	-0.007265384
Mup19	1	8.36E-04	2E-14	0.003450852	-0.007001504	-0.007278881
1500015L2	1	1.00E+00	1E-07	-0.002556389	-0.001660928	-0.007286166
E330011O2	0.017967085	2.04E-01	1	-0.009648345	-0.013339387	-0.007287071

Rhd	1	1.00E+00	0.0005	-0.003278414	0.00229042	-0.007300379
Kank2	0.000149437	8.22E-03	1	-0.018257597	-0.023834404	-0.00731074
Usp10	1	3.31E-05	1	0.019061767	-0.044137523	-0.00732362
Olfm3	1	1.00E+00	1E-05	-0.003412652	-0.006916445	-0.007323962
Tle6	1	8.37E-02	8E-12	0.000663535	-0.006317187	-0.007331317
Zfp619	6.89361E-05	1.00E+00	1	-0.009755913	-0.009102073	-0.007331493
Six5	1	1.00E+00	3E-08	-0.000815046	-0.005228787	-0.007343795
BC048644	1	2.80E-02	9E-07	-0.003071891	-0.00795123	-0.007354366
Ctsb	1	3.61E-57	1	-0.00746882	0.1644184	-0.007374381
1700029I1!	1	1.02E-10	0.0933	-0.000782263	-0.017000931	-0.007397898
Pm20d2	1	1.00E+00	3E-06	0.000853682	-0.004637762	-0.007410424
4930534D2	1	7.17E-03	3E-13	-0.001784659	-0.006792444	-0.007457731
Tcaf1	1	2.80E-05	5E-06	-0.003906946	-0.009677461	-0.007469236
Fam53a	1	6.85E-08	1	0.000355169	-0.034873788	-0.007497389
Jund	0.002833465	5.29E-01	1	-0.010949693	0.017754111	-0.007521033
Cyp27a1	2.7336E-137	2.07E-66	1	0.194534524	-0.206374087	-0.007521299
Gm48094	1	1.00E+00	0.0005	-0.003156295	-0.00623957	-0.007525664
Sar1a	1	8.27E-06	1	0.005240696	-0.023386672	-0.007534481
Elf5	1.02714E-35	1.00E+00	0.0252	-0.015398083	-0.005674751	-0.007549518
Nbea	1	3.41E-12	1	0.007770963	-0.047774346	-0.007560583
Cfap74	2.02065E-19	1.00E+00	2E-07	-0.008955135	-0.005600638	-0.007563927
1700019A0	0.004065427	3.75E-01	1	-0.008455636	-0.011875901	-0.007597964
Zscan2	1	6.44E-03	0.1738	0.001095707	-0.011663591	-0.007601768
Brd1	0.012805394	2.69E-11	1	0.026987375	-0.053070883	-0.007605018
Rsad2	0.001139328	4.63E-03	1	-0.008510552	0.022097574	-0.007605563
Phc1	1	5.90E-03	3E-14	0.000994103	-0.006379585	-0.007621675
Gm47123	1	1.00E+00	0.0001	-0.003220541	-0.006289592	-0.007627689
Ralyl	1.2855E-06	1.00E+00	2E-08	-0.005802762	-0.005523607	-0.007628883
Ppp1r3g	1	1.00E+00	5E-12	-0.001807209	-0.005434243	-0.007657425
Gm15892	1.1855E-05	1.51E-07	0.0003	-0.006866551	-0.012777766	-0.007659734
Ak6	1	3.46E-02	1	-0.001340477	-0.018287885	-0.007661351
Fsd1l	0.201154638	1.00E+00	5E-06	-0.004474117	-0.004102921	-0.007673878
Irf7	1	1.00E+00	0.0017	-0.001284504	-0.005087026	-0.007682518
S1pr2	4.79541E-08	1.00E+00	5E-05	-0.007511173	0.005126119	-0.007685117
Gm20616	1	1.00E+00	0.0002	0.001299946	-0.002912443	-0.007701534
Asb5	1	3.80E-06	0.1233	-0.002372376	-0.015951033	-0.007711425
Ang	1	4.41E-05	0.5604	-0.00545989	-0.015034054	-0.00772038
Etfb	1	3.41E-14	1	-0.002610711	-0.050088096	-0.007721336
Rcan2	1	4.61E-06	0.533	0.002395031	-0.015674768	-0.00774838
Zbp1	1	1.00E+00	3E-10	-0.002957983	0.002496965	-0.007755082
Cyp21a1	1	2.24E-04	1E-13	-0.000704809	-0.007845741	-0.007767747
Ccnb2	5.98702E-07	1.00E+00	1	-0.012816314	-0.002344945	-0.00778278
Plscr3	1	1.00E+00	0.0335	0.001673399	-0.009309364	-0.00781116
Fitm1	1	6.41E-04	1E-14	-0.002980484	-0.007426794	-0.007814705
Fam25c	1.18395E-15	3.17E-04	0.0006	-0.010044785	-0.010566661	-0.007897683
Setmar	1	1.24E-06	0.2514	-0.000358956	-0.016625549	-0.007919728
Abraxas2	1	1.64E-02	1	0.00635933	-0.020935725	-0.007924147

Awat2	0.000175283	1.00E+00	1	-0.012003728	-0.00510695	-0.007941915
Ubap1l	1	8.37E-03	0.1789	-0.006032858	-0.012701549	-0.007955598
1700045H1	0.008181063	4.49E-02	1	-0.010411998	-0.014983686	-0.007965969
Pde6g	1	1.00E+00	0.0007	-0.003533595	-0.007075022	-0.007966916
Atg101	4.61773E-05	1.00E+00	1	-0.013418105	-0.012372938	-0.0079685
Klhdc7b	0.03241202	3.94E-04	8E-11	-0.00425084	-0.008454152	-0.007978653
Fbrsl1	1	3.80E-61	1	0.013930624	-0.139544554	-0.007988576
Gdf9	1	4.57E-03	2E-05	-0.004341868	-0.00940026	-0.00801477
Gm45235	1	1.00E+00	2E-06	0.000351192	0.004444575	-0.008063895
Kdm8	1	2.58E-02	1	0.001613429	-0.015653239	-0.008086721
4921539H0	1	1.00E+00	2E-10	-0.000447041	-0.003693	-0.008165483
Bmp5	1.29151E-14	1.29E-02	2E-10	0.011429776	-0.005732118	-0.008206046
Cblb	1.45055E-20	4.17E-45	1	0.058057789	0.18155442	-0.008247731
Gm47725	1	2.38E-06	4E-18	-0.002002776	-0.008432671	-0.008265828
Epn2	1.61106E-05	6.14E-05	1	-0.019852144	-0.02865394	-0.008270291
Shpk	0.04252647	4.62E-11	1	-0.017058818	-0.047603866	-0.008270712
Fbxo8	0.005150558	1.00E+00	1	-0.026169904	-0.013714652	-0.008282565
Gm11867	1.56335E-08	1.00E+00	1	-0.011244265	-0.001839752	-0.008300441
Zbtb14	1	1.00E+00	0.0254	-0.003501789	-0.001883551	-0.008302135
Snx9	3.98086E-09	3.67E-01	1	-0.036213874	-0.023565073	-0.008331983
Hmgn5	1	1.88E-08	0.314	-0.003012036	-0.020281237	-0.008337585
Wnt9a	0.006934812	3.21E-05	2E-16	-0.004082805	-0.008300465	-0.008364422
Irf2bp1	0.001526485	1.00E+00	0.0413	-0.007848468	-0.000826625	-0.008403623
Car5b	0.003971723	1.11E-01	2E-09	-0.005167015	-0.00762932	-0.008405145
Map1lc3a	1	1.00E+00	0.0029	-0.000561	-0.007947391	-0.008410241
0610040F0	0.021566952	1.00E+00	0.0002	-0.005993577	-0.006077521	-0.008428743
Cldn3	1	1.00E+00	0.0006	-0.004740657	0.002598016	-0.008436817
Mib1	0.114964198	1.23E-06	1	-0.022317689	-0.047575588	-0.008453154
Prkar1a	1	9.54E-03	1	-0.011818542	-0.022957073	-0.008466943
Gm26964	1	3.51E-02	5E-11	-0.003181805	-0.007592969	-0.008486971
Prdx4	1	7.70E-09	1	-0.004385347	-0.022879313	-0.008518282
Dcaf6	5.3385E-130	5.02E-23	1	0.159722321	-0.092088113	-0.008535163
Gm14798	1	1.00E+00	0.0226	0.002198496	-0.0025396	-0.008539828
Ccdc151	0.672556501	2.54E-07	3E-16	-0.003421692	-0.009443975	-0.008563186
Themis	0.013240662	1.00E+00	0.0035	-0.00763456	-0.003885565	-0.008563301
Popdc3	1	1.57E-01	1E-11	0.000727515	-0.007273184	-0.008603757
Dennd3	1	1.75E-03	0.6888	-0.004949395	-0.012530495	-0.008609108
Atxn7l3b	1	3.56E-04	1	-0.000746545	-0.027209961	-0.008626748
Meiob	2.52873E-34	5.93E-08	1	-0.049962503	0.065520032	-0.008661474
Zfp655	5.90726E-44	2.18E-04	1	-0.053042415	-0.032035679	-0.008704666
Gm17249	1	1.00E+00	0.0005	-0.002859962	-0.0084895	-0.008716418
Gm45282	1	1.03E-06	4E-20	-0.003093073	-0.008736418	-0.008736418
Map4k5	1	2.39E-06	1	-0.011222483	-0.041678368	-0.008760253
Cdyl2	4.01432E-05	1.00E+00	1	-0.019263426	-0.002222875	-0.008777288
Nap1l4	1	3.86E-08	1	0.004029629	-0.039447116	-0.008788919
1700011L2	1	4.32E-01	3E-08	-0.000549538	-0.007982642	-0.008789901
Gm15527	8.02817E-05	2.83E-02	4E-06	-0.00661103	-0.009185877	-0.008801579

Csnk2a1	0.440335166	1.25E-07	1	0.022628515	-0.052366527	-0.008829714
Slc6a9	2.8758E-08	1.00E+00	0.2972	-0.020777293	-0.004982692	-0.008836948
Gm31036	1	1.00E+00	4E-07	-0.004199388	-0.004237516	-0.008840151
Pex6	1	1.02E-02	1	0.014500538	-0.028449676	-0.008846784
Srebf2	1.79528E-32	2.83E-55	1	0.068183508	-0.112108651	-0.008857749
Calu	1	2.33E-11	1	-0.008678261	-0.039138066	-0.008884523
Dcp1a	1	1.28E-03	1	0.002116489	-0.031147087	-0.008918914
Lrrc4c	0.003762082	2.55E-02	1E-04	0.00825272	-0.010613343	-0.008927839
Gm28960	1	1.00E+00	4E-14	-0.00202477	-0.005785686	-0.008949443
Rgr	1	7.12E-01	2E-09	-0.002990169	-0.007258911	-0.008953624
Gm10701	1	1.00E+00	5E-13	-0.002693975	-0.004647783	-0.008963166
Gm10681	0.000334412	1.16E-06	2E-19	-0.004366278	-0.009049138	-0.008965717
A930038B1	1	8.62E-05	2E-08	-0.001570985	-0.010886008	-0.008979294
Gm11773	1	6.13E-05	5E-16	0.000893739	-0.008799893	-0.009011228
Plekhb1	1	7.26E-05	8E-11	0.000639627	-0.009944356	-0.009032287
Igflr1	7.96158E-06	1.00E+00	0.5897	0.012751428	-0.004414392	-0.009033589
Fopnl	1	1.81E-02	1	-0.004770214	-0.015427882	-0.009035316
Gm28050	0.008598647	1.00E+00	0.001	-0.007150356	-0.009115115	-0.009048833
Dio3os	2.9128E-05	1.22E-06	1E-19	-0.004811771	-0.009161681	-0.009054352
Leng1	1	1.01E-05	1	0.007446449	-0.021928134	-0.009076833
Zfp236	2.38439E-10	3.97E-07	1	0.043046303	-0.054301896	-0.009090808
Trim56	4.27579E-10	1.00E+00	1	-0.022039402	0.002532585	-0.009103828
Gm38416	1.63069E-07	1.07E-04	0.0003	-0.008914502	-0.013110035	-0.009134059
Mrps36	1	1.00E+00	0.0137	-6.13492E-05	-0.004739948	-0.009137647
Bok	1	1.00E+00	4E-08	-0.002380098	-0.005500074	-0.009151286
Srcin1	1	1.00E+00	4E-08	0.000595698	-0.007290144	-0.00918089
Gm28876	1	5.34E-02	3E-09	-0.001850907	-0.008452136	-0.009228099
2010315B0	1	2.34E-03	1	-0.012625226	-0.027784114	-0.009228963
Enho	5.29769E-17	1.01E-03	2E-13	-0.008071143	-0.008688822	-0.009230642
Gm17057	0.441068467	1.00E+00	2E-10	-0.004381437	-0.006392583	-0.009233497
Cadm4	0.393252723	2.12E-02	1E-14	-0.003886405	-0.007732339	-0.009254645
Gm36011	1.71562E-09	3.88E-02	2E-18	-0.005935649	-0.00694314	-0.009283383
Nr4a2	1	2.03E-03	2E-16	-0.00309584	-0.008149215	-0.009292226
Chchd7	1	8.16E-04	0.0712	-0.00391552	-0.014877826	-0.009299877
Cnpy3	1	5.50E-04	1	0.002917772	-0.023832664	-0.009309768
Suox	1	1.30E-04	9E-13	-0.003212605	-0.009769493	-0.009312979
Ank1	1	4.80E-03	5E-11	-0.001569282	-0.008480317	-0.0093187
Aars2	1	5.06E-04	1	0.007806543	-0.017505835	-0.00932122
Mef2b	1	1.00E+00	0.003	-0.005728473	-0.005530819	-0.009375792
Gm16548	1	1.55E-05	0.2525	-0.006013641	-0.0187454	-0.009385166
Sult1c2	1	4.72E-06	5E-16	-0.001378249	-0.00971382	-0.009387783
Arl10	0.008434322	1.00E+00	1E-05	-0.006522088	-0.005367973	-0.009390974
Fbxo42	0.043059392	1.44E-03	1	0.023182676	-0.032296483	-0.009394129
Ube2a	1	1.46E-02	1	-0.001401407	-0.015951425	-0.009397012
Fam126a	1	8.29E-27	1	-0.005681727	-0.040334393	-0.009403374
Ranbp17	1.30401E-12	9.88E-05	1	-0.017778771	-0.017848905	-0.009407644
Arl5a	1.0045E-13	1.58E-15	1	-0.037673578	-0.059942831	-0.00941361

Gm45051	1	2.78E-02	2E-06	-0.001516501	-0.009809184	-0.009427958
Colgalt2	0.001451332	3.87E-04	4E-11	-0.005395308	-0.009362374	-0.009454542
Zfp946	0.070315459	1.13E-13	1	-0.011718393	-0.035616308	-0.009456443
Plp1	1	2.54E-04	0.0023	-0.003807924	-0.01399301	-0.009495085
Gm10550	1	1.00E+00	0.0022	-0.004557333	-0.000153474	-0.009514742
Nmi	0.000539847	1.00E+00	1	-0.012421781	0.001745915	-0.009536085
Cuedc2	1	1.14E-03	0.0975	-0.001319054	-0.015542509	-0.009538183
Ccdc169	0.662051888	1.00E+00	5E-10	-0.004340439	-0.003459143	-0.009563372
Eepd1	3.4926E-18	1.00E+00	1	0.049939988	0.048449704	-0.009592345
Gm20619	1	1.83E-01	2E-08	-0.003237686	-0.008465245	-0.009596301
Adssl1	1	7.27E-01	2E-05	-0.004009828	-0.007742501	-0.009597205
Gm47771	1.99788E-12	8.24E-03	2E-11	-0.00770193	-0.008919339	-0.009613682
Zscan22	2.47306E-05	9.34E-06	1	0.017531564	-0.025669121	-0.009614532
4930481B0	1	1.29E-01	1E-05	-0.003132692	-0.009574267	-0.009614729
Rasgrp2	1	1.00E+00	0.0106	-0.001081754	0.000621302	-0.009626254
Runx2	1	1.00E+00	0.0003	-0.001057967	0.019770556	-0.009639008
Nos1ap	4.32414E-11	1.00E+00	1	-0.047324621	0.006085727	-0.009661497
Zfp936	4.93846E-05	4.77E-02	6E-08	-0.006735061	-0.00947812	-0.009669167
Mief2	1	1.54E-07	1	0.0056629	-0.029557634	-0.00967426
Zfp654	0.401467001	7.14E-08	1	-0.018590767	-0.04853031	-0.009693263
Zfp189	1	2.79E-06	1	0.006566689	-0.022307488	-0.00970135
Tmub1	1	2.97E-03	5E-09	-0.000851075	-0.009562768	-0.009705828
4930505N2	1	1.00E+00	0.0233	-0.00246234	-0.007171315	-0.009720716
Bcap31	0.007960472	1.00E+00	1	-0.01638942	-0.016081908	-0.009724655
Fbxo33	0.01171309	1.00E+00	1	-0.012084922	-0.010181524	-0.009748769
AV099323	0.019137032	5.16E-02	1E-05	-0.006180737	-0.010721493	-0.009765095
Cryz	1	1.17E-04	1	0.002858519	-0.023866143	-0.00976571
Vcl	1	9.62E-17	0.148	-0.004340068	-0.058209956	-0.009766662
Egfem1	3.99636E-11	1.23E-06	1E-09	0.0109519	-0.012114707	-0.009795181
Serpina12	3.81175E-20	8.51E-07	1E-22	-0.00754157	-0.009414519	-0.009821894
Gm47376	1	1.00E+00	0.0097	-0.000588839	-5.09704E-06	-0.009822708
Lypla2	4.34945E-06	1.00E+00	1	0.017756839	0.002691538	-0.009858914
Gm16567	1	9.88E-01	2E-09	-0.000348751	-0.00784503	-0.009920143
Zfp68	1	3.07E-05	1	-0.006024504	-0.020389575	-0.009954908
Zfp825	1	6.43E-04	1	0.004254016	-0.02174899	-0.009957222
Ccdc30	0.000686107	3.79E-04	0.0007	-0.008571949	-0.014755789	-0.009977177
Nr1d1	1	1.32E-05	3E-06	0.005873997	-0.014046851	-0.009977588
Gm45253	0.000993952	1.00E+00	0.0007	-0.008285411	-0.008757362	-0.010021222
Serpina3n	1.56925E-90	1.65E-13	0.3735	0.066212374	-0.034459086	-0.010060861
Rnpc3	9.01701E-05	7.36E-06	1	0.028884779	-0.040489316	-0.010063954
F13b	1	4.27E-04	1	-0.008715742	-0.036922415	-0.010068821
Cs	7.46512E-14	1.15E-09	1	0.045970116	-0.048744717	-0.010146443
Peli3	0.000845615	6.63E-04	9E-12	-0.006060288	-0.010014005	-0.010170009
Prss48	1	7.74E-02	0.0012	0.000165907	0.013708761	-0.010172116
4933433G1	1	1.00E+00	0.0074	-0.001920894	0.007039399	-0.010188625
4933406C1	0.062814149	1.00E+00	0.0016	-0.007365701	-0.002718206	-0.010204052
Gnal	1	1.00E+00	2E-06	0.001491851	-0.001768321	-0.010205538

Prrg1	0.056916892	3.73E-02	0.0041	-0.007739066	-0.011011405	-0.01021064
Mtfp1	1	1.00E+00	0.0387	-0.002658606	-0.010413151	-0.010211293
E430024P1	6.6591E-05	5.14E-01	0.0135	-0.009835838	-0.011706433	-0.010236987
2610008E1	1	4.58E-09	1	-0.010211638	-0.04402206	-0.010243742
Gm30097	1	1.00E+00	0.0125	-0.00199591	-0.009470353	-0.010251879
Actn4	6.04205E-40	1.00E+00	1	0.075945705	-0.018901088	-0.01025662
Noa1	1	1.00E+00	0.046	0.000874434	-0.0048937	-0.010259406
Klf1	0.062854514	7.10E-06	3E-10	-0.005430073	-0.012052461	-0.010291026
Rdh5	0.000645024	9.27E-04	1	-0.013554716	-0.021541966	-0.010301517
Gvin1	7.32413E-30	1.00E+00	4E-09	-0.013065667	0.013713177	-0.010315339
Fkbp9	1	8.68E-07	1	-0.005650554	-0.024495548	-0.010317681
Gm47689	1	1.00E+00	5E-06	0.003102818	-0.008986914	-0.010332097
Tdrd7	0.001699506	7.21E-02	1	-0.021740257	0.044931646	-0.010341429
Gm17133	1	1.00E+00	5E-06	-0.003764275	-0.004165675	-0.010361254
5430403G1	2.79304E-05	7.42E-07	7E-05	-0.00898822	-0.01663952	-0.010371008
Sema6b	1	1.00E+00	1E-06	-0.002559507	-0.001514893	-0.010374919
Fam107a	0.177447025	3.62E-07	2E-21	-0.004138626	-0.010272247	-0.010382781
Cbfa2t3	0.000125843	1.00E+00	8E-12	0.008183214	-0.003263069	-0.01038413
Nlgn2	0.270502807	6.14E-05	5E-16	-0.004205993	-0.00975397	-0.010389915
Vmn1r90	1	1.07E-08	6E-25	0.001126399	-0.010424904	-0.010424904
D830032E0	1	1.00E+00	6E-14	-0.000844012	-0.005398419	-0.010427013
Zc3h12a	1	1.00E+00	0.0064	-0.003590476	0.009443401	-0.010437167
Dzank1	1	1.67E-06	2E-19	-0.000553465	-0.010195923	-0.010442986
Maged1	1	1.39E-15	0.6963	0.001828105	-0.033256984	-0.010443442
Gnai3	2.24955E-13	1.00E+00	1	-0.035652318	-0.012909268	-0.010464623
Trip10	1	2.56E-04	0.0074	0.001532717	-0.016712705	-0.010471331
Prcp	1	1.00E+00	0.0043	-0.000317036	0.01675228	-0.010472247
Gm50306	0.00854688	2.17E-07	5E-22	-0.00439027	-0.010352579	-0.010499957
Proca1	0.005532227	5.51E-02	3E-10	-0.005881535	-0.009208569	-0.010528436
Epha7	0.033981112	6.23E-02	6E-15	0.006011852	-0.007087565	-0.01054564
Lsamp	1.10062E-22	1.00E+00	0.0005	0.020856763	-0.008159964	-0.010549577
Kcnb2	1	5.86E-02	2E-17	-0.003376469	-0.007883034	-0.010565594
Haus7	1	7.76E-08	0.0084	-0.00140512	-0.020728958	-0.010574418
D630039A0	1	9.78E-02	8E-06	-0.001275891	-0.010840915	-0.010584686
Slc39a1	0.081982195	6.60E-01	4E-05	-0.007054254	-0.009670145	-0.010590671
Crip2	1	1.68E-03	0.0047	-0.003018968	-0.014997608	-0.010603183
Zfp280d	1	8.48E-06	1	0.006058282	-0.044470046	-0.010610188
Wars2	1	2.62E-08	1	0.008953809	-0.042371687	-0.010633506
Tmem245	1	2.18E-05	1	-0.009677918	-0.048504124	-0.010634862
Zfp30	1	1.89E-08	3E-05	-0.004169446	-0.0174331	-0.010644846
Mfsd7a	1	6.86E-01	7E-05	-0.002720362	-0.01008657	-0.010647171
Yeats2	1	1.87E-13	1	-0.013888711	-0.052952494	-0.010678069
Gm47368	1	1.00E+00	7E-05	-0.001035818	-0.003637136	-0.010707241
Mir670hg	0.002884795	2.06E-02	0.0001	-0.008223858	-0.013027704	-0.010718095
Ugcg	0.002889164	3.83E-05	1	-0.017159447	-0.024209833	-0.010722752
Timm17b	1	1.00E+00	0.0005	0.001489243	-0.006141086	-0.010729852
Gm41668	0.009703075	8.83E-09	2E-22	-0.004505171	-0.011131578	-0.010741585

4930484I04	1	1.00E+00	0.0001	0.005004932	-0.007883027	-0.010748022
Gm26885	0.000111544	1.00E+00	5E-10	-0.007300975	-0.006997396	-0.010756305
Gm31084	0.000366664	1.00E+00	0.3259	-0.013137348	-0.009766177	-0.01076232
Catsper2	1	6.30E-07	1	-0.006583694	-0.034022172	-0.010775976
Setd1b	1	6.00E-04	1	0.002309733	-0.028383509	-0.010821993
Nol4	0.023668541	1.00E+00	9E-05	-0.007422982	-0.00675065	-0.010826229
Mtx2	0.008578581	1.00E+00	1	-0.023031722	-0.018774292	-0.010833604
Cidec	1	1.00E+00	0.0088	0.000953814	-0.012463051	-0.010859895
Gm4409	0.80940303	1.00E+00	6E-06	-0.006049887	-0.004600918	-0.010903808
Tppp	1	4.58E-04	1E-15	-0.002108377	-0.009986376	-0.010917223
Gpr135	1	1.44E-05	0.0277	-0.002642577	-0.019419411	-0.010938999
Taf8	1	1.29E-05	1	0.010685034	-0.024618035	-0.010956832
Gm36908	1	1.00E+00	2E-14	0.004634257	-0.006141094	-0.010978707
Gm15890	1	5.25E-01	3E-17	-0.004078687	-0.007307142	-0.010979075
Gm35696	2.53473E-23	1.00E+00	1	-0.0416975	-0.006377887	-0.010985248
Maea	1	1.87E-07	1	-0.00833319	-0.034414433	-0.011013319
Gm21887	6.69709E-07	1.00E+00	0.2691	-0.013254798	0.005623118	-0.011017074
Gm22146	1.80278E-13	4.04E-07	1	0.027008636	-0.029199039	-0.011025522
Dyrk1b	1	3.90E-05	0.0066	-0.00235228	-0.018759261	-0.011028547
Gm11613	3.76758E-10	1.00E+00	0.0101	-0.014013089	-0.004274693	-0.011036305
Mad2l2	1	1.00E+00	0.0004	0.004333277	-0.00873719	-0.011038376
Gpbp1	1	4.51E-02	1	0.00543857	-0.027460656	-0.011039065
Dusp22	1.51039E-07	1.00E+00	1	-0.021398721	-0.017412203	-0.011069772
Zfp384	1	1.66E-08	1	0.014377772	-0.040444195	-0.011109871
Rbbp9	1	2.07E-03	0.0002	-0.004886225	-0.01443178	-0.011150572
C330007P0	7.77097E-05	3.46E-07	1	-0.018728653	-0.033948748	-0.011168559
Tubgcp3	1	2.94E-09	1	0.011832387	-0.040190043	-0.011185283
Purb	1	9.06E-04	1	0.004191537	-0.026399283	-0.011197304
Snapin	1	1.92E-02	0.0001	0.001244649	-0.012564122	-0.011210067
Gm32872	1	6.13E-05	1E-25	-0.001844272	-0.009250169	-0.011240463
Gm11337	1.90003E-08	3.09E-05	6E-05	-0.011259569	-0.015832587	-0.011243732
Pias3	1	1.00E+00	2E-06	-0.00065028	-0.003598511	-0.011257427
Gm45895	0.000148906	2.51E-09	2E-26	-0.005169795	-0.011281678	-0.011281678
Tuft1	2.82998E-46	6.65E-05	1	-0.044403343	-0.029572508	-0.011318962
Zap70	1	5.11E-03	4E-18	-0.00413923	-0.008925847	-0.01132227
Rfx2	1	8.73E-05	4E-16	-0.000947086	-0.01058876	-0.011340799
Gm28875	1	7.09E-02	5E-16	-0.001755969	-0.008290686	-0.011378525
Gm49494	1	4.45E-01	7E-05	-0.005353763	-0.011467288	-0.011435208
Cfdp1	1	3.81E-03	1	-4.9043E-05	-0.03344007	-0.011453318
Magix	1	1.09E-06	7E-06	-0.00283293	-0.016729101	-0.011470519
Zc3h12b	0.566326986	1.00E+00	0.0001	-0.006818476	-0.006752515	-0.011493083
Clic5	2.57776E-10	1.53E-05	3E-21	-0.007473846	-0.010020525	-0.011493711
Gm15336	5.19509E-05	2.21E-04	3E-11	-0.007234567	-0.011236439	-0.011507546
Gm34333	2.43971E-06	2.60E-08	2E-27	-0.005570046	-0.010899317	-0.011510379
Cdon	1	1.00E+00	2E-12	0.000651305	-0.007832166	-0.011515913
F3	1	1.50E-01	1E-12	-0.003838102	-0.008995073	-0.01153484
A330040F1	0.000290716	3.47E-05	1	-0.023149709	0.056295343	-0.011540816

AW554918	1	1.12E-02	1	-0.014389562	-0.028828223	-0.011559251
Gm17231	1	1.02E-03	0.0002	0.006344089	-0.014841143	-0.011573819
Sftpd	1	1.19E-06	1E-22	-0.003121802	-0.010813574	-0.011589394
Selenow	0.020664605	1.00E+00	0.2709	-0.011699311	0.015005559	-0.011589735
Efnb1	1	1.00E+00	0.0002	0.000757819	-0.006741454	-0.011652044
Vegfb	1	1.41E-05	1E-06	0.002425928	-0.015185593	-0.011665654
Extl1	1	1.00E+00	1E-05	-0.00646617	0.002136485	-0.011683093
Gm6614	0.545544241	1.39E-08	1E-23	0.005185265	-0.011720343	-0.011691146
Nrxn3	1.69764E-14	3.22E-02	7E-10	0.016110365	-0.009548887	-0.011698611
Sytl3	1	1.00E+00	1E-10	-0.001577871	-0.002505491	-0.011721844
Ppme1	0.032780887	7.45E-16	1	0.020735156	-0.058293529	-0.011730749
Gm49926	1	2.17E-03	6E-12	-0.001491799	-0.011195797	-0.011751814
Gna14	1	1.41E-02	1E-17	-0.002599273	-0.008912038	-0.011760336
Ces4a	0.021121249	3.81E-09	1E-25	-0.00456284	-0.01174356	-0.011770672
E130310I04	1	1.00E+00	3E-11	-0.004204063	-0.00784539	-0.011963773
Gm15401	1	2.50E-04	5E-15	-0.002274973	-0.011344374	-0.011971854
P2ry1	1	5.01E-06	4E-22	-0.000136385	-0.010742381	-0.011985078
Gstm6	1	1.92E-01	2E-08	-0.004373542	-0.010325371	-0.011987686
Ttc21b	1	5.42E-01	1E-06	-0.002780546	-0.009505802	-0.011988118
Gm46348	1	1.00E+00	2E-06	0.001506336	-0.00420752	-0.011993418
Gm13919	1.78606E-15	1.00E+00	0.0002	-0.015583224	-0.005857381	-0.012006953
Cul7	1	1.33E-05	8E-15	-0.002886772	-0.011836174	-0.012034571
Zfp667	1	8.08E-03	2E-11	-0.004060175	-0.01083277	-0.012042241
Spata24	0.00044673	7.18E-10	1E-06	-0.008607331	-0.020010012	-0.012052501
Dhx58	1	1.00E+00	6E-09	-0.003777847	-0.007490938	-0.01206157
Fhod1	1	1.00E+00	7E-14	-0.003420001	-0.00404414	-0.012066323
Gtf2e1	1.4183E-10	8.64E-05	1	-0.029566205	-0.033791767	-0.012071164
Tex261	1	1.00E+00	0.0053	-0.001644634	-0.011061877	-0.012090993
Pik3r1	7.85695E-30	1.00E+00	0.0004	-0.074059663	-0.01054144	-0.012092575
Impa2	1	1.00E+00	1E-09	0.003015881	-0.008144549	-0.012106653
Dnah7b	1	1.00E+00	2E-08	0.003737314	-0.001796298	-0.012141542
Trim47	1	1.00E+00	2E-06	-0.004431891	0.003941894	-0.012189413
Plcb3	1	1.00E+00	0.003	-0.00488169	-0.012200835	-0.012192796
Gm4316	6.52705E-25	5.44E-04	1	-0.033272296	0.029010419	-0.012208678
Gm13021	1	1.17E-11	1E-18	-0.001986896	-0.014947263	-0.012210504
H1f0	1	8.60E-04	0.0077	0.000755088	-0.019513438	-0.012229148
1700113H0	2.91726E-07	7.44E-02	7E-19	-0.007195054	-0.008617138	-0.012235817
Cpt1b	1.6464E-08	1.73E-04	0.1577	0.01837729	-0.020813579	-0.012242253
4930483K1	1	3.08E-02	9E-12	-0.003686766	-0.010548712	-0.01224774
Ubtf	0.007020048	9.72E-01	1	0.019400405	-0.016731377	-0.012267095
Astn2	1	1.00E+00	1E-06	-3.59833E-05	-0.005312431	-0.012269008
Trappc6b	1	5.98E-05	1	0.013696094	-0.026560088	-0.01227744
Syf2	1	4.39E-03	0.2964	0.003252154	-0.019094319	-0.012290274
Pomt2	1	6.60E-03	0.0056	0.002520326	-0.015956737	-0.012322532
Gm17023	1.35985E-12	4.96E-04	5E-11	-0.010716349	-0.012621673	-0.012341393
Lcp1	1	1.02E-09	1	0.021324472	0.107193521	-0.012355097
Gm12724	0.15780764	8.20E-04	2E-19	-0.004939554	-0.00989991	-0.012403651

Gchfr	1	1.01E-04	7E-12	-0.004074365	-0.012265574	-0.012426986
Fam149b	2.8363E-05	1.00E+00	1	-0.020429764	-0.010302762	-0.012471405
Mapk4	0.000279222	1.52E-06	4E-11	-0.014996378	-0.015829315	-0.012479542
Smad2	1	2.92E-10	1	0.001857221	-0.041656322	-0.012512503
Dcdc2b	0.483411192	1.85E-08	2E-10	-0.005937861	-0.016919138	-0.012574865
Gm960	0.051658181	3.34E-03	2E-10	-0.006526415	-0.011581446	-0.012575566
Zfp24	1	1.41E-06	1	0.006110522	-0.031911299	-0.012582234
Arhgef9	1.26623E-24	6.87E-21	0.3435	-0.026831451	-0.041891888	-0.012610327
Gm50138	1	9.32E-08	6E-17	-0.003132491	-0.013607826	-0.012641536
Azin2	3.40406E-08	2.01E-07	1E-18	-0.007870195	-0.013148622	-0.012666463
Smarcc1	1	6.48E-03	1	-0.002375646	-0.03094893	-0.012708548
Col20a1	1	1.00E+00	1E-06	-0.001297246	0.005392496	-0.012728466
Vezf1	1	1.89E-02	0.0406	-0.000246577	-0.018388482	-0.012741696
Arl2	1	8.92E-01	0.0009	-0.003199607	-0.012747735	-0.012788908
Serpini1	1	1.87E-03	8E-21	-0.001385	-0.009617018	-0.012801055
44260	3.50964E-23	1.63E-02	1	-0.047382069	-0.031474113	-0.012808972
Nfatc1	8.20683E-05	1.00E+00	1	-0.017901439	0.002323043	-0.012817502
Capn8	1.02609E-05	6.33E-08	9E-23	-0.006434023	-0.012646835	-0.012826752
Zfp85os	1	2.24E-02	9E-10	-0.00116812	-0.01169575	-0.012827804
Zfp563	1	1.00E+00	0.0098	-0.008331046	-0.010603425	-0.012842898
Tcf12	1.95872E-13	1.00E+00	1	-0.054079596	0.045738332	-0.012880148
1700018L0	8.22755E-33	1.00E+00	3E-09	-0.016589055	-0.003873262	-0.012902978
Izumo4	0.623441237	3.23E-14	0.0036	-0.008805528	-0.029486336	-0.012930193
Eno3	1	1.81E-01	9E-08	-0.001981446	-0.011727851	-0.012937486
Slain2	1	2.58E-09	1	0.018489175	-0.048502939	-0.013001398
Med15	1	7.57E-17	1	0.006502925	-0.070825912	-0.013034434
Zbtb9	1	1.00E+00	4E-06	0.000935119	-0.005797445	-0.013054082
Cela1	0.002270164	4.97E-02	2E-14	-0.00681375	-0.010261614	-0.013054974
Slc12a5	0.013418286	8.28E-08	1E-21	-0.005535524	-0.012749824	-0.013065155
Atxn7l3	1	2.47E-04	0.0759	0.009377879	-0.022133275	-0.013142066
Ccdc61	1	1.04E-05	0.1291	-0.007577986	-0.024048675	-0.013160582
Gm44662	1	1.11E-02	9E-22	0.003159432	-0.009512028	-0.013174229
Zfp260	1	4.32E-03	0.7349	-0.004890305	-0.023349367	-0.013185523
Myh10	8.96008E-07	3.07E-02	0.0055	-0.014678814	-0.017817874	-0.013187526
Htatsf1	1	8.10E-11	0.0635	0.001271695	-0.031974004	-0.013207886
Cep250	0.000568098	1.00E-04	1	-0.018544416	-0.027135644	-0.01321862
Gm35339	1	5.14E-10	2E-10	0.001225737	-0.018230523	-0.013222196
Zkscan5	1	1.55E-04	1	0.000501667	-0.025535501	-0.013234425
Uck1	1	8.93E-10	0.0969	-0.007700475	-0.030073214	-0.013240146
4933404O1	1	5.07E-10	3E-17	-0.003646747	-0.015568243	-0.01325095
1810053B2	7.15605E-26	2.81E-10	2E-15	0.019044549	-0.016240106	-0.013255442
Bad	1	1.41E-06	9E-05	0.002303346	-0.020033511	-0.013260285
Slc29a3	1	1.00E+00	9E-08	-0.006440214	0.000820382	-0.013314588
Zfp963	1	1.40E-05	0.0049	-0.004793747	-0.02223017	-0.013325293
Fgfrl1	1	6.30E-03	8E-08	-0.002356486	-0.013619391	-0.013326194
Gm595	1	4.91E-10	6E-25	0.001015337	-0.013774253	-0.013343109
Gstm7	1	1.98E-06	1E-06	-0.000283	-0.018486538	-0.01334726

Csrnp2	0.001229926	7.13E-01	7E-09	-0.008421661	-0.010813036	-0.013348663
Sphk2	1	1.29E-05	0.3143	-0.003781832	-0.026233599	-0.013356959
Gm44421	1	1.95E-08	6E-18	-0.002612553	-0.014497124	-0.013379284
Gm43936	1	1.09E-09	4E-20	-0.002039319	-0.014832063	-0.013381384
Trmt61b	1	3.56E-02	1	-0.005326385	-0.027193483	-0.013418986
Skint5	1	1.00E+00	3E-11	-0.001046933	-0.008243362	-0.013443308
Gm4673	1	6.83E-05	3E-14	-0.000828494	-0.013017647	-0.013449081
Slc35f1	1	1.97E-02	3E-17	0.000320066	-0.010656695	-0.013457571
Gpr12	1	2.58E-12	5E-32	-0.003831045	-0.013802799	-0.013503737
Gm47772	1	2.33E-02	1E-09	-0.006169444	-0.012777549	-0.013517682
Gm21781	1	1.00E+00	1E-06	0.000386747	-0.007949353	-0.013535945
4930599N2	1	1.00E+00	0.0001	-0.007652364	0.019608447	-0.013539538
Zfp266	0.332480946	4.60E-02	1	-0.016750256	-0.026368563	-0.013578159
Nfe2l3	1.50172E-13	1.22E-09	5E-21	-0.009459545	-0.01479632	-0.013595904
Wdr62	0.004246826	7.93E-05	5E-09	-0.008340217	-0.014276154	-0.01360423
Klhl7	1	1.44E-14	1	-0.002934283	-0.049500976	-0.013625218
Tor3a	1	1.00E+00	0.0013	-0.003469775	-0.000880994	-0.013700824
Dtwd2	1	1.39E-01	0.0211	0.007352388	-0.016010318	-0.013708087
9630028H0	1	1.00E+00	0.0067	-0.0049756	-0.007864089	-0.013731312
Gm10069	0.068145297	3.79E-09	1	-0.016702964	-0.041169184	-0.01374478
Slc29a1	6.1112E-303	7.03E-12	1	0.22696789	-0.074447763	-0.013746891
Ppp2cb	0.002412492	3.25E-02	1	-0.017139013	-0.022047665	-0.01375378
Ammecr1	0.311778071	1.00E+00	0.0011	-0.010301559	-0.008011164	-0.013777569
Eva1c	1	4.18E-03	3E-21	-0.002776506	-0.009490526	-0.01380687
Gm10649	5.13245E-27	1.00E+00	0.0005	-0.021832513	-0.009776439	-0.013826602
Jchain	0.000349754	1.14E-11	2E-23	-0.006149565	-0.015161924	-0.013835804
Tgds	1	2.70E-03	0.0233	0.003432526	-0.019892368	-0.013838018
Bhmt2	5.0926E-133	7.18E-93	1	0.14627406	-0.178607396	-0.013873325
Gm3336	1	1.69E-01	1E-13	-0.00447976	-0.010453511	-0.013878665
Tfg	1	2.23E-13	1	0.00207592	-0.049791703	-0.013883872
Anxa4	3.39076E-07	6.91E-01	1	-0.021320781	0.037870319	-0.013884183
Cpeb1	1	4.20E-11	0.0106	-0.006288303	-0.031374408	-0.013909609
Kcnip1	1	4.07E-02	2E-14	-0.003154784	-0.01083552	-0.013918224
8430426J0	3.77801E-14	5.41E-02	2E-05	-0.016543977	-0.01501521	-0.013955988
Usp35	1	1.67E-09	1E-14	-0.001686162	-0.016870772	-0.013968286
Dennd4b	1	1.00E+00	1E-06	-0.001041741	0.002173542	-0.013983046
Oxsr1	1	4.64E-01	7E-08	-0.002931941	-0.011884026	-0.013984135
Fgfr1	0.021983127	2.42E-03	1E-21	-0.006101026	-0.010568637	-0.014019723
Snx16	1	1.00E+00	0.0263	-0.005719025	-0.006739888	-0.014022722
Crtc2	3.45445E-09	7.23E-02	0.0466	0.022950262	-0.018303435	-0.014028687
Slc16a12	0.640681947	5.99E-21	1	0.016281633	-0.074627905	-0.014036866
Smim19	1	8.15E-01	0.0011	-0.008176985	-0.015172567	-0.014083353
Gbp7	1.20274E-11	1.30E-01	3E-14	-0.01135605	-0.006493706	-0.014090855
Iws1	1	4.28E-15	1	-0.001702373	-0.071265469	-0.014102716
Hpx	2.95845E-89	8.54E-04	1	-0.093704701	-0.029312321	-0.014115911
Slc13a5	0.106824719	2.28E-10	2E-30	-0.004819831	-0.013579214	-0.014123117
Rab32	1	2.71E-01	0.0004	-0.007851696	-0.01188123	-0.014127645

Fem1c	2.24729E-22	1.00E+00	1	-0.033290801	-0.008881106	-0.01413243
Gm14097	0.139709939	1.26E-08	3E-29	-0.00460142	-0.013001492	-0.014147842
Cdc14a	1	1.02E-01	1E-12	-0.00205975	-0.008900729	-0.014151017
AC163685.	1	1.00E+00	0.0008	-0.000480949	-0.010500775	-0.014184498
Gm36860	1	2.15E-07	1E-31	-0.002024286	-0.012103656	-0.014204484
Tmem37	0.02271807	1.16E-07	4E-23	0.007005448	-0.012873278	-0.014206889
Hhex	1.4497E-35	4.88E-21	1	-0.05609122	-0.072962499	-0.014244626
Card10	1	5.26E-01	0.0002	-0.007719936	-0.013781452	-0.0142652
Acadm	3.36552E-52	1.00E+00	1	0.097704112	-0.000988663	-0.014267326
Rdh14	0.000586042	5.97E-03	6E-06	-0.010353585	-0.01501729	-0.014286414
Rps6kc1	1	2.79E-05	1	-0.008932327	-0.034761733	-0.014318077
Gm42604	0.004224651	5.01E-11	3E-24	-0.006091399	-0.015402019	-0.014367463
Pknox1	7.17865E-08	9.00E-19	1	-0.024575201	-0.054387243	-0.014377664
Rilp	1	2.42E-12	0.0002	0.005232891	-0.028205516	-0.014389333
Cmtm6	0.002033253	1.00E+00	7E-07	-0.009863217	-0.005167172	-0.014395245
Gm26542	1	1.00E+00	0.0124	0.001005178	-0.006233735	-0.014398613
Tram1	1	1.30E-13	1	0.006468675	-0.046253544	-0.014401142
Sh2b2	6.23243E-07	1.00E+00	1E-14	-0.009300029	0.00298142	-0.014404734
Gm14372	4.25201E-09	1.20E-03	5E-21	-0.008607795	-0.011089693	-0.014460335
C9orf72	0.143506058	1.28E-02	0.9569	-0.014770324	-0.02116445	-0.014492588
B3glct	1	1.00E+00	6E-11	0.00068154	-0.006319799	-0.014553843
Xaf1	7.23192E-08	1.00E+00	1E-17	-0.00926437	0.002045373	-0.014559015
4930401C1	1	8.41E-05	1E-22	0.001194915	-0.011478201	-0.014564913
1700007F1	0.000620723	3.58E-06	1E-22	-0.006652821	-0.012975349	-0.014568144
Tcn2	1	2.31E-06	0.0001	-0.004990171	-0.019837874	-0.014570919
Rdh16f2	1.6268E-159	5.70E-85	0.0031	-0.162071809	-0.180977849	-0.014598501
Fzd5	9.92889E-05	4.88E-07	2E-21	-0.007072555	-0.013941983	-0.014630081
Csdc2	2.67591E-26	1.22E-06	9E-25	-0.012082667	-0.013112354	-0.014640086
Aph1a	1	1.00E+00	0.0047	-0.005886954	-0.012848203	-0.014646108
Tsga10	1	2.97E-01	5E-07	-0.002980745	-0.011534228	-0.014646329
Gm47271	1	1.00E+00	5E-08	0.001983761	-0.002494739	-0.014651466
Gm15611	1.86825E-53	1.00E+00	7E-20	-0.017408863	-0.002169578	-0.014655731
Pald1	9.4129E-06	7.05E-10	0.0049	-0.016324031	-0.030889745	-0.014686089
Leng9	0.163249337	1.00E+00	1E-08	-0.007830983	-0.008221518	-0.014692671
Zfp964	1	4.18E-09	2E-22	-0.004312736	-0.015005971	-0.014709273
Cbx7	1	1.00E+00	1E-07	0.000675773	-0.008403942	-0.014715345
Gm14966	1	3.41E-08	0.0091	0.003420798	-0.028403594	-0.014724371
Rilpl1	1	3.38E-05	1E-20	-0.004954658	-0.011571549	-0.014724847
Napb	1	1.04E-08	0.1093	0.001689948	-0.03303448	-0.01474905
Gm12798	1	4.04E-11	7E-32	-0.002437969	-0.014180075	-0.014796552
Tgfbr3	1	1.32E-06	1E-10	-0.003071534	-0.013725416	-0.014807537
Zkscan17	1	1.00E+00	1E-08	-0.004265793	-0.008592566	-0.014814527
Sh3bgrl2	4.01357E-25	8.79E-08	1	-0.046106048	-0.042143206	-0.014817134
Zfp335os	1	6.43E-05	0.0176	-0.004233771	-0.024183793	-0.014831802
Fam117a	1	1.35E-07	1E-09	-0.005661738	-0.017557687	-0.014850623
Gm49189	0.006964337	1.66E-01	1E-10	-0.008376304	-0.01230299	-0.014857246
2900097C1	1	9.12E-03	0.0658	-0.000649086	-0.019206702	-0.014878724

Tmem39b	1	1.06E-04	4E-05	-0.002539054	-0.019372626	-0.014892374
Sfr1	1	2.01E-02	2E-05	0.003727512	-0.016299436	-0.014907738
Gm9968	1	2.45E-05	5E-13	-0.0033515	-0.015697683	-0.014909249
1500035N2	0.032849586	1.00E+00	0.1308	0.014434698	0.003980082	-0.014950121
Jade2	2.85447E-05	1.00E+00	0.0321	-0.01699588	-0.013935316	-0.014950885
Zfp324	1	8.02E-06	6E-07	-0.006542047	-0.019550623	-0.014959701
Calm1	1	1.00E+00	0.0011	-0.008656188	-0.006585054	-0.014967916
Fam124a	0.020275917	6.62E-01	2E-10	-0.008183324	-0.00743373	-0.015004978
A430033K0	1	1.14E-08	0.0095	0.00057204	-0.029153618	-0.015039243
Gm9993	1	1.40E-04	8E-08	-0.005587869	-0.018092805	-0.015074968
Gm47469	1	5.03E-11	8E-07	-0.000460256	-0.025171227	-0.01513624
Rbms3	4.74415E-51	8.03E-02	8E-09	0.037060288	-0.009075804	-0.015168713
Trim14	7.58179E-92	1.00E+00	1	-0.102401782	0.043906102	-0.015171531
6430590A0	1	6.24E-04	1	-0.000251579	-0.02806871	-0.015207429
Zbtb8os	1	4.07E-03	0.0219	-0.007839101	-0.023024809	-0.015212019
Dnaja1	1	8.01E-25	1	0.013435227	-0.076144367	-0.015252164
Gm40770	4.29138E-07	1.00E+00	0.0008	-0.015265897	-0.009681817	-0.015258916
Zbtb37	1	2.76E-01	5E-05	-0.006799259	-0.013678406	-0.015337466
Olfm2	1	7.46E-03	6E-07	-0.006766545	-0.015972	-0.015373077
Gm29394	0.189384646	2.04E-06	8E-07	-0.008240822	-0.021246156	-0.015425168
Mospd1	4.42925E-05	4.44E-01	7E-05	-0.012878241	-0.012845819	-0.015426563
Lonrf1	2.68229E-09	1.00E+00	3E-07	-0.013822592	-0.003616015	-0.015429054
Wdr89	1	9.11E-08	5E-06	0.001253432	-0.023184536	-0.015432971
Gm46559	1	1.71E-01	3E-16	0.003703942	-0.010804641	-0.015442186
AA386476	1	2.43E-03	9E-06	-0.00512827	-0.017818706	-0.01550287
Atxn2l	1.14697E-23	1.64E-12	1	0.045224758	-0.045470507	-0.01550297
Selenos	2.6926E-06	1.37E-07	0.2518	0.021754249	-0.033015749	-0.015508878
H2-Q4	1	1.00E+00	0.0005	0.00055324	-0.002580581	-0.01553605
Letm2	1	3.44E-04	0.0077	-0.002588247	-0.022969693	-0.015538869
F11r	1	7.95E-08	1	0.002216441	-0.046589992	-0.015588011
Masp2	1	1.94E-103	1	-0.01931113	-0.184290338	-0.015607965
Lcmt1	1	8.93E-08	0.0013	-0.004974551	-0.026314025	-0.015608866
Prickle3	1	2.18E-03	1E-05	-0.006129934	-0.017855057	-0.015629195
Pithd1	1	1.00E+00	0.0003	0.007652453	-0.0137833	-0.015656247
Rcor3	0.01339905	1.17E-06	1	-0.017224231	-0.034392892	-0.015660275
Hnf4g	0.371550634	4.25E-06	3E-15	-0.006404504	-0.014053335	-0.015674211
Kif1bp	1	1.00E+00	0.0074	0.005955369	-0.014860032	-0.015703367
Sike1	0.008657825	1.00E+00	0.0013	-0.01197424	-0.012689543	-0.015734106
Mpst	0.226345525	1.90E-03	2E-14	-0.006776653	-0.013639859	-0.015735422
1700066M	1	2.09E-08	6E-08	-0.001456592	-0.022123269	-0.015751295
Kdelr1	1	9.81E-03	1	-0.007456708	-0.025342623	-0.015764017
Mup17	1	5.90E-01	4E-23	0.000289421	-0.007100926	-0.01576951
9430091E2	0.009077505	1.00E+00	0.0176	-0.013969941	-0.017018585	-0.015782681
Gm11944	0.095307443	2.01E-10	7E-30	-0.005564256	-0.015225967	-0.015796717
Fbxo46	1	1.00E+00	0.006	-0.006628727	-0.008589594	-0.015801415
Gm34408	1	1.00E+00	0.0015	-0.003108036	0.006305934	-0.015864223
Gnpda1	9.7923E-10	1.00E+00	0.0007	-0.018189569	0.007798051	-0.015873867

Chst13	1	1.00E+00	4E-06	-0.004307645	-0.013555629	-0.015881036
Degs1	1	1.00E+00	2E-06	0.005787734	-0.010804876	-0.01588795
Rnaset2a	1.114E-05	5.13E-01	0.0003	-0.013898064	-0.013718106	-0.0158915
Zfp60	1	1.15E-12	0.103	0.007102523	-0.042665508	-0.015917909
Mup12	2.41111E-12	3.65E-09	3E-35	-0.0089492	-0.013906756	-0.015933895
Cep68	1	1.00E+00	0.0155	-0.006330382	-0.010174881	-0.015944436
Mxi1	1.73835E-11	1.00E+00	0.273	-0.030336755	-0.017666546	-0.015963535
Clcn4	4.89741E-06	1.12E-03	0.0711	0.019854769	-0.023390902	-0.015973484
Bst2	1	1.00E+00	4E-06	0.000556359	-0.001442576	-0.015994957
Plekha8	3.47865E-06	2.45E-03	1	0.02548627	-0.028215576	-0.016045206
Tmem11	1	3.76E-05	0.0967	-0.006155718	-0.028761766	-0.016054435
Fam184a	1	9.45E-14	1E-28	0.005019046	-0.017665078	-0.016071325
Gm12473	1	3.79E-14	6E-34	0.000632657	-0.016655405	-0.016088837
Tmem120a	0.000456187	4.61E-05	0.0136	-0.01756224	-0.028253491	-0.016125987
Ube2n	0.966939319	1.13E-04	0.5849	-0.01332857	-0.028797539	-0.016143514
Sytl1	1	1.92E-04	4E-20	-0.004366758	-0.013079256	-0.016199628
Alg9	0.015328224	1.00E+00	0.0403	-0.013757844	-0.010708098	-0.016203029
Flt3l	1	7.38E-02	6E-11	-0.006142487	-0.012307202	-0.016219666
Steap2	1	1.00E+00	2E-16	-0.00329358	-0.009033822	-0.016408735
A130010J1	1	3.36E-06	7E-19	-0.005426785	-0.015660992	-0.016470615
Lzts3	1	1.00E+00	1E-09	-0.005293005	-0.009376709	-0.016477537
Neu2	1	1.00E+00	0.0005	-0.007337055	-0.009514721	-0.016493918
Gm46545	4.98592E-14	1.39E-09	5E-36	-0.009493778	-0.014425291	-0.016552115
Hdc	0.044396011	1.00E+00	8E-16	-0.007571802	0.0016594	-0.016592872
Zfp354b	1	1.61E-09	9E-17	-0.004593145	-0.018935786	-0.016600415
Foxj2	1	1.00E+00	3E-05	-0.001590573	-0.011878483	-0.016616852
Pink1	1	3.78E-03	0.005	0.009752641	-0.022374462	-0.016640484
Aamp	1	2.16E-09	0.0113	-0.000598316	-0.031930915	-0.016647324
Zfp420	1	2.96E-09	1E-08	-0.005180045	-0.022798293	-0.016651553
Gm20404	1	1.00E+00	2E-10	0.001543031	0.003364642	-0.016673879
A530072M	1	9.63E-14	0.0257	-0.012579828	-0.042641128	-0.0166801
Tmem263	0.115367955	6.32E-12	7E-09	-0.008534066	-0.025476107	-0.016692427
Pura	0.099634507	1.56E-07	1	0.019560991	-0.043106639	-0.016703141
Coa7	0.000237267	1.00E-02	0.0002	-0.013878649	-0.019931215	-0.01676422
Gm26916	1.47024E-09	1.53E-01	3E-20	-0.010465434	-0.009699153	-0.016765109
Ddx11	1	6.82E-15	0.0005	-0.009189935	-0.037486698	-0.016768815
Sec22c	0.262870468	3.61E-09	3E-13	-0.00748818	-0.020670791	-0.016782556
Tcp11l1	4.85286E-10	1.00E+00	1E-10	-0.014050579	-2.88548E-05	-0.016783143
Serpina7	5.42627E-26	1.75E-12	5E-36	-0.012390335	-0.016499834	-0.016909297
4930593A0	4.11599E-08	2.58E-15	9E-15	-0.010964249	-0.024397023	-0.016952678
Egln2	1	5.59E-04	0.0503	-0.00288325	-0.024019251	-0.016971105
Irf1	1	1.00E+00	5E-07	0.004452525	0.005125925	-0.016995785
Mmd2	1	7.73E-13	7E-38	0.002521333	-0.016441087	-0.017042416
Gm28376	1	3.02E-08	1E-22	-0.003660433	-0.016537997	-0.017088454
Hes6	1	1.43E-09	5E-17	-0.004532337	-0.019085817	-0.01709594
Rabac1	1	3.35E-05	6E-07	0.004954297	-0.019964662	-0.017115992
Odf3b	1	1.30E-12	9E-19	8.42633E-05	-0.020862831	-0.017161026

Emp2	1	8.48E-08	4E-33	-0.00347902	-0.014027971	-0.017168914
Sub1	1.44087E-07	1.35E-10	0.0005	-0.018202447	-0.034051772	-0.017181855
Adamts7	1	3.33E-14	4E-13	-0.001113019	-0.025449617	-0.017283981
Gm16364.1	1	8.47E-12	1E-19	-0.005540256	-0.020025681	-0.017302261
Hexb	1.03087E-06	1.00E+00	7E-18	-0.009949903	0.002959505	-0.017324851
Gm43598	1	1.31E-14	1E-18	-0.000160124	-0.022713614	-0.017353127
Slc25a44	1	8.67E-09	1E-05	0.007304201	-0.027446082	-0.017362458
Gm45411	1	6.79E-02	2E-21	-0.001561957	-0.01108507	-0.017377959
Gm16158	0.003627107	2.45E-07	5E-06	0.013946915	-0.025300957	-0.017384482
Auh	3.0268E-06	1.43E-06	1	-0.026990772	-0.043123815	-0.017420988
Spice1	1	5.18E-07	2E-06	-0.006378529	-0.024754022	-0.017431667
Gm14636	1	5.48E-01	4E-12	-0.004965944	-0.008753458	-0.017498235
Larp1	0.001830888	1.00E+00	1	-0.03182594	0.036385919	-0.017531577
Zfp787	0.590708661	3.21E-07	1	-0.0156675	-0.040981087	-0.017568899
BC049987	2.74051E-07	9.72E-16	1E-17	-0.010778632	-0.024419862	-0.017574491
Tollip	1	3.30E-03	6E-06	-0.001975849	-0.017666075	-0.01757796
Neto2	1	2.72E-02	5E-10	0.00011986	-0.015071128	-0.017586387
Hamp2	0.95717693	3.00E-05	3E-26	0.010029059	-0.014072918	-0.017586388
Vps9d1	1	7.74E-13	0.0058	0.003912998	-0.039792077	-0.017611919
Pank3	1	5.98E-07	0.007	0.000715005	-0.029891254	-0.01761986
Carhsp1	6.32805E-06	4.72E-05	2E-09	-0.012393405	-0.019298916	-0.017627904
Mex3c	0.003592545	2.44E-02	6E-06	-0.012415037	-0.017081162	-0.017648309
Rprd2	1	6.99E-08	1	0.010296341	-0.04692061	-0.017672834
Repin1	1	6.96E-03	3E-07	-0.007912093	-0.018782477	-0.017708908
Trim3	1	1.00E+00	2E-07	-0.003013427	-0.007625045	-0.017724322
Fbxo28	1	6.38E-06	0.5088	-0.010162171	-0.034347897	-0.017738387
Hif1an	1	7.06E-05	1E-05	-0.005174125	-0.022798602	-0.017776141
Itgb1	3.4013E-06	1.00E+00	1	-0.027996956	0.051111352	-0.017791605
Galnt1	1	6.03E-09	0.2196	0.000385524	-0.035713442	-0.01780344
Abi2	1	4.85E-04	3E-10	-0.003246056	-0.017399105	-0.017829574
Mfsd13b	1.59219E-17	6.72E-05	5E-07	-0.022370245	-0.017216978	-0.01784073
AI987944	1	1.04E-23	0.0001	-0.004019612	-0.04552686	-0.017895339
Cldn12	1	1.63E-06	8E-07	-0.008354904	-0.02488567	-0.01794782
Tapbp	3.92077E-06	1.00E+00	1E-06	-0.014545762	0.010525455	-0.017968967
Adamts6	3.25781E-06	1.00E+00	0.0002	0.023591963	-0.001324375	-0.018018006
Zmiz1os1	1	1.28E-03	7E-07	-0.008068217	-0.020857902	-0.018019148
Cep44	1	3.88E-14	1	0.011985139	-0.053269169	-0.018039424
Bcl2l2	1	1.72E-05	1E-05	-0.000967754	-0.024440644	-0.018040323
Gfpt1	0.190252467	3.20E-15	0.0398	0.017913586	-0.045116118	-0.018052945
Clic4	5.5789E-06	1.00E+00	1	-0.0459617	-0.012000569	-0.018063645
Enpp6	0.759679984	2.28E-10	1E-35	-0.00505985	-0.016108851	-0.018072537
Sertad3	1	1.44E-04	8E-16	-0.005083945	-0.016368098	-0.018087009
Csrp3	1	4.74E-17	2E-43	-0.002305783	-0.018314396	-0.018100977
Fam192a	1	1.88E-04	0.1843	0.001688009	-0.029928626	-0.018131138
Bag3	0.009000064	6.66E-11	1	-0.01938524	-0.051213983	-0.018145004
Rsf1os1	1	1.00E+00	3E-10	0.001385065	-0.003540444	-0.018154323
Dpp9	3.73401E-15	1.58E-05	1	0.038496187	-0.036199086	-0.018160801

Stxbp5l	0.007947581	2.55E-09	1E-32	-0.006414064	-0.016264565	-0.018170326
Pltp	0.004753541	1.00E+00	4E-14	0.011670303	-0.000165269	-0.0181828
Iqsec1	0.878223525	1.00E+00	0.0004	-0.012622843	0.025016457	-0.018190531
Zfp574	1	1.30E-02	1E-06	-0.004484127	-0.017994399	-0.018263571
Pttg1	1	1.00E+00	3E-06	0.003380991	-0.007478644	-0.018267378
Gcat	1	1.15E-14	1E-16	-0.003855804	-0.024763053	-0.018282222
1700093J2	4.51799E-12	1.10E-08	3E-11	-0.015687562	-0.023859287	-0.018284316
Cerkl	1	1.00E+00	0.0173	0.000858424	-0.017414776	-0.018294568
Cbx3	1	1.00E+00	0.0006	-0.005071067	0.002487876	-0.018297323
Zfp775	1	5.26E-12	2E-09	-0.006887018	-0.027495661	-0.018307829
Pqlc2	9.50089E-10	1.00E+00	0.1091	-0.024539377	0.000607915	-0.018353609
Hjv	1	4.49E-27	1	0.012627078	-0.070387037	-0.018370597
Vps41	4.03053E-23	9.16E-10	1	-0.05802265	-0.065745175	-0.018415835
Acot11	6.61065E-13	9.28E-07	1E-19	-0.012776926	-0.017204672	-0.018458081
Top1mt	3.75092E-09	7.63E-09	0.0009	-0.020199183	-0.034472179	-0.018502046
Vegfc	1	1.35E-06	0.0094	0.003041181	-0.032529324	-0.018505454
Pip5k1a	1	9.42E-14	1	0.002794423	-0.056873568	-0.018512136
Gm48226	1	4.88E-12	1E-14	0.002671246	-0.024040067	-0.018556383
Pcgf2	1	2.11E-05	2E-09	0.003968278	-0.021052551	-0.018589309
Ppp1r15b	0.000646127	2.51E-03	5E-05	-0.015396499	-0.021024241	-0.018590097
Ilkap	1	1.65E-07	0.5433	0.010665748	-0.037633834	-0.018606944
Ube2j1	1	8.85E-03	0.0041	0.000678678	-0.024295217	-0.018617706
Vps37b	1	1.00E+00	0.0072	-0.000492677	-0.015503767	-0.018621181
Cul3	4.9666E-05	5.17E-07	1	-0.027938493	-0.047431341	-0.018621531
Stx6	1.55772E-06	1.07E-04	1	-0.024756407	-0.031544451	-0.018666213
Slc17a4	4.24101E-20	6.92E-67	3E-05	0.046151387	-0.112285506	-0.018678752
Stat5a	1	1.76E-01	0.0051	0.001160126	-0.021989362	-0.018719038
Desi1	1	5.17E-03	0.27	0.015475559	-0.028316173	-0.018745803
Cldn34c1	0.003959919	2.03E-13	4E-36	-0.006729719	-0.018135841	-0.018746896
Mettl8	0.004287173	1.00E+00	0.0146	-0.01672815	-0.011661624	-0.018753944
Copg2	7.29616E-23	4.99E-05	1	-0.051697071	-0.040544558	-0.018798933
A430090L1	1	5.49E-06	5E-12	-0.006384075	-0.019966444	-0.018806897
Pmf1	1	2.27E-06	1E-11	0.004220414	-0.021216643	-0.018823765
Wdr66	1	1.64E-07	1E-21	-3.76488E-05	-0.01787969	-0.018845175
Gm7298	1	6.73E-05	3E-26	0.00362672	-0.014525522	-0.018872616
Ifitm3	1	3.38E-02	6E-26	-0.004037379	-0.010530243	-0.018882599
Nhs	1	1.14E-10	3E-35	-0.001482516	-0.016672236	-0.018883603
Eny2	1	7.85E-10	0.1109	0.002441708	-0.043151895	-0.018909587
Acbd3	1	1.01E-02	0.9264	0.002033338	-0.025922912	-0.018930194
Dnah8	1	4.42E-07	1E-33	-0.002396692	-0.014183647	-0.018944198
Naprt	1	9.39E-16	0.0001	0.004602375	-0.04202661	-0.018956245
Irf5	1.47977E-36	1.00E+00	2E-29	-0.017080722	0.003391187	-0.018959143
Cdk2	1	8.37E-07	4E-14	-0.002815077	-0.019540924	-0.018964899
Atg4d	1	2.55E-01	0.002	-0.004097093	-0.020645723	-0.018966338
Gm45250	1	3.79E-06	6E-17	-0.003057311	-0.018282287	-0.018975119
Dsp	3.23996E-10	1.00E+00	0.1673	-0.026386896	-0.002305286	-0.019092411
Pld2	1	2.60E-04	1E-15	-0.006016842	-0.015603901	-0.019142219

4933423P2	0.008731442	3.53E-01	4E-21	-0.007898093	-0.010802186	-0.019224826
Fam204a	1	5.01E-04	0.0027	-0.010637068	-0.026198241	-0.019252519
Dhrs11	2.04576E-18	2.28E-07	0.0003	-0.026289174	-0.029531241	-0.019265636
Trim30d	1	1.00E+00	8E-19	-0.006648363	0.030775902	-0.019291408
Rab18	0.000194895	1.48E-04	0.0119	-0.018072497	-0.030017265	-0.019300606
Vps72	1	1.00E+00	0.0005	-0.000129022	-0.009607503	-0.019335857
Gm28370	1	2.05E-07	4E-27	0.004263109	-0.016553152	-0.0193411
Plekhg2	1	2.74E-08	3E-21	-0.005940395	-0.018182499	-0.019348559
Klhl21	1	1.00E+00	2E-14	0.000103999	-0.004228977	-0.019382331
Pogk	1	1.00E+00	3E-14	-0.007301041	-0.00953568	-0.019388425
Gm17168	1	8.39E-15	5E-25	0.000299178	-0.022658225	-0.019404955
Slbp	0.078180194	6.42E-04	4E-07	-0.011693386	-0.020414931	-0.019434248
Gm15417	1	7.25E-07	7E-12	-0.00283104	-0.022488296	-0.019443478
Sypl	1	1.00E+00	7E-05	-0.005047164	-0.009042013	-0.019512858
Slc27a1	1	1.00E+00	2E-21	-0.006482298	0.001661004	-0.01953715
Ntm	1.89104E-33	7.62E-16	3E-36	0.022870481	-0.019768319	-0.019553777
Ogfrl1	1	4.26E-08	4E-22	-0.004875224	-0.016501001	-0.019584688
Tbc1d32	4.9379E-15	3.07E-05	0.4288	-0.037189782	-0.037018219	-0.019622413
Gria3	1.53982E-11	9.28E-16	7E-24	-0.013383229	-0.025166752	-0.019631344
Rgs12	1.35756E-22	5.41E-17	1E-25	-0.015204245	-0.023135688	-0.019642565
Zdhhc20	2.44658E-07	1.00E+00	0.0732	-0.024562013	-0.007279065	-0.019729299
Fkbp2	1	1.70E-16	1E-11	-0.002892983	-0.030930469	-0.019744405
Sigmar1	1	1.48E-12	0.0014	0.010266723	-0.041447889	-0.019751056
Ube2j2	1	6.90E-04	0.673	-0.006707086	-0.033174464	-0.019753675
Fam20b	0.012408363	1.53E-03	0.0009	-0.015074265	-0.024837463	-0.019780617
Proser3	1	1.19E-07	4E-15	0.001677593	-0.020571429	-0.019841776
Plxnb1	6.09353E-52	2.49E-13	0.001	-0.050157283	-0.044625685	-0.019847794
Nab2	7.7514E-12	1.45E-02	4E-23	-0.012581671	-0.012236141	-0.019852625
Parp11	1	5.47E-01	1E-11	-0.007352237	-0.005519014	-0.019868431
Ythdf1	1	3.58E-08	0.0029	-0.009302232	-0.035211848	-0.019869585
Hdac1	8.09362E-07	9.90E-09	0.0021	-0.020348177	-0.035260072	-0.019900995
AW011738	3.08832E-09	3.76E-03	3E-21	-0.011453482	-0.013807506	-0.019927395
Sft2d2	1	1.24E-05	2E-10	-0.003330945	-0.019289955	-0.019962393
Daglb	0.241886737	1.00E+00	1E-05	-0.012160862	-0.005264445	-0.019965264
Ctcfl	1	2.78E-17	6E-22	0.003154688	-0.025697514	-0.019968575
Zfp516	1.1569E-25	1.00E+00	9E-06	-0.034035055	-0.004407482	-0.019987916
1700034P1	1	4.51E-05	1E-11	-0.005182296	-0.02036705	-0.020009911
5830444B0	1.2472E-32	1.00E+00	6E-35	-0.015632138	-0.00861582	-0.020016095
Hnrnph2	1.66731E-05	1.06E-01	5E-08	-0.015425466	-0.01569149	-0.020032204
4930455C1	1	1.00E+00	1E-07	0.005909334	-0.008798869	-0.020033661
Pgrmc1	5.49277E-05	4.37E-08	1	-0.026899918	-0.045517355	-0.02010682
Gm11437	4.58055E-20	7.95E-21	1E-21	0.021809885	-0.02869604	-0.020154034
Madd	1	1.58E-01	0.0004	-0.002987402	-0.014231805	-0.020154896
Nasp	1	1.00E+00	0.0003	5.54203E-05	-0.009864399	-0.020202754
Maf	1	1.00E+00	4E-14	-0.008239228	0.020857209	-0.0202427
Ube2g2	1	4.82E-04	0.0721	0.012814058	-0.032139012	-0.020268657
Zfp821	1	1.10E-02	1E-20	-0.00580804	-0.012379887	-0.020287772

Med26	1	6.97E-14	1	0.007126757	-0.060718019	-0.020292084
Casc4	1	8.38E-13	7E-40	-0.003233587	-0.01870638	-0.020294747
Ikzf4	1.72682E-20	8.57E-15	4E-42	-0.012569267	-0.01957149	-0.020325624
Usp42	1	1.61E-10	3E-07	-0.000869802	-0.032011701	-0.020329886
Zswim3	1	6.46E-04	5E-11	-0.007134055	-0.018870542	-0.020342974
Arih2	1	4.33E-04	1	-0.005681565	-0.035757531	-0.02034491
Lmtk2	1	8.53E-06	0.2143	0.002422323	-0.036099719	-0.020382561
Rnf19b	0.717701148	1.00E+00	2E-06	-0.011182481	0.013405105	-0.020413579
Spink10	0.001705032	1.34E-10	1E-06	-0.014680318	-0.03305601	-0.020443631
Ap4s1	1	1.00E+00	1E-05	-0.002451119	-0.015746735	-0.020447653
Yaf2	1	2.35E-10	0.4036	-0.004880458	-0.049421055	-0.020461929
Gm45669	1	1.67E-06	4E-36	-0.003298937	-0.015307741	-0.020471396
Nfyb	0.003558817	1.00E+00	0.0001	-0.015702941	-0.013698075	-0.020479216
Lrrc42	1	1.07E-05	1E-08	-0.002231576	-0.023722009	-0.020482403
Top1	5.13846E-35	1.00E+00	1	0.075514095	0.017953658	-0.020485083
Cxxc5	1	5.07E-18	0.2214	-0.013474462	-0.068906991	-0.020512781
Prxl2a	3.0938E-12	3.24E-09	5E-15	-0.015833018	-0.02371016	-0.020519602
Trp53bp1	0.013112022	2.57E-22	0.0043	-0.016482601	-0.058342111	-0.020534889
Iqcb1	1	2.18E-10	6E-05	-0.008871595	-0.037471295	-0.020539392
Cfap77	9.29593E-11	2.05E-13	4E-28	-0.011909091	-0.021762568	-0.020552546
Gmcl1	1	3.66E-01	0.0007	0.007046838	-0.020107141	-0.020570497
Tsc22d1	4.4589E-07	1.00E+00	4E-07	-0.016666067	0.024026451	-0.02057053
Lrp3	1	5.91E-05	4E-21	-0.003492107	-0.017250271	-0.020617038
Gm39469	1.42433E-06	5.18E-14	0.0218	-0.022226961	-0.047493395	-0.020630874
Pgpep1	2.13195E-07	1.00E+00	8E-08	-0.016956811	-0.01185663	-0.020657374
Fam53c	0.256003122	3.86E-01	1E-08	-0.010667715	-0.016133367	-0.020679909
Cspg5	2.40406E-17	2.74E-15	1E-45	-0.011688115	-0.019492213	-0.020681668
Mapkap1	9.82847E-07	1.00E+00	1	-0.040947324	0.022025595	-0.020716265
Zscan26	1	7.30E-55	1	0.014917975	-0.12644256	-0.020759192
Lsm12	2.61361E-12	3.08E-05	2E-06	-0.022087711	-0.027127097	-0.020762036
Pcyt1a	0.047536136	4.97E-02	0.4224	-0.020309005	-0.0262594	-0.02077687
Actn3	3.46302E-16	1.00E+00	1E-21	-0.015341037	-0.010993546	-0.02080962
Mtr	1	7.37E-09	0.0075	-0.011822866	-0.037911547	-0.020835444
Gm44696	0.002210035	7.03E-10	3E-18	-0.009996756	-0.023437746	-0.020859244
Glyat	8.28971E-19	2.50E-02	1	-0.053152139	-0.033242909	-0.020860444
1700037F0	3.20208E-05	9.87E-04	7E-09	-0.014643953	-0.021650041	-0.02086307
Laptm4b	1	1.00E+00	0.0002	-0.005749432	-0.016886005	-0.020886172
N4bp2os	1	3.70E-03	3E-27	0.000730889	-0.013950309	-0.020898896
Fnta	1	1.00E+00	5E-07	-0.001481356	-0.014322411	-0.020902346
Prox2	6.43482E-05	1.00E+00	4E-11	-0.012940672	-0.012399209	-0.020933402
Fiz1	1	3.70E-18	3E-07	-0.009352177	-0.041469627	-0.020934042
Alad	1	2.36E-05	0.0001	0.001448472	-0.028051601	-0.020934765
Pbx2	1	5.77E-06	2E-17	-0.00441004	-0.01906521	-0.020941221
F2r	1	2.45E-08	5E-29	0.006857487	-0.018420605	-0.021017791
Kctd20	1	3.37E-04	0.0022	-0.012690579	-0.029199362	-0.021044231
Jup	1	7.16E-03	6E-08	-0.006072869	-0.019963235	-0.021057608
Prkra	0.057121381	3.89E-09	2E-11	-0.010323538	-0.026088793	-0.021060905

Zfp972	8.68939E-05	1.06E-01	2E-05	-0.017554854	-0.021357906	-0.021074356
Parg	1	7.67E-08	0.2509	0.007363837	-0.045584214	-0.021095161
As3mt	2.07576E-60	2.16E-25	2E-05	0.056159016	-0.050201538	-0.021108352
Zfp977	1	3.25E-19	2E-17	-0.00315013	-0.031389451	-0.021126734
Sh3bp2	2.39107E-23	1.00E+00	0.0037	-0.036768564	0.015127623	-0.021170463
Kdelr2	1	1.61E-12	0.0022	0.008739614	-0.046801133	-0.021171754
Acly	2.47925E-07	2.35E-41	0.0002	0.034353157	-0.085488544	-0.021191784
2210039B0	1	2.57E-12	2E-29	0.000827655	-0.02164519	-0.021216427
A930005H1	1	4.41E-10	7E-09	-0.003973207	-0.03002283	-0.021217483
Arhgef40	0.707400735	2.87E-07	6E-16	-0.008261474	-0.021608538	-0.021270996
Whrn	1	1.05E-17	5E-46	-0.001246394	-0.020803917	-0.021301099
Mindy2	1.1587E-22	1.00E+00	0.0615	0.053044633	0.02058024	-0.021313964
Cfap36	1	2.15E-05	5E-13	-5.10257E-05	-0.020966294	-0.021339507
Tuba4a	1	2.58E-07	2E-05	-0.001018384	-0.030435017	-0.021357989
Smap2	0.619824097	8.00E-08	0.1937	0.021055143	-0.041261726	-0.021360784
Pip4k2b	0.000943606	6.56E-07	0.0001	0.018481159	-0.031333796	-0.021367314
AA986860	1	2.36E-06	0.0006	-0.011052065	-0.032566796	-0.021400068
Lmln	5.00481E-14	1.00E+00	2E-07	-0.021868773	-0.015747467	-0.02140662
Tap1	1	1.00E+00	8E-22	-0.00453118	-0.004784593	-0.021427025
Hsf2	3.93753E-05	5.88E-49	0.1922	0.026998635	-0.101316593	-0.021436045
Ctsc	0.99365398	1.00E+00	4E-08	0.006525679	0.005137579	-0.021436986
Asap2	3.84865E-79	1.00E+00	0.4738	-0.111057457	0.00240816	-0.021448915
Tsacc	1	1.90E-02	5E-06	0.000327228	-0.022071298	-0.021460723
Nsun7	1.13229E-10	3.13E-14	2E-34	-0.011377821	-0.020910252	-0.021481766
Mob4	0.000890702	1.00E+00	0.0073	-0.019322639	-0.010370901	-0.021491052
Fam76a	1	1.00E+00	8E-07	-0.005728914	-0.002202881	-0.021544085
Naa60	1	7.96E-04	0.0058	0.007707282	-0.029643343	-0.021544789
Phf7	1	4.09E-06	7E-14	-0.001150086	-0.021759783	-0.021557669
Mms19	0.402485884	1.13E-07	0.3013	-0.017003551	-0.039233411	-0.021602325
1700113B0	1	1.36E-05	2E-34	0.003257108	-0.015418708	-0.02161289
Fam71d	1.48202E-17	3.64E-11	4E-20	-0.016907983	-0.024569964	-0.021657909
Casp9	1	1.02E-03	2E-15	-0.003656556	-0.01739457	-0.021679087
2510002D2	1	1.46E-06	2E-05	-0.007247782	-0.032363456	-0.021696446
Prxl2c	1	3.54E-08	0.383	0.018022807	-0.047269081	-0.02171384
Nxt2	1	2.31E-07	9E-28	-0.0051689	-0.017860975	-0.021718228
Syap1	1	1.90E-30	0.1309	0.004314861	-0.080065335	-0.021719379
Pgm2l1	1	1.00E+00	2E-30	0.000405415	-0.005828904	-0.021730627
Zfp276	1	5.76E-15	0.0348	0.006875777	-0.0542904	-0.021751033
Mpp4	0.455162778	1.09E-27	2E-06	-0.011862549	-0.054936113	-0.021758145
Cep97	1	3.30E-05	4E-10	0.007462124	-0.022989469	-0.021759322
Mthfsl	1	3.15E-01	4E-05	0.01089453	-0.019312587	-0.021797676
Porcn	1	7.29E-14	7E-38	0.002421462	-0.021175753	-0.021815053
Gpr137c	0.061582983	4.41E-12	4E-30	-0.007688741	-0.021117215	-0.021821509
Med28	1	8.81E-10	4E-13	-0.004523118	-0.02667788	-0.021826123
Xpnpep3	1	1.35E-22	0.472	-0.002484112	-0.077901011	-0.021841716
Ifi27	1	2.48E-06	3E-19	-0.000450106	-0.018788793	-0.021972696
C1qtnf9	1	3.07E-05	8E-31	-0.006159728	-0.01594658	-0.022081241

Ccl27a	0.016987656	1.89E-16	3E-31	-0.007713838	-0.024257623	-0.022081308
Usf2	1.53771E-05	6.36E-03	0.0148	0.026792292	-0.029509786	-0.022081414
Gm28182	1.54209E-72	7.39E-18	1E-49	-0.020337697	-0.021581661	-0.022126681
4921524J1	1	3.80E-12	7E-05	-0.005289502	-0.040498299	-0.022156889
Serpina1a	5.05662E-08	2.17E-142	0.0008	-0.035630177	-0.219401288	-0.022172085
Dennd6a	1	1.00E+00	0.0308	-0.013404239	-0.011176577	-0.022178242
Fam228a	1	2.18E-17	2E-23	-0.004178034	-0.027595164	-0.022203252
Ppp2r5d	1	4.00E-08	5E-13	-0.005744867	-0.02504481	-0.022213915
Oasl1	1	1.00E+00	4E-28	-0.006052729	0.001712232	-0.022215792
Map2k7	1	1.15E-27	0.001	0.003161757	-0.065697556	-0.022241645
Gm17753	1	1.90E-03	0.0004	-0.014334655	-0.028070583	-0.022256191
Egln3	1	1.00E+00	1E-13	-0.00364299	-0.003482537	-0.022273665
Snta1	1	1.00E+00	0.0037	0.000164734	-0.022101618	-0.022287542
4932412D2	4.34403E-31	2.58E-11	2E-24	-0.019922444	-0.023182161	-0.022315008
Tmem94	1	2.12E-10	2E-09	-0.004635788	-0.031310289	-0.022369675
Kremen1	9.0758E-07	1.00E+00	5E-07	-0.017785215	-0.012484584	-0.0223783
Nsun4	1	2.33E-11	5E-10	-0.003688788	-0.032655561	-0.022382754
Cwc25	1	4.11E-08	2E-07	-0.006479825	-0.031690684	-0.022418
Usp54	1	1.14E-02	5E-09	-0.006368423	-0.020402204	-0.022447192
Ptges3	0.283190157	1.90E-03	0.272	-0.018796087	-0.032694371	-0.022449821
Jag1	0.039946064	2.88E-01	1E-24	-0.008388236	-0.011696476	-0.022498081
4930408O1	0.002461001	2.97E-06	3E-30	-0.008437438	-0.017315003	-0.022537726
St7l	0.001630894	1.00E+00	1	0.027434196	0.017480634	-0.022561195
Kdm1b	1	8.43E-07	1E-08	-0.010123641	-0.026896383	-0.022600681
Wasf1	1	1.39E-15	7E-23	0.001058454	-0.027935657	-0.022647617
Pou2f1	1.84811E-13	1.00E+00	1	-0.042299537	-0.012898231	-0.022742798
Arl3	1	2.05E-09	3E-08	0.007376462	-0.033885633	-0.022745258
Tent5c	6.97438E-06	8.27E-11	7E-25	-0.011269864	-0.022373457	-0.022760166
Zfp281	1	1.56E-19	1E-06	0.006607458	-0.046954683	-0.022761227
Btd	4.40277E-33	6.56E-07	0.0027	-0.049616236	-0.038005226	-0.022775296
Zfp362	1	4.41E-06	3E-14	-0.003937429	-0.021017539	-0.022870752
Gm31356	4.7421E-113	1.00E+00	3E-05	0.094113467	-3.74967E-05	-0.022876247
Mllt6	1	1.00E+00	3E-11	-0.010245101	-0.009381788	-0.022880779
Itga7	0.011884161	3.79E-04	5E-27	-0.008833656	-0.016395615	-0.022901944
Hint2	1	2.27E-10	3E-10	0.002880187	-0.031406312	-0.022946055
Mras	1.04819E-12	1.29E-04	3E-21	-0.015700688	-0.018949571	-0.022984568
Gcnt7	3.74892E-08	5.01E-01	1E-06	0.020740812	-0.020110079	-0.023068271
Neurl2	1.13319E-22	3.93E-15	1E-39	0.02043495	-0.022366651	-0.023093816
Zmym3	1	6.68E-08	6E-16	-0.001973826	-0.023874847	-0.023134512
Gas1	2.85176E-05	2.71E-18	1E-56	0.009768662	-0.020990943	-0.023171843
Rbm4b	1	6.81E-03	4E-08	-0.000565472	-0.021908835	-0.023187495
Igip	1	1.75E-06	7E-24	-0.003174065	-0.019157774	-0.023202273
5330439K0	1	1.57E-13	2E-37	-0.004289061	-0.021862496	-0.023207254
Ctso	0.004321979	9.22E-02	1E-05	-0.015622581	-0.020742152	-0.023233039
Gm13267	1	1.56E-03	2E-21	-0.006268306	-0.017147903	-0.023247409
Orm1	6.27473E-09	1.00E+00	2E-15	-0.016400498	-0.009210421	-0.023289957
Dusp10	1.65483E-20	2.83E-18	1E-15	-0.022140862	-0.035397564	-0.023301125

Stom	1	1.54E-08	6E-21	-0.006959135	-0.021937515	-0.023328445
Amot	0.000196583	2.29E-19	6E-14	-0.013050344	-0.037587905	-0.023342125
Tfcp2	1	1.52E-09	0.0192	0.005141505	-0.047347874	-0.023352712
Hmgn3	6.40741E-10	3.63E-18	3E-55	-0.01017155	-0.021801044	-0.023373115
Acy1	1	4.97E-17	5E-20	-0.004732595	-0.030635675	-0.023397929
Sacs	1	6.99E-15	4E-43	-0.000457313	-0.021271521	-0.023410026
Tmem256	1	3.48E-14	3E-18	-0.005979066	-0.027941499	-0.023417308
Ddb2	0.00342251	1.80E-12	3E-08	-0.014775077	-0.038562241	-0.023433109
Lag3	1.0757E-09	2.61E-21	3E-53	-0.010012315	-0.023510944	-0.023434055
Socs5	1	1.14E-06	5E-07	0.006050093	-0.028285935	-0.023514603
Hist1h2bc	1.14953E-07	4.28E-03	1E-08	-0.020920316	-0.022764165	-0.023525772
Fkbp14	0.026305325	5.47E-23	3E-17	0.011383763	-0.036963564	-0.023546964
Atp11a	0.056434542	7.85E-01	8E-46	0.007927979	-0.006344342	-0.023565852
Rab11a	1	1.06E-05	8E-08	-0.009186162	-0.026115298	-0.023600825
Pde1a	1	1.39E-12	5E-33	-0.003538464	-0.022412133	-0.023634392
Tead3	1	6.22E-09	5E-05	0.004360869	-0.040876962	-0.023653841
Gm46367	1	1.00E+00	6E-16	0.009435937	-0.010643649	-0.023692159
Gm45470	6.75768E-06	9.75E-24	9E-57	-0.008536462	-0.024398374	-0.023731004
Aqp8	1	1.09E-12	4E-46	-0.002838397	-0.02002914	-0.023737761
Ndfip2	1	3.03E-05	0.0044	0.016243486	-0.034433444	-0.023745831
Zmym4	2.6616E-10	2.15E-01	0.5197	-0.03791234	-0.02502127	-0.023770862
Aida	1	7.85E-04	2E-13	-0.008041748	-0.019171275	-0.02379221
Cfap61	1.18712E-31	5.46E-19	1E-24	-0.021479582	-0.02988509	-0.023811425
Dazap2	1	5.34E-02	2E-20	0.000209925	-0.014320744	-0.023842053
Ints7	1	1.20E-12	0.0015	-0.002223823	-0.047341971	-0.023867991
Fst	1	5.97E-20	6E-43	-0.000817203	-0.025928101	-0.023892163
Nploc4	5.24615E-15	1.30E-16	1	0.050185671	-0.076798325	-0.023921646
Atp11b	1	7.98E-09	1	-0.019626163	-0.053266452	-0.02393098
Lmbr1l	1.3591E-07	1.00E+00	0.0012	-0.026272233	-0.009621402	-0.023936298
Gm20536	0.570208169	1.00E+00	1E-18	0.01026437	0.000739339	-0.023953233
Trpc4ap	1	8.03E-06	0.0337	0.020918121	-0.037019137	-0.023998284
Bicd2	1	7.92E-04	3E-05	0.004077974	-0.027544331	-0.024002516
Gm26839	1.2282E-06	7.36E-06	2E-21	-0.013037454	-0.018950212	-0.024019602
4430402I1;	0.360237234	2.21E-06	6E-26	-0.008245164	-0.019758474	-0.024089095
Snx4	1	1.25E-09	0.0008	0.00898013	-0.04035941	-0.024092659
Tmem123	1	2.74E-04	8E-14	-0.002786399	-0.020854067	-0.024110572
Rnf41	1	1.64E-03	3E-10	-0.001309397	-0.020183286	-0.024135526
Tpd52l2	1	1.83E-05	3E-09	-0.005904747	-0.025982745	-0.024156038
Spon2	1	8.38E-18	1E-48	-0.001295143	-0.022845015	-0.024202545
Cpsf6	1	5.97E-09	0.0727	-0.01166229	-0.049315086	-0.024211329
Olfr323	4.76995E-05	1.00E+00	2E-35	-0.009750554	-0.008548975	-0.024277649
Gpatch2l	1	6.44E-01	0.0004	0.016550665	-0.020135977	-0.024305033
Cyp7a1	2.19983E-37	8.53E-30	5E-28	-0.047735828	-0.061020655	-0.024353664
Syt3	1	1.20E-23	2E-58	-0.003199825	-0.024593697	-0.024369429
Gm19522	1	5.06E-02	4E-31	-0.005448022	-0.012762316	-0.024372337
Parp12	0.020683111	8.88E-01	2E-10	-0.013300122	-0.009969745	-0.024381319
Hint3	1	2.99E-14	2E-10	0.001704775	-0.037647446	-0.024402486

Il13ra1	0.0003652	1.00E+00	2E-10	-0.015010753	-0.008166822	-0.024409559
Mme	3.39843E-06	3.49E-11	4E-34	-0.010880193	-0.021957212	-0.024431164
Dusp12	1	1.30E-07	2E-05	0.001411073	-0.036147451	-0.02450555
Pskh1	0.002881474	1.33E-06	0.0011	-0.019206036	-0.040518685	-0.024518295
Pex16	1	4.74E-19	5E-06	-0.005096893	-0.053861211	-0.024562572
Baiap2l1	1.3574E-162	2.34E-45	0.7236	0.188577207	-0.144255086	-0.024589582
B3gnt2	1.27545E-20	3.32E-02	0.0418	-0.045322364	-0.025965514	-0.02461752
Gm43569	9.87339E-09	1.26E-18	4E-40	-0.011233476	-0.026021847	-0.024656948
Ddit3	0.073609914	7.97E-14	5E-19	-0.010229202	-0.030816367	-0.024741172
Gm21917	2.26314E-09	6.42E-20	5E-46	-0.01106125	-0.025648332	-0.024750183
Ppm1a	1	1.20E-12	0.0461	-0.012167176	-0.056282685	-0.024761648
Ddx3x	1.01653E-18	8.74E-13	0.0033	-0.043549972	-0.055832505	-0.024794081
Mapkapk3	2.60858E-46	1.00E+00	1E-22	-0.02742584	0.017233639	-0.024799695
Bloc1s5	0.000303097	1.47E-10	3E-10	-0.014688925	-0.03418178	-0.024806877
Fam114a2	1	1.15E-11	0.0191	0.001280261	-0.051455969	-0.024852734
Gm16541	1	1.00E+00	4E-17	0.005418399	-0.012382627	-0.024872383
Wdr4	1	2.34E-23	7E-05	0.003026263	-0.061792928	-0.024875106
Zfp579	1	6.25E-15	1E-35	-0.001563449	-0.023484918	-0.024966542
Trmt9b	3.70946E-11	3.53E-02	0.7796	-0.037589064	-0.033073415	-0.024984661
Cib3	4.07952E-84	1.23E-16	9E-44	0.049400876	-0.024359027	-0.025002632
Syncrip	1	1.00E+00	0.0181	0.003750085	-0.0022753	-0.025089399
Ifnar1	0.086336319	1.26E-02	2E-08	-0.013857592	-0.019757725	-0.025138748
Ppp3cb	0.074454089	1.00E+00	0.0056	-0.017766638	-0.006622226	-0.025142661
Tent5a	1	1.00E+00	6E-32	-0.003531612	-0.00489812	-0.025193947
Selenoo	1	6.33E-24	0.002	-0.013540375	-0.070926121	-0.025260398
Map4k2	1	5.55E-10	3E-21	0.002086496	-0.024101469	-0.025285286
Heg1	1	2.61E-03	2E-29	-0.000513626	-0.009906596	-0.025311511
Oaz1	1	3.33E-07	1E-07	0.002968361	-0.031666336	-0.025339313
Gm15343	0.002831097	9.54E-15	6E-45	-0.008360288	-0.023166824	-0.025392371
Slc22a14	0.535865646	1.00E+00	1E-06	-0.013151322	0.006413671	-0.025443026
Gm16193	1	1.00E+00	3E-07	-0.001158589	-0.004773786	-0.025472502
Nim1k	1.22554E-06	9.35E-33	8E-08	-0.020603708	-0.063250746	-0.025476439
Pcnt	1	4.19E-02	8E-06	0.000790878	-0.022225962	-0.025496369
Pkig	0.034214533	1.28E-02	0.0007	-0.018961742	-0.026446962	-0.025512579
Megf9	7.35886E-29	4.64E-34	1	0.068966537	-0.138253736	-0.025517732
Nr4a1	9.98254E-57	8.48E-07	7E-55	-0.020764292	-0.014315228	-0.02552422
Mpnd	1	2.21E-09	9E-18	-0.007735117	-0.027592503	-0.025532601
Zfp809	1.182E-34	3.43E-08	0.2639	-0.068153971	-0.054600049	-0.025576108
Tmem175	1	1.00E+00	3E-06	-0.008317067	-0.009407625	-0.025576818
Efcab2	1	1.49E-02	3E-22	-0.007703935	-0.015904127	-0.025621581
Ing1	1	5.17E-16	2E-23	-0.002249174	-0.030687826	-0.025690372
Kcnk10	3.98683E-37	1.12E-19	8E-53	-0.018290676	-0.025325874	-0.025744102
Brms1l	1	3.39E-09	4E-10	0.000952578	-0.033589699	-0.025783775
Abcd1	3.90078E-05	1.16E-01	5E-16	-0.014610683	-0.01647907	-0.0257842
Trim34a	0.528434092	3.67E-01	8E-30	-0.007894221	-0.009542712	-0.025789224
Mbl2	4.4803E-53	1.10E-23	4E-07	-0.050742647	-0.057823775	-0.025805932
Nod1	0.08917169	1.00E+00	2E-16	-0.011145527	-0.002617707	-0.025841588

Cdc42ep1	5.19106E-08	8.10E-07	2E-13	-0.01718598	-0.028118052	-0.025859958
Sptssa	1	4.34E-05	2E-10	-0.008639131	-0.026009941	-0.02590687
Slc6a16	1	7.58E-25	6E-62	-0.00452053	-0.026144051	-0.025957744
Zdhhc17	1	1.10E-03	9E-06	-0.00860852	-0.028559731	-0.026004714
Rab40c	0.000193893	4.00E-03	0.0148	0.030944581	-0.032550603	-0.026024349
Lect2	0.000907556	9.42E-25	4E-41	-0.008899447	-0.030098076	-0.026036451
Ankrd46	1	7.45E-01	5E-12	0.005900288	-0.01686518	-0.026050787
Ddo	0.011041418	1.00E+00	0.0001	-0.017727938	0.006491602	-0.026089284
Dffa	1	1.37E-13	1E-07	-0.008724145	-0.0461123	-0.02610688
Gm34280	1	7.10E-18	6E-51	-0.005627933	-0.024750562	-0.026189585
Pde6d	0.805486091	3.07E-05	8E-13	-0.010536843	-0.025048433	-0.026204191
Ube2z	1	1.00E+00	1E-14	-0.008841092	-0.013683357	-0.026214094
Ppm1d	1	1.21E-06	1E-15	-0.006891302	-0.025406102	-0.026218818
Pip5k1c	1	1.00E+00	7E-13	-0.010461356	-0.005665217	-0.026279921
Casp8	0.020928928	1.00E+00	5E-05	-0.017453021	0.002619819	-0.026346362
Spen	0.530165634	6.67E-18	0.0266	0.023312612	-0.072171516	-0.026376539
Zc3h12d	1.52048E-18	4.33E-08	9E-24	-0.02006708	-0.023916512	-0.026402112
Hmgn1	1.39314E-05	9.27E-09	1E-16	-0.014951885	-0.028187091	-0.026415501
Sin3b	1	3.30E-01	1E-11	-0.008365397	-0.016453552	-0.026498842
Shmt2	1	3.27E-28	3E-08	0.001273746	-0.060891787	-0.026519664
Nudt6	1	4.12E-03	2E-18	-0.005955021	-0.018828843	-0.026524809
Gm47167	1	1.00E+00	6E-12	0.008020545	-0.003743661	-0.02657761
Ndrg1	3.5114E-15	1.00E+00	7E-25	-0.022221943	0.009693196	-0.026582058
Tnrc6a	1	6.64E-06	0.0013	-0.005773769	0.084562077	-0.026652614
Slc12a2	1	7.35E-04	3E-26	-0.005620134	-0.017179519	-0.026723967
AY074887	2.2049E-14	6.03E-16	3E-48	-0.013524953	-0.024569205	-0.026737471
Isca1	0.003676651	2.41E-10	1E-08	-0.016097744	-0.039226335	-0.026739861
Gsdmd	1	2.39E-09	5E-30	-0.003897484	-0.020882183	-0.026755367
Map7	0.001068925	1.47E-17	0.0668	-0.040324986	-0.081890038	-0.026767021
Znrf2	1	2.86E-07	0.0003	0.012004597	-0.038045127	-0.026812074
Kat7	1	3.36E-11	5E-05	0.005444575	-0.045546219	-0.026814508
Lrrc3	1	1.58E-21	3E-36	-0.00403898	-0.030617366	-0.026843115
Lhpp	8.58043E-09	9.82E-33	2E-08	-0.056002604	-0.124670732	-0.026845031
Tmem220	1	1.38E-14	2E-20	0.006882807	-0.032438698	-0.026876778
Txnrd3	0.864766991	1.91E-21	3E-51	-0.006381836	-0.026956465	-0.026901404
Cep70	3.59456E-31	1.00E+00	1E-11	-0.034568619	0.009772026	-0.026910523
Prpf6	1.65475E-23	4.39E-13	0.0453	-0.055145119	-0.065889877	-0.026915448
Tmem88b	1	8.09E-11	3E-43	-0.005672363	-0.022058844	-0.026920374
Patz1	5.29835E-07	1.15E-19	4E-50	-0.010619036	-0.025474854	-0.026980643
Slc16a4	1	1.92E-03	2E-33	-0.006354629	-0.016851006	-0.027021486
Zfp869	1	1.79E-09	9E-08	0.006623977	-0.03880528	-0.027086239
C730034F0	1	3.65E-14	1E-28	0.005203881	-0.028621173	-0.027098481
Rasa3	1.16537E-09	1.00E+00	5E-25	-0.018551372	0.056019963	-0.027101588
Khdrbs1	1	1.00E+00	5E-06	0.00032055	-0.011023723	-0.027151929
Chic1	3.8607E-13	1.77E-19	3E-57	-0.012419315	-0.025438656	-0.027226374
Ddx46	1.50917E-24	4.71E-23	0.0449	0.061867822	-0.084301734	-0.027277564
Sclt1	1	4.26E-04	0.0004	-0.003611774	-0.034658014	-0.027304064

Lta4h	1	8.44E-07	9E-11	0.001039517	-0.030045256	-0.027319771
Rcc1	1.35884E-06	1.88E-12	2E-16	-0.016485237	-0.031490704	-0.027340414
Platr22	3.16007E-46	2.44E-53	0.0001	-0.065331435	-0.1173082	-0.027351971
Stt3b	1	1.76E-19	0.0006	0.017837302	-0.067812707	-0.0273568
Edc3	0.000374222	5.07E-10	0.0012	-0.023695645	-0.050160366	-0.027388443
Cnksr3	1	3.15E-34	0.0009	0.015448382	-0.082897728	-0.027398879
Tgif1	1	6.40E-02	3E-16	-0.000167457	-0.017205826	-0.027400086
Hic2	1	1.39E-17	3E-21	-0.003394169	-0.035379437	-0.027426643
Irgm2	1	9.99E-09	7E-14	-0.004121323	-0.029100955	-0.027452095
Polr3g	1	4.09E-10	6E-14	0.008140394	-0.033011514	-0.02752332
Zfp974	0.102736203	8.30E-17	2E-17	-0.010789773	-0.037630624	-0.027528107
Snx32	1	1.87E-22	8E-26	-0.004535746	-0.033683275	-0.027540657
Rexo5	1	6.10E-08	4E-14	-0.005936178	-0.029739372	-0.027547608
Uimc1	1	1.21E-10	0.0483	0.01780421	-0.058000585	-0.027553503
Ing5	1	1.17E-07	2E-27	0.002838341	-0.023165609	-0.027580733
Polg2	1	2.74E-07	2E-14	-0.003002657	-0.029128505	-0.027598665
Sgce	0.000383917	8.92E-20	1E-44	-0.00979983	-0.027042151	-0.027607606
Gm16675	1.09191E-08	1.04E-05	1E-31	-0.014028444	-0.019309234	-0.027616756
Tecr	0.00038432	1.28E-39	2E-05	0.024818712	-0.086639652	-0.027622325
Nrp2	1.45045E-28	1.00E+00	8E-41	0.026406188	0.02252104	-0.02763726
Ptpmt1	1	1.68E-06	8E-09	-0.010702485	-0.033023966	-0.027645771
Klhdc7a	4.22325E-21	1.07E-22	4E-63	-0.014849309	-0.026648003	-0.027662133
Arid3b	1	1.12E-11	3E-14	-0.001430601	-0.032727942	-0.027766291
1810058I24	1	7.48E-08	2E-24	-0.003551741	-0.023314453	-0.027771819
Tmem59	1	2.81E-03	7E-09	-0.001162083	-0.025472481	-0.02786022
Lgals4	1.63406E-19	3.11E-36	0.0001	0.052115014	-0.091320802	-0.027873285
Abl2	0.595678463	7.95E-04	0.0094	-0.020543423	-0.027116361	-0.027906737
Lrg1	1	1.00E+00	1E-17	-0.00891549	0.010840304	-0.027922166
Ccdc107	0.383157325	6.84E-23	9E-16	0.012569421	-0.04471546	-0.027940096
Dlg3	1	8.45E-33	2E-10	-0.00127962	-0.062211006	-0.027946772
Tlcd1	0.003925311	4.54E-28	2E-10	0.01728607	-0.056870768	-0.027956044
Swi5	1	6.30E-03	6E-13	-0.005323203	-0.023054359	-0.027964365
Pllp	0.008037048	3.38E-04	3E-16	0.014450714	-0.023204596	-0.027977778
Sucnr1	1	4.63E-27	2E-70	0.000149616	-0.027751251	-0.027991782
Gt(ROSA)2(	3.16773E-06	1.86E-04	2E-15	-0.016372321	-0.021344162	-0.028038828
Sult1b1	1.52044E-14	3.37E-25	7E-21	-0.019423084	-0.042584156	-0.028049322
Sumo3	1	3.08E-07	4E-16	-0.001248071	-0.028084254	-0.02805152
D830024N(	1	2.19E-08	3E-23	0.006090752	-0.026112583	-0.028120998
Gm16291	1	1.00E+00	2E-30	-0.003999633	-0.009578446	-0.028138327
Ociad2	1	2.05E-06	1E-28	-0.007488436	-0.021814612	-0.028155356
Hsdl2	1	1.26E-09	0.0017	0.008716277	-0.056245726	-0.028157727
Klf7	1	1.00E+00	5E-29	0.002138917	0.005196938	-0.028173813
Purg	7.83569E-14	1.00E+00	5E-09	-0.029259472	-0.018679453	-0.028218566
Osbpl11	1	2.48E-14	0.0047	-0.00276889	-0.062470817	-0.028221367
D830025C(	0.128381855	4.49E-12	3E-34	-0.008890682	-0.025984116	-0.028228168
Mfsd4a	1	1.00E+00	1E-17	-0.000285878	-0.012270499	-0.02824473
Ensa	1	3.03E-07	5E-12	-0.005577807	-0.029572185	-0.028255952

3110056K0	1	4.80E-01	6E-10	-0.009810306	-0.01836903	-0.02825867
Gprasp1	1	2.71E-30	0.0001	-0.013662617	-0.07976608	-0.028260742
Gm16576	6.39983E-06	1.10E-10	6E-39	-0.010984525	-0.023575817	-0.028295527
Gm15640	1	7.31E-09	3E-49	0.007826692	-0.020189246	-0.028328239
Hyal2	0.431875697	1.16E-16	2E-25	-0.009452281	-0.032848943	-0.028361741
Gm15879	3.19725E-09	3.73E-09	9E-22	-0.01625641	-0.02801609	-0.028385218
Map7d1	0.000165045	1.00E+00	5E-19	-0.014123928	-0.006156334	-0.028399956
Retreg2	0.027488727	2.01E-19	1E-12	-0.013492014	-0.046254775	-0.028418037
Pcsk4	1	1.85E-26	6E-20	0.008337811	-0.044869786	-0.028430848
Pfkl	1	1.14E-19	5E-34	-0.000826469	-0.031650372	-0.028447175
Pts	1	3.13E-08	3E-13	-0.005492303	-0.031004901	-0.028481772
Siae	3.2424E-23	2.25E-09	2E-16	-0.027546675	-0.030954297	-0.02853035
Acap2	0.013175204	6.80E-06	0.0001	0.024675334	-0.027180678	-0.028556387
Gm26759	1	7.44E-01	4E-33	-0.001691119	-0.012269863	-0.028575794
Suclg1	1	3.74E-03	9E-06	0.002757264	-0.028893774	-0.028604778
Gm10125	1	3.23E-18	1E-54	-0.004544198	-0.024957368	-0.028688759
Gm42984	0.863505938	3.99E-04	1E-27	-0.008592738	-0.019078545	-0.028704393
Tle3	1.3673E-31	1.00E+00	6E-20	-0.029666238	-0.004492294	-0.028717436
Gm12216	6.89842E-15	1.53E-02	2E-28	-0.017945442	-0.014196757	-0.028739693
Zrsr2	1	6.31E-12	5E-11	0.010584773	-0.038345154	-0.02876676
Gbp10	5.45142E-38	1.72E-19	5E-53	-0.020337217	-0.028183656	-0.028805951
Foxa1	1	2.91E-24	3E-35	-0.007398812	-0.034876196	-0.028816069
Slc25a34	1	5.99E-13	1E-18	-0.004918693	-0.034157925	-0.028821159
Dnajb4	5.29946E-14	8.56E-13	3E-08	-0.030093346	-0.046388655	-0.028827743
Blcap	1	2.23E-15	9E-07	-0.010150923	-0.04976929	-0.028875111
Mier3	1	1.64E-06	9E-09	-0.011368266	-0.034617599	-0.028999438
Gm11967	1	2.58E-22	2E-60	-0.005274855	-0.027924655	-0.029049457
Gtf2ird2	1	4.83E-12	9E-13	0.003452355	-0.037476091	-0.029063177
Klhl13	8.07691E-08	4.79E-19	8E-39	-0.012623789	-0.030204356	-0.029105435
Cldn2	4.18108E-68	7.29E-21	3E-45	-0.027543519	-0.030056907	-0.029105908
Cyp2d40	1	2.70E-18	8E-59	-0.005221659	-0.025756167	-0.029115823
Midn	5.13704E-50	9.37E-03	4E-14	-0.042873991	-0.022109297	-0.029154459
Mapre1	1	2.90E-13	2E-14	-0.0095174	-0.036611349	-0.029258948
Ywhag	0.767060951	1.00E+00	7E-08	-0.01610287	0.009283762	-0.029309284
Amotl2	8.55474E-06	2.61E-11	3E-13	0.020310113	-0.037830108	-0.029344942
Zbtb4	4.85509E-11	2.48E-08	6E-24	-0.01757567	-0.02256223	-0.029389305
E2f8	2.06336E-11	5.48E-23	2E-59	-0.012909298	-0.028144597	-0.029416339
Gabarapl2	1	3.68E-15	1E-18	-0.003197727	-0.036516106	-0.029468168
Ces1g	2.70012E-33	4.26E-01	4E-12	-0.03893905	-0.017540293	-0.029470876
44448	2.98832E-29	5.68E-30	0.0136	-0.065083907	-0.105066784	-0.029577733
Rab9	0.078064446	2.17E-15	7E-06	-0.017885663	-0.056200933	-0.029590387
Slc13a2	0.01385148	1.51E-27	6E-70	-0.007339218	-0.029596349	-0.029623461
Ctsl	1	3.54E-07	2E-07	0.009369736	-0.033034808	-0.029641637
Taf13	1.45999E-06	9.09E-07	9E-20	-0.016133437	-0.027148503	-0.029688061
Mettl24	0.000228858	1.58E-07	3E-43	-0.010635784	-0.021065159	-0.029720666
Fam199x	0.210882207	3.57E-08	6E-28	-0.009760755	-0.025343731	-0.029739385
Picalm	7.30838E-13	1.45E-34	0.0001	0.048650354	0.151469523	-0.029745064

F11	5.91075E-05	4.16E-84	0.0011	-0.035112002	-0.190638404	-0.029826347
Gm19461	1	2.52E-08	3E-46	-0.004087786	-0.021737447	-0.029826835
Gm36975	0.002547009	2.27E-01	3E-08	0.020756757	0.033924649	-0.029849981
Snx1	1	8.63E-01	3E-12	-0.003805626	-0.013501965	-0.02985849
Plekhh3	1	6.58E-20	3E-35	-0.006922767	-0.033205033	-0.029919503
Srebf1	1	7.43E-14	2E-08	-0.011050835	-0.048721437	-0.029945005
Fga	1.6288E-270	7.57E-94	7E-07	0.247825154	-0.210626872	-0.029961382
Fam207a	1	4.45E-15	2E-07	0.008626109	-0.055248497	-0.030015955
Dact2	1	1.17E-20	3E-40	0.00287415	-0.032920722	-0.030068321
Gbp6	1.45259E-30	5.56E-07	2E-35	-0.023121755	-0.022459015	-0.030111758
Sos1	0.007097792	1.00E+00	0.0131	0.027949122	-0.021276269	-0.030175891
Pigl	6.06159E-06	5.27E-09	1E-09	-0.020754027	-0.037428531	-0.030219193
D2hgdh	1	2.04E-12	5E-14	0.002579877	-0.039213907	-0.030223602
Mindy4	4.4311E-05	1.78E-17	2E-26	-0.01311581	-0.035561165	-0.030225464
S1pr1	1	9.49E-10	4E-30	-0.008410099	-0.025739962	-0.030234289
Mgmt	5.416E-118	1.78E-36	0.0288	-0.161887073	-0.138630276	-0.030329076
Snx18	0.000723377	1.00E+00	5E-10	0.020595283	-0.01550551	-0.030335785
Gm20300	1	4.60E-22	6E-37	-0.006948526	-0.034237764	-0.030384544
Rab2b	1	8.24E-08	8E-14	-0.002025888	-0.030825882	-0.030409093
Ppp1r2	1	1.00E+00	4E-13	-0.006902489	-0.008175952	-0.03046919
Sqor	8.62206E-63	1.29E-05	3E-07	-0.062384822	-0.035372056	-0.030470327
Brdt	7.40376E-21	1.57E-21	9E-25	-0.023437129	-0.040697648	-0.030481409
Wdfy3	0.008736769	8.73E-01	0.0001	-0.03510566	0.080348385	-0.030595113
Bcl7c	5.18626E-38	3.12E-46	8E-16	0.044392526	-0.068803988	-0.030647566
Plppr1	1	3.11E-01	1E-25	0.009913203	-0.016223132	-0.030678941
Psme1	0.864234899	3.70E-05	1E-26	-0.009567394	-0.020770256	-0.030711703
A230072C0	1	2.29E-16	1E-16	-0.009075462	-0.041758554	-0.030753412
Btbd19	1.85411E-78	1.64E-13	6E-38	-0.033709857	-0.028779453	-0.030768272
Fas	4.70852E-18	1.90E-06	8E-17	-0.02719262	-0.025186406	-0.030781141
BC024978	1	1.24E-18	1E-23	-0.005818866	-0.039060723	-0.030801538
9130016M	1	1.67E-18	3E-06	0.007635016	-0.061198279	-0.030803562
Nuak1	9.9442E-06	2.68E-13	2E-39	-0.012087392	-0.026084746	-0.030820652
Tpmt	4.52022E-36	5.01E-31	5E-09	-0.045923976	-0.072763206	-0.0308521
Zfp110	1	7.90E-20	2E-07	0.010445372	-0.059438089	-0.030867531
Gm39363	4.10841E-32	3.56E-20	5E-24	-0.029253509	-0.040717228	-0.030869285
Pik3ca	1	1.00E+00	0.0097	0.019437215	-0.001678557	-0.03088388
Zfp629	4.54847E-07	2.01E-18	1E-27	-0.014753783	-0.035504372	-0.0309429
Hnrnpr	1	1.30E-12	1E-04	-0.001349941	-0.058590827	-0.030948284
Rnf170	1	3.23E-12	7E-17	-0.010385088	-0.037643574	-0.030952431
Triobp	0.416060153	3.54E-09	4E-27	-0.009945562	-0.026909293	-0.030968459
Unc13b	1	2.11E-20	3E-24	0.009250042	-0.042297249	-0.03100843
Ssh2	1.90683E-14	1.00E+00	4E-05	-0.047604319	0.054307621	-0.031057183
Aqp11	6.49847E-06	1.00E+00	5E-22	-0.01570708	-0.006231819	-0.031099041
Slc26a1	1	3.11E-04	8E-14	-0.008298639	-0.026220763	-0.031124332
H2-Q6	1	1.50E-12	3E-35	-0.004679265	-0.026313811	-0.031176752
Gm8251	1	4.95E-11	2E-23	-0.005711112	-0.031730467	-0.031178779
Rcbtb2	8.7793E-05	1.00E+00	1E-11	-0.01982479	0.011991869	-0.031180621

P2rx4	1	1.00E+00	4E-07	-0.015226932	0.034911132	-0.031180872
Snx21	1.9744E-05	8.24E-09	1E-19	-0.016255951	-0.031100232	-0.031190943
Gata6	0.887485355	6.58E-16	9E-20	0.0125111	-0.038179957	-0.031216349
Pex5	1	1.09E-10	7E-08	-0.00159378	-0.046629198	-0.031219904
Prss53	1	1.01E-31	2E-29	0.004086398	-0.045225888	-0.031261332
Spg20	4.15096E-31	5.53E-14	7E-13	0.044172283	-0.043386415	-0.031264519
Zfp354a	1	9.68E-31	4E-42	0.002219748	-0.039621583	-0.031306342
Gm46218	1	3.66E-16	5E-20	-0.004107206	-0.039595679	-0.031309375
Ascc1	1	8.35E-17	5E-09	-0.004562902	-0.052598641	-0.031321698
Wdr82	1.42771E-07	1.47E-12	6E-07	-0.026268194	-0.050441084	-0.031343397
Bdnf	0.000116446	2.79E-27	5E-74	-0.009724897	-0.030356729	-0.03138809
Frs2	1	1.00E+00	0.0008	0.000376391	-0.015196877	-0.031398523
Mark2	0.279737147	1.00E+00	0.0024	0.024634979	-0.004838716	-0.03144309
Nuak2	1	3.41E-12	1E-48	-0.001438982	-0.02351511	-0.031482378
Slc12a4	1	3.63E-22	3E-49	-0.005235918	-0.031528154	-0.031487973
Klf13	0	1.00E+00	0.0044	0.213616938	-0.008253448	-0.0314898
Cd164	1	2.43E-18	4E-17	0.007838551	-0.04333817	-0.031502469
Usp28	0.501624739	7.23E-06	1E-14	-0.013431289	-0.026170405	-0.031506226
Hmox2	1.12707E-44	2.31E-08	0.0002	0.069489169	-0.046029307	-0.031537555
Kdm6b	1	2.19E-06	1E-09	-0.011214687	-0.033456165	-0.031541642
Arid4a	3.63739E-20	1.00E+00	0.0002	0.060137528	0.041003006	-0.031597332
Ptprs	1	6.53E-16	1E-61	-0.006320698	-0.024907006	-0.031598922
Lyn	1	2.44E-51	1E-08	-0.009708732	0.222071238	-0.031623566
Slc25a10	1	2.00E-24	8E-13	-0.001195771	-0.056872412	-0.031631728
Thra	2.80684E-20	1.41E-18	2E-21	-0.025739904	-0.040386345	-0.031646888
Snx2	1	1.00E+00	2E-09	0.008326217	-0.002557422	-0.031658479
Kmt2a	1	6.79E-01	0.003	-0.022599765	0.053906086	-0.031659952
Hnrnpd	1	4.49E-10	1E-06	0.002725626	-0.045597465	-0.031665719
Eif5b	4.35155E-26	6.74E-10	0.0032	0.064980971	-0.05529651	-0.031684573
Qser1	0.247941221	2.04E-05	4E-08	-0.01665201	-0.036349344	-0.031691892
Cdc42bpb	1	1.07E-01	0.0053	0.013937889	-0.040514789	-0.031703907
B4galnt3	2.50627E-07	1.48E-27	7E-63	-0.011200535	-0.032491963	-0.031710864
Dync1li1	1	1.97E-03	4E-08	-0.010074661	-0.026422298	-0.031769502
Nsd2	0.677415547	7.90E-10	6E-09	-0.01755111	-0.043047	-0.031826822
Gm44686	1	1.00E+00	1E-23	0.0041045	-0.010575182	-0.031832998
Uap1	1	2.61E-26	4E-07	-0.015897404	-0.072848136	-0.031874988
Pxmp2	6.18735E-18	1.80E-34	5E-07	-0.042009177	-0.090424193	-0.03189809
Vwa3b	0.163535823	3.90E-29	5E-24	-0.011472004	-0.048270635	-0.031900141
Slc10a2	1	1.95E-24	2E-65	-0.003289985	-0.031897482	-0.031973328
Mphosph9	1	1.04E-19	6E-16	-0.006736574	-0.049420697	-0.031992259
Cd99l2	1.52541E-12	1.17E-11	7E-11	-0.028130298	-0.042824485	-0.032003258
Clstn3	4.50108E-08	7.12E-19	4E-41	-0.01376517	-0.03222783	-0.032025681
Nudcd3	1	3.98E-01	3E-05	-0.003756152	-0.020592436	-0.032045904
Dym	6.89601E-08	1.09E-02	0.0007	-0.042579665	-0.030511898	-0.032070486
Gm26684	1.14195E-20	5.73E-19	3E-34	-0.021230141	-0.034750493	-0.032109252
Cops4	1	1.60E-08	1E-12	0.012274611	-0.034660936	-0.032133671
Gm45609	1	3.03E-18	2E-26	-0.006929731	-0.038635222	-0.03214399

Cnot8	0.09138649	4.75E-07	1E-11	-0.015135714	-0.036195625	-0.032214569
Pole4	1	3.37E-13	5E-06	0.009433582	-0.054798173	-0.032274222
Zfp36l1	6.36271E-53	4.96E-36	3E-08	-0.085913795	-0.103443016	-0.03228999
Ebp	1	2.86E-28	5E-29	0.004229958	-0.046166282	-0.032334182
Prob1	1	2.33E-32	1E-66	-0.002264074	-0.034843001	-0.032388061
Akr7a5	1	2.38E-15	1E-13	-0.00551909	-0.046625526	-0.032421022
Psmd11	1.57704E-13	1.00E+00	0.0004	0.047328699	-0.017085522	-0.032433644
Rsu1	3.64146E-09	1.00E+00	5E-09	-0.028936595	0.024259165	-0.032446604
Pdxk	9.65014E-08	5.63E-11	5E-27	-0.01668467	-0.029452131	-0.032456464
Kdm5b	3.1359E-99	1.20E-36	0.0008	-0.108667142	-0.10943649	-0.032508384
Flot2	1	7.21E-03	9E-15	-0.012575352	-0.025286682	-0.032519091
Gm4876	7.99318E-64	8.35E-14	4E-36	-0.033300638	-0.030623579	-0.032528907
Spata2l	1	1.61E-24	3E-35	-0.003104873	-0.03954051	-0.032543173
Oxsm	1.58106E-12	5.16E-12	1E-25	-0.019941067	-0.033457213	-0.032652244
H2-Q7	1	9.95E-07	3E-42	0.000422768	-0.018158437	-0.03265549
Letmd1	1	8.20E-14	5E-28	-0.001546711	-0.033781795	-0.032671522
Asgr1	9.77859E-66	7.12E-12	2E-07	-0.068836323	-0.050912013	-0.032737489
Mov10	6.9092E-62	5.44E-08	4E-12	-0.055496512	-0.035644993	-0.032737896
Nudt4	1	4.30E-32	8E-06	0.007025211	-0.092599427	-0.032759552
Fez2	1	3.52E-07	2E-21	-0.005190116	-0.026573921	-0.032807043
Bace1	1	9.72E-23	4E-08	-0.005592215	-0.069731051	-0.032817099
Got2	3.17776E-18	5.88E-36	2E-06	0.05268135	-0.099586312	-0.032833586
Map2k4	1.50504E-11	9.89E-36	0.0019	-0.04211857	-0.108242703	-0.032839756
Akap10	1	2.46E-02	2E-05	0.02026207	-0.019984617	-0.032839816
Tiprl	1	4.83E-17	1E-17	0.008737818	-0.044567746	-0.032874487
Pdpr	1	5.85E-07	6E-11	0.003665288	-0.036193107	-0.032886347
Gm15614	1	1.00E+00	4E-23	0.007951915	-0.003669573	-0.032900982
Zfp217	1.21458E-06	8.56E-04	3E-26	-0.016307009	-0.019073849	-0.032906714
Gm40040	4.41533E-23	1.77E-06	5E-28	-0.024934752	-0.025762154	-0.032931118
Tmco4	2.37239E-21	1.00E+00	1E-08	-0.039435462	0.005148345	-0.032986123
Klf11	0.68438696	2.85E-12	2E-51	-0.007917096	-0.025883566	-0.032990884
Rab28	0.007350752	3.83E-22	5E-08	-0.019774236	-0.064691569	-0.032999052
Eloa	1	1.34E-21	1E-12	-0.007428295	-0.056233085	-0.033065637
Gm13481	0.214967896	9.72E-19	7E-59	-0.010063045	-0.029899714	-0.033082513
Akap7	0.008049894	2.91E-03	3E-15	-0.014675073	-0.020892932	-0.033108298
Adar	1	1.21E-08	1E-14	-0.007162331	-0.032515341	-0.033248558
Mmp15	1	5.14E-10	5E-38	0.003720743	-0.027261601	-0.033260542
Fras1	2.28487E-05	2.33E-22	9E-60	-0.011280043	-0.032039652	-0.03330641
Tasor	1	1.00E+00	0.0028	-0.004770392	0.009077994	-0.033317761
Gm42413	1	2.66E-13	6E-53	0.004962234	-0.027184078	-0.033382857
Ppp2r5c	3.79656E-05	9.90E-07	8E-05	-0.028286666	-0.043308728	-0.033455615
Ctnnbip1	5.56477E-08	9.69E-10	2E-23	-0.017797938	-0.031682344	-0.033519594
Dcaf17	1	1.00E+00	7E-11	-0.013704096	-0.017340371	-0.033536914
Ptgds	1.51524E-05	5.91E-31	1E-80	0.011734893	-0.033087526	-0.033546903
Plekhg6	1.11961E-05	1.85E-20	2E-42	-0.012656895	-0.035245426	-0.033549637
Copz1	1	1.91E-19	1E-08	0.010750324	-0.061264531	-0.033591525
Zfyve21	1	3.09E-27	1E-29	-0.005503441	-0.04521075	-0.033784701

Csnk1d	1	7.38E-05	3E-06	0.011860414	-0.036860303	-0.033821284
Gm48383	0.757116167	5.82E-29	5E-54	0.009306406	-0.036569356	-0.033872905
Zfp207	0.00032117	6.65E-05	0.0007	-0.028952999	-0.046439963	-0.033884684
Adra1a	2.18863E-11	8.14E-19	2E-12	-0.043487385	-0.067516411	-0.033899119
Lactb2	0.000291976	2.81E-76	6E-05	0.029208342	-0.167404329	-0.033903191
Ppig	0.276093083	4.40E-11	2E-07	0.021289324	-0.043617175	-0.033903588
Eif2ak1	1	1.50E-12	2E-09	-0.010778713	-0.051772903	-0.033944501
Gm15889	0.35284758	8.85E-16	2E-62	-0.008204608	-0.027770947	-0.033945797
Zfp141	1	3.47E-13	1E-14	0.000100587	-0.043701739	-0.03399147
Kcnrg	3.40437E-13	3.68E-25	4E-76	-0.013952899	-0.030954632	-0.034009799
Cenpv	2.44639E-08	9.15E-01	8E-06	-0.030775981	-0.023669026	-0.034101711
Tjp1	1	9.34E-45	3E-07	0.002692764	-0.098993576	-0.034149418
Tnk2	1	6.83E-08	2E-15	-0.006369308	-0.036459999	-0.034268411
Phc3	1	2.56E-21	0.0001	0.003732809	-0.079531382	-0.0342856
Rab5b	1	1.74E-12	9E-14	0.006030121	-0.043948469	-0.034326002
Dmwd	1	8.44E-30	5E-42	0.000328595	-0.041417689	-0.034338866
Foxk1	1	1.71E-02	1E-08	0.014327463	-0.027175344	-0.034388253
Crlf3	0.111851102	1.00E+00	2E-19	-0.0139297	0.001910284	-0.034460776
Cyth3	6.40217E-08	1.00E+00	6E-26	-0.017929536	-0.006406126	-0.034462041
Pgpep1l	0.139704867	2.64E-31	4E-39	-0.01046241	-0.044377339	-0.034490863
Prcc	1	1.98E-13	3E-17	0.009138874	-0.042729592	-0.034615769
Odf2	1	1.63E-03	3E-11	-0.004456481	-0.023911593	-0.034678927
Btrc	2.90411E-57	3.83E-16	0.0003	-0.087783892	-0.076942561	-0.034715681
Plekhf1	1	3.55E-21	2E-78	0.006595068	-0.029401551	-0.034774447
Sycp3	0.003925202	7.28E-09	6E-57	-0.009944153	-0.023457401	-0.034829252
Tmed10	1	5.34E-18	3E-08	0.00868128	-0.05917293	-0.034835896
1700024B1	0.023419063	1.72E-28	2E-79	-0.008402065	-0.033132213	-0.034860428
Tab3	7.63851E-05	1.03E-10	2E-17	-0.017614504	-0.0395891	-0.034914781
Ctnnbl1	1	1.89E-33	5E-09	-0.004868442	-0.084751087	-0.034922753
Sec24a	9.05937E-84	1.57E-24	0.0003	0.112990247	-0.092937305	-0.034927106
Hsd17b10	1	4.42E-25	8E-43	0.004055295	-0.039615768	-0.034933375
4933408B1	1	1.48E-28	9E-47	-0.007169368	-0.040246786	-0.034943855
Ldlrad3	1.66535E-57	5.16E-05	1E-05	-0.077986746	-0.033117622	-0.034947238
Ddrgk1	1	5.43E-18	2E-18	0.004601901	-0.046066884	-0.034982411
Oaz2	3.1665E-10	1.11E-07	2E-22	-0.021303202	-0.031705206	-0.035081545
Rest	0.013529355	5.04E-24	5E-17	-0.015877732	-0.054903283	-0.035102869
Immt	1	1.82E-27	3E-07	0.013742204	-0.079186635	-0.035153103
Pigu	0.000196878	7.19E-09	5E-08	0.026310706	-0.047036905	-0.035168771
Stx7	1	3.66E-01	4E-15	0.004093853	-0.014141295	-0.035193764
Clip1	1	7.49E-05	0.0146	-0.0049552	0.056958549	-0.035201819
Tbc1d2b	1	2.54E-19	8E-10	0.017922189	-0.060257526	-0.035204888
Platr4	4.14562E-21	1.00E+00	1E-15	-0.031659513	-0.016276676	-0.035208767
Mapk6	1	1.44E-04	2E-19	-0.000726748	-0.02415375	-0.035208973
Hdac6	1	3.97E-27	1E-34	-0.006428709	-0.044740896	-0.035230083
Anxa6	1	6.97E-34	6E-13	-0.006480227	-0.076965667	-0.035250437
Fam210b	1	1.56E-19	7E-30	0.006176759	-0.040593866	-0.03527626
Crim1	0.449634406	1.64E-27	2E-20	-0.015611177	-0.05906254	-0.035279831

Wdr59	1	7.62E-16	2E-12	-0.012279211	-0.050073925	-0.0352912
Elf2	2.37173E-11	2.15E-33	3E-05	0.046993524	0.146943561	-0.035296761
Rmdn1	1.2674E-41	3.27E-32	4E-05	0.076349642	-0.09834954	-0.03529982
C730027H1	1	1.08E-20	3E-56	0.003794823	-0.032921165	-0.035303688
Cables1	1.93771E-64	6.77E-17	2E-36	-0.036319875	-0.035073174	-0.035316061
Cys1	1	9.07E-34	5E-83	0.005571865	-0.035127559	-0.035472074
Kansl3	0.0428664	5.42E-03	1E-05	-0.021573553	-0.036463021	-0.035498822
Gm4841	1	2.47E-09	5E-37	0.007976165	-0.027510182	-0.035564468
Lrfn3	1	1.39E-14	1E-27	-0.007359525	-0.038438104	-0.035565596
Gm31718	1.3413E-07	1.00E+00	1E-28	-0.018106715	-0.00544437	-0.03559482
Fancl	1.33521E-06	3.64E-20	5E-13	-0.022633712	-0.055074292	-0.035605414
Calm2	0.392729803	1.00E+00	1E-15	-0.014887087	-0.005956894	-0.035708624
Kifc2	1	2.66E-42	1E-34	0.006382432	-0.054252701	-0.035737667
Esrra	1	9.60E-09	9E-16	-0.007185462	-0.037780409	-0.035802307
Klc4	1	5.83E-17	3E-15	0.001681392	-0.050750651	-0.035810007
Rnf138	0.813967402	1.87E-12	6E-18	-0.01313164	-0.03907657	-0.035813781
Gm48321	1	1.00E+00	3E-19	-0.002221096	-0.00764291	-0.03587406
Ipo8	0.139325951	2.10E-09	6E-09	-0.017280619	-0.045574298	-0.035884054
Ip6k1	1	4.85E-04	3E-05	0.013807875	-0.033446552	-0.035913792
Pbx3	0.001610453	1.00E+00	8E-06	-0.025512022	-0.010773591	-0.035954493
Abca9	0.166079683	3.38E-30	6E-35	0.012316566	-0.046480646	-0.035964742
Gm11639	7.65638E-10	6.32E-26	1E-71	-0.01415065	-0.034401001	-0.035966534
Ccm2	1	2.31E-09	3E-11	-0.003997086	-0.04062665	-0.036037198
Ttc7	1.70003E-40	1.00E+00	2E-06	-0.075230521	-0.000156461	-0.036093074
Pdzd8	1	1.58E-18	5E-13	-0.008950402	-0.055940439	-0.036158866
Ccdc117	2.46191E-08	2.40E-08	1E-21	-0.020455221	-0.032676254	-0.036164882
Zyg11a	6.0505E-124	3.59E-07	1E-09	-0.097436826	-0.039158371	-0.036166641
Mup22	0.148045065	1.05E-28	9E-82	0.009125094	-0.033903456	-0.036255657
Gm44997	4.38489E-08	6.50E-29	4E-46	-0.014852303	-0.041948179	-0.036325611
AI838599	1	2.46E-16	2E-43	-0.006495853	-0.033671375	-0.036333244
Nrbp1	1	1.87E-12	2E-14	0.003690136	-0.040630757	-0.036420252
4931414P1	0.001652992	2.97E-17	6E-41	-0.011977382	-0.035387336	-0.036462766
Usp25	1	2.51E-07	3E-08	-0.00700246	-0.033640187	-0.03646925
Gm45589	7.50863E-30	5.58E-14	1E-44	-0.025984466	-0.029353185	-0.036481688
Tex12	0.007559423	2.71E-20	2E-77	-0.00902462	-0.030420637	-0.036517217
Sesn3	3.20648E-46	4.40E-36	4E-14	-0.055700384	-0.07842837	-0.036541432
Farp2	4.92622E-10	5.93E-24	6E-06	-0.03599724	-0.084733637	-0.036636619
Prrc2b	1	5.22E-06	7E-06	-0.004737515	-0.043824287	-0.036647289
Clcn2	1.86078E-05	8.09E-13	5E-37	-0.014529764	-0.032978638	-0.036665082
Rbm12b1	1	2.43E-23	2E-23	0.004913974	-0.050294607	-0.036678292
B3gat3	1	6.05E-27	6E-11	-0.005587987	-0.070575221	-0.036697593
2810030D1	1	2.07E-29	3E-35	0.000406685	-0.048775674	-0.036714581
Ptpra	1	5.24E-07	4E-09	-0.015468788	-0.032034669	-0.036766637
Ackr4	2.58376E-30	1.00E+00	1E-11	0.053069081	-0.015693685	-0.036767599
Kcnt2	1	1.89E-27	5E-70	-0.006652535	-0.035639074	-0.036803621
Ubc	1	1.03E-47	3E-06	-0.005582821	-0.119366548	-0.036807894
Cul1	3.63792E-08	1.13E-01	8E-05	-0.039668832	-0.032247854	-0.03688074

Tmem205	0.084712019	2.50E-23	2E-07	0.022487782	-0.075170022	-0.036941611
Ahnak	1.33921E-28	3.24E-02	5E-28	-0.033393358	0.001597083	-0.037016566
Ccdc6	0.061555576	1.40E-06	8E-10	-0.018183223	-0.041004569	-0.037046626
Rnf115	1	1.00E+00	7E-11	0.008870695	-0.013899418	-0.037067406
Gm16833	1	4.55E-12	1E-43	0.00797559	-0.031225822	-0.037081087
Gm50063	9.72781E-19	1.18E-33	2E-79	-0.016987734	-0.037650985	-0.037085538
Zfp369	1	3.45E-24	3E-13	0.002765529	-0.065442903	-0.037091879
Bnip3l	1	1.46E-05	5E-16	0.003443528	-0.02980006	-0.037105919
Mnd1	2.79599E-11	1.86E-22	9E-35	-0.019471237	-0.044408955	-0.037114173
Pkp2	9.2732E-09	4.20E-73	2E-06	-0.034193076	-0.146458777	-0.037116628
Lipe	0.090704147	4.94E-28	2E-29	-0.01158124	-0.049741065	-0.037163871
Hivep1	1.10727E-53	1.41E-02	1E-09	-0.08639477	0.076211747	-0.037170001
Cpsf7	1	3.09E-28	1E-09	0.015891838	-0.076064464	-0.037176403
Cebpe	1	7.67E-19	3E-58	-0.002211359	-0.033120611	-0.037180473
Klf10	3.7761E-25	6.29E-14	1E-67	0.029133725	-0.025065484	-0.037189138
Gm5916	5.91868E-07	1.94E-34	2E-90	-0.011181272	-0.036256881	-0.037235271
Sftpa1	0.091406236	2.41E-35	1E-88	-0.008129852	-0.037127381	-0.037328985
Kctd2	1	6.04E-18	4E-22	-0.000463947	-0.046530199	-0.037335785
Gorasp2	1	4.81E-19	6E-11	0.001411824	-0.061299836	-0.037362756
Ddx42	1	2.95E-14	1E-08	0.009967769	-0.058928933	-0.037411507
Prkaca	1	5.98E-07	2E-28	0.006073777	-0.02928453	-0.037493549
Tax1bp1	7.93134E-10	1.00E+00	0.0002	0.041079248	-0.015538722	-0.037523072
Gm16551	2.7949E-97	1.74E-26	1E-80	-0.032333922	-0.034526153	-0.037526272
Pglyrp2	2.04268E-10	1.83E-31	8E-63	-0.015042061	-0.039622295	-0.037622536
Pknox2	5.76062E-07	2.24E-33	5E-76	0.015750165	-0.038579049	-0.037643907
Rbm14	1	7.47E-17	2E-14	-0.000706456	-0.05359863	-0.037651218
Retreg3	0.003040228	4.50E-07	3E-14	-0.018011544	-0.034392322	-0.037653131
Hspb11	5.19738E-12	2.30E-16	3E-38	-0.019111266	-0.036569231	-0.037662657
Nrg4	1.10365E-32	5.08E-01	9E-08	-0.0687286	-0.017111617	-0.037666515
Acadl	0.00022899	1.56E-16	6E-10	0.030828516	-0.063413492	-0.037674825
Nxph1	1.16788E-08	1.06E-30	2E-85	0.016029381	-0.035530467	-0.037747244
F10	1.08E-152	9.08E-24	2E-08	-0.136965225	-0.080106284	-0.037750955
Rbbp8	0.001446096	3.08E-09	1E-09	0.022374451	-0.045463684	-0.037761341
Gm16121	1	5.62E-24	4E-68	-0.00726753	-0.035082168	-0.037761804
Cdv3	1	3.18E-03	1E-15	-0.002070596	-0.024614048	-0.037789502
Gm26549	0.105513354	1.14E-15	1E-20	-0.013506361	-0.045713586	-0.037816301
Herc6	9.84433E-29	3.25E-02	3E-20	-0.035268263	-0.00435755	-0.037824305
Wnt5b	0.003094457	1.28E-35	1E-23	-0.016210722	-0.063685605	-0.037872038
Nfix	1.23265E-16	4.69E-39	6E-06	-0.051400037	-0.124092147	-0.037876013
Zfp523	1	1.80E-29	4E-23	-0.000493501	-0.058680895	-0.037944414
Kank1	1	3.80E-33	0.0001	-0.010689347	-0.109761923	-0.037980783
Atf2	1	3.71E-02	2E-09	-0.008701176	-0.02518548	-0.037998351
Wdcp	1	3.20E-20	3E-20	-0.003641686	-0.051709662	-0.03800439
Aspdh	4.99262E-13	1.35E-79	2E-20	0.030638036	-0.102588615	-0.038011575
Fgd4	3.36136E-50	1.00E+00	0.0004	-0.099089929	0.053195061	-0.03803484
Fam168b	0.970883837	3.10E-03	2E-14	-0.014492477	-0.026537511	-0.0380383
Rab21	0.146768365	5.56E-01	3E-10	-0.017622586	-0.019357682	-0.038055835

Gm44787	1.38222E-54	1.12E-13	4E-33	-0.040995939	-0.036259648	-0.038128653
Fam117b	1	1.98E-10	4E-27	-0.008399227	-0.03165248	-0.038155741
Fat1	2.3797E-17	1.53E-22	1E-09	-0.040885714	-0.075295236	-0.038207872
Arsa	1	2.79E-14	5E-43	0.003669972	-0.033305958	-0.038312916
2700069I1	1	3.21E-29	1E-70	-0.001976969	-0.038393862	-0.038461172
1700112D2	1	4.69E-28	9E-42	0.008881029	-0.046069405	-0.038465327
Taok1	1	6.93E-02	5E-06	-0.00034493	0.049176701	-0.038491716
Pccb	1	1.45E-29	5E-19	0.00015584	-0.062977272	-0.038542283
Dcaf7	1	6.84E-24	2E-14	-0.007883158	-0.065277699	-0.038546082
Gm34667	1.05662E-19	1.04E-51	1	-0.059208742	-0.215906864	-0.038583475
Rprd1b	1	7.42E-16	2E-09	-0.007853237	-0.060261252	-0.038597395
Cdh4	1	1.67E-27	1E-55	-0.002875525	-0.038835691	-0.038629638
Serpine2	1.22251E-10	9.83E-31	3E-86	-0.01399692	-0.036848743	-0.038697074
Psip1	1	1.60E-03	5E-21	0.001264421	-0.024408206	-0.038697176
Dcdc2a	1.30068E-17	1.65E-29	7E-84	-0.017187035	-0.035082656	-0.038723729
Dgkd	1	1.09E-03	6E-11	-0.008808006	-0.024765122	-0.038737283
Tmem143	1	2.47E-15	1E-25	0.005531488	-0.04393622	-0.038765886
Tent4a	1	3.66E-08	3E-20	0.01017853	-0.036220858	-0.038836499
Ar	1	2.17E-19	6E-64	-0.007530397	-0.034302342	-0.038902461
Sumo1	1	2.78E-09	4E-09	0.004995189	-0.04882279	-0.038925207
Wdr20	1	3.78E-18	1E-05	0.013998119	-0.078095338	-0.038951743
Kcp	2.16827E-16	1.13E-34	5E-89	-0.016252069	-0.038338019	-0.038962155
Nr1h3	0.12545338	6.59E-27	3E-14	0.019705397	-0.065585773	-0.039034801
Ly6g2	1	3.02E-13	3E-20	-0.003742759	-0.045479692	-0.039067301
Tmem64	1	1.73E-15	3E-31	-0.005907628	-0.040064728	-0.039082262
Crat	3.33494E-72	6.54E-21	3E-29	0.065870582	-0.048104751	-0.039155324
S100a13	0.000326191	1.00E-25	3E-58	-0.012130039	-0.039109712	-0.039157561
Stbd1	1	1.63E-27	7E-64	-0.004103055	-0.039079646	-0.03917545
Dnmt3b	6.34543E-21	1.04E-23	1E-34	-0.026397875	-0.046609524	-0.03926755
Snx7	1.17304E-05	2.06E-19	6E-16	-0.022170356	-0.059783304	-0.039345099
Paqr3	3.47421E-07	1.88E-22	7E-41	-0.016166156	-0.041542476	-0.039365714
Tns2	1	1.16E-09	3E-42	-9.04153E-05	-0.029071744	-0.039402088
H2-Ke6	1.35172E-08	1.40E-26	8E-28	0.022306376	-0.052532433	-0.039418008
Echs1	1	1.32E-38	4E-15	0.003320872	-0.079678213	-0.03950283
Tle5	1	6.30E-09	3E-13	-0.008251363	-0.042683362	-0.039537815
Plxnb2	1	5.81E-17	4E-19	-0.001728082	-0.047960197	-0.039576144
Gm16984	1	3.17E-05	3E-27	0.009943783	-0.029583938	-0.039623802
Mavs	1	8.05E-31	7E-14	-0.00892138	-0.080052768	-0.039639335
Gm13449	0.012725409	5.12E-17	6E-68	-0.0098873	-0.031625347	-0.03973321
Ctnnb1	0.145717524	1.00E+00	1E-13	-0.018101866	-0.000662217	-0.039764896
Zbtb7b	1	3.74E-30	4E-23	0.002746926	-0.063082281	-0.039767796
Gin1	2.54912E-74	7.60E-22	1	0.100924583	-0.103285763	-0.03979972
Ago1	0.003274131	3.31E-23	5E-26	-0.015079522	-0.050835862	-0.039834592
Zfp606	0.24106338	2.85E-12	7E-36	-0.011114897	-0.035031599	-0.039859968
Tom1	1	1.00E+00	1E-13	0.008481569	-0.006579989	-0.03990029
Nr6a1os	3.52983E-08	5.76E-06	7E-54	-0.015518415	-0.023153737	-0.039925623
Faf2	1	9.71E-29	9E-08	0.005862344	-0.093185612	-0.039939092

Klhl15	1.00466E-14	5.64E-07	3E-23	-0.027385484	-0.030733847	-0.039948454
Ctbp1	1	2.78E-12	8E-29	-0.008884128	-0.038151858	-0.039974586
Glb1	2.40276E-28	7.92E-10	3E-23	-0.037117718	-0.033715606	-0.040152993
Josd2	1	5.50E-37	3E-17	-0.00140533	-0.07585479	-0.040154118
Gigyf1	1	6.81E-05	1E-13	0.004063678	-0.029784608	-0.04021864
Acrbp	1	1.03E-28	1E-22	0.000464016	-0.059300269	-0.040232623
Kcnc3	1	4.21E-41	8E-28	0.003565878	-0.067279652	-0.040369086
Ncoa5	1	5.24E-22	1E-09	-0.00917155	-0.076301677	-0.040408773
Akap17b	1.82543E-13	2.22E-37	5E-40	-0.021305463	-0.055037778	-0.040475006
Glul	1.16722E-06	1.68E-25	3E-36	0.018970138	-0.044914662	-0.040503355
Il6st	0.008074105	1.00E+00	9E-11	-0.021706025	-0.010462941	-0.040505012
Gm37336	5.99713E-06	2.36E-41	8E-77	-0.012045031	-0.045218874	-0.040506017
Zfp653	1	2.99E-35	6E-29	-0.001484654	-0.060785016	-0.040527378
Taco1	0.007854351	1.38E-07	4E-15	-0.017429912	-0.038304456	-0.040631758
Pfkfb2	1	3.58E-27	9E-05	0.012871794	-0.100857976	-0.040637562
Dio1	1	1.78E-34	2E-74	0.003156391	-0.041679845	-0.040651951
Notch2	1	2.43E-07	1E-09	0.019555316	-0.037499537	-0.04065597
Plbd1	1	4.10E-36	4E-17	0.013631402	-0.072787186	-0.040689224
Ndel1	0.042709114	1.00E+00	9E-12	-0.020683418	-0.016116058	-0.040772861
D730045B(	1.72494E-09	8.42E-12	8E-80	-0.014422129	-0.025778028	-0.040860842
Tarsl2	1	6.87E-21	2E-36	-0.004080744	-0.045294229	-0.040889269
Srd5a2	2.52493E-37	9.20E-08	1E-39	-0.03284142	-0.02900984	-0.040910584
Bdh2	1	1.36E-27	5E-89	-0.0072378	-0.036036633	-0.04091524
Lemd3	1	1.79E-20	2E-11	0.016817704	-0.072535556	-0.040929376
Ercc5	1	1.47E-46	3E-12	-0.005640513	-0.100396603	-0.040949275
Socs4	0.000754471	3.44E-10	3E-27	-0.016164845	-0.03638786	-0.040959556
Pgm1	0.418785842	2.39E-14	8E-11	-0.022685591	-0.067872065	-0.040985393
Sucla2	1	2.30E-20	7E-12	-0.003699165	-0.069033278	-0.040996869
Arhgef37	0.448925027	2.21E-33	4E-10	-0.020937485	-0.090178342	-0.041008647
Mup1	9.36691E-63	4.52E-36	7E-98	-0.028387447	-0.039742816	-0.041063518
Agtpbp1	1	1.39E-09	3E-25	0.008827781	-0.033910947	-0.041072139
Hmgcl	0.005134288	4.60E-08	4E-09	0.027129562	-0.046288673	-0.041110247
Nav2	1	1.98E-77	6E-11	0.013835571	-0.190629362	-0.041121426
Pogz	1	5.83E-16	8E-13	-0.013024976	-0.05748541	-0.041131335
Cebpa	1	1.22E-08	1E-50	0.00060455	-0.028013859	-0.041140383
Rhoq	7.58898E-12	2.49E-11	9E-45	-0.019674766	-0.027766188	-0.041163233
Stxbp3	1	4.17E-01	2E-19	0.000355508	-0.016453954	-0.041197192
Tlcd2	0.196996073	2.90E-26	5E-41	-0.0116801	-0.047564162	-0.041218195
Pias2	1	7.53E-25	1E-08	-0.015864093	-0.087945859	-0.041310004
Acvr2a	1	1.26E-19	1E-10	-0.005145551	-0.072246362	-0.041336627
Ccni	5.97724E-17	1.08E-26	7E-15	-0.035524772	-0.068247208	-0.041346607
Dennd1b	7.42995E-10	1.00E+00	8E-07	-0.045649768	0.055528239	-0.041364478
Trim41	1	4.59E-15	2E-16	-0.000947197	-0.050313178	-0.041374202
Prr12	0.001216529	9.79E-19	1E-32	-0.014908315	-0.044995022	-0.0414229
Pgrmc2	1	4.69E-30	3E-51	-0.006189135	-0.046628906	-0.041426797
Dynll2	0.036486071	4.78E-10	7E-30	-0.013598894	-0.035039151	-0.041440204
Bend7	9.1893E-27	4.02E-29	2E-10	-0.050822086	-0.086379741	-0.0414737

Fn3k	1.23901E-12	1.86E-27	8E-59	-0.017926407	-0.04253921	-0.041485489
Mcm9	1	4.37E-11	7E-22	0.002285705	-0.039966856	-0.041495216
Plk3	4.05258E-30	5.90E-08	2E-19	-0.048797369	-0.037748532	-0.04150577
Rab4b	1	2.97E-23	4E-33	-0.001017665	-0.047021644	-0.041529889
Olfr1034	2.89564E-08	1.13E-32	5E-74	-0.014086127	-0.042128709	-0.041611091
Pitpna	4.96676E-05	1.00E+00	3E-18	0.025198795	0.021366143	-0.041696602
Bphl	1	2.86E-22	6E-08	-0.01546359	-0.083032952	-0.041741248
Aldh3a2	1	6.86E-34	0.1102	0.006610701	-0.122637312	-0.041750254
Setd2	1	1.00E+00	1E-07	0.011465163	0.00529194	-0.041771671
Cideb	0.001281122	2.95E-32	2E-27	0.018140524	-0.06081261	-0.041774269
Ephb4	0.185892432	5.34E-26	2E-52	-0.01053006	-0.04218563	-0.041776113
Nrep	2.91514E-87	5.54E-38	3E-95	-0.032656929	-0.040994714	-0.04179653
Tsen34	0.011347009	1.98E-21	1E-42	-0.012301155	-0.041790432	-0.041899037
Hnmt	0.020525121	1.17E-10	2E-58	-0.010749814	-0.02881932	-0.041921461
Klhdc10	0.002027087	3.10E-34	5E-07	0.029978176	-0.109212343	-0.041934623
Gas7	5.03842E-17	1.99E-23	5E-59	-0.021363833	-0.037673109	-0.041991978
1110059E2	4.85681E-13	1.62E-21	3E-10	-0.041662487	-0.079627658	-0.042022757
Sgpp1	1	5.93E-11	1E-38	0.007007633	-0.032102941	-0.042040297
Cab39l	0.086973383	9.73E-14	1E-05	0.022887547	-0.08841946	-0.042046492
Cnnm4	1	7.96E-19	6E-75	-0.005528683	-0.033171076	-0.042058233
Lrrn3	1	1.00E+00	6E-35	0.01142528	-0.014135094	-0.042123547
Habp4	1	2.20E-29	3E-19	-0.006861198	-0.069908726	-0.042148519
Tmc4	1	5.13E-39	4E-41	-0.004728747	-0.056989716	-0.042150374
Fxn	1	3.60E-17	2E-30	0.002723944	-0.046484301	-0.042154451
B230206L0	0.0020917	2.27E-03	4E-33	-0.01464262	-0.023081157	-0.042275563
Phf1	1	2.05E-19	6E-42	0.008440153	-0.041443445	-0.042303745
Angptl4	2.31544E-63	1.84E-54	4E-10	0.089819486	-0.122480591	-0.042401829
Slk	1	2.46E-32	8E-12	-0.008089443	-0.08504486	-0.042417096
Pank2	1	5.01E-12	2E-16	-0.013099306	-0.047472446	-0.042437131
Alpl	1	2.66E-20	2E-32	0.000416884	-0.048886459	-0.042442467
Isoc1	1	6.76E-28	2E-56	-0.006892186	-0.044628048	-0.042458316
Tnrc18	1	3.65E-01	5E-09	0.000261505	-0.025330303	-0.04250058
Zfx	1	3.05E-21	6E-11	0.012904423	-0.07369436	-0.042541704
Sdc1	9.68897E-43	1.32E-26	9E-30	-0.043581352	-0.056895056	-0.042576075
Kdm7a	2.43273E-05	6.13E-02	6E-13	-0.026365232	-0.017811032	-0.042595255
Gm49668	1	1.84E-25	3E-36	0.010029108	-0.052546149	-0.042659935
Arf3	9.00001E-12	1.22E-11	4E-19	-0.028570675	-0.04337229	-0.042697207
Cyp8b1	1	1.41E-52	2E-36	0.012517148	-0.070145171	-0.042698153
Mplkip	0.182626513	1.47E-07	8E-31	-0.01261373	-0.032106696	-0.042732418
Wbp1l	0	1.89E-33	8E-09	0.27089149	-0.114149705	-0.042760966
Gm15318	0.311165966	1.62E-17	3E-46	-0.010358703	-0.0406854	-0.042794112
Fbxo34	1	2.89E-38	4E-07	-0.01138208	-0.120527984	-0.042810178
Taf4	1	2.11E-13	3E-16	0.010686687	-0.051965477	-0.042835302
Gm43597	1	8.00E-36	3E-56	-0.00694707	-0.049888174	-0.042848826
Ccdc12	1	4.49E-16	5E-17	0.002527454	-0.052389599	-0.042891721
Dstyk	1	4.60E-19	3E-15	0.00149264	-0.062022719	-0.042930435
Samd8	1	5.95E-07	3E-19	0.011188141	-0.034634091	-0.042957473

Igfals	1.31513E-45	8.07E-28	1E-46	-0.034628281	-0.048003586	-0.042969878
Ptbp3	2.04821E-48	1.33E-09	2E-09	-0.083497122	0.086243409	-0.043060188
Ak4	3.28678E-27	1.91E-07	3E-17	-0.044225272	-0.042249736	-0.043108265
Adal	1	2.37E-18	6E-29	0.00673962	-0.049883163	-0.043191113
Gm32569	5.25903E-13	5.90E-36	4E-91	-0.016563292	-0.042472474	-0.043235362
Wasf2	1	1.00E+00	3E-11	0.00724339	0.025230371	-0.043261587
Hsf5	6.19691E-24	7.92E-40	4E-51	-0.025642603	-0.055114395	-0.04333788
Slc39a8	5.58536E-14	4.11E-09	4E-22	-0.030082035	-0.040942995	-0.043412422
Tmem38a	0.734922131	2.42E-16	1E-54	-0.009546576	-0.03698957	-0.043470331
Ubr3	7.40783E-09	3.21E-11	2E-09	0.038906688	-0.055773745	-0.043478711
Tango6	0.024644254	6.10E-19	1E-09	0.027963102	-0.078650142	-0.043517723
Cirbp	1	1.88E-25	7E-42	-0.001433814	-0.047004304	-0.043534993
Mosmo	1	6.78E-07	2E-16	0.006307213	-0.040172165	-0.043554431
Flvcr2	1.97594E-17	3.41E-37	1E-53	-0.0224245	-0.052028062	-0.043591563
Agrn	1	3.73E-37	6E-29	0.000486395	-0.066579959	-0.043605914
Rnf146	1	2.13E-12	3E-20	-0.001699485	-0.047091247	-0.043673742
Nqo2	0.004387956	3.14E-12	2E-28	-0.015977895	-0.040704761	-0.04372528
Prickle1	1.9542E-10	1.02E-40	1E-21	0.030310062	-0.074606252	-0.043725673
Prkd3	0.632601131	2.36E-54	5E-10	0.021581432	-0.149400567	-0.043787133
Cyp2u1	2.31091E-07	2.14E-16	7E-58	-0.016093737	-0.036283129	-0.04387297
4930594M	4.51342E-18	4.51E-30	5E-58	-0.022130774	-0.047232715	-0.043953756
Chrna4	3.7403E-102	2.18E-60	2E-57	0.074255684	-0.068424257	-0.043963401
Kctd21	0.937726034	1.36E-22	2E-53	-0.009313301	-0.043002606	-0.043973387
Mreg	5.66207E-53	1.00E+00	0.0004	-0.090773271	-0.018151074	-0.043996446
Dtx2	1	1.27E-17	6E-44	-0.006325264	-0.041372887	-0.044018234
Pou6f1	2.86219E-09	6.82E-15	5E-35	-0.021073857	-0.041244656	-0.044076053
Nadk	6.90619E-38	2.32E-22	3E-10	0.071317088	-0.076993149	-0.044085148
Vamp5	0.000228839	4.38E-31	1E-83	-0.01173948	-0.04079609	-0.044108074
Dnajc3	1.14745E-07	4.57E-38	2E-09	0.04047028	-0.11014668	-0.044135965
Cand1	0.173739334	1.70E-22	5E-19	-0.016486652	-0.064000147	-0.044183112
Hs3st3b1	1	2.07E-77	2E-17	0.014297258	-0.125896402	-0.044199945
Itga5	1	1.52E-05	3E-23	0.009583231	-0.028491373	-0.044231489
Scaf4	1	4.28E-15	5E-09	0.009292986	-0.069333655	-0.044366431
Pdlim1	1	1.34E-29	9E-31	-0.010056975	-0.061656943	-0.044384901
Rarres1	1.20493E-93	9.91E-23	1E-38	-0.053634516	-0.049474846	-0.04438765
Relch	1	3.14E-29	3E-10	0.000819213	-0.094055302	-0.044483071
Albfm1	0	2.33E-108	0.0004	-0.281432144	-0.245753109	-0.044484695
Zswim6	8.00345E-10	7.12E-05	3E-13	0.037820587	0.071405847	-0.044485459
Thrsp	5.10773E-22	1.25E-39	4E-39	-0.029416533	-0.061772963	-0.044527912
Cisd1	2.28186E-12	3.49E-18	1E-27	0.030738602	-0.049638607	-0.044536425
Inhbe	0.165030485	6.30E-28	2E-58	-0.010154243	-0.04609053	-0.044563271
Taf3	1	9.90E-27	7E-08	-0.00879055	-0.09341233	-0.044574375
Nphp3	1	2.27E-34	1E-37	-0.0109529	-0.060693464	-0.044795591
Dgka	0.844335121	4.59E-28	3E-71	-0.009341142	-0.039525851	-0.044896155
4931406P1	1	7.13E-04	4E-12	0.000745826	-0.031709173	-0.044900113
Snx27	1	2.29E-03	4E-21	-0.010321525	-0.01044463	-0.044900422
Maco1	0.013060094	2.34E-12	8E-13	0.023983009	-0.055815597	-0.044932149

Trp53inp1	1	3.01E-49	1E-09	0.011936803	-0.146324571	-0.044970706
Shf	8.51672E-23	2.14E-50	6E-52	-0.025858859	-0.063086279	-0.044976155
Adam22	9.54308E-43	1.34E-22	1E-70	-0.031163151	-0.041177673	-0.045030334
Slc10a5	1.65141E-05	5.09E-23	5E-28	-0.019516675	-0.057145174	-0.04513517
Adap2os	4.91932E-32	8.82E-01	6E-61	-0.027941562	-0.012448767	-0.045168856
Ccnyl1	4.1678E-09	3.42E-22	5E-45	-0.019784549	-0.046985831	-0.045187997
Map3k21	5.26972E-11	1.50E-14	1E-43	-0.021073396	-0.039647984	-0.045278657
Gcnt4	1.75594E-22	3.63E-38	2E-96	-0.020519929	-0.044336281	-0.045361403
Plcl1	1	9.73E-21	2E-70	0.005995782	-0.034140706	-0.04536575
Vapa	0.01701896	2.04E-05	2E-12	-0.023382149	-0.038468343	-0.045414986
Olfml1	2.18966E-23	5.75E-37	2E-36	-0.031798624	-0.064843026	-0.045436366
4930517O1	1	1.00E+00	1E-21	0.011656227	-0.017624007	-0.045441904
Tmem265	1	7.44E-34	3E-34	-0.00016636	-0.061986654	-0.045480319
2610035D1	3.15177E-07	1.00E+00	9E-26	-0.025329763	0.005260096	-0.045616579
Amer1	8.02817E-05	9.07E-32	2E-66	-0.01359464	-0.047724643	-0.045635645
Zfp975	1.80314E-18	1.17E-63	4E-17	-0.038864473	-0.112435392	-0.045699594
Rufy4	1.68866E-07	7.85E-43	6E-94	-0.01391097	-0.046677656	-0.045713432
1810034E1	1	1.00E+00	4E-18	-0.006270052	-0.019359805	-0.04581295
Sgk3	1.00039E-06	3.73E-09	2E-15	-0.027459271	-0.041484992	-0.045857286
Cdc42ep5	3.30873E-09	1.76E-34	2E-86	-0.016222479	-0.044925291	-0.045920801
Cyb5r3	1	4.11E-15	3E-39	-0.009601777	-0.041876206	-0.045955157
Zbtb10	9.4724E-28	2.28E-33	2E-60	-0.027161698	-0.050889626	-0.045976454
Cacng7	0.002923435	9.24E-46	3E-115	-0.010395929	-0.045788841	-0.046103062
Rbm15	1	9.28E-22	4E-33	-0.009839666	-0.053275321	-0.046112351
Rnd1	1	9.30E-22	4E-58	-0.006973066	-0.040770148	-0.046126692
Acbd4	1	3.26E-30	1E-34	-0.008837284	-0.06099624	-0.046197568
Adam11	1	3.35E-45	1E-118	0.004413853	-0.045071348	-0.046200318
Ankrd17	1	1.41E-21	2E-10	-0.022390408	-0.073212048	-0.046201169
Ing2	1	1.06E-32	1E-35	-0.009261268	-0.060445643	-0.046215153
Txndc15	0.070509482	4.59E-15	1E-22	-0.016996703	-0.052887805	-0.046218683
Gm15563	9.7376E-05	3.37E-01	1E-53	0.015882756	-0.018409408	-0.046220822
F7	4.08126E-15	9.24E-08	1E-26	-0.030387795	-0.02922993	-0.046231892
Cpne2	1	2.11E-28	4E-94	-0.00504698	-0.035830704	-0.046256455
Wdr81	1	4.50E-09	1E-30	0.000245508	-0.034383594	-0.046273499
Atp2b1	0.003660227	1.00E+00	6E-24	0.024314097	0.062383955	-0.046289154
Fam228b	1	3.69E-39	4E-44	-0.007054682	-0.061956252	-0.046290137
Osbpl1a	2.18409E-26	5.23E-02	2E-09	0.062703887	-0.021792208	-0.046339368
Tbc1d5	1	2.31E-01	4E-11	-0.027248408	-0.011579999	-0.04643935
Tmem30a	1.15908E-06	8.56E-34	7E-14	-0.028578494	-0.089303917	-0.046472317
Scara5	0.362306744	5.99E-13	9E-84	0.013942538	-0.03030925	-0.046547315
Dipk2a	1.09714E-13	1.59E-15	2E-54	-0.021799167	-0.038156117	-0.046553928
Ski	5.70782E-09	1.36E-32	3E-25	0.030231956	-0.065098892	-0.046574639
Adck1	2.13342E-08	3.03E-37	3E-17	-0.028382494	-0.088790728	-0.046602335
Mtpn	1	6.42E-10	2E-30	-0.012101299	-0.038412473	-0.046612429
Mtmr4	1	7.90E-37	1E-29	-0.006186264	-0.070491082	-0.046713422
Anpep	1	1.51E-01	2E-54	0.005686446	-0.010424181	-0.046764641
Mtm1	1.34534E-14	1.00E+00	9E-16	-0.039697432	-0.005643576	-0.046788442

Inpp5a	1	1.64E-15	3E-24	0.013028919	-0.044607999	-0.046801133
Gm47467	6.31712E-07	2.55E-36	2E-91	-0.013912546	-0.045324635	-0.046828863
Gabpb2	1	4.79E-25	4E-12	0.002832505	-0.087152504	-0.046923207
Tbc1d4	0.000399675	3.76E-19	1E-50	-0.015565729	-0.040502217	-0.046951605
Ap2b1	1	5.55E-03	4E-16	0.008984056	-0.026418115	-0.047043246
Hsd17b13	2.82935E-49	2.12E-08	3E-11	0.113750315	-0.052104559	-0.047043857
Fcho2	1	4.69E-05	3E-10	0.015232412	0.072862011	-0.047096391
Babam2	1	1.00E+00	1E-08	-0.000177885	-0.005517303	-0.047097516
Aldh8a1	1	1.44E-121	7E-10	0.018600225	-0.213903983	-0.047112283
Gpbp1l1	1	1.72E-26	5E-12	0.006715717	-0.087777536	-0.047162902
Pigv	1	1.51E-20	5E-44	-0.003708517	-0.045941476	-0.047179768
2610037D0	1	1.00E+00	1E-19	-0.014063889	-0.001727516	-0.047249859
Meaf6	0.107531596	3.65E-22	3E-47	0.01454603	-0.045274874	-0.047325037
Ccdc157	0.013276571	2.93E-39	8E-50	-0.012879082	-0.059544885	-0.047338755
Foxk2	0.017134307	3.26E-50	5E-14	0.022527861	-0.109760407	-0.047355833
Gm15962	5.64062E-51	1.55E-37	6E-115	-0.02776309	-0.043132885	-0.047361653
Topors	6.25742E-13	1.60E-16	3E-28	-0.029138624	-0.051896325	-0.04740226
Ttll5	1	4.90E-41	7E-13	0.00690887	-0.112064413	-0.047419833
Dcps	1	2.76E-16	9E-17	-0.015615465	-0.061411124	-0.047458295
Pym1	1	9.13E-20	2E-39	0.005331401	-0.048585565	-0.047472431
Tmem164	1	3.77E-02	2E-28	-0.01274931	0.010623114	-0.047615702
Ascc3	1	7.50E-07	6E-10	-0.009341388	-0.043284044	-0.047708527
Taok2	1	3.91E-42	2E-18	-0.002561709	-0.093446036	-0.047722875
Ccnf	0.093992574	5.17E-34	6E-57	0.013078148	-0.054297906	-0.047890676
Arhgap17	1	1.00E+00	1E-25	0.014515249	0.026950627	-0.047916383
Mob2	1	2.14E-08	6E-15	0.005550834	-0.047153242	-0.047930575
1010001N0	0.453635616	3.83E-10	3E-22	0.01825179	-0.046353805	-0.047956795
Mepce	1	4.61E-26	3E-51	-0.00155953	-0.049726039	-0.04801819
Rlf	2.5076E-07	1.88E-05	1E-08	-0.03764947	-0.048364586	-0.04802847
Alg11	1	2.22E-22	7E-33	0.002819106	-0.056462458	-0.048041507
Sfmbt1	0.015018085	7.18E-11	8E-09	-0.02732879	-0.068376217	-0.048076842
Lactb	1	4.39E-16	6E-32	-0.007900679	-0.047543576	-0.048158203
Gm12353	0.000438455	1.00E+00	4E-19	0.023541011	-0.01576981	-0.048199203
Rsrc2	1	1.42E-13	3E-12	0.017682272	-0.057395064	-0.048209518
Pxylp1	4.67351E-14	4.65E-12	6E-30	-0.029576231	-0.045440223	-0.048251605
Alas2	0.068584214	3.55E-36	3E-56	-0.011418007	-0.055976562	-0.048379342
Trim33	1	4.53E-20	8E-14	0.012074212	-0.074419635	-0.04839369
Grid1	1.7273E-35	3.16E-31	1E-82	-0.02902261	-0.046424427	-0.048439748
Kpna1	1	1.61E-14	3E-17	-0.001164562	-0.057509086	-0.048641878
Galnt2	0.004126404	3.77E-29	5E-16	-0.027067364	-0.086018114	-0.048748014
Crcp	4.23941E-29	6.05E-15	2E-19	0.055170972	-0.058487407	-0.048824589
4930404H1	7.92602E-23	1.84E-83	2E-18	-0.051488099	-0.148770031	-0.04883078
Pbrm1	1	1.20E-12	1E-13	0.004151439	-0.05723634	-0.048904401
Ythdf2	1	8.85E-15	3E-21	-0.004326538	-0.05475791	-0.048922636
Lonrf3	1	1.61E-03	2E-38	-0.003665291	-0.023605266	-0.048926715
Srek1	2.82479E-05	1.00E+00	9E-11	0.033486926	0.006250237	-0.049001584
Marveld1	3.86981E-23	8.13E-18	6E-74	-0.024414695	-0.037234847	-0.049018684

E130308A1	0.00110644	8.10E-43	6E-14	-0.024738603	-0.10931615	-0.04907915
Ndrg3	0.074090719	2.76E-22	1E-16	0.023129868	-0.07736088	-0.049118954
Crebbp	1	1.98E-18	2E-14	0.016934313	-0.064916566	-0.049173304
Dsg1c	8.49039E-48	7.66E-47	2E-118	-0.027978946	-0.048703371	-0.049219058
Gm11844	1	4.30E-24	8E-54	0.000195231	-0.04888426	-0.049246535
Insig1	0.001046527	9.31E-108	8E-25	0.028048446	-0.147362845	-0.049353009
Gpam	5.703E-210	7.27E-34	2E-14	0.19684811	-0.116206917	-0.049406288
Pacsin3	1	9.17E-31	7E-40	-0.005206943	-0.061947658	-0.049410148
Thap2	4.11447E-05	3.10E-22	8E-26	-0.021734496	-0.062396309	-0.049419411
Ccdc25	0.02471546	2.59E-12	4E-26	-0.016948078	-0.048035323	-0.049448552
Dnajc18	1	1.35E-38	5E-76	-0.001447513	-0.051755863	-0.049474033
Ranbp3l	1	7.42E-43	2E-109	-0.00557682	-0.048642637	-0.049479076
Rabep2	1	1.08E-32	2E-44	0.002513251	-0.059763838	-0.049585688
Clybl	0.243512072	9.31E-19	1E-06	-0.032506263	-0.095896143	-0.04959133
Atxn3	1	6.95E-14	1E-28	-0.013490958	-0.047876044	-0.049647686
Abi1	1	4.13E-03	1E-13	0.013441823	0.07770483	-0.049651205
Gm47371	1	1.43E-01	2E-35	0.005188943	-0.02299343	-0.049711143
Gpr155	1.56178E-43	2.76E-29	2E-95	-0.029297724	-0.043278938	-0.049881471
Rapgef6	0.545154601	1.00E+00	1E-11	-0.020177344	-0.002413774	-0.049945062
Pdcd7	1	4.81E-22	1E-43	-0.008470956	-0.050036696	-0.050089111
Ube2d3	4.20732E-30	3.84E-07	5E-11	-0.064328995	-0.049438717	-0.05009768
Stx1b	8.42141E-18	1.02E-34	6E-64	-0.023281575	-0.053969732	-0.050107932
Cdkl5	7.1626E-20	7.65E-32	2E-22	-0.042545788	-0.083370436	-0.050132786
Dnajc22	1	5.92E-44	6E-27	-0.012561228	-0.086281683	-0.050232817
Map3k1	3.95603E-05	2.09E-01	2E-23	-0.023584442	-0.015796878	-0.050243631
Dpy19l3	5.57816E-58	1.18E-48	2E-104	-0.03256398	-0.052091748	-0.050252157
Tst	2.1957E-05	7.02E-29	1E-56	-0.016248024	-0.052123814	-0.050333245
Slc36a1	1	1.06E-16	2E-55	-0.009991927	-0.038631291	-0.050339417
Pctp	4.88777E-08	4.67E-38	1E-34	0.024611072	-0.073157128	-0.050353689
Igf1os	1.32645E-96	3.08E-38	5E-44	-0.058661501	-0.065036024	-0.050411472
Lsm14a	1	3.02E-07	1E-19	0.015999509	-0.040533784	-0.050425735
Apof	1	5.90E-43	3E-66	-0.007913746	-0.059209369	-0.050508979
Rp9	0.025059494	8.46E-18	3E-17	-0.020198004	-0.067108371	-0.050540998
Nfic	1	1.00E+00	4E-36	-0.010064388	0.041399683	-0.050541065
Ambp	5.43146E-12	1.41E-38	4E-17	-0.038252133	-0.097607688	-0.050557446
Iffo1	1	7.37E-27	6E-96	-0.002804251	-0.038104632	-0.050634402
Wnk1	1	1.00E+00	2E-12	-0.001557972	0.053532591	-0.050707475
Gjc3	1	1.76E-28	4E-121	0.0007398	-0.03970914	-0.050775238
Cnnm3	1	5.46E-31	4E-32	0.002420219	-0.069159445	-0.050783786
4833411C0	0.000259344	4.32E-65	4E-15	0.028134781	-0.140372467	-0.050878822
Exoc6	4.65539E-06	2.82E-12	9E-14	-0.030468754	-0.060312246	-0.050908702
Adgrf1	2.181E-78	4.15E-51	3E-126	-0.034440682	-0.050946382	-0.05093665
Parp14	5.19614E-05	1.00E+00	8E-44	-0.018842265	0.02975839	-0.05111275
Commd1	4.43143E-07	5.75E-08	9E-19	-0.028283259	-0.044796199	-0.051144162
Ubr5	1	1.00E+00	6E-13	-0.02018802	-0.003693278	-0.051146697
B230307C2	0.373416635	3.09E-23	6E-32	-0.014412774	-0.057899747	-0.051188686
Pnrc1	1	4.44E-20	2E-20	-0.005967764	-0.06696201	-0.051278567

Rsbn1l	1.09594E-07	2.31E-16	1E-15	0.036167911	-0.064789027	-0.051283038
Chac2	1	1.53E-28	9E-37	-0.003487908	-0.065270836	-0.051505587
Rad51d	1	6.37E-21	3E-32	-0.007079367	-0.056989174	-0.05179174
Pclo	4.98776E-11	1.88E-42	6E-66	0.022097209	-0.060548318	-0.05185335
Ptdss1	1	8.33E-28	1E-42	-0.004167848	-0.059201543	-0.051857661
Slc35d2	1.31781E-20	7.08E-26	5E-11	-0.055241956	-0.098115252	-0.051968898
Ppp6r2	1	5.09E-30	3E-14	0.004147348	-0.094710198	-0.05209201
Ppp2r2a	1	3.39E-16	2E-16	0.005029711	-0.067203251	-0.05212052
Rabgap1	1	7.98E-11	1E-11	-0.007955656	-0.066242466	-0.052177915
Lman2l	1	7.33E-25	4E-50	-0.006642941	-0.052413569	-0.052181433
Shfl	3.84303E-11	1.04E-80	9E-16	-0.042221489	-0.161490769	-0.052394182
Acss3	1	4.14E-13	2E-09	0.007430905	-0.069544688	-0.052429216
Uhrf2	1	1.00E+00	3E-18	-0.013044811	-0.01016657	-0.052432549
2900089D1	0.003729771	3.68E-11	3E-81	-0.013403056	-0.026293138	-0.052462059
Gm46329	3.38449E-34	5.01E-33	4E-111	-0.027599098	-0.045906347	-0.052495178
Hbs1l	1	6.57E-40	1E-13	0.013324017	-0.114821983	-0.052555004
Tmem53	0.421184135	7.32E-47	1E-55	-0.011750672	-0.069129422	-0.052617898
Rnf11	8.63826E-86	2.08E-08	3E-20	-0.082950612	-0.041966807	-0.052825061
Cnot6l	0.001623508	4.81E-08	1E-18	-0.024052656	-0.041201745	-0.05289927
Fdxr	1	2.24E-42	2E-38	-0.003894973	-0.076562072	-0.053002201
Atp1b1	6.61784E-32	1.70E-35	6E-18	-0.062757636	-0.099700021	-0.053064584
Il31ra	1	1.80E-13	5E-19	0.017521484	-0.051449313	-0.05307922
Mup14	3.35233E-27	2.67E-46	5E-133	-0.022690199	-0.049029713	-0.053116369
Ndst1	1.2592E-40	7.99E-10	7E-14	-0.072184495	-0.052395841	-0.053118338
Gm45301	1	2.23E-54	1E-132	-0.000247802	-0.053206251	-0.053212783
Ube2e1	2.63232E-27	8.52E-12	8E-21	-0.049992627	-0.055028545	-0.053237712
Atp7b	1	3.03E-13	7E-33	-0.013641637	-0.04953048	-0.053282424
Zkscan3	1	2.81E-10	2E-11	-0.022121705	-0.063506127	-0.053314669
Tm4sf1	0.002435696	2.51E-51	8E-56	-0.01509255	-0.07281939	-0.053616407
Serpina11	2.11595E-22	3.44E-32	2E-74	-0.026618953	-0.053216922	-0.053709569
Zbtb7a	5.94288E-21	1.00E+00	1E-31	-0.039430022	0.001600843	-0.053976386
Slc3a1	3.22132E-18	1.00E-38	5E-28	0.045865271	-0.087602978	-0.054020416
Serpind1	0.018114065	2.05E-63	7E-31	0.021265659	-0.105774195	-0.054038492
Itprid2	1	1.52E-14	5E-37	0.000140588	-0.046440689	-0.054060637
Gm47348	8.23353E-43	1.24E-29	8E-85	-0.033283015	-0.048944718	-0.054065151
Gm17021	6.79953E-18	1.18E-31	6E-79	-0.023593351	-0.051492668	-0.054147985
Nt5c2	0.000100961	6.85E-11	2E-17	0.030717984	-0.05425906	-0.054151069
Grk4	1	3.86E-40	8E-77	0.007172863	-0.057600222	-0.054159244
Ptma	0.000221923	4.75E-06	2E-45	-0.017705458	-0.028617708	-0.054218441
Gm29724	1	7.23E-30	4E-44	-0.00850293	-0.063086049	-0.054263161
Sdsl	1	2.85E-35	4E-59	0.001575968	-0.060561865	-0.054269042
Reck	1	4.92E-42	3E-120	0.001297613	-0.050581042	-0.054364331
Dtx4	0.063702209	2.04E-13	2E-56	0.015190474	-0.037052328	-0.054429858
Thsd7b	1	1.68E-35	4E-108	0.003792012	-0.048374547	-0.054521311
Cldn1	3.18292E-09	9.87E-46	6E-107	0.019929337	-0.05437054	-0.054522381
Apon	0.246605978	5.27E-49	3E-110	-0.009690264	-0.055097649	-0.054548598
Syde2	1	1.16E-38	2E-78	0.006283673	-0.057100874	-0.054655809

F9	1	5.53E-23	5E-23	-0.010925521	-0.073997157	-0.054673524
Tlr5	2.08891E-29	1.71E-56	3E-95	-0.027480574	-0.062596534	-0.054812515
Dnm3	7.88122E-05	9.93E-40	4E-66	-0.01614309	-0.061295397	-0.054842673
Pcmtd2	7.03668E-13	2.93E-27	1E-26	-0.032882023	-0.073873961	-0.054853575
Ncor2	2.63647E-11	6.87E-12	1E-22	-0.03277729	-0.054850239	-0.054989148
Rnf144b	9.78486E-44	3.54E-32	9E-08	-0.099465239	-0.135309947	-0.055016838
Gprc5c	1.07391E-45	6.40E-31	4E-48	-0.044708391	-0.06286844	-0.055089646
Ggps1	1.29435E-06	1.57E-24	2E-31	-0.023890059	-0.066523627	-0.05510389
Socs2	6.70338E-66	7.67E-60	1E-66	-0.045808072	-0.073971641	-0.055112358
Farp1	2.17483E-63	9.15E-23	9E-12	-0.100328888	-0.096518031	-0.05515082
Podn	1.7845E-75	1.42E-40	3E-76	-0.045503155	-0.059498721	-0.055282492
Maml1	0.116672839	7.14E-28	4E-21	-0.019333417	-0.080703481	-0.055314601
Jph1	1.48154E-11	8.34E-47	5E-105	-0.019298848	-0.056973367	-0.055405377
4930549G2	1	5.30E-22	1E-70	-0.002254579	-0.045544403	-0.055519793
Ppp1cb	1.30536E-07	3.01E-05	5E-21	-0.030476949	-0.034999476	-0.055592642
Akap11	1.42568E-06	3.41E-25	7E-24	-0.027855968	-0.07481888	-0.055759538
Gpt2	0	3.11E-27	7E-11	0.308377454	-0.121747582	-0.055780187
Cpox	7.38927E-45	4.23E-50	1E-26	0.068630315	-0.09970677	-0.055817705
Gm17276	9.39188E-54	3.96E-07	6E-44	-0.049990877	-0.039039275	-0.055932605
Bbx	1.62156E-19	4.04E-03	2E-17	-0.05091103	-0.030981453	-0.056002554
Gm13522	5.9915E-48	1.90E-46	7E-121	-0.031503883	-0.054129365	-0.056105764
Kifc3	0.000142911	8.76E-24	2E-56	-0.018072106	-0.052138742	-0.056126372
Rbm4	1	9.23E-25	1E-27	0.004290429	-0.070581468	-0.056195441
Ttc39b	4.79641E-16	1.39E-17	1E-21	-0.040323215	-0.063981474	-0.056235362
Slc25a48	8.33473E-06	3.65E-10	2E-82	-0.015651537	-0.030914201	-0.056254221
Rarres2	1.16775E-08	1.57E-43	5E-91	-0.017349045	-0.058129072	-0.056275213
Slc23a1	0.067527204	1.21E-115	1E-28	-0.016789764	-0.157399606	-0.056312807
9530068E0	2.9778E-05	3.07E-29	1E-59	-0.017319975	-0.05589146	-0.056320755
Mrnip	1	1.37E-12	8E-31	-0.009598189	-0.049287506	-0.056345934
Gna13	0.08783766	1.00E+00	1E-47	-0.01515722	0.002008338	-0.056417218
Zbtb2	4.92485E-09	4.62E-31	5E-47	-0.023360156	-0.064163813	-0.056473593
Cbfb	4.03782E-12	2.76E-19	6E-39	-0.029130462	-0.052009918	-0.056511567
Arl6ip6	1	7.17E-10	2E-41	-0.011822304	-0.041180779	-0.056596562
Mocs2	0.006286759	1.30E-56	1E-22	0.025649652	-0.116704532	-0.056649889
Gm32828	6.28086E-31	1.26E-55	1E-61	-0.036025972	-0.076413775	-0.056653197
1190005I0	0.003878056	6.77E-15	2E-54	-0.016369385	-0.04500371	-0.056816315
Avpr1a	0.061946749	1.55E-54	2E-110	-0.010653323	-0.060530785	-0.056822464
Dnajc7	1	1.03E-16	3E-17	0.012643212	-0.067171598	-0.056847991
Sema5b	2.41102E-12	2.44E-42	4E-99	-0.020039942	-0.056651646	-0.056851187
Smlr1	1	1.24E-27	5E-39	0.004471614	-0.064960976	-0.057162543
Ttc3	2.47919E-10	4.13E-18	7E-25	-0.033041206	-0.06177984	-0.057327067
Gpsm2	9.30183E-13	2.46E-48	6E-67	-0.023089178	-0.069887598	-0.057451809
Ddx10	1	2.11E-26	4E-23	-0.011473087	-0.079246704	-0.057452298
Irf9	1	2.25E-22	6E-65	0.006630026	-0.050477479	-0.057579641
Csrp2	3.22814E-31	4.85E-40	1E-68	-0.033838659	-0.063488304	-0.057580606
Slc44a1	1.72299E-44	1.00E+00	2E-25	-0.060982065	-0.013741262	-0.057603446
Klhl5	6.04658E-59	5.69E-21	7E-34	-0.062348451	-0.061577273	-0.057615305

Lrit2	1.91822E-09	1.06E-35	1E-117	-0.016696455	-0.049528479	-0.05762215
Nxpe2	2.05946E-05	4.38E-56	3E-136	0.016674497	-0.057690502	-0.057632487
Rnf185	1	7.35E-15	1E-26	-0.00663496	-0.058460279	-0.05768797
Gm30784	0.000134808	1.25E-36	3E-53	0.019924707	-0.068909085	-0.057712346
Arl5b	1	9.74E-40	3E-39	-0.01160898	-0.080843745	-0.057802717
Gm15998	1.64715E-11	3.72E-59	3E-112	-0.018149845	-0.063354415	-0.057806367
Tk1	0.028350029	8.22E-56	4E-120	-0.011014786	-0.060693015	-0.057922504
Ptpre	3.38103E-06	5.68E-05	1E-101	-0.016959342	-0.010408133	-0.057947759
Lrrc28	2.64354E-15	5.80E-32	5E-14	0.056650594	-0.115411059	-0.057987761
Nit2	1	1.43E-43	5E-56	2.26037E-05	-0.071760398	-0.057997048
5031439G0	4.35729E-11	2.04E-08	4E-36	-0.029589897	-0.030621794	-0.058063943
Gnaq	9.90291E-37	1.00E+00	3E-16	-0.083076667	0.065098513	-0.058115272
Zmat1	9.63689E-10	1.72E-45	1E-49	-0.024907022	-0.077934176	-0.058149899
Kcmf1	1	4.52E-03	2E-15	-0.017572714	-0.03465309	-0.058167842
Stx2	0.000275573	8.70E-25	6E-78	-0.015181321	-0.048115291	-0.058326348
Alkbh5	1	3.17E-16	8E-46	-0.008880779	-0.049102419	-0.058332226
Gm2061	1	2.44E-49	1E-146	0.008772367	-0.053281285	-0.058440602
Ctcf	1	1.44E-24	2E-29	0.010112025	-0.068993617	-0.058456936
Socs7	1	1.08E-20	1E-40	-0.011960962	-0.056854747	-0.058472042
Pitx3	0.127689066	1.43E-61	4E-155	-0.009357173	-0.057880698	-0.05849176
Smc1a	0.13159268	8.54E-54	6E-21	0.024696525	-0.12092344	-0.058499654
Tpm3	1	5.40E-14	1E-33	-0.004551197	-0.042282919	-0.058550049
6720427I07	4.5568E-17	5.27E-20	1E-29	-0.037335516	-0.065722267	-0.05868679
Nt5c3	1	8.24E-27	8E-49	0.001769687	-0.057874126	-0.058691572
Gch1	1.78108E-72	1.75E-57	7E-07	-0.12902431	-0.180005429	-0.058694962
Gm30881	1.78853E-44	1.64E-37	4E-80	-0.037369065	-0.058708204	-0.05878522
Tpd52l1	1.31663E-20	2.55E-46	3E-59	-0.030581845	-0.074134515	-0.058824771
Cdc42	3.91475E-12	1.00E+00	2E-29	-0.033292651	-0.007593712	-0.058838838
Rbpms2	1.24357E-11	1.23E-20	2E-31	-0.031325447	-0.064189843	-0.058871934
Atl3	1	1.69E-33	9E-15	0.022326209	-0.115028324	-0.058934615
Parp16	1	1.45E-10	6E-38	0.016691795	-0.046148682	-0.058937314
Mitf	1	1.13E-08	2E-15	0.012252751	0.155217725	-0.059035142
Smarcd2	1	3.34E-26	2E-48	0.004503258	-0.060872267	-0.059044704
Lama3	1.84437E-48	1.57E-60	1E-66	-0.044916557	-0.079891045	-0.059147548
Prkce	2.32735E-08	4.82E-02	5E-13	-0.046232242	-0.022708557	-0.059150083
Xpa	1	2.15E-28	1E-33	-0.003293369	-0.074362769	-0.059155219
Rbpj	1	1.33E-16	1E-32	-0.011871522	-0.054953073	-0.059168249
Zbtb46	2.05579E-06	2.03E-41	5E-111	-0.01597737	-0.05283768	-0.059427871
Trhde	0.008259413	3.87E-52	6E-118	-0.014283375	-0.060793023	-0.059481079
Usp9x	1	1.00E+00	2E-16	0.004419277	-0.004596372	-0.059589689
Gm11476	0.000667566	6.75E-50	3E-85	-0.015296526	-0.067093474	-0.059676404
Dgcr2	1	4.54E-18	3E-28	-0.002609349	-0.065025266	-0.059724425
Rspry1	1	2.23E-32	4E-23	-0.001988186	-0.089823945	-0.059830408
Med13l	1	1.00E+00	5E-16	-0.0131037	0.034861191	-0.059956387
Mgam	1.09085E-05	2.56E-72	5E-27	-0.027917827	-0.136865794	-0.059956895
Smg7	1	3.72E-28	2E-21	-0.002268471	-0.08819849	-0.059957833
Paxx	1	1.05E-60	2E-72	0.008136053	-0.077314889	-0.060053109

Magt1	1	1.14E-61	1E-21	0.010227778	-0.134580121	-0.060064305
Brpf3	1	3.92E-32	2E-31	0.003824827	-0.081891333	-0.060094896
Gng12	8.5652E-08	1.00E+00	3E-15	-0.039991175	-0.030142587	-0.060146416
Acnat2	3.31592E-88	8.80E-37	5E-72	0.073587256	-0.062647405	-0.06034369
Phldb3	3.97726E-32	2.82E-43	1E-97	-0.030554848	-0.060223697	-0.060447175
Slc15a5	8.4017E-33	2.01E-30	4E-136	-0.027576031	-0.045874014	-0.060507947
5330438D1	4.47168E-06	1.00E+00	3E-45	0.023251972	0.001220299	-0.060661355
Sema4a	3.27256E-05	4.74E-24	3E-36	-0.023080398	-0.068671554	-0.060667409
Gm32624	3.32557E-52	7.39E-25	2E-27	-0.068845859	-0.074678057	-0.060691771
Sos2	1	6.68E-09	3E-23	0.003744261	-0.054241718	-0.060704712
5430402O1	4.58589E-07	9.76E-20	1E-61	-0.020645057	-0.050751667	-0.060769459
Rnf216	0.396997623	1.00E+00	2E-16	-0.022694281	-0.005427479	-0.060772518
Ankhd1	0.004318734	5.85E-12	5E-19	0.026571545	-0.05276428	-0.060959197
Zdhhc9	0.000765464	2.40E-17	7E-42	-0.020117259	-0.055535198	-0.061124451
Gm16552	1	1.00E+00	5E-71	0.011066542	-0.011628331	-0.061303321
Tesmin	0.027805277	1.27E-31	1E-66	-0.013740048	-0.060845766	-0.061340507
Urgcp	1	1.04E-12	4E-23	-0.002272578	-0.06143766	-0.061465114
H3f3a	0.006020204	3.65E-10	2E-59	-0.016082381	-0.034037626	-0.061502677
B4galt5	3.8744E-36	1.00E+00	3E-28	-0.062651082	0.018625239	-0.061546487
Lrit1	2.6092E-07	5.30E-44	3E-104	-0.01660312	-0.059948971	-0.06155833
D530033B1	1	6.18E-19	7E-90	0.011873971	-0.043714743	-0.061650605
Armc9	0.000206231	3.26E-26	7E-41	-0.021762701	-0.066664578	-0.061737828
Ranbp9	1	1.00E+00	7E-18	-0.017387133	0.045742533	-0.061744844
Apoa5	5.87496E-06	8.05E-94	5E-14	0.03763934	-0.20950686	-0.061768477
Stard4	1	2.00E-84	3E-71	0.010270924	-0.095570304	-0.061821778
Tmem50a	1.9711E-139	7.32E-15	1E-31	0.12427127	-0.057605901	-0.06186097
Sardhos	3.22573E-10	2.89E-41	4E-99	-0.019380811	-0.059856006	-0.061917382
Sptbn2	1	1.39E-17	3E-37	-0.009585729	-0.059618802	-0.061925566
Cbx5	1	7.25E-41	6E-33	-0.007678846	-0.093779435	-0.062171749
Hunk	7.28283E-09	1.70E-31	2E-73	-0.021699933	-0.06046139	-0.062310533
Ube2d2a	1	1.85E-20	2E-22	-0.001455753	-0.075698033	-0.062321482
Gm20045	1.48615E-17	5.34E-46	2E-74	-0.027121438	-0.071183416	-0.062554134
Rnase4	1	2.18E-113	6E-17	-0.015300021	-0.1969312	-0.062569708
Eif4e2	1	1.44E-45	3E-54	-0.010880893	-0.07974176	-0.062641214
Grm8	1	4.69E-24	5E-88	0.006346527	-0.054409663	-0.062652114
Jade1	1	2.27E-25	5E-48	-0.004596236	-0.064184666	-0.062788425
Grtp1	1	6.96E-44	3E-56	0.001465351	-0.078426873	-0.062856214
Fam162a	1	1.46E-23	3E-22	-0.01833774	-0.082493138	-0.062976913
Fxyd1	1.2423E-38	3.92E-63	7E-39	0.060698489	-0.110233216	-0.062977072
Jmy	1	3.36E-19	2E-27	-0.000117704	-0.072377657	-0.063289913
Ston1	1	2.95E-23	2E-64	-0.011449323	-0.054575919	-0.063318203
Adcy6	1	1.72E-33	5E-45	-0.005248045	-0.075890707	-0.063359415
Axin1	1	8.14E-23	3E-41	0.000320039	-0.064343894	-0.063393657
Ttc38	1.02159E-05	2.21E-42	2E-39	-0.022073474	-0.090165504	-0.063411387
Ccdc57	0.000178322	3.90E-19	7E-79	-0.016484888	-0.046336806	-0.063463458
Pbld2	0.025912418	7.67E-14	1E-15	-0.032330946	-0.084006831	-0.063484237
Amfr	1	1.43E-59	5E-23	0.001913828	-0.131119346	-0.063504235

Senp7	1	2.27E-37	7E-33	0.004359225	-0.088236189	-0.063539873
Gda	0.004119703	5.73E-23	8E-84	-0.014955617	-0.040448422	-0.063634688
Cep85	2.6296E-215	1.00E+00	8E-19	0.190807317	-0.013622297	-0.06377394
Bckdhb	1.41808E-81	6.61E-118	3E-17	-0.123773588	-0.230547093	-0.063857386
Tmem170b	0.000120693	3.52E-19	3E-57	-0.019219634	-0.052879095	-0.063869356
Akt2	1	1.25E-31	4E-36	0.000111744	-0.081372064	-0.063913703
Atf6	2.4497E-81	1.00E+00	9E-18	0.119819264	-0.019517513	-0.064003583
Rap2a	1.02521E-10	4.09E-32	1E-101	-0.020451188	-0.052057457	-0.064008926
Pnpla6	1	2.41E-58	5E-33	0.004839243	-0.113053499	-0.064019138
Shroom2	0.355432833	4.71E-01	2E-17	-0.023469542	-0.035822405	-0.064082199
Acot7	1	3.55E-45	2E-51	-0.013697743	-0.083723715	-0.064084654
Mboat7	4.4263E-10	2.58E-46	3E-80	-0.02237532	-0.070203415	-0.064111793
Tmtc4	8.09205E-46	3.11E-43	3E-119	-0.035014523	-0.058661537	-0.064138426
Cog5	1	1.00E+00	2E-19	-0.013492474	0.020976956	-0.064157875
Sp1	1	7.66E-19	8E-65	0.003800976	-0.048382301	-0.064262927
Gm27003	1	1.64E-49	9E-79	-0.008287723	-0.073095539	-0.064271733
Bche	1	5.41E-43	2E-26	0.007002753	-0.103872837	-0.064287547
Zcchc7	1	6.16E-11	1E-20	0.01561502	-0.062521015	-0.064461724
Hsd11b1	1.82163E-08	6.31E-17	1E-51	-0.027968276	-0.055806828	-0.064507424
Gm13483	1	1.96E-37	3E-23	0.003511212	-0.104089103	-0.064613876
Slc17a5	2.83555E-14	7.13E-07	4E-29	-0.039364392	-0.044702339	-0.064734574
Cpt2	0.000706455	3.33E-27	4E-38	-0.021670735	-0.076241132	-0.064783105
Kdm1a	1	1.29E-12	5E-39	-0.002122707	-0.048687948	-0.064830405
Atg14	1	2.11E-28	8E-47	0.013393936	-0.070382233	-0.064865389
Ablim2	0.144532365	1.01E-49	2E-119	-0.011543844	-0.063482419	-0.064870579
Upf2	1	1.52E-50	1E-20	-0.006795073	-0.130523214	-0.064885478
Hdac4	3.99928E-05	4.57E-11	2E-24	-0.026940441	-0.056787591	-0.064978069
Sp4	3.8771E-20	7.35E-34	5E-86	-0.029009678	-0.058980437	-0.065102896
Rab11b	1	1.75E-26	4E-41	0.006631657	-0.070634693	-0.065107926
Cab39	1	1.54E-10	4E-26	-0.00256271	-0.05270408	-0.065144837
Isoc2a	1	1.00E+00	7E-30	-0.016533498	0.011686474	-0.065236081
Cbx1	1	1.51E-20	7E-44	0.007029928	-0.061882114	-0.065241299
Lgals8	1	1.26E-53	7E-20	0.00224623	-0.137296433	-0.06526088
Brd2	0.000170301	1.57E-27	6E-28	-0.025924035	-0.083473628	-0.065283958
Nceh1	2.79198E-60	6.96E-01	9E-35	-0.073519706	-0.014444972	-0.065386055
Nab1	0.000212006	1.84E-26	2E-45	-0.021369096	-0.069233902	-0.065395475
Spsb4	1.73996E-25	2.67E-39	1E-122	-0.027952855	-0.054186678	-0.065400996
Kmt5b	1	5.84E-30	3E-22	0.015338414	-0.097394279	-0.06543562
Gstk1	1	4.05E-64	1E-117	0.005791147	-0.072128231	-0.065456921
Pdia3	1	4.10E-48	8E-29	0.024947981	-0.112213756	-0.065470292
Mcur1	1	3.41E-35	6E-79	-0.001745813	-0.063569183	-0.065532921
Map3k13	1.5241E-10	4.79E-07	4E-16	-0.050887482	-0.058846014	-0.065597131
Mprip	3.75444E-38	4.88E-25	9E-19	0.075848807	-0.099763091	-0.065637212
Fam214a	1.803E-134	1.05E-48	0.001	0.198974436	-0.217960802	-0.065670403
Coq10b	1.5544E-154	9.48E-19	4E-48	-0.089782922	-0.057167518	-0.065682613
Wrn	5.21321E-13	6.40E-23	3E-40	-0.032747697	-0.064874093	-0.065763245
E030030I06	1	4.76E-44	3E-79	-0.010419935	-0.071281348	-0.065774923

Paox	0.44095684	9.44E-43	8E-49	-0.015309037	-0.083829701	-0.065849605
Hspb8	1	1.30E-31	2E-38	-0.006177811	-0.080945727	-0.065907544
Arhgef3	1	2.26E-91	2E-18	-0.006927376	-0.2086754	-0.066034681
Exd1	5.04592E-22	6.50E-47	2E-70	-0.033475018	-0.076858389	-0.066085891
Amn1	1.93388E-20	3.05E-31	1E-30	-0.046835209	-0.087108527	-0.066170597
Coq9	1	3.72E-43	4E-44	0.008553385	-0.090863928	-0.066202469
Zfp771	1	1.15E-30	3E-53	-0.006319137	-0.071820331	-0.06621533
Hp1bp3	1	8.97E-67	3E-27	0.020051616	-0.135930615	-0.066216364
Dennd5a	9.97008E-10	5.18E-50	3E-35	-0.031732411	-0.099978066	-0.066234124
Gm765	0.018135849	2.45E-55	8E-134	-0.013846053	-0.066293641	-0.066240666
G0s2	1.58029E-05	6.29E-54	3E-66	0.035543422	-0.085818461	-0.066267728
Brap	2.11011E-57	3.39E-38	6E-18	-0.096102863	-0.130789904	-0.06639686
Cdc42ep4	7.62886E-51	8.72E-21	4E-51	0.065080118	-0.059750149	-0.066402703
Cd81	1	6.28E-42	3E-75	-0.010763168	-0.068829431	-0.066468694
Grb7	0.000199493	3.15E-48	2E-55	0.022339921	-0.087040943	-0.066512304
Rab6a	0.005877394	1.23E-27	2E-33	-0.022153522	-0.079499526	-0.066533117
Tet3	5.56563E-14	1.00E+00	3E-23	-0.043420634	0.048051144	-0.066550733
44449	1.09056E-09	6.57E-46	7E-67	-0.024691076	-0.077678742	-0.066614001
Mup21	1	3.71E-38	9E-126	0.004608738	-0.056419961	-0.066760379
Ces2a	7.12769E-07	3.28E-52	7E-74	-0.021018839	-0.080878302	-0.066855824
Cyp2j8	1	1.26E-48	4E-117	-0.004304593	-0.066098316	-0.066916768
Spns2	1.46979E-16	6.86E-83	6E-84	-0.027164121	-0.095481173	-0.06695428
Inhba	3.53596E-28	5.86E-48	4E-102	-0.032812155	-0.068736156	-0.067086984
Zc3h6	9.84629E-09	1.00E+00	2E-46	-0.027292748	-0.003300949	-0.067209604
Sdccag8	1	1.52E-06	9E-20	-0.011519832	-0.058609023	-0.067522503
Nostrin	1	1.79E-39	2E-51	0.014394431	-0.079228517	-0.067589269
Rasgef1b	3.28311E-46	1.00E+00	2E-47	0.064547864	0.02724137	-0.067614304
Sod1	3.13182E-14	8.70E-09	9E-34	-0.038510365	-0.042475762	-0.067628388
Stx16	1	1.98E-25	7E-29	0.001842247	-0.07875749	-0.067636194
Txnrd2	1	3.06E-24	2E-59	-3.42231E-05	-0.061371715	-0.067710908
C1rl	1	3.87E-35	3E-33	-0.003370184	-0.095482278	-0.06771575
9430038I0	0.000187703	2.38E-52	4E-77	-0.01829731	-0.079953122	-0.06781562
Bco2	0.062325035	3.16E-52	5E-132	-0.011573015	-0.06498794	-0.067873366
Wac	1	6.02E-01	2E-22	-0.023872398	-0.024744504	-0.067963707
Tpst2	1	1.97E-13	3E-34	0.001885649	-0.055460585	-0.068011154
Ctsh	1	6.54E-15	6E-32	0.008098326	-0.056052851	-0.068036786
Cyld	3.48795E-05	6.83E-22	4E-54	-0.022468032	-0.058362496	-0.068063537
Rassf3	2.857E-84	4.17E-23	2E-20	-0.1156172	-0.096283656	-0.068149157
Flnb	1	1.00E+00	3E-21	0.001212418	0.000556661	-0.068384573
Ppp1r21	2.92237E-05	9.27E-19	3E-47	-0.022906338	-0.057258526	-0.068478039
Rheb	0.188897122	8.62E-18	2E-40	-0.017383487	-0.062010719	-0.06860872
Mllt3	1.71056E-09	1.21E-45	3E-40	-0.032055167	-0.092123006	-0.068664473
Pdp2	0.000495484	8.25E-53	1E-107	-0.015164168	-0.071638009	-0.068735262
Hebp1	1	1.00E+00	5E-20	-0.003787133	-0.005296202	-0.068806408
Hhat	5.3476E-71	7.07E-49	3E-39	-0.074467202	-0.101456507	-0.068814908
Sltm	1	1.00E+00	7E-23	0.015841565	-0.00126777	-0.068823865
Mapk1ip1l	1.36536E-16	2.40E-47	8E-33	-0.042434747	-0.109079819	-0.068884431

Rara	0.000428768	2.63E-25	1E-106	-0.016047961	-0.047448952	-0.068904414
Siah2	9.03335E-06	2.60E-41	8E-78	-0.019317144	-0.07240942	-0.069068939
Neo1	3.57858E-11	2.45E-59	2E-30	-0.036540567	-0.128545988	-0.069084589
Skil	1	1.83E-06	2E-71	0.001549594	-0.026470087	-0.069175561
Ncoa4	0.004131853	3.90E-36	3E-37	-0.022994535	-0.091380164	-0.069237451
D430042O(	5.40144E-09	3.68E-33	7E-33	-0.032729126	-0.092856496	-0.069255679
Arrdc3	0	2.90E-38	8E-69	0.212025143	-0.072431529	-0.069271629
Atg16l2	0.003542486	2.43E-27	2E-56	0.021791661	-0.067016155	-0.069288329
Slc12a9	1	3.27E-50	2E-87	0.003073809	-0.075136325	-0.069306439
Ube2e3	3.91729E-06	4.18E-34	2E-50	-0.024048888	-0.079061339	-0.069334633
Btg1	1	9.85E-46	3E-96	-0.010717984	-0.065760659	-0.069389982
Crkl	1	1.37E-27	1E-39	0.00168017	-0.077803683	-0.069410578
Coq10a	1	5.94E-53	7E-90	-9.23334E-05	-0.075600396	-0.069419093
Dhfr	4.40664E-55	1.19E-50	4E-64	-0.053962015	-0.087294827	-0.069604864
Kdm2a	1	8.63E-02	1E-23	-0.008885909	-0.026186269	-0.069721249
Sh3rf1	1.21541E-16	1.93E-29	2E-58	-0.032977456	-0.065489891	-0.069792191
Grk3	2.73702E-13	3.36E-35	3E-109	-0.02551191	-0.055087091	-0.069994381
Arntl	6.00469E-08	5.43E-15	2E-22	0.038281778	-0.075944354	-0.070181371
Cep85l	0	3.73E-56	1E-19	0.278698455	-0.172801655	-0.070251942
Masp1	5.52304E-08	6.72E-64	2E-22	-0.041987314	-0.168081041	-0.07028312
Fahd1	1.13336E-09	2.29E-56	1E-81	-0.022992365	-0.083199257	-0.070291136
Tspan33	0.015102778	1.79E-54	2E-120	-0.013593093	-0.070717825	-0.070302644
Dedd	1	8.46E-32	1E-50	-0.005432158	-0.07735951	-0.070390389
Slc35e3	1.78019E-06	6.99E-54	7E-112	-0.017894236	-0.071914593	-0.070412484
Stk24	1	3.48E-12	1E-29	-0.013573355	-0.057073705	-0.070452341
Nhej1	1	1.12E-47	9E-79	-0.00066299	-0.077819313	-0.07051019
Btbd7	1.79527E-09	2.29E-01	9E-25	-0.040974346	-0.02811216	-0.070539206
A330023F2	0.000373383	9.79E-05	4E-24	-0.031796545	0.069080951	-0.070542929
Sort1	9.831E-67	2.70E-44	3E-67	-0.060280486	-0.080903981	-0.070543742
Syne3	9.71526E-15	1.23E-34	1E-97	-0.026471429	-0.062620575	-0.070628747
Tent2	7.80657E-06	2.07E-19	2E-34	-0.027982431	-0.066423585	-0.070673921
E130307A1	4.12001E-06	7.32E-39	1E-104	-0.019163351	-0.062385092	-0.070825494
Tnfrsf1b	1	3.50E-10	8E-38	-0.008551901	-0.042740816	-0.070928978
Gm10644	0.000101033	5.86E-44	4E-107	-0.016062234	-0.067454645	-0.070932101
Man2a2	2.37476E-12	1.59E-17	5E-78	-0.027364667	-0.045734324	-0.07093645
Map2k6	8.80395E-58	6.52E-12	2E-43	-0.071728422	-0.056805656	-0.071339277
Tle2	1	6.36E-63	4E-121	0.007562509	-0.075787559	-0.071367792
Erf	6.91986E-34	2.39E-34	5E-95	-0.037174896	-0.063786594	-0.071386713
AU040320	1	8.22E-47	3E-34	-0.016312835	-0.110858932	-0.071436595
Cisd2	0.005640587	1.87E-40	4E-62	-0.018487875	-0.079591621	-0.071514543
Tnks	5.43431E-05	1.65E-09	9E-24	-0.032120613	-0.064308942	-0.071653669
Rc3h1	1	1.00E+00	5E-26	0.004346578	-0.021600919	-0.07177801
Bid	1	2.74E-33	1E-134	-0.004913938	-0.052443973	-0.071889867
Tcim	1	1.15E-62	1E-137	0.001208786	-0.072675773	-0.072100434
Arl13b	1	7.08E-48	1E-82	-0.00677082	-0.078854295	-0.072126718
Car14	7.70078E-41	9.45E-73	9E-178	-0.032180931	-0.071765643	-0.072209867
Morf4l1	1	7.55E-22	6E-33	0.013399305	-0.076696258	-0.072213999

Pex14	9.71197E-06	7.97E-30	2E-25	-0.043907398	-0.098570096	-0.0722485
Pm20d1	1.51553E-10	3.62E-54	1E-52	-0.028763128	-0.10013814	-0.072286557
Cdk13	1	1.00E+00	2E-24	-0.018304546	0.007501586	-0.07247166
Qrich1	1	1.41E-42	1E-30	0.003133248	-0.111149622	-0.072507422
Rab7	1	1.00E+00	5E-28	0.001257563	-0.021948472	-0.072707018
Fam47e	0.000775452	2.70E-36	2E-125	-0.015840178	-0.061650549	-0.072769084
Flrt1	3.82251E-15	3.62E-40	2E-119	-0.024059604	-0.0643389	-0.072786021
Zmynd11	1.66563E-13	1.00E-04	1E-24	-0.045849694	-0.04775094	-0.072840675
Gm19696	1	2.75E-66	6E-119	-0.009609536	-0.07964116	-0.072885587
Dock5	1.19793E-23	1.31E-12	2E-23	-0.061676413	-0.07198576	-0.072974203
Rps6ka5	0.000347564	1.07E-30	7E-79	-0.019503529	-0.062009476	-0.07301924
Adgrv1	0.02535433	1.00E+00	1E-112	-0.01455317	0.009113	-0.073094337
Naa50	4.45026E-11	1.59E-18	6E-62	-0.028495521	-0.056422619	-0.073133867
Mccc2	1	2.44E-47	1E-38	-0.007094113	-0.108558881	-0.073143475
Slc15a4	2.44268E-12	2.13E-11	1E-29	-0.042513386	-0.058447917	-0.073182474
Ttc19	1	5.63E-22	6E-40	0.00854876	-0.075141319	-0.073197534
Bri3	0.358604663	1.00E+00	6E-35	0.021552244	0.012772297	-0.073220461
Snap23	1	2.35E-29	1E-51	-0.008976512	-0.070501352	-0.073320482
Sdhaf3	0.000206811	2.18E-44	8E-44	-0.023800241	-0.100710684	-0.073322512
Mpdz	1.07356E-24	8.12E-70	6E-88	-0.035421167	-0.091272274	-0.073367675
Kif1b	5.01563E-24	9.06E-16	9E-28	0.06510106	-0.072154095	-0.073402339
Crk	1	5.64E-12	7E-46	-0.004150227	-0.048408697	-0.073427331
Fads1	0.002064695	2.42E-69	3E-114	0.017612999	-0.083787057	-0.073496268
Slc25a20	1	8.35E-65	1E-23	-0.020096005	-0.170020885	-0.073651154
Mipol1	3.66861E-05	9.33E-68	1E-30	-0.033351039	-0.144797629	-0.07379398
Appbp2	1	2.60E-27	4E-44	0.005454558	-0.074687742	-0.073864119
Shld1	1	5.22E-12	1E-60	-0.012509795	-0.048378111	-0.073868575
Prlr	2.7991E-122	2.51E-266	1E-10	-0.215429767	-0.471376826	-0.073971892
Usp6nl	6.343E-220	2.11E-21	2E-23	-0.193563165	-0.091663417	-0.074145282
Sin3a	1	3.28E-29	1E-44	0.002122761	-0.080932401	-0.074208339
Steap4	0.002688062	1.34E-23	2E-114	-0.020857453	-0.047378022	-0.074238364
Bcl3	1.16306E-05	1.35E-21	3E-121	-0.017752342	-0.045987286	-0.074242967
Tpcn1	5.5618E-44	1.79E-31	8E-69	-0.051511279	-0.071881424	-0.074305826
Bcl2l13	0.999798035	3.90E-43	3E-44	-0.017293696	-0.099892254	-0.074363009
B430010I2	5.87495E-12	3.88E-09	4E-51	-0.03208155	-0.046406933	-0.074371708
Nisch	0.018229918	2.99E-16	3E-26	0.025343244	-0.071243477	-0.074415589
Atp8b1	0.009466003	6.24E-63	4E-34	-0.026415848	-0.143643034	-0.074455539
Ap3b1	0.008361694	1.07E-03	3E-25	-0.036529584	-0.030013535	-0.07450213
Gask1a	5.90952E-64	3.21E-73	2E-139	0.058633868	-0.081169403	-0.074605454
Bik	1	5.24E-36	3E-104	-0.00243815	-0.065712444	-0.074710199
Inpp5f	1	2.16E-58	1E-39	0.011835072	-0.121484723	-0.074801158
Ddx6	1	3.33E-02	2E-31	0.002539649	-0.029903116	-0.07481149
Camk2g	0.021976947	3.37E-45	3E-53	-0.018948795	-0.094011355	-0.074844295
C130074G1	1	6.21E-35	3E-65	-0.002028642	-0.078771124	-0.074938597
Cish	0	1.47E-68	1E-149	-0.083138779	-0.076441728	-0.075018153
Tars	1	1.07E-47	9E-55	0.007893898	-0.098247086	-0.075087768
Zmiz1	0.000235119	1.00E+00	4E-20	0.031611117	0.021949949	-0.075092734

Acvr2b	3.12104E-31	1.03E-43	3E-110	-0.036132797	-0.070507979	-0.075202831
Hadha	1	4.89E-28	3E-30	0.00124446	-0.099066134	-0.075249203
Cutal	1	6.69E-47	5E-82	-0.006342813	-0.08246607	-0.075391834
Prkaa2	2.25542E-42	6.58E-02	2E-26	-0.080418687	-0.030595433	-0.075473522
Sgcz	1	5.63E-45	5E-95	-0.017010203	-0.076508483	-0.075514753
Rab14	0.303653435	1.95E-42	1E-36	0.023363661	-0.105195855	-0.075527161
Slc11a2	0.955300809	3.97E-25	5E-84	-0.013648568	-0.059000921	-0.075712242
Mapk1	1	4.58E-26	6E-35	0.003715194	-0.086663052	-0.075724323
Decr2	1.76829E-08	2.43E-66	2E-35	-0.033532475	-0.136986636	-0.075792749
Afg1l	1	4.50E-38	2E-34	-0.013511195	-0.106023035	-0.075842367
Mgat1	1	5.95E-31	2E-63	-0.00201144	-0.071573886	-0.075989504
Hexim1	1.34588E-16	3.13E-60	4E-130	-0.02546407	-0.07721271	-0.076116263
Tecpr2	1	8.34E-30	4E-43	0.013162366	-0.08577101	-0.076205766
Mbd2	3.55971E-15	4.90E-13	3E-49	-0.038998954	-0.049666549	-0.076290329
Gstt1	0.022160498	1.97E-39	2E-119	-0.014520672	-0.066091873	-0.076318922
Prkar2a	1	1.36E-22	4E-47	0.006776483	-0.070898464	-0.076430849
Pspc1	1	8.34E-22	1E-46	-0.00671811	-0.068413025	-0.076434767
Fermt2	1	4.13E-53	2E-26	0.013512202	-0.145599441	-0.07658813
Pnldc1	1.93704E-21	9.45E-60	3E-67	-0.039029216	-0.100871821	-0.076597232
Golph3	2.55727E-16	1.85E-35	1E-58	-0.036543246	-0.082956131	-0.076628023
Dnajc19	0.000757145	3.91E-18	6E-61	-0.022093258	-0.059382661	-0.076933764
Ube2w	0.028893573	1.86E-36	5E-45	-0.020954224	-0.093545898	-0.076937114
Herc3	1	4.41E-30	2E-97	-0.01070045	-0.060623046	-0.076974242
Pttg1ip	1	1.84E-53	2E-93	-0.010658212	-0.083544696	-0.077203626
Fbxo11	0.001036816	1.00E+00	6E-26	-0.030415301	-0.007445412	-0.077221588
Ppp2ca	7.26103E-30	3.43E-26	6E-39	-0.056518344	-0.083874496	-0.077361532
Gm35164	6.6962E-121	2.11E-80	2E-186	-0.053172652	-0.078709547	-0.07744738
Tex14	8.5069E-182	2.02E-15	2E-33	-0.145826205	0.108305779	-0.077716134
Smagp	1	3.21E-50	7E-148	-0.004863806	-0.068658	-0.077803898
Gnas	0.144384522	1.53E-10	3E-37	-0.021317932	-0.052486356	-0.077822758
Dhcr24	1.58641E-26	5.89E-183	1E-36	0.066123794	-0.253608289	-0.077886384
Atg5	9.32828E-10	2.09E-64	2E-33	-0.037689925	-0.144717756	-0.077900872
Tbl1x	2.46959E-56	1.00E+00	2E-28	-0.091350517	-0.020573917	-0.078020698
Gatad2a	1	1.01E-20	8E-38	0.014290471	-0.07421449	-0.07803752
H2-Q10	1.05229E-42	1.14E-227	2E-22	0.083561689	-0.322077388	-0.078076704
Gm13944	1	1.73E-21	1E-132	0.010428418	-0.05038394	-0.078097192
Khdrbs3	1.25787E-18	2.73E-80	2E-49	-0.041269831	-0.134047132	-0.078138088
Atf5	1	3.70E-67	2E-40	0.019817567	-0.140813097	-0.078185369
Cgn	1	9.27E-53	9E-68	0.000415584	-0.102390057	-0.07819793
Bmt2	2.07328E-10	8.09E-46	4E-46	-0.033108645	-0.103332059	-0.078299927
Spast	1	3.32E-36	1E-49	0.009330567	-0.090814601	-0.078368105
Lipa	0.000768861	1.28E-07	2E-62	0.024922495	-0.022924805	-0.078455014
C330002G(	1	1.37E-39	2E-139	-0.00843181	-0.06386995	-0.078494289
Mastl	2.33187E-58	2.98E-56	8E-120	-0.047530224	-0.079280293	-0.078522435
Whamm	1	4.47E-75	8E-50	0.006668827	-0.131573501	-0.078775094
Arf4	0.001333655	3.12E-28	3E-50	-0.021572427	-0.078256559	-0.078830195
Trim44	4.01724E-05	5.80E-23	2E-42	-0.025809941	-0.075157176	-0.078884368

Pcmtd1	3.14936E-36	1.20E-03	8E-28	-0.080866343	-0.038457895	-0.078961852
Nck1	1	6.04E-48	8E-40	-0.007039886	-0.11446442	-0.079002301
Sorbs2os	3.43824E-14	9.00E-02	1E-51	0.035841414	-0.021309033	-0.079003536
Wrnip1	0.046584242	1.33E-62	2E-117	0.015635664	-0.083675607	-0.0790152
Asb13	5.41258E-09	3.33E-22	5E-60	0.029794214	-0.067883513	-0.079022111
Cask	2.09453E-09	2.86E-33	2E-50	-0.033408007	-0.08468092	-0.079039641
St6galnac6	3.79227E-28	5.83E-76	3E-198	-0.028985331	-0.075567695	-0.079196934
Ireb2	1.285E-34	1.71E-25	6E-33	-0.068358662	-0.090644169	-0.07966831
Luzp1	0.001599158	4.43E-40	5E-47	0.026056372	-0.094697076	-0.079774314
Cradd	1	1.00E+00	9E-23	-0.02335074	-0.00826631	-0.079790105
Atg3	0.002157958	1.26E-21	4E-51	0.02524565	-0.070198071	-0.079823469
Arhgap23	1	4.70E-76	2E-73	-0.011905416	-0.107156094	-0.079862107
Eea1	1	5.34E-13	7E-33	-0.015322513	-0.064958324	-0.079863886
Ppp4r4	8.32304E-18	1.94E-81	4E-87	-0.033383607	-0.108360494	-0.079924727
Ttc6	1	5.79E-46	3E-93	-0.003548158	-0.0803283	-0.07999015
Canx	1	8.05E-56	5E-36	-0.008957472	-0.137026774	-0.080142679
Lmbrd2	3.21383E-09	6.50E-31	1E-47	-0.03151594	-0.088428838	-0.080377082
Cxadr	2.89309E-74	6.20E-45	1E-38	-0.102847891	-0.127788091	-0.080526654
Gm16157	1.23511E-32	1.77E-62	1E-53	-0.053931831	-0.11771796	-0.080561228
1810008I1	0.000191317	7.95E-57	4E-103	0.021942813	-0.086055097	-0.080652886
Ppp1r10	7.1372E-84	1.05E-15	3E-37	-0.095830979	-0.069880741	-0.080715477
Trim25	9.96566E-27	1.78E-14	2E-46	0.057388045	-0.051052011	-0.080736007
Pik3c2g	1	2.73E-97	8E-10	0.008432252	-0.258788724	-0.080745196
Gabarapl1	9.99399E-09	1.05E-24	1E-49	0.037310815	-0.075274093	-0.080810599
Ino80d	1	7.88E-18	8E-42	-0.005923225	-0.068893523	-0.080827883
Szrd1	1	1.08E-27	3E-59	-0.011512473	-0.076422044	-0.080832376
Fam222a	2.15778E-92	2.87E-43	4E-81	-0.072546215	-0.085427284	-0.080855832
Impa1	1.24085E-20	1.83E-54	4E-50	-0.045326523	-0.114513069	-0.080878023
Dnmt3a	2.48683E-72	4.19E-04	2E-30	-0.108801721	0.112477638	-0.080954205
Cbx4	0.000161665	1.16E-31	5E-130	-0.017405381	-0.060967068	-0.080979308
Umad1	3.17875E-20	3.28E-52	3E-34	-0.053996259	-0.130246786	-0.080996134
Prkacb	1	2.33E-62	2E-50	-0.017626994	-0.121117628	-0.081006241
Gm15638	1	3.84E-45	2E-105	0.002768215	-0.078406084	-0.08107561
Gm48877	1	6.56E-04	1E-105	-0.007150353	-0.02660234	-0.081076164
Nfatc3	3.50935E-05	2.83E-23	6E-40	0.033791356	-0.08105505	-0.08118214
Zfp512	0.052251266	5.35E-42	4E-73	-0.016622463	-0.08704477	-0.081213132
Abcg2	1	2.41E-20	3E-67	0.003745634	-0.064989016	-0.081310996
Tnks1bp1	0.000707013	2.90E-52	2E-132	-0.016232342	-0.076282831	-0.081317456
H2afv	1	2.73E-35	4E-99	-0.004486092	-0.071054729	-0.081332198
Bud13	1.05953E-25	3.22E-42	3E-75	-0.043671529	-0.085860609	-0.081386854
Rnf135	2.56915E-36	5.33E-34	3E-84	-0.046449612	-0.074277005	-0.081417813
Gm37240	1	1.00E+00	3E-36	-0.018274135	0.02108682	-0.081543671
Ei24	3.16769E-67	9.53E-48	3E-40	0.09532567	-0.121443384	-0.08163014
Homer1	4.63719E-07	1.32E-56	3E-71	-0.025368037	-0.10055388	-0.081638797
Pcbp4	2.39561E-05	6.76E-73	1E-129	-0.017244564	-0.089122304	-0.081702858
Dcun1d3	1	1.36E-35	1E-47	-0.01397366	-0.096142697	-0.081733189
Dipk1a	1.09961E-47	1.26E-38	4E-90	-0.053002934	-0.074147261	-0.081814501

Fam13a	1.4692E-133	1.66E-25	8E-37	0.173354306	-0.089047399	-0.081914454
Nap1l1	0.635821728	5.74E-32	2E-97	0.014403114	-0.066994753	-0.081935302
Wipi1	4.40715E-08	6.06E-72	2E-149	-0.019874672	-0.084282482	-0.082136763
Bmp2k	7.35846E-10	8.44E-19	9E-94	-0.027321494	-0.045046159	-0.082227687
Reep6	0.097455644	4.04E-57	5E-58	0.020160686	-0.111712186	-0.082298598
Gns	3.21656E-27	8.51E-15	2E-62	0.059230536	-0.03087542	-0.08241866
Atf1	1	1.34E-24	6E-44	0.010429161	-0.080881017	-0.08255921
Sh3bgrl	2.55516E-24	9.04E-73	4E-36	-0.061436898	-0.158023632	-0.082575032
Ethe1	2.3905E-143	1.75E-52	4E-145	-0.066763525	-0.075944985	-0.082604535
Timd2	2.34922E-06	2.11E-71	2E-47	-0.030250015	-0.136187007	-0.082607903
Aamdc	5.93059E-16	1.56E-43	1E-79	-0.034278922	-0.088437388	-0.082660143
Hmbox1	1	3.03E-48	1E-49	-0.001514839	-0.108285341	-0.082783777
Dcun1d1	2.4392E-126	1.51E-20	3E-32	-0.128341631	-0.089511046	-0.082913979
Arih1	1	1.00E+00	9E-32	0.015193217	0.017439007	-0.082972629
Msantd2	0.144295222	5.73E-30	5E-67	-0.017636939	-0.076393821	-0.083051443
Mapk8	1	8.38E-53	1E-59	-0.015319979	-0.107467462	-0.083105222
Snx3	1	3.18E-36	2E-63	-0.012610186	-0.086504354	-0.083143596
Cpeb3	1.20235E-27	1.76E-02	1E-27	-0.075506338	-0.035744161	-0.083264846
Sgsm2	4.58158E-23	1.94E-48	3E-102	-0.03608386	-0.082076696	-0.083329061
Sap130	5.05652E-13	2.91E-66	1E-43	-0.040197275	-0.138965337	-0.083549125
Avl9	0.003906076	5.06E-22	1E-57	-0.021266561	-0.070475652	-0.083594975
Rcor1	2.18829E-05	1.20E-13	4E-32	-0.032863745	-0.079830371	-0.08364308
Foxa3	8.629E-27	4.29E-53	7E-52	-0.052933526	-0.116452653	-0.083684311
Mb21d2	2.80116E-81	7.29E-10	4E-42	-0.104687778	-0.033860166	-0.083693153
Gm47431	2.62718E-14	3.42E-55	2E-168	-0.026010933	-0.07429726	-0.083705636
Akap8l	1	2.98E-34	4E-33	0.022057923	-0.113092527	-0.084025227
Rtf2	5.25146E-05	2.84E-61	7E-49	0.031143979	-0.12635325	-0.084176551
Kidins220	7.36044E-09	1.08E-14	8E-43	-0.035516742	-0.060727262	-0.08425536
Zfp710	5.60492E-28	1.00E+00	2E-67	-0.047813528	0.019449208	-0.084362087
Cpn2	0.701718204	8.90E-67	2E-139	-0.012762019	-0.086247703	-0.08444461
Dtnb	3.81265E-45	2.65E-02	3E-27	-0.091566037	-0.042121857	-0.08459318
Mlycd	1	6.16E-19	2E-78	-0.001216392	-0.059712304	-0.084763282
Abcb10	1	1.78E-54	1E-98	-0.004213264	-0.090088385	-0.084786249
Casd1	1	9.08E-40	2E-55	-0.014906823	-0.097030534	-0.084812034
Gapvd1	0.439994261	1.00E+00	1E-35	0.023921146	-0.000821903	-0.084903332
Mthfd1	1	5.30E-103	4E-32	0.017714985	-0.204886816	-0.085018571
Fbxw7	0.000621239	3.31E-12	3E-46	-0.026289526	-0.058014756	-0.085181908
Epc2	0.053080365	1.50E-42	2E-57	-0.019557084	-0.098208437	-0.085253743
Pcdh1	1	1.44E-21	6E-97	0.003442474	-0.060269474	-0.085272716
C4a	5.52807E-68	4.90E-25	2E-149	-0.05053347	-0.049137761	-0.085315792
Sgk2	1.24739E-19	1.07E-25	2E-47	0.056492947	-0.087016847	-0.085370903
Ero1lb	1	3.53E-63	1E-39	0.007663871	-0.143424363	-0.085376061
Rin3	3.74819E-08	7.09E-09	3E-54	-0.031238583	-0.040544787	-0.085655554
1810013L2	6.70899E-23	4.56E-08	3E-52	-0.049308042	-0.042479676	-0.085764
Ccdc138	1.82442E-19	9.73E-59	2E-94	-0.034872173	-0.095343167	-0.085859774
44258	0.003621859	6.79E-38	8E-54	-0.025689509	-0.093557826	-0.085986786
Eri3	1	3.95E-10	4E-37	-0.003107773	-0.067120922	-0.086010341

Elk4	1	6.16E-39	7E-52	-0.013460452	-0.099067761	-0.086015815
Braf	1	7.67E-33	3E-40	-0.004714232	-0.100412354	-0.086219165
Abl1	0.099660656	4.77E-54	1E-37	-0.02265143	-0.139384828	-0.086236075
Cat	1.88672E-92	1.70E-115	3E-24	0.112579701	-0.251286404	-0.086367894
Amy1	0.068136016	3.75E-61	3E-48	-0.022055329	-0.130571203	-0.086485792
Hopx	4.40289E-25	9.19E-50	2E-169	0.037640309	-0.072304287	-0.08661787
Prdx1	2.03034E-56	2.37E-02	9E-40	-0.086936495	0.064156675	-0.086701637
Camta2	1	1.92E-81	2E-62	-0.011273704	-0.132284633	-0.086709718
Klf15	6.50954E-06	1.86E-59	5E-40	0.031169969	-0.139361116	-0.086838596
Phf8	3.46001E-18	1.26E-32	6E-50	-0.046176206	-0.094341332	-0.087051109
Ubl3	1	5.73E-22	3E-53	-0.012418187	-0.070328058	-0.087060179
Sar1b	3.59223E-73	9.98E-63	7E-40	0.106023255	-0.143778226	-0.087088806
Txndc11	1	2.21E-80	2E-29	-0.006179917	-0.188381517	-0.08713368
Adgra3	1	8.88E-62	8E-74	-0.008671655	-0.110594401	-0.087218173
Proc	1	1.60E-35	8E-33	0.02054924	-0.124880582	-0.087259111
Cep350	1	1.25E-04	1E-37	-0.01425994	-0.036453598	-0.087330369
Slc22a18	5.23053E-14	1.37E-46	3E-59	-0.037266995	-0.107786748	-0.087421528
Klb	5.00828E-13	8.45E-151	6E-66	0.033890207	-0.179528917	-0.08747961
Dusp1	7.79199E-15	1.17E-32	9E-157	-0.025168618	-0.055537197	-0.087633947
Phyhd1	1.00067E-08	2.44E-47	7E-50	-0.033262583	-0.113827462	-0.0876846
Gm16124	1	5.93E-15	1E-85	-0.00197355	-0.046138669	-0.087793469
Ddx60	3.68173E-12	1.42E-17	1E-155	-0.027404387	-0.02919674	-0.087883766
Sarnp	1	5.72E-55	3E-52	-0.00210911	-0.121103038	-0.088029485
Smim14	0.001204973	1.88E-37	6E-49	0.032343023	-0.102313339	-0.08804558
Prr14	1	3.30E-42	1E-84	0.000822167	-0.087130996	-0.08808821
Serpina10	3.46565E-14	2.46E-65	2E-101	-0.030974688	-0.10002217	-0.088143108
Ildr2	1	9.35E-69	2E-167	0.006182884	-0.086024827	-0.088281534
Golga4	5.59238E-09	5.08E-19	5E-37	0.040580395	-0.07570414	-0.088297766
Zfp91	1	3.27E-23	2E-49	0.004314938	-0.080197138	-0.088388954
Paxbp1	1	3.83E-58	6E-47	0.005637467	-0.127014789	-0.088584694
Hip1r	4.96647E-14	2.78E-43	1E-52	-0.039204689	-0.109193547	-0.088616685
Ccdc50	1.5821E-12	1.79E-61	2E-42	-0.042530488	-0.143957174	-0.088824108
Cfhr1	3.91513E-14	1.00E+00	1E-49	-0.042613908	-0.012497415	-0.089048857
Tfeb	1	6.18E-06	2E-87	-0.008477432	-0.024570173	-0.089099596
Cda	3.24094E-24	3.00E-66	2E-54	-0.056322572	-0.134493334	-0.089226322
Hpd	4.38558E-40	4.37E-144	1E-20	0.093803798	-0.304960577	-0.089259157
Gm45425	1	1.86E-96	1E-153	-0.007057398	-0.104608512	-0.089394086
Mtmr3	1	1.00E+00	1E-40	-0.021417895	-0.012979972	-0.089452298
Mbd1	5.10041E-12	9.22E-16	5E-17	-0.060884314	-0.099801949	-0.089469649
Apol9a	2.30185E-64	1.94E-60	2E-180	-0.046887353	-0.079098251	-0.089491545
Memo1	1	8.30E-52	1E-55	-0.01041172	-0.115006256	-0.089514293
Gm12940	1.52473E-22	1.56E-70	4E-128	-0.034485844	-0.097183166	-0.089671322
Fam114a1	6.02687E-13	9.98E-50	6E-92	-0.032619425	-0.087638815	-0.089714886
Sik1	1.79438E-82	1.31E-64	1E-162	-0.052557703	-0.083928894	-0.089748862
Rb1	1	7.95E-09	4E-78	-0.00240031	-0.03542798	-0.089771725
Ralgps1	7.74312E-14	1.13E-70	3E-56	-0.038337452	-0.139137292	-0.089972835
Fbxo3	4.18649E-05	4.26E-41	2E-52	-0.028257319	-0.106099275	-0.090091782

Dennd4a	5.53624E-08	1.20E-06	9E-36	-0.042431318	0.087277314	-0.090386176
Papola	1	6.49E-03	1E-39	0.001726632	-0.031103584	-0.090444662
Ifrd1	0.001254191	6.25E-34	8E-56	-0.02650425	-0.09234721	-0.09045847
Gm20732	3.81015E-10	6.48E-21	4E-71	-0.03207734	-0.067185982	-0.090541609
Pcnx	0.037683254	8.65E-38	2E-50	-0.022570819	-0.102351234	-0.090542367
Arnt	2.38611E-08	9.87E-30	3E-41	-0.038327937	-0.103908114	-0.090666254
R3hdm1	1	1.93E-12	5E-39	-0.005551058	-0.071256592	-0.090759688
Tgoln1	1	3.63E-30	2E-96	-0.00448707	-0.072485286	-0.09079567
Acsl5	1.06435E-17	2.47E-45	1E-54	-0.050316961	-0.116354882	-0.091035584
Bbox1	3.99474E-54	1.23E-84	7E-42	0.093454631	-0.174847743	-0.091122115
Tec	1	1.75E-13	1E-37	0.009993686	-0.074602306	-0.09113
2310015A1	2.22243E-17	4.01E-39	4E-135	-0.031511693	-0.073460595	-0.091144637
Prdm2	5.82663E-70	1.19E-04	5E-37	-0.110413034	-0.042284167	-0.091151354
Akr1d1	1	2.51E-244	2E-59	-0.013577345	-0.295363679	-0.091152367
Lnx2	5.4866E-228	1.35E-09	6E-34	-0.198668091	-0.070524888	-0.091300535
Cyp3a25	0	3.28E-196	1	0.5273421	-0.496755242	-0.091424797
Bcl9l	0.005198108	3.22E-68	2E-58	0.026230945	-0.135710776	-0.091629539
Fgb	1.18946E-47	4.21E-230	4E-37	0.097509319	-0.346031593	-0.091729111
Arhgef18	1.77009E-31	1.74E-57	5E-43	-0.065947755	-0.136847338	-0.092112437
Dop1b	1.5017E-142	1.87E-13	2E-24	-0.161727803	-0.088908581	-0.092167807
Slc17a8	1	8.68E-80	6E-58	0.012720325	-0.155127273	-0.092192534
Rab43	0.31595973	2.32E-21	2E-43	0.024966027	-0.087267248	-0.092196347
Tprkb	1	1.33E-75	3E-71	0.01009436	-0.130933027	-0.092230112
Il18	6.04577E-09	4.29E-64	5E-194	-0.020311209	-0.07816664	-0.092265428
Pdlim5	1.09591E-15	1.77E-13	1E-38	-0.054875278	-0.071530267	-0.092287206
Ergic1	1.06743E-13	1.10E-23	2E-56	-0.040472195	-0.07696738	-0.092291995
Pim1	0.043298538	1.66E-19	4E-142	-0.01478053	-0.044752868	-0.092311387
Irs1	1	4.28E-59	2E-42	-0.024666366	-0.15098855	-0.092385128
Gm826	3.34451E-05	7.15E-35	5E-116	-0.024562255	-0.076671685	-0.092478639
Upf3b	1	1.86E-75	8E-53	0.020334077	-0.147146541	-0.092649005
Trio	1.39023E-14	5.45E-68	2E-37	-0.055787753	-0.164485639	-0.092662698
Cntrl	2.71771E-19	6.75E-62	7E-54	0.05429599	-0.131964638	-0.092694117
Crebrf	4.27008E-37	1.17E-04	6E-43	-0.073969209	-0.044223264	-0.092818358
Zfp467	6.50139E-07	4.68E-71	3E-183	-0.019428241	-0.085408299	-0.092879162
Rap1b	2.90781E-10	1.13E-12	9E-104	-0.028393649	-0.03390886	-0.093094923
Foxj3	1	3.04E-40	3E-74	5.59127E-05	-0.093449628	-0.093194222
Elavl1	1.86918E-07	5.22E-54	3E-56	0.037039647	-0.123120854	-0.093235139
Dsc2	1.15931E-21	1.86E-33	3E-95	-0.041037852	-0.079358835	-0.093291184
Lrrfip2	0.000236344	1.00E+00	1E-40	-0.036292285	-0.014633892	-0.093430187
AU022252	1	2.50E-72	1E-96	0.007082492	-0.113947406	-0.093750195
Zfp976	5.06919E-11	2.00E-95	1E-74	-0.033335243	-0.144711869	-0.093862841
Ctdspl2	1	1.01E-40	3E-65	-0.009644733	-0.100740629	-0.093869045
Wipf2	1	1.57E-34	3E-69	-0.007657697	-0.090226082	-0.094209707
Csad	0.517967582	2.70E-21	8E-47	-0.023115494	-0.082243566	-0.094326273
Kmt2c	1	5.44E-08	3E-49	-0.012239657	-0.041395415	-0.094412579
Zbtb43	1	1.81E-56	9E-79	-0.003659824	-0.111454242	-0.094424842
Trp53bp2	1	1.84E-51	5E-85	0.008653416	-0.103457984	-0.094442504

Ubxn7	1	4.98E-33	4E-61	-0.011445528	-0.094910347	-0.095094568
Rnf38	3.59533E-21	2.08E-21	2E-54	-0.052322386	-0.07829993	-0.09537807
Tmem163	0.023492057	9.15E-66	1E-208	-0.013868007	-0.080754808	-0.095576855
Inhbc	1	5.08E-58	1E-137	-0.007465066	-0.092314495	-0.095682892
Galm	1.1512E-13	4.88E-56	2E-38	-0.049709186	-0.157198371	-0.095906817
Hnrnph3	1	6.32E-28	2E-69	0.012224223	-0.08361162	-0.095957268
Pcgf3	0.643937061	7.61E-39	2E-69	-0.017347365	-0.099439773	-0.096160083
Smarcc2	1	4.83E-43	7E-61	0.006939842	-0.108133578	-0.096169254
Marf1	0.007037874	4.39E-33	2E-44	-0.030215761	-0.111421222	-0.096246452
Kat6a	1	4.05E-43	1E-57	0.013263859	-0.108775419	-0.096310065
4930523C0	1.00178E-12	2.11E-47	3E-67	-0.037950331	-0.10985365	-0.096452612
Ppp1r13b	0.008475155	2.60E-70	4E-69	-0.022747747	-0.131311083	-0.096996453
Paqr7	2.95266E-41	5.46E-86	6E-203	-0.041301028	-0.095758723	-0.097076224
Lclat1	8.72775E-33	6.08E-79	5E-68	-0.058091396	-0.143525722	-0.097131757
Glce	0.158159305	1.34E-46	1E-65	0.021276877	-0.111580239	-0.097203316
Eci1	1	1.08E-72	8E-79	-0.013648548	-0.129977806	-0.097318617
Ube2i	1	3.07E-70	8E-64	0.014056983	-0.138414975	-0.097341151
Lypla1	1	6.93E-57	7E-51	0.015747297	-0.138345966	-0.097379595
1810026B0	1	6.80E-35	9E-80	0.000737427	-0.085580338	-0.097521942
Bhlhe40	8.82027E-10	7.57E-59	3E-196	-0.023310185	-0.080431069	-0.097758076
Ap1g1	1.43533E-08	2.66E-26	5E-47	0.04151607	-0.102047143	-0.097786707
Car1	3.46428E-80	4.21E-90	5E-242	-0.051293217	-0.092660703	-0.097794523
Arl8b	1	1.70E-42	2E-66	-0.011519092	-0.102968238	-0.097833446
4933406I1	4.05883E-32	2.56E-171	8E-76	-0.05797717	-0.210516883	-0.097894456
Tmc7	9.0961E-40	4.02E-89	5E-133	0.054433767	-0.115792153	-0.098033452
Phlda1	2.56197E-41	4.40E-74	3E-132	-0.050128561	-0.107979317	-0.098666598
Kdm3b	0.073280284	1.53E-23	6E-61	-0.021259956	-0.080368732	-0.098848705
Gm30849	6.38434E-18	1.08E-51	4E-141	-0.032742557	-0.087143995	-0.098916182
Zdhhc14	0.20869576	1.08E-15	3E-67	-0.022183514	-0.048776486	-0.099141359
Gtf2a1	1.07161E-15	2.71E-42	2E-95	-0.03667565	-0.090487397	-0.09920184
Amacr	0.000384319	3.43E-85	2E-89	-0.024091587	-0.133441861	-0.099218185
Slc27a5	1	1.17E-97	3E-101	-0.001018603	-0.135627267	-0.099378592
Bcorl1	1.90704E-18	1.23E-60	4E-131	-0.035388723	-0.097342697	-0.099550801
Snap47	2.51284E-11	1.18E-63	7E-93	-0.031943512	-0.115705813	-0.099730301
Tshz3	3.89966E-73	1.52E-85	2E-180	-0.05629256	-0.099483326	-0.099807337
Fam234a	1	2.13E-48	2E-77	0.000631371	-0.105173412	-0.09987845
Anks4b	1	7.57E-85	2E-117	-0.008575593	-0.119933566	-0.099949353
Tiam2	1.03784E-20	1.08E-68	7E-210	-0.033316617	-0.088701428	-0.099985392
Tcp11l2	2.28684E-22	2.18E-50	2E-90	-0.044833256	-0.103936435	-0.100006028
Zcwpw2	1	7.85E-82	4E-185	0.013800181	-0.099493319	-0.100365622
44261	1	7.92E-73	1E-53	0.006493541	-0.159834886	-0.100434666
Peli2	6.5238E-44	6.57E-76	4E-43	-0.085629288	-0.180668142	-0.100437206
Slc49a4	7.14693E-73	2.19E-28	7E-58	0.106499981	-0.095186458	-0.100568461
Ptch1	1	1.17E-25	1E-78	0.011198483	-0.081766571	-0.100736495
Pcbd2	1	2.56E-37	1E-60	-0.007472479	-0.108578527	-0.100822365
Nt5dc3	1	7.48E-61	1E-84	-0.009348376	-0.12073604	-0.100891014
Map3k3	1	3.13E-39	1E-77	-0.004156992	-0.09147458	-0.100893545

Ctnnd1	1	1.06E-06	1E-49	-0.020160924	-0.050955656	-0.101014452
Mettl7b	1.4214E-198	2.44E-87	4E-67	-0.141924186	-0.158807675	-0.10113181
Sfxn2	1	6.66E-69	3E-73	-0.014255142	-0.133374847	-0.101207355
P4ha1	1	3.84E-58	2E-99	-0.007311233	-0.104436664	-0.10126769
Phf20	1	8.26E-34	9E-76	-0.010398315	-0.088149672	-0.101328942
Ncor1	1	6.42E-23	2E-57	-0.022970237	-0.072962557	-0.101364975
Ubtd1	1	9.64E-31	7E-128	-0.015703779	-0.068599181	-0.101524852
Fkbp5	2.97603E-33	1.00E+00	1	0.088884942	-0.135886637	-0.101792241
Als2	1	1.01E-93	1E-54	0.015208354	-0.179903278	-0.101931961
Abhd14b	2.93707E-16	1.52E-62	4E-163	-0.030161691	-0.091676375	-0.101978115
Gc	1.5094E-263	2.13E-169	4E-21	0.216061051	-0.343164615	-0.102023454
Irs2	1.36021E-74	2.84E-29	4E-43	-0.115312809	-0.108755942	-0.102027752
Heca	4.44386E-08	3.36E-36	8E-78	-0.032739825	-0.095371113	-0.102058117
Proser2	2.71481E-58	2.80E-68	7E-73	0.087515336	-0.13533241	-0.102228336
Dcaf11	3.1697E-05	3.41E-121	2E-64	0.032981248	-0.191145391	-0.102316585
Stat5b	1.06314E-09	2.66E-18	4E-40	-0.050584915	-0.080753968	-0.102321734
Bmpr1a	1	2.77E-58	1E-53	-0.012846105	-0.143373193	-0.102400646
Gm47200	2.62189E-11	1.82E-56	6E-163	-0.025804246	-0.089981138	-0.102443472
Tmem266	1.05906E-10	5.89E-84	3E-187	-0.024261049	-0.102761984	-0.102647968
Glcci1	1	4.08E-70	3E-86	-0.00926132	-0.127094018	-0.10265165
Mn1	1	4.77E-45	9E-106	-0.011644635	-0.095395682	-0.102739207
Stag2	1	1.00E+00	8E-48	0.003399445	-0.019483243	-0.102950425
Ak3	1	2.27E-62	5E-84	0.015048799	-0.122468681	-0.103078369
Slc40a1	2.61228E-41	1.00E+00	9E-112	-0.056747451	0.016694937	-0.103116377
Spred2	1.3058E-05	1.94E-16	1E-50	-0.035863662	-0.079206327	-0.103191899
Gm16066	1.28286E-05	1.56E-52	3E-114	-0.024710127	-0.099052227	-0.103226147
Irf6	3.19705E-05	4.94E-82	3E-183	-0.01929087	-0.10304417	-0.103416444
St3gal4	1	2.93E-14	2E-32	-0.017321171	-0.09467477	-0.10361481
Fignl2	7.96119E-10	3.25E-83	2E-164	-0.024369281	-0.108554292	-0.103746103
Atl2	6.28412E-07	1.95E-99	2E-51	0.04073188	-0.195381238	-0.104014964
Fxr1	1	2.41E-84	9E-62	-0.000353996	-0.162795036	-0.104015274
Tmie	2.14152E-21	9.91E-54	2E-138	-0.037458823	-0.095014208	-0.104057355
Slc30a9	1	3.44E-46	2E-62	0.01579582	-0.119098561	-0.104114869
Abtb2	0.001136835	1.00E+00	2E-18	-0.045353054	-0.024855698	-0.104172853
Scd1	6.89636E-09	3.51E-20	1E-113	-0.030564889	-0.05563238	-0.104268764
Sephs2	1	1.48E-71	7E-92	-0.00555739	-0.126208556	-0.104335716
Kpna3	1	3.77E-28	3E-86	0.013268988	-0.083052439	-0.10448427
Rnf2	1.08479E-08	1.01E-69	5E-133	-0.026492496	-0.108008924	-0.104548389
Mtfr1	0.001153379	2.77E-62	2E-77	-0.024454912	-0.13005769	-0.104671239
Steap3	2.82634E-83	2.46E-64	3E-177	-0.06221032	-0.093854437	-0.10479386
Pcmt1	1	2.34E-37	4E-63	-0.018598347	-0.108600713	-0.104894783
Pms1	3.7264E-217	1.05E-66	3E-89	-0.129359053	-0.126267387	-0.105213612
Herc4	2.42739E-83	8.16E-03	8E-51	-0.120536342	-0.02651027	-0.105231206
Lasp1	6.04419E-09	2.35E-43	6E-175	-0.024750219	-0.076226733	-0.105262976
Fbxo9	1	1.19E-58	3E-78	0.008786626	-0.125731105	-0.105358315
Slco2a1	1.61084E-05	5.42E-99	6E-81	0.031965545	-0.159930011	-0.105387773
Ncald	3.74405E-07	9.63E-94	1E-215	-0.022031203	-0.103033818	-0.105504512

Helz2	1	4.64E-84	8E-110	-0.009301315	-0.126002244	-0.105564851
Chic2	1	7.99E-39	8E-79	-0.009038729	-0.100290865	-0.105837276
Ptpn2	1	1.45E-53	8E-68	0.005730222	-0.127784725	-0.105991475
Lap3	1.60683E-16	2.52E-72	3E-96	-0.038914626	-0.126113999	-0.106043767
Simc1	3.23512E-27	2.47E-34	2E-54	0.070066755	-0.116188899	-0.106084517
Ube2u	0.007097792	1.44E-112	4E-262	-0.015465541	-0.107248674	-0.106098023
Slc35e2	9.40006E-45	2.62E-127	2E-63	0.082177456	-0.202500364	-0.106189094
Mical3	4.3178E-114	7.00E-46	4E-48	-0.145070994	-0.136696428	-0.106237867
Inmt	5.93058E-32	7.81E-118	4E-283	-0.035326642	-0.106280272	-0.106305163
Fcgrt	1	5.97E-66	5E-192	-0.010094855	-0.092090917	-0.106312656
Trim28	1	1.83E-27	1E-37	-0.020807684	-0.120243057	-0.106381324
Fh1	4.9026E-101	3.75E-76	1E-96	-0.089826653	-0.130508089	-0.106458466
Slc9a3r1	1.2047E-35	1.04E-59	2E-198	-0.041689698	-0.086575825	-0.106491174
E230016M	3.91498E-60	2.49E-41	7E-158	-0.058950446	-0.0823788	-0.106711728
Gm40438	6.7835E-129	1.47E-64	2E-60	-0.142259075	-0.159737507	-0.106914224
Cnot2	1	4.90E-36	3E-64	-0.004135086	-0.107940367	-0.10694242
Rassf5	1	4.41E-76	1E-196	-0.004708661	-0.098195625	-0.107123154
Nfe2l1	1.94202E-12	7.85E-73	2E-77	-0.038657141	-0.142762798	-0.107525271
Arl6ip1	1.57615E-18	1.64E-71	8E-115	-0.039821337	-0.118252408	-0.107629944
Nucks1	8.3724E-117	4.67E-57	5E-73	0.12133303	-0.128432225	-0.107662138
Cd47	1	6.88E-60	5E-159	0.002839624	-0.089337259	-0.108198587
Prox1	1.01481E-90	4.68E-89	1E-41	0.140996968	-0.222352519	-0.108268769
Fbxl20	1	1.00E+00	9E-51	-0.005907047	0.00649052	-0.108614754
Ptp4a2	3.0052E-09	1.97E-13	5E-81	-0.035588623	-0.049721132	-0.10875066
Ocln	1.05141E-43	1.02E-64	4E-90	0.075962522	-0.129544944	-0.108853129
Mapk15	1	7.74E-52	1E-215	0.006632944	-0.078538081	-0.109100237
Tbc1d14	1	1.69E-41	1E-110	-0.014306624	-0.086363137	-0.109122039
Tpst1	1	1.11E-88	7E-179	-0.009472819	-0.112001959	-0.109431223
Slc25a32	2.15831E-06	9.74E-53	4E-97	0.0314419	-0.113337296	-0.109457685
Apobec1	2.3193E-16	2.69E-03	3E-149	-0.037323351	0.03824032	-0.109690309
Zswim5	1	2.71E-58	6E-133	-0.012638492	-0.105759797	-0.110015318
Tor1aip2	2.38105E-39	3.36E-36	9E-70	-0.069369417	-0.106269834	-0.110138034
Nat8f1	0.000141845	9.56E-87	4E-205	-0.020214387	-0.105784963	-0.110148614
Gm50237	1	1.00E-93	4E-175	-0.00612606	-0.117011002	-0.110220271
Ppfibp1	2.1294E-15	2.04E-50	5E-65	-0.049884389	-0.129695309	-0.110267013
Adh7	0.475025711	1.62E-62	2E-167	-0.015274154	-0.098737767	-0.110362285
Spaca6	1	7.45E-115	5E-269	-0.003934748	-0.109544534	-0.110565016
Tnpo3	1	1.20E-67	1E-74	6.40975E-05	-0.140386112	-0.11065015
Nectin2	1.18269E-75	4.75E-54	2E-168	-0.065325861	-0.093909576	-0.110680877
Reps2	2.44036E-86	1.63E-39	6E-56	-0.119346439	-0.124408718	-0.110773763
Rfx4	2.84827E-24	8.53E-104	4E-225	-0.035095436	-0.11327268	-0.110937042
BC028777	4.12399E-41	2.08E-50	2E-178	0.055809136	-0.08942589	-0.11095488
Mpc2	1	1.50E-65	6E-71	0.005263891	-0.144842451	-0.111204079
Mrpl18	1	1.43E-212	4E-65	-0.003491731	-0.275326358	-0.111250569
Slc31a1	1	7.05E-73	4E-97	-0.005067254	-0.134296632	-0.111252405
Pbld1	5.918E-14	3.27E-178	2E-40	0.045885427	-0.299691207	-0.111381283
Dyrk2	1	1.50E-79	6E-178	-0.006723491	-0.108737675	-0.112144682

Mfsd4b1	4.53025E-10	1.49E-75	2E-224	-0.024268435	-0.099161879	-0.112404198
Efna1	9.45767E-19	1.87E-30	5E-136	-0.03991281	-0.078752402	-0.112714204
Klhl24	0.005108255	1.65E-18	8E-91	-0.022467818	-0.068239679	-0.112876491
Gpcpd1	4.0619E-176	6.55E-07	6E-49	-0.187571164	-0.063367788	-0.112934949
Noct	1	1.50E-25	2E-50	-0.014171693	-0.114144565	-0.113146172
Nr2c2	1	2.15E-09	4E-76	0.001976009	-0.054411616	-0.113162232
Slc25a15	2.1997E-114	5.57E-141	9E-58	-0.13607611	-0.242226195	-0.11335143
Sipa1l3	0.000296525	3.23E-58	9E-88	-0.024744529	-0.12094931	-0.113391777
Yy1	1	1.05E-42	5E-82	-0.016817558	-0.110552602	-0.113471903
Slc25a33	1	3.36E-10	2E-125	0.002798568	-0.044377829	-0.113570686
Abhd15	1.34952E-11	2.93E-47	2E-128	-0.033865318	-0.098373998	-0.113713677
Nek7	7.23316E-12	2.30E-07	5E-59	-0.052376959	-0.048841258	-0.113863236
Dazap1	1	2.12E-42	8E-81	-0.001753034	-0.114094878	-0.113923882
Heatr6	1	7.92E-64	1E-96	0.00160951	-0.128437415	-0.114112715
Ric8b	1	1.02E-70	5E-94	-0.012339272	-0.137723634	-0.114383915
Serinc5	3.09698E-39	3.19E-62	8E-130	-0.055864086	-0.113858019	-0.114411294
Acsf2	3.72765E-71	1.19E-35	1E-62	-0.103474878	-0.118193666	-0.114623273
Ap3s1	1	1.03E-39	9E-94	-0.010559677	-0.102787096	-0.114652603
Macrod2	0.625229461	2.18E-47	1E-100	-0.023714617	-0.111189807	-0.115121494
C1ra	3.60895E-22	2.64E-94	9E-209	-0.035648146	-0.115164193	-0.115165784
Ppp2r1b	8.31785E-40	3.29E-94	2E-209	-0.045740456	-0.114871744	-0.115204808
Enpep	4.21327E-19	9.96E-84	2E-249	-0.033239811	-0.102471157	-0.115302322
Ormdl3	1	4.20E-83	2E-172	-0.005238581	-0.116763437	-0.115384063
Zyg11b	1.84019E-12	4.96E-62	2E-95	-0.038436486	-0.129031656	-0.115438012
R3hdm2	1	1.39E-19	2E-62	-0.004266399	-0.084404013	-0.115448011
Bmpr2	0.993762382	4.48E-28	8E-85	-0.019631834	-0.08499143	-0.115470911
Nid2	0.738429145	9.05E-123	6E-280	-0.012630538	-0.117249442	-0.115982979
Rgn	1	4.06E-107	6E-218	0.006117599	-0.119693499	-0.116036479
Gfod2	2.0825E-06	2.37E-37	1E-111	-0.028924386	-0.098705395	-0.116223479
Rhot1	1	9.52E-74	3E-74	-0.015447033	-0.160604559	-0.116263342
B130055M	1	2.85E-81	1E-197	-0.001461766	-0.110177792	-0.116403984
Hrg	0.0043342	5.70E-52	3E-102	0.039874898	-0.118248971	-0.116741516
Hsp90ab1	1.25624E-32	2.95E-69	3E-71	-0.067677946	-0.154573932	-0.116895442
Wdr33	1	5.04E-19	1E-68	-0.020218352	-0.080291529	-0.116924552
Senp5	1.84556E-06	1.37E-54	3E-76	-0.032701855	-0.133492383	-0.116991098
B4galt1	7.7881E-181	3.91E-62	4E-64	-0.158568519	-0.15135887	-0.117069592
Tspan14	7.49655E-30	2.51E-46	2E-137	-0.04958881	-0.092844559	-0.117118766
Iigp1	2.6827E-150	8.10E-34	6E-66	-0.145869808	-0.107579995	-0.117240269
Ddhd1	1	1.97E-21	4E-105	0.01360141	-0.046664235	-0.117539278
Lbp	3.24748E-63	1.31E-101	1E-129	0.083825899	-0.148961526	-0.117844261
Hlf	7.07987E-24	3.09E-64	5E-78	0.062714575	-0.14620986	-0.11787267
Tmem19	2.87316E-11	1.31E-64	3E-135	-0.032626792	-0.115438836	-0.117887769
Gata4	2.46548E-51	6.66E-39	1E-111	-0.070081672	-0.099665574	-0.118367008
Rsbn1	1	2.18E-68	3E-96	-0.011056123	-0.136895019	-0.11842198
Plin4	1.97734E-17	1.03E-114	2E-238	-0.034166924	-0.123418238	-0.118495048
Cyp2c67	0.005912737	2.12E-102	2E-32	-0.064841788	-0.247071351	-0.118579467
Vkorc1l1	1	1.31E-71	2E-80	0.007308921	-0.151164302	-0.118644132

Itch	1.88733E-22	1.29E-16	7E-70	0.061926601	-0.064206042	-0.118652889
Mup7	2.5262E-128	8.69E-129	0	-0.06742196	-0.11825742	-0.118812173
Insc	2.55096E-67	4.15E-54	6E-125	-0.079006486	-0.108644474	-0.118926764
Fmnl2	9.08669E-13	7.38E-04	4E-106	-0.042511369	0.140016402	-0.119372747
Rhoa	1	1.31E-49	3E-117	-0.009224843	-0.105684931	-0.119495683
Sdr9c7	1	4.48E-74	1E-168	-0.011138491	-0.115763135	-0.120336401
Pxdc1	1	5.17E-97	5E-194	0.00328654	-0.124860082	-0.120346128
Adarb1	1	1.28E-83	2E-181	-0.003546376	-0.116910838	-0.12036467
Camk2n1	7.88168E-07	1.17E-103	1E-250	-0.02182902	-0.1163109	-0.120394128
Cyp4v3	3.57761E-13	8.38E-45	2E-107	-0.039127216	-0.111778509	-0.120429369
Npr2	1	2.67E-123	4E-254	0.014773915	-0.12576235	-0.120701476
L2hgdh	1	7.22E-87	9E-126	-0.01038283	-0.141405466	-0.120704859
Ppp6c	1	4.09E-65	1E-112	-0.016826696	-0.12771484	-0.120909692
Glyctk	2.4385E-07	3.00E-80	1E-150	0.032344035	-0.12733245	-0.120961138
Hipk3	1	6.60E-22	3E-113	-0.011612588	-0.074642159	-0.121027073
Tlk1	1	1.00E+00	2E-73	-0.009427532	-0.020712466	-0.121964368
Nfil3	1	2.52E-123	2E-130	0.012976784	-0.164697537	-0.122113381
Ipmk	1	3.96E-42	5E-103	0.0066283	-0.109104103	-0.122126122
Nrbp2	2.98862E-28	2.59E-120	3E-235	0.046189763	-0.128490845	-0.122175919
Nat8f2	1	1.20E-52	3E-230	0.002950656	-0.088508396	-0.122436081
Erp44	1	6.35E-92	1E-98	0.019137998	-0.16108801	-0.122548003
Tmem241	0.086499466	1.57E-90	3E-136	-0.019223502	-0.141063162	-0.123097952
Fpgs	0.014323345	8.59E-113	7E-48	0.027379096	-0.244372938	-0.12360575
Apol7a	1.09543E-19	8.17E-109	2E-125	-0.044099123	-0.160063654	-0.123820934
4930556N1	1	1.79E-32	3E-158	0.007560971	-0.082062984	-0.123909906
Cmtm4	0.008806713	6.06E-46	2E-132	0.023496747	-0.104098217	-0.124277291
Agpat2	3.43733E-34	7.74E-117	2E-236	-0.042891944	-0.129224908	-0.124388071
Qdpr	0.064841877	2.01E-105	9E-213	-0.016901866	-0.128393551	-0.124401262
Tra2a	1.59378E-05	4.98E-07	1E-85	-0.032576122	-0.044715135	-0.124507137
Mttp	1	6.06E-112	2E-76	0.005034608	-0.204861674	-0.124520673
Cyp4a14	0	0.00E+00	9E-35	0.726562354	-0.667968278	-0.124602158
Adhfe1	2.71235E-90	8.39E-73	5E-87	-0.106522723	-0.154559128	-0.124606318
Aff1	2.42782E-17	1.00E+00	1E-86	-0.050923451	0.031359354	-0.124814045
Dcakd	1	2.36E-60	6E-96	-0.014459685	-0.137428586	-0.124820871
Pum1	1	2.03E-09	8E-75	-0.01634353	-0.050868149	-0.124827503
Nnmt	2.57986E-54	5.40E-108	4E-158	-0.067109445	-0.148947712	-0.124849049
Cyp4f17	7.33657E-13	9.06E-97	5E-113	-0.03891645	-0.159083711	-0.124974568
Adck5	6.64795E-39	3.01E-95	6E-183	-0.052924649	-0.130470315	-0.125032927
Zfp318	0.682429586	1.39E-94	1E-125	-0.016903588	-0.150993286	-0.125194937
Crp	2.66113E-57	5.63E-58	4E-17	0.105268158	-0.237027392	-0.125257213
Spag9	1	7.77E-23	2E-71	0.014950197	-0.076497554	-0.125587931
Ppp2r5a	1	7.97E-07	3E-68	-0.023042988	-0.05672019	-0.126009143
Prpf4b	2.0051E-129	1.15E-163	5E-79	0.132785748	-0.250092782	-0.126079583
Asap3	0.220256892	4.14E-123	7E-186	-0.016795301	-0.148781222	-0.126097198
Ngef	6.98469E-33	8.68E-52	5E-81	-0.069769382	-0.139633965	-0.126325875
Atg7	1.57419E-26	2.16E-01	1E-66	-0.071261396	-0.02772567	-0.126550805
Arhgap35	1.94084E-06	5.28E-124	2E-82	-0.03362711	-0.213480644	-0.126576022

Cfap20	6.20095E-08	2.02E-88	4E-186	0.02993312	-0.126150087	-0.126799768
Pde8a	9.7528E-32	3.63E-40	9E-77	0.075156363	-0.126101394	-0.126936
Acss2	2.20108E-05	7.79E-212	3E-97	0.059415463	-0.291448212	-0.127139444
Rprd1a	9.27599E-08	3.67E-63	7E-87	-0.041223538	-0.14626286	-0.127207491
Reps1	3.31596E-08	2.89E-36	7E-92	-0.03630878	-0.106134833	-0.12731687
Zfhx2	1	7.32E-105	2E-182	0.004174056	-0.138083584	-0.127340708
Apoe	0.028195315	2.54E-32	2E-54	0.022891537	-0.11178166	-0.127374844
Gse1	3.74682E-98	4.38E-80	2E-93	-0.11608821	-0.165211401	-0.127651233
Wdtc1	1	9.81E-28	2E-90	0.008145493	-0.099294784	-0.127653375
Myo1d	0.001362504	8.88E-51	2E-106	-0.026107536	-0.122278257	-0.127667718
Hsd17b11	1.13422E-13	1.46E-75	4E-133	-0.04004427	-0.132268684	-0.127723948
Slc10a1	1.55097E-54	2.45E-196	2E-91	0.092087978	-0.248652491	-0.127727842
Tedc2	2.514E-140	8.07E-91	2E-140	0.127965554	-0.144993491	-0.127918236
Zfp644	8.85364E-17	3.18E-16	3E-78	-0.054325511	-0.080979684	-0.128551522
Car5a	1	1.78E-137	2E-112	-0.003625481	-0.200968648	-0.128558608
Ugt3a2	1	2.87E-54	5E-94	0.005880017	-0.136406724	-0.128601973
Plin5	1	2.09E-51	8E-159	-0.004443065	-0.10783258	-0.128724912
Syn3	0.157978196	4.12E-59	7E-86	-0.024075124	-0.147776588	-0.12891928
Stag1	1	1.00E+00	4E-76	0.003304566	-0.011893433	-0.128951075
Fyco1	0.00037415	2.90E-65	1E-112	-0.026187854	-0.136238808	-0.129428583
Cth	0	2.73E-83	8E-26	-0.435038962	-0.29032455	-0.129727514
Fut8	1	1.60E-114	6E-224	-0.008548615	-0.132871878	-0.12987046
R3hcc1l	1	8.20E-58	1E-102	-0.008131822	-0.132507409	-0.130213007
Ube2b	1	1.78E-41	4E-80	-0.020951303	-0.123251628	-0.130762049
Brd3	1	7.89E-69	5E-95	-0.012301676	-0.151678095	-0.130813658
Adgrl2	1	1.48E-19	5E-68	-0.008106878	-0.103299384	-0.131236236
Zc3hav1	4.26795E-71	7.39E-16	3E-126	-0.084625918	-0.055631726	-0.13153176
Ak2	1	2.74E-82	1E-109	-0.009771959	-0.155349797	-0.131539771
Scap	0.101442455	1.16E-125	5E-115	-0.022337049	-0.186986382	-0.13155531
Paics	1	2.77E-70	2E-151	0.008626825	-0.126301384	-0.131624854
Tcf20	6.30495E-22	8.05E-20	2E-79	-0.063150442	-0.087870223	-0.13166869
5031425E2	5.21172E-61	2.31E-68	1E-69	-0.109451601	-0.169419201	-0.131736822
Nsmce2	9.67541E-30	1.00E+00	2E-70	-0.076232601	0.058350678	-0.131875626
Scnn1a	6.66426E-05	1.66E-92	7E-234	-0.022504459	-0.122878861	-0.131903518
Hnrnpc	1.20172E-05	1.71E-28	3E-89	-0.033683593	-0.103553365	-0.132236101
Pabpn1	1	1.08E-134	1E-78	-0.010836424	-0.232063414	-0.132271303
Larp1b	1	1.28E-172	4E-70	-0.024230038	-0.282755286	-0.13246015
Gm12910	3.37309E-29	7.37E-121	0	-0.03711646	-0.119080824	-0.132648737
Qprt	1	4.37E-138	3E-231	-0.006345564	-0.148328439	-0.132760785
Bnip3	2.9363E-140	1.34E-12	3E-115	-0.130951794	-0.059568256	-0.133005224
Gm30835	3.18309E-57	3.29E-72	6E-237	-0.060529243	-0.111491197	-0.133005871
Tnrc6c	0.000638942	1.00E+00	1E-81	-0.034160134	-0.010009768	-0.133307847
Sp3	2.67097E-05	3.40E-44	3E-128	-0.028385957	-0.10616153	-0.133411515
Pptc7	1	1.97E-34	8E-82	0.004177917	-0.118778316	-0.133414633
Sbf2	5.17643E-15	1.03E-01	4E-85	-0.052345132	-0.02872784	-0.13344829
Col4a3bp	1	1.68E-38	2E-107	0.004482902	-0.106611673	-0.133623942
Camta1	1.86423E-11	1.06E-84	1E-198	-0.032335532	-0.120528879	-0.133680409

Inpp5b	1	1.02E-108	2E-168	0.017714272	-0.15019604	-0.133951005
Ncam2	1.17595E-60	1.00E+00	1E-85	-0.093093928	-0.02279255	-0.133976688
Atat1	1	2.21E-88	2E-186	-0.013684274	-0.132688261	-0.134032719
Ncoa3	1	1.68E-30	1E-164	-0.015324324	-0.067638714	-0.134125427
Rev3l	3.16509E-05	6.86E-42	6E-89	-0.033537927	-0.129682492	-0.134757964
Eif4b	1	7.54E-66	3E-133	-0.007999091	-0.131887231	-0.135017356
Stk38	1.93338E-12	1.52E-29	5E-93	0.046753812	-0.104493314	-0.135044949
Dnmbp	0.000238384	1.41E-29	4E-76	0.032710995	-0.111419199	-0.13530961
Pds5b	0.002147214	8.00E-69	1E-137	-0.023671881	-0.133059485	-0.135405408
Dhdh	1.44637E-10	3.03E-67	3E-85	-0.043990459	-0.168550776	-0.135614462
Pklr	9.994E-27	2.63E-101	5E-247	-0.047290542	-0.131139841	-0.135690456
Rnf103	7.96381E-39	4.02E-73	3E-127	-0.065318599	-0.14294507	-0.135714299
Rabep1	2.27838E-10	5.77E-22	4E-75	0.041563734	-0.085328925	-0.135736825
Chd7	1	5.11E-18	6E-77	0.010940863	-0.081291473	-0.135762257
Mob1b	9.36345E-15	4.07E-59	2E-156	-0.039126456	-0.117433096	-0.135859602
Cmbl	1	2.95E-115	4E-219	-0.014805039	-0.14380501	-0.136020531
Sdr42e1	2.89465E-37	1.14E-67	1E-124	-0.066074347	-0.138358644	-0.136264725
Asxl1	1	4.38E-05	2E-98	0.015148397	-0.047497272	-0.136302013
Fbxl17	1.13854E-29	3.78E-239	8E-90	0.052654831	-0.295572826	-0.136499226
Invs	2.94999E-11	4.50E-93	1E-158	-0.034268382	-0.146175718	-0.136598337
Snrk	5.93667E-16	1.63E-65	5E-154	0.045491038	-0.119970395	-0.136615127
Pwwp2a	8.38368E-07	3.48E-37	1E-133	-0.02978543	-0.097752739	-0.136713977
St13	1	2.25E-80	5E-127	-0.004370448	-0.147027539	-0.137120519
Serpinc1	8.15748E-17	4.38E-188	4E-48	-0.058754439	-0.318034127	-0.137237736
Ppp6r3	1	4.61E-18	1E-96	-0.004894645	-0.078074002	-0.137465943
Ceacam1	1	7.22E-148	1E-152	-0.009983374	-0.187302225	-0.137684089
Fam210a	1.03333E-23	1.52E-34	3E-137	-0.050254172	-0.098701362	-0.137758163
Zfpm1	1	1.14E-94	2E-211	0.008363279	-0.132738454	-0.13785547
Oip5os1	4.41506E-38	3.00E-59	7E-164	-0.058952127	-0.116973812	-0.138000845
Ablim1	1	1.11E-69	3E-83	0.008012084	-0.178939083	-0.138157746
Gm30301	1	7.03E-114	2E-284	-0.012507062	-0.130587773	-0.1384562
Tln2	5.477E-50	4.50E-65	2E-110	-0.077356357	-0.146615608	-0.138578838
Fgfr4	3.60863E-52	4.41E-123	7E-177	-0.065036024	-0.164371892	-0.138859659
Msl2	6.44541E-06	2.19E-94	1E-143	-0.027670187	-0.155558852	-0.138914459
Rybp	2.56934E-11	1.88E-83	2E-186	-0.033603609	-0.12995934	-0.139090154
Mup2	1.38024E-25	1.24E-17	2E-166	-0.051347555	-0.072076455	-0.139121627
Rnf125	3.77296E-28	3.38E-38	2E-35	-0.102823849	-0.20067613	-0.139280629
Gm31121	2.32249E-09	6.97E-118	0	-0.029076561	-0.135127598	-0.13933452
Nr1d2	1.41368E-31	1.37E-78	3E-224	-0.048631209	-0.119791154	-0.13939648
Gm31583	2.48035E-95	2.94E-122	0	-0.067949543	-0.130826996	-0.139549683
Ppp4r3a	1.0005E-112	1.86E-40	2E-90	-0.133880055	-0.128297604	-0.139864234
Tmem131	1.54175E-35	3.03E-37	3E-97	0.076439539	-0.119897608	-0.139871801
Faah	1.59471E-44	3.20E-163	1E-107	0.082762052	-0.239054794	-0.139970683
Gm31522	0.011909716	1.38E-131	0	-0.016883877	-0.13742487	-0.139982479
Tsc1	1.10444E-08	2.57E-119	1E-137	-0.032616134	-0.180551981	-0.140001021
Arhgef19	1	2.17E-164	8E-188	-0.014636185	-0.185099473	-0.140054815
Jak1	1	2.20E-20	3E-90	-0.000341783	-0.082056845	-0.140392429

Ldlrad4	4.18004E-95	1.97E-85	1E-57	-0.154390724	-0.217810814	-0.140866628
Shoc2	1	6.84E-66	7E-129	-0.008835587	-0.135769019	-0.140961347
Hsd17b12	4.25531E-25	2.22E-199	9E-101	-0.062079854	-0.269141945	-0.141209245
Spin1	8.57378E-26	1.73E-82	1E-170	-0.049485582	-0.139557036	-0.141285868
Gm45083	3.47183E-47	8.82E-97	4E-261	-0.056215549	-0.128573782	-0.141361572
B2m	2.20148E-15	1.91E-51	9E-190	-0.0408622	-0.097247626	-0.141533377
Atf7	0.004688673	1.03E-03	2E-101	-0.028728272	-0.044436015	-0.141725691
Epb41l4b	1	7.76E-137	1E-128	0.006791553	-0.202764291	-0.141891369
Map2k1	1	5.01E-70	2E-112	0.000592551	-0.151950673	-0.141984365
Gpt	1	3.25E-146	1E-204	-0.000709854	-0.17028666	-0.142026263
Mindy3	1	4.07E-62	5E-145	0.007660065	-0.129528043	-0.142245363
Klhl3	8.24642E-23	3.07E-174	2E-127	0.058332186	-0.234333867	-0.142494382
Ywhaq	0.0002235	5.21E-84	3E-252	-0.022203826	-0.120928872	-0.142499943
Bag4	1	2.24E-91	2E-189	0.003573944	-0.140803692	-0.14255822
Nectin1	1.38682E-41	4.98E-104	9E-116	0.081774654	-0.188565241	-0.142611608
Dock8	1.00312E-32	6.28E-25	6E-161	-0.060579795	-0.049960258	-0.14262301
Gm49797	0.003037187	1.00E+00	4E-106	-0.027370661	-0.013836902	-0.142857304
Nacc2	3.09021E-17	4.17E-93	3E-198	-0.038811266	-0.139506726	-0.142885872
Exph5	4.00632E-19	1.49E-69	8E-157	-0.048106171	-0.135650126	-0.142897156
Acad11	3.69251E-63	5.72E-52	6E-96	0.097353371	-0.136941673	-0.143071188
Ppargc1a	6.70499E-26	1.30E-53	1E-67	0.063906574	-0.162883948	-0.1433701
Aldh7a1	5.64142E-33	1.21E-116	2E-105	-0.069214174	-0.206525255	-0.143516794
Nufip2	8.70602E-12	2.24E-41	2E-118	-0.041669484	-0.110564128	-0.143798838
Ccdc152	2.24999E-92	1.41E-101	7E-291	-0.074738118	-0.129182608	-0.143926216
Numa1	1	5.79E-69	7E-122	-0.000955463	-0.148775169	-0.143986767
Zfp697	1.85902E-55	2.02E-136	3E-251	-0.061262919	-0.155151447	-0.144101953
Pls3	1	1.44E-101	8E-134	-0.004268693	-0.172771725	-0.144119391
Ctif	3.55653E-45	7.26E-91	2E-79	0.076351226	-0.199966713	-0.144133192
Arid4b	1	1.35E-47	9E-99	-0.009736744	-0.121365578	-0.144138449
Tob2	1	6.60E-84	9E-191	-0.005833196	-0.137079116	-0.144261468
Cyp4f13	1	8.33E-93	7E-137	-0.006990676	-0.163366899	-0.144406606
Usp32	0.01384673	4.52E-87	2E-113	-0.02599577	-0.17002257	-0.144713593
Gm3734	0.001219135	1.08E-53	4E-132	0.028757658	-0.134341665	-0.144730987
Rnf19a	1	3.38E-91	5E-118	-0.003237082	-0.175115733	-0.145123838
Pter	5.10803E-13	9.80E-106	8E-108	0.050138833	-0.197201918	-0.145177629
Tmbim6	1	2.16E-71	5E-112	-0.003410555	-0.157718055	-0.145375338
Nf1	0.223790322	5.27E-43	2E-98	-0.028828767	-0.122017803	-0.145439772
Rbms1	2.11432E-23	1.00E+00	7E-60	0.074304778	0.043444233	-0.145513398
Nrf1	1	3.34E-24	6E-108	0.008084917	-0.097706782	-0.145540776
Msh3	0.706573268	9.71E-75	2E-121	-0.020309476	-0.157405859	-0.145834703
Sfpq	1	8.34E-24	3E-117	-0.016827012	-0.090664634	-0.146116939
Diaph1	1.65962E-34	2.37E-11	1E-105	0.076288757	-0.065050379	-0.146220646
Scarf1	1	1.89E-170	1E-174	0.011585343	-0.202360229	-0.146224708
Shld2	6.1491E-204	7.24E-47	2E-57	0.218435614	-0.178336485	-0.146424773
Srd5a1	1.07846E-25	2.67E-131	9E-280	0.050495395	-0.149351185	-0.146462956
Etfdh	1	1.86E-85	4E-117	0.001339322	-0.174265805	-0.14664118
Rab1a	2.27453E-06	2.50E-58	2E-143	-0.02986019	-0.126347372	-0.146697962

Arl4a	3.57322E-14	2.24E-113	1E-237	-0.034478612	-0.1456811	-0.14672904
Slc20a2	1.02342E-07	8.19E-59	1E-89	-0.042858089	-0.164751207	-0.147242471
Itsn1	1	1.55E-10	1E-115	-0.017965317	-0.054470077	-0.147251297
Cacul1	1	2.44E-85	1E-119	-0.0189013	-0.169966321	-0.147353953
Arid1a	1	6.09E-09	3E-110	-0.002951641	-0.060625156	-0.147479919
Ywhaz	1.81951E-62	5.39E-70	3E-185	-0.074135608	-0.124621114	-0.147513503
S100pbp	3.59432E-09	7.78E-120	2E-219	-0.031225295	-0.155781288	-0.14793995
Bicral	3.72891E-11	2.09E-81	5E-116	-0.042405717	-0.171179929	-0.148110944
Tef	9.02242E-30	4.28E-66	4E-253	-0.047027187	-0.112856232	-0.148281104
Gm47889	5.71185E-40	2.49E-85	1E-180	0.060805915	-0.146771283	-0.148396138
Selenoi	1.11197E-24	2.74E-90	6E-129	-0.058588411	-0.170266335	-0.148533529
Ccdc58	1.33135E-22	2.22E-34	2E-112	-0.058527762	-0.11414924	-0.148611185
Jarid2	1	3.30E-76	7E-119	-0.006155832	-0.160372903	-0.148739066
Ahcyl1	1	4.66E-89	3E-141	0.002211437	-0.164271204	-0.148989573
Acadsb	3.00938E-60	1.27E-122	2E-160	-0.077928704	-0.182163641	-0.149025971
Ebpl	1.51361E-11	6.14E-151	0	-0.030763163	-0.153215991	-0.149417975
Fam20c	1	3.20E-58	5E-239	-0.013903605	-0.088959978	-0.149459449
Paip2	1	7.69E-76	7E-114	-0.018615183	-0.167592716	-0.149577945
Snrnp70	7.56235E-08	2.33E-57	4E-105	0.033803846	-0.139406372	-0.149638285
Gm35188	1.08909E-40	5.01E-21	2E-162	0.066058293	-0.082074826	-0.150307771
Cbfa2t2	1.71982E-33	3.42E-53	2E-114	-0.070994291	-0.144681885	-0.15035172
Fbf1	5.57354E-16	2.17E-146	2E-201	-0.042960617	-0.180408322	-0.150423967
Kat2b	0.000224272	1.66E-09	5E-96	0.028898071	-0.05450669	-0.150499519
Rtf1	0.009944881	1.36E-83	1E-153	0.027524697	-0.15274247	-0.150525552
Slc45a3	5.07984E-66	4.53E-134	1E-205	0.086014819	-0.172460214	-0.15058406
Vmp1	1	2.80E-03	3E-98	0.013971406	-0.031861606	-0.150643026
Ambra1	4.14599E-10	7.91E-28	2E-105	0.041396011	-0.089551604	-0.150678266
Taf15	2.20538E-15	2.54E-41	6E-114	-0.051475372	-0.125584428	-0.150739778
Atp2a2	2.85906E-05	2.75E-80	2E-98	0.033528521	-0.184129018	-0.150910209
1700012D1	3.77436E-08	2.75E-90	2E-203	-0.031448325	-0.144752548	-0.15109837
Tmem25	4.15294E-05	2.12E-181	0	-0.020799297	-0.167277275	-0.151197622
Lin52	0.288329699	9.60E-32	3E-99	-0.026494641	-0.120980071	-0.151284468
Nsd3	1.85618E-06	7.57E-31	1E-115	-0.04436854	-0.094104147	-0.151527638
Dag1	1	3.09E-99	4E-178	-0.010325314	-0.160286115	-0.151978483
Akr1c20	0.016297032	9.72E-158	2E-218	0.023535986	-0.183040313	-0.151991766
Cyp4a12b	5.22511E-31	5.98E-151	0	-0.049313043	-0.153199408	-0.152058874
Cpne1	1.02282E-10	9.33E-98	9E-149	-0.038319425	-0.170549371	-0.152112379
Tmcc1	1	2.62E-12	9E-102	0.018427375	-0.062114606	-0.152148612
Sc5d	0.547936096	6.72E-234	1E-187	0.028448693	-0.252314619	-0.152696525
Yes1	9.10729E-16	2.91E-66	2E-144	-0.046232358	-0.142469364	-0.15283299
Polr2a	1.66091E-35	4.31E-89	4E-128	-0.071497385	-0.174211612	-0.152870274
Fer	4.80868E-23	1.03E-114	8E-157	-0.053318831	-0.180242805	-0.153127391
Mecp2	1	1.28E-61	2E-179	-0.015265832	-0.125070269	-0.153836965
Xpo7	0.231335676	9.00E-27	5E-109	0.026065903	-0.101364764	-0.153882102
Apoc1	1	9.13E-184	1E-86	-0.02517763	-0.300840167	-0.153956093
Smc5	0.108456317	4.60E-56	2E-126	-0.024213569	-0.138301755	-0.1543342
Mef2d	4.87291E-09	1.28E-16	3E-129	-0.039695574	-0.076913747	-0.154665446

Cyp4f14	1.4138E-63	3.84E-177	0	-0.05652601	-0.152965852	-0.154673348
Homer2	1	2.80E-162	0	0.00178812	-0.158978632	-0.154847897
Tob1	1.1176E-117	4.99E-122	3E-199	-0.09971127	-0.171639718	-0.155030444
Fhod3	3.52057E-29	1.21E-141	0	-0.043491177	-0.149158437	-0.155198518
4732471J0	3.06788E-37	1.01E-92	2E-187	-0.063556555	-0.156271089	-0.155431084
Creb3l2	0.001074686	4.32E-162	4E-144	0.035429395	-0.233544418	-0.155725406
Tram2	2.9584E-10	9.21E-51	1E-196	-0.036491992	-0.111933346	-0.156180965
Gm27216	4.61442E-40	2.38E-167	0	-0.049463418	-0.159103474	-0.156335691
Wdfy2	0.002549001	2.26E-74	5E-166	-0.0240971	-0.140623002	-0.156436788
Afdn	1.15334E-07	6.94E-52	5E-121	-0.040449879	-0.135858056	-0.156469523
St3gal3	1.0143E-265	2.85E-156	2E-89	-0.225817841	-0.295570564	-0.156550872
Ggact	4.94925E-27	3.89E-62	1E-152	-0.059570383	-0.142066779	-0.15669114
Trim7	4.51493E-38	1.60E-121	2E-273	-0.053973372	-0.153806229	-0.156971955
Aldh6a1	1	2.37E-151	2E-164	-0.002507146	-0.208042218	-0.157869439
Tmed5	3.41654E-80	3.21E-58	1E-167	-0.092162011	-0.131888473	-0.158156883
Kif1c	0.174240171	2.77E-106	1E-176	-0.019441086	-0.170868514	-0.158435411
Rsf1	1	1.42E-73	2E-136	-0.014347141	-0.159352697	-0.159101918
Khk	1.31587E-07	2.84E-122	2E-298	-0.02761053	-0.150381673	-0.159618694
Pla1a	1.37229E-37	1.12E-179	7E-284	-0.054420492	-0.183697219	-0.159748258
Serpinf1	0.002885977	1.24E-156	0	-0.019393829	-0.158407474	-0.159872509
Arhgap29	5.28577E-78	1.39E-181	4E-128	-0.106699682	-0.260783044	-0.159925221
Ahcy	1.119E-156	2.07E-195	2E-148	0.152635863	-0.253153676	-0.159960026
Slc38a3	7.73711E-69	4.55E-78	2E-96	-0.117459839	-0.199543482	-0.160279744
Mup11	1.43244E-41	3.29E-159	0	-0.048780288	-0.154679042	-0.160307828
Clec2h	3.1319E-212	1.30E-140	0	-0.107402022	-0.156644148	-0.160627709
Zfp444	1	5.97E-144	1E-273	-0.012778551	-0.168126201	-0.160628455
Atrx	2.53284E-13	3.74E-53	5E-114	0.047848433	-0.134021152	-0.160910033
AY036118	1	1.00E+00	3E-117	0.014844975	0.087608845	-0.161689161
Pex7	0.398692013	5.39E-99	2E-159	-0.019664879	-0.177390185	-0.161717653
Zfp652	6.43422E-32	5.82E-16	5E-129	-0.069967435	-0.076220999	-0.161953414
Tbl1xr1	1	1.00E+00	5E-122	0.017995086	-0.003509194	-0.162490839
Ap2a2	7.04532E-16	2.07E-98	2E-157	0.053088147	-0.176973006	-0.162569123
Nfyc	1.83471E-15	1.35E-120	2E-144	-0.049109159	-0.204871641	-0.162764359
Strbp	4.41691E-36	3.55E-01	1E-120	-0.085251651	0.015180139	-0.162821301
Usf3	1	3.10E-143	1E-149	-0.010632238	-0.218877091	-0.163071815
Prdx6	1.44251E-22	8.75E-84	7E-173	-0.052846894	-0.15772405	-0.164053313
Fam169b	1.96901E-20	8.41E-157	5E-107	-0.065051376	-0.26981501	-0.164363678
Lrp6	1.62596E-38	5.06E-16	1E-127	-0.079736684	-0.079096087	-0.164502472
Pde4b	6.4046E-214	1.59E-80	8E-253	-0.129463575	-0.130066658	-0.164743725
C8b	2.3945E-173	1.56E-147	0	-0.105783061	-0.164548524	-0.16542877
Tfdp2	7.47896E-71	6.07E-108	5E-168	-0.093912343	-0.186134885	-0.165783181
Dnase2b	5.12141E-35	3.77E-95	1E-199	-0.061839447	-0.162518093	-0.166192397
Mfhas1	6.53062E-81	5.88E-29	1E-162	-0.101101844	-0.09845107	-0.16625061
Celsr1	1.79579E-06	6.98E-167	0	-0.023943429	-0.164669625	-0.166358917
Psen2	1.19631E-09	3.32E-125	8E-216	0.038016376	-0.177960424	-0.166922063
Nsd1	1	8.80E-13	2E-132	-0.007342475	-0.049239514	-0.166948444
E130102H2	1	1.13E-112	6E-273	0.019458032	-0.155753789	-0.167088535

C3	0	4.76E-65	3E-89	-0.384544767	-0.201499201	-0.167170976
Iqsec2	1.148E-32	4.05E-131	3E-247	-0.057542249	-0.173089668	-0.167195958
Gckr	5.48232E-57	1.94E-289	4E-117	0.090446717	-0.357892467	-0.167402501
Litaf	1.89647E-87	1.72E-22	4E-208	-0.097378896	-0.048414457	-0.167526159
Zpr1	4.1103E-12	3.13E-110	4E-120	-0.050788145	-0.217264611	-0.167726874
Zbtb44	1.66557E-06	2.54E-83	2E-166	-0.032830434	-0.165941635	-0.168186002
Gm32461	7.60952E-15	8.48E-168	9E-243	-0.043189454	-0.204418731	-0.168249592
Rnf130	1	2.51E-73	4E-177	-0.011548274	-0.146134195	-0.168407925
Map3k2	1	1.93E-84	5E-170	-0.010957458	-0.16418573	-0.168568133
Habp2	1	1.52E-201	4E-233	0.000131305	-0.221445838	-0.168943494
Pdilt	3.04718E-79	7.94E-149	2E-297	-0.082084222	-0.176140287	-0.169338133
Sned1	1	2.39E-180	1E-262	0.003649227	-0.200734394	-0.169452334
Fah	3.57033E-11	5.62E-203	4E-136	-0.04709373	-0.290074492	-0.169890736
Msrb1	3.51885E-22	3.97E-123	3E-286	-0.044238552	-0.156594056	-0.16990577
Luc7l2	1	8.59E-199	8E-158	0.013465276	-0.267063618	-0.170003468
Skap2	1	3.02E-09	2E-243	-0.01202146	0.001777102	-0.17021052
Prkcz	1.17387E-63	5.84E-141	7E-244	-0.077492577	-0.18484032	-0.170652672
Entpd8	1	1.94E-202	1E-265	-0.000916858	-0.211541351	-0.171114381
Pafah1b1	1	5.54E-30	1E-135	-0.011111298	-0.099424033	-0.171121342
Dlg1	8.57646E-18	1.67E-19	2E-131	-0.062901374	-0.077054667	-0.171314065
Rbm6	0.287875895	1.40E-58	7E-137	0.022786184	-0.140869164	-0.171730443
1600020E0	6.32744E-07	8.13E-91	3E-158	0.038740576	-0.180572065	-0.17173928
Upb1	0.032086868	1.17E-109	5E-123	0.026820252	-0.216929288	-0.171979024
Tanc2	1	4.44E-130	3E-249	0.015672354	-0.166615613	-0.172225826
Scai	1.9309E-46	1.89E-149	0	-0.058834719	-0.164762429	-0.172450201
Sik2	1	2.01E-87	8E-180	-0.01519127	-0.166163375	-0.172505329
Hnf4aos	1.9642E-254	2.10E-260	4E-39	-0.250671705	-0.467076701	-0.172588297
Abhd17c	6.79684E-26	9.46E-109	5E-169	-0.063388043	-0.192133833	-0.17274319
Foxn3	3.69971E-31	1.86E-02	1E-94	-0.090282067	-0.031432175	-0.172871584
Gfod1	1	1.38E-115	2E-253	-0.015587974	-0.160895291	-0.173019705
Cacna1d	2.2733E-33	5.74E-121	0	-0.05031893	-0.139078328	-0.173077986
Peli1	1	2.71E-25	4E-174	-0.002882428	-0.085487059	-0.1733758
Rabgef1	1	1.61E-147	4E-176	0.01489975	-0.220620921	-0.173383877
Fmo5	1.48949E-45	4.49E-21	8E-08	0.146423227	-0.308213059	-0.173545879
Pcca	1	8.45E-255	3E-126	-0.002210102	-0.334418259	-0.173887024
Grk5	1	1.14E-44	5E-207	-0.0129535	-0.102116462	-0.174045167
Dcun1d4	1	6.04E-160	0	-0.006880542	-0.180922678	-0.17414547
Lcor	1	7.89E-17	1E-140	-0.021470118	-0.069619162	-0.174260331
Tesk2	1	4.25E-168	3E-144	-0.014836191	-0.258033787	-0.174339101
Supt3	1.48363E-05	9.20E-128	3E-159	-0.034140133	-0.211797421	-0.174585719
Ppargc1b	5.04533E-24	4.19E-33	3E-133	-0.071000104	-0.117929912	-0.174844209
Arhgef11	7.53616E-12	4.57E-55	9E-155	-0.044847681	-0.146462433	-0.175451665
Sipa1l1	7.04774E-42	8.43E-46	2E-122	-0.093021099	-0.135184029	-0.175693515
Pias1	1.55117E-10	5.36E-25	2E-171	-0.041349116	-0.090536763	-0.176580158
Ddx17	1	1.96E-70	2E-158	-0.028030247	-0.14712272	-0.177171922
Srsf11	5.72601E-09	1.48E-64	3E-153	0.035330895	-0.145659463	-0.177310349
Gpd1	0.188542903	2.17E-133	0	-0.014852309	-0.170005673	-0.178066399

1600014C1	1.57456E-10	2.11E-122	4E-268	0.038657493	-0.171817819	-0.17813811
Srgap3	1.3278E-107	1.14E-168	0	-0.084935143	-0.175434039	-0.178480573
Mcm10	3.26372E-97	7.51E-186	0	-0.080374055	-0.18347416	-0.178486161
Itih1	1	3.44E-232	2E-172	-0.011741302	-0.288375997	-0.178586694
Rap1a	0.244528711	1.15E-70	3E-207	-0.020536452	-0.139286595	-0.178591832
Ankrd28	0.001589861	1.24E-64	6E-158	-0.031075558	-0.157821795	-0.179027412
Agxt	1	3.56E-155	6E-247	-0.009245817	-0.200761115	-0.179520289
B630019A1	4.1184E-172	9.26E-101	3E-112	-0.186652005	-0.234311427	-0.179648557
Map2k5	0.004537262	5.26E-74	3E-149	-0.035766974	-0.157099041	-0.180135558
Synj2bp	4.85136E-19	1.18E-164	3E-204	-0.049330218	-0.223398164	-0.180246671
Gm31814	2.20218E-80	1.19E-79	1E-239	-0.092534758	-0.151626487	-0.180314429
Pcyt2	0	0.00E+00	5E-117	0.214892266	-0.443442296	-0.180370458
Pnisr	1.52825E-14	1.95E-80	3E-160	0.044950829	-0.162605252	-0.18045035
Gm15622	9.1298E-184	1.11E-181	0	0.154324929	-0.194155338	-0.180479585
Ralgapa2	2.66823E-49	6.79E-09	5E-140	0.079425239	-0.064574998	-0.180524279
Rbms2	1	7.95E-64	9E-201	-0.002992071	-0.128114764	-0.181099692
Dbi	0.000148572	8.79E-115	3E-228	0.030891132	-0.178278082	-0.181135949
Gm11266	6.023E-101	1.76E-161	3E-273	-0.09933591	-0.202473344	-0.181142559
Spata13	6.61244E-11	3.63E-42	5E-119	-0.054537684	-0.151255484	-0.181241971
Dixdc1	1	3.38E-191	0	0.003207326	-0.19012955	-0.181913739
Gatad2b	1	2.91E-29	8E-161	0.000598473	-0.108080531	-0.182351579
Gm26708	1.55157E-34	2.26E-167	0	-0.053697417	-0.177541839	-0.182532682
Ywhae	1.55639E-11	7.61E-81	3E-249	-0.037684423	-0.14535172	-0.183258902
Rmnd5a	2.5978E-23	2.20E-66	4E-162	-0.061792053	-0.162557132	-0.183293849
Efna5	1	4.82E-70	1E-169	-0.017724286	-0.163817867	-0.183403907
Fam193b	5.54957E-20	1.75E-149	2E-178	0.056233141	-0.231974567	-0.183451436
Coq8a	3.34777E-05	1.42E-146	5E-207	0.032501963	-0.216144462	-0.183482877
Adnp	2.22749E-12	4.58E-157	2E-201	-0.041860543	-0.222415373	-0.183513575
C730002L0	0.957048305	2.42E-15	4E-133	-0.040377272	-0.085057744	-0.183516251
Zfp277	2.04568E-46	6.13E-122	7E-201	-0.077760355	-0.197624002	-0.183731752
Mbtd1	0.003510607	1.37E-44	3E-167	-0.028590308	-0.13066274	-0.184167755
Lnpep	0.012198793	2.21E-29	1E-207	-0.024944857	-0.082328628	-0.184169403
Mfsd2a	9.91945E-72	1.23E-148	4E-121	0.130545882	-0.26798669	-0.184886034
Pde4c	6.20492E-28	4.75E-132	7E-231	-0.059063558	-0.198085786	-0.185535084
Ubn2	0.000492437	3.82E-21	2E-152	0.029989036	-0.084460761	-0.185720761
Hsd17b4	1	2.45E-50	3E-154	-0.011318603	-0.135370014	-0.185784166
Ptprf	8.89934E-09	4.28E-128	2E-220	-0.036338797	-0.200220515	-0.185819846
Vps13c	3.06879E-06	2.52E-20	6E-190	-0.033288196	-0.080710082	-0.186437902
Zhx2	2.28264E-07	3.83E-44	5E-154	-0.040577001	-0.138504298	-0.186663291
Fam193a	1	4.20E-92	6E-169	0.008217503	-0.173786873	-0.186699547
Cd302	5.34918E-97	1.35E-81	2E-151	-0.127498725	-0.189479947	-0.18743867
Gm26917	6.99036E-92	7.53E-86	4E-155	0.094767871	-0.255513732	-0.187560664
Rad23b	2.55919E-05	1.32E-142	4E-194	-0.032314547	-0.224564752	-0.188213799
Pygl	0.000520415	6.65E-103	1E-143	-0.034225471	-0.218288379	-0.188257637
Cyp2c50	8.86352E-13	1.14E-171	0	-0.038180708	-0.203409157	-0.188354659
Gigyf2	1	4.11E-61	6E-168	0.00660087	-0.143240772	-0.18856607
9030616G1	0	4.14E-170	7E-299	-0.164019102	-0.205854457	-0.188716521

Peg3	1	2.00E-207	0	0.008909931	-0.215426982	-0.188742459
Acat1	2.47845E-67	2.10E-52	3E-141	0.106384355	-0.146016126	-0.188970975
Ppp2r3a	0.101484724	5.47E-95	2E-152	-0.030724191	-0.20345617	-0.189210282
Zfp148	1	3.10E-59	1E-165	-0.028977198	-0.142585297	-0.1896359
Smurf1	2.56783E-27	2.17E-113	9E-279	-0.053651435	-0.171230208	-0.189822079
Etfbkmt	1	5.20E-94	2E-158	-0.011659783	-0.202904515	-0.190048041
Rassf8	2.82992E-33	1.83E-101	5E-227	-0.065714973	-0.173982661	-0.190840265
Tsc22d2	0.00017372	5.76E-84	3E-195	-0.031766756	-0.174364024	-0.191869247
Rtp3	0.30848881	4.00E-217	0	-0.017857063	-0.208511291	-0.192363813
Gpr39	5.28968E-06	2.13E-171	2E-193	-0.039215086	-0.248854776	-0.192568943
Zbtb20	1.2974E-151	7.99E-69	2E-267	-0.105368047	-0.19082165	-0.192838193
Klhl2	3.45833E-06	2.70E-90	4E-218	-0.033918768	-0.171538698	-0.19353533
Cux1	0.000121588	3.04E-11	6E-166	-0.036753301	-0.058868253	-0.193711978
Stxbp6	3.4063E-117	8.72E-131	3E-187	-0.129901544	-0.223588615	-0.194044415
Gm10658	1	4.84E-170	0	0.014780215	-0.181901915	-0.194364921
Dennd1a	3.73398E-09	7.00E-26	4E-160	-0.04437424	-0.085348633	-0.194516676
Rdx	1.33775E-08	5.16E-89	4E-169	-0.044766546	-0.193900719	-0.194517288
Mocs1	1	4.29E-135	1E-212	-0.016687199	-0.216098527	-0.194609267
Clu	2.13833E-93	1.95E-76	4E-300	-0.095062413	-0.141679302	-0.194625308
Cyp2c70	5.4027E-12	8.24E-195	1E-213	-0.057768884	-0.260612343	-0.194715885
Dhrs4	1	3.42E-158	3E-280	0.013075191	-0.207562851	-0.194827148
Plce1	2.0246E-103	9.39E-144	4E-253	-0.108692013	-0.207253341	-0.194920116
Gm30262	1.5441E-153	5.21E-199	0	-0.100212608	-0.189137664	-0.195309621
Cpn1	1	3.10E-146	4E-214	-0.011225417	-0.224613318	-0.195579292
Pnkd	1	3.27E-150	0	0.004741272	-0.187706545	-0.195882924
Gm40264	2.48686E-75	5.13E-121	0	-0.085943451	-0.172355679	-0.195965797
Pros1	7.44202E-19	1.60E-218	0	-0.042823474	-0.22126385	-0.19606066
Inca1	8.50356E-15	1.48E-201	3E-224	-0.046268081	-0.259495199	-0.197229704
Stim1	1	2.81E-93	8E-198	-0.016988885	-0.188086905	-0.197402228
2810459M	1	4.98E-151	0	-0.005982766	-0.185198509	-0.197738851
Gjb1	0.172509185	1.39E-202	2E-178	-0.0267602	-0.293996392	-0.197815254
Gab1	5.35261E-10	1.55E-163	3E-261	-0.038245699	-0.220131138	-0.197821453
Agfg2	1.12565E-92	6.67E-147	1E-242	0.119476834	-0.21656076	-0.198864502
Proz	3.54788E-41	1.70E-202	1E-277	-0.068327584	-0.240394211	-0.199016923
Ugt3a1	1	3.58E-208	0	-0.007403603	-0.193493575	-0.199238121
Atp11c	2.58832E-27	2.25E-121	4E-128	-0.074663383	-0.277765187	-0.199433127
Gm15261	7.32965E-65	1.28E-129	0	-0.078122195	-0.17688544	-0.199537862
Ugt2b1	5.12455E-54	1.38E-64	4E-242	-0.086097254	-0.141979324	-0.199694024
Rnf13	6.94719E-06	3.73E-133	5E-234	-0.032661306	-0.207377159	-0.199862321
9930021J0	1	1.52E-78	6E-189	-0.012288829	-0.178109518	-0.200095064
Rida	1	6.64E-118	3E-189	-0.005366415	-0.218351289	-0.200425677
Fam13b	1	8.13E-54	2E-214	-0.016011481	-0.136741621	-0.20092377
Susd4	6.4764E-208	5.76E-183	0	-0.12604926	-0.199729027	-0.201263137
Adtrp	1.92714E-16	1.43E-97	1E-164	-0.058656634	-0.216140543	-0.201334468
Serpina3k	1.0546E-140	2.76E-227	0	-0.0939531	-0.198205334	-0.201622703
Mafg	1	5.62E-50	3E-188	-0.023432384	-0.145200151	-0.20211984
Trip4	0.767665281	4.44E-139	6E-185	-0.023601668	-0.243733665	-0.202447542

Kat6b	1	1.07E-84	1E-178	-0.012035206	-0.190406816	-0.202554798
Shmt1	1.00109E-11	6.29E-186	0	0.040272811	-0.220760971	-0.203175365
Hagh	4.45494E-66	1.29E-164	8E-232	-0.09354081	-0.239609352	-0.204076385
Nlk	1	2.76E-133	2E-239	-0.011957519	-0.211134381	-0.204169223
Mapk14	1	1.24E-39	2E-206	0.006700128	-0.1240474	-0.204342691
Uri1	1	9.51E-90	2E-231	-0.020030411	-0.177272792	-0.204762569
Zfyve1	1.34436E-59	6.03E-82	1E-173	-0.108302164	-0.187483104	-0.20478667
Ash1l	1	2.25E-102	7E-190	0.017575478	-0.192852848	-0.204805519
Wdr45b	2.45545E-48	7.91E-157	2E-293	-0.075069262	-0.211580327	-0.204884495
Ccl25	1	1.94E-134	4E-246	-0.002232506	-0.212804764	-0.205209766
Wfdc21	1	4.26E-243	0	-0.001636614	-0.203984755	-0.20533688
Raph1	0.000189554	1.38E-48	1E-169	-0.039295277	-0.139289166	-0.205943905
Saa4	1	1.69E-168	0	-0.001352242	-0.193691971	-0.20635247
Irf2bp2	1	2.56E-125	0	0.001987181	-0.179749818	-0.20641773
2310001H1	4.30316E-17	8.86E-175	0	-0.045886029	-0.216717436	-0.206550503
Sox6	0.000246257	1.53E-169	3E-184	-0.037533736	-0.266093503	-0.206944284
Fign	1	2.21E-145	4E-192	-0.024891813	-0.249074054	-0.207052659
Arhgef26	3.48878E-37	1.04E-33	1E-112	0.100756602	-0.152199379	-0.207906013
Tmem150a	7.46303E-16	2.63E-192	0	0.047070232	-0.219410267	-0.208996448
Paqr9	4.29519E-25	2.41E-231	0	-0.047783302	-0.204944968	-0.209488773
Tle4	1	1.39E-128	8E-240	0.013133449	-0.209767638	-0.20967347
Ldah	2.33735E-08	1.27E-173	2E-204	-0.04079494	-0.271851269	-0.211078119
Tspan12	1	1.04E-123	3E-161	-0.029791068	-0.25234608	-0.211106974
Them4	1	3.48E-208	0	0.0182529	-0.24388767	-0.211293868
Rrbp1	1	4.10E-126	1E-152	-0.027750178	-0.248231867	-0.21175789
Erc1	5.68887E-10	3.68E-82	3E-196	-0.049289559	-0.177531425	-0.211950865
Lpgat1	1.14558E-07	8.80E-116	1E-198	-0.042665437	-0.224949722	-0.212008576
5730522E0	2.63661E-18	1.42E-54	5E-120	-0.075206981	-0.197680194	-0.212167775
Stra6l	1	7.04E-174	3E-217	-0.019279683	-0.259275671	-0.212259445
Stat3	1.92775E-08	6.42E-08	4E-180	-0.047384743	-0.061158816	-0.212328747
Gm12909	1.6872E-27	6.73E-153	0	0.054525937	-0.173145885	-0.212329124
Larp4b	1.1612E-06	3.89E-42	4E-194	-0.045745041	-0.123527482	-0.212479875
Chd6	1.98094E-10	2.88E-119	2E-233	-0.043202325	-0.213107907	-0.212715333
Aadac	1	7.70E-241	0	0.002001547	-0.241286221	-0.213608391
Mmd	1.19355E-17	6.92E-147	2E-259	0.051657937	-0.226935264	-0.213624153
Osbpl9	1	2.83E-28	2E-193	-0.022564218	-0.096331707	-0.213908651
Agt	3.88452E-10	3.76E-249	0	-0.033051499	-0.239721069	-0.214328951
Enox2	1.18279E-40	4.40E-39	5E-191	-0.087468454	-0.132670432	-0.215647352
Agmo	6.9436E-189	6.28E-129	1E-143	-0.188524523	-0.285468614	-0.216195975
Slc6a13	1.27679E-19	0.00E+00	0	0.055268324	-0.3357089	-0.216247311
Slc22a28	1.3348E-49	4.29E-259	0	-0.06017659	-0.21603701	-0.216314386
Psd3	1.51809E-58	3.66E-85	6E-206	-0.091215156	-0.184936749	-0.217498706
Rcl1	1	1.02E-105	2E-190	-0.011903168	-0.225022811	-0.217802209
Herpud1	2.1135E-274	1.58E-119	3E-205	0.2263471	-0.230594475	-0.218211029
Apoh	0.033768285	3.02E-181	6E-276	0.026364648	-0.247261956	-0.218624767
Aff4	0.00646701	3.97E-44	2E-230	-0.031046859	-0.127414028	-0.219098529
Syne1	1.1572E-22	2.83E-38	6E-198	-0.069007819	-0.127157539	-0.219423419

Pten	8.42309E-12	1.73E-41	1E-223	-0.049388984	-0.126353913	-0.219986972
Anp32a	0.011610207	6.79E-126	0	-0.024205311	-0.196011711	-0.220387085
Gpd2	0.025411574	2.29E-188	0	0.026839218	-0.227822632	-0.220571094
Stau1	1	2.23E-162	1E-286	0.021609127	-0.232858375	-0.220672154
Gsk3b	5.20524E-32	1.25E-28	1E-214	0.071844091	-0.101303658	-0.220895038
St8sia3os	1.3974E-79	8.91E-115	4E-195	-0.124835065	-0.237570889	-0.221088355
Gpatch8	0.036041477	4.45E-123	2E-225	-0.031595929	-0.217223563	-0.2215826
Zfp871	1	4.34E-106	1E-274	0.011804794	-0.190290966	-0.221783833
Sirt3	1	1.89E-190	3E-258	-0.004937851	-0.267058346	-0.222295868
Dcaf5	3.06448E-37	6.46E-87	1E-231	-0.077679618	-0.191236752	-0.222388507
Fgg	6.58896E-25	0.00E+00	2E-212	0.070702189	-0.393472406	-0.223050522
Atxn10	1	8.98E-152	1E-260	-0.013776637	-0.235376829	-0.223068501
Prkag2	1	8.11E-149	2E-141	-0.006208219	-0.29644539	-0.223228492
Hs6st1	1.74322E-52	8.24E-58	8E-273	-0.086006016	-0.146234558	-0.223256737
Trp53inp2	1.10816E-16	3.24E-169	0	0.047098108	-0.218555277	-0.223715695
Jpx	1	5.07E-169	0	0.002804122	-0.222731073	-0.223890671
Golgb1	1	2.09E-189	2E-220	-0.004264537	-0.282865279	-0.223982446
Rnf111	1	9.68E-47	2E-252	-0.001825533	-0.130411108	-0.224206344
Epb41l5	1	4.06E-172	3E-271	0.002959427	-0.250781465	-0.224336201
44263	0.099297958	7.11E-143	0	0.026027081	-0.214895214	-0.224431893
Nadk2	1	2.31E-152	0	-0.002499555	-0.220955075	-0.224494655
Slc25a23	2.4952E-20	4.42E-212	0	-0.045937633	-0.230562954	-0.225283096
Gm47719	1	6.50E-116	0	-0.010974103	-0.183257875	-0.225550156
Gm13775	7.37657E-05	2.14E-66	9E-153	-0.025070624	-0.194562779	-0.225780718
Otulin	6.2013E-146	6.61E-46	1E-266	-0.142815628	-0.12099748	-0.226318078
Cdc14b	3.00132E-31	9.92E-159	3E-229	-0.073244726	-0.261826567	-0.226480672
Acsm3	1	3.35E-187	0	0.015513989	-0.239127884	-0.226751426
Rtn3	0.080819395	1.87E-61	2E-206	0.025478046	-0.160667025	-0.227608419
Zkscan1	1	1.17E-148	0	0.015445476	-0.217191105	-0.227885827
Bcas3	1	2.23E-63	3E-191	-0.003819898	-0.160181023	-0.227901095
Apol9b	4.0264E-120	7.27E-221	0	-0.095039144	-0.214616061	-0.228042047
Agtr1a	1	2.54E-212	7E-264	-0.016972515	-0.283076009	-0.228091041
Nr0b2	1.41775E-80	3.89E-158	8E-284	-0.109317675	-0.241961185	-0.228126683
Pxmp4	5.14992E-11	2.49E-205	5E-229	0.039386078	-0.307309553	-0.228202475
Ifnar2	1	5.81E-143	0	-0.007758998	-0.195850462	-0.22877776
Ndfip1	9.429E-113	3.39E-60	7E-257	-0.129028498	-0.156615153	-0.228881082
Nars2	3.01334E-37	1.66E-186	4E-239	-0.078983448	-0.282182789	-0.229709449
Chd2	1	5.28E-26	6E-221	-0.023151188	-0.090098717	-0.230941168
Baat	8.67465E-80	1.08E-248	0	0.109315776	-0.275725794	-0.231294275
Cyth1	0.260862716	1.01E-37	1E-273	-0.025033364	-0.111945457	-0.231805004
Aadat	9.48137E-26	3.31E-245	0	-0.053129832	-0.243258346	-0.232835168
Cgnl1	1	3.44E-120	2E-233	-0.015209504	-0.223619535	-0.232885501
Rbbp4	4.75493E-07	9.77E-198	0	-0.034299144	-0.250991531	-0.233654284
Znrf3	1	4.14E-79	2E-223	-0.01526369	-0.186904195	-0.233814464
Eps8l2	1	5.90E-80	7E-212	-0.005303672	-0.194992104	-0.234040937
Lrig1	6.30324E-43	8.69E-160	0	-0.076390129	-0.231587776	-0.23406818
Camk1d	6.79545E-45	1.00E+00	1E-238	-0.096703035	0.014912385	-0.234382305

Sh3pxd2a	1.11821E-24	9.22E-168	1E-216	-0.069682672	-0.282441699	-0.234661401
Adrb3	1.72558E-42	2.12E-238	0	-0.067804442	-0.238004941	-0.235604083
Cdk19	5.87388E-63	5.44E-106	0	-0.093490103	-0.193263376	-0.236654425
Gm16573	1.45504E-46	2.15E-133	2E-263	0.074993599	-0.238899484	-0.237696603
Zfp395	1	3.82E-216	0	-0.004965521	-0.277454978	-0.238980629
Srrm2	1.84059E-77	1.24E-209	3E-303	0.085918414	-0.262939369	-0.239572572
1700001C1	6.04701E-16	3.28E-296	0	-0.039938013	-0.24767091	-0.240213922
Zbtb7c	1	1.42E-183	0	0.001212269	-0.222229464	-0.240481073
Ube2r2	1	2.06E-80	4E-253	-0.024890763	-0.183554077	-0.241074158
Fnip1	1.55949E-09	2.74E-36	1E-301	-0.041615343	-0.111789295	-0.242482417
N4bp2l2	0.000509084	1.56E-113	3E-276	-0.033490077	-0.212906414	-0.24276174
Chd4	8.38818E-10	3.20E-143	2E-250	-0.047616671	-0.256013558	-0.242790108
Serpinf2	1	5.93E-255	0	-0.010458218	-0.289750125	-0.242811042
Qk	3.67237E-26	2.13E-26	2E-295	-0.06517889	-0.093533799	-0.242890284
Rbm33	0.394121015	2.99E-121	2E-290	0.021213131	-0.20856559	-0.243782001
Phactr4	1	9.00E-71	6E-282	0.018700953	-0.166568935	-0.24460235
Gm47465	1.57739E-86	8.68E-225	0	-0.0943748	-0.234181937	-0.245005639
Rai14	2.767E-112	3.19E-131	2E-228	-0.148048411	-0.254330408	-0.245352929
Pan3	1	2.85E-51	2E-274	0.009647126	-0.126305722	-0.245619557
Olfr1033	1.25493E-55	1.20E-140	1E-196	-0.114280779	-0.287057205	-0.245642233
Ppm1b	9.49314E-09	1.04E-124	2E-253	-0.048199812	-0.228015575	-0.245831221
Mkln1	0.040120071	3.88E-82	6E-275	-0.031626396	-0.167951221	-0.246053861
Cyp2j6	1	5.34E-297	0	-0.013746978	-0.259905907	-0.24616834
Lrrfip1	1	1.21E-154	0	0.01563284	-0.215262219	-0.246184421
Xpr1	5.30346E-15	2.34E-189	0	-0.047460974	-0.244333068	-0.246656218
Ftcd	1.52061E-57	6.96E-227	1E-174	0.105011539	-0.364992486	-0.247286779
Mcu	1	3.28E-119	1E-243	-0.020815523	-0.231697103	-0.247375353
Rab10	1	1.73E-27	2E-265	0.006952642	-0.101944189	-0.247436482
Dexi	1.81293E-34	1.33E-275	0	-0.057534239	-0.255309553	-0.247473525
St6gal1	4.34917E-34	4.77E-142	0	-0.067792142	-0.198939754	-0.248407307
Acot12	7.00289E-24	2.74E-75	3E-256	0.060440894	-0.174674373	-0.248593454
Ddi2	8.97294E-49	3.23E-238	0	0.073874914	-0.304598898	-0.249023559
Ppp2r5e	2.6412E-292	7.31E-70	9E-216	0.245080486	-0.183778608	-0.249693819
Pbx1	6.4484E-18	3.03E-57	2E-231	-0.065181753	-0.170322319	-0.250044459
Gm31333	5.23543E-18	1.93E-292	4E-299	-0.055109184	-0.344928087	-0.25038183
Cryl1	8.39163E-17	4.22E-43	4E-133	0.080020904	-0.190113015	-0.250389415
Gcnt2	9.43913E-92	3.16E-49	8E-205	0.153193346	-0.168769601	-0.25047709
Itpr2	1.2185E-48	4.96E-191	1E-270	-0.093158264	-0.281986625	-0.250860329
Ahcyl2	6.12794E-39	1.42E-134	3E-296	-0.080434718	-0.235648547	-0.251070339
Asl	1.2597E-222	1.60E-243	3E-207	0.238039913	-0.399880172	-0.251919524
Cbs	3.0003E-34	6.51E-243	2E-257	0.075859979	-0.336198269	-0.252121635
1700028E1	6.79721E-81	1.27E-163	0	-0.097973925	-0.209587835	-0.25217125
Arhgap32	4.24601E-30	5.43E-208	0	-0.063550031	-0.259077047	-0.253002035
Cyp2d9	1	4.44E-263	8E-271	0.018517699	-0.35029521	-0.253643705
Sec63	1	1.33E-124	6E-285	-0.020508659	-0.225616669	-0.253823789
Hp	1.91415E-56	1.32E-150	0	0.103452817	-0.203501357	-0.253921428
Zfp609	4.1382E-106	1.26E-136	7E-291	-0.13209685	-0.244866041	-0.25433854

Pipox	3.80542E-14	4.33E-237	0	-0.047868186	-0.302544993	-0.254469199
Gm4952	1	0.00E+00	1E-266	-0.00420199	-0.407957593	-0.254999672
Zcchc24	7.69686E-09	5.43E-187	0	-0.039227237	-0.264673807	-0.255425173
Rnf214	1	8.15E-226	0	-0.018896298	-0.304142032	-0.255748639
Mbnl1	1	2.44E-29	1E-263	0.000291402	-0.088437378	-0.255790303
Ehmt1	0.064153102	1.10E-127	3E-284	0.023577467	-0.22057454	-0.256064807
Vegfa	4.40353E-11	0.00E+00	4E-295	0.043418473	-0.394972989	-0.256184813
Plcb1	1.5026E-161	1.84E-193	2E-195	0.172317133	-0.359159697	-0.257600002
Usp3	0.057274122	3.59E-69	0	0.024899411	-0.162280977	-0.258347734
Cd1d1	1	4.02E-274	0	-0.017479646	-0.29844061	-0.258489798
Ppm1l	1	5.22E-262	2E-301	0.008883727	-0.335949491	-0.258613848
Arhgap6	5.61759E-87	2.66E-86	4E-206	-0.151897911	-0.233346843	-0.258685006
Lonp2	1	2.07E-206	0	-0.016633457	-0.29757873	-0.259752239
Sardh	6.72274E-70	1.11E-287	0	-0.092094882	-0.296331886	-0.259859485
Parva	1.40076E-12	4.82E-161	1E-265	-0.052879029	-0.282686994	-0.259901649
Hipk2	0	2.23E-69	6E-248	-0.310987785	-0.173779655	-0.260228406
Nfat5	1.42981E-33	1.20E-101	0	-0.075238896	-0.20074718	-0.26032578
Stat2	1	5.30E-238	0	0.022967875	-0.300491149	-0.260566938
Onecut2	2.44808E-38	7.75E-188	5E-239	-0.095480415	-0.314237105	-0.26107455
Gm37494	8.0496E-30	4.20E-254	0	-0.064648614	-0.293943886	-0.261242322
Gm30117	5.8622E-133	8.00E-200	0	-0.145786728	-0.278455277	-0.261599216
Ahdc1	3.09921E-24	8.34E-275	0	-0.060620416	-0.314379328	-0.262133354
Abcb11	1.53525E-51	0.00E+00	5E-81	0.082755392	-0.666232313	-0.263955862
Tacc1	5.51941E-10	3.01E-25	0	-0.046872605	-0.106470662	-0.264679616
Serpina3m	3.04247E-08	0.00E+00	0	-0.043903021	-0.296701166	-0.264708592
Ptprg	1.75049E-80	8.52E-171	0	-0.114829259	-0.275483197	-0.265089359
Daam1	4.83015E-79	3.08E-122	2E-273	-0.124301389	-0.24156748	-0.265476671
Ces1d	0.000118171	7.73E-173	0	-0.031260271	-0.234583532	-0.265549731
Tmprss6	1	1.78E-151	0	-0.001143437	-0.253967034	-0.266148245
Rdh7	1.15242E-80	3.75E-202	0	0.113081956	-0.298567217	-0.266529595
Gucd1	0.000596237	3.21E-182	0	0.031776563	-0.273474634	-0.266655986
Wsb1	1.2356E-163	4.83E-124	0	-0.152163671	-0.209764871	-0.267909974
Rad54l2	6.70526E-26	1.30E-146	9E-272	0.070384234	-0.271587879	-0.268220902
Cyp2d26	7.5437E-110	0.00E+00	0	0.128601179	-0.311623642	-0.269804304
Fam168a	1	1.27E-189	0	-0.01724347	-0.242154663	-0.270094364
Afmid	1.77473E-15	4.59E-287	0	-0.04228816	-0.272189692	-0.270447044
Slc7a2	1.6904E-150	1.25E-240	5E-104	0.252025989	-0.584226828	-0.270507464
Rtn4rl1	1	5.95E-210	0	-0.011685985	-0.275845134	-0.272219234
Ndrg2	1	0.00E+00	0	-0.001997049	-0.403512362	-0.272408919
Ccnd3	0.069730193	6.18E-16	0	-0.032223446	-0.069677367	-0.272501755
Zranb1	1	4.88E-162	0	0.007935589	-0.252281674	-0.273173101
Tor1aip1	9.12769E-19	1.47E-204	0	-0.054318468	-0.272165201	-0.273750417
Tmtc2	1	1.27E-50	5E-197	-0.008128425	-0.187169319	-0.273933678
Gstz1	1	0.00E+00	0	0.017249998	-0.358512395	-0.275004491
Rtp4	1.1632E-56	2.04E-279	0	-0.080808083	-0.275060818	-0.276497788
Sntb1	1	7.15E-66	1E-208	-0.010484099	-0.201513562	-0.276512937
Caln1	0.06938791	7.85E-126	1E-275	-0.034166099	-0.267300425	-0.276535107

Insr	1	4.21E-155	0	-0.022195691	-0.240162211	-0.276736162
C730036E1	1.66969E-47	0.00E+00	0	-0.065662797	-0.276628452	-0.277760626
Rere	1	6.41E-201	0	0.000857869	-0.27561787	-0.278036694
Plekha6	1.12575E-09	1.50E-249	0	-0.040299745	-0.294993179	-0.278177941
Tmem56	4.3542E-18	2.29E-225	8E-217	0.062631565	-0.375159832	-0.278557783
Sh3d19	0.002404537	4.09E-62	3E-276	0.027010409	-0.184356969	-0.278709746
Selenbp2	0	4.27E-286	0	-0.197251066	-0.274387598	-0.278766452
Gm11342	0.002834914	9.51E-279	0	0.025573268	-0.260235173	-0.278805309
Qsox1	0	4.78E-285	0	-0.224299907	-0.348965396	-0.27904019
Atxn1	2.91433E-63	0.00E+00	8E-197	-0.103589987	-0.441599771	-0.27946098
Dhrs3	1	4.00E-53	0	-0.00350773	-0.158576217	-0.279946971
Cyp4a12a	7.5045E-159	0.00E+00	0	-0.129656945	-0.281046084	-0.28049898
Syt1	3.34515E-22	7.29E-170	0	-0.061828606	-0.237829764	-0.280642396
Lamp2	1	2.08E-120	0	0.011776547	-0.225122172	-0.280888623
Il15ra	1.36228E-97	2.00E-179	0	0.12284832	-0.248558541	-0.281435484
Slco2b1	0.423982197	0.00E+00	0	-0.017351866	-0.29919962	-0.281998416
Pank1	2.88538E-38	8.98E-215	7E-234	-0.103865672	-0.365740134	-0.282002664
Gm12718	0	1.37E-259	0	-0.177202593	-0.274457326	-0.2828138
4932438A1	1	4.16E-106	0	-0.023819839	-0.193936123	-0.283245741
N4bp2	1	3.59E-248	0	0.012485863	-0.271926539	-0.285218399
Gm29571	1	1.27E-216	0	-0.017350835	-0.283237256	-0.286184417
Elovl2	8.73931E-32	0.00E+00	1E-293	0.084056464	-0.438796321	-0.288077433
Sash1	1.87733E-65	4.61E-07	0	-0.112152234	-0.046672705	-0.288231463
Hoga1	9.80257E-40	0.00E+00	4E-299	-0.090265863	-0.428739613	-0.288511977
Fads6	7.81555E-23	3.13E-275	0	-0.057794817	-0.311032716	-0.290150415
Slc30a10	3.37574E-15	0.00E+00	0	0.044960326	-0.281645857	-0.29042403
Ggnbp2	1	3.55E-210	0	-0.027542249	-0.288332786	-0.290829584
Slc12a7	7.78231E-08	0.00E+00	0	-0.040978381	-0.360275752	-0.291104116
Luc7l3	1	3.52E-135	0	0.012281892	-0.223076495	-0.291363439
Ugt2a3	0	5.83E-265	0	-0.21658626	-0.288548728	-0.291364087
Ces1e	6.03607E-21	0.00E+00	0	-0.050887792	-0.285938128	-0.291905702
Rfx3	1.08767E-16	1.05E-212	0	-0.053376	-0.274707553	-0.292259127
Rreb1	1	1.85E-72	0	0.000975371	-0.167024886	-0.292521696
Kng1	0	4.03E-151	3E-222	-0.360741907	-0.330917875	-0.293297996
Sord	1	6.91E-134	9E-270	0.008665632	-0.279121488	-0.293678512
Scarb2	1	1.50E-43	0	-0.009201176	-0.135783177	-0.294355013
Cyp2j9	1	0.00E+00	0	0.012205383	-0.307176323	-0.294580733
Smurf2	3.68628E-18	2.25E-136	0	-0.059768383	-0.234133589	-0.295405209
Gys2	0.210374269	2.29E-284	0	-0.024810044	-0.297450024	-0.295844284
Hibadh	2.6083E-144	7.27E-238	0	-0.166632436	-0.330999544	-0.296434409
Creb3l3	2.1312E-33	7.83E-179	0	0.067918719	-0.275159055	-0.29701042
Brd4	1	4.77E-102	0	0.012335934	-0.188312035	-0.297869353
Tmem131l	9.1305E-06	9.59E-32	0	0.038273618	-0.119342114	-0.299152075
Gpat4	4.19346E-85	9.35E-277	0	-0.116936816	-0.338674088	-0.299569842
Itih4	1.2053E-295	9.87E-172	0	-0.192173248	-0.245562246	-0.300070148
Pde9a	4.74859E-38	0.00E+00	0	-0.063412057	-0.308946011	-0.300102998
Kif13b	5.65058E-37	3.53E-207	0	0.072395974	-0.285102546	-0.300351526

Chp1	6.7812E-169	1.38E-279	0	-0.169479809	-0.365700338	-0.300626714
Cspp1	3.23973E-15	4.80E-171	0	0.050787441	-0.265120211	-0.302086646
Sox5	1	0.00E+00	2E-265	-0.022672188	-0.50471641	-0.302600732
Mllt10	1	1.36E-193	0	0.008610311	-0.27314533	-0.302811199
Adap2	8.5848E-185	1.88E-109	0	-0.155946224	-0.18109285	-0.302815286
Kalrn	0.015441736	1.18E-268	0	-0.024111864	-0.289475418	-0.304668078
Kng2	2.88164E-29	1.89E-209	0	-0.086163654	-0.349095756	-0.304899802
Dgat2	2.1856E-165	9.85E-290	0	0.175707514	-0.348873521	-0.305204426
Ranbp10	5.08546E-22	1.88E-177	0	-0.068151688	-0.282889754	-0.305338477
Numb	6.61593E-05	4.47E-181	0	-0.038410766	-0.281006338	-0.305342134
Adh4	7.6354E-172	2.72E-265	0	-0.154167469	-0.302523838	-0.30610225
AI182371	2.50687E-45	8.51E-303	0	-0.099821279	-0.412400861	-0.306573353
Nr3c1	1.1999E-138	1.54E-112	0	-0.152622812	-0.215235107	-0.307580645
Slc25a30	2.6218E-187	1.68E-244	0	-0.170646726	-0.295742252	-0.308948886
Hamp	2.40194E-44	2.34E-136	0	0.149258205	-0.273767695	-0.308966824
Ube2g1	2.42079E-08	4.29E-104	0	-0.044154594	-0.211937459	-0.310193321
Ripor2	6.2215E-199	7.94E-181	0	-0.174349087	-0.25873061	-0.310196034
Lyst	1	1.19E-61	0	-0.026712423	-0.134230681	-0.311139249
Mbl1	7.0788E-211	0.00E+00	0	-0.147674649	-0.316742944	-0.311936288
Egfros	0	4.11E-258	0	-0.182812665	-0.266837819	-0.312330214
4930402H2	0.003666316	5.74E-140	0	-0.034825388	-0.25676583	-0.31280226
Ncoa1	0.000171233	3.71E-124	0	-0.037335223	-0.224311338	-0.312851834
Inpp4a	6.84649E-18	1.63E-220	0	0.058136397	-0.291194686	-0.313575159
Slc25a47	1	1.48E-302	0	-0.020608494	-0.351608199	-0.313582195
Afm	1.123E-106	0.00E+00	0	-0.131420755	-0.384758966	-0.314197799
Rufy3	1.08438E-23	1.38E-55	0	-0.065508914	-0.136675383	-0.314574564
Spop	3.58727E-11	8.08E-84	0	0.045446883	-0.181312706	-0.314732185
Eva1a	7.33683E-11	5.34E-154	0	-0.054132233	-0.271102514	-0.315061435
Ass1	2.7552E-174	1.36E-179	1E-122	0.301038672	-0.491976161	-0.315175683
Tspan9	1.99038E-28	1.07E-288	0	-0.065075938	-0.339149933	-0.315912083
Cyp3a13	9.23637E-94	3.25E-239	0	0.136464124	-0.298994289	-0.316649449
Slc17a3	9.93051E-28	0.00E+00	0	-0.067701294	-0.367269089	-0.317039778
Mapkapk2	3.5649E-249	4.23E-245	0	-0.211604142	-0.339316889	-0.31705224
Mtus1	2.5493E-227	0.00E+00	0	-0.188372837	-0.448814229	-0.317198475
Acsm1	0	0.00E+00	0	-0.22907903	-0.388331643	-0.319045902
Cyp4a32	1	1.32E-175	0	-0.015806016	-0.289305539	-0.319136345
Gk	5.1701E-155	0.00E+00	0	0.181665002	-0.389589072	-0.319364984
Cyp2c38	3.4037E-192	6.21E-144	1E-250	0.346386255	-0.409394347	-0.320152455
Smg6	1	5.60E-66	0	-0.012392163	-0.170125802	-0.321047778
Apbb2	8.29709E-92	2.08E-201	0	-0.134682745	-0.304340322	-0.321562166
Slc47a1	9.32436E-45	0.00E+00	0	-0.082399133	-0.398566131	-0.321803542
Pigr	4.98783E-19	0.00E+00	0	-0.052122718	-0.305469217	-0.32203645
Hsd3b5	0	0.00E+00	0	-0.192015679	-0.322056144	-0.322056144
Cyp2f2	9.2475E-28	2.64E-108	0	0.093456354	-0.270092167	-0.322292661
Fgd6	3.21616E-10	1.33E-123	0	-0.0530303	-0.22628832	-0.322386519
Irf2	9.08563E-20	1.44E-90	0	0.061225094	-0.193472243	-0.322480897
Gldc	6.97284E-96	1.22E-160	2E-257	0.163463044	-0.345123998	-0.323373869

Rabgap1l	2.4433E-175	5.83E-250	0	-0.145089512	-0.345885863	-0.324015665
Large1	0.269834979	8.68E-186	0	-0.025344527	-0.269243179	-0.324810717
Arhgap26	2.87115E-85	1.03E-162	0	-0.113064021	-0.248982109	-0.326044551
Msra	0.000212212	6.54E-208	0	-0.036016945	-0.323320352	-0.326456914
Abcd3	5.36731E-25	1.06E-149	0	-0.071739606	-0.252371736	-0.327035352
Lars2	1	2.37E-188	0	0.015802178	-0.305258321	-0.327260948
Gphn	1	0.00E+00	0	0.000368874	-0.316559701	-0.327494055
Foxp1	9.61004E-10	1.41E-128	0	-0.040088795	-0.250456943	-0.327989612
Btbd9	1	2.66E-110	0	-0.024982002	-0.219031727	-0.329492548
Mafb	2.77742E-87	4.62E-227	0	-0.120877904	-0.297298524	-0.329862311
Tcea3	1.1944E-113	0.00E+00	0	-0.115914935	-0.32260042	-0.330372213
Ppp3ca	1.69622E-25	5.49E-100	0	-0.073927676	-0.217250574	-0.330667689
Pard3b	1.05281E-41	2.28E-156	0	-0.102864545	-0.302093111	-0.331325952
Dnajc12	1.6771E-115	7.41E-207	0	-0.167644293	-0.346039743	-0.331624728
Utrn	1	1.65E-138	0	-0.020193414	-0.239957925	-0.333576029
Gm49417	2.04489E-44	0.00E+00	0	-0.083656969	-0.369054098	-0.33455579
Etfa	0.010855576	2.77E-230	0	-0.030779281	-0.318636321	-0.334897434
Tmem219	1	1.75E-161	0	0.014176176	-0.280916317	-0.335569592
Vwa8	3.09817E-25	0.00E+00	0	0.042765547	-0.449117315	-0.335577397
Zfand3	1	3.00E-148	0	-0.005843742	-0.229726554	-0.337561103
Iqgap2	1.849E-251	0.00E+00	0	0.161620591	-0.479211045	-0.337820581
Pdia5	1.2336E-08	6.82E-298	0	-0.048606131	-0.38734551	-0.338467064
Caprin1	0.30172101	2.00E-197	0	-0.030532505	-0.290183677	-0.338753733
Arhgef12	0.057074696	3.78E-156	0	-0.031340032	-0.234693053	-0.339018582
Serping1	1	5.55E-240	0	-0.002029358	-0.311424521	-0.340147643
Optn	2.23469E-74	2.20E-251	0	-0.118587078	-0.329621146	-0.340469318
Nipsnap1	1.00285E-07	0.00E+00	0	-0.038365351	-0.385022942	-0.341214134
Alas1	0	2.52E-126	1E-245	-0.546988106	-0.337517293	-0.341537226
Slc25a42	6.18154E-14	3.86E-165	0	-0.054684009	-0.277283814	-0.343489097
Man2a1	6.1671E-192	8.67E-72	0	0.213594819	-0.208009431	-0.343494426
Ido2	5.43784E-92	9.79E-258	0	-0.130708989	-0.362399986	-0.345403024
Mgst1	3.11851E-56	2.86E-272	0	-0.104664952	-0.363654626	-0.347215298
Maob	1	0.00E+00	0	-0.016622428	-0.41285559	-0.348480648
Fam222b	0.014570334	1.83E-164	0	0.026712947	-0.264777746	-0.349531018
Pecr	6.4101E-109	0.00E+00	0	0.151837181	-0.399640877	-0.349831435
Cpt1a	6.66918E-05	5.34E-98	0	-0.040908876	-0.2145574	-0.350801359
Glo1	1.81807E-14	0.00E+00	0	0.050250628	-0.360046405	-0.351946664
Ypel2	2.01924E-26	5.01E-163	0	-0.078704245	-0.294512611	-0.352231485
Acat3	4.95933E-84	2.89E-272	0	-0.125740981	-0.373628343	-0.352682052
Adra1b	3.6711E-71	2.31E-116	0	-0.129259263	-0.255509087	-0.354080955
Lims2	6.60357E-09	0.00E+00	0	0.043498497	-0.370267776	-0.354176298
Epas1	1.5699E-199	6.18E-158	0	0.204283111	-0.283836579	-0.354776682
Ssbp3	7.5076E-174	8.47E-242	0	-0.175144728	-0.341348455	-0.355260127
Palld	1	1.98E-163	0	0.007844801	-0.278072857	-0.355698496
Gabbr2	1.97318E-14	0.00E+00	0	-0.049137107	-0.394512357	-0.356454446
Bckdha	1	7.20E-187	0	0.00197304	-0.290462922	-0.357516319
Ankrd33b	0.207315245	1.37E-293	0	-0.022698949	-0.329938614	-0.359426973

Phf21a	1.49887E-18	2.33E-124	0	-0.065191778	-0.23207695	-0.360696875
Vgll4	1.0737E-75	6.12E-169	0	-0.129778739	-0.295137049	-0.361028433
Insig2	3.9672E-184	0.00E+00	0	0.220183711	-0.53848378	-0.361245792
Hivep2	1.0282E-114	6.85E-143	0	-0.149359154	-0.267687126	-0.3623755
Evi5	1	0.00E+00	0	-0.013548027	-0.428279672	-0.363159166
Mia2	4.5682E-178	0.00E+00	0	0.148841971	-0.371180149	-0.363198081
Adcy9	1.40796E-82	8.71E-52	0	-0.125136991	-0.176039276	-0.364240412
Zhx3	1.25629E-84	0.00E+00	0	-0.125811002	-0.446424875	-0.364790309
Plxna2	3.56232E-61	2.98E-187	0	-0.132309625	-0.345201814	-0.365503002
Cflar	1	5.08E-236	0	0.004058928	-0.319508527	-0.365793434
Znrf1	2.33084E-07	3.08E-78	0	-0.044335757	-0.178862599	-0.365981053
Ube2h	2.60945E-45	7.67E-271	0	-0.095010261	-0.361195193	-0.366040539
Nr1h4	5.56087E-45	3.84E-213	0	-0.087707705	-0.377641371	-0.367117876
Slc17a2	1	0.00E+00	0	-0.014957283	-0.43888931	-0.367268203
Rdh16	0.041991155	0.00E+00	0	-0.035195218	-0.394256573	-0.367839732
Gramd3	1	3.68E-141	0	0.029724033	-0.287402656	-0.367890626
Fggy	8.5287E-117	1.18E-267	0	-0.135284547	-0.380377222	-0.368148876
Erc2	1.009E-199	7.35E-203	0	-0.234757219	-0.382428382	-0.368219531
0610043K1	0.177817309	0.00E+00	0	0.026561223	-0.342775845	-0.369296396
Akr1c6	1.30446E-22	2.01E-151	5E-215	-0.105686233	-0.417443068	-0.369691684
Gck	1.6401E-141	0.00E+00	0	0.161653064	-0.355052793	-0.371033077
Fads2	5.09895E-25	0.00E+00	0	0.077312766	-0.466813665	-0.371042177
C4bp	7.18477E-45	0.00E+00	0	-0.092044635	-0.400303287	-0.37122861
Celf1	1	1.71E-165	0	0.017341698	-0.255605211	-0.371867676
Dpy19l1	1	6.89E-247	0	-0.019187034	-0.32741455	-0.372444254
Ece1	0.104968507	1.95E-283	0	0.023424102	-0.360795975	-0.37257569
Amdhd1	3.25515E-11	6.20E-280	0	0.056055911	-0.426392379	-0.372831836
Tcf25	2.81554E-47	1.79E-301	0	0.080204073	-0.357139193	-0.376805065
Kyat1	2.63827E-26	0.00E+00	0	-0.084512566	-0.490330664	-0.377291772
Hook3	6.78002E-06	7.10E-204	0	-0.040202507	-0.283139973	-0.377377965
Dip2c	7.90115E-21	9.74E-212	0	-0.064956895	-0.321947616	-0.377683154
Smoc1	6.4165E-09	1.16E-241	0	0.049700721	-0.403647952	-0.379083804
Map3k5	4.2673E-141	8.42E-64	0	0.197218456	-0.213702462	-0.379508961
Aldob	4.54284E-73	0.00E+00	0	0.124093168	-0.425115409	-0.37985209
Kyat3	1.57735E-88	0.00E+00	0	-0.148084188	-0.742472105	-0.379982859
Tfr2	1	0.00E+00	0	-0.000227533	-0.391380051	-0.383215807
Haao	0.095318033	0.00E+00	0	-0.025283294	-0.419968596	-0.383535233
Mlxipl	1.64158E-19	1.07E-182	0	0.053416371	-0.340265246	-0.384182345
Cyp2c23	2.0413E-135	0.00E+00	0	-0.132870681	-0.386134201	-0.384645445
Erbb4	0	2.39E-49	0	-0.313579247	-0.226776252	-0.387910998
Mthfs	8.58334E-42	1.98E-269	0	-0.089027395	-0.329335347	-0.389418287
Ttc7b	1	9.69E-279	0	0.00767484	-0.323242322	-0.390418061
Naaladl2	1.08132E-29	3.01E-177	0	-0.070460493	-0.363192581	-0.390556106
Fam107b	0.063587305	1.10E-176	0	-0.033370056	-0.317727278	-0.391059226
Fgf1	2.78961E-12	0.00E+00	0	-0.044518582	-0.394856976	-0.391409644
Arhgap5	1.2902E-106	1.31E-151	0	0.136525503	-0.270782016	-0.392519331
Abcc6	1	0.00E+00	0	-0.013611544	-0.419067074	-0.392723041

Echdc2	3.16918E-18	1.58E-289	0	0.052244325	-0.367105444	-0.394078768
Ptprd	1.0776E-299	0.00E+00	0	-0.171198219	-0.527507207	-0.39451928
Akap1	0.000122922	0.00E+00	0	-0.036449191	-0.379726981	-0.39623321
Ptms	3.782E-21	0.00E+00	0	-0.056175599	-0.3764012	-0.396645688
Apob	2.84809E-71	0.00E+00	0	-0.106192741	-0.61865819	-0.398055175
Lgals9	1.73006E-62	2.66E-207	0	-0.109101107	-0.320816086	-0.39896161
Cdk8	1	5.49E-209	0	-0.012870624	-0.291680396	-0.399236013
Kansl1l	0.001226875	5.53E-200	0	-0.033288769	-0.299132547	-0.399438442
Gm3839	4.14727E-36	0.00E+00	0	-0.082285376	-0.392381201	-0.399464005
BC024386	1.84052E-83	0.00E+00	0	0.109868889	-0.458457363	-0.400278813
Slc2a9	4.59769E-23	2.25E-294	0	-0.064541457	-0.356199986	-0.400556985
Atxn7l1	2.6838E-116	1.29E-213	0	-0.151508711	-0.319670927	-0.400913521
Acaa2	1	0.00E+00	0	-0.012867453	-0.429866845	-0.401126419
Cxcl12	1	0.00E+00	0	-0.018539198	-0.409654865	-0.401382611
Ell2	2.75808E-05	3.03E-248	0	0.036598312	-0.354826034	-0.401454103
Gls2	1	0.00E+00	0	-0.015728863	-0.416072536	-0.401626557
Kcnn2	5.74829E-05	1.98E-152	0	0.037060537	-0.302355135	-0.401815918
Svil	1.53991E-28	5.34E-216	0	-0.081146855	-0.339458932	-0.402836207
Rin2	4.31234E-25	2.99E-137	0	-0.080807878	-0.28858805	-0.406066707
Bcl6	0	0.00E+00	0	0.41448602	-0.458179642	-0.406293201
Ppm1k	8.34721E-92	0.00E+00	0	-0.126717604	-0.449433732	-0.406890002
Rnf152	5.0649E-188	0.00E+00	0	-0.190386668	-0.418870678	-0.407390199
Atrn	1	0.00E+00	0	-0.020462651	-0.388095921	-0.407610146
Txlng	1.48774E-16	2.34E-226	0	0.050551403	-0.317830042	-0.407637346
Smarca2	0.011016955	0.00E+00	0	-0.035323233	-0.388808708	-0.408274563
Pdcd4	2.28066E-54	0.00E+00	0	-0.093884102	-0.405641519	-0.413853007
Fam20a	8.65569E-18	0.00E+00	0	0.065079689	-0.461331635	-0.414128869
Pard3	1.4918E-113	0.00E+00	0	-0.107562196	-0.461453478	-0.415225918
Gna12	3.2317E-119	2.38E-154	0	-0.152814673	-0.281896967	-0.415770426
Pzp	1.8127E-139	0.00E+00	2E-259	-0.182168099	-0.687781617	-0.416810254
Eda	2.08741E-34	0.00E+00	0	-0.094862207	-0.485712151	-0.419687312
Slc25a25	0	0.00E+00	0	-0.271483406	-0.459599623	-0.422525543
Slc25a51	2.36699E-14	0.00E+00	0	0.056086462	-0.419356049	-0.423107857
Apoa2	8.43334E-35	0.00E+00	0	0.073339152	-0.418582572	-0.423824882
Pcsk5	1.86301E-45	0.00E+00	0	-0.095966671	-0.517350965	-0.424181215
C1s1	2.28386E-06	0.00E+00	0	-0.036759359	-0.449560691	-0.424524522
Vwce	1.07354E-70	0.00E+00	0	-0.098098642	-0.433307964	-0.425199416
Kcnk5	6.4359E-124	0.00E+00	0	-0.179755997	-0.433752778	-0.427223113
Kmo	0.002127671	0.00E+00	0	-0.037868656	-0.480292821	-0.428122659
Ern1	7.1467E-216	2.76E-205	0	-0.21853409	-0.330845749	-0.428651311
Chpt1	1.18025E-05	1.17E-221	0	-0.047036226	-0.345731534	-0.42917227
Mir99ahg	5.9495E-173	7.17E-245	0	-0.197698149	-0.37375952	-0.429320194
Cdh2	3.75585E-59	0.00E+00	0	-0.109326823	-0.476903673	-0.429372151
Ttr	1.02418E-80	0.00E+00	0	0.133525183	-0.638697766	-0.430213583
Dhtkd1	1.00668E-95	0.00E+00	0	-0.132721693	-0.410890085	-0.431324586
Lpcat3	3.11768E-44	8.18E-258	0	-0.102105469	-0.388839507	-0.432075476
Nectin3	9.064E-129	0.00E+00	0	-0.134116893	-0.419388769	-0.435119425

Glud1	4.438E-295	0.00E+00	0	0.271036712	-0.643531468	-0.4358983
C4b	2.06921E-38	7.10E-182	0	-0.087146122	-0.307640951	-0.437362045
Lurap1l	1.62881E-21	7.88E-132	0	-0.072918524	-0.272661649	-0.438218577
Fchsd2	4.84738E-24	1.01E-191	0	-0.077162153	-0.322329717	-0.440390792
Atrnl1	9.10571E-95	7.14E-234	0	-0.135616825	-0.361999137	-0.442823077
Cyp2c29	4.43874E-21	1.21E-228	0	0.110128156	-0.492965113	-0.444205534
Pid1	2.35499E-73	0.00E+00	0	0.091104621	-0.656083812	-0.445728157
Garem1	1	9.43E-168	0	0.000784745	-0.315495988	-0.445747776
Pde4dip	0.000100927	0.00E+00	0	0.031210797	-0.460135141	-0.447336088
Tnrc6b	1	5.37E-182	0	0.011059679	-0.259850444	-0.449016598
Ulk2	0.223228009	2.14E-233	0	-0.028708324	-0.331262144	-0.450349848
Pcx	1	0.00E+00	0	0.011263511	-0.426107275	-0.450674194
Cobll1	3.51858E-91	5.54E-236	0	0.149865237	-0.403177624	-0.451124688
Dock4	1.5309E-121	1.61E-179	0	-0.163880836	-0.330370863	-0.452212334
Onecut1	0	0.00E+00	0	-0.381507186	-0.433709643	-0.452360837
Col5a3	0.187967041	0.00E+00	0	0.027519448	-0.451883537	-0.452775108
Gm2788	4.7787E-292	0.00E+00	0	-0.233004819	-0.455395374	-0.454248051
Ptprk	3.5853E-144	0.00E+00	0	-0.128964861	-0.508623412	-0.454874215
Scarb1	1	3.81E-183	0	0.002641608	-0.32400181	-0.455004134
Lima1	1.04823E-40	2.19E-289	0	-0.092255758	-0.38509094	-0.455635573
Cfhr2	3.90146E-06	0.00E+00	0	0.033563089	-0.427510279	-0.456657995
Cadm1	9.76906E-11	2.90E-128	0	0.055264144	-0.257830549	-0.457866502
Suclg2	4.11621E-18	1.31E-299	0	-0.06109391	-0.369147248	-0.459941048
Gm4951	1.88527E-53	0.00E+00	0	-0.099053812	-0.522839444	-0.46417272
Sfxn5	7.91631E-45	0.00E+00	0	-0.08872069	-0.422028737	-0.465679755
Ehbp1	1.33372E-56	0.00E+00	0	-0.106275365	-0.417463235	-0.466184005
Nrp1	0	1.83E-244	0	-0.313581373	-0.366879295	-0.467870636
Zfp385b	5.60416E-07	1.30E-236	0	0.04285128	-0.368454473	-0.46796742
Nr6a1	3.38467E-45	0.00E+00	0	-0.0951539	-0.429382455	-0.468455344
Apoa1	1	0.00E+00	0	0.005643699	-0.485949012	-0.469496633
Lifr	2.14554E-68	0.00E+00	0	-0.143037548	-0.463666991	-0.472580378
Snx29	0.000585036	1.01E-190	0	-0.043483032	-0.310490599	-0.476813895
Shb	9.3965E-164	0.00E+00	0	0.199105147	-0.440923309	-0.47798683
Kansl1	5.68852E-07	6.06E-179	0	-0.042280844	-0.290896879	-0.478866644
Slc2a2	1	0.00E+00	0	0.001837296	-0.585004287	-0.481779264
F830016B0	1	0.00E+00	0	-0.001615176	-0.542486345	-0.481791575
Mup20	5.5088E-150	0.00E+00	0	0.199963718	-0.389394264	-0.481932138
Oaf	1.0741E-171	0.00E+00	0	-0.178878857	-0.508124527	-0.482753623
Phlpp1	1	0.00E+00	0	0.008375026	-0.404941365	-0.483744249
Gm4756	1.16745E-47	0.00E+00	0	-0.08853888	-0.437039336	-0.485904212
Adk	3.3021E-190	0.00E+00	0	0.153744371	-0.779750509	-0.488998023
Cmss1	1.42994E-34	8.32E-200	0	-0.079836182	-0.313367618	-0.489007388
Hykk	3.55699E-30	0.00E+00	0	-0.077048738	-0.519951066	-0.489384488
Gm4788	1	3.74E-266	0	0.000189742	-0.351398432	-0.491067053
Gm13773	0	0.00E+00	0	-0.238651495	-0.479962021	-0.49276134
Zfand6	4.17088E-11	1.23E-284	0	-0.055165219	-0.368576565	-0.493100738
Atxn2	3.43249E-28	0.00E+00	0	-0.062173315	-0.389266105	-0.49328633

Hacl1	0	0.00E+00	0	-0.709420395	-0.618360959	-0.494659979
Myo1e	1	8.09E-98	0	0.015484444	-0.282650729	-0.495211287
Sds	3.90755E-31	1.02E-244	0	0.073045241	-0.520626059	-0.495429061
Ugt2b5	1.4613E-23	4.05E-119	0	-0.089177145	-0.29745218	-0.500986617
Klkb1	0.023884255	0.00E+00	0	0.027338493	-0.536583246	-0.502550204
Cabyr	4.7555E-265	0.00E+00	0	-0.205834449	-0.495033773	-0.504883961
Enpp2	3.109E-291	0.00E+00	0	-0.262605835	-0.494023306	-0.505499265
Car8	5.27712E-13	0.00E+00	0	-0.067400349	-0.526212754	-0.506794201
Enpp3	2.98214E-33	0.00E+00	0	-0.078091865	-0.46896985	-0.50798806
Ugt2b36	1	4.44E-236	0	-0.00523825	-0.37477449	-0.508857794
Lrp1	9.3885E-179	9.65E-165	0	-0.174655152	-0.273885547	-0.509518362
Akap13	8.58498E-21	0.00E+00	0	-0.061965251	-0.410620732	-0.509554029
Aspg	3.4429E-104	0.00E+00	0	0.143321239	-0.531452022	-0.510296
Gm36041	1.4587E-127	7.28E-126	0	-0.223027774	-0.36618568	-0.510875633
Macrod1	3.304E-213	0.00E+00	0	-0.201872979	-0.500078971	-0.511975616
St3gal5	0	1.22E-170	0	0.547874478	-0.414125237	-0.521370197
Bmp1	4.46839E-07	0.00E+00	0	0.039149738	-0.559557173	-0.521635413
C6	2.24011E-88	0.00E+00	0	-0.143932676	-0.485738797	-0.521664206
Igfbp4	3.60377E-06	0.00E+00	0	-0.039275555	-0.523420123	-0.523877168
Ephx2	1	0.00E+00	0	-0.010251628	-0.435917914	-0.524622078
Sorbs1	3.14989E-88	0.00E+00	0	-0.136894778	-0.443442994	-0.527632915
Chd3	1.58148E-11	0.00E+00	0	-0.054591144	-0.56460413	-0.528476818
Itih3	1	0.00E+00	0	-0.024484416	-0.661823022	-0.529123701
Sec14l2	5.80472E-66	0.00E+00	0	-0.108613579	-0.529050303	-0.529223307
Xiap	1.59175E-09	0.00E+00	0	-0.052818936	-0.440962527	-0.529708879
Sorbs2	2.01206E-46	0.00E+00	0	0.089715948	-0.545204924	-0.530935559
Upp2	0.000324756	0.00E+00	0	0.032675396	-0.556295562	-0.534471422
Sgms2	8.0426E-192	0.00E+00	0	-0.218815801	-0.503228733	-0.5380965
Glt1d1	1	0.00E+00	0	0.005336036	-0.473503242	-0.540072771
Arl15	7.26709E-31	0.00E+00	0	-0.084229809	-0.478716592	-0.541551488
Zfhx4	9.40371E-39	0.00E+00	0	-0.088850149	-0.558076771	-0.542649078
Slc16a10	2.3913E-57	6.84E-245	0	0.143655668	-0.468470909	-0.543402857
Sdc2	3.35574E-29	0.00E+00	0	0.086478567	-0.595871998	-0.545364354
Lin7a	3.2391E-78	0.00E+00	0	-0.161185975	-0.522993794	-0.547124074
Clpx	0	0.00E+00	0	0.218735979	-0.529673276	-0.548795233
Gm31508	1	0.00E+00	0	-0.017931719	-0.631282544	-0.55045105
Pde3b	4.55616E-53	0.00E+00	0	-0.103027639	-0.474356968	-0.551421759
Cecr2	5.53585E-07	0.00E+00	0	-0.042650059	-0.540438977	-0.553203343
Pdgfrl	0.000182314	0.00E+00	0	-0.120149369	-0.549231157	-0.554889112
Nox4	1.242E-236	0.00E+00	0	-0.22117968	-0.52141547	-0.558410536
Magi1	1.36992E-26	0.00E+00	0	-0.060762333	-0.684901954	-0.560622501
Hal	1	0.00E+00	0	-0.0403048	-0.628940717	-0.562534292
Cadps2	0.005838506	0.00E+00	0	-0.03312103	-0.56222166	-0.564447927
Dleu2	2.8303E-222	1.24E-210	0	-0.22138088	-0.348637272	-0.567768322
Serpina1e	0	0.00E+00	0	-0.421709117	-0.567049354	-0.568046092
Osbpl8	1.04017E-41	0.00E+00	0	-0.092540242	-0.370346743	-0.568974161
Cyp2c54	1.23208E-18	0.00E+00	0	-0.082885636	-0.540120676	-0.569643496

Nudt7	0	0.00E+00	0	-0.619888042	-0.644259432	-0.571234722
Bach2	0.234968896	2.03E-171	0	-0.035190334	-0.335698494	-0.572028869
Cps1	0	4.46E-234	4E-98	0.660167106	-0.820045783	-0.572156083
Dlgap1	8.71744E-17	0.00E+00	0	-0.070901675	-0.568416407	-0.57315942
Eci2	2.79489E-61	0.00E+00	0	0.089646446	-0.753199808	-0.573331537
Slc16a2	2.06449E-63	0.00E+00	0	-0.141046938	-0.486449621	-0.574113959
Cyp4f15	5.67706E-29	0.00E+00	0	-0.072869853	-0.566864493	-0.575733145
4732465J0	1	0.00E+00	0	0.009228643	-0.466392782	-0.577804607
Elovl3	5.3333E-174	0.00E+00	0	-0.178089913	-0.577267181	-0.577938861
Cmtm8	1.48601E-51	0.00E+00	0	-0.108199677	-0.496676689	-0.579636216
Peak1	3.5407E-146	0.00E+00	0	0.169143634	-0.410125206	-0.580057065
Gas2	2.28959E-60	0.00E+00	0	-0.125414602	-0.563402081	-0.581081896
Ror1	1.4459E-160	0.00E+00	0	-0.197674476	-0.590766415	-0.582568459
Abat	1.46952E-35	0.00E+00	0	-0.090122207	-0.629694334	-0.583021168
Plcxd2	8.9682E-115	0.00E+00	0	-0.180370666	-0.565665062	-0.583575376
Gm42418	5.20231E-58	1.63E-209	0	-0.109835269	-0.295911323	-0.584306463
Serpina1c	2.8568E-199	0.00E+00	0	-0.187682359	-0.609865947	-0.58763603
Acbd5	1.17621E-55	0.00E+00	0	-0.10691897	-0.522597707	-0.588977558
Hmgcs2	0	0.00E+00	0	0.230450348	-0.742413822	-0.593820812
Hsd3b2	0	0.00E+00	0	-0.261295309	-0.600350572	-0.594544829
Sco2	6.7597E-156	0.00E+00	0	0.158713806	-0.618534243	-0.59798629
Adipor2	2.46039E-72	0.00E+00	0	-0.140036776	-0.654402222	-0.598643277
Gprin3	1.11811E-20	0.00E+00	0	-0.080473971	-0.585103885	-0.599130936
Gm48633	6.8347E-277	0.00E+00	0	-0.24927245	-0.687128119	-0.603178979
Uroc1	1	0.00E+00	0	-0.018793626	-0.642291754	-0.603475815
Ces3b	0	0.00E+00	0	-0.303911018	-0.603709091	-0.60481192
Slc4a4	6.02445E-31	5.70E-249	0	0.088177939	-0.453433521	-0.608403064
Arid5b	5.13647E-10	3.90E-302	0	-0.047599118	-0.43295903	-0.614838221
Hgd	5.08279E-54	0.00E+00	0	0.090145851	-0.66240527	-0.616129042
Ctcflos	1.73017E-75	0.00E+00	0	0.162054019	-0.678987436	-0.622950608
Zfand4	3.83466E-24	0.00E+00	0	-0.092028064	-0.582309886	-0.625147766
Gm49431	1	0.00E+00	0	-0.015707426	-0.631365718	-0.625627857
Il1rap	1	0.00E+00	0	-0.010298428	-0.569941955	-0.631153191
Slc38a4	1	0.00E+00	0	-0.000963068	-0.689175818	-0.631865058
Syne2	1.25897E-56	0.00E+00	0	-0.104596913	-0.552440021	-0.63205422
Phyh	5.93167E-34	0.00E+00	0	-0.094268079	-0.662181193	-0.638753376
Thrb	6.77222E-62	0.00E+00	0	-0.076422759	-0.623744286	-0.639309528
Chn2	1.39231E-48	0.00E+00	0	-0.100569452	-0.679410594	-0.640802569
Ptprj	3.67697E-47	1.70E-276	0	-0.108352075	-0.409835487	-0.6408465
Gm19951	7.7923E-234	4.01E-282	0	-0.240081847	-0.427153682	-0.640898058
Rbfox2	1.40499E-36	0.00E+00	0	-0.08622242	-0.591529802	-0.640979496
Igfbp2	1.84845E-23	0.00E+00	0	-0.117634926	-0.757812453	-0.641043859
Crot	5.072E-173	9.82E-286	0	-0.20485631	-0.513475328	-0.641069044
Otc	3.7448E-106	0.00E+00	0	-0.155476874	-0.668672801	-0.643429665
Acaa1b	3.81112E-96	0.00E+00	0	0.154738775	-0.651512977	-0.644117901
Tshz2	8.96448E-49	2.32E-207	0	-0.119365634	-0.383433696	-0.646155682
Gpc4	3.71473E-79	0.00E+00	0	-0.137000747	-0.668056665	-0.649076282

Mylk	3.9916E-108	0.00E+00	0	-0.149947255	-0.552056202	-0.649908849
Gm50136	1	0.00E+00	0	0.025148869	-0.636486755	-0.651788977
Gm48099	8.487E-251	2.63E-105	0	-0.248834396	-0.278722219	-0.655205845
Acsl1	0	0.00E+00	0	-0.375542379	-0.778528961	-0.659339801
Col27a1	8.61573E-68	0.00E+00	0	-0.110815313	-0.656524967	-0.660488164
Got1	0	0.00E+00	0	-0.471279184	-0.718356034	-0.675711816
Necab1	1	0.00E+00	0	-0.024366971	-0.665713772	-0.6898195
Sema4g	1.88866E-19	0.00E+00	0	0.052173158	-0.73306475	-0.69590636
Car3	0	0.00E+00	0	-0.324425503	-0.69620199	-0.699947574
Plg	1	0.00E+00	0	0.017092998	-0.749904955	-0.706663731
Ppp1r3b	1.65117E-56	0.00E+00	0	0.09894732	-0.584014527	-0.708535599
Klf12	0	0.00E+00	0	-0.289686773	-0.714333388	-0.712420137
Neb	2.6523E-250	0.00E+00	0	-0.243163563	-0.697698451	-0.718838787
Clmn	0	0.00E+00	0	-0.247384089	-0.565471391	-0.719735504
Scp2	0	0.00E+00	0	-0.574955283	-0.619486654	-0.722416056
Acox1	1.2859E-199	0.00E+00	0	-0.194809091	-0.756731252	-0.732563271
Clec2d	4.02786E-60	0.00E+00	0	0.100703842	-0.663224147	-0.738824658
Rnf169	9.7359E-24	0.00E+00	0	0.061589796	-0.581268812	-0.75219877
Arhgap42	3.98811E-11	0.00E+00	0	-0.04606695	-0.749669859	-0.753087874
Shank2	0	0.00E+00	0	-0.316057128	-0.718061687	-0.753195665
5033403H0	4.66678E-14	0.00E+00	0	-0.058247084	-0.763366826	-0.757955967
Errfi1	1.22604E-94	0.00E+00	0	-0.076353591	-0.949622699	-0.759173587
Slc39a14	1	0.00E+00	0	0.016956757	-0.676563371	-0.759312585
Prodh2	1	0.00E+00	0	-0.014735396	-0.746813269	-0.769359501
Sntg2	0	0.00E+00	0	-0.285722423	-0.745770433	-0.775339075
Gnmt	1	0.00E+00	0	-0.017261865	-0.799091411	-0.781964935
Slco1a1	0.000220962	0.00E+00	0	-0.044339047	-0.738089486	-0.784365364
Cfh	0	0.00E+00	0	-0.670740864	-0.771159168	-0.7894145
Phldb2	1.89019E-33	0.00E+00	0	-0.101515661	-0.70303403	-0.79045527
Nlrp6	7.94793E-14	0.00E+00	0	-0.060251025	-0.834496691	-0.796698065
Slc25a13	1.9458E-84	0.00E+00	0	0.094921404	-0.795008702	-0.79687733
Dpys	1	0.00E+00	0	-0.002764765	-0.62182541	-0.799024869
Keg1	0	0.00E+00	0	-0.399911294	-0.763901817	-0.806650107
Mup3	1	0.00E+00	0	0.000312889	-0.80114939	-0.808037325
Lipc	0.027739187	0.00E+00	0	-0.011177255	-0.870158247	-0.819617132
Grb14	1.75229E-07	0.00E+00	0	0.039814671	-0.738363186	-0.831244795
Col18a1	2.81215E-64	0.00E+00	0	0.088621529	-0.816563344	-0.831939665
Ablim3	6.06853E-14	0.00E+00	0	-0.066261397	-0.791585915	-0.840056774
Slc27a2	1	0.00E+00	0	-0.012972341	-0.894343389	-0.843881362
Immp2l	5.0421E-262	0.00E+00	0	-0.118046596	-0.953841964	-0.847508314
Arhgap24	2.11337E-74	0.00E+00	0	-0.149217277	-0.677650835	-0.852269287
Mcc	4.23082E-50	0.00E+00	0	0.10973091	-0.792559384	-0.881297382
Cyp2e1	1.06263E-05	0.00E+00	0	0.059261879	-0.857828294	-0.881374276
Chsy3	8.96918E-84	0.00E+00	0	-0.147957931	-0.747703269	-0.882890065
Slco1b2	3.57538E-36	0.00E+00	0	0.112964804	-0.997087758	-0.884312865
Carmil1	0.009237212	0.00E+00	0	0.039708766	-0.810200814	-0.888138633
Dapk1	0	0.00E+00	0	-0.385243795	-0.708702461	-0.891481036

Ppara	2.6326E-200	0.00E+00	0	0.199007045	-0.925217355	-0.899105365
Mat1a	1	0.00E+00	0	0.021088698	-1.112974764	-0.911010437
Sik3	1.33096E-22	0.00E+00	0	0.051069351	-0.753111684	-0.912570837
Zeb1	3.77791E-18	0.00E+00	0	-0.066526036	-0.828239392	-0.914963806
Cyp7b1	0	0.00E+00	0	-0.413200554	-0.646626844	-0.922696012
Nfib	0.04757281	0.00E+00	0	-0.026850256	-0.834582703	-0.924437596
Dlc1	1	0.00E+00	0	0.026345802	-0.745544723	-0.928759499
Hao1	1.1346E-141	0.00E+00	0	-0.193681146	-0.942435718	-0.942045724
G6pc	0	0.00E+00	0	-0.553114265	-1.123057926	-0.942773515
Azgp1	3.92458E-88	0.00E+00	0	0.101779889	-0.797811817	-0.94378422
Gm20319	1.1561E-39	0.00E+00	0	-0.097553921	-0.846983221	-0.949581369
Neat1	1.77814E-47	0.00E+00	0	0.07101377	-1.152864014	-0.955329386
Colec12	2.86236E-05	0.00E+00	0	-0.052321926	-0.828518428	-0.978264733
Tns1	1.09461E-40	0.00E+00	0	0.105831491	-0.892135369	-0.987718562
Uox	1.0741E-112	0.00E+00	0	-0.185737644	-0.864858115	-0.988304311
Hnf1aos1	1	0.00E+00	0	-0.017228284	-0.992074382	-0.988983584
Hsd3b3	0	0.00E+00	0	-0.418854669	-0.94490194	-1.012146685
Hc	3.42548E-90	0.00E+00	0	-0.132457885	-0.98156566	-1.023366682
Bhmt	0	0.00E+00	0	0.572522911	-1.062335369	-1.023406967
C8a	1.06271E-57	0.00E+00	0	-0.121171178	-0.917399798	-1.03807622
Sugct	1	0.00E+00	0	-3.81539E-05	-1.187534365	-1.039216579
Cyp2j5	3.46165E-11	0.00E+00	0	0.038741598	-0.868740163	-1.044174983
B3galt1	0	0.00E+00	0	-0.368649947	-0.804490423	-1.081970654
Pck1	6.05209E-05	0.00E+00	0	0.018970275	-1.434390314	-1.092444667
C9	0	0.00E+00	0	-0.411187459	-1.014434384	-1.093073918
Prr16	0.581226941	0.00E+00	0	0.036658997	-0.974031722	-1.123908918
Gm36264	0	0.00E+00	0	-0.370065692	-1.12709278	-1.126660847
Rhobtb1	1.88226E-14	0.00E+00	0	-0.065376884	-1.112360747	-1.200476464
Pah	3.25965E-70	0.00E+00	0	-0.147682979	-1.128802172	-1.210252904
9030622O[illegible]	0	0.00E+00	0	-0.507837971	-1.146216208	-1.212161731
Slc22a30	0.006750683	0.00E+00	0	-0.035062038	-1.068478719	-1.293770392
Gfra1	0	0.00E+00	0	0.445057987	-1.112284479	-1.322659555
Aass	0	0.00E+00	0	-0.498741864	-1.22472681	-1.329445316
Mug2	1	0.00E+00	0	-0.019790938	-1.219814039	-1.385296303
Igf1	0	0.00E+00	0	-1.17334955	-1.197967366	-1.400483057
Ces3a	1.185E-245	0.00E+00	0	-0.262841691	-1.351681105	-1.460160396
Kynu	6.4034E-07	0.00E+00	0	0.042766783	-1.438031633	-1.564271152
Cmah	8.96709E-12	0.00E+00	0	-0.061855117	-1.403155559	-1.57423311
Sult2a8	1.4776E-203	0.00E+00	0	-0.285503641	-1.783733748	-1.910900895
Ghr	0	0.00E+00	0	-0.345516112	-1.799613353	-1.925247451
Mug1	0	0.00E+00	0	0.356064831	-2.172714983	-2.396986246
Ttc39c	0	0.00E+00	0	-0.494191259	-2.251227846	-2.409915228
Egfr	0	0.00E+00	0	-1.188057741	-2.434921571	-2.832203202

Gene	Adj. P-Value Control vs TCDD	Adj. P-Value Control vs T+S	Log2FC TCDD	-log10(P-value)	Log2FC T+S	-log10(P-value)
Reln	0.00E+00	0	2.120245	#NUM!	2.3827768	#NUM!
Fmo3	0.00E+00	0	1.995999	#NUM!	2.8636125	#NUM!
Airn	0.00E+00	0	1.656621	#NUM!	2.3216102	#NUM!
Cyp1a2	0.00E+00	0	1.599452	#NUM!	1.9927484	#NUM!
Esrrg	0.00E+00	0	1.590254	#NUM!	1.8092504	#NUM!
Nrg1	0.00E+00	0	1.490354	#NUM!	1.9554854	#NUM!
Selenbp1	0.00E+00	0	1.348606	#NUM!	1.8742851	#NUM!
Gm20528	0.00E+00	0	1.111783	#NUM!	0.9987948	#NUM!
Cyp1a1	0.00E+00	0	1.102879	#NUM!	1.4046165	#NUM!
Pvt1	0.00E+00	0	1.072734	#NUM!	0.9545946	#NUM!
Samd4	0.00E+00	0	1.068456	#NUM!	1.2787976	#NUM!
Por	0.00E+00	0	1.056809	#NUM!	1.3648403	#NUM!
Abcc4	0.00E+00	0	1.025759	#NUM!	1.1493599	#NUM!
Ugdh	0.00E+00	0	0.999622	#NUM!	1.2549668	#NUM!
Nfe2l2	0.00E+00	0	0.96537	#NUM!	1.3800852	#NUM!
Tbc1d8	0.00E+00	0	0.964311	#NUM!	1.2259171	#NUM!
Tiparp	0.00E+00	0	0.950408	#NUM!	1.2376086	#NUM!
Them7	0.00E+00	0	0.935454	#NUM!	1.240966	#NUM!
Myom1	0.00E+00	0	0.875006	#NUM!	1.1183673	#NUM!
Gclc	0.00E+00	0	0.861738	#NUM!	1.0569585	#NUM!
Lrmda	0.00E+00	0	0.84167	#NUM!	0.5695547	#NUM!
Eif4g3	0.00E+00	0	0.836642	#NUM!	1.0577722	#NUM!
Robo1	0.00E+00	0	0.812386	#NUM!	1.0486525	#NUM!
Dst	0.00E+00	0	0.806723	#NUM!	1.0980962	#NUM!
Nrip1	0.00E+00	0	0.795856	#NUM!	1.0294764	#NUM!
Cyp2b9	0.00E+00	0	0.753668	#NUM!	1.8739664	#NUM!
Htatip2	0.00E+00	0	0.748587	#NUM!	0.9794391	#NUM!
Ftl1	0.00E+00	0	0.747912	#NUM!	0.6609483	#NUM!
Arhgap10	0.00E+00	0	0.696518	#NUM!	0.5535575	#NUM!
Pik3ap1	0.00E+00	0	0.694917	#NUM!	0.8444553	#NUM!
Zfhx3	0.00E+00	0	0.69187	#NUM!	0.9271392	#NUM!
Cyp2a5	0.00E+00	0	0.687573	#NUM!	0.8492466	#NUM!
Sult5a1	0.00E+00	5.5E-280	0.685232	#NUM!	0.4122448	279.25643
Msrb3	0.00E+00	0	0.675778	#NUM!	0.9888694	#NUM!
Tbcel	0.00E+00	0	0.654365	#NUM!	0.7620655	#NUM!
Osbpl3	0.00E+00	0	0.650514	#NUM!	0.5038106	#NUM!
Tanc1	0.00E+00	0	0.648632	#NUM!	0.8459864	#NUM!
Txnrd1	0.00E+00	0	0.639586	#NUM!	0.5545451	#NUM!
9130230L2	0.00E+00	0	0.639017	#NUM!	0.5028289	#NUM!
Prkca	0.00E+00	0	0.636242	#NUM!	0.8973288	#NUM!
Ahrr	0.00E+00	0	0.633343	#NUM!	0.8826209	#NUM!
Hjurp	0.00E+00	0	0.628918	#NUM!	1.00792	#NUM!
Nipal2	0.00E+00	0	0.620549	#NUM!	0.8072225	#NUM!

Wipf3	0.00E+00	0	0.616544	#NUM!	0.8471584	#NUM!
Glis3	0.00E+00	0	0.612401	#NUM!	0.7901262	#NUM!
Gstm3	0.00E+00	0	0.610492	#NUM!	0.7102214	#NUM!
Chd9	3.34E-230	0	0.582077	229.47577	0.9128097	#NUM!
Abhd6	0.00E+00	0	0.572801	#NUM!	0.814255	#NUM!
Adamts17	0.00E+00	0	0.571826	#NUM!	0.8604853	#NUM!
Npc1	0.00E+00	0	0.563842	#NUM!	0.7396611	#NUM!
Entpd5	0.00E+00	0	0.556441	#NUM!	0.6413151	#NUM!
Cyb5a	0.00E+00	0	0.551305	#NUM!	0.8829937	#NUM!
Lpin2	0.00E+00	0	0.545018	#NUM!	0.9032714	#NUM!
Aig1	0.00E+00	0	0.542168	#NUM!	0.66344	#NUM!
Slc7a11	0.00E+00	0	0.541359	#NUM!	0.3493084	#NUM!
Ppard	0.00E+00	0	0.540847	#NUM!	0.7781614	#NUM!
Bdh1	0.00E+00	0	0.535706	#NUM!	0.6440694	#NUM!
Kcnq1ot1	0.00E+00	0	0.534926	#NUM!	0.8929422	#NUM!
Gsr	0.00E+00	0	0.517295	#NUM!	0.436537	#NUM!
Aox1	3.94E-299	0	0.514634	298.4047	0.8039359	#NUM!
Acacb	0.00E+00	0	0.512022	#NUM!	0.7210727	#NUM!
Hacd3	0.00E+00	0	0.496453	#NUM!	0.6665446	#NUM!
Fabp12	0.00E+00	0	0.496181	#NUM!	0.8150591	#NUM!
Zfp704	0.00E+00	0	0.49298	#NUM!	0.7480151	#NUM!
Hectd2os	0.00E+00	0	0.491707	#NUM!	0.5907544	#NUM!
Rapgef4os1	0.00E+00	0	0.490299	#NUM!	0.5720385	#NUM!
Aopep	0.00E+00	0	0.489956	#NUM!	0.3986239	#NUM!
Ctdspl	0.00E+00	0	0.483297	#NUM!	1.0326777	#NUM!
Cyfip2	0.00E+00	0	0.482436	#NUM!	0.6885267	#NUM!
Patj	0.00E+00	0	0.479373	#NUM!	0.6699844	#NUM!
Abcc3	0.00E+00	0	0.479025	#NUM!	0.6213044	#NUM!
Hsd17b6	1.77E-188	0	0.47558	187.75112	0.7322614	#NUM!
Adh1	0.00E+00	0	0.474963	#NUM!	0.8351908	#NUM!
Zeb2	0.00E+00	7.3E-104	0.474726	#NUM!	0.0925832	103.13792
Xdh	0.00E+00	0	0.467017	#NUM!	0.5864463	#NUM!
Ankrd12	0.00E+00	0	0.46551	#NUM!	0.3736051	#NUM!
Ecpas	0.00E+00	0	0.464931	#NUM!	0.5740751	#NUM!
Acyp2	0.00E+00	0	0.461135	#NUM!	0.5905342	#NUM!
Cyp1b1	0.00E+00	0	0.460035	#NUM!	0.6285474	#NUM!
Pitpnc1	2.90E-226	0	0.45682	225.53725	0.6226619	#NUM!
Nlrp12	0.00E+00	0	0.455547	#NUM!	0.6691555	#NUM!
Etl4	2.03E-294	0	0.454393	293.69191	0.5394225	#NUM!
Eif1a	0.00E+00	0	0.444915	#NUM!	0.5595003	#NUM!
Txnip	0.00E+00	0	0.436471	#NUM!	0.6078105	#NUM!
Fbp1	3.80E-183	0	0.436322	182.42064	0.642387	#NUM!
Srgap2	0.00E+00	0	0.425781	#NUM!	0.5160763	#NUM!
Gramd1c	3.73E-251	0	0.424149	250.4281	0.834632	#NUM!
Hpgd	0.00E+00	0	0.421429	#NUM!	0.7388322	#NUM!
D630045J1	0.00E+00	0	0.419393	#NUM!	0.541904	#NUM!
Tbc1d16	0.00E+00	0	0.417311	#NUM!	0.7673007	#NUM!

Cdk6	0.00E+00	0	0.413372	#NUM!	0.5522482	#NUM!
Metap1d	0.00E+00	0	0.410339	#NUM!	0.573246	#NUM!
Rpl10a	0.00E+00	0	0.410192	#NUM!	0.49928	#NUM!
Arid1b	3.71E-299	0	0.409018	298.43038	0.6228359	#NUM!
Gm37273	0.00E+00	0	0.408999	#NUM!	0.5157344	#NUM!
Cldn14	0.00E+00	0	0.407667	#NUM!	0.6440544	#NUM!
Herc1	0.00E+00	0	0.405647	#NUM!	0.4745326	#NUM!
Fabp1	2.63E-222	0	0.404398	221.58025	0.5657434	#NUM!
Mapre3	2.13E-230	0	0.402724	229.67202	0.5106503	#NUM!
Son	0.00E+00	0	0.402626	#NUM!	0.7314098	#NUM!
Cers6	5.99E-252	0	0.400685	251.22245	0.5390436	#NUM!
Lrp4	0.00E+00	0	0.40024	#NUM!	0.6223026	#NUM!
Dpf3	0.00E+00	0	0.399408	#NUM!	0.5533769	#NUM!
Gtf2i	4.60E-282	0	0.398844	281.33682	0.6040068	#NUM!
Plin2	2.28E-193	5.17E-82	0.395803	192.64254	0.2190401	81.28641
Setbp1	0.00E+00	0	0.395392	#NUM!	0.6877893	#NUM!
Gpnmb	0.00E+00	0	0.395087	#NUM!	0.117997	#NUM!
Ism2	0.00E+00	0	0.391037	#NUM!	0.5266763	#NUM!
2900026A(	4.11E-211	0	0.387711	210.38605	0.6312099	#NUM!
Shroom3	2.19E-130	0	0.384135	129.65857	0.6380669	#NUM!
Mybl1	0.00E+00	0	0.383381	#NUM!	0.6313474	#NUM!
Pde4d	5.54E-149	0	0.379272	148.25631	0.5844664	#NUM!
Plekhm3	0.00E+00	0	0.378908	#NUM!	0.2991839	#NUM!
Notch1	0.00E+00	0	0.37741	#NUM!	0.690665	#NUM!
Lgmn	0.00E+00	3.1E-172	0.377216	#NUM!	0.1182296	171.51009
St3gal1	7.39E-182	0	0.37267	181.1311	0.7053943	#NUM!
Cdh22	0.00E+00	0	0.371575	#NUM!	0.5431764	#NUM!
Nup155	0.00E+00	0	0.369099	#NUM!	0.6882442	#NUM!
Ppm1h	0.00E+00	0	0.369033	#NUM!	0.2378886	#NUM!
Gstm2	0.00E+00	0	0.368443	#NUM!	0.4450003	#NUM!
Sytl5	0.00E+00	0	0.366296	#NUM!	0.5745564	#NUM!
Chrm3	0.00E+00	0	0.36594	#NUM!	0.5510004	#NUM!
Igf2r	0.00E+00	0	0.364658	#NUM!	0.5438407	#NUM!
Lmo7	1.50E-267	0	0.362375	266.82367	0.7610077	#NUM!
Sybu	0.00E+00	0	0.356704	#NUM!	0.4495982	#NUM!
Furin	1.18E-134	0	0.356066	133.9264	0.5579487	#NUM!
Ces1f	2.95E-189	0	0.354483	188.53032	0.4906463	#NUM!
Rock2	9.44E-220	4.1E-166	0.350141	219.02497	0.2219867	165.38308
Ube2e2	1.03E-111	0	0.349732	110.98699	0.5278005	#NUM!
Gsta3	5.54E-131	0	0.34827	130.25612	0.4981207	#NUM!
Pawr	1.27E-174	0	0.346912	173.89747	0.5192564	#NUM!
Parm1	0.00E+00	0	0.345585	#NUM!	0.4388886	#NUM!
Exoc3	0.00E+00	0	0.343394	#NUM!	0.5757104	#NUM!
Arfgef2	9.71E-271	0	0.341542	270.0129	0.4629312	#NUM!
Pdk4	0.00E+00	0	0.341296	#NUM!	0.5405771	#NUM!
Gm34777	0.00E+00	0	0.340262	#NUM!	0.4006621	#NUM!
Me1	1.11E-193	0	0.339704	192.9546	0.4442192	#NUM!

Abr	0.00E+00	2.2E-194	0.338123	#NUM!	0.0892234	193.65136
Tmlhe	1.01E-269	0	0.337481	268.99585	0.5802585	#NUM!
Mast2	8.14E-223	0	0.337038	222.0895	0.4807868	#NUM!
Sgk1	0.00E+00	0	0.334725	#NUM!	0.633247	#NUM!
Tacc2	9.95E-137	0	0.334705	136.00204	0.4124484	#NUM!
Tbc1d1	0.00E+00	0	0.326917	#NUM!	0.4426712	#NUM!
B4galt6	0.00E+00	0	0.326733	#NUM!	0.4386085	#NUM!
Pon3	7.48E-241	0	0.324122	240.12636	0.5259634	#NUM!
4833422C1	0.00E+00	0	0.32405	#NUM!	0.3744803	#NUM!
Ppp1r9a	7.01E-176	0	0.323212	175.15447	0.4275816	#NUM!
Zbtb16	9.51E-66	0	0.322363	65.021697	0.7389816	#NUM!
Lrch1	1.60E-216	2.2E-209	0.320137	215.79515	0.2209459	208.64874
Ddc	3.20E-72	2.01E-79	0.316659	71.494353	0.2133009	78.696802
Gas5	0.00E+00	0	0.313771	#NUM!	0.3564598	#NUM!
Slc1a5	0.00E+00	0	0.31172	#NUM!	0.4634902	#NUM!
Crybg1	2.74E-217	0	0.311472	216.56153	0.4108098	#NUM!
Eya3	1.10E-178	0	0.309711	177.95888	0.4694125	#NUM!
Ahr	4.08E-171	0	0.306999	170.38885	0.650751	#NUM!
Acox2	7.85E-135	0	0.305357	134.10539	0.4350132	#NUM!
Tpr	2.27E-198	0	0.303464	197.64372	0.4075001	#NUM!
Fnip2	4.17E-136	2.93E-13	0.303345	135.37993	0.0628845	12.5333
Snx24	4.70E-131	1.81E-47	0.302468	130.32829	0.1243352	46.741691
Rictor	2.52E-217	0	0.301421	216.59864	0.4111418	#NUM!
Slc25a21	9.00E-88	0	0.299362	87.04593	0.5745464	#NUM!
Myo5b	1.11E-155	0	0.297498	154.95521	0.3820092	#NUM!
Snd1	1.90E-150	0	0.297001	149.72026	0.4032335	#NUM!
Tmem214	3.44E-288	0	0.29674	287.46337	0.4006862	#NUM!
Shtn1	8.35E-182	5.6E-206	0.296567	181.07845	0.2251341	205.25313
Elmo1	2.77E-110	2.52E-64	0.296366	109.55805	0.1250127	63.599357
Jazf1	0.00E+00	0	0.293965	#NUM!	0.4103383	#NUM!
Abcb4	4.59E-160	0	0.292826	159.33846	0.6085906	#NUM!
Snx10	2.44E-224	1.8E-300	0.292501	223.61194	0.2304272	299.73855
Trpm8	0.00E+00	0	0.29203	#NUM!	0.3648879	#NUM!
Zup1	0.00E+00	0	0.289941	#NUM!	0.3312628	#NUM!
Eml4	2.86E-194	0	0.289858	193.54435	0.5759721	#NUM!
Slc8a1	1.38E-294	3.18E-62	0.289368	293.86116	0.0525538	61.496903
Fubp1	1.46E-194	0	0.28894	193.83598	0.3279763	#NUM!
Iqgap1	0.00E+00	1.3E-162	0.287823	#NUM!	0.0891983	161.87182
Fnbp1	9.06E-167	6.01E-14	0.286868	166.04282	0.0607469	13.221045
Tnnc1	0.00E+00	0	0.2865	#NUM!	0.4259348	#NUM!
Tyw1	2.50E-282	0	0.285059	281.60161	0.3289031	#NUM!
Hdac8	2.32E-113	0	0.284789	112.63458	0.3661556	#NUM!
Lncpint	8.53E-169	5.5E-191	0.283826	168.06895	0.2032349	190.25763
Gm42047	1.49E-74	0	0.283627	73.827855	0.5843512	#NUM!
N4bp2l1	2.01E-128	0	0.282778	127.69652	0.3849834	#NUM!
Tmprss2	0.00E+00	0	0.282657	#NUM!	0.3459994	#NUM!
Psme4	3.02E-156	0	0.281346	155.52067	0.3526279	#NUM!

Lilr4b	0.00E+00	1.4E-198	0.281146	#NUM!	0.0618841	197.83869
Pnpla7	5.32E-138	1.72E-24	0.279911	137.2743	0.0789644	23.764365
Gpld1	6.27E-142	0	0.276915	141.20296	0.4414088	#NUM!
Atoh8	1.85E-276	0	0.276869	275.7331	0.4484835	#NUM!
Psap	8.20E-136	2.77E-17	0.276579	135.08628	0.0553593	16.557315
Mgll	2.70E-51	0	0.276113	50.568065	0.6157522	#NUM!
Ranbp2	2.16E-183	0	0.274318	182.66613	0.3252993	#NUM!
B3galnt2	5.83E-193	0	0.274115	192.23457	0.3968619	#NUM!
Pkp4	2.56E-90	0	0.272629	89.591965	0.4132067	#NUM!
Gipc2	0.00E+00	0	0.270774	#NUM!	0.3106617	#NUM!
Pdgfc	2.28E-128	1.22E-84	0.270649	127.6417	0.1320103	83.912573
F8	0.00E+00	0	0.270253	#NUM!	0.3084716	#NUM!
Mertk	2.51E-83	1.81E-54	0.268174	82.601139	0.1073322	53.742155
Smg1	4.31E-124	1.1E-133	0.2679	123.366	0.1915213	132.97076
Serinc3	3.48E-191	1.1E-219	0.266813	190.45831	0.194505	218.95645
Fyb	0.00E+00	5.3E-82	0.265836	#NUM!	0.0629245	81.275629
Antxr2	3.47E-131	0	0.265065	130.46018	0.5176631	#NUM!
Magi3	1.15E-77	0	0.264376	76.93863	0.6748527	#NUM!
Rb1cc1	3.45E-150	0	0.264289	149.46235	0.3101827	#NUM!
Gstt2	7.08E-182	0	0.263063	181.15023	0.4858232	#NUM!
Map4	1.64E-154	4.1E-121	0.262698	153.78438	0.1603144	120.38409
Nbr1	2.63E-150	0	0.262202	149.58039	0.3744584	#NUM!
Slc35f5	2.08E-297	0	0.260042	296.68194	0.3175532	#NUM!
Rab11fip3	1.26E-159	0	0.259443	158.89942	0.3804942	#NUM!
Yars	1.36E-194	0	0.257906	193.86499	0.3940476	#NUM!
Gnl3	0.00E+00	0	0.256815	#NUM!	0.3296317	#NUM!
Gab2	2.33E-126	0.000625	0.256102	125.63244	0.0356872	3.204432
Rapgef2	1.04E-95	0	0.254662	94.984146	0.3623003	#NUM!
Mrtfb	6.75E-88	3.66E-09	0.253998	87.170876	0.0695943	8.4370377
Map4k3	6.27E-122	1.9E-130	0.253864	121.20288	0.1835118	129.71589
Dock2	0.00E+00	5.2E-71	0.253702	#NUM!	0.0422654	70.284121
Sertad2	3.50E-131	0	0.253601	130.4563	0.3158696	#NUM!
Lilrb4a	0.00E+00	3.8E-171	0.252498	#NUM!	0.0611	170.42536
Gm37359	0.00E+00	0	0.252057	#NUM!	0.4227362	#NUM!
Fmo1	8.55E-127	0	0.251498	126.06791	0.3331828	#NUM!
Tapt1	1.24E-68	0	0.25144	67.905597	0.4100243	#NUM!
Vtcn1	6.65E-247	0	0.251196	246.17714	0.4642542	#NUM!
Slc25a3	5.31E-159	0	0.250544	158.27478	0.3160873	#NUM!
Tgfa	4.89E-131	0	0.24978	130.311	0.4171357	#NUM!
Myo1f	0.00E+00	6.9E-99	0.248936	#NUM!	0.041453	98.161887
Scfd2	4.91E-124	0	0.248346	123.30866	0.2958302	#NUM!
Xxylt1	3.76E-268	0	0.246273	267.42433	0.3049351	#NUM!
App	1.10E-226	7.4E-243	0.245395	225.95723	0.1719977	242.1292
Rplp1	0.00E+00	0	0.244574	#NUM!	0.1712019	#NUM!
Cd84	0.00E+00	9.93E-90	0.241783	#NUM!	0.0432311	89.002871
Tardbp	3.32E-149	0	0.241667	148.47924	0.2965342	#NUM!
Sfswap	7.61E-152	0	0.240905	151.11852	0.3061067	#NUM!

Bptf	4.25E-128	0	0.240311	127.37162	0.2978154	#NUM!
Gm42031	2.42E-121	0	0.240074	120.6164	0.3246602	#NUM!
Hnrnpa3	2.89E-146	0	0.239785	145.53969	0.3658854	#NUM!
Dop1a	1.43E-146	0	0.238917	145.84592	0.3488953	#NUM!
Ccs	3.38E-105	0	0.238406	104.47145	0.4247113	#NUM!
Trak2	1.44E-169	0	0.2381	168.84155	0.4016506	#NUM!
D10Wsu10	0.00E+00	0	0.236358	#NUM!	0.1731604	#NUM!
Phip	1.09E-106	6.28E-79	0.234322	105.96085	0.135228	78.202007
Sec61a2	1.84E-153	0	0.232823	152.73456	0.3135063	#NUM!
Tnfaip8l1	1.24E-126	0	0.232051	125.90758	0.3980682	#NUM!
Mad1l1	2.27E-111	0	0.231987	110.64365	0.2850301	#NUM!
Tcirg1	1.51E-176	2.1E-171	0.231594	175.82166	0.1327142	170.67422
Fndc3b	6.21E-93	0	0.230046	92.20669	0.3329525	#NUM!
Comt	3.43E-78	0	0.22967	77.464197	0.423627	#NUM!
Stard13	4.83E-69	0	0.229398	68.316134	0.4666605	#NUM!
Galnt10	4.67E-260	0	0.229129	259.33088	0.3042213	#NUM!
Smchd1	6.13E-121	0	0.228987	120.21239	0.2860926	#NUM!
Bin1	4.01E-198	0	0.228798	197.39676	0.2890769	#NUM!
Hacd2	1.60E-75	3.2E-276	0.228575	74.794703	0.2988741	275.49226
Pdzrn3	6.66E-39	0	0.226101	38.176342	0.6281267	#NUM!
Washc2	1.07E-116	0	0.225896	115.97211	0.3050448	#NUM!
Pick1	6.22E-254	0	0.225499	253.20651	0.3236986	#NUM!
Dock10	0.00E+00	1.09E-64	0.224861	#NUM!	0.0413102	63.960701
Man1a	5.91E-84	0	0.224474	83.228501	0.3004481	#NUM!
Cyp2a4	0.00E+00	0	0.224295	#NUM!	0.1862424	#NUM!
Pkm	0.00E+00	0	0.223607	#NUM!	0.2738519	#NUM!
Abcg8	7.11E-110	0	0.222207	109.14801	0.3403376	#NUM!
Lyn	2.44E-51	1.19E-08	0.222071	50.612959	-0.0316236	7.9233931
Apc	3.71E-105	0	0.219983	104.4304	0.2892954	#NUM!
Runx1	0.00E+00	4.09E-91	0.219662	#NUM!	0.0486867	90.388709
Rtn4	2.34E-194	3.8E-156	0.217481	193.63079	0.1222425	155.4154
Kdm5a	1.86E-112	0	0.217404	111.73059	0.2764934	#NUM!
Tmcc3	8.10E-103	2E-20	0.217288	102.09138	0.0587859	19.69828
Snx30	4.90E-163	0	0.216726	162.30972	0.2679787	#NUM!
Grb2	1.49E-98	2.79E-87	0.214502	97.825664	0.1382776	86.555089
Eif4a2	3.69E-139	0	0.214464	138.43338	0.2664292	#NUM!
Rbm47	3.45E-83	2.34E-62	0.214447	82.461733	0.1228302	61.630639
Frrs1	1.25E-142	8.4E-118	0.211518	141.90383	0.1156029	117.07549
BC005537	5.17E-75	1.82E-42	0.210762	74.286731	0.0933907	41.740483
Prpf39	2.76E-119	0	0.210419	118.5593	0.3249943	#NUM!
Hexa	1.32E-256	3.8E-213	0.210278	255.88072	0.1227651	212.42014
Slc46a3	9.03E-151	0	0.210135	150.04447	0.3349588	#NUM!
Setd4	4.53E-246	0	0.209297	245.34343	0.3681536	#NUM!
Akap9	2.26E-80	0	0.208849	79.646609	0.3628961	#NUM!
Mid1	1.80E-187	0	0.208831	186.74427	0.2956547	#NUM!
Mettl15	1.20E-151	0	0.208577	150.91922	0.2687922	#NUM!
Parp9	6.09E-106	0	0.208349	105.21528	0.2967713	#NUM!

Dgkh	1.79E-123	0	0.206989	122.74616	0.2786239	#NUM!
Slc16a7	9.35E-91	0	0.206783	90.029148	0.4101948	#NUM!
Iars	1.68E-212	0	0.206199	211.77383	0.2602253	#NUM!
Pacs2	1.03E-166	0	0.205793	165.98724	0.2450676	#NUM!
Mpp7	1.93E-95	0	0.205758	94.714395	0.2861333	#NUM!
Ralgapb	7.20E-115	0	0.205083	114.14268	0.2619386	#NUM!
Tpm1	6.92E-218	0	0.20502	217.15993	0.2936075	#NUM!
Tafa2	1.20E-239	0	0.204764	238.92246	0.6547764	#NUM!
Pan2	1.47E-136	0	0.204363	135.8337	0.259887	#NUM!
Cep192	0.00E+00	0	0.203992	#NUM!	0.2398426	#NUM!
Coro7	4.83E-169	0	0.203723	168.31625	0.312846	#NUM!
Fgfr2	2.04E-68	1	0.203341	67.690923	0.0034681	0
Fbxw9	0.00E+00	0	0.203319	#NUM!	0.3502599	#NUM!
Arl6ip5	1.94E-160	0	0.202465	159.71287	0.3230111	#NUM!
Ssbp2	3.57E-176	0	0.202316	175.44697	0.3224102	#NUM!
Acot4	4.04E-220	0	0.202189	219.39353	0.299158	#NUM!
Ptprc	0.00E+00	7.5E-89	0.202164	#NUM!	0.0469328	88.124897
Cabin1	1.07E-157	0	0.201971	156.97105	0.3000634	#NUM!
Gsg1l	0.00E+00	0	0.200652	#NUM!	0.2856726	#NUM!
1110051M	2.35E-245	0	0.199702	244.62806	0.2414433	#NUM!
Ccdc85c	1.30E-152	0	0.199495	151.88769	0.3198427	#NUM!
Lrrc4	1.45E-120	4.8E-129	0.199153	119.83903	0.1349775	128.31647
Epg5	2.56E-116	0	0.198882	115.59208	0.3324851	#NUM!
Mmp12	0.00E+00	7E-190	0.198423	#NUM!	0.0585349	189.15211
Cyp2c68	4.62E-52	0	0.198383	51.335633	0.6105883	#NUM!
D1Ertd622	1.34E-161	1.3E-174	0.198154	160.87194	0.1356877	173.89885
Sirpa	0.00E+00	4.1E-142	0.198129	#NUM!	0.0532601	141.3831
0610010F0	5.62E-138	0	0.197932	137.25057	0.2599496	#NUM!
Etv6	2.40E-51	0	0.197662	50.619718	0.4179123	#NUM!
Utp14a	3.35E-155	0	0.197099	154.47558	0.2775114	#NUM!
Tmem62	0.00E+00	0	0.197029	#NUM!	0.2888442	#NUM!
Rpsa	2.71E-192	1.1E-225	0.196404	191.56662	0.1460551	224.94911
Eif5	5.02E-91	0	0.195069	90.29964	0.2820042	#NUM!
Trpv4	0.00E+00	0	0.194516	#NUM!	0.2588423	#NUM!
Fbxw8	4.93E-95	0	0.19417	94.307084	0.3783455	#NUM!
Pard3bos3	1.15E-275	2.3E-231	0.193921	274.93874	0.1247953	230.63797
Maml3	5.88E-56	1	0.193836	55.230507	-0.0071557	0
Igfbp1	4.33E-69	0	0.193589	68.363494	0.6193114	#NUM!
Rpl3	3.45E-283	0	0.193404	282.46248	0.2310677	#NUM!
Ctsd	1.11E-264	2.4E-117	0.192947	263.95435	0.0720521	116.62702
Vti1a	1.13E-61	0	0.192882	60.948381	0.277855	#NUM!
Tfrc	4.03E-185	0	0.192363	184.3949	0.2497982	#NUM!
Kdm3a	1.66E-101	0	0.191839	100.77866	0.294239	#NUM!
Iffo2	9.10E-194	0	0.191754	193.04101	0.3473828	#NUM!
Aldh2	1.35E-61	0	0.191005	60.870102	0.291455	#NUM!
Hspd1	1.15E-134	0	0.190212	133.94069	0.2363642	#NUM!
Xrcc4	1.20E-67	0	0.190174	66.920359	0.3208309	#NUM!

Elovl5	2.06E-43	0	0.189539	42.686009	0.6947698	#NUM!	
Srsf10	5.28E-120	0	0.18947	119.2771	0.4065168	#NUM!	
Lrrc20	6.97E-82	0	0.189046	81.157075	0.3079753	#NUM!	
4732463B0	0.00E+00	0	0.189027	#NUM!	0.273154	#NUM!	
Rps29	8.00E-238	5.2E-268	0.188805	237.09704	0.136665	267.28808	
Sult2a2	0.00E+00	0	0.188754	#NUM!	0.6081696	#NUM!	
Actb	1.38E-164	1.9E-104	0.188038	163.86052	0.0926439	103.71692	
Slc22a27	0.00E+00	0	0.187674	#NUM!	0.4120269	#NUM!	
Eef1a1	2.26E-101	0	0.186808	100.64573	0.238439	#NUM!	
Ccdc93	4.15E-111	0	0.185996	110.38167	0.2595102	#NUM!	
Myh9	3.73E-64	5.79E-58	0.185978	63.428843	0.1130394	57.237315	
Pxk	3.95E-78	0	0.185883	77.403278	0.2820495	#NUM!	
Mecr	2.16E-110	0	0.185446	109.66628	0.2851175	#NUM!	
Cyp39a1	4.57E-72	6.1E-284	0.184681	71.34008	0.2471945	283.21457	
Rif1	7.42E-77	0	0.183806	76.129638	0.2920241	#NUM!	
Sel1l3	2.40E-72	0	0.183662	71.619766	0.305919	#NUM!	
Eefsec	1.17E-64	1.62E-60	0.183522	63.932466	0.1238041	59.79081	
Prkcb	3.75E-258	3.66E-44	0.18332	257.42591	0.0301419	43.436991	
Dync1h1	1.94E-62	5.57E-37	0.183018	61.713221	0.0973212	36.253924	
Gm41804	0.00E+00	0	0.182876	#NUM!	0.2464467	#NUM!	
Cblb	4.17E-45	1	0.181554	44.379846	-0.0082477		0
Swt1	2.29E-78	0	0.181337	77.640575	0.2928786	#NUM!	
Itga9	3.12E-226	2.25E-58	0.181331	225.50537	0.0400099	57.646907	
Ctps2	6.76E-174	0	0.18106	173.16974	0.2223451	#NUM!	
Herc2	1.00E-73	0	0.179433	72.99853	0.2719658	#NUM!	
Gm43700	0.00E+00	0	0.178339	#NUM!	0.4581927	#NUM!	
Sf3b2	1.76E-102	0	0.178158	101.75538	0.2627705	#NUM!	
Rpl36	8.87E-189	0	0.178095	188.05225	0.2466069	#NUM!	
Riok2	6.13E-113	0	0.176405	112.2122	0.2203454	#NUM!	
Ikbke	2.27E-137	0	0.176373	136.64398	0.2394725	#NUM!	
Lpl	0.00E+00	1.6E-275	0.176066	#NUM!	0.0927617	274.80696	
Rhbdd1	1.20E-84	3.4E-298	0.17583	83.921554	0.2290617	297.47315	
Lgals3	0.00E+00	7.8E-114	0.175711	#NUM!	0.0341553	113.10978	
Pitpnm2	3.42E-54	3.7E-236	0.175708	53.466103	0.2604473	235.43548	
Mal2	3.60E-101	0	0.175149	100.44401	0.2682671	#NUM!	
Mdm2	1.92E-60	1.5E-276	0.175029	59.71728	0.243988	275.8206	
Arhgef10l	1.20E-53	4.8E-189	0.174963	52.919656	0.2242947	188.31518	
Pdcd11	5.93E-117	0	0.174752	116.22699	0.2465735	#NUM!	
Ctss	0.00E+00	7.4E-80	0.174271	#NUM!	0.0337151	79.131005	
Sorbs3	1.52E-115	0	0.173373	114.81707	0.2196057	#NUM!	
Ptges	0.00E+00	0	0.173101	#NUM!	0.3827475	#NUM!	
Tgfbr2	5.63E-171	0	0.171963	170.2498	0.2190546	#NUM!	
Gm43449	1.47E-228	0	0.171946	227.83218	0.294214	#NUM!	
Ascc2	4.98E-94	0	0.170816	93.302739	0.2286964	#NUM!	
Malat1	2.64E-132	0	0.170611	131.57765	0.4860367	#NUM!	
Kpna4	5.01E-54	2.99E-09	0.170587	53.30002	0.048977	8.5237291	
Plcl2	8.49E-53	1.94E-07	0.170573	52.071031	0.0482957	6.7116822	

Fyb2	2.50E-52	4.4E-246	0.170132	51.60167	0.2422978	245.36114
Gm13657	0.00E+00	0	0.169105	#NUM!	0.3032202	#NUM!
Dgkz	4.80E-105	8.6E-108	0.168079	104.31864	0.1050625	107.06513
Stk40	1.65E-49	2.7E-298	0.167786	48.781984	0.2596157	297.57601
Rptor	1.67E-55	4.7E-266	0.167568	54.776162	0.2452711	265.33041
Rtn4rl2	0.00E+00	0	0.166218	#NUM!	0.2055153	#NUM!
Marveld3	0.00E+00	0	0.165934	#NUM!	0.2166667	#NUM!
Usp4	1.52E-76	0	0.165629	75.817939	0.2917632	#NUM!
Mast4	7.11E-57	0	0.165585	56.148062	0.2601092	#NUM!
Fam185a	5.21E-134	0	0.164623	133.28298	0.247426	#NUM!
Ctsb	3.61E-57	1	0.164418	56.443044	-0.0073744	0
Mpped2	0.00E+00	0	0.163908	#NUM!	0.2988627	#NUM!
Polr1a	1.41E-104	0	0.163808	103.85019	0.2413585	#NUM!
Zmym2	9.28E-61	1.98E-41	0.163323	60.03252	0.0952863	40.702754
Inpp5d	5.02E-302	4.12E-51	0.162625	301.29937	0.0281192	50.38545
Hipk1	2.82E-61	5.7E-292	0.162436	60.550373	0.2296382	291.24594
Gsta1	0.00E+00	0	0.162196	#NUM!	0.1027924	#NUM!
Prdm16	0.00E+00	0	0.161925	#NUM!	0.1100861	#NUM!
Tango2	7.04E-48	0	0.161699	47.152323	0.3137275	#NUM!
Gm26740	3.59E-277	1.43E-59	0.161691	276.44464	0.0338993	58.843801
Rftn1	1.64E-270	2.09E-49	0.161662	269.78634	0.0275592	48.67891
Osgin1	1.75E-30	3.8E-255	0.161612	29.758176	0.3175193	254.42303
Irak2	2.08E-38	1	0.16135	37.681193	0.0155587	0
Gm36419	8.80E-129	0	0.160971	128.05551	0.400039	#NUM!
Ypel3	4.69E-112	0	0.160955	111.32852	0.2162224	#NUM!
Gm15738	1.20E-111	1.23E-33	0.160324	110.92057	0.054293	32.909703
Stk10	2.69E-254	3E-200	0.159895	253.57082	0.092413	199.52932
Rps10	7.66E-191	1.8E-204	0.159843	190.1156	0.1164839	203.73325
Srsf2	8.30E-53	1.5E-215	0.159495	52.0811	0.2120939	214.8167
Plbd2	2.69E-173	0	0.159468	172.57046	0.2256053	#NUM!
Myo9b	7.63E-50	1.1E-15	0.159083	49.117594	0.0512153	14.95735
Cdk11b	5.48E-75	4.4E-242	0.159073	74.261579	0.2039197	241.35693
Bcl9	9.58E-89	0	0.158984	88.018637	0.2593429	#NUM!
Pou2f2	1.28E-258	6.46E-82	0.158967	257.89241	0.0436443	81.189485
Wdr91	7.47E-69	1.7E-257	0.15895	68.126937	0.2085619	256.76729
Celf2	2.28E-36	7.01E-09	0.158017	35.642206	0.0512968	8.1545788
Dclk3	1.78E-212	0	0.157922	211.74987	0.2191693	#NUM!
Itpk1	3.59E-57	0	0.157552	56.445029	0.3162477	#NUM!
Micu1	7.13E-54	1.2E-246	0.157542	53.146793	0.2292538	245.9126
Gm47283	1.38E-26	0	0.157396	25.861474	0.3692004	#NUM!
Cbr1	0.00E+00	0	0.157208	#NUM!	0.3147423	#NUM!
Nedd4l	6.48E-51	0	0.156993	50.188709	0.374936	#NUM!
Stimate	6.38E-122	0	0.156704	121.19543	0.2659752	#NUM!
Rps26	3.09E-173	1E-168	0.156276	172.50993	0.1043573	167.98476
Prom1	0.00E+00	0	0.155721	#NUM!	0.3910168	#NUM!
Stab2	1.15E-107	8.55E-08	0.155501	106.93935	0.0340508	7.0682549
Tmem135	8.86E-45	1.3E-162	0.155368	44.052464	0.205826	161.87202

Pde6c	0.00E+00	0	0.155256	#NUM!	0.2618881	#NUM!
1700042O1	9.99E-104	2.49E-62	0.155238	103.00036	0.0772267	61.60363
Mitf	1.13E-08	2.2E-15	0.155218	7.9466415	-0.0590351	14.658551
Wdr3	7.69E-156	0	0.155031	155.11404	0.1954422	#NUM!
Dram2	7.54E-66	0	0.154768	65.122671	0.2585967	#NUM!
5330439B1	0.00E+00	0	0.154214	#NUM!	0.191937	#NUM!
Arhgap15	1.98E-185	2.34E-43	0.154056	184.7035	0.0389482	42.630595
Gm44507	0.00E+00	8.8E-134	0.154021	#NUM!	0.0499473	133.05579
Hes1	0.00E+00	3.1E-178	0.153387	#NUM!	0.0747047	177.5054
Lrrk1	5.39E-76	0	0.15307	75.268431	0.2107307	#NUM!
Gon4l	1.32E-58	1.3E-237	0.152928	57.879762	0.2161991	236.89953
Kif21a	1.22E-36	6.3E-268	0.152652	35.915005	0.2687475	267.20242
Ipo7	1.40E-90	2.3E-287	0.152645	89.853153	0.1942869	286.63485
Atg2a	3.31E-82	3.4E-276	0.152366	81.47978	0.2007069	275.46497
Pum2	1.45E-54	4.83E-36	0.152259	53.839838	0.0880543	35.316421
C2cd2	5.19E-33	1	0.151628	32.284562	0.0185274	0
Cox7c	3.22E-86	0	0.151619	85.491909	0.2272449	#NUM!
Picalm	1.45E-34	0.000119	0.15147	33.837449	-0.0297451	3.9241359
Tbcd	4.05E-60	1.1E-293	0.151262	59.392954	0.22603	292.94807
Ptpn4	2.15E-59	0	0.151186	58.666923	0.3128201	#NUM!
Mgat5	3.88E-10	1	0.150597	9.4108223	-0.005606	0
Usp18	6.72E-206	0	0.149524	205.17271	0.2613326	#NUM!
Ttc41	6.92E-198	0	0.149381	197.15972	0.2218613	#NUM!
Cd5l	5.18E-290	1.21E-94	0.149364	289.28567	0.0436002	93.918383
Mindy1	1.93E-50	6.9E-292	0.149296	49.714557	0.2442374	291.16081
Mpp6	1.80E-39	5.4E-159	0.148754	38.745917	0.2112932	158.2668
Ttc27	2.68E-77	8.4E-247	0.148635	76.572199	0.1847069	246.07608
Rxra	2.57E-26	2E-281	0.147824	25.590295	0.3318112	280.69226
Apbb1ip	1.15E-301	1.2E-70	0.147494	300.93949	0.0313966	69.919588
Tubgcp5	3.46E-160	0	0.147458	159.46047	0.2225304	#NUM!
Ptcd3	1.26E-83	0	0.147353	82.901137	0.2074706	#NUM!
Pla2g7	0.00E+00	2.4E-250	0.147065	#NUM!	0.0696415	249.62588
Pgap1	1.08E-88	0	0.147024	87.968404	0.2575518	#NUM!
Elf2	2.15E-33	3.41E-05	0.146944	32.668226	-0.0352968	4.4672275
Rhou	7.38E-84	1.9E-285	0.146663	83.131894	0.1951826	284.7215
Mdn1	3.26E-79	0	0.146306	78.486719	0.223836	#NUM!
Maml2	8.63E-139	5.63E-20	0.146049	138.06378	0.025877	19.249359
Cplane1	8.63E-96	0	0.146017	95.063867	0.2117042	#NUM!
Zfp791	2.76E-135	0	0.145615	134.55928	0.3213123	#NUM!
Mroh2a	0.00E+00	0	0.145571	#NUM!	0.1860339	#NUM!
AI463229	3.10E-67	0	0.145327	66.509254	0.2357706	#NUM!
Top2b	2.17E-64	4.5E-216	0.145315	63.66381	0.1823881	215.34779
Hmgb2	5.38E-214	0	0.1453	213.26938	0.195503	#NUM!
Micos10	3.58E-71	0	0.145037	70.445544	0.2603285	#NUM!
Ifi207	5.07E-281	2.08E-84	0.14441	280.29522	0.0384725	83.682978
Mmp27	0.00E+00	5.3E-168	0.144053	#NUM!	0.0493795	167.27583
Heatr1	1.82E-174	0	0.143843	173.7403	0.1822708	#NUM!

Serpinb6a	1.08E-97	0	0.14357	96.967616	0.189095	#NUM!
Srpk1	4.90E-83	0	0.143222	82.309947	0.2483075	#NUM!
4732419C1	1.21E-94	0	0.143149	93.9181	0.2229644	#NUM!
Myof	0.00E+00	1.57E-73	0.143125	#NUM!	0.024677	72.802884
Gtf2ird1	2.95E-32	0	0.142712	31.530209	0.3357767	#NUM!
Ntf3	1.37E-208	0	0.142677	207.86408	0.3334753	#NUM!
Derl1	8.04E-87	0	0.142631	86.094502	0.2395736	#NUM!
Hgfac	1.23E-56	0	0.142618	55.911847	0.3979365	#NUM!
Traf3	1.47E-45	7E-214	0.142393	44.832449	0.2031298	213.15522
Impact	2.70E-89	9.2E-295	0.142379	88.569127	0.1872467	294.03706
Hip1	1.03E-234	6.39E-43	0.142009	233.98927	0.0259287	42.194433
Nfx1	9.02E-45	2.9E-222	0.14155	44.044895	0.2141683	221.54236
Terb1	3.57E-271	0	0.141077	270.44781	0.1701422	#NUM!
Nrn1	3.04E-64	0	0.140902	63.516941	0.2300474	#NUM!
Abhd4	5.07E-162	0	0.140771	161.29518	0.1920992	#NUM!
H6pd	1.60E-38	2.5E-264	0.140769	37.795856	0.2386639	263.60068
Pip4k2a	2.64E-211	2.6E-47	0.14034	210.57878	0.0315054	46.584586
Kif16b	1.82E-24	3.3E-226	0.140174	23.740368	0.2413383	225.48028
Vav3	1.04E-198	2.54E-33	0.140071	197.98338	0.0242074	32.594447
Rai1	9.47E-45	4.6E-201	0.140041	44.023778	0.2107341	200.33422
Fmnl2	7.38E-04	3.8E-106	0.140016	3.1319311	-0.1193727	105.41609
Tm4sf4	1.02E-65	0	0.139953	64.991739	0.2404437	#NUM!
Phkb	1.88E-33	4.2E-159	0.139926	32.726752	0.2025714	158.38135
Polk	1.33E-92	0	0.139871	91.875948	0.2342567	#NUM!
Lacc1	5.17E-246	0	0.139775	245.28689	0.3125127	#NUM!
Actr3	5.23E-55	7.17E-14	0.139766	54.281469	0.0448326	13.144779
Cdc42bpg	2.06E-220	0	0.139415	219.68686	0.206833	#NUM!
Gtpbp2	7.39E-80	7.6E-264	0.139221	79.131647	0.1754244	263.11859
Csf3r	3.74E-273	3.67E-57	0.139094	272.42719	0.0267164	56.435727
Itfg1	4.46E-47	2.6E-235	0.139083	46.350618	0.209633	234.5781
Mon2	1.93E-45	8.6E-190	0.139064	44.715194	0.1999232	189.06722
Rnf217	3.48E-23	0	0.138797	22.458278	0.3871602	#NUM!
Tafa5	1.98E-176	0	0.138629	175.70394	0.2501719	#NUM!
Trappc9	1.54E-29	1.2E-185	0.138247	28.812595	0.2242795	184.9248
9130409I2	1.28E-135	1.9E-131	0.138211	134.89207	0.0964467	130.71115
Cerk	4.86E-253	4.69E-36	0.138207	252.31373	0.021865	35.328515
Eif2a	7.81E-101	0	0.138199	100.10759	0.1836787	#NUM!
Utp20	1.47E-96	0	0.13802	95.832628	0.2153668	#NUM!
Slc22a3	4.96E-202	0	0.137657	201.30418	0.1974607	#NUM!
Entpd1	4.48E-293	1.88E-78	0.137476	292.34826	0.0300342	77.724883
2410131K1	1.10E-214	0	0.137379	213.95865	0.1902071	#NUM!
Mfn2	1.67E-65	0	0.136567	64.776044	0.2667076	#NUM!
Ubr2	4.37E-31	1.5E-193	0.136524	30.359387	0.2252662	192.82032
Btg3	2.29E-135	0	0.136128	134.64102	0.1766803	#NUM!
Adgb	7.46E-234	3.8E-138	0.13604	233.12707	0.0544351	137.42344
Fgf21	0.00E+00	0	0.135822	#NUM!	0.1955205	#NUM!
Mrc1	4.07E-110	8.37E-23	0.135801	109.39019	0.034285	22.077126

Gm33543	3.58E-30	7.9E-222	0.135673	29.446525	0.218401	221.10344
Scaf8	7.17E-43	9.2E-28	0.135585	42.14418	0.0796793	27.036322
Gsta2	1.17E-46	0.000797	0.135385	45.931587	0.0327557	3.0985382
Arhgap22	3.70E-242	7.42E-61	0.135284	241.43223	0.0311216	60.129336
Myo9a	3.17E-66	1	0.13489	65.499417	0.0081192	0
Adgre5	2.21E-263	1.74E-68	0.134812	262.65638	0.0339682	67.760693
Cdkn1a	4.04E-120	0	0.134749	119.39361	0.1666358	#NUM!
Agpat3	1.79E-28	2.2E-232	0.134486	27.74607	0.2463554	231.66496
Ubxn4	8.39E-36	1.6E-142	0.13425	35.07648	0.1840335	141.80099
Atm	9.32E-81	2.94E-57	0.134234	80.03047	0.0763369	56.53221
Abcg1	0.00E+00	2.6E-107	0.134211	#NUM!	0.0323246	106.58897
Adamts14	0.00E+00	0	0.134176	#NUM!	0.3165624	#NUM!
Ptpn9	2.57E-33	0	0.134033	32.590503	0.2594284	#NUM!
Tox	4.14E-23	0	0.133954	22.383307	0.4387185	#NUM!
H2afj	6.46E-213	0	0.133036	212.18981	0.1789143	#NUM!
4833420G1	2.04E-50	0	0.133006	49.690072	0.2352575	#NUM!
Nop56	3.48E-104	0	0.132848	103.45842	0.1850499	#NUM!
1700086O(	1.69E-152	0	0.132337	151.77093	0.1657645	#NUM!
Dph5	4.06E-71	0	0.132325	70.39107	0.2107338	#NUM!
Ublcp1	2.05E-90	1.1E-271	0.132306	89.688523	0.1726182	270.94942
Gars	1.15E-139	0	0.132182	138.9388	0.1863562	#NUM!
Gtf2a2	4.10E-70	0	0.131795	69.386993	0.2130286	#NUM!
Gpr137b	1.76E-267	4.73E-82	0.131491	266.75367	0.0300186	81.325421
Lars	1.91E-55	7.8E-251	0.130757	54.717833	0.1961424	250.11056
Sh3gl1	4.85E-106	0	0.130483	105.31397	0.1978844	#NUM!
Clcn3	3.43E-36	3.2E-176	0.130292	35.465012	0.1847276	175.49095
Lrpprc	1.02E-29	1.2E-140	0.129838	28.991033	0.1935	139.93242
Wipf1	7.78E-206	1.89E-34	0.129806	205.10917	0.0242328	33.723885
Baz2b	7.26E-18	1	0.12961	17.13923	0.0138486	0
Ano6	9.52E-38	0.004852	0.129407	37.021408	0.0299702	2.3140713
Alkbh1	2.21E-73	2.1E-274	0.129244	72.655219	0.1859194	273.67811
Gm42711	0.00E+00	0	0.129162	#NUM!	0.4099201	#NUM!
Tent4b	5.98E-32	1	0.128837	31.223247	-0.0029308	0
Rbm26	1.58E-34	4.8E-126	0.12852	33.800271	0.1632624	125.31444
Gm12498	0.00E+00	0	0.128484	#NUM!	0.0789381	#NUM!
Orc3	4.19E-52	2.9E-222	0.128412	51.377819	0.1841113	221.53271
Slc9a9	1.12E-114	3.09E-24	0.128283	113.94907	0.0273833	23.509574
Man2b1	1.36E-86	2.1E-301	0.128079	85.867065	0.1697649	300.67495
Erbb3	1.02E-38	0	0.128058	37.989511	0.2458877	#NUM!
4930581F2	6.23E-30	8.87E-14	0.127943	29.205573	0.057537	13.052138
Gadd45g	2.49E-99	0	0.127822	98.604103	0.3011314	#NUM!
Wwtr1	1.32E-38	6.7E-288	0.126842	37.878946	0.23655	287.17671
Syk	1.60E-277	9.16E-47	0.126241	276.79453	0.0204646	46.038296
Meis1	1.09E-74	0	0.125803	73.964104	0.2018238	#NUM!
Cnbd2	3.03E-179	8.4E-168	0.125497	178.51799	0.0848026	167.07378
Fhit	1.02E-11	1.1E-295	0.125195	10.990532	0.3637507	294.95282
Traf5	9.07E-64	1E-228	0.125114	63.042474	0.166466	227.98811

Atp9a	4.78E-39	8.5E-236	0.12496	38.320224	0.2078166	235.06849
Ndor1	4.25E-107	0	0.124506	106.37142	0.1660641	#NUM!
H2-D1	2.25E-68	1.97E-25	0.124392	67.647404	0.0440614	24.705642
Sult1d1	1.27E-38	0	0.124276	37.894787	0.2676966	#NUM!
Cbr3	0.00E+00	0	0.124048	#NUM!	0.1560277	#NUM!
Brip1os	2.62E-26	1.01E-14	0.123968	25.580896	0.0599188	13.996882
Ccdc125	4.75E-61	1.5E-247	0.123863	60.323345	0.172526	246.81067
Atg4c	4.12E-48	4.5E-208	0.123844	47.385597	0.1787749	207.35114
Knop1	1.41E-70	7.5E-251	0.123573	69.851368	0.1675817	250.12335
Ext1	3.06E-11	0	0.12335	10.5149	0.363701	#NUM!
Fto	4.58E-21	2.1E-276	0.123269	20.33891	0.2564761	275.6697
Abcc5	1.62E-200	3.9E-142	0.123082	199.79086	0.0606271	141.41267
Sdk1	2.76E-87	0	0.122711	86.559183	0.2723228	#NUM!
Xpnpep1	2.34E-91	0	0.122625	90.630948	0.2055173	#NUM!
Prep	4.60E-70	3E-253	0.122465	69.336889	0.1737211	252.52503
Ksr2	2.50E-218	3.03E-40	0.122172	217.6017	0.017346	39.519273
Enpp1	7.23E-62	1.8E-292	0.122135	61.140844	0.1951175	291.75593
Slf1	3.41E-78	0	0.12166	77.466809	0.1897571	#NUM!
Nqo1	0.00E+00	0	0.121566	#NUM!	0.2033792	#NUM!
Mpp2	0.00E+00	0	0.121453	#NUM!	0.1885015	#NUM!
Rpl9	1.28E-116	0	0.121206	115.89151	0.1471689	#NUM!
Cul4a	1.56E-32	2.1E-144	0.121138	31.807157	0.1754119	143.67956
Igsf11	5.85E-38	0	0.121084	37.23295	0.2986849	#NUM!
Gm12153	0.00E+00	1E-158	0.120486	#NUM!	0.056602	157.98703
Sh3kbp1	1.64E-180	3.48E-27	0.120433	179.78389	0.0223755	26.457882
Dapk2	4.76E-45	0	0.120426	44.322732	0.2551887	#NUM!
Fbxl5	3.58E-96	5.48E-67	0.120368	95.446602	0.0634855	66.261492
Cox10	1.89E-42	1.5E-285	0.120275	41.723527	0.2096447	284.8181
Tmem51	3.19E-73	1.52E-32	0.120154	72.495946	0.0504824	31.816751
Fus	1.16E-23	1	0.119594	22.935272	0.0160976	0
Lgr4	2.21E-13	0.012792	0.119429	12.656121	0.0458774	1.8930486
Rgl1	1.59E-123	2.23E-28	0.119378	122.79783	0.0245716	27.651565
Kdm2b	1.63E-50	2.3E-241	0.118905	49.787192	0.1818573	240.64407
Slc13a3	0.00E+00	0	0.118819	#NUM!	0.2170858	#NUM!
Hnrnpa2b1	5.22E-19	2.3E-94	0.118788	18.282369	0.1544518	93.637913
Epb41l1	4.41E-207	4E-139	0.118535	206.35516	0.0639704	138.40142
Soat1	3.33E-283	5.37E-59	0.118507	282.47807	0.0230266	58.269853
Stau2	5.80E-23	0	0.118498	22.236908	0.2975789	#NUM!
Man1c1	1.16E-219	7.74E-45	0.117986	218.93718	0.0240214	44.111199
Desi2	3.70E-22	1.3E-229	0.117846	21.431884	0.2552948	228.87273
Ccdc162	6.89E-62	0	0.117604	61.161937	0.2915116	#NUM!
Zfp973	3.28E-60	0	0.117585	59.484669	0.225354	#NUM!
Nptx1	0.00E+00	0	0.11756	#NUM!	0.1831394	#NUM!
Nop58	5.51E-67	0	0.117467	66.258491	0.1877295	#NUM!
Rasa2	2.57E-60	1.7E-201	0.117414	59.589246	0.151223	200.76297
Morc2a	5.24E-49	3.5E-220	0.117388	48.280638	0.177803	219.45073
Smarcad1	7.36E-52	1.6E-240	0.117361	51.133212	0.1776212	239.79893

Arhgap25	3.71E-271	9.35E-52	0.117078	270.43117	0.0196654	51.02902
Mef2a	4.19E-15	1	0.117075	14.378098	0.005769	0
Eef2	6.47E-52	1.9E-204	0.117022	51.189191	0.1558139	203.72551
A330015K(	1.33E-300	0	0.116928	299.87646	0.1696542	#NUM!
Vars	5.08E-166	0	0.116807	165.29403	0.149494	#NUM!
Slc43a2	6.25E-47	1	0.116723	46.203935	0.0046483	0
Uhrf1bp1l	3.46E-30	0	0.116186	29.461143	0.3424662	#NUM!
Gm3776	0.00E+00	0	0.116152	#NUM!	0.0791816	#NUM!
Gpr107	9.95E-39	8.1E-216	0.116084	38.002392	0.1877372	215.09352
Col4a3	4.10E-241	0	0.115535	240.38752	0.217564	#NUM!
Rpl10	7.48E-149	0	0.11526	148.1263	0.1839372	#NUM!
Wdr7	3.26E-25	1	0.115172	24.4873	0.0242288	0
Wwox	2.61E-07	0	0.115117	6.5829141	0.5101702	#NUM!
Oplah	1.28E-52	1.1E-179	0.114755	51.892881	0.155389	178.9438
Lpin3	1.61E-231	0	0.114472	230.79256	0.1668152	#NUM!
Zfp612	1.65E-57	2.6E-225	0.114376	56.78147	0.1646162	224.59334
Eif4g2	5.24E-32	5.1E-174	0.114286	31.28107	0.1710928	173.2959
Fli1	1.66E-153	2.6E-21	0.114252	152.77979	0.0227437	20.585492
Gmds	1.08E-129	0	0.114201	128.96475	0.1465959	#NUM!
Cdk17	1.45E-25	1	0.114089	24.839417	0.0064559	0
Zfp64	5.91E-38	9.8E-226	0.114012	37.22854	0.1936336	225.00847
Gm10475	0.00E+00	0	0.113742	#NUM!	0.0835896	#NUM!
Ankrd11	6.93E-14	1	0.113467	13.159108	0.0261973	0
Pdss1	1.05E-141	0	0.113435	140.97739	0.169154	#NUM!
Alb	2.66E-07	0	0.113328	6.5743629	0.4558405	#NUM!
Wdr43	3.05E-47	1.6E-168	0.113309	46.515631	0.1519479	167.80122
Xylt1	1.58E-164	7.88E-09	0.113165	163.80232	0.0120148	8.1035737
Snx13	1.88E-27	1.04E-11	0.113024	26.725673	0.0531511	10.983796
Riok3	1.47E-25	3E-131	0.113005	24.832963	0.1577561	130.51993
Evl	1.89E-224	2.51E-33	0.112935	223.72443	0.0166213	32.600589
Xpo6	5.11E-26	4.9E-161	0.112715	25.291493	0.1735431	160.30771
Dnmt3a	4.19E-04	1.94E-30	0.112478	3.3776347	-0.0809542	29.712032
Gm41409	0.00E+00	0	0.112277	#NUM!	0.1592644	#NUM!
Gm14964	0.00E+00	1.1E-263	0.111962	#NUM!	0.0669668	262.9767
Gramd4	7.34E-72	0	0.111957	71.134295	0.1873937	#NUM!
Epn1	8.44E-50	0	0.111953	49.07359	0.1942884	#NUM!
Cd300a	4.92E-246	4.88E-42	0.111687	245.30846	0.0196405	41.311727
Anks1	1.99E-30	1.27E-14	0.111533	29.700069	0.0541388	13.897187
Mapre2	1.11E-83	1.92E-39	0.111522	82.95362	0.0472168	38.716468
Abcd2	1.70E-121	0	0.111205	120.77011	0.2791748	#NUM!
Sidt2	1.24E-52	5.8E-267	0.111013	51.906413	0.1791119	266.2364
Ldhd	6.17E-96	0	0.110842	95.20952	0.1996789	#NUM!
Pigx	3.22E-108	0	0.110677	107.4918	0.19709	#NUM!
Ddb1	1.63E-59	7.8E-298	0.110392	58.787273	0.1812892	297.10659
Unc93b1	9.83E-59	3.05E-32	0.110392	58.007414	0.0491418	31.516379
Gdf15	1.74E-126	1.9E-113	0.11036	125.75834	0.0761328	112.72995
Cpb2	6.00E-03	6.5E-224	0.1103	2.221768	0.2696969	223.18788

Taf1a	2.46E-75	3.9E-281	0.1101	74.608203	0.1603147	280.41103
Brwd1	4.52E-18	1.9E-109	0.110021	17.345098	0.1632817	108.72899
Ankrd6	1.89E-199	1.4E-147	0.109943	198.7236	0.0647993	146.86811
Ssb	2.19E-53	2.7E-291	0.109841	52.659557	0.184452	290.57134
Tbxas1	1.85E-157	2.74E-22	0.109774	156.73328	0.0199685	21.562895
Lamc1	3.16E-35	1.8E-197	0.109645	34.50051	0.1729543	196.73494
Trmt1	1.60E-70	0	0.109562	69.795802	0.225943	#NUM!
Tnpo1	2.99E-24	1	0.10951	23.524765	0.0048358	0
Mrtfa	1.24E-16	1	0.109504	15.904955	0.0136967	0
Secisbp2l	1.77E-37	2.4E-297	0.109477	36.751485	0.2123102	296.6256
Mcph1	3.48E-36	2.3E-181	0.109178	35.458989	0.1708889	180.63898
Mical2	3.16E-15	3.3E-199	0.108883	14.50041	0.243266	198.48542
Gart	1.15E-87	6.1E-250	0.10881	86.94027	0.1415048	249.21282
Slc20a1	1.88E-86	0	0.108567	85.726771	0.1997819	#NUM!
Tex14	2.02E-15	1.95E-33	0.108306	14.694969	-0.0777161	32.709527
Cltc	1.83E-18	1.6E-115	0.108272	17.737004	0.1511186	114.7936
Afg3l2	4.29E-32	1.6E-247	0.108165	31.367996	0.2052978	246.80725
Dcaf8	2.89E-21	8.55E-96	0.107992	20.538558	0.1446444	95.068056
Myo5a	3.09E-161	2.09E-41	0.107883	160.51025	0.0239115	40.679925
Prim2	9.82E-73	7.9E-224	0.107517	72.008027	0.1446283	223.10206
Cemip2	3.79E-68	8E-275	0.107386	67.421811	0.1521922	274.09948
Lcp1	1.02E-09	1	0.107194	8.9912798	-0.0123551	0
Zfp981	3.22E-38	8.6E-251	0.107192	37.491875	0.1806009	250.06527
Rars2	1.56E-37	7.8E-175	0.107149	36.806699	0.1602472	174.1093
Cd44	2.93E-193	3.73E-43	0.10704	192.53383	0.022753	42.42875
Rapgef4	2.66E-37	0	0.106762	36.57557	0.3768413	#NUM!
Cdk7	4.60E-37	3.7E-280	0.10657	36.336932	0.2059065	279.4375
Atg2b	1.43E-30	0	0.106557	29.845119	0.2717659	#NUM!
Ttc37	1.66E-35	0	0.106485	34.779158	0.2522079	#NUM!
Nrros	3.61E-161	1.71E-14	0.106303	160.44305	0.0157545	13.766573
Nomo1	4.81E-92	0	0.10617	91.31744	0.1792057	#NUM!
2810403D2	2.25E-37	3E-154	0.106123	36.647421	0.1426267	153.52028
44450	5.33E-88	2.3E-257	0.105846	87.273625	0.1355993	256.632
Wdr25	4.00E-54	2.4E-234	0.105825	53.398408	0.1596304	233.62282
1200007C1	0.00E+00	4.7E-270	0.105755	#NUM!	0.0778181	269.33109
Rab31	1.07E-175	6.33E-65	0.105674	174.9715	0.0353774	64.198583
Cyp2b13	6.54E-301	0	0.105163	300.1845	0.2811619	#NUM!
Abcc1	1.01E-259	6.84E-59	0.104765	258.99686	0.0200771	58.164722
Arhgap45	2.41E-159	1.62E-19	0.104425	158.61833	0.0189892	18.791033
Rab8b	1.13E-197	3.27E-33	0.104139	196.94853	0.0190853	32.485548
Sugp1	5.25E-57	0	0.104074	56.279681	0.2364021	#NUM!
Arap2	2.61E-35	1.7E-218	0.103938	34.582761	0.1769232	217.77541
Rsad1	8.20E-61	9.3E-300	0.103778	60.086197	0.1729484	299.03234
Hnrnpdl	1.02E-24	1.6E-141	0.103672	23.992841	0.1530961	140.79851
Ctse	3.66E-121	2.12E-98	0.103647	120.43708	0.0625504	97.6746
Rnf123	2.76E-40	2.1E-181	0.103329	39.559238	0.1535584	180.67717
Trappc12	2.07E-50	4.4E-179	0.103205	49.684161	0.1394797	178.35586

Zmynd12	5.77E-170	0	0.102683	169.23894	0.1431858	#NUM!
Gm47990	0.00E+00	0	0.102626	#NUM!	0.1429815	#NUM!
Dnajc10	6.17E-65	1.3E-264	0.102589	64.209461	0.1592215	263.89755
Slc25a22	3.26E-17	0	0.102532	16.486454	0.4006403	#NUM!
Brca2	1.25E-77	6.7E-259	0.10225	76.902465	0.14469	258.17227
Arhgap31	2.66E-134	6.88E-26	0.101967	133.57447	0.0226226	25.162603
Thrap3	2.00E-23	1E-159	0.101957	22.698851	0.1771712	158.98171
Nt5e	8.46E-110	0	0.101606	109.07249	0.2805732	#NUM!
4632427E1	9.02E-29	0	0.101564	28.044765	0.2482267	#NUM!
Tomm40	3.97E-38	1.7E-270	0.101329	37.401709	0.1916315	269.76012
Fryl	2.65E-18	1	0.100924	17.576395	0.0260358	0
Trim30a	7.26E-45	1	0.100852	44.139006	0.0030119	0
Frmd6	1.30E-53	9.1E-252	0.100653	52.887633	0.1603614	251.0406
Alg14	4.86E-22	1.6E-158	0.100605	21.313007	0.172563	157.78386
Ano10	4.41E-57	0	0.100563	56.355832	0.1863947	#NUM!
Pik3r5	1.12E-242	1.75E-35	0.100237	241.9513	0.0138303	34.758124
Pik3r3	1.93E-218	0	0.10021	217.71534	0.1564796	#NUM!
Ago2	4.59E-27	3.2E-144	0.100019	26.337756	0.1391865	143.50071
Pkn1	8.15E-47	1.12E-14	0.100001	46.088642	0.035201	13.952467
Gadd45b	0.00E+00	0	0.099862	#NUM!	0.2060184	#NUM!
Cdk14	2.77E-127	1.89E-10	0.099721	126.55676	0.014248	9.7246763
Sorl1	5.69E-202	5.36E-33	0.099714	201.24504	0.0188063	32.270856
Zfp746	3.83E-43	9.6E-264	0.099644	42.417058	0.17653	263.01836
Lcp2	1.44E-219	1.25E-51	0.099578	218.84275	0.0230742	50.902734
Sf3b3	2.00E-38	1.1E-183	0.099566	37.699515	0.1528924	182.97168
Ddx5	1.12E-06	8.68E-86	0.099421	5.9498436	0.1470751	85.061616
Ctsa	4.36E-36	1.58E-24	0.099413	35.36099	0.0480515	23.802399
Samhd1	2.14E-51	2.13E-06	0.099356	50.669965	0.0206774	5.6717827
Pinx1	9.51E-68	5E-274	0.099101	67.021868	0.1502436	273.29976
Ppid	3.02E-76	0	0.098908	75.519724	0.1866354	#NUM!
Ddx39b	7.03E-22	1.7E-131	0.098869	21.153243	0.1519264	130.75745
Ctr9	1.06E-43	4.7E-271	0.098545	42.974386	0.182648	270.32914
Lgr5	3.73E-13	6E-215	0.098261	12.427869	0.2219176	214.21984
Sars	2.50E-54	3.2E-211	0.098243	53.601769	0.1421597	210.49544
Birc6	3.39E-08	1.67E-96	0.098211	7.4694286	0.1512969	95.777029
Xpo5	5.17E-66	3.1E-214	0.098208	65.286579	0.1319186	213.51489
Lekr1	3.39E-246	0	0.098146	245.46948	0.1305043	#NUM!
1110038B1	2.33E-166	0	0.098142	165.63319	0.1275121	#NUM!
Sys1	6.76E-70	4.1E-236	0.097876	69.169868	0.1368413	235.38714
Crybg2	4.70E-264	0	0.097683	263.32747	0.1261141	#NUM!
Micu3	2.58E-35	1.4E-139	0.096929	34.589213	0.1302793	138.84775
Mndal	4.18E-69	4.77E-06	0.09681	68.378617	0.0156546	5.3213736
Snhg20	6.35E-47	1E-237	0.096646	46.196982	0.1586772	236.97893
Nol10	2.46E-59	1.5E-194	0.096435	58.609559	0.1308184	193.81117
Cd9	1.93E-51	5.37E-20	0.096326	50.713353	0.0378506	19.269992
Nedd9	6.07E-115	2.99E-52	0.096312	114.21684	0.0360701	51.524979
Trps1	5.04E-148	2.84E-30	0.096017	147.29726	0.018126	29.546847

Pop1	4.55E-67	3.1E-282	0.095999	66.342005	0.1536479	281.51288
Tert	1.88E-71	2.5E-232	0.095742	70.72631	0.1338813	231.60985
Rpl39	9.96E-165	1.5E-123	0.095551	164.00195	0.057578	122.82398
Copb1	4.57E-29	8.2E-192	0.095453	28.3402	0.1692674	191.08457
Naa25	9.52E-31	2.3E-212	0.095228	30.021363	0.1731633	211.64477
C2cd5	4.12E-43	7.5E-257	0.095168	42.385364	0.170537	256.12345
Ythdf3	2.63E-16	1	0.095138	15.579472	0.0121782	0
Gm5150	6.91E-196	2.31E-76	0.09511	195.16061	0.0286291	75.636696
Tfdp1	6.62E-43	3.7E-178	0.095079	42.179331	0.1362277	177.43132
Atp8a1	3.91E-46	1	0.095003	45.40762	0.0054498	0
Cyc1	9.60E-81	0	0.094947	80.017694	0.154744	#NUM!
Slx4ip	2.76E-22	6.3E-145	0.094878	21.558496	0.1484364	144.20033
Dyrk1a	9.12E-13	1	0.094827	12.039805	0.0083704	0
Gm10501	1.15E-119	6.38E-82	0.094791	118.94003	0.0543196	81.19511
Plcg2	1.02E-263	1.2E-205	0.094535	262.99033	0.0570785	204.91773
Zbtb11	1.51E-16	1.11E-95	0.094467	15.819604	0.1405183	94.955058
Aoah	6.97E-171	9.96E-59	0.094408	170.15691	0.0242529	58.001668
Copa	1.40E-15	1.1E-256	0.094298	14.85485	0.2270222	255.94284
Txn2	1.73E-32	2.6E-225	0.09422	31.76278	0.1747365	224.57954
Sfxn1	3.71E-10	2.6E-249	0.094216	9.4302521	0.2718462	248.59105
Gm34654	2.78E-127	0	0.094178	126.55543	0.1393688	#NUM!
Git2	2.56E-15	1.4E-104	0.094159	14.591244	0.1371112	103.8429
Net1	9.93E-32	0	0.094137	31.002974	0.2290299	#NUM!
Rtn4ip1	1.71E-60	3.5E-256	0.093935	59.767478	0.148217	255.45514
Phf20l1	8.31E-12	1	0.093782	11.080391	0.0305541	0
Ncf2	1.35E-233	6.83E-53	0.093671	232.87011	0.0212634	52.165759
St18	1.52E-158	1.99E-19	0.09337	157.81892	0.0116564	18.701435
Trrap	3.18E-18	1.5E-160	0.093322	17.497501	0.1655237	159.8238
Msn	2.68E-189	2.4E-27	0.09329	188.57204	0.0165695	26.619924
Chst11	3.66E-198	2.23E-17	0.093205	197.43652	0.0097054	16.650796
Mta3	2.17E-32	4.3E-136	0.092878	31.663953	0.1260408	135.36836
Mtpap	3.52E-44	9.6E-172	0.092811	43.453795	0.1319382	171.01848
Apex2	1.52E-11	0	0.092761	10.818994	0.3262096	#NUM!
Anapc10	7.77E-33	4.3E-180	0.092715	32.109301	0.1491671	179.36909
Filip1l	3.48E-34	0.000131	0.092699	33.458086	0.0264524	3.8815633
Eef1b2	4.12E-108	1.2E-276	0.092591	107.38467	0.1164218	275.90494
Basp1	6.30E-229	7.12E-44	0.092567	228.20044	0.0165509	43.147414
Rab24	1.60E-55	3.4E-224	0.092435	54.796866	0.134118	223.47097
Mast1	5.16E-182	1.6E-153	0.092375	181.28763	0.0627771	152.79827
Ppan	3.87E-107	0	0.092323	106.41193	0.1690533	#NUM!
Nemf	3.71E-18	1.9E-201	0.092248	17.430826	0.1979635	200.71555
Snhg15	5.83E-205	0	0.092218	204.23397	0.1251404	#NUM!
Tcerg1	1.59E-24	1.6E-245	0.092197	23.799858	0.195223	244.78727
Echdc3	8.71E-11	2.2E-149	0.092161	10.060196	0.1832984	148.65031
Zmiz2	1.10E-75	0	0.092	74.959967	0.1673056	#NUM!
Rcc2	1.27E-57	7.85E-35	0.091832	56.895907	0.0492944	34.105349
Aldh1a7	6.00E-27	4E-145	0.091775	26.221936	0.1416684	144.39588

Pgm3	9.84E-52	0	0.09165	51.007051	0.2224641	#NUM!
Plaa	7.57E-25	1.2E-147	0.09161	24.12063	0.1487438	146.92089
Morrbid	3.45E-124	8.75E-13	0.091485	123.46213	0.013931	12.058117
Hk3	6.34E-201	2.44E-29	0.091388	200.19797	0.0139783	28.612559
Serpine1	2.01E-220	0	0.091335	219.69618	0.2660414	#NUM!
Itpkb	1.72E-126	1.36E-06	0.091316	125.76453	0.0102518	5.8656513
Tpcn2	2.41E-113	7.21E-25	0.09128	112.61721	0.0203411	24.142055
Unk	2.66E-36	1.5E-161	0.091252	35.574594	0.1359849	160.8294
Mettl27	5.02E-58	2.1E-185	0.091236	57.299199	0.1235289	184.67035
Spag1	6.52E-66	0	0.091195	65.185532	0.1582601	#NUM!
Tmem243	1.98E-08	1	0.091132	7.7030946	-0.0041912	0
Nampt	1.20E-13	1	0.091074	12.921606	0.0163403	0
Zfp943	7.44E-36	3.3E-185	0.09079	35.128447	0.1477119	184.48115
Zfp282	8.28E-47	5.9E-225	0.090727	46.082125	0.1471737	224.22991
Cdip1	8.09E-11	1.8E-249	0.090551	10.091852	0.2923137	248.75247
Ercc6l2	2.57E-28	3.1E-126	0.090196	27.590087	0.1301726	125.50917
Stx8	1.83E-11	1	0.090052	10.738296	0.0071277	0
Rnf128	4.06E-16	8.68E-93	0.089925	15.391613	0.1259224	92.06162
Sap30	4.02E-269	0	0.089777	268.39627	0.1405676	#NUM!
Sqstm1	6.83E-11	1	0.089696	10.165852	-0.000769	0
Anapc1	1.59E-27	2.4E-178	0.089626	26.799064	0.15971	177.62658
Epb41l2	1.37E-60	1	0.089518	59.86292	0.0096709	0
Prkag1	1.65E-31	7.7E-186	0.089467	30.782362	0.1485527	185.11589
Stradb	1.73E-47	8.4E-271	0.089341	46.761142	0.1666884	270.07332
Gtdc1	1.59E-05	3.8E-253	0.089335	4.797561	0.2774068	252.42546
Col4a5	9.33E-89	0	0.089244	88.030279	0.1892275	#NUM!
Aqp9	1.31E-07	5.8E-289	0.089148	6.8811851	0.3374204	288.23419
Ints2	3.97E-85	0	0.088899	84.401058	0.1749694	#NUM!
Mlkl	3.12E-143	0	0.088881	142.50644	0.1345235	#NUM!
Ubash3b	3.01E-142	6.72E-22	0.088875	141.52117	0.0178383	21.172545
Ucp2	0.00E+00	0	0.088831	#NUM!	0.1364751	#NUM!
Araf	1.27E-27	1.1E-164	0.088718	26.894744	0.1490818	163.97042
Igsf5	7.12E-14	6.4E-123	0.088557	13.147221	0.170668	122.1954
Xndc1	3.97E-49	3.1E-183	0.088326	48.401752	0.1236475	182.51434
F5	1.64E-06	1.2E-130	0.088218	5.7855125	0.1970838	129.91278
Pik3cd	4.63E-199	2.45E-27	0.08807	198.33396	0.0144574	26.611542
Ltn1	3.00E-28	4.5E-202	0.088026	27.523228	0.1592923	201.34707
Map4k4	4.77E-17	1	0.08755	16.321229	0.0223575	0
Rgs1	2.39E-232	1.78E-69	0.087418	231.62135	0.0208771	68.749369
Dennd4a	1.20E-06	9.36E-36	0.087277	5.9193462	-0.0903862	35.028612
Uvssa	6.27E-27	1E-156	0.087049	26.202892	0.1434744	155.99032
Srsf4	1.47E-11	1.12E-69	0.086795	10.832486	0.1287678	68.949689
Tars2	1.06E-66	3.4E-252	0.086766	65.973791	0.1313013	251.46821
Arhgef5	1.59E-49	7.1E-244	0.086754	48.799118	0.1481216	243.14704
Prpf8	2.54E-41	2.6E-242	0.086528	40.59479	0.1540001	241.59175
Ik	1.39E-25	5.9E-240	0.08648	24.857381	0.175814	239.22581
Nudt5	1.69E-53	1.8E-190	0.086352	52.77194	0.1233206	189.74546

Slc16a11	2.49E-164	0	0.086343	163.60315	0.1449074	#NUM!
Actn1	2.91E-23	4.3E-188	0.086294	22.535661	0.1528162	187.37107
Slc30a7	4.34E-26	1.5E-124	0.086248	25.362548	0.1242847	123.83519
Ptbp3	1.33E-09	1.79E-09	0.086243	8.8764625	-0.0430602	8.7474625
Srrt	1.10E-18	2.5E-277	0.086226	17.959026	0.2160199	276.59569
Psma1	8.00E-44	3.3E-281	0.086212	43.096948	0.1675449	280.47919
Bsg	3.89E-45	3.5E-242	0.08602	44.409629	0.1458737	241.45919
Gca	7.24E-97	3.7E-271	0.086008	96.140269	0.117966	270.42716
Msr1	4.46E-151	4.12E-40	0.085942	150.35059	0.0213934	39.385378
Rpl4	2.31E-72	1.1E-244	0.085885	71.635722	0.1189646	243.96997
Rhbdd2	1.21E-68	9.3E-277	0.085711	67.915987	0.1374915	276.0302
Cd74	1.68E-188	4.73E-58	0.085632	187.77462	0.0253264	57.32506
Ccdc148	2.44E-95	2.53E-64	0.085451	94.612389	0.0386933	63.59763
Ly6e	6.36E-109	6.48E-66	0.085381	108.19636	0.042867	65.188475
Tmsb4x	5.02E-157	1.65E-33	0.085291	156.29949	0.0224146	32.782117
1810064F2	3.01E-37	0.607841	0.08513	36.521253	0.0129101	0.2162103
A730090N1	8.01E-226	0	0.085089	225.09618	0.1468746	#NUM!
Synpo	1.53E-91	0	0.085001	90.814358	0.1527805	#NUM!
Tnrc6a	6.64E-06	0.001306	0.084562	5.1778051	-0.0266526	2.8840986
Rab3gap2	7.73E-18	2.7E-156	0.084541	17.111677	0.1437946	155.57617
Atp5b	8.97E-24	4.2E-206	0.084485	23.047381	0.1709783	205.37329
Fam129b	1.36E-222	1.5E-102	0.084468	221.86763	0.0290478	101.83628
Csf2rb	2.93E-119	5.21E-08	0.084447	118.53386	0.011678	7.2828904
Ppcdc	1.94E-28	3.3E-210	0.084209	27.712232	0.1576366	209.47537
Adgre1	1.21E-141	1.96E-45	0.083932	140.91898	0.0243149	44.708285
Sympk	4.94E-34	1.7E-172	0.083844	33.306352	0.1358804	171.75922
Parp6	7.34E-46	2.9E-165	0.083818	45.134024	0.116299	164.53425
Edem1	3.35E-15	7.2E-301	0.08352	14.474763	0.2462934	300.14317
Ggcx	7.44E-39	0	0.083375	38.12814	0.2164935	#NUM!
Iars2	2.83E-14	8.4E-93	0.083309	13.54891	0.131977	92.075627
Mcts1	1.90E-26	1.4E-172	0.083296	25.721246	0.1489161	171.86007
Cct6a	2.66E-38	1.9E-178	0.083226	37.574832	0.126734	177.7307
Dalrd3	2.28E-68	0	0.083202	67.641737	0.1574079	#NUM!
Eif2s2	1.12E-20	1.1E-170	0.083158	19.949258	0.1537483	169.97798
Axin2	1.06E-88	0	0.083021	87.974851	0.1410255	#NUM!
Supt16	1.90E-18	3.8E-103	0.082828	17.721854	0.1233117	102.42566
Irak1	1.81E-32	2.3E-189	0.082525	31.742077	0.1356978	188.64098
Eef1g	8.33E-40	4.4E-200	0.082486	39.07953	0.1344757	199.35831
Adora1	6.35E-31	1	0.082314	30.19753	0.0007729	0
Mt1	2.31E-86	0	0.082288	85.636117	0.1791516	#NUM!
Wdfy4	6.94E-146	4.03E-29	0.08223	145.15887	0.0175203	28.395001
Acer2	1.21E-79	0	0.082192	78.915788	0.1503731	#NUM!
Lmbr1	6.15E-14	1.1E-81	0.082154	13.211034	0.1247916	80.959506
Cd300lb	1.34E-224	7.73E-31	0.081929	223.87217	0.0101349	30.11194
Klhdc2	7.90E-53	1.7E-213	0.081809	52.102644	0.1255142	212.75755
Slc35a3	3.59E-10	1.3E-121	0.08162	9.4447605	0.1586796	120.89438
Hnrnpl	1.05E-15	2.9E-114	0.081403	14.980008	0.1359403	113.53952

Tinag	2.60E-281	0	0.081402	280.58498	0.1146684	#NUM!
Gulo	4.12E-11	7.1E-80	0.081395	10.384885	0.1277223	79.148714
Gcfc2	5.50E-42	2.8E-213	0.081359	41.259299	0.134558	212.55009
Arfgap2	1.21E-28	2.3E-215	0.08126	27.915947	0.1564993	214.63477
Stxbp4	5.52E-31	4E-232	0.081137	30.258443	0.1630241	231.39679
Ankrd44	4.50E-05	1	0.081095	4.3465094	-0.0025069	0
Med25	8.02E-70	7.6E-275	0.080987	69.095563	0.1311911	274.1175
Tsc22d3	6.08E-15	6.33E-64	0.08096	14.216334	0.1507299	63.198442
Ifi204	9.76E-138	1.2E-29	0.080842	137.01048	0.0168076	28.921801
Snhg12	1.28E-73	4.2E-214	0.080791	72.893395	0.1070636	213.37286
Prkcd	3.49E-21	1	0.080594	20.457794	-0.0005116	0
Dtd1	1.87E-15	1.1E-104	0.08054	14.727247	0.1298971	103.947
Itgb5	1.91E-16	0.009322	0.080479	15.719598	0.0252388	2.0304968
Notum	1.08E-38	5.1E-278	0.080406	37.968034	0.1692728	277.29401
Trim2	5.69E-09	1.8E-265	0.080361	8.2447055	0.2746291	264.75088
Ikbkg	3.83E-11	4.47E-92	0.080239	10.416688	0.1340085	91.349905
Cd274	1.09E-127	8.48E-37	0.080213	126.9639	0.0199732	36.071503
Stab1	3.75E-64	7.13E-06	0.079771	63.426434	0.0155964	5.1468084
Tuba8	0.00E+00	0	0.079739	#NUM!	0.1249641	#NUM!
Sp100	1.34E-63	1	0.079678	62.871539	0.0077504	0
Copg1	5.35E-54	7.5E-277	0.079654	53.271818	0.1437917	276.12216
Map1lc3b	2.54E-32	1.8E-141	0.079362	31.596013	0.1171043	140.75136
Dhdds	1.77E-27	2E-202	0.079322	26.75087	0.1535216	201.70381
Flna	4.99E-137	4.69E-23	0.07926	136.30184	0.0172889	22.329276
Arhgef2	4.71E-229	1.1E-131	0.079115	228.32724	0.0371581	130.95284
Ikzf1	1.20E-135	5.79E-22	0.079092	134.92155	0.0152093	21.237415
Cebpz	9.37E-23	1.7E-197	0.079062	22.028467	0.1652609	196.77492
Usp37	4.94E-21	1.2E-182	0.078706	20.306659	0.157047	181.91708
Nckap1l	4.35E-197	5.88E-36	0.078597	196.36119	0.0155754	35.230439
Hdac9	8.34E-75	9.16E-21	0.078484	74.078651	0.022042	20.037915
Cherp	2.24E-34	2E-154	0.078441	33.649484	0.1202988	153.70521
Mettl7a1	5.92E-17	6.5E-102	0.078346	16.227661	0.1226922	101.18679
Gm14296	2.96E-29	2.4E-194	0.078333	28.529057	0.1423406	193.61519
Bet1	6.09E-26	3.1E-120	0.078263	25.215463	0.113434	119.51408
Nek9	3.15E-21	1.3E-132	0.078261	20.501735	0.1265299	131.86967
Glyr1	3.11E-12	2.1E-185	0.078172	11.506979	0.1885087	184.67011
Slc11a1	1.83E-203	1.42E-45	0.078103	202.73696	0.0182318	44.849002
Senp2	3.25E-18	3.5E-173	0.077917	17.487502	0.1528402	172.45471
Tnfaip8l3	1.86E-177	0	0.077876	176.73073	0.1098875	#NUM!
Nme2	1.31E-55	9.5E-179	0.077754	54.882415	0.1051187	178.02322
Abi1	4.13E-03	1.04E-13	0.077705	2.383527	-0.0496512	12.983221
Tbc1d22a	3.61E-07	1	0.077695	6.442061	0.0052278	0
Enc1	2.33E-177	0	0.077687	176.6327	0.1278143	#NUM!
Cdkn1b	2.53E-57	5.7E-236	0.077576	56.597553	0.1226425	235.24787
Elmsan1	6.14E-11	2.3E-111	0.07748	10.211585	0.1379498	110.64036
Nob1	1.39E-43	2E-183	0.077441	42.857498	0.1161691	182.69777
Tfb2m	2.70E-57	1E-235	0.077417	56.569001	0.1263723	234.99179

Mettl1	3.38E-76	1.3E-285	0.077395	75.471067	0.1276267	284.88091
Csf1r	9.05E-163	3.18E-48	0.077354	162.04318	0.0207975	47.497876
Gm17494	1.08E-88	4.52E-69	0.077349	87.96757	0.0491697	68.345311
Idh3b	2.27E-47	1.8E-175	0.077205	46.643162	0.1127701	174.73848
Exosc10	3.95E-32	6.7E-180	0.077172	31.403185	0.1318651	179.17266
Hps5	4.32E-26	7E-130	0.077128	25.364692	0.1150441	129.15668
Atp6v0d2	2.54E-75	0.000313	0.077052	74.594563	0.0101916	3.5045923
Dse	1.88E-111	3.42E-23	0.076911	110.72604	0.0173066	22.466099
Pbdc1	1.07E-32	4.3E-135	0.076895	31.970805	0.1097658	134.36369
Slc15a3	1.39E-200	4.07E-26	0.076767	199.85598	0.0104616	25.390571
Tlr4	1.72E-172	6.19E-39	0.07674	171.76523	0.0178016	38.208338
Cfb	2.80E-26	1.5E-133	0.07661	25.553535	0.1188944	132.83036
Ube2k	1.34E-05	1	0.0766	4.8720289	0.0131291	0
Rint1	2.18E-19	9.9E-199	0.07658	18.662257	0.1624111	198.00292
Prmt9	1.24E-36	1E-142	0.076538	35.905748	0.1073002	141.99621
Ndufa10	1.21E-28	1E-165	0.076352	27.916324	0.1305372	164.99777
Cluh	7.58E-10	1E-207	0.076321	9.1203248	0.214126	206.98419
Tbx3os1	4.07E-24	2.4E-280	0.076292	23.390879	0.2148152	279.62775
Gm1976	2.25E-28	6.2E-159	0.076279	27.647401	0.1301304	158.20417
Hivep1	1.41E-02	1.01E-09	0.076212	1.851664	-0.03717	8.9955584
Dnaja2	9.22E-17	6.6E-124	0.076209	16.035178	0.1334187	123.18016
Cpped1	1.14E-09	3.48E-77	0.07619	8.9444101	0.1394981	76.458897
Etnk1	9.22E-10	0	0.075751	9.0351373	0.2916769	#NUM!
Stxbp5	6.38E-11	2.98E-74	0.075477	10.195113	0.1066087	73.526037
Hilpda	2.97E-227	0	0.075341	226.52788	0.1019947	#NUM!
Dhps	3.93E-73	0	0.075267	72.405737	0.1707332	#NUM!
Cd38	1.09E-98	6.9E-30	0.075137	97.961615	0.0208418	29.16139
Gramd1b	6.69E-120	2.63E-28	0.075132	119.17441	0.0191386	27.579924
Aldh1l1	3.52E-03	1.4E-151	0.075116	2.4530282	0.2431635	150.84498
Pnpla2	1.93E-60	5E-198	0.075088	59.715117	0.1050081	197.30093
Gm26632	5.86E-38	5.88E-24	0.075033	37.23226	0.0434401	23.230396
Mdh2	5.96E-35	6.8E-152	0.075023	34.224959	0.1150655	151.16546
Kcnq1	1.15E-59	2.2E-199	0.074889	58.941115	0.1032026	198.64966
Gpi1	2.60E-20	3.5E-119	0.074848	19.585663	0.1263619	118.45427
Pepd	6.66E-14	5.8E-229	0.074756	13.176265	0.1928516	228.23462
6030443J0	1.21E-12	4.8E-276	0.074689	11.917709	0.2190847	275.31851
Wdr44	3.73E-25	5.7E-138	0.074628	24.428866	0.1176277	137.24121
Trmt13	6.03E-22	7E-169	0.074604	21.219556	0.1427519	168.15214
Hck	5.65E-163	1.9E-28	0.074558	162.24802	0.0136997	27.722017
Dctn2	2.17E-30	4.1E-142	0.074468	29.662767	0.1141879	141.39024
Eif1	5.34E-20	4.1E-175	0.074449	19.272431	0.1443281	174.38349
Nid1	4.52E-204	0	0.074337	203.3448	0.1373619	#NUM!
Tnfrsf19	1.62E-167	0	0.074214	166.79039	0.1255932	#NUM!
Gm14403	1.32E-73	0	0.074202	72.878883	0.1341486	#NUM!
Mtrex	1.62E-25	5.2E-121	0.074116	24.789625	0.1132964	120.2812
Esr1	1.49E-03	8.7E-203	0.074109	2.8258163	0.2269092	202.0624
Acaca	6.44E-06	5.1E-300	0.074101	5.1908204	0.2873493	299.28966

Fndc9	0.00E+00	0	0.073953	#NUM!	0.0959537	#NUM!
Mapk8ip3	1.09E-15	6.2E-162	0.073784	14.961475	0.1521281	161.20614
Grpel2	2.25E-18	4.8E-151	0.073485	17.647632	0.1424845	150.32029
Nkd1	1.93E-149	0	0.073384	148.71448	0.171417	#NUM!
Gm26911	8.25E-182	0	0.073374	181.0834	0.1987125	#NUM!
Snrnp200	3.92E-31	1.8E-126	0.073353	30.406388	0.1059441	125.7527
Crebl2	1.48E-14	8.3E-101	0.073314	13.830308	0.1161317	100.08147
Tbc1d19	3.61E-101	3.1E-297	0.073237	100.44229	0.1066196	296.50892
Stard9	3.90E-75	3.74E-11	0.073232	74.408549	0.0166761	10.426859
Dctn6	1.01E-49	3.6E-246	0.073038	48.99669	0.1301947	245.4429
Spi1	1.14E-190	9.08E-19	0.072982	189.94169	0.0093242	18.042049
Chdh	6.62E-07	9.4E-279	0.072981	6.1788415	0.268547	278.02621
Upf1	5.46E-39	5E-149	0.072921	38.262645	0.1043535	148.30138
Fcho2	4.69E-05	2.53E-10	0.072862	4.3288958	-0.0470964	9.5977012
Fem1b	3.80E-43	9.8E-218	0.072861	42.419674	0.128955	217.00744
Fam83a	3.03E-261	0	0.072746	260.51856	0.0999196	#NUM!
Secisbp2	3.67E-17	8.9E-126	0.072634	16.43578	0.1285259	125.05145
Thoc1	8.06E-18	2.9E-135	0.072555	17.093574	0.1318585	134.53246
Huwe1	6.48E-03	5.82E-84	0.072502	2.1882569	0.1463629	83.234929
Mirt1	7.81E-170	3.18E-18	0.072432	169.10726	0.0091262	17.498231
Nupl2	2.24E-40	8.2E-196	0.072401	39.650543	0.1221434	195.0839
Il6ra	2.71E-03	5.7E-140	0.072357	2.5678273	0.2322639	139.24608
Ccdc66	1.57E-64	6.6E-199	0.07206	63.805068	0.0988495	198.17806
Phf14	6.97E-05	1	0.071815	4.1564769	0.0091617	0
Ice2	6.09E-22	9E-138	0.071618	21.215685	0.1243089	137.0446
Cyb5rl	1.35E-79	8.2E-274	0.071611	78.870484	0.1129014	273.08687
Bank1	6.27E-85	2E-20	0.071589	84.202733	0.021219	19.697918
Ces1h	1.50E-300	1.8E-203	0.07156	299.82414	0.0464556	202.74234
Gm36283	2.99E-186	0	0.071553	185.52407	0.1030083	#NUM!
Sh3pxd2b	1.16E-124	1.22E-20	0.07148	123.93718	0.0146229	19.913287
Dstn	1.94E-18	1.54E-96	0.071453	17.712637	0.1079784	95.812348
Gpx1	3.43E-09	1.1E-112	0.071435	8.4647873	0.1242619	111.96141
Fastkd1	1.82E-30	6.2E-156	0.071428	29.741056	0.1197097	155.20564
Zswim6	7.12E-05	2.54E-13	0.071406	4.1477662	-0.0444855	12.595784
Pef1	1.02E-35	1.1E-167	0.071373	34.989506	0.1134833	166.97497
Aasdh	1.16E-24	4E-111	0.071341	23.935973	0.1037407	110.39617
Tubgcp2	2.75E-47	2.8E-237	0.071333	46.561269	0.1260559	236.55695
Xylt2	3.52E-35	2.9E-218	0.071214	34.452873	0.1343142	217.54299
Dock11	2.73E-85	0.001081	0.071133	84.564534	0.0110513	2.9663159
Parp8	4.24E-119	3.28E-42	0.071016	118.37221	0.0208191	41.483526
Atp8b4	3.70E-59	6.89E-05	0.070931	58.43163	0.0142667	4.1619824
Mars	5.75E-58	3.6E-218	0.070895	57.240598	0.110474	217.44427
Foxn2	1.41E-11	7.78E-84	0.070848	10.850384	0.1080609	83.108877
Psmd5	1.16E-49	2.2E-198	0.070767	48.934748	0.1127683	197.65176
Mapk9	2.24E-13	1.36E-78	0.070621	12.649233	0.1057819	77.865935
Tm9sf4	2.81E-16	9.7E-151	0.070618	15.551837	0.1338703	150.0141
Ccdc88a	1.54E-67	1	0.070578	66.813399	0.007398	0

Ralbp1	3.36E-14	3.7E-179	0.07018	13.473646	0.1542477	178.42845
Ddx1	1.26E-28	1.6E-220	0.070138	27.898634	0.1428244	219.78472
Nudcd2	1.27E-76	5.5E-221	0.070127	75.896205	0.097824	220.26079
Cenpc1	4.10E-37	3.9E-238	0.070055	36.387509	0.1350808	237.41116
Tfec	1.61E-121	1.14E-69	0.069966	120.79221	0.0296404	68.944203
Lsg1	1.76E-19	2.3E-202	0.069846	18.754094	0.155271	201.64355
Ptpn6	9.15E-71	7.45E-30	0.069751	70.038576	0.0273704	29.127859
Elf4	6.69E-158	6.96E-59	0.069743	157.17443	0.0233361	58.157232
Snhg17	1.99E-69	7.6E-286	0.069739	68.700103	0.1216737	285.12166
Sdhb	4.09E-15	1.4E-107	0.069552	14.388689	0.1185693	106.84926
Slamf7	8.33E-181	4.29E-37	0.06954	180.07916	0.0126948	36.367049
Gusb	4.12E-43	1.7E-07	0.069534	42.384943	0.0176127	6.7699417
Fam49a	7.76E-96	1.57E-13	0.069278	95.109939	0.0127597	12.802926
Kcnk13	2.65E-148	1.01E-49	0.069236	147.57673	0.0184512	48.994705
Mfge8	1.38E-287	0	0.0692	286.86077	0.1088092	#NUM!
G6pdx	1.80E-140	4.7E-89	0.069142	139.74524	0.0380469	88.3277
A330023F2	9.79E-05	4E-24	0.069081	4.0090376	-0.0705429	23.398397
Gak	8.45E-09	5.7E-100	0.068879	8.0733416	0.1279875	99.24752
Abcg5	2.25E-03	0	0.068823	2.6476837	0.3019079	#NUM!
Snhg8	3.73E-49	4.2E-173	0.068798	48.42787	0.0988739	172.37441
Ifi203	7.97E-80	1.29E-13	0.068775	79.098499	0.0143369	12.889006
Vav1	1.90E-179	2.65E-43	0.068757	178.72088	0.0160652	42.576052
Prex1	6.39E-79	1	0.068745	78.19476	0.0044701	0
Aim2	6.07E-143	8.44E-56	0.068653	142.21696	0.0232358	55.073453
Abcg3	1.82E-125	3.21E-34	0.068634	124.74064	0.0168802	33.493665
Slc30a5	5.80E-17	8.1E-107	0.068624	16.236497	0.1130576	106.09191
2610020C0	2.39E-38	1.05E-21	0.068296	37.622364	0.0366567	20.9782
Igf1r	1.87E-84	8.8E-20	0.068202	83.728205	0.0189679	19.055554
Rev1	2.41E-07	6.56E-92	0.068144	6.6176273	0.1311069	91.182826
Pigk	1.98E-22	2.6E-114	0.067928	21.702703	0.1031863	113.59302
Myo1b	1.03E-02	3.6E-215	0.067873	1.9871096	0.2597225	214.44017
Arrb1	2.61E-155	2.4E-109	0.067585	154.58298	0.0341096	108.61549
Apba3	7.51E-47	5.2E-204	0.067518	46.124599	0.1103783	203.28049
Pirb	1.58E-166	1.8E-33	0.067438	165.80006	0.014839	32.745382
Gpatch2	9.88E-07	3.01E-98	0.067425	6.0050663	0.1481152	97.520936
Gm12185	7.09E-104	2.39E-07	0.067347	103.14963	0.0110989	6.6217026
Mir142hg	2.04E-179	9.47E-37	0.067236	178.68963	0.0140799	36.023553
Cyth4	9.07E-165	9.76E-29	0.067171	164.04261	0.0132806	28.010354
Lpin1	1.86E-23	3.7E-114	0.06712	22.731161	0.2660563	113.43248
Sec24b	3.88E-08	5.58E-97	0.066942	7.4106808	0.1245017	96.253255
Hsph1	1.14E-30	1.8E-282	0.066941	29.942885	0.1584743	281.74922
Golga5	2.82E-12	1.9E-121	0.066819	11.550125	0.1287055	120.71389
9530046B1	4.93E-50	8.1E-257	0.066765	49.306947	0.1239464	256.08982
Fam111a	1.79E-75	2.03E-06	0.066518	74.746452	0.0145632	5.6918201
Cln3	1.56E-35	0.017296	0.066507	34.807165	0.0139169	1.7620509
Lair1	2.78E-161	6.82E-46	0.066494	160.5552	0.016853	45.166162
Nol11	1.40E-35	2.5E-161	0.066415	34.853436	0.1050141	160.60922

Csf2ra	1.69E-161	8.68E-63	0.066368	160.77192	0.0226264	62.061368
Mpeg1	7.78E-166	2.45E-32	0.066364	165.10888	0.0147453	31.611451
Mrps7	3.16E-48	2.7E-283	0.06633	47.500488	0.1312142	282.56587
Trip11	4.26E-06	4.42E-49	0.066324	5.3703336	0.1043909	48.354865
Abca3	2.88E-18	5.6E-95	0.066319	17.540007	0.1006578	94.251838
Ibtk	1.50E-08	4.9E-144	0.066237	7.8251995	0.1551101	143.31092
Otulinl	3.19E-168	6.67E-37	0.06622	167.49673	0.0140733	36.175927
Slc44a3	4.66E-36	6.7E-179	0.066211	35.331455	0.1119966	178.1738
Gls	7.76E-68	5.6E-16	0.066179	67.11026	0.018183	15.251945
Cdk5rap1	7.05E-35	1.6E-159	0.065767	34.151836	0.1063869	158.80074
Meiob	5.93E-08	1	0.06552	7.2267652	-0.0086615	0
Elp1	1.16E-23	2.1E-173	0.065487	22.936593	0.1284989	172.67865
Gpr141	6.38E-141	4.4E-31	0.065292	140.19507	0.0127283	30.356995
Abca6	2.99E-05	0	0.065264	4.5242851	0.3356136	#NUM!
Usp20	4.64E-22	9.9E-130	0.065175	21.333778	0.1116897	129.00573
Ddx56	2.38E-51	6.9E-177	0.065139	50.624243	0.0954539	176.15874
Brd7	9.64E-14	8.3E-123	0.064967	13.015732	0.1197385	122.0831
Zfp160	2.89E-15	2.5E-144	0.064916	14.539569	0.1317812	143.5945
Uqcrc1	2.15E-22	5.8E-121	0.064772	21.667347	0.1045114	120.23496
Ddx24	4.03E-13	6E-141	0.064771	12.39437	0.1311991	140.22186
Prkch	2.31E-75	6.5E-15	0.064739	74.635981	0.0149157	14.187285
Fmo2	2.52E-110	0	0.064354	109.59819	0.1051093	#NUM!
Tug1	7.78E-19	1.1E-177	0.064344	18.109164	0.1344521	176.96965
Sptan1	2.71E-05	5.72E-88	0.064326	4.5676803	0.1327115	87.242234
Mphosph1	1.53E-49	2.2E-193	0.064309	48.816401	0.1013709	192.64942
Nav1	3.63E-41	1	0.064292	40.440433	0.0094147	0
Prdx1	2.37E-02	9.37E-40	0.064157	1.6246404	-0.0867016	39.028154
Arhgap30	3.18E-151	6.12E-30	0.064044	150.49696	0.0148067	29.212965
Thoc7	1.42E-23	9.5E-120	0.064042	22.848854	0.1021784	119.02385
Cars	1.33E-15	3.2E-147	0.063839	14.876698	0.1293825	146.49173
Cd300lf	2.30E-163	5.71E-36	0.063792	162.638	0.013012	35.24367
Gm42375	4.77E-241	1.2E-138	0.06376	240.32147	0.0349118	137.93091
Cyp2c55	6.30E-73	5.3E-290	0.063654	72.200528	0.1213517	289.27671
Mipep	1.31E-21	4.1E-162	0.063549	20.883363	0.1242871	161.38431
Gfm1	1.29E-21	1.3E-170	0.063539	20.889943	0.128411	169.87127
Taf2	8.03E-19	3.6E-115	0.063401	18.095468	0.1023018	114.44155
Ggta1	7.13E-142	8.85E-25	0.063347	141.14717	0.0109167	24.052856
Phrf1	1.95E-10	5.8E-111	0.063335	9.7108995	0.1259057	110.23674
Sec11a	2.17E-07	5.24E-86	0.063243	6.6637777	0.1191106	85.280665
Ankrd13c	8.63E-06	5.6E-100	0.063185	5.0638997	0.154345	99.254159
Atp5d	4.84E-30	3.2E-200	0.062961	29.315582	0.1211757	199.48917
Cyp4a31	2.07E-53	0	0.062827	52.683844	0.1379727	#NUM!
Gpr89	4.23E-22	2.6E-135	0.062799	21.373727	0.1099129	134.59306
Acot3	1.01E-204	0	0.06273	203.99434	0.1359327	#NUM!
Map2k2	1.26E-07	4.71E-94	0.062722	6.8981731	0.1241953	93.327439
Aars	3.42E-10	6.3E-75	0.062632	9.4662221	0.1023091	74.200607
Ttc14	7.36E-05	1.3E-104	0.062621	4.1333373	0.1351461	103.89438

Rell1	7.99E-06	3.13E-80	0.06259	5.0973763	0.1196837	79.505015
Vipas39	2.17E-21	4.2E-167	0.062504	20.664126	0.1276118	166.37943
Greb1l	1.07E-35	0	0.062471	34.971121	0.2887563	#NUM!
Scfd1	7.34E-07	0	0.062465	6.1345149	0.2479563	#NUM!
Clec16a	8.08E-04	8.82E-85	0.062462	3.0923901	0.1364214	84.054433
Usp16	1.70E-11	5.4E-115	0.062452	10.769171	0.1235803	114.26483
Nbas	6.99E-06	4.6E-294	0.062443	5.1552177	0.2453425	293.33436
Cd180	1.40E-108	2.31E-33	0.062431	107.85356	0.0175042	32.635801
Gpatch3	1.40E-37	9.8E-161	0.062421	36.854964	0.1001154	160.00966
Twsg1	2.02E-41	2.3E-224	0.062357	40.694083	0.1168619	223.64755
Fbp2	2.68E-151	0	0.062325	150.57144	0.1205517	#NUM!
Adcy7	1.22E-99	3.79E-08	0.062308	98.913469	0.0110575	7.421624
Atp13a1	1.40E-23	6.8E-136	0.062233	22.853715	0.1075592	135.16904
Ptk2	6.92E-08	1	0.06205	7.159593	0.015708	0
Zfp143	1.90E-21	3.9E-103	0.062013	20.721023	0.0927578	102.41349
Hpse2	7.47E-58	0	0.062011	57.126659	0.3894909	#NUM!
Orc4	2.52E-15	2.1E-94	0.061901	14.598435	0.1011579	93.678172
Ccdc122	6.26E-21	1.3E-261	0.0619	20.203149	0.161505	260.88421
Akt3	1.68E-70	4.03E-07	0.061826	69.774281	0.0114647	6.3952244
Grhpr	3.22E-05	2.24E-63	0.061781	4.4921174	0.1038321	62.65001
Alg8	2.18E-69	1.4E-214	0.061761	68.661389	0.0901092	213.83904
Vldlr	2.97E-137	0	0.061736	136.52693	0.126694	#NUM!
H2afy	7.94E-08	3.22E-83	0.061719	7.1002247	0.1072266	82.492011
Pam	1.48E-35	2.94E-11	0.061717	34.829079	0.0241916	10.532275
Xkr9	8.11E-05	3.1E-144	0.061678	4.090917	0.1622204	143.51357
Dsg2	2.16E-07	9.7E-150	0.061637	6.6665001	0.1640863	149.01473
5033421B0	4.61E-38	1	0.061592	37.336408	0.0038385	0
Cttnbp2nl	5.45E-48	1.45E-07	0.061564	47.263685	0.0161774	6.837581
Ddx52	5.08E-29	1.5E-119	0.06152	28.293937	0.0918035	118.82933
Tmem147	1.06E-48	1.1E-261	0.061417	47.974042	0.1176422	260.96835
Ecd	2.34E-14	1.94E-98	0.061174	13.631136	0.1079873	97.711449
Tubb4b	3.03E-50	8.4E-163	0.061051	49.519128	0.0841185	162.07452
Gm14164	7.25E-166	7.2E-119	0.061018	165.13994	0.0390699	118.14299
Cars2	1.81E-22	6.6E-187	0.060998	21.742996	0.1299868	186.17983
Mms22l	9.75E-54	9.9E-185	0.060961	53.010827	0.0913201	184.00366
Chchd6	7.50E-41	2.3E-150	0.060897	40.125176	0.0889482	149.63891
Slc38a1	1.98E-146	7.41E-53	0.060799	145.70232	0.019496	52.130091
Sdad1	1.35E-29	1.2E-177	0.060696	28.868682	0.1127718	176.91562
Ehbp1l1	1.51E-41	1	0.060628	40.820074	0.0076735	0
Rexo2	4.96E-29	1.8E-146	0.060626	28.304302	0.1020174	145.73634
Cybb	1.90E-134	7.32E-26	0.060442	133.72159	0.0111827	25.135512
Stpg2	2.92E-68	5.6E-204	0.060426	67.534219	0.0892435	203.252
Cd59a	9.75E-18	5.4E-132	0.06035	17.011016	0.1115257	131.27002
Abcf1	2.29E-11	9.64E-77	0.060347	10.639278	0.096044	76.015825
Chid1	1.19E-37	7.4E-160	0.060332	36.925633	0.096213	159.1281
Il7r	3.29E-131	1.89E-33	0.060253	130.48339	0.0141549	32.723176
Nvl	8.70E-10	1.92E-82	0.060229	9.0606377	0.106464	81.716962

Slc37a2	2.29E-72	1.48E-16	0.060202	71.639529	0.0173878	15.828755
Plekho2	1.12E-122	8.28E-12	0.060193	121.95052	0.0095553	11.082195
Itga1	2.27E-04	2.2E-177	0.060075	3.6431169	0.1947622	176.66036
Bmp6	4.60E-92	0	0.060028	91.337297	0.1300371	#NUM!
Cct8	1.39E-19	1.8E-136	0.059978	18.856956	0.1095531	135.73843
Ndufs2	3.67E-26	8.8E-136	0.059964	25.435616	0.0989434	135.05324
Rfx7	1.52E-02	3.24E-82	0.059926	1.8190365	0.1510709	81.490022
Ccdc120	3.67E-164	0	0.059924	163.43555	0.0905109	#NUM!
Pex26	7.82E-22	2.4E-135	0.059813	21.107038	0.1067742	134.62221
Rasa4	1.16E-146	1.79E-22	0.059803	145.93447	0.0093593	21.748352
Cacna1b	2.95E-192	0	0.059802	191.53089	0.1022568	#NUM!
Khdc4	4.38E-04	1E-79	0.059618	3.3583667	0.1263361	78.998219
Gm12092	3.43E-225	2.3E-115	0.059566	224.46429	0.0293148	114.63635
Gm31763	3.00E-36	2.27E-14	0.059483	35.5231	0.0262203	13.643772
B430306N(	8.18E-162	9.83E-27	0.059449	161.08722	0.0096866	26.007486
Fnbp1l	1.01E-08	1.61E-88	0.059415	7.9969332	0.1061808	87.793148
Il10rb	9.90E-36	1	0.059399	35.004358	0.0027368	0
Snx14	2.13E-11	1.3E-108	0.059342	10.672191	0.1170156	107.89403
Aco2	1.55E-03	1.4E-180	0.059269	2.8085727	0.2078089	179.86559
Ube4b	2.37E-03	5.79E-45	0.059256	2.6254894	0.1041486	44.237266
Itgam	9.35E-150	3.79E-18	0.059215	149.02911	0.0080827	17.420839
Uqcr10	1.26E-33	4E-231	0.059126	32.897947	0.1171667	230.40263
Ddx50	9.11E-06	6.3E-103	0.059061	5.040258	0.127697	102.19826
Slc16a5	5.33E-96	0	0.059053	95.272968	0.1231493	#NUM!
Ssr3	4.23E-24	1.8E-157	0.059045	23.373815	0.1103817	156.73654
Pwp1	1.30E-36	7.7E-176	0.059043	35.885529	0.1022264	175.11573
Eif2ak4	1.84E-12	7.2E-106	0.058946	11.735508	0.1050684	105.1442
Asns	4.08E-188	0	0.058845	187.38987	0.0824066	#NUM!
Il10ra	5.86E-118	1.68E-17	0.058602	117.23246	0.0098321	16.773893
St7	4.97E-04	4E-112	0.058593	3.3032555	0.1523743	111.39983
Napa	1.40E-13	4.48E-94	0.05855	12.852374	0.0958225	93.348926
Cramp1l	3.60E-08	1.46E-62	0.058467	7.4442649	0.0914974	61.835338
Dhx8	3.54E-14	2.2E-99	0.058437	13.451005	0.1022686	98.659793
Capn10	1.57E-29	1.7E-217	0.058403	28.803381	0.1243688	216.77739
Nop14	7.98E-19	8.6E-104	0.058315	18.098169	0.0926746	103.06499
BC025920	6.40E-89	3.77E-60	0.058272	88.193547	0.0271585	59.423778
Nup205	4.11E-33	1.8E-135	0.058251	32.38588	0.0869587	134.75459
Trmt2a	4.55E-53	1.7E-195	0.058244	52.342167	0.0878759	194.76008
Gm41541	7.95E-100	8.1E-249	0.058065	99.099379	0.0846924	248.09133
Gm49980	4.43E-05	3.18E-98	0.058046	4.353963	0.1564895	97.497295
Cox19	2.48E-15	2.3E-137	0.058018	14.605166	0.1152758	136.64306
Ly86	2.13E-95	2.22E-23	0.057989	94.67132	0.0140426	22.654502
Axl	2.51E-136	2.21E-27	0.057903	135.60072	0.012673	26.656365
Dnajc11	1.36E-04	2.51E-98	0.057892	3.8675435	0.1408483	97.601012
Zc3h7b	2.83E-17	3.11E-94	0.057818	16.548763	0.0915995	93.507267
Hspa4l	1.31E-29	2.4E-242	0.057813	28.88121	0.1363872	241.61976
Lyz2	1.65E-151	2.12E-28	0.057773	150.78344	0.0133039	27.674565

Htra1	3.49E-176	5.06E-68	0.057687	175.45681	0.0206555	67.295575
Npat	4.82E-22	6.5E-135	0.057682	21.316801	0.1000681	134.18628
Zfp426	2.24E-17	3.3E-113	0.057587	16.6503	0.1030894	112.47963
Npepl1	1.74E-18	1.8E-135	0.057385	17.759189	0.1045027	134.74947
Clec4d	6.33E-166	3.77E-41	0.057384	165.19881	0.0127287	40.423097
Nbl1	1.08E-222	0	0.057271	221.9648	0.0803164	#NUM!
Naa40	1.31E-21	1.7E-99	0.057242	20.883887	0.0851779	98.772226
Ttyh2	1.24E-03	3.79E-76	0.057214	2.9062194	0.1108208	75.421789
Tia1	5.37E-15	9.8E-130	0.057084	14.270261	0.1119325	129.00846
Slc33a1	8.85E-08	7.44E-72	0.057002	7.0531593	0.0995441	71.128707
Cotl1	2.97E-93	9E-10	0.056999	92.527842	0.0102337	9.0455521
Adcy1	1.79E-92	0	0.056977	91.746095	0.1121443	#NUM!
Clip1	7.49E-05	0.014646	0.056959	4.1253414	-0.0352018	1.8342855
Trim24	5.88E-10	0	0.056911	9.2307308	0.2384607	#NUM!
Fan1	2.29E-26	2E-143	0.056869	25.640772	0.0995857	142.70764
Anxa3	1.00E-117	5.5E-18	0.056791	116.99796	0.0111593	17.259278
Stard5	8.75E-05	5.3E-160	0.056741	4.0579694	0.173786	159.27712
Epb41l4aos	6.48E-73	1.6E-224	0.056697	72.18862	0.0872081	223.80131
Mpp1	1.67E-27	2E-116	0.056566	26.777379	0.0791469	115.6944
Rxylt1	4.86E-26	5.7E-137	0.056557	25.313415	0.0989349	136.24794
Ccz1	1.06E-09	1.87E-88	0.056534	8.9732435	0.0999533	87.728069
Brix1	9.01E-22	1.3E-100	0.056514	21.045385	0.0860669	99.875112
Ttf2	4.49E-41	5.8E-174	0.056493	40.347853	0.0912865	173.23305
Eif3g	4.32E-57	7E-204	0.056481	56.36412	0.0884152	203.15644
Tmem248	3.07E-07	4.5E-114	0.056468	6.5129793	0.1261992	113.34379
A330040F1	3.47E-05	1	0.056295	4.4598937	-0.0115408	0
Frmd4a	6.66E-61	1	0.05629	60.176767	0.0058736	0
Snx8	7.14E-14	0.136285	0.056272	13.146285	0.0164754	0.8655521
Rab4a	3.42E-13	7.5E-102	0.05623	12.466567	0.106169	101.12266
Ccdc47	1.85E-19	2E-116	0.056036	18.732602	0.098363	115.69919
Gnpat	5.11E-15	2.2E-87	0.055977	14.29148	0.0894893	86.656789
Sapcd2	2.20E-245	0	0.055903	244.65706	0.0835476	#NUM!
Laptm5	6.94E-130	7.05E-23	0.05564	129.15867	0.0115986	22.151898
Kdm6a	8.23E-03	1.48E-50	0.055635	2.0846584	0.0990055	49.830187
Psmd13	1.49E-16	9.7E-178	0.055597	15.827762	0.123263	177.01477
Uqcrc2	2.11E-08	2.9E-117	0.055588	7.6749367	0.126618	116.53043
Eif2d	2.47E-22	1.2E-142	0.055531	21.608094	0.1030593	141.90499
Gucy2c	8.12E-182	4.4E-99	0.055502	181.09043	0.0289655	98.352511
Eipr1	7.34E-13	7.4E-180	0.055354	12.134454	0.1379884	179.12823
Epsti1	1.23E-75	3.28E-18	0.055347	74.911839	0.0130938	17.483477
Atp5a1	8.51E-05	1.35E-75	0.055343	4.0700305	0.1162588	74.868857
2610507B1	4.41E-05	2.49E-56	0.055334	4.3559753	0.0975442	55.603495
Zc2hc1a	9.00E-145	0	0.055191	144.04591	0.0889123	#NUM!
Psmc3	1.95E-17	5.7E-267	0.055134	16.71059	0.1525658	266.24099
Gab3	2.29E-98	2.64E-28	0.055052	97.640656	0.0154933	27.578448
Cep295	5.78E-14	2.4E-111	0.055031	13.238327	0.1002155	110.62341
Mybbp1a	2.02E-22	2.1E-139	0.054975	21.695306	0.0996145	138.68307

Efl1	4.59E-06	9.29E-73	0.054975	5.3383446	0.1072322	72.032161
Ss18	2.25E-10	5.11E-76	0.054889	9.6479319	0.0941407	75.291953
Gm15943	6.50E-90	2.35E-09	0.054854	89.187357	0.0086832	8.6285578
Gskip	7.04E-15	2.72E-87	0.054832	14.152493	0.0851462	86.564917
Irf8	3.33E-84	2.88E-10	0.05482	83.478135	0.0112666	9.5411088
Szt2	4.32E-25	4.4E-146	0.054751	24.364528	0.0989713	145.36083
Ankib1	1.01E-03	5.77E-70	0.054708	2.9936994	0.1096304	69.23894
Eif2ak3	3.20E-07	7.78E-66	0.054629	6.4941949	0.0913326	65.109132
Fbxw5	5.07E-32	3.2E-181	0.054436	31.294951	0.0981644	180.49737
Cyba	3.24E-124	2.24E-20	0.054399	123.4895	0.0108983	19.65
Rapgef5	8.57E-37	0.028071	0.054327	36.067154	0.0108536	1.5517468
Trnt1	1.01E-33	1E-165	0.054314	32.995921	0.0952976	164.98593
Wnk3	1.04E-61	9.07E-32	0.054283	60.984027	0.0275063	31.04227
Slc38a9	1.77E-04	3.66E-71	0.05425	3.7527831	0.1084942	70.436871
Zfp672	2.02E-15	4.5E-177	0.054214	14.694493	0.1270999	176.34378
Itga4	1.73E-90	5.99E-22	0.054191	89.761481	0.0145687	21.222223
Pomgnt1	2.17E-37	3E-147	0.054117	36.662985	0.084083	146.52136
Pus10	4.04E-06	1.64E-84	0.053999	5.3938977	0.1173603	83.785045
Elp3	3.18E-13	1.26E-78	0.053996	12.497907	0.085092	77.899578
Dnase1l1	3.63E-37	2.89E-17	0.053774	36.440429	0.0221466	16.539735
Rars	2.92E-15	4.4E-130	0.053758	14.53535	0.1082372	129.35746
Wdr45	5.15E-25	3.8E-142	0.053748	24.288391	0.0937922	141.41484
Usp19	1.27E-12	8.3E-133	0.053733	11.896966	0.1183874	132.08005
Zfp692	1.25E-28	6E-229	0.053648	27.90235	0.1217257	228.22141
Slc9a8	3.44E-05	2.2E-53	0.053526	4.4634415	0.0893099	52.657522
Cltb	7.40E-13	2.91E-78	0.053428	12.131008	0.0892728	77.53664
Ubac2	8.77E-04	3.59E-84	0.053407	3.0567682	0.1392661	83.445158
Tmem267	7.01E-17	1	0.053343	16.154553	0.0050623	0
Atp5g1	1.03E-33	4.8E-130	0.053318	32.985423	0.078872	129.31866
Ehd4	1.67E-70	1.18E-45	0.053238	69.777535	0.0253924	44.928884
Csf2rb2	3.40E-160	1.68E-31	0.053221	159.46858	0.0093004	30.775074
Lonp1	1.23E-16	3.2E-157	0.053067	15.909374	0.1124128	156.49678
Oasl2	4.95E-83	4.17E-07	0.053002	82.305394	0.0084774	6.3800402
Slco3a1	1.56E-39	1	0.052968	38.805801	0.0039648	0
Tbrg1	7.23E-32	1.5E-252	0.052884	31.140987	0.1176808	251.83248
Ufl1	1.29E-07	8.6E-103	0.052821	6.8883377	0.1184474	102.06724
mt-Atp6	4.94E-35	1.8E-165	0.052722	34.306266	0.0827476	164.74811
Psmd6	1.30E-29	2.3E-122	0.052718	28.886396	0.0792984	121.64432
Srsf6	1.23E-04	1.2E-171	0.052674	3.9091602	0.1578673	170.90877
Nom1	8.71E-12	2.3E-136	0.052624	11.059771	0.1214931	135.64502
Dcbld1	3.91E-25	1.5E-208	0.052462	24.407807	0.1178658	207.81108
Rras2	9.16E-04	3.34E-76	0.052392	3.038239	0.1164604	75.476232
Sema6d	1.54E-26	1	0.052339	25.811736	0.0098679	0
Coro2a	2.00E-114	3.93E-21	0.052208	113.69842	0.0117434	20.405057
Pdhb	1.95E-13	2.6E-158	0.052053	12.711055	0.1234284	157.57683
Ddx18	9.94E-24	4E-140	0.051976	23.002502	0.0963015	139.39875
Ndufv1	2.15E-31	1.5E-179	0.051901	30.667998	0.0973566	178.8173

Dtymk	1.45E-42	4.1E-151	0.05185	41.837829	0.0776984	150.39163
Snapc3	3.81E-04	2.46E-69	0.051837	3.4186747	0.1014364	68.609152
Gm15441	2.81E-57	0	0.051787	56.551393	0.1345743	#NUM!
Pgap2	4.46E-11	4.6E-85	0.051738	10.350507	0.0913139	84.337441
Dld	3.88E-20	1.6E-110	0.051713	19.410676	0.0853326	109.80826
Arhgap1	1.00E-20	1.8E-112	0.051667	19.999963	0.0849175	111.75525
Psmc4	1.42E-20	9.7E-137	0.051663	19.847706	0.096152	136.01151
Dvl1	1.33E-05	1.1E-184	0.051617	4.8773177	0.1628658	183.94113
Ipo4	3.83E-49	1.2E-207	0.051592	48.417177	0.0867762	206.93128
Xpo4	1.30E-04	1.03E-53	0.051574	3.8872934	0.0912767	52.988143
Tnfaip8	1.54E-81	0.000239	0.051539	80.813133	0.0069557	3.6209303
Neurl3	1.16E-114	2.33E-41	0.051496	113.93579	0.0167218	40.632815
Fgr	1.77E-23	1	0.051456	22.751765	-0.0006889	0
Rmnd5b	1.80E-28	2E-120	0.05136	27.745365	0.0780413	119.7003
Ube2f	1.43E-05	5.46E-57	0.051275	4.8446357	0.0867702	56.262451
Lias	5.69E-15	4.2E-97	0.051252	14.244893	0.0889212	96.376337
Socs6	1.06E-10	2.7E-108	0.051043	9.9730134	0.0979808	107.57539
Srp19	1.29E-20	3.2E-110	0.051019	19.888148	0.0834926	109.49811
Rnf126	4.11E-30	4E-155	0.050994	29.385695	0.0903531	154.40224
P2rx7	3.95E-87	2.57E-09	0.050977	86.403268	0.0097291	8.5900335
Aprt	1.62E-34	1.1E-136	0.050944	33.789539	0.0762968	135.94099
Hgs	1.18E-12	1.05E-83	0.05085	11.928946	0.0850178	82.977674
Csde1	1.57E-02	5.75E-89	0.05084	1.8053554	0.123884	88.240557
Tnfrsf11a	6.44E-119	1.11E-33	0.050825	118.19096	0.0129196	32.954755
Ncln	2.09E-28	1.1E-117	0.0508	27.67914	0.0788251	116.97531
Timm13	3.32E-34	6.5E-135	0.050754	33.479075	0.0755035	134.18472
Cops5	1.71E-12	7.23E-88	0.050727	11.767719	0.0896226	87.140665
Gm11973	5.39E-19	0.108386	0.050531	18.268085	0.015402	0.9650278
B230217O1	3.00E-13	2.48E-76	0.05044	12.52247	0.0811142	75.606079
Nat8f6	1.44E-73	1	0.050334	72.840897	0.0036671	0
Slc7a8	3.41E-124	1.52E-39	0.050255	123.46705	0.0139086	38.817105
Itih5	1.20E-08	0	0.050237	7.92005	0.3516798	#NUM!
Nars	6.03E-07	1.97E-66	0.050102	6.2196818	0.0826739	65.705207
Plekha2	1.83E-101	0.131856	0.050046	100.7375	0.0041729	0.8798999
Grhl1	1.22E-95	1E-277	0.050016	94.911887	0.0846478	276.98136
Plek	1.51E-118	7.09E-22	0.050002	117.82054	0.0089009	21.149303
Eif3b	1.64E-13	9.6E-151	0.049991	12.785611	0.1161579	150.01605
Ddx55	3.91E-14	5.2E-114	0.049712	13.407722	0.0952094	113.28098
Mt2	2.41E-58	2.1E-274	0.049584	57.618813	0.1051697	273.66784
Gng2	1.09E-101	4.31E-36	0.049481	100.96179	0.0164604	35.365556
Slc35g1	2.62E-14	2.3E-106	0.049473	13.581942	0.09254	105.6326
Rpap2	3.80E-08	4.57E-69	0.049472	7.4201476	0.0858116	68.339709
Nup88	1.75E-12	2.37E-78	0.049459	11.756346	0.0828617	77.625033
Ncbp1	2.65E-17	5E-100	0.049399	16.577399	0.0875208	99.303942
Anapc15	2.61E-25	1.2E-07	0.049364	24.583852	0.0147394	6.920461
Nrd1	2.25E-02	2.01E-71	0.049228	1.6468911	0.10796	70.697314
Zfyve16	4.99E-17	8.8E-124	0.04898	16.301525	0.0933553	123.05477

Zfp438	7.32E-07	1.11E-69	0.048974	6.1353417	0.0909866	68.953089
Slfn4	1.34E-120	3.76E-24	0.048955	119.87148	0.0088738	23.424914
Clec4f	2.57E-37	2.51E-07	0.048913	36.589868	0.0141967	6.6000866
Nedd1	4.62E-04	9.23E-51	0.048913	3.3355446	0.0848193	50.0348
Gm49961	2.99E-12	2.9E-209	0.048833	11.523653	0.1473165	208.54248
Bcat2	1.58E-07	8.08E-80	0.04881	6.8000724	0.0951975	79.092852
Gm4258	5.52E-88	1.4E-220	0.04869	87.258021	0.0713478	219.84107
Dus1l	6.11E-20	0	0.048652	19.214125	0.1918851	#NUM!
Ptprt	1.23E-84	3.62E-35	0.048614	83.909025	0.0217832	34.440999
AU020206	1.89E-63	3.53E-25	0.048601	62.722629	0.0183677	24.452259
Pla2g4a	6.81E-103	1.41E-34	0.048566	102.16699	0.0137047	33.851955
Hmgcr	3.45E-03	0	0.048342	2.4620693	0.4284241	#NUM!
Col6a3	1.84E-166	0	0.048237	165.73437	0.1178451	#NUM!
mt-Nd1	9.64E-36	6.9E-131	0.048237	35.016128	0.0684848	130.16343
Dmtf1	4.58E-04	1.95E-50	0.048221	3.3389966	0.0841576	49.709858
Gcn1	1.28E-04	9.11E-88	0.048183	3.8911942	0.1123009	87.040708
Mlh1	1.57E-14	9.9E-108	0.047915	13.805339	0.090627	107.00466
Itgal	5.18E-89	2.13E-26	0.047707	88.285804	0.0143063	25.670916
Yipf5	1.57E-13	2.7E-116	0.047692	12.803423	0.0985873	115.57366
Sall1	1.24E-07	7.1E-130	0.047684	6.9052337	0.1148307	129.14956
Gm20658	1.38E-131	3.24E-53	0.047677	130.85913	0.0153325	52.489127
Cox5b	2.02E-30	1.1E-156	0.047616	29.695285	0.0797991	155.96315
Tecpr1	4.19E-02	1	0.047616	1.378142	-0.0017496	0
Nfam1	6.07E-122	3.74E-24	0.047608	121.21708	0.0107306	23.427347
Fbxo36	1.25E-09	1.1E-117	0.047552	8.9047029	0.1098204	116.97644
Nsun2	2.17E-06	1.2E-68	0.047527	5.6639054	0.0889773	67.919279
Mrps10	6.39E-14	1.16E-88	0.047422	13.194185	0.0824249	87.937299
Clasrp	9.99E-07	7.4E-78	0.04731	6.0003391	0.0939561	77.1308
Farsb	1.43E-06	4.8E-94	0.047259	5.8435597	0.1036185	93.318735
Alpk1	2.24E-50	5.26E-08	0.047191	49.649271	0.0116404	7.2787189
Polg	1.72E-10	2.23E-83	0.047112	9.7634848	0.0803616	82.651888
Farsa	2.43E-11	1.67E-92	0.047098	10.614786	0.089413	91.776644
Zfp930	2.56E-37	2.1E-159	0.04707	36.59188	0.0775819	158.67406
Usp38	1.17E-08	3.63E-96	0.047027	7.9304552	0.099256	95.440196
Sdc3	1.23E-78	4.47E-06	0.047005	77.910809	0.0080806	5.3499608
Rexo1	6.18E-06	1.1E-128	0.046964	5.2091186	0.129434	127.94741
Csnk1g2	2.23E-06	1.01E-83	0.046928	5.6510311	0.0966764	82.997003
Dvl3	1.68E-13	1.3E-100	0.046923	12.775954	0.0870109	99.88062
Xlr3a	1.69E-101	8.25E-27	0.046894	100.77146	0.0168808	26.083422
Cd48	7.72E-124	1.51E-28	0.04683	123.11224	0.0107967	27.821663
Rcsd1	1.68E-69	6.23E-23	0.046801	68.775029	0.0151521	22.205745
1110032A0	1.26E-19	1.1E-110	0.046783	18.900981	0.0819178	109.97541
BC052040	1.39E-03	1.08E-55	0.046718	2.8581882	0.0926327	54.965777
Trappc10	3.42E-04	1.1E-95	0.046716	3.4659252	0.1139606	94.960401
Ssr4	7.04E-34	0	0.04665	33.152617	0.1423417	#NUM!
Zfp131	6.69E-06	2.45E-84	0.046648	5.1746481	0.0980655	83.611035
Rnf167	2.00E-16	1.8E-120	0.046324	15.698701	0.0837087	119.73461

Cxcl1	1.48E-37	0.000893	0.046214	36.828726	0.0152022	3.0493683
Tlr7	2.67E-123	2.03E-31	0.04606	122.57349	0.0110258	30.692837
Eme2	7.07E-79	5.1E-239	0.046024	78.150292	0.0764186	238.29614
Parp2	1.56E-35	2.3E-163	0.046007	34.807099	0.0784342	162.63567
Fblim1	1.03E-124	2.59E-20	0.045957	123.98679	0.0091023	19.587297
Car2	5.01E-113	2.1E-238	0.045937	112.30012	0.0676691	237.67339
Ciao3	1.70E-17	2.1E-113	0.045891	16.769475	0.0809303	112.68469
Mtif3	6.46E-14	4.78E-81	0.04576	13.189853	0.0762209	80.320161
Trpm2	2.29E-91	9.61E-25	0.045667	90.6395	0.0124052	24.017228
Sec24c	3.73E-04	1.46E-87	0.045566	3.4284584	0.1063811	86.837098
Commd3	4.05E-29	3.1E-127	0.045554	28.392338	0.073464	126.51367
Slfn5	1.68E-64	0.002395	0.045432	63.775374	0.0059016	2.6206888
Stx18	6.56E-03	7.8E-112	0.045398	2.1832605	0.1346843	111.10815
Gm34829	1.97E-154	0	0.04538	153.70642	0.078415	#NUM!
Capns1	5.71E-18	2.92E-88	0.045312	17.243153	0.0700956	87.534596
Gm48236	1.20E-114	1.1E-287	0.04531	113.92033	0.0816438	286.9498
Lpxn	4.44E-30	2.15E-12	0.045307	29.352527	0.0188643	11.666986
Chordc1	8.47E-06	7.22E-88	0.045289	5.0722473	0.0975004	87.141616
Atox1	6.46E-07	4.53E-63	0.045237	6.1900525	0.0772234	62.344201
Dhx30	4.71E-08	1.63E-88	0.045215	7.3265336	0.0899381	87.787799
Ptpn23	5.13E-17	3.3E-95	0.045212	16.289896	0.0720815	94.482004
Smc6	9.83E-03	9.7E-64	0.045199	2.0072518	0.0903186	63.013234
Ddx49	1.44E-13	1.03E-94	0.045181	12.840369	0.0772459	93.989076
Slc38a10	1.36E-08	2.44E-82	0.045151	7.8662016	0.0819991	81.612563
Mrpl15	3.85E-11	1.6E-125	0.045137	10.414878	0.102367	124.79375
Spata17	3.11E-25	8.6E-269	0.045125	24.506988	0.1257775	268.06633
Camkk2	1.30E-12	2.3E-120	0.045074	11.886541	0.0913837	119.63721
Ptgs1	1.31E-78	1.23E-20	0.045061	77.882657	0.01301	19.910256
Gm16559	3.26E-72	9.3E-213	0.044929	71.486153	0.07055	212.03001
Uggt1	2.75E-03	1.5E-131	0.044898	2.561287	0.1411534	130.82359
Mthfr	1.09E-02	2.53E-71	0.044858	1.962481	0.1032816	70.597489
Pak1ip1	2.53E-12	4.03E-90	0.044783	11.59673	0.0813055	89.394273
Als2cl	5.45E-04	5.74E-71	0.044704	3.2636991	0.1002157	70.241309
Cln6	7.37E-107	1.7E-284	0.044701	106.13229	0.0770616	283.78249
Sult2a1	6.14E-157	0	0.044696	156.21168	0.1890144	#NUM!
Gm43948	1.34E-77	1.6E-283	0.044501	76.872379	0.0884156	282.8013
Lsp1	5.22E-108	2.4E-18	0.044371	107.28213	0.0093583	17.619677
Wdr41	2.91E-06	4.43E-75	0.044348	5.5364456	0.0844807	74.353943
Rarb	5.31E-04	4.3E-161	0.044278	3.2750845	0.1290542	160.36286
E230029C0	4.75E-54	5.46E-12	0.044225	53.323179	0.0116894	11.262469
Usp5	5.59E-16	1.7E-124	0.044224	15.252906	0.0896777	123.76068
Atf3	9.97E-103	4.81E-38	0.044183	102.0013	0.0159962	37.317622
Sorcs3	5.52E-104	4.38E-39	0.04415	103.25781	0.017212	38.358996
Htt	1.09E-03	4.4E-136	0.044102	2.9642655	0.1299315	135.35364
Itgb2	1.81E-113	1.37E-16	0.044094	112.74326	0.0076206	15.862928
Terf2	1.48E-03	1.03E-48	0.044087	2.8288797	0.0814353	47.98563
Tmed7	4.07E-17	4.3E-167	0.044065	16.390247	0.1053988	166.36803

Psph	4.44E-36	1.6E-249	0.044041	35.352322	0.1009747	248.80821
Minpp1	6.19E-08	1.6E-123	0.043892	7.2083857	0.1156993	122.79213
Cd53	3.37E-108	1.29E-19	0.043766	107.47209	0.009912	18.888961
AU019990	3.05E-154	4.48E-89	0.043761	153.51623	0.0223401	88.348329
Zfp54	1.74E-45	1.6E-185	0.043738	44.758288	0.0767363	184.78404
Fermt3	1.14E-26	0.02343	0.043707	25.941926	0.0105775	1.630227
Slc22a29	1.30E-129	0	0.043679	128.8846	0.0926693	#NUM!
Gnpnat1	1.17E-10	4.54E-76	0.043664	9.9313805	0.0744176	75.342803
Cops7b	7.09E-04	3.5E-168	0.043664	3.1493482	0.1460535	167.45183
Mrps18b	1.24E-36	8.4E-143	0.04359	35.90581	0.0709773	142.0734
C1qb	1.29E-100	5.91E-33	0.04357	99.890399	0.0141461	32.228184
Qrsl1	3.58E-33	1.3E-156	0.043529	32.445651	0.0778475	155.87679
Kcnj8	5.91E-177	3.1E-296	0.043482	176.22824	0.0682689	295.5094
Btk	2.81E-105	1.49E-15	0.043338	104.5513	0.0079255	14.826222
A630001G2	4.35E-110	5.62E-22	0.043266	109.36134	0.0100342	21.250102
Riok1	1.64E-06	1.42E-61	0.043257	5.7863309	0.075768	60.846411
Mthfsd	1.84E-28	2.8E-124	0.043188	27.735398	0.0715624	123.55221
Ddx58	1.27E-05	1.61E-88	0.04314	4.8978766	0.0916072	87.792475
Gm28375	4.46E-24	0.000218	0.04311	23.350593	0.0146771	3.6609319
Casp4	7.77E-100	5.83E-23	0.043057	99.109567	0.010195	22.234387
Igsf6	1.19E-57	1.88E-07	0.042944	56.92446	0.0090713	6.7269649
Dhx33	1.80E-13	8.98E-76	0.042831	12.743703	0.0678729	75.046518
Srpr	4.03E-05	1.5E-121	0.042817	4.3949319	0.1131873	120.82534
Ccng1	2.31E-34	2.9E-157	0.042803	33.636823	0.0743178	156.53655
Hpgds	4.99E-59	2.36E-17	0.04277	58.302301	0.0148572	16.626233
Marcks	1.15E-74	4.47E-06	0.042665	73.940829	0.0077995	5.3501595
Gm16556	2.74E-126	4.48E-26	0.042566	125.56157	0.0084409	25.349042
Srp72	9.19E-03	3.9E-228	0.042309	2.036725	0.1927898	227.4036
Dnase1l3	1.93E-55	1.2E-156	0.042283	54.71455	0.0611114	155.90776
Hells	6.23E-14	3.8E-101	0.042191	13.205218	0.0816283	100.41577
Med24	3.19E-28	7E-146	0.042107	27.495664	0.0764892	145.15314
Gm19710	4.85E-15	1	0.042106	14.314358	0.0096232	0
Ccnd1	3.42E-81	1.7E-267	0.042086	80.466259	0.0779622	266.77959
Psmc6	1.28E-02	1.22E-45	0.042059	1.8936284	0.073984	44.912999
Mlh3	1.40E-08	1.07E-92	0.042026	7.8549159	0.087379	91.971349
Trub1	3.97E-07	8.3E-125	0.042021	6.4006759	0.1123482	124.08307
Prepl	7.36E-15	6.4E-105	0.041994	14.133255	0.0796214	104.19079
Ftsj3	3.93E-12	1.4E-106	0.041962	11.405949	0.0836929	105.85859
Tgfbi	4.65E-58	3.35E-07	0.041871	57.332096	0.0077164	6.4753604
Hars	6.36E-09	2.86E-93	0.041787	8.1964854	0.0821141	92.543878
Fbxl4	1.17E-04	6E-114	0.041744	3.9302423	0.1113059	113.22237
Tmem161b	6.40E-03	3.7E-65	0.041671	2.1936909	0.0918408	64.431912
Traf7	5.05E-11	1.1E-122	0.04165	10.296398	0.0938307	121.97422
Marco	3.57E-131	4.13E-77	0.041621	130.44731	0.0218903	76.383652
Ipo13	2.74E-30	1.4E-126	0.04159	29.562032	0.0653676	125.85611
H2-Ab1	3.95E-104	5.92E-24	0.041555	103.40386	0.0109131	23.227746
AB124611	4.22E-75	0.000444	0.041553	74.374539	0.0059802	3.3526063

Zfp330	2.01E-18	1.79E-92	0.041499	17.696378	0.0668096	91.745973
Sirpb1b	2.99E-96	7E-24	0.041457	95.524994	0.0097881	23.154804
Dynll1	9.13E-75	8.5E-191	0.041443	74.039731	0.0610714	190.07233
Slfn2	1.08E-98	7.19E-24	0.041365	97.965089	0.0110784	23.143209
Ms4a6c	2.44E-104	1.28E-46	0.041318	103.61334	0.0165307	45.893289
Nono	1.07E-05	2.69E-65	0.041078	4.9706307	0.0775205	64.570077
Glipr1	1.26E-79	5.18E-26	0.041077	78.899547	0.0134788	25.285486
Smg5	3.13E-05	1.01E-69	0.041068	4.5047047	0.0822331	68.994758
Utp11	4.32E-11	2.7E-145	0.041059	10.364555	0.1073258	144.56666
Pkp1	7.92E-170	1.9E-286	0.040916	169.10113	0.0668943	285.7295
Gle1	6.59E-05	2.22E-87	0.040877	4.1811924	0.0975388	86.653341
H2-Aa	3.75E-96	2.4E-26	0.040873	95.425967	0.0122563	25.6195
Nlrp1b	4.42E-84	3.98E-09	0.040858	83.354666	0.006737	8.4002036
Parvb	7.60E-109	3.08E-39	0.040799	108.11923	0.0153386	38.511228
Ccdc59	2.93E-10	4.1E-81	0.040775	9.5326468	0.0754796	80.387124
Polb	1.56E-02	2.47E-72	0.040724	1.8068592	0.0953951	71.607311
Oxct1	7.51E-53	1.42E-20	0.040716	52.124626	0.0141564	19.84704
Ap1b1	1.67E-03	9.1E-99	0.040711	2.7764809	0.1101571	98.04283
Fbxo45	1.72E-15	1.6E-126	0.040606	14.764689	0.0831971	125.80685
Acadvl	1.61E-07	1.48E-61	0.040588	6.7940021	0.0713634	60.830306
Ciz1	1.32E-07	1.15E-60	0.040587	6.8786478	0.0699937	59.938239
Ufsp2	1.11E-06	1.9E-120	0.040561	5.9557161	0.1003239	119.71014
Fbh1	1.42E-08	6.44E-68	0.040444	7.848848	0.0705499	67.191042
Fam72a	4.64E-77	6.8E-202	0.040413	76.333055	0.064332	201.16777
Zswim4	7.76E-10	1.17E-71	0.040407	9.1102757	0.0679814	70.931913
Cycs	3.18E-25	8.6E-109	0.040312	24.497206	0.060803	108.0671
Ier2	1.29E-21	5.41E-06	0.040302	20.887944	0.0170691	5.2671263
Plxnc1	1.43E-23	4.24E-07	0.04027	22.845559	0.0140595	6.3728837
Cope	5.97E-08	4.2E-133	0.040265	7.2238109	0.1031304	132.37533
Eif2b4	8.35E-11	5.8E-141	0.040245	10.078443	0.1011369	140.23973
Arrb2	2.33E-17	1	0.040171	16.631968	0.0026717	0
Creb5	1.42E-85	1.92E-12	0.040167	84.849107	0.0068866	11.717568
Nt5c	1.00E-43	4.1E-152	0.040162	42.998319	0.0637627	151.3888
Ulbp1	4.94E-89	5.09E-26	0.040146	88.306613	0.0117873	25.292862
Fbxl7	3.72E-09	1	0.040125	8.4299515	0.0096253	0
Exosc8	2.06E-31	2.1E-143	0.040086	30.686257	0.0703569	142.67123
Galt	7.38E-24	6.9E-152	0.040031	23.131956	0.0782681	151.15865
Rtcb	1.27E-13	1.99E-81	0.040021	12.895259	0.0647321	80.70211
Adap1	7.84E-125	7.82E-74	0.039922	124.10583	0.0204459	73.106751
Chmp7	3.71E-23	4E-172	0.039842	22.430849	0.0848218	171.40329
AU022793	4.35E-122	2.8E-32	0.039806	121.36201	0.010694	31.552282
Blmh	2.06E-02	2.8E-124	0.039791	1.6854073	0.1264836	123.55657
Gm34411	1.67E-55	2.03E-29	0.039741	54.776517	0.0212026	28.693186
Gm15886	7.79E-23	8.56E-08	0.039666	22.108572	0.0165802	7.0672746
Selplg	2.01E-94	5.76E-20	0.039651	93.696074	0.0093956	19.239423
Qars	8.11E-19	4E-110	0.039636	18.091122	0.0726636	109.40016
Gm13561	1.32E-26	1.77E-10	0.039599	25.88101	0.0181633	9.7526523

Trappc11	1.45E-07	1.72E-77	0.039411	6.8397646	0.0791638	76.765248
Aox3	1.73E-12	0	0.039377	11.76262	0.3445399	#NUM!
Fam92a	1.26E-31	1.2E-184	0.03936	30.900484	0.0803097	183.91435
6030458C1	4.52E-15	1.2E-123	0.039347	14.345139	0.0834592	122.92782
Hspa14	1.13E-15	1.9E-123	0.039343	14.946225	0.0803023	122.73182
Psmd2	7.96E-07	3.54E-73	0.039319	6.0992672	0.0742505	72.450428
Atp5j2	6.25E-08	3.66E-85	0.039301	7.2042526	0.0775586	84.436735
Ipo11	5.45E-03	3.12E-68	0.039233	2.2632992	0.0879679	67.505749
A630089N(	2.36E-09	4.5E-155	0.039168	8.6272609	0.1116714	154.34655
Zfp951	3.04E-06	3.33E-81	0.039158	5.5167999	0.0850706	80.477933
Copb2	9.37E-10	1.5E-124	0.039122	9.0284624	0.0958672	123.82978
Usp1	3.46E-14	1.7E-128	0.039085	13.46068	0.0859302	127.76428
Junb	1.10E-17	1	0.039064	16.959812	0.0104371	0
Eif3e	7.36E-10	1.38E-73	0.03902	9.1332376	0.0719566	72.860057
Ms4a6d	2.48E-123	8.39E-31	0.038814	122.60518	0.0097843	30.076164
Psmd8	7.79E-14	3E-122	0.03872	13.10842	0.0769587	121.51596
Slc44a2	1.27E-39	0.015056	0.038561	38.896334	0.0073338	1.8222958
Il15	2.35E-14	1	0.038555	13.629466	0.0032977	0
Gm20559	1.36E-34	0.910972	0.038486	33.865105	0.0065668	0.0404948
Cmtm7	7.26E-76	8.09E-34	0.038414	75.139151	0.0155519	33.092299
Foxred1	7.00E-14	7E-140	0.03837	13.154719	0.0833518	139.15784
Card11	5.81E-52	0.876807	0.038288	51.235795	0.0044466	0.0570958
Apobec1	2.69E-03	3.2E-149	0.03824	2.569815	-0.1096903	148.49989
Gm42067	4.27E-31	1.5E-126	0.038207	30.369704	0.0636953	125.83546
Timm10b	9.80E-28	3.2E-145	0.038206	27.008764	0.0681339	144.49818
Oas2	1.10E-67	1.56E-09	0.038203	66.956655	0.007996	8.8063167
Exosc1	4.34E-17	2.1E-90	0.038143	16.362602	0.0655299	89.678573
Vsir	5.89E-90	2.15E-14	0.03808	89.229916	0.0069167	13.667178
H2-Eb1	3.11E-104	2.85E-30	0.037831	103.50687	0.0111412	29.545291
Mmp13	1.87E-107	2.83E-37	0.037826	106.7276	0.0099444	36.548618
Mtmr10	3.41E-02	2.34E-92	0.037803	1.4671216	0.1170975	91.631572
Gm13391	1.12E-122	1.7E-271	0.037789	121.95102	0.0669358	270.7739
Klhl20	7.74E-04	8.01E-48	0.037745	3.1110439	0.0667851	47.096108
Slc25a36	5.83E-60	8.92E-09	0.037724	59.234602	0.0090366	8.0494938
Gm42477	1.76E-43	1.4E-151	0.037689	42.754644	0.0612577	150.84693
Flad1	1.87E-09	7.1E-101	0.037668	8.7290708	0.0842464	100.15119
Ctu2	2.00E-31	3.8E-117	0.037662	30.699075	0.0583665	116.42083
Fam129a	4.87E-67	2.77E-25	0.03764	66.312061	0.0133075	24.557155
Ccr1	1.19E-111	1.41E-17	0.037628	110.92572	0.004922	16.851487
Rap1gap	3.02E-41	4.6E-174	0.037622	40.520129	0.06574	173.33834
Tubb2a	6.55E-09	1.2E-118	0.03762	8.1839045	0.0926469	117.90411
Pomt1	1.40E-23	1E-142	0.03754	22.853397	0.074578	141.9928
Jdp2	4.77E-88	8.51E-14	0.037474	87.321709	0.0074653	13.070269
Ankrd16	2.54E-05	6.01E-82	0.037441	4.5947881	0.0851835	81.221225
Il1r2	1.48E-83	3.29E-15	0.037358	82.830037	0.0075016	14.483308
Cct3	5.85E-05	2.18E-50	0.037233	4.232846	0.0680717	49.66113
9930111J2	1.50E-83	1.52E-20	0.037192	82.822522	0.009868	19.818287

Tspan5	2.71E-23	1	0.037176	22.567268	0.0057955	0
Ncf4	4.96E-112	2.99E-20	0.037129	111.30415	0.0066896	19.523752
Gm37168	1.34E-112	4.27E-14	0.036959	111.87396	0.0046138	13.369355
Gm14569	1.74E-120	2.87E-66	0.036949	119.75976	0.0197175	65.542859
Mylip	1.33E-28	1	0.036946	27.876024	0.0044533	0
Gm17749	1.97E-97	2.27E-06	0.036896	96.704947	0.0032665	5.6434533
Fastk	6.62E-11	8.1E-121	0.036859	10.179255	0.0851885	120.09083
Ms4a7	5.45E-116	9.21E-41	0.036834	115.26322	0.0109456	40.035652
Kitl	1.65E-45	3.85E-07	0.0368	44.783562	0.0076939	6.4145785
Hmcn2	2.47E-16	1.66E-93	0.036758	15.60665	0.0656283	92.780676
Uck2	3.05E-07	7.95E-83	0.036739	6.5160387	0.0756021	82.099901
Cc2d1a	1.14E-18	2.1E-102	0.036695	17.941464	0.0634864	101.67194
Ddit4	4.27E-16	3.5E-166	0.036592	15.369596	0.0921754	165.4508
Mpzl3	1.35E-07	1.46E-83	0.036532	6.8690676	0.0767097	82.834217
Atad1	1.09E-02	1.57E-59	0.036519	1.9644071	0.0811142	58.804548
Gm14221	1.05E-82	6.21E-17	0.036482	81.977587	0.008072	16.207105
Gm28905	5.42E-38	3.8E-233	0.036445	37.266058	0.0902286	232.42212
Smc2	2.37E-33	7.4E-134	0.036407	32.625671	0.0592667	133.13215
Setd6	1.99E-32	1.7E-134	0.036405	31.700239	0.0617407	133.78086
Zmat3	2.99E-14	1.9E-146	0.036303	13.524237	0.0851196	145.71036
Adam8	6.11E-89	4.38E-39	0.03628	88.213771	0.0137356	38.358996
Il1f9	2.80E-100	2.54E-13	0.036244	99.553258	0.0046719	12.595784
Urb1	8.15E-17	3.16E-93	0.036232	16.088688	0.0624655	92.500169
Ndufaf2	2.20E-02	1.63E-45	0.0362	1.6579953	0.0725226	44.788904
Cped1	3.47E-04	3.3E-242	0.036198	3.4596064	0.220463	241.4868
Snx19	3.66E-04	1.71E-93	0.036141	3.4367423	0.0933911	92.767807
Afp	1.34E-89	3.7E-212	0.036081	88.87212	0.059344	211.43525
Tnfaip3	3.77E-53	3.85E-11	0.036069	52.423881	0.0111387	10.414839
Blnk	2.12E-67	2.19E-05	0.036041	66.673348	0.0061056	4.6604764
Snf8	3.32E-07	8.43E-66	0.036011	6.4788984	0.0665073	65.073999
Gm14410	8.86E-08	3.21E-60	0.035936	7.0525413	0.0593428	59.493432
Vps11	1.92E-05	2.22E-53	0.035932	4.7163397	0.0672211	52.653443
Zfp112	1.52E-18	1.23E-89	0.035913	17.817948	0.0609269	88.911812
Twnk	2.41E-14	1.99E-77	0.035891	13.617958	0.0609822	76.700384
Ints4	6.53E-05	6.35E-72	0.035808	4.184845	0.0804282	71.197028
Edc4	6.19E-08	4.51E-86	0.035783	7.2081022	0.0726215	85.345386
Exoc7	4.15E-11	9.63E-77	0.035781	10.381882	0.0641924	76.016156
Zfand2a	2.19E-20	6.44E-96	0.035746	19.658748	0.0556154	95.190933
Vcam1	2.42E-69	1.05E-23	0.035696	68.615934	0.0128527	22.978107
Lig3	6.80E-07	5.5E-101	0.035515	6.1673456	0.0869106	100.25919
Gm44767	4.52E-19	3.5E-106	0.035506	18.344754	0.0647624	105.4576
Trim12a	7.52E-04	1	0.035499	3.1236644	-0.0062393	0
Cmklr1	7.97E-86	6.67E-06	0.03549	85.098639	0.0038009	5.1758045
Rps6kb2	2.35E-08	3.8E-124	0.035468	7.6297853	0.0907187	123.42003
Trim11	2.68E-03	7.27E-50	0.035455	2.5726643	0.0674357	49.138511
Mat2b	5.24E-09	8.93E-73	0.035454	8.2803302	0.0637854	72.049389
Recql5	4.60E-02	2E-126	0.035437	1.3373284	0.1206333	125.70761

Gm49970	2.16E-07	1.01E-84	0.035397	6.6654241	0.0767429	83.995677
Spns1	7.21E-08	8.64E-77	0.035357	7.1422869	0.0694377	76.063601
Nrxn2	2.99E-131	1.25E-61	0.03533	130.52488	0.0169754	60.904044
Mettl16	1.03E-02	5.44E-46	0.035313	1.9884612	0.0690222	45.264742
Abi3	1.80E-84	2.33E-22	0.035276	83.743737	0.009406	21.632756
Asah2	2.99E-04	5.04E-60	0.035251	3.5247558	0.0679343	59.297956
BC003965	3.58E-12	2.6E-99	0.035235	11.446465	0.071673	98.58592
Mrto4	2.00E-36	1E-137	0.03522	35.698346	0.0582428	136.99342
Fdx1	2.51E-02	3.9E-107	0.035097	1.5998437	0.109359	106.41056
Ntmt1	4.78E-08	4.18E-63	0.03505	7.3202787	0.0644557	62.378464
Mfsd12	1.78E-56	5.53E-09	0.035038	55.750346	0.0082108	8.2574384
Abcc12	1.40E-75	3.32E-06	0.03503	74.85372	0.0062805	5.4784031
Micos13	3.41E-24	1.5E-120	0.034979	23.467298	0.0597256	119.82278
Plxdc1	1.32E-41	1	0.03489	40.878097	0.0036695	0
Mios	4.31E-07	1.39E-58	0.034831	6.3658902	0.0641086	57.857465
Clec1b	2.91E-78	7.36E-24	0.034831	77.535443	0.0103133	23.132983
Ccdc17	8.90E-52	3.9E-188	0.034822	51.050763	0.0636045	187.41421
Trap1	2.02E-02	1.37E-86	0.034784	1.6947319	0.0961002	85.862485
Atp8b5	7.12E-67	7.2E-193	0.034767	66.147368	0.0579374	192.13999
Tom1l1	2.54E-02	5.26E-58	0.03475	1.5958992	0.0808658	57.278638
Themis2	6.01E-75	1.34E-10	0.034703	74.22087	0.0064206	9.8717986
Sla	1.37E-83	3.5E-19	0.034698	82.862872	0.0086077	18.456244
Slfn8	1.72E-64	4.79E-07	0.03467	63.764045	0.0055402	6.3192747
Usp36	2.53E-02	7.25E-61	0.034665	1.5961157	0.0823113	60.139584
Klhl26	1.79E-05	7.5E-113	0.034631	4.748284	0.0985548	112.12229
Yipf3	3.70E-17	4.88E-92	0.034604	16.432142	0.0575874	91.311457
Bet1l	2.40E-07	8.1E-136	0.034602	6.6195767	0.09981	135.09015
Plod1	5.41E-04	5.91E-49	0.03456	3.266728	0.0595267	48.228616
M6pr	1.94E-09	3.87E-68	0.034476	8.7132428	0.0584189	67.412454
Tgfb1	6.88E-61	1.3E-05	0.03441	60.162157	0.0062667	4.8865097
Kin	4.93E-04	3.81E-67	0.034394	3.3072963	0.0754092	66.419087
B3gntl1	3.53E-04	1.05E-47	0.034363	3.4527467	0.0577343	46.979037
Plpp5	4.47E-09	2.4E-166	0.034358	8.3497855	0.1007868	165.62857
Uggt2	2.40E-19	2.6E-122	0.034349	18.619551	0.0682739	121.58127
C3ar1	2.08E-106	6.6E-21	0.034246	105.68167	0.0067044	20.180179
Mnt	1.32E-09	1	0.034228	8.879887	0.003872	0
Pfkp	4.09E-91	4E-54	0.034189	90.387865	0.0184444	53.397525
Gm16000	8.03E-48	3.15E-06	0.034107	47.095402	0.0094649	5.5021594
Acot8	1.22E-05	7.07E-64	0.034012	4.9118875	0.0712138	63.150288
Tmem273	6.10E-89	8.16E-22	0.033866	88.214593	0.0089834	21.088252
Tifa	2.63E-17	0.019361	0.033854	16.579291	0.0112585	1.7130802
Rab7b	3.40E-109	6.37E-30	0.033785	108.46848	0.0087595	29.196149
Mpv17l	7.85E-03	4.22E-68	0.033759	2.1051044	0.0847849	67.374636
Fam71f2	4.42E-90	4.29E-42	0.03364	89.355057	0.0163422	41.367402
Pde1b	3.58E-99	4.83E-29	0.033609	98.445652	0.010585	28.316052
Emb	6.92E-81	1.2E-13	0.03354	80.159971	0.0068365	12.919448
Apobec3	1.07E-62	2.35E-22	0.033502	61.970063	0.0111447	21.628146

Mettl6	1.13E-03	4.12E-88	0.033458	2.9466388	0.0882603	87.385041
0610009E0	6.96E-14	1.67E-86	0.033332	13.157355	0.0611229	85.776942
Ahnak2	4.12E-89	5.05E-42	0.033328	88.384578	0.0127743	41.296534
Tnik	4.14E-13	8.1E-193	0.033161	12.383028	0.1027502	192.09295
Hgf	3.42E-10	1	0.032997	9.4662348	0.0040735	0
Tm6sf1	1.10E-78	5.53E-28	0.032989	77.957518	0.011539	27.257357
Tnfrsf10b	3.05E-98	5E-246	0.03298	97.516197	0.061392	245.30233
Prkcsh	1.85E-11	6.4E-116	0.032953	10.733102	0.0746156	115.19206
Flrt2	3.29E-80	1.14E-15	0.032928	79.482983	0.0062387	14.941797
Bcl2	6.13E-25	0.003466	0.032839	24.212621	0.0088841	2.4601715
Bbs5	3.61E-26	5.6E-107	0.032829	25.4419	0.0546839	106.24833
Adrm1	1.98E-10	4.3E-135	0.03279	9.7030958	0.0831694	134.37096
Ppp1r12c	4.87E-04	9.83E-47	0.03278	3.3127649	0.0536243	46.007591
Eri2	1.10E-10	2.72E-72	0.032645	9.9586924	0.0596389	71.565645
Rrnad1	4.80E-02	5.24E-73	0.032634	1.3188424	0.085042	72.281065
Ddt	1.64E-10	3.7E-145	0.032605	9.7846027	0.0835255	144.43114
Tyrobp	8.22E-90	1.84E-31	0.032524	89.085235	0.0113786	30.734651
Ubl5	2.97E-19	6.23E-90	0.032471	18.526817	0.049813	89.205419
Tatdn1	5.78E-03	7.77E-43	0.032426	2.2381851	0.0624035	42.109449
F630028O1	1.97E-94	3.41E-24	0.032388	93.705627	0.0083688	23.467338
Phospho2	5.71E-11	5.4E-77	0.032338	10.243262	0.0589446	76.267679
Sharpin	8.06E-12	2.3E-115	0.032288	11.093807	0.0732462	114.63119
Sh2d3c	1.91E-13	1	0.032286	12.719642	0.001866	0
Amz1	6.47E-70	3.65E-10	0.032247	69.188934	0.0073619	9.4372188
Btbd11	8.44E-38	4.73E-08	0.032239	37.073665	0.0079347	7.3255883
Clptm1l	8.00E-08	4.1E-106	0.032181	7.0969342	0.0750622	105.38256
Snapc4	3.39E-07	2.41E-88	0.032165	6.4698733	0.0723973	87.618521
Uckl1	1.96E-05	5.62E-61	0.032162	4.7082033	0.0614704	60.250219
Gdpd2	1.23E-130	2.4E-208	0.032156	129.91027	0.04997	207.61616
C1qa	3.49E-53	3.33E-13	0.03213	52.457147	0.0098197	12.477162
Coro1a	6.80E-68	2.4E-09	0.03211	67.167402	0.0067034	8.6206653
Trim68	1.29E-58	5.2E-173	0.032109	57.889386	0.0538855	172.2845
Golga7	1.46E-03	3.06E-68	0.031994	2.8362818	0.0727592	67.513608
Il1rn	1.33E-91	1.63E-32	0.031991	90.876253	0.0113395	31.787628
Wdr83	3.61E-12	9.8E-140	0.031967	11.442358	0.0787277	139.00673
Lst1	3.99E-87	8.75E-25	0.031931	86.399058	0.0086212	24.057863
Vps52	4.32E-07	6.73E-73	0.031927	6.3644531	0.0623009	72.171789
Ndufa6	3.52E-05	1.47E-60	0.031909	4.4530261	0.0619578	59.831521
Ptafr	4.86E-88	3.81E-07	0.031881	87.313086	0.003869	6.4194917
Iscu	1.12E-08	5.6E-107	0.031848	7.9505279	0.0714816	106.25338
Morf4l2	3.25E-02	3.23E-81	0.031802	1.4881119	0.0817295	80.490245
Apeh	1.75E-09	4.11E-70	0.031783	8.7581931	0.0599226	69.386331
E330020D1	1.98E-87	2.88E-22	0.031697	86.702475	0.0089645	21.540938
Slc35b1	3.09E-11	8E-153	0.031657	10.510266	0.0880233	152.0972
H2-T24	1.57E-34	1	0.031635	33.804018	0.0044392	0
Ints14	1.06E-06	7.97E-61	0.031613	5.9728128	0.0597986	60.09863
C1qc	6.57E-75	4.05E-25	0.031581	74.182269	0.010899	24.392256

Drg2	1.76E-09	1.2E-102	0.03156	8.7550169	0.0705503	101.9151
Lpcat2	1.10E-83	2.65E-06	0.031532	82.958691	0.0042331	5.5769197
Stxbp2	3.54E-02	3.06E-41	0.031529	1.450514	0.0548987	40.514464
Hdgfl2	4.26E-02	5.44E-56	0.031384	1.3704478	0.0754559	55.264084
Fgd2	1.90E-63	1.26E-20	0.031376	62.72052	0.0100818	19.898325
Gm12602	1.79E-60	1.5E-197	0.031357	59.748296	0.0591982	196.83153
Dusp14	1.11E-84	9.8E-218	0.031323	83.956047	0.0571944	217.00744
Rbm19	4.18E-09	2.18E-64	0.031316	8.3786575	0.0575384	63.66071
Coro6	1.72E-110	1.8E-197	0.031308	109.76512	0.0474748	196.74337
Dgat1	4.42E-03	2.23E-98	0.031307	2.3541332	0.0855137	97.652572
Ing3	4.05E-04	8.58E-71	0.031287	3.3925676	0.0723627	70.066317
Il1b	2.61E-68	2.96E-14	0.03128	67.58266	0.005475	13.528862
Rab36	1.95E-47	4.4E-153	0.031217	46.709905	0.0524343	152.35242
Lrmp	6.77E-63	1.74E-11	0.031207	62.169213	0.0084595	10.760306
Ift20	2.15E-06	2.7E-103	0.031203	5.6666688	0.0758906	102.57392
mt-Cytb	8.35E-17	5.8E-103	0.031165	16.078093	0.0558894	102.23335
Arfip2	3.10E-08	3.2E-222	0.031161	7.508408	0.1160833	221.4974
Trpm6	1.27E-40	0	0.031024	39.896648	0.0980509	#NUM!
Tenm4	2.91E-42	1	0.031009	41.535925	0.0019691	0
Dnajb11	1.52E-04	1.3E-109	0.030978	3.8189607	0.0920825	108.89526
Calhm2	8.13E-41	3.32E-16	0.03087	40.090107	0.0121501	15.478502
St8sia4	6.05E-55	1.42E-13	0.030823	54.218175	0.0086222	12.8487
Mrpl3	1.71E-02	1.12E-45	0.0308	1.7667987	0.0622392	44.951228
Pop5	3.42E-26	5.2E-150	0.030695	25.46579	0.062396	149.28463
Ppib	5.70E-06	5.9E-110	0.030657	5.2438133	0.0813852	109.22815
Mri1	3.37E-02	1.46E-43	0.030655	1.4721341	0.0624828	42.835041
Cd300c2	9.20E-90	5.55E-22	0.030642	89.036082	0.0083159	21.255764
Tsc2	1.11E-06	5.9E-126	0.030524	5.9541197	0.0863831	125.23261
Hacd4	1.63E-43	5.86E-05	0.030506	42.788001	0.0064339	4.2317871
Gm21188	7.66E-87	3.93E-09	0.030456	86.115582	0.0033979	8.4055224
Cd63	6.83E-25	0.000471	0.030293	24.165288	0.0095314	3.3273713
Ampd3	1.03E-63	0.006632	0.030235	62.987198	0.0040756	2.1783385
Myl12a	1.35E-11	3.9E-114	0.030186	10.870218	0.0688887	113.40983
Gm30505	5.74E-09	1.3E-137	0.030173	8.2412306	0.0863769	136.87279
Gm10447	1.50E-77	1.5E-260	0.030142	76.824215	0.0662102	259.81016
Cd200r1	2.84E-66	2.51E-13	0.030029	65.546693	0.0067579	12.600736
Adam19	1.28E-28	0.001358	0.029968	27.892901	0.0073065	2.8670502
Cd86	6.44E-54	5.61E-09	0.029946	53.191168	0.0061633	8.2511557
Fcgr2b	1.26E-17	1	0.029907	16.898763	0.0059725	0
Letm1	9.13E-04	1.19E-79	0.029868	3.0393199	0.0729775	78.925016
Sipa1	8.42E-14	0.882018	0.029798	13.07473	0.0064568	0.0545227
Tmem82	5.23E-03	2.7E-112	0.029781	2.2817586	0.0937655	111.5734
Nfia	1.23E-11	5.9E-210	0.029766	10.908673	0.1987781	209.22778
Rfng	3.69E-29	1.5E-114	0.029744	28.432821	0.0489202	113.81685
Mto1	2.31E-03	2.56E-45	0.029614	2.6360309	0.0595124	44.590937
Tmem86b	9.18E-05	2.24E-88	0.029597	4.0371689	0.0743148	87.648941
Irf2bpl	1.94E-20	1	0.029547	19.712927	0.0072347	0

Srgn	1.63E-43	9.45E-06	0.02953	42.788088	0.0060083	5.0247746
Aff3	5.02E-16	0.227621	0.029482	15.299647	0.0081527	0.6427885
Stk16	3.58E-04	5.9E-171	0.029467	3.4458971	0.1129413	170.22593
Sult1e1	1.61E-60	6.8E-226	0.029458	59.794327	0.0806152	225.16889
Tlr13	2.68E-91	8.31E-23	0.029349	90.571122	0.007352	22.080472
Cd93	5.97E-80	4.3E-13	0.02926	79.224373	0.0060725	12.366345
Lhfpl2	8.14E-27	1	0.029218	26.089628	0.004158	0
Gbp8	3.86E-26	1.73E-07	0.02916	25.413875	0.0103298	6.7624647
Pigq	5.17E-10	3.2E-105	0.029142	9.2866995	0.0672905	104.48833
Eif3d	1.58E-08	6.98E-88	0.029085	7.800764	0.0618731	87.156176
Zfp560	9.62E-06	3.53E-57	0.029072	5.0167138	0.0556011	56.451737
Phb2	8.47E-04	7.3E-59	0.029036	3.0721797	0.0629917	58.136634
Gm4316	5.44E-04	1	0.02901	3.26458	-0.0122087	0
Myo1g	6.06E-72	6.89E-24	0.02901	71.217517	0.0105586	23.161601
BC029722	6.38E-44	3.4E-135	0.028936	43.195121	0.0457906	134.46487
Stoml2	1.89E-08	8.73E-64	0.028902	7.7228034	0.0545592	63.059158
Mrps35	3.51E-08	8.75E-60	0.028741	7.4549227	0.0523963	59.057782
Dapp1	2.33E-59	2.31E-10	0.028642	58.633129	0.006847	9.6373246
Ercc8	1.53E-07	4.16E-65	0.028641	6.8143533	0.0566211	64.380397
Bach2os	5.76E-12	1	0.028528	11.239934	0.0028789	0
Ust	3.65E-21	7.86E-05	0.028507	20.438189	0.0094377	4.1044199
Clec4n	8.49E-73	8.5E-20	0.028386	72.07106	0.0078725	19.070463
Mctp1	1.43E-26	1	0.028375	25.845207	0.0048081	0
Ms4a6b	2.51E-60	1.49E-21	0.028346	59.60005	0.0098696	20.825955
Dennd1c	6.02E-61	2.12E-09	0.028307	60.220464	0.0061808	8.6741289
Smarce1	5.43E-03	2.97E-48	0.028301	2.2653138	0.0585855	47.527063
Snx20	1.33E-82	4.06E-19	0.028208	81.876417	0.0064352	18.391343
Rpp21	4.23E-10	2.28E-78	0.028109	9.3741658	0.0538259	77.641537
Far1	1.77E-39	1	0.028071	38.752348	0.0038892	0
Glis1	7.67E-64	1.52E-25	0.027949	63.115087	0.0119423	24.817638
Soga1	2.46E-56	0.002156	0.02788	55.608209	0.0043464	2.6664389
Prpf31	7.73E-15	5.64E-78	0.027797	14.11186	0.047424	77.248647
Ndufa2	4.46E-06	5.62E-72	0.02778	5.3507984	0.0560446	71.249995
Fcgr4	1.94E-76	1.02E-18	0.027755	75.713032	0.0071726	17.990423
Gstt3	3.59E-12	1.4E-104	0.027718	11.445002	0.0610915	103.85342
Trpv2	7.45E-81	1.02E-32	0.027714	80.128079	0.0107775	31.991697
Serpina6	1.21E-16	7.13E-95	0.027691	15.918219	0.0518468	94.146812
Ncf1	3.36E-59	2.45E-10	0.027569	58.473249	0.0065248	9.6100845
Cenpt	2.98E-43	1.9E-181	0.027567	42.525645	0.0557443	180.71043
Dnmt3aos	2.97E-27	1	0.027441	26.527922	0.0034115	0
Blzf1	2.09E-05	2.9E-68	0.027441	4.6802442	0.0628203	67.537784
Bag6	7.01E-03	9.24E-98	0.027433	2.1541912	0.081165	97.034128
Tmem208	3.40E-04	6.88E-65	0.027392	3.469089	0.0589095	64.16225
Scamp3	2.88E-06	8.12E-65	0.02732	5.5412927	0.0554301	64.090685
Gm46516	4.66E-12	1	0.0273	11.33162	0.0052726	0
Mtx1	1.10E-09	9.64E-74	0.027252	8.9585812	0.0529863	73.015955
Wdr74	1.04E-09	4.06E-83	0.027194	8.9836132	0.0565109	82.391753

P2ry6	3.94E-69	5.68E-13	0.027184	68.404806	0.0064616	12.245403
Clic1	5.88E-12	1	0.02715	11.230789	0.0069788	0
Wls	3.21E-31	0.007361	0.027124	30.493338	0.0059708	2.1330613
Gm46224	2.92E-81	8.99E-18	0.027081	80.53476	0.0068083	17.046077
4933439K1	1.09E-73	1.79E-12	0.027027	72.960929	0.0046394	11.746643
Gm4070	1.91E-18	1	0.026953	17.719794	-0.0011829	0
Rnf150	8.94E-14	1	0.026944	13.048572	-0.0007944	0
Capg	6.16E-78	7.08E-13	0.026924	77.210313	0.0050169	12.150108
Zfp408	7.06E-04	2.32E-95	0.026913	3.1513338	0.0750602	94.635282
1700109H(	1.31E-02	2.03E-43	0.026852	1.8817379	0.0568577	42.692068
Fgd3	4.54E-72	1.75E-13	0.026806	71.343241	0.0054588	12.756024
Ctsk	1.00E-05	1	0.026717	4.998397	-0.0027296	0
Tagln2	3.78E-66	3.33E-30	0.026636	65.422225	0.0118642	29.477164
Trmu	2.40E-04	4.35E-77	0.026606	3.6198731	0.0668603	76.361662
Chpf2	7.74E-05	1.53E-76	0.02658	4.1115061	0.0638514	75.815155
Vwf	3.40E-55	1.82E-18	0.026576	54.468635	0.0078604	17.74011
Cytip	2.29E-62	1.13E-25	0.026573	61.639836	0.0096254	24.948531
Sec13	3.26E-13	1.89E-75	0.02656	12.486603	0.0462657	74.72429
D930048N:	9.80E-08	1.24E-61	0.026542	7.0086789	0.052612	60.907306
Scyl1	2.21E-08	7.32E-70	0.026495	7.6550803	0.051471	69.135217
Dpysl2	3.47E-44	7.9E-174	0.026449	43.459445	0.0522292	173.10406
Man2c1	8.14E-06	9.26E-68	0.026412	5.0895115	0.0577689	67.033376
Sirt4	1.03E-09	5.1E-81	0.026349	8.9860275	0.0535407	80.292427
Sp140	2.09E-13	1	0.026294	12.679382	0.0025571	0
Gm13912	6.05E-81	1.32E-29	0.026288	80.218068	0.0109474	28.879452
Pacc1	2.87E-23	0.000115	0.026188	22.541946	0.0083421	3.9402558
Rnf180	6.53E-75	3.65E-27	0.026034	74.185261	0.0092729	26.438097
Gm13599	4.32E-75	1.12E-11	0.026029	74.365017	0.0061866	10.950903
Zfp760	1.52E-07	4.64E-68	0.026028	6.8171876	0.0527132	67.333168
Mfsd8	4.69E-02	3.34E-61	0.025978	1.3284809	0.0612926	60.476779
Ccr5	4.93E-53	1.92E-10	0.025972	52.306886	0.0064963	9.7177035
Mrpl19	3.51E-12	1.23E-76	0.025913	11.45471	0.0474462	75.911342
Tpm4	1.27E-06	1	0.025884	5.8956197	0.0006564	0
Fcgr3	2.02E-66	1.05E-25	0.025857	65.694216	0.0106133	24.980255
Gm26760	8.56E-66	1.26E-17	0.025787	65.067555	0.0093454	16.900441
Trem2	2.80E-100	8.36E-17	0.025773	99.553594	0.0046102	16.077888
Galnt6	2.84E-67	2.89E-05	0.025712	66.547421	0.0039531	4.538676
Slc39a7	4.36E-02	2.81E-60	0.025611	1.3603023	0.0609197	59.551598
Dcxr	9.38E-12	3.2E-186	0.025601	11.027895	0.08163	185.49105
Mgat4a	8.97E-59	5E-18	0.025577	58.047255	0.0078485	17.301298
Mdfi	2.84E-100	2.92E-41	0.025526	99.546121	0.0112382	40.534143
Gapdh	2.40E-10	4.3E-72	0.025455	9.6199231	0.0479988	71.366759
Higd1a	4.57E-02	1.76E-41	0.025427	1.3403336	0.0546502	40.755151
Gm14325	1.29E-06	4.66E-84	0.025388	5.8889635	0.0627177	83.331783
Triap1	8.22E-27	6.8E-108	0.025371	26.085359	0.0439708	107.16754
Bud31	1.22E-08	2.89E-64	0.025364	7.9139636	0.0475898	63.538609
Plcb2	2.52E-69	1.26E-07	0.025354	68.598566	0.0042415	6.899922

Trim30b	2.02E-61	1.11E-06	0.025295	60.694986	0.0039213	5.9556723
Vim	7.32E-55	1.96E-13	0.025251	54.135486	0.0068796	12.708493
Zc3h8	7.61E-05	1.59E-80	0.025226	4.1186796	0.0636539	79.799808
Rhoh	7.42E-52	4.8E-08	0.025225	51.129332	0.0046917	7.3190362
Ifi209	1.44E-41	1	0.025177	40.841264	0.0026503	0
E230032D2	1.10E-63	4.95E-05	0.025173	62.957793	0.0036788	4.3053794
Acod1	9.55E-58	2.38E-06	0.025167	57.020189	0.0026618	5.6237078
Tut1	2.18E-03	1.66E-51	0.025083	2.6611743	0.0540129	50.779133
Sgsm3	2.67E-04	4.15E-50	0.025077	3.5737767	0.0513524	49.381435
Smpd4	1.39E-03	2.22E-72	0.025063	2.8579056	0.063904	71.653705
Relb	1.52E-11	1	0.024955	10.819231	0.0051582	0
Gm1123	5.54E-19	0.417416	0.024955	18.256283	0.0060454	0.3794304
Gm33699	9.25E-51	3.9E-180	0.024914	50.033741	0.0514183	179.40738
Ppp1r15a	3.47E-10	2.53E-67	0.024815	9.4600787	0.0458224	66.596486
Arhgap9	7.40E-61	1.03E-09	0.024771	60.130919	0.005657	8.9881694
Pyroxd1	2.72E-02	6.71E-62	0.024771	1.5655573	0.0614997	61.17358
Gm16322	4.24E-99	3E-184	0.024739	98.372658	0.0441699	183.52052
Gm26510	1.41E-48	0.313639	0.024711	47.850265	0.0037837	0.5035699
Zfp317	1.07E-02	7.2E-50	0.024691	1.9714681	0.0537807	49.142902
Trim5	3.80E-13	1	0.024643	12.420051	0.0046014	0
Ccdc88b	5.06E-54	2.44E-21	0.024541	53.295825	0.0087104	20.611754
Treml4	8.31E-72	7.56E-10	0.024536	71.080619	0.0038871	9.1213851
Asb6	3.27E-14	2.73E-77	0.024531	13.485776	0.0425766	76.563553
Nop10	5.30E-18	8.27E-95	0.024517	17.276132	0.044789	94.082628
Apba1	1.37E-34	0.000607	0.024374	33.864318	0.0049282	3.2168474
Gltp	4.33E-83	2.61E-43	0.024365	82.363012	0.0116369	42.582805
Prr5l	2.85E-34	2.74E-12	0.024329	33.545222	0.0086808	11.562794
Fmnl1	2.59E-44	6.5E-06	0.024285	43.585909	0.0052907	5.1870459
Tmed4	8.23E-10	9.58E-68	0.02428	9.0846202	0.0474494	67.018483
Zfp955b	1.13E-04	8.1E-101	0.024233	3.9452522	0.0717595	100.09074
Pkib	9.87E-40	4.5E-12	0.024225	39.005676	0.0083848	11.346624
Rims3	6.80E-72	4.61E-13	0.024224	71.167584	0.0052291	12.33597
Kif5a	2.95E-03	3.81E-47	0.024184	2.5301308	0.0461822	46.418932
Slc28a2	1.16E-56	1.61E-16	0.024144	55.934375	0.0073133	15.792987
Mrpl13	8.89E-05	6.97E-50	0.024084	4.0509289	0.0483298	49.156471
Unc5b	8.94E-19	2.8E-121	0.024077	18.048734	0.0578903	120.54695
Stap1	2.35E-20	0.018392	0.024054	19.628469	0.0058962	1.7353644
E030042O2	3.20E-50	1.1E-180	0.023969	49.495333	0.0503439	179.97067
Rassf2	8.95E-47	5.69E-07	0.023931	46.047975	0.0057821	6.2447611
Fxyd5	1.62E-45	0.000638	0.023891	44.790007	0.0045519	3.1951128
Loxl3	4.13E-60	2.54E-12	0.023734	59.383821	0.0058904	11.594385
Ly9	1.13E-68	4.28E-11	0.023701	67.945419	0.0048226	10.368666
Slfn1	2.12E-54	7.06E-09	0.023592	53.674358	0.0044755	8.150978
Mmp8	8.55E-74	9.14E-24	0.023537	73.068142	0.0068266	23.039109
Llph	2.02E-05	6.66E-52	0.023502	4.6954554	0.0458511	51.176335
Tmx2	8.15E-12	2.19E-78	0.023454	11.088593	0.0449555	77.659281
Pdzd11	1.68E-12	6.2E-100	0.023445	11.775382	0.0512397	99.205178

Arap3	3.53E-17	1	0.023411	16.452537	0.0025895	0
Ints10	4.59E-08	2.69E-75	0.023409	7.3378777	0.0484947	74.570986
Gnat3	6.82E-75	1.52E-17	0.023322	74.166402	0.0055563	16.816802
Eef1aknmt	6.46E-13	1.12E-74	0.023319	12.189627	0.0442422	73.949966
S100a9	1.15E-59	9.52E-25	0.023272	58.938595	0.009023	24.021309
Ap2m1	4.53E-04	1.72E-69	0.023271	3.3436425	0.0560071	68.76364
Cd37	1.30E-47	8.48E-08	0.023235	46.885572	0.0054917	7.071487
Rpgrip1l	1.28E-03	1.9E-58	0.023002	2.8938669	0.0544907	57.720124
Naip6	2.78E-59	1.57E-05	0.022923	58.555459	0.0040668	4.8042738
AI314278	1.68E-82	4.1E-223	0.022916	81.775122	0.0533783	222.38808
Polm	5.86E-04	1.55E-50	0.022896	3.2318024	0.0484763	49.810148
Trerf1	2.28E-19	0.017734	0.022849	18.642501	0.0059464	1.751193
Rbfa	4.99E-07	2.48E-86	0.022831	6.3015883	0.0545437	85.605917
Vps28	1.72E-14	2.41E-77	0.022823	13.765159	0.0403527	76.618852
Ezr	2.93E-26	0.008154	0.022797	25.532642	0.0051764	2.0886069
Trappc6a	8.65E-13	1.35E-79	0.022776	12.063173	0.0437679	78.869856
Sectm1a	1.60E-73	3.4E-172	0.022766	72.794915	0.0427105	171.46596
Nlrp3	5.71E-60	6.74E-13	0.022689	59.243301	0.0050735	12.171403
Oas3	1.89E-62	0.039184	0.022642	61.723245	0.0022746	1.4068894
C5ar2	2.24E-65	4.07E-20	0.02261	64.650299	0.0072015	19.390928
Pik3r6	3.70E-40	1.07E-06	0.022607	39.43217	0.0048229	5.9717463
Gabrb3	4.67E-13	5.79E-84	0.022602	12.330505	0.0451741	83.237638
Hbb-bs	1.95E-11	1	0.022582	10.709696	0.0034979	0
2700062C0	1.58E-08	1.42E-61	0.022578	7.8023015	0.0418069	60.848933
Gtf3a	8.33E-30	2.03E-09	0.022551	29.079557	0.0092001	8.693031
Itgax	3.43E-73	3.08E-07	0.022463	72.464721	0.0031397	6.5113928
Clec9a	8.90E-42	6.12E-15	0.02246	41.05085	0.0081997	14.213587
Nr2c2ap	2.95E-02	3.32E-47	0.022444	1.5299419	0.0485356	46.478708
Cxcl2	3.26E-53	9.17E-07	0.022415	52.486133	0.0033885	6.0376465
Wars	2.23E-04	2.58E-70	0.022411	3.6514632	0.0528519	69.588978
Rps6ka2	2.39E-26	0.001609	0.022355	25.620987	0.0051373	2.7934483
Naip5	5.44E-40	4.93E-08	0.022318	39.264284	0.0064442	7.3072447
C5ar1	6.38E-70	5.59E-14	0.022207	69.195416	0.0044773	13.252744
Ap5b1	9.97E-60	5E-161	0.022196	59.00145	0.041458	160.29855
Slc30a1	2.34E-07	1	0.022118	6.6302371	0.0026733	0
Morc4	1.18E-28	1.3E-145	0.022098	27.927782	0.051229	144.87116
Rsad2	4.63E-03	1	0.022098	2.3340865	-0.0076056	0
Lrrc59	1.36E-03	2.68E-48	0.021989	2.8672454	0.0450401	47.57111
Meig1	1.31E-37	0.025751	0.02197	36.882451	0.0045862	1.5892082
Shisa5	1.67E-19	0.214583	0.021911	18.77656	0.0050895	0.6684051
Fbxw17	1.23E-54	6.92E-25	0.02191	53.90887	0.0096187	24.160168
Gpn3	3.05E-05	1.04E-51	0.021889	4.5158369	0.0420647	50.98388
Galk1	1.28E-05	3.49E-90	0.021885	4.8914301	0.0563343	89.457476
Acot6	5.29E-50	4.7E-138	0.021867	49.276776	0.0379445	137.32723
Sp110	9.39E-32	0.450772	0.021864	31.027394	0.0034306	0.3460429
Lrrc27	1.65E-42	0.002166	0.021848	41.78219	0.003952	2.6644133
Tmem236	5.76E-62	1.2E-30	0.021845	61.239583	0.010999	29.920013

Irak3	1.65E-32	3.69E-05	0.02179	31.783156	0.0059581	4.4335193
Zfp335	9.89E-04	3.14E-47	0.021726	3.0049177	0.0454758	46.5029
Kif5c	4.75E-75	9.7E-157	0.021668	74.323093	0.0408198	156.01279
Renbp	4.26E-10	1	0.021636	9.3702786	-0.0002249	0
Arhgdib	1.10E-38	7.66E-09	0.021517	37.958929	0.0062512	8.1160221
Kif24	8.90E-03	8.6E-124	0.021473	2.0508056	0.0854199	123.0666
Wdr18	9.74E-04	2.84E-61	0.021436	3.0114919	0.0506666	60.546318
Tmem258	5.26E-14	5.82E-96	0.021392	13.279405	0.0451237	95.235414
Gm20663	1.54E-02	1	0.02134	1.8128438	-0.0043075	0
Spic	8.40E-60	8.57E-14	0.021295	59.075818	0.005255	13.066894
Ndufb11	5.42E-07	1.19E-68	0.021215	6.2661202	0.0448198	67.925436
Ifi211	2.74E-40	0.065205	0.021202	39.561682	0.0026356	1.18572
Mpg	8.46E-07	5.94E-68	0.02107	6.0726904	0.0461913	67.225877
Sult2a5	2.53E-78	0	0.021056	77.597193	0.0849076	#NUM!
Adra2a	1.97E-87	4.6E-201	0.020979	86.704885	0.0464496	200.33934
Cd68	1.52E-23	1	0.020915	22.818954	0.0013713	0
Use1	2.18E-03	2.9E-78	0.0209	2.6606416	0.0559629	77.537371
Chaf1b	5.52E-30	4.2E-137	0.020707	29.258113	0.0443752	136.37999
Cxcr5	5.21E-27	0.001313	0.020648	26.282776	0.0060903	2.8817162
Pira2	6.65E-54	3.84E-06	0.020629	53.177462	0.0038845	5.4158441
Cd83	1.30E-57	2.18E-10	0.020538	56.887521	0.0049187	9.6613751
Nxpe4	2.34E-12	1	0.020458	11.631219	1.603E-05	0
Kif21b	2.00E-39	2.75E-08	0.020431	38.698864	0.0057864	7.5600821
Uba7	5.67E-11	1	0.020315	10.246147	0.004054	0
Ndufaf7	1.42E-10	1.4E-66	0.020255	9.8484264	0.0387643	65.852879
Pld4	2.65E-33	3.47E-09	0.020194	32.576837	0.0071901	8.4594914
Gm36231	1.56E-11	9.7E-143	0.020187	10.807055	0.0603274	142.01491
Endov	1.02E-03	3.75E-81	0.02015	2.9920351	0.0550599	80.425408
Ttll7	2.66E-37	1.6E-126	0.020149	36.575641	0.0394061	125.80416
44447	5.25E-38	0.031439	0.020092	37.279939	0.003588	1.5025323
Nlrc4	8.74E-38	8.06E-09	0.019931	37.058386	0.0065046	8.0935101
1700095A2	3.99E-07	1.4E-92	0.019911	6.3992464	0.0535237	91.85495
Agpat4	6.96E-22	1	0.019897	21.157104	0.0011864	0
Slc35a4	4.93E-05	9.85E-67	0.019884	4.3067279	0.0474612	66.006454
Mefv	1.55E-58	2.9E-15	0.019824	57.810126	0.004879	14.537811
Nat8f3	7.27E-56	8.87E-08	0.019756	55.13829	0.0049148	7.0519792
Rftn2	1.86E-36	1.41E-09	0.019687	35.730477	0.007337	8.8523149
Gngt2	9.20E-49	2.06E-17	0.01968	48.036396	0.0077347	16.685136
Hvcn1	3.29E-43	1.36E-06	0.019583	42.483341	0.00439	5.8672288
Plxdc2	1.97E-10	1	0.019569	9.7065529	0.0016344	0
Ciita	1.18E-19	0.131055	0.019558	18.928368	0.0043059	0.8825462
Gcnt1	6.82E-45	7.61E-05	0.019515	44.166314	0.0033703	4.1187789
Gm2814	2.66E-07	3.23E-69	0.019515	6.5757374	0.041783	68.49095
Ifit2	4.38E-21	1	0.019458	20.358266	0.0011211	0
Spp1	7.18E-42	7.09E-16	0.01945	41.144117	0.0075637	15.149657
Cep89	3.09E-02	2.08E-45	0.019278	1.5104619	0.0452311	44.681017
Pilra	6.13E-45	2.91E-10	0.019187	44.21245	0.0053818	9.5361019

Gm2245	1.38E-56	2.96E-09	0.019082	55.858908	0.0037075	8.5286152
Cd80	3.88E-43	2.1E-12	0.019047	42.410887	0.0061929	11.678764
Trim35	3.98E-37	5.88E-07	0.019042	36.399901	0.0047828	6.2303899
Sec61b	1.07E-03	6.9E-62	0.019039	2.9700369	0.0454015	61.161204
Sell	1.68E-38	3.96E-07	0.019037	37.775117	0.0050607	6.4026217
Plaur	1.75E-48	0.000303	0.019019	47.756183	0.0031782	3.519097
6820408C1	1.17E-73	2.7E-168	0.019001	72.932904	0.0393175	167.56446
Cxcl16	2.39E-46	8.65E-09	0.018996	45.622137	0.0047015	8.0627581
Spout1	9.17E-09	2.87E-80	0.018983	8.0374486	0.0428416	79.541544
Fermt1	1.09E-36	3.3E-194	0.018794	35.961609	0.0540963	193.48729
Nsun5	1.02E-07	1.11E-71	0.018793	6.9901738	0.0421056	70.955569
Siglec1	5.12E-39	1.61E-11	0.018756	38.29076	0.0056497	10.791962
Hdac10	4.60E-05	3.38E-53	0.018744	4.3376826	0.0389541	52.470884
Aph1c	5.90E-19	1	0.018728	18.229013	0.0008939	0
Cox18	7.77E-05	3.28E-70	0.018701	4.1093551	0.0479459	69.484332
6430548M	1.39E-07	1	0.018679	6.8580689	-0.0013583	0
Eid1	1.34E-46	2.5E-145	0.018672	45.873373	0.0371976	144.5966
Kcnk6	8.35E-46	2.38E-14	0.018583	45.078367	0.0062293	13.623781
Dhrs1	2.16E-02	1.62E-53	0.01851	1.6656077	0.0450623	52.790266
Ifi213	2.12E-17	1	0.018488	16.673206	0.0010961	0
Olfr56	1.67E-09	1	0.018241	8.7761738	-0.000324	0
Gpr35	4.40E-03	3E-62	0.018195	2.357039	0.0451158	61.522663
Akna	9.32E-37	0.00162	0.018103	36.030439	0.0037729	2.7903513
Gpn1	1.14E-02	2.31E-43	0.018035	1.9431959	0.0393685	42.637028
Satb1	6.68E-20	0.05409	0.017962	19.175042	0.0047461	1.2668812
Hist1h2be	1.96E-04	1	0.017887	3.7070062	-0.0013571	0
Spata6	6.67E-26	1.05E-06	0.017836	25.175617	0.0052252	5.980603
Nfkbid	3.48E-51	1.29E-07	0.017802	50.458621	0.0033116	6.8907603
Aldh3b3	1.36E-70	3.8E-136	0.017747	69.867311	0.0309721	135.4246
Hdac7	6.94E-14	1	0.017687	13.158745	0.0043836	0
Cd244a	7.96E-49	1.35E-09	0.017674	48.098927	0.003948	8.8694894
Fhad1	3.30E-11	1	0.017662	10.481037	0.004301	0
Elac2	4.29E-04	6.08E-71	0.017568	3.3674061	0.0488479	70.216334
Cnn2	7.32E-33	1.05E-09	0.017534	32.13553	0.0062633	8.9774202
Tlr1	1.82E-55	7.36E-14	0.017519	54.739756	0.00485	13.13301
Gm38604	2.90E-07	1	0.017472	6.5378672	0.0014472	0
Prpf38a	7.30E-03	1.33E-51	0.017364	2.1368927	0.0410508	50.874769
Gm43661	3.98E-44	3.82E-10	0.017322	43.400478	0.0040923	9.4174541
Ppp1r18	1.25E-31	2.29E-05	0.017286	30.90187	0.0045369	4.6395236
Bsn	3.36E-20	1	0.017259	19.473826	0.0026883	0
Tlr8	1.04E-46	3.53E-10	0.017207	45.981721	0.0046393	9.4526772
Bin2	9.45E-15	1	0.01717	14.024738	0.0025407	0
Cd247	1.79E-04	2.2E-101	0.017134	3.7471816	0.0508414	100.65996
Ms4a4c	7.89E-35	0.223155	0.017125	34.103198	0.002172	0.6513934
Clec12a	6.12E-40	5.26E-10	0.017119	39.212956	0.0051626	9.2790883
Sars2	2.91E-03	4.32E-45	0.016917	2.5353871	0.0385176	44.364968
Mvk	1.22E-09	7.3E-240	0.016911	8.9138951	0.0861118	239.13646

Rasl11b	1.23E-20	1.56E-97	0.016895	19.910307	0.0347333	96.805637
Lyzl4	1.13E-37	0.055908	0.016827	36.948768	0.0025291	1.2525256
Eln	8.87E-29	3.7E-154	0.016795	28.052301	0.0466922	153.43729
Pole	3.42E-08	2.97E-62	0.016736	7.4665416	0.0338862	61.526614
2700046G(	2.16E-25	1.2E-105	0.016655	24.664752	0.0335007	104.90713
Mki67	1.29E-30	2.3E-136	0.016621	29.889411	0.0513283	135.63286
Rap2b	1.05E-35	8.68E-08	0.01659	34.979885	0.0047387	7.0613151
Nfatc2	2.20E-41	2.01E-09	0.016456	40.656866	0.0040314	8.6978134
Hcls1	1.40E-05	1	0.016443	4.8531477	-0.0013088	0
Bcl2a1b	3.35E-48	1.25E-18	0.016406	47.474507	0.0063898	17.904252
Emp1	6.58E-48	2.31E-10	0.016345	47.181631	0.0037189	9.6365706
Surf2	4.08E-04	7.71E-62	0.016338	3.3892284	0.0390468	61.112737
Pstpip1	2.24E-48	1.36E-10	0.016267	47.649278	0.0040835	9.865721
Gm40645	4.86E-44	9.55E-05	0.016258	43.31344	0.003026	4.0199028
Ints6l	3.53E-04	1	0.016257	3.4518914	-0.0005324	0
Fastkd2	2.80E-02	1.59E-54	0.016241	1.5529699	0.0441906	53.799937
Ifi27l2b	2.09E-41	4.3E-138	0.016218	40.679334	0.0361592	137.36244
Fabp5	1.31E-24	0.017103	0.016214	23.88196	0.0035707	1.7669344
Tmem156	2.84E-41	1.17E-05	0.016173	40.54655	0.0031717	4.9324294
Pdgfb	4.86E-48	1.88E-05	0.016155	47.313427	0.0027503	4.7258644
Ighm	8.41E-23	5.93E-05	0.016149	22.075208	0.0050211	4.2268964
Zwilch	1.98E-12	1.69E-76	0.01614	11.704204	0.0340251	75.771709
Pycard	9.53E-08	1	0.016122	7.0207269	0.0012652	0
Ppdpf	1.33E-03	5.04E-48	0.015998	2.8772373	0.0338718	47.29796
Casp1	1.26E-38	1.42E-10	0.01599	37.900795	0.0052787	9.846699
Zfp820	5.52E-07	2.7E-127	0.015974	6.2584427	0.0541198	126.56981
A930014D(	4.71E-46	8.61E-08	0.015961	45.326593	0.003489	7.0650905
Gale	1.96E-09	2.1E-225	0.015894	8.7081966	0.0751058	224.67406
Gm26770	2.92E-19	4.28E-93	0.015875	18.534561	0.0327224	92.368794
Gm39556	6.30E-37	3.59E-12	0.015873	36.200371	0.0054397	11.444482
Isyna1	1.19E-19	2.72E-88	0.015833	18.922937	0.0302904	87.566055
Il1a	1.10E-27	0.000142	0.0158	26.960267	0.0042736	3.8488524
Disc1	1.45E-34	1.79E-05	0.015786	33.837794	0.003623	4.7462839
Havcr2	3.19E-44	4.69E-11	0.015673	43.495718	0.0040723	10.329041
1600022D1	6.04E-56	7.22E-23	0.015555	55.219193	0.0070438	22.141262
Tnfrsf21	3.01E-21	1	0.015545	20.521736	0.0014367	0
Msantd3	4.81E-61	3.6E-136	0.015545	60.31749	0.0309567	135.44456
Raet1e	2.03E-32	0.00312	0.015528	31.693387	0.0035715	2.5058378
Ndufaf5	3.59E-04	1.4E-46	0.015323	3.445475	0.0329086	45.85518
Tsr3	2.68E-03	2.33E-73	0.015152	2.571087	0.0426736	72.632975
Kcnab2	2.43E-46	7.45E-12	0.015137	45.613652	0.0044252	11.127792
Cfap44	1.39E-57	4.5E-201	0.015104	56.857593	0.0485057	200.34992
Tpx2	1.66E-13	3E-91	0.015094	12.778971	0.0364584	90.52323
Gm19325	1.84E-40	1.22E-07	0.015042	39.736247	0.0039621	6.9141517
Thbs1	1.90E-34	8.82E-06	0.014977	33.721128	0.0027977	5.054538
Fam171a1	2.20E-10	2.42E-85	0.014912	9.6572966	0.0330811	84.616122
Plau	3.71E-44	0.001332	0.014864	43.430499	0.0019245	2.8753999

Lrrk2	2.51E-07	1	0.014821	6.601011	0.0019268	0
Gm26693	9.26E-09	1	0.014737	8.0332139	0.0023086	0
Naxe	1.77E-04	2.5E-54	0.014699	3.7514569	0.0333887	53.601965
Gm45469	1.35E-03	1	0.014688	2.8712251	-0.0038265	0
Clec4e	8.75E-39	1	0.014671	38.058122	0.0012053	0
Ly6a	2.32E-40	6.61E-11	0.014643	39.634948	0.0046534	10.179878
Traf3ip3	1.04E-13	1	0.014567	12.983654	0.0005028	0
Gm45510	1.48E-03	1	0.014539	2.8294008	-0.0060569	0
S100a8	6.97E-38	9.62E-13	0.014471	37.156892	0.0054936	12.016813
A230028O(	4.08E-43	0.000449	0.014459	42.389765	0.0023854	3.347664
Arg2	6.11E-23	1	0.014416	22.214005	0.0018663	0
Dubr	4.23E-38	0.010325	0.014411	37.373237	0.0025319	1.9861293
Mcoln2	1.88E-40	6.96E-06	0.014322	39.726649	0.0031426	5.1573896
Clec4a3	1.11E-20	0.160461	0.014274	19.954216	0.0034094	0.7946297
Sytl2	6.63E-37	1.4E-166	0.014247	36.17865	0.0460238	165.83901
Hba-a1	5.11E-04	1	0.014224	3.2913742	-0.0005073	0
AI662270	1.98E-39	2.76E-08	0.014108	38.702318	0.0039113	7.5591366
Gm35154	1.40E-32	3.99E-07	0.01399	31.853983	0.0038829	6.3986038
H2-DMb1	1.17E-28	2.16E-06	0.013965	27.930871	0.0041914	5.6653074
Sirpb1c	1.51E-42	1.7E-14	0.013951	41.821966	0.004517	13.768832
Gm10974	1.77E-21	0.000242	0.013948	20.751665	0.0048919	3.6156207
Ankrd55	3.08E-25	4.36E-05	0.013901	24.511567	0.0040805	4.3609828
Slc44a4	3.86E-55	1.65E-21	0.013852	54.413641	0.0058184	20.781955
Ighd	1.50E-16	0.30277	0.013748	15.82489	0.0030602	0.5188872
Ap1s2	3.04E-24	7.39E-05	0.013744	23.516522	0.0043278	4.1313693
Sat2	7.57E-06	1.69E-62	0.013688	5.1209324	0.033944	61.771286
Oas1a	1.25E-29	1	0.013678	28.90299	0.0016977	0
Cracr2a	2.08E-27	2.48E-06	0.013623	26.680994	0.0045633	5.6055919
Cnrip1	2.48E-20	0.056981	0.013622	19.604955	0.0029052	1.2442725
Prdm1	9.00E-38	8.52E-11	0.013551	37.045669	0.0048274	10.069602
Trpv1	2.12E-23	2.4E-126	0.013439	22.674113	0.0360097	125.61963
Itpripl2	1.14E-25	0.549895	0.013391	24.941965	0.0028807	0.2597206
Col1a1	2.95E-39	0.000166	0.013364	38.530034	0.0020367	3.7799402
Surf1	2.94E-06	1.22E-55	0.013347	5.5322855	0.0292997	54.915191
Orai2	6.99E-20	0.024627	0.013181	19.15527	0.0033326	1.6085937
Urod	2.12E-03	2.66E-59	0.01316	2.6730831	0.0350164	58.574929
Col3a1	1.88E-10	1	0.013106	9.7267013	0.0003765	0
AC142100.	4.50E-26	0.002406	0.013029	25.347247	0.0026166	2.6187517
Tifab	8.92E-41	2.49E-06	0.013016	40.049758	0.0031724	5.6033395
Pde8b	2.26E-12	1	0.01291	11.645561	0.0028078	0
Cyp4f18	1.09E-29	7.98E-08	0.012797	28.960721	0.0037663	7.097993
Ifi206	1.60E-19	1	0.012793	18.795659	0.0010121	0
Insyn2b	1.92E-24	0.65597	0.012785	23.717058	0.0023213	0.1831162
Fos	1.06E-24	4.41E-05	0.012696	23.97515	0.0041162	4.3558415
Ms4a4b	3.47E-19	0.073864	0.012564	18.459236	0.0036495	1.1315657
Nlrc3	2.85E-13	1	0.012534	12.544483	0.0022606	0
Il1rl2	1.62E-09	1	0.01246	8.7910552	6.611E-05	0

Smim1	5.99E-06	9.32E-81	0.01241	5.2222558	0.0354327	80.030456
Cd33	1.10E-33	0.121947	0.012346	32.958643	0.0018489	0.9138303
Lrrc25	4.78E-34	4.59E-08	0.012344	33.320849	0.0039797	7.337749
Stx11	1.75E-31	0.488864	0.012323	30.757617	0.0015682	0.3108117
Anxa1	1.85E-10	1	0.012274	9.7317678	-0.0001665	0
Hba-a2	4.30E-02	1	0.012238	1.3664723	-0.0048313	0
Dnlz	2.27E-08	1.08E-59	0.012203	7.6447668	0.0266134	58.967121
Cd19	1.76E-19	1	0.012159	18.755574	0.001398	0
Rgs10	5.80E-36	1.01E-06	0.012037	35.236825	0.0029737	5.9955006
Trim30c	9.81E-12	1	0.011994	11.008459	0.0011142	0
Tex9	3.82E-05	1.6E-50	0.011986	4.4175031	0.0267017	49.796513
Lilra6	1.00E-34	0.107023	0.011963	33.999854	0.0014275	0.9705235
Gm48767	9.98E-11	8.49E-71	0.011924	10.000764	0.0268883	70.071081
Gm5086	3.13E-24	1	0.011921	23.50414	0.0008655	0
Gm2164	3.10E-24	1	0.011832	23.508109	0.0007155	0
Gm36640	1.45E-38	1.5E-08	0.011804	37.838474	0.0034488	7.8248785
Zfp641	2.95E-15	2E-105	0.011796	14.53054	0.0326143	104.7066
Grb10	2.38E-06	9E-147	0.011762	5.6239315	0.0510553	146.04439
Gm4131	3.31E-08	1	0.011638	7.4806881	-0.0001027	0
Zeb2os	1.59E-33	0.000248	0.011603	32.797853	0.0023303	3.6061644
8030453O2	4.23E-30	0.12281	0.011579	29.373605	0.0022987	0.9107673
Bhlhe41	4.74E-25	0.000219	0.011562	24.324244	0.0036676	3.6594375
Gm10734	3.40E-03	2.82E-60	0.01155	2.4681717	0.0329336	59.549421
Gm33103	1.11E-33	1.31E-05	0.011546	32.953595	0.0025303	4.8841337
Slirp	9.90E-03	1.4E-47	0.011464	2.0045609	0.0283403	46.855087
Lpcat1	1.38E-21	0.000929	0.011461	20.858882	0.0033705	3.0319459
Pygm	4.58E-21	1	0.011434	20.33891	0.0024121	0
Ak8	2.73E-20	1	0.011427	19.564549	0.0007272	0
Tnfrsf26	9.95E-18	0.766407	0.011369	17.00236	0.0022563	0.1155406
Ptpro	1.45E-27	0.017955	0.011357	26.837636	0.0025002	1.7458186
Metrnl	1.81E-06	1	0.01132	5.7422243	0.0007627	0
Gsn	1.45E-24	0.056167	0.011256	23.838664	0.0023903	1.2505157
Fkbp11	2.74E-05	1.17E-96	0.01123	4.5628451	0.0392661	95.932625
Dgkg	1.12E-08	1	0.011217	7.9517464	0.0003406	0
Crip1	1.36E-15	0.965295	0.011153	14.865505	0.0028742	0.0153398
Il3ra	3.36E-12	1	0.011015	11.47316	0.0014014	0
Nlrp1a	6.46E-29	1	0.011004	28.189502	0.0012805	0
Hoxb4	1.75E-13	1	0.010998	12.757742	0.00141	0
Ccl4	5.25E-37	8.96E-12	0.010903	36.279431	0.003477	11.04761
Gm13212	2.79E-26	4.5E-05	0.010889	25.554954	0.0031209	4.3465984
Sh3tc1	1.13E-07	1	0.010873	6.9463893	0.0017377	0
Trim6	4.09E-28	3.17E-95	0.010858	27.388058	0.0246911	94.499262
Cd200r4	2.55E-32	5.74E-05	0.010781	31.594264	0.0028006	4.2407443
Ntn1	3.08E-05	1	0.010774	4.5116921	4.695E-05	0
Ptger2	4.07E-23	0.001041	0.010709	22.390411	0.0030326	2.9826567
Lad1	6.69E-07	1	0.010704	6.1748198	0.0003984	0
Was	1.18E-29	0.000214	0.010659	28.929116	0.0025001	3.670594

Gm49965	3.94E-17	0.180004	0.010643	16.404398	0.0024034	0.7447188
Rasal3	2.97E-23	0.008821	0.010627	22.527716	0.0025129	2.0544583
Olfr111	4.16E-34	0.025088	0.010623	33.3805	0.0014571	1.6005262
Gm45606	2.50E-35	1.57E-07	0.01062	34.602126	0.0028534	6.8042797
Fabp7	8.63E-28	4.43E-05	0.010612	27.064219	0.0027055	4.3535172
Olfr267	1.60E-02	5.65E-41	0.010599	1.7966147	0.0226299	40.247781
Hpse	3.98E-20	0.230148	0.010575	19.399984	0.0020066	0.6379923
Lingo4	3.40E-05	1	0.010515	4.4685003	-0.000295	0
Saa1	8.96E-03	0.822909	0.010379	2.0474729	-0.0034885	0.0846484
Cd40	1.31E-17	1	0.010374	16.881112	0.0014353	0
Akr1b7	4.89E-36	3.9E-161	0.010367	35.310958	0.038881	160.40788
Fam78b	1.09E-10	1	0.010337	9.9619197	0.0006312	0
Arl4c	5.41E-27	0.048464	0.010303	26.266592	0.0021311	1.3145812
Ccr2	2.91E-24	0.000351	0.010282	23.535958	0.0024279	3.4544201
Zfp831	5.45E-13	1	0.010212	12.263271	0.0019151	0
Slamf8	5.24E-32	1	0.010203	31.280765	0.0005268	0
Cdh23	2.89E-14	1	0.010175	13.53978	0.0013472	0
Ankdd1a	1.89E-33	1	0.010125	32.72293	0.0003712	0
Slc25a43	6.09E-27	0.697249	0.010112	26.215385	0.0018306	0.1566119
Procr	1.13E-31	7.01E-07	0.010108	30.946877	0.0028308	6.1542073
Mical1	4.18E-21	1	0.01007	20.378878	0.0017789	0
Cnr2	2.59E-18	0.123778	0.01001	17.586193	0.0024145	0.907355
Slc16a3	2.88E-24	1	0.009976	23.540213	0.0011009	0
Cxcr4	1.00E-29	0.000352	0.009888	28.999539	0.0022688	3.4539699
Col1a2	9.85E-13	1	0.009828	12.006655	0.0009944	0
5031425F1	1.99E-29	0.00452	0.009801	28.702211	0.0016728	2.3448151
Sash3	4.13E-25	0.552206	0.009797	24.384107	0.0017507	0.2578988
Gm13986	2.53E-06	1	0.009782	5.5969256	-0.0001421	0
4930430E1	4.60E-36	0.000509	0.009743	35.337228	0.0016134	3.2929942
Pdlim4	2.30E-30	0.00034	0.009708	29.637856	0.0018742	3.468559
Rab3il1	3.72E-11	1	0.009693	10.429656	-7.691E-05	0
Gm14548	1.46E-17	1	0.009673	16.835188	0.0015267	0
9630014M	3.10E-10	4.29E-60	0.00962	9.5080976	0.0209207	59.367232
Scimp	4.64E-26	2.54E-05	0.009551	25.333117	0.0030614	4.5949658
Gm49101	2.68E-31	3.17E-07	0.009485	30.571472	0.0028202	6.4989246
Tmem229b	2.86E-22	0.004214	0.009468	21.543856	0.0024139	2.3752901
Gm12708	3.08E-33	1.42E-05	0.009458	32.511	0.0021549	4.8474374
Dusp2	5.12E-26	0.004325	0.009401	25.291035	0.0022815	2.3640412
Syn1	2.46E-16	1	0.009303	15.609098	0.0010976	0
Alox5ap	5.48E-20	1	0.009197	19.261314	0.0008925	0
Mapk12	3.46E-06	1	0.009184	5.4605661	0.0007149	0
Rgs19	5.06E-26	0.000214	0.009171	25.296213	0.0023874	3.6686932
Batf3	1.04E-18	1	0.009076	17.98389	0.0019024	0
Ckb	1.54E-12	1	0.00907	11.811349	0.0012438	0
AC160336.	1.33E-03	1.01E-59	0.008967	2.8768939	0.025561	58.995227
Mep1a	3.08E-33	1.6E-131	0.008778	32.511	0.0326984	130.79354
Lockd	6.80E-11	1.52E-60	0.008727	10.167604	0.0205595	59.817788

Cgas	5.71E-09	1	0.008715	8.2430837	0.0012481	0
Gdpd5	1.63E-07	1	0.008678	6.7867627	0.0005334	0
Vinac1	2.37E-10	1	0.008672	9.6250708	0.0013881	0
Rab37	5.00E-14	1	0.008615	13.300704	0.0006034	0
Smoc2	3.63E-13	6.44E-63	0.008495	12.439775	0.018791	62.191147
Faiml	2.07E-30	0.007477	0.00844	29.684591	0.0012655	2.1262725
Nos1	1.74E-12	1	0.008375	11.758266	0.0005662	0
Emp3	1.87E-22	0.147578	0.008339	21.728433	0.0017096	0.8309783
Gspt2	2.29E-24	3.3E-125	0.008262	23.640466	0.0303024	124.4775
Riiad1	1.06E-03	1	0.008217	2.97486	-0.00184	0
Gm32364	1.77E-19	0.056527	0.008181	18.751402	0.0019526	1.2477434
Alox5	1.93E-03	1	0.008174	2.71359	1.744E-05	0
Il2rg	1.73E-13	1	0.008122	12.762025	0.0007229	0
Gm10521	2.52E-24	1	0.008121	23.598048	0.0009956	0
Gm41556	6.13E-18	0.683161	0.008036	17.212714	0.0019713	0.1654767
Gm16096	8.14E-24	0.000509	0.007994	23.089262	0.0015158	3.2929942
Timd4	1.97E-03	1	0.007977	2.7045305	-0.000122	0
Gm2415	2.30E-07	3.4E-69	0.007774	6.6389591	0.022679	68.468785
Chek1	1.19E-02	4.2E-48	0.007757	1.9226573	0.0222196	47.37666
Gm42962	6.78E-19	1	0.007691	18.168804	0.0012911	0
Saa2	5.01E-06	1	0.007494	5.3000753	-0.0004038	0
Zfp248	4.65E-03	1.67E-52	0.007397	2.3324007	0.0220692	51.777836
Gm21860	1.30E-08	1	0.007336	7.8862386	0.0003351	0
Galnt12	4.73E-15	1	0.007332	14.324707	0.0012827	0
Runx3	7.22E-21	0.016694	0.007327	20.141365	0.001723	1.7774522
4930455G(	1.28E-26	0.13732	0.007317	25.891638	0.0012138	0.8622667
Chil1	1.15E-12	1	0.007278	11.937898	0.000698	0
Gm11755	1.09E-20	8.98E-92	0.007189	19.961857	0.0236124	91.046848
Akr1c18	1.97E-15	1	0.007031	14.704882	0.0010176	0
Bst1	1.07E-13	1	0.00698	12.969234	0.0004222	0
Mamld1	1.34E-04	1	0.00697	3.8718328	-0.0002594	0
Syngr1	7.75E-20	1	0.006912	19.110765	0.0004306	0
Tnf	7.13E-23	0.018354	0.006895	22.146932	0.0012309	1.736273
Gm36161	2.97E-21	1	0.006831	20.527398	0.000538	0
Xlr4a	1.21E-26	1	0.006791	25.91622	0.0007162	0
Mak	1.20E-14	1	0.006759	13.922343	0.0010769	0
Slc2a6	2.06E-14	1	0.006748	13.685698	0.0007672	0
Hbb-bt	5.98E-05	1	0.006686	4.2234241	-0.0002855	0
Mcub	5.37E-11	1	0.006605	10.270313	0.0006569	0
Kcnj6	9.43E-07	1	0.006592	6.0253044	0.0004842	0
Nrg3	2.77E-03	1	0.006526	2.5574156	-0.0001121	0
Gm37229	1.23E-05	1	0.00651	4.9092056	0.0001622	0
Hcar2	3.68E-18	0.6721	0.006483	17.434192	0.0008484	0.1725661
Adam33	2.73E-06	1	0.006424	5.5641952	0.0002869	0
Siglecf	8.14E-24	0.000509	0.006418	23.089262	0.0015646	3.2929942
Mtmr11	7.35E-03	1.72E-57	0.006396	2.1337892	0.0216325	56.765474
Trem3	4.96E-11	1	0.006381	10.304475	0.0003862	0

Trim29	5.47E-21	0.018354	0.006334	20.262001	0.0012797	1.736273
Phf11a	6.35E-19	1	0.006266	18.196996	0.0003472	0
4833407H1	3.22E-17	1	0.006184	16.491982	0.0005005	0
Nat8	8.50E-05	1	0.006139	4.0706133	-0.000931	0
Gm44993	2.16E-16	1	0.006107	15.664771	0.0006717	0
Hlx	1.83E-09	1	0.006106	8.7385293	0.0008883	0
St6galnac4	1.00E-07	1	0.006042	6.9980273	0.0006259	0
Cyp4f39	2.31E-03	1	0.00603	2.6356409	-0.0008988	0
Tspan2	3.40E-06	1	0.006009	5.4681189	0.0002201	0
Hmmr	1.87E-07	8.72E-51	0.005988	6.7286854	0.0169255	50.059702
Tspan32	4.30E-09	1	0.005949	8.3667599	0.0006456	0
A530064D0	3.68E-18	0.111054	0.005876	17.434192	0.001064	0.9544654
Ccl2	1.59E-13	1	0.005828	12.799797	0.0005479	0
Gm26520	1.31E-11	1	0.00571	10.881231	0.0006073	0
Cenpf	3.06E-03	2.13E-57	0.005619	2.5145184	0.0236787	56.672094
Gm30198	7.59E-17	0.630621	0.005591	16.119801	0.0008703	0.2002317
Gm10863	1.83E-10	1	0.005572	9.7381238	0.0004929	0
Gm5122	1.48E-06	6.12E-49	0.005486	5.8308439	0.0148864	48.213271
Treml1	4.20E-19	0.6721	0.0054	18.37654	0.0007508	0.1725661
Gm15091	4.59E-03	1	0.005389	2.3379793	-0.0004472	0
Gm49198	1.17E-02	1	0.005155	1.9332441	-0.000675	0
Hmx1	4.80E-20	0.6721	0.005008	19.319151	0.0007508	0.1725661
Slc36a2	2.82E-16	1	0.004789	15.549025	0.0007996	0
Ceacam16	1.59E-13	1	0.004768	12.799723	0.0005268	0
Gm41492	3.74E-03	1.69E-45	0.004723	2.4267716	0.0154287	44.771902
Ticam2	2.17E-14	1	0.004704	13.662639	0.0001668	0
Gm36486	3.22E-17	1	0.004635	16.491982	0.0001322	0
Gm10800	2.18E-02	0.210298	0.004623	1.6624925	-0.0018331	0.6771653
Treml2	4.78E-07	1	0.004547	6.3206692	0.0004003	0
A530032D1	4.59E-10	1	0.004542	9.3377446	0.0001691	0
Ppp1r1c	3.62E-05	1	0.00441	4.4413803	4.602E-05	0
Arl11	2.17E-14	1	0.004143	13.662639	0.0006327	0
Xlr3b	6.37E-10	1	0.00411	9.195791	-0.0001533	0
Gtsf1	8.97E-04	1	0.003943	3.0471896	-0.0002661	0
AI427809	1.91E-13	1	0.003939	12.719451	0.0005493	0
Nectin4	4.90E-09	1	0.003804	8.3096934	0.0002962	0
Ctbp2	1.91E-13	1	0.003701	12.719451	0.0004659	0
Gm34517	6.47E-12	3.79E-60	0.003691	11.189102	0.0141924	59.421686
Clec5a	1.38E-05	1	0.003687	4.8599867	-2.111E-05	0
Trim15	2.17E-14	1	0.003666	13.662639	0.0001668	0
Egr3	1.30E-10	1	0.003597	9.8873951	0.0002991	0
Tns4	3.27E-03	1	0.003511	2.4850301	-0.0003382	0
Lipn	1.68E-12	1	0.003497	11.775654	0.0002503	0
Gm45463	2.13E-07	1	0.003417	6.6708208	0.0002164	0
BC023719	1.47E-11	1	0.003174	10.832195	0	0
Sbk2	6.92E-06	1	0.002222	5.1599816	0	0
Mycbpap	1.14E-03	7.31E-37	0.002133	2.9432323	0.0095427	36.136065

Skap2	3.02E-09	2.5E-243	0.001777	8.5204896	-0.1702105	242.60537
Gm30146	5.44E-04	2.24E-35	0.001426	3.2644557	0.0084885	34.649589
Csf3	4.31E-02	3.5E-29	0.001018	1.3656934	0.0072656	28.45576
Tm6sf2	6.97E-03	1	-0.010017	2.1565249	0.0054244	0
Ptpre	5.68E-05	1.5E-101	-0.010408	4.2459327	-0.0579478	100.83098
Rdh11	6.89E-03	1	-0.012493	2.1619464	0.0024445	0
Dusp23	1.75E-02	1	-0.012696	1.7573218	0.000461	0
Snai2	1.64E-06	1	-0.013174	5.7859609	-0.0041009	0
Nr4a1	8.48E-07	7.28E-55	-0.014315	6.0718449	-0.0255242	54.137945
E230001N(	7.58E-06	0.000161	-0.015821	5.1200506	0.0122671	3.7925644
Grem2	3.01E-05	1.41E-06	-0.016704	4.5213043	0.0748912	5.8513649
1700029I1!	1.02E-10	0.093328	-0.017001	9.9895246	-0.0073979	1.0299867
Ftsj1	4.88E-02	1	-0.018307	1.3116968	0.0123785	0
Ticam1	1.64E-03	1	-0.01837	2.7852763	0.002496	0
Zfp263	2.48E-02	1	-0.018464	1.6058055	0.0120823	0
Calr	1.23E-02	1	-0.018661	1.9113216	0.0195015	0
Eaf2	5.83E-03	1	-0.018894	2.2342728	0.0095747	0
Ring1	1.75E-03	1	-0.0191	2.7568428	-2.847E-06	0
Cd59b	1.77E-04	1	-0.019427	3.7515359	0.0041776	0
Rdh9	4.63E-06	1	-0.019461	5.3345535	0.0031461	0
Plppr2	4.51E-04	1	-0.020483	3.3455624	0.0054476	0
Cyhr1	4.40E-03	1	-0.020944	2.3562695	0.0106739	0
Sun2	4.33E-02	0.018255	-0.021416	1.363754	0.0289378	1.7386283
Wbp2	3.70E-03	1	-0.021861	2.432253	0.004509	0
Dlgap4	2.03E-02	1	-0.021889	1.6917242	0.0039652	0
Akap5	2.20E-04	1	-0.02201	3.6577377	0.0079541	0
Fgfr3	1.74E-02	1	-0.022589	1.7590267	0.0055301	0
Nfxl1	3.93E-02	6.41E-07	-0.022734	1.4053049	0.0329123	6.1929369
Lipa	1.28E-07	1.96E-62	-0.022925	6.8940574	-0.078455	61.708111
Nr6a1os	5.76E-06	6.71E-54	-0.023154	5.2396266	-0.0399256	53.173349
Pdia6	1.16E-03	1.16E-08	-0.023241	2.9371847	0.0313665	7.9360776
Phkg2	8.19E-03	5.52E-08	-0.023269	2.0866389	0.028134	7.2580345
Gm6712	1.56E-02	4E-13	-0.023408	1.8056679	0.0350797	12.397528
Sycp3	7.28E-09	6.01E-57	-0.023457	8.1376197	-0.0348293	56.221456
Mitd1	2.84E-03	1	-0.023525	2.5472921	0.0053947	0
Vnn1	1.52E-05	1	-0.023617	4.8191342	-0.0026428	0
Cdc26	2.50E-02	1	-0.023861	1.6014376	0.0108703	0
Trabd	4.14E-02	6.71E-11	-0.023941	1.3831941	0.0402347	10.173255
Pdia4	1.40E-02	1.28E-07	-0.024214	1.8551489	0.0313759	6.8937279
Tfeb	6.18E-06	1.84E-87	-0.02457	5.2091169	-0.0890996	86.735951
Pcbd1	2.89E-04	1	-0.024749	3.5393727	0.0024204	0
E430024I0!	4.70E-03	1	-0.024879	2.3278557	0.0041668	0
Zfp219	9.21E-03	1	-0.024984	2.0358649	0.0038785	0
Dnhd1	2.73E-06	1	-0.025009	5.5640314	-0.0017482	0
Klf10	6.29E-14	1.46E-67	-0.025065	13.201005	-0.0371891	66.835829
Prrc2a	6.21E-04	1	-0.025597	3.2070218	0.0007263	0
Zfp1	7.96E-04	1	-0.025687	3.0991696	0.0060118	0

Kmt5c	2.30E-06	1	-0.025759	5.637549	-0.0028972	0
D730045B(	8.42E-12	7.75E-80	-0.025778	11.074749	-0.0408608	79.110467
Top3b	2.19E-02	1.16E-05	-0.025844	1.6598925	0.0270832	4.9368579
Depp1	1.70E-07	1	-0.026089	6.7687842	0.0008307	0
Spcs2	1.10E-04	0.056281	-0.026152	3.9568289	0.0192117	1.2496353
2900089D1	3.68E-11	2.6E-81	-0.026293	10.43359	-0.0524621	80.585638
C920021L1	8.79E-06	1	-0.026374	5.0559099	-0.0035755	0
Skil	1.83E-06	1.88E-71	-0.02647	5.7382669	-0.0691756	70.726609
Herc4	8.16E-03	7.68E-51	-0.02651	2.088309	-0.1052312	50.114424
Nmrk1	2.50E-02	1.9E-14	-0.026535	1.6021119	0.0474741	13.722304
Gm48877	6.56E-04	1.5E-105	-0.026602	3.1833553	-0.0810762	104.83778
Vps37c	3.23E-04	1	-0.026612	3.4908533	-0.0019174	0
Abcd4	1.16E-02	9.68E-11	-0.026663	1.9364683	0.0358515	10.014172
Ttc36	1.58E-05	1	-0.026745	4.8000286	0.0001156	0
Sppl2b	6.30E-08	1	-0.026804	7.2003273	-0.00403	0
Atg12	7.12E-06	1	-0.027384	5.1478034	0.0020048	0
Lman1	2.23E-02	7.15E-33	-0.027401	1.6517033	0.0727956	32.145542
Impg2	3.13E-10	1	-0.02754	9.5041719	-0.004055	0
Pdzk1	2.02E-03	2.76E-08	-0.027727	2.6949938	0.0366111	7.5583395
Leap2	6.11E-12	1	-0.027856	11.214271	-0.0060513	0
Tgfbr3l	6.36E-15	1	-0.027897	14.196213	-0.0011845	0
Apoc3	1.08E-03	3.9E-16	-0.028126	2.9650424	0.0642758	15.408514
Psma4	2.28E-03	7.34E-12	-0.028168	2.64216	0.0345032	11.134219
Cep164	4.23E-03	1	-0.028334	2.3735107	0.0095511	0
Tmem144	1.16E-11	1	-0.028337	10.936155	-0.0061801	0
Zfyve27	1.92E-05	1	-0.028342	4.7164098	-0.0034757	0
Sbk1	1.53E-05	1	-0.028437	4.8162918	0.0056185	0
Aifm2	1.45E-02	0.11369	-0.028442	1.8374159	0.0193339	0.9442765
Ap3d1	2.84E-04	1	-0.028962	3.546526	0.0058924	0
Mettl26	2.83E-05	1	-0.029143	4.5486582	-0.0019483	0
Ddx60	1.42E-17	1.1E-155	-0.029197	16.84905	-0.0878838	154.94757
Zfp595	1.37E-06	1	-0.029439	5.8641439	-0.0034973	0
Izumo4	3.23E-14	0.003599	-0.029486	13.490191	-0.0129302	2.4438342
Mief2	1.54E-07	1	-0.029558	6.8136665	-0.0096743	0
Tox4	5.01E-04	1	-0.029816	3.3005141	0.007051	0
Usp49	2.22E-05	1	-0.029818	4.6527827	0.0119537	0
P3h1	4.47E-10	1	-0.029963	9.3494196	0.0115008	0
Arl4d	7.76E-11	1	-0.029991	10.110175	-0.0008293	0
Zfp326	7.04E-05	1	-0.030077	4.1522395	0.0055576	0
Ampd2	1.66E-09	1	-0.030155	8.7798508	-0.005329	0
Pitpnb	1.54E-03	2.9E-07	-0.030239	2.8117274	0.034999	6.5369444
Prrc1	6.06E-05	1	-0.030281	4.217184	0.0039969	0
Dera	3.93E-02	0.057797	-0.030285	1.4053421	0.022661	1.2380953
Scara5	5.99E-13	9E-84	-0.030309	12.222794	-0.0465473	83.045985
Dap3	8.62E-05	1	-0.030322	4.0643184	0.0040069	0
Ick	1.50E-02	0.005427	-0.030437	1.8238604	0.0260271	2.2654731
Hsd17b7	2.49E-09	3.44E-14	-0.030472	8.6034108	0.0321571	13.463607

Gtf3c2	1.18E-03	3.88E-05	-0.030581	2.9267726	0.0329736	4.4112039
Dnajc24	3.44E-04	1	-0.030747	3.463248	-0.0026328	0
Slc25a48	3.65E-10	1.7E-82	-0.030914	9.4373188	-0.0562542	81.77017
Ahsa2	6.85E-10	1	-0.030968	9.1644707	0.0032338	0
Rbm10	1.59E-03	0.274406	-0.031034	2.7998193	0.0240191	0.5616061
Tm7sf2	4.90E-18	1	-0.03108	17.309921	0.0063459	0
Ufd1	1.42E-03	1	-0.031081	2.8477396	0.0148547	0
Papola	6.49E-03	1.37E-39	-0.031104	2.1879233	-0.0904447	38.862067
Foxn3	1.86E-02	1.22E-94	-0.031432	1.7313851	-0.1728716	93.912798
Pcsk9	2.17E-17	1.05E-08	-0.031501	16.663409	0.021327	7.9793261
Mfsd14a	2.21E-05	0.003756	-0.031641	4.6560506	0.0238248	2.4253174
Rhod	3.14E-05	1	-0.031755	4.5034541	0.0058697	0
Vmp1	2.80E-03	2.72E-98	-0.031862	2.5526884	-0.150643	97.564862
Arap1	6.49E-05	1	-0.032079	4.1876701	-0.0039328	0
Tmem29	1.02E-06	0.20231	-0.03213	5.9908756	0.0187061	0.6939825
Miga2	6.70E-04	0.00205	-0.03213	3.1737661	0.0324012	2.6882925
E2f3	2.69E-06	3.37E-40	-0.032675	5.5695546	0.0860892	39.471821
Shprh	2.88E-02	1	-0.032738	1.5412824	0.0111697	0
Dot1l	1.60E-05	1	-0.032768	4.7960283	-0.0006165	0
Pfdn2	1.01E-03	1	-0.032769	2.9939141	0.0110556	0
Nxf1	1.01E-05	3.1E-10	-0.032801	4.9950532	0.0365056	9.5087627
Gm14327	1.49E-09	1	-0.033222	8.8263241	0.0019246	0
C8g	3.34E-08	1	-0.033222	7.4756463	0.0081848	0
Maged1	1.39E-15	0.696293	-0.033257	14.857721	-0.0104434	0.1572081
Pyroxd2	5.63E-08	1	-0.033326	7.2493695	-0.0022259	0
Uso1	2.41E-03	6.89E-10	-0.033351	2.6177962	0.0490075	9.1617635
Cox16	1.56E-02	8.8E-128	-0.033508	1.8076159	0.1962822	127.05716
Bnip2	4.04E-06	1	-0.033681	5.3933021	-0.0052043	0
Txndc5	4.03E-07	8.15E-05	-0.033719	6.3949977	0.0326752	4.0885898
Psma7	1.61E-08	1	-0.033741	7.7930816	0.0073685	0
Rap1b	1.13E-12	8.7E-104	-0.033909	11.946466	-0.0930949	103.05946
Mccc1	4.81E-05	1	-0.034051	4.3179075	0.0088017	0
Ppp1r37	5.01E-04	0.028712	-0.03434	3.3001816	0.0239596	1.5419335
Maea	1.87E-07	1	-0.034414	6.7289614	-0.0110133	0
Trir	1.41E-06	1	-0.03444	5.8517082	-0.0062342	0
Serpina3n	1.65E-13	0.37351	-0.034459	12.783237	-0.0100609	0.4276972
Rgl3	7.32E-06	1	-0.034515	5.1357696	-0.0070225	0
Scrn3	1.54E-03	3.7E-07	-0.034644	2.8124504	0.0390011	6.4321863
Fam53a	6.85E-08	1	-0.034874	7.1645326	-0.0074974	0
Psmb1	3.18E-05	1	-0.034888	4.4975734	0.0026368	0
Fasn	7.84E-12	0.174897	-0.035019	11.105701	0.0165945	0.7572166
Txndc12	9.67E-10	1	-0.035342	9.0144364	-0.0065913	0
Rb1	7.95E-09	3.83E-78	-0.035428	8.0997133	-0.0897717	77.417164
Usp48	1.66E-05	1	-0.035467	4.7798289	0.0031315	0
Baz2a	4.23E-04	0.000143	-0.035471	3.3739448	0.0380605	3.8437701
Epm2aip1	1.47E-11	1	-0.035556	10.833221	-0.0041839	0
Zfp946	1.13E-13	1	-0.035616	12.947567	-0.0094564	0

Ralgps2	3.71E-06	5.16E-19	-0.035632	5.4306812	0.0804664	18.28757
Ndufaf6	1.67E-04	1	-0.035857	3.7763203	0.0084371	0
Fnbp4	1.92E-04	3.92E-05	-0.036742	3.7159758	0.0381909	4.4069324
Tbc1d8b	2.33E-07	1	-0.036927	6.6323999	-0.0015822	0
Clk3	1.24E-07	1	-0.036947	6.9080658	-0.0005965	0
Fkbp14	5.47E-23	2.93E-17	-0.036964	22.261838	-0.023547	16.532545
Mdh1	2.41E-07	0.529385	-0.037422	6.6176273	0.0219963	0.2762283
Atg13	5.93E-05	0.000231	-0.037479	4.2266459	0.0298506	3.6372992
Ddx11	6.82E-15	0.000547	-0.037487	14.166303	-0.0167688	3.2623824
Idi1	4.65E-25	3.8E-06	-0.0375	24.332334	0.0221205	5.4207257
Mtch2	1.23E-03	1	-0.03751	2.9109123	0.0138063	0
Fip1l1	2.47E-04	6.07E-30	-0.037749	3.6081624	0.0750684	29.2166
Banp	4.79E-04	1.65E-69	-0.037829	3.3195407	0.12283	68.781923
Phf11c	6.21E-10	1	-0.038024	9.207253	0.0354451	0
Emsy	5.43E-06	1	-0.038625	5.2653793	-0.0058942	0
Msi2	9.85E-05	1	-0.038722	4.0065542	0.0199767	0
Prpf40b	2.82E-06	1	-0.038826	5.5501837	-0.0029251	0
B4galnt1	7.93E-16	1	-0.03888	15.100488	-0.0023865	0
Efr3a	2.45E-05	1	-0.038977	4.6109449	0.0111112	0
Il11ra1	3.47E-09	1	-0.039008	8.459408	0.0132827	0
Ncapd3	2.00E-07	2.85E-33	-0.039076	6.6991791	0.072013	32.544933
Calu	2.33E-11	1	-0.039138	10.631996	-0.0088845	0
Ncoa2	1.01E-05	1	-0.03924	4.9959626	0.0080807	0
Nap1l4	3.86E-08	1	-0.039447	7.4129416	-0.0087889	0
Gjc3	1.76E-28	4E-121	-0.039709	27.755031	-0.0507752	120.39555
Vps9d1	7.74E-13	0.005781	-0.039792	12.111371	-0.0176119	2.2379884
Sox13	2.29E-11	1	-0.039805	10.64025	-0.0039828	0
Srbd1	1.70E-03	4.27E-05	-0.040019	2.7693126	0.0420949	4.3695338
Ganc	1.17E-06	1	-0.040071	5.9313123	-0.0033136	0
Chchd3	1.58E-03	1.76E-74	-0.04014	2.8008567	0.1309405	73.75388
Tubgcp3	2.94E-09	1	-0.04019	8.531308	-0.0111853	0
Cept1	9.51E-08	1	-0.040239	7.0217498	0.0256332	0
Sugp2	2.52E-08	1	-0.040242	7.5990856	-0.0020606	0
Fam126a	8.29E-27	1	-0.040334	26.081583	-0.0094034	0
Chd8	9.28E-06	9.51E-08	-0.040343	5.0326122	0.0429404	7.0220383
Ccser2	3.60E-08	1	-0.040365	7.4435235	-0.0027856	0
Zfp384	1.66E-08	1	-0.040444	7.7800868	-0.0111099	0
Gzf1	3.47E-11	1	-0.04073	10.459748	-0.0060101	0
Pced1b	5.63E-07	1	-0.040888	6.2496712	0.0152294	0
Chkb	3.97E-07	6.75E-06	-0.04104	6.4008223	0.0325502	5.1704416
Gripap1	3.94E-07	1	-0.041156	6.4050036	0.0226016	0
Fiz1	3.70E-18	3.06E-07	-0.04147	17.431373	-0.020934	6.5136285
Smad2	2.92E-10	1	-0.041656	9.5339062	-0.0125125	0
Arhgef9	6.87E-21	0.34352	-0.041892	20.163298	-0.0126103	0.4640476
Naprt	9.39E-16	0.000135	-0.042027	15.027296	-0.0189562	3.8710119
Wars2	2.62E-08	1	-0.042372	7.5817356	-0.0106335	0
Trmt2b	5.51E-10	1	-0.042496	9.2590411	-0.0055197	0

Sult1b1	3.37E-25	7.12E-21	-0.042584	24.472062	-0.0280493	20.14771
A530072M	9.63E-14	0.025689	-0.042641	13.016162	-0.0166801	1.5902607
Zfp60	1.15E-12	0.102978	-0.042666	11.940361	-0.0159179	0.9872574
Rab3ip	2.06E-06	1.2E-11	-0.043307	5.6865076	0.0499647	10.921769
Hook1	3.23E-08	2.85E-10	-0.043366	7.4914373	0.0372214	9.5455858
1810055G(	1.54E-10	2.04E-09	-0.043379	9.8133798	0.037725	8.6909116
Trf	1.05E-03	1.6E-198	-0.043473	2.9781051	0.4228304	197.78721
Zfp598	3.13E-12	1	-0.043571	11.50408	0.0006835	0
D530033B1	6.18E-19	6.81E-90	-0.043715	18.209209	-0.0616506	89.1666
Slc19a2	2.78E-06	3.39E-18	-0.044017	5.556591	0.0567036	17.470035
2610008E1	4.58E-09	1	-0.044022	8.3393084	-0.0102437	0
Slc25a33	3.36E-10	1.7E-125	-0.044378	9.4735077	-0.1135707	124.78032
Atf7	1.03E-03	1.6E-101	-0.044436	2.9858183	-0.1417257	100.78578
Tra2a	4.98E-07	1.25E-85	-0.044715	6.3028306	-0.1245071	84.90397
Ccdc107	6.84E-23	9.06E-16	-0.044715	22.164934	-0.0279401	15.042835
Pim1	1.66E-19	3.5E-142	-0.044753	18.780599	-0.0923114	141.45215
Lman2	8.13E-10	1	-0.044757	9.0897775	0.0012581	0
Pcsk4	1.85E-26	5.69E-20	-0.04487	25.733558	-0.0284308	19.244607
Echdc1	1.68E-09	0.879297	-0.044876	8.7743087	0.0188035	0.0558643
Slc22a1	4.05E-03	1	-0.044947	2.3923681	0.0103202	0
Cdadc1	2.70E-07	1	-0.044982	6.5681515	0.0063616	0
Acbd6	3.68E-07	1	-0.045016	6.4336582	-0.0040273	0
Bmp2k	8.44E-19	8.93E-94	-0.045046	18.073566	-0.0822277	93.049375
Itih2	1.57E-09	0	-0.045058	8.8047854	0.8651086	#NUM!
Gfpt1	3.20E-15	0.03984	-0.045116	14.494385	-0.0180529	1.3996854
Prss53	1.01E-31	1.97E-29	-0.045226	30.994492	-0.0312613	28.706029
Fyttd1	2.98E-08	1	-0.045304	7.5261904	-0.0041401	0
Atxn2l	1.64E-12	1	-0.045471	11.784338	-0.015503	0
AI987944	1.04E-23	0.000105	-0.045527	22.982081	-0.0178953	3.9792135
1110019D1	7.27E-11	1	-0.045615	10.138226	0.0023711	0
Cyp2c37	1.45E-14	1	-0.045801	13.837385	0.0187232	0
Slc15a5	2.01E-30	4.5E-136	-0.045874	29.697328	-0.0605079	135.34694
Gm46329	5.01E-33	4.2E-111	-0.045906	32.300549	-0.0524952	110.37749
Bcl3	1.35E-21	3.3E-121	-0.045987	20.870999	-0.074243	120.48062
Gm16124	5.93E-15	1.24E-85	-0.046139	14.227196	-0.0877935	84.907776
Ext2	5.88E-05	1.07E-21	-0.046171	4.2308888	0.0643309	20.972494
Tram1	1.30E-13	1	-0.046254	12.885527	-0.0144011	0
Sash1	4.61E-07	0	-0.046673	6.3359505	-0.2882315	#NUM!
Zfp281	1.56E-19	1.09E-06	-0.046955	18.806321	-0.0227612	5.9635528
Papss2	2.54E-11	9.27E-30	-0.047015	10.594934	0.1181921	29.032714
Arsg	2.57E-09	1	-0.047373	8.5904391	0.0134235	0
Steap4	1.34E-23	2.2E-114	-0.047378	22.873219	-0.0742384	113.65117
Vcpip1	4.98E-10	1	-0.047449	9.3029307	-0.001658	0
Rara	2.63E-25	9.8E-107	-0.047449	24.579653	-0.0689044	106.01085
Gm39469	5.18E-14	0.021776	-0.047493	13.285794	-0.0206309	1.6620139
Asxl1	4.38E-05	2.23E-98	-0.047497	4.3585446	-0.136302	97.652421
Psmb7	2.55E-08	1	-0.047545	7.5926733	0.0166278	0

Mib1	1.23E-06	1	-0.047576	5.9107972	-0.0084532	0
Vps37a	2.22E-07	1	-0.047601	6.652749	0.0154368	0
Shpk	4.62E-11	1	-0.047604	10.335602	-0.0082707	0
Erg28	1.66E-21	1	-0.047645	20.780676	0.0167281	0
Nbea	3.41E-12	1	-0.047774	11.466951	-0.0075606	0
Vwa3b	3.90E-29	4.97E-24	-0.048271	28.408723	-0.0319001	23.303471
Zfp410	1.17E-10	1	-0.048394	9.9325795	0.0014499	0
Litaf	1.72E-22	4.1E-208	-0.048414	21.76533	-0.1675262	207.39064
Slain2	2.58E-09	1	-0.048503	8.5879194	-0.0130014	0
Zfp654	7.14E-08	1	-0.04853	7.146445	-0.0096933	0
Cs	1.15E-09	1	-0.048745	8.9394025	-0.0101464	0
Mrpl1	4.79E-11	1	-0.048803	10.319459	0.0044969	0
Nek7	2.30E-07	4.51E-59	-0.048841	6.6386329	-0.1138632	58.346284
Mup14	2.67E-46	5E-133	-0.04903	45.573639	-0.0531164	132.3013
C4a	4.90E-25	2.4E-149	-0.049138	24.309618	-0.0853158	148.62675
Atf4	9.48E-12	1	-0.04922	11.023182	0.0066418	0
Nsd1	8.80E-13	1.9E-132	-0.04924	12.05575	-0.1669484	131.72738
Klhl7	1.44E-14	1	-0.049501	13.841893	-0.0136252	0
D930016D(	1.04E-09	1	-0.049687	8.9821365	0.0063154	0
Ptp4a2	1.97E-13	4.73E-81	-0.049721	12.704706	-0.1087507	80.325273
Scly	2.67E-09	1E-36	-0.049788	8.5732544	0.0844482	35.999759
Tfg	2.23E-13	1	-0.049792	12.65203	-0.0138839	0
Gm42109	4.95E-14	1	-0.049849	13.305104	0.002699	0
Dock8	6.28E-25	5.5E-161	-0.04996	24.201714	-0.142623	160.25877
Etfb	3.41E-14	1	-0.050088	13.467455	-0.0077213	0
As3mt	2.16E-25	1.93E-05	-0.050202	24.666382	-0.0211084	4.7147858
Cabcoco1	7.42E-15	1	-0.050219	14.129594	-0.0022863	0
Gm13944	1.73E-21	1E-132	-0.050384	20.762926	-0.0780972	131.98413
Gtf3c1	2.89E-08	2.9E-19	-0.050578	7.5391699	0.0579535	18.537083
Asb7	2.30E-15	1	-0.050708	14.639134	0.0139337	0
Sfi1	4.22E-13	0.007841	-0.050818	12.374605	0.0290497	2.1056126
Gbf1	4.13E-08	1	-0.050852	7.3838727	-0.0066546	0
Zfp592	2.34E-08	1	-0.050864	7.630095	-0.0063608	0
Pum1	2.03E-09	8.44E-75	-0.050868	8.6921102	-0.1248275	74.073454
Ctnnd1	1.06E-06	1.11E-49	-0.050956	5.9735348	-0.1010145	48.955555
Bag3	6.66E-11	1	-0.051214	10.176828	-0.018145	0
Ndufs3	7.98E-12	1	-0.051576	11.097735	0.0005132	0
Fbxw11	1.48E-09	1	-0.051634	8.8308951	0.0221108	0
Plcg1	3.74E-12	1	-0.051806	11.426665	0.0159776	0
Selenop	1.11E-04	1	-0.051847	3.9544841	0.0291952	0
U2surp	1.59E-08	1	-0.05205	7.7975349	0.0241837	0
Zfyve9	1.08E-08	1	-0.05225	7.9670876	0.0056418	0
Apoc4	2.99E-12	6.23E-89	-0.052356	11.524436	0.1398674	88.205675
Csnk2a1	1.25E-07	1	-0.052367	6.9039907	-0.0088297	0
Zfp707	8.43E-18	1	-0.052407	17.074324	0.0028485	0
Bid	2.74E-33	9.8E-135	-0.052444	32.562396	-0.0718899	134.00698
Slc25a38	1.98E-24	1	-0.05257	23.703708	-0.0021551	0

Fbxw4	1.85E-11	1	-0.052794	10.732449	0.0001713	0
Yeats2	1.87E-13	1	-0.052952	12.729164	-0.0106781	0
Brd1	2.69E-11	1	-0.053071	10.571018	-0.007605	0
Cep44	3.88E-14	1	-0.053269	13.411498	-0.0180394	0
Gm2061	2.44E-49	1.1E-146	-0.053281	48.611791	-0.0584406	145.94112
Ola1	2.93E-13	1	-0.053515	12.533044	0.0019133	0
Pkhd1	4.97E-08	1.54E-19	-0.053643	7.3038067	0.076959	18.813627
Pex16	4.74E-19	4.99E-06	-0.053861	18.324192	-0.0245626	5.3022894
Kifc2	2.66E-42	1.15E-34	-0.054253	41.575594	-0.0357377	33.940574
Acaa1a	6.77E-12	1	-0.054272	11.169361	-0.0033226	0
Zfp276	5.76E-15	0.034774	-0.05429	14.239614	-0.021751	1.4587456
Zfp236	3.97E-07	1	-0.054302	6.4008223	-0.0090908	0
Nmt1	8.30E-13	1	-0.054357	12.081069	-0.0034084	0
Pknox1	9.00E-19	1	-0.054387	18.045896	-0.0143777	0
Nr2c2	2.15E-09	4.16E-76	-0.054412	8.6681217	-0.1131622	75.381404
Met	4.77E-06	1.42E-11	-0.054459	5.3210746	0.0588245	10.84827
Itsn1	1.55E-10	1.3E-115	-0.05447	9.8107336	-0.1472513	114.89251
Kat2b	1.66E-09	4.57E-96	-0.054507	8.7807562	-0.1504995	95.33962
Tmem260	1.42E-09	1	-0.054553	8.847275	0.0048392	0
Cstf3	1.33E-05	1	-0.054818	4.8755523	-0.0023756	0
Mpp4	1.09E-27	1.63E-06	-0.054936	26.964038	-0.0217581	5.7872828
Akap17b	2.22E-37	5.4E-40	-0.055038	36.654341	-0.040475	39.267792
Dusp1	1.17E-32	9.3E-157	-0.055537	31.930975	-0.0876339	156.0321
Zc3hav1	7.39E-16	2.6E-126	-0.055632	15.131525	-0.1315318	125.58724
Scd1	3.51E-20	1.1E-113	-0.055632	19.45461	-0.1042688	112.94806
Dhx36	4.56E-09	2.21E-07	-0.05569	8.3408044	0.0419095	6.656547
Tet2	4.17E-15	1	-0.055868	14.379825	0.004623	0
Dmgdh	3.51E-05	2.18E-23	-0.056007	4.4551581	0.0939266	22.660784
Ppm1a	1.20E-12	0.046097	-0.056283	11.920349	-0.0247616	1.3363309
Ppp2r5a	7.97E-07	2.56E-68	-0.05672	6.09857	-0.1260091	67.590988
Tlcd1	4.54E-28	1.53E-10	-0.056871	27.342857	-0.027956	9.8144719
Slc25a10	2.00E-24	8.11E-13	-0.056872	23.699518	-0.0316317	12.090873
Pip5k1a	9.42E-14	1	-0.056874	13.026077	-0.0185121	0
Tmc4	5.13E-39	4.19E-41	-0.05699	38.290157	-0.0421504	40.378068
Aven	1.37E-09	1	-0.05761	8.8637193	-0.0018124	0
Mbl2	1.10E-23	4.11E-07	-0.057824	22.958245	-0.0258059	6.3862488
Vcl	9.62E-17	0.147957	-0.05821	16.016827	-0.0097667	0.8298634
Ppme1	7.45E-16	1	-0.058294	15.128085	-0.0117307	0
Trp53bp1	2.57E-22	0.004324	-0.058342	21.590871	-0.0205349	2.3641629
Scaper	4.01E-11	0.000464	-0.058591	10.396693	0.0403197	3.3338403
Zfp523	1.80E-29	3.69E-23	-0.058681	28.744223	-0.0379444	22.433363
Cux1	3.04E-11	6.5E-166	-0.058868	10.517537	-0.193712	165.18939
Tmed10	5.34E-18	3.18E-08	-0.059173	17.272303	-0.0348359	7.498116
Pja2	1.72E-15	1	-0.059309	14.765041	0.004265	0
Zfp110	7.90E-20	2.41E-07	-0.059438	19.102236	-0.0308675	6.6176283
Bnip3	1.34E-12	3.2E-115	-0.059568	11.874404	-0.1330052	114.49387
Stx5a	2.45E-12	6.11E-53	-0.059778	11.61039	0.1018618	52.213984

Hmg20a	1.85E-11	1	-0.059879	10.7325	-0.005957	0
Arl5a	1.58E-15	1	-0.059943	14.802459	-0.0094136	0
Nhsl1	1.06E-06	1	-0.059984	5.9733574	0.0177532	0
Pcdh1	1.44E-21	6.03E-97	-0.060269	20.842854	-0.0852727	96.219624
Esyt2	1.62E-13	1	-0.06048	12.789276	0.0095022	0
Arid1a	6.09E-09	2.7E-110	-0.060625	8.2150932	-0.1474799	109.57214
Med26	6.97E-14	1	-0.060718	13.156916	-0.0202921	0
Aco1	3.17E-13	1	-0.060728	12.49961	0.0103109	0
Zfp653	2.99E-35	6.47E-29	-0.060785	34.524158	-0.0405274	28.188803
Cideb	2.95E-32	1.86E-27	-0.060813	31.529662	-0.0417743	26.731626
Shmt2	3.27E-28	3.44E-08	-0.060892	27.485727	-0.0265197	7.4628515
Fdft1	3.76E-30	4.83E-58	-0.060936	29.424576	0.0996483	57.316319
Cbx4	1.16E-31	4.7E-130	-0.060967	30.933968	-0.0809793	129.32864
Stat3	6.42E-08	3.6E-180	-0.061159	7.192724	-0.2123287	179.44071
9130016M	1.67E-18	3.12E-06	-0.061198	17.776885	-0.0308036	5.5062076
Zfp780b	2.31E-18	1	-0.061231	17.636084	-0.0025249	0
Copz1	1.91E-19	1.06E-08	-0.061265	18.717832	-0.0335915	7.9742041
Thrsp	1.25E-39	3.9E-39	-0.061773	38.901495	-0.0445279	38.408396
Wdr4	2.34E-23	6.59E-05	-0.061793	22.630144	-0.0248751	4.1811612
Tmcc1	2.62E-12	9.2E-102	-0.062115	11.582341	-0.1521486	101.03801
Dlg3	8.45E-33	1.5E-10	-0.062211	32.073211	-0.0279468	9.8237952
Tead1	6.32E-13	6.1E-34	-0.062379	12.199323	0.0876171	33.214792
Osbpl11	2.48E-14	0.004681	-0.062471	13.606029	-0.0282214	2.3296669
Pccb	1.45E-29	4.55E-19	-0.062977	28.839697	-0.0385423	18.341787
Zbtb7b	3.74E-30	3.97E-23	-0.063082	29.427026	-0.0397678	22.401162
Shf	2.14E-50	5.66E-52	-0.063086	49.668945	-0.0449762	51.247431
Nim1k	9.35E-33	8.29E-08	-0.063251	32.029314	-0.0254764	7.0812314
Mtss1	4.36E-02	1.5E-24	-0.063372	1.3609722	0.1172293	23.82459
Wnt5b	1.28E-35	1.26E-23	-0.063686	34.893282	-0.037872	22.89956
Pmm2	1.69E-17	1	-0.063726	16.772154	0.0161844	0
C330002G(	1.37E-39	1.9E-139	-0.06387	38.862179	-0.0784943	138.7235
Cobl	7.29E-09	0.715233	-0.064174	8.137156	0.0281534	0.1455527
4930470G(	1.13E-27	1	-0.064364	26.946621	0.0059639	0
Ralgapa2	6.79E-09	4.9E-140	-0.064575	8.1681823	-0.1805243	139.3089
Rab28	3.83E-22	4.8E-08	-0.064692	21.41686	-0.0329991	7.3191455
Olfml1	5.75E-37	1.68E-36	-0.064843	36.239978	-0.0454364	35.775297
Diaph1	2.37E-11	1.5E-105	-0.06505	10.625896	-0.1462206	104.82831
Ski	1.36E-32	2.87E-25	-0.065099	31.865053	-0.0465746	24.542233
Dcaf7	6.84E-24	1.57E-14	-0.065278	23.165246	-0.0385461	13.803901
Zfp369	3.45E-24	3.06E-13	-0.065443	23.461744	-0.0370919	12.514855
Nr1h3	6.59E-27	2.77E-14	-0.065586	26.181145	-0.0390348	13.557802
Map2k7	1.15E-27	0.000976	-0.065698	26.93847	-0.0222416	3.0104315
Vps41	9.16E-10	1	-0.065745	9.0382266	-0.0184158	0
Prpf6	4.39E-13	0.045295	-0.06589	12.357425	-0.0269154	1.3439455
Agrn	3.73E-37	6.28E-29	-0.06658	36.428062	-0.0436059	28.202368
Wwc1	7.47E-08	1	-0.066721	7.126689	0.0225769	0
Supt5	3.50E-16	1	-0.066969	15.456	0.0161765	0

Kcnc3	4.21E-41	8.05E-28	-0.06728	40.375436	-0.0403691	27.09427
Cyp51	1.02E-43	0.004412	-0.067318	42.991767	0.0331036	2.3553623
Nsdhl	4.82E-48	6.5E-151	-0.067434	47.316675	0.1415817	150.18813
Ncoa3	1.68E-30	1.3E-164	-0.067639	29.775502	-0.1341254	163.90303
Rbpms	5.02E-08	1	-0.067661	7.299689	0.0034096	0
Stt3b	1.76E-19	0.000594	-0.067813	18.754944	-0.0273568	3.2259985
Hibch	1.60E-14	1	-0.068176	13.795936	0.0206981	0
Ccni	1.08E-26	6.89E-15	-0.068247	25.965786	-0.0413466	14.161524
Chrna4	2.18E-60	2E-57	-0.068424	59.661336	-0.0439634	56.699098
Ubtd1	9.64E-31	6.9E-128	-0.068599	30.015938	-0.1015249	127.16031
Wasl	1.06E-14	1	-0.068707	13.97306	0.0064194	0
Bcl7c	3.12E-46	8.03E-16	-0.068804	45.505854	-0.0306476	15.095116
Cxxc5	5.07E-18	0.221359	-0.068907	17.295379	-0.0205128	0.6549029
Epb41	3.44E-14	0.008027	-0.069007	13.463004	0.0513809	2.0954356
Taok3	9.54E-12	1	-0.069572	11.020544	0.0049711	0
Lcor	7.89E-17	9.9E-141	-0.069619	16.102838	-0.1742603	140.00445
Ccnd3	6.18E-16	0	-0.069677	15.208735	-0.2725018	#NUM!
Bace1	9.72E-23	4.26E-08	-0.069731	22.012546	-0.0328171	7.3702611
Habp4	2.20E-29	3.46E-19	-0.069909	28.657115	-0.0421485	18.460462
Cyp8b1	1.41E-52	1.78E-36	-0.070145	51.851987	-0.0426982	35.750598
Hjv	4.49E-27	1	-0.070387	26.347442	-0.0183706	0
Mtmr4	7.90E-37	1.32E-29	-0.070491	36.10254	-0.0467134	28.878524
B3gat3	6.05E-27	6.15E-11	-0.070575	26.218439	-0.0366976	10.210845
Med15	7.57E-17	1	-0.070826	16.120859	-0.0130344	0
Selenoo	6.33E-24	0.001995	-0.070926	23.198872	-0.0252604	2.6999485
Sult1a1	8.88E-43	0.000147	-0.071103	42.05138	0.0260869	3.8331234
Iws1	4.28E-15	1	-0.071265	14.368506	-0.0141027	0
Mup2	1.24E-17	2E-166	-0.072076	16.906641	-0.1391216	165.70406
Spen	6.67E-18	0.026639	-0.072172	17.176159	-0.0263765	1.5744811
Hopx	9.19E-50	2E-169	-0.072304	49.03665	-0.0866179	168.6988
Tpmt	5.01E-31	4.69E-09	-0.072763	30.300132	-0.0308521	8.3285711
Plbd1	4.10E-36	4E-17	-0.072787	35.387155	-0.0406892	16.398432
Tm4sf1	2.51E-51	8.3E-56	-0.072819	50.60028	-0.0536164	55.080976
Uap1	2.61E-26	4.06E-07	-0.072848	25.582755	-0.031875	6.3914419
Hhex	4.88E-21	1	-0.072962	20.311668	-0.0142446	0
Pctp	4.67E-38	1.38E-34	-0.073157	37.330851	-0.0503537	33.861113
Ankrd17	1.41E-21	1.69E-10	-0.073212	20.852044	-0.0462012	9.7709758
Zfx	3.05E-21	6.34E-11	-0.073694	20.515575	-0.0425417	10.198042
Socs2	7.67E-60	9.72E-67	-0.073972	59.115172	-0.0551124	66.012243
Slc29a1	7.03E-12	1	-0.074448	11.1528	-0.0137469	0
Prickle1	1.02E-40	1.36E-21	-0.074606	39.991915	-0.0437257	20.866466
Slc16a12	5.99E-21	1	-0.074628	20.222711	-0.0140369	0
Hipk3	6.60E-22	2.8E-113	-0.074642	21.180649	-0.1210271	112.55866
Mob3b	6.37E-06	1	-0.074921	5.1959871	0.01474	0
F12	1.35E-17	1.36E-30	-0.075069	16.869096	0.0777022	29.865578
Tmem205	2.50E-23	2.37E-07	-0.07517	22.602838	-0.0369416	6.6244569
Fat1	1.53E-22	1.01E-09	-0.075295	21.816004	-0.0382079	8.9946814

0610040J0	3.62E-12	1.55E-20	-0.075534	11.441238	0.0817881	19.809005
Acsl3	5.75E-38	1	-0.075559	37.240406	-0.0034396	0
Pnpla8	1.52E-15	1	-0.075737	14.819181	0.003483	0
Ppip5k2	1.60E-11	1	-0.07577	10.796786	0.0122726	0
Josd2	5.50E-37	3.05E-17	-0.075855	36.259914	-0.0401541	16.515817
Cpsf7	3.09E-28	9.73E-10	-0.076064	27.510277	-0.0371764	9.011951
Dnaja1	8.01E-25	1	-0.076144	24.096464	-0.0152522	0
Zfp652	5.82E-16	4.7E-129	-0.076221	15.235361	-0.1619534	128.332
Lasp1	2.35E-43	5.5E-175	-0.076227	42.629169	-0.105263	174.25676
Hdlbp	5.40E-19	1.34E-27	-0.076281	18.2679	0.0888428	26.874479
Ncoa5	5.24E-22	1.33E-09	-0.076302	21.280724	-0.0404088	8.8762364
Gm32828	1.26E-55	1.26E-61	-0.076414	54.899674	-0.0566532	60.898201
Fdxr	2.24E-42	2.12E-38	-0.076562	41.649076	-0.0530022	37.674051
Nploc4	1.30E-16	1	-0.076798	15.886866	-0.0239216	0
Mef2d	1.28E-16	2.6E-129	-0.076914	15.893914	-0.1546654	128.58598
Btrc	3.83E-16	0.000329	-0.076943	15.41662	-0.0347157	3.4826568
Anxa6	6.97E-34	6.13E-13	-0.076966	33.156907	-0.0352504	12.212845
Nadk	2.32E-22	2.76E-10	-0.076993	21.634902	-0.0440851	9.5594967
Dlg1	1.67E-19	2E-131	-0.077055	18.777966	-0.1713141	130.69754
Paxx	1.05E-60	2.4E-72	-0.077315	59.978035	-0.0600531	71.619782
Fdps	2.16E-51	2.37E-24	-0.077691	50.666	0.0565116	23.625727
Xpnpep3	1.35E-22	0.471952	-0.077901	21.869304	-0.0218417	0.3261022
Ppp6r3	4.61E-18	1.07E-96	-0.078074	17.33634	-0.1374659	95.971842
Wdr20	3.78E-18	1.05E-05	-0.078095	17.422566	-0.0389517	4.9771568
Sesn3	4.40E-36	4.42E-14	-0.078428	35.356991	-0.0365414	13.354228
Mapk15	7.74E-52	1.4E-215	-0.078538	51.111384	-0.1091002	214.84695
Efna1	1.87E-30	5.2E-136	-0.078752	29.727852	-0.1127142	135.28612
Lrp6	5.06E-16	1.3E-127	-0.079096	15.2956	-0.1645025	126.89999
Immt	1.82E-27	2.87E-07	-0.079187	26.740397	-0.0351531	6.542533
Phc3	2.56E-21	0.000125	-0.079531	20.592547	-0.0342856	3.9015687
1110059E2	1.62E-21	2.84E-10	-0.079628	20.790985	-0.0420228	9.5462085
Echs1	1.32E-38	3.58E-15	-0.079678	37.878525	-0.0395028	14.446578
Gprasp1	2.71E-30	0.000102	-0.079766	29.566424	-0.0282607	3.9913438
Lama3	1.57E-60	1E-66	-0.079891	59.803906	-0.0591475	65.998909
Mavs	8.05E-31	6.99E-14	-0.080053	30.094015	-0.0396393	13.155281
Syap1	1.90E-30	0.130874	-0.080065	29.722054	-0.0217194	0.8831454
F10	9.08E-24	2.44E-08	-0.080106	23.042067	-0.037751	7.6123283
Bhlhe40	7.57E-59	3.2E-196	-0.080431	58.12087	-0.0977581	195.49539
Yap1	4.29E-15	1	-0.080592	14.367176	0.0195084	0
Vps13c	2.52E-20	5.7E-190	-0.08071	19.59915	-0.1864379	189.24247
Tmem163	9.15E-66	9.7E-209	-0.080755	65.038659	-0.0955769	208.01135
Zfp644	3.18E-16	2.52E-78	-0.08098	15.497014	-0.1285515	77.598884
Map7	1.47E-17	0.066751	-0.08189	16.833565	-0.026767	1.1755394
4930556N1	1.79E-32	2.6E-158	-0.082063	31.74785	-0.1239099	157.59208
Gm35188	5.01E-21	1.9E-162	-0.082075	20.300052	-0.1503078	161.715
Lnpep	2.21E-29	1.2E-207	-0.082329	28.656261	-0.1841694	206.91545
E230016M	2.49E-41	6.9E-158	-0.082379	40.603733	-0.1067117	157.15888

Fam126b	6.63E-21	1	-0.082554	20.178741	-0.0041376	0
Cnksr3	3.15E-34	0.000875	-0.082898	33.501751	-0.0273989	3.0580154
Bphl	2.86E-22	5.59E-08	-0.083033	21.543626	-0.0417412	7.2525071
Cdkl5	7.65E-32	1.9E-22	-0.08337	31.116556	-0.0501328	21.720207
Ddx46	4.71E-23	0.044878	-0.084302	22.326722	-0.0272776	1.3479708
Ubn2	3.82E-21	2.4E-152	-0.084461	20.418029	-0.1857208	151.62059
Farp2	5.93E-24	5.89E-06	-0.084734	23.226733	-0.0366366	5.2295845
Ctnnbl1	1.89E-33	5.34E-09	-0.084751	32.723542	-0.0349228	8.2728239
Slk	2.46E-32	7.59E-12	-0.085045	31.608444	-0.0424171	11.119935
C730002L0	2.42E-15	3.6E-133	-0.085058	14.616658	-0.1835163	132.43962
Dennd1a	7.00E-26	4.2E-160	-0.085349	25.15511	-0.1945167	159.37184
Peli1	2.71E-25	3.6E-174	-0.085487	24.567049	-0.1733758	173.44437
Acly	2.35E-41	0.000169	-0.085489	40.628506	-0.0211918	3.7732507
Galnt2	3.77E-29	4.95E-16	-0.086018	28.42378	-0.048748	15.305738
Fam120c	3.23E-34	1	-0.086257	33.490702	-0.0029643	0
Dnajc22	5.92E-44	6.25E-27	-0.086282	43.227723	-0.0502328	26.203959
Bend7	4.02E-29	1.8E-10	-0.08638	28.395296	-0.0414737	9.7455772
Slc9a3r1	1.04E-59	1.8E-198	-0.086576	58.981169	-0.1064912	197.73486
Tecr	1.28E-39	1.98E-05	-0.08664	38.892095	-0.0276223	4.7040713
Sel1l	1.00E-21	1	-0.086691	20.999957	0.0062204	0
Gabpb2	4.79E-25	3.89E-12	-0.087153	24.319253	-0.0469232	11.410162
Slc3a1	1.00E-38	4.87E-28	-0.087603	37.998096	-0.0540204	27.312669
Gpbp1l1	1.72E-26	5.07E-12	-0.087778	25.763873	-0.0471629	11.294637
Pias2	7.53E-25	1.02E-08	-0.087946	24.123291	-0.04131	7.992423
Mbnl1	2.44E-29	1E-263	-0.088437	28.612074	-0.2557903	262.99305
Nat8f2	1.20E-52	2.7E-230	-0.088508	51.922584	-0.1224361	229.57276
Tiam2	1.08E-68	6.8E-210	-0.088701	67.967284	-0.0999854	209.16914
Adck1	3.03E-37	2.51E-17	-0.088791	36.519201	-0.0466023	16.600267
Fam20c	3.20E-58	5.2E-239	-0.08896	57.495159	-0.1494594	238.28427
Tle1	6.97E-19	5.58E-49	-0.089196	18.156975	0.124578	48.253219
Tmem30a	8.56E-34	7.38E-14	-0.089304	33.067489	-0.0464723	13.131712
BC028777	2.08E-50	1.5E-178	-0.089426	49.681085	-0.1109549	177.82036
Ambra1	7.91E-28	2.5E-105	-0.089552	27.101811	-0.1506783	104.60833
Rspry1	2.23E-32	3.61E-23	-0.089824	31.651212	-0.0598304	22.442575
Chd2	5.28E-26	5.8E-221	-0.090099	25.277152	-0.2309412	220.23371
Ttc38	2.21E-42	2.19E-39	-0.090166	41.655207	-0.0634114	38.660408
Arhgef37	2.21E-33	4.34E-10	-0.090178	32.656187	-0.0410086	9.362576
Pxmp2	1.80E-34	5.1E-07	-0.090424	33.745015	-0.0318981	6.2921728
Pias1	5.36E-25	2.4E-171	-0.090537	24.271151	-0.1765802	170.61625
Sfpq	8.34E-24	2.9E-117	-0.090665	23.07884	-0.1461169	116.53124
Bcr	3.14E-29	1	-0.091246	28.503238	0.0269628	0
Mpdz	8.12E-70	5.84E-88	-0.091272	69.090459	-0.0733677	87.233861
Lgals4	3.11E-36	0.000101	-0.091321	35.506873	-0.0278733	3.9938979
Dcaf6	5.02E-23	1	-0.092088	22.299067	-0.0085352	0
Mllt3	1.21E-45	3.38E-40	-0.092123	44.916295	-0.0686645	39.471391
Nudt4	4.30E-32	7.92E-06	-0.092599	31.366404	-0.0327596	5.1014555
Car1	4.21E-90	5.4E-242	-0.092661	89.375711	-0.0977945	241.26954

Sec24a	1.57E-24	0.000294	-0.092937	23.803195	-0.0349271	3.5318888
Faf2	9.71E-29	8.56E-08	-0.093186	28.012993	-0.0399391	7.0674426
Taf3	9.90E-27	7E-08	-0.093412	26.004182	-0.0445744	7.154838
Taok2	3.91E-42	1.79E-18	-0.093446	41.408358	-0.0477229	17.748174
Qk	2.13E-26	2E-295	-0.093534	25.67222	-0.2428903	294.70251
1300017J0	1.77E-25	2.87E-08	-0.09372	24.751349	0.0480998	7.5416739
Cbx5	7.25E-41	5.82E-33	-0.093779	40.139605	-0.0621717	32.234751
Prodh	5.27E-39	6.96E-22	-0.093962	38.277932	0.0638167	21.157391
Relch	3.14E-29	3.14E-10	-0.094055	28.502948	-0.0444831	9.5035253
Ppp6r2	5.09E-30	2.86E-14	-0.09471	29.292864	-0.052092	13.543437
Spns2	6.86E-83	6.47E-84	-0.095481	82.16352	-0.0669543	83.189059
Stard4	2.00E-84	2.6E-71	-0.09557	83.69963	-0.0618218	70.585838
Nat10	2.34E-37	0.177963	-0.095693	36.630121	0.0290638	0.7496709
Clybl	9.31E-19	1.01E-06	-0.095896	18.031085	-0.0495913	5.9968838
Firre	6.21E-33	1	-0.096251	32.206788	-0.006392	0
Osbpl9	2.83E-28	1.7E-193	-0.096332	27.547559	-0.2139087	192.78121
Msmo1	1.34E-47	7.95E-70	-0.096471	46.872057	0.1319764	69.09957
Farp1	9.15E-23	8.8E-12	-0.096518	22.038651	-0.0551508	11.055624
B2m	1.91E-51	8.6E-190	-0.097248	50.718796	-0.1415334	189.06467
Kmt5b	5.84E-30	3.39E-22	-0.097394	29.233246	-0.0654356	21.470318
Ambp	1.41E-38	4.4E-17	-0.097608	37.850034	-0.0505574	16.356683
Nrf1	3.34E-24	6.4E-108	-0.097707	23.476898	-0.1455408	107.19147
Slc35d2	7.08E-26	5.09E-11	-0.098115	25.150042	-0.0519689	10.293314
Rmdn1	3.27E-32	4.15E-05	-0.09835	31.485482	-0.0352998	4.3818672
Mfhas1	5.88E-29	1.3E-162	-0.098451	28.230839	-0.1662506	161.87528
Fam210a	1.52E-34	3.2E-137	-0.098701	33.817103	-0.1377582	136.50065
Tjp1	9.34E-45	2.92E-07	-0.098994	44.029599	-0.0341494	6.5341741
Pafah1b1	5.54E-30	1.3E-135	-0.099424	29.25622	-0.1711213	134.89383
Got2	5.88E-36	1.82E-06	-0.099586	35.230261	-0.0328336	5.7392264
Atp1b1	1.70E-35	6.49E-18	-0.0997	34.769211	-0.0530646	17.187935
Cpox	4.23E-50	9.88E-27	-0.099707	49.374027	-0.0558177	26.005288
Dennd5a	5.18E-50	3.02E-35	-0.099978	49.285887	-0.0662341	34.519896
Pm20d1	3.62E-54	1.22E-52	-0.100138	53.441023	-0.0722866	51.914307
Ercc5	1.47E-46	3.35E-12	-0.100397	45.831598	-0.0409493	11.474647
Pfkfb2	3.58E-27	9.16E-05	-0.100858	26.445796	-0.0406376	4.0381154
Pnldc1	9.45E-60	2.54E-67	-0.100872	59.024422	-0.0765972	66.594695
Gsk3b	1.25E-28	1.2E-214	-0.101304	27.902526	-0.220895	213.93067
Hsf2	5.88E-49	0.192223	-0.101317	48.230618	-0.021436	0.7161941
Xpo7	9.00E-27	5.3E-109	-0.101365	26.045645	-0.1538821	108.27325
Hhat	7.07E-49	3.31E-39	-0.101457	48.150332	-0.0688149	38.479733
Rab10	1.73E-27	1.9E-265	-0.101944	26.762388	-0.2474365	264.73028
Grk5	1.14E-44	5.4E-207	-0.102116	43.941645	-0.1740452	206.26667
Enpep	9.96E-84	1.9E-249	-0.102471	83.001648	-0.1153023	248.72635
Aspdh	1.35E-79	2.34E-20	-0.102589	78.868492	-0.0380116	19.630518
Gin1	7.60E-22	1	-0.103286	21.119204	-0.0397997	0
Zfp36l1	4.96E-36	2.73E-08	-0.103443	35.304237	-0.03229	7.5645376
Cnn3	1.91E-34	1	-0.10366	33.720043	0.0098685	0

Bche	5.41E-43	2.08E-26	-0.103873	42.267121	-0.0642875	25.681101
Gm13483	1.96E-37	2.55E-23	-0.104089	36.707112	-0.0646139	22.594209
Gm45425	1.86E-96	1.1E-153	-0.104609	95.730985	-0.0893941	152.95626
44448	5.68E-30	0.013583	-0.105067	29.245353	-0.0295777	1.8670188
Rab14	1.95E-42	1.48E-36	-0.105196	41.709341	-0.0755272	35.829778
Pfkfb1	1.63E-35	3.06E-13	-0.105629	34.789137	0.0528166	12.51489
Serpind1	2.05E-63	6.79E-31	-0.105774	62.68734	-0.0540385	30.168217
Tacc1	3.01E-25	0	-0.106471	24.520971	-0.2646796	#NUM!
Arhgap23	4.70E-76	1.73E-73	-0.107156	75.327783	-0.0798621	72.761528
Gatad2b	2.91E-29	8E-161	-0.108081	28.535508	-0.1823516	160.09665
Map2k4	9.89E-36	0.001884	-0.108243	35.004782	-0.0328398	2.7249246
Ppp4r4	1.94E-81	3.61E-87	-0.10836	80.713313	-0.0799247	86.442034
Mccc2	2.44E-47	1.14E-38	-0.108559	46.612842	-0.0731435	37.941996
Mapk1ip1l	2.40E-47	7.64E-33	-0.10908	46.619389	-0.0688844	32.117076
Klhdc10	3.10E-34	5.25E-07	-0.109212	33.509201	-0.0419346	6.2802304
E130308A1	8.10E-43	6.42E-14	-0.109316	42.091485	-0.0490792	13.19218
Kdm5b	1.20E-36	0.000835	-0.109436	35.92134	-0.0325084	3.0783422
Foxk2	3.26E-50	5.23E-14	-0.10976	49.486547	-0.0473558	13.281426
Kank1	3.80E-33	0.000103	-0.109762	32.420595	-0.0379808	3.986317
Etnk2	4.42E-12	4.3E-66	-0.110102	11.354853	0.15135	65.366035
Dnajc3	4.57E-38	1.53E-09	-0.110147	37.340293	-0.044136	8.815477
Fxyd1	3.92E-63	6.53E-39	-0.110233	62.407216	-0.0629771	38.185283
AU040320	8.22E-47	3.09E-34	-0.110859	46.085036	-0.0714366	33.510571
Elovl6	4.12E-35	1	-0.11094	34.385529	0.0182791	0
Qrich1	1.41E-42	1.23E-30	-0.11115	41.851717	-0.0725074	29.909708
Gm30835	3.29E-72	5.7E-237	-0.111491	71.482981	-0.1330059	236.24236
Reep6	4.04E-57	5E-58	-0.111712	56.393912	-0.0822986	57.301424
Fnip1	2.74E-36	1.4E-301	-0.111789	35.562607	-0.2424824	300.86122
Tram2	9.21E-51	9.7E-197	-0.111933	50.035875	-0.156181	196.01503
Cyth1	1.01E-37	1E-273	-0.111945	36.996745	-0.231805	272.99514
Ttll5	4.90E-41	7.33E-13	-0.112064	40.310239	-0.0474198	12.134655
Srebf2	2.83E-55	1	-0.112109	54.548814	-0.0088577	0
Pdia3	4.10E-48	7.51E-29	-0.112214	47.387657	-0.0654703	28.124245
Slc17a4	6.92E-67	3.03E-05	-0.112286	66.160124	-0.0186788	4.51806
Zfp975	1.17E-63	3.81E-17	-0.112435	62.933559	-0.0456996	16.419116
Gcgr	9.91E-29	1	-0.11267	28.003817	0.0005406	0
Tef	4.28E-66	4.3E-253	-0.112856	65.368364	-0.1482811	252.36863
F2	5.92E-39	0.281264	-0.113042	38.227357	0.0300257	0.5508853
Pnpla6	2.41E-58	5.22E-33	-0.113053	57.617725	-0.0640191	32.281932
Apoc2	3.42E-66	1	-0.113462	65.465444	0.0258555	0
Wbp1l	1.89E-33	7.55E-09	-0.11415	32.72329	-0.042761	8.1219781
Arhgef7	1.78E-42	1.76E-13	-0.114354	41.74843	0.0672839	12.754245
Nck1	6.04E-48	7.79E-40	-0.114464	47.219277	-0.0790023	39.108412
Impa1	1.83E-54	3.63E-50	-0.114513	53.737076	-0.080878	49.44033
Hbs1l	6.57E-40	1.47E-13	-0.114822	39.182354	-0.052555	12.83179
Atl3	1.69E-33	8.92E-15	-0.115028	32.771011	-0.0589346	14.0495
Lrrc28	5.80E-32	4.81E-14	-0.115411	31.236351	-0.0579878	13.317534

Gpam	7.27E-34	2.41E-14	-0.116207	33.138693	-0.0494063	13.617824
Foxa3	4.29E-53	6.55E-52	-0.116453	52.367849	-0.0836843	51.183452
Mocs2	1.30E-56	1.17E-22	-0.116705	55.885166	-0.0566499	21.930368
Platr22	2.44E-53	0.00014	-0.117308	52.612694	-0.027352	3.8540116
Gm16157	1.77E-62	1.01E-53	-0.117718	61.750977	-0.0805612	52.994919
Agmat	4.36E-44	1	-0.118443	43.36016	-0.0006225	0
Gm12910	7.37E-121	0	-0.119081	120.13281	-0.1326487	#NUM!
Tmem131l	9.59E-32	0	-0.119342	31.018325	-0.2991521	#NUM!
Ubc	1.03E-47	2.71E-06	-0.119367	46.988028	-0.0368079	5.5665168
Fbxo34	2.89E-38	4.23E-07	-0.120528	37.538601	-0.0428102	6.3731823
Smc1a	8.54E-54	5.74E-21	-0.120923	53.068307	-0.0584997	20.240982
Otulin	6.61E-46	1E-266	-0.120997	45.179789	-0.2263181	265.99138
Sarnp	5.72E-55	2.86E-52	-0.121103	54.24275	-0.0880295	51.544131
Prkacb	2.33E-62	2.38E-50	-0.121118	61.632475	-0.0810062	49.623938
Ei24	9.53E-48	2.8E-40	-0.121443	47.02086	-0.0816301	39.552138
Inpp5f	2.16E-58	1.34E-39	-0.121485	57.665729	-0.0748012	38.872072
Ergic2	2.50E-52	1	-0.121505	51.60264	0.0090909	0
Gpt2	3.11E-27	6.65E-11	-0.121748	26.507599	-0.0557802	10.177352
Angptl4	1.84E-54	4.32E-10	-0.122481	53.735088	-0.0424018	9.364833
Aldh3a2	6.86E-34	0.110155	-0.122637	33.163406	-0.0417503	0.9579968
Larp4b	3.89E-42	4.2E-194	-0.123527	41.410569	-0.2124799	193.38062
Mapk14	1.24E-39	1.7E-206	-0.124047	38.906715	-0.2043427	205.768
Nfix	4.69E-39	6.49E-06	-0.124092	38.328547	-0.037876	5.1880736
Lhpp	9.82E-33	1.53E-08	-0.124671	32.007898	-0.026845	7.816653
Hs3st3b1	2.07E-77	1.75E-17	-0.125896	76.684806	-0.0441999	16.756046
Helz2	4.64E-84	7.7E-110	-0.126002	83.333623	-0.1055649	109.11315
Pan3	2.85E-51	1.6E-274	-0.126306	50.544444	-0.2456196	273.78944
Rtf2	2.84E-61	7.29E-49	-0.126353	60.546576	-0.0841766	48.1375
Pten	1.73E-41	1.3E-223	-0.126354	40.761432	-0.219987	222.87183
Zscan26	7.30E-55	1	-0.126443	54.136748	-0.0207592	0
Paxbp1	3.83E-58	5.91E-47	-0.127015	57.416961	-0.0885847	46.228716
Syne1	2.83E-38	5.6E-198	-0.127158	37.547987	-0.2194234	197.25093
Aff4	3.97E-44	2.4E-230	-0.127414	43.40098	-0.2190985	229.61521
Cxadr	6.20E-45	1.04E-38	-0.127788	44.207526	-0.0805267	37.984033
Neo1	2.45E-59	1.6E-30	-0.128546	58.610348	-0.0690846	29.794776
Eci1	1.08E-72	7.71E-79	-0.129978	71.965637	-0.0973186	78.113099
Umad1	3.28E-52	3.01E-34	-0.130247	51.483528	-0.0809961	33.521721
Rnf111	9.68E-47	1.5E-252	-0.130411	46.014084	-0.2242063	251.82349
Upf2	1.52E-50	1.12E-20	-0.130523	49.81737	-0.0648855	19.949049
Amy1	3.75E-61	3.26E-48	-0.130571	60.42617	-0.0864858	47.486976
Mbtd1	1.37E-44	3.4E-167	-0.130663	43.862732	-0.1841678	166.46977
Brap	3.39E-38	5.56E-18	-0.13079	37.470048	-0.0663969	17.254774
Gm31583	2.94E-122	0	-0.130827	121.53127	-0.1395497	#NUM!
Tprkb	1.33E-75	2.79E-71	-0.130933	74.877182	-0.0922301	70.554404
Baiap2	4.54E-49	1.63E-74	-0.130961	48.342782	0.1437586	73.787659
Amfr	1.43E-59	5.25E-23	-0.131119	58.845012	-0.0635042	22.280095
Ppp1r13b	2.60E-70	3.53E-69	-0.131311	69.585533	-0.0969965	68.45207

Whamm	4.47E-75	8.14E-50	-0.131574	74.34988	-0.0787751	49.089285
Cntrl	6.75E-62	7.16E-54	-0.131965	61.170546	-0.0926941	53.145033
Camta2	1.92E-81	1.7E-62	-0.132285	80.716347	-0.0867097	61.770697
Enox2	4.40E-39	4.5E-191	-0.13267	38.356975	-0.2156474	190.34435
Cyp2d10	1.11E-61	1	-0.132682	60.954538	-0.0057505	0
Sfxn2	6.66E-69	3.36E-73	-0.133375	68.176709	-0.1012074	72.473481
Amacr	3.43E-85	2.16E-89	-0.133442	84.464592	-0.0992182	88.666445
Khdrbs3	2.73E-80	1.71E-49	-0.134047	79.563139	-0.0781381	48.767166
Lyst	1.19E-61	0	-0.134231	60.923929	-0.3111392	#NUM!
Cda	3.00E-66	1.74E-54	-0.134493	65.522853	-0.0892263	53.760223
Magt1	1.14E-61	1.16E-21	-0.13458	60.943771	-0.0600643	20.933909
0610005C1	3.46E-47	1	-0.134901	46.460513	0.0089842	0
Gm31121	6.97E-118	0	-0.135128	117.15651	-0.1393345	#NUM!
Rnf144b	3.54E-32	8.99E-08	-0.13531	31.450746	-0.0550168	7.0461563
Proser2	2.80E-68	6.91E-73	-0.135332	67.552957	-0.1022283	72.160289
Slc27a5	1.17E-97	2.9E-101	-0.135627	96.932408	-0.0993786	100.53106
Bcl9l	3.22E-68	1.74E-58	-0.135711	67.491708	-0.0916295	57.758644
Scarb2	1.50E-43	0	-0.135783	42.823648	-0.294355	#NUM!
Hp1bp3	8.97E-67	3.46E-27	-0.135931	66.047258	-0.0662164	26.460958
Timd2	2.11E-71	1.52E-47	-0.136187	70.676216	-0.0826079	46.819097
Rufy3	1.38E-55	0	-0.136675	54.859137	-0.3145746	#NUM!
Fam13b	8.13E-54	2.1E-214	-0.136742	53.090081	-0.2009238	213.68189
Arhgef18	1.74E-57	5.37E-43	-0.136847	56.758598	-0.0921124	42.269978
Mgam	2.56E-72	4.51E-27	-0.136866	71.592125	-0.0599569	26.346146
Decr2	2.43E-66	1.65E-35	-0.136987	65.613689	-0.0757927	34.781229
Canx	8.05E-56	4.93E-36	-0.137027	55.094441	-0.0801427	35.307407
Lgals8	1.26E-53	7.01E-20	-0.137296	52.898453	-0.0652609	19.154576
Hsp90b1	2.05E-48	1	-0.13747	47.68826	0.0231744	0
Gcdh	7.56E-69	1	-0.137848	68.121719	0.0010492	0
Megf9	4.64E-34	1	-0.138254	33.333692	-0.0255177	0
Lypla1	6.93E-57	6.55E-51	-0.138346	56.159092	-0.0973796	50.183961
Ube2i	3.07E-70	7.66E-64	-0.138415	69.513124	-0.0973412	63.115765
Zhx2	3.83E-44	5.1E-154	-0.138504	43.416453	-0.1866633	153.29095
Mgmt	1.78E-36	0.028761	-0.13863	35.748896	-0.0303291	1.5411934
Sap130	2.91E-66	1.15E-43	-0.138965	65.536432	-0.0835491	42.939336
Cacna1d	5.74E-121	0	-0.139078	120.24138	-0.173078	#NUM!
Ralgps1	1.13E-70	2.72E-56	-0.139137	69.945256	-0.0899728	55.564657
Raph1	1.38E-48	1.3E-169	-0.139289	47.86125	-0.2059439	168.87691
Klf15	1.86E-59	4.63E-40	-0.139361	58.730595	-0.0868386	39.334511
Abl1	4.77E-54	1.07E-37	-0.139385	53.321319	-0.0862361	36.971708
Fbrsl1	3.80E-61	1	-0.139545	60.419975	-0.0079886	0
4833411C0	4.32E-65	3.69E-15	-0.140372	64.364618	-0.0508788	14.433133
Tnpo3	1.20E-67	1.33E-74	-0.140386	66.92189	-0.1106501	73.876385
Atf5	3.70E-67	1.54E-40	-0.140813	66.432025	-0.0781854	39.813394
Clu	1.95E-76	4.4E-300	-0.141679	75.710413	-0.1946253	299.35374
Ugt2b1	1.38E-64	4.4E-242	-0.141979	63.860816	-0.199694	241.35196
Nfe2l1	7.85E-73	1.91E-77	-0.142763	72.10497	-0.1075253	76.717954

Bmpr1a	2.77E-58	1.05E-53	-0.143373	57.557394	-0.1024006	52.980561
Ero1lb	3.53E-63	1.46E-39	-0.143424	62.452486	-0.0853761	38.83604
Lclat1	6.08E-79	5.12E-68	-0.143526	78.215833	-0.0971318	67.290733
Ldlr	1.54E-45	0.022857	-0.143594	44.813208	0.0460308	1.640973
Atp8b1	6.24E-63	3.61E-34	-0.143643	62.204893	-0.0744555	33.442081
Sar1b	9.98E-63	7.46E-40	-0.143778	62.000989	-0.0870888	39.127297
Ccdc50	1.79E-61	2.25E-42	-0.143957	60.747356	-0.0888241	41.647367
Baiap2l1	2.34E-45	0.723626	-0.144255	44.630179	-0.0245896	0.1404861
Zfp976	2.00E-95	1.35E-74	-0.144712	94.699886	-0.0938628	73.870565
Atg5	2.09E-64	2.06E-33	-0.144718	63.678924	-0.0779009	32.686534
Mipol1	9.33E-68	1.27E-30	-0.144798	67.030311	-0.073794	29.89762
Mpc2	1.50E-65	5.63E-71	-0.144842	64.825079	-0.1112041	70.249707
Mafg	5.62E-50	3E-188	-0.1452	49.250055	-0.2021198	187.52191
Fermt2	4.13E-53	1.51E-26	-0.145599	52.383903	-0.0765881	25.821403
Hs6st1	8.24E-58	8.1E-273	-0.146235	57.084331	-0.2232567	272.08949
Trp53inp1	3.01E-49	9.78E-10	-0.146325	48.521617	-0.0449707	9.0097179
Pkp2	4.20E-73	2.36E-06	-0.146459	72.377055	-0.0371166	5.6267139
Hsd17b2	2.37E-29	2.82E-66	-0.146793	28.625888	0.1834237	65.549601
Upf3b	1.86E-75	8.19E-53	-0.147147	74.731163	-0.092649	52.086707
Insig1	9.31E-108	7.79E-25	-0.147363	107.03098	-0.049353	24.108286
Chka	6.56E-36	0.000322	-0.147677	35.182828	0.0555021	3.4922653
4930404H1	1.84E-83	1.62E-18	-0.14877	82.735483	-0.0488308	17.789749
Asap3	4.14E-123	7.1E-186	-0.148781	122.38254	-0.1260972	185.14865
Nnmt	5.40E-108	3.8E-158	-0.148948	107.26798	-0.124849	157.42358
Lbp	1.31E-101	1.2E-129	-0.148962	100.88363	-0.1178443	128.93112
Prkd3	2.36E-54	5E-10	-0.149401	53.626481	-0.0437871	9.3013028
Irs1	4.28E-59	2.18E-42	-0.150989	58.369007	-0.0923851	41.661812
Vkorc1l1	1.31E-71	1.67E-80	-0.151164	70.881274	-0.1186441	79.776179
B4galt1	3.91E-62	3.85E-64	-0.151359	61.407701	-0.1170696	63.415058
Hsp90ab1	2.95E-69	2.66E-71	-0.154574	68.53032	-0.1168954	70.574849
Slc17a8	8.68E-80	5.68E-58	-0.155127	79.061365	-0.0921925	57.246015
Tenm3	7.45E-14	1.9E-154	-0.156329	13.127938	0.3056865	153.72706
Ndfip1	3.39E-60	7.4E-257	-0.156615	59.469303	-0.2288811	256.13054
Galm	4.88E-56	1.55E-38	-0.157198	55.311794	-0.0959068	37.810991
Slc23a1	1.21E-115	1.19E-28	-0.1574	114.91809	-0.0563128	27.924639
Sh3bgrl	9.04E-73	4E-36	-0.158024	72.043775	-0.082575	35.398104
Dhrs3	4.00E-53	0	-0.158576	52.397954	-0.279947	#NUM!
Mettl7b	2.44E-87	3.8E-67	-0.158808	86.612342	-0.1011318	66.420494
Cyp4f17	9.06E-97	4.7E-113	-0.159084	96.042836	-0.1249746	112.32442
Serpina1b	2.48E-80	1.18E-13	-0.159611	79.605064	0.0854161	12.927599
Gm40438	1.47E-64	1.83E-60	-0.159738	63.832098	-0.1069142	59.738042
Tfpi2	5.86E-63	1	-0.159744	62.231999	-0.0034586	0
44261	7.92E-73	1.15E-53	-0.159835	72.101231	-0.1004347	52.940757
Slco2a1	5.42E-99	6.09E-81	-0.15993	98.265629	-0.1053878	80.215585
Apol7a	8.17E-109	2.1E-125	-0.160064	108.0878	-0.1238209	124.67168
Sdc4	8.17E-56	2.83E-84	-0.160445	55.087773	0.1767367	83.547578
Rhot1	9.52E-74	2.9E-74	-0.160605	73.021335	-0.1162633	73.537483

Rtn3	1.87E-61	2.1E-206	-0.160667	60.728153	-0.2276084	205.67066
Hmgcs1	4.95E-122	1	-0.160788	121.30501	0.0265888	0
Erp44	6.35E-92	1.4E-98	-0.161088	91.197469	-0.122548	97.854868
Shfl	1.04E-80	9.34E-16	-0.161491	79.982864	-0.0523942	15.029534
Slco1a4	3.00E-55	2.4E-295	-0.162066	54.523549	0.3939729	294.61092
Usp3	3.59E-69	0	-0.162281	68.444471	-0.2583477	#NUM!
Fxr1	2.41E-84	8.62E-62	-0.162795	83.617432	-0.1040153	61.064417
Cyp2d22	1.08E-82	1	-0.163801	81.968281	0.0015502	0
Trio	5.45E-68	1.85E-37	-0.164486	67.263899	-0.0926627	36.733068
Nfil3	2.52E-123	2.5E-130	-0.164698	122.59927	-0.1221134	129.60611
Gse1	4.38E-80	2.03E-93	-0.165211	79.358661	-0.1276512	92.691641
Phactr4	9.00E-71	6.5E-282	-0.166569	70.045856	-0.2446023	281.18961
Rreb1	1.85E-72	0	-0.167025	71.732561	-0.2925217	#NUM!
Tmem25	2.12E-181	0	-0.167277	180.6736	-0.1511976	#NUM!
Lactb2	2.81E-76	5.97E-05	-0.167404	75.550713	-0.0339032	4.224239
Mkln1	3.88E-82	6.1E-275	-0.167951	81.411003	-0.2460539	274.21245
Masp1	6.72E-64	1.71E-22	-0.168081	63.172924	-0.0702831	21.766438
Lpp	1.88E-71	6.42E-57	-0.16858	70.725523	0.1259188	56.19254
Gcnt2	3.16E-49	8.2E-205	-0.16877	48.500896	-0.2504771	204.08756
Slc25a20	8.35E-65	1.26E-23	-0.170021	64.078131	-0.0736512	22.900614
Smg6	5.60E-66	0	-0.170126	65.251607	-0.3210478	#NUM!
Gpt	3.25E-146	9.8E-205	-0.170287	145.48766	-0.1420263	204.00842
Pbx1	3.03E-57	2E-231	-0.170322	56.518337	-0.2500445	230.69673
Slc45a3	4.53E-134	1E-205	-0.17246	133.34405	-0.1505841	205.00061
Pls3	1.44E-101	7.8E-134	-0.172772	100.84126	-0.1441194	133.11023
Cep85l	3.73E-56	1.25E-19	-0.172802	55.427884	-0.0702519	18.901645
Gm12909	6.73E-153	0	-0.173146	152.17217	-0.2123291	#NUM!
Pon1	7.78E-74	1	-0.173705	73.109063	0.013839	0
Hipk2	2.23E-69	6E-248	-0.17378	68.651033	-0.2602284	247.22346
Acot12	2.74E-75	2.6E-256	-0.174674	74.561744	-0.2485935	255.59289
Bbox1	1.23E-84	6.75E-42	-0.174848	83.910771	-0.0911221	41.170377
Adcy9	8.71E-52	0	-0.176039	51.060183	-0.3642404	#NUM!
Gm15261	1.28E-129	0	-0.176885	128.89449	-0.1995379	#NUM!
Bhmt2	7.18E-93	1	-0.178607	92.143877	-0.0138733	0
Znrf1	3.08E-78	0	-0.178863	77.511452	-0.3659811	#NUM!
Klb	8.45E-151	5.57E-66	-0.179529	150.07289	-0.0874796	65.254013
Als2	1.01E-93	1.41E-54	-0.179903	92.995953	-0.101932	53.849792
Gch1	1.75E-57	7.38E-07	-0.180005	56.757687	-0.058695	6.1319651
Fer	1.03E-114	7.6E-157	-0.180243	113.98902	-0.1531274	156.12081
Fbf1	2.17E-146	2E-201	-0.180408	145.66349	-0.150424	200.70434
Tsc1	2.57E-119	1.1E-137	-0.180552	118.59011	-0.140001	136.97447
Peli2	6.57E-76	4.47E-43	-0.180668	75.182754	-0.1004372	42.349735
Rdh16f2	5.70E-85	0.003107	-0.180978	84.244441	-0.0145985	2.507617
Adap2	1.88E-109	0	-0.181093	108.72636	-0.3028153	#NUM!
Spop	8.08E-84	0	-0.181313	83.092819	-0.3147322	#NUM!
Acadsb	1.27E-122	1.5E-160	-0.182164	121.89592	-0.149026	159.82258
Akr1c20	9.72E-158	2.2E-218	-0.18304	157.01236	-0.1519918	217.65914

Gm47719	6.50E-116	0	-0.183258	115.18726	-0.2255502	#NUM!
Ube2r2	2.06E-80	4.1E-253	-0.183554	79.68538	-0.2410742	252.38827
Pla1a	1.12E-179	6.8E-284	-0.183697	178.94938	-0.1597483	283.16753
Ppp2r5e	7.31E-70	8.6E-216	-0.183779	69.136346	-0.2496938	215.06791
Masp2	1.94E-103	1	-0.18429	102.71318	-0.015608	0
Sh3d19	4.09E-62	3.2E-276	-0.184357	61.387772	-0.2787097	275.49349
Arhgef19	2.17E-164	7.9E-188	-0.185099	163.6628	-0.1400548	187.10034
Znrf3	4.14E-79	2E-223	-0.186904	78.383459	-0.2338145	222.68959
Scap	1.16E-125	4.9E-115	-0.186986	124.93437	-0.1315553	114.30544
Ceacam1	7.22E-148	1.3E-152	-0.187302	147.14135	-0.1376841	151.87436
Brd4	4.77E-102	0	-0.188312	101.3214	-0.2978694	#NUM!
Txndc11	2.21E-80	2.27E-29	-0.188382	79.656316	-0.0871337	28.643754
Nectin1	4.98E-104	9.4E-116	-0.188565	103.30268	-0.1426116	115.02527
Dennd5b	5.02E-82	1	-0.189361	81.299459	-0.0060323	0
Nav2	1.98E-77	6.27E-11	-0.190629	76.703406	-0.0411214	10.202453
F11	4.16E-84	0.00109	-0.190638	83.380727	-0.0298263	2.9627522
Dcaf11	3.41E-121	2.33E-64	-0.191145	120.4675	-0.1023166	63.63213
Irf2	1.44E-90	0	-0.193472	89.840171	-0.3224809	#NUM!
4932438A1	4.16E-106	0	-0.193936	105.38114	-0.2832457	#NUM!
Gm15622	1.11E-181	0	-0.194155	180.95294	-0.1804796	#NUM!
Atl2	1.95E-99	1.72E-51	-0.195381	98.711018	-0.104015	50.763419
Rnase4	2.18E-113	5.91E-17	-0.196931	112.66241	-0.0625697	16.228341
Pter	9.80E-106	8.4E-108	-0.197202	105.00893	-0.1451776	107.07476
St6gal1	4.77E-142	0	-0.19894	141.32143	-0.2484073	#NUM!
Pemt	1.02E-96	1	-0.199877	95.989971	0.0213659	0
Ctif	7.26E-91	2.45E-79	-0.199967	90.139257	-0.1441332	78.610003
Rnf125	3.38E-38	1.78E-35	-0.200676	37.470688	-0.1392806	34.75038
Sned1	2.39E-180	1.1E-262	-0.200734	179.62161	-0.1694523	261.94257
Nfat5	1.20E-101	0	-0.200747	100.91936	-0.2603258	#NUM!
Car5a	1.78E-137	1.8E-112	-0.200969	136.75021	-0.1285586	111.74285
Scarf1	1.89E-170	1.1E-174	-0.20236	169.72343	-0.1462247	173.97439
Slc35e2	2.62E-127	1.79E-63	-0.2025	126.58245	-0.1061891	62.746774
Epb41l4b	7.76E-137	1.1E-128	-0.202764	136.11001	-0.1418914	127.94234
Hp	1.32E-150	0	-0.203501	149.87793	-0.2539214	#NUM!
Gm32461	8.48E-168	8.9E-243	-0.204419	167.07163	-0.1682496	242.04878
Mttp	6.06E-112	2.29E-76	-0.204862	111.21779	-0.1245207	75.639802
Nfyc	1.35E-120	1.6E-144	-0.204872	119.87037	-0.1627644	143.80894
Mthfd1	5.30E-103	4.07E-32	-0.204887	102.27539	-0.0850186	31.390529
Cyp27a1	2.07E-66	1	-0.206374	65.682998	-0.0075213	0
Aldh7a1	1.21E-116	2.1E-105	-0.206525	115.9155	-0.1435168	104.67706
Man2a1	8.67E-72	0	-0.208009	71.062211	-0.3434944	#NUM!
Aldh6a1	2.37E-151	2.2E-164	-0.208042	150.62439	-0.1578694	163.66021
Arhgef3	2.26E-91	2.44E-18	-0.208675	90.646621	-0.0660347	17.61298
Apoa5	8.05E-94	5.47E-14	-0.209507	93.093972	-0.0617685	13.261901
1700028E1	1.27E-163	0	-0.209588	162.8979	-0.2521712	#NUM!
Agxt2	5.18E-97	1	-0.209739	96.285912	-0.0009409	0
Wsb1	4.83E-124	0	-0.209765	123.31628	-0.26791	#NUM!

4933406I1:	2.56E-171	7.78E-76	-0.210517	170.59115	-0.0978945	75.10882
Fga	7.57E-94	6.68E-07	-0.210627	93.121176	-0.0299614	6.175202
Entpd8	1.94E-202	1.2E-265	-0.211541	201.71299	-0.1711144	264.93681
Supt3	9.20E-128	3.3E-159	-0.211797	127.036	-0.1745857	158.48602
Ube2g1	4.29E-104	0	-0.211937	103.36755	-0.3101933	#NUM!
Arhgap35	5.28E-124	1.93E-82	-0.213481	123.27744	-0.126576	81.714128
Map3k5	8.42E-64	0	-0.213702	63.074457	-0.379509	#NUM!
Aldh8a1	1.44E-121	6.69E-10	-0.213904	120.84291	-0.0471123	9.1746817
Cpt1a	5.34E-98	0	-0.214557	97.272151	-0.3508014	#NUM!
Apol9b	7.27E-221	0	-0.214616	220.13834	-0.228042	#NUM!
Nr3c1	1.54E-112	0	-0.215235	111.81139	-0.3075806	#NUM!
Peg3	2.00E-207	0	-0.215427	206.69805	-0.1887425	#NUM!
Gm34667	1.04E-51	1	-0.215907	50.983706	-0.0385835	0
Coq8a	1.42E-146	4.6E-207	-0.216144	145.84733	-0.1834829	206.34198
Ppp3ca	5.49E-100	0	-0.217251	99.260699	-0.3306677	#NUM!
Zpr1	3.13E-110	3.7E-120	-0.217265	109.50383	-0.1677269	119.43588
Ldlrad4	1.97E-85	1.2E-57	-0.217811	84.705121	-0.1408666	56.91938
Fam214a	1.05E-48	0.001026	-0.217961	47.97779	-0.0656704	2.9888923
Usf3	3.10E-143	1E-149	-0.218877	142.50839	-0.1630718	148.98996
Btbd9	2.66E-110	0	-0.219032	109.5758	-0.3294925	#NUM!
Serpina1a	2.17E-142	0.000761	-0.219401	141.66435	-0.0221721	3.1184268
Rabgef1	1.61E-147	4.4E-176	-0.220621	146.79419	-0.1733839	175.35416
Pros1	1.60E-218	0	-0.221264	217.79496	-0.1960607	#NUM!
Habp2	1.52E-201	3.9E-233	-0.221446	200.81889	-0.1689435	232.41265
Zbtb7c	1.42E-183	0	-0.222229	182.84637	-0.2404811	#NUM!
Prox1	4.68E-89	1.27E-41	-0.222353	88.329925	-0.1082688	40.896701
Adnp	4.58E-157	1.6E-201	-0.222415	156.33955	-0.1835136	200.78429
Luc7l3	3.52E-135	0	-0.223076	134.45393	-0.2913634	#NUM!
Synj2bp	1.18E-164	2.6E-204	-0.223398	163.92839	-0.1802467	203.58828
Ncoa1	3.71E-124	0	-0.224311	123.43016	-0.3128518	#NUM!
Rad23b	1.32E-142	3.8E-194	-0.224565	141.87909	-0.1882138	193.42354
Fgd6	1.33E-123	0	-0.226288	122.87552	-0.3223865	#NUM!
Erbb4	2.39E-49	0	-0.226776	48.622021	-0.387911	#NUM!
Zfand3	3.00E-148	0	-0.229727	147.52328	-0.3375611	#NUM!
Bckdhb	6.61E-118	3E-17	-0.230547	117.17991	-0.0638574	16.522803
Fam193b	1.75E-149	2E-178	-0.231975	148.75717	-0.1834514	177.68904
Pabpn1	1.08E-134	1.07E-78	-0.232063	133.96745	-0.1322713	77.972431
Phf21a	2.33E-124	0	-0.232077	123.6333	-0.3606969	#NUM!
Creb3l2	4.32E-162	4E-144	-0.233544	161.36463	-0.1557254	143.40328
Smurf2	2.25E-136	0	-0.234134	135.64728	-0.2954052	#NUM!
B630019A1	9.26E-101	2.9E-112	-0.234311	100.03359	-0.1796486	111.53741
Klhl3	3.07E-174	1.8E-127	-0.234334	173.51331	-0.1424944	126.73323
Ces1d	7.73E-173	0	-0.234584	172.11177	-0.2655497	#NUM!
Arhgef12	3.78E-156	0	-0.234693	155.4224	-0.3390186	#NUM!
Crp	5.63E-58	4.01E-17	-0.237027	57.249168	-0.1252572	16.397368
Syt1	7.29E-170	0	-0.23783	169.13727	-0.2806424	#NUM!
Faah	3.20E-163	1.2E-107	-0.239055	162.49493	-0.1399707	106.90516

A1cf	9.92E-99	1	-0.239331	98.00339	-0.0043637	0
Hagh	1.29E-164	8.2E-232	-0.239609	163.88828	-0.2040764	231.0874
Agt	3.76E-249	0	-0.239721	248.42506	-0.214329	#NUM!
Utrn	1.65E-138	0	-0.239958	137.78237	-0.333576	#NUM!
Proz	1.70E-202	1.5E-277	-0.240394	201.76891	-0.1990169	276.82907
Aadac	7.70E-241	0	-0.241286	240.11341	-0.2136084	#NUM!
Slc25a15	5.57E-141	8.61E-58	-0.242226	140.25402	-0.1133514	57.065166
Them4	3.48E-208	0	-0.243888	207.45782	-0.2112939	#NUM!
Fpgs	8.59E-113	6.56E-48	-0.244373	112.06611	-0.1236057	47.183295
Ahsg	9.37E-78	2.8E-209	-0.24466	77.02848	0.3006935	208.56006
Itih4	9.87E-172	0	-0.245562	171.00576	-0.3000701	#NUM!
Arg1	7.50E-46	3.03E-20	-0.245639	45.124681	0.0676556	19.518062
Albfm1	2.33E-108	0.00037	-0.245753	107.63339	-0.0444847	3.4321895
Cyp2c67	2.12E-102	2.02E-32	-0.247071	101.67416	-0.1185795	31.695012
Slc10a1	2.45E-196	1.61E-91	-0.248652	195.61042	-0.1277278	90.792354
Gpr39	2.13E-171	1.8E-193	-0.248855	170.67164	-0.1925689	192.73901
Arhgap26	1.03E-162	0	-0.248982	161.98894	-0.3260446	#NUM!
Fign	2.21E-145	4.3E-192	-0.249074	144.65652	-0.2070527	191.36417
Prpf4b	1.15E-163	5E-79	-0.250093	162.94039	-0.1260796	78.300966
Foxp1	1.41E-128	0	-0.250457	127.84985	-0.3279896	#NUM!
Cat	1.70E-115	3.44E-24	-0.251286	114.76959	-0.0863679	23.46338
Tat	2.97E-56	2.2E-24	-0.251333	55.527507	0.1466828	23.658357
Sc5d	6.72E-234	1.3E-187	-0.252315	233.17273	-0.1526965	186.8722
Abcd3	1.06E-149	0	-0.252372	148.97347	-0.3270354	#NUM!
Ahcy	2.07E-195	2.4E-148	-0.253154	194.68384	-0.15996	147.62163
Dhcr24	5.89E-183	1.01E-36	-0.253608	182.23016	-0.0778864	35.995101
Adra1b	2.31E-116	0	-0.255509	115.63718	-0.354081	#NUM!
Celf1	1.71E-165	0	-0.255605	164.76813	-0.3718677	#NUM!
4930402H2	5.74E-140	0	-0.256766	139.24127	-0.3128023	#NUM!
Cadm1	2.90E-128	0	-0.257831	127.53817	-0.4578665	#NUM!
Tesk2	4.25E-168	3.2E-144	-0.258034	167.37165	-0.1743391	143.50009
Ripor2	7.94E-181	0	-0.258731	180.09996	-0.310196	#NUM!
Pik3c2g	2.73E-97	8.16E-10	-0.258789	96.564321	-0.0807452	9.0883284
Stra6l	7.04E-174	2.8E-217	-0.259276	173.1524	-0.2122594	216.55774
Inca1	1.48E-201	2.7E-224	-0.259495	200.82836	-0.1972297	223.57248
Tnrc6b	5.37E-182	0	-0.25985	181.26999	-0.4490166	#NUM!
Gm11342	9.51E-279	0	-0.260235	278.02165	-0.2788053	#NUM!
Cyp2c70	8.24E-195	1.1E-213	-0.260612	194.08389	-0.1947159	212.94437
Arhgap29	1.39E-181	4.4E-128	-0.260783	180.85733	-0.1599252	127.35469
Cdc14b	9.92E-159	3.1E-229	-0.261827	158.00344	-0.2264807	228.50793
Fam222b	1.83E-164	0	-0.264778	163.73797	-0.349531	#NUM!
Sox6	1.53E-169	2.5E-184	-0.266094	168.81412	-0.2069443	183.59591
Egfros	4.11E-258	0	-0.266838	257.38635	-0.3123302	#NUM!
Sirt3	1.89E-190	2.6E-258	-0.267058	189.72272	-0.2222959	257.58132
Luc7l2	8.59E-199	8.2E-158	-0.267064	198.06583	-0.1700035	157.08367
Hivep2	6.85E-143	0	-0.267687	142.16434	-0.3623755	#NUM!
Mfsd2a	1.23E-148	3.8E-121	-0.267987	147.91144	-0.184886	120.42279

Hsd17b12	2.22E-199	9E-101	-0.269142	198.65275	-0.1412092	100.04738
Large1	8.68E-186	0	-0.269243	185.06142	-0.3248107	#NUM!
Fam169b	8.41E-157	4.6E-107	-0.269815	156.07504	-0.1643637	106.34034
Arhgap5	1.31E-151	0	-0.270782	150.88308	-0.3925193	#NUM!
Ldah	1.27E-173	1.8E-204	-0.271851	172.89663	-0.2110781	203.73868
Lurap1l	7.88E-132	0	-0.272662	131.10335	-0.4382186	#NUM!
Lrp1	9.65E-165	0	-0.273886	164.01567	-0.5095184	#NUM!
Mrpl18	1.43E-212	4.06E-65	-0.275326	211.84324	-0.1112506	64.391396
Baat	1.08E-248	0	-0.275726	247.96742	-0.2312943	#NUM!
Slc25a42	3.86E-165	0	-0.277284	164.41336	-0.3434891	#NUM!
Zfp395	3.82E-216	0	-0.277455	215.41842	-0.2389806	#NUM!
Atp11c	2.25E-121	3.8E-128	-0.277765	120.64851	-0.1994331	127.42451
Palld	1.98E-163	0	-0.278073	162.70293	-0.3556985	#NUM!
Tdo2	1.46E-70	0.000155	-0.278508	69.835861	0.0542646	3.8091979
Gm48099	2.63E-105	0	-0.278722	104.5807	-0.6552058	#NUM!
Tmem219	1.75E-161	0	-0.280916	160.75732	-0.3355696	#NUM!
Slc30a10	0.00E+00	0	-0.281646	#NUM!	-0.290424	#NUM!
Gna12	2.38E-154	0	-0.281897	153.62326	-0.4157704	#NUM!
Nars2	1.66E-186	4.1E-239	-0.282183	185.7803	-0.2297094	238.38997
Myo1e	8.09E-98	0	-0.282651	97.092127	-0.4952113	#NUM!
Larp1b	1.28E-172	3.62E-70	-0.282755	171.89285	-0.1324602	69.4415
Golgb1	2.09E-189	1.8E-220	-0.282865	188.67997	-0.2239824	219.73546
Agtr1a	2.54E-212	6.6E-264	-0.283076	211.59596	-0.228091	263.17718
Hook3	7.10E-204	0	-0.28314	203.14861	-0.377378	#NUM!
Epas1	6.18E-158	0	-0.283837	157.20918	-0.3547767	#NUM!
Agmo	6.28E-129	1.4E-143	-0.285469	128.20238	-0.216196	142.8644
Gramd3	3.68E-141	0	-0.287403	140.43428	-0.3678906	#NUM!
Itih1	3.44E-232	2.1E-172	-0.288376	231.46326	-0.1785867	171.68028
Rin2	2.99E-137	0	-0.288588	136.52436	-0.4060667	#NUM!
Serpinf2	5.93E-255	0	-0.28975	254.22695	-0.242811	#NUM!
Fah	5.62E-203	4.2E-136	-0.290074	202.25062	-0.1698907	135.37991
Cth	2.73E-83	8.15E-26	-0.290325	82.563734	-0.1297275	25.089092
Bckdha	7.20E-187	0	-0.290463	186.14263	-0.3575163	#NUM!
Kansl1	6.06E-179	0	-0.290897	178.21755	-0.4788666	#NUM!
Acss2	7.79E-212	3.3E-97	-0.291448	211.10838	-0.1271394	96.481193
Cdk8	5.49E-209	0	-0.29168	208.26077	-0.399236	#NUM!
Gm37494	4.20E-254	0	-0.293944	253.37651	-0.2612423	#NUM!
Gjb1	1.39E-202	2.1E-178	-0.293996	201.85798	-0.1978153	177.67085
Ypel2	5.01E-163	0	-0.294513	162.29986	-0.3522315	#NUM!
Vgll4	6.12E-169	0	-0.295137	168.2131	-0.3610284	#NUM!
Akr1d1	2.51E-244	1.54E-59	-0.295364	243.60096	-0.0911524	58.811585
St3gal3	2.85E-156	2.39E-89	-0.295571	155.54549	-0.1565509	88.621364
Fbxl17	3.78E-239	7.57E-90	-0.295573	238.42283	-0.1364992	89.121072
Gm42418	1.63E-209	0	-0.295911	208.78707	-0.5843065	#NUM!
Sardh	1.11E-287	0	-0.296332	286.95577	-0.2598595	#NUM!
Prkag2	8.11E-149	2E-141	-0.296445	148.09099	-0.2232285	140.70404
Serpina3m	0.00E+00	0	-0.296701	#NUM!	-0.2647086	#NUM!

Ugt2b5	4.05E-119	0	-0.297452	118.39231	-0.5009866	#NUM!
Cd1d1	4.02E-274	0	-0.298441	273.39548	-0.2584898	#NUM!
Kansl1l	5.53E-200	0	-0.299133	199.25741	-0.3994384	#NUM!
Pbld1	3.27E-178	1.55E-40	-0.299691	177.48583	-0.1113813	39.809446
Stat2	5.30E-238	0	-0.300491	237.27545	-0.2605669	#NUM!
Apoc1	9.13E-184	1.02E-86	-0.30084	183.03944	-0.1539561	85.990446
Kcnn2	1.98E-152	0	-0.302355	151.7033	-0.4018159	#NUM!
Pipox	4.33E-237	0	-0.302545	236.36377	-0.2544692	#NUM!
Rnf214	8.15E-226	0	-0.304142	225.08903	-0.2557486	#NUM!
Ddi2	3.23E-238	0	-0.304599	237.4907	-0.2490236	#NUM!
Hpd	4.37E-144	1.13E-20	-0.304961	143.35949	-0.0892592	19.947253
Pigr	0.00E+00	0	-0.305469	#NUM!	-0.3220364	#NUM!
Cyp2j9	0.00E+00	0	-0.307176	#NUM!	-0.2945807	#NUM!
Pxmp4	2.49E-205	4.8E-229	-0.30731	204.60327	-0.2282025	228.31554
C4b	7.10E-182	0	-0.307641	181.14851	-0.437362	#NUM!
Fmo5	4.49E-21	7.5E-08	-0.308213	20.347956	-0.1735459	7.12471
Snx29	1.01E-190	0	-0.310491	189.99554	-0.4768139	#NUM!
Cyp2d26	0.00E+00	0	-0.311624	#NUM!	-0.2698043	#NUM!
Cmss1	8.32E-200	0	-0.313368	199.07995	-0.4890074	#NUM!
Onecut2	7.75E-188	4.8E-239	-0.314237	187.11096	-0.2610746	238.31883
Ahdc1	8.34E-275	0	-0.314379	274.07891	-0.2621334	#NUM!
Garem1	9.43E-168	0	-0.315496	167.0256	-0.4457478	#NUM!
Fam107b	1.10E-176	0	-0.317727	175.96058	-0.3910592	#NUM!
Txlng	2.34E-226	0	-0.31783	225.63117	-0.4076373	#NUM!
Serpinc1	4.38E-188	3.93E-48	-0.318034	187.35876	-0.1372377	47.406
Atxn7l1	1.29E-213	0	-0.319671	212.89021	-0.4009135	#NUM!
Lgals9	2.66E-207	0	-0.320816	206.5749	-0.3989616	#NUM!
H2-Q10	1.14E-227	1.55E-22	-0.322077	226.94396	-0.0780767	21.810608
Fchsd2	1.01E-191	0	-0.32233	190.99546	-0.4403908	#NUM!
Ttc7b	9.69E-279	0	-0.323242	278.0138	-0.3904181	#NUM!
Scarb1	3.81E-183	0	-0.324002	182.4187	-0.4550041	#NUM!
Dpy19l1	6.89E-247	0	-0.327415	246.16184	-0.3724443	#NUM!
Dock4	1.61E-179	0	-0.330371	178.7926	-0.4522123	#NUM!
Ern1	2.76E-205	0	-0.330846	204.55928	-0.4286513	#NUM!
Ulk2	2.14E-233	0	-0.331262	232.66962	-0.4503498	#NUM!
Pcca	8.45E-255	3.2E-126	-0.334418	254.07301	-0.173887	125.49932
Bach2	2.03E-171	0	-0.335698	170.69169	-0.5720289	#NUM!
Slc6a13	0.00E+00	0	-0.335709	#NUM!	-0.2162473	#NUM!
Ppm1l	5.22E-262	1.7E-301	-0.335949	261.28267	-0.2586138	300.77946
Cbs	6.51E-243	2.3E-257	-0.336198	242.18628	-0.2521216	256.64114
Abcc2	6.43E-181	1	-0.338342	180.19169	0.0102927	0
Gpat4	9.35E-277	0	-0.338674	276.02934	-0.2995698	#NUM!
Svil	5.34E-216	0	-0.339459	215.27235	-0.4028362	#NUM!
0610043K1	0.00E+00	0	-0.342776	#NUM!	-0.3692964	#NUM!
Gc	2.13E-169	4.48E-21	-0.343165	168.67123	-0.1020235	20.349114
Gm31333	1.93E-292	4E-299	-0.344928	291.71425	-0.2503818	298.40163
Chpt1	1.17E-221	0	-0.345732	220.93245	-0.4291723	#NUM!

Fgb	4.21E-230	4.47E-37	-0.346032	229.3756	-0.0917291	36.350145
Dnajc12	7.41E-207	0	-0.34604	206.13038	-0.3316247	#NUM!
Dleu2	1.24E-210	0	-0.348637	209.90627	-0.5677683	#NUM!
Dgat2	9.85E-290	0	-0.348874	289.00653	-0.3052044	#NUM!
Qsox1	4.78E-285	0	-0.348965	284.32057	-0.2790402	#NUM!
Cyp2d9	4.44E-263	7.7E-271	-0.350295	262.35217	-0.2536437	270.11502
Gm4788	3.74E-266	0	-0.351398	265.42725	-0.4910671	#NUM!
Slc25a47	1.48E-302	0	-0.351608	301.82869	-0.3135822	#NUM!
Gck	0.00E+00	0	-0.355053	#NUM!	-0.3710331	#NUM!
Slc2a9	2.25E-294	0	-0.3562	293.647	-0.400557	#NUM!
Gckr	1.94E-289	4.2E-117	-0.357892	288.71195	-0.1674025	116.37907
Gstz1	0.00E+00	0	-0.358512	#NUM!	-0.2750045	#NUM!
Plcb1	1.84E-193	1.9E-195	-0.35916	192.7361	-0.2576	194.72226
Slc12a7	0.00E+00	0	-0.360276	#NUM!	-0.2911041	#NUM!
Atrnl1	7.14E-234	0	-0.361999	233.1464	-0.4428231	#NUM!
Fn1	6.95E-154	6.76E-53	-0.364814	153.1579	0.1752242	52.169982
Ftcd	6.96E-227	1.5E-174	-0.364992	226.15725	-0.2472868	173.82994
Chp1	1.38E-279	0	-0.3657	278.86106	-0.3006267	#NUM!
Pank1	8.98E-215	7.2E-234	-0.36574	214.04664	-0.2820027	233.14447
Gm36041	7.28E-126	0	-0.366186	125.13777	-0.5108756	#NUM!
Nrp1	1.83E-244	0	-0.366879	243.73794	-0.4678706	#NUM!
Slc17a3	0.00E+00	0	-0.367269	#NUM!	-0.3170398	#NUM!
Zfp385b	1.30E-236	0	-0.368454	235.88624	-0.4679674	#NUM!
Zfand6	1.23E-284	0	-0.368577	283.9109	-0.4931007	#NUM!
Gm49417	0.00E+00	0	-0.369054	#NUM!	-0.3345558	#NUM!
Suclg2	1.31E-299	0	-0.369147	298.88419	-0.459941	#NUM!
Osbpl8	0.00E+00	0	-0.370347	#NUM!	-0.5689742	#NUM!
Mir99ahg	7.17E-245	0	-0.37376	244.14422	-0.4293202	#NUM!
Ugt2b36	4.44E-236	0	-0.374774	235.35266	-0.5088578	#NUM!
Tmem56	2.29E-225	8.2E-217	-0.37516	224.63994	-0.2785578	216.08696
Tshz2	2.32E-207	0	-0.383434	206.63514	-0.6461557	#NUM!
Afm	0.00E+00	0	-0.384759	#NUM!	-0.3141978	#NUM!
Nipsnap1	0.00E+00	0	-0.385023	#NUM!	-0.3412141	#NUM!
Lima1	2.19E-289	0	-0.385091	288.65932	-0.4556356	#NUM!
Pdia5	6.82E-298	0	-0.387346	297.16629	-0.3384671	#NUM!
Acsm1	0.00E+00	0	-0.388332	#NUM!	-0.3190459	#NUM!
Atxn2	0.00E+00	0	-0.389266	#NUM!	-0.4932863	#NUM!
Mup20	0.00E+00	0	-0.389394	#NUM!	-0.4819321	#NUM!
Gk	0.00E+00	0	-0.389589	#NUM!	-0.319365	#NUM!
Gm3839	0.00E+00	0	-0.392381	#NUM!	-0.399464	#NUM!
Fgg	0.00E+00	1.8E-212	-0.393472	#NUM!	-0.2230505	211.74514
Gabbr2	0.00E+00	0	-0.394512	#NUM!	-0.3564544	#NUM!
Vegfa	0.00E+00	4E-295	-0.394973	#NUM!	-0.2561848	294.40266
Dpyd	2.81E-91	0	-0.396996	90.550808	0.3216613	#NUM!
Slc47a1	0.00E+00	0	-0.398566	#NUM!	-0.3218035	#NUM!
Pecr	0.00E+00	0	-0.399641	#NUM!	-0.3498314	#NUM!
Asl	1.60E-243	2.8E-207	-0.39988	242.79483	-0.2519195	206.54978

Ndrg2	0.00E+00	0	-0.403512	#NUM!	-0.2724089	#NUM!
Phlpp1	0.00E+00	0	-0.404941	#NUM!	-0.4837442	#NUM!
Gm4952	0.00E+00	1.2E-266	-0.407958	#NUM!	-0.2549997	265.92404
Ptprj	1.70E-276	0	-0.409835	275.76834	-0.6408465	#NUM!
Peak1	0.00E+00	0	-0.410125	#NUM!	-0.5800571	#NUM!
Akap13	0.00E+00	0	-0.410621	#NUM!	-0.509554	#NUM!
AI182371	8.51E-303	0	-0.412401	302.07021	-0.3065734	#NUM!
Maob	0.00E+00	0	-0.412856	#NUM!	-0.3484806	#NUM!
St3gal5	1.22E-170	0	-0.414125	169.91243	-0.5213702	#NUM!
Ehbp1	0.00E+00	0	-0.417463	#NUM!	-0.466184	#NUM!
Haao	0.00E+00	0	-0.419969	#NUM!	-0.3835352	#NUM!
Gm19951	4.01E-282	0	-0.427154	281.39687	-0.6408981	#NUM!
Evi5	0.00E+00	0	-0.42828	#NUM!	-0.3631592	#NUM!
Hoga1	0.00E+00	4E-299	-0.42874	#NUM!	-0.288512	298.4005
Acaa2	0.00E+00	0	-0.429867	#NUM!	-0.4011264	#NUM!
Arid5b	3.90E-302	0	-0.432959	301.40853	-0.6148382	#NUM!
Onecut1	0.00E+00	0	-0.43371	#NUM!	-0.4523608	#NUM!
Ephx2	0.00E+00	0	-0.435918	#NUM!	-0.5246221	#NUM!
Gm4756	0.00E+00	0	-0.437039	#NUM!	-0.4859042	#NUM!
Elovl2	0.00E+00	1E-293	-0.438796	#NUM!	-0.2880774	292.99861
Slc17a2	0.00E+00	0	-0.438889	#NUM!	-0.3672682	#NUM!
Shb	0.00E+00	0	-0.440923	#NUM!	-0.4779868	#NUM!
Xiap	0.00E+00	0	-0.440963	#NUM!	-0.5297089	#NUM!
Atxn1	0.00E+00	7.5E-197	-0.4416	#NUM!	-0.279461	196.12262
Pcyt2	0.00E+00	4.9E-117	-0.443442	#NUM!	-0.1803705	116.30999
Sorbs1	0.00E+00	0	-0.443443	#NUM!	-0.5276329	#NUM!
Zhx3	0.00E+00	0	-0.446425	#NUM!	-0.3647903	#NUM!
Mtus1	0.00E+00	0	-0.448814	#NUM!	-0.3171985	#NUM!
Vwa8	0.00E+00	0	-0.449117	#NUM!	-0.3355774	#NUM!
Ppm1k	0.00E+00	0	-0.449434	#NUM!	-0.40689	#NUM!
Slc4a4	5.70E-249	0	-0.453434	248.24392	-0.6084031	#NUM!
Bcl6	0.00E+00	0	-0.45818	#NUM!	-0.4062932	#NUM!
BC024386	0.00E+00	0	-0.458457	#NUM!	-0.4002788	#NUM!
Slc25a25	0.00E+00	0	-0.4596	#NUM!	-0.4225255	#NUM!
Fam20a	0.00E+00	0	-0.461332	#NUM!	-0.4141289	#NUM!
4732465J0	0.00E+00	0	-0.466393	#NUM!	-0.5778046	#NUM!
Fads2	0.00E+00	0	-0.466814	#NUM!	-0.3710422	#NUM!
Hnf4aos	2.10E-260	4.23E-39	-0.467077	259.67801	-0.1725883	38.373516
Slc16a10	6.84E-245	0	-0.468471	244.16489	-0.5434029	#NUM!
Enpp3	0.00E+00	0	-0.46897	#NUM!	-0.5079881	#NUM!
Prlr	2.51E-266	1.1E-10	-0.471377	265.60061	-0.0739719	9.9592331
Glt1d1	0.00E+00	0	-0.473503	#NUM!	-0.5400728	#NUM!
Pde3b	0.00E+00	0	-0.474357	#NUM!	-0.5514218	#NUM!
Iqgap2	0.00E+00	0	-0.479211	#NUM!	-0.3378206	#NUM!
Kmo	0.00E+00	0	-0.480293	#NUM!	-0.4281227	#NUM!
Eda	0.00E+00	0	-0.485712	#NUM!	-0.4196873	#NUM!
C6	0.00E+00	0	-0.485739	#NUM!	-0.5216642	#NUM!

Slc16a2	0.00E+00	0	-0.48645	#NUM!	-0.574114	#NUM!
Kyat1	0.00E+00	0	-0.490331	#NUM!	-0.3772918	#NUM!
Ass1	1.36E-179	1.2E-122	-0.491976	178.86539	-0.3151757	121.90477
Cmtm8	0.00E+00	0	-0.496677	#NUM!	-0.5796362	#NUM!
Cyp3a25	3.28E-196	1	-0.496755	195.48466	-0.0914248	0
Sox5	0.00E+00	1.7E-265	-0.504716	#NUM!	-0.3026007	264.765
Ptprk	0.00E+00	0	-0.508623	#NUM!	-0.4548742	#NUM!
Crot	9.82E-286	0	-0.513475	285.00784	-0.641069	#NUM!
Pcsk5	0.00E+00	0	-0.517351	#NUM!	-0.4241812	#NUM!
Nox4	0.00E+00	0	-0.521415	#NUM!	-0.5584105	#NUM!
Acbd5	0.00E+00	0	-0.522598	#NUM!	-0.5889776	#NUM!
Gm4951	0.00E+00	0	-0.522839	#NUM!	-0.4641727	#NUM!
Car8	0.00E+00	0	-0.526213	#NUM!	-0.5067942	#NUM!
Ptprd	0.00E+00	0	-0.527507	#NUM!	-0.3945193	#NUM!
Insig2	0.00E+00	0	-0.538484	#NUM!	-0.3612458	#NUM!
Cyp2c54	0.00E+00	0	-0.540121	#NUM!	-0.5696435	#NUM!
F830016B0	0.00E+00	0	-0.542486	#NUM!	-0.4817916	#NUM!
Mylk	0.00E+00	0	-0.552056	#NUM!	-0.6499088	#NUM!
Syne2	0.00E+00	0	-0.55244	#NUM!	-0.6320542	#NUM!
Bmp1	0.00E+00	0	-0.559557	#NUM!	-0.5216354	#NUM!
Clmn	0.00E+00	0	-0.565471	#NUM!	-0.7197355	#NUM!
Il1rap	0.00E+00	0	-0.569942	#NUM!	-0.6311532	#NUM!
Rnf169	0.00E+00	0	-0.581269	#NUM!	-0.7521988	#NUM!
Zfand4	0.00E+00	0	-0.58231	#NUM!	-0.6251478	#NUM!
Ppp1r3b	0.00E+00	0	-0.584015	#NUM!	-0.7085356	#NUM!
Slc7a2	1.25E-240	4.6E-104	-0.584227	239.90398	-0.2705075	103.34134
Slc2a2	0.00E+00	0	-0.585004	#NUM!	-0.4817793	#NUM!
Rbfox2	0.00E+00	0	-0.59153	#NUM!	-0.6409795	#NUM!
Serpina1c	0.00E+00	0	-0.609866	#NUM!	-0.587636	#NUM!
Hacl1	0.00E+00	0	-0.618361	#NUM!	-0.49466	#NUM!
Apob	0.00E+00	0	-0.618658	#NUM!	-0.3980552	#NUM!
Scp2	0.00E+00	0	-0.619487	#NUM!	-0.7224161	#NUM!
Dpys	0.00E+00	0	-0.621825	#NUM!	-0.7990249	#NUM!
Hal	0.00E+00	0	-0.628941	#NUM!	-0.5625343	#NUM!
Abat	0.00E+00	0	-0.629694	#NUM!	-0.5830212	#NUM!
Gm31508	0.00E+00	0	-0.631283	#NUM!	-0.5504511	#NUM!
Ttr	0.00E+00	0	-0.638698	#NUM!	-0.4302136	#NUM!
Glud1	0.00E+00	0	-0.643531	#NUM!	-0.4358983	#NUM!
Nudt7	0.00E+00	0	-0.644259	#NUM!	-0.5712347	#NUM!
Cyp7b1	0.00E+00	0	-0.646627	#NUM!	-0.922696	#NUM!
Pid1	0.00E+00	0	-0.656084	#NUM!	-0.4457282	#NUM!
Itih3	0.00E+00	0	-0.661823	#NUM!	-0.5291237	#NUM!
Hgd	0.00E+00	0	-0.662405	#NUM!	-0.616129	#NUM!
Clec2d	0.00E+00	0	-0.663224	#NUM!	-0.7388247	#NUM!
Abcb11	0.00E+00	4.71E-81	-0.666232	#NUM!	-0.2639559	80.326858
Cyp4a14	0.00E+00	9.41E-35	-0.667968	#NUM!	-0.1246022	34.026196
Slc39a14	0.00E+00	0	-0.676563	#NUM!	-0.7593126	#NUM!

Arhgap24	0.00E+00	0	-0.677651	#NUM!	-0.8522693	#NUM!
Ctcflos	0.00E+00	0	-0.678987	#NUM!	-0.6229506	#NUM!
Magi1	0.00E+00	0	-0.684902	#NUM!	-0.5606225	#NUM!
Gm48633	0.00E+00	0	-0.687128	#NUM!	-0.603179	#NUM!
Pzp	0.00E+00	1.9E-259	-0.687782	#NUM!	-0.4168103	258.73216
Phldb2	0.00E+00	0	-0.703034	#NUM!	-0.7904553	#NUM!
Dapk1	0.00E+00	0	-0.708702	#NUM!	-0.891481	#NUM!
Shank2	0.00E+00	0	-0.718062	#NUM!	-0.7531957	#NUM!
Got1	0.00E+00	0	-0.718356	#NUM!	-0.6757118	#NUM!
Slco1a1	0.00E+00	0	-0.738089	#NUM!	-0.7843654	#NUM!
Grb14	0.00E+00	0	-0.738363	#NUM!	-0.8312448	#NUM!
Hmgcs2	0.00E+00	0	-0.742414	#NUM!	-0.5938208	#NUM!
Kyat3	0.00E+00	0	-0.742472	#NUM!	-0.3799829	#NUM!
Dlc1	0.00E+00	0	-0.745545	#NUM!	-0.9287595	#NUM!
Sntg2	0.00E+00	0	-0.74577	#NUM!	-0.7753391	#NUM!
Chsy3	0.00E+00	0	-0.747703	#NUM!	-0.8828901	#NUM!
Sik3	0.00E+00	0	-0.753112	#NUM!	-0.9125708	#NUM!
Eci2	0.00E+00	0	-0.7532	#NUM!	-0.5733315	#NUM!
Igfbp2	0.00E+00	0	-0.757812	#NUM!	-0.6410439	#NUM!
Keg1	0.00E+00	0	-0.763902	#NUM!	-0.8066501	#NUM!
Acsl1	0.00E+00	0	-0.778529	#NUM!	-0.6593398	#NUM!
Adk	0.00E+00	0	-0.779751	#NUM!	-0.488998	#NUM!
Ablim3	0.00E+00	0	-0.791586	#NUM!	-0.8400568	#NUM!
Mcc	0.00E+00	0	-0.792559	#NUM!	-0.8812974	#NUM!
Azgp1	0.00E+00	0	-0.797812	#NUM!	-0.9437842	#NUM!
Mup3	0.00E+00	0	-0.801149	#NUM!	-0.8080373	#NUM!
B3galt1	0.00E+00	0	-0.80449	#NUM!	-1.0819707	#NUM!
Carmil1	0.00E+00	0	-0.810201	#NUM!	-0.8881386	#NUM!
Cps1	4.46E-234	3.98E-98	-0.820046	233.35032	-0.5721561	97.39987
Zeb1	0.00E+00	0	-0.828239	#NUM!	-0.9149638	#NUM!
Colec12	0.00E+00	0	-0.828518	#NUM!	-0.9782647	#NUM!
Nfib	0.00E+00	0	-0.834583	#NUM!	-0.9244376	#NUM!
Gm20319	0.00E+00	0	-0.846983	#NUM!	-0.9495814	#NUM!
Uox	0.00E+00	0	-0.864858	#NUM!	-0.9883043	#NUM!
Cyp2j5	0.00E+00	0	-0.86874	#NUM!	-1.044175	#NUM!
Tns1	0.00E+00	0	-0.892135	#NUM!	-0.9877186	#NUM!
Slc27a2	0.00E+00	0	-0.894343	#NUM!	-0.8438814	#NUM!
C8a	0.00E+00	0	-0.9174	#NUM!	-1.0380762	#NUM!
Hsd3b3	0.00E+00	0	-0.944902	#NUM!	-1.0121467	#NUM!
Errfi1	0.00E+00	0	-0.949623	#NUM!	-0.7591736	#NUM!
Immp2l	0.00E+00	0	-0.953842	#NUM!	-0.8475083	#NUM!
Prr16	0.00E+00	0	-0.974032	#NUM!	-1.1239089	#NUM!
Hc	0.00E+00	0	-0.981566	#NUM!	-1.0233667	#NUM!
Slco1b2	0.00E+00	0	-0.997088	#NUM!	-0.8843129	#NUM!
C9	0.00E+00	0	-1.014434	#NUM!	-1.0930739	#NUM!
Bhmt	0.00E+00	0	-1.062335	#NUM!	-1.023407	#NUM!
Slc22a30	0.00E+00	0	-1.068479	#NUM!	-1.2937704	#NUM!

Gfra1	0.00E+00	0	-1.112284	#NUM!	-1.3226596	#NUM!
Rhobtb1	0.00E+00	0	-1.112361	#NUM!	-1.2004765	#NUM!
Mat1a	0.00E+00	0	-1.112975	#NUM!	-0.9110104	#NUM!
G6pc	0.00E+00	0	-1.123058	#NUM!	-0.9427735	#NUM!
Pah	0.00E+00	0	-1.128802	#NUM!	-1.2102529	#NUM!
Neat1	0.00E+00	0	-1.152864	#NUM!	-0.9553294	#NUM!
Sugct	0.00E+00	0	-1.187534	#NUM!	-1.0392166	#NUM!
Igf1	0.00E+00	0	-1.197967	#NUM!	-1.4004831	#NUM!
Mug2	0.00E+00	0	-1.219814	#NUM!	-1.3852963	#NUM!
Aass	0.00E+00	0	-1.224727	#NUM!	-1.3294453	#NUM!
Ces3a	0.00E+00	0	-1.351681	#NUM!	-1.4601604	#NUM!
Cmah	0.00E+00	0	-1.403156	#NUM!	-1.5742331	#NUM!
Pck1	0.00E+00	0	-1.43439	#NUM!	-1.0924447	#NUM!
Kynu	0.00E+00	0	-1.438032	#NUM!	-1.5642712	#NUM!
Sult2a8	0.00E+00	0	-1.783734	#NUM!	-1.9109009	#NUM!
Ghr	0.00E+00	0	-1.799613	#NUM!	-1.9252475	#NUM!
Mug1	0.00E+00	0	-2.172715	#NUM!	-2.3969862	#NUM!
Ttc39c	0.00E+00	0	-2.251228	#NUM!	-2.4099152	#NUM!
Egfr	0.00E+00	0	-2.434922	#NUM!	-2.8322032	#NUM!

Top 25 Volcano Plot Gene Clusters from DAVID

DEG Group	Cluster	Description	Score	DEG Group	Cluster	Description	Score
Upregulated	1	Lipid Metabolism	26.20	Downregulated	1	Plasma Membrane	18.05
	2	Endoplasmic Reticulum	21.27		2	Pleckstrin Homology Domain	13.72
	3	Membrane	15.49		3	GTPase Activity	8.68
	4	Metal Binding	11.78		4	Src Homology Domain	8.25
	5	Glycoproteins	9.60		5	Ubl Conjugation	7.16
	6	Cholesterol/Steroid Metabolism	9.59		6	Transmembrane	7.14
	7	NADP	7.71		7	Protein Kinase Sites	6.22
	8	Mitochondrion	7.26		8	SH3 Domain	5.02
	9	ATP Binding	6.91		9	Metal Binding	4.60
	10	Lipid Transport/Cholesterol Homeostasis	5.83		10	Focal Adhesion	4.59
	11	Drug Metabolism	5.21		11	Protein Kinase Sites	4.29
	12	Metabolic Processes	4.94		12	Chemokine Signaling	3.92
	13	PDZ Domain	4.08		13	Lysosome	3.87
	14	Fatty Acid Metabolism	4.07		14	Cell Junction	3.78
	15	WW Domain	4.00		15	Endocytosis	3.77
	16	ABC Transporters/Bile Transport	3.90		16	Differentiation	3.57
	17	Ubl conjugation	3.80		17	Transcription Regulation	3.40
	18	Flavoproteins	3.72		18	Focal Adhesion	3.28
	19	DNA Binding/Transcription Regulation	3.60		19	Golgi Apparatus	3.21
	20	Calponin Homology Domain	3.53		20	Lipid Transport	3.03
	21	NAD/NADP	3.48		21	Immune System Process	2.93
	22	Peroxisome	3.46		22	Calcium	2.76
	23	Ligase Activity / Biotin	3.29		23	Cytokinesis	2.76
	24	Fibronectin	3.27		24	Growth Hormone Synthesis	2.74
	25	Cholesterol Efflux / HDL and LDL	3.25		25	Alpha Macroglobulin	2.73

TCDD			
**DEG Group**	**Cluster**	**Description**	**Score**
Upregulated	No significant clusters		
Downregulated	1	Endoplasmic reticiulum	2.85
	2	Oxidoreductase activity	2.33
	3	Lipid metabolism	1.63
	4	Mitochondrion	1.41

TCDD + Statin			
**DEG Group**	**Cluster**	**Description**	**Score**
Upregulated	1	Endoplasmic reticiulum	5.52
	2	Peroxisome	4.19
	3	Transport	2.85
	4	ATP/nucleotide binding	2.58
	5	Metal binding	1.96
	6	Oxidoreductase activity	1.48
	7	AAA domain	1.34
	8	Transcription regulation	1.33
	9	Lipid metabolism	1.31
Downregulated	1	Cell division	1.64

Gene	V_C_vs_adj_V_S	V_C_vs_adj_T_C	V_C_vs_adj_T_S	V_C_vs_calculatedFCV_S	V_C_vs_calculatedFCT_C	V_C_vs_calculatedFCT_S
Gpnmb	1	1.62E-123	7.4584E-140	-0.002206049	1.468548011	1.522064152
Lgmn	1	6.83E-94	7.6757E-116	-0.07925184	1.081848914	1.193516613
Abr	1	4.84E-122	6.6755E-127	-0.0249424	1.178980771	1.138554118
Cd84	1	7.25E-111	7.0774E-104	-0.067118938	1.10356787	1.025837046
Lilr4b	1	2.14E-112	1.6449E-113	0.029117646	0.994910657	0.979477466
Mmp27	1	4.26E-67	4.478E-111	0.001698584	0.685753403	0.972984643
Fmo3	1	9.97E-34	1.27E-153	0.018685953	0.328858519	0.968962285
Lilrb4a	1	9.40E-115	1.5653E-117	0.092237472	0.937708758	0.960161828
Lrmda	1	2.34E-80	2.10757E-77	-0.01291663	0.988787667	0.918618646
Adgb	1	3.30E-16	7.70784E-52	-0.029239134	0.518098493	0.863236884
Mertk	1	4.43E-46	1.48976E-68	0.048316051	0.717749073	0.83693243
Reln	1	1.56E-26	1.1542E-120	0.030489181	0.333351268	0.804744037
Mgat5	1	1.59E-74	2.16866E-73	-0.102277067	0.834003593	0.797289949
Apobec1	1	8.18E-77	4.72545E-74	-0.010129513	0.811268191	0.772488185
Arhgap10	1	1.68E-82	1.18977E-79	-0.005646544	0.86166203	0.767623056
Gm5150	1	1.82E-37	3.74847E-63	-0.074639771	0.585124999	0.766457165
Arhgap22	1	1.18E-69	5.69793E-75	0.031500267	0.723652871	0.745309758
Cyp1a2	1	2.97E-81	5.9199E-108	-0.017172756	0.557775652	0.737916746
Runx1	1	9.59E-61	1.57043E-57	0.002561014	0.782553482	0.734924449
Myo1f	1	2.56E-106	8.18272E-67	0.027696568	0.996639118	0.725520931
Pou2f2	1	2.03E-13	1.46805E-39	-0.014809263	0.411142093	0.717148327
Pla2g7	1	6.70E-79	9.9674E-80	0.033586718	0.730182814	0.70850356
Psap	1	1.07E-101	1.84741E-70	0.086690803	0.89632113	0.704305864
Fyb	1	7.08E-50	4.7677E-53	-0.07156906	0.67686266	0.693608652
Cd36	1	3.13E-59	1.93855E-63	-0.007050211	0.734561711	0.687734422
Gab2	1	2.90E-66	1.72842E-51	-0.066714044	0.791066155	0.683810175
Mmp12	1	3.40E-54	8.27914E-58	0.002767055	0.64324965	0.671041839
Dock10	1	1.61E-57	1.10576E-49	0.050618135	0.739583746	0.670947131
Slc8a1	1	6.73E-53	5.37911E-35	-0.101810472	0.85155254	0.665599756
Nrg1	1	1.21E-30	1.94545E-93	0.012087622	0.244957104	0.655710502
Cadm1	1	1.95E-19	2.03766E-37	-0.021389371	0.539114963	0.648208572
Cdh18	1	8.27E-22	1.35184E-80	-0.039042252	0.316833487	0.641596694
Ankrd12	1	2.23E-54	2.03729E-55	0.050216525	0.599310888	0.63629107
Itga9	1	3.46E-19	8.51039E-31	0.046825651	0.512409074	0.619028661
Sirpa	1	5.84E-66	7.10793E-56	0.035697419	0.703206824	0.616256084
Airn	1	2.10E-20	6.23335E-50	0.020873403	0.465285692	0.61559563
Fmn1	1	2.12E-63	1.23956E-52	0.064783199	0.718672518	0.601683819
Pvt1	1	7.19E-32	1.24953E-34	0.020992382	0.61533102	0.600188894
Man1c1	1	7.71E-51	6.94284E-56	0.013914147	0.580568724	0.598243634
Lpl	1	9.56E-77	1.89649E-64	0.011016389	0.73908452	0.593516818
Fnip2	1	1.10E-52	3.88915E-44	-0.010052325	0.67554736	0.593074542
Aoah	1	3.02E-32	1.35694E-32	0.072342573	0.602116227	0.591614283
Ccdc148	1	3.50E-14	8.62816E-27	0.070767022	0.43056599	0.569722598
Mitf	1	3.13E-54	2.3507E-35	0.029828888	0.769956927	0.563816609
Ifi207	1	1.60E-48	2.26581E-56	0.025684558	0.516034914	0.56273069
Vav3	1	2.83E-69	1.12921E-42	-0.01715239	0.773530058	0.56120507
Myof	1	4.11E-79	5.70483E-57	0.028904507	0.756360296	0.560896972
Lgals3	1	2.69E-83	2.9809E-57	-0.000427844	0.76191156	0.56050936
Gsr	1	1.05E-34	3.13751E-48	-0.056870575	0.470721803	0.559019685
Zeb2	1	2.77E-56	8.18822E-27	-0.114421114	0.822474091	0.556678299
Tgfbr1	1	2.97E-42	1.03233E-38	0.018476676	0.57939289	0.555404119
Iqgap1	1	3.50E-91	1.75501E-45	-0.017644802	0.817652357	0.547175469
Sat1	1	7.32E-51	3.22449E-48	0.061151966	0.546790522	0.545942942
Wwp1	1	2.12E-09	8.24365E-35	0.018132221	0.324863509	0.541837679
Cerk	1	5.26E-65	1.48805E-51	0.044783348	0.665173147	0.534854276
Ctss	1	1.08E-51	3.43183E-43	0.069805394	0.601938384	0.533564139
BC005537	1	1.97E-44	4.13506E-45	-0.056979542	0.531606652	0.528526598
Rftn1	1	3.21E-54	4.1784E-39	-0.10567973	0.66693459	0.526304356
Cblb	1	3.15E-22	8.39737E-28	0.048999095	0.468714167	0.526062543
Tcirg1	1	1.52E-54	4.5229E-51	-0.023371961	0.539989458	0.513271778
Fgd4	1	1.99E-32	8.34022E-35	-0.085040802	0.510621698	0.489678575
Rgs1	1	1.96E-49	2.88922E-58	0.033962763	0.473490942	0.489663898

Abca1	1	9.24E-49	3.55621E-30	0.019208995	0.698569089	0.485726563
Kcnq1ot1	0.298863675	1.14E-22	1.10387E-35	0.154001987	0.427986933	0.481459452
Ctsb	1	7.07E-59	2.63582E-35	-0.027907944	0.66044137	0.479974261
Gpr137b	1	5.10E-54	4.2916E-48	0.000400946	0.520240762	0.478033085
Itgav	1	4.23E-39	5.27583E-32	-0.026697567	0.516655966	0.474551243
Lrp1	1	6.67E-26	1.27428E-41	-0.048613897	0.379909594	0.473046454
Zfp704	1	1.05E-36	1.64148E-35	-0.01401123	0.484246643	0.469904268
Frmd4b	1	8.73E-13	1.09775E-19	-0.032892606	0.388702491	0.467796013
Plekhm2	1	1.50E-47	2.58011E-36	0.072658194	0.55715854	0.463266448
Fth1	1	2.74E-65	1.70956E-56	-0.005203068	0.49279308	0.454060778
Tnfaip2	1	4.27E-40	5.15218E-40	-0.025160581	0.441372124	0.450895252
Sh3pxd2b	1	2.78E-64	7.21393E-58	0.020653642	0.488430348	0.444673276
Elmo1	1	4.48E-58	1.01461E-25	-0.094544704	0.664820152	0.437913454
Dock4	1	6.24E-11	8.83893E-29	0.043668051	0.294656035	0.4346475
Ftl1	1	4.56E-52	1.87007E-39	0.102228939	0.488853252	0.431037446
Pnpla7	1	5.42E-30	1.41273E-30	0.009645226	0.438052091	0.42911329
Bcl2l1	1	5.74E-34	2.52371E-30	0.023527659	0.461567999	0.427988894
Txnrd1	1	8.17E-38	9.35716E-31	-0.01688341	0.516433112	0.425625617
Hip1	1	7.48E-40	9.28804E-27	-0.040124859	0.535235685	0.42486824
Aff1	1	1.42E-41	6.71863E-25	-0.083628787	0.545246425	0.418147645
Eepd1	1	1.63E-44	5.53375E-26	-0.015451755	0.586097748	0.417961957
Plin2	1	4.00E-33	5.91562E-29	-0.002009196	0.469292408	0.417300182
Filip1l	1	3.24E-18	5.21196E-27	0.009621905	0.330095852	0.415840517
Zmiz1	1	2.09E-28	2.64376E-32	0.046366306	0.400610499	0.415815937
Nampt	1	9.24E-29	3.84198E-32	0.03299945	0.375863764	0.414505219
Snx24	1	8.15E-26	3.4329E-23	0.064397842	0.458197032	0.414270994
Rasgef1b	1	2.91E-22	1.78843E-17	0.067325091	0.48401862	0.4084883
Msr1	1	9.47E-36	2.88966E-22	-0.067457201	0.528208171	0.407061561
Fkbp5	1.46381E-08	5.55E-01	4.20733E-16	0.418715264	0.174972654	0.406347758
Tbxas1	1	6.05E-49	8.30862E-22	-0.007300843	0.653284816	0.406245672
Ksr2	1	1.54E-32	4.47651E-17	0.117079047	0.666451885	0.405027996
Aopep	1	2.00E-14	6.85064E-31	-0.009270601	0.314335285	0.402248639
Evl	1	4.79E-51	9.49676E-27	0.001841791	0.603062904	0.402030134
Snx5	1	6.90E-47	9.11233E-32	0.007295633	0.49416524	0.39880098
Cyp2b9	1	9.14E-04	1.53078E-51	0.002159501	0.057955869	0.395924193
Kcnk13	1	2.05E-31	1.45464E-22	-0.037382162	0.492644592	0.394837179
Fmnl2	1	1.89E-23	9.77111E-11	-0.078504499	0.56169173	0.394382338
Mrc1	1	4.57E-02	3.67085E-13	-0.061074982	0.223721079	0.386651618
Cd38	1	6.31E-24	1.10923E-20	0.015491296	0.431369924	0.386607205
Actr3	1	4.11E-48	3.16456E-27	0.012842674	0.51569526	0.382473362
Dgkz	1	1.26E-38	8.8064E-28	-0.04697945	0.472057737	0.381856693
5033421BC	1	4.09E-30	6.39308E-31	-0.034454604	0.375330754	0.376496171
Abcg3	1	3.50E-14	1.93643E-15	0.022320536	0.376043842	0.376327479
Dnmt3a	1	4.10E-30	3.31264E-20	-0.004550035	0.484467562	0.373875524
Myo9b	1	2.28E-52	1.90163E-28	0.058774602	0.514595934	0.373673353
Sh3kbp1	1	2.90E-32	3.84039E-19	-0.04452566	0.496698251	0.372779651
Gns	1	3.91E-26	2.77571E-27	0.043515273	0.367152883	0.37260965
Atp8a1	1	2.35E-31	2.87548E-18	-0.005273093	0.481744281	0.37025982
Slc6a6	1	8.92E-40	1.67777E-25	-0.023375892	0.467108786	0.369068535
Pgap1	1	2.53E-31	6.50191E-32	-0.002976801	0.348176582	0.366116597
Apbb1ip	1	8.44E-49	5.0716E-20	-0.103316558	0.573567397	0.36536275
Myo9a	1	3.04E-24	4.70665E-12	0.03797667	0.516679779	0.363253229
Frrs1	1	1.20E-40	9.98627E-33	-0.01527769	0.406814769	0.363044667
Selenbp1	1	1.58E-01	6.45123E-28	0.054082674	0.109859078	0.362004523
Ahrr	1	1.64E-35	1.03244E-45	-0.00779504	0.301603123	0.361587571
Arhgap25	1	6.00E-49	1.22131E-20	0.021072212	0.599987383	0.357019808
Dhx9	1	5.91E-27	1.01087E-25	0.005979636	0.354421304	0.352973003
Csf2rb	1	1.60E-47	1.06577E-30	0.048686536	0.442134012	0.349140986
Tfec	1	1.55E-19	7.25682E-24	0.011731375	0.323697951	0.347287882
Tiparp	1	2.92E-24	1.79637E-35	-0.009100499	0.254671584	0.346246653
Entpd1	1	5.10E-31	1.62986E-13	0.056667746	0.535451996	0.34611687
Tmem104	1	4.51E-33	1.1494E-28	0.047079956	0.37170123	0.345706897
Cyp1a1	1	9.90E-37	1.76792E-46	0.002192839	0.264516227	0.345340689

Slc43a2	1	4.02E-20	3.62148E-16	-0.010714453	0.391727126	0.344823951
Lacc1	1	2.76E-21	1.27942E-33	0.065784763	0.251883164	0.34185666
Gclc	1	1.00E+00	7.03532E-23	0.020266344	-0.070963797	0.340196511
Malat1	7.5E-14	1.76E-75	3.75885E-56	0.22251005	0.401069156	0.339056768
Rcbtb2	1	1.22E-19	5.07729E-27	-0.021157151	0.277095068	0.338808307
St18	1	4.23E-34	3.74526E-25	0.002585545	0.453867054	0.338197401
Dst	1	7.60E-06	4.85587E-14	0.094734172	0.274527986	0.336915296
Nfe2l2	1	2.18E-05	8.10814E-22	-0.008122979	0.201404152	0.334911314
Atp6v1a	1	1.99E-30	4.61954E-27	0.02544398	0.347894599	0.334589381
Ms4a4a	1	1.58E-25	1.94163E-35	0.01019845	0.264332639	0.333074187
Gusb	1	9.43E-38	2.00712E-35	0.028607219	0.347410194	0.332814073
Tns3	1	1.62E-22	1.21411E-15	-0.008153118	0.407970886	0.332446674
Sema6d	1	7.29E-09	7.51766E-10	0.101380523	0.334145178	0.332176344
Capzb	1	7.68E-29	8.21782E-20	0.044438659	0.386627672	0.330021539
Abcg1	1	7.25E-44	1.05957E-22	0.013317887	0.491459632	0.329770722
Cttnbp2nl	1	3.61E-29	9.49143E-26	0.012395019	0.350225346	0.329100834
Ctsd	1	1.98E-65	3.21263E-35	0.04881184	0.481920845	0.326393256
Tlr4	1	1.20E-32	6.0763E-33	0.036141041	0.309267856	0.326092459
Atp6v1b2	1	1.56E-39	8.08457E-25	-0.03071119	0.435024275	0.325461464
Dram2	1	1.73E-16	3.04454E-21	-0.005722834	0.278304701	0.325331241
Ppm1h	1	3.13E-25	1.02797E-12	-0.156169337	0.44514987	0.325006873
Tbc1d23	1	2.78E-31	2.68581E-22	0.017225751	0.369231174	0.324970443
Gm50020	1	2.54E-21	1.36522E-33	-0.001408531	0.228869054	0.324962004
Inpp5d	1	3.95E-46	6.04904E-16	0.001424702	0.557547095	0.324346536
Wdr91	1	2.03E-22	3.4546E-29	0.0308021	0.264975268	0.324252639
Gab3	1	1.10E-12	5.0255E-18	0.008507788	0.275315939	0.323708574
Mthfs	1	8.71E-08	7.44716E-10	-0.006296628	0.277589074	0.323112188
Slc38a6	1	3.37E-15	4.58239E-18	-0.076673722	0.290090832	0.322900263
Adgre1	1	4.40E-04	8.09924E-08	-0.091018426	0.269810624	0.322557694
Slco3a1	1	8.77E-21	6.04358E-13	-0.005462075	0.406980139	0.321851484
Gpr141	1	1.55E-22	6.01696E-18	0.011060216	0.370649438	0.320546285
Stab1	1	3.32E-33	2.79914E-24	0.012567932	0.365259729	0.320516796
Adgre5	1	2.79E-16	3.42078E-17	0.077411157	0.313465899	0.319777702
Mdm2	1	3.57E-23	1.27521E-22	0.02512237	0.311149066	0.31872538
Atp6v0d2	1	2.78E-31	1.0633E-24	0.004385678	0.414474878	0.318145882
Pik3cb	1	1.12E-37	1.16682E-21	-0.002078014	0.437099589	0.312933549
Pkn1	1	2.59E-21	5.20372E-22	0.041756768	0.294983862	0.310890757
Esrrg	1	1.00E+00	4.91709E-20	-0.007224483	-0.034195302	0.310315398
Resf1	1	2.51E-11	2.6532E-17	0.031539205	0.242775141	0.310206537
Phc2	1	2.18E-19	3.17523E-17	0.028685208	0.329278893	0.308126964
Atp6v0b	1	2.23E-22	9.31938E-22	0.058663894	0.299707611	0.307413602
Fam172a	1	1.06E-19	3.89662E-19	0.07413052	0.317854444	0.307199679
Dock2	1	5.44E-52	2.30161E-10	-0.07970349	0.672609796	0.306326783
Myo5a	1	2.37E-33	1.45426E-17	-0.033840117	0.462382822	0.305103166
Irak2	1	1.66E-16	1.1754E-14	-0.013944423	0.324512254	0.304295986
Tbc1d22a	1	1.11E-38	5.40342E-16	0.008766808	0.471331207	0.303270521
Tpcn2	1	2.96E-21	5.09993E-24	-0.028804543	0.271704234	0.303142771
Parp8	1	5.70E-28	1.23664E-12	-0.053549415	0.442235047	0.302240784
Galnt7	1	3.11E-29	1.37638E-21	0.020068789	0.366767417	0.30122752
Abcc5	1	7.87E-33	1.36352E-20	-0.063991839	0.412067041	0.300329991
Ggta1	1	2.69E-32	3.25384E-25	-0.068377413	0.352826503	0.299941996
Csf2ra	1	3.17E-43	1.74468E-31	0.074208667	0.345108879	0.297792904
Dip2b	1	2.97E-31	2.3612E-15	0.100732239	0.421502136	0.297441051
Atp8b4	1	2.71E-30	1.74724E-20	0.008319525	0.38496575	0.29450229
P2rx4	1	3.12E-25	8.67884E-17	-0.011671042	0.360868607	0.294211796
Skap2	1	1.45E-50	1.58244E-12	-0.098930257	0.578121865	0.29232497
Cd5l	1	1.02E-06	0.007199547	-0.039429466	0.419499505	0.290398032
Blvrb	1	1.03E-24	4.84274E-23	-0.001095125	0.307454882	0.290145227
Shtn1	1	1.57E-18	2.54936E-15	0.021125315	0.316150511	0.289574693
Serinc3	1	4.32E-08	1.43191E-15	0.075409556	0.213617969	0.289507751
Rbm47	1	6.66E-26	1.59486E-16	-0.03894301	0.361976038	0.287989379
Ptpn12	1	6.81E-26	1.32992E-13	-0.034192843	0.387791333	0.287575675
Atp2b1	1	2.66E-25	7.96077E-11	-0.065412823	0.435704395	0.286466738

Camk2d	1	1.53E-22	1.82436E-10	-0.050917953	0.421600518	0.285673904
Adap2	1	1.06E-18	1.56728E-11	0.020725684	0.366053902	0.285277642
Ubash3b	1	9.56E-30	3.89628E-12	-0.040277756	0.463566726	0.283501807
Rgl1	1	6.01E-09	4.75858E-09	-0.048855182	0.305578592	0.280659835
Basp1	1	6.66E-32	1.42823E-22	-0.00856165	0.378803843	0.280544076
Ms4a6c	1	2.60E-12	1.62883E-17	-0.010077129	0.223910634	0.279315778
Washc2	1	1.29E-14	8.1076E-17	0.018120578	0.247341577	0.279289013
Fnbp1	1	1.31E-20	1.41404E-10	-0.08756656	0.386194843	0.278753064
Wdfy3	1	1.13E-06	1.18519E-10	0.049792864	0.242460372	0.27796044
Tlr7	1	2.30E-14	1.43332E-20	-0.048566867	0.218761284	0.277657806
Lipa	1	1.82E-33	4.83561E-19	0.016885168	0.384308723	0.277518612
Ms4a7	1	3.56E-25	4.58867E-31	-0.003441284	0.222374932	0.277187586
Dnajc13	1	3.54E-17	4.0445E-16	0.041048972	0.275523897	0.276326827
Itgb5	1	2.09E-20	3.60088E-12	0.001209471	0.353536083	0.276001237
Mrtfa	1	9.64E-24	6.96472E-11	-0.00690596	0.390914772	0.274268695
Ranbp2	1	1.47E-13	1.24458E-15	0.017254039	0.246563892	0.273782442
Pik3r5	1	1.52E-33	7.63546E-16	-0.015108835	0.421399122	0.273367554
Otulinl	1	9.18E-29	4.18717E-18	-0.013748007	0.360560368	0.273296919
Gng2	1	3.31E-22	1.22481E-11	-0.020028934	0.35927061	0.271992417
Marco	1	3.03E-20	1.48468E-22	0.03552063	0.249855596	0.271881551
Abcc1	1	1.35E-35	5.45797E-21	-0.017847534	0.363979197	0.269845683
Zfc3h1	1	1.33E-12	1.04046E-14	0.032828235	0.246112339	0.26945981
Mga	1	1.67E-10	1.00601E-16	-0.044103077	0.201856783	0.268435669
Fam219a	1	1.92E-18	1.28894E-13	-0.007354947	0.32810967	0.268268538
Rpl10a	1	3.64E-26	2.46839E-26	0.066561094	0.244475277	0.268052741
Abhd12	1	3.50E-30	5.00935E-14	0.003162848	0.41220919	0.265961454
Colgalt1	1	1.51E-22	4.20094E-20	0.043070875	0.278823124	0.265661513
Wipf1	1	2.07E-29	5.33561E-09	0.052959298	0.455232624	0.264243242
Cers6	1	7.39E-24	9.72839E-08	-0.05294145	0.444455412	0.263609906
Maml2	1	7.69E-09	1.08192E-05	-0.096717371	0.321287018	0.261671825
Abcc4	1	2.64E-16	7.96478E-16	-0.039106479	0.248399178	0.261323568
Atp1b3	1	7.94E-16	2.49036E-15	0.013678345	0.236608835	0.260214568
Phip	1	6.12E-12	3.77904E-11	-0.062599303	0.263586075	0.260136149
Csf1r	1	1.14E-11	8.76599E-10	0.023892604	0.269622638	0.258835337
Mapre2	1	2.87E-15	1.22633E-13	0.021668883	0.275324699	0.258742921
Trpm7	1	1.17E-02	7.80728E-17	0.042973975	0.131736177	0.258511415
Xdh	1	1.00E+00	4.71182E-12	0.03095827	0.117438016	0.258205454
Rbm39	1	1.15E-10	1.03153E-17	0.031179347	0.203523294	0.258049917
Ano6	1	5.70E-14	2.81509E-07	0.037169707	0.355549478	0.257584376
Ulbp1	1	2.01E-17	1.51919E-25	0.003773788	0.185455139	0.257421919
Pparg	1	2.51E-30	4.97357E-17	-0.032216982	0.38071325	0.257353629
Birc3	1	3.43E-15	2.34831E-10	0.012574452	0.295998387	0.256280718
Vcam1	1	2.72E-03	8.66671E-08	0.109523176	0.191722416	0.255713978
Ncf2	1	3.14E-35	3.08378E-16	0.025060613	0.389031076	0.254882886
Atp6v0a1	1	9.68E-50	1.75778E-15	0.015720215	0.51363107	0.254451368
Ms4a6d	1	1.76E-19	8.17462E-25	0.020982907	0.208899139	0.254187052
Snx30	1	3.83E-18	1.63204E-15	-0.006071854	0.266172872	0.251496735
Washc4	1	5.32E-21	4.59398E-17	0.045689434	0.266792791	0.250781004
Ddhd1	1	6.62E-11	5.77627E-06	0.090991881	0.329063381	0.25036504
Ifi204	1	3.23E-16	3.18247E-14	-0.033334455	0.261440068	0.250132788
Srgap2	1	1.94E-14	5.22526E-11	-0.03793019	0.306250159	0.249474016
Herc1	1	1.37E-05	2.7129E-14	0.029877125	0.172011931	0.249019866
Mvb12b	1	4.75E-20	9.43672E-19	-0.005097377	0.254139953	0.248835005
Vps54	1	1.33E-16	7.26377E-11	-0.041158166	0.298384818	0.248207694
Cd300lb	1	1.10E-42	2.11671E-21	-0.000893459	0.390542978	0.24768666
Dnm2	1	6.12E-19	1.93325E-12	0.031743785	0.298990643	0.247283829
Brip1os	1	2.22E-05	2.22051E-10	0.006595518	0.176679644	0.24706728
Hexa	1	3.87E-30	4.4959E-16	0.016961729	0.336577771	0.246809512
B430306N(	1	4.62E-34	1.30018E-24	0.011378492	0.304383665	0.246459533
Vmp1	1	2.80E-06	1.12726E-09	0.127678957	0.202069747	0.246436637
Pla2g4a	1	1.91E-14	2.09862E-11	-0.066211991	0.287305167	0.245635922
Lcp1	1	8.20E-31	8.88606E-10	0.038141753	0.431938306	0.245488611
Soat1	1	3.59E-26	8.46975E-13	0.001144366	0.355386049	0.244955391

Zup1	1	8.14E-09	5.10581E-13	-0.044240239	0.194241822	0.244468525
Myh9	1	4.68E-22	8.24361E-09	0.008292989	0.365490905	0.243060648
Cd44	1	6.88E-40	1.42826E-05	-0.023513964	0.577271069	0.241657783
Arhgap31	1	1.70E-20	1.9472E-11	-0.071869545	0.329607146	0.241588615
Ptbp3	1	2.01E-25	3.17049E-11	0.001532614	0.350966384	0.240847865
Dmxl1	1	1.20E-05	2.74173E-11	-0.014975487	0.187692189	0.240129178
H2-D1	1	2.43E-25	2.00825E-17	0.060282424	0.290048002	0.239875399
Nckap1l	1	1.72E-33	4.29018E-15	0.0198032	0.365587474	0.239503273
Gm26740	1	1.62E-09	1.02303E-05	-0.123385398	0.295368982	0.238540836
Ms4a6b	1	4.17E-10	2.01337E-19	-0.031068577	0.160268528	0.237885399
Gstm1	1	5.79E-21	2.72127E-15	-0.044180892	0.268294568	0.236605946
Elf2	1	1.93E-16	1.15867E-09	0.02820448	0.301433226	0.23600058
Mdfic	1	1.21E-22	1.05521E-10	-0.079451045	0.350282551	0.235279039
Maf	1	2.91E-02	1.30252E-07	-0.023252688	0.156450613	0.23479478
Akap10	1	2.54E-17	1.53696E-09	0.010770176	0.298853952	0.233360274
Ptpn1	1	2.22E-21	3.22244E-08	0.075100305	0.352319618	0.232619739
Litaf	1	1.30E-31	7.11517E-08	0.001640205	0.4404708	0.232465923
Baz1a	1	6.21E-21	7.42411E-11	0.008225236	0.313578041	0.232026896
Slamf7	1	1.38E-27	3.79932E-22	0.003450111	0.267896185	0.23191391
Exoc3	1	6.54E-22	1.0927E-21	0.021994841	0.222255742	0.231897155
Npc1	1	4.05E-09	1.06981E-10	-0.018067879	0.193761264	0.231028535
Ttyh2	1	8.06E-15	1.87253E-13	0.011545212	0.240008447	0.230160245
Tpp2	1	1.85E-08	2.64555E-10	-0.009409451	0.202647242	0.229809323
Nisch	1	2.54E-03	1.08203E-09	0.104557269	0.15100691	0.229554913
Rps14	1	1.73E-16	9.83236E-17	-0.00103443	0.224443017	0.229102295
Cbl	1	8.13E-24	2.22486E-08	-0.068762433	0.369385675	0.228826106
Plekhm3	0.711099411	4.99E-31	1.51286E-07	-0.178855103	0.458078918	0.22774865
Dync1h1	1	1.12E-12	4.38262E-10	0.018550311	0.235679482	0.227611444
Tbc1d9	1	3.21E-21	4.70005E-09	-0.00786595	0.342328176	0.227558967
Cd274	1	1.91E-31	1.66603E-18	0.038907157	0.318851159	0.22690762
Slc12a6	1	5.29E-12	4.81656E-08	-0.039457755	0.265054373	0.226477889
Hpcal1	1	1.65E-21	0.000159841	-0.106875272	0.428374011	0.225854842
Gm42047	1	1.00E+00	4.292E-15	0.019718665	0.068798785	0.22520496
Nceh1	1	6.08E-18	1.32873E-10	-0.009646203	0.295885372	0.223851799
Amz1	1	2.09E-34	6.15646E-22	-0.00665424	0.303884957	0.223364757
Rap1gds1	1	1.33E-13	8.87099E-06	0.060517355	0.298066674	0.221932273
Epb41l2	1	5.31E-27	1.8613E-09	-0.057238851	0.36844251	0.219676252
Fam49a	1	5.00E-08	9.96762E-06	-0.017393446	0.256331928	0.218855841
Specc1	1	1.28E-24	9.96237E-14	-0.054374605	0.320287114	0.218226131
Tmem140	1	5.97E-12	5.74936E-15	0.034118159	0.18725058	0.217719147
Rab3gap2	1	6.47E-14	4.45634E-12	-0.022288151	0.222137219	0.216821199
Rufy3	1	5.63E-12	2.34789E-07	-0.002432799	0.272289313	0.216330037
Trafd1	1	5.09E-08	6.07257E-13	0.102140683	0.164762742	0.216314959
Gsap	1	1.07E-22	6.73195E-07	-0.045569993	0.372854944	0.216066388
Mmp13	1	9.17E-15	8.65205E-20	0	0.172585555	0.215065527
Cept1	1	6.32E-08	5.13752E-07	0.090468586	0.222309221	0.214124576
Dab2	1	3.01E-01	2.81065E-08	-0.022819853	0.12198061	0.213847546
Cybb	1	2.02E-16	8.7396E-11	-0.022547279	0.274368952	0.213573593
Ppard	1	6.50E-09	8.96972E-10	0.042016537	0.200100373	0.212645686
Cltc	1	1.05E-19	5.61343E-09	0.056324532	0.284063302	0.212255242
Fam20c	1	1.49E-27	2.00313E-10	0.004839246	0.369441135	0.211992363
Fermt3	1	1.32E-17	3.4796E-11	0.008811458	0.262552468	0.211971574
Mpeg1	1	1.06E-22	9.79641E-20	-0.005800901	0.230316104	0.211766664
Tbk1	1	6.09E-14	1.94863E-10	-0.020961144	0.232886303	0.211593281
D10Wsu10	1	1.85E-17	4.73706E-21	-0.009739871	0.189385554	0.211538486
Hnrnpa3	1	3.19E-11	4.90139E-10	0.043641467	0.203032599	0.210934402
Hk3	1	1.72E-22	2.34299E-11	-0.015852024	0.300138491	0.210011512
Slc37a2	1	8.65E-30	2.69138E-22	-0.002425578	0.2682279	0.209793333
Tasor2	1	1.88E-16	4.3901E-14	0.041866325	0.215518105	0.209790535
Hgsnat	1	1.00E+00	9.13475E-06	-0.005117216	0.127064105	0.209665193
Slc11a1	1	1.43E-21	1.39331E-15	0.016990999	0.243446776	0.209617098
Arpc2	1	5.61E-19	1.35664E-08	0.015065549	0.298578382	0.209613534
Mroh1	1	1.11E-15	2.36011E-15	-0.027907486	0.207496258	0.209263917

Pgd	1	1.02E-19	3.70737E-18	0.020684432	0.211321215	0.208794122
Vrk2	1	5.32E-18	0.000312646	-0.070150951	0.365218167	0.208743245
Vav1	1	7.98E-24	1.86827E-11	0.034018685	0.294361017	0.208486309
Ctsa	1	1.95E-16	3.20544E-13	0.033132616	0.216823868	0.207827133
Myom1	1	1.47E-08	7.83374E-21	-0.010436764	0.107103963	0.207297811
Aig1	1	6.17E-08	2.39088E-07	0.051415825	0.220419968	0.206124208
Dock11	1	1.07E-17	1.23531E-08	-0.04430026	0.287548229	0.20567177
Klf6	1	9.15E-14	7.3766E-15	-0.019880245	0.177870129	0.205425113
Fndc3b	1	1.45E-14	6.76244E-07	0.014685257	0.288396343	0.205255212
Exoc4	1	3.51E-09	3.73947E-08	-0.061911298	0.219650142	0.204926319
Unc93b1	1	2.52E-22	1.80787E-07	-0.00685006	0.328346198	0.20413436
Dtnbp1	1	6.10E-18	4.74838E-09	0.00467083	0.277969745	0.203881074
Mlxip	1	1.51E-13	5.33645E-07	-0.00263145	0.264579723	0.203333276
Rapgef5	1	8.38E-21	4.18828E-13	-0.006525618	0.279887205	0.203237555
Flna	1	5.45E-20	1.97429E-13	-0.019034449	0.235782885	0.201555273
Slc7a11	1	2.33E-16	3.37666E-17	-0.001802325	0.212812558	0.201006495
Erbin	1	4.12E-08	2.27566E-07	0.058694836	0.205281089	0.200525443
Gm14221	1	1.78E-10	4.0937E-13	0.006384067	0.177420162	0.199905204
Anxa4	1	4.68E-24	1.37406E-14	0.002541547	0.270082586	0.199871085
Cyp2a5	1	1.77E-25	1.68381E-19	0.009179508	0.219088713	0.199567888
Pikfyve	1	3.22E-08	4.04873E-07	-0.008984176	0.207342732	0.199171835
Cyfip1	1	8.68E-20	1.75117E-08	-0.027715124	0.30611167	0.199087389
Clec1b	1	1.15E-11	9.55974E-13	-0.004677741	0.190549721	0.198880795
Hpgds	1	2.89E-21	1.06017E-17	0.010421886	0.218638056	0.198798331
Coro7	1	3.99E-16	3.42421E-07	0.020283332	0.280201288	0.198523854
E230029CC	1	5.00E-10	0.000146983	0.007131615	0.272707653	0.198183421
Clcn7	1	8.15E-13	2.61284E-15	-0.001488071	0.166470411	0.197225645
Agap1	1	2.04E-07	0.015713613	-0.019023935	0.290473017	0.196854156
C1qb	1	7.89E-21	1.24926E-15	0.0831681	0.231157505	0.196785938
Smchd1	1	5.69E-15	4.45185E-06	-0.076536419	0.279190118	0.1967603
Rnf150	1	2.05E-15	1.17602E-06	0.016381295	0.323081261	0.196560129
Ndst1	1	6.32E-07	5.33117E-07	-0.025038433	0.194625914	0.19654561
Tmem273	1	1.21E-14	1.41398E-14	-0.029540408	0.198692977	0.195761773
Coro1c	1	7.82E-26	3.99244E-12	-0.012953453	0.291219948	0.195662091
Tmem87b	1	9.65E-14	9.73023E-10	0.043057428	0.21779183	0.195604306
Gm16556	1	6.56E-18	7.86082E-22	0.012872139	0.173105858	0.195427292
A330023F2	1	4.44E-06	2.80119E-05	-0.045371361	0.199214629	0.195062428
Zfp992	1	3.21E-18	1.00042E-17	-0.005386771	0.197200348	0.194914118
Pirb	1	8.40E-20	1.79442E-10	0.000438273	0.255976667	0.194588233
Ccny	1	4.84E-03	0.000657641	0.0062354	0.177457855	0.19451708
Gm20528	1	1.00E+00	1.91092E-06	0.036701312	0.069508354	0.194450496
Ankrd24	1	1.14E-02	2.14152E-09	-0.003984444	0.115640694	0.194310137
Lrp12	1	1.80E-18	1.92499E-13	-0.025320618	0.250397752	0.194289114
Sirpb1b	1	4.83E-13	3.56126E-13	-0.025616396	0.204333644	0.193743634
Tbc1d16	1	2.57E-05	6.33349E-11	0.035631311	0.131015244	0.19266024
Pik3r1	1	1.74E-01	0.003346348	0.057729517	0.163299227	0.192501873
Igf1	2.34834E-15	1.00E+00	0.889263623	-0.475457878	0.136659323	0.192343783
Man2b1	1	2.84E-10	1.25424E-10	0.013447018	0.186363033	0.192249575
Ccr1	1	2.43E-21	7.32864E-22	0.006343421	0.176256041	0.192203994
Tnfrsf11a	1	5.51E-23	1.39323E-07	-0.049732624	0.337570396	0.191690654
Taf1d	1	4.68E-13	4.13299E-09	0.067429882	0.208854614	0.191339134
Slc7a8	1	1.24E-12	1.54353E-05	-0.047388424	0.302989951	0.191296398
Krit1	1	5.25E-07	2.78162E-09	0.054794435	0.155147913	0.191150168
Lcorl	1	7.28E-07	4.37783E-05	0.041860765	0.203340328	0.190996463
Arhgap19	1	1.80E-14	5.35458E-17	0.013421785	0.169872944	0.190806549
Rtn4	1	2.98E-26	7.3017E-06	-0.091243286	0.371637498	0.190516849
Appl2	1	2.71E-12	7.09296E-12	-0.001716396	0.186670328	0.190496262
Gas2l3	1	1.99E-33	4.68624E-20	0.020787188	0.270494094	0.189924732
Sh3bp2	1	2.17E-21	2.5894E-09	0.001096074	0.285471272	0.189061932
Anxa3	1	1.02E-22	2.46034E-13	-0.014244155	0.263337328	0.188683593
Coro2a	1	1.82E-14	5.05146E-08	0.065640202	0.245595255	0.187437639
Syk	1	1.26E-32	0.000111664	-0.004488488	0.450697287	0.186380589
Tmem51	1	9.20E-14	9.68337E-06	0.023046785	0.278583669	0.186180448

Rasa3	1	1.93E-12	0.000693387	-0.047533377	0.288619872	0.186033873
Prpf39	1	1.92E-06	2.03413E-08	0.018282288	0.155036311	0.186026709
Rock1	1	1.09E-10	9.58182E-07	0.084947784	0.224387058	0.185660983
9130230L2	1	4.85E-05	1.72979E-12	-0.012690226	0.10882805	0.185565078
Rab31	1	9.73E-21	2.09055E-06	-0.019420208	0.32906724	0.185325536
Ttc14	1	4.92E-06	8.13472E-08	0.03407924	0.15299378	0.185175343
Vps35	1	2.65E-18	9.09152E-13	0.02748136	0.210484188	0.185124173
Fblim1	1	1.76E-27	3.86806E-18	0.021786464	0.237971622	0.18473957
Gnaq	1	6.97E-20	2.56455E-05	-0.120564578	0.322171424	0.184040898
Dennd4c	1	2.77E-07	0.000139214	-0.041347889	0.206786603	0.183337369
Sgk1	1	1.24E-10	1.24262E-13	0.012522407	0.165162946	0.18290839
Sdcbp	1	1.74E-26	9.09865E-11	-0.017598443	0.297026995	0.182823686
Rreb1	1	9.83E-18	9.64537E-05	0.017171362	0.325202777	0.182536473
Dse	1	2.36E-10	0.000142863	-0.030676658	0.253954285	0.181472269
Ikbke	1	4.09E-20	3.7829E-11	-0.018312124	0.248852874	0.18146627
Gtpbp2	1	3.56E-08	8.47455E-11	0.044969487	0.146087219	0.181284437
Bach2os	1	4.72E-12	1.71015E-09	0.004088859	0.197509285	0.180184334
Atp6v0d1	1	1.44E-18	2.49796E-08	0.017886937	0.252271649	0.179969311
Mbnl2	1	1.38E-11	1.92461E-05	0.000374895	0.259107744	0.179532519
Sash1	1	4.56E-07	0.000581293	-0.032810244	0.23433288	0.179488671
Ncoa7	1	7.73E-16	1.88837E-08	0.008064792	0.237213823	0.179470446
Stard9	1	7.91E-09	7.62995E-06	-0.019584861	0.208544139	0.179180766
Ric1	1	5.36E-13	1.13282E-05	-0.01568116	0.242488299	0.178817464
0610010F0	1	7.14E-06	8.77301E-11	0.015125782	0.126186575	0.178732051
Cd200r1	1	1.79E-19	8.00232E-16	-0.012358177	0.205883889	0.178569801
Cln3	1	2.36E-15	1.27272E-11	0.0053133	0.193699725	0.178227254
Arfgef2	1	5.89E-09	3.44717E-06	0.08108424	0.194167441	0.177881695
Wnk1	1	5.86E-10	2.88775E-05	0.033219523	0.229999973	0.17759787
Tep1	1	2.88E-15	3.02538E-08	0.00443502	0.226190042	0.177149413
Htatip2	1	2.61E-04	1.14985E-12	-0.006515701	0.095379116	0.176772959
Csf2rb2	1	6.12E-25	8.03247E-19	0.013608274	0.209313762	0.176470728
Nfic	1	5.43E-03	1.08984E-06	-0.006996269	0.107930129	0.175763108
Cep70	1	4.46E-16	1.23963E-12	0.013398257	0.19819814	0.175754315
Fam129b	1	4.68E-23	4.92881E-12	-0.018735334	0.285042	0.175038126
Itsn2	1	2.39E-06	9.95379E-06	-0.009686597	0.184654215	0.17483168
Vps8	1	4.09E-20	3.11391E-09	0.024302459	0.25167298	0.174585528
Slc15a3	1	4.45E-25	8.27738E-07	-0.080147385	0.332362426	0.174477068
Pkib	1	6.83E-20	2.64828E-15	0.010003566	0.209877046	0.174463229
Xrcc4	1	3.61E-08	0.000478531	-0.019555805	0.220851689	0.174216196
Picalm	1	3.17E-26	0.000907397	0.027984547	0.395410341	0.174015694
Rsrp1	0.672940568	1.00E+00	2.63797E-05	0.162787138	0.049065949	0.173931012
Dennd1b	1	2.38E-14	0.001461769	-0.014343832	0.2898896	0.173367287
Stk4	1	6.77E-14	1.43109E-06	-0.040872197	0.235178772	0.173197181
Por	1	1.00E+00	0.000467216	0.082574387	0.003121786	0.172995727
Itpkb	1	2.23E-23	0.000426293	-0.021217296	0.38088677	0.17247665
Tmem106a	1	1.27E-16	1.49739E-09	0.002751234	0.227072054	0.172464257
Ewsr1	1	1.84E-11	2.47547E-05	0.027816478	0.219522746	0.172375152
Tbc1d14	1	3.40E-10	5.94721E-05	-0.028812894	0.220827055	0.17183888
Slx4ip	1	8.89E-13	4.37621E-08	-0.019328853	0.208904874	0.17181243
Wdr26	1	3.82E-07	6.95985E-05	0.047855764	0.184418538	0.17175779
Txnip	1	1.37E-06	7.53007E-11	0.03763341	0.12456485	0.171411791
Cyth4	1	8.63E-24	6.15137E-08	0.017695303	0.305046722	0.171034463
Akr1a1	1	1.92E-17	4.95368E-07	0.001819325	0.244483896	0.170474743
Otulin	1	1.78E-01	0.003823137	-0.083160383	0.13042831	0.170459077
Arid4a	1	6.85E-08	0.001188951	0.069555582	0.218141739	0.170369747
Ugdh	1	1.00E+00	0.000127613	0.030217985	-0.021548289	0.169652488
Aim2	1	2.58E-14	1.90986E-07	-0.060998066	0.223658279	0.169598614
Pkm	1	3.70E-22	2.52831E-15	0.014976281	0.2098035	0.169303773
Plod1	1	1.40E-08	5.44882E-10	0.055453689	0.150167318	0.169127189
Adcy7	1	5.19E-17	8.38728E-06	-0.024848454	0.261427249	0.168753144
Gm15943	1	1.60E-19	2.18749E-14	-0.011392951	0.219871028	0.168487687
Pik3ap1	1	9.96E-06	0.003695622	-0.015576361	0.207288786	0.167992527
Igsf6	1	1.95E-16	5.45085E-11	-0.013911084	0.212939277	0.167761185

Pitpna	1	1.04E-14	0.003623635	-0.013116907	0.295603583	0.167701508
P2rx7	1	3.77E-14	1.3499E-08	0.030116167	0.199838194	0.166802298
Map4k3	1	2.69E-01	0.000361056	0.039672696	0.136659903	0.166757349
Rasa4	1	2.53E-19	7.61098E-10	0.022732992	0.236961675	0.166597102
Gstm3	1	1.45E-16	1.40677E-18	-0.004458645	0.141767947	0.16655904
Tmlhe	1	4.65E-01	7.2062E-07	-0.033071151	0.092959429	0.166266033
4930599N2	1	7.97E-14	3.27186E-09	0.015501209	0.197155424	0.166239764
Gmip	1	3.70E-08	6.46645E-07	0.013625485	0.164358082	0.16607864
Btaf1	1	4.93E-08	5.81948E-05	0.000950434	0.190202161	0.16593087
Creb5	1	2.59E-18	7.42772E-10	0.003522304	0.236020748	0.165729151
Arglu1	1	8.98E-05	0.000154026	0.010181371	0.153182179	0.165688252
Abl2	1	1.13E-08	0.002655634	0.026480778	0.232801244	0.165369845
Il10rb	1	1.55E-22	1.21013E-08	-0.000736069	0.273090798	0.165237917
Git2	1	3.27E-09	0.000373824	-0.021812408	0.216136871	0.165167697
Rpl41	1	7.67E-19	1.6182E-12	0.022382196	0.201359015	0.164932919
Rragc	1	4.25E-16	5.02076E-12	0.015371015	0.186013851	0.163515048
Cd180	1	7.29E-19	5.11676E-05	-0.042993673	0.303878077	0.16346973
Pitpnc1	1	5.84E-03	1	0.053611149	0.214776167	0.16342269
Hck	1	4.10E-10	0.055411195	0.032343746	0.268040981	0.163367328
Nipa2	1	4.41E-13	1.93058E-06	0.019906014	0.21567312	0.163181338
AU020206	1	1.99E-18	2.00511E-12	0.019320078	0.20296804	0.162965787
Card11	1	4.32E-15	1.47151E-08	-0.020986613	0.24493456	0.162453364
Creg1	1	4.76E-03	6.53473E-05	0.0089321	0.120123148	0.162154981
Fam111a	1	2.47E-09	3.59173E-09	-0.007542499	0.16253364	0.161936729
Clasp2	1	9.19E-09	0.000755359	-0.052857331	0.206042207	0.161306036
Snx27	1	2.22E-13	4.76612E-06	-0.030874244	0.215466027	0.161240111
Dnase1l1	1	4.02E-18	1.90373E-14	0.010517874	0.175284167	0.161119366
Chst11	1	1.37E-26	4.96256E-08	-0.037604364	0.404341178	0.16095156
Lpxn	1	2.16E-18	2.10313E-11	0.01635061	0.205397567	0.16081507
Gm43305	0.006098853	1.00E+00	3.44372E-05	0.201481347	0.064255113	0.160393545
Npepps	1	7.45E-07	0.001587772	-0.041907141	0.193307696	0.159868512
Ncoa6	1	2.65E-04	0.001159142	-0.051518009	0.154159955	0.159524565
Ehbp1l1	1	2.02E-10	8.32992E-08	-0.039384608	0.180344874	0.159462183
Actb	1	3.62E-19	4.37615E-06	-0.026140954	0.279993307	0.159460289
Dglucy	1	1.00E+00	0.000505089	-0.028275604	0.086822027	0.159418128
Samd9l	1	8.91E-06	6.36693E-08	0.021728736	0.127290707	0.159179117
Ubap2l	1	1.36E-06	0.000533482	0.041715088	0.177415554	0.15883785
Top1	1	7.98E-06	0.027739429	0.066956237	0.201815727	0.157672062
Arhgap17	1	3.24E-05	0.040755453	-0.019055149	0.205855411	0.157656728
Frmd4a	1	7.55E-13	0.000105985	0.025431947	0.25386549	0.156980092
Atp6v1h	1	5.50E-09	5.02867E-05	-0.044671718	0.185296131	0.156708304
Jmjd1c	1	3.82E-03	6.60236E-05	-0.03331707	0.136873188	0.156550186
Dennd1a	1	7.34E-11	0.002281313	-0.038224924	0.236895995	0.156438441
Map4	1	1.06E-14	0.00681166	-0.038806214	0.271994952	0.156176555
Il7r	1	6.04E-15	8.88128E-09	-0.007536655	0.215100532	0.155775223
Vps13c	1	2.72E-04	0.009829498	-0.05099	0.173777222	0.155749981
Pip4k2a	1	1.33E-25	0.167045009	-0.045485599	0.465217377	0.155471117
Dop1a	1	3.57E-01	0.000232026	-0.045273855	0.103235948	0.155434084
Rb1cc1	1	1.00E+00	0.005346941	0.014331704	0.071341893	0.154646036
Ptk2b	1	4.29E-16	0.073074719	-0.114706816	0.323767613	0.154612212
Foxn2	1	1.50E-11	4.25181E-05	-0.023175426	0.202868437	0.153759328
Ttc17	1	9.25E-05	0.001569428	0.040287797	0.15160509	0.153262665
Glipr1	1	6.97E-18	2.5855E-16	0.004875132	0.157313402	0.153050341
Prex1	1	2.12E-12	0.00249693	-0.003503156	0.253890263	0.152889424
Tbc1d1	1	7.40E-14	0.06093954	-0.073492786	0.300644394	0.152398355
Galk2	1	9.04E-12	2.00314E-07	-0.034062507	0.184853658	0.152288249
Atp6v1c1	1	6.33E-15	8.26493E-09	0.000925207	0.188364406	0.152214268
Impact	1	4.40E-16	1.03142E-08	0.035746681	0.195408848	0.152162629
Apol7c	1	1.76E-09	4.19086E-12	0.007962044	0.133410627	0.15174675
Cd48	1	5.44E-15	1.44509E-08	-0.016602873	0.198026663	0.151569036
Tecpr1	1	2.41E-09	1.15953E-07	-0.004015217	0.149496544	0.15154573
Rab7b	1	1.11E-26	1.8158E-12	-0.006764518	0.25055592	0.150521126
Gm38832	1	1.70E-05	1.83313E-15	-0.003141794	0.076006789	0.150270284

Eya3	1	1.92E-05	0.000147094	0.021868482	0.152166289	0.150172355
Mir142hg	1	5.05E-23	2.89247E-09	-0.006940257	0.249041713	0.149925047
Mycbp2	1	2.70E-03	0.005623375	0.063541813	0.159188062	0.149288078
Actr2	1	2.34E-22	0.000254693	0.001797696	0.298620665	0.148839863
Gm37168	1	4.35E-27	3.20719E-17	0	0.215935844	0.148818733
Trim14	1	3.07E-02	0.023909236	-0.0288194	0.150205152	0.148811012
Dclre1c	1	6.94E-09	0.001011113	0.024318752	0.193045026	0.148700888
Cep128	1	2.09E-04	0.001080517	-0.042755093	0.162585088	0.148567722
Nrp2	1	5.89E-23	5.90011E-11	0.014654067	0.253450044	0.148192373
Vps35l	1	2.71E-11	4.18436E-05	-0.017917517	0.195816415	0.148147633
Plekhm1	1	5.40E-12	1.14811E-05	-0.039077583	0.201639095	0.14797736
AU022793	1	1.21E-11	4.00261E-16	0	0.103225216	0.147958482
Arhgap45	1	2.45E-11	8.39931E-05	-0.072354879	0.219130448	0.14791024
Morrbid	1	4.83E-16	0.005783094	-0.002592748	0.321804115	0.147624058
Piezo1	1	9.43E-14	1.0256E-05	0.009782026	0.219136878	0.147541232
Nlrp1b	1	2.29E-09	0.004882424	0.043886971	0.218326144	0.147173878
Stxbp5	1	1.77E-04	0.000537606	-0.035105591	0.144306947	0.146868845
Wdfy4	1	9.92E-16	0.175446475	-0.04304478	0.328350356	0.146423024
Pdpk1	1	9.98E-05	0.000732021	-0.000364627	0.1482753	0.146388306
B4galt6	1	5.59E-17	1.20902E-07	-0.018866713	0.235782305	0.146348707
Slc38a1	1	1.32E-06	1.62803E-05	-0.039504866	0.152338219	0.145571299
Sgpl1	1	7.03E-11	2.52972E-05	0.012273907	0.196634099	0.145544224
Adam8	1	7.16E-19	1.49002E-11	0.000228148	0.201933806	0.145521582
CAAA0111!	1	2.88E-12	3.53566E-07	0.026725843	0.185658921	0.145503744
Pak2	1	3.46E-14	0.012851581	0.050765751	0.261167422	0.145288508
Notch1	1	8.10E-08	3.9155E-10	-0.000301649	0.119664303	0.145204854
Slf2	1	1.27E-09	0.001703194	-0.014426583	0.201309367	0.145090297
Itfg1	1	4.93E-11	1.0082E-05	0.056939011	0.178970774	0.145029611
Ogt	1	6.66E-09	0.030020677	-0.034562701	0.215197175	0.144853858
Renbp	1	1.50E-13	1.61569E-12	0.009552279	0.146948542	0.144828383
Micu1	1	5.97E-08	6.81021E-05	0.012679993	0.162989125	0.144826579
Srsf2	1	8.92E-01	0.007564666	0.067373785	0.102455661	0.14477603
Slc25a3	1	1.21E-02	3.40827E-05	0.095022391	0.104825026	0.144653996
Gclm	1	3.95E-02	0.000354012	0.029827695	0.117158478	0.144280281
Fbxl5	1	9.38E-16	5.87649E-07	-0.024172894	0.219849839	0.144068112
Msrb3	1	1.00E+00	3.5485E-08	0.014443679	0.024248236	0.143278465
Klf7	1	2.40E-13	0.009988096	0.010537672	0.258238821	0.14309743
Arhgef1	1	1.95E-10	5.20467E-05	0.017755368	0.186951078	0.143077902
Ptpn6	1	4.65E-16	5.19012E-09	0.02930125	0.185114115	0.143046477
Snx8	1	2.85E-15	4.54526E-05	-0.021149547	0.233168761	0.142748536
Clcn5	1	6.50E-13	1.90872E-06	0.037887129	0.201280979	0.142706947
Agap3	1	7.78E-09	2.50427E-06	0.029076934	0.159267832	0.14266272
Lnpep	1	9.97E-11	0.147735262	-0.083734044	0.256844897	0.14260769
9930111J2	1	2.50E-03	6.37301E-07	-0.00223549	0.096749632	0.142602334
Prkcb	1	2.69E-18	1	-0.089952889	0.409925755	0.142314841
Nsf	1	2.52E-10	0.008308292	0.028508086	0.22672021	0.142186618
Arpc1b	1	1.60E-12	1.64702E-05	-0.015011596	0.195474108	0.14190201
Haus8	1	1.73E-13	9.46666E-13	0.011336966	0.143032202	0.141747322
Rsu1	1	7.16E-11	0.012078868	-0.034980275	0.227820883	0.141735917
F630028O1	1	1.02E-13	3.3668E-07	0.012931059	0.194459652	0.14167408
Pik3cd	1	5.40E-16	0.557147887	-0.076006191	0.313634551	0.14165905
Parvb	1	3.84E-17	5.54429E-08	-0.014915082	0.207202988	0.14121074
Alpk1	1	2.08E-08	0.01208545	0.000566984	0.215660892	0.141201865
Tanc2	1	8.93E-03	1	0.041919506	0.224124534	0.141197399
Il15	1	1.98E-08	0.001466404	-0.004598682	0.200168249	0.141192943
Wwp2	1	1.32E-10	0.002103796	-0.043107887	0.208649501	0.140486343
Loxl3	1	7.69E-21	1.844E-13	0.01330045	0.180928093	0.140471393
Ptpra	1	2.65E-12	0.010364074	-0.04684255	0.232220816	0.140269446
Stx8	1	4.20E-08	0.104053858	-0.020464527	0.222667138	0.139884011
Lyn	1	5.86E-13	0.556788194	-0.040015555	0.313078571	0.13912079
Fes	1	4.92E-09	1.19608E-08	0.0325465	0.141717109	0.139088162
Pot1b	1	4.71E-05	0.018056989	0.009793416	0.165985042	0.138973456
Gm11099	1	1.64E-10	2.65974E-09	0.007244487	0.164630159	0.138920621

Vac14	1	8.17E-11	4.63023E-07	0.00292073	0.161671007	0.138451742
Snd1	1	1.00E+00	0.007526642	0.028624943	0.067362269	0.138007258
Vps13b	1	8.10E-04	0.000883685	0.005884245	0.135364871	0.137802984
Cfp	1	1.94E-10	4.69608E-09	0.064719841	0.139203922	0.137521493
Btbd1	1	5.42E-11	7.00315E-05	0.066384775	0.181209493	0.137369093
Slc26a11	1	9.96E-09	2.52063E-07	-0.010874988	0.137035775	0.137275281
B3galnt1	1	2.60E-03	2.48869E-09	-0.008527282	0.080574499	0.137135013
Sptlc2	1	2.17E-12	0.005887172	-0.015379796	0.227917359	0.137011047
Psme4	1	1.00E+00	0.007603699	0.010322508	0.097115133	0.136843186
Arhgap30	1	2.14E-12	0.003544163	-0.070319821	0.22525493	0.136743798
Robo1	1	1.00E+00	0.000533714	0.012903943	0.013401461	0.136075055
Elf4	1	1.22E-12	0.037336095	0.06041359	0.251042466	0.135968288
Enox2	1	5.16E-05	1	-0.052506725	0.21327693	0.135665998
C3ar1	1	5.29E-14	1.9764E-12	0.003487907	0.146853435	0.135414998
5031439G(	1	7.85E-17	0.014068521	0.028598192	0.269550369	0.135398966
Ttll3	1	3.20E-07	5.53984E-07	0.009803407	0.132432898	0.135292065
Cd300c2	1	9.00E-16	3.69059E-07	0.035883059	0.196450047	0.134978346
Prkcd	1	2.90E-18	0.002919422	-0.034523336	0.282623138	0.134948629
Ly6e	1	1.49E-07	7.57913E-06	0.018762936	0.141582911	0.134664807
Il6st	1	4.96E-08	0.022312315	-0.0388459	0.195213742	0.134484435
Pum2	1	3.19E-09	0.053327879	0.015522513	0.209632713	0.13434426
Pde1b	1	2.15E-11	3.75599E-10	0.030375087	0.143336697	0.133862201
Acer3	1	1.24E-14	1	-0.055183361	0.324489831	0.133313725
Eif1a	1	2.00E-06	1.45895E-07	0.002405809	0.111902827	0.133301483
Wdr11	1	6.30E-10	1.66757E-06	0.014484413	0.153563756	0.133090189
Akr1b10	1	8.42E-10	1.60205E-13	0.020219264	0.107452871	0.132892884
Arrb2	1	8.37E-06	0.000210716	0.021542794	0.140637369	0.132607766
Casp4	1	1.20E-06	6.42786E-08	8.79675E-05	0.115952709	0.132271079
Tpr	1	7.94E-04	0.068410342	0.100255861	0.153605697	0.132160709
Atr	1	2.86E-07	1.07871E-06	0.018995107	0.129452958	0.131431483
Apaf1	1	8.90E-14	2.91003E-05	-0.037591909	0.196138475	0.131405214
Fcgr4	1	1.42E-08	8.32328E-09	0.020324646	0.127401458	0.131231306
Ankfy1	1	8.03E-10	0.015926667	-0.035278212	0.198781317	0.130706479
Mgat4a	1	1.34E-08	0.005187116	-0.042586555	0.186902377	0.130666665
Aftph	1	6.56E-10	0.62599926	0.034812068	0.233763694	0.130656481
Slc25a36	1	2.60E-07	1.72156E-06	-0.011827212	0.13443476	0.130368909
Snx13	1	2.03E-05	0.145616901	0.01097292	0.178303128	0.130361681
Lcp2	1	2.52E-03	1	-0.161576766	0.191230676	0.129507916
Slc8b1	1	2.87E-04	0.512886329	0.092833414	0.169949332	0.129256567
Gm13391	1	1.58E-11	1.54175E-14	-0.002800393	0.106818744	0.129118089
Nck2	1	8.02E-09	0.000559001	-0.020896762	0.183100337	0.129086866
Nek6	1	1.31E-11	3.47126E-06	0.038024341	0.169949506	0.128640421
N4bp1	1	1.77E-15	9.05731E-06	0.05278302	0.210772	0.12794981
Ehd4	1	6.60E-14	0.000140156	-0.033740311	0.21676782	0.127797068
Orc3	1	6.80E-08	3.67118E-05	0.01394118	0.138007602	0.127466745
Kpna4	1	7.48E-11	0.396627138	-0.082547131	0.244356593	0.127385399
Abi1	1	1.75E-17	0.038911429	0.022008517	0.279364638	0.127341008
Nsmce2	1	1.58E-09	0.430494607	-0.013490239	0.233065064	0.127329496
Arhgap39	1	7.25E-08	0.233320041	0.037785413	0.211674077	0.127316084
Plgrkt	1	1.95E-11	2.18763E-05	0.009588541	0.164210315	0.12723299
Stab2	1	1.16E-03	0.015203231	0.00296659	0.125170996	0.127054639
Msn	1	2.33E-16	0.003485739	-0.075444157	0.25765308	0.12698095
Eea1	1	1.11E-03	0.780814731	0.021566614	0.15797988	0.126861283
Igf2bp2	1	6.01E-14	7.29813E-09	-0.004106553	0.173296351	0.126672523
Ecpas	1	1.00E+00	0.003520493	0.013781312	0.065952406	0.126503177
Spred1	1	1.22E-10	0.066003043	-0.031126889	0.219170121	0.126354857
Cdk11b	1	1.00E+00	0.008113783	0.044449393	0.072388831	0.126284504
A630001G	1	1.31E-12	5.11244E-07	0.005744279	0.163963197	0.126110621
D1Ertd622	1	2.52E-06	0.002623061	-0.04113965	0.152425914	0.126073252
Jaml	1	2.84E-06	4.03527E-07	-0.02095896	0.116341269	0.125801363
Col4a3bp	1	6.55E-06	0.197718458	0.017609185	0.166625313	0.125634499
Itch	1	6.43E-01	0.020097588	0.0502765	0.105822791	0.125491553
Mef2a	1	8.11E-10	0.109328628	-0.030923568	0.228238003	0.125301889

Eif5b	1	7.13E-05	0.441589142	0.057894044	0.164487392	0.125093861
Nxpe4	1	5.67E-05	0.000157511	-0.001017315	0.12477894	0.124692314
Riok3	1	3.65E-04	0.139471946	-0.039326779	0.145958135	0.124401599
Zswim8	1	7.40E-08	3.49223E-06	0.041234751	0.129422917	0.124393803
Ralgapb	1	1.42E-04	0.011219864	-0.016540153	0.1343367	0.124311657
Dpp7	1	4.48E-10	1.57061E-13	0.001286972	0.097804133	0.124176908
Exoc5	1	6.78E-08	0.000417186	-0.001521019	0.14642364	0.124119006
Galnt6	1	2.20E-20	4.32746E-10	0.041327933	0.195649099	0.12408048
Nagk	1	1.99E-11	5.33496E-08	0.034210106	0.141707412	0.123959539
Smg1	1	2.01E-05	0.005864138	0.014464271	0.156990111	0.123939515
Synj1	1	3.69E-07	0.214641088	-0.084035773	0.189037453	0.123872172
Cmip	1	4.48E-15	0.540870937	0.004841212	0.288313449	0.123769986
Mylip	1	2.89E-18	9.74094E-06	0.00163939	0.222956906	0.123446792
Tank	1	1.18E-07	0.08235545	0.001057673	0.198529721	0.123305221
Rps6kb1	1	2.42E-03	0.008423064	0.06758087	0.116994097	0.123043594
Arhgef2	1	1.63E-13	0.000382415	-0.006689425	0.19246054	0.12245237
Cd300a	1	5.42E-13	0.574352761	-0.087627107	0.270879995	0.122449408
Gna13	1	2.98E-13	0.005117749	-0.023941309	0.208598916	0.12243412
Mfhas1	1	4.45E-08	1	-0.010135522	0.224046649	0.122366259
Itgb1	1	8.43E-06	0.202323335	-0.097187888	0.170917339	0.122195831
Rasa1	1	6.10E-07	0.189111043	-0.02949223	0.190734747	0.122083356
P2ry14	1	2.12E-12	1.92057E-07	-0.008002387	0.170798921	0.121805591
Cd74	1	7.35E-18	0.000351224	0.044869253	0.256882715	0.121779798
Pkn2	1	1.72E-03	0.453531625	0.003268287	0.139771938	0.121730689
Rpl3	1	2.46E-12	6.10545E-08	0.042442789	0.1465776	0.121703723
Sla	1	2.66E-06	0.000897135	-0.036491507	0.144362899	0.121650466
Opa1	1	7.59E-06	0.001875317	0.012076668	0.133798843	0.121448357
Axl	1	5.62E-11	0.008096312	0.020472325	0.213112014	0.121420006
Map3k20	1	1.59E-03	0.07091346	0.017808107	0.14117506	0.121416409
Tlr8	1	3.95E-09	1.06005E-05	0.012290871	0.14394016	0.12140913
Tlr13	1	9.85E-16	4.95337E-13	0.000451241	0.139531627	0.120973729
2510009E0	1	2.11E-02	0.298579111	-0.062527848	0.143005234	0.12086092
Dnase2a	1	6.78E-07	1.36372E-05	-0.017879919	0.121866168	0.120685871
Ankrd11	1	9.26E-05	0.032400689	0.004126002	0.152647123	0.120640923
Agl	1	1.11E-03	0.002075731	-0.019142512	0.111549149	0.120627835
Fnip1	1	6.30E-10	0.888336609	0.005770882	0.229602037	0.120557328
Cyfip2	1	4.49E-09	4.71826E-07	-0.00522664	0.133056826	0.120505435
Jak2	1	9.94E-14	0.016968914	-0.019156466	0.231504487	0.120328777
Cdk6	1	1.17E-02	1	-0.040163055	0.171984766	0.12013949
Gak	1	1.23E-04	0.065373615	0.003700177	0.139453421	0.119962559
Lmbrd1	1	5.86E-03	0.035057303	0.079778004	0.115671879	0.119787816
Atp7a	1	1.53E-06	0.077103071	-0.013945838	0.173243251	0.119227979
Gm31718	1	1.90E-10	2.2128E-09	0.017315652	0.122452758	0.119103902
Gm12905	1	1.23E-09	8.93181E-07	-0.012345603	0.128432799	0.119066283
Asah1	1	4.80E-10	0.009282697	-0.009517967	0.182713625	0.118637139
Gm15489	1	2.45E-06	9.40322E-08	0.009710948	0.101339225	0.118569571
Hipk1	1	1.95E-06	0.023382162	0.047342146	0.151756857	0.118489539
Tnpo1	1	5.93E-06	0.82097911	-0.018276508	0.175823866	0.118290948
Cyp1b1	1	1.79E-05	2.28217E-13	0	0.056219042	0.118020195
Oxct1	1	6.23E-11	0.000826267	-0.028392801	0.181053607	0.118002831
Vwa5a	1	1.09E-09	4.00569E-06	-0.015360873	0.140773352	0.117999406
Gas5	1	5.24E-05	0.000292791	0.043558864	0.113555475	0.1178973
Fus	1	5.70E-08	0.553970679	0.030931984	0.202250611	0.117891596
Hacd4	1	5.12E-10	3.06441E-06	-0.033262085	0.148732187	0.117666342
Arhgap18	1	4.94E-09	0.257909122	-0.089036353	0.209168372	0.117622032
Eif2ak4	1	8.58E-08	0.000113582	0.030520399	0.139775541	0.11751813
Gatad2a	1	3.72E-04	0.273055184	0.031574515	0.150951062	0.117415172
Tmem164	1	4.07E-17	1	-0.09420713	0.347158929	0.117275917
Cd93	1	1.13E-11	8.34888E-11	-0.007685573	0.121717446	0.117220873
Snx2	1	5.09E-07	1	-0.03470439	0.197721713	0.117186754
Asap1	1	1.42E-12	0.604384259	-0.112571604	0.272308517	0.117077812
Lars	1	1.41E-02	0.003334805	-0.012944655	0.100551921	0.116786341
Rmnd1	1	5.98E-02	0.003643783	-0.040514667	0.095145391	0.115704332

Ctsh	1	2.63E-05	0.608215467	0.04977334	0.159622915	0.115640231
Birc6	1	1.00E+00	0.031699988	-0.005221601	0.0328503	0.115635698
P2ry12	1	5.85E-05	0.009328217	-0.031023583	0.1470498	0.115475004
Slc9a9	1	4.73E-05	1	-0.079040836	0.275667131	0.115320152
Swap70	1	1.64E-10	0.000874735	-0.011140721	0.16999703	0.115286263
Pign	1	8.46E-05	0.710469656	-0.03491636	0.149917512	0.115228178
Phactr2	1	2.26E-07	0.209050511	-0.045226246	0.210650691	0.114923015
Mirt1	1	1.48E-15	8.22831E-08	-0.01318047	0.18349719	0.114405612
Dnajc5	1	5.07E-06	0.184010531	0.018905037	0.157127193	0.11426382
Cotl1	1	5.20E-23	0.039494839	0.001587251	0.305428025	0.114121482
C5ar2	1	1.08E-09	4.0562E-11	0.004875132	0.107200419	0.113695325
Usp8	1	4.10E-05	0.052115569	-0.005963489	0.141348847	0.113445416
Zfand5	1	5.33E-04	0.097058378	0.003964052	0.135641207	0.113367511
Cd80	1	9.02E-09	9.34559E-05	0.024905951	0.142054726	0.113278098
Hnrnpm	1	2.13E-05	1	0.026547933	0.166441156	0.113162072
Wls	1	1.63E-17	1.06731E-06	0.016426037	0.188785923	0.113149963
Atf3	1	1.86E-19	2.4995E-09	0.008875212	0.184776108	0.113039985
Itprid2	1	1.22E-06	0.004377267	-0.007186497	0.140876544	0.11289433
Fli1	1	2.08E-11	1	-0.107475992	0.297140846	0.112872773
Mtdh	1	1.43E-02	1	0.013178962	0.146654514	0.112566458
Uhrf2	1	1.92E-05	0.144472881	-0.025046961	0.149462359	0.112565968
Pacc1	1	3.28E-11	1.64768E-09	-0.004507265	0.126629442	0.112195847
Fam13b	1	2.23E-03	1	0.010374048	0.155161938	0.112030667
Arrb1	1	8.88E-12	0.00036806	-0.012855272	0.175588299	0.111752505
Smc4	1	8.87E-06	0.000136494	0.029550061	0.123653787	0.111686763
Ppt1	1	4.74E-09	0.000461609	0.01745213	0.153314791	0.111473809
Stx12	1	1.65E-13	2.67183E-05	0.017952117	0.164796164	0.111459684
Tmem181a	1	1.52E-11	0.003354549	-0.010934953	0.174990619	0.11134783
Washc5	1	3.24E-09	3.12731E-06	0.002590138	0.120920354	0.11105552
Pdcd6ip	1	1.79E-05	0.145470765	0.029251377	0.141461977	0.111008058
Ermard	1	6.23E-02	0.013283074	-0.002883273	0.098402379	0.110954214
Parp9	1	3.52E-02	0.517334276	-0.010419434	0.12409181	0.110877149
Ptp4a2	1	1.80E-08	1	-0.052587368	0.21398307	0.110751717
Hps3	1	5.80E-07	0.090584221	0.042809514	0.16035919	0.110587693
Clec4d	1	5.83E-12	2.48442E-08	-0.001087525	0.137527389	0.110390101
Tmsb4x	1	4.56E-15	0.000334855	0.018677114	0.196415156	0.110346663
Lrif1	1	2.91E-05	5.63412E-06	0.009827721	0.100759955	0.110345037
A930007I1	1	8.74E-14	1.17327E-08	0.017846211	0.145498284	0.110192988
Cyba	1	2.55E-13	0.000591554	-0.014190165	0.179637462	0.110101485
Ankib1	1	4.16E-03	0.599717504	-0.025725042	0.130384142	0.109912184
Dis3l2	1	8.16E-05	1	0.030329812	0.170218418	0.10986775
Eif2s2	1	8.04E-03	0.006368168	0.007563035	0.099622714	0.109860672
Eml4	1	1.01E-05	1	-0.024971608	0.181029853	0.109631503
Vps26a	1	1.12E-08	0.000212766	0.028639693	0.144059283	0.109336281
Tsc22d1	1	5.92E-18	0.002463013	-0.006449469	0.237045936	0.109333955
Tmem65	1	2.44E-09	0.010457248	-0.005592583	0.181414894	0.109111511
Ston2	1	4.70E-02	1	0.056860863	0.157328377	0.109045549
Plek	1	2.18E-16	0.000384133	0.013481017	0.23424296	0.109016264
Tex10	1	9.27E-07	9.28779E-06	0.010010648	0.106226597	0.108512366
Polk	1	1.06E-04	3.91425E-05	0.014686095	0.095663514	0.108490704
Cop1	1	4.24E-09	1	-0.028115168	0.213454919	0.108446822
Plekha1	1	5.42E-15	0.000240159	0.03833825	0.190195556	0.10822687
Slfn2	1	6.90E-13	1.03556E-05	-0.001299018	0.168712701	0.108090866
Eif4a2	1	1.21E-02	0.060222276	0.024404172	0.104743721	0.108078226
Gdpd1	1	2.85E-09	6.17345E-07	-0.005826179	0.125684339	0.107626015
Plxna1	1	4.16E-12	1.08261E-06	-0.014091882	0.144697876	0.10746733
Tpd52	1	5.29E-20	1	-0.035461672	0.353962911	0.107310977
Ciita	1	1.08E-06	0.041634089	0.019925934	0.177343863	0.107230681
Kitl	1	9.91E-09	1.55793E-06	-0.009448147	0.134460463	0.107228704
Smc6	1	8.41E-06	0.004835004	0.009365542	0.122801491	0.107208102
Zfp516	1	5.29E-10	0.065393495	-0.002131524	0.195833249	0.107201683
Fam120a	1	5.73E-05	1	-0.002492958	0.169887421	0.107001336
Blvra	1	2.81E-09	0.000109637	-0.010815439	0.140621555	0.106767771

St7	1	1.22E-02	0.355545949	0.054430728	0.119247748	0.106620795
Plekho2	1	1.53E-14	0.018469523	-0.051632943	0.218373945	0.106617318
Asb2	1	3.02E-06	4.3534E-05	0.004182191	0.110695728	0.106479525
Lst1	1	7.93E-04	0.000772552	0.011884048	0.103469852	0.10642026
Glis3	1	1.00E+00	9.37386E-06	-0.018983175	0.05798546	0.106372915
Mapkapk3	1	3.50E-11	0.000154935	-0.019603045	0.158054555	0.106360153
Rab7	1	9.45E-07	1	0.059017459	0.196467904	0.106359637
Wipf3	1	1.00E+00	5.66746E-05	-0.013899385	0.023787193	0.106293889
Abi3	1	5.63E-11	0.008097162	-0.01645953	0.180334442	0.106225731
Rad51b	1	2.07E-03	1	-0.026439306	0.187144865	0.106189412
Edem1	1	1.83E-02	0.042838526	0.020331001	0.099683813	0.10615534
Ago2	1	1.47E-08	0.080285477	0.056380455	0.173954055	0.106029433
Sirt7	1	5.41E-02	0.00391421	0.016986493	0.086985298	0.106006875
Nrp1	1	2.26E-08	1	-0.100347467	0.253505481	0.10600095
Stap1	1	9.96E-10	1.56147E-05	-0.008270811	0.145970767	0.105816688
Ucp2	1	2.71E-10	1.06805E-05	0.033939421	0.137954285	0.105532956
Tmem189	1	2.50E-10	0.000658097	-0.029167282	0.156161957	0.105223871
Cdk14	1	1.90E-14	1	-0.087500069	0.404942044	0.105223151
Trappc8	1	3.15E-03	1	0.017227437	0.133994808	0.104880887
Vsir	1	1.51E-10	0.000866377	-0.018991063	0.159127436	0.104828175
Mcl1	1	1.18E-06	0.042299723	-0.056760276	0.150071006	0.104684284
Clec4n	1	1.30E-09	4.48061E-07	-0.00657616	0.131231704	0.104506949
Gnl3	1	3.59E-10	4.542E-05	0.043957179	0.139360589	0.104429194
Tpp1	1	8.73E-03	8.10381E-06	0.000173938	0.075182108	0.104287317
Asph	1	1.84E-04	1	-0.003635503	0.168112084	0.103251313
Ctdspl	1	1.00E+00	0.000679697	-0.011255784	0.014685491	0.103015968
C5ar1	1	1.85E-18	6.90514E-10	0.005898203	0.163440064	0.102775437
Zfp638	1	5.80E-03	1	-0.017260265	0.153493429	0.102759433
Tlr1	1	7.62E-14	7.36976E-09	-0.003773725	0.134092461	0.102734905
Mau2	1	1.15E-02	0.2871307	0.001313026	0.106763656	0.10238059
Angel2	1	1.06E-05	2.3493E-05	0.027922149	0.09963285	0.102351058
P2ry6	1	9.68E-12	0.002385843	0.014625817	0.178384128	0.102086366
BC025920	1	7.35E-06	1.54342E-08	0.004377343	0.085642189	0.102008714
Igf2r	1	2.26E-07	0.000725631	-0.007692148	0.127370035	0.101642468
Mark2	1	4.28E-06	1	0.031444262	0.177981091	0.10151184
Esd	1	1.39E-06	0.000195049	0.016940108	0.116393333	0.101479667
Grb2	1	1.33E-17	1	0.03575387	0.321988029	0.10140035
Clec9a	1	5.92E-04	0.5386677	0.008306681	0.13925772	0.101394204
Gtf2h1	1	1.95E-03	0.009433566	0.028120747	0.094711732	0.101315517
Parp4	1	7.27E-04	0.356848535	0.036459679	0.121404836	0.101241495
Ipo7	1	9.39E-07	0.011050661	0.036944107	0.132290113	0.101053217
Srxn1	1	1.87E-10	1.62161E-09	-0.003404487	0.101631415	0.10091746
Rpl13a	1	2.91E-05	0.00566584	0.016782498	0.12280309	0.100856728
Rab20	1	1.11E-07	0.034661223	-0.017442222	0.15586689	0.100849526
Neurl3	1	1.52E-05	0.006654763	-0.008602857	0.121671485	0.100829253
Nek9	1	1.09E-03	0.020302799	0.024698625	0.107474564	0.100784456
Myo1g	1	5.94E-08	5.0866E-05	0.021292295	0.122147993	0.100608386
Acap2	1	2.17E-07	1	0.053650881	0.209462757	0.10030507
Sf3b1	1	1.32E-05	1	0.052719272	0.173845846	0.10028177
Lrrc14b	1	7.94E-04	2.31548E-08	0.004133913	0.059973097	0.100223929
Alkbh1	1	4.80E-08	0.006414943	0.076894829	0.135089811	0.100164192
Stx7	1	1.51E-04	1	-0.049784573	0.146969518	0.10013653
Adap1	1	9.78E-11	0.003000559	-0.008298502	0.158210458	0.100122225
Mmp8	1	2.99E-11	4.78627E-10	0	0.112900361	0.100035213
Bcl2l11	1	2.47E-05	0.002818878	-0.024150643	0.116189137	0.09997833
Hltf	1	3.53E-04	0.143412851	-0.009196649	0.125598312	0.099971034
Mpp1	1	6.34E-08	0.000200819	0.02105428	0.123462204	0.099834584
Zzef1	1	5.06E-04	1	0.011721671	0.145693156	0.099825941
Cd9	1	7.99E-13	0.000558225	-0.011824048	0.177780786	0.099628854
Atm	1	2.40E-04	0.005967982	0.02521287	0.104284154	0.09954367
Eif4a1	1	4.65E-06	1	0.017654685	0.143732879	0.099485002
Taldo1	1	8.95E-11	3.66519E-05	0.030661646	0.13982061	0.099319295
Vps4b	1	1.06E-03	0.045615674	0.044667179	0.10445557	0.099135367

Sulf2	1	4.47E-07	0.00828669	0.031462523	0.138402635	0.099056428
Aak1	1	2.03E-04	1	0.026537562	0.145986411	0.098874613
Hmox1	1	3.62E-14	0.000226038	-0.012442648	0.198883176	0.098501992
Sestd1	1	5.53E-07	0.01550373	-0.039265327	0.134986573	0.098400671
Bcl2a1b	1	1.21E-12	7.86263E-10	0.004901552	0.116562275	0.098150969
Nrros	1	1.24E-18	1	-0.005001139	0.335959771	0.09792785
Gls	1	9.23E-13	0.028372947	-0.044904109	0.195377789	0.097740067
Rusc2	1	2.43E-03	1	-0.012739666	0.127289602	0.097702537
Sbno2	1	7.64E-09	1	0.001539407	0.188956997	0.097661596
Cap1	1	8.87E-14	0.015378227	-0.040107228	0.19032396	0.097557017
Trpv2	1	3.38E-12	3.17772E-07	0.009938813	0.123552876	0.096768574
Slc3a2	1	4.77E-10	5.18884E-07	0.010501235	0.109647152	0.096607943
Aph1c	1	7.92E-11	4.47369E-06	0.009126042	0.130238593	0.096417245
Tenm4	1	7.29E-10	0.01363788	-0.005595581	0.185754642	0.096411276
Adam9	1	5.74E-07	0.012931663	0.036592014	0.129972938	0.096328145
Scaf8	1	2.24E-02	1	0.051286189	0.131387334	0.095941226
Thap3	1	4.87E-03	0.001059264	0.021797124	0.082306144	0.095594598
Kif23	1	9.62E-05	0.00031199	0.012391453	0.105199211	0.095404467
Spopl	1	3.52E-04	0.92428099	-0.049496276	0.128246132	0.095390526
E230032D2	1	5.51E-04	1.74405E-05	0.002665595	0.081042788	0.095378369
Nipbl	1	1.89E-02	1	-0.010024545	0.128664462	0.095369262
Gm1123	1	8.93E-08	7.52602E-09	-0.00419713	0.091012877	0.095365745
Fndc3a	1	8.09E-03	1	-0.015785164	0.151482956	0.095234707
Sfswap	1	4.39E-02	1	0.005862951	0.114185024	0.095164459
Pip4p2	1	1.45E-05	0.000381568	0.004954006	0.100233033	0.094973647
Rin3	1	2.83E-07	1	-0.029662906	0.197687431	0.094963075
Heatr5a	1	6.87E-07	0.744954433	-0.024129804	0.17267439	0.094818169
Irf5	1	8.77E-12	0.002824846	0.009932345	0.161549714	0.094761977
Cyth3	1	1.46E-09	0.470449333	-0.017379286	0.184358496	0.094702716
Pdk4	1	1.00E+00	8.62485E-09	0.014015487	0.032685087	0.09466713
Dnajb14	1	1.28E-05	0.11750885	-0.033575355	0.135122179	0.094626325
Emb	1	5.76E-10	0.000365374	0.005370001	0.150387404	0.094594645
Tgfbi	1	1.35E-09	1	-0.006700323	0.205162985	0.094579789
Mtpn	1	3.99E-06	0.01047177	0.013183381	0.116801081	0.094457703
Sh2d4b	1	4.13E-09	1.75498E-08	0.006172929	0.106340118	0.094378307
Klhl6	1	3.70E-03	0.007060315	-0.039554208	0.092754814	0.094306999
Zfp991	1	1.90E-09	3.69657E-05	-0.003288056	0.122007857	0.094290628
Ctnnb1	1	6.66E-07	0.195160281	-0.016822599	0.142036383	0.094257784
Slc28a2	1	1.08E-02	8.70391E-05	-0.010945198	0.069738323	0.094062402
Cc2d1b	1	7.18E-06	4.05215E-05	0.005596373	0.096465846	0.093960658
Dctn5	1	3.65E-06	6.6766E-05	0.027720391	0.094688073	0.093958499
Rps9	1	3.40E-04	0.035644126	0.040558715	0.106452982	0.093881716
C1qc	1	1.50E-07	6.82822E-05	-0.000741412	0.110342673	0.093822305
Aen	1	1.64E-02	0.000445356	-0.013322157	0.07154461	0.093517406
Spidr	1	9.06E-04	1	-0.003748451	0.130766411	0.093499945
Hpse2	1	1.00E+00	8.48106E-09	0.002977147	0.007890337	0.09337728
Rnf115	1	5.77E-07	0.501866215	0.043472976	0.151192358	0.093220197
Guf1	1	1.63E-03	2.8872E-06	-0.007446646	0.070972683	0.093184831
Gdi2	1	2.03E-04	1	0.036928728	0.149080798	0.09315413
Dpp8	1	8.77E-03	1	-0.031483268	0.122807414	0.093038321
H2afj	1	1.47E-03	2.92707E-05	0.004475987	0.074085323	0.092935422
Atad2	1	2.22E-04	0.01811543	0.00951299	0.11941781	0.092923064
Vapb	1	5.31E-06	0.213003322	-0.021049565	0.129074223	0.092879916
Vezt	1	4.07E-04	0.240885887	0.013571615	0.118306963	0.092561582
Cd300lf	1	1.07E-03	1	0.029132076	0.148038026	0.09226877
Mfsd1	1	4.07E-04	0.000220943	-0.022326624	0.085542437	0.092206237
Ly9	1	1.64E-13	2.50057E-08	-0.002062632	0.123958155	0.092042779
Skp2	1	2.04E-08	9.15137E-07	-0.005197508	0.103371019	0.091872479
Ctsz	1	4.02E-15	0.010196117	-0.001284809	0.190873194	0.091803674
Ipo5	1	2.21E-06	1.0707E-05	0.035619054	0.091643601	0.091721818
Ccdc88a	1	1.61E-07	1	-0.072542545	0.214442794	0.091689873
Zfp397	1	3.76E-03	0.000939949	0.037231131	0.081348739	0.091530373
Trem2	1	2.92E-16	8.43576E-11	0.006917637	0.120872538	0.091483127

Adamdec1	1	1.25E-02	7.3322E-07	0.001568616	0.058989673	0.09147794
Atg7	1	1.31E-09	1	0.008997891	0.243343205	0.091254853
Srsf7	1	1.47E-02	0.066429961	0.041980548	0.090557474	0.091096885
Sipa1	1	1.61E-04	0.001347477	0.012513324	0.091754927	0.090869646
Itga5	1	1.78E-07	0.041694443	-0.018261639	0.146948015	0.090793308
Ncf4	1	1.21E-09	0.000167446	-0.00479429	0.131030708	0.090778059
Asxl2	1	5.54E-03	1	0.079423831	0.143734062	0.090737036
Snhg20	1	1.00E+00	0.025050234	-0.003785253	0.052054678	0.090736001
Junos	1	1.49E-02	7.6848E-06	-0.005091203	0.063433136	0.090722409
Gm26510	1	4.79E-07	0.091623202	0.034650701	0.132363902	0.090672813
Anpep	1	1.34E-13	4.91598E-05	0.028010845	0.15263307	0.090608596
Exoc1	1	1.34E-06	0.000218631	0.005620094	0.101747226	0.090401524
Rps8	1	1.38E-06	0.088278396	0.021500558	0.132925857	0.090293852
Fabp12	1	1.00E+00	3.2188E-08	0.00275872	0.02539989	0.090273229
Rab8b	1	2.64E-23	1	-0.091135618	0.361461592	0.090251594
Ankrd49	1	5.21E-04	1.79305E-06	-0.005128	0.066359742	0.090164333
Sorl1	1	1.18E-15	0.171994221	-0.014948484	0.261707589	0.09007804
Rnf149	1	1.19E-12	1	-0.027789982	0.210432336	0.090013304
Gm4258	1	9.38E-11	0.009101336	-0.023017208	0.167990954	0.089907674
Plxdc1	1	1.17E-09	0.000450364	-0.006359631	0.143731606	0.08976504
Arfgef1	1	2.41E-02	1	0.063901697	0.129118002	0.08971402
H2-T24	1	2.34E-06	0.000572739	-0.004854367	0.106204628	0.089620075
Adam19	1	8.46E-05	1	-0.007623827	0.152020846	0.089518807
Spic	1	5.51E-04	0.001400964	-0.003330818	0.092016705	0.089493619
Fcgr3	1	6.41E-04	0.000363775	-0.034508758	0.087652405	0.089315904
Atp6ap2	1	1.27E-07	0.000941457	0.028369737	0.115107889	0.089219494
Hnrnpf	1	1.61E-06	1	0.01307979	0.142666646	0.088937981
Adamts17	1	1.00E+00	5.8518E-08	0.002993816	0.026761069	0.088818943
Tug1	1	5.11E-01	0.002602818	0.00648405	0.060642343	0.088752278
Sh3glb1	1	2.22E-05	1	-0.019311022	0.159019932	0.088669312
Pacrg	1	9.04E-09	1.89726E-06	0.003551363	0.112981769	0.088298618
E330020D1	1	3.14E-03	0.003327749	0.012388053	0.076460326	0.08822397
Gm46224	1	2.84E-06	0.002707394	-0.003386177	0.11153863	0.088178444
Jdp2	0.039106775	1.24E-03	1	0.184375692	0.15104196	0.088053303
Pag1	1	6.45E-06	1	0.009176166	0.222327167	0.087970215
Bin3	1	9.69E-06	1	-0.038059672	0.138903709	0.087744885
Cyth1	1	8.73E-06	1	0.017672519	0.21023323	0.087709289
Rpl12	1	1.75E-09	1.43629E-05	0.032166273	0.109546224	0.087606467
C130050O1	1	1.07E-06	0.013302265	-0.013561089	0.118143735	0.087298065
Hspa4	1	4.89E-06	1	0.0407952	0.149727169	0.087204837
Ncapg2	1	1.96E-10	3.96345E-06	-0.026198216	0.117652445	0.087060652
Rnf180	1	4.04E-05	1	0.075526998	0.179269344	0.087046119
Lyzl4	1	7.84E-09	8.61177E-07	0.001289015	0.103842766	0.086993913
Gm2245	1	1.57E-16	0.001546966	0.01527674	0.197008365	0.086911919
Mfsd12	1	5.98E-16	4.49648E-08	0.008221278	0.138520064	0.086911279
Rngtt	1	9.78E-08	1	-0.010403333	0.181435758	0.086832942
Nars	1	1.43E-02	0.042547079	0.021114978	0.083565702	0.086801937
Wdr1	1	1.04E-09	0.004650121	-0.00848795	0.137340251	0.086711901
H2-Eb1	1	3.25E-11	0.002921267	-0.005745794	0.171942355	0.086672029
Susd1	1	9.04E-03	0.003521533	-0.007784349	0.079552962	0.086471668
Galc	1	1.56E-06	0.049992089	-0.002415751	0.127670924	0.086450155
Kif16b	1	9.85E-03	1	0.026375767	0.127575561	0.086288218
Napa	1	2.41E-02	0.019135366	0.013784225	0.077060809	0.086194129
Fkbp15	1	3.75E-02	1	-0.048771223	0.125312186	0.086106359
Rpl37a	1	8.77E-08	0.002396989	0.032318204	0.120297923	0.086085881
Gm19325	1	7.24E-04	1.11617E-07	0.008678468	0.055923561	0.085968755
Tcp11l1	1	3.14E-07	9.90647E-06	0.005819526	0.094301316	0.085873353
Apobec3	1	1.11E-08	1	0.061631186	0.175751682	0.085622358
Mybl1	1	3.59E-01	1.74306E-07	-0.003058449	0.033989887	0.085402868
Csf3r	1	1.37E-08	1	-0.033799412	0.275314298	0.085350294
Casp8	1	3.25E-04	1	0.008822519	0.135781094	0.085212505
Tom1	1	6.09E-05	1	-0.017457716	0.145845916	0.084735907
Soga1	1	8.37E-07	0.091676451	-0.047428279	0.126481762	0.084557577

Lamp1	1	1.75E-07	1	-0.028116209	0.150022334	0.084491385
Plekho1	1	1.35E-11	0.030469317	0.019213505	0.155613366	0.084449896
Blnk	1	7.97E-15	0.001791142	-0.006802236	0.192248826	0.08432757
Cenpc1	1	3.63E-06	0.006733779	-0.011748665	0.10518048	0.084287114
Ppp1r12c	1	2.57E-04	0.087715268	0.001611723	0.100152234	0.083997119
Cdc27	1	3.37E-05	1	0.02049617	0.118733386	0.083976453
Zfp994	1	8.82E-03	3.1903E-05	0.008359819	0.060431071	0.083955252
Dctn4	1	1.74E-05	0.094243132	0.04537005	0.111258018	0.083634348
Iqsec1	1	4.14E-10	1	0.024350659	0.189551816	0.083567959
Xylt1	1	4.88E-11	1	-0.052211904	0.262811588	0.083551701
Inf2	1	2.68E-08	0.020694097	0.006343466	0.1321381	0.083118578
mt-Co1	1	4.27E-04	1	0.006140459	0.114027731	0.083024174
Slc1a5	1	2.43E-04	1.737E-05	0.000164991	0.071466806	0.083009562
Cndp2	1	1.59E-07	0.00134382	-0.013312406	0.108010785	0.082758559
Clec4a2	1	1.71E-04	3.28324E-05	0.006961039	0.077533806	0.082700933
4933439K1	1	7.24E-04	4.0624E-06	-0.002054298	0.064996733	0.082689734
Dhx34	1	2.63E-07	4.08185E-05	0.004187693	0.090184591	0.082492621
Smad6	1	8.28E-07	0.433840966	0.033289512	0.141083844	0.082454681
Zfp62	1	1.74E-02	0.421577747	-0.007364969	0.085824202	0.082097497
Smarca4	1	1.09E-02	1	0.031045221	0.105216032	0.081815684
Gnptab	1	4.36E-09	0.534231697	-0.010672991	0.161983979	0.081645799
Tbcd	1	6.87E-03	1	0.021004694	0.10284572	0.081585636
Rnf166	1	3.28E-05	0.001917937	0.01886168	0.088162344	0.081369319
Psmc1	1	1.14E-03	0.231476687	0.017702306	0.098700912	0.081202039
Rab39	1	1.49E-03	0.651311983	-0.018357847	0.110774562	0.08118744
Nptn	1	4.40E-10	1	-0.064542111	0.226108229	0.081088408
Mdn1	1	4.40E-05	1	0.009822102	0.120154027	0.081054991
Xpo1	1	3.38E-05	0.917355788	-0.003977612	0.126618373	0.080853065
44447	1	2.80E-08	5.28674E-07	0.026068355	0.088506908	0.080670452
Tmem87a	1	2.45E-04	1	0.054108847	0.126229748	0.080448102
Stam2	1	2.98E-06	0.77578185	0.012090412	0.12779641	0.080437403
Tcerg1	1	3.42E-05	1	-0.007132274	0.136210818	0.079932675
Cep192	1	3.62E-06	0.098683126	-0.028015808	0.112389889	0.079708839
Stambpl1	1	2.99E-07	0.431380023	-0.032071668	0.137801642	0.079705859
Slc23a2	1	1.68E-03	1	-0.006960741	0.140987345	0.079614138
Zmym6	1	8.72E-04	0.039503958	-0.029722965	0.088138155	0.07959013
Trp53cor1	1	1.13E-01	1.58267E-05	-0.003086912	0.047378265	0.079568315
Atp13a2	1	6.79E-06	0.018682751	0.047502757	0.107043633	0.079561279
Celf2	1	3.44E-08	1	-0.051710851	0.218939526	0.079507729
Ptger2	1	5.97E-05	0.000390547	-0.012687944	0.080179573	0.079398921
Bcap29	1	1.39E-07	0.000622172	0.026777623	0.105465202	0.079393926
Rpl17	1	8.48E-08	0.005204381	0.04163898	0.109136742	0.079390999
Plaa	1	3.16E-02	0.796202639	0.01927766	0.085042316	0.079257061
Lrrc27	1	7.07E-09	8.80086E-08	-8.3345E-06	0.088666289	0.079212571
Mefv	1	2.36E-10	6.66087E-05	-0.01161345	0.118750787	0.07919471
Sft2d1	1	1.60E-05	1	-0.007789355	0.125446999	0.078919305
Htt	1	1.53E-07	1	-0.002806692	0.160112521	0.078849181
Mvp	1	4.95E-03	1	-0.025314042	0.105694149	0.078595976
Gstm2	1	1.93E-07	7.86491E-07	-0.003096662	0.082360524	0.078591071
Capg	1	6.59E-13	1.89982E-05	-0.004263806	0.125513347	0.078333849
Ehd1	1	4.37E-05	0.03246324	0.019199384	0.100368174	0.078287773
G2e3	1	2.76E-04	0.006496464	0.018941507	0.085524765	0.078263983
Ppig	1	1.79E-02	1	-0.001422806	0.111741358	0.078156822
Slc30a7	1	2.61E-05	1	-0.021330551	0.139228911	0.07812559
Appl1	1	3.54E-05	0.549727961	-0.0016458	0.113597901	0.078104329
Laptm4a	1	1.90E-04	0.207016091	0.020229479	0.100418491	0.078015207
Rpl10	1	1.61E-02	0.002017885	0.003020311	0.061976165	0.078001128
Cep170	1	5.05E-08	1	-0.014883893	0.153685707	0.0779692
Ssb	1	2.95E-02	0.687330816	0.050945864	0.084848062	0.077928484
Pbdc1	1	7.09E-04	0.06750718	0.004258141	0.093614336	0.077889031
Mknk1	1	5.11E-04	0.58447837	-0.009134281	0.103438778	0.077724876
Gnai2	1	1.29E-06	1	-0.034094996	0.145497597	0.077706432
Rpl13	1	4.44E-08	0.126180084	-0.017971242	0.128662874	0.077506853

Kcnk6	1	1.22E-03	0.020270144	0.003397168	0.085021691	0.077483866
44262	1	1.08E-03	1	0.024450142	0.140621997	0.077388539
Capza1	1	3.21E-03	1	0.00402316	0.127651673	0.077359314
Rhoh	1	5.43E-07	1	-0.016807914	0.143941881	0.077295933
Gnb1	1	1.24E-12	1	-0.018605594	0.245476265	0.077287985
Tyrobp	1	7.48E-06	0.005741594	0.004305867	0.099655971	0.076931877
Ralgapa1	1	3.19E-04	1	0.005450712	0.150760842	0.076827685
Vps26c	1	1.02E-04	0.038033084	-0.004764382	0.094355138	0.076781432
Gmeb1	1	5.04E-03	1	-0.016185826	0.095513285	0.076683097
Ptprc	1	5.44E-14	1	-0.196277254	0.336070194	0.076635566
Rhbdf2	1	9.74E-09	1	-0.000488109	0.159539479	0.076463039
Casp1	1	5.41E-05	2.73919E-05	0.002336016	0.070843125	0.076287129
Dennd1c	1	6.97E-06	0.005059812	0.040798165	0.097495392	0.076125196
Arpc5	1	3.45E-05	0.022199592	-0.007887464	0.097479271	0.075975501
Znfx1	1	1.43E-03	1	0.019737328	0.116281092	0.075974776
H2-DMb1	1	3.19E-07	0.00027074	0.026884137	0.093542338	0.0758769
Zkscan17	1	1.51E-02	0.006814623	0.012387754	0.07190224	0.07585083
Utp14a	1	2.80E-02	0.267946587	0.002563421	0.080429714	0.075838197
Cd53	1	4.10E-08	0.521213017	-0.034757554	0.14067615	0.075464274
Ccdc82	1	2.20E-03	0.202868172	-0.028380334	0.089827765	0.075284659
Tnks	1	2.33E-04	1	-0.045467911	0.158535037	0.075234787
Wdr41	1	2.76E-03	1	-0.03716818	0.121963966	0.075070565
Myo1c	1	4.14E-07	0.009529199	-0.026148781	0.107172958	0.075031066
44446	1	1.77E-06	1	0.03082648	0.142085277	0.074779519
Oasl2	1	7.12E-04	1	8.42431E-05	0.115837781	0.074639848
Rpl38	1	2.82E-06	0.200269296	0.0193131	0.118956951	0.074286376
Abce1	1	1.86E-05	0.065672007	0.033901446	0.098721653	0.074201035
Stx4a	1	1.90E-06	1	0.028325396	0.124729068	0.074117138
Jun	1	8.92E-05	0.001724112	0.006971416	0.080234049	0.074105007
Pfkfb3	1	2.49E-07	1	-0.01380676	0.182918265	0.07403519
Peli1	1	1.62E-03	1	0.024001995	0.174877499	0.073889332
H3f3b	1	3.46E-03	1	-0.003900321	0.100168446	0.073866553
Fam168a	1	2.69E-03	1	-0.00677955	0.162389156	0.073849234
Synj2	1	3.04E-09	0.000186655	0.010020115	0.108451392	0.073814908
Tmem267	1	1.96E-05	1	-0.000311523	0.139026623	0.073728273
Npm1	1	7.55E-07	0.005798166	0.039865177	0.097358329	0.073350048
Cdkn1a	1	1.00E+00	0.004862046	0.040821602	0.033977735	0.073257574
Prcp	1	9.93E-07	0.570133097	-0.020373411	0.128354426	0.073208489
Rxylt1	1	1.29E-01	0.037868673	0.00451499	0.062258891	0.07316902
Ap1s2	1	5.30E-07	0.031792579	-0.024866917	0.112588267	0.073135726
Tyk2	1	5.34E-03	1	0.01159486	0.095271988	0.072687136
Casp8ap2	1	4.92E-03	0.289322635	0.009041372	0.085452874	0.072687008
Rilpl2	1	1.95E-06	0.70322817	-0.022967218	0.127350891	0.07265326
Map1s	1	5.13E-04	0.001969978	0.025144164	0.073030626	0.072360813
Laptm5	1	8.25E-05	0.833968538	-0.006881657	0.116769432	0.07227318
Katnbl1	1	1.16E-02	0.23401591	-0.024610874	0.081907416	0.072224028
Tubgcp5	1	3.49E-02	0.39530154	-0.009349055	0.078924019	0.072186385
Snx20	1	1.97E-05	0.145986248	0.014073666	0.107237936	0.072119381
Gramd1b	1	5.01E-10	1	-0.028792438	0.205759804	0.07202412
Prdx1	1	1.03E-03	1	-0.03557855	0.159190143	0.071585985
Cfl2	1	6.21E-03	0.000828098	0.013313362	0.062654027	0.071442446
G6pdx	1	1.88E-05	0.006039117	-0.004962443	0.087500805	0.071294324
Psme2	1	7.21E-02	0.021113219	-0.010308839	0.062758021	0.071271895
Pfkp	1	1.06E-07	0.034826603	0.00975819	0.111664224	0.071088327
Gm26569	1	8.29E-03	0.000115178	0.001300182	0.059519055	0.071064396
Kmt5a	1	1.96E-07	0.075918955	-0.021563801	0.114767807	0.07105224
Supt20	1	2.10E-02	1	0.027421812	0.097539042	0.07078323
Flcn	1	7.88E-03	0.179300642	0.036260319	0.075747233	0.070628249
Dnmt3aos	1	3.04E-07	1.05056E-05	-2.50035E-05	0.078544021	0.070425416
Nsun6	1	2.59E-01	0.042042627	0.007748567	0.058958594	0.070359529
Rttn	1	1.26E-04	0.059533134	-0.017793832	0.091312496	0.070286836
Fcer1g	1	1.34E-04	1	0.040492607	0.110074088	0.070143467
Gnat3	1	1.19E-05	1.23238E-06	0	0.062911167	0.070135572

Gpr107	1	4.95E-04	1	0.003277523	0.113436461	0.0700905
Pold3	1	4.29E-05	0.054503654	0.020561546	0.092894151	0.069688113
Odf2	1	8.35E-04	1	0.001663002	0.115084965	0.069657168
Rplp1	1	1.23E-08	0.91943134	-0.007856856	0.136298792	0.069614396
Ndor1	1	6.59E-04	0.020748145	0.021008837	0.075344079	0.069514065
Uba6	1	1.66E-03	0.827430569	0.009942585	0.087155478	0.069415736
Treml4	1	1.10E-07	0.072197783	0.013791053	0.122099381	0.069331487
Atp8a2	1	1.00E+00	0.001194796	-0.019889311	0.037208589	0.069256189
Med15	1	3.99E-02	1	0.029186099	0.121364634	0.069223816
Rb1	1	3.26E-05	1	-0.029244821	0.164614067	0.069120278
Hps5	1	7.53E-03	1	0.026427341	0.085195611	0.069097446
C1qa	1	1.70E-03	0.2343022	0.017322287	0.087083715	0.068999726
Gm26944	1	3.37E-02	0.062213005	0.009893681	0.064541608	0.068885651
Bax	1	1.47E-05	0.000282296	0.021819802	0.076808453	0.068828317
Btk	1	1.23E-05	1	-0.027389109	0.137669839	0.068774798
Ino80	1	2.00E-03	1	0.048358918	0.133793515	0.068645225
Eef1a1	1	1.29E-02	1	0.006992322	0.105756272	0.068624302
Actg1	1	3.82E-08	0.00117991	0.004455888	0.100480747	0.068596633
Anxa5	1	8.64E-07	0.02115245	0.017833942	0.100332467	0.068591195
Ptbp2	1	2.80E-02	1	-0.034771045	0.105518395	0.068583403
Fap	1	2.20E-01	0.003593212	-0.008209171	0.050512648	0.068477833
Arl8a	1	8.98E-08	0.217147927	-0.002062558	0.113638029	0.0682418
Hexb	1	5.20E-08	0.015813251	0.009302796	0.109547538	0.068160812
Clec12a	1	2.70E-08	0.0113023	0.02084951	0.111322191	0.068151056
Lrch1	1	2.78E-06	1	-0.097522831	0.20983154	0.068059418
Dph5	1	2.36E-03	0.767234311	0.028731429	0.086926948	0.068018255
Rps12	1	3.04E-06	0.04720309	-0.001995314	0.098175563	0.067992103
Pi4k2a	1	2.48E-06	1	0.006941972	0.136121719	0.067949788
Nipal2	1	8.45E-01	2.27349E-05	0.001383527	0.026868645	0.067920383
Nin	1	2.03E-08	1	-0.005279983	0.143012777	0.067902443
Arhgdib	1	1.70E-07	0.034233004	-0.005404379	0.108117211	0.067820257
Rab32	1	3.60E-03	0.618065888	-0.001200076	0.085709232	0.067625014
Commd3	1	4.42E-03	0.000192379	0.013930725	0.054836988	0.067461543
Hnrnpll	1	8.14E-03	1	-0.012265968	0.105020588	0.067268557
Tnfaip3	1	4.22E-08	0.003671375	0.014051628	0.100919061	0.067176489
Gramd1a	1	4.20E-03	0.199768577	0.022370162	0.077663151	0.06690639
Usp14	1	1.03E-05	0.363034482	0.057781465	0.102926233	0.066870924
Nlrc4	1	2.12E-03	0.124130968	-0.015711907	0.07985464	0.066865072
Nfatc1	1	4.70E-02	1	0.009875853	0.103855679	0.066862815
Rpl6	1	2.24E-04	1	0.035205714	0.09608152	0.066800617
Cd200r4	1	3.41E-04	2.08451E-05	0.000527668	0.054722545	0.066560202
Snx10	1	1.08E-08	1	-0.057665608	0.214076023	0.066557576
Naa20	1	3.72E-07	0.017114064	0.031224265	0.098839757	0.066435191
Gm33103	1	6.07E-05	3.98308E-05	-0.001400196	0.066037	0.066433504
Cxcl16	1	6.51E-06	0.000137399	0.008469584	0.073071802	0.066420704
Tm9sf4	1	1.16E-02	1	0.028105256	0.090418125	0.066370654
Acp5	1	3.68E-02	0.047693351	0.02662037	0.064343111	0.066287265
Fbxw17	1	1.70E-03	0.021226869	0.011603118	0.073662003	0.066240551
Fuca2	1	6.33E-06	0.000660608	0.004782036	0.078187128	0.066141499
Sp140	1	4.21E-06	1	0.00824941	0.117241301	0.065938349
Gm20658	1	1.19E-03	0.000452517	0.004150582	0.061902912	0.065910496
Dot1l	1	3.80E-03	1	0.031743364	0.108168324	0.065886511
Arhgap15	1	2.01E-08	1	-0.148512686	0.334923085	0.065557316
Ppp4r1	1	3.83E-02	1	-0.082424742	0.126517083	0.065516946
Sirpb1c	1	2.26E-04	0.001878879	-0.027062358	0.078045906	0.065512735
Aldh3b1	1	3.68E-05	0.00741734	-0.011544388	0.079921472	0.065359131
Pdgfb	1	1.49E-10	0.009367326	0.023744207	0.130363814	0.065299709
Ifngr2	1	3.92E-11	0.783938301	-0.004883597	0.150494381	0.065223811
Etf1	1	1.57E-02	1	0.023526082	0.105956248	0.065221544
Mthfsl	1	3.31E-05	0.288223377	0.038145332	0.093653742	0.065134251
Camk1	1	2.53E-02	0.006404892	-0.009569976	0.055865209	0.0649899
Dtx4	1	9.91E-04	0.247894793	0.027935596	0.085549681	0.064941799
Rps19	1	9.93E-06	0.647546962	0.019473812	0.101391697	0.064866075

Bin1	1	2.00E-02	1	-0.01190703	0.083374103	0.064848767
Dip2a	1	4.89E-06	0.000586552	0.041470828	0.079944157	0.064804333
Gcnt1	1	3.17E-05	0.053351356	0.014439173	0.090968702	0.06449335
Gm20275	1	1.13E-14	0.479611473	0.034035588	0.173099013	0.064351115
Smpdl3a	1	8.52E-03	0.038457994	-0.007015294	0.068973037	0.064277968
Efhd2	1	1.10E-10	0.177332861	0.007201609	0.146071263	0.064240672
Flvcr1	1	1.60E-04	0.031774141	0.01054896	0.081649183	0.064202769
Vamp4	1	1.59E-04	1	0.024413607	0.098853559	0.064094838
Tlk1	1	3.79E-03	1	0.015007969	0.141870333	0.063851026
Tspan5	1	1.65E-04	1	-0.010720841	0.131719755	0.06368243
Cytip	1	1.49E-06	1	-0.058269396	0.131622052	0.063643454
Rab2a	1	1.25E-04	1	-0.005605865	0.156094072	0.063555651
Heatr1	1	3.75E-02	0.232931563	0.002135677	0.064304208	0.063509156
Topbp1	1	7.72E-04	0.891322165	0.009208045	0.089707626	0.063304225
Clic1	1	6.72E-07	0.36367699	0.013520994	0.111595523	0.06327154
Dnmt1	1	9.05E-03	0.157041145	0.020142808	0.073012743	0.063262187
Virma	1	9.43E-04	1	0.019512697	0.108090205	0.063246457
Ddx21	1	1.51E-04	0.179887612	0.040329628	0.087523168	0.063128376
Gm29685	1	3.72E-01	0.000272264	-0.004989772	0.033994391	0.063124435
Gm37233	1	1.88E-02	0.000117396	-0.008621021	0.044901575	0.063112818
Ascc2	1	3.26E-02	1	0.023570185	0.089477108	0.06307342
App	1	1.01E-13	1	-0.071521533	0.301014426	0.063044562
Relch	1	2.61E-05	1	0.057775517	0.158637883	0.062924802
Gm17749	1	6.71E-09	0.000133487	0.002171295	0.099965664	0.062886468
Itgb2	1	9.30E-13	0.714041116	-0.025971679	0.167828544	0.062746374
Gm43700	1	1.00E+00	1.65756E-05	-0.001391862	0.01229332	0.062715553
Tafa2	1	1.00E+00	0.014038863	0.026154293	-0.004775578	0.062692816
Gbe1	1	6.54E-04	1	0.13156707	-0.142277253	0.062687009
Ctdp1	1	1.27E-03	1	0.013202553	0.088324522	0.06268666
Otud4	1	3.56E-02	1	0.053586601	0.084926733	0.062622323
Sipa1l2	1	1.27E-03	1	-0.057028613	0.123912926	0.062511808
Slc7a1	1	2.15E-04	0.010800242	-0.007352833	0.080041056	0.062493932
Sh3gl1	1	5.87E-04	0.843040036	0.004681785	0.086046573	0.062367703
Hoxb4	1	1.13E-02	0.004202075	-0.003454494	0.054196394	0.062360521
Hacd3	1	8.12E-04	1	0.048994248	-0.096850468	0.062178136
Marcks	1	3.20E-11	1	0.003812446	0.155251137	0.062172771
Arl6ip5	1	4.28E-03	1	0.003313451	0.084031076	0.062108171
Ilk	1	8.01E-04	0.001062931	0.015615159	0.059535273	0.062015478
Itpk1	1	3.60E-02	1	0.022628845	0.112437115	0.061960297
Irak4	1	1.07E-04	1	0.01991654	0.102030451	0.06175805
Rc3h2	1	6.91E-04	1	0.026161524	0.115452785	0.061685272
Vwf	1	1.04E-04	0.000253547	0.002985481	0.063744016	0.06164283
Gm37273	1	1.00E+00	3.54886E-05	0.001383527	0.019892998	0.061495192
Rps16	1	2.36E-04	0.067276439	0.050606069	0.078135844	0.061467248
Nup153	1	6.41E-04	1	-0.032752309	0.104311597	0.060985836
Siah1a	1	3.47E-04	0.193248135	0.026378983	0.077692621	0.060980644
Gdf15	1	1.16E-02	0.006144839	0.009894962	0.054794297	0.060965124
Rps29	1	8.01E-08	1	-0.010196469	0.126417891	0.060837267
Pnpt1	1	8.24E-04	0.385176886	0.019888481	0.085451465	0.060574377
Tubgcp4	1	6.91E-03	1	-0.01607852	0.086639313	0.060270358
Plk2	1	2.79E-07	0.001962782	0.004681709	0.095953785	0.060253398
Ago3	1	2.56E-02	1	0.023806147	0.120438511	0.060147808
Gm19619	1	3.20E-01	0.000106745	0.001348773	0.032765825	0.060033616
B230219D2	1	4.43E-03	1	0.067700655	0.082552893	0.060029542
Mtpap	1	4.00E-04	1	0.028117615	0.087673642	0.05987066
Plaur	1	3.47E-11	0.112578405	-0.005680999	0.140305219	0.059853418
Kif3b	1	2.39E-04	1	0.046935682	0.104154712	0.059831074
Pik3r6	1	1.35E-04	1	-0.016801273	0.106606709	0.059756048
Exoc2	1	2.23E-04	1	-0.016500323	0.131538751	0.059586083
Syncrip	1	2.03E-02	1	0.012367177	0.114257387	0.059406174
Cwc27	1	3.14E-02	1	0.020026906	0.118206348	0.059359106
Spint1	1	3.51E-09	1.86455E-05	0.001383527	0.083404289	0.059331679
B4galt5	1	6.16E-08	1	-0.080225082	0.235207139	0.059296534

6330562C2	1	4.45E-05	0.01697979	9.68685E-05	0.074843216	0.059295352
Tfe3	1	1.36E-02	1	0.004086025	0.073612004	0.059210927
4930503L1	1	1.31E-03	0.015101615	-0.007916599	0.063368314	0.059185866
Grn	1	3.13E-06	1	0.010121385	0.13085995	0.059170227
Relb	1	4.65E-03	1	-0.008045388	0.087539586	0.05916508
Rps27a	1	6.75E-04	1	0.017370043	0.089547832	0.058865273
Mmp14	1	7.70E-07	0.04001455	0.02306762	0.093554532	0.05880575
Ccl6	1	5.86E-05	1	0.001863753	0.103536668	0.058686245
Brox	1	2.53E-04	1	-0.013732291	0.087990693	0.058670526
Smarcad1	1	2.08E-02	1	0.009480552	0.093692529	0.058525023
Pgs1	1	2.85E-04	1	0.032694033	0.087978132	0.058505481
Il18bp	1	2.42E-04	0.035401802	0.038534697	0.074151834	0.058116774
Dnajc16	1	8.13E-05	0.006923988	0.031895036	0.070346262	0.058037357
Gart	1	7.00E-04	1	0.018143421	0.076926146	0.058016417
Plekhg1	1	2.84E-04	1	-0.052146499	0.138789423	0.057968037
Brd7	1	2.25E-02	1	-0.003682355	0.087245307	0.057760208
Tet3	1	1.96E-02	1	-0.105182157	0.164802629	0.057681103
Rpl28	1	2.11E-03	0.310331508	0.007902988	0.074013896	0.057563334
Npl	1	2.24E-01	0.023887208	-0.004208923	0.04829335	0.057532887
Gngt2	1	1.69E-05	0.194176366	0.02341114	0.088585391	0.057522325
Lin54	1	3.42E-02	1	0.020916529	0.072255203	0.057471808
Hsf3	1	2.66E-03	9.2992E-05	-0.003597911	0.047221135	0.057467031
Wdr36	1	2.37E-02	0.204797391	0.008270182	0.062705505	0.057400258
Eef1g	1	1.57E-02	1	0.000438643	0.075131891	0.057395295
Zcchc2	1	2.38E-02	1	-0.018695684	0.111874149	0.057371091
Slc39a11	1	4.62E-02	1	0.019765794	0.126047182	0.057331486
Capn2	1	2.96E-03	1	-0.007306449	0.087316943	0.057289252
Adam17	1	5.31E-05	1	-0.011294349	0.159659029	0.057252392
Trmt2a	1	9.74E-02	0.009372464	0.008945828	0.047461683	0.057029913
Etv6	1	1.06E-03	1	0.025191916	0.202221596	0.056815459
Spsb4	1	1.28E-04	1	-0.011843878	0.110362076	0.056652606
Map3k8	1	2.21E-07	1	0.005349559	0.130195312	0.05609247
Sae1	1	2.45E-02	1	-0.011700969	0.082079736	0.055918856
Ap4e1	1	3.83E-04	0.768514199	0.039233097	0.077511549	0.055896788
Adamts10	1	1.10E-02	0.23779723	0.009991413	0.062304706	0.055885982
Zfp993	1	3.27E-07	0.16353292	-0.012254788	0.09537778	0.055703429
Thumpd3	1	8.61E-04	1	-0.001552587	0.090464914	0.055649324
Ncl	1	2.55E-03	1	0.049894477	0.101720585	0.0555854
Orc4	1	9.49E-04	1	0.018480381	0.093770192	0.055465776
Nlrp1a	1	4.11E-03	0.206092747	-0.004398006	0.063205538	0.055278486
Rps26	1	1.53E-06	0.124984867	0.029811486	0.094505485	0.055255238
Ocrl	1	1.50E-02	1	0.015645443	0.081087569	0.055112779
Rps20	1	1.43E-03	1	-0.010801991	0.085052565	0.055045798
Atp6v0c	1	6.26E-04	1	0.020220353	0.086856368	0.055044704
Gadd45b	1	1.97E-03	0.000655781	0.005482686	0.054315494	0.055012398
Rps6ka3	1	2.33E-11	1	-0.032691081	0.220735005	0.054823731
Trim35	1	3.04E-06	0.055682202	0.018514702	0.083480913	0.054790377
Pip5k1c	1	3.44E-04	1	-0.009059749	0.09920142	0.054703548
Rpl23	1	2.70E-02	1	0.019826392	0.080452761	0.054618843
Gm6209	1	1.84E-02	0.014541215	-0.000844066	0.055550229	0.054374137
H2-Aa	1	1.27E-08	1	0.010070464	0.156759832	0.054292449
Chmp3	1	1.87E-02	1	0.037460667	0.086153915	0.054224965
Disp1	1	3.25E-05	1	0.008605606	0.09529775	0.053817696
Zranb3	1	4.75E-02	1	-0.016281218	0.074751873	0.053746984
Pitpnm1	1	3.18E-03	0.181399186	0.026488242	0.066320622	0.053491778
Ostm1	1	3.63E-03	0.438246347	0.008796673	0.065208622	0.053091533
Pawr	1	4.26E-02	1	0.061907904	-0.045771253	0.052924463
Cmpk1	1	1.64E-04	1	0.043446732	0.088410231	0.052826669
Glg1	1	1.50E-03	1	0.01012477	0.139280654	0.052778038
Rpl8	1	1.14E-05	0.712007269	0.034133947	0.090068688	0.052741139
Id2	1	1.05E-02	1	0.011669079	0.086262162	0.052711613
Rnf216	1	2.39E-02	1	0.037760062	0.145028029	0.052631408
Dram1	1	1.78E-07	1	-0.021123339	0.118764593	0.05257716

Bsn	1	1.00E+00	0.003692535	-0.004175585	0.027778777	0.052404081
Lrrc25	1	3.26E-05	1	0.009507148	0.100055693	0.052392934
Sult2a2	1	1.00E+00	0.001589607	0	0.011484584	0.052359772
Vps18	1	2.60E-04	1	-0.003564498	0.080095953	0.052278232
Slc36a1	1	3.49E-02	1	0.023677502	0.067083046	0.05225816
Cdc42se2	1	1.13E-07	1	0.000305345	0.179348662	0.052140217
Daglb	1	7.62E-06	1	-0.033060463	0.132649539	0.052085603
Gm42711	1	1.00E+00	0.000255102	0	0.007204534	0.052081683
Flrt2	1	5.58E-08	0.143489743	-0.004915198	0.115543717	0.051802963
Bin2	1	1.85E-03	1	-0.005893844	0.111786854	0.05164516
Aatk	1	7.46E-04	0.458967223	0.022120708	0.073667544	0.051622821
Fabp7	1	6.83E-02	0.012700378	-0.007815169	0.046267739	0.051513677
Tspan4	1	3.01E-02	0.032278296	0.015340672	0.049219795	0.051484472
Fgd3	1	8.33E-06	1	-0.029157143	0.108718794	0.051363201
Rap2b	1	1.51E-03	0.118824705	-0.013751853	0.063754313	0.051182554
Traf3	1	1.83E-05	1	-0.07504833	0.180478615	0.051135551
Smad2	1	1.15E-02	1	-0.019863169	0.112673179	0.051070714
Sntb2	1	2.17E-03	1	-0.007321701	0.112370857	0.05105127
Ap1s1	1	5.43E-03	0.030543628	0.018337947	0.054226129	0.051030618
Dync1i2	1	7.03E-03	1	0.004730051	0.105369525	0.051025389
Pip4p1	1	1.16E-03	1	0.030949035	0.084157312	0.051017844
Kdm7a	1	8.10E-10	1	-0.011050941	0.223650403	0.050949952
Zmat3	1	1.00E+00	0.049992089	0.005750254	0.035025466	0.050907431
Rack1	1	7.26E-04	1	0.013850901	0.084397266	0.050800105
Pla2g15	1	3.57E-03	0.448307626	0.012338285	0.066702469	0.0507729
Gm29282	1	3.06E-02	0.005117749	-0.007230575	0.046571004	0.050742822
Vta1	1	3.97E-04	1	-0.004762846	0.092196516	0.050619026
A230028O(	1	1.12E-04	0.000701088	0	0.054152242	0.050544746
Golph3l	1	9.80E-04	1	0.037122887	0.081049438	0.050524102
Rnaseh2b	1	6.13E-05	0.043819526	0.010932602	0.0665177	0.05046102
Npc2	1	8.08E-05	1	-0.033830293	0.116720664	0.050455767
Dubr	1	6.78E-06	0.007944135	0.007668607	0.067241429	0.050441423
Plau	1	8.30E-08	0.003681816	-0.003606245	0.090537243	0.050319169
Gm45606	1	1.91E-01	0.00187717	-0.006967644	0.03707369	0.050305366
Dennd2a	1	4.22E-09	0.019070466	0.012347424	0.097045785	0.050100831
Mllt6	1	3.48E-02	1	0.006802849	0.072713289	0.049901169
Vdac2	1	1.94E-04	1	-0.008414581	0.100180433	0.049883421
Gm16897	1	1.00E+00	0.001259606	0.004133913	0.028475976	0.049876193
Naip2	1	1.62E-04	1	-0.013074103	0.115405708	0.049841673
Nup88	1	4.24E-02	1	0.031146422	0.061592708	0.049799561
Agtpbp1	1	1.76E-04	1	0.030541555	0.11446875	0.04978507
Ninj1	1	4.40E-05	0.007722029	0.03275923	0.063877254	0.049673344
Plxdc2	1	6.60E-03	1	-0.073374603	0.141883003	0.049629645
Plekhf2	1	5.24E-04	0.972956865	0.022049396	0.071043553	0.049513473
Snx29	1	1.38E-02	1	0.009692421	0.148305308	0.049442419
Mdc1	1	3.63E-02	0.246199251	0.023259628	0.051150087	0.049298368
Cstb	1	1.21E-06	0.00155332	0.011834441	0.067061927	0.04916208
Zfp984	1	6.67E-05	0.704178128	-0.004630211	0.077717253	0.049102576
Ywhag	1	2.02E-03	1	0.000271699	0.120549261	0.049059161
C9orf72	1	3.35E-02	1	-0.008649675	0.086225605	0.048973845
Patl1	1	4.53E-03	1	0.044901333	0.088146622	0.048764709
Cyp2a4	1	4.24E-08	0.001023182	0.001383527	0.074917354	0.04863054
Snhg1	1	7.10E-04	0.17565154	0.012024167	0.062892083	0.048627195
Rala	1	2.00E-02	1	0.009823906	0.095731073	0.048533072
H2-Ab1	1	1.93E-06	1	0.001000185	0.110654631	0.048408523
Lmo7	1	4.09E-04	1	-0.026134864	-0.047902822	0.048278677
Rpl5	1	4.31E-04	1	-0.00651002	0.074535608	0.048163191
Pwwp2b	1	2.05E-05	0.00639687	0.005710864	0.063367097	0.048158102
Rpl26	1	1.22E-02	1	0.024480551	0.066210924	0.047986237
Lmo2	1	9.24E-06	1	0.005460039	0.086979218	0.047961423
Slc48a1	1	7.39E-03	1	0.004144604	0.065274698	0.047948771
Ubl3	1	2.56E-05	1	0.025982647	0.164433287	0.047819248
Dtx3	1	4.59E-02	0.009952093	0.001854897	0.042265121	0.047774962

Ahnak2	1	6.00E-05	0.012672075	-0.001400196	0.069367434	0.04768307
Pdss1	1	4.31E-03	1	0.008895821	0.062814295	0.047555478
Fem1b	1	2.67E-04	1	-0.001424498	0.085684834	0.047498067
Nhlrc3	1	2.11E-02	1	-0.019056607	0.065924186	0.04746551
Sag	1	6.18E-02	0.004778937	0.014490762	0.038736712	0.04742896
Uap1l1	1	8.18E-05	0.000492971	0.018204282	0.051556615	0.047300984
Rab11fip5	1	3.31E-03	0.135404075	-0.004255471	0.057803551	0.047207857
Ankrd66	1	5.48E-02	0.000793609	0.002767055	0.032985182	0.046920225
Baz2b	1	1.87E-05	1	0.064477195	0.168529135	0.046683284
Sh3bp1	1	1.83E-02	1	-0.005007544	0.062766699	0.046628509
Mre11a	1	4.43E-02	1	-0.003036889	0.062241375	0.046407103
Cd83	1	1.42E-07	1	-0.007047743	0.12515451	0.046402748
Rnf19b	1	1.55E-02	1	0.003512006	0.113864068	0.046270077
Dusp3	1	1.98E-02	1	0.013339067	0.088556438	0.046269404
Chmp4b	1	9.52E-04	1	-0.013371241	0.119724437	0.046195395
Rpl30	1	1.61E-04	0.325461824	0.031513066	0.066425548	0.046176534
Il1a	1	2.87E-07	0.139127218	0.014996677	0.087067878	0.046087827
Cbr1	1	6.49E-01	0.002731816	0.018760412	0.030290258	0.046044668
Mapk6	1	2.75E-02	1	-0.002073725	0.071965996	0.045993966
Cnot6	1	2.19E-02	1	0.00575701	0.078888681	0.045929338
Gm15523	1	6.57E-03	0.057781312	0.012363525	0.052445012	0.045911875
Lrmp	1	2.06E-02	1	0.047289151	0.103434779	0.045876225
Prom1	1	1.00E+00	0.001087399	0.003576366	0.008544562	0.045866095
Cd68	1	1.32E-05	0.053472977	-0.007027401	0.069513849	0.04584661
Havcr2	1	2.52E-03	1	0.017711582	0.081046872	0.045551555
Pam	1	1.74E-05	1	-0.017490824	0.125658419	0.045499618
Fmn2	1	1.00E+00	0.040017332	0.002146291	0.019429121	0.045398257
B230303O:	1	1.17E-02	0.001390875	-0.005234619	0.039316985	0.045393048
Itgax	1	1.83E-08	0.791979558	-0.002737283	0.107821531	0.045261363
Adap2os	1	5.30E-10	1	0.036296698	0.122261952	0.045078196
Ifi30	1	2.68E-03	1	0.004543244	0.074494562	0.044986698
St8sia4	1	7.48E-04	1	-0.075840742	0.148254296	0.044728541
Sumf1	1	1.70E-03	1	-0.027267401	0.101948146	0.044723575
Irf8	1	4.80E-05	1	0.032161881	0.15393714	0.044626921
Taok1	1	3.84E-02	1	0.013421854	0.137093235	0.044465184
Clta	1	3.65E-06	1	-0.006751778	0.136828522	0.044310837
Ppp1r9b	1	3.51E-06	1	-0.011073463	0.112782311	0.044129108
Tmem165	1	5.11E-08	1	-0.000920179	0.120393165	0.044041528
Parm1	1	1.00E+00	0.01863126	-0.001391862	0.012744909	0.044010811
Sybu	1	1.00E+00	0.036723828	-0.000599219	0.012530025	0.043858131
Plcb2	1	9.82E-03	1	-0.003331933	0.07929074	0.04383724
Psen1	1	4.50E-04	1	0.00149558	0.122719829	0.043790215
Eif2s3y	1	1.27E-02	1	-0.019697599	0.104262568	0.043734485
Far1	1	4.10E-04	1	-0.043735166	0.109546358	0.043688246
Acot9	1	2.59E-02	1	-0.00189791	0.063204499	0.04324352
Rps10	1	3.31E-02	1	0.004624278	0.072974365	0.043236927
8030453O:	1	3.10E-03	1	0.00296771	0.070060031	0.04316834
Cog5	1	1.81E-04	1	0.013019509	0.163819136	0.043151528
Nlrp3	1	8.90E-03	1	-0.001110499	0.102204048	0.043050721
Txn1	1	8.81E-05	1	0.018747248	0.093010214	0.043032038
Diaph3	1	2.00E-02	1	-0.037021164	0.085605364	0.043023448
Lpin2	4.75701E-05	2.25E-19	1	0.235015178	-0.303075439	0.042963679
Zeb2os	1	3.32E-03	0.867339924	0.010799547	0.059233836	0.042963013
Cab39	1	4.88E-02	1	0.004914798	0.118603746	0.042936261
Pfn1	1	4.93E-05	1	0.006982942	0.080752981	0.04271878
Rpl27a	1	2.94E-04	1	0.002493031	0.079623434	0.042714951
Tmem154	1	3.90E-03	1	-0.014219763	0.073853689	0.042617985
Capza2	1	2.27E-05	1	-0.041475715	0.139302196	0.0425559
Serpine1	1	1.00E+00	0.012865055	0.001375193	0.014581875	0.042499503
Gm13710	1	8.77E-04	0.498772378	0.015250066	0.066147481	0.042321868
Slc36a4	1	1.32E-02	0.324571508	0.012323896	0.05102296	0.042173134
Gm40645	1	7.06E-03	1	-0.033865569	0.08979959	0.042109164
Galnt3	1	4.93E-03	1	0.019932983	0.067067475	0.04193464

Arpc4	1	1.78E-03	1	0.01555658	0.073754762	0.041855708
Itpripl2	1	1.68E-04	1	0.009748222	0.068396722	0.04185468
Prkch	1	1.14E-03	1	-0.007490398	0.130807712	0.041845115
Urb1	1	4.59E-02	0.104212164	0.027812546	0.041133032	0.041793495
Sec14l1	1	7.69E-04	1	0.007140817	0.115931482	0.0416645
Gm20663	1	2.28E-02	1	0.009364884	0.114917845	0.041554299
AB124611	1	9.75E-05	1	0.026574562	0.141468053	0.041521123
Cyld	1	4.39E-02	1	0.030303046	0.083148405	0.041453846
Eif2s1	1	2.33E-03	1	0.005848302	0.080796737	0.041369018
Nfya	1	6.13E-05	1	0.021032348	0.094228078	0.041297952
Milr1	1	7.68E-05	1	0.009152462	0.073041898	0.041290424
Gm21188	1	7.23E-09	1	-0.027551894	0.137845131	0.041235164
Cdh23	1	2.19E-04	1	-0.007565117	0.076186038	0.040876195
Il4ra	1	1.96E-02	1	0.018562966	0.072518755	0.040797013
Smox	1	1.25E-05	1	-0.00439999	0.107068104	0.040631833
Cd63	1	8.46E-07	0.058190491	-0.002808727	0.071706032	0.040548182
Hat1	1	7.84E-03	1	-0.001112155	0.087675689	0.040456355
Hgf	1	3.51E-02	1	0.002901931	0.073868804	0.040429124
Ptafr	1	2.17E-08	1	0.009805147	0.119223836	0.040248246
Malt1	1	1.30E-04	1	-0.035884443	0.154085729	0.040200343
Akap7	1	1.06E-02	1	-0.005923895	0.097506108	0.039886801
Scd2	1	1.34E-08	0.027309087	0.014032156	0.082392107	0.039748439
Snx1	1	3.26E-04	1	-0.026530237	0.115100857	0.039737585
Tmem168	1	6.04E-03	1	-0.006819829	0.066666362	0.039628324
Mcoln2	1	2.00E-06	1	-0.000383074	0.081212979	0.039459218
Ilf3	1	1.42E-04	1	0.00591128	0.091438564	0.039346772
Matk	1	7.97E-05	0.45309991	-0.005066199	0.06281651	0.039309218
Gpr35	1	1.19E-03	1	0.017300011	0.076108433	0.039136517
Plekha2	1	7.49E-04	1	-0.056525185	0.11748899	0.038999827
Tmem156	1	4.44E-02	1	-0.021589982	0.065812292	0.038919929
Clip2	1	2.95E-03	1	-0.011178222	0.064319328	0.03881311
Atpif1	1	1.36E-02	1	-0.007120497	0.056974953	0.038752686
Rgs10	1	2.15E-04	1	-0.00915003	0.085622836	0.038741247
Hcls1	1	3.08E-04	1	0.0098886	0.091329442	0.038663874
Rab3il1	1	8.11E-04	1	-0.013682345	0.066917395	0.038264444
Ipo13	1	3.88E-02	0.320639643	0.006250325	0.043194007	0.038056523
Cyb5a	1	4.72E-11	1	0.135246931	-0.175285307	0.038051328
Lyz2	1	3.07E-10	1	-0.015831076	0.150357595	0.038003558
Rpsa	1	2.29E-02	1	-0.012284786	0.085783513	0.03786291
Cysltr1	1	1.19E-02	1	-0.001518296	0.059186777	0.037827873
Rps6ka2	1	2.27E-02	1	-0.009924989	0.079233601	0.037763306
Emp1	1	8.63E-07	0.485739029	-0.004998107	0.089575736	0.037589764
Slc35f6	1	5.77E-04	1	-0.016180652	0.084395627	0.037515473
Mapk7	1	9.07E-04	0.065160965	0.006545179	0.047897317	0.03743057
Ola1	1	4.41E-02	1	0.034061622	0.089238349	0.037254286
Sema4d	1	3.00E-09	1	-0.055760412	0.161533615	0.037201462
Atp6ap1	1	8.81E-04	1	0.010024722	0.073261961	0.037188684
Rpl35	1	4.80E-03	1	0.016127023	0.05389855	0.037000746
Zfas1	1	6.84E-03	1	0.023297841	0.051787559	0.036975497
Inip	1	4.76E-02	1	0.010305768	0.052751122	0.036967314
Selenon	1	3.27E-02	0.04849775	0.009659688	0.036615987	0.036910535
Rplp2	1	7.45E-03	1	-0.022301799	0.075974042	0.036819217
Nfkb1	1	2.63E-04	1	0.022901942	0.192819159	0.036777547
4930469K1	1	4.50E-03	1	0.000391425	0.061715724	0.036663992
Actr10	0.539046327	4.51E-02	1	0.066910292	0.061775292	0.036620172
Eps15	1	1.73E-03	1	-0.02986832	0.131120519	0.036492208
Gm15964	1	6.59E-05	0.766851183	-0.001463413	0.061527321	0.036116192
Il21r	1	2.05E-06	1	0.021646745	0.076809008	0.036048392
Agpat5	1	8.37E-03	1	-0.034908874	0.073367327	0.035634206
Gm12708	1	1.99E-01	0.034958642	0.000800977	0.032130576	0.035574047
Il1r2	1	2.18E-03	1	0.001772625	0.078038419	0.035481754
Tfeb	1	7.77E-06	1	-0.050389041	0.179312837	0.035435964
Slc29a3	1	3.18E-04	1	-0.005070761	0.086504062	0.035424109

Hpf1	1	7.25E-04	1	0.014056056	0.069698266	0.035102376
Rgs19	1	2.48E-02	0.060875085	0.011021671	0.036682251	0.034980189
Coro1a	1	2.56E-03	1	-0.007244219	0.078540439	0.034897541
Gm8113	1	1.01E-03	0.614820078	0.00766719	0.053146856	0.034823438
Tmem202	1	2.09E-03	0.205655435	0.003311391	0.049277413	0.034617519
Gm13657	1	1.00E+00	0.035619034	0	0.02347356	0.034365284
Smim3	1	2.60E-05	1	-0.018630326	0.084981212	0.034312957
Cdt1	1	6.49E-03	0.057340169	-8.3345E-06	0.038855116	0.034304312
Tapbp	1	3.24E-03	1	0.028372839	0.080497112	0.034195817
Atp11a	1	1.91E-02	1	0.012915314	0.076982859	0.034070561
Arpc3	1	1.50E-02	1	0.012634824	0.069292122	0.033909994
Gm14636	1	3.22E-03	1	0.002585738	0.080603486	0.033773749
Rps28	1	2.28E-02	1	-0.006073733	0.076506243	0.033763041
Tkt	1	8.80E-03	1	0.018076896	0.082676089	0.033688223
Lpcat1	1	1.31E-04	1	0.008015824	0.072126057	0.033540205
Agps	1	1.23E-06	1	-0.041296448	0.153671445	0.033463455
Tifab	1	1.18E-02	1	-0.013838657	0.068030805	0.033202891
Patj	1	2.48E-02	1	0.024026464	-0.047095537	0.033042717
Csrp1	1	1.49E-03	1	0.009434105	0.064097462	0.032788956
Grk2	1	4.15E-02	1	-0.011792586	0.093348579	0.032760074
Olfr111	1	1.12E-04	0.138338029	0	0.054797874	0.032645922
Gm45894	1	2.82E-04	1	0.01116485	0.078078744	0.032644357
Ikzf1	1	6.89E-04	1	-0.088158265	0.18422077	0.032590465
Gm13684	1	3.05E-04	1	-0.012007423	0.067815173	0.032337192
Igsf8	1	2.33E-03	0.868412328	0.01874516	0.047697438	0.032287293
Gla	1	4.01E-03	0.229884914	0.003554822	0.043293735	0.032197165
Spg21	1	6.69E-03	1	0.003266023	0.06066473	0.03218642
Ralgds	1	2.24E-04	0.486124586	0.00849663	0.057543611	0.031885488
Mamld1	1	3.37E-03	1	0.009454233	0.06528456	0.031733975
Gm13067	1	3.91E-03	0.079388696	0	0.039030766	0.031665794
Ccl4	1	4.22E-02	0.240130491	0.006343421	0.037270013	0.031421275
Lat2	1	1.14E-03	1	0.002314711	0.067520321	0.031209297
Il1rn	1	1.36E-03	1	0.004360674	0.065367296	0.030997488
Card19	1	9.13E-03	1	0.001894526	0.045995261	0.030685659
Vat1	1	8.96E-06	0.129934218	0.002192839	0.05552522	0.030085302
Lysmd4	1	5.23E-06	1	0.049337406	0.11038954	0.029947022
Platr25	1	6.92E-03	1	-0.011002332	0.07335714	0.02994277
Rp2	1	4.85E-02	1	-0.022794835	0.082771946	0.029762908
Gm10521	1	1.54E-02	1	-0.005600785	0.044189562	0.02948898
Anxa2	1	2.13E-06	1	-0.014899806	0.100037707	0.029435839
4930430E1	1	2.84E-02	0.146900017	0	0.033531563	0.029277312
Syn1	1	2.35E-02	1	0.003673235	0.064963528	0.029247597
Fbxl2	1	4.42E-02	1	0.007013297	0.046432921	0.029159
Tm6sf1	1	8.34E-05	1	-0.000977068	0.150325565	0.028672055
Igfbp1	1	1.83E-04	1	-0.017938421	-0.069190337	0.028517948
Ssbp4	1	4.88E-03	1	0.007638489	0.054721375	0.028505016
Slc6a8	1	3.93E-02	1	-2.50035E-05	0.038955939	0.028222454
Galns	1	1.76E-02	1	-0.001286659	0.058351023	0.028168567
Utp6	1	4.97E-02	1	0.011924153	0.060045089	0.027647883
Add1	1	1.72E-02	1	-0.0151305	0.091858856	0.027384089
Prkx	1	3.68E-03	1	0.003971309	0.062408949	0.027381744
Tle3	1	2.42E-02	1	0.008091536	0.075597578	0.027321476
Spi1	1	1.35E-06	1	-0.002583204	0.188956563	0.026980255
Slc7a7	1	5.61E-03	1	-0.01770069	0.131081774	0.026809093
Khdrbs1	1	1.27E-03	1	-0.063383672	0.1407289	0.026708065
Qk	1	3.39E-07	1	-0.039584455	0.206285835	0.026674462
Faiml	1	1.88E-03	0.530625417	0	0.042995376	0.02663317
Cxcl2	1	1.27E-03	1	0.006343421	0.068262027	0.026515592
Tgif1	1	4.07E-02	1	0.015747408	0.059003589	0.026307707
Plxnc1	1	3.35E-02	1	-0.059855726	0.097882626	0.026202672
Suco	1	9.60E-03	1	-0.026888413	0.092561149	0.026106242
Gm13963	1	5.31E-03	1	-8.3345E-06	0.040931897	0.026075466
Acpp	1	3.70E-02	1	0.001911195	0.050664477	0.025946428

Nuak1	1	4.88E-02	1	-0.000800363	0.075687628	0.025370474
Lims1	1	7.53E-06	1	-0.073200964	0.182372801	0.024832313
Cdc42	1	2.01E-07	1	-0.035950754	0.193775431	0.024757503
Metrnl	1	3.23E-02	1	-0.01280746	0.065426292	0.024711833
Tnfaip8	1	1.13E-06	1	-0.061939706	0.179787396	0.02456981
Crlf3	1	1.21E-04	1	-0.044003406	0.135267682	0.024185545
Nfkbid	1	5.29E-03	1	-0.000465554	0.059485976	0.02414147
Adam10	1	1.62E-03	1	-0.065296669	0.155141521	0.024000449
Dapp1	1	4.57E-04	1	0.033463182	0.129714542	0.023958662
Pld3	1	1.82E-02	1	0.003882791	0.069586323	0.023867964
Arih1	1	2.79E-02	1	-0.016030382	0.129118698	0.023756068
Ttyh3	1	1.91E-03	1	0.035823117	0.071057925	0.023633271
Tnf	1	2.16E-03	1	0.011748711	0.048879125	0.023202438
Ostf1	1	5.51E-04	1	-0.028894536	0.128227987	0.023186641
Gm32364	1	7.91E-03	1	0.001375193	0.040403415	0.023137666
Anxa1	1	1.36E-02	1	-0.008056556	0.063452055	0.023115264
Insyn2b	1	5.37E-08	1	-0.011897583	0.1114443	0.023010141
Tusc3	1	5.57E-03	1	-0.012431543	0.070516495	0.022933848
Fabp5	1	6.57E-04	1	-1.6669E-05	0.05389233	0.022922946
Tubb6	1	1.75E-03	1	0.001902861	0.050300328	0.022896007
Tpm4	1	2.30E-02	1	-0.012130635	0.072775025	0.022610278
Gm7480	1	5.79E-05	1	0.00551744	0.054224339	0.02232104
Gm26520	1	3.29E-04	1	0.00277937	0.07608054	0.021766225
Fam102b	1	1.52E-02	1	-0.008609585	0.073171154	0.021267672
Ptpn7	1	1.81E-02	1	-0.00721249	0.044184976	0.020425584
Cdk17	1	1.42E-02	1	-0.016838811	0.143301192	0.02008315
Gm42962	1	9.19E-03	1	-0.023189297	0.066826728	0.018475312
Dennd4b	1	2.09E-04	1	0.008464546	0.094671854	0.018290297
Slc15a2	0.000267187	1.00E+00	1	0.087207086	0.031931454	0.017124019
4930404N1	1	3.23E-02	1	0.003311391	0.036061765	0.016070524
4933421D2	1	1.18E-03	1	0	0.042803218	0.016030683
Itgam	1	1.30E-05	1	-0.042965578	0.120963047	0.015957323
Pias1	1	2.36E-02	1	0.032322109	0.150092046	0.012461549
Slc27a1	1	3.93E-02	1	0.001619266	0.065027158	0.011859173
Reep5	1	4.02E-02	1	-0.025800322	0.066070042	0.010839805
Tgfa	1	3.20E-06	1	-0.004250282	-0.035014549	0.009387055
Gm13708	1	4.39E-02	1	0.007633852	0.042785561	0.00931369
Diaph2	1	4.30E-03	1	0.040718588	0.153314881	0.008159773
Il10ra	1	1.79E-02	1	-0.002160116	0.098068844	0.007846317
Pdzrn3	1	3.26E-14	1	-0.01941926	-0.143026029	0.003657818
Itih2	1	7.20E-38	1	-0.025925231	-0.237060527	-0.000920594
Cyp2c68	1	2.04E-13	1	0.002432122	-0.092879222	-0.001309637
4930455H(	1	8.25E-03	1	-0.001408531	-0.004175585	-0.001685441
Bcl2	1	2.83E-04	1	-0.043339756	0.090596326	-0.00172137
Runx2	1	4.42E-04	1	-0.029876881	0.106309263	-0.001839478
Wwtr1	1	4.58E-08	1	0.015803231	-0.050099165	-0.002109506
Itih5	1	8.72E-14	1	-0.012029206	-0.036425745	-0.002303073
Gm1600	1	8.25E-03	1	-0.002792058	-0.004175585	-0.002327604
9430002A1	1	8.25E-03	1	-0.004175585	-0.004175585	-0.003559591
4930524O(	1	8.25E-03	1	-0.001408531	-0.004175585	-0.00371359
Fam107a	1	8.25E-03	0.255114056	-0.002792058	-0.004175585	-0.004021587
C86187	1	1.00E+00	0.003618128	-0.001408531	-0.003423561	-0.004175585
Tcf24	1	1.00E+00	0.003618128	-0.001408531	-0.003683526	-0.004175585
3100003L0	1	1.00E+00	0.003618128	-0.004175585	-0.003683526	-0.004175585
Gm41168	1	1.00E+00	0.003618128	-0.004175585	-0.003683526	-0.004175585
C2cd4d	1	4.71E-01	0.003618128	-0.004175585	-0.004011566	-0.004175585
Gjc1	1	4.71E-01	0.003618128	-0.004175585	-0.004011566	-0.004175585
Unc45bos	1	4.71E-01	0.003618128	-0.004175585	-0.004011566	-0.004175585
Gpr12	1	8.25E-03	0.003618128	0.002742051	-0.004175585	-0.004175585
Sowaha	1	8.25E-03	0.003618128	-0.000599219	-0.004175585	-0.004175585
Gm12963	1	8.25E-03	0.003618128	-0.002792058	-0.004175585	-0.004175585
Gm17830	1	8.25E-03	0.003618128	-0.004175585	-0.004175585	-0.004175585
Gm47725	1	8.25E-03	0.003618128	-0.004175585	-0.004175585	-0.004175585

Snorc	1	8.25E-03	0.003618128	-0.004175585	-0.004175585	-0.004175585
Ugt2b38	1	8.25E-03	0.003618128	-0.004175585	-0.004175585	-0.004175585
Gm15401	1	7.71E-03	1	-0.005567447	-0.005403428	-0.00446329
Gm30373	1	7.76E-03	1	-0.005567447	-0.005307482	-0.004643456
Gm36435	1	1.06E-04	1	-0.005567447	-0.005567447	-0.004707371
B4galnt3	1	1.06E-04	1	-0.002800393	-0.005567447	-0.004797455
Vmn1r90	1	8.25E-03	0.254779404	0.002737176	-0.004989772	-0.004835774
Gm30363	1	7.71E-03	1	-0.00418392	-0.005403428	-0.004951453
A930038B1	1	4.70E-01	0.003618128	-0.002222718	-0.004825753	-0.004989772
Pgr	1	8.25E-03	0.003618128	-0.001413406	-0.004989772	-0.004989772
Scn4a	1	7.71E-03	0.548956108	-0.005567447	-0.005403428	-0.005105452
Gm44646	1	1.06E-04	0.548956108	-0.005567447	-0.005567447	-0.005105452
Gm11832	1	3.04E-02	1	-0.0014252	-0.00646725	-0.005149074
Gm36231	1	2.47E-04	1	0.006746937	-0.010054831	-0.005242194
Klf1	1	7.71E-03	0.064549675	-0.000607554	-0.005403428	-0.00525945
Gm41668	1	1.06E-04	0.064549675	-0.005567447	-0.005567447	-0.00525945
Gimd1	1	1.34E-01	0.00321725	0.003543028	-0.005239408	-0.005323365
Gm39157	1	1.00E+00	0.003197181	-0.001416865	-0.004815423	-0.005413449
Gm38079	1	1.35E-01	0.003197181	-0.002800393	-0.005143463	-0.005413449
Gm16567	1	7.71E-03	0.003197181	0.005735867	-0.005403428	-0.005413449
Hfm1	1	1.00E+00	3.50815E-05	-0.005567447	-0.004815423	-0.005567447
Atp1b4	1	1.00E+00	3.50815E-05	-0.005567447	-0.004979443	-0.005567447
Spock1	1	1.00E+00	3.50815E-05	-0.00418392	-0.005075388	-0.005567447
Vmo1	1	7.71E-03	3.50815E-05	-0.002800393	-0.005403428	-0.005567447
Gm45282	1	7.71E-03	3.50815E-05	-0.005567447	-0.005403428	-0.005567447
Hs3st6	1	7.71E-03	3.50815E-05	-0.005567447	-0.005403428	-0.005567447
Gm13021	1	1.06E-04	3.50815E-05	0.000201758	-0.005567447	-0.005567447
4930477N(	1	1.06E-04	3.50815E-05	-0.001991081	-0.005567447	-0.005567447
Gm42604	1	1.06E-04	3.50815E-05	-0.002800393	-0.005567447	-0.005567447
Gm45895	1	1.06E-04	3.50815E-05	-0.002800393	-0.005567447	-0.005567447
Gm10030	1	3.05E-02	1	0.000767639	-0.00630323	-0.005637238
A930002C(	1	1.18E-04	1	-4.16725E-05	-0.006795289	-0.005701153
Gm45349	1	3.04E-02	1	-0.005575782	-0.00646725	-0.005727321
Gm47882	1	1.67E-02	1	-0.001215107	-0.009466827	-0.005756526
Sytl4	1	6.36E-03	1	-0.003391277	-0.007599147	-0.005770944
Nudt12os	1	3.57E-02	1	0.00133352	-0.007435127	-0.005861027
Gm43691	1	1.78E-04	1	0.019541261	-0.009805196	-0.005948844
Gm29683	1	3.04E-02	0.424960521	-0.004192254	-0.00646725	-0.00600915
Gm8251	1	2.60E-02	1	0.000113537	-0.014571896	-0.006044316
Fbxl21	1	3.04E-02	0.421075949	0.001341855	-0.00646725	-0.006189317
4933421A(	1	2.83E-03	0.421075949	-4.16725E-05	-0.00663127	-0.006189317
Cadm3	1	7.70E-03	0.003192192	-0.00361458	-0.006217615	-0.006227636
1700024B1	1	3.04E-02	0.090251498	-4.16725E-05	-0.00646725	-0.006253232
C1ql3	1	1.38E-06	0.090251498	0.001341855	-0.006959309	-0.006253232
Gm21691	1	7.66E-04	1	-0.001433534	-0.007763166	-0.006323022
Mycn	1	1.06E-04	3.50815E-05	-0.00361458	-0.006381634	-0.006381634
Trim80	1	1.94E-01	0.012957627	-0.004192254	-0.006015394	-0.00640723
Rgs16	1	1.38E-06	0.012957627	0.012645169	-0.006959309	-0.00640723
Npnt	1	1.26E-03	1	-0.004208923	-0.008922934	-0.006456728
1810053B2	1	1.38E-06	0.001072167	0.009878115	-0.006959309	-0.006471145
Gm26684	1	5.36E-05	1	0.003526359	-0.008023131	-0.006477021
Gm49926	1	8.30E-01	0.012883751	-0.000615888	-0.006043265	-0.006497314
Ccdc151	1	1.92E-01	0.012883751	-0.0014252	-0.006207285	-0.006497314
Gdf2	1	1.91E-01	0.012883751	-0.006959309	-0.00630323	-0.006497314
Gm2350	1	3.04E-02	0.012883751	-0.006959309	-0.00646725	-0.006497314
Mup22	1	1.18E-04	0.012883751	0.013219385	-0.006795289	-0.006497314
Gm4755	1	1.18E-04	0.012883751	-0.004192254	-0.006795289	-0.006497314
Capn8	1	1.91E-01	0.001064714	-0.0014252	-0.00630323	-0.006561229
1700113B(	1	7.70E-03	0.064607249	0.002960478	-0.006795289	-0.006561229
G6pc2	1	1.75E-06	1	-0.005584116	-0.008187151	-0.006631019
4921539H(	1	1.00E+00	0.001057475	-0.005575782	-0.0057833	-0.006651312
Emp2	1	1.92E-01	0.001057475	-0.004192254	-0.006207285	-0.006651312
Cyp2c40	1	1.38E-06	0.001057475	-0.006959309	-0.006959309	-0.006651312

Ehhadh	1	7.50E-05	1	0.10756744	-0.073847024	-0.006737543
Gm50138	1	3.04E-02	3.74089E-05	-0.0014252	-0.00646725	-0.006805311
Csdc2	1	2.83E-03	3.74089E-05	-0.004192254	-0.00663127	-0.006805311
Serpina9	1	1.19E-04	3.74089E-05	-0.006959309	-0.006699344	-0.006805311
Csrp3	1	1.38E-06	3.74089E-05	0.004344005	-0.006959309	-0.006805311
Gm12473	1	1.18E-04	3.46163E-07	-0.004192254	-0.006795289	-0.006959309
Gm45470	1	1.38E-06	3.46163E-07	0.003534694	-0.006959309	-0.006959309
Gm32872	1	1.38E-06	3.46163E-07	0.001341855	-0.006959309	-0.006959309
Cd209f	1	1.38E-06	3.46163E-07	-0.0014252	-0.006959309	-0.006959309
Gm36011	1	1.38E-06	3.46163E-07	-0.003382943	-0.006959309	-0.006959309
A730071L1	1	1.75E-06	0.696490441	-0.005584116	-0.008187151	-0.007119183
Gm31600	1	1.38E-06	0.089406462	-0.005006441	-0.007773496	-0.007157502
Gm16291	1	7.61E-04	0.066440249	0.011018211	-0.007859112	-0.007247013
Gm44696	1	1.69E-05	1	-0.004208923	-0.009250974	-0.007252889
Gm48199	1	1.28E-03	1	0.004901552	-0.008731044	-0.007342972
Chrd	1	1.26E-03	1	0.001325186	-0.008922934	-0.007342972
Cstad	1	1.00E+00	0.001054654	-0.006389969	-0.004793271	-0.007465499
Odf3b	1	1.19E-04	0.001054654	0.014362941	-0.007513531	-0.007465499
Gm44096	1	1.38E-06	0.001054654	-0.00419713	-0.007773496	-0.007465499
Cttnbp2	1	1.00E+00	0.014569025	-0.006158332	-0.006779048	-0.007491095
Snai2	1	3.05E-02	1	0.004673374	-0.007903733	-0.007509694
Car5b	1	1.00E+00	0.014480381	-0.004200589	-0.006259118	-0.007581178
Zfp618	1	3.14E-04	1	-0.007014191	-0.013732446	-0.00765174
Gm45253	1	2.70E-02	1	-0.001441869	-0.00849895	-0.007741052
Myrf	1	1.18E-02	1	0.008787159	-0.012244639	-0.007762116
Gm12798	1	8.30E-01	3.46163E-07	-0.000855859	-0.006761507	-0.007773496
Gm15411	1	1.78E-04	1	-0.004208923	-0.009086954	-0.007804968
Ociad2	1	3.06E-03	1	0.001308517	-0.010694669	-0.007880634
Gm43598	1	1.69E-05	1	0.00409224	-0.009250974	-0.007895051
Gm15756	1	1.26E-03	0.730965162	0.001325186	-0.008922934	-0.007958966
Vim	1	2.19E-02	1	-0.046761131	0.061636624	-0.007981692
Gm595	1	7.61E-04	1.54323E-05	0.001568616	-0.007859112	-0.008043174
Fam25c	1	1.25E-03	1	-0.006406638	-0.009737121	-0.008194906
Gm16587	1	1.75E-06	4.21921E-07	-0.004200589	-0.008187151	-0.008197172
Prob1	1	1.79E-04	0.305579424	-5.83415E-05	-0.008991009	-0.008203048
D630039A(	1	9.44E-07	0.114890246	0.002943809	-0.009414993	-0.008266963
Gm47389	1	1.49E-01	3.46249E-09	-0.008351171	-0.007367053	-0.008351171
Cyp21a1	1	1.83E-08	3.46249E-09	-0.005584116	-0.008351171	-0.008351171
Zc2hc1c	1	9.44E-07	0.114268545	-0.006975978	-0.009414993	-0.008357046
Epha4	1	1.00E+00	0.014408837	-0.003631249	-0.00766131	-0.008395365
Slc39a2	1	1.48E-01	0.014408837	0.002712172	-0.00818124	-0.008395365
Zfp354b	1	1.75E-06	0.014408837	0.002137957	-0.009001338	-0.008395365
Srrm4os	1	6.17E-08	1	0.017066779	-0.016758593	-0.008396057
Large2	1	6.51E-03	1	-0.006406638	-0.009573102	-0.008401241
Mtnr1a	1	1.80E-04	1	0.000510999	-0.00970925	-0.008465156
Tmem217	1	1.00E+00	0.037267114	-0.002825396	-0.006898956	-0.008511045
Sptlc3	1	6.63E-01	0.037267114	-0.004208923	-0.00800689	-0.008511045
Apol7d	1	1.28E-03	0.037267114	-0.004783139	-0.008731044	-0.008511045
Colgalt2	1	1.21E-03	1	-0.012763268	-0.01142324	-0.008616888
Fam71d	1	1.68E-03	1	-0.011151563	-0.011950382	-0.008630333
Six5	1	2.64E-01	0.010305828	-0.006975978	-0.008074965	-0.008665043
Gm14372	1	1.26E-03	0.010305828	-0.005592451	-0.008922934	-0.008665043
Wfdc2	1	5.33E-05	0.688064676	-0.009979545	-0.009651505	-0.008747557
Gm45044	1	4.77E-05	1	-0.007237494	-0.012583008	-0.008776762
Gpr135	1	1.77E-04	0.115431212	0.000510999	-0.009901141	-0.008787492
Gm15527	1	1.69E-05	0.00233212	0.001325186	-0.009250974	-0.008819042
AW495222	1	6.37E-03	1.53873E-05	0.000519333	-0.008317388	-0.008857361
Jchain	1	2.80E-03	1	-0.004681948	-0.009888018	-0.008934493
Dpf3	1	1.51E-15	1	-0.034805445	-0.066873178	-0.008975555
Mmp11	1	1.78E-04	5.24207E-05	-0.005592451	-0.009086954	-0.009036955
Tex12	1	1.00E+00	3.46249E-09	-0.003631249	-0.006937157	-0.009165358
Cys1	1	1.50E-01	3.46249E-09	0.001902861	-0.007893404	-0.009165358
Mup16	1	1.75E-06	3.46249E-09	-0.007781831	-0.009001338	-0.009165358

C1qtnf7	1	1.00E+00	0.002349264	-0.005019651	-0.007587324	-0.009209552
Eya2	1	1.22E-03	1	0.001547072	-0.010609053	-0.009214856
Tfcp2l1	1	9.17E-03	1	0.00049433	-0.011248891	-0.009284646
Fgfrl1	1	3.04E-03	1	0.004884883	-0.010790614	-0.009330535
Adgrf1	1	2.55E-08	2.08742E-07	-0.004208923	-0.009579013	-0.009435036
Itga7	1	1.22E-03	0.018682751	0.00016842	-0.009890811	-0.009504826
Lect2	1	9.48E-07	0.115790702	-0.006411513	-0.010947422	-0.009535004
1700018L0	1	8.99E-02	0.002334393	-0.007790165	-0.009245062	-0.009543145
Pcdh12	1	9.15E-02	0.002318112	-0.009173692	-0.008957226	-0.009633229
Gm47368	1	1.25E-03	0.0004102	-0.00502311	-0.009737121	-0.009697144
Tnfaip8l1	1	4.20E-05	1	0.022300922	-0.034155573	-0.009721618
1700045H1	1	9.06E-03	0.471184422	0.013421143	-0.01072254	-0.009728615
Cacng7	1	1.28E-03	3.49582E-11	-0.001206773	-0.008731044	-0.009743033
Klhdc7a	1	2.44E-10	3.49582E-11	-0.006975978	-0.009743033	-0.009743033
BC049987	1	6.47E-03	0.037419503	-0.005837297	-0.010387288	-0.009869169
Syt3	1	1.26E-03	5.17234E-05	0.002703838	-0.009641176	-0.009941226
Lag3	1	1.00E+00	4.33431E-06	-6.32169E-05	-0.006181224	-0.010095224
1110020A2	1	2.70E-02	1	-0.005625789	-0.01207029	-0.010112278
Tmem184a	1	1.20E-02	1	-0.007815169	-0.012052748	-0.010124429
Gal3st1	1	9.04E-03	1	0.001877857	-0.011440781	-0.01017089
Plekhb1	1	3.89E-05	1.00933E-05	-0.009751367	-0.010218851	-0.010184735
1700109K2	1	1.02E-07	0.860176406	-0.009190361	-0.012588919	-0.010324889
Gm28182	1	3.27E-12	9.90647E-06	-0.011134894	-0.011134894	-0.010364902
Gm44851	1	6.60E-01	1.02554E-06	-0.006984313	-0.009002639	-0.010428817
Casc4	1	1.00E+00	0.002304304	-0.005837297	-0.008295236	-0.010447416
Gm38416	1	1.68E-05	0.002304304	-0.00779504	-0.010879348	-0.010447416
Tppp	1	4.86E-03	0.001639215	-0.011949081	-0.010540979	-0.010473012
Slc13a2	1	3.43E-07	1.01506E-06	-0.002598635	-0.010642835	-0.010518901
Mir670hg	1	1.25E-02	1	-0.001240111	-0.0124262	-0.010548106
Plekhh2	1	4.93E-02	0.001625702	-0.009182027	-0.010021049	-0.01062701
Gm8016	1	2.54E-08	5.14721E-05	-0.009987879	-0.011207387	-0.010755413
Acss2os	1	1.66E-02	2.67925E-09	-0.005600785	-0.009562772	-0.010826897
Plcl2	1	8.28E-03	1	-0.135128308	0.172772595	-0.010835773
Gm9968	1	1.00E+00	0.047545977	-8.3345E-05	-0.007234014	-0.010850227
Myl4	1	4.79E-03	1	-0.010810401	-0.012169353	-0.010869398
Gm15962	1	3.43E-07	5.14961E-11	-0.002833731	-0.010642835	-0.010890813
Rufy4	1	1.00E+00	0.02301958	0.001295307	-0.009564315	-0.010946507
Gm28643	1	1.00E+00	0.002341258	-0.006146837	-0.006732485	-0.010974869
Zbed5	1	1.00E+00	0.009773925	-0.003424615	-0.010118294	-0.011068141
F2r	1	5.68E-01	6.10243E-05	0.007077722	-0.009902441	-0.011114602
Gm45301	1	3.65E-10	3.54947E-13	0.008469584	-0.010970875	-0.011134894
Spata22	1	2.46E-02	0.022352276	-0.007806834	-0.011180816	-0.011158796
Gchfr	1	2.44E-02	0.022352276	0.009500781	-0.011276762	-0.011158796
Dsg1c	1	2.54E-08	4.64353E-09	-0.007220825	-0.011207387	-0.011217408
Gm40040	1	8.81E-04	0.003079315	0.004644912	-0.011768821	-0.011222711
Sult1b1	1	8.81E-04	0.008644098	0.006028439	-0.011768821	-0.011312795
Gm11337	1	3.65E-10	1.00987E-06	-0.0077985	-0.011785062	-0.011333087
Mup12	1	1.76E-04	3.49582E-11	-0.007220825	-0.010715328	-0.011371407
Sftpa1	1	2.44E-10	3.49582E-11	-0.003070243	-0.011371407	-0.011371407
Olfr1034	1	1.01E-07	2.20032E-07	-0.004799808	-0.011870678	-0.011486514
Gm44421	1	3.27E-12	6.8175E-08	-0.009182027	-0.011949081	-0.011487086
Gstm7	1	6.19E-02	0.00386558	-8.3345E-05	-0.010842447	-0.011582473
Stbd1	1	1.20E-02	0.001451235	-8.3345E-05	-0.011334506	-0.011736471
Cyp46a1	1	7.94E-06	0.000932936	-0.011957416	-0.012356825	-0.011800958
Slc6a12	1	4.31E-03	1	0.022443398	-0.018891351	-0.011868281
Fam228a	1	1.19E-02	0.000496001	-0.007000982	-0.011526397	-0.01189047
Stxbp5l	1	1.27E-01	9.8841E-06	-0.009186902	-0.010863107	-0.011903193
Gm45411	1	1.00E+00	0.001429029	-0.001466872	-0.005114091	-0.011916638
Bdnf	1	1.76E-04	3.49582E-11	-0.0077985	-0.011293003	-0.011949081
Dact2	1	2.59E-10	1.18651E-05	0.01537401	-0.012916959	-0.011992704
1500026H1	1	2.72E-02	1	0.011304492	-0.014331289	-0.012014911
Gm45609	1	2.30E-03	0.994109812	0.003714907	-0.013351604	-0.012030623
Gm11437	1	2.28E-04	1.00676E-09	0.007312818	-0.010926763	-0.012064761

Chic1	1	1.00E+00	0.000488008	-0.006765886	-0.009474548	-0.012070636
Mal2	1	1.33E-04	1	0.012361006	-0.036025522	-0.012075502
Mettl24	1	1.05E-07	3.31364E-11	0.007651938	-0.011582841	-0.012218759
Gm8883	1	2.15E-09	4.10629E-05	-0.000657561	-0.013262539	-0.01228855
4933417D1	1	1.18E-02	9.79903E-06	-0.009768036	-0.011622343	-0.012352465
Gm11639	1	1.27E-01	2.66242E-09	-0.001459954	-0.010863107	-0.012455271
D130043K2	1	3.23E-07	0.069626707	-0.00920703	-0.014620619	-0.012506957
Gm11967	1	3.64E-10	5.13152E-11	-0.007803375	-0.012599249	-0.012519187
Thsd1	1	1.38E-02	1	-0.001774936	-0.019199846	-0.012575252
Myh14	1	1.99E-03	1	0.001707157	-0.017775695	-0.012577197
Slc6a16	1	1.57E-08	5.05903E-11	-0.003652793	-0.012435229	-0.01260927
Nat8f4	1	2.85E-01	0.000254819	-0.004470439	-0.011502944	-0.012679061
Gm19461	1	2.41E-07	3.21128E-07	-0.004233927	-0.012742609	-0.012750545
Fst	1	3.64E-10	3.54947E-13	0.002690628	-0.012599249	-0.012763268
Gas1	1	1.03E-07	1.00236E-09	0.001538737	-0.012492974	-0.012878948
Clstn3	1	2.61E-03	0.149937125	-0.004020375	-0.013988502	-0.012972068
Gm32511	1	3.01E-10	1	-0.010491646	-0.031922911	-0.012972113
Sytl1	1	2.48E-07	1	-0.011186318	-0.017754341	-0.012972562
Lncbate1	1	1.04E-06	0.461680976	-0.012210597	-0.015445135	-0.012984791
Gm47802	1	1.00E+00	0.035793381	-0.010590558	-0.009740231	-0.013044697
Ccdc149	1	1.40E-06	0.948106128	-0.005069658	-0.016117786	-0.013170261
Rab17	1	1.02E-04	0.003916802	-0.007014191	-0.01396454	-0.013172527
Cyp2d40	1	2.55E-10	3.62054E-15	-0.004804684	-0.013012904	-0.013340943
Serpine2	1	2.33E-07	1.92719E-06	0.004297457	-0.013748687	-0.013410734
S1pr5	1	4.75E-03	5.05026E-11	-0.007234035	-0.012265298	-0.013423457
Gm32569	1	1.05E-04	3.27315E-07	-0.011159898	-0.012958462	-0.013540752
Gstm6	1	4.60E-03	0.000149483	-0.005052989	-0.013318791	-0.013672842
Tmem88b	1	6.10E-10	3.23643E-07	-0.000665895	-0.014490381	-0.013744327
Gm34280	1	2.63E-08	5.86573E-15	-0.007575197	-0.01309852	-0.01376462
Cidec	1	4.97E-08	0.000235306	0.006246866	-0.014976529	-0.013765113
Dtx1	1	2.40E-04	9.94462E-07	-0.009431749	-0.013311579	-0.013775648
Gm15318	1	1.70E-06	0.000638962	0.022614098	-0.014456599	-0.01381469
C1qtnf9	1	5.86E-03	3.20027E-07	-0.01531048	-0.011918602	-0.01383441
Spon2	1	4.28E-11	2.43218E-05	-0.003672921	-0.015468588	-0.01394252
Ranbp3l	1	3.00E-03	1.80184E-08	-0.004367622	-0.012900717	-0.014026728
Suox	1	6.19E-10	8.00722E-09	-0.012543425	-0.014394436	-0.014052324
Cldn12	1	6.28E-03	0.096678323	0.011413393	-0.015381664	-0.014061236
2810030D1	1	3.26E-07	5.4875E-08	0.012256042	-0.013806432	-0.014078492
Nsun7	1	3.22E-07	5.4875E-08	-0.009776371	-0.013902377	-0.014078492
Atoh8	1	8.19E-10	1	-0.004737933	-0.042569876	-0.014134305
Atxn7l1os2	1	1.00E+00	0.007057792	-0.014749474	-0.010599831	-0.014192477
Slc15a5	1	8.82E-06	1.39804E-11	-0.009198696	-0.013420648	-0.01427081
Gm46348	1	3.20E-07	2.36132E-05	-0.013357612	-0.014716564	-0.014276685
Magix	1	4.37E-11	8.79018E-10	-0.002049423	-0.014558456	-0.014386489
1700019A0	1	2.37E-01	0.026654532	-0.011413079	-0.013700634	-0.014428417
Fitm2	1	5.46E-03	1	0.015717768	-0.023106947	-0.014536489
Gm15640	1	1.03E-15	7.4998E-11	-0.004242261	-0.01514646	-0.014540487
2310030G0	1	2.63E-07	1	0.005637896	-0.019316644	-0.014548278
Spry4	1	8.34E-14	0.013135729	-0.012568429	-0.018337928	-0.014569693
Sh2d4a	1	1.44E-09	1	0.009537281	-0.026298045	-0.014572879
Mmd2	1	7.31E-14	5.85538E-15	-0.003664587	-0.014568785	-0.014578807
Mapk4	1	2.47E-04	0.000279992	0.002311252	-0.015233886	-0.014584031
Kcnt2	1	1.00E+00	0.003882871	0.004618492	-0.009798961	-0.01468465
Gm26871	1	8.76E-03	1	0.002322807	-0.024400069	-0.014692698
Tlr5	1	1.00E+00	0.005807239	-0.004842897	-0.012669464	-0.014704292
Lrp3	1	6.15E-10	5.50517E-08	-0.005630664	-0.015208623	-0.014802596
Ctcfl	1	1.03E-15	5.50517E-08	-0.009781246	-0.015960647	-0.014802596
AI467606	1	1.00E+00	3.97246E-06	-0.012556635	-0.011104222	-0.014872386
Rai2	1	1.15E-02	0.385759383	-0.008890871	-0.015982147	-0.014978802
D830025C0	1	4.11E-03	0.229627283	-0.00704407	-0.01670118	-0.015029994
2210039B0	1	5.06E-08	1.55787E-06	0.002091409	-0.015598825	-0.015128619
Pcsk9	1	2.21E-14	0.427060802	0.024669575	-0.020543976	-0.01519801
Ccnl2	1	1.96E-02	1	-0.007689088	-0.126911533	-0.015251234

Rtkn	1	2.05E-04	0.375083368	-0.009248703	-0.018271663	-0.015253211
Gjc3	1	8.05E-18	3.79899E-19	0.009828108	-0.01531048	-0.01531048
Plekhf1	1	3.16E-07	7.45076E-11	-0.00286361	-0.014812509	-0.015354674
Gm16121	1	4.28E-11	7.45076E-11	0.000477661	-0.015468588	-0.015354674
Gm30097	1	1.21E-08	1.82975E-05	-0.008174416	-0.016358061	-0.015386146
Fam210b	1	2.13E-05	0.160052313	0.022464942	-0.018945283	-0.015432805
Wasf1	1	2.64E-01	0.01915887	-0.005081452	-0.015351791	-0.015447795
Gm765	1	1.58E-06	3.99571E-05	0.00381264	-0.015931283	-0.015455214
Cish	1	2.20E-04	1	-0.021127649	-0.019780129	-0.015489723
Nhs	1	1.00E+00	0.007010844	-0.004263806	-0.012864327	-0.015494256
Rph3al	1	8.24E-06	1	-0.007878385	-0.020889097	-0.015521194
BB365896	1	1.90E-05	1.77886E-10	-0.008401178	-0.01428225	-0.015534269
Gm14455	1	1.00E+00	0.00164201	-0.009000397	-0.013085389	-0.015601221
Gm15343	1	7.30E-14	4.07209E-13	-0.007588407	-0.015960647	-0.015636503
Dll1	1	6.08E-06	0.000122234	-0.013142644	-0.016313949	-0.015647682
Gm13481	1	8.87E-03	5.90174E-05	0.004386855	-0.015015239	-0.015673128
Gm13267	1	2.73E-13	4.99737E-05	-0.013374281	-0.018088292	-0.015686001
Oat	1	1.15E-03	2.06761E-06	-0.005407362	-0.014717864	-0.015694143
Gm28055	1	1.07E-03	1	0.016416136	-0.022821719	-0.015766619
Itga3	1	4.16E-01	0.037758638	-0.015348693	-0.014787749	-0.015787835
Igsf11	1	4.66E-12	1	0.006881541	-0.049180301	-0.015860304
Lgalsl	1	1.11E-08	2.22665E-05	-0.00703432	-0.017421884	-0.015865596
Plscr2	1	8.50E-06	1	0.016664442	-0.022465969	-0.015884886
Gm26608	1	1.09E-03	1	0.005142701	-0.019732108	-0.015911133
Tc2n	1	2.90E-02	1	0.003379008	-0.024058397	-0.015951609
Gata6	1	2.96E-07	0.782908927	0.023813923	-0.021229736	-0.016020937
Cib3	1	8.05E-18	7.50982E-11	0.024908338	-0.016938854	-0.016078778
1810019D2	1	1.85E-06	0.778398408	-0.007878385	-0.020969771	-0.01611102
Gm15336	1	1.00E+00	0.000290157	-0.011995629	-0.012162133	-0.016192191
Il17rc	1	1.00E+00	0.03202827	0.008178189	-0.012580206	-0.016198639
Gm16548	1	4.38E-10	8.44764E-06	0.00150194	-0.017558032	-0.016289844
Foxa1	1	2.13E-09	8.55015E-09	0.000126747	-0.016522081	-0.016310137
Arl4d	1	1.08E-06	0.140473663	0.013121414	-0.019944149	-0.01632363
Rcan2	1	5.18E-06	0.026236637	0.013218148	-0.018545075	-0.016356646
Pdzk1	1	1.02E-05	1	-0.001076805	-0.032530776	-0.016372211
Gm31084	1	4.70E-10	0.000821902	-0.006807558	-0.018429964	-0.016475886
Gm13613	1	4.12E-13	0.003396487	-0.004623054	-0.020629592	-0.01649334
Myorg	1	1.63E-04	5.26364E-08	-0.014180134	-0.015774475	-0.016520481
Slc17a1	1	3.55E-09	1	0.021009863	-0.027954343	-0.016610561
Pde1a	1	6.55E-01	0.010301741	0.00449434	-0.015058043	-0.016633894
Gm11789	1	1.11E-07	0.091588934	-0.000948955	-0.020504282	-0.016657796
B230206L0	1	1.16E-07	0.001575938	0.020048801	-0.018711889	-0.016685658
Lzts3	1	1.78E-03	0.000422228	-0.011193236	-0.01635345	-0.016719968
Gltpd2	1	5.10E-04	0.000636204	-0.007848507	-0.016636077	-0.016796034
Bdh2	1	1.17E-10	4.05038E-08	0.001487522	-0.017260982	-0.01682905
Vangl1	1	3.78E-10	1	-0.013990169	-0.023283588	-0.016947366
Aqp11	1	4.43E-04	0.611341169	-0.004889445	-0.021179712	-0.016994998
Gm33543	1	7.74E-08	1	0.002228847	-0.046775837	-0.017007138
Arhgef9	1	7.49E-06	0.300259376	-0.008816724	-0.022344422	-0.01701464
Pglyrp2	1	3.10E-10	2.08331E-06	-0.000353195	-0.018314475	-0.017052267
Vnn1	1	5.75E-06	0.143772744	0.018319862	-0.019570697	-0.017056448
Scara5	1	1.00E-07	3.31364E-11	0.019637488	-0.016732881	-0.017086825
Plekhg6	1	2.78E-13	1.62359E-09	-0.007840172	-0.017992347	-0.017098156
Flvcr2	1	3.10E-07	0.001604172	-0.007927019	-0.018492392	-0.017160407
Mkx	1	8.34E-14	1.47171E-09	-0.016719011	-0.018406002	-0.017187667
Gm45425	1	1.53E-14	1.60149E-09	0.017194391	-0.018156366	-0.017188239
4930408O1	1	3.68E-12	2.07475E-14	-0.007025985	-0.017110085	-0.017234128
Gm45669	1	2.66E-01	0.003338395	-0.013399285	-0.015619477	-0.017307527
Sucnr1	1	1.09E-19	6.62268E-19	-0.010598892	-0.017516529	-0.01736253
4930556M	1	1.34E-02	1	0.008135042	-0.021806745	-0.017415937
Trps1	1	4.84E-16	1	-0.1555968	0.344926049	-0.01743558
Cdc42ep5	1	4.23E-11	4.62549E-15	-0.004491983	-0.017096962	-0.017445044
Gm4841	1	1.72E-04	0.005727524	0.002051152	-0.01854917	-0.017475543

Gm15611	1	2.00E-19	9.57202E-16	-0.018094204	-0.017930184	-0.01747821
4732419C1	1	2.22E-07	1	-0.01102018	-0.035349583	-0.017484466
Gm47348	1	1.51E-14	3.12005E-17	-0.00125678	-0.017438125	-0.017542125
Hsd17b7	1	7.99E-09	0.57965514	0.031049137	-0.026404373	-0.017626901
Olfm2	1	1.03E-08	1.76696E-11	-0.002070967	-0.017500288	-0.017650234
L3hypdh	1	3.15E-02	1	0.005935343	-0.020247428	-0.017669203
Aldh5a1	1	1.52E-03	1	-0.00080208	-0.025624794	-0.017759979
Sh3yl1	1	4.53E-15	0.376317201	-0.010438807	-0.026558401	-0.017772732
Cldn1	1	2.17E-04	0.00062028	-0.001172257	-0.017417151	-0.017846733
D130020LC	1	8.64E-03	0.000243731	-0.004631388	-0.017496977	-0.017859034
Itga8	1	1.00E+00	0.012672075	-0.008204295	-0.012954692	-0.017923521
1700007F1	1	8.05E-18	4.48832E-12	-0.010266064	-0.018567228	-0.017951234
Serpinb2	1	1.00E+00	0.001129498	-0.01559354	-0.012184728	-0.017956459
Repin1	1	2.16E-03	1	0.004221031	-0.020879544	-0.017959746
Lrit2	1	3.72E-12	3.10694E-13	0.003463142	-0.017828327	-0.0179844
Prss53	1	6.10E-07	8.12795E-06	-0.001509961	-0.018558199	-0.018008301
Gm46559	1	3.46E-14	5.35535E-17	-0.012796606	-0.017838657	-0.018022719
Tesmin	1	3.43E-02	0.072865862	0.018638854	-0.018515896	-0.018046463
Lrrc3	1	1.12E-06	5.42901E-08	-0.011193236	-0.017637737	-0.018079786
Alas2	1	1.00E+00	0.003843629	0.002160678	-0.01641412	-0.018160606
Gm13449	1	5.46E-15	7.5023E-13	-0.007608535	-0.018665967	-0.018163994
Gm15889	1	3.82E-11	3.10523E-17	-0.005647333	-0.017664307	-0.018356312
Plekhh3	1	7.66E-05	0.002273542	-0.001298452	-0.019229211	-0.018379241
Gm46329	1	5.94E-10	6.20684E-17	-0.009221656	-0.017747129	-0.018413229
Slc18b1	1	8.44E-07	1	-0.004331898	-0.025167316	-0.018430482
Tox2	1	8.54E-04	0.008387377	-0.015891286	-0.019172325	-0.018490438
Pgpep1l	1	9.98E-05	2.63852E-10	-0.006229883	-0.016923913	-0.018516186
Mup17	1	9.85E-01	6.64859E-14	0.000459576	-0.013976679	-0.018528909
Sardhos	1	8.99E-12	5.40348E-15	-0.000116683	-0.017982017	-0.018562074
Gm43597	1	1.08E-01	0.000528957	-0.007288917	-0.017562258	-0.018596433
Ift81	1	5.08E-04	0.014434218	-0.014584513	-0.020272883	-0.018611771
Gcnt4	1	9.28E-16	2.39465E-15	-0.0086509	-0.018816863	-0.018682907
Gm4876	1	1.00E+00	0.005460082	-0.015601874	-0.015675728	-0.018691819
Gm20300	1	6.35E-03	0.015900754	-0.006039919	-0.01958051	-0.018710568
Gm2061	1	2.05E-19	6.99693E-21	0.015786069	-0.018648425	-0.018754392
Gm49961	1	2.92E-04	1	-0.003834109	-0.030289466	-0.018870359
Sall1	1	2.69E-12	1	0.021771211	-0.035444421	-0.018884627
Gm28040	1	9.27E-13	0.000114534	-0.002564119	-0.02149873	-0.018930598
Nlrp12	1	5.53E-04	1	0.086605882	-0.055687758	-0.018936332
Slc16a1	1	6.12E-03	0.212198951	-0.000755532	-0.021735235	-0.019046476
Dtnbos	1	6.13E-03	0.00149217	-0.008915875	-0.018012877	-0.019154387
Sgce	1	3.22E-04	0.005750106	-0.010650315	-0.019986407	-0.01915969
Gm10649	1	7.78E-20	1.59913E-06	-0.01950761	-0.022427897	-0.019181677
Notum	1	8.44E-03	1	-0.001651021	-0.028878933	-0.019290404
Inhbe	1	8.36E-14	2.60845E-10	0.001257094	-0.01993843	-0.019330373
Gm13944	1	1.29E-07	1.12645E-12	0.014754663	-0.017226084	-0.019337942
Gm49970	1	1.06E-09	0.163981832	-0.003315745	-0.025853268	-0.01936849
Grid1	1	1.98E-04	4.72013E-05	-0.009718268	-0.019931026	-0.019380443
Snx33	1	1.49E-03	1	-0.017649881	-0.025869309	-0.019414749
Gng7	1	1.72E-03	0.044026411	-0.020355135	-0.021181013	-0.019519373
Slc25a34	1	4.57E-04	0.000101666	0.00147552	-0.019413889	-0.019597784
Pard3bos1	1	5.43E-03	0.003487793	0.006422204	-0.019103901	-0.019621686
Angptl8	1	5.22E-03	0.402525917	0.010687129	-0.021753611	-0.019676988
Pitx3	1	1.48E-21	4.01188E-23	-0.014188468	-0.019722577	-0.019722577
Tpd52l1	1	3.73E-09	0.001248532	0.000652999	-0.022959967	-0.019778444
1810064F2	1	7.18E-04	0.374060124	-0.012075515	-0.023253403	-0.019783912
D530033B:	1	1.00E+00	0.008419442	0.010680269	-0.013221854	-0.019892508
A930001CC	1	2.34E-10	0.032443434	-0.010910415	-0.02608336	-0.01992089
Abcg8	1	2.38E-07	1	0.012804103	-0.0581488	-0.019966229
A430090L1	1	1.99E-02	2.60552E-09	-0.010397134	-0.017343287	-0.020015585
Pex11g	1	1.90E-02	1	0.004513603	-0.024224509	-0.020058456
Gm35339	1	7.47E-01	6.3083E-07	-0.007060739	-0.016009679	-0.020059779
Irf2bp1	1	1.00E+00	0.003459369	-0.006711301	-0.0142866	-0.020103503

Rarres1	1	1.31E-02	0.321510256	-0.011098395	-0.021835924	-0.020186017
Amot	1	1.11E-08	3.56851E-05	0.009422312	-0.022057046	-0.02020693
Cc2d2a	1	1.05E-01	0.001381207	-0.015382031	-0.016839502	-0.020213378
Gm14327	1	7.38E-08	0.003473395	-0.005702216	-0.023137619	-0.020269151
Idi1	1	2.44E-06	1	0.038748815	-0.031321916	-0.020317105
Gm30505	1	5.14E-11	0.119597486	0.002594073	-0.025839636	-0.020339514
Rgs18	1	1.00E+00	0.002020319	-0.013435247	-0.010595269	-0.020340486
D730045B(	1	3.21E-08	4.41277E-11	-0.008671028	-0.01995006	-0.020350322
Gm47467	1	7.41E-07	2.77099E-07	0.010582537	-0.02060744	-0.020355626
Rdh9	1	3.13E-10	2.77099E-07	-0.005445575	-0.021207776	-0.020355626
Pllp	1	3.52E-17	3.80477E-06	-0.000383074	-0.023632286	-0.020475886
B930025P(	1	7.72E-10	4.61386E-05	0.004218988	-0.023257017	-0.020532382
Zfp977	1	1.86E-12	4.19725E-09	-0.00671816	-0.021844311	-0.020650177
C430014B1	1	1.19E-02	0.008659908	-0.010655191	-0.020793928	-0.020679955
Lrrfip1	1	3.34E-02	1	-0.013115244	0.138559798	-0.020705808
Txnrd3	1	2.27E-04	1.01092E-09	-0.004863025	-0.018667777	-0.02074783
1500035N2	1	1.00E+00	0.011571789	0.002573945	-0.017855538	-0.020762861
Zfp354a	1	1.34E-16	2.7591E-11	-0.008207754	-0.022349493	-0.020837342
Lrrn3	1	3.75E-05	0.002125606	0.008136517	-0.021835791	-0.020911886
4931428L1	1	3.22E-06	1	0.018072736	-0.032912652	-0.021044537
933016201	1	7.81E-09	0.007722029	-0.014023507	-0.025514899	-0.021166574
Slc24a3	1	1.00E+00	4.00265E-05	-0.009609367	-0.014165993	-0.021171107
Krba1	1	1.15E-04	0.121234298	-0.003200136	-0.024682978	-0.021228795
Lipg	1	7.68E-20	1	-0.002472126	-0.041048768	-0.021312277
Dio1	1	1.44E-19	1.62878E-14	-0.008204295	-0.022591917	-0.021337656
Plag1	1	1.00E+00	1.13682E-12	-0.014547716	-0.015566674	-0.021363824
Ccdc157	1	1.07E-03	0.000222355	-0.004327023	-0.021509052	-0.021364595
Gm29394	1	3.85E-01	0.00216499	-0.005715425	-0.019479162	-0.021498873
Gm44787	1	1.76E-14	4.76006E-08	-0.015373697	-0.023899463	-0.021593489
4933408B1	1	1.00E-02	8.7663E-06	-0.013074791	-0.0199622	-0.02159807
4930517O1	1	3.15E-09	0.021278637	0.02530677	-0.02726709	-0.021638454
Ptgds	1	2.01E-23	7.3508E-23	-0.010860408	-0.021928626	-0.021774628
Gm45589	1	1.16E-14	6.57116E-07	-0.013652466	-0.024368069	-0.021780425
Depp1	1	2.55E-08	0.050484423	0.035681494	-0.026829472	-0.021787149
Egln3	1	5.89E-03	7.75508E-06	-0.001779811	-0.01813792	-0.021821859
Spata2l	1	1.64E-04	1.33324E-08	0.006766199	-0.020273489	-0.02187589
Gm16984	1	2.86E-04	4.86627E-05	0.014939752	-0.021368485	-0.021938833
Hdac11	1	4.35E-04	0.04654911	-0.006302851	-0.024133174	-0.022037106
Dhfr	1	3.17E-02	1	-0.004423578	-0.025737482	-0.022045645
Gm13522	1	7.98E-11	7.66722E-12	-0.015833511	-0.022234513	-0.022080358
Gm16035	1	2.89E-12	8.81146E-05	-0.008705782	-0.025562129	-0.022109563
Rab30	1	1.31E-15	1	0.015720454	-0.067959706	-0.02213947
Pknox2	1	1.98E-06	1.39887E-09	0.007814066	-0.021348343	-0.022210376
Podn	1	1.07E-07	0.000698956	-0.015623419	-0.024290175	-0.02228973
3110009E1	1	1.00E+00	0.021006799	-0.020042435	-0.011252117	-0.022298642
Plppr2	1	7.44E-04	0.001301004	-0.000409494	-0.022644067	-0.022333925
Gm16193	1	3.98E-10	0.000540766	0.001772968	-0.025921343	-0.022372966
Impg2	1	3.71E-04	0.009844891	-0.00791314	-0.023849247	-0.022391564
Gm50163	1	1.00E+00	0.010576712	-0.014617851	-0.016398203	-0.02242433
Gm26632	1	6.02E-02	0.029260107	0.019398159	-0.023066743	-0.022515984
Spata24	1	1.98E-02	0.000513618	-0.018633904	-0.021897252	-0.022603602
Avpr1a	1	4.35E-23	2.24569E-17	0.004242575	-0.023570123	-0.022820173
Fdft1	1	5.37E-05	1	0.082697233	-0.047171394	-0.022870072
Gm16158	1	3.71E-05	1.45874E-06	0.004791787	-0.022388534	-0.022887126
Foxa2	1	1.53E-07	0.037501318	0.010051171	-0.028307459	-0.022921084
Acy1	1	5.46E-03	0.125909764	-0.002619002	-0.02459536	-0.022923922
Gm9733	1	1.00E+00	4.83383E-05	-0.010208378	-0.019201849	-0.022933586
1700112D2	1	1.58E-11	8.58207E-09	-0.004204287	-0.024084651	-0.022964336
Tle2	1	1.48E-01	0.00084815	0.0094985	-0.021361081	-0.023045305
Zfp612	1	2.83E-06	1	0.019336985	-0.032502905	-0.023079211
Fkbp14	1	7.51E-04	3.01599E-06	0.007896545	-0.021925825	-0.023085891
Ccl27a	1	6.31E-08	3.57316E-11	-0.009263329	-0.022574182	-0.023100308
Gm42984	1	1.00E+00	0.011746256	-0.017382863	-0.014413904	-0.023198904

Hyal2	1	2.39E-02	0.00078735	-0.003774351	-0.021934847	-0.023256943
Pir	1	3.52E-06	1	-0.004924361	-0.058770855	-0.023311656
Cdhr5	1	2.57E-15	1.11671E-07	-0.016427855	-0.025499966	-0.023311946
Ramp1	1	1.00E+00	0.02150965	-0.021169322	-0.020887265	-0.02341353
Dcdc2a	1	2.58E-12	2.85792E-12	-0.013189192	-0.023923426	-0.023883173
Frmd5	1	1.00E+00	9.3157E-06	-0.017534136	-0.019607139	-0.023922414
Efcab5	1	1.00E+00	0.000119815	-0.022129822	-0.02148672	-0.023936312
4930594M	1	5.91E-04	0.000125417	-0.019221329	-0.024663434	-0.023961154
Dab2ip	1	2.07E-01	0.006520792	-0.000565568	-0.020467424	-0.023965485
Ace	1	1.13E-01	2.91531E-06	-0.01395088	-0.020458993	-0.023989548
Gm16833	1	5.42E-13	5.98268E-12	-0.000396284	-0.024758272	-0.024004006
Sema5b	1	1.75E-01	7.18349E-12	-0.010284208	-0.019449614	-0.024044121
Shroom1	1	7.54E-18	1	0.009207419	-0.060343761	-0.024054481
Gm15879	1	2.30E-01	0.023758409	0.00812875	-0.023597136	-0.024179169
AA986860	1	6.67E-05	0.020660569	0.005318039	-0.027539194	-0.024182105
Meg3	1	1.94E-05	4.1219E-09	0.00925429	-0.023046224	-0.024184595
Traf4	1	4.02E-05	0.027344042	0.008909658	-0.02682407	-0.024282046
Dmd	1	4.01E-06	1	0.030304863	-0.068771825	-0.024294191
Izumo4	1	4.95E-07	0.184432084	-0.015100626	-0.029597464	-0.024319393
C730027H:	1	6.76E-19	4.88105E-15	-0.000730528	-0.025120094	-0.024320145
Slc10a2	1	2.99E-21	4.25396E-27	0.008415269	-0.024018203	-0.024346242
AI838599	1	1.58E-11	1.38487E-18	-0.010072641	-0.02336641	-0.024512463
Sorbs3	1	5.08E-03	1	-0.014391568	-0.039937689	-0.024538392
Apom	1	1.57E-02	0.032789196	-0.016041008	-0.025473705	-0.024739087
Gcat	1	1.99E-10	1.95636E-13	-0.000503767	-0.024255883	-0.024915846
Porcn	1	1.00E-08	3.43383E-14	-0.021156112	-0.024137276	-0.025069272
Fdps	1	8.32E-04	1	0.066270445	-0.037641104	-0.025084049
Exd1	1	8.08E-08	0.000484294	0.011311962	-0.028891377	-0.025174065
Cdc42ep1	1	3.32E-09	2.98538E-07	-0.002150853	-0.026261012	-0.025281404
Lrit1	1	1.34E-15	5.92103E-16	-0.001779811	-0.025346276	-0.02528833
Tjp3	1	1.00E+00	0.018670898	0.02191895	-0.023059339	-0.025296042
Ptpre	1	6.53E-05	1	-0.023628267	0.113813466	-0.025413789
Ston1	1	3.39E-05	0.004455302	0.003914383	-0.029427355	-0.02545144
Platr4	1	3.84E-15	3.76985E-07	0.001193877	-0.028883324	-0.025459383
Mnd1	1	1.53E-16	7.42962E-09	-0.018751764	-0.028540537	-0.025466381
Pacsin3	1	2.51E-11	3.6998E-09	0.018502908	-0.026882799	-0.025481291
Cyp2u1	1	2.26E-09	5.8073E-15	-0.009275123	-0.023927844	-0.025494093
Gm50063	1	1.01E-28	1.00588E-28	-0.02033359	-0.02570368	-0.025559703
Thpo	1	7.39E-15	0.000875086	-0.005753639	-0.032273103	-0.025596698
Rorc	1	6.16E-04	0.043333053	-0.025623092	-0.029222704	-0.02562934
Zfhx3	1	1.04E-02	1	-0.09532411	-0.161580426	-0.025687075
Zkscan7	1	7.28E-03	1	-0.015034248	-0.03239657	-0.025695843
Tpmt	1	7.01E-09	0.175233194	0.006520489	-0.037262868	-0.025767126
Apbb3	1	6.44E-03	1	0.007480135	-0.034440091	-0.025829639
Hopx	1	1.93E-07	1.7708E-10	0.023573744	-0.024263604	-0.025865927
Tomm40l	1	8.45E-03	1	0.0141503	-0.036355178	-0.025923009
Cpeb2	1	1.75E-06	1	0.12286927	-0.158411656	-0.025997141
Map3k21	1	4.48E-24	4.40434E-13	0.013554808	-0.028385547	-0.026025307
Sox13	1	3.58E-11	5.21785E-05	0.00255586	-0.030033272	-0.02602665
Spns3	1	1.00E+00	0.003483742	-0.021435713	-0.011826825	-0.026094574
Arhgef5	1	2.53E-07	1	-0.016068293	-0.04384964	-0.026269726
St8sia6	1	1.00E+00	8.39725E-05	-0.028056004	-0.017573368	-0.026422389
Fn3k	1	1.06E-13	1.29396E-23	-0.011232866	-0.025267872	-0.026455831
Cebpe	1	5.98E-13	8.88369E-31	-0.008696031	-0.02419372	-0.026527888
Mmp15	1	3.55E-15	6.62935E-13	-0.008949213	-0.027377976	-0.026569816
Akap17b	1	4.81E-03	6.7001E-08	-0.006890903	-0.021443397	-0.026593868
Frk	1	2.08E-12	0.126744067	-0.01629073	-0.03666799	-0.026797906
Ranbp17	1	3.03E-05	0.003815176	-0.011992409	-0.028615495	-0.026825697
Gm10644	1	1.41E-08	3.56743E-06	0.001620353	-0.030059326	-0.026968817
9630013D2	1	2.33E-12	0.000884045	-0.018233847	-0.034470123	-0.026985149
E330011O2	1	5.46E-13	2.61947E-19	-0.016199677	-0.025835217	-0.027021355
Gpsm2	1	1.07E-01	0.006879443	-0.02045169	-0.025895049	-0.027028944
H1f0	1	2.04E-02	7.55081E-05	0.004157814	-0.02419176	-0.027090222

Ackr4	1	4.87E-05	0.005455957	0.046640366	-0.030537463	-0.027104437
Slc6a9	1	6.77E-06	0.003780358	-0.020903169	-0.030788915	-0.027161183
Gm39363	1	2.10E-14	8.01434E-09	-0.009301542	-0.02899602	-0.027171965
Wdr93	1	4.90E-03	0.005603002	-0.014771257	-0.026973659	-0.027217502
Slc25a27	1	4.19E-06	0.004895961	-0.005551881	-0.031044462	-0.027247257
Nr2f2	1	1.20E-02	0.000884909	-0.018357999	-0.025799136	-0.027278157
Rilp	1	1.70E-10	3.31631E-06	-0.014513201	-0.030099343	-0.027285378
Farp2	1	2.76E-02	1	0.05054169	-0.054945513	-0.027424697
Rhou	1	5.59E-05	1	-0.030744173	-0.041911327	-0.027500618
Itga1	1	2.89E-04	1	0.006542184	-0.057217962	-0.027552771
Sdc1	1	1.00E+00	0.000171681	0.002749283	-0.012073654	-0.027659676
Serpina11	1	9.39E-12	1.44181E-13	0.002584322	-0.027276119	-0.027718168
Serpina3n	1	4.03E-11	2.60673E-06	0.019396742	-0.030563531	-0.027721205
Fam228b	1	4.60E-06	2.93528E-07	0.008556701	-0.026618732	-0.027798565
Pdk2	1	2.05E-05	0.807891084	-0.011823989	-0.035674828	-0.02780526
Gask1a	1	2.45E-22	4.47069E-24	0.009276614	-0.027807872	-0.027873861
Echdc1	1	2.74E-04	1	0.004882678	-0.047356898	-0.027931015
Gm15614	1	2.50E-05	6.53633E-07	0.011080326	-0.026521818	-0.02794661
Ebp	1	4.45E-02	0.000884904	9.31706E-05	-0.027308208	-0.028056141
Gm32828	1	2.38E-17	1.7537E-14	-0.002147394	-0.029087547	-0.028141272
Gm16551	1	6.20E-25	8.45776E-29	-0.026926733	-0.027982221	-0.028156262
Gm35164	1	7.26E-27	4.50974E-31	-0.020009097	-0.028146241	-0.02831026
Olfml1	1	1.47E-21	2.83677E-17	-0.007680087	-0.028959313	-0.028392924
2510002D2	1	1.99E-02	0.073417727	-0.004685093	-0.030412751	-0.028401578
Cyp3a59	5.25438E-05	4.41E-21	1	0.171499414	-0.078196328	-0.02840392
Kcnrg	1	1.00E+00	6.04373E-06	-0.019824008	-0.017755491	-0.028409177
Lrg1	1	1.00E+00	0.005532483	-0.017116711	-0.001722324	-0.02849156
Lrfn3	1	7.13E-16	1.71661E-11	3.36516E-05	-0.02978513	-0.028506909
Apof	1	1.43E-08	0.000193432	-0.019498098	-0.03257899	-0.028534741
Llgl2	1	1.01E-07	0.035886132	-0.001120521	-0.037206933	-0.028622035
BC065397	1	2.94E-01	0.003865442	-0.005002669	-0.025719238	-0.028767121
Rmdn2	1	2.47E-07	1	0.041702174	-0.074884705	-0.028829376
Mup1	1	1.38E-30	4.7744E-35	-0.021735203	-0.028723916	-0.028887935
Gm49668	1	1.36E-13	6.42455E-10	0.012475647	-0.031141206	-0.029031125
Hunk	1	1.85E-13	2.57343E-09	0.000586323	-0.030746126	-0.029048008
Padi2	1	1.00E+00	3.64385E-06	-0.013794196	-0.02331991	-0.029067209
Car14	1	1.15E-28	1.21113E-32	-0.001559968	-0.028973551	-0.02906753
Cpb2	1	2.56E-07	1	-0.007155206	-0.064452182	-0.029110487
Aqp8	1	5.11E-25	4.64131E-33	-0.019439756	-0.028632388	-0.029124447
Ildr2	1	1.92E-18	5.52074E-20	0.015267705	-0.029097877	-0.029129751
Slc26a1	1	4.63E-05	2.13902E-07	0.015807166	-0.027929396	-0.029221177
Srd5a2	1	3.84E-22	6.88957E-14	-0.015314178	-0.031823856	-0.029525565
Gm11844	1	1.20E-20	1.45912E-16	-0.005732094	-0.030807448	-0.029533134
Gm47431	1	1.31E-28	4.19922E-27	-0.004671018	-0.029855812	-0.029663803
Flrt1	1	8.03E-14	6.63122E-14	-0.011737812	-0.029799877	-0.029705731
Inhba	1	1.90E-10	5.88153E-11	-0.010590796	-0.029912573	-0.029738668
Msmo1	1	4.47E-08	1	0.083225595	-0.060703617	-0.029748542
Wnt5b	1	2.83E-06	4.74042E-05	-0.018147908	-0.032554229	-0.029790231
Mettl26	1	1.17E-07	0.035573581	0.011576698	-0.037949743	-0.029814982
Rarres2	1	7.43E-16	2.84775E-17	-0.007921474	-0.029709843	-0.029841703
Grik5	1	1.18E-05	0.006624239	-0.000880549	-0.033028739	-0.029888333
Rnf24	1	8.18E-02	0.007252355	-0.014869645	-0.027734059	-0.030120862
Grtp1	1	9.03E-06	4.62558E-11	0.011894513	-0.026868368	-0.030212643
Neu2	1	2.17E-13	1.5088E-12	-0.008979091	-0.03069968	-0.030237517
Ccdc192	1	2.51E-07	1.95042E-06	-0.011925661	-0.030238258	-0.030306087
Pex19	1	9.67E-06	1	0.033849396	-0.051044163	-0.030377181
Gm47371	1	1.30E-05	3.27619E-08	0.000341476	-0.029462616	-0.030446889
Pdp2	1	4.08E-07	7.67974E-08	0.011641332	-0.030628295	-0.030472485
Adcy6	1	4.20E-06	0.000134897	0.002527158	-0.02989184	-0.030535703
Bcar1	1	4.53E-17	0.000209915	-0.029004618	-0.040389572	-0.030594238
Dnah12	1	1.00E+00	3.35188E-06	-0.013725857	-0.024237294	-0.030596508
Cabcoco1	1	9.86E-12	8.23451E-06	-0.003279187	-0.034359294	-0.030637755
Zbtb10	1	5.28E-03	1.75685E-05	-0.018933633	-0.027467018	-0.030689277

Pctp	1	6.98E-01	0.042909625	0.026077003	-0.027969869	-0.030723583
Inhbc	1	1.39E-14	1.42819E-11	0.004272215	-0.032752039	-0.030863396
Hectd2	1	3.06E-07	0.209167478	0.006337204	-0.041578546	-0.030991271
Mpzl2	1	4.80E-11	0.850872097	0.002475676	-0.050214631	-0.031045123
1190005I0	1	1.76E-04	0.000113737	-0.005115029	-0.03090682	-0.031046771
Fras1	1	4.28E-08	5.8521E-12	-0.024226086	-0.030394994	-0.03105118
4930578G1	1	1.34E-04	8.06167E-09	-0.009540946	-0.028203368	-0.031149397
Tmtc4	1	1.00E+00	0.00443587	-0.012839993	-0.025843697	-0.031250369
Ly6g2	1	4.80E-15	2.25203E-09	0.003447651	-0.034027296	-0.031254628
Thrsp	1	7.24E-07	8.22803E-07	-0.015928023	-0.031441691	-0.03133452
Ube2u	1	2.54E-32	1.24652E-34	0.004770243	-0.03134362	-0.031363662
Eppk1	1	1.39E-14	0.002093302	0.011353396	-0.040941	-0.031375832
Brdt	1	2.95E-06	5.28846E-08	-0.011793902	-0.030637834	-0.031427798
Gm30849	1	3.57E-12	8.58978E-12	-0.002081583	-0.031996897	-0.031457276
Tbc1d30	1	1.48E-06	0.002499141	-0.018634143	-0.035889381	-0.031478638
Aifm2	1	2.31E-06	0.046849711	0.004580592	-0.041633771	-0.031508685
Tmem82	1	1.23E-06	0.004978082	-0.005403589	-0.037488252	-0.031541652
Leap2	1	1.23E-21	8.94231E-10	0.015113674	-0.036204964	-0.031550475
Slc25a10	1	8.96E-04	0.12534043	0.012678582	-0.036411113	-0.031608454
Gm45083	1	1.16E-26	4.6281E-24	0.001652275	-0.032069073	-0.031613047
Gm33037	1	1.27E-04	0.006802465	-0.016577249	-0.035736386	-0.031639798
Pcgf2	1	1.07E-06	4.82346E-13	-0.012439639	-0.028338633	-0.031642824
Pxylp1	1	1.55E-04	6.4023E-06	0.017041538	-0.030750013	-0.031687789
Jph1	1	8.67E-23	9.48568E-31	-0.012644856	-0.031005251	-0.031697255
1700037F0	1	4.23E-12	1.28796E-09	-0.015105502	-0.032803872	-0.031717768
Slc22a18	1	4.35E-03	1	0.004994316	-0.04476307	-0.03180612
Syde2	1	1.01E-29	4.23072E-21	0.003601683	-0.033788974	-0.031964666
Chrna4	1	1.73E-31	4.62948E-21	0.033802982	-0.034462594	-0.032080346
Slc37a4	1	8.39E-05	0.317377046	0.030954178	-0.043282005	-0.032132895
Slc39a8	1	1.22E-07	0.002923535	-0.006897952	-0.040540268	-0.032202518
Adam11	1	2.85E-06	3.23695E-16	-0.005182882	-0.026865129	-0.032220821
Fam49b	1	9.05E-06	1	-0.03145577	0.248850849	-0.032254507
Me1	1	2.97E-11	1	0.053778918	-0.14515001	-0.032261055
Tm4sf1	1	1.57E-14	1.26857E-12	-0.006336189	-0.03324098	-0.032276845
Pclo	1	2.79E-12	1.21862E-23	0.008393426	-0.030427259	-0.032293306
Gm5524	1	1.15E-17	2.73395E-07	-0.007986107	-0.038535339	-0.03239476
Pm20d1	1	2.44E-19	0.000137754	0.043886103	-0.042010224	-0.032469533
Plcg2	1	1.03E-02	1	0.006952719	0.142770552	-0.032606164
Esrp2	1	2.88E-16	5.95437E-06	-0.007667116	-0.039968705	-0.032673429
Carhsp1	1	1.60E-03	0.000132793	-0.029808428	-0.032117719	-0.032746552
Alpl	1	1.22E-27	5.97708E-14	0.013607409	-0.036029315	-0.032828923
Bri3bp	1	1.00E+00	4.89657E-05	-0.03395901	-0.02425684	-0.033014042
Apol9a	1	4.19E-19	9.61381E-23	-0.013366186	-0.032647403	-0.033028089
Trhde	1	2.19E-11	9.14743E-31	-0.011714283	-0.029875296	-0.033049605
Gm31356	1	1.66E-07	0.003546761	0.042370716	-0.03978234	-0.033175086
Chst13	1	2.12E-34	2.02567E-25	-0.024542514	-0.034381921	-0.033184503
Wipi1	1	4.02E-02	0.000347393	-0.004103959	-0.030349797	-0.033301746
Ephb4	1	2.78E-11	1.80719E-11	-0.018569508	-0.033604612	-0.033314248
Pcbd1	1	5.04E-12	1.14183E-08	0.003538466	-0.036745546	-0.03336905
Comt	1	2.19E-04	1	-0.029928374	-0.146016974	-0.033578202
Tmem266	1	1.06E-13	1.97398E-18	-0.006703563	-0.031620997	-0.033627272
S100a6	1	1.00E+00	1.4338E-07	-0.02770442	-0.018728064	-0.033662296
Dsg2	1	1.26E-18	0.318158199	0.025481004	-0.061649643	-0.033662749
Gm30301	1	7.08E-25	8.84183E-26	0.016857865	-0.033687117	-0.033755181
Tbx3	1	5.17E-08	0.005466812	-0.006890515	-0.041071044	-0.034123195
Rhobtb3	1	3.24E-16	2.45451E-08	-0.009719132	-0.037944217	-0.034128035
Mpp4	1	5.67E-18	3.69E-09	-0.00037702	-0.039657697	-0.034319209
Pla2g12b	1	2.36E-14	9.33374E-08	0.002838681	-0.037903223	-0.034327122
Zmat1	1	1.00E+00	0.036008416	0.019543589	-0.028908125	-0.034407538
Naprt	1	5.10E-12	2.55005E-06	-0.00385707	-0.038893059	-0.034438347
Gm16552	1	1.00E+00	9.1525E-06	0.002344903	-0.024296793	-0.034467025
Iyd	1	7.83E-21	4.55822E-15	-0.005773767	-0.035937609	-0.034516631
Csrp2	1	1.06E-10	1.37516E-09	-0.016083889	-0.035865433	-0.034549845

Tcim	1	7.14E-07	1.96336E-10	0.031100321	-0.032700198	-0.034642195
Ccng2	1	8.54E-03	0.105930178	-0.022100429	-0.038926982	-0.034676921
Nfyb	1	7.55E-02	0.019119203	-0.016192446	-0.032990655	-0.034806419
Mup14	1	1.01E-29	1.10988E-42	-0.022346216	-0.033788974	-0.034879059
Gria3	1	1.00E+00	1.7524E-08	-0.011379987	-0.019961155	-0.034979932
Zc3h12b	1	1.00E+00	0.00145528	-0.022716869	-0.025729135	-0.034982762
Cmya5	1	4.79E-04	7.50939E-08	-0.021865096	-0.02892193	-0.034999533
Adora1	1	5.83E-28	6.31987E-12	-0.01652441	-0.040587712	-0.035010913
Gm15998	1	4.20E-42	4.76228E-35	-0.019009821	-0.035847244	-0.035077252
Shf	1	3.57E-24	3.75737E-21	-0.030329804	-0.035978782	-0.03512675
Aldh1a7	1	1.08E-08	0.071104482	-0.017489466	-0.05087252	-0.035130696
Stard4	1	6.76E-07	2.3475E-05	0.01918437	-0.038596792	-0.035253178
Amotl2	1	1.00E-21	1.23338E-13	0.003647366	-0.039415976	-0.035317766
Cyp2ab1	1	5.63E-06	4.07301E-16	-0.027096017	-0.03066408	-0.035396428
Ccdc28a	1	8.12E-03	0.042868735	0.000853654	-0.037458227	-0.035406051
Tmem53	1	7.40E-21	6.72254E-11	-0.001857713	-0.039624749	-0.035595892
Nrxn1	1	1.89E-02	1	0.063597665	-0.043251024	-0.035637908
Erbb3	1	1.23E-09	1	0.027146056	-0.068921573	-0.035809969
Cdh4	1	5.14E-02	2.8215E-07	-0.021180936	-0.031402183	-0.035906469
Zfp101	1	3.33E-02	0.13908825	-0.030426598	-0.03842852	-0.035930839
Cox16	1	3.60E-02	1	0.028452899	-0.13495018	-0.036187557
1810055G(	1	2.66E-17	0.00175933	0.012663956	-0.050734372	-0.036232912
Alg11	1	4.21E-05	0.002375464	0.020054617	-0.040612058	-0.036297878
Kif5a	1	1.91E-01	0.005493584	-0.032395269	-0.033570945	-0.036339036
Homer2	1	4.50E-31	5.36506E-31	0.027207796	-0.036754259	-0.036366307
Echdc3	1	3.10E-05	1	-0.025103116	-0.064167175	-0.036419412
Dgka	1	1.00E+00	0.000427115	0.00250724	-0.01817889	-0.036441663
1810008I1	1	5.62E-34	3.14665E-24	0.03218415	-0.037926359	-0.036552349
Siah2	1	1.00E+00	0.000112586	-0.013029346	-0.02786013	-0.036602153
Kcnc3	1	1.03E-21	1.16858E-19	-0.000712682	-0.0367424	-0.036619724
Tom1l1	1	1.62E-25	0.021361814	-0.010323524	-0.061584357	-0.03664452
Cyp2j8	1	2.87E-36	1.00502E-35	-0.011868882	-0.036996683	-0.036738792
Kifc2	1	6.25E-21	4.52542E-17	-0.012133857	-0.03815826	-0.036786023
Gm26549	1	8.82E-03	0.010206131	-0.009680605	-0.03761703	-0.036956313
Dnm3	1	1.97E-06	1.86377E-10	-0.018149325	-0.034758977	-0.036960425
Pcsk4	1	9.05E-29	1.24689E-22	-0.009355008	-0.03867689	-0.036972465
Fmo1	1	3.58E-10	1	0.021531912	-0.072576282	-0.037003654
Ebpl	1	3.45E-05	2.31463E-05	0.02354242	-0.037590433	-0.037007766
Ddo	1	3.81E-25	1.71496E-12	-0.006154559	-0.04240192	-0.03705931
Kctd15	1	6.15E-23	6.68804E-15	-0.013522259	-0.039148799	-0.037100468
Ank3	1	9.44E-03	1	0.049127359	-0.066409123	-0.037178088
Tbx3os1	1	5.32E-10	0.207105293	-0.027044445	-0.052271026	-0.037189567
Ago1	1	1.00E+00	0.045629068	0.002756515	-0.025641398	-0.037243396
Tmem64	1	8.06E-03	0.001183684	-0.006141588	-0.037018469	-0.037274448
Asgr2	1	1.03E-12	8.19841E-08	-0.011396097	-0.042163116	-0.0374117
Retsat	1	3.07E-07	0.003453837	0.006573268	-0.04552627	-0.037460117
Ar	1	1.00E-18	2.69958E-26	-0.015477751	-0.03564404	-0.037473924
Ifitm3	1	1.00E+00	4.71872E-09	-0.015763062	-0.019676653	-0.037561289
6430590A(	1	2.66E-02	3.48097E-05	-0.01802462	-0.033496443	-0.037688024
Gpld1	1	2.24E-07	1	0.074150192	-0.112245493	-0.037691301
C1rl	1	8.77E-10	0.000146109	0.05544257	-0.044741454	-0.037819758
Steap3	1	6.43E-01	8.04394E-05	-0.037603468	-0.030048371	-0.038033513
Apoc2	1	6.36E-09	0.374196286	0.035459446	-0.062089422	-0.038171772
Adra1a	1	7.90E-16	0.710291176	-0.008013452	-0.066947832	-0.038278386
Bahcc1	1	6.90E-04	0.000495957	-0.018366573	-0.038958132	-0.038344225
Tead3	1	8.32E-20	6.39818E-17	0.003881045	-0.039324449	-0.03834991
Pnpo	1	2.70E-06	0.000134725	-0.008454912	-0.041067128	-0.038370221
Zmym3	1	2.21E-01	3.439E-06	0.003704052	-0.031627979	-0.038420493
Isca1	1	5.23E-05	0.000445573	-0.010659753	-0.041391154	-0.038468645
Camsap3	1	2.72E-20	1.6617E-06	-0.010470341	-0.046693126	-0.038484408
Fdxr	1	1.53E-02	0.014320794	0.007295015	-0.039733925	-0.03850803
Ces1g	1	1.74E-11	8.10021E-11	-0.031415316	-0.039683602	-0.038556944
Gm20619	1	8.47E-04	3.02183E-07	-0.033340566	-0.036161929	-0.038591709

Maged1	1	3.30E-13	5.61039E-07	-0.012305348	-0.04255625	-0.038622418
Slc25a48	1	5.42E-26	1.33021E-30	-0.01971454	-0.037897127	-0.038661246
Hnf1a	1	2.03E-16	8.42159E-13	-0.00248392	-0.041310033	-0.038699263
Rab4a	1	1.85E-08	0.003801416	-0.000811517	-0.051114707	-0.038762666
Gstk1	1	7.04E-19	9.8123E-19	0.001698584	-0.038959324	-0.038851368
Tlcd1	1	4.18E-14	2.14394E-07	0.017200415	-0.043958118	-0.038953401
Fads1	1	6.22E-01	8.72932E-07	-0.0005776	-0.02986857	-0.038959198
Gstt1	1	1.78E-20	1.1257E-15	-0.004424994	-0.040878328	-0.038998519
Apon	1	4.24E-18	2.92694E-25	-0.010743411	-0.037086257	-0.039097145
Hnf1b	1	2.09E-18	0.000118738	-0.002228935	-0.053008495	-0.03911611
Hyal5	1	1.00E+00	0.023044266	0.012321318	-0.018370912	-0.039128085
Elmod3	1	7.01E-06	0.317358791	0.005334783	-0.053414951	-0.039158483
Spns2	1	9.85E-19	9.70138E-26	-0.010283597	-0.037915837	-0.03948193
Miga2	1	9.74E-03	0.591531216	0.036638622	-0.04732339	-0.03955561
Pxdc1	1	5.40E-05	0.027466236	0.033530613	-0.046103356	-0.039601067
Flt1	1	1.82E-02	0.00068102	-0.015092812	-0.037066135	-0.039713971
Gm30881	1	1.00E+00	0.014310885	-0.041825602	-0.03216887	-0.039716781
Tns2	1	1.45E-16	5.34937E-16	-0.014927331	-0.040438102	-0.039722051
Asb13	1	1.06E-02	1	0.01493188	-0.050470473	-0.039775285
5430402O:	1	1.02E-04	6.33048E-07	-0.014869853	-0.037678471	-0.039793828
Acnat2	1	3.03E-33	3.98033E-18	0.019907741	-0.042900036	-0.039806831
E230016M	1	1.00E+00	8.31732E-05	-0.025310629	-0.025563351	-0.039816199
Gpr155	1	5.13E-03	2.77578E-10	-0.028341003	-0.030108069	-0.039817698
Gm48877	1	3.57E-20	7.87732E-15	0.007542144	-0.041928581	-0.03981801
Tnks1bp1	1	4.10E-11	2.0205E-11	-0.016806054	-0.039937523	-0.039841339
Cideb	1	6.44E-16	3.82295E-11	0.005809536	-0.043473085	-0.039922559
Lama3	1	5.69E-28	6.81068E-15	-0.029677416	-0.043960082	-0.039964368
Cisd1	1	3.56E-04	1.31293E-05	0.022469342	-0.038701809	-0.040273404
Fam47e	1	1.01E-26	1.2763E-37	-0.017085416	-0.039022471	-0.040336702
Tgfbr3	1	1.23E-02	0.000636223	0.000268586	-0.038447554	-0.040367751
AI661453	1	2.23E-18	1.13534E-09	-0.010831632	-0.047319516	-0.040391073
C4a	1	5.24E-20	4.37053E-25	-0.025406708	-0.039075604	-0.040501879
Steap4	1	4.18E-03	1.42638E-12	-0.016001617	-0.02723768	-0.040541784
Gm15883	1	3.94E-15	4.28336E-10	0.012250064	-0.044580236	-0.040614369
Tuba4a	1	3.45E-02	0.000390171	-0.018650259	-0.036668003	-0.040615259
Spint2	1	9.55E-06	0.043275782	-0.01161154	-0.050369025	-0.040750441
N4bp2l1	0.029579645	2.31E-01	1	0.177026854	-0.094816996	-0.040751911
Gm10069	1	4.94E-19	4.75939E-12	0.011447044	-0.045290611	-0.040938077
Trim2	1	1.89E-20	1	0.029569818	-0.097159537	-0.041030677
Klf9	1	1.40E-06	1	-0.012515167	-0.144386225	-0.04106362
Smagp	1	1.00E+00	0.014084531	0.024604407	-0.029542813	-0.041265963
Pcbp4	1	3.95E-32	1.70376E-22	-0.01826231	-0.043323875	-0.041277747
Fdx1	1	6.86E-07	0.027202365	-0.039581578	-0.052994373	-0.041279677
Acmsd	1	2.01E-15	3.95139E-11	-0.017687766	-0.044390173	-0.041285786
Slc22a15	1	1.00E+00	0.009795715	-0.000633928	-0.024450501	-0.041403662
Gm12910	1	6.97E-40	5.92332E-53	0.004372782	-0.040498648	-0.041414692
Xkr9	1	4.85E-27	0.025358306	0.013959026	-0.078343643	-0.041457289
Cblc	1	2.42E-27	6.37609E-15	-0.001926373	-0.046745943	-0.041538083
Zfp523	1	3.99E-03	9.43873E-05	-0.016346478	-0.038833517	-0.04173515
Pfkfb1	0.00639355	6.83E-11	1	0.130506959	-0.076043575	-0.04183686
Gm31121	1	1.82E-43	1.33691E-42	-0.017326803	-0.042137351	-0.041849397
Cpn2	1	1.55E-29	4.83009E-25	0.007452536	-0.042682544	-0.041860575
Bik	1	3.76E-33	1.36112E-32	-0.004418702	-0.042000088	-0.041914034
Klhl12	1	1.30E-03	0.0750362	-0.031861786	-0.049513812	-0.041940277
Josd2	1	1.19E-02	0.002800531	0.014557007	-0.042229849	-0.042033517
Acads	1	7.66E-04	0.000182529	-0.015455893	-0.042207482	-0.042107339
Osgin1	0.001675267	5.40E-03	1	-0.129865716	-0.094367175	-0.042325412
Serpind1	1	1.22E-17	8.41257E-09	0.012539177	-0.051248067	-0.042352173
Rhpn2	1	1.52E-26	8.33416E-06	0.002002294	-0.057916939	-0.042356354
Hadh	1	1.76E-04	0.041411153	-0.028904529	-0.051381071	-0.042489749
Esr1	1	1.43E-04	1	-0.032912811	-0.065691406	-0.042535798
Orm1	1	4.26E-02	2.75921E-16	-0.019682008	-0.028973356	-0.042581762
Xkr6	1	3.92E-08	1	-0.008721182	-0.061434684	-0.042642352

Fam222a	1	1.42E-15	9.58374E-13	-0.016812345	-0.044203481	-0.042818525
Rfx4	1	5.86E-29	7.61247E-40	-0.026449072	-0.041810612	-0.042868774
Gm19696	1	1.07E-33	3.15149E-23	-0.016554289	-0.045246506	-0.042871811
Agpat2	1	8.56E-18	1.16437E-15	0.001641659	-0.044885282	-0.042953353
Gm17276	1	3.38E-18	5.49931E-16	-0.024064226	-0.044169976	-0.043096666
Gm35696	1	2.88E-16	4.23992E-13	-0.025906152	-0.045628794	-0.043161537
Dmwd	1	1.75E-04	4.87888E-09	0.000475769	-0.039256305	-0.043187048
Vegfd	1	9.04E-16	3.9026E-09	-0.02434587	-0.048328106	-0.043190635
Nhsl2	1	1.37E-05	1.77833E-10	-0.02477728	-0.037952237	-0.043231393
Plcg1	1	2.90E-03	0.158570682	-0.02218868	-0.051985868	-0.043235436
Hexim1	1	3.02E-10	2.35379E-13	-0.006790576	-0.040334331	-0.043259805
Hook1	1	2.10E-24	0.000103769	0.003357777	-0.063556686	-0.043346773
Cfi	1	2.23E-12	0.145775305	0.017024842	-0.066988771	-0.043371446
Gm16157	1	1.07E-20	5.59998E-12	-0.011068144	-0.048827983	-0.04337339
Atp9a	1	1.90E-12	0.000783376	0.026799287	-0.055010867	-0.043561167
Ptgis	1	1.00E+00	3.24146E-06	-0.016175777	-0.02492717	-0.043648526
Gfod2	1	9.30E-12	1.11962E-09	0.004214426	-0.045710728	-0.043879607
Fgfr3	1	3.51E-22	4.75602E-15	-0.021552396	-0.048115652	-0.043976739
C8g	1	7.00E-13	3.99457E-08	0.020508139	-0.048300833	-0.043997975
Ak3	1	8.32E-03	0.031851423	0.034389694	-0.047481733	-0.044004838
Shpk	1	1.08E-13	8.41265E-08	-0.002517914	-0.05194137	-0.044088978
Gm20045	1	1.00E+00	0.046479791	-0.049758005	-0.036707837	-0.04418886
Sult1d1	1	6.27E-18	1	-0.026812494	-0.080909924	-0.044226568
Nid2	1	3.49E-07	9.11432E-11	0.000451555	-0.04144007	-0.04431638
Gm42109	1	2.06E-39	4.62654E-14	-0.034358514	-0.053711164	-0.044405719
Shmt2	1	5.11E-07	3.83352E-05	-0.019569888	-0.048598613	-0.044544398
Slc25a22	1	1.75E-05	1	0.035816851	-0.106309138	-0.044619777
Slc10a5	1	1.48E-29	2.30057E-16	-0.020503113	-0.049693686	-0.044690958
Klhdc8b	1	8.84E-15	4.66579E-12	-0.008207441	-0.04732542	-0.044730198
Chp2	1	4.19E-13	3.41678E-23	-0.022100192	-0.040531439	-0.044855937
Gm36975	1	5.01E-01	0.003734116	0.012101236	-0.035590441	-0.044862822
Gemin7	1	2.53E-02	3.44297E-05	-0.031904398	-0.038362609	-0.04495779
Ormdl3	1	5.51E-09	4.64703E-08	-0.008005058	-0.047603058	-0.044996337
Ntm	1	3.30E-23	6.97419E-28	-0.012633301	-0.04454743	-0.045111448
Gm30784	1	1.71E-35	5.45354E-37	-0.010541653	-0.045154469	-0.045216671
Rabep2	1	1.00E+00	0.002707394	-0.00347738	-0.032759525	-0.045245143
Fhod3	1	2.66E-27	4.52561E-41	0.020029002	-0.04327119	-0.045407223
Arl13b	1	1.00E+00	0.041353654	-0.040336246	-0.026189209	-0.045450492
Car1	1	3.83E-47	2.1254E-48	-0.031418717	-0.045543371	-0.045511222
Acsl3	1	1.41E-06	9.03506E-05	0.005848271	-0.048882775	-0.045648354
Chd9	1	1.60E-06	1	0.104504241	-0.235392122	-0.045740506
Zfp707	1	3.13E-15	7.20336E-07	-0.001108964	-0.055785791	-0.045742226
Abhd3	1	2.36E-11	5.81753E-07	-0.014957449	-0.051274494	-0.045808179
Adh7	1	2.64E-27	2.85766E-22	-0.00389591	-0.047935585	-0.045942667
Bco2	1	2.37E-04	2.11471E-05	-0.005029327	-0.045810278	-0.045959313
Dennd3	1	1.00E+00	3.29461E-06	-0.032305362	-0.025464154	-0.04598174
Parp16	1	1.84E-03	0.009623752	-0.016542971	-0.050298916	-0.046060171
Irf6	1	3.24E-38	2.29995E-30	-0.011371093	-0.048091883	-0.046099504
Abcg5	1	1.46E-27	0.044202191	0.046776189	-0.096016844	-0.046108198
Ggnbp1	1	2.05E-14	0.000128457	-2.51659E-05	-0.06124728	-0.046124965
Cutal	1	9.46E-33	1.65633E-27	0.006267308	-0.047787806	-0.046169294
Abca8a	1	4.28E-12	1	-0.038919662	-0.114269603	-0.04620709
Mtx3	1	9.35E-05	0.000907789	-0.025667283	-0.050341478	-0.046290887
Taco1	1	1.00E+00	0.034749535	-0.017894085	-0.02071114	-0.046521771
Sdsl	1	1.16E-28	2.07621E-24	-0.001111559	-0.047331154	-0.046535353
Celsr1	1	3.05E-20	9.6081E-30	-0.001684359	-0.04415462	-0.046606838
Rwdd3	1	6.76E-07	3.66032E-06	-0.019351461	-0.047894246	-0.046764214
Fam126b	1	9.12E-03	1	0.069211051	-0.064573617	-0.046890485
Garnl3	1	1.00E+00	0.006017024	-0.050518169	-0.022529129	-0.046911699
Fcgrt	1	1.00E+00	0.001029405	0.028463026	-0.03362086	-0.046970347
Snhg11	1	1.86E-05	0.064310445	-0.025949716	-0.059298761	-0.047049905
Mbl2	1	5.45E-13	3.91939E-09	-0.018046954	-0.050920255	-0.047051824
Gstp1	1	2.28E-04	2.32152E-09	-0.019546926	-0.041048877	-0.047086284

Alad	1	8.16E-11	1.9059E-09	0.01228458	-0.049788447	-0.047088001
Cdc42ep4	1	2.73E-03	0.000182849	0.002075441	-0.045161033	-0.047088715
9130016M	1	1.82E-18	1.11335E-06	0.035899445	-0.059328807	-0.0471311
Grem2	1	2.34E-23	0.003088307	-0.029014637	-0.071618527	-0.047134473
Trp53inp1	1	6.26E-07	1	0.047976516	-0.124979264	-0.047148189
Bcl7a	1	2.39E-07	5.88504E-06	0.025237783	-0.049868439	-0.047204294
Gm31522	1	2.90E-33	7.80512E-30	-0.006985415	-0.048187828	-0.04722983
Bdh1	1	7.71E-17	1	0.004023987	-0.1028693	-0.047313144
Hsf2	1	3.79E-05	0.001249864	0.034198238	-0.054022631	-0.047441418
Gm17753	1	4.04E-32	9.8827E-24	-0.005044341	-0.049507707	-0.047523409
Gm31583	1	5.70E-41	8.86397E-49	-0.016789385	-0.046654609	-0.047596438
Ppa1	1	1.46E-09	0.003577423	0.022633482	-0.060898252	-0.047614622
Camk2b	1	2.59E-15	0.082363646	0.050695396	-0.086097193	-0.047935588
Hjv	1	2.53E-25	9.52038E-07	0.011908081	-0.061746565	-0.047954401
Gm30835	1	1.85E-11	3.62405E-14	-0.026964468	-0.047007523	-0.047959887
Zswim5	1	7.22E-29	1.99733E-16	-0.001960501	-0.052339647	-0.048030792
Stk39	1	1.00E+00	0.034830242	-0.000278675	-0.02679786	-0.048090393
Pklr	1	6.44E-42	2.32612E-36	0.004765488	-0.049133878	-0.04809571
Slc22a14	1	8.10E-03	1.45559E-06	-0.025626237	-0.040397782	-0.048292396
Atp7b	1	1.52E-26	2.07758E-15	0.005615457	-0.05369985	-0.048304629
G0s2	1	4.12E-18	2.99088E-14	0.068272067	-0.050591536	-0.048305773
Gm15638	1	8.63E-38	7.90194E-28	-0.002859837	-0.051063589	-0.048337024
Slc23a1	1	2.23E-32	2.29672E-16	-0.004846042	-0.054108578	-0.0483425
Rgl3	1	3.52E-06	7.33162E-05	-0.007871392	-0.052305303	-0.048380314
Arl5b	1	1.00E+00	0.02987097	-0.024707012	-0.043460107	-0.048403011
Fbf1	1	1.60E-02	0.048542565	0.018884373	-0.053112993	-0.048598384
Vps13d	1	3.78E-02	1	0.027253263	-0.132279614	-0.048651189
Chac2	1	7.37E-05	1.97907E-07	0.016980125	-0.045701816	-0.048855894
Cdkl5	1	3.45E-08	0.001899167	-0.005823474	-0.057998944	-0.048907418
Tjp2	1	4.31E-07	0.391802577	-0.018332831	-0.074383484	-0.048925146
Etnppl	1	9.35E-12	1	-0.038527327	-0.12672645	-0.048926445
Cmbl	1	1.73E-14	1.69115E-13	0.027321692	-0.049534719	-0.048934741
Ccdc25	1	1.09E-01	0.018256362	-0.015198983	-0.047946115	-0.049124737
Mill2	1	8.45E-04	5.60874E-06	-0.039885944	-0.046552546	-0.049190615
Bud13	1	3.66E-04	2.75803E-06	-0.036566218	-0.047071614	-0.049345212
Ttc36	1	7.65E-05	0.002974944	-0.004078045	-0.054348724	-0.049371346
Aspdh	1	1.41E-43	1.3369E-24	0.011311724	-0.053040661	-0.049414464
Asic5	1	9.98E-14	9.64639E-07	0.028867974	-0.058923273	-0.049473865
Zbtb43	1	1.68E-02	0.002675471	-0.002567741	-0.047330993	-0.049497606
Hspb8	1	4.66E-19	1.90204E-12	0.012185431	-0.054894508	-0.049600625
Fahd1	1	5.44E-13	2.26732E-16	-0.005194137	-0.047335091	-0.049698858
Tpst1	1	1.00E+00	0.012533863	-0.009893008	-0.023264839	-0.049710347
Zfp266	1	1.83E-02	0.005123535	-0.029393044	-0.051993131	-0.049876512
Gm12940	1	1.00E+00	1.89524E-05	-0.002858688	-0.036612451	-0.049966307
Rarb	1	1.02E-04	2.89806E-06	-0.034907397	-0.047511746	-0.0499746
Smlr1	1	2.78E-42	2.30095E-22	0.007600828	-0.056330868	-0.050013752
Prpf40b	1	1.17E-01	3.66922E-07	-0.009585705	-0.039704094	-0.050132337
Grk4	1	1.19E-01	0.001395366	-0.012066479	-0.044483005	-0.05014589
Armc9	1	3.70E-02	0.006462905	-0.028748751	-0.049753786	-0.050208194
Pyroxd2	1	3.54E-04	0.007231123	-0.019552069	-0.055086342	-0.050257692
Smarcd2	1	1.00E+00	0.016211987	-0.005685709	-0.033009511	-0.050375065
Nqo2	1	4.62E-04	0.000675124	-0.034550431	-0.052450922	-0.0505592
Slc16a12	1	9.00E-18	5.23989E-07	-0.006454527	-0.06174726	-0.050614453
Aamdc	1	7.34E-03	1.00259E-06	-0.019831402	-0.043622515	-0.050666034
Pccb	1	1.33E-01	1.94318E-06	0.012074249	-0.041451342	-0.050735379
Scnn1a	1	2.28E-11	5.54883E-17	-0.013634857	-0.047674774	-0.050819247
Pex6	1	3.20E-02	0.638705122	-0.006887277	-0.061192955	-0.050896686
Spaca6	1	1.43E-15	2.46811E-21	-0.006235861	-0.04858589	-0.050959101
Gkap1	1	5.26E-01	0.018471271	-0.02281074	-0.044965356	-0.051084036
Lrig3	1	2.62E-12	2.91271E-08	-0.009587748	-0.057957419	-0.051086483
Gprc5c	1	6.40E-07	1.96279E-08	-0.0171233	-0.049732645	-0.051207192
Pttg1ip	1	2.30E-03	6.24692E-06	-0.02589892	-0.045715846	-0.051230989
Tmc7	1	2.24E-39	3.22963E-26	0.028951541	-0.053799897	-0.051249722

Hmgcs1	1	2.39E-05	1	0.033017292	-0.076087945	-0.051264732
AI463229	1	5.74E-17	6.27819E-07	-0.015383373	-0.06393576	-0.051294055
Il15ra	1	5.65E-03	1	0.080840965	-0.095211715	-0.051371736
Pcdh1	1	7.44E-26	8.3647E-26	-0.000925054	-0.052001399	-0.051401454
Tmie	1	1.71E-45	4.93459E-26	-0.003355032	-0.056183089	-0.051408452
Dsp	1	2.36E-22	1.18148E-12	-0.015864493	-0.058001062	-0.051495917
Tst	1	2.59E-12	1.69821E-19	-0.024248138	-0.047534385	-0.051546189
Akr1c14	1	9.94E-10	0.008113783	-0.027544577	-0.075339004	-0.051549048
Cep44	1	9.17E-04	0.002065267	-0.017794668	-0.054487602	-0.051668253
Mrnip	1	8.73E-09	2.85318E-05	0.006298752	-0.058297219	-0.051736752
Adamts6	1	5.78E-04	7.73932E-05	0.002496402	-0.049636652	-0.051784672
Agrn	1	1.17E-09	3.03425E-12	-0.021885463	-0.049312533	-0.051789497
Sesn3	1	2.91E-07	6.62358E-07	-0.016107118	-0.054164011	-0.051901862
Ptpn21	1	1.59E-09	2.31615E-05	-0.051255934	-0.059461114	-0.051916497
Acot7	1	6.58E-01	2.34458E-05	-0.027571052	-0.037940195	-0.051952659
Cbx4	1	6.13E-01	5.39515E-06	-0.015616426	-0.039480497	-0.051993386
Etfb	1	5.89E-03	0.012822233	-0.011274134	-0.055881352	-0.052002081
Nr1d2	1	4.49E-02	0.007164072	-0.040034219	-0.05094616	-0.052013286
Ppm1e	1	1.00E+00	0.001781111	-0.011879489	-0.038035421	-0.052038245
Nudt12	1	2.13E-09	1.21392E-05	-0.013935451	-0.061439818	-0.052083166
Acbd4	1	4.33E-14	5.15244E-15	-0.029713602	-0.053162969	-0.052188127
Psmd9	1	4.56E-08	0.04275339	0.0184228	-0.080619094	-0.052431803
Ccdc152	1	1.63E-22	1.73437E-35	-0.013232579	-0.049729411	-0.052513683
Btd	1	3.04E-11	8.60032E-07	-0.014777294	-0.060558342	-0.052592326
Pex11a	1	1.34E-09	0.297654556	0.075741631	-0.083932274	-0.052612923
Cyp4f17	1	1.54E-20	7.05478E-11	0.011747714	-0.064264081	-0.052624568
Nit1	1	3.16E-03	0.000710351	0.006377537	-0.053532843	-0.052789289
Mapk15	1	2.03E-32	4.78342E-35	0.00461393	-0.052703593	-0.05283333
Plin4	1	1.73E-38	4.79872E-40	-0.015443444	-0.05308328	-0.052949431
Gm27216	1	3.23E-58	2.21364E-55	-0.018416341	-0.053603079	-0.05295297
Gm26708	1	5.24E-34	6.63413E-27	0.006689535	-0.055141735	-0.052985062
Tlcd2	1	3.90E-10	4.45162E-15	-0.04271541	-0.050557354	-0.053121677
Mup21	1	5.19E-38	1.30739E-56	-0.016373241	-0.051759492	-0.053341636
Slc17a4	1	1.09E-47	1.00652E-14	0.026800137	-0.066553345	-0.053372498
Trp53bp2	1	2.65E-01	0.000598952	0.016854452	-0.046093024	-0.053390817
Sec23a	1	4.55E-03	0.86545865	0.018469179	-0.070877727	-0.053425377
BC028777	1	7.29E-29	8.92902E-26	0.009119453	-0.054505479	-0.0534982
Pnldc1	1	2.40E-24	1.13141E-14	-0.021638573	-0.059267104	-0.053504345
Mfsd4b1	1	2.17E-44	5.8735E-35	-0.035177576	-0.056447472	-0.053556861
Brap	1	4.09E-05	1	0.014440059	-0.096738463	-0.053629622
Akr7a5	1	1.51E-08	3.30605E-09	0.008543016	-0.054659929	-0.053721335
Qprt	1	1.04E-12	5.76054E-14	0.013308859	-0.054613691	-0.053889702
Bche	1	2.37E-18	1.18855E-09	0.045726762	-0.063845363	-0.05390967
Nme7	1	1.00E+00	0.017520254	-0.048556643	-0.048826777	-0.053967979
Lactb	1	8.44E-02	0.001080269	-0.038950124	-0.051135841	-0.054204482
Prickle1	1	2.41E-02	0.042822453	0.02171384	-0.056191468	-0.054255044
Mpzl1	1	1.53E-02	3.43687E-08	0.007097688	-0.042270899	-0.054383893
2310001H1	1	1.00E+00	0.013448074	-0.034912139	-0.020323134	-0.05439661
Meiob	1	2.11E-16	4.76654E-12	-0.02895713	-0.058870523	-0.054427884
Akap9	1	4.25E-07	1	0.027154364	-0.162976352	-0.054448402
Ltbp1	1	1.27E-03	1.45506E-06	-0.053003101	-0.052267121	-0.054569313
Ugt2b34	1	3.69E-10	2.52143E-07	-0.026293533	-0.058164732	-0.054584949
Atf5	1	7.61E-14	0.000226986	0.080157427	-0.079164877	-0.054814035
Pdlim1	1	5.67E-16	1.56941E-09	-0.037383864	-0.061516976	-0.055034692
Coq3	1	1.24E-01	0.00522021	0.013430086	-0.051112536	-0.055103929
Mst1	1	1.20E-12	0.117700427	0.060529152	-0.082493162	-0.055136983
Lsr	1	2.14E-10	8.57585E-10	-0.013455299	-0.05606124	-0.05514591
Nxn	1	1.00E+00	0.01006014	-0.043085138	-0.035969131	-0.055160245
Uap1	1	7.62E-04	0.000334884	-0.00639587	-0.055881427	-0.05529032
Ccdc138	1	4.32E-02	1.84734E-10	-0.02977415	-0.040075785	-0.055601603
Slc19a2	1	1.71E-17	0.000341963	0.037950777	-0.080788007	-0.055603713
Zyg11a	1	1.86E-35	7.01434E-19	-0.037446842	-0.063986775	-0.055615479
Ivd	1	4.68E-05	0.13097541	0.032845678	-0.077826096	-0.055623527

Gm29724	1	9.58E-13	1.25588E-17	-0.026992572	-0.053318083	-0.055647983
Gm47200	1	7.97E-34	1.69426E-37	-0.026309115	-0.054758745	-0.055703127
Proser2	1	1.14E-21	1.76589E-12	0.061556739	-0.064192009	-0.055738298
Ndrg3	1	1.69E-04	0.010443438	0.028654705	-0.064731245	-0.055773326
Abhd15	1	3.44E-04	0.001580945	-0.018922002	-0.060970579	-0.055803249
D230025D:	1	1.67E-03	1	0.059084596	-0.090208708	-0.055806253
Zfhx2	1	1.80E-04	9.67903E-07	0.005542834	-0.050777851	-0.055871757
Tbc1d4	1	1.00E+00	0.00538676	-0.008245235	-0.017201154	-0.056024609
Syne3	1	1.00E+00	3.78815E-10	-0.032638162	-0.035815503	-0.056053423
Hint2	1	2.91E-11	8.85741E-11	-0.012176109	-0.057137629	-0.056116545
Shroom2	1	9.64E-05	0.057822045	0.006120523	-0.078785379	-0.056339665
Anks4b	1	5.91E-43	1.99652E-27	0.004335746	-0.060694581	-0.056347526
Gm30262	1	2.34E-58	6.99E-53	-0.021102332	-0.057369427	-0.05641548
Ppp4r4	1	1.84E-32	3.3246E-28	-0.013532249	-0.057464898	-0.056422548
Nfil3	1	5.15E-11	1.20689E-09	0.004058948	-0.059785576	-0.056448833
Klb	1	3.60E-59	1.43772E-19	0.000605556	-0.067123299	-0.056464054
Dnajc22	1	9.60E-33	1.36202E-16	-0.024630405	-0.063640357	-0.05660187
Cyp4a12b	1	1.14E-52	8.97674E-51	-0.003007591	-0.05695362	-0.056617634
Ak4	1	2.21E-24	4.36637E-18	-0.038707692	-0.061023254	-0.056664641
Enpep	1	1.72E-47	1.22484E-49	-0.022725831	-0.056815715	-0.056895502
Sdr9c7	1	1.66E-60	3.6621E-35	-0.003273759	-0.061698703	-0.056906109
Nsmf	1	1.56E-03	2.49319E-05	0.015027571	-0.053721018	-0.056937614
Ugt3a1	1	3.93E-60	2.74555E-60	0.024452088	-0.057520324	-0.057062224
Slco1a4	1	1.48E-58	1	0.094805581	-0.186450249	-0.057151852
Slc25a30	1	3.64E-01	0.000459891	0.004036855	-0.050243413	-0.05717373
Cyp8b1	1	1.04E-36	2.37996E-24	-0.021272407	-0.060874827	-0.057190274
Zfp507	1	9.19E-16	7.7388E-10	-0.000872691	-0.066208212	-0.057294891
Sema4a	1	1.00E+00	1.87201E-06	-0.019742302	-0.012656905	-0.057309327
Pex5	1	5.62E-03	7.01213E-06	-0.010642143	-0.051174024	-0.057310838
Sfxn1	1	7.59E-06	1	-0.041711539	-0.101559295	-0.057484162
Nrbp2	1	4.60E-55	1.08574E-47	0.030572148	-0.058785384	-0.057485429
Sgcz	1	3.74E-12	4.95603E-18	0.027804506	-0.055292715	-0.057487005
Serpina10	1	2.72E-38	3.76726E-35	-0.010307423	-0.059306991	-0.057531987
Cfap20	1	2.34E-01	0.000182415	-0.007809278	-0.049409882	-0.057573328
Chdh	1	7.20E-23	0.025401364	-0.00830137	-0.116175665	-0.057641304
Neo1	1	5.69E-19	0.000225313	0.018356199	-0.086937647	-0.057656784
Gm15261	1	1.00E+00	3.59344E-08	-0.005637789	-0.031184831	-0.057784042
Adgra3	1	9.64E-20	2.67839E-10	-0.010547006	-0.067215775	-0.057873639
Gm15622	0.842923661	8.53E-47	4.00818E-37	0.080218018	-0.060181664	-0.057893394
Tmem205	1	4.35E-04	0.000166664	0.025320995	-0.060666769	-0.057926159
Slc3a1	1	7.48E-44	8.60127E-23	0.026805012	-0.065754697	-0.057983746
Als2cl	1	1.48E-05	0.000446959	-0.004223951	-0.064507763	-0.058029471
Tedc2	0.00435804	3.93E-20	8.04429E-15	0.118794061	-0.06356129	-0.058137865
Cmtm4	1	1.47E-03	0.00055722	0.026394596	-0.060926639	-0.058228023
Bace1	1	1.07E-17	1.20852E-13	0.015646531	-0.063779743	-0.058357922
Gfod1	1	1.00E+00	0.037152462	-0.065207661	0.026749909	-0.05850436
Plk3	1	1.18E-01	4.39285E-06	0.006573793	-0.046720401	-0.058550576
Sult1a1	1	3.43E-11	6.46522E-06	0.030444118	-0.067819628	-0.058618578
Plxnb1	1	5.92E-45	4.62282E-26	-0.037091053	-0.064758949	-0.058916451
Scap	1	7.60E-01	0.031405352	0.009229263	-0.054568137	-0.058923794
Gm13814	1	4.25E-01	0.000638116	-0.036519131	-0.049945236	-0.059088188
Gtf2i	1	7.09E-03	1	0.031225531	-0.110225532	-0.059094313
Phf11c	1	6.55E-06	0.001003351	0.014857855	-0.069878396	-0.059170029
Pex16	1	1.17E-06	2.56363E-11	-0.013539404	-0.0536309	-0.059184282
Brpf3	1	6.45E-01	0.000541879	-0.007917999	-0.048089158	-0.059554758
Ctdsp2	1	1.00E+00	0.014395566	-0.059336583	-0.049915555	-0.05958838
Mcm10	1	2.11E-02	1.55141E-12	-0.027948953	-0.040288958	-0.059625913
B430010I2:	1	8.70E-09	4.46085E-08	-0.008610149	-0.060796303	-0.059699758
Trim7	1	6.91E-06	6.92854E-14	-0.016200809	-0.047982783	-0.059701101
Camk2n1	1	2.66E-35	2.56951E-43	-0.012026611	-0.058429148	-0.059767043
Impa1	1	4.64E-01	0.000704776	-0.013614712	-0.052381189	-0.059832013
D930016D(	1	4.71E-04	0.00766142	-0.018923715	-0.068391353	-0.060020071
Cpt2	1	5.33E-08	1.16174E-05	-0.009169218	-0.067561465	-0.060105202

Pgrmc1	1	6.45E-02	0.001743735	-0.012611232	-0.057284204	-0.060150762
Serinc5	1	1.00E+00	0.008933977	-0.022127194	-0.042257024	-0.060176895
Nat8f2	1	1.70E-21	1.90338E-23	0.004197534	-0.060652396	-0.060213476
Cyp39a1	1	1.02E-22	0.994761782	0.072576214	-0.110091488	-0.060305039
Trmt9b	1	8.54E-22	3.15457E-16	-0.01084168	-0.064741849	-0.060321579
Macrod2	1	3.76E-09	9.07262E-08	-0.006471016	-0.062508326	-0.060323913
Rhod	1	2.30E-25	1.37779E-17	-0.022383281	-0.065050405	-0.060771651
1010001N(	1	1.26E-39	2.48461E-33	-0.014822128	-0.062730553	-0.060772003
Hacd2	1	2.69E-07	1	0.03019324	-0.139698415	-0.060776433
Scrn3	1	2.39E-09	0.000104366	-0.020000595	-0.075703588	-0.061006574
Dyrk2	1	1.20E-01	0.001840612	-0.031717192	-0.056860237	-0.061103749
Khdrbs3	1	1.56E-43	2.53272E-14	-0.017903538	-0.076022587	-0.061186152
Hdgf	1	7.14E-02	6.24672E-08	-0.027059174	-0.047058292	-0.061431328
Grhpr	1	4.72E-22	5.55036E-09	0.016366772	-0.07806148	-0.061509417
Ttc6	1	2.63E-38	5.14365E-31	-0.013884637	-0.063329528	-0.061521082
Tef	1	1.58E-07	5.35952E-08	0.01356869	-0.062578113	-0.061645161
Mpdz	1	1.76E-37	1.98677E-27	-0.044832627	-0.06514966	-0.061704756
1700001C1	1	2.57E-38	7.54918E-35	0.000737835	-0.062965062	-0.061715164
Nat8f1	1	2.10E-18	2.22367E-27	0.009602048	-0.057965786	-0.061859899
Slco2b1	1	3.84E-15	2.58455E-12	-0.013781656	-0.059035023	-0.061868585
Plin5	1	1.67E-28	3.38378E-27	0.030138724	-0.063721031	-0.061980239
Tprkb	1	1.03E-04	6.95453E-05	-0.011029288	-0.063577435	-0.061991309
Grb7	1	1.55E-37	4.63386E-25	0.003703188	-0.067030464	-0.062019672
Slc12a2	1	1.00E+00	0.002551489	-0.038031135	-0.047389724	-0.062137194
Rab6a	1	1.00E+00	0.018265286	-0.003614159	-0.042229121	-0.062309275
4930523CC	1	3.18E-03	6.86428E-08	0.021156951	-0.052561113	-0.062426404
Ces2a	1	6.77E-46	6.02951E-35	-0.018749408	-0.065288094	-0.062459711
Cyp7a1	1	1.08E-21	1.41019E-19	-0.042325046	-0.064491628	-0.062527133
Slc17a8	1	7.28E-28	3.40825E-15	0.007835045	-0.069736571	-0.062590005
Habp4	1	6.20E-08	3.10834E-11	-0.019587421	-0.058910333	-0.062608623
Gulo	1	7.89E-09	0.000104294	-0.043407993	-0.074206876	-0.062682575
Smad9	1	1.05E-19	1.06171E-10	-0.02664667	-0.072891372	-0.06272395
Fbp1	1	1.41E-15	1	-0.120495682	-0.210916482	-0.062811108
Ptch1	1	6.19E-10	3.26961E-09	0.001834547	-0.065860988	-0.063065641
Platr22	1	1.97E-34	8.9164E-22	-0.032791345	-0.070314113	-0.063126066
Prkcq	1	1.23E-01	1.69744E-06	-0.00184465	-0.049207069	-0.063174621
Pex1	1	5.52E-08	0.118375026	-0.037719806	-0.095691814	-0.063241546
Ercc5	1	2.66E-03	0.006009731	-0.0275278	-0.070368535	-0.063258716
F13b	1	1.17E-28	1.18824E-17	-0.010762599	-0.070320195	-0.063434018
Psen2	1	1.00E+00	0.016889236	0.035032198	-0.038323005	-0.063447685
AU022252	1	6.65E-13	4.9124E-11	-0.007354891	-0.06629181	-0.063501737
Decr1	1	2.02E-11	2.23211E-05	0.015654358	-0.08281014	-0.063641761
Fam210a	1	2.17E-01	0.004839517	-0.027991399	-0.059080574	-0.063655661
Dixdc1	1	5.98E-55	2.75971E-51	0.018523977	-0.064689574	-0.063763606
Pim3	1	1.02E-14	1.28466E-11	0.054674245	-0.069382123	-0.064272328
Dexi	1	6.25E-03	8.14527E-09	0.008437261	-0.048479301	-0.0642767
Fh1	1	1.46E-09	1.72184E-08	-0.021272093	-0.067941531	-0.064343611
Inmt	1	4.35E-72	1.46651E-74	-0.020330607	-0.064630529	-0.06447653
1810034E1	1	9.69E-06	2.95066E-10	-0.027854917	-0.058174442	-0.064553363
Nat10	1	7.84E-03	0.014995955	-0.021027156	-0.069397673	-0.064621621
Slc22a28	1	3.29E-70	6.72426E-75	-0.000961225	-0.064630529	-0.064630529
Papss2	1	2.66E-06	0.058956328	0.046181887	-0.087265516	-0.06526282
Gabarapl1	1	1.65E-06	2.51147E-07	-0.006236576	-0.068879286	-0.065378572
Fyb2	1	6.36E-20	7.48009E-06	-0.002782131	-0.091028485	-0.065565452
Ppp1r3c	1	1.28E-21	5.5078E-09	-0.03725649	-0.079030009	-0.065573672
Ppme1	1	1.00E+00	0.00595831	-0.019597169	-0.049894723	-0.065575579
Srd5a1	1	2.55E-33	2.8103E-42	0.012263035	-0.064227196	-0.065652943
Hgfac	1	2.88E-16	0.373707466	-0.059228306	-0.116535997	-0.065671703
Foxp4	1	6.05E-03	4.18212E-08	-0.029254501	-0.047019147	-0.065711933
Phlda1	1	6.60E-31	5.86715E-32	-0.022351418	-0.066098311	-0.065765885
Pald1	1	1.00E+00	0.000430692	-0.025669308	-0.037710503	-0.065807092
Dsc2	1	5.17E-35	3.15796E-33	-0.011048879	-0.066979931	-0.065843145
Apoc4	1	2.13E-07	0.060648858	0.024415886	-0.095128648	-0.065866706

Mocs2	1	4.60E-09	1.08381E-08	0.021053969	-0.070552507	-0.066099382
Fam135a	1	2.63E-10	0.261355764	0.05431325	-0.124600805	-0.066205833
Atat1	1	8.73E-08	4.67677E-12	-0.005296582	-0.060563607	-0.066210438
Cyp4f14	1	2.44E-71	6.73041E-83	-0.026684405	-0.065871494	-0.066363553
Fgfr1	1	1.22E-02	1.44491E-09	-0.013761211	-0.05409047	-0.066403501
Gm36251	1	3.44E-37	4.61892E-10	-0.013348263	-0.094076808	-0.066428602
Arhgap23	1	1.00E+00	0.000411868	-0.037461154	-0.035219926	-0.066535828
Gm3734	1	3.18E-38	1.63561E-25	0.019919135	-0.071870246	-0.066685123
Nacc2	1	1.74E-02	2.48564E-05	-0.037419212	-0.058455576	-0.066746133
C130074G1	1	1.20E-35	1.15002E-27	-0.002658318	-0.069141322	-0.066824225
Mn1	1	8.02E-42	4.21912E-31	-0.018563694	-0.069837964	-0.066909899
Rbpms2	1	1.40E-42	3.62228E-25	-0.024821174	-0.074298777	-0.066921354
Fignl2	1	1.46E-07	1.04033E-20	-0.016083783	-0.05318905	-0.066921677
Gm32063	1	4.58E-47	2.00193E-16	-0.007039149	-0.083925715	-0.067075394
Inpp5b	1	6.25E-01	0.005188179	-0.042205171	-0.057367605	-0.067092356
Adarb1	1	1.62E-17	2.0109E-13	-0.01184902	-0.074143759	-0.067094931
Rnf19a	1	3.35E-01	0.028518855	0.014187609	-0.064261864	-0.067224802
Kank2	1	3.33E-14	6.22498E-17	-0.014968063	-0.065967598	-0.067235347
Aadat	1	1.64E-60	1.29266E-41	0.007399458	-0.070462869	-0.06727432
Gm32624	1	5.38E-54	3.02778E-25	-0.034621685	-0.075963542	-0.067314704
Hadhb	1	3.95E-03	0.009750399	0.025101452	-0.073113968	-0.067360589
Lcat	1	2.74E-08	2.68038E-08	-0.010832346	-0.068853522	-0.067511367
Nectin2	1	2.13E-24	3.83288E-34	-0.029175557	-0.064767778	-0.067512276
Slc31a1	1	6.63E-02	0.000191841	-0.043650871	-0.058332819	-0.067597969
Ddrgk1	1	2.44E-04	5.79721E-11	-0.01783634	-0.056640745	-0.067847073
1700028E1	1	6.92E-08	1.67386E-08	-0.05474384	-0.068104329	-0.068450378
Galm	1	1.76E-04	0.016841632	-0.027340596	-0.080479912	-0.068485199
Rnf2	1	1.00E+00	0.001158114	-0.022549462	-0.035970143	-0.068681482
Ttc23	1	1.58E-09	0.031501702	-0.031186554	-0.101670428	-0.068696997
Ttc38	1	4.04E-18	3.34108E-15	-0.013731499	-0.073327425	-0.069196443
Angptl3	1	1.47E-06	1.62331E-07	0.037441522	-0.069258266	-0.069199115
Aldh3a2	1	7.16E-03	0.758038477	0.032601595	-0.085211873	-0.069248326
Gm12909	1	9.59E-61	3.78841E-76	0.026469352	-0.068172519	-0.069409385
Adhfe1	1	9.02E-13	4.29164E-06	-0.01849718	-0.090645201	-0.06947477
C1ra	1	8.58E-29	6.84615E-37	-0.037140434	-0.067633023	-0.0695892
Cry2	1	1.89E-18	3.13724E-14	-0.022772646	-0.075868917	-0.069626342
Ip6k2	1	8.44E-10	0.000117937	0.05908336	-0.091900528	-0.069720296
Gramd1c	1	6.99E-43	1	-0.002941954	-0.288145111	-0.069726632
Susd4	1	1.45E-56	3.02344E-64	-0.046152668	-0.069393802	-0.069761926
Coq10b	1	1.83E-02	1.86522E-05	-0.062723508	-0.062757815	-0.069850864
Fetub	1	3.97E-10	8.35775E-14	-0.039299203	-0.065022086	-0.069981548
F9	1	2.85E-38	9.08621E-18	0.013500777	-0.082529374	-0.069996519
Aldh4a1	1	5.21E-13	0.02600758	-0.0096778	-0.111521498	-0.07007174
AC149090.	1.12642E-05	1.00E+00	1	0.239154379	-0.038294025	-0.070233817
Hpx	1	1.00E+00	0.043198076	-0.041869833	-0.041366672	-0.070353723
Zcchc14	1	1.43E-12	1.14906E-05	0.006275451	-0.090953437	-0.070392636
Zfpm1	1	2.05E-17	5.26732E-31	0.011285006	-0.062622168	-0.070420371
Slc25a32	1	5.20E-05	9.99912E-07	0.022114085	-0.069712971	-0.070451507
Timd2	1	1.82E-40	8.78241E-25	0.003429031	-0.077462506	-0.070487323
1600014C1	1	3.59E-15	1.5583E-14	0.007889674	-0.073081801	-0.070497746
C8b	1	6.44E-75	1.31501E-74	-0.044366521	-0.071096155	-0.070525844
Mipol1	1	1.10E-15	6.44173E-08	0.030823641	-0.089666313	-0.070610549
Pcbd2	1	1.00E+00	0.003208595	-0.01257348	-0.055944414	-0.070788471
Ces1f	1	5.18E-13	0.370285774	-0.010671022	-0.136664824	-0.070905722
Zfp697	1	2.95E-29	6.26661E-39	-0.040394766	-0.068798378	-0.070956166
Bphl	1	1.81E-23	2.41206E-07	-0.008860214	-0.097624629	-0.070972558
Ak2	1	1.00E+00	0.00593338	0.009132172	-0.043443264	-0.07098032
Hsd11b1	1	1.27E-06	6.71532E-18	0.016340499	-0.054731548	-0.071369971
Sar1b	1	3.03E-03	0.012702863	0.081014046	-0.078661488	-0.071411229
Gm50237	1	5.43E-33	6.41007E-34	-0.001299794	-0.071685583	-0.071424059
Rtp4	1	3.67E-03	7.82756E-05	-0.040147457	-0.069176073	-0.071717343
Abhd14b	1	7.57E-28	9.23524E-26	-0.030862272	-0.073199162	-0.071849078
Fam114a2	1	1.00E+00	0.022165278	-0.052142922	-0.052099749	-0.071995859

Sptbn2	1	2.78E-32	6.57675E-32	-0.00985068	-0.072456869	-0.072015627
Mup7	1	1.78E-71	1.46579E-78	-0.033169497	-0.071850445	-0.072120431
Prodh	1	6.88E-18	5.92405E-10	0.035374103	-0.085666515	-0.072138618
Bag4	1	1.47E-09	9.91386E-11	0.035213959	-0.069810094	-0.072203454
Tram2	1	1.00E+00	2.33286E-05	-0.027140923	-0.052210842	-0.072260472
Dcaf1	1	5.60E-07	0.001278705	-0.006907374	-0.090973934	-0.072264796
1700012D1	1	2.02E-09	4.16191E-15	-0.006262817	-0.063154235	-0.072540973
Asap3	1	4.73E-45	1.88097E-48	0.001390223	-0.072639362	-0.072573312
Pxmp2	1	1.38E-27	2.1615E-16	0.003426808	-0.081780652	-0.072664405
4930556N1	1	1.10E-29	9.25502E-31	-0.004998269	-0.072530153	-0.072851974
Etl4	1	7.70E-10	0.170115362	0.003200691	-0.127946533	-0.073026238
Cda	1	1.17E-52	1.76805E-27	-0.012721817	-0.082475222	-0.073124045
Ywhaq	1	1.00E+00	0.007571451	-0.031343167	-0.028153041	-0.073276539
Nnmt	1	8.71E-33	6.73428E-31	-0.012728677	-0.075951905	-0.073644551
Vegfc	1	1.49E-19	2.41017E-12	-0.014422694	-0.07872488	-0.073783793
Insig1	1	1.16E-18	5.26317E-10	0.023166995	-0.086333625	-0.07380941
Insc	1	2.38E-55	5.17093E-29	-0.033648621	-0.082656975	-0.073815454
Selenop	1	2.44E-04	1	0.093389062	-0.127500687	-0.07401441
Fat1	1	1.86E-51	5.37304E-24	-0.021672775	-0.087619853	-0.074261959
Foxp2	1	2.15E-29	0.015566558	0.022025343	-0.14185722	-0.074317229
E130307A1	1	1.00E+00	0.011124858	-0.009406337	-0.015936169	-0.074398002
Cgn	1	1.16E-37	1.98798E-28	-0.015882026	-0.080357767	-0.07439801
Pde1c	1	2.89E-04	2.70458E-06	-0.030780761	-0.069218418	-0.074463624
Nfe2l1	1	1.00E+00	0.002273825	-0.034601779	-0.061161116	-0.074531992
Tmtc1	1	1.00E+00	0.00086293	-0.00764919	-0.048164595	-0.074576865
Arl4a	1	1.24E-18	1.68512E-21	-0.014309325	-0.073269363	-0.074626896
Snta1	1	4.55E-03	0.002964242	0.03470119	-0.076569383	-0.074764969
Siglech	1	1.00E+00	0.001071963	-0.018747531	-0.046341395	-0.074815606
Acadsb	1	3.78E-08	1.22153E-07	-0.027694042	-0.078889376	-0.074831257
Arsg	1	1.03E-10	2.04491E-13	-0.009953049	-0.071736517	-0.074860295
Elovl6	1	1.48E-11	0.000240244	0.05646652	-0.09222197	-0.074907404
Ubxn7	1	1.00E+00	0.029763033	-0.021902635	-0.051508155	-0.074988007
Npr2	1	3.77E-72	8.75282E-60	-0.008281257	-0.077342282	-0.075192009
Spin1	1	1.00E+00	0.017386506	-0.056097083	-0.061471197	-0.075205941
Gm47889	1	2.35E-32	1.20676E-28	0.011686107	-0.077063817	-0.075317943
Vcl	1	7.77E-06	0.000454652	-0.015298785	-0.077801501	-0.075333191
Sfxn5	1	1.00E+00	0.031611782	-0.040098844	-0.02164398	-0.075462145
Jmy	1	9.16E-09	0.00029437	0.013885671	-0.098445481	-0.075590734
Qdpr	1	1.54E-13	1.54173E-21	0.002976954	-0.069398962	-0.075847877
Gm16066	1	1.71E-05	7.5079E-09	-0.01261821	-0.069721248	-0.075982294
Cyp2c37	1	2.49E-44	1.04156E-18	-0.045039228	-0.089770166	-0.076204961
Bcl2l13	1	1.84E-04	1.05274E-06	-0.042063694	-0.071775083	-0.076305924
Peli2	1	9.49E-03	0.003037792	-0.003934717	-0.069749434	-0.076597973
Sned1	1	9.29E-38	1.18868E-27	0.020312137	-0.082070798	-0.076763884
Rnf43	1	1.60E-20	1.73367E-08	0.005780481	-0.096603304	-0.076799372
Nr4a1	1	1.00E+00	1.87206E-06	-0.036614567	-0.037221423	-0.077107912
Plbd1	1	1.00E+00	0.003336043	0.024827565	-0.001087528	-0.077171735
Gm11342	1	3.04E-34	6.73039E-53	0.036398218	-0.072407317	-0.077221922
Sp4	1	1.12E-02	5.69003E-12	-0.041452083	-0.054159512	-0.077227988
Fxyd1	1	8.01E-37	9.7824E-29	0.009963832	-0.080770763	-0.077261884
Isoc2a	1	2.82E-13	9.58112E-09	-0.022436895	-0.088242182	-0.07747471
Foxa3	1	1.10E-55	2.02697E-26	-0.007462433	-0.088861274	-0.077507708
Eci1	1	1.23E-19	4.98156E-19	0.011573316	-0.079338044	-0.077550549
Ncam2	1	6.95E-15	4.45666E-22	-0.013876882	-0.074604412	-0.078442459
Sorbs2os	1	2.76E-30	2.42996E-32	-0.006097559	-0.078031069	-0.078483934
Tkfc	1	2.33E-23	1.44708E-12	-0.043168856	-0.093164476	-0.078760404
Cyp2a12	1	4.64E-30	5.28214E-17	0.039521295	-0.090119308	-0.078853393
Peg3	1	7.21E-69	1.08689E-62	0.025478262	-0.080410065	-0.079129889
Paics	1	1.02E-08	3.09609E-13	0.025557507	-0.072778617	-0.079203872
Pfkfb2	1	2.14E-08	0.000113121	0.022720093	-0.095136281	-0.07934266
Dcun1d4	1	4.77E-12	9.35052E-16	-0.011784314	-0.076416058	-0.079387573
Angptl4	1	4.55E-22	7.27589E-13	0.064946453	-0.094415756	-0.079418397
Serpinf1	1	1.09E-67	3.64721E-71	-0.016629536	-0.079825705	-0.079625592

Selenoi	1	2.39E-07	3.09427E-06	-0.04206399	-0.087389827	-0.079839793
1600020E0	1	3.78E-04	0.006193516	-0.021984386	-0.091918814	-0.080076811
Cdip1	1	2.66E-06	1	0.123530899	-0.154063448	-0.080136711
Cbfa2t3	1	1.00E+00	0.014961973	-0.042770765	-0.00202916	-0.080231223
Pla1a	1	4.90E-55	6.3979E-55	-0.008946885	-0.07978652	-0.080238198
Acsl5	1	1.00E+00	0.008612864	-0.024242004	-0.036316982	-0.080533197
Bnip3	1	1.00E-15	2.51338E-11	-0.016696122	-0.090527536	-0.080593872
Cpox	1	1.38E-18	4.10224E-16	0.058917239	-0.085854366	-0.080716563
Sult5a1	1	1.00E+00	0.001875827	-0.055957991	-0.051677872	-0.080751225
Cyp2j6	1	6.85E-58	1.91797E-41	0.025845098	-0.08614861	-0.080905432
Tob2	1	3.75E-01	1.29343E-08	-0.024254082	-0.056621844	-0.080920927
Apol7a	1	1.09E-42	5.16843E-33	-0.031035806	-0.085306869	-0.08119789
Decr2	1	6.71E-17	6.55974E-11	0.043186933	-0.092414803	-0.081478268
Mccc2	1	1.76E-14	9.98445E-11	-0.025869338	-0.08919109	-0.08168691
Tesk2	1	2.50E-06	1.79589E-07	-0.009334534	-0.079271713	-0.081907221
Tmem170b	1	1.00E+00	0.021208104	-0.05624937	-0.019510092	-0.081997306
Mocs1	1	2.36E-02	0.018853543	0.007031405	-0.085919323	-0.082059438
Hibch	1	4.87E-12	3.83749E-09	-0.019457292	-0.093300736	-0.082190594
Eif4b	1	1.00E+00	0.00051984	-0.025807252	-0.05215531	-0.082397211
Hsd17b11	1	1.67E-01	0.000238348	-0.044488835	-0.068645397	-0.082557615
Hhex	1	4.19E-16	1.4338E-11	-0.040314208	-0.093480709	-0.082873653
Rgn	1	1.25E-35	9.2739E-48	-0.008183497	-0.080506193	-0.082966046
Sgk2	1	7.28E-39	6.51114E-22	0.051065052	-0.094811741	-0.08300056
Acsf2	1	2.39E-06	1.20841E-05	0.007392009	-0.091901875	-0.083010811
Dock9	1	2.34E-18	8.37625E-14	-0.05071905	-0.09046987	-0.083092502
Tjp1	1	2.54E-27	6.99996E-12	-0.021316439	-0.106323529	-0.083123069
Rasgrp2	1	1.00E+00	0.00224756	-0.012662894	-0.050449929	-0.083250673
Anp32a	1	1.00E+00	0.00016794	-0.037319449	-0.052705168	-0.083663651
Elovl5	1	2.70E-03	1	-0.041976899	-0.169938918	-0.083733579
Ccs	1	8.85E-09	0.366499491	-0.06075647	-0.149921839	-0.083911688
Mup2	1	1.65E-58	3.3861E-50	-0.033485954	-0.085942237	-0.083944248
Pros1	1	4.90E-06	2.28477E-07	0.005868904	-0.083242479	-0.084042292
Rbbp4	1	1.00E+00	0.005526294	-0.021582316	-0.071150027	-0.084735656
4833411C0	1	5.98E-40	5.20917E-18	0.00306528	-0.105211509	-0.084871055
Auh	1	1.00E+00	0.01754604	-0.064005569	-0.069576998	-0.084930406
Bend7	1	9.56E-53	3.76891E-27	-0.031210024	-0.09879019	-0.084964692
Trip4	1	7.43E-01	0.006268347	0.007270375	-0.074553921	-0.08500568
Zfp976	1	3.70E-19	9.05599E-23	-0.040798399	-0.08426317	-0.085100929
Zfp512	1	1.00E+00	2.98017E-06	-0.026469451	-0.057599704	-0.085394971
Ralgps2	1	3.49E-10	8.1791E-05	-0.02688182	-0.108308199	-0.085529226
Slc29a1	1	4.08E-04	1	0.07346464	-0.126751598	-0.085662437
Fam13a	1	1.20E-08	0.000676299	0.078994217	-0.10903347	-0.085667144
Gpd1	1	8.12E-48	1.43603E-41	-0.002433211	-0.087821937	-0.085785973
Igsf5	1	7.80E-29	2.17796E-06	0.031692598	-0.134398241	-0.085803735
Gas7	1	1.00E+00	0.001239607	-0.047382992	-0.0299665	-0.085840774
Plekha7	1	2.37E-35	1.51963E-14	-0.039660657	-0.103002639	-0.085905855
Lap3	1	9.97E-15	3.02121E-13	-0.023882193	-0.091681203	-0.086094355
Sdr42e1	1	5.78E-37	1.00832E-25	-0.028017643	-0.094776296	-0.086142443
Atp1b1	1	4.76E-30	1.15718E-17	-0.009615076	-0.096926325	-0.086245814
Tcp11l2	1	5.40E-13	9.7428E-13	-0.040150765	-0.087175235	-0.086261994
C2cd2	1	1.22E-04	0.000849158	0.047466262	-0.101782645	-0.086280528
Gm31814	1	1.21E-47	4.65892E-44	-0.037935356	-0.087047807	-0.086495038
Glyctk	1	2.33E-54	1.59183E-49	0.002315965	-0.088719606	-0.086565951
Apol9b	1	1.67E-63	8.41601E-83	-0.049657439	-0.084532059	-0.086662111
Tiam2	1	1.40E-29	2.5959E-49	-0.046361731	-0.081167241	-0.08668629
Hsdl2	1	5.95E-12	2.57219E-10	0.043066469	-0.094749207	-0.086759338
Glcci1	1	1.00E+00	0.005279255	-0.000874486	-0.068491142	-0.086925463
Zbtb44	1	7.74E-01	0.0019689	0.012137412	-0.075015046	-0.087490355
Mef2c	1	1.00E+00	0.00087093	-0.075706923	-0.048755524	-0.087638564
Gm10658	1	1.22E-45	7.71728E-55	-0.025256059	-0.086834837	-0.087676534
C730036E1	1	2.37E-100	5.7814E-107	-0.027282447	-0.087714505	-0.087714505
Sfxn2	1	7.33E-15	4.44641E-15	-0.007003038	-0.089440916	-0.087757963
Amy1	1	1.45E-10	3.36875E-11	-0.023395571	-0.089445647	-0.087890558

Aox1	1	1.11E-38	1	-0.004842181	-0.332283713	-0.088007098
4933406I1	1	5.94E-41	1.53419E-20	-0.015245143	-0.105016822	-0.088341782
Dnase2b	1	8.18E-58	4.45978E-44	0.003420099	-0.093409388	-0.088422123
Klhl3	1	6.65E-38	1.96645E-17	0.01838909	-0.106301589	-0.088517405
Mtfr1	1	1.09E-15	9.38411E-15	0.005007533	-0.094184341	-0.088600402
Pls3	1	1.45E-28	3.79743E-21	0.030273538	-0.096691744	-0.088759872
Spred2	1	1.00E+00	0.006186598	-0.056515609	-0.065127351	-0.088776532
Prkcz	1	2.00E-45	3.30294E-32	-0.041230944	-0.095590167	-0.088790797
Kcnq1	1	1.00E+00	0.000471848	0.009600248	-0.029197569	-0.08883168
9030616G1	1	2.70E-97	2.74022E-65	-0.056494071	-0.094581304	-0.089012895
Nfyc	1	1.00E+00	0.001043896	-0.091460673	-0.041538167	-0.08908929
L2hgdh	1	7.93E-33	5.6063E-26	-0.014536114	-0.095583264	-0.0891212
Gm13483	1	2.56E-20	2.28904E-12	-0.014278429	-0.099958352	-0.089179015
Gm11266	1	6.41E-91	4.06424E-54	-0.051678399	-0.096457178	-0.089208399
Rtp3	1	1.80E-78	4.32063E-78	-0.020733809	-0.089956146	-0.089263974
Clec2h	0.017297883	4.06E-75	3.85936E-78	-0.076167252	-0.089510466	-0.089386532
Rybp	1	1.00E+00	1.25819E-07	-0.031703354	-0.029351553	-0.089405595
Pde4b	1	1.00E+00	7.67789E-05	-0.064416361	-0.018815521	-0.089462732
Nars2	1	8.67E-06	0.000114218	-0.000436609	-0.100518664	-0.089579098
Reep6	1	2.54E-25	9.24813E-19	0.009545214	-0.096022542	-0.089601371
Zkscan1	1	1.09E-08	2.47461E-08	0.041944928	-0.095466715	-0.08960314
Abhd17c	1	9.70E-04	2.52863E-08	-0.038092485	-0.078247299	-0.089752844
H6pd	1	9.82E-10	4.83853E-06	-0.038364797	-0.107608606	-0.090082923
Sh3rf1	1	1.00E+00	0.011865337	-0.062173157	-0.067379871	-0.090089257
Slc22a1	1	1.07E-29	2.13817E-13	-0.021159865	-0.111159791	-0.09009259
Gata4	1	1.89E-24	2.18179E-18	-0.031147953	-0.096033085	-0.090117758
Smim14	1	1.00E+00	0.001340596	-1.38455E-05	-0.064194343	-0.090233878
Gab1	1	1.00E+00	0.000527089	-0.044624243	-0.034999406	-0.090278542
Pdilt	1	3.16E-86	3.97029E-73	-0.05958301	-0.092270737	-0.090532617
Akr1c20	1	1.42E-78	1.02062E-44	-0.006902336	-0.098592151	-0.09075484
Fads6	1	5.72E-31	4.08321E-16	-0.000216293	-0.112041118	-0.091113335
Tmem163	1	5.56E-03	1.22278E-11	0.009142521	-0.065544533	-0.091386606
Cxxc5	1	3.00E-30	2.43143E-28	-0.022608744	-0.093514885	-0.091481635
Ccdc58	1	4.16E-03	2.3499E-07	-0.007519616	-0.078814948	-0.091507829
Ces1c	1	9.07E-22	0.002707394	-0.038049725	-0.15582956	-0.091524207
Anxa6	1	3.04E-02	7.77989E-05	-0.051906781	-0.084392382	-0.091542232
Mbd1	1	3.84E-02	0.358279658	-0.067263651	-0.106596288	-0.091590863
Gm32461	1	1.55E-88	2.23542E-55	-0.008432635	-0.098837576	-0.091792201
Inpp5f	1	1.00E+00	6.21214E-06	-0.01370422	-0.057216114	-0.091961882
Fbxo21	1	6.53E-21	6.50674E-12	0.006306284	-0.109989403	-0.092097604
Efna1	1	8.97E-56	3.11013E-48	-0.038453363	-0.095419592	-0.092707614
2810459M	1	4.03E-66	2.33556E-47	0.007870171	-0.09960355	-0.09275345
Klhdc10	1	1.00E+00	0.02250808	0.019273746	-0.071483375	-0.092792594
Myo1d	1	3.07E-16	5.89728E-22	-0.011499716	-0.080420963	-0.092905123
Yes1	1	2.06E-34	1.54468E-17	-0.003829246	-0.114352903	-0.093079107
Ube2w	1	1.00E+00	0.029519303	-0.062665008	-0.056018468	-0.093200677
Kif1b	1	1.74E-09	1	0.088668029	-0.204720299	-0.093229799
Adrb3	1	3.11E-96	3.98316E-90	-0.047373218	-0.094380199	-0.093376146
Mpc2	1	7.63E-11	1.15563E-09	0.021318779	-0.099022137	-0.093475852
Asgr1	0.884657372	3.17E-24	1.90003E-26	-0.066158262	-0.094123637	-0.093721553
Fbxl17	1	1.22E-03	1	0.070531243	-0.160222296	-0.093734686
Prdx6	1	9.14E-11	2.30673E-14	-0.031317358	-0.086837995	-0.093767461
Gpt	1	3.32E-33	1.25877E-32	0.0268856	-0.095832714	-0.093984409
Cyp4a12a	1	4.32E-94	1.3028E-102	-0.020874005	-0.093899961	-0.094074002
Amacr	1	2.43E-46	4.36523E-35	-0.035176875	-0.100673931	-0.094114783
Cobl	1	2.99E-42	1.46294E-13	-0.044580381	-0.126758743	-0.094122619
Prkn	1	7.45E-20	1	0.063293957	-0.332174507	-0.094247246
Mb21d2	1	1.64E-33	1.37348E-11	-0.061693473	-0.131137703	-0.094328847
Kank1	1	9.51E-42	3.81425E-21	0.005351335	-0.108775784	-0.094374902
Habp2	1	8.49E-66	3.11489E-41	0.014123986	-0.102116469	-0.094387823
Aplp2	1	1.07E-04	0.000197787	-0.04286969	-0.100111727	-0.094448238
Bcl9l	1	6.05E-17	3.88267E-16	0.019756976	-0.098213634	-0.094763636
Got2	1	1.41E-08	3.99617E-07	-0.010007629	-0.103696983	-0.09489182

Smad3	1	1.00E+00	0.000767444	0.005715028	-0.065463563	-0.094984134
Palmd	1	1.49E-42	7.0099E-18	-0.000890803	-0.118240955	-0.095162513
Dnajc3	1	1.00E+00	0.006138949	0.007461768	-0.068143514	-0.095197635
Ugt3a2	1	2.92E-38	5.97683E-23	0.020281382	-0.107934368	-0.095328565
Exph5	1	1.31E-77	6.78085E-53	-0.032014402	-0.101605568	-0.095356408
Mllt3	1	6.29E-03	1.11506E-05	-0.011305486	-0.07884058	-0.095546888
Apoc3	1	7.80E-05	3.81128E-05	0.020498722	-0.100444063	-0.09568057
Tead1	1	7.88E-38	1.83509E-12	-0.051566112	-0.127160892	-0.095701602
Ei24	1	5.05E-15	5.85335E-11	0.025759453	-0.108243546	-0.095853757
Gm30117	1	5.95E-65	5.08656E-43	-0.057487308	-0.101603549	-0.095889462
Abca8b	1	3.08E-30	0.17562742	0.015780773	-0.259304676	-0.095893017
Kifc3	1	1.83E-10	3.2065E-13	-0.021762961	-0.09299352	-0.096004405
Pkp2	1	6.79E-62	1.08661E-21	-0.025510508	-0.121186452	-0.096069625
Pde4c	1	3.52E-72	2.43172E-42	-0.004079178	-0.105119699	-0.096100231
Them4	1	5.13E-33	1.10044E-32	0.009544646	-0.096958763	-0.096262792
Ptpn3	1	7.99E-17	2.83078E-10	-0.069051553	-0.109590799	-0.096299767
Tars	1	4.00E-09	3.21468E-16	-0.038455465	-0.085557591	-0.096464957
Lgals4	1	7.43E-44	5.84407E-25	0.035986503	-0.10975399	-0.096501245
4930404H1	0.559854022	2.06E-64	9.97816E-34	-0.067735794	-0.108577672	-0.096622403
Ggact	1	3.44E-10	7.46035E-15	-0.032347198	-0.090283637	-0.096793281
Rxra	1	1.02E-07	1	-0.017157864	-0.192646205	-0.096915506
Pnkd	1	2.28E-16	1.20585E-24	0.001948113	-0.089670223	-0.096917701
Atl2	1	7.86E-15	4.71072E-06	0.086246983	-0.130462736	-0.09697697
Bmpr1a	1	6.79E-14	5.57869E-06	-0.024813401	-0.131941882	-0.097040372
Acaa1a	1	3.79E-12	1.40122E-11	-0.006292561	-0.101106082	-0.097217589
Sod1	1	2.27E-03	1.45118E-06	-0.054229885	-0.086207929	-0.097224897
Zhx2	1	1.00E+00	0.031425519	0.013583482	-0.079492239	-0.097498044
Lgr4	1	1.55E-17	0.001299643	-0.045751267	-0.1647498	-0.098322021
Jpx	1	2.46E-10	1.60946E-14	0.000267648	-0.092649149	-0.098404076
Phyhd1	1	3.30E-15	9.18224E-15	-0.013820515	-0.102866558	-0.098476145
Ocln	1	1.46E-64	4.22456E-36	0.002615216	-0.109874743	-0.098592662
Serpina3k	1	1.97E-90	5.4673E-104	-0.028035131	-0.09767845	-0.09860243
Myo5b	1	2.99E-26	1.45905E-07	-0.007769376	-0.151153404	-0.09884525
Tmem25	1	3.84E-101	2.38644E-82	-0.035096989	-0.101262596	-0.098954693
Cdc14b	1	1.75E-06	8.06259E-07	0.011853008	-0.1035995	-0.098993181
Slit1	1	1.42E-06	7.40658E-09	-0.028216968	-0.090307312	-0.099129361
Fam193b	1	8.70E-01	0.005702013	0.028325258	-0.085222786	-0.099180785
Gm47465	1	3.27E-92	4.99342E-92	-0.031839705	-0.099776948	-0.099244912
Magi3	1	1.61E-50	0.09653593	0.044882108	-0.329772491	-0.099789248
Acsm5	1	3.25E-35	1.14598E-15	-0.027648908	-0.130695449	-0.099924289
Srrm2	0.342667752	2.21E-04	1	0.141885608	-0.163783548	-0.100326109
2210408F2	1	5.76E-05	1	-0.09651192	-0.150589698	-0.100343273
Bcr	1	1.40E-18	3.79714E-17	-0.035000598	-0.103226133	-0.100493211
Ppip5k2	1	2.19E-03	0.001510476	0.002906616	-0.106040186	-0.100506694
Bbox1	1	2.79E-62	2.04557E-21	0.049329133	-0.127089415	-0.100576212
Ifi213	1	6.51E-02	3.44149E-08	-0.047426046	-0.078069215	-0.100633757
Kdm5b	1	3.78E-07	1.29313E-08	-0.053767019	-0.098637712	-0.100729267
Agtr1a	1	8.09E-62	1.34664E-25	0.038876235	-0.119132416	-0.100801474
Lrrc28	1	7.62E-13	7.51208E-07	-0.001765246	-0.126384405	-0.100866089
Lbp	1	8.52E-42	4.49307E-31	-0.002582664	-0.108225457	-0.100880181
Pter	1	1.24E-19	1.1391E-12	0.001663876	-0.119455064	-0.101187209
Arhgap35	1	2.46E-03	0.0003173	-0.054680992	-0.09997786	-0.101390444
Slc35d2	1	5.01E-09	2.49158E-10	-0.044791832	-0.10090973	-0.101417361
Adgrl2	1	7.78E-06	0.000162252	0.06586681	-0.115676904	-0.101597649
Saa4	1	2.45E-81	5.67318E-90	0.005376218	-0.100869785	-0.101603566
Ptk2	1	1.08E-03	0.015038834	-0.044055423	-0.11687964	-0.101739462
Epb41l4b	1	1.21E-67	2.25171E-36	0.016719493	-0.113965369	-0.101933697
Rnf220	1	1.00E+00	0.031303393	-0.026237383	-0.061778928	-0.102022504
Mettl7b	1	4.11E-23	5.23421E-25	-0.017083833	-0.102003261	-0.102405539
Crim1	1	5.94E-07	1.44144E-05	-0.062133617	-0.112829998	-0.102696075
Otud7b	1	2.91E-06	6.98517E-06	0.011072906	-0.109530843	-0.102742176
Slc10a1	1	4.02E-45	3.43518E-23	0.055250955	-0.119499557	-0.102868492
Hlf	1	1.77E-50	1.65729E-41	0.07076426	-0.108492313	-0.103322716

Arhgef19	1	2.04E-84	3.69916E-76	-0.021414331	-0.10554906	-0.10333263
Syt1	1	5.67E-66	6.12502E-70	-0.031567021	-0.103418939	-0.103582516
A330040F1	1	1.00E+00	4.45771E-08	-0.05314213	-0.049173799	-0.103947999
Gm12718	1	1.19E-44	1.48769E-52	-0.057552267	-0.102950658	-0.104062417
Cd163	1	1.35E-14	0.001433377	-0.029355047	-0.198148769	-0.104197223
Agmat	1	7.90E-67	1.4279E-23	0.017935688	-0.131788234	-0.10420194
Cacna1e	1	1.44E-04	7.82259E-17	-0.017832613	-0.082357633	-0.10421145
Syn3	1	4.00E-56	6.84653E-34	-0.026397065	-0.115600393	-0.104253241
Cd302	1	1.00E+00	0.01026979	-0.088794722	-0.091133639	-0.104271533
Slc12a7	1	1.21E-04	0.000622172	-0.000360688	-0.115876044	-0.104363421
Aldh7a1	1	3.70E-17	2.82802E-15	0.016398367	-0.111528697	-0.104496331
Cyp2d10	1	6.30E-39	2.68476E-16	0.038947044	-0.132329812	-0.10495173
Gm35188	1	2.23E-10	5.41095E-21	-0.004361431	-0.090301191	-0.105027421
Cyp2c70	1	3.51E-58	4.57687E-34	0.011180337	-0.116549273	-0.105215979
Cnn3	1	1.55E-44	3.62077E-19	-0.021980928	-0.131467759	-0.105342826
Pgm1	1	6.47E-11	5.00819E-08	0.007624584	-0.120965083	-0.105457388
Nt5dc1	1	1.00E+00	0.000217869	-0.042867117	-0.041488305	-0.1054805
Serpina3m	1	5.11E-83	7.09867E-76	0.024546064	-0.108334408	-0.105663521
Nckap1	1	3.55E-13	2.28128E-08	0.034859927	-0.131665128	-0.105997211
Gm40264	1	4.05E-78	6.62767E-74	-0.037814902	-0.106475942	-0.106001819
Wdr45b	1	1.00E+00	9.2846E-08	-0.040994116	-0.067006534	-0.106308245
Npas2	1	5.16E-41	3.21707E-14	0.019049219	-0.1440287	-0.106348589
Tfdp2	1	3.71E-17	3.7241E-15	-0.037516826	-0.114539191	-0.10668491
Ralgps1	1	8.65E-03	7.09037E-12	-0.011216673	-0.071053805	-0.106876359
Kalrn	1	8.31E-21	3.10494E-39	0.014616618	-0.096904742	-0.107041304
Csad	1	2.61E-05	4.21384E-07	-0.001072462	-0.107127242	-0.107066617
Ceacam1	1	6.42E-33	2.34228E-39	-0.029701166	-0.103259372	-0.107200717
Scarf1	1	4.90E-13	3.54862E-16	-0.030559709	-0.104925294	-0.107819543
Dhrs4	1	1.96E-34	1.63081E-31	-0.023108203	-0.111723091	-0.107849472
Dap	1	1.00E-12	6.43783E-12	-0.078745981	-0.114565115	-0.107995802
Tmbim6	1	1.00E+00	0.010701952	-0.007362822	-0.063361846	-0.108215587
Arsb	1	5.24E-09	2.65438E-10	-0.033958323	-0.102300791	-0.10824607
Ccm2	1	1.00E+00	0.009528839	-0.026467928	-0.049754576	-0.108488924
Mthfd1	1	1.32E-16	2.07097E-11	0.021230353	-0.123927727	-0.108537141
Atp2a2	1	1.00E+00	0.021057808	0.031615907	-0.080517631	-0.108608785
Inca1	1	1.97E-50	2.00183E-23	0.011205824	-0.132063073	-0.108728393
Slc25a51	1	1.95E-01	3.14123E-07	0.079192089	-0.085243397	-0.108919055
Egfros	0.449413497	7.28E-100	1.1097E-101	-0.067315639	-0.109342635	-0.10895919
Dnmbp	1	6.68E-08	0.0035753	0.052523652	-0.150758627	-0.109137662
Slco2a1	1	5.15E-21	7.03798E-21	-0.009307713	-0.113789772	-0.109531124
Cyp2c23	1	5.04E-92	4.8272E-109	-0.046460688	-0.108626531	-0.109730376
Wdtc1	1	2.44E-09	1.24316E-11	-0.021419679	-0.109115985	-0.10976641
Atp8b1	1	1.50E-58	1.23229E-26	-0.020164555	-0.12979965	-0.109808777
Nhsl1	1	3.10E-28	8.46335E-10	-0.014571362	-0.151308482	-0.109877613
Khk	1	4.43E-37	9.61519E-40	-0.048420144	-0.109056386	-0.109899682
Hrg	1	5.12E-34	4.90466E-26	0.031218635	-0.116757537	-0.110005126
Slc45a3	1	2.12E-08	1.0032E-16	0.018093609	-0.093025903	-0.110309543
St3gal5	0.003010564	1.00E+00	1	0.251786798	-0.099275449	-0.110765303
Ankrd33b	1	2.68E-12	9.26818E-27	-0.021333295	-0.075328651	-0.110834201
Slc15a4	1	1.06E-02	1.4998E-06	-0.07046098	-0.095256249	-0.111163921
Rbms2	1	1.16E-01	0.000147411	0.060371392	-0.096261056	-0.111270588
Glyat	1	3.67E-43	9.94237E-19	-0.007421535	-0.140098706	-0.111835951
Sc5d	1	9.67E-32	1.67461E-27	0.016736779	-0.117385307	-0.111904216
Irs1	1	5.39E-48	8.13117E-26	-0.005897204	-0.131156242	-0.112002577
Arhgef10l	1	2.71E-08	1.03067E-15	-0.011150874	-0.098670317	-0.11207119
Gch1	1	4.78E-08	8.36961E-07	-0.018326116	-0.121008831	-0.11219897
Rnf217	1	2.41E-28	0.001897322	-0.023928181	-0.24438165	-0.112364424
Map2k6	1	6.07E-04	1.38359E-12	-0.09169103	-0.077091129	-0.112726022
Il31ra	1	6.30E-02	1.42186E-07	-0.003963775	-0.085911383	-0.112751584
Gne	1	4.58E-10	6.30233E-07	0.113928064	-0.131677683	-0.112790823
Hip1r	1	2.81E-31	1.80814E-33	-0.054372056	-0.111602644	-0.112836798
Pkhd1	1	2.19E-36	3.60217E-18	-0.012923879	-0.136669611	-0.11292612
Rnase4	1	3.38E-34	8.54737E-16	0.009408271	-0.13997436	-0.11319561

Kif21a	1	3.58E-22	8.22301E-09	0.049359461	-0.160948749	-0.113310191
Mprip	1	5.55E-03	0.001358968	0.087159497	-0.11496949	-0.113717453
Lrig1	1	2.14E-79	5.91314E-44	-0.023333082	-0.126007854	-0.113837742
Abca9	1	1.72E-03	5.2564E-05	-0.074556347	-0.10575984	-0.114225772
Agpat3	1	2.75E-09	0.032194858	0.024510786	-0.177360116	-0.114323427
Tln2	1	4.32E-57	4.32132E-44	-0.056694999	-0.120523443	-0.114364987
Yap1	1	7.65E-51	1.61468E-15	0.017399789	-0.160531387	-0.114467653
Reps2	1	1.98E-31	7.86698E-13	-0.033999085	-0.152978937	-0.114469832
Stau1	1	1.00E+00	9.08904E-05	-0.020952275	-0.080962023	-0.114513676
Slc27a5	1	2.10E-59	2.80045E-59	-0.029375777	-0.114872382	-0.114600157
Cyp2c50	1	3.01E-90	7.49004E-77	-0.020620674	-0.117438112	-0.114707496
Shmt1	1	2.98E-31	1.41523E-34	-0.007741794	-0.115561118	-0.115041666
Ankrd28	1	9.15E-07	3.41357E-12	0.013027847	-0.10139534	-0.115090737
Hs3st3b1	1	6.81E-25	5.00038E-24	-0.040494123	-0.11507213	-0.115388222
Lhpp	1	2.60E-12	5.20814E-08	-0.058542207	-0.134736306	-0.11564163
Chuk	1	3.28E-01	0.001805422	0.024683569	-0.105315828	-0.115870885
Plce1	1	1.27E-38	1.73201E-38	-0.030204864	-0.117255944	-0.116105313
C730002L0	1	2.00E-35	4.89886E-34	-0.01893313	-0.118720882	-0.116116841
Adh4	1	6.43E-71	1.13776E-81	-0.061113171	-0.114883289	-0.116400968
Gpr39	1	7.25E-79	1.20346E-48	-0.006825866	-0.126464203	-0.116418543
Kansl1l	1	1.00E+00	0.001115882	0.022878882	-0.075700438	-0.116485471
Ube2g1	1	1.00E+00	0.000658335	0.051662153	-0.060081967	-0.116548305
Grm8	1	1.10E-06	2.44353E-17	-0.004038283	-0.093834625	-0.116851485
Hsd3b7	1	3.15E-12	3.04572E-06	0.048220367	-0.150601599	-0.116867916
Nedd4l	1	3.83E-11	1	0.158727479	-0.248504994	-0.117234941
Tcea3	1	7.02E-106	9.52652E-92	-0.023812625	-0.120199367	-0.117692801
Nectin1	1	4.88E-18	5.03649E-25	0.044134344	-0.103276728	-0.117861488
Dnajc12	1	3.62E-36	2.62185E-19	0.005631526	-0.136195232	-0.117861969
Slc25a23	1	6.57E-78	2.92018E-59	-0.055690112	-0.12365846	-0.117924231
Ddx5	1	1.56E-03	1	0.082165466	-0.159284704	-0.117979076
Il6ra	1	1.08E-03	1	0.142662211	-0.163836454	-0.118040314
Numb	1	3.45E-02	0.15631716	-0.009476935	-0.12126704	-0.118088946
Agt	1	8.61E-90	1.70363E-83	0.006310802	-0.120234768	-0.118128307
Trp53inp2	1	1.74E-34	2.39079E-34	0.019054787	-0.118231938	-0.118277492
Fgfr4	1	2.95E-92	5.58707E-66	-0.064476452	-0.124280817	-0.118341609
Gm40438	1	4.72E-67	6.4468E-34	-0.06533571	-0.136571392	-0.118362585
Coq8a	1	1.43E-59	8.90338E-33	-0.013530816	-0.135846426	-0.118386641
Aldh1a1	1	4.99E-21	0.00242353	0.098971688	-0.221000787	-0.118495397
Psmd11	1	2.38E-02	0.000338282	0.004936979	-0.112335844	-0.118684392
Ube2r2	1	1.00E+00	0.00189392	-0.036968234	-0.078463221	-0.118862403
Iigp1	1	7.90E-14	4.26837E-10	0.035743686	-0.135658797	-0.119095715
Slc35e2	1	1.71E-24	2.07745E-16	0.008330929	-0.13777297	-0.119177411
Cxadr	1	1.95E-54	1.08628E-31	0.002165902	-0.13208748	-0.119266496
Prox1os	1	5.26E-22	3.29165E-14	0.078317865	-0.142091719	-0.119342167
Klf15	1	1.28E-59	5.70001E-40	0.036311759	-0.131658415	-0.119439835
Agxt	1	2.55E-81	8.66595E-77	-0.009292251	-0.1218469	-0.11943997
Lgals9	1	1.00E+00	0.000157104	-0.00283478	-0.078581706	-0.119512297
Ppp6r2	1	2.76E-07	4.04553E-10	-0.022288451	-0.114447969	-0.119595364
Prkaa2	1	1.53E-68	1.55335E-30	-0.033230224	-0.148996304	-0.119847374
Stim1	1	1.00E+00	0.002761345	-0.075879366	-0.050719362	-0.119904026
Pde8a	1	1.00E+00	0.021749265	-0.060214162	-0.091014483	-0.119961875
Gcdh	1	7.02E-47	4.07223E-22	-0.037779742	-0.150224676	-0.12002686
Zbtb7c	1	6.30E-27	1.73997E-46	-0.029947561	-0.108330452	-0.120191454
Gm47719	1	6.65E-43	3.42919E-47	-0.035761989	-0.116193125	-0.120308157
Mgam	1	1.40E-65	1.45882E-44	-0.017147423	-0.127724933	-0.12068455
Ccdc88c	1	6.87E-06	3.11806E-18	-0.062089132	-0.098758592	-0.12099965
Nadk2	1	1.01E-13	7.17363E-20	-0.010359905	-0.110479226	-0.121017077
Plekhg3	1	5.14E-10	1.09192E-10	0.006683053	-0.119319354	-0.121411264
Zkscan3	1	7.68E-05	0.000520414	0.027329999	-0.13130856	-0.121464614
Fign	1	7.30E-70	5.10634E-27	0.023318228	-0.150307207	-0.121595919
Ap3m1	1	1.06E-05	0.004099782	0.015532683	-0.14570778	-0.121631431
Sec16b	1	7.35E-51	5.91874E-13	-0.009532339	-0.178154573	-0.121704524
Aldh2	1	4.10E-11	0.065943522	-0.016790036	-0.200457313	-0.12190872

Rai14	1	1.67E-02	6.63738E-11	-0.010417442	-0.07585408	-0.121926524
Pde4d	1	1.81E-41	0.161667377	0.044466408	-0.381084442	-0.122081571
Masp2	1	2.41E-67	1.95908E-21	-0.009953302	-0.162749933	-0.122087628
Gpatch8	1	4.56E-01	0.033405298	-0.054214554	-0.106429642	-0.122206971
Zfp395	1	7.00E-06	2.92403E-11	-0.021285478	-0.103884526	-0.12244074
Ppargc1b	1	1.00E+00	4.61035E-05	0.071874763	-0.06808928	-0.122855282
Tsc22d3	0.079936507	1.50E-14	0.483172328	0.164871901	-0.233436813	-0.122993351
Gpd2	1	1.00E+00	2.00247E-12	-0.035765802	-0.066524114	-0.123078423
Pde9a	1	1.20E-123	3.85E-121	-0.040173624	-0.124147278	-0.123218971
Gabbr2	1	8.24E-52	1.43174E-71	-0.03152244	-0.116835639	-0.123371047
Ypel2	1	8.74E-03	4.11934E-07	-0.020862478	-0.10299818	-0.123452407
St5	1	1.43E-21	7.32897E-13	0.030755003	-0.156652984	-0.123677035
Xylb	1	1.68E-14	8.40609E-10	0.050156681	-0.152816655	-0.123850285
Pemt	1	2.42E-49	2.01283E-23	0.020423242	-0.150146222	-0.124110589
Sardh	1	1.11E-77	1.26619E-64	-0.021660862	-0.128973021	-0.124202064
Ccnd3	0.002862734	1.00E+00	0.649570338	0.26999874	0.008765342	-0.12421343
Mttp	1	4.82E-57	2.36557E-25	0.012033012	-0.152283638	-0.124334988
Hsd3b5	1.00324E-05	7.06E-127	3.5688E-135	-0.101172413	-0.124353257	-0.124353257
F12	1	8.97E-48	3.66556E-20	0.023415706	-0.157113092	-0.124493175
Rfx3	1	9.78E-03	1.89466E-11	-0.047094754	-0.084641526	-0.124539774
Entpd8	1	2.02E-100	2.73783E-84	0.003661219	-0.128699617	-0.124770826
Gm49980	1	2.54E-11	6.52348E-10	0.077989564	-0.138638403	-0.124828625
Car5a	1	2.63E-94	7.2587E-56	-0.02613819	-0.136623549	-0.124992319
0610040J0	1	7.59E-13	2.19694E-08	0.065354659	-0.145784219	-0.125014269
Rad23b	1	4.02E-02	2.22126E-06	-0.022293623	-0.101793464	-0.125049738
Stat2	1	7.43E-04	0.012541468	-0.049065454	-0.14256298	-0.125158381
Acsm3	1	3.10E-83	1.31104E-65	0.011607381	-0.131355687	-0.125212036
Asap2	1	2.63E-27	6.33034E-18	-0.068112664	-0.143880498	-0.125213382
Slc25a20	1	2.23E-13	1.60793E-10	0.025094432	-0.14021184	-0.125449682
Cyp2c67	1	8.13E-59	5.38134E-35	0.007885558	-0.14004223	-0.125493707
1300017J0	1	2.84E-32	1.40515E-17	-0.057003158	-0.152422298	-0.125889884
Caln1	1	5.28E-62	4.9037E-44	0.007785532	-0.132882821	-0.126109332
Faah	1	5.77E-77	2.20552E-37	0.014917538	-0.146956567	-0.126626948
Gm29571	1	2.58E-103	1.14958E-75	0.034497418	-0.132809395	-0.127008949
Fgf1	1	6.96E-96	5.1579E-108	0.008606817	-0.126089392	-0.127199352
Rdh16f2	1	1.43E-55	1.79038E-25	-0.006508198	-0.154366049	-0.127250085
Rdx	1	9.69E-04	0.000159781	-0.115767774	-0.130451336	-0.12758316
Gigyf2	1	3.97E-02	0.005718932	0.016402271	-0.116464138	-0.128143671
Gm35154	1	1.00E+00	6.96016E-08	-0.102512576	-0.023777258	-0.128314597
Bicral	1	3.04E-01	5.62777E-06	-0.025780752	-0.096540807	-0.128337072
Ncald	1	1.33E-16	1.13606E-21	-0.056726262	-0.11308647	-0.128527536
Ldlr	1	1.19E-12	5.08665E-07	0.013455931	-0.159458798	-0.128883171
Ces1e	1	6.13E-120	6.0323E-128	-0.051507667	-0.128850001	-0.128924657
Slc25a33	1	9.04E-14	2.11577E-22	-0.058164404	-0.112156069	-0.128990329
Ptprf	1	3.76E-50	1.68139E-52	-0.020127669	-0.129089142	-0.129018517
Dcaf11	1	3.27E-24	1.60267E-21	-0.019153992	-0.136363219	-0.129020407
Chchd3	1	2.23E-13	0.294657158	-0.024638317	-0.246562003	-0.129127443
Mup11	1	3.36E-116	1.3004E-136	-0.058312258	-0.127979562	-0.12929649
Ces1d	1	1.36E-55	7.62883E-87	-0.056894376	-0.122992994	-0.12964894
Dhdh	1	8.49E-25	2.10729E-17	0.005849825	-0.150628668	-0.129936565
Efna5	1	2.94E-51	8.89086E-32	0.029906814	-0.141341666	-0.130174795
Prpf4b	1	2.10E-10	0.14044002	0.083068393	-0.21717422	-0.130263739
0610043K1	1	1.82E-30	2.44817E-51	0.014281419	-0.119371375	-0.130383117
Wfdc21	1	2.91E-118	2.7262E-126	-0.011609466	-0.130161175	-0.130447236
4930402H2	1	4.38E-01	0.000149142	0.032031242	-0.094524122	-0.13048609
Ddc	1	1.62E-25	7.38594E-05	-0.006268771	-0.235091158	-0.130532483
Proz	1	1.24E-82	1.17577E-61	-0.029603612	-0.136549927	-0.130536115
Aldh6a1	1	3.82E-46	2.17227E-37	0.019846138	-0.139802098	-0.130641868
Chd4	1	5.22E-01	2.07366E-05	-0.067633421	-0.096231898	-0.130661561
Ugt2a3	0.000165739	3.19E-93	5.3026E-111	-0.094684667	-0.129362907	-0.131011137
Dipk1a	1	1.00E+00	1.65446E-08	-0.088006134	-0.070964987	-0.131034322
Gys2	1	3.74E-88	6.07489E-78	0.003133522	-0.133682905	-0.131179949
Prox1	1	1.05E-39	3.99558E-17	0.126241739	-0.172973002	-0.131415323

Dhcr24	1	1.96E-65	6.6914E-28	0.026213634	-0.158468748	-0.131521871
Pon1	1	2.63E-57	2.21689E-22	0.028805156	-0.17194726	-0.131913722
Ngef	1	4.27E-72	3.09916E-44	-0.011179618	-0.14512767	-0.131922951
Sec14l4	1	1.61E-65	5.61422E-23	-0.031679705	-0.170347036	-0.131980001
Epb41l5	1	2.34E-56	1.43888E-37	0.066317502	-0.145193942	-0.132147472
Ahcy	1	1.36E-73	4.44555E-36	0.066556983	-0.155299106	-0.132171162
Tmem243	1	7.69E-04	1.24077E-05	0.028757039	-0.124465562	-0.13242698
Gck	1	5.01E-102	3.662E-124	0.103846567	-0.130668627	-0.132458652
Hagh	1	1.08E-24	2.48012E-26	-0.04144758	-0.132913658	-0.132481317
Farp1	1	1.81E-28	9.63551E-19	-0.068364021	-0.151591591	-0.132537645
Selenbp2	0.021045487	1.68E-105	5.3484E-120	-0.089543984	-0.131831008	-0.132680787
Qsox1	1	1.30E-37	1.8932E-24	-0.068853313	-0.147306741	-0.132692013
Arhgef37	1	5.56E-15	4.7478E-17	-0.037510233	-0.123291228	-0.132813611
Slc25a15	1	7.70E-57	2.79215E-28	-0.027222495	-0.158730438	-0.132831087
Slc30a10	1	3.98E-149	4.1113E-150	0.010867151	-0.133661334	-0.132975299
Etfa	1	1.07E-06	6.48717E-09	0.015076703	-0.130274078	-0.133297867
Phlpp1	1	1.64E-02	0.000569527	0.107003475	-0.104484518	-0.133353412
Gk	0.001402662	1.46E-17	3.28645E-16	0.190917106	-0.133518543	-0.1334944
Hsd17b13	1	1.46E-31	1.28415E-17	0.075306515	-0.159943809	-0.133556118
Ppargc1a	1	2.13E-68	1.09792E-26	0.053935989	-0.16051457	-0.133575307
Paqr9	1	2.23E-18	8.86533E-27	-0.056212174	-0.122219465	-0.133980494
Hook3	1	1.06E-01	0.000872633	0.058897511	-0.115162515	-0.134212157
Rnf214	1	1.26E-05	1.16822E-08	0.01468376	-0.122424693	-0.134260378
Plac8	1	4.34E-09	2.27837E-31	-0.048377887	-0.093271402	-0.134283055
Mia3	0.168182511	4.05E-13	1.30743E-05	0.156507389	-0.18402277	-0.134462105
Fgd6	1	7.33E-09	0.001720323	-0.032362923	-0.183182181	-0.134473955
Dtnb	1	9.24E-14	5.39875E-10	-0.053749135	-0.154581304	-0.134811035
Wwox	1	4.47E-16	1	-0.133619139	-0.372703113	-0.13514902
Tmem150a	1	7.55E-63	1.46381E-69	-0.005153074	-0.134811907	-0.136018711
Cyp2j9	1	3.90E-133	4.6817E-138	-0.004534296	-0.13651313	-0.136184935
Dennd4a	1	1.00E+00	0.001285826	-0.122445117	-0.025271471	-0.136551993
Clu	1	4.88E-23	7.5827E-48	-0.059703808	-0.117342146	-0.137355069
Sephs2	1	1.13E-33	3.70057E-34	-0.023685159	-0.138007038	-0.137491052
Srr	1	7.35E-14	8.19279E-05	0.043943044	-0.202179631	-0.13754222
Dmpk	1	2.75E-01	0.000259938	0.079917123	-0.109348025	-0.138103862
Gm16573	1	4.73E-60	7.75087E-46	0.048089252	-0.14590419	-0.13813353
Cfhr1	1	5.96E-65	2.8023E-45	0.004483056	-0.150376625	-0.138406703
Sil1	1	2.92E-02	0.000145147	0.077003668	-0.114301321	-0.13882109
Pex7	1	3.14E-10	4.77885E-18	-0.049079269	-0.118293501	-0.139052188
Jarid2	1	1.00E+00	0.004996429	-0.072323988	-0.0023468	-0.139313556
Fbrsl1	1	3.07E-05	2.12509E-12	0.002005964	-0.114343971	-0.140274569
Etfdh	1	5.29E-22	1.88029E-17	0.014337887	-0.152981861	-0.140433983
Tfpi2	1	3.45E-88	1.94557E-41	-0.007028965	-0.167197314	-0.140913949
Cryl1	1	1.00E+00	0.000173521	0.0471813	-0.106028353	-0.141236278
Wwc2	1	1.03E-08	1.63553E-06	-0.060946542	-0.158644599	-0.141703531
Ambp	1	1.10E-23	1.05879E-22	-0.042523935	-0.144401747	-0.141758437
Cyp4a10	6.29465E-23	3.76E-40	8.17284E-05	0.469057221	-0.29057618	-0.141940944
Cyp3a13	1	5.11E-89	6.09242E-97	0.102156315	-0.140698789	-0.142020563
Rtn4rl1	1	1.04E-44	2.59996E-48	-0.01712133	-0.139595023	-0.142120318
Cyp2d22	1	7.67E-63	2.64011E-30	0.026580193	-0.173197022	-0.142806993
Megf9	1	3.56E-28	3.53608E-14	0.0470464	-0.171904401	-0.143155433
Rnf152	1	1.45E-103	2.20882E-67	-0.043453644	-0.153500307	-0.143272803
Cpeb3	1	1.90E-09	1.9629E-05	-0.010576612	-0.178084582	-0.143565145
Shfl	1	3.12E-38	1.58855E-29	-0.028616278	-0.158863778	-0.143590753
Gm3839	1	6.74E-134	5.7651E-145	-0.033842052	-0.143440981	-0.143615022
Akr1d1	1	6.49E-80	3.70093E-30	-0.003580897	-0.176819795	-0.144020438
Brwd1	1	1.30E-12	0.000314514	-0.01347262	-0.20626726	-0.144567044
Ppp1r9a	1	4.93E-02	0.054501821	-0.103191265	-0.148607144	-0.144690126
Zpr1	1	6.81E-23	9.55163E-18	-0.021009665	-0.162135736	-0.145040555
Srgap3	1	4.39E-04	2.78889E-14	-0.038576082	-0.097270857	-0.145766147
Macrod1	1	1.05E-40	1.46029E-27	-0.038438673	-0.166102387	-0.145775982
N4bp2	1	6.89E-12	3.90253E-24	-0.032520119	-0.124612469	-0.145999401
Prlr	1	1.06E-82	2.48068E-14	-0.027491121	-0.21377995	-0.146052254

Ddx17	1	5.13E-03	0.012590053	0.059212309	-0.144466303	-0.146698543
Mbl1	1	3.66E-129	1.6874E-128	-0.047973093	-0.147917336	-0.146867168
Tob1	1	1.63E-83	1.56944E-59	-0.064830318	-0.157295403	-0.147309189
Apba1	1	1.00E+00	0.028918796	-0.07737407	-0.029928352	-0.147407854
Gin1	1	4.47E-19	1.37776E-24	-0.003852876	-0.141455128	-0.147717115
Osbpl1a	1	4.25E-14	1.92832E-05	0.083308673	-0.209861246	-0.147856955
Cpn1	1	5.01E-79	2.67656E-53	0.008837439	-0.161813665	-0.149031753
Bcl11a	1	1.00E+00	7.90353E-12	-0.051373954	-0.095792269	-0.149270651
Bhmt2	1	1.32E-72	2.87879E-29	0.06272936	-0.183369443	-0.149589462
Ttpa	1	8.62E-39	2.12675E-14	0.122506283	-0.208425872	-0.149821299
Gpat4	1	7.15E-14	1.57568E-19	-0.023236644	-0.143006073	-0.149926375
Acss3	1	6.10E-47	9.49301E-24	0.042647545	-0.180003803	-0.150018588
Tle1	1	4.86E-32	1.23353E-06	0.06096075	-0.27583721	-0.150089392
Pcolce2	1	6.64E-06	1.20457E-15	-0.093655779	-0.105829952	-0.150355864
Znrf3	1	3.98E-07	1.80036E-08	0.031418058	-0.150367428	-0.150364957
Acadm	1	1.46E-15	5.34776E-11	0.063208962	-0.174585418	-0.15039779
Fam169b	1	1.98E-57	6.06893E-36	0.004773943	-0.167053956	-0.151078236
Stxbp6	1	4.02E-44	1.09853E-36	-0.052753224	-0.155200172	-0.151477882
Albfm1	1	1.96E-73	1.23507E-22	-0.095645797	-0.192106829	-0.151750354
Dock5	1	1.00E+00	0.000502357	-0.063906704	-0.073256129	-0.152005812
Ido2	1	3.06E-68	1.35224E-36	-0.002956626	-0.172209325	-0.152049867
Rbm33	1	2.96E-01	9.36585E-06	0.042823322	-0.109992279	-0.152451197
Ugt2b1	0.251401546	1.00E-56	6.97959E-74	-0.093245033	-0.147441752	-0.152554682
Slc25a21	1	2.30E-60	0.014841398	0.072156961	-0.471384109	-0.152576152
Nr0b2	1	5.53E-87	1.8302E-82	-0.077304443	-0.154075108	-0.153046508
Bcas3	1	1.32E-10	0.005705832	-0.079341769	-0.24161553	-0.153285024
Epb41	1	1.79E-20	0.007583178	0.03111209	-0.299899257	-0.153695616
Abcb4	0.031933171	1.39E-70	0.012974199	0.204903496	-0.582524801	-0.154148704
Slc17a3	1	2.22E-98	1.45079E-60	-0.03189876	-0.169806902	-0.154234071
Serpina1b	1	1.32E-25	3.87409E-15	-0.018554073	-0.18760923	-0.154534747
Arhgap29	1	4.73E-46	6.57329E-29	-0.039642612	-0.180387861	-0.155558291
Fam222b	1	8.43E-03	4.13547E-08	0.08109714	-0.117859726	-0.155632101
St8sia3os	1	2.05E-83	2.55717E-58	-0.079298145	-0.165248949	-0.155735043
Spop	1	1.00E+00	0.003810408	0.017197211	-0.034857157	-0.156252348
Man1a	1	1.17E-32	0.000290846	0.027663485	-0.345961307	-0.156495401
Add3	1	1.00E+00	0.000174583	-0.138189432	-0.108077321	-0.156688313
Ablim1	1	3.78E-43	1.95706E-32	-0.014331983	-0.170289763	-0.156836974
Vtn	1	1.59E-20	5.66892E-25	-0.015618881	-0.152770014	-0.157114442
Plekha6	1	7.56E-60	2.96023E-51	-0.030972031	-0.162793553	-0.157486049
Tmprss6	1	7.00E-68	5.51944E-52	0.014381209	-0.169705466	-0.157662864
Chp1	1	1.00E-16	3.11519E-15	-0.061384686	-0.165034692	-0.15773398
Myo1e	1	1.80E-06	0.000170881	0.053984636	-0.146516412	-0.157905741
Map3k13	1	4.05E-25	2.32898E-17	-0.011631411	-0.183824607	-0.158537268
Etfbkmt	1	1.41E-42	1.25414E-38	0.018688372	-0.166161955	-0.158679894
Baat	1	1.04E-123	3.83001E-84	0.025792723	-0.169889275	-0.158704221
Cep85	1	6.93E-09	7.12256E-11	0.105008411	-0.156131333	-0.15878815
Gm31333	1	8.99E-107	2.80585E-52	0.000380469	-0.182604488	-0.159176754
Myo18a	1	2.21E-09	1.11786E-08	-0.001619114	-0.163311486	-0.159337319
Fam129a	1	1.00E+00	0.002512386	-0.124689812	-0.023958874	-0.159341253
Apoa5	1	4.41E-59	2.90608E-22	0.036375826	-0.208189595	-0.159366708
Pitpnm2	1	3.39E-28	1.57263E-17	0.000391386	-0.194960226	-0.159436621
Prkd3	1	1.66E-16	7.98029E-13	0.015376646	-0.179324198	-0.159537011
Slc6a13	1	5.80E-143	6.84547E-77	0.052789846	-0.176014064	-0.159699009
Elp4	1	3.17E-06	3.04907E-07	-0.038992502	-0.154196769	-0.159722279
Ahdc1	1	1.17E-36	1.70141E-43	-0.038452152	-0.155449468	-0.159735079
Epsti1	1	1.00E+00	0.01261813	-0.132450372	-0.020170248	-0.160456829
Onecut1	0.000107474	4.67E-124	3.2986E-134	-0.126549118	-0.160445566	-0.160615555
Rdh16	1	1.71E-99	7.73063E-81	-0.017353474	-0.166580694	-0.160738952
Kcnk5	1	5.10E-111	2.35248E-74	-0.028572645	-0.169544363	-0.160856999
Hsp90ab1	1	6.04E-02	1.7938E-11	-0.029657717	-0.106072804	-0.16112837
Serpinf2	1	6.32E-93	1.46398E-50	-0.047863134	-0.184930411	-0.161170662
Gbf1	1	3.37E-08	4.07082E-05	0.034170832	-0.186463109	-0.161387977
Aadac	1	7.09E-128	9.4963E-105	-0.05725385	-0.166252663	-0.161763885

Ddi2	1	6.06E-10	0.000573018	0.086566753	-0.222268309	-0.161906666
Glo1	1	7.19E-52	1.03655E-47	0.024413194	-0.168457962	-0.162490192
Zfand3	1	1.00E+00	0.001422858	-0.084692355	-0.084089604	-0.162821036
Aqp9	1	6.47E-39	3.44472E-10	-0.029442483	-0.266915499	-0.162898959
Chka	1	5.24E-36	0.002383027	-0.055189871	-0.362701921	-0.163339317
Cyp2d26	1	5.03E-82	1.04536E-78	0.09829277	-0.167875411	-0.164082096
Arhgef18	1	3.80E-11	1.50278E-18	-0.096899913	-0.139781942	-0.164326098
Zfyve1	1	4.12E-10	3.29785E-09	-0.010315645	-0.173672112	-0.164511863
Plcl1	1	1.00E+00	4.4475E-07	-0.01151214	-0.076022665	-0.164544244
Gm13773	0.032224146	5.52E-154	2.3717E-160	-0.091012623	-0.16472737	-0.164595224
Slc20a2	1	4.16E-09	4.35075E-13	-0.044937415	-0.152133312	-0.164889821
F11	1	5.94E-84	3.68034E-35	-0.016005282	-0.204038872	-0.16495072
Dgat2	1	1.92E-81	1.36257E-58	0.041266665	-0.175574427	-0.165030884
Inpp4a	1	2.65E-02	1.7289E-09	-0.000885176	-0.120279781	-0.165166616
Deptor	1	1.74E-41	0.000250772	-0.014984156	-0.379066483	-0.165565415
Arhgap32	1	2.41E-39	2.0305E-35	-0.028393107	-0.169069231	-0.166109803
Akap8l	1	9.57E-10	6.11685E-09	0.042380417	-0.179288011	-0.166607582
Smurf2	1	2.53E-01	2.45892E-05	0.046161399	-0.117256419	-0.166673027
Gm4756	1	5.49E-93	2.3711E-126	-0.005405419	-0.161968901	-0.166810163
2900026A(	1	2.26E-36	1.23596E-19	-0.004663497	-0.214595057	-0.166859826
Rida	1	1.35E-50	2.47466E-37	0.019988244	-0.18324192	-0.167416003
Fabp1	1	3.90E-14	5.49092E-07	0.040861454	-0.221120926	-0.168219031
N4bp2l2	1	6.21E-05	1.26217E-10	-0.035219047	-0.141974588	-0.16857892
Ppm1l	1	6.85E-57	1.34915E-46	-0.016262639	-0.174492363	-0.168604796
Tom1l2	1	2.95E-14	5.58918E-09	-0.015754739	-0.200688749	-0.168723162
Cxcl12	1	1.12E-122	1.6675E-132	-0.040161561	-0.167956398	-0.168765674
Abtb2	1	4.89E-11	1.35404E-10	0.020760095	-0.146236906	-0.168927857
Aldh8a1	1	8.33E-94	1.9141E-42	-0.005299993	-0.204319089	-0.169012946
Heg1	1	1.78E-01	1.61684E-10	-0.04248901	-0.107556053	-0.169925849
Maob	1	2.91E-138	3.39567E-88	0.019888635	-0.182239797	-0.169978585
Hdlbp	1	4.59E-13	1.24127E-07	0.031342968	-0.206540888	-0.170100496
Gm49417	1	1.03E-81	2.01112E-75	-0.072496446	-0.174368964	-0.170237181
Masp1	1	8.85E-49	5.80264E-35	-0.043448663	-0.191631892	-0.171031582
Sirt3	1	3.41E-47	1.86091E-34	-0.015289124	-0.186661873	-0.171115619
St6gal1	1	1.91E-01	2.21551E-14	-0.081363576	-0.093680531	-0.171599675
Golgb1	1	5.35E-12	5.77013E-08	-0.002216131	-0.202424137	-0.171705366
Zranb1	1	5.77E-11	1.47972E-09	0.047345409	-0.183454936	-0.171711028
Pipox	1	5.25E-105	8.47932E-71	-0.029136029	-0.188178006	-0.172105751
Ssbp3	1	4.04E-15	2.73576E-17	-0.07765328	-0.168024446	-0.172121481
Grk5	1	1.00E+00	6.76571E-11	-0.082663956	-0.039884888	-0.172226626
Ppm1k	1	1.03E-49	1.87601E-40	-0.070754327	-0.183920554	-0.17231647
Ranbp10	1	2.61E-07	1.00598E-12	-0.000708621	-0.151190621	-0.17252315
Acss2	1	8.79E-29	1.70618E-28	0.007040135	-0.176326544	-0.172783616
Plxna2	1	1.83E-41	6.23642E-34	-0.096219499	-0.183556816	-0.172846064
Stat5b	1	6.11E-08	5.48666E-06	0.017054691	-0.186253882	-0.172975258
Trf	1	4.01E-16	0.000292941	-0.076161759	-0.281628689	-0.173565101
Arid4b	1	4.36E-08	1.02164E-06	-0.004994675	-0.182570579	-0.173913073
Tox	1	3.39E-51	7.61034E-05	-0.01132563	-0.372004658	-0.174726393
Kat2b	1	7.72E-11	4.11218E-06	-0.03581591	-0.208325978	-0.174755565
Hsd17b2	1	2.44E-41	2.29798E-12	-0.026421554	-0.270979953	-0.174802587
Cspp1	1	4.89E-08	6.03622E-09	0.029514813	-0.169913737	-0.1749685
Stat3	1	1.00E+00	1.23565E-07	-0.029335658	-0.077634246	-0.17520131
Adtrp	1	2.53E-97	6.90836E-53	-0.016973693	-0.19719265	-0.175288027
Slc25a25	1	3.39E-05	9.83727E-16	-0.086301359	-0.127292054	-0.175449041
Phactr4	1	1.21E-06	9.61561E-15	0.046350577	-0.144354059	-0.1756428
Nipsnap1	1	3.12E-89	1.55166E-63	-0.017000193	-0.188604117	-0.175749165
Ppp2r5e	0.145637886	3.49E-14	5.1198E-07	0.166517019	-0.230898654	-0.175789975
Plpp3	1	2.00E-18	0.000327428	-0.030832256	-0.287650744	-0.17586424
Nek7	1	4.09E-07	9.02852E-07	0.032022643	-0.172959886	-0.176007514
Pecr	1	5.29E-75	1.45451E-57	0.079488296	-0.188513667	-0.17606361
Atrn	1	1.73E-09	2.06441E-09	0.063995994	-0.179029191	-0.176189086
Ddah1	1	2.76E-29	1.92737E-14	0.023224408	-0.22698847	-0.176478176
Ehmt1	1	5.41E-13	1.85749E-07	-0.016961506	-0.216182265	-0.176672967

Shroom3	1	1.18E-68	6.96744E-08	-0.059474709	-0.407766986	-0.177014217
Acox2	1	5.81E-38	8.62762E-13	0.062687194	-0.267201973	-0.17708894
Proc	1	8.15E-49	1.15066E-32	0.029104986	-0.203315288	-0.177217575
Tmem219	1	1.47E-11	1.19454E-13	0.061934858	-0.17504903	-0.177269989
Ext1	1	9.15E-04	0.002228523	-0.095345897	-0.189588909	-0.177480235
Nos1ap	1	1.45E-08	2.32811E-17	-0.106808246	-0.147019624	-0.177898482
Apbb2	1	1.58E-19	4.21604E-14	-0.052055059	-0.189756665	-0.178398416
F2	1	1.43E-80	1.12721E-41	-0.014674875	-0.206554983	-0.178513464
Ccdc141	1	8.58E-47	1.29381E-19	0.115360813	-0.247199355	-0.178967256
Rcl1	1	7.22E-35	1.56104E-25	-0.05197997	-0.201082641	-0.179424575
Pdia5	1	3.39E-38	9.67552E-44	-0.040985226	-0.17657182	-0.179515695
Pigr	1	4.33E-134	1.1205E-162	-0.039182355	-0.176916282	-0.179520387
Mcu	1	1.42E-10	2.99744E-10	0.00111411	-0.183547738	-0.179885216
Xiap	1	8.26E-06	3.2079E-08	-0.015552253	-0.155627601	-0.180175747
Hs6st1	1	4.77E-02	4.31511E-14	-0.043649622	-0.111053724	-0.180411031
Hpn	1	4.33E-29	3.10977E-16	0.00453055	-0.230019005	-0.181851285
Mfsd2a	1	1.21E-123	1.40163E-85	0.109812316	-0.192696708	-0.182485086
Etnk2	1	3.24E-39	4.9404E-24	0.024557299	-0.218676042	-0.18293146
Trim28	1	3.62E-26	6.44908E-20	0.000168658	-0.210263754	-0.183543496
Ppp2r3a	1	4.66E-28	7.0925E-21	0.039267951	-0.207177128	-0.183555499
Luc7l3	1	2.25E-04	2.00363E-05	0.021707127	-0.164702341	-0.183555691
Slc25a42	1	5.51E-78	1.70267E-68	-0.028063149	-0.19226155	-0.184006182
Daam1	1	3.33E-10	8.1474E-14	-0.063614654	-0.170570387	-0.184055243
Caprin1	1	3.26E-10	1.46725E-07	0.029094918	-0.201972883	-0.184164215
Serpina1a	1	3.27E-53	5.44835E-38	-0.049385785	-0.204662318	-0.184230272
Lamp2	1	6.34E-13	1.55262E-07	0.068665727	-0.227877625	-0.184545837
5730522E0	1	5.64E-15	3.21631E-17	0.054982148	-0.183353107	-0.184605133
Dbi	1	7.74E-31	8.53195E-46	0.007427853	-0.169541859	-0.184756081
Mllt10	1	5.83E-13	1.64409E-06	-0.073552587	-0.237030318	-0.185380313
Sec24a	1	9.79E-30	5.93352E-08	0.071985	-0.308340046	-0.185540909
4932438A1	1	6.28E-16	1.47075E-07	-0.033890168	-0.244666599	-0.18593801
Gm2788	1	4.87E-145	1.3796E-170	-0.083118893	-0.1848421	-0.186498062
Bckdha	1	2.96E-25	2.3331E-26	0.005766233	-0.19111014	-0.186867914
Pex14	1	6.61E-20	4.87298E-09	0.045149044	-0.252744198	-0.187263978
Foxn3	1	6.04E-03	0.032821637	-0.022825817	-0.218515215	-0.187822114
Itih1	1	1.48E-107	2.18012E-75	0.032127692	-0.200765432	-0.187900729
Spata13	1	1.37E-21	2.5041E-30	-0.021820609	-0.175653225	-0.188912889
Cyp4a32	1	5.39E-90	2.21682E-64	0.009572391	-0.203557164	-0.189129419
Atxn1	1	9.11E-05	0.00049797	-0.070048431	-0.218655839	-0.189171426
Nectin3	1	7.40E-151	3.3611E-143	-0.054580041	-0.193462781	-0.190389762
Mgll	1	3.06E-54	9.68725E-14	0.091479534	-0.333122513	-0.190432994
Ggnbp2	1	1.86E-11	1.83995E-08	0.013495308	-0.209556543	-0.190978327
Sec63	1	3.22E-05	4.42563E-10	-0.034433942	-0.155240701	-0.19099201
Pdgfrl	1	5.39E-93	3.4973E-117	0.00746398	-0.188644874	-0.19148168
Sh3pxd2a	1	1.77E-03	4.25601E-12	-0.064537971	-0.134676947	-0.191512006
Stard10	1	2.17E-16	4.73013E-15	0.061234084	-0.205364128	-0.191670412
Shld2	0.504708161	4.73E-15	5.06742E-11	0.154354187	-0.220510226	-0.19192929
Dcaf6	1	1.92E-22	4.67778E-10	0.117710736	-0.261045868	-0.192062194
Hp	1	8.00E-18	2.77923E-57	0.040027772	-0.143044163	-0.192820217
Flnb	1	5.79E-19	3.57643E-15	0.015296755	-0.207901625	-0.192950168
0610005C1	1	2.58E-46	4.76189E-21	0.037930991	-0.255758904	-0.193065756
Slc17a2	1	8.85E-158	1.79884E-86	0.040904368	-0.211552491	-0.19310719
Dpp4	1	1.74E-24	1.09279E-16	-0.038687461	-0.215391471	-0.193208479
Gucd1	1	1.37E-41	1.75717E-32	-0.026687097	-0.210990087	-0.193515287
Arntl	1	7.90E-16	1.08906E-19	0.043167825	-0.186170919	-0.193659407
Cd1d1	1	1.44E-58	3.04506E-53	-0.009088985	-0.200842694	-0.193852361
Lims2	1	4.00E-114	5.72246E-78	-0.007522677	-0.208601013	-0.194186294
Zfand6	1	1.25E-10	4.99249E-10	-0.001604183	-0.194766736	-0.194288602
Mctp2	1	3.04E-25	1.43327E-13	-0.067801729	-0.201458893	-0.194315123
Fermt2	1	2.30E-45	5.99964E-27	0.017189432	-0.237203169	-0.194817569
Mrpl18	1	1.88E-68	1.4119E-44	-0.031649556	-0.216795262	-0.194982289
Dmgdh	1	8.46E-42	3.22916E-26	0.078929306	-0.231275214	-0.19556162
Pygl	1	3.08E-08	5.56706E-24	-0.021811437	-0.132008176	-0.196334408

Car8	1	6.77E-76	1.00765E-85	-0.044426913	-0.193563907	-0.196427009
Il1r1	1.94772E-05	3.63E-47	0.000189208	0.251542804	-0.427909236	-0.196788139
Dpy19l1	1	6.36E-11	1.85816E-18	-0.031328306	-0.167281713	-0.19701771
Plxna4	1	6.21E-16	4.03474E-24	-0.079344489	-0.178259851	-0.19702286
Itih4	0.083288431	1.41E-101	2.9318E-120	-0.101781051	-0.195079626	-0.197264471
Afm	1	1.83E-130	8.90518E-79	-0.069261403	-0.214149252	-0.197407211
Gm4952	1	6.27E-148	1.45967E-69	0.006998394	-0.220637454	-0.197734294
Fhit	1	2.02E-52	3.41067E-06	0.058277938	-0.433129742	-0.197757422
Olfr1033	1	6.95E-16	2.08526E-27	-0.012236441	-0.167624074	-0.197794543
Slc2a9	1	3.21E-26	1.1523E-37	-0.066304355	-0.184113476	-0.197846071
Acad11	1	5.65E-38	1.08515E-22	0.133674181	-0.241240295	-0.198315933
Slc47a1	1	2.63E-156	9.1422E-100	-0.021938003	-0.214536429	-0.198481529
Pbld2	1	1.45E-39	6.53861E-30	-0.035779097	-0.225227475	-0.199020333
Afdn	1	1.47E-19	2.74237E-19	0.005137924	-0.207743906	-0.199555702
Gls2	1	1.05E-133	5.6823E-149	0.001489267	-0.19860441	-0.199599917
Prkca	1	3.75E-22	4.36847E-05	-0.046600552	-0.310051507	-0.19987122
Nr5a2	1	1.52E-52	3.37416E-19	0.086770362	-0.293395889	-0.200399474
Apoe	1	4.45E-05	6.08997E-06	0.022623002	-0.185904114	-0.200586377
Hamp	1	2.15E-59	1.53036E-72	0.061078254	-0.197871164	-0.200625827
Tcf25	1	6.54E-12	2.27355E-08	0.073818499	-0.22121943	-0.201096911
Vwce	1	1.15E-188	3.2375E-152	-0.083097604	-0.207420567	-0.201314115
Tacc2	1	8.33E-55	8.53193E-13	0.108437394	-0.362860176	-0.201338317
Gpt2	0.005598386	6.03E-32	7.02799E-17	0.178798741	-0.257244198	-0.201797262
Strbp	1	1.09E-30	1.10855E-12	0.027898032	-0.287406691	-0.202144394
Wwc1	1	4.81E-42	5.08845E-31	0.003428547	-0.226407946	-0.202226526
Cyp2f2	1	2.06E-53	6.41011E-61	0.091382985	-0.201089673	-0.202912525
Apoh	1	7.77E-83	1.26332E-71	0.009514586	-0.213265868	-0.204673272
Atxn2	1	1.46E-21	1.85368E-08	0.056543431	-0.295133934	-0.204873736
Gjb1	1	3.91E-88	5.55323E-60	-0.030693295	-0.228634208	-0.206351329
Dlgap1	1	2.50E-82	3.2155E-113	0.017560692	-0.200389122	-0.206378423
Chpt1	1	5.22E-38	4.8347E-41	0.002841123	-0.206273467	-0.206793177
Phf21a	1	7.98E-04	1.2873E-06	-0.066393575	-0.173471467	-0.207504203
Col5a3	1	2.78E-191	1.8499E-185	0.041556848	-0.20977277	-0.207648667
Foxo1	1	1.26E-37	1.76583E-12	0.096670816	-0.320925853	-0.207693955
Ptprj	1	1.00E+00	0.020442919	-0.104423907	-0.058935533	-0.207711078
Wbp1l	1	3.88E-22	1.20412E-09	0.138219065	-0.284949092	-0.207955073
Lin7a	1	6.35E-151	2.2887E-150	-0.027540255	-0.209319508	-0.208151004
5031425E2	1	4.32E-17	1.64911E-13	0.01447343	-0.229779178	-0.208838712
Fads2	1	4.17E-129	2.89031E-95	0.062420244	-0.218515419	-0.208860161
Pdcd4	1	3.46E-24	1.90258E-32	-0.037493787	-0.193586786	-0.209093479
Gstz1	1	3.39E-59	1.72017E-46	-0.008813259	-0.227370453	-0.20916438
Nfix	1	4.50E-28	1.78268E-25	-0.07299877	-0.21860709	-0.20919277
Bcl6	0.240715449	5.14E-07	2.36053E-24	0.169468791	-0.143691374	-0.209293187
Slc25a47	1	3.53E-83	1.35848E-58	0.028298275	-0.227591172	-0.209395787
Pcsk6	1	4.59E-53	4.7363E-23	0.036704574	-0.286768214	-0.209600171
Nr1i2	1	7.58E-40	3.61187E-24	0.121358426	-0.25023755	-0.209914482
Cald1	1	1.80E-60	6.47438E-17	0.004909133	-0.34534341	-0.21054459
Gcnt2	1	7.47E-19	2.47564E-33	0.104555543	-0.163512555	-0.210865635
Insr	1	6.16E-26	6.34835E-11	0.005108864	-0.30121873	-0.211752748
Rbp4	1	3.72E-42	3.10825E-15	0.068148097	-0.318091443	-0.212069269
Large1	1	1.00E+00	2.19325E-09	0.09100217	-0.095741315	-0.212927947
Herpud1	1	7.79E-32	1.48157E-29	0.11921213	-0.221474565	-0.213115193
Enpp3	1	2.05E-135	1.198E-142	-0.062801646	-0.21416063	-0.21419381
Hibadh	1	1.52E-15	3.88883E-14	-0.040041206	-0.223026349	-0.214234438
Fry	1	1.00E+00	4.88339E-24	-0.100789973	-0.086704332	-0.214523548
Gm50136	1	2.32E-202	5.7605E-201	0.007253787	-0.215989217	-0.214696945
Abat	1	1.36E-134	2.6633E-109	-0.00453925	-0.223654462	-0.214910416
Vgll4	1	1.91E-16	3.97557E-13	-0.037519443	-0.234640405	-0.215399264
Ptms	1	3.82E-38	2.31863E-54	-0.02520159	-0.201356808	-0.215429239
Rapgef4	1	1.91E-71	2.12664E-05	0.158578474	-0.616537047	-0.215431025
Gsta3	1	1.28E-46	9.83299E-13	0.052237087	-0.353032449	-0.215455251
Hivep2	1	1.00E+00	4.82927E-11	-0.146589537	-0.074266876	-0.215672743
Nedd4	1	6.75E-45	1.45555E-21	-0.024492365	-0.283721981	-0.216667554

Vegfa	1	2.32E-60	1.69003E-52	0.029673013	-0.227865758	-0.2167031
Elovl2	1	3.28E-144	1.51154E-57	0.051360278	-0.254983564	-0.216853949
B630019A1	1	1.07E-61	6.66085E-42	-0.068691376	-0.247184494	-0.217039017
Tenm3	1	8.13E-64	2.39784E-11	0.043547157	-0.390321042	-0.217059989
Rad54l2	1	1.81E-23	9.44945E-20	0.03113764	-0.238165587	-0.218502378
Zcchc24	1	1.72E-26	9.13485E-40	-0.082315917	-0.198030167	-0.218562048
Cabyr	1	3.09E-102	2.8144E-121	-0.088649887	-0.216199274	-0.218648588
Parva	1	1.65E-76	3.88174E-73	-0.021129987	-0.227400989	-0.219183703
Nox4	1	6.44E-174	3.353E-180	-0.092605674	-0.220271704	-0.220061834
Stard13	1	5.15E-56	2.41373E-09	-0.072892159	-0.460808748	-0.220384897
Cadps2	1	3.51E-128	1.759E-111	0.009185793	-0.227491037	-0.220571406
Fam20a	1	3.18E-109	6.44082E-95	0.048232597	-0.228637923	-0.221020823
Gcgr	1	3.58E-52	2.29343E-30	0.004653623	-0.266717853	-0.221044682
Ralgapa2	1	5.03E-23	1.4352E-09	0.083933815	-0.3256558	-0.22174098
Acaa2	1	7.63E-49	8.14345E-51	0.010998268	-0.225363997	-0.221801485
Epas1	1	3.50E-31	7.69077E-37	0.088706726	-0.216220977	-0.221823342
Nfia	0.000215958	1.31E-60	7.18254E-08	0.262364077	-0.626351056	-0.223543447
Clybl	1	8.90E-20	2.34285E-20	0.039025668	-0.223673351	-0.224198288
Sipa1l1	1	3.33E-19	2.08282E-14	0.058708888	-0.249611787	-0.224534235
Cbs	1	7.47E-88	1.28036E-53	0.025547601	-0.258326791	-0.225262629
Lactb2	1	9.15E-39	2.05786E-16	0.032884018	-0.317809526	-0.225451362
Mtss1	1	3.33E-16	0.000252735	0.007006654	-0.361465974	-0.225832358
Cdc42bpa	1	1.12E-54	1.84894E-29	0.029001884	-0.285378102	-0.227049906
Acsm1	1	3.65E-127	9.101E-103	-0.101678338	-0.23768811	-0.228416892
Gpam	1	1.73E-48	4.37176E-25	0.084494453	-0.29351326	-0.228911525
Timd4	1	1.73E-18	1.41344E-29	-0.042372809	-0.188738942	-0.229583774
Clec4f	1	2.10E-16	8.14937E-07	0.0911596	-0.324692105	-0.230193987
Myo6	1	7.20E-61	5.08083E-24	0.01449751	-0.327042059	-0.230293024
Eda	1	4.68E-136	3.2147E-109	-0.04595286	-0.239523315	-0.231593171
Cdo1	0.01079978	1.99E-57	5.57125E-15	0.198927201	-0.400257255	-0.231834072
Neb	1	4.47E-121	1.2645E-129	-0.081830283	-0.232597402	-0.232658809
Eps8l2	1	1.58E-51	1.29595E-44	0.021301455	-0.249493085	-0.232765914
Pcca	1	1.54E-30	6.75313E-14	0.003672449	-0.322818829	-0.233856487
Fam107b	1	1.28E-04	2.72822E-22	-0.060336111	-0.153067712	-0.234146238
Ces3b	1	5.66E-203	2.2105E-218	-0.082633732	-0.234290153	-0.234464194
Gas2	1	7.13E-71	4.79433E-68	-0.014751426	-0.238946098	-0.234603898
Pcsk5	1	2.36E-146	1.30592E-69	-0.031253944	-0.268156542	-0.234987801
Tnrc6b	1	1.77E-13	2.91756E-11	-0.020146451	-0.268730147	-0.236071102
Cep85l	0.001668865	3.48E-44	3.1042E-14	0.196063846	-0.378598096	-0.236159462
Zfp385b	1	6.75E-68	2.39708E-65	0.06510545	-0.242337751	-0.236480135
Tfr2	1	2.80E-138	4.90853E-92	0.008182699	-0.255869011	-0.236554033
Hsd3b2	1	2.84E-210	9.0259E-226	-0.093247761	-0.236810757	-0.236962514
Serping1	1	7.02E-132	6.8999E-126	-0.073731577	-0.240975763	-0.237003719
Cpt1a	1	1.68E-21	1.59046E-17	0.060452879	-0.259491459	-0.237269885
Kat6b	1	2.59E-12	3.85735E-21	-0.0305836	-0.200624206	-0.237923835
Akap1	1	2.56E-78	2.83309E-66	-0.023560546	-0.250318063	-0.237994357
Raph1	1	6.46E-17	3.02134E-20	0.011694134	-0.222001315	-0.23830072
Slc2a2	1	4.87E-201	7.194E-107	0.024701454	-0.261867073	-0.238622233
Kcnn2	1	2.40E-54	2.49613E-45	0.115777128	-0.256541628	-0.238873344
Oaf	1	1.16E-133	2.7857E-112	-0.034171111	-0.245676748	-0.23893673
C4bp	1	1.07E-92	1.0214E-80	0.03860221	-0.247457377	-0.239172593
Acat1	1	4.06E-33	5.88626E-21	0.121146588	-0.284933443	-0.239178996
Cgnl1	1	4.23E-43	2.64638E-42	-0.006428695	-0.246516134	-0.239317418
Mgst1	1	2.41E-38	7.54361E-38	-0.024361262	-0.246724943	-0.239607314
Pbx1	1	8.41E-15	7.17425E-20	-0.051399346	-0.181259734	-0.239662211
Bach2	1	6.38E-13	3.49942E-13	-0.013287216	-0.229048183	-0.240531426
C3	1	1.83E-20	2.83481E-25	-0.116002175	-0.227086185	-0.240644398
Cyp2c54	1	6.75E-181	1.3621E-211	-0.089381237	-0.238661444	-0.241000993
Txlng	1	5.55E-30	2.97416E-19	0.082110136	-0.287123341	-0.241142363
Scd1	1	3.38E-09	2.55023E-64	-0.045540142	-0.142062528	-0.242677052
Adgre4	1	3.34E-18	1.61219E-19	-0.047849785	-0.226385126	-0.243088763
Gm49431	1	9.47E-229	6.2989E-212	-0.004850214	-0.246705221	-0.243298086
Gm13775	1	8.14E-87	2.67158E-72	-0.032384044	-0.254761428	-0.243825824

Ripor2	1	5.35E-07	4.81766E-38	-0.118096801	-0.106750763	-0.243997592
Zbtb16	1.68818E-06	6.47E-76	6.79096E-05	0.351729167	-0.661635936	-0.24468242
Myo1b	0.46635597	1.20E-72	3.32806E-14	0.152611294	-0.462912453	-0.244964216
Lrba	1	1.81E-25	1.32698E-21	-0.040228119	-0.259066617	-0.24528063
Tmem131l	1	1.71E-05	4.0072E-21	-0.016590429	-0.144561019	-0.24536884
Cyp27a1	1	3.02E-45	3.9368E-14	0.134347735	-0.390640703	-0.245837857
Igfbp4	1	3.75E-89	4.8529E-107	-0.019822288	-0.241457194	-0.246635719
Ece1	1	8.48E-20	7.94224E-27	0.036495165	-0.226561796	-0.24674587
Fga	1	2.17E-73	1.00252E-42	0.083731676	-0.292946243	-0.24736852
Bmp1	1	2.39E-177	8.2334E-133	0.040830503	-0.25924884	-0.247933664
Pabpn1	1	1.32E-33	1.20894E-20	-0.009170329	-0.301451514	-0.248028155
Mical3	1	1.15E-41	1.91987E-35	-0.093573792	-0.26507127	-0.24882194
Upb1	1	1.11E-57	5.63265E-43	0.047607083	-0.278344272	-0.249095861
Ppm1b	1	2.37E-29	6.36086E-22	-0.001946651	-0.280986361	-0.249427378
C1s1	1	1.52E-162	6.8037E-129	-0.008021077	-0.261061074	-0.249470416
Bckdhb	1	4.47E-58	1.32129E-17	-0.022879065	-0.412816738	-0.249567361
Slc38a3	1	3.52E-68	7.42812E-53	-0.060501697	-0.27079181	-0.250355419
Acnat1	3.78477E-05	1.29E-84	8.30921E-26	0.242688802	-0.405661371	-0.251185622
Gprin3	1	2.56E-151	6.677E-120	0.012846053	-0.260628977	-0.251422524
Sgms2	1	1.64E-31	8.64794E-62	-0.08534611	-0.201700016	-0.251917274
Nr6a1	1	1.04E-22	1.47213E-19	0.003193635	-0.266824313	-0.251970912
Plcxd2	1	1.10E-43	2.34375E-49	-0.013199458	-0.252629718	-0.252013436
Tspan12	1	5.66E-70	6.10563E-47	-0.014064671	-0.288893436	-0.252625181
Cth	1	1.45E-57	8.79795E-26	-0.136895294	-0.330800705	-0.2530458
Otc	1	1.24E-178	9.0166E-137	0.003089183	-0.265187384	-0.25339043
Enpp2	1	1.32E-102	2.04433E-90	-0.112554731	-0.263288107	-0.254582115
Sdc4	1	3.73E-65	4.02464E-29	0.100717817	-0.346604174	-0.25646276
Ehbp1	1	2.60E-30	1.14224E-26	-0.02093939	-0.2744425	-0.256663167
Zfhx4	1	7.71E-140	8.1415E-101	0.043669906	-0.270353031	-0.257617585
Optn	1	3.42E-33	1.20003E-27	0.018046718	-0.279112807	-0.258497183
Ctcflos	1	8.01E-173	1.1481E-119	0.065168701	-0.271592426	-0.258950486
Arhgap26	1	3.07E-01	1.49391E-21	-0.083751815	-0.113350302	-0.259132354
Kng2	1	1.63E-87	1.13331E-62	-0.012354024	-0.280852584	-0.259231286
Tcf7l1	1	3.83E-31	1.32036E-30	-0.064828718	-0.272940049	-0.259510381
Cecr2	1	1.27E-144	2.804E-126	-0.027954618	-0.267556327	-0.260104879
Arhgef3	1	9.76E-27	1.92369E-17	-0.009200121	-0.304084386	-0.260189683
Sec14l2	1	5.57E-131	4.9728E-112	-0.033631881	-0.272341388	-0.262197118
Arhgef12	1	4.65E-45	1.77854E-17	-0.004657497	-0.397387224	-0.262489362
Acat3	1	4.25E-91	2.03514E-75	-0.064744208	-0.278124765	-0.26386821
Ndrg2	1	2.10E-129	1.3706E-101	-0.067294282	-0.278985739	-0.264163988
Onecut2	1	8.80E-59	6.63203E-42	0.013693822	-0.298239416	-0.264904753
Haao	1	4.06E-102	7.0446E-106	-0.095177564	-0.265110528	-0.264910653
Klkb1	1	6.54E-112	2.45314E-96	0.037414394	-0.275723526	-0.265627254
Gm48633	1	4.02E-157	9.8833E-100	-0.053449038	-0.282914687	-0.265660959
Arhgap5	1	1.94E-35	9.28487E-21	0.142168613	-0.330947151	-0.266121704
Lpin1	0.000125487	3.12E-43	4.86022E-06	0.208463379	-0.473455304	-0.266999972
Dhtkd1	1	2.16E-69	3.20861E-51	-0.03198462	-0.296005708	-0.268061002
Fah	1	9.47E-86	3.7813E-75	-0.035321924	-0.284823397	-0.269838189
Baiap2l1	0.193262294	7.05E-80	7.80923E-27	0.159498966	-0.410432421	-0.271160513
Cyp2c38	3.64926E-06	2.97E-79	2.15416E-48	0.289521049	-0.308412168	-0.27167685
Sntg2	1	1.52E-184	4.0385E-220	-0.104682514	-0.269041568	-0.272431207
Cat	1	2.61E-59	2.22218E-14	0.130534018	-0.532221172	-0.273494922
Cyp4f15	1	5.19E-193	3.9022E-190	-0.006255523	-0.276513718	-0.273600392
Tex14	1	3.82E-20	1.94939E-31	-0.07432962	-0.238203161	-0.273753841
Tspan9	1	1.96E-47	1.23344E-52	-0.041705948	-0.269931548	-0.274917185
Ube2e2	1	1.06E-65	5.32591E-13	0.052412375	-0.532596456	-0.275101226
Rdh7	1	5.11E-85	1.70781E-63	0.084611188	-0.300301021	-0.275689184
Ell2	1	5.04E-27	7.8358E-23	0.115402828	-0.288213876	-0.275689727
Lpcat3	1	1.34E-19	5.02098E-25	0.026124887	-0.253450203	-0.276269217
Slc22a23	1	1.15E-44	9.9496E-26	-0.060831038	-0.343030016	-0.27634741
Ror1	1	3.70E-65	2.01869E-74	-0.049350208	-0.274599678	-0.276643684
Col27a1	1	1.66E-118	3.2269E-183	-0.055387846	-0.262986244	-0.276765578
Fchsd2	1	1.86E-27	2.09346E-25	-0.019960258	-0.281121187	-0.279702934

Slc16a2	1	2.18E-78	5.83171E-87	-0.125883061	-0.281747734	-0.280595193
Gramd3	1	1.19E-57	5.2335E-30	0.111672858	-0.360707136	-0.280742093
Cped1	1	2.62E-85	1.26587E-19	0.033492521	-0.52666583	-0.282423354
Cmtm8	1	1.35E-56	5.59953E-82	-0.05105541	-0.264234636	-0.282788861
Abcd3	1	2.96E-52	4.51069E-24	0.072664457	-0.387132147	-0.283168089
Elovl3	1	6.74E-258	2.2277E-274	-0.086351769	-0.286739395	-0.286739395
Erc2	1	1.09E-63	1.14901E-55	-0.084816081	-0.304232025	-0.287067603
Pank1	1	2.81E-47	5.4043E-33	0.048074459	-0.327300689	-0.287406593
Ern1	1	4.95E-23	7.73583E-24	-0.070697341	-0.285153757	-0.288763519
Asl	1	1.97E-56	8.09693E-51	0.133672547	-0.304849859	-0.29011159
Aldh1l1	1	1.19E-79	9.82952E-31	0.065400226	-0.414912044	-0.292030396
Cyp2d9	1	3.09E-136	8.34122E-96	-0.051663113	-0.315089336	-0.292038814
Rora	1	6.73E-68	6.76434E-24	0.070743839	-0.466959325	-0.29216965
Hykk	1	1.15E-171	8.1416E-125	-0.040262838	-0.310364396	-0.292543375
Hal	1	2.20E-148	2.8454E-91	0.011864484	-0.315129919	-0.29292616
5033403H(	1	2.75E-193	1.8675E-182	-0.0093105	-0.297662266	-0.293141832
Gldc	1	1.58E-68	3.64112E-48	0.144627636	-0.331279772	-0.293636679
Ncoa1	1	1.45E-09	4.54379E-18	-0.052986843	-0.227313296	-0.294409143
Lifr	1	1.13E-27	2.05062E-31	-0.003385379	-0.284559367	-0.294956681
Cp	0.246381953	3.39E-77	8.8017E-19	0.175785097	-0.562055801	-0.295445764
Gm4951	1	7.79E-57	4.11677E-16	-0.008016696	-0.523096924	-0.295632066
Hoga1	1	8.68E-73	1.0403E-46	-0.041830064	-0.347138858	-0.296623554
Necab1	1	2.17E-181	1.1218E-157	0.029816436	-0.304915914	-0.297194195
Dapk1	1	4.85E-05	9.09447E-10	-0.059457666	-0.241986023	-0.297302908
Plekha5	1	7.98E-73	3.61181E-24	-0.023061998	-0.474769249	-0.297353475
Ube2h	1	2.21E-31	3.71609E-21	-0.065463243	-0.348446233	-0.298125686
F5	1	6.90E-78	8.56501E-42	-0.028245478	-0.376053027	-0.298436997
Shb	1	3.65E-42	1.94947E-42	0.133260977	-0.303540741	-0.298966927
Kmo	1	5.91E-64	1.04136E-78	-0.031293519	-0.286989695	-0.30049095
Got1	1	3.77E-59	1.55294E-58	-0.141017436	-0.305485442	-0.301408619
Msi2	1	1.85E-31	1.90587E-24	-0.001446319	-0.33551015	-0.303098947
Hnf4a	1	1.08E-83	3.66933E-37	0.06558316	-0.406427294	-0.303997925
Gpc4	1	1.44E-187	8.2576E-145	-0.054408134	-0.316073349	-0.304468585
Zfand4	1	7.81E-39	5.86985E-61	-0.02108788	-0.27668935	-0.304667426
Prkce	1	2.21E-34	1.86627E-21	-0.058566317	-0.364093237	-0.305947505
Map7	1	3.51E-87	1.47653E-40	0.002787268	-0.406159251	-0.305948823
4732465J0	1	2.14E-76	1.76075E-97	0.09846322	-0.293069276	-0.3061302
Pcx	1	1.71E-70	7.57413E-39	0.12352239	-0.373323922	-0.306880553
C4b	1	1.87E-78	1.39984E-76	-0.049869567	-0.315813591	-0.307131043
Erc1	1	1.08E-64	4.01337E-29	-0.009664593	-0.427182746	-0.307672914
Acot12	1	7.00E-80	2.29153E-55	0.093570808	-0.351030145	-0.310795677
Il1rap	1	6.59E-57	9.43428E-90	-0.037405537	-0.277215635	-0.311874043
Dennd5b	0.150046166	2.72E-86	1.45712E-27	0.17470288	-0.514634167	-0.313235255
Lonp2	1	9.33E-60	2.59258E-23	0.085021748	-0.507905871	-0.313505383
H2-Q10	1	3.08E-74	2.16704E-53	0.084101865	-0.348626448	-0.313760634
Fgg	1	1.05E-137	3.76814E-95	-0.021359739	-0.341094367	-0.315455883
Luc7l2	1	8.19E-26	2.90689E-23	0.035277123	-0.339048295	-0.315541981
Arhgap24	1	2.63E-03	2.39462E-30	-0.017492098	-0.116326754	-0.315846056
Tex2	1	1.88E-45	2.86555E-29	-0.055619037	-0.364153845	-0.316351203
Nudt7	7.73995E-08	9.08E-110	1.19727E-86	-0.235018943	-0.334668	-0.317449469
Acbd5	1	4.44E-34	6.47242E-31	-0.012545888	-0.332083528	-0.318742585
Suclg2	1	4.80E-52	5.01494E-33	-0.044096378	-0.382747784	-0.318775591
Cyp3a11	0.124903269	1.15E-75	2.20791E-20	0.207962861	-0.570959389	-0.31931815
AI182371	1	1.59E-111	4.67024E-86	-0.085719449	-0.341370143	-0.319711988
St3gal3	1	5.40E-47	1.87898E-25	-0.063000381	-0.425408307	-0.320062511
Ppp1r3b	1	3.31E-63	1.88803E-65	0.108226785	-0.324551038	-0.322940137
Gm26917	1	1.84E-35	2.71733E-25	0.093202837	-0.403338824	-0.323158275
Creb3l3	1	4.44E-72	6.94978E-56	0.111726003	-0.356129182	-0.324080015
Pde4dip	1	8.16E-70	1.7315E-42	0.042492593	-0.391357741	-0.326388997
Nr1i3	1	1.51E-88	1.37755E-30	-0.014591128	-0.493676788	-0.326417281
Igfbp2	1	3.65E-191	6.3102E-107	-0.059880373	-0.354743478	-0.326417737
Utrn	1	6.21E-15	2.6373E-22	-0.06755148	-0.281904923	-0.326756575
Keg1	0.025414753	1.84E-232	2.6192E-255	-0.167914328	-0.325734476	-0.327186284

Arl15	1	2.79E-27	2.56366E-21	-0.1503691	-0.37824993	-0.327648202
Tmtc2	1	4.84E-73	5.46394E-31	0.035069591	-0.460260729	-0.330324767
Arg1	0.001047649	3.91E-92	7.43366E-24	0.243055307	-0.568573094	-0.330918723
Fn1	1	6.61E-116	6.69948E-20	-0.083517958	-0.638080439	-0.331140442
Mir99ahg	1	1.48E-71	7.86637E-50	-0.076074239	-0.373395732	-0.331359999
Clec2d	1	6.42E-19	1.86181E-30	0.080436691	-0.269932883	-0.331486105
Amdhd1	1	1.12E-154	7.8698E-101	-0.031967049	-0.362641482	-0.332233862
Shank2	1	1.71E-93	4.0388E-109	-0.113973547	-0.329765559	-0.33231903
AY036118	0.106202877	2.22E-15	1.01367E-23	-0.18355891	-0.274997311	-0.333113062
Fgb	1	2.73E-85	4.24746E-56	0.08546283	-0.385182084	-0.334038396
Abca6	1	2.19E-106	1.63939E-33	0.07315931	-0.547638142	-0.334070381
Nr3c2	1	6.12E-86	1.55988E-36	-0.077117845	-0.469737218	-0.334080914
Ahsg	1	3.23E-93	8.8614E-22	0.134701201	-0.594039175	-0.335907092
Gckr	1	1.77E-92	1.62237E-47	0.081580493	-0.423898446	-0.336787722
Sord	1	5.71E-71	2.56463E-46	0.008166733	-0.392171619	-0.336875332
Msra	1	2.32E-47	3.72547E-31	0.052313612	-0.406771311	-0.336956452
Ulk2	1	6.49E-25	1.2582E-29	-0.072202039	-0.31381711	-0.338839289
Tshz2	1	1.81E-73	2.38743E-52	-0.111679338	-0.375275287	-0.339170628
Rnf125	1	9.79E-77	4.24875E-35	-0.080316224	-0.439210825	-0.339342432
Sorbs1	1	1.70E-89	4.4255E-52	-0.021478139	-0.410018573	-0.33940343
Psd3	1	1.80E-50	3.47148E-20	-0.098044506	-0.549485338	-0.340833322
Agxt2	1	2.60E-103	5.97157E-46	-0.04346507	-0.4573253	-0.341640369
Rere	1	3.65E-30	8.10461E-27	-0.011503169	-0.378696045	-0.341915961
Lurap1l	1	1.43E-70	8.27937E-51	0.022894434	-0.37998377	-0.342045878
Itpr1	1	2.93E-34	2.28256E-25	-0.030067659	-0.37164078	-0.343498181
Dlc1	1	3.15E-39	6.30674E-19	0.009676779	-0.490490437	-0.343655918
Sox6	1	6.15E-105	1.09102E-43	0.006592819	-0.478298442	-0.34402686
Pde3b	1	1.50E-66	3.97317E-74	-0.02761916	-0.338079254	-0.344326752
Ablim3	1	1.78E-228	5.9287E-224	-0.0401149	-0.350296099	-0.346740912
Car3	4.25529E-07	5.58E-290	0	-0.203009077	-0.345729668	-0.346893689
Cyp4a14	1.54782E-36	1.39E-147	1.40967E-29	0.668342337	-0.660232786	-0.34725796
Cyp2c29	1	1.37E-98	6.13821E-90	0.076712077	-0.36362324	-0.347506517
Evi5	1	6.71E-58	1.39263E-32	0.061100359	-0.451404297	-0.348080676
F830016B0	1	7.89E-81	5.00693E-79	0.017250695	-0.36131009	-0.349514307
Aspg	1	4.54E-184	2.8128E-166	0.04473166	-0.359609457	-0.349694206
Larp1b	1	2.49E-85	2.22477E-42	0.05119572	-0.45769363	-0.349769412
Pbld1	1	7.36E-104	2.30639E-38	0.049896688	-0.522980163	-0.350454671
Insig2	1	6.56E-96	9.16512E-40	0.13849604	-0.493858706	-0.351075112
Ctif	1	1.93E-76	4.43747E-41	0.058756311	-0.459232139	-0.351407824
Glud1	4.38638E-05	2.46E-53	3.70613E-28	0.243267177	-0.460718965	-0.352372967
Fggy	1	2.51E-63	6.83384E-30	0.01159095	-0.501253057	-0.353530743
Sntb1	1	4.11E-83	1.08627E-34	0.015459091	-0.509143087	-0.354251278
Aldob	1	3.36E-101	1.00211E-83	0.077315641	-0.378919676	-0.354311308
Pxmp4	1	4.96E-100	9.78628E-41	0.096783073	-0.503611857	-0.35467224
Sco2	1	2.82E-247	1.031E-234	0.083617771	-0.360147656	-0.355134333
Zbtb20	1	7.20E-51	8.799E-37	0.02348803	-0.474686119	-0.355460275
Fpgs	1	3.59E-101	6.35087E-46	0.082747647	-0.487927582	-0.356531751
C6	1	2.97E-32	1.07496E-30	-0.006447625	-0.362055113	-0.3567352
Sh3d19	1	1.14E-95	3.54899E-35	0.003816098	-0.542433142	-0.357062683
Gm4788	1	1.91E-84	4.53951E-61	0.108923618	-0.397751669	-0.357194433
Syne2	1	1.19E-41	6.89536E-52	-0.08358095	-0.342590431	-0.362029215
Eva1a	1	2.42E-79	6.32251E-73	-0.027928907	-0.378611571	-0.363350449
Abcc6	1	1.90E-98	1.00146E-72	0.014878393	-0.400484774	-0.363597349
Pard3b	1	1.90E-81	1.22491E-38	-0.037536155	-0.495073352	-0.364539351
Gm29966	1.00491E-10	2.12E-115	1.25857E-23	0.330140086	-0.819941575	-0.365553389
Mob3b	1	1.68E-66	3.03118E-32	0.018362413	-0.511972007	-0.367263186
Kyat1	1	1.33E-98	3.35755E-58	-0.033503905	-0.437211568	-0.367338705
Adra1b	1	1.04E-78	2.00941E-59	-0.038978026	-0.403841642	-0.36898713
Sds	0.852890134	6.38E-135	1.5149E-116	0.15912862	-0.380587566	-0.369116467
Garem1	1	2.36E-98	1.85093E-50	0.059466358	-0.462111892	-0.369914599
Hao1	1	4.71E-300	0	-0.050040223	-0.369975716	-0.370577637
Smarca2	1	5.81E-55	3.69927E-41	-0.020594636	-0.42290675	-0.370793989
Atp11c	1	4.82E-68	2.1762E-27	-0.007105627	-0.589870017	-0.37242507

Adcy9	1	2.92E-56	6.79117E-39	-0.074740007	-0.438555257	-0.372726231
Grb14	1	2.40E-139	3.3119E-183	0.062121625	-0.367448091	-0.376937084
Upp2	1	3.98E-57	1.25849E-68	-0.036974347	-0.372465317	-0.379369596
Sdc2	1	1.28E-88	1.7919E-49	0.113982887	-0.464557104	-0.38029239
Aox3	1	2.43E-95	2.60735E-18	-0.170588239	-0.785201646	-0.380386932
Clmn	1	1.84E-88	3.26043E-92	-0.060808506	-0.382710224	-0.381550024
Akap13	1	3.91E-37	9.59579E-35	-0.048716951	-0.40461699	-0.382604341
Gm34667	1	1.10E-106	1.5922E-41	-0.023613609	-0.567085416	-0.386258744
Mia2	1	1.47E-54	2.06315E-37	0.043176612	-0.475658664	-0.387797756
Ugt2b5	1	1.08E-63	6.1216E-84	-0.029053174	-0.370336934	-0.387906114
Alas1	0.000111614	9.49E-41	1.30961E-46	-0.249918226	-0.378608311	-0.389433493
Gm31508	1	9.98E-103	1.53133E-46	0.019954837	-0.526586362	-0.391184355
A1cf	1	3.30E-105	1.97642E-33	0.026744916	-0.638021785	-0.391410658
Slc39a14	1	2.61E-77	4.50889E-92	0.063754065	-0.379283048	-0.391410771
Mup20	1	8.70E-162	0	0.102412769	-0.365285714	-0.392247793
Ephx2	1	4.40E-105	1.55713E-64	0.044280177	-0.46082115	-0.393907775
Uroc1	1	6.41E-193	5.0967E-183	-0.002344427	-0.402494995	-0.394769612
Arhgef26	1	1.55E-105	8.47467E-55	0.045812392	-0.493389679	-0.397978464
Cfhr2	1	1.02E-98	5.57897E-66	0.124326766	-0.458215336	-0.399577975
Map3k5	1	1.92E-40	2.4725E-33	0.017139546	-0.450274012	-0.402239058
Rbpms	1	8.19E-83	1.78962E-39	0.001554732	-0.580737254	-0.403771069
St3gal4	1	3.99E-22	1.28661E-25	-0.138139063	-0.411368937	-0.40391021
Ptprg	1	6.59E-113	1.21635E-45	-0.01926902	-0.598044228	-0.404103137
Cobll1	1	4.11E-104	3.29352E-54	0.153356856	-0.516422155	-0.404690637
Adipor2	1	5.91E-66	1.42395E-38	0.048258187	-0.506610461	-0.405121276
Echdc2	1	1.78E-112	6.6205E-121	0.051800431	-0.406567062	-0.407274304
Glt1d1	1	5.51E-108	7.99224E-68	0.020817501	-0.470850148	-0.407343846
Serpinc1	1	1.84E-105	5.84111E-53	0.012125746	-0.535509702	-0.407927312
Sorbs2	0.025712155	6.95E-102	4.87272E-37	0.20826702	-0.623444891	-0.40817776
Fam214a	1	2.40E-85	1.20455E-33	0.130947303	-0.625185487	-0.408884413
Dpyd	1	2.98E-157	4.08134E-26	0.122437687	-1.345247749	-0.409733886
Agmo	1	3.37E-66	1.35627E-32	-0.047426543	-0.58732498	-0.410750446
Smoc1	1	1.56E-115	1.27117E-58	0.038089129	-0.523062538	-0.412460866
Ldlrad4	1	4.96E-65	8.45546E-53	-0.108113694	-0.44885849	-0.412908531
Sik3	1	7.27E-34	1.64665E-41	-0.022799792	-0.394502279	-0.41453967
Pik3c2g	1	5.79E-128	3.83306E-56	0.013856236	-0.554584774	-0.415710683
Pcyt2	1	2.26E-110	1.13124E-51	0.102705408	-0.567213608	-0.415881191
Rbfox2	1	2.56E-100	3.15896E-59	0.007660076	-0.50462814	-0.416780081
Gm36264	1	7.12E-289	7.1268E-261	-0.153886283	-0.42392239	-0.417653061
Hpd	1	2.35E-116	4.9743E-54	0.018984396	-0.567752077	-0.41831584
Slc4a4	0.792471042	3.14E-106	2.81787E-53	0.168525405	-0.556999193	-0.419233737
Ugt2b36	1	2.12E-84	9.49915E-76	0.044254976	-0.435118023	-0.419239084
Hnf1aos1	1	3.64E-97	1.84513E-83	0.035978117	-0.438043143	-0.419971885
Cdh2	1	1.82E-111	5.09554E-43	0.075947514	-0.637560765	-0.423311595
Apoc1	1	6.47E-55	1.69135E-48	-0.065496152	-0.449572579	-0.42412552
Mtus1	1	1.71E-94	8.50885E-42	0.013055659	-0.627418892	-0.425853312
Serpina1e	1.86961E-24	0.00E+00	0	-0.328668739	-0.425096846	-0.42601289
Iqgap2	1	5.66E-40	8.62081E-33	0.094375997	-0.505504062	-0.42782194
Tmem56	1	2.22E-101	4.80929E-47	0.107513627	-0.600126834	-0.42887342
Crp	0.01366223	2.04E-101	1.67422E-46	0.223882704	-0.610850882	-0.429300035
Nlrp6	1	7.04E-136	9.3092E-145	0.001479911	-0.429676297	-0.429424633
Prkag2	1	8.02E-67	7.95303E-56	-0.057149165	-0.468873291	-0.430549527
Rabgap1l	1	6.70E-63	1.91133E-34	0.016125001	-0.577061177	-0.431708819
Gm36041	1	5.10E-100	4.43993E-63	-0.067038344	-0.500798424	-0.432995641
Zhx3	1	6.63E-112	1.30368E-64	-0.029141356	-0.543943935	-0.437343348
Plcb1	1	4.29E-81	2.42858E-38	0.122756272	-0.603715105	-0.441874683
Nr1h4	1	1.57E-118	2.0542E-51	0.03497889	-0.626702777	-0.441927648
BC024386	1	9.68E-131	1.2558E-125	0.066070904	-0.453214178	-0.442928777
Pid1	1	7.84E-43	6.46136E-30	0.164541897	-0.583622076	-0.443389059
Erbb4	1	1.91E-106	1.55905E-49	-0.049436904	-0.60871864	-0.443788413
Chsy3	1	1.06E-102	4.2111E-108	-0.006194481	-0.448854948	-0.447867675
Mylk	1	2.26E-127	2.97318E-81	-0.079091702	-0.516422348	-0.448673137
Phldb2	1	1.69E-114	4.69468E-73	-0.051495097	-0.522056616	-0.450360478

Kng1	1	1.63E-86	9.19704E-71	-0.16353438	-0.484086003	-0.450639343
Carmil1	1	2.73E-78	1.1255E-103	-0.011600288	-0.426324761	-0.451067544
C9	0.010926606	6.29E-264	0	-0.203631197	-0.44545266	-0.451850737
Zeb1	1	2.28E-71	9.48628E-69	-0.075280851	-0.461422784	-0.452908904
Hgd	0.683094806	7.97E-121	1.24352E-66	0.156926437	-0.573099393	-0.452927906
Vwa8	1	2.58E-75	1.60724E-37	0.152000978	-0.665600212	-0.454200132
Sema4g	1	1.39E-163	4.8347E-185	-0.028786735	-0.454463237	-0.458501293
Hnf4aos	1	5.44E-124	6.61807E-58	-0.14337708	-0.620446801	-0.461758331
C8a	1	1.74E-182	1.8379E-266	-0.029349247	-0.450183382	-0.465351193
Slco1a1	1	9.12E-156	2.7177E-166	0.001595516	-0.468229203	-0.46755678
Gm20319	1	5.07E-122	4.5289E-100	0.068448813	-0.494050868	-0.468344745
Ftcd	1	5.78E-130	3.21897E-97	0.022537818	-0.514198849	-0.468420712
Kyat3	1	6.17E-133	9.90494E-63	0.026686845	-0.618122194	-0.468611416
Naaladl2	1	1.04E-113	1.38978E-53	0.070724144	-0.642450772	-0.469077396
B3galt1	1	2.27E-101	5.29891E-71	-0.073196367	-0.527886947	-0.470101602
Serpina1c	1	2.28E-175	4.3665E-216	-0.064303241	-0.460411954	-0.471104856
Peak1	1	3.60E-16	3.1875E-44	0.001466661	-0.301988734	-0.471177589
Ttr	1	1.87E-101	1.40105E-58	0.003689615	-0.592807628	-0.47122419
Gc	1	3.65E-117	5.66767E-58	0.120555063	-0.667701812	-0.472946024
Prodh2	1	2.55E-141	1.1849E-136	0.062731403	-0.484480667	-0.475822276
Slc38a4	1	1.70E-131	8.23973E-67	0.035537624	-0.608262113	-0.475957381
Lars2	1	1.56E-71	8.42725E-48	-0.029306675	-0.598114069	-0.47804266
Tns1	1	5.45E-100	1.01345E-71	0.128926631	-0.545745723	-0.481108174
Sox5	1	1.96E-110	3.40319E-42	0.055814606	-0.743462744	-0.482784557
Atrnl1	1	1.71E-36	2.93442E-48	-0.102713764	-0.437475653	-0.482981487
Cdk8	1	4.47E-56	2.78318E-53	-0.096441906	-0.493331917	-0.487273563
Clpx	5.8874E-05	5.22E-107	9.49688E-60	0.229257539	-0.660039865	-0.493671515
Tdo2	9.83392E-17	1.94E-118	1.65072E-46	0.444028791	-0.777686068	-0.499588412
Crot	1	1.04E-110	1.45045E-63	-0.070785774	-0.625475259	-0.506489558
Mcc	1	5.92E-124	5.02654E-80	0.060170168	-0.597079633	-0.507877325
Cyp7b1	1	1.01E-110	2.3327E-124	-0.144398664	-0.502458746	-0.509009204
Chn2	1	2.14E-92	4.1396E-89	0.057128105	-0.523364984	-0.513871955
Pard3	1	1.20E-120	4.41668E-50	-0.001663908	-0.778545041	-0.518327781
Arhgap42	1	1.10E-136	7.44828E-78	0.032544591	-0.657035943	-0.519994164
Nfib	1	9.03E-131	1.1843E-80	0.044335797	-0.637303802	-0.52057157
Eci2	1	4.97E-122	8.10505E-68	0.144915919	-0.682220257	-0.525187966
Foxp1	1	4.58E-59	2.34452E-51	-0.125689306	-0.60601572	-0.526958204
Phyh	1	3.70E-143	6.04945E-98	0.0017023	-0.605861662	-0.528930829
Ass1	1	2.08E-106	5.44724E-49	0.115226197	-0.709803486	-0.528972726
Tat	0.000651392	1.68E-113	1.55772E-43	0.280566654	-0.83091852	-0.531985629
Dpys	1	4.54E-138	3.68724E-82	0.055357856	-0.65119289	-0.539203729
Lipc	1	1.69E-126	2.34816E-70	-0.028049287	-0.661897987	-0.541251749
Hacl1	7.81512E-08	3.95E-128	2.16462E-56	-0.326369403	-0.746408627	-0.546288264
Scp2	0.001314299	9.70E-137	8.90217E-83	-0.208315327	-0.667782098	-0.547427909
Slc25a13	1	6.58E-111	4.99287E-67	0.112440609	-0.698995377	-0.551409606
Hsd3b3	1	1.72E-140	3.6323E-140	-0.180474729	-0.561237476	-0.557442232
Col18a1	1	1.78E-150	1.6204E-101	0.112603821	-0.638080744	-0.558130693
Sugct	1	3.33E-133	3.96373E-85	0.116252758	-0.686471514	-0.569114876
Thrb	1	1.58E-60	2.3349E-50	0.077599876	-0.674264561	-0.569908837
Acsl1	1	4.81E-86	3.53319E-41	-0.094775634	-0.761026454	-0.57204073
Cfh	7.9615E-07	2.67E-63	1.18305E-57	-0.302027962	-0.599557732	-0.575492758
Akr1c6	1	1.30E-129	5.94585E-83	-0.133650079	-0.681226131	-0.581573231
Abcc2	1	4.10E-131	2.60374E-55	0.169515109	-0.9364391	-0.585089843
Camk1d	1	1.51E-51	8.08431E-93	-0.152735546	-0.430386804	-0.586769746
Itih3	1	2.26E-186	1.2185E-123	0.010172966	-0.670623298	-0.588287317
Alb	1	2.18E-114	9.22389E-59	0.172762371	-0.915619419	-0.592804672
Abcb11	1	1.82E-149	3.34161E-66	0.104938594	-0.874665323	-0.593306918
Cyp2j5	1	2.94E-164	2.815E-126	0.076400622	-0.642388917	-0.595717585
Plg	1	1.37E-142	1.80404E-81	0.078734562	-0.748825262	-0.597757506
Ptprk	1	1.55E-138	5.19015E-75	-0.019634791	-0.822287246	-0.598284816
Acox1	1	4.46E-124	6.75191E-67	0.022869455	-0.825531259	-0.598437758
Mlxipl	1	2.62E-139	2.88059E-93	-0.040044547	-0.742937272	-0.610573016
Magi1	1	1.74E-137	8.2314E-63	-0.012681737	-0.910009937	-0.613822228

Cps1	6.00525E-28	6.82E-163	2.67614E-52	0.633117547	-1.198908105	-0.615459794
Azgp1	1	4.57E-172	2.0656E-153	0.035987616	-0.641207727	-0.618600672
Pah	1	5.12E-164	3.372E-121	-0.047757912	-0.674431832	-0.618894824
Uox	1	9.42E-148	7.543E-110	-0.009493024	-0.675754633	-0.621768847
Apoa1	1	7.67E-146	9.1472E-161	-0.017004879	-0.621228157	-0.631342487
Fmo5	0.675443894	8.23E-100	3.35266E-45	0.186817556	-0.84462009	-0.631911328
Bhmt	4.34046E-13	6.81E-179	6.6124E-119	0.41735652	-0.7213005	-0.636860665
Slc7a2	1	7.15E-122	4.92227E-49	0.173453263	-1.002006401	-0.637162025
Klf12	1	1.63E-138	1.99529E-79	-0.116484601	-0.860479052	-0.661145938
Slc27a2	1	5.47E-168	3.1667E-103	0.128550147	-0.824587867	-0.663027453
Hc	1	1.14E-185	1.1696E-135	-0.06121119	-0.746116568	-0.674580987
Cyp3a25	1.82378E-25	1.84E-185	2.48124E-81	0.477326893	-1.135491858	-0.675913842
Gphn	1	2.54E-146	1.6875E-128	-0.021028707	-0.785980945	-0.683867696
Cyp2e1	1	1.55E-153	5.6626E-119	0.089070934	-0.744374772	-0.687672582
Gnmt	1	3.62E-195	1.7269E-133	-0.02767625	-0.785589237	-0.690737994
Prr16	0.195387193	2.21E-167	1.5892E-108	0.190693968	-0.829762927	-0.691211211
Apob	1	4.61E-154	1.4812E-99	0.027960781	-0.868892342	-0.69231517
Ptprd	1	8.05E-154	2.2148E-83	-0.026683047	-0.961698312	-0.69251974
Acaa1b	1	3.12E-180	2.7827E-122	0.03327111	-0.826968151	-0.692939819
Mup3	1	0.00E+00	0	-0.072602632	-0.696009066	-0.69889493
Mat1a	1	2.12E-207	4.2313E-129	0.184405637	-0.847847758	-0.702350941
Apoa2	1	4.08E-142	8.8541E-133	-0.014641615	-0.723813139	-0.704044513
Hmgcs2	2.09082E-06	8.09E-159	1.65145E-81	0.321543422	-1.029206596	-0.710343455
Ppara	1	3.85E-146	4.40022E-90	0.213373891	-0.897492876	-0.717468866
G6pc	0.280333203	1.56E-186	7.0861E-112	-0.196825722	-0.873515718	-0.724004037
Rhobtb1	1	2.89E-139	2.1689E-104	-0.029070723	-0.828356046	-0.727595837
9030622O2	0.232230208	4.10E-163	2.3869E-100	-0.227929882	-0.880210414	-0.727895682
Aass	1	8.71E-184	3.8724E-144	-0.143737469	-0.775816534	-0.728073223
Cmah	1	8.43E-168	6.5278E-146	0.000578228	-0.764032547	-0.739409692
Neat1	1	1.45E-80	1.82895E-56	-0.026547269	-0.935922065	-0.750933551
Pzp	1	9.85E-157	4.45027E-89	-0.070869284	-0.99951475	-0.757152245
Slc22a30	1	7.77E-215	4.2306E-204	-0.043438475	-0.773058598	-0.762689648
Gm19951	3.7617E-09	9.37E-75	7.6625E-105	-0.338140892	-0.654132758	-0.765021271
Cmss1	0.080517169	3.37E-119	1.758E-137	-0.169016596	-0.747915795	-0.778516278
Gfra1	0.000777608	5.57E-153	1.6509E-107	0.301563392	-0.912646171	-0.787398124
Gm42418	4.20406E-14	4.36E-149	1.9154E-163	-0.319613693	-0.797832167	-0.808489555
Kynu	1	9.40E-122	4.135E-104	0.137888812	-0.903889054	-0.813906394
Gm48099	1.15938E-07	2.36E-32	3.9978E-219	-0.346937909	-0.455460743	-0.824028902
Adk	1.00408E-06	3.70E-183	4.56376E-90	0.330842177	-1.430053234	-0.849079616
Ces3a	0.002381953	3.39E-254	5.588E-256	-0.251999927	-0.865825221	-0.864010942
Pck1	1	4.06E-248	3.4091E-162	0.093107381	-1.087314581	-0.913375407
Mug2	1	2.80E-190	1.5157E-144	-0.015001716	-1.074422751	-0.931078266
Errfi1	1	6.58E-185	3.8297E-110	0.130425129	-1.369140339	-0.957854006
Sult2a8	1	6.43E-255	6.3618E-204	-0.184228197	-1.027163805	-0.96228166
Slco1b2	1	9.91E-215	4.441E-156	0.16737664	-1.171116065	-0.964551534
Immp2l	1	1.54E-168	3.8287E-130	0.027383334	-1.297240719	-0.99694424
Ttc39c	1	4.11E-217	9.8676E-198	-0.231347287	-1.399538578	-1.3041925
Ghr	1	1.27E-230	7.934E-193	-0.198085628	-1.652291819	-1.418464046
Egfr	1.59064E-13	3.48E-257	3.3711E-234	-0.579197585	-1.679673211	-1.574300849
Mug1	5.06558E-19	1.01E-282	2.248E-260	0.494868888	-2.183578816	-1.959281617

Gene	Adj. P-Value Control vs TCDD	Adj. P-Value Control vs T+S	log2FC TCDD	-log10(P-value)	Log2FC T+S	-log10(P-value)
Mug1	1.01E-282	2.2E-260	-2.183578816	281.9943523	-1.959281617	259.6482041
Egfr	3.48E-257	3.4E-234	-1.679673211	256.4581749	-1.574300849	233.4722313
Ghr	1.27E-230	7.9E-193	-1.652291819	229.8948144	-1.418464046	192.1005064
Adk	3.70E-183	4.56E-90	-1.430053234	182.4313065	-0.849079616	89.34067719
Ttc39c	4.11E-217	9.9E-198	-1.399538578	216.3863449	-1.3041925	197.0057897
Errfi1	6.58E-185	3.8E-110	-1.369140339	184.181967	-0.957854006	109.416835
Dpyd	2.98E-157	4.08E-26	-1.345247749	156.5255482	-0.409733886	25.38919689
Immp2l	1.54E-168	3.8E-130	-1.297240719	167.8134196	-0.99694424	129.4169456
Cps1	6.82E-163	2.68E-52	-1.198908105	162.166115	-0.615459794	51.57249102
Slco1b2	9.91E-215	4.4E-156	-1.171116065	214.0041286	-0.964551534	155.3525241
Cyp3a25	1.84E-185	2.48E-81	-1.135491858	184.7354489	-0.675913842	80.60533129
Pck1	4.06E-248	3.4E-162	-1.087314581	247.3910045	-0.913375407	161.467362
Mug2	2.80E-190	1.5E-144	-1.074422751	189.553464	-0.931078266	143.8193827
Hmgcs2	8.09E-159	1.65E-81	-1.029206596	158.0918178	-0.710343455	80.78213345
Sult2a8	6.43E-255	6.4E-204	-1.027163805	254.1914542	-0.96228166	203.1964233
Slc7a2	7.15E-122	4.92E-49	-1.002006401	121.1458873	-0.637162025	48.30783477
Pzp	9.85E-157	4.45E-89	-0.99951475	156.006578	-0.757152245	88.35161387
Ptprd	8.05E-154	2.21E-83	-0.961698312	153.0942221	-0.69251974	82.65466646
Abcc2	4.10E-131	2.6E-55	-0.9364391	130.3869519	-0.585089843	54.58440187
Neat1	1.45E-80	1.83E-56	-0.935922065	79.83862448	-0.750933551	55.737799
Alb	2.18E-114	9.22E-59	-0.915619419	113.6607289	-0.592804672	58.03508566
Gfra1	5.57E-153	1.7E-107	-0.912646171	152.2545287	-0.787398124	106.7822706
Magi1	1.74E-137	8.23E-63	-0.910009937	136.7598694	-0.613822228	62.0845264
Kynu	9.40E-122	4.1E-104	-0.903889054	121.0268893	-0.813906394	103.3835254
Ppara	3.85E-146	4.4E-90	-0.897492876	145.4145231	-0.717468866	89.3565254
9030622O2	4.10E-163	2.4E-100	-0.880210414	162.3877387	-0.727895682	99.62216638
Abcb11	1.82E-149	3.34E-66	-0.874665323	148.7409448	-0.593306918	65.47604368
G6pc	1.56E-186	7.1E-112	-0.873515718	185.8070249	-0.724004037	111.1495917
Apob	4.61E-154	1.5E-99	-0.868892342	153.3359663	-0.69231517	98.82938421
Klf12	1.63E-138	2E-79	-0.860479052	137.7890769	-0.661145938	78.69999472
Mat1a	2.12E-207	4.2E-129	-0.847847758	206.6741163	-0.702350941	128.3735296
Fmo5	8.23E-100	3.35E-45	-0.84462009	99.08485896	-0.631911328	44.47461099
Tat	1.68E-113	1.56E-43	-0.83091852	112.7738263	-0.531985629	42.80751052
Prr16	2.21E-167	1.6E-108	-0.829762927	166.6549944	-0.691211211	107.7988281
Rhobtb1	2.89E-139	2.2E-104	-0.828356046	138.5388491	-0.727595837	103.6637616
Acaa1b	3.12E-180	2.8E-122	-0.826968151	179.5052211	-0.692939819	121.555532
Acox1	4.46E-124	6.75E-67	-0.825531259	123.3503311	-0.598437758	66.17057333
Slc27a2	5.47E-168	3.2E-103	-0.824587867	167.262365	-0.663027453	102.4993921
Ptprk	1.55E-138	5.19E-75	-0.822287246	137.810463	-0.598284816	74.28481983
Gm29966	2.12E-115	1.26E-23	-0.819941575	114.673513	-0.365553389	22.90012322
Gphn	2.54E-146	1.7E-128	-0.785980945	145.5956071	-0.683867696	127.7727559
Gnmt	3.62E-195	1.7E-133	-0.785589237	194.4409573	-0.690737994	132.7627417
Aox3	2.43E-95	2.61E-18	-0.785201646	94.61380952	-0.380386932	17.58380032

Pard3	1.20E-120	4.42E-50	-0.778545041	119.92009	-0.518327781	49.35490436
Tdo2	1.94E-118	1.65E-46	-0.777686068	117.7114989	-0.499588412	45.78232655
Aass	8.71E-184	3.9E-144	-0.775816534	183.0597373	-0.728073223	143.4120194
Cmah	8.43E-168	6.5E-146	-0.764032547	167.0743521	-0.739409692	145.1852312
Acsl1	4.81E-86	3.53E-41	-0.761026454	85.31798415	-0.57204073	40.45183316
Plg	1.37E-142	1.8E-81	-0.748825262	141.864349	-0.597757506	80.74375466
Hacl1	3.95E-128	2.16E-56	-0.746408627	127.403371	-0.546288264	55.6646193
Hc	1.14E-185	1.2E-135	-0.746116568	184.9415911	-0.674580987	134.9319686
Cyp2e1	1.55E-153	5.7E-119	-0.744374772	152.8100285	-0.687672582	118.2469837
Sox5	1.96E-110	3.4E-42	-0.743462744	109.7074771	-0.482784557	41.46811319
Mlxipl	2.62E-139	2.88E-93	-0.742937272	138.5815369	-0.610573016	92.54051906
Bhmt	6.81E-179	6.6E-119	-0.7213005	178.1665794	-0.636860665	118.1796384
Ass1	2.08E-106	5.45E-49	-0.709803486	105.6813137	-0.528972726	48.26382336
Slc25a13	6.58E-111	4.99E-67	-0.698995377	110.182085	-0.551409606	66.30164995
Sugct	3.33E-133	3.96E-85	-0.686471514	132.4772749	-0.569114876	84.40189552
Eci2	4.97E-122	8.11E-68	-0.682220257	121.3036701	-0.525187966	67.09124436
Akr1c6	1.30E-129	5.95E-83	-0.681226131	128.8849797	-0.581573231	82.22578624
Uox	9.42E-148	7.5E-110	-0.675754633	147.0257458	-0.621768847	109.1224533
Pah	5.12E-164	3.4E-121	-0.674431832	163.2906502	-0.618894824	120.4721068
Thrb	1.58E-60	2.33E-50	-0.674264561	59.80269525	-0.569908837	49.63173173
Itih3	2.26E-186	1.2E-123	-0.670623298	185.64648	-0.588287317	122.9141749
Scp2	9.70E-137	8.9E-83	-0.667782098	136.0133588	-0.547427909	82.05050428
Gc	3.65E-117	5.67E-58	-0.667701812	116.4380372	-0.472946024	57.24659552
Vwa8	2.58E-75	1.61E-37	-0.665600212	74.58775527	-0.454200132	36.79391828
Lipc	1.69E-126	2.35E-70	-0.661897987	125.7709779	-0.541251749	69.62927287
Zbtb16	6.47E-76	6.79E-05	-0.661635936	75.18916321	-0.24468242	4.168068622
Cyp4a14	1.39E-147	1.41E-29	-0.660232786	146.8568017	-0.34725796	28.85088272
Clpx	5.22E-107	9.5E-60	-0.660039865	106.2823532	-0.493671515	59.02241895
Arhgap42	1.10E-136	7.45E-78	-0.657035943	135.958859	-0.519994164	77.12794399
Gm19951	9.37E-75	7.7E-105	-0.654132758	74.02822987	-0.765021271	104.1156285
Dpys	4.54E-138	3.69E-82	-0.65119289	137.3434075	-0.539203729	81.43329813
Naaladl2	1.04E-113	1.39E-53	-0.642450772	112.9815273	-0.469077396	52.85705275
Cyp2j5	2.94E-164	2.8E-126	-0.642388917	163.5322497	-0.595717585	125.5505185
Col18a1	1.78E-150	1.6E-101	-0.638080744	149.750599	-0.558130693	100.7903865
Fn1	6.61E-116	6.7E-20	-0.638080439	115.1797433	-0.331140442	19.17395885
A1cf	3.30E-105	1.98E-33	-0.638021785	104.4812708	-0.391410658	32.70412142
Cdh2	1.82E-111	5.1E-43	-0.637560765	110.7388056	-0.423311595	42.29280947
Nfib	9.03E-131	1.18E-80	-0.637303802	130.0441129	-0.52057157	79.92653767
Mtus1	1.71E-94	8.51E-42	-0.627418892	93.76818158	-0.425853312	41.07012932
Nr1h4	1.57E-118	2.05E-51	-0.626702777	117.8029004	-0.441927648	50.68735757
Nfia	1.31E-60	7.18E-08	-0.626351056	59.88385723	-0.223543447	7.14372218
Crot	1.04E-110	1.45E-63	-0.625475259	109.9840365	-0.506489558	62.83849713
Fam214a	2.40E-85	1.2E-33	-0.625185487	84.61905593	-0.408884413	32.91917391
Sorbs2	6.95E-102	4.87E-37	-0.623444891	101.1582938	-0.40817776	36.31222845
Hnf4aos	5.44E-124	6.62E-58	-0.620446801	123.2645316	-0.461758331	57.17926879
Kyat3	6.17E-133	9.9E-63	-0.618122194	132.2098329	-0.468611416	62.00414818
Rapgef4	1.91E-71	2.13E-05	-0.616537047	70.72005216	-0.215431025	4.672305487

Crp	2.04E-101	1.67E-46	-0.610850882	100.6905762	-0.429300035	45.77618796
Erbb4	1.91E-106	1.56E-49	-0.60871864	105.7179237	-0.443788413	48.8071393
Slc38a4	1.70E-131	8.24E-67	-0.608262113	130.7694259	-0.475957381	66.08408706
Foxp1	4.58E-59	2.34E-51	-0.60601572	58.33895121	-0.526958204	50.62994692
Phyh	3.70E-143	6.05E-98	-0.605861662	142.4315447	-0.528930829	97.21828378
Plcb1	4.29E-81	2.43E-38	-0.603715105	80.3675505	-0.441874683	37.61464804
Tmem56	2.22E-101	4.81E-47	-0.600126834	100.6536869	-0.42887342	46.31791872
Lars2	1.56E-71	8.43E-48	-0.598114069	70.80775719	-0.47804266	47.07431395
Ptprg	6.59E-113	1.22E-45	-0.598044228	112.1812582	-0.404103137	44.91494092
Mcc	5.92E-124	5.03E-80	-0.597079633	123.2279733	-0.507877325	79.29873044
Ahsg	3.23E-93	8.86E-22	-0.594039175	92.49065406	-0.335907092	21.0524976
Ttr	1.87E-101	1.4E-58	-0.592807628	100.7270507	-0.47122419	57.85354484
Atp11c	4.82E-68	2.18E-27	-0.589870017	67.31685519	-0.37242507	26.66230148
Agmo	3.37E-66	1.36E-32	-0.58732498	65.47186801	-0.410750446	31.8676541
Pid1	7.84E-43	6.46E-30	-0.583622076	42.105932	-0.443389059	29.18967624
Abcb4	1.39E-70	0.012974	-0.582524801	69.85830225	-0.154148704	1.886919461
Rbpms	8.19E-83	1.79E-39	-0.580737254	82.08646634	-0.403771069	38.74723986
Rabgap1l	6.70E-63	1.91E-34	-0.577061177	62.17378685	-0.431708819	33.71866324
Hgd	7.97E-121	1.24E-66	-0.573099393	120.098697	-0.452927906	65.9053461
Cyp3a11	1.15E-75	2.21E-20	-0.570959389	74.93946	-0.31931815	19.65601886
Arg1	3.91E-92	7.43E-24	-0.568573094	91.40744233	-0.330918723	23.12879711
Hpd	2.35E-116	4.97E-54	-0.567752077	115.6297066	-0.41831584	53.30326808
Pcyt2	2.26E-110	1.13E-51	-0.567213608	109.6454635	-0.415881191	50.94644437
Gm34667	1.10E-106	1.59E-41	-0.567085416	105.9581107	-0.386258744	40.79800107
Cp	3.39E-77	8.8E-19	-0.562055801	76.46968757	-0.295445764	18.05543339
44256	5.33E-150	2.3E-117	-0.56120509	149.2735247	-0.519908944	116.6405446
Slc4a4	3.14E-106	2.82E-53	-0.556999193	105.5029329	-0.419233737	52.55007872
Pik3c2g	5.79E-128	3.83E-56	-0.554584774	127.2371758	-0.415710683	55.41645421
Psd3	1.80E-50	3.47E-20	-0.549485338	49.7458142	-0.340833322	19.459485
Abca6	2.19E-106	1.64E-33	-0.547638142	105.6590176	-0.334070381	32.78531865
Tns1	5.45E-100	1.01E-71	-0.545745723	99.26394483	-0.481108174	70.99419628
Zhx3	6.63E-112	1.3E-64	-0.543943935	111.1784708	-0.437343348	63.88482936
Sh3d19	1.14E-95	3.55E-35	-0.542433142	94.94343118	-0.357062683	34.44989574
Serpinc1	1.84E-105	5.84E-53	-0.535509702	104.7350925	-0.407927312	52.23350471
Ube2e2	1.06E-65	5.33E-13	-0.532596456	64.97285438	-0.275101226	12.27360647
Cat	2.61E-59	2.22E-14	-0.532221172	58.58381198	-0.273494922	13.6532199
B3galt1	2.27E-101	5.3E-71	-0.527886947	100.643888	-0.470101602	70.27581373
Cped1	2.62E-85	1.27E-19	-0.52666583	84.5809971	-0.282423354	18.89761097
Gm31508	9.98E-103	1.53E-46	-0.526586362	102.001007	-0.391184355	45.81493004
Gm4951	7.79E-57	4.12E-16	-0.523096924	56.1082097	-0.295632066	15.38544346
Smoc1	1.56E-115	1.27E-58	-0.523062538	114.8078605	-0.412460866	57.89579772
Pbld1	7.36E-104	2.31E-38	-0.522980163	103.1330719	-0.350454671	37.63706811
Phldb2	1.69E-114	4.69E-73	-0.522056616	113.7731495	-0.450360478	72.3283936
Mylk	2.26E-127	2.97E-81	-0.516422348	126.6456632	-0.448673137	80.52677908
Cobll1	4.11E-104	3.29E-54	-0.516422155	103.385738	-0.404690637	53.48233935
Dennd5b	2.72E-86	1.46E-27	-0.514634167	85.56463681	-0.313235255	26.8365056
Ftcd	5.78E-130	3.22E-97	-0.514198849	129.2380493	-0.468420712	96.4922827

Mob3b	1.68E-66	3.03E-32	-0.511972007	65.77473785	-0.367263186	31.51838865
Sntb1	4.11E-83	1.09E-34	-0.509143087	82.38641322	-0.354251278	33.964061
Lonp2	9.33E-60	2.59E-23	-0.507905871	59.02992108	-0.313505383	22.58626766
Adipor2	5.91E-66	1.42E-38	-0.506610461	65.22809215	-0.405121276	37.84650552
Rbfox2	2.56E-100	3.16E-59	-0.50462814	99.5920475	-0.416780081	58.5004556
Pxmp4	4.96E-100	9.79E-41	-0.503611857	99.30454335	-0.35467224	40.00938218
Fggy	2.51E-63	6.83E-30	-0.501253057	62.5999204	-0.353530743	29.16533498
Gm36041	5.10E-100	4.44E-63	-0.500798424	99.29264144	-0.432995641	62.35262433
Pard3b	1.90E-81	1.22E-38	-0.495073352	80.72165173	-0.364539351	37.91189446
Insig2	6.56E-96	9.17E-40	-0.493858706	95.18296147	-0.351075112	39.03786169
Nr1i3	1.51E-88	1.38E-30	-0.493676788	87.81975444	-0.326417281	29.86089275
Arhgef26	1.55E-105	8.47E-55	-0.493389679	104.8090024	-0.397978464	54.07187697
Dlc1	3.15E-39	6.31E-19	-0.490490437	38.5019732	-0.343655918	18.20019505
Fpgs	3.59E-101	6.35E-46	-0.487927582	100.4446771	-0.356531751	45.19716652
Sox6	6.15E-105	1.09E-43	-0.478298442	104.2111883	-0.34402686	42.96216613
Mia2	1.47E-54	2.06E-37	-0.475658664	53.83246252	-0.387797756	36.68546845
Plekha5	7.98E-73	3.61E-24	-0.474769249	72.09790261	-0.297353475	23.44227493
Zbtb20	7.20E-51	8.8E-37	-0.474686119	50.14253325	-0.355460275	36.05556686
Lpin1	3.12E-43	4.86E-06	-0.473455304	42.50584637	-0.266999972	5.313344386
Slc25a21	2.30E-60	0.014841	-0.471384109	59.63771427	-0.152576152	1.828525176
Glt1d1	5.51E-108	7.99E-68	-0.470850148	107.2588084	-0.407343846	67.09733128
Nr3c2	6.12E-86	1.56E-36	-0.469737218	85.21295296	-0.334080914	35.80691001
Rora	6.73E-68	6.76E-24	-0.466959325	67.17171291	-0.29216965	23.16977448
Sdc2	1.28E-88	1.79E-49	-0.464557104	87.89306224	-0.38029239	48.74668698
Myo1b	1.20E-72	3.33E-14	-0.462912453	71.9222893	-0.244964216	13.47780886
Garem1	2.36E-98	1.85E-50	-0.462111892	97.62628954	-0.369914599	49.73261094
Ephx2	4.40E-105	1.56E-64	-0.46082115	104.3568436	-0.393907775	63.80767403
Stard13	5.15E-56	2.41E-09	-0.460808748	55.28825882	-0.220384897	8.617311985
Glud1	2.46E-53	3.71E-28	-0.460718965	52.60988281	-0.352372967	27.43107904
Tmtc2	4.84E-73	5.46E-31	-0.460260729	72.31486256	-0.330324767	30.26249432
Ctif	1.93E-76	4.44E-41	-0.459232139	75.7145575	-0.351407824	40.35286481
Cfhr2	1.02E-98	5.58E-66	-0.458215336	97.98944728	-0.399577975	65.25344606
Larp1b	2.49E-85	2.22E-42	-0.45769363	84.60330517	-0.349769412	41.65271536
Agxt2	2.60E-103	5.97E-46	-0.4573253	102.5852824	-0.341640369	45.22391141
Gm48099	2.36E-32	4E-219	-0.455460743	31.62748984	-0.824028902	218.3981791
Evi5	6.71E-58	1.39E-32	-0.451404297	57.173373	-0.348080676	31.85616427
C8a	1.74E-182	1.8E-266	-0.450183382	181.7602798	-0.465351193	265.735683
Ldlrad4	4.96E-65	8.46E-53	-0.44885849	64.30461147	-0.412908531	52.07286282
Rnf125	9.79E-77	4.25E-35	-0.439210825	76.00927188	-0.339342432	34.37173931
Adcy9	2.92E-56	6.79E-39	-0.438555257	55.53463277	-0.372726231	38.16805569
Kyat1	1.33E-98	3.36E-58	-0.437211568	97.87728483	-0.367338705	57.47397736
Fhit	2.02E-52	3.41E-06	-0.433129742	51.69474915	-0.197757422	5.467159746
Camk1d	1.51E-51	8.08E-93	-0.430386804	50.82006112	-0.586769746	92.09235695
Il1r1	3.63E-47	0.000189	-0.427909236	46.44019861	-0.196788139	3.723059619
Erc1	1.08E-64	4.01E-29	-0.427182746	63.96614642	-0.307672914	28.39649127
St3gal3	5.40E-47	1.88E-25	-0.425408307	46.26781608	-0.320062511	24.72607897
Gckr	1.77E-92	1.62E-47	-0.423898446	91.75137856	-0.336787722	46.78984971

Smarca2	5.81E-55	3.7E-41	-0.42290675	54.23595148	-0.370793989	40.43188363
Aldh1l1	1.19E-79	9.83E-31	-0.414912044	78.92332945	-0.292030396	30.00746774
Bckdhb	4.47E-58	1.32E-17	-0.412816738	57.34956697	-0.249567361	16.87900335
Baiap2l1	7.05E-80	7.81E-27	-0.410432421	79.15183803	-0.271160513	26.10739161
Sorbs1	1.70E-89	4.43E-52	-0.410018573	88.76875349	-0.33940343	51.35403736
Shroom3	1.18E-68	6.97E-08	-0.407766986	67.92684585	-0.177014217	7.156926962
Msra	2.32E-47	3.73E-31	-0.406771311	46.6348087	-0.336956452	30.42881929
Hnf4a	1.08E-83	3.67E-37	-0.406427294	82.96624467	-0.303997925	36.43541301
Map7	3.51E-87	1.48E-40	-0.406159251	86.45429774	-0.305948823	39.83075708
Acnat1	1.29E-84	8.31E-26	-0.405661371	83.8908979	-0.251185622	25.08044045
Gm26917	1.84E-35	2.72E-25	-0.403338824	34.73549622	-0.323158275	24.56585726
Abcc6	1.90E-98	1E-72	-0.400484774	97.72217944	-0.363597349	71.99936543
Cdo1	1.99E-57	5.57E-15	-0.400257255	56.70094825	-0.231834072	14.25404698
Gm4788	1.91E-84	4.54E-61	-0.397751669	83.71849145	-0.357194433	60.34299063
Arhgef12	4.65E-45	1.78E-17	-0.397387224	44.33297065	-0.262489362	16.74993645
Sord	5.71E-71	2.56E-46	-0.392171619	70.2431336	-0.336875332	45.59097486
Pde4dip	8.16E-70	1.73E-42	-0.391357741	69.08832841	-0.326388997	41.76157813
Cyp27a1	3.02E-45	3.94E-14	-0.390640703	44.52010596	-0.245837857	13.40485638
Tenm3	8.13E-64	2.4E-11	-0.390321042	63.09007587	-0.217059989	10.62017978
Abcd3	2.96E-52	4.51E-24	-0.387132147	51.52817376	-0.283168089	23.34575658
Fgb	2.73E-85	4.25E-56	-0.385182084	84.56413606	-0.334038396	55.3718705
Suclg2	4.80E-52	5.01E-33	-0.382747784	51.31885808	-0.318775591	32.29973395
Deptor	1.74E-41	0.000251	-0.379066483	40.75895664	-0.165565415	3.600721372
Cep85l	3.48E-44	3.1E-14	-0.378598096	43.45791548	-0.236159462	13.50805062
F5	6.90E-78	8.57E-42	-0.376053027	77.16125571	-0.298436997	41.06727191
Tshz2	1.81E-73	2.39E-52	-0.375275287	72.74295083	-0.339170628	51.62206972
Mir99ahg	1.48E-71	7.87E-50	-0.373395732	70.8287019	-0.331359999	49.1042257
Pcx	1.71E-70	7.57E-39	-0.373323922	69.76668282	-0.306880553	38.12066721
Tox	3.39E-51	7.61E-05	-0.372004658	50.46946532	-0.174726393	4.118595922
Itpr1	2.93E-34	2.28E-25	-0.37164078	33.53340181	-0.343498181	24.64157706
Mup20	8.70E-162	0	-0.365285714	161.0605462	-0.392247793	#NUM!
Tex2	1.88E-45	2.87E-29	-0.364153845	44.72566692	-0.316351203	28.54279126
Prkce	2.21E-34	1.87E-21	-0.364093237	33.655757	-0.305947505	20.72902502
Tacc2	8.33E-55	8.53E-13	-0.362860176	54.07953859	-0.201338317	12.06895294
Chka	5.24E-36	0.002383	-0.362701921	35.28064084	-0.163339317	2.622871056
Amdhd1	1.12E-154	7.9E-101	-0.362641482	153.951294	-0.332233862	100.1040337
Mtss1	3.33E-16	0.000253	-0.361465974	15.47743604	-0.225832358	3.597334908
Gramd3	1.19E-57	5.23E-30	-0.360707136	56.92433405	-0.280742093	29.28120737
Igfbp2	3.65E-191	6.3E-107	-0.354743478	190.4375715	-0.326417737	106.1999592
Gsta3	1.28E-46	9.83E-13	-0.353032449	45.89153388	-0.215455251	12.00731458
Acot12	7.00E-80	2.29E-55	-0.351030145	79.15468617	-0.310795677	54.63987522
Ube2h	2.21E-31	3.72E-21	-0.348446233	30.65608973	-0.298125686	20.42991402
Hoga1	8.68E-73	1.04E-46	-0.347138858	72.0615224	-0.296623554	45.98284007
Sdc4	3.73E-65	4.02E-29	-0.346604174	64.42844401	-0.25646276	28.39527257
Man1a	1.17E-32	0.000291	-0.345961307	31.93331121	-0.156495401	3.536337618
Cald1	1.80E-60	6.47E-17	-0.34534341	59.74518409	-0.21054459	16.18880159
Slc22a23	1.15E-44	9.95E-26	-0.343030016	43.94118568	-0.27634741	25.00219429

Fgg	1.05E-137	3.77E-95	-0.341094367	136.9782436	-0.315455883	94.4238727
Mgll	3.06E-54	9.69E-14	-0.333122513	53.51383439	-0.190432994	13.01379939
Arhgap5	1.94E-35	9.28E-21	-0.330947151	34.71210403	-0.266121704	20.03222416
Cth	1.45E-57	8.8E-26	-0.330800705	56.84003645	-0.2530458	25.05561838
Myo6	7.20E-61	5.08E-24	-0.327042059	60.14293087	-0.230293024	23.29406561
Ralgapa2	5.03E-23	1.44E-09	-0.3256558	22.29814744	-0.22174098	8.843086429
Clec4f	2.10E-16	8.15E-07	-0.324692105	15.67719032	-0.230193987	6.088875993
Pcca	1.54E-30	6.75E-14	-0.322818829	29.81321174	-0.233856487	13.17049475
Foxo1	1.26E-37	1.77E-12	-0.320925853	36.90125177	-0.207693955	11.7530517
Rbp4	3.72E-42	3.11E-15	-0.318091443	41.42993098	-0.212069269	14.50748389
Lactb2	9.15E-39	2.06E-16	-0.317809526	38.03871662	-0.225451362	15.68658514
Hal	2.20E-148	2.85E-91	-0.315129919	147.6580908	-0.29292616	90.54585734
Prkca	3.75E-22	4.37E-05	-0.310051507	21.42609261	-0.19987122	4.359670835
Cyp2c38	2.97E-79	2.15E-48	-0.308412168	78.52673985	-0.27167685	47.66672232
Sec24a	9.79E-30	5.93E-08	-0.308340046	29.0090798	-0.185540909	7.226687514
Peak1	3.60E-16	3.19E-44	-0.301988734	15.44342461	-0.471177589	43.49654954
Insr	6.16E-26	6.35E-11	-0.30121873	25.21046722	-0.211752748	10.19733911
Epb41	1.79E-20	0.007583	-0.299899257	19.74600498	-0.153695616	2.120148761
Atxn2	1.46E-21	1.85E-08	-0.295133934	20.83526244	-0.204873736	7.731965333
Gpam	1.73E-48	4.37E-25	-0.29351326	47.76234366	-0.228911525	24.35934344
Nr5a2	1.52E-52	3.37E-19	-0.293395889	51.81804809	-0.200399474	18.47183431
Fga	2.17E-73	1E-42	-0.292946243	72.66327152	-0.24736852	41.99890738
Cyp4a10	3.76E-40	8.17E-05	-0.29057618	39.42526082	-0.141940944	4.087626916
Plpp3	2.00E-18	0.000327	-0.287650744	17.69806393	-0.17586424	3.484884364
Strbp	1.09E-30	1.11E-12	-0.287406691	29.9640289	-0.202144394	11.95524616
Pcsk6	4.59E-53	4.74E-23	-0.286768214	52.33839953	-0.209600171	22.32456037
Cdc42bpa	1.12E-54	1.85E-29	-0.285378102	53.95036732	-0.227049906	28.73307714
Wbp1l	3.88E-22	1.2E-09	-0.284949092	21.41137147	-0.207955073	8.919330438
Nedd4	6.75E-45	1.46E-21	-0.283721981	44.17073741	-0.216667554	20.83697339
Gm48633	4.02E-157	9.9E-100	-0.282914687	156.3956906	-0.265660959	99.00509812
Trf	4.01E-16	0.000293	-0.281628689	15.39693192	-0.173565101	3.533220531
Il1rap	6.59E-57	9.43E-90	-0.277215635	56.18130271	-0.311874043	89.02529102
Zfand4	7.81E-39	5.87E-61	-0.27668935	38.10715224	-0.304667426	60.23137291
Tle1	4.86E-32	1.23E-06	-0.27583721	31.31359127	-0.150089392	5.908850861
Ctcflos	8.01E-173	1.1E-119	-0.271592426	172.0962157	-0.258950486	118.9400115
Hsd17b2	2.44E-41	2.3E-12	-0.270979953	40.61237355	-0.174802587	11.63865426
Clec2d	6.42E-19	1.86E-30	-0.269932883	18.19265528	-0.331486105	29.73006502
Pcsk5	2.36E-146	1.31E-69	-0.268156542	145.6277134	-0.234987801	68.88408411
Acox2	5.81E-38	8.63E-13	-0.267201973	37.23562427	-0.17708894	12.06410892
Aqp9	6.47E-39	3.44E-10	-0.266915499	38.18919034	-0.162898959	9.46284574
Gcgr	3.58E-52	2.29E-30	-0.266717853	51.44649421	-0.221044682	29.63951387
Col27a1	1.66E-118	3.2E-183	-0.262986244	117.7809124	-0.276765578	182.4912101
Slc2a2	4.87E-201	7.2E-107	-0.261867073	200.3124945	-0.238622233	106.1430322
Dcaf6	1.92E-22	4.68E-10	-0.261045868	21.71637053	-0.192062194	9.329960237
Cbs	7.47E-88	1.28E-53	-0.258326791	87.12653909	-0.225262629	52.89266893
Gpt2	6.03E-32	7.03E-17	-0.257244198	31.21975559	-0.201797262	16.15316913
Tfr2	2.80E-138	4.91E-92	-0.255869011	137.552627	-0.236554033	91.30904829

0610005C1	2.58E-46	4.76E-21	-0.255758904	45.58804338	-0.193065756	20.32222087
Elovl2	3.28E-144	1.51E-57	-0.254983564	143.4837275	-0.216853949	56.82057943
Pex14	6.61E-20	4.87E-09	-0.252744198	19.17984357	-0.187263978	8.312205179
Ccdc141	8.58E-47	1.29E-19	-0.247199355	46.06657828	-0.178967256	18.8881299
4932438A1	6.28E-16	1.47E-07	-0.244666599	15.20195394	-0.18593801	6.832459693
Rnf217	2.41E-28	0.001897	-0.24438165	27.61883855	-0.112364424	2.721859067
Bcas3	1.32E-10	0.005706	-0.24161553	9.880235979	-0.153285024	2.243681031
Fermt2	2.30E-45	6E-27	-0.237203169	44.63867905	-0.194817569	26.22187511
Ddc	1.62E-25	7.39E-05	-0.235091158	24.79004667	-0.130532483	4.131594219
Ppp2r5e	3.49E-14	5.12E-07	-0.230898654	13.45760382	-0.175789975	6.290747276
Ncoa1	1.45E-09	4.54E-18	-0.227313296	8.839610266	-0.294409143	17.3425814
Ddah1	2.76E-29	1.93E-14	-0.22698847	28.5595838	-0.176478176	13.71503571
Ddi2	6.06E-10	0.000573	-0.222268309	9.217761201	-0.161906666	3.241831677
Aldh1a1	4.99E-21	0.002424	-0.221000787	20.30180658	-0.118495397	2.615551589
Gm4952	6.27E-148	1.46E-69	-0.220637454	147.2024666	-0.197734294	68.83574581
2900026A0	2.26E-36	1.24E-19	-0.214595057	35.64664765	-0.166859826	18.9079944
Slc47a1	2.63E-156	9.1E-100	-0.214536429	155.5797742	-0.198481529	99.0389492
Afm	1.83E-130	8.91E-79	-0.214149252	129.7366941	-0.197407211	78.0503574
Prlr	1.06E-82	2.48E-14	-0.21377995	81.97491826	-0.146052254	13.60542988
Slc17a2	8.85E-158	1.8E-86	-0.211552491	157.0532744	-0.19310719	85.74500819
Osbpl1a	4.25E-14	1.93E-05	-0.209861246	13.37131976	-0.147856955	4.71482102
Ttpa	8.62E-39	2.13E-14	-0.208425872	38.06455216	-0.149821299	13.67228446
Apoa5	4.41E-59	2.91E-22	-0.208189595	58.35591894	-0.159366708	21.53669225
F2	1.43E-80	1.13E-41	-0.206554983	79.84397177	-0.178513464	40.94799536
Brwd1	1.30E-12	0.000315	-0.20626726	11.88739552	-0.144567044	3.502359902
Aldh8a1	8.33E-94	1.91E-42	-0.204319089	93.07954677	-0.169012946	41.71803572
F11	5.94E-84	3.68E-35	-0.204038872	83.22621259	-0.16495072	34.43411168
Srr	7.35E-14	8.19E-05	-0.202179631	13.13342814	-0.13754222	4.086568409
Sgms2	1.64E-31	8.65E-62	-0.201700016	30.78497758	-0.251917274	61.06308721
Cd163	1.35E-14	0.001433	-0.198148769	13.87100285	-0.104197223	2.843639645
Adtrp	2.53E-97	6.91E-53	-0.19719265	96.59684112	-0.175288027	52.16062482
Albfm1	1.96E-73	1.24E-22	-0.192106829	72.70783288	-0.151750354	21.90830734
Serpinf2	6.32E-93	1.46E-50	-0.184930411	92.19910157	-0.161170662	49.83446476
Bhmt2	1.32E-72	2.88E-29	-0.183369443	71.87802461	-0.149589462	28.54079072
Gm31333	8.99E-107	2.81E-52	-0.182604488	106.0464412	-0.159176754	51.55193528
Maob	2.91E-138	3.4E-88	-0.182239797	137.5362907	-0.169978585	87.46907396
Acss3	6.10E-47	9.49E-24	-0.180003803	46.21438275	-0.150018588	23.02259599
Sec16b	7.35E-51	5.92E-13	-0.178154573	50.13391656	-0.121704524	12.22777065
Agpat3	2.75E-09	0.032195	-0.177360116	8.560425854	-0.114323427	1.492213484
Akr1d1	6.49E-80	3.7E-30	-0.176819795	79.18769751	-0.144020438	29.43168875
Slc6a13	5.80E-143	6.85E-77	-0.176014064	142.2365213	-0.159699009	76.16459671
Cyp2d22	7.67E-63	2.64E-30	-0.173197022	62.11543273	-0.142806993	29.57837728
Prox1	1.05E-39	4E-17	-0.172973002	38.97993988	-0.131415323	16.39842008
Ido2	3.06E-68	1.35E-36	-0.172209325	67.51470846	-0.152049867	35.86894606
Pon1	2.63E-57	2.22E-22	-0.17194726	56.57930888	-0.131913722	21.65425504
Megf9	3.56E-28	3.54E-14	-0.171904401	27.44906221	-0.143155433	13.45147759
Sec14l4	1.61E-65	5.61E-23	-0.170347036	64.79316616	-0.131980001	22.25071077

Slc17a3	2.22E-98	1.45E-60	-0.169806902	97.65437463	-0.154234071	59.8383962
Tfpi2	3.45E-88	1.95E-41	-0.167197314	87.46157956	-0.140913949	40.71095283
Lgr4	1.55E-17	0.0013	-0.1647498	16.81008915	-0.098322021	2.886176059
Masp2	2.41E-67	1.96E-21	-0.162749933	66.61720802	-0.122087628	20.70794769
Kif21a	3.58E-22	8.22E-09	-0.160948749	21.44598516	-0.113310191	8.084969146
Yap1	7.65E-51	1.61E-15	-0.160531387	50.11653644	-0.114467653	14.79191233
Ppargc1a	2.13E-68	1.1E-26	-0.16051457	67.67107701	-0.133575307	25.95942942
Slc25a15	7.70E-57	2.79E-28	-0.158730438	56.11342718	-0.132831087	27.55406081
Dhcr24	1.96E-65	6.69E-28	-0.158468748	64.70738187	-0.131521871	27.17448291
F12	8.97E-48	3.67E-20	-0.157113092	47.04720729	-0.124493175	19.43586022
Ces1c	9.07E-22	0.002707	-0.15582956	21.04262147	-0.091524207	2.567448593
Ahcy	1.36E-73	4.45E-36	-0.155299106	72.86731586	-0.132171162	35.35207477
Rdh16f2	1.43E-55	1.79E-25	-0.154366049	54.84588209	-0.127250085	24.74705576
Fam107b	1.28E-04	2.73E-22	-0.153067712	3.893831005	-0.234146238	21.56412017
Reps2	1.98E-31	7.87E-13	-0.152978937	30.70299873	-0.114469832	12.10419204
Mttp	4.82E-57	2.37E-25	-0.152283638	56.31678018	-0.124334988	24.6260649
Nhsl1	3.10E-28	8.46E-10	-0.151308482	27.50826436	-0.109877613	9.07245751
Myo5b	2.99E-26	1.46E-07	-0.151153404	25.52422186	-0.09884525	6.83593028
Fign	7.30E-70	5.11E-27	-0.150307207	69.13644784	-0.121595919	26.29189062
Gcdh	7.02E-47	4.07E-22	-0.150224676	46.15342815	-0.12002686	21.39016741
Pemt	2.42E-49	2.01E-23	-0.150146222	48.61554504	-0.124110589	22.6961924
Prkaa2	1.53E-68	1.55E-30	-0.148996304	67.81406382	-0.119847374	29.80873202
Faah	5.77E-77	2.21E-37	-0.146956567	76.23899143	-0.126626948	36.65648982
Tmem131l	1.71E-05	4.01E-21	-0.144561019	4.766284216	-0.24536884	20.3971589
Phactr4	1.21E-06	9.62E-15	-0.144354059	5.916985881	-0.1756428	14.01702322
Npas2	5.16E-41	3.22E-14	-0.1440287	40.28716132	-0.106348589	13.49253963
Bcl6	5.14E-07	2.36E-24	-0.143691374	6.289382467	-0.209293187	23.62699137
Hp	8.00E-18	2.78E-57	-0.143044163	17.09707679	-0.192820217	56.55607611
Scd1	3.38E-09	2.55E-64	-0.142062528	8.471401559	-0.242677052	63.59342076
Foxp2	2.15E-29	0.015567	-0.14185722	28.66672066	-0.074317229	1.807807397
Glyat	3.67E-43	9.94E-19	-0.140098706	42.43479197	-0.111835951	18.00251021
Rnase4	3.38E-34	8.55E-16	-0.13997436	33.47086511	-0.11319561	15.06816768
Pkhd1	2.19E-36	3.6E-18	-0.136669611	35.65981468	-0.11292612	17.44343598
Car5a	2.63E-94	7.26E-56	-0.136623549	93.580004	-0.124992319	55.13914126
Gm40438	4.72E-67	6.45E-34	-0.136571392	66.32630208	-0.118362585	33.190656
Coq8a	1.43E-59	8.9E-33	-0.135846426	58.84471601	-0.118386641	32.05044504
Sh3pxd2a	1.77E-03	4.26E-12	-0.134676947	2.752990088	-0.191512006	11.37099775
Igsf5	7.80E-29	2.18E-06	-0.134398241	28.10785614	-0.085803735	5.661949594
Cyp2d10	6.30E-39	2.68E-16	-0.132329812	38.20040834	-0.10495173	15.57109427
Inca1	1.97E-50	2E-23	-0.132063073	49.70443651	-0.108728393	22.69857272
Pygl	3.08E-08	5.57E-24	-0.132008176	7.511434749	-0.196334408	23.25437389
Agmat	7.90E-67	1.43E-23	-0.131788234	66.1022605	-0.10420194	22.84530358
Cnn3	1.55E-44	3.62E-19	-0.131467759	43.80856026	-0.105342826	18.44119867
Mb21d2	1.64E-33	1.37E-11	-0.131137703	32.78592647	-0.094328847	10.86217828
Acsm5	3.25E-35	1.15E-15	-0.130695449	34.48780527	-0.099924289	14.94082371
Atp8b1	1.50E-58	1.23E-26	-0.12979965	57.82438403	-0.109808777	25.90928807
Slc25a25	3.39E-05	9.84E-16	-0.127292054	4.469411868	-0.175449041	15.00712523

Tead1	7.88E-38	1.84E-12	-0.127160892	37.1035353	-0.095701602	11.73634159
Bbox1	2.79E-62	2.05E-21	-0.127089415	61.55389649	-0.100576212	20.68918661
Cobl	2.99E-42	1.46E-13	-0.126758743	41.52378599	-0.094122619	12.83477229
Lrig1	2.14E-79	5.91E-44	-0.126007854	78.66994113	-0.113837742	43.22818211
N4bp2	6.89E-12	3.9E-24	-0.124612469	11.161927	-0.145999401	23.40865355
Pkp2	6.79E-62	1.09E-21	-0.121186452	61.16798079	-0.096069625	20.96392492
Inpp4a	2.65E-02	1.73E-09	-0.120279781	1.576636566	-0.165166616	8.762230771
Slc10a1	4.02E-45	3.44E-23	-0.119499557	44.39602149	-0.102868492	22.46405098
Agtr1a	8.09E-62	1.35E-25	-0.119132416	61.09196366	-0.100801474	24.87074967
Palmd	1.49E-42	7.01E-18	-0.118240955	41.8271004	-0.095162513	17.15428844
Clu	4.88E-23	7.58E-48	-0.117342146	22.31122988	-0.137355069	47.12017624
Arhgap24	2.63E-03	2.39E-30	-0.116326754	2.580780061	-0.315846056	29.62076383
Chdh	7.20E-23	0.025401	-0.116175665	22.14290765	-0.057641304	1.595142966
Epb41l4b	1.21E-67	2.25E-36	-0.113965369	66.91833445	-0.101933697	35.64748716
Aldh4a1	5.21E-13	0.026008	-0.111521498	12.28355962	-0.07007174	1.584900057
Slc22a1	1.07E-29	2.14E-13	-0.111159791	28.96903553	-0.09009259	12.66995681
Hs6st1	4.77E-02	4.32E-14	-0.111053724	1.321162055	-0.180411031	13.36500772
4930404H1	2.06E-64	9.98E-34	-0.108577672	63.68558571	-0.096622403	33.00094938
Ripor2	5.35E-07	4.82E-38	-0.106750763	6.271867848	-0.243997592	37.31716364
Tjp1	2.54E-27	7E-12	-0.106323529	26.59512126	-0.083123069	11.15490437
Klhl3	6.65E-38	1.97E-17	-0.106301589	37.17731637	-0.088517405	16.70631705
Pcolce2	6.64E-06	1.2E-15	-0.105829952	5.177984479	-0.150355864	14.91916658
4833411C0	5.98E-40	5.21E-18	-0.105211509	39.22351945	-0.084871055	17.28323122
Plekha7	2.37E-35	1.52E-14	-0.103002639	34.62572861	-0.085905855	13.81826261
Bend7	9.56E-53	3.77E-27	-0.09879019	52.01975288	-0.084964692	26.42378382
Ccdc88c	6.87E-06	3.12E-18	-0.098758592	5.163106147	-0.12099965	17.50611606
Bphl	1.81E-23	2.41E-07	-0.097624629	22.74256967	-0.070972558	6.617612496
Srgap3	4.39E-04	2.79E-14	-0.097270857	3.357402453	-0.145766147	13.55456928
Abcg5	1.46E-27	0.044202	-0.096016844	26.83453813	-0.046108198	1.354556199
Gm36251	3.44E-37	4.62E-10	-0.094076808	36.46319964	-0.066428602	9.335459492
Plac8	4.34E-09	2.28E-31	-0.093271402	8.36204264	-0.134283055	30.64237544
Fyb2	6.36E-20	7.48E-06	-0.091028485	19.19648239	-0.065565452	5.126093157
Cyp2c37	2.49E-44	1.04E-18	-0.089770166	43.6042228	-0.076204961	17.98231506
Foxa3	1.10E-55	2.03E-26	-0.088861274	54.95934098	-0.077507708	25.69315305
Fat1	1.86E-51	5.37E-24	-0.087619853	50.72960543	-0.074261959	23.26977967
Neo1	5.69E-19	0.000225	-0.086937647	18.24483031	-0.057656784	3.647214092
Rfx3	9.78E-03	1.89E-11	-0.084641526	2.009488613	-0.124539774	10.72246874
Gm32063	4.58E-47	2E-16	-0.083925715	46.3393935	-0.067075394	15.69855068
Slc19a2	1.71E-17	0.000342	-0.080788007	16.76763311	-0.055603713	3.466021384
Xkr9	4.85E-27	0.025358	-0.078343643	26.31434485	-0.041457289	1.595879758
Map2k6	6.07E-04	1.38E-12	-0.077091129	3.217029067	-0.112726022	11.85899109
Khdrbs3	1.56E-43	2.53E-14	-0.076022587	42.80574167	-0.061186152	13.59641306
Gm32624	5.38E-54	3.03E-25	-0.075963542	53.26883049	-0.067314704	24.5188763
Rai14	1.67E-02	6.64E-11	-0.07585408	1.77664193	-0.121926524	10.17800352
Grem2	2.34E-23	0.003088	-0.071618527	22.63093355	-0.047134473	2.510279528
Ralgps1	8.65E-03	7.09E-12	-0.071053805	2.063191247	-0.106876359	11.14933138
Klb	3.60E-59	1.44E-19	-0.067123299	58.44323296	-0.056464054	18.84232613

Slc17a4	1.09E-47	1.01E-14	-0.066553345	46.96104862	-0.053372498	13.99717901
Hook1	2.10E-24	0.000104	-0.063556686	23.67758806	-0.043346773	3.98393141
Hjv	2.53E-25	9.52E-07	-0.061746565	24.59602372	-0.047954401	6.021345706
Tom1l1	1.62E-25	0.021362	-0.061584357	24.78941609	-0.03664452	1.670361878
Rhpn2	1.52E-26	8.33E-06	-0.057916939	25.81800192	-0.042356354	5.079138363
Sp4	1.12E-02	5.69E-12	-0.054159512	1.948946865	-0.077227988	11.24488575
Gm42109	2.06E-39	4.63E-14	-0.053711164	38.68552039	-0.044405719	13.33474411
Hnf1b	2.09E-18	0.000119	-0.053008495	17.68034529	-0.03911611	3.925410215
1810055G(	2.66E-17	0.001759	-0.050734372	16.57450425	-0.036232912	2.754652697
Pm20d1	2.44E-19	0.000138	-0.042010224	18.61336278	-0.032469533	3.860897224
Mcm10	2.11E-02	1.55E-12	-0.040288958	1.67482067	-0.059625913	11.80927369
Orm1	4.26E-02	2.76E-16	-0.028973356	1.37099456	-0.042581762	15.55921561
Gm19325	7.24E-04	1.12E-07	0.055923561	3.14010053	0.085968755	6.952270659
Cyp1b1	1.79E-05	2.28E-13	0.056219042	4.747962273	0.118020195	12.64165114
Cyp2b9	9.14E-04	1.53E-51	0.057955869	3.039105278	0.395924193	50.81508774
Adamdec1	1.25E-02	7.33E-07	0.058989673	1.904740461	0.09147794	6.134765422
Lrrc14b	7.94E-04	2.32E-08	0.059973097	3.100170922	0.100223929	7.635359878
Cyp2a4	4.24E-08	0.001023	0.074917354	7.373002725	0.04863054	2.99004722
Gm38832	1.70E-05	1.83E-15	0.076006789	4.769018282	0.150270284	14.73680774
B3galnt1	2.60E-03	2.49E-09	0.080574499	2.585024322	0.137135013	8.604029277
Scd2	1.34E-08	0.027309	0.082392107	7.87274524	0.039748439	1.563692823
Spint1	3.51E-09	1.86E-05	0.083404289	8.454207814	0.059331679	4.729425756
Plau	8.30E-08	0.003682	0.090537243	7.080929368	0.050319169	2.433937935
Mmp14	7.70E-07	0.040015	0.093554532	6.113579202	0.05880575	1.397782061
Htatip2	2.61E-04	1.15E-12	0.095379116	3.583662271	0.176772959	11.93935919
Plk2	2.79E-07	0.001963	0.095953785	6.553965788	0.060253398	2.707127975
Dennd2a	4.22E-09	0.01907	0.097045785	8.37426408	0.050100831	1.719638702
Naa20	3.72E-07	0.017114	0.098839757	6.429688608	0.066435191	1.766646848
Gm17749	6.71E-09	0.000133	0.099965664	8.173010752	0.062886468	3.874559438
Anxa5	8.64E-07	0.021152	0.100332467	6.063466044	0.068591195	1.674639328
Actg1	3.82E-08	0.00118	0.100480747	7.417554391	0.068596633	2.928151232
Tnfaip3	4.22E-08	0.003671	0.100919061	7.374315621	0.067176489	2.435171286
AU022793	1.21E-11	4E-16	0.103225216	10.9176418	0.147958482	15.39765696
Myom1	1.47E-08	7.83E-21	0.107103963	7.834003057	0.207297811	20.10603082
Arhgdib	1.70E-07	0.034233	0.108117211	6.770276729	0.067820257	1.465554987
Synj2	3.04E-09	0.000187	0.108451392	8.516467356	0.073814908	3.728960509
9130230L2	4.85E-05	1.73E-12	0.10882805	4.314468916	0.185565078	11.7620074
Rpl17	8.48E-08	0.005204	0.109136742	7.071842717	0.079390999	2.283630954
Hexb	5.20E-08	0.015813	0.109547538	7.284251737	0.068160812	1.800978835
Clec12a	2.70E-08	0.011302	0.111322191	7.56831688	0.068151056	1.946833167
Pfkp	1.06E-07	0.034827	0.111664224	6.975951402	0.071088327	1.45808889
Ap1s2	5.30E-07	0.031793	0.112588267	6.275475898	0.073135726	1.497674244
Ankrd24	1.14E-02	2.14E-09	0.115640694	1.943547612	0.194310137	8.669278374
Mefv	2.36E-10	6.66E-05	0.118750787	9.626788586	0.07919471	4.176468803
Trem2	2.92E-16	8.44E-11	0.120872538	15.53440583	0.091483127	10.07387585
Ly9	1.64E-13	2.5E-08	0.123958155	12.78487305	0.092042779	7.601961342
Capg	6.59E-13	1.9E-05	0.125513347	12.18092425	0.078333849	4.721288442

Pdgfb	1.49E-10	0.009367	0.130363814	9.827122061	0.065299709	2.028384384
Tbc1d16	2.57E-05	6.33E-11	0.131015244	4.590899122	0.19266024	10.19835723
Ncf4	1.21E-09	0.000167	0.131030708	8.917666022	0.090778059	3.776124371
Trpm7	1.17E-02	7.81E-17	0.131736177	1.933249893	0.258511415	16.10750038
Inf2	2.68E-08	0.020694	0.1321381	7.572025973	0.083118578	1.68415353
Wdr1	1.04E-09	0.00465	0.137340251	8.982244771	0.086711901	2.332535785
Mfsd12	5.98E-16	4.5E-08	0.138520064	15.22333506	0.086911279	7.347127704
Taldo1	8.95E-11	3.67E-05	0.13982061	10.04809955	0.099319295	4.435903021
Plxdc1	1.17E-09	0.00045	0.143731606	8.931141964	0.08976504	3.346436478
Plxna1	4.16E-12	1.08E-06	0.144697876	11.38059311	0.10746733	5.965528676
Itga5	1.78E-07	0.041694	0.146948015	6.749231623	0.090793308	1.379921826
Emb	5.76E-10	0.000365	0.150387404	9.23988203	0.094594645	3.437261874
Nisch	2.54E-03	1.08E-09	0.15100691	2.594848621	0.229554913	8.965761911
Anpep	1.34E-13	4.92E-05	0.15263307	12.8742096	0.090608596	4.308390221
Plekho1	1.35E-11	0.030469	0.155613366	10.87090202	0.084449896	1.516137276
Rab20	1.11E-07	0.034661	0.15586689	6.954615478	0.100849526	1.460156116
Tmem189	2.50E-10	0.000658	0.156161957	9.601866391	0.105223871	3.181710032
Maf	2.91E-02	1.3E-07	0.156450613	1.536652598	0.23479478	6.88521431
Mapkapk3	3.50E-11	0.000155	0.158054555	10.45597084	0.106360153	3.809850055
Adap1	9.78E-11	0.003001	0.158210458	10.00953712	0.100122225	2.522797878
Vsir	1.51E-10	0.000866	0.159127436	9.822030146	0.104828175	3.062293275
Ms4a6b	4.17E-10	2.01E-19	0.160268528	9.379354441	0.237885399	18.69607562
Irf5	8.77E-12	0.002825	0.161549714	11.0572213	0.094761977	2.549005161
C5ar1	1.85E-18	6.91E-10	0.163440064	17.73347764	0.102775437	9.160827354
Plgrkt	1.95E-11	2.19E-05	0.164210315	10.71050853	0.12723299	4.660025489
Stx12	1.65E-13	2.67E-05	0.164796164	12.78245493	0.111459684	4.573191581
Gm4258	9.38E-11	0.009101	0.167990954	10.02776107	0.089907674	2.040894867
Slfn2	6.90E-13	1.04E-05	0.168712701	12.16142442	0.108090866	4.98482422
Swap70	1.64E-10	0.000875	0.16999703	9.784650201	0.115286263	3.058123416
H2-Eb1	3.25E-11	0.002921	0.171942355	10.48857823	0.086672029	2.534428783
Herc1	1.37E-05	2.71E-14	0.172011931	4.862135297	0.249019866	13.56656548
Tmem181a	1.52E-11	0.003355	0.174990619	10.8171838	0.11134783	2.474365818
Arrb1	8.88E-12	0.000368	0.175588299	11.05170329	0.111752505	3.434081527
Ciita	1.08E-06	0.041634	0.177343863	5.967198358	0.107230681	1.380550932
Cd9	7.99E-13	0.000558	0.177780786	12.09730307	0.099628854	3.253190973
P2ry6	9.68E-12	0.002386	0.178384128	11.01390142	0.102086366	2.622358072
Cyba	2.55E-13	0.000592	0.179637462	12.59377829	0.110101485	3.228005671
Abi3	5.63E-11	0.008097	0.180334442	10.24965679	0.106225731	2.091667157
Loxl3	7.69E-21	1.84E-13	0.180928093	20.11393371	0.140471393	12.73424007
Oxct1	6.23E-11	0.000826	0.181053607	10.20584448	0.118002831	3.082879713
Tmem65	2.44E-09	0.010457	0.181414894	8.613303336	0.109111511	1.980582572
Asah1	4.80E-10	0.009283	0.182713625	9.318997533	0.118637139	2.032325845
Mirt1	1.48E-15	8.23E-08	0.18349719	14.83050094	0.114405612	7.084689309
Atf3	1.86E-19	2.5E-09	0.184776108	18.72984643	0.113039985	8.602146685
Ptpn6	4.65E-16	5.19E-09	0.185114115	15.33294583	0.143046477	8.28482255
Ulbp1	2.01E-17	1.52E-25	0.185455139	16.69609688	0.257421919	24.81838721
Tenm4	7.29E-10	0.013638	0.185754642	9.137555209	0.096411276	1.865253131

Mgat4a	1.34E-08	0.005187	0.186902377	7.872976823	0.130666665	2.285074055
Wls	1.63E-17	1.07E-06	0.188785923	16.78687998	0.113149963	5.971707939
Plekha1	5.42E-15	0.00024	0.190195556	14.26561092	0.10822687	3.619500255
Cap1	8.87E-14	0.015378	0.19032396	13.05203586	0.097557017	1.813093732
Ctsz	4.02E-15	0.010196	0.190873194	14.39570613	0.091803674	1.991565192
Blnk	7.97E-15	0.001791	0.192248826	14.09881093	0.08432757	2.746870005
Arhgef2	1.63E-13	0.000382	0.19246054	12.78910203	0.12245237	3.417465142
F630028O1	1.02E-13	3.37E-07	0.194459652	12.98999402	0.14167408	6.472782229
Il6st	4.96E-08	0.022312	0.195213742	7.304679479	0.134484435	1.651455368
Gls	9.23E-13	0.028373	0.195377789	12.03474319	0.097740067	1.547095557
Impact	4.40E-16	1.03E-08	0.195408848	15.35623378	0.152162629	7.986562624
Arpc1b	1.60E-12	1.65E-05	0.195474108	11.79467587	0.14190201	4.783301906
Galnt6	2.20E-20	4.33E-10	0.195649099	19.65812172	0.12408048	9.363766626
Apaf1	8.90E-14	2.91E-05	0.196138475	13.05049169	0.131405214	4.536102897
Tmsb4x	4.56E-15	0.000335	0.196415156	14.34093646	0.110346663	3.475143116
Cd300c2	9.00E-16	3.69E-07	0.196450047	15.04583874	0.134978346	6.432903709
Gm2245	1.57E-16	0.001547	0.197008365	15.80477632	0.086911919	2.8105192
Ankfy1	8.03E-10	0.015927	0.198781317	9.095296016	0.130706479	1.797875087
Hmox1	3.62E-14	0.000226	0.198883176	13.44154954	0.098501992	3.645818022
Clcn5	6.50E-13	1.91E-06	0.201280979	12.18723112	0.142706947	5.719258079
Slf2	1.27E-09	0.001703	0.201309367	8.896475647	0.145090297	2.768735906
Nfe2l2	2.18E-05	8.11E-22	0.201404152	4.660788687	0.334911314	21.09107858
Plekhm1	5.40E-12	1.15E-05	0.201639095	11.26753617	0.14797736	4.940016363
Mga	1.67E-10	1.01E-16	0.201856783	9.776227331	0.268435669	15.99739555
Adam8	7.16E-19	1.49E-11	0.201933806	18.14524281	0.145521582	10.82680803
Foxn2	1.50E-11	4.25E-05	0.202868437	10.82264371	0.153759328	4.371425897
Lpxn	2.16E-18	2.1E-11	0.205397567	17.66523076	0.16081507	10.6771333
Parvb	3.84E-17	5.54E-08	0.207202988	16.41589336	0.14121074	7.256154149
Gna13	2.98E-13	0.005118	0.208598916	12.52646837	0.12243412	2.290920984
Wwp2	1.32E-10	0.002104	0.208649501	9.878350879	0.140486343	2.676996318
N4bp1	1.77E-15	9.06E-06	0.210772	14.75099038	0.12794981	5.043000739
Axl	5.62E-11	0.008096	0.213112014	10.25053215	0.121420006	2.091712739
Serinc3	4.32E-08	1.43E-15	0.213617969	7.364206224	0.289507751	14.84408462
Il7r	6.04E-15	8.88E-09	0.215100532	14.21874577	0.155775223	8.051524667
Ogt	6.66E-09	0.030021	0.215197175	8.17633864	0.144853858	1.522579524
Snx27	2.22E-13	4.77E-06	0.215466027	12.65416341	0.161240111	5.321835012
Alpk1	2.08E-08	0.012085	0.215660892	7.682799148	0.141201865	1.91773718
Nipa2	4.41E-13	1.93E-06	0.21567312	12.35535736	0.163181338	5.714313253
Gm37168	4.35E-27	3.21E-17	0.215935844	26.36186251	0.148818733	16.49387504
Ehd4	6.60E-14	0.00014	0.21676782	13.18074318	0.127797068	3.85338932
Nlrp1b	2.29E-09	0.004882	0.218326144	8.639744731	0.147173878	2.311364552
Plekho2	1.53E-14	0.01847	0.218373945	13.81479282	0.106617318	1.733544331
Arhgap45	2.45E-11	8.4E-05	0.219130448	10.61066508	0.14791024	4.07575622
Piezo1	9.43E-14	1.03E-05	0.219136878	13.02553367	0.147541232	4.989023672
Fbxl5	9.38E-16	5.88E-07	0.219849839	15.02798681	0.144068112	6.230881898
Mylip	2.89E-18	9.74E-06	0.222956906	17.53976513	0.123446792	5.011399192
Aim2	2.58E-14	1.91E-07	0.223658279	13.58798072	0.169598614	6.718998056

Mrc1	4.57E-02	3.67E-13	0.223721079	1.34029519	0.386651618	12.43523281
Arhgap30	2.14E-12	0.003544	0.22525493	11.67034713	0.136743798	2.450486267
Nsf	2.52E-10	0.008308	0.22672021	9.597811099	0.142186618	2.080488231
Tmem106a	1.27E-16	1.5E-09	0.227072054	15.89560657	0.172464257	8.82466475
Rsu1	7.16E-11	0.012079	0.227820883	10.14498854	0.141735917	1.917973773
Sptlc2	2.17E-12	0.005887	0.227917359	11.66365684	0.137011047	2.230093276
Gm50020	2.54E-21	1.37E-33	0.228869054	20.59565951	0.324962004	32.8647962
Jak2	9.94E-14	0.016969	0.231504487	13.00246076	0.120328777	1.770345939
Ptpra	2.65E-12	0.010364	0.232220816	11.57635691	0.140269446	1.984469474
Snx8	2.85E-15	4.55E-05	0.233168761	14.54571222	0.142748536	4.342441732
Plek	2.18E-16	0.000384	0.23424296	15.66192758	0.109016264	3.415518552
Stk4	6.77E-14	1.43E-06	0.235178772	13.1696339	0.173197181	5.844333553
B4galt6	5.59E-17	1.21E-07	0.235782305	16.25281779	0.146348707	6.91756529
Creb5	2.59E-18	7.43E-10	0.236020748	17.58620593	0.165729151	9.129144669
Dennd1a	7.34E-11	0.002281	0.236895995	10.13428404	0.156438441	2.641815134
Rasa4	2.53E-19	7.61E-10	0.236961675	18.59700512	0.166597102	9.118559412
Tsc22d1	5.92E-18	0.002463	0.237045936	17.22760828	0.109333955	2.608533228
Ncoa7	7.73E-16	1.89E-08	0.237213823	15.11185566	0.179470446	7.723913752
Fblim1	1.76E-27	3.87E-18	0.237971622	26.7541246	0.18473957	17.41250649
Ric1	5.36E-13	1.13E-05	0.242488299	12.27099785	0.178817464	4.945837212
Akr1a1	1.92E-17	4.95E-07	0.244483896	16.71601307	0.170474743	6.305072431
Card11	4.32E-15	1.47E-08	0.24493456	14.36448344	0.162453364	7.832235406
Nrg1	1.21E-30	1.95E-93	0.244957104	29.91743703	0.655710502	92.71097893
Ikbke	4.09E-20	3.78E-11	0.248852874	19.38816477	0.18146627	10.4221755
Mir142hg	5.05E-23	2.89E-09	0.249041713	22.29665557	0.149925047	8.538730569
Rab7b	1.11E-26	1.82E-12	0.25055592	25.95502643	0.150521126	11.74093174
Elf4	1.22E-12	0.037336	0.251042466	11.91415851	0.135968288	1.427871107
Vps8	4.09E-20	3.11E-09	0.25167298	19.38816373	0.174585528	8.506693362
Lacc1	2.76E-21	1.28E-33	0.251883164	20.55901328	0.34185666	32.8929852
Atp6v0d1	1.44E-18	2.5E-08	0.252271649	17.84181989	0.179969311	7.602414752
Nrp2	5.89E-23	5.9E-11	0.253450044	22.22970051	0.148192373	10.22913965
Frmd4a	7.55E-13	0.000106	0.25386549	12.12182585	0.156980092	3.974756741
Prex1	2.12E-12	0.002497	0.253890263	11.67285573	0.152889424	2.602593646
Tiparp	2.92E-24	1.8E-35	0.254671584	23.53507737	0.346246653	34.74560324
Pirb	8.40E-20	1.79E-10	0.255976667	19.07591066	0.194588233	9.746074698
Cd74	7.35E-18	0.000351	0.256882715	17.1339428	0.121779798	3.454415468
Msn	2.33E-16	0.003486	0.25765308	15.63248431	0.12698095	2.457705116
Klf7	2.40E-13	0.009988	0.258238821	12.61899029	0.14309743	2.000517271
Mbnl2	1.38E-11	1.92E-05	0.259107744	10.85964532	0.179532519	4.715657684
Pak2	3.46E-14	0.012852	0.261167422	13.46133269	0.145288508	1.891043457
Adcy7	5.19E-17	8.39E-06	0.261427249	16.28495337	0.168753144	5.0763788
Anxa3	1.02E-22	2.46E-13	0.263337328	21.99208384	0.188683593	12.60900548
Ms4a4a	1.58E-25	1.94E-35	0.264332639	24.80081948	0.333074187	34.71183304
Cyp1a1	9.90E-37	1.77E-46	0.264516227	36.00444899	0.345340689	45.75253745
Slc37a2	8.65E-30	2.69E-22	0.2682279	29.06318289	0.209793333	21.57002429
5031439G0	7.85E-17	0.014069	0.269550369	16.10517692	0.135398966	1.851751542
Anxa4	4.68E-24	1.37E-14	0.270082586	23.32991655	0.199871085	13.86199432

Gas2l3	1.99E-33	4.69E-20	0.270494094	32.7004869	0.189924732	19.32917542
Map4	1.06E-14	0.006812	0.271994952	13.97437052	0.156176555	2.166747038
Il10rb	1.55E-22	1.21E-08	0.273090798	21.81059255	0.165237917	7.9171679
Dtnbp1	6.10E-18	4.75E-09	0.277969745	17.21501831	0.203881074	8.323454966
Tmem51	9.20E-14	9.68E-06	0.278583669	13.0361748	0.186180448	5.013973651
Smchd1	5.69E-15	4.45E-06	0.279190118	14.24474003	0.1967603	5.351459611
Abi1	1.75E-17	0.038911	0.279364638	16.75656145	0.127341008	1.409922819
Rapgef5	8.38E-21	4.19E-13	0.279887205	20.07664148	0.203237555	12.37796379
Actb	3.62E-19	4.38E-06	0.279993307	18.44080587	0.159460289	5.358907791
Coro7	3.99E-16	3.42E-07	0.280201288	15.39863099	0.198523854	6.465439115
Prkcd	2.90E-18	0.002919	0.282623138	17.53784512	0.134948629	2.5347031
Cltc	1.05E-19	5.61E-09	0.284063302	18.97981093	0.212255242	8.250771623
Fam129b	4.68E-23	4.93E-12	0.285042	22.32930883	0.175038126	11.30725773
Sh3bp2	2.17E-21	2.59E-09	0.285471272	20.6637803	0.189061932	8.586800478
Dock11	1.07E-17	1.24E-08	0.287548229	16.97100817	0.20567177	7.908223213
Fndc3b	1.45E-14	6.76E-07	0.288396343	13.83822457	0.205255212	6.169896689
Rasa3	1.93E-12	0.000693	0.288619872	11.71460844	0.186033873	3.159024171
Dennd1b	2.38E-14	0.001462	0.2898896	13.62419678	0.173367287	2.835121213
Coro1c	7.82E-26	3.99E-12	0.291219948	25.10658382	0.195662091	11.39876112
Vav1	7.98E-24	1.87E-11	0.294361017	23.0977682	0.208486309	10.72855955
Dock4	6.24E-11	8.84E-29	0.294656035	10.20491196	0.4346475	28.05360014
Pitpna	1.04E-14	0.003624	0.295603583	13.98440909	0.167701508	2.440855581
Nceh1	6.08E-18	1.33E-10	0.295885372	17.21621814	0.223851799	9.876561975
Sdcbp	1.74E-26	9.1E-11	0.297026995	25.75871265	0.182823686	10.04102302
Arpc2	5.61E-19	1.36E-08	0.298578382	18.25078625	0.209613534	7.867535273
Actr2	2.34E-22	0.000255	0.298620665	21.6314946	0.148839863	3.593983501
Hk3	1.72E-22	2.34E-11	0.300138491	21.76523921	0.210011512	10.63022893
Ahrr	1.64E-35	1.03E-45	0.301603123	34.78615813	0.361587571	44.98613478
Slc7a8	1.24E-12	1.54E-05	0.302989951	11.90644407	0.191296398	4.811484893
Cd180	7.29E-19	5.12E-05	0.303878077	18.1371495	0.16346973	4.291005038
Amz1	2.09E-34	6.16E-22	0.303884957	33.68069644	0.223364757	21.21066907
B430306N(	4.62E-34	1.3E-24	0.304383665	33.33531774	0.246459533	23.88599732
Cyth4	8.63E-24	6.15E-08	0.305046722	23.06407576	0.171034463	7.211027852
Cotl1	5.20E-23	0.039495	0.305428025	22.2839206	0.114121482	1.403459652
Cyfip1	8.68E-20	1.75E-08	0.30611167	19.06170649	0.199087389	7.756670513
Baz1a	6.21E-21	7.42E-11	0.313578041	20.20707065	0.232026896	10.12935565
Aopep	2.00E-14	6.85E-31	0.314335285	13.69852018	0.402248639	30.16426899
Cdh18	8.27E-22	1.35E-80	0.316833487	21.08232431	0.641596694	79.86907612
Cd274	1.91E-31	1.67E-18	0.318851159	30.7198514	0.22690762	17.77831661
Specc1	1.28E-24	9.96E-14	0.320287114	23.89365142	0.218226131	13.00163721
Morrbid	4.83E-16	0.005783	0.321804115	15.31627027	0.147624058	2.237839734
Gnaq	6.97E-20	2.56E-05	0.322171424	19.15652087	0.184040898	4.590989115
Rnf150	2.05E-15	1.18E-06	0.323081261	14.68848581	0.196560129	5.929585738
Wwp1	2.12E-09	8.24E-35	0.324863509	8.672809944	0.541837679	34.08388029
Rreb1	9.83E-18	9.65E-05	0.325202777	17.00761743	0.182536473	4.015680982
Unc93b1	2.52E-22	1.81E-07	0.328346198	21.59879207	0.20413436	6.742831742
Fmo3	9.97E-34	1.3E-153	0.328858519	33.0014843	0.968962285	152.896187

Rab31	9.73E-21	2.09E-06	0.32906724	20.01201475	0.185325536	5.679739533
Arhgap31	1.70E-20	1.95E-11	0.329607146	19.76915149	0.241588615	10.71058861
Filip1l	3.24E-18	5.21E-27	0.330095852	17.48948735	0.415840517	26.28299929
Slc15a3	4.45E-25	8.28E-07	0.332362426	24.35181225	0.174477068	6.082107311
Reln	1.56E-26	1.2E-120	0.333351268	25.80706859	0.804744037	119.9377046
Hexa	3.87E-30	4.5E-16	0.336577771	29.41262652	0.246809512	15.34718338
Tnfrsf11a	5.51E-23	1.39E-07	0.337570396	22.25914989	0.191690654	6.855976511
Tbc1d9	3.21E-21	4.7E-09	0.342328176	20.49358611	0.227558967	8.32789749
Mdfic	1.21E-22	1.06E-10	0.350282551	21.91631449	0.235279039	9.976660276
Ptbp3	2.01E-25	3.17E-11	0.350966384	24.69644747	0.240847865	10.49887296
Ptpn1	2.22E-21	3.22E-08	0.352319618	20.65429958	0.232619739	7.491814977
Itgb5	2.09E-20	3.6E-12	0.353536083	19.68086872	0.276001237	11.44359148
Soat1	3.59E-26	8.47E-13	0.355386049	25.44491804	0.244955391	12.07212956
Ano6	5.70E-14	2.82E-07	0.355549478	13.24381455	0.257584376	6.550507698
Gng2	3.31E-22	1.22E-11	0.35927061	21.4806003	0.271992417	10.91193237
Otulinl	9.18E-29	4.19E-18	0.360560368	28.03729123	0.273296919	17.37807949
Rbm47	6.66E-26	1.59E-16	0.361976038	25.17672021	0.287989379	15.79727827
Abcc1	1.35E-35	5.46E-21	0.363979197	34.86892152	0.269845683	20.26296886
Vrk2	5.32E-18	0.000313	0.365218167	17.27403111	0.208743245	3.504947025
Myh9	4.68E-22	8.24E-09	0.365490905	21.32986967	0.243060648	8.083882408
Nckap1l	1.72E-33	4.29E-15	0.365587474	32.76394361	0.239503273	14.36752479
Adap2	1.06E-18	1.57E-11	0.366053902	17.97602717	0.285277642	10.80485474
Epb41l2	5.31E-27	1.86E-09	0.36844251	26.27520374	0.219676252	8.730184713
Cbl	8.13E-24	2.22E-08	0.369385675	23.08970346	0.228826106	7.652697597
Fam20c	1.49E-27	2E-10	0.369441135	26.82656681	0.211992363	9.698291151
Rtn4	2.98E-26	7.3E-06	0.371637498	25.52527224	0.190516849	5.1365762
Gsap	1.07E-22	6.73E-07	0.372854944	21.97061334	0.216066388	6.171859286
Basp1	6.66E-32	1.43E-22	0.378803843	31.17653798	0.280544076	21.84520185
Lrp1	6.67E-26	1.27E-41	0.379909594	25.17569069	0.473046454	40.8947343
Pparg	2.51E-30	4.97E-17	0.38071325	29.60030228	0.257353629	16.30333198
Itpkb	2.23E-23	0.000426	0.38088677	22.65089598	0.17247665	3.37029201
Lipa	1.82E-33	4.84E-19	0.384308723	32.74038319	0.277518612	18.31554886
Atp8b4	2.71E-30	1.75E-20	0.38496575	29.56764853	0.29450229	19.75764625
Fnbp1	1.31E-20	1.41E-10	0.386194843	19.88305438	0.278753064	9.849539611
Ptpn12	6.81E-26	1.33E-13	0.387791333	25.16681839	0.287575675	12.87617495
Ncf2	3.14E-35	3.08E-16	0.389031076	34.50308405	0.254882886	15.51091608
Cd300lb	1.10E-42	2.12E-21	0.390542978	41.95697517	0.24768666	20.67433873
Mrtfa	9.64E-24	6.96E-11	0.390914772	23.01585468	0.274268695	10.15709655
Picalm	3.17E-26	0.000907	0.395410341	25.49905743	0.174015694	3.0422028
Malat1	1.76E-75	3.76E-56	0.401069156	74.75420579	0.339056768	55.42494493
Chst11	1.37E-26	4.96E-08	0.404341178	25.86301439	0.16095156	7.304294664
44256	1.93E-18	7.63E-06	0.406235043	17.71426967	0.259652023	5.1172736
Slco3a1	8.77E-21	6.04E-13	0.406980139	20.05682898	0.321851484	12.21870563
Pou2f2	2.03E-13	1.47E-39	0.411142093	12.69237913	0.717148327	38.83326027
Abcc5	7.87E-33	1.36E-20	0.412067041	32.10400124	0.300329991	19.86533726
Abhd12	3.50E-30	5.01E-14	0.41220919	29.45599651	0.265961454	13.30021884
Atp6v0d2	2.78E-31	1.06E-24	0.414474878	30.55552583	0.318145882	23.97334568

Pik3r5	1.52E-33	7.64E-16	0.421399122	32.81868261	0.273367554	15.11716453
Dip2b	2.97E-31	2.36E-15	0.421502136	30.52773984	0.297441051	14.62686761
Camk2d	1.53E-22	1.82E-10	0.421600518	21.81599093	0.285673904	9.738888852
Hpcal1	1.65E-21	0.00016	0.428374011	20.78358366	0.225854842	3.796311627
Ccdc148	3.50E-14	8.63E-27	0.43056599	13.45641196	0.569722598	26.06408179
Lcp1	8.20E-31	8.89E-10	0.431938306	30.08643707	0.245488611	9.051290523
Atp6v1b2	1.56E-39	8.08E-25	0.435024275	38.80679711	0.325461464	24.09234331
Atp2b1	2.66E-25	7.96E-11	0.435704395	24.57497522	0.286466738	10.09904486
Pik3cb	1.12E-37	1.17E-21	0.437099589	36.94906913	0.312933549	20.93299535
Litaf	1.30E-31	7.12E-08	0.4404708	30.88680739	0.232465923	7.147814869
Csf2rb	1.60E-47	1.07E-30	0.442134012	46.79635113	0.349140986	29.97233456
Parp8	5.70E-28	1.24E-12	0.442235047	27.24413694	0.302240784	11.90775605
Cers6	7.39E-24	9.73E-08	0.444455412	23.13118836	0.263609906	7.011958886
Ppm1h	3.13E-25	1.03E-12	0.44514987	24.50425848	0.325006873	11.98801842
Syk	1.26E-32	0.000112	0.450697287	31.90104458	0.186380589	3.952085312
St18	4.23E-34	3.75E-25	0.453867054	33.3734054	0.338197401	24.42651812
Wipf1	2.07E-29	5.34E-09	0.455232624	28.68486402	0.264243242	8.272815746
Plekhm3	4.99E-31	1.51E-07	0.458078918	30.30160605	0.22774865	6.820200984
Myo5a	2.37E-33	1.45E-17	0.462382822	32.62527432	0.305103166	16.8373592
Ubash3b	9.56E-30	3.9E-12	0.463566726	29.01967403	0.283501807	11.40935023
Airn	2.10E-20	6.23E-50	0.465285692	19.67724841	0.61559563	49.20527831
Slc6a6	8.92E-40	1.68E-25	0.467108786	39.04987115	0.369068535	24.77526879
Gsr	1.05E-34	3.14E-48	0.470721803	33.97814827	0.559019685	47.50341526
Tbc1d22a	1.11E-38	5.4E-16	0.471331207	37.95382234	0.303270521	15.26733138
Dgkz	1.26E-38	8.81E-28	0.472057737	37.89998486	0.381856693	27.05520179
Atp8a1	2.35E-31	2.88E-18	0.481744281	30.6282451	0.37025982	17.54128921
Ctsd	1.98E-65	3.21E-35	0.481920845	64.7026433	0.326393256	34.49313954
Dnmt3a	4.10E-30	3.31E-20	0.484467562	29.38724081	0.373875524	19.47982525
Abcg1	7.25E-44	1.06E-22	0.491459632	43.13948183	0.329770722	21.97487048
Kcnk13	2.05E-31	1.45E-22	0.492644592	30.68804868	0.394837179	21.83724526
Snx5	6.90E-47	9.11E-32	0.49416524	46.16141898	0.39880098	31.04037036
Sh3kbp1	2.90E-32	3.84E-19	0.496698251	31.53705926	0.372779651	18.41562469
Atp6v0a1	9.68E-50	1.76E-15	0.51363107	49.01418333	0.254451368	14.75503509
Myo9b	2.28E-52	1.9E-28	0.514595934	51.64291467	0.373673353	27.72087302
Actr3	4.11E-48	3.16E-27	0.51569526	47.38594622	0.382473362	26.49968655
Myo9a	3.04E-24	4.71E-12	0.516679779	23.51780083	0.363253229	11.32728782
Adgb	3.30E-16	7.71E-52	0.518098493	15.48096436	0.863236884	51.11306739
Msr1	9.47E-36	2.89E-22	0.528208171	35.02363521	0.407061561	21.53915278
Hip1	7.48E-40	9.29E-27	0.535235685	39.12624319	0.42486824	26.03207591
Entpd1	5.10E-31	1.63E-13	0.535451996	30.29277828	0.34611687	12.78784931
Aff1	1.42E-41	6.72E-25	0.545246425	40.84816142	0.418147645	24.17271905
Plekhm2	1.50E-47	2.58E-36	0.55715854	46.82415699	0.463266448	35.58836179
Inpp5d	3.95E-46	6.05E-16	0.557547095	45.40375159	0.324346536	15.21831348
Cyp1a2	2.97E-81	5.9E-108	0.557775652	80.52661485	0.737916746	107.2276857
Fmnl2	1.89E-23	9.77E-11	0.56169173	22.72407922	0.394382338	10.01005631
Apbb1ip	8.44E-49	5.07E-20	0.573567397	48.07350227	0.36536275	19.29485512
Cd44	6.88E-40	1.43E-05	0.577271069	39.16257123	0.241657783	4.845191847

Skap2	1.45E-50	1.58E-12	0.578121865	49.83739743	0.29232497	11.80067155
Gm5150	1.82E-37	3.75E-63	0.585124999	36.73999705	0.766457165	62.42614616
Eepd1	1.63E-44	5.53E-26	0.586097748	43.78798182	0.417961957	25.25698046
Arhgap25	6.00E-49	1.22E-20	0.599987383	48.22148915	0.357019808	19.91317319
Evl	4.79E-51	9.5E-27	0.603062904	50.31930062	0.402030134	26.02242434
Tbxas1	6.05E-49	8.31E-22	0.653284816	48.21824713	0.406245672	21.08047131
Ctsb	7.07E-59	2.64E-35	0.66044137	58.15078674	0.479974261	34.57908423
Elmo1	4.48E-58	1.01E-25	0.664820152	57.34894752	0.437913454	24.99370288
Cerk	5.26E-65	1.49E-51	0.665173147	64.27864114	0.534854276	50.82738252
Ksr2	1.54E-32	4.48E-17	0.666451885	31.81366926	0.405027996	16.34906045
Rftn1	3.21E-54	4.18E-39	0.66693459	53.4941119	0.526304356	38.37899001
Dock2	5.44E-52	2.3E-10	0.672609796	51.26473742	0.306326783	9.63796848
Mmp27	4.26E-67	4.5E-111	0.685753403	66.3709027	0.972984643	110.348919
Abca1	9.24E-49	3.56E-30	0.698569089	48.03447481	0.485726563	29.44901224
Sirpa	5.84E-66	7.11E-56	0.703206824	65.23367625	0.616256084	55.14825712
Fmn1	2.12E-63	1.24E-52	0.718672518	62.67404794	0.601683819	51.90673287
Lpl	9.56E-77	1.9E-64	0.73908452	76.01969582	0.593516818	63.72204865
Myof	4.11E-79	5.7E-57	0.756360296	78.38568095	0.560896972	56.24375722
Lgals3	2.69E-83	2.98E-57	0.76191156	82.56949111	0.56050936	56.52565261
Mitf	3.13E-54	2.35E-35	0.769956927	53.50376528	0.563816609	34.62880309
Vav3	2.83E-69	1.13E-42	0.773530058	68.54807435	0.56120507	41.94722615
Gab2	2.90E-66	1.73E-51	0.791066155	65.53703136	0.683810175	50.76235085
Iqgap1	3.50E-91	1.76E-45	0.817652357	90.45544745	0.547175469	44.75571921
Zeb2	2.77E-56	8.19E-27	0.822474091	55.55675005	0.556678299	26.08681053
Slc8a1	6.73E-53	5.38E-35	0.85155254	52.17219866	0.665599756	34.26928963
Psap	1.07E-101	1.85E-70	0.89632113	100.9693208	0.704305864	69.73343575
Lrmda	2.34E-80	2.11E-77	0.988787667	79.63064601	0.918618646	76.67621863
Myo1f	2.56E-106	8.18E-67	0.996639118	105.5922622	0.725520931	66.08710225

Top 25 Volcano Plot Gene Clusters from DAVID

DEG Group	Cluster	Description	Score	DEG Group	Cluster	Description	Score
Upregulated	1	Polar Residues	10.73	Downregulated	1	Plasma Membrane	20.52
	2	Endoplasmic Reticulum	9.13		2	Actin Binding	10.80
	3	Lipid Metabolism	8.08		3	Pleckstrin Homology Domain	10.29
	4	Transmembrane	8.08		4	Cytoskeleton	6.61
	5	Nucleotide Binding	6.83		5	Src Homology Domain	5.59
	6	Mitochondrion	5.81		6	Lysosome	5.00
	7	Fatty Acid Metabolism	5.73		7	Transmembrane	4.87
	8	Oxidoreductase Activity	4.64		8	Myosin	4.83
	9	Glycoprotein	4.18		9	Endoplasmic Reticulum	4.74
	10	Nuclear Receptor/Transcrip	4.04		10	ATP Binding	4.59
	11	Cholesterol/Steroid Metabo	3.98		11	Rho GTPase	4.12
	12	Tyrosine Metabolism	3.48		12	Protein Kinase	3.73
	13	ABC Transporters	3.27		13	Cell Junction	3.65
	14	Cell Membrane	3.25		14	Chemokine Signaling	3.61
	15	Urea Cycle	3.14		15	Metal Binding	3.53
	16	Drug Metabolism	3.13		16	B Cell Receptor Signaling	3.31
	17	Pyridoxal Phosphate-Deper	3.13		17	Actin	3.23
	18	GTPase Regulation	3.07		18	Calponin Homology	2.66
	19	Cell Junction	2.95		19	Lipid Metabolism	2.46
	20	Tryptophan Metabolism	2.83		20	Immune System Process	2.45
	21	Alpha 2 Macroglobulin	2.72		21	Apoptosis	2.35
	22	PDZ Domain	2.65		22	Lipoprotein	2.29
	23	Insulin Signaling Pathway	2.59		23	Cell Surface	2.19
	24	Actin Binding	2.58		24	Glycoprotein	2.14
	25	Cartilage Development	2.52		25	Ras-Association	2.14

TCDD

DEG Group	Cluster	Description	Score
Upregulated	1	Endosome	8.23
	2	Transport	4.40
	3	Pleckstrin homology domain	4.28
	4	Cell-cell adhesion	4.23
	5	HEAT repeat	3.32
	6	Lipid binding	3.10
	7	Immunity	3.08
	8	Kinase activity	2.79
	9	Src homology domains	2.72
	10	B/T cell receptor pathway	2.64
	11	Leishmaniasis/pertussis	2.61
	12	Metal binding	2.45
	13	Cell division	2.37
	14	Cytoskeleton	2.36
	15	Diseases (tuberculosis, etc)	2.36
	16	AH/BAR domain	2.27
	17	Ubiquitin complex	2.06
	18	SEC7	1.99
	19	mRNA binding	1.93
	20	Cell-cell adhesion	1.85
	21	Vesicles	1.76
	22	Ubiquitination	1.58
	23	Leishmaniasis; infection	1.56
	24	GTP binding	1.51
	25	Notch signaling	1.49
	26	Various signaling pathways	1.45
	27	Cell projection	1.35
Downregulated	1	WW domain	2.32
	2	Oxidoreductase activity	1.51

TCDD + Statin

DEG Group	Cluster	Description	Score
Upregulated	1	Apoptosis	1.84
	2	Oxidoreductase	1.66
	3	Nucleus	1.60
Downregulated	1	Sarcoplasmic reticulum	1.54
	2	Ubiquitin activity	1.51

TCDD + Statin			
**DEG Group**	**Cluster**	**Description**	**Score**
Upregulated	1	Cell Division	7.87
	2	Cytoskeleton	4.68
	3	Microtubule	3.83
	4	ATP Binding	2.22
	5	PDZ Domain	2.21
	6	Methyltransferase	1.72
	7	Actin Binding	1.59
	8	Endoplasmic Reticulum	1.53
	9	IQ Domain	1.52
	10	Ubl Conjugation	1.37
	11	Nuclear Pore Complex	1.34
Downregulated	1	Metal-Binding	1.85
	2	Protein Lipidation	1.76
	3	Complement Pathway	1.57
	4	Chromo Domain	1.49
	5	GTPase Activation	1.43
	6	Protein Kinase	1.42
	7	Transcription	1.35
	8	Endoplasmic Reticulum	1.34

Gene	T Cells		Hepatic Stellate Cells		Hepatic Portal Fibroblasts		Liver Dendritic Cells		Endothelial Cell of Hepatic Sinusoid		Central Hepatocytes		Cholangiocytes		B Cells	
	TCDD	T+S	TCDD	T+S	TCDD	T+S	TCDD	T+S	TCDD	T+S	TCDD	T+S	TCDD	T+S	TCDD	T+S
Ahr	0.01	0.15	0.05	0.10	-0.07	-0.02	0.02	0.32	-0.10	-0.04	0.59	0.63	0.13	0.19	0.00	0.13
Ahrr	0.27	0.38	0.35	0.39	0.22	0.29	0.55	0.73	0.15	0.22	1.33	1.36	0.25	0.37	0.06	0.18
Cyp1a1	0.22	0.39	0.29	0.43	0.19	0.35	0.89	1.24	0.86	0.92	1.56	1.49	0.64	0.66	0.29	0.42
Cyp1a2	0.51	0.78	0.45	0.61	0.48	0.94	1.16	1.47	0.54	0.68	1.81	1.94	0.45	0.71	0.55	0.76
Cyp1b1	0.06	0.14	0.22	0.22	0.12	0.28	0.36	0.60	0.19	0.22	0.86	0.95	0.19	0.23	0.07	0.13
